# Evolutionary Dynamics and Expression Divergence of the *MADS-Box* Gene Family During Recent Speciation of AA-Genome *Oryza* Species

**DOI:** 10.3390/plants14030379

**Published:** 2025-01-26

**Authors:** Jiaqi Tian, Lizhi Gao

**Affiliations:** 1 Engineering Research Center for Selecting and Breeding New Tropical Crop Varieties, Ministry of Education, Tropical Biodiversity and Genomics Research Center, Hainan University, Haikou 570228, China; tjq921111@163.com; 2 Institution of Genomics and Bioinformatics, South China Agricultural University, Guangzhou 510642, China

**Keywords:** AA-genome *Oryza* species, *MADS-box* gene family, gene duplication, gene expression divergence, speciation

## Abstract

To investigate the evolutionary trajectory during the recent speciation of AA-genome *Oryza* species, we conducted a comprehensive analysis of the *MADS-box* gene family across eight *Oryza* species. We identified 1093 *MADS-box* genes in total and systematically examined their evolutionary history, gene family expansion, and expression divergence. Our results revealed that extensive lineage-specific expansions occurred in AA-genome *Oryza* species, which were primarily generated by proximal and tandem duplications, with a particularly notable episode in Type-I genes. Despite the significant expansion, Type-I genes were generally expressed at low levels or not expressed across various organs. In contrast, the expansion of Type-II genes was primarily observed in the AG, AGL12, SOC1, GGM13, and MIKC* subfamilies, which exhibited high levels of expression in reproductive organs such as panicles and stigmas. Additionally, we found species-specific gene expression in the two out-crossing wild rice species, *Oryza rufipogon* and *Oryza longistaminata*. Notably, a unique *MADS-box* gene in *O. longistaminata* exhibited high expression levels in rhizomes and stems, which may be associated with the species’ distinctive rhizomatous growth habit.

## 1. Introduction

The *MADS-box* gene family plays a role in all stages of plant development and is among the most extensively studied gene families. It is crucial for defining floral organ identity, controlling flowering time, supporting vegetative growth, and contributing to the development of seeds, fruits, pollen, and embryo sacs [1,2,3]. MADS-box transcription factors are generally divided into two types, Type-I and Type-II, based on their protein structures. Type-I proteins possess a single MADS-box domain and can be further subdivided into Mα, Mβ, and Mγ subgroups [4,5]. Type-II genes, including the MEF2-like genes in animals and fungi and MIKC-type genes in plants, have products containing MADS (M), Intervening (I), Keratin-like (K), and C-terminal (C) domains [6]. Plant MIKC genes are further subdivided into MIKCc-type and MIKC*-type, with MIKCc-type divided into fourteen different subfamilies [7,8,9]. MIKCc-type MADS-box transcription factors are further categorized into five distinct classes, A, B, C, D, and E, based on their homologous functions in floral organ identity [10,11]. MADS-box transcription factors and their complexes determine the identity of floral organs, with floral patterning explained by the ABC or ABCDE model [12]. In the ABC model, the letters represent A (*APETALA2*, *AP2*), B (*APETALA3* and *PISTILLA*, *AP3*/*PI*), and C (*AGAMOUS*, *AG*).

To date, a total of 75 *MADS-box* genes have been identified in the *Nipponbare* genome of *Oryza sativa*, which play important roles in various developmental processes of rice [8]. Among them, 16 genes are primarily divided into five classes according to the ABCDE model. Class A (*AP1*) genes, including *OsMADS14* [13,14], *OsMADS15* [15,16], *OsMADS18* [17,18], and *OsMADS20* [19], regulate the development of panicles and glumes and induce early flowering under short-day conditions. Class B (*PI*/*GLO*/*AP3*) genes, such as *OsMADS2* [20], *OsMADS4* [21], and *OsMADS16* [19,22,23,24], specify the identities of petals and stamens. Class C (*AG*) genes, including *OsMADS3* [21,25,26,27] and *OsMADS58* [28], modulate the development of stamens and carpels. Class D (*AGL*) genes, comprising *OsMADS13* [27,29,30], *OsMADS21*, and *OsMADS29* [31], regulate the development of ovules. Class E (*SEP3*/*AGL9*/*AGL2*) genes, such as *OsMADS1* [32,33,34,35,36], *OsMADS5* [37], *OsMADS7*/*8* [37,38], and *OsMADS34* [39], control the development of floral organs and induce early flowering, determine the development of floral organs, and induce early flowering [40,41]. The *AGL6* genes, such as *OsMADS6*/*17* [42], control the development of floral organs and meristematic organs. The *AGL17* genes, including *OsMADS25* [43] and *OsMADS23*/*27*/*57*/*61* [44,45], regulate root development. The *TM3-like* genes, such as *OsMADS50* [46] and *OsMADS51*/*56* [47,48], control flowering time. The *STMADS11-like* genes, including *OsMADS22* [49,50] and *OsMADS47*/*55* [51], respectively, regulate inflorescence reversal and flowering time. The *AGL12* genes, such as *OsMADS26* [50,52], enhance drought resistance and resistance to rice pathogens. The *Mγ* genes, like *OsMADS87* [53], determine seed size and thermosensitivity. It is noteworthy that most of the aforementioned *MADS-box* genes belong to Type-II, with only one gene (*OsMADS87*) classified as Type-I.

The genus *Oryza* encompasses about 24 wild species, covering 11 genome types: AA-, BB-, CC-, BBCC-, CCDD-, EE-, FF-, GG-, HHJJ-, HHKK-, and KKLL-. Among the eight AA-genome *Oryza* species, Asian cultivated rice (*O. sativa*) and African cultivated rice (*Oryza glaberrima*) are cultivated species. *O. rufipogon*, *Oryza nivara*, *Oryza barthii*, *O. longistaminata*, *Oryza glumaepatula*, and *Oryza meridionalis* are their wild relatives [54,55,56], which are recognized as crucial germplasm resources for the genetic improvement of cultivated rice [54]. The genome sequencing and annotation of AA-genome *Oryza* species have formed a solid foundation to obtain comprehensive insights into species differentiation, genomic variation, and innovation in the genus *Oryza* [55,56,57,58,59]. While prior studies have characterized *MADS-box* genes in individual *Oryza* species, such as *O. sativa*, or in specific AA-genome subgroups, a comprehensive comparative analysis covering the entire AA-genome group of *Oryza* has been lacking [60]. There is a gap to be filled by systematically characterizing *MADS-box* genes across high-quality representative *Oryza* genomes. Such a broad-scale investigation will provide an unprecedented opportunity to understand the evolutionary trajectories, duplication patterns, and expression divergences of *MADS-box* genes during speciation events in *Oryza*. With this regard, it is essential to examine key evolutionary pressures such as “neutral selection”, “positive selection”, and “purifying selection”. Neutral selection involves no impact on fitness, positive selection favors advantageous mutations, and purifying selection eliminates deleterious variants to maintain the functional integrity of genes [61,62]. Detection of these selective pressures may offer insights into the genetic diversification and functional specialization of *MADS-box* genes in the genus *Oryza*.

In this study, we systematically characterized *MADS-box* genes in the fifteen high-quality genomes from the ten *Oryza* species including all eight AA-genome species and two closely related species. We identified a total of 1093 *MADS-box* genes, which significantly expanded the known repertoire compared to previous studies. We also conducted comprehensive analyses of the origin, evolution, and domain structures of the *MADS-box* gene family across the *Oryza* AA-genomes. Our results revealed patterns of amplification and expression divergence in the *MADS-box* gene family, particularly highlighting unique expressed genes in *O. longistaminata* and *O. rufipogon*. This study not only establishes a foundation for understanding the evolution and expression divergence of the *MADS-box* gene family during the recent speciation of AA-genome *Oryza* species but also guides future functional studies and breeding programs.

## 2. Results

### 2.1. Identification of the MADS-Box Gene Family

**Figure 1:**
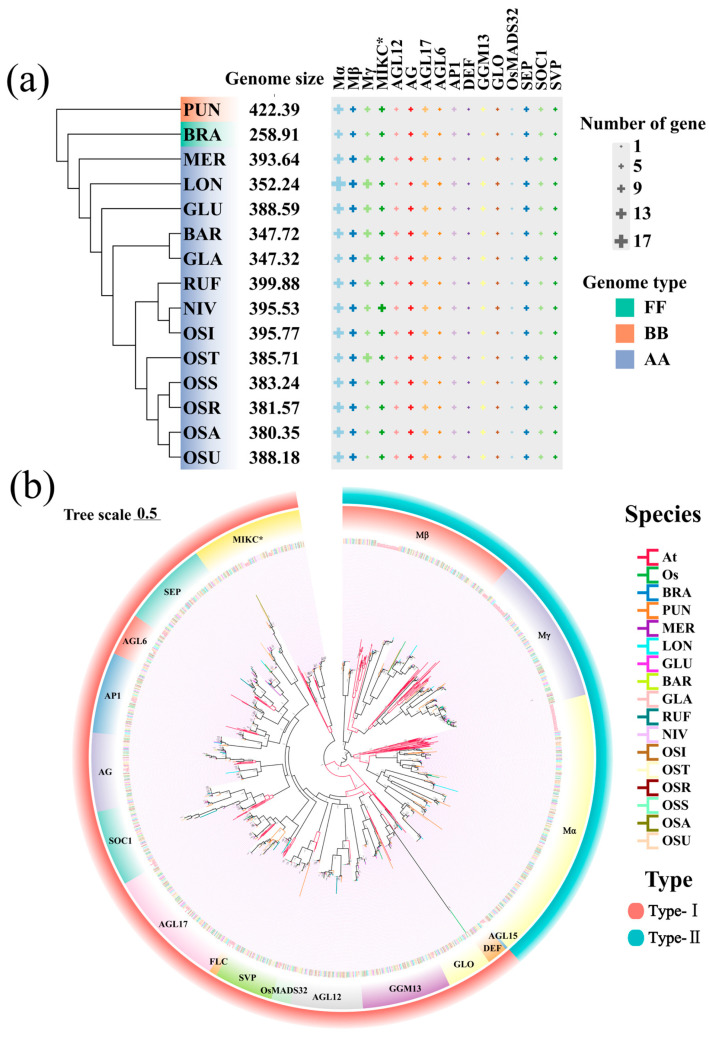
Evolution of *MADS-box* genes across AA-genome *Oryza* species. (**a**) The distribution of *MADS-box* subgroup genes across AA-genome *Oryza* species using BB- and FF-genome *Oryza* species as outgroups. The size of the cross indicates the number of genes. (**b**) The phylogenetic tree of *MADS-box* genes within different *Oryza* genomes together with *Arabidopsis thaliana*, where the outermost layer represents the classification of subfamilies (Type-I and Type-II), and the next layer represents the classification into 18 subfamilies. The abbreviations of these species are given as follows: Nipponbare of *O. sativa* (Os), *A. thaliana* (At), *O. brachyantha* (BRA), *O. punctata* (PUN), *O. meridionalis* (MER), *O. longistaminata* (LON), *O. glumaepatula* (GLU), *O. barthii* (BAR), *O. glaberrima* (GLA), *O. nivara* (NIV), and *O. rufipogon* (RUF). *O. sativa* was further divided into Temperate *Japonica* (OST), Tropical *Japonica* (OSR), Subtropical *Japonica* (OSS), *Aus* (OSU), and *Aromatic* (OSA).

To understand the evolutionary pathway of MADS-box transcription factors, we analyzed fifteen high-quality genomes from eight AA-genome *Oryza* species, as well as the FF-genome species (*Oryza brachyantha*) and the BB-genome species (*Oryza punctata*) as outgroups. (Figure 1a, Table S1). Among them, the *O. sativa* genomes were further sampled by including five subspecies: *Indica* (OSI), Temperate *Japonica* (OST), Tropical *Japonica* (OSR, including Subtropical *Japonica* OSS), *Aus* (OSU), and *Aromatic* (OSA), resulting in six genome assemblies. Using Hidden Markov Model (HMM) and BLAST searches, this study identified a total of 1430 *MADS-box* genes. After screening for conserved domains, 1093 *MADS-box* genes were finally confirmed (Table S2). Among them, the AA-genome *Oryza* species contained a total of 535 *MADS-box* genes with *O. meridionalis* of 73 genes, *O. longistaminata* of 87 genes, *O. glumipatula* of 78 genes, *O. barthii* of 76 genes, *O. glaberrima* of 74 genes, *O. rufipogon* of 69 genes, *O. nivara* of 78 genes, and *O. sativa* of 75 genes. Our results showed that the number of *MADS-box* genes was relatively conserved across these genomes. For instance, *O. brachyantha* had the fewest *MADS-box* genes (62), while *O. longistaminata* harbored the most (87). The study presented detailed information on the phosphorylation sites, amino acid counts, molecular weights, isoelectric points, aliphatic indices, hydrophilicity indices, and subcellular localizations of these genes (Table S3). These results indicate that *MADS-box* genes exhibited to some extent the conservation during evolution, with a trend of amplification in the AA-genome species compared to the BB- and FF-genome species.

### 2.2. Classification and Phylogenetic Analysis of the MADS-Box Gene Family

To elucidate the phylogenetic relationships of MADS-box transcription factors in AA-genome *Oryza* species, we constructed a phylogenetic tree using 1168 *MADS-box* genes from *Oryza* along with 109 *MADS-box* genes from *Arabidopsis thaliana* (Figure 1b). The *MADS-box* genes in *Oryza* were classified into two major categories: 402 Type-I genes and 691 Type-II genes (Table S2). Further phylogenetic analysis divided the Type-I genes into Mα, Mβ, and Mγ subgroups, while the Type-II genes were divided into 13 subgroups, including MIKC*, AG, AGL12, AGL17, AGL6, AP1, DEF, GGM13, GLO, OsMADS32, SEP, SOC1, and SVP. Compared to the dicot species *A. thaliana*, the *Oryza* genomes lacked the FLC and AGL15 subgroups but had an additional OsMADS32 subgroup. Evolutionary analysis of *MADS-box* genes across the 15 *Oryza* genomes revealed that the Type-I genes in the AA-genomes significantly expanded compared to FF- and BB-genomes with 20 and 24 genes, respectively. Among AA-genome *Oryza* species, *O. longistaminata* exhibited the most notable expansion of Type-I genes with a total of 38.

### 2.3. Structural and Regulatory Complexity of MADS-Box Genes

Structural diversity and cis-regulatory complexity of *MADS-box* genes were investigated across AA-genome *Oryza* species. Our results showed that Type-I and Type-II *MADS-box* genes exhibited discernable structural differences (Supplementary Figure S1, Table S4). Specifically, Type-I genes were characterized by a simpler structure, containing one to five coding DNA sequences (CDS), whereas Type-II genes harbored a more complex architecture with one to fifteen CDS. Despite these differences, genes within the same phylogenetic subgroup generally shared similar CDS features, particularly in terms of intron and exon length. The result suggested that the structural conservation within subgroups may have largely contributed to maintaining their functional roles in the AA-genome *Oryza* species.

We further identified several conserved motifs in MADS-box proteins, with motifs 1 and 6 aligning with the MIKC-type MADS domain. Motif 1 was universally present across all newly identified subgroups, while motif 6 was mainly found in the Mγ subgroup. Other motifs, such as 2, 5, 8, 9, and 10, were exclusive to Type-II genes, further highlighting their functional and structural distinctions. In addition, we observed 62 unique cis-regulatory elements (cis-elements) in the *Oryza MADS-box* genes (Supplementary Figure S2, Table S5), which could be classified into five major categories based on their roles in environmental adaptation. Our results showed that the most prevalent cis-elements were core promoter elements, although their distribution varied among species, indicating diverse regulatory roles. Moreover, we identified abiotic stress-responsive elements and organ-specific elements including CAT-box, which play critical roles in flower development, leaf formation, and root growth. These findings provide crucial insights into the functional diversification of *MADS-box* genes in the genus *Oryza*.

### 2.4. Chromosomal Locations of MADS-Box Genes

**Figure 2:**
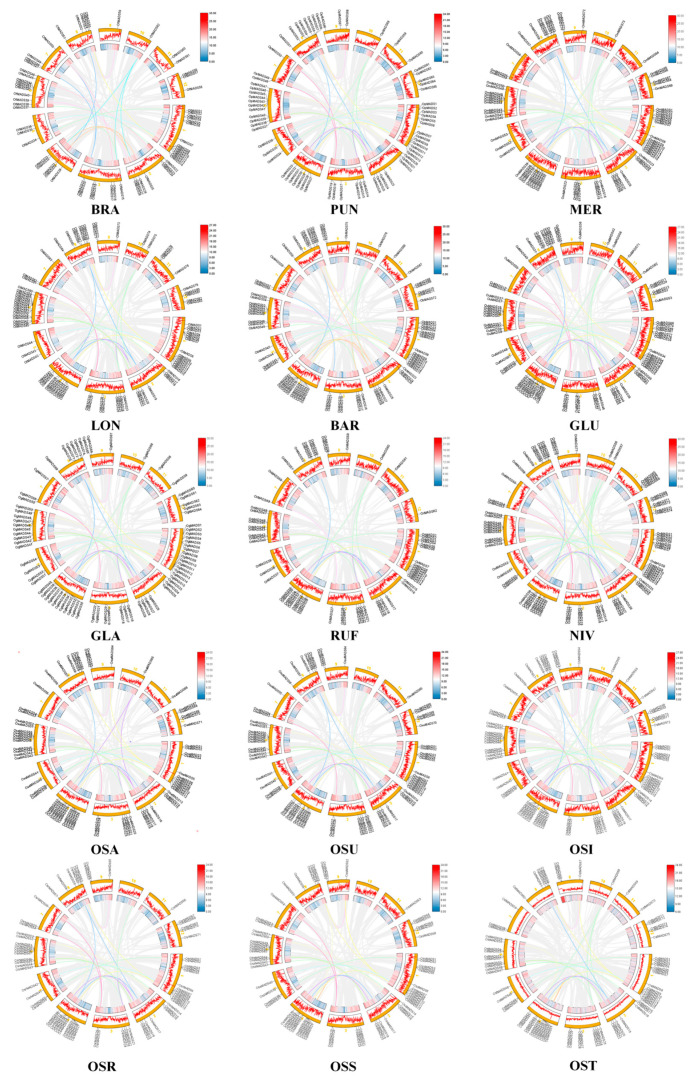
Distribution of *MADS-box* genes in the fifteen *Oryza* genomes. The outermost layer displays gene names, the next layer represents chromosomes, the third layer is a line plot of gene density, the fourth layer is a heatmap of gene density, and the innermost layer illustrates syntenic connections between genes. The differently-colored lines in the innermost layer represent syntenic relationships within the genome, with the same color indicating genes that are syntenic to each other. The abbreviations of the species are as follows: *O. brachyantha* (BRA), *O. punctata* (PUN), *O. meridionalis* (MER), *O. longistaminata* (LON), *O. glumaepatula* (GLU), *O. barthii* (BAR), *O. glaberrima* (GLA), *O. nivara* (NIV), and *O. rufipogon* (RUF). *O. sativa* is further represented by Temperate *Japonica* (OST), Tropical *Japonica* (OSR), Subtropical *Japonica* (OSS), *Aus* (OSU), and *Aromatic* (OSA).

To position the genomic localization of the identified *MADS-box* genes in AA-genome *Oryza* species, we employed Advanced Circos program in TBtools (v2.075) to illustrate their physical distribution across chromosomes (Figure 2, Table S6). The results revealed a significant uneven distribution of *MADS-box* genes across the 12 chromosomes. Notably, Chromosome 1 harbored the largest number of *MADS-box* genes, followed by Chromosome 6. There were significant differences in the number of *MADS-box* genes on Chromosome 4 among different genomes (AA-, BB-, and FF-genomes). For instance, Chromosome 4 contained only seven and five *MADS-box* genes in *O. punctata* (BB-genome) and *O. brachyantha* (FF-genome), respectively, whereas the AA-genomes exhibited a remarkably high gene density. We surprisingly observed differences among the subspecies of *O. sativa*. For example, no *MADS-box* genes were detected on Chromosome 10 in the *Aus*, Subtropical *Japonica*, and Tropical *Japonica* subspecies, while 1–2 genes in the *Aromatic*, Temperate *Japonica*, and *Indica* genomes were located on this chromosome. Additionally, we found that some *MADS-box* genes could not be mapped to any known chromosomes in genomes such as *O. longistaminata*, *O. rufipogon*, and *O. nivara*. Notably, Chromosome 6 in the *O. longistaminata* genome harbored 16 *MADS-box* genes, which was the largest number detected among all these examined genomes.

### 2.5. Comparative Genomic and Molecular Evolutionary Analyses of Specifically Amplified MADS-Box Genes

**Figure 3:**
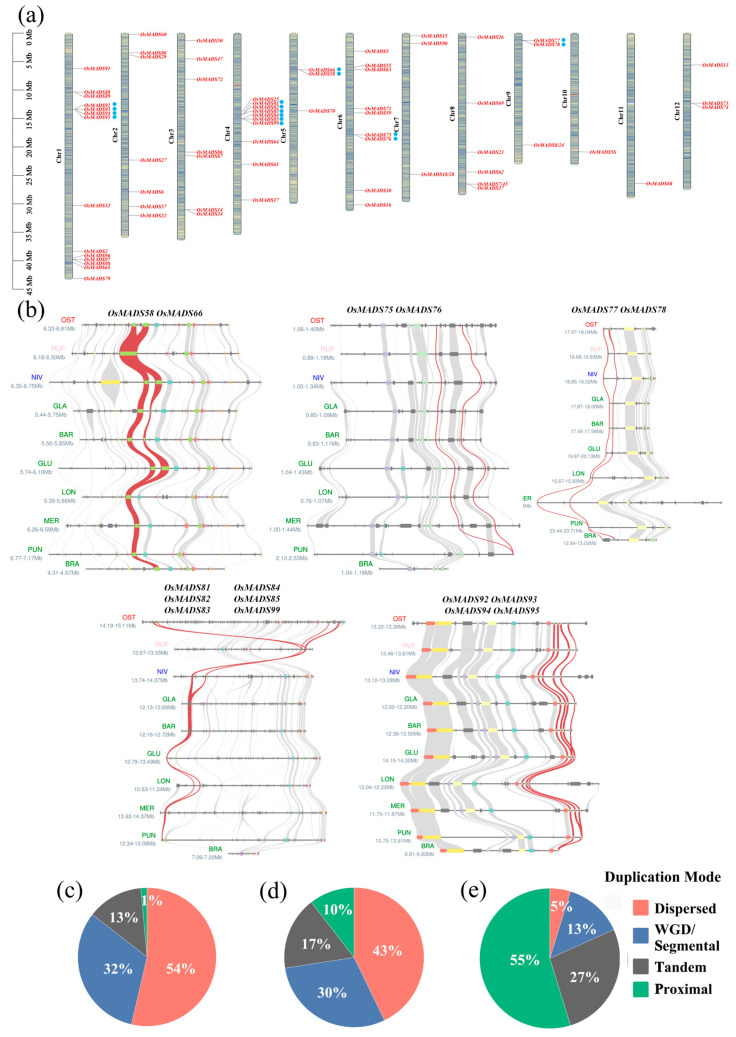
Expansion, genomic synteny, and expression patterns of *MADS-box* genes. (**a**) Chromosomal mapping of *MADS-box* genes on the twelve chromosomes of *O. sativa*, showing specific expansion of AG, Mα, Mγ, and Mβ subfamily genes. Note that the expanded genes are marked with blue dots. (**b**) Genomic synteny analysis of the expanded *MADS-box* genes across *Oryza* species. (**c**) Duplication modes of all *MADS-box* genes in the BB-genome. (**d**) Duplication modes of all *MADS-box* genes in the AA-genomes. (**e**) Duplication modes of *MADS-box* genes specifically expanded in the AA-genomes.

Our results showed that *MADS-box* genes in the AA-genome species have undergone significant expansion compared to the BB- and FF-genome species (Table S7) through the integration of chromosomal mapping (Figure 3a and Figure S3) and genomic synteny analysis (Figure 3b and Figure S4, Table S7). Phylogenetic analysis of *MADS-box* genes showed that the expanded genes in the AA-genomes were mainly Type-I *MADS-box* genes (Figure 1b). Interestingly, our results suggested that three genes from *O. longistaminata* (*OlMADS85*, *OlMADS86*, *OlMADS87*) and one gene from *O. rufipogon* (*OrMADS65*) had no genomic synteny with genes in other species, indicating that they were species-specific genes. Furthermore, 13 *MADS-box* genes (including *OgMADS31*, *OgMADS33*, *OgMADS62*, *OgMADS63*, *ObMADS31*, *ObMADS33*, *ObMADS70*, *ObMADS71*, *OuMADS3*, *OuMADS2*, *OlMADS34*, *OlMADS74*, and *OmMADS33*) clearly showed genomic synteny relationships only in the AA-genomes and confirmed that the expanded *MADS-box* genes in the AA-genomes were lineage-specific and mainly Type-I. Additionally, genomic synteny analyses showed that all *MADS-box* genes in the *O. glaberrima* genome exhibited one-to-one synteny with its wild progenitor *O. barthii*, indicating that there was no specific expansion or contraction occurred during the domestication of *O. glaberrima*, with all genes being derived from its ancestor.

In addition to patterns of the expansion described above, detailed orthologous and paralogous analyses suggested notable lineage-specific and species-specific variations across *Oryza* species (Table S8). Orthogroup OG0000000, for instance, contained six paralogs in *O. sativa* (*OsMADS81*, *OsMADS82*, *OsMADS83*, *OsMADS84*, *OsMADS85*, *OsMADS87*), while *O. punctata* and *O. brachyantha* only had two (*OpMADS17*, *OpMADS28*) and one (*OfMADS19*) orthologs, respectively. Interestingly, certain orthogroups contained genes that were either absent or exhibited no direct orthologous relationships with *O. sativa*. For example, *OsMADS23* was completely absent in *O. glaberrima* and *O. barthii*, while genes such as *OlMADS87* in *O. longistaminata* and *OrMADS65* in *O. rufipogon* lacked genomic synteny with *O. sativa*, suggesting species-specific evolution among AA-genome species. Further analyses revealed that some *MADS-box* genes maintained orthology across AA-genome species but were absent in BB- and FF-genome species, underscoring their lineage-specific expansion in AA-genome species. These genes, including *OgMADS31* and *OuMADS3*, demonstrated synteny relationships only among AA-genome species. The absence of synteny or orthology for specific *MADS-box* genes in non-AA genomes highlights the unique evolutionary trajectories of the AA-genome species.

Further analyses of the duplication modes of the specifically amplified *MADS-box* genes revealed that the expansion of *MADS-box* genes in the BB-genome species, *O. punctata*, primarily occurred through dispersed duplication and whole-genome duplication (WGD), with tandem and proximal duplications together accounting for only ~14% of all *MADS-box* genes (Figure 3c, Table S9). However, although dispersed duplication and WGD were also predominant in the AA-genome *Oryza* species, tandem and proximal duplications reached ~27% (Figure 3d), twice more than that in the BB-genome. Additional classification of the expanded *MADS-box* genes in the AA-genome *Oryza* species compared to *O. punctata* showed that proximal duplication accounted for ~55%, followed by tandem duplication, indicating that the expansion of *MADS-box* genes in the AA-genome *Oryza* species was primarily due to extensive proximal and tandem duplications (Figure 3e).

Generally speaking, a *Ka*/*Ks* ratio lower than 1 indicates that these genes have undergone purifying selection subjected to functional constraint; a *Ka*/*Ks* ratio equal to 1 suggests these genes are under neutral selection; and a *Ka*/*Ks* ratio greater than 1 suggests that these genes have undergone positive selection [63,64]. The selection pressure analyses revealed that most *MADS-box* genes were under purifying selection (0 < *Ka*/*Ks* < 1) (Table S10). However, our results indicated that six *MADS-box* genes showed signals of positive selection (*Ka*/*Ks* > 1) (Table 1). Among them, five belonged to Type-I genes, and one belonged to the SEP subgroup of Type-II. These findings indicate that the majority of *MADS-box* genes have undergone conservative evolution under functional constraints, which is consistent with their key roles in reproduction and development. However, a few genes have experienced positive selection, possibly related to environmental adaptation or other types of selection forces. Notably, Type-I genes were predominant among the six positively selected genes, while only one gene was from the SEP subgroup of Type-II, possibly reflecting the relatively flexible adaptability of Type-I genes in evolution as well as the importance of the SEP subgroup in specific evolutionary contexts. To simplify the analyses across species, we compared all the other seven AA-genome *Oryza* species to *O. sativa* due to its comprehensive and accurate annotation as a reference genome (Table S11). Our results showed that three *MADS-box* gene pairs, *OsMADS88*/*ObMADS73*, *OsMADS87*/*OnMADS75*, and *OsMADS75*/*OuMADS23*, indicated signals of positive selection (*Ka/Ks* > 1) (Table 1 and Table S11). Notably, *OsMADS88/ObMADS73* and *OsMADS87/OnMADS75* exhibited signals of positive selection detected by both within-species and across-species comparisons (Table 1 and Table S11). Additionally, *OsMADS75*/*OuMADS23*, belonging to the SEP subgroup of Type-II genes, demonstrated strong positive selection across species.

### 2.6. Expression Patterns of Specifically Amplified MADS-Box Genes

**Figure 4:**
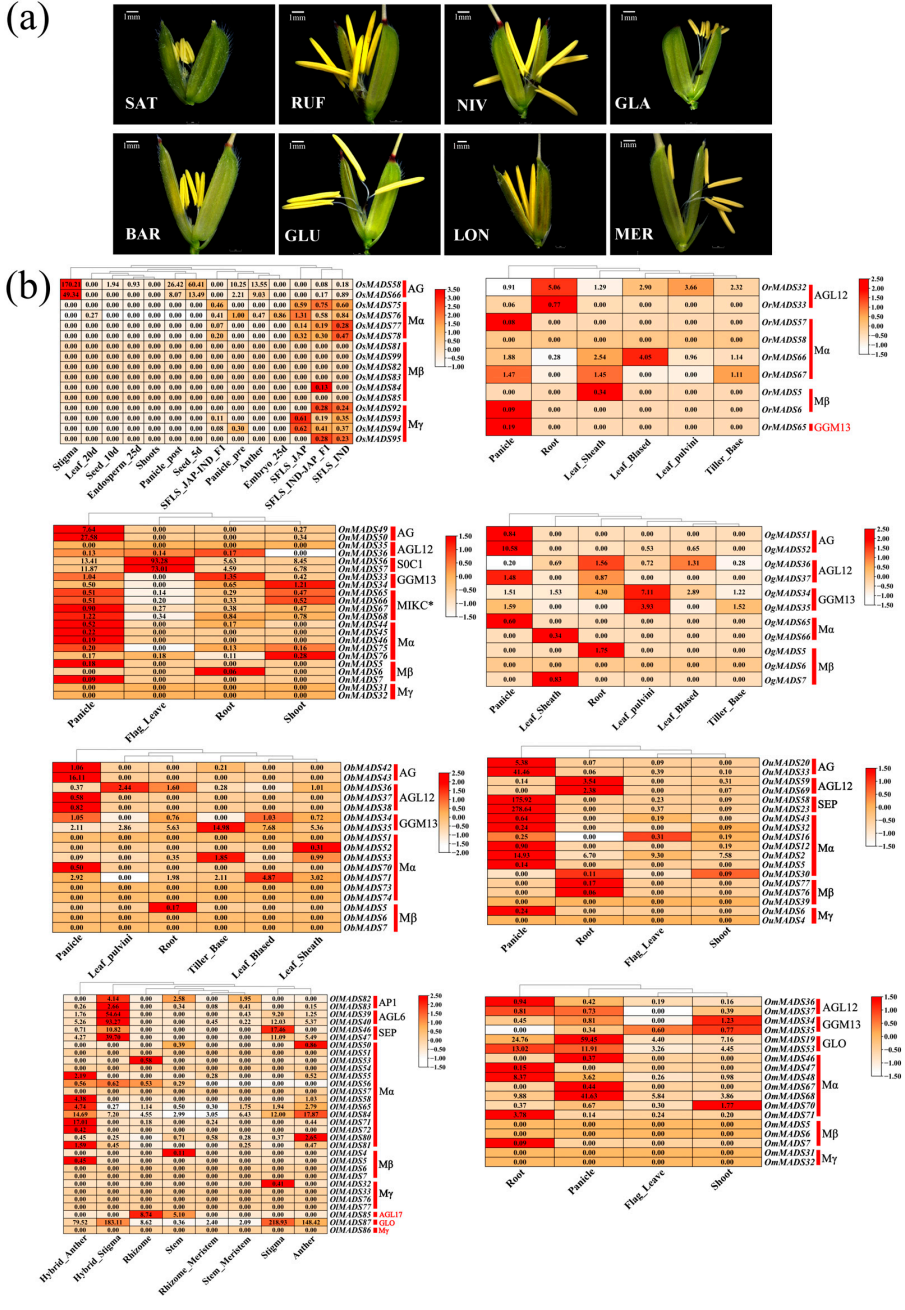
Floral organ phenotypes and expression profiling of *MADS-box* genes in different organs of the AA-genome *Oryza* species. (**a**) Phenotypic differences in floral organs of the eight AA-genome *Oryza* species, including *O. meridionalis* (MER), *O. longistaminata* (LON), *O. glumaepatula* (GLU), *O. barthii* (BAR), *O. glaberrima* (GLA), *O. nivara* (NIV), *O. rufipogon* (RUF), and *O. sativa* (SAT). The white bar represents a scale of 1 mm. (**b**) Heatmap of the expression of specifically amplified genes in the eight AA-genome *Oryza* species.

Floral traits have remarkably diversified during recent speciation of the AA-genome *Oryza* species (Figure 4a). The *MADS-box* gene family, known for its crucial role in regulating plant phenotypes, particularly floral organ traits, provides an unprecedented opportunity to illuminate gene functionality through expression patterns. To better elucidate the functions of *MADS-box* genes that have undergone specific expansion in the AA-genome *Oryza* species, we examined gene expression profiling by analyzing the transcriptomic data (Figure 4b, Figures S3 and S5). Despite the significant expansion of Type-I genes in the AA-genome species, these genes did not exhibit notable expression divergence across organs. However, the expanded Type-II *MADS-box* genes primarily occurred in the AG, AGL12, SOC1, GGM13, and MIKC* subfamilies, which were highly expressed in reproductive organs such as spikelets and stigmas. Our results showed that the gene expression patterns exhibited certain specificities across these species. The expanded AG subfamily genes showed obvious expression differences across multiple organs, particularly in spikelets and stigmas, and were notably expressed in five-day-old germinating seeds of rice. This finding suggested their potential role in reproductive organ development and early seedling growth. The SEP subfamily genes in *O. glumaepatula* exhibited high levels of expression in spikelets and stigmas, and they were expressed at the highest levels in stigmas observed in *O. longistaminata* and *O. sativa*, indicating an essential role in floral organ differentiation and functionality. The GGM13 subfamily genes were also extensively and highly expressed in *O. glaberrima*, further indicating functional divergence across these species. Further analyses of species-specific expression profiles revealed that *O. rufipogon* contained a unique *MADS-box* gene belonging to the GGM13 subfamily, which was minimally expressed only in spikelets. There were three specific genes in *O. longistaminata* belonging to the AGL17, GLO, and Mγ subfamilies, respectively, which exhibited contrasting patterns of gene expression. The AGL17 subfamily genes were highly expressed in rhizomes and stems, potentially related to the unique rhizomatous characteristics of *O. longistaminata*. The GLO subfamily genes were expressed across various organs, with notable expression in stamens and pistils, suggesting their potential role in floral organ development. The Mγ subfamily genes, however, were not expressed in any organs.

### 2.7. Functional Analysis of Duplicated Genes and Their Association with Regulatory Networks

To further investigate the relationships between the expansion of *MADS-box* genes and their functional roles in the *Oryza* AA-genome species, we conducted GO enrichment analysis on duplicated *MADS-box* genes across eight species and analyzed their interaction networks using predicted protein-protein interactions (Supplementary Figure S6). The results indicated that the expanded *MADS-box* genes in these *Oryza* species are broadly enriched in pathways related to transcriptional regulation, with “Positive regulation of transcription by RNA polymerase II” as a key pathway. Specifically, 6 of the 16 duplicated genes in *O. sativa*, 13 of 18 in *O. meridionalis*, 14 of 28 in *O. longistaminata*, 12 of 18 in *O. glumipatula*, 13 of 17 in *O. barthii*, 10 of 11 in *O. glaberrima*, 5 of 9 in *O. rufipogon*, and 10 of 22 in *O. nivara* were enriched in this pathway. These findings suggest that the duplicated *MADS-box* genes have extensive and essential roles in transcriptional regulation across various biological processes.

Further analyses revealed differences in the enrichment of duplicated genes in pathways related to floral organ development among rice and wild-related species. For example, duplicated genes in *O. meridionalis* and *O. barthii* were significantly enriched in “flower development” and “floral organ morphogenesis”, while duplicated genes in *O. glumipatula* were enriched in “specification of floral organ identity” and “specification of stamen identity”. In *O. longistaminata*, duplicated genes are predominantly enriched in “flower development” and “specification of floral organ identity”. These enrichment results indicated that *MADS-box* gene expansion plays a vital role in floral organ formation, differentiation, and specialization, potentially enhancing the formation of reproductive isolation in these AA-genome *Oryza* species.

STRING-based interaction network analysis revealed that several duplicated genes share high protein sequence similarity with key *MADS-box* genes in *O. sativa* (e.g., *MADS2*, *MADS3*, *MADS5*, and *MADS6*) and occupied critical positions within regulatory networks. These duplicated genes appear to contribute to increased regulatory diversity and precision, potentially acting in concert to regulate floral organ development. In contrast, duplicated genes in *O. rufipogon* and *O. nivara* were primarily enriched in transcriptional regulation and showed fewer enrichments in pathways related to floral organ development, suggesting that their functional differentiation in floral organ regulation may be relatively limited.

## 3. Discussion

The *MADS-box* gene family, divided into Type-I and Type-II based on protein structure, plays a central role in plant development by regulating floral organ identity, controlling flowering time, and responding to both abiotic and biotic stresses [6,65,66,67]. Type-II genes are more extensively characterized due to their critical role in defining floral organ identity, while Type-I genes are involved in broader developmental and adaptive processes [6,68]. In this study, we highlight the expansion of *MADS-box* genes in AA-genome *Oryza* species, which offer essential raw materials for reproductive success and adaptive evolution. Prior research has shown that *MADS-box* genes play significant roles in adaptive radiation and speciation across various plant lineages [69,70]. Unlike previous studies that primarily focused on Asian cultivated rice or several *Oryza* species [8,60], we comprehensively conducted a comparative analysis across all eight AA-genome species and relatives. We presented novel insights into the specific mechanisms driving the expansion of these genes in the AA-genome *Oryza* species, particularly through gene duplication and functional diversification, contributing to reproductive success and environmental adaptability.

Gene duplication and subsequent neofunctionalization are key processes driving the evolution of genome complexity and adaptability in plants [71]. Our results showed a significant expansion of Type-I *MADS-box* genes in AA-genome species, likely driven by the need for increased genetic redundancy and subfunctionalization of reproductive systems to adapt to diverse environmental pressures [72,73]. These expansions may have facilitated the acquisition of novel reproductive traits, contributing to the evolutionary success and ecological versatility of *Oryza* species. This large-scale investigation enabled us to reveal the predominant role of proximal and tandem duplications in driving the expansion of the *MADS-box* gene family, providing novel insights into the evolution of this important gene family during recent speciation. Our findings revealed that proximal and tandem duplications are the predominant mechanisms driving *MADS-box* gene expansion in AA-genome *Oryza* species, in line with previous studies [74,75]. These duplication events have not only increased genetic diversity but also generated species-specific *MADS-box* genes, contributing to functional diversity and species-specific adaptations [76,77]. Such expansions have likely played a pivotal role in the adaptive evolution of these species, enabling them to thrive in diverse ecological niches in pan-tropical regions of the Earth.

Positive selection detected in several Type-I *MADS-box* genes further underscores their critical role in enabling species to adapt to specific ecological niches [78,79,80]. These selective pressures, likely driven by both biotic and abiotic stresses, have promoted functional divergence in these genes, allowing for the enhancement of traits essential for survival under environmental challenges [81]. In this study, some Type-I *MADS-box* genes exhibited signals of positive selection detected not only within species but also across the AA-genome species. For example, *OsMADS88*/*ObMADS73* and *OsMADS87*/*OnMADS75* under strong positive selection highlight their roles in driving adaptive evolution. Importantly, *OsMADS87*, a Type-I Mγ gene known to regulate seed size and thermosensitivity [53], was identified as a key player under positive selection. This finding aligns with its crucial role in reproduction and stress responses, further emphasizing its importance in adaptive evolution. *OsMADS75*/*OuMADS23* were under strong positive selection, suggesting their pivotal role in reproductive specialization and environmental adaptation. These genes enriched in pathways related to transcriptional regulation and reproductive development, likely support species-specific adaptations in response to environmental pressures, contributing to reproductive success and resilience in fluctuating climates. The identification of positively selected genes suggests potential targets for rice improvement strategies aimed at increasing stress resilience and productivity in fluctuating climates [8,82,83,84,85,86,87,88]. Accordingly, our findings indicate that selection signals detected in Type-I genes could be leveraged in rice breeding programs to enhance germplasm exploration in the AA-genome *Oryza* species.

Expression of *MADS-box* genes in this study indicated that their expansion, particularly Type-II genes, may be intricately linked to the development of reproductive organs in AA-genome *Oryza* species. The high expression of AG subfamily genes in reproductive organs, including panicles and stigmas, across various *Oryza* species underscores their pivotal role in floral organ identity and development [68,89]. This pattern of expression divergence highlights how the expanded Type-II genes contribute to floral structure diversification, a critical factor in the reproductive success and ecological adaptation of these species [90,91,92,93]. Moreover, several *MADS-box* genes were expressed in non-floral organs, such as leaves and roots, suggesting broader functional roles beyond reproduction. For example, SOC1 subfamily members integrate environmental signals to regulate developmental transitions, including flowering time, which demonstrates the multifunctionality of *MADS-box* genes in plant adaptability [94,95,96]. Collectively, the specifically amplified *MADS-box* genes in the AA-genome *Oryza* species revealed complex expression patterns, largely varying across species and organs. Type-I genes generally exhibited low levels of expression or no expression, while the expanded subfamilies of Type-II genes displayed high levels of expression in reproductive organs, indicating that they function in the reproductive and developmental processes.

In this study, duplicated *MADS-box* genes in AA-genome *Oryza* species, particularly *O. meridionalis*, *O. glumipatula*, and *O. barthii*, showed significant functional enrichment in floral organ development pathways, with protein similarities to key *MADS-box* genes in *O. sativa*, such as *MADS2*, *MADS5*, and *MADS6*. These similarities suggest that the duplicated genes might contribute to a more nuanced regulatory landscape, potentially enhancing adaptability in floral morphogenesis [6,7,67]. In contrast, *O. rufipogon*, *O. nivara*, and *O. sativa* displayed fewer enrichments in floral regulatory pathways, suggesting a more conserved role in transcriptional regulation. This divergence highlights how gene duplication events in specific lineages may foster increased complexity and diversification in regulatory networks, directly impacting species-specific floral traits. Such findings highlight the adaptive potentials of gene duplications in response to evolutionary pressures and support a broad role of *MADS-box* genes in the development and diversification of reproductive organs during speciation [73,97].

In conclusion, our comprehensive analysis of the evolutionary dynamics and expression divergence of the *MADS-box* gene family across AA-genome *Oryza* species provides new insights into how they have facilitated recent plant speciation. Our findings, particularly the expansion of Type-I genes through tandem and proximal duplications, coupled with positive selection and species-specific expression patterns, highlight the role of *MADS-box* genes in driving functional diversification, reproductive success, and environmental adaptability. Comprehensive GO enrichment and interaction network analyses further underscored the pivotal role of *MADS-box* gene expansion in reproductive organ development in the AA-genome *Oryza* species. Thus, these duplicated genes likely play essential roles in the regulation of floral organ development and may have facilitated their adaptation to diverse environments. It is our hope that these results will form a strong foundation for future research and potential applications in rice breeding programs aimed at enhancing stress resilience and agricultural sustainability.

## 4. Materials and Methods

### 4.1. Data Collection and Plant Materials

This study was conducted using high-quality reference genomes of *Oryza* species. The genomes of *O. sativa* subgroups Temperate *Japonica*, Subtropical *Japonica*, Tropical *Japonica*, *Aus*, *Aromatic*, and *Indica*, as well as *O. nivara*, *O. glaberrima*, *O. barthii*, *O. glumaepatula*, *O. meridionalis*, *O. punctata*, and *O. brachyantha*, were downloaded from the Gramene *ORYZA* release 8 databases (https://oryza-ensembl.gramene.org/index.html) accessed on 10 September 2023. The *O. sativa* MSU7 genome was downloaded from RGAP (Rice Genome Annotation Project Database, http://rice.uga.edu/) [98,99] accessed on 10 September 2023. The *O. rufipogon* genome data file was obtained from NCBI (National Center for Biotechnology Information, https://www.ncbi.nlm.nih.gov/) under the accession number PRJCA002346 [58] accessed on 10 September 2023, while the genome data for *O. longistaminata* were downloaded from the *O. longistaminata* Information Resource (http://olinfres.nig.ac.jp/) [100] accessed on 10 September 2023. The expression matrix for *O. sativa* was retrieved from the RGAP (Rice Genome Annotation Project Database, http://rice.uga.edu/) accessed on 5 May 2024. This matrix was generated from a curated set of 80 mRNA-seq libraries available in the NCBI Sequence Read Archive (SRA). Transcript abundances were quantified using Kallisto (v0.51.0) and reported as Transcripts Per Million (TPM) for each gene model. Detailed information about the mRNA-seq libraries and the RNA-seq Coverage tracks aligned to the genome assembly can be accessed via the Genome Browser on the RGAP website. The transcriptome data for *O. rufipogon*, *O. nivara*, *O. glaberrima*, *O. barthii*, *O. glumaepatula*, *O. meridionalis*, and *O. longistaminata* were downloaded from NCBI (National Center for Biotechnology Information, https://www.ncbi.nlm.nih.gov/) under project numbers PRJNA478144, PRJNA264484, PRJNA264496, SRP011058, and PRJNA264483 [60,101] accessed on 5 May 2024, respectively.

All *Oryza* materials used in this study were grown between September 2020 and September 2021 at the experimental station of South China Agricultural University. For *O. sativa*, a 6 × 4 plot arrangement was employed, maintaining both row and plant spacing at 20 cm, following standard field management practices. For wild *Oryza* species, a planting configuration with 60 cm between rows and 80 cm between plants was employed, with each wild rice plant individually isolated using open-ended polyvinyl chloride (PVC) tubes.

### 4.2. Identification of MADS-Box Family Genes

MADS-box proteins for *O. sativa* and *A. thaliana* were obtained from the Plant Transcription Factor Database v3.0 (http://plntfdb.bio.uni-potsdam.de/v3.0/) accessed on 15 September 2023 and the Phytozome 13 database (http://www.phytozome.net) accessed on 15 September 2023. To identify conserved domains within MADS-box proteins, we downloaded hidden Markov model (HMM) profiles for the SRF-type transcription factor (PF00319) and K-box region (PF01486) from the Pfam 37.0 database (http://pfam.xfam.org/) accessed on 15 September 2023. We then performed HMM searches on the protein sequences of 15 *Oryza* species using HMMER version 2.3.2 (http://hmmer.janelia.org/) [102] with specific parameters: --cut_tc to set trusted cutoff values and --domtblout to specify output files. Using PF00319 and PF01486 as search models, we filtered results to retain significant domain matches based on an E-value threshold of 1 × 10^−5^, ensuring statistical significance. Next, we utilized the makedb command in the BLAST software [103] package (blastn: 2.10.0+) to create a protein sequence database. Using MADS-box protein sequences from *A. thaliana* and *O. sativa* as query sequences, we conducted a blastp search across the protein databases of 15 different *Oryza* genomes with an E-value threshold of 1 × 10^−5^. The BLAST results were further filtered based on sequence identity, retaining only matches with over 30% identity. Gene IDs obtained from both HMMER and BLAST searches were merged and deduplicated to yield a list of candidate *MADS-box* gene IDs. Subsequently, the protein sequences of the identified candidate *MADS-box* genes were extracted from the *Oryza* protein sequences and saved in FASTA format. These sequences were then uploaded to the NCBI’s CD-search (Conserved Domains search) [104] and the SMART database (http://smart.embl.de/) [105] to predict conserved domains. From the resulting data, we manually selected sequences containing the SRF domain and MADS superfamily domain for further analysis and validation. Finally, the identified MADS-box protein sequences were submitted to the ExPASy platform (https://www.expasy.org/) [106], where the ProtParam tool (https://web.expasy.org/protparam/, accessed on 20 September 2023) [107] was used to compute physicochemical properties, including amino acids number, isoelectric point (pI), and molecular weight (MWs). Additionally, the hydrophobicity profiles of these proteins were analyzed using ProtScale (https://web.expasy.org/protscale/, accessed on 20 September 2023) [106]. Subcellular localization of the proteins was predicted using the Plant-mPLoc tool (http://www.csbio.sjtu.edu.cn/bioinf/plant-multi/, accessed on 20 September 2023) [108].

### 4.3. Multiple Sequence Alignment and Phylogenetic Tree Construction

Multiple sequence alignment of MADS-box proteins was performed using MAFFT (v7.475) [109] with the --localpair mode for local alignment and the --maxiterate 1000 parameter to ensure high accuracy. Following alignment, TrimAl (v1.4.rev15) [110] was used to process the alignment file, generating a PHYLIP-formatted output suitable for phylogenetic analysis. The parameter -phylip_paml was used to format the alignment, and sequences were filtered using -gt 0.6 to retain only those with more than 60% similarity to the majority of sequences. For maximum likelihood tree construction, FastTree (v2.1.10) [111] was employed with the default JTT model, and the output was saved in Newick format. Tree visualization was enhanced using Chiplot (https://www.chiplot.online/, accessed on 12 January 2025) [112].

### 4.4. Identification of Conserved Motifs in Protein Sequences of MADS-Box Gene Family and Analysis of Cis-Acting Regulatory Elements

To identify conserved motifs within the MADS-box protein sequences of *Oryza* species, the MEME Suite (https://meme-suite.org/meme/tools/meme), accessed on 20 September 2023 [113] was used to detect motifs across all MADS-box protein sequences obtained in this study. The MEME parameters were configured as follows: minimum motif width set to 6 amino acids, maximum width to 200, and the number of motifs to 10, with all other settings at default values. The motif prediction results from MEME and NCBI CD-search were visualized using the Gene Structure View (Advanced) tool in TBtools v2.075 [114]. For the analysis of cis-regulatory elements, the 2000 bp upstream sequences of each gene were extracted as putative promoter regions using seqkit v2.0.0 [115] with the --up-stream 2000 parameter, based on GTF annotations converted from GFF3 files using gffread-0.12.7 [116]. Gene IDs in these sequences were then standardized with awk to ensure consistency. The extracted sequences were uploaded to the PlantCARE database (http://bioinformatics.psb.ugent.be/webtools/plantcare/html/), accessed on 20 September 2023 [117] for cis-acting regulatory element analysis.

### 4.5. Chromosomal Location, Duplication Mode, and Molecular Evolutionary Analysis of MADS-Box Genes

The MCScanX toolkit (University of Georgia, Athens, GA, USA) [118] was used to analyze gene duplication events and identify homologous chromosomal regions. For both intra-species and genome-wide comparisons, global BLAST was performed with an E-value threshold of less than 1 × 10^−10^, retaining the top five results for each query. To enhance efficiency, the Diamond alignment tool (v2.08) [119] was used as an alternative to BLAST, maintaining the same E-value threshold and top-five result retention. Visualization of chromosomal positions, gene duplication events, and collinearity relationships was achieved using the Advanced Circos program in TBtools (v2.075) [114]. Positive selection analysis was conducted by identifying orthologous gene pairs, with *Ka*/*Ks* ratios calculated using the PAML software package (v4.9i) [120] and KaKs_Calculator (version 3.0) [121], ensuring robust assessment of evolutionary rates. For collinearity analysis among *Oryza* species, the MCScan (Python version) toolkit [118] was employed. CDS, protein, and GFF3 files were processed and converted into BED format using JCVI’s format conversion tools. Collinearity analysis identified orthologous genes and detected collinear blocks with a designated reference species. The resulting collinear blocks were merged and refined, and target genes with their flanking regions were extracted for visualization using JCVI’s synteny visualization module, with a custom layout file defining species order and color coding.

### 4.6. Gene Expression Analysis

HISAT2.2.0 [122] was employed to align reads to the reference genome, with index construction utilizing exon and splice site information extracted from the annotated GTF file. The alignment process was executed with two threads, and strand-specificity was set to reverse forward (RF) for the RNA-seq data. FeatureCounts 1.6.2 [123] was used for quantifying exon reads, specifying gene identifiers as the attribute and employing a custom script for normalization by Transcripts Per Million (TPM). The results were subsequently merged to compile gene expression data for downstream differential expression analysis. This workflow ensured a systematic and reproducible approach to RNA-seq data processing, enhancing the accuracy of transcript quantification and subsequent analysis. Gene expression heatmaps were generated using TBtools v2.075 [114] with Log2 (TPM + 1) scale [114].

### 4.7. Protein Similarity and GO Enrichment Analysis

Duplicated MADS-box protein sequences for each of the AA-genome *Oryza* species were analyzed in STRING (https://string-db.org/, accessed on 4 November 2024) using *Oryza sativa* as the reference species. STRING was used to identify the best-matching orthologous proteins and perform GO enrichment analysis for Biological Processes, followed by interaction network construction.

## 5. Conclusions

This study provides a comprehensive analysis of the *MADS-box* gene family in the AA-genome *Oryza* species by examining fifteen high-quality genomes, including diverse genome types and subspecies of *O. sativa*. We identified a total of 1093 *MADS-box* genes, revealing general trends of gene amplification from FF-genomes to AA-genomes. Phylogenetic analysis uncovered two primary groups, with Type-I genes experiencing significant expansion predominantly through proximal and tandem duplications, while Type-II genes remained relatively stable. This distinction indicates divergent evolutionary paths between Type-I and Type-II genes. Most of the identified *MADS-box* genes were found to be under purifying selection, reflecting functional conservation, while a smaller subset exhibited positive selection signals, suggesting adaptive significance. Furthermore, interaction network analysis highlighted that several duplicated genes occupy critical positions in regulatory pathways for floral development, aligning with their high expression in reproductive organs. These findings underscore the role of *MADS-box* genes in reproductive system variation and recent speciation events in AA-genome *Oryza* species.

## Figures and Tables

**Table 1:** Estimation of *Ka*/*Ks* values exceeding one of *MADS-box* duplicated gene pairs in the genus *Oryza*.

Duplicated Gene Pairs	*Ka*	*Ks*	*Ka*/*Ks*	Duplication Mode	Subfamily
*OlMADS65/OlMADS84*	0.00983233	0.0070629	1.39211	SD	Mα
*OuMADS58/OuMADS23*	0.0330073	0.0264149	1.24957	TD	SEP
*OstMADS32/OstMADS33*	0.0169867	0.0134423	1.26367	SD	Mγ
*OssMADS28/OssMADS30*	0.00924201	0.00764212	1.20935	Proximal	Mγ
*OsrMADS31/OsrMADS28*	0.0448498	0.0336014	1.33476	SD	Mγ
*OsaMADS30/OsaMADS27*	0.0545886	0.0290186	1.88116	Proximal	Mγ

## Data Availability

No new data were created within the article.

## Supplementary Materials

The following supporting information can be downloaded at: https://www.mdpi.com/article/10.3390/plants14030379/s1. Supplementary Figure S1: Gene structure of *MADS-box* gene: (a) gene structure of Type-I *MADS-box* gene, (b) gene structure of Type-Ⅱ *MADS-box* gene; Supplementary Figure S2: Distribution of cis-regulatory elements in *MADS-box* genes: (a) proportion of cis-acting elements in different genome types, (b) bar chart of proportion of cis-acting elements in different genome types; Supplementary Figure S3: Chromosomal distribution and collinear gene synteny heatmaps of the *MADS-box* gene family across AA-genome *Oryza* species; Supplementary Figure S4: Synteny map of *MADS-box* genes in AA-genome *Oryza* species; Supplementary Figure S5: Expression heatmap of *MADS-box* genes in AA-genome *Oryza* species; Supplementary Figure S6: GO enrichment and interaction network analysis of *MADS-box* duplicated genes in AA-genome *Oryza* species. Each page shows GO enrichment analysis and STRING-based interaction network for duplicated *MADS-box* genes in eight AA-genome *Oryza* species. Duplicated genes are marked in red within the interaction network. Network edges represent interaction types. Known interactions (blue for curated databases and purple for experimental data), predicted interactions (dark green for gene neighborhood, red for gene fusions, and dark blue for gene co-occurrence), and other interactions (light green for text mining, black for co-expression, and cyan for protein homology) are presented; Table S1: Detailed information on the 15 genomes used in this study; Table S2: *MADS-box* genes in *Oryza* genus; Table S3: Basic information of *MADS-box* genes and their proteins in *Oryza* genus; Table S4: Gene structure analysis of *MADS-box* genes in *Oryza*; Table S5: Cis-regulatory element analysis of *MADS-box* genes in *Oryza*; Table S6: Chromosomal location of *MADS-box* genes in the *Oryza* genus; Table S7: *Oryza* AA-genome *MADS-box* gene collinearity analysis; Table S8: Cross-species analysis of *MADS-Box* genes using Orthofinder and MCscan to identify orthologs and paralogs in *Oryza* species; Table S9: Duplication modes of *MADS-box* genes in the 10 *Oryza* species; Table S10: Intraspecies gene duplication modes and evolutionary analysis of *MADS-box* genes in *Oryza* species; Table S11: Evolutionary analysis of *MADS-Box* gene pairs across AA-genome *Oryza* species.
