# Supplementary material for: Evolutionary Dynamics and Expression Divergence of the MADS-Box Gene Family During Recent Speciation of AA-Genome Oryza Species
Source: Plants (Basel). 2025 Jan 26;14(3):379. doi: 10.3390/plants14030379 (PMC11820988; doi:10.3390/plants14030379)
Supplement: Supplementary file 1 [file plants-14-00379-s001.zip › Supplementary Figure S2.pdf]

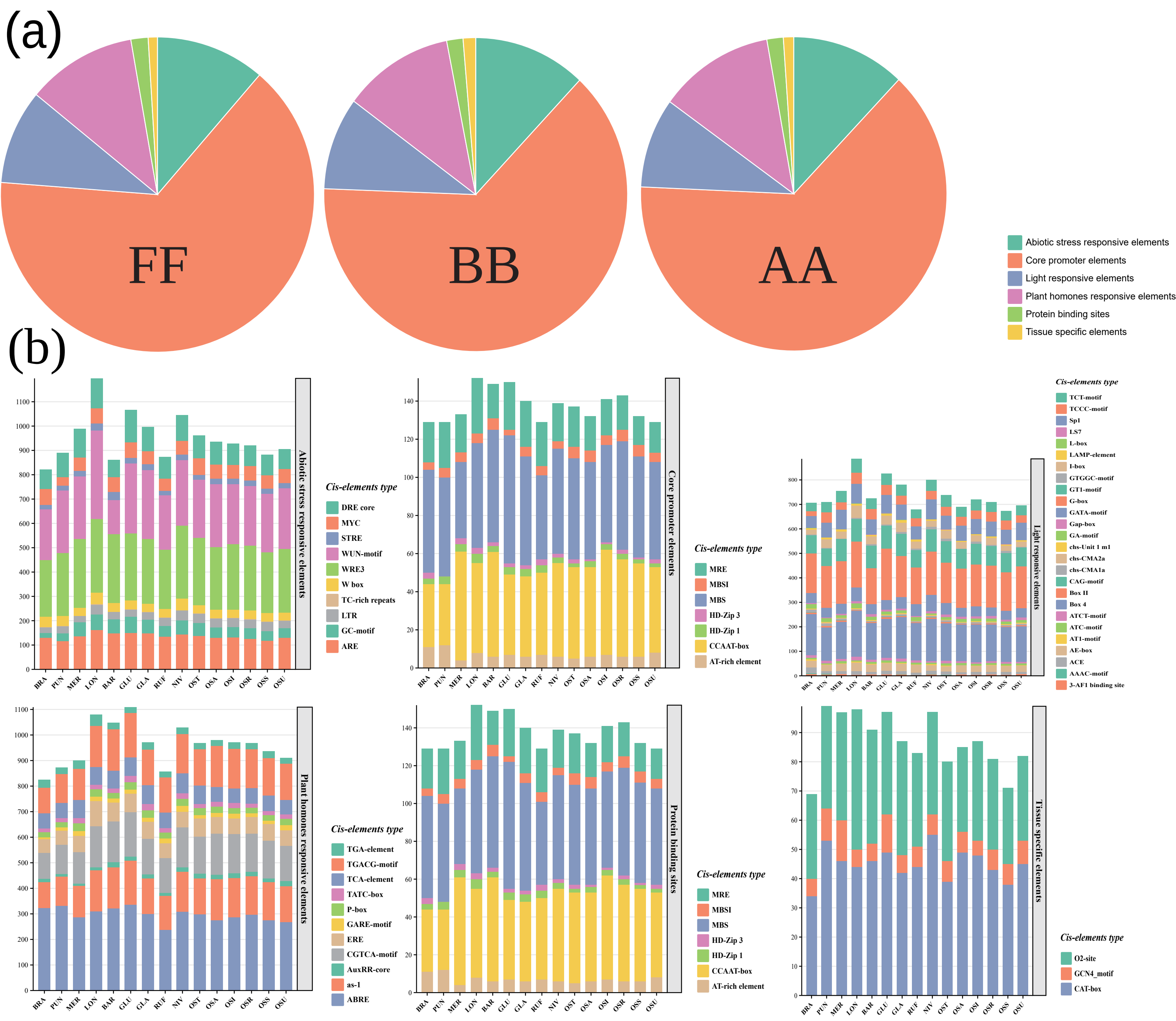

(a)
FF
BB
AA
Abiotic stress responsive elements
Core promoter elements
Light responsive elements
Plant homones responsive elements
Protein binding sites
Tissue specific elements
(b)
Abiotic stress responsive elements
Cis-elements type
DRE core
MYC
STRE
WUN-motif
WRE3
W box
TC-rich repeats
LTR
GC-motif
ARE
Core promoter elements
Cis-elements type
MRE
MBSI
MBS
HD-Zip 3
HD-Zip 1
CCAAT-box
AT-rich element
Light responsive elements
Cis-elements type
TCT-motif
TCCC-motif
Sp1
LS7
L-box
LAMP-element
I-box
GTGGC-motif
GT1-motif
G-box
GATA-motif
Gap-box
GA-motif
chs-Unit 1 m1
chs-CMA2a
chs-CMA1a
CAG-motif
Box II
Box 4
ATCT-motif
ATC-motif
AT1-motif
AE-box
ACE
AAAC-motif
3-AF1 binding site
Plant homones responsive elements
Cis-elements type
TGA-element
TGACG-motif
TCA-element
TATC-box
P-box
GARE-motif
ERE
CGTCA-motif
AuxRR-core
as-1
ABRE
Protein binding sites
Cis-elements type
MRE
MBSI
MBS
HD-Zip 3
HD-Zip 1
CCAAT-box
AT-rich element
Tissue specific elements
Cis-elements type
O2-site
GCN4_motif
CAT-box
BRA PUN MER LON BAR GLU GLA RUF NIV OST OSA OSI OSR OSS OSU
