# Supplementary material for: Evolutionary Dynamics and Expression Divergence of the MADS-Box Gene Family During Recent Speciation of AA-Genome Oryza Species
Source: Plants (Basel). 2025 Jan 26;14(3):379. doi: 10.3390/plants14030379 (PMC11820988; doi:10.3390/plants14030379)
Supplement: Supplementary file 1 [file plants-14-00379-s001.zip › Supplementary Figure S4.pdf]

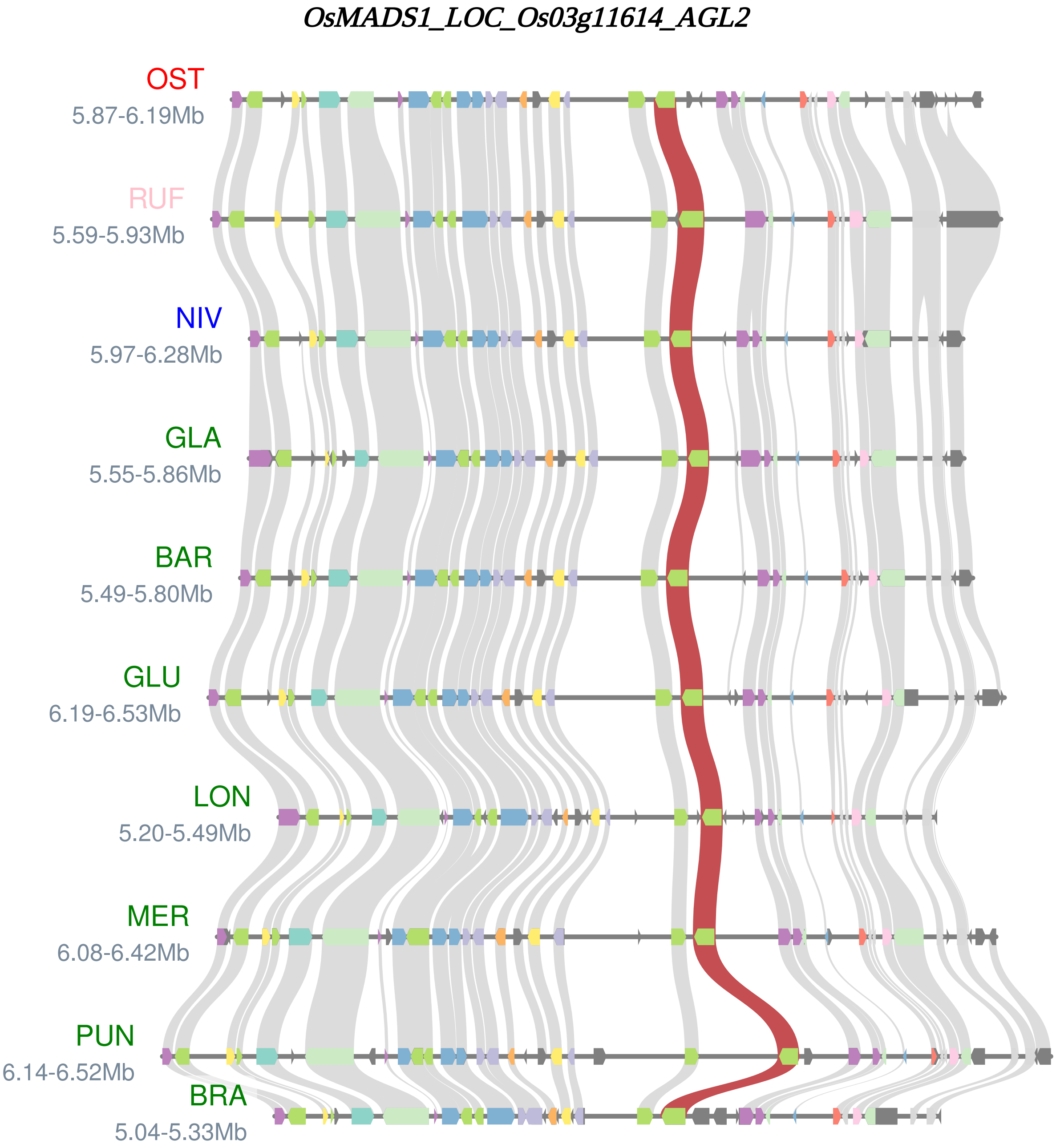
OsMADS1_LOC_Os03g11614_AGL2
OST
5.87-6.19Mb
RUF
5.59-5.93Mb
NIV
5.97-6.28Mb
GLA
5.55-5.86Mb
BAR
5.49-5.80Mb
GLU
6.19-6.53Mb
LON
5.20-5.49Mb
MER
6.08-6.42Mb
PUN
6.14-6.52Mb
BRA
5.04-5.33Mb

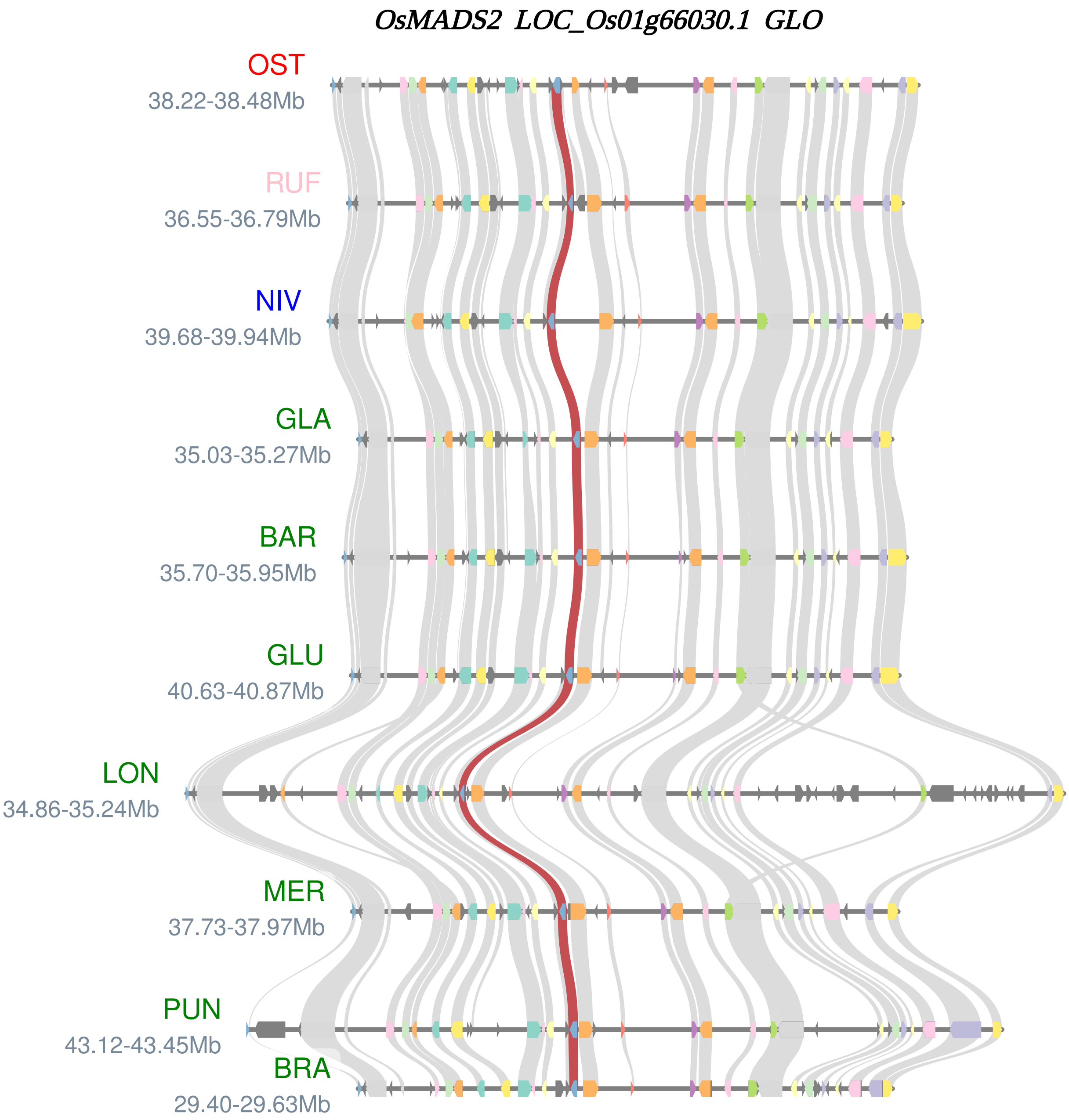
OsMADS2 LOC_Os01g66030.1 GLO
OST
38.22-38.48Mb
RUF
36.55-36.79Mb
NIV
39.68-39.94Mb
GLA
35.03-35.27Mb
BAR
35.70-35.95Mb
GLU
40.63-40.87Mb
LON
34.86-35.24Mb
MER
37.73-37.97Mb
PUN
43.12-43.45Mb
BRA
29.40-29.63Mb

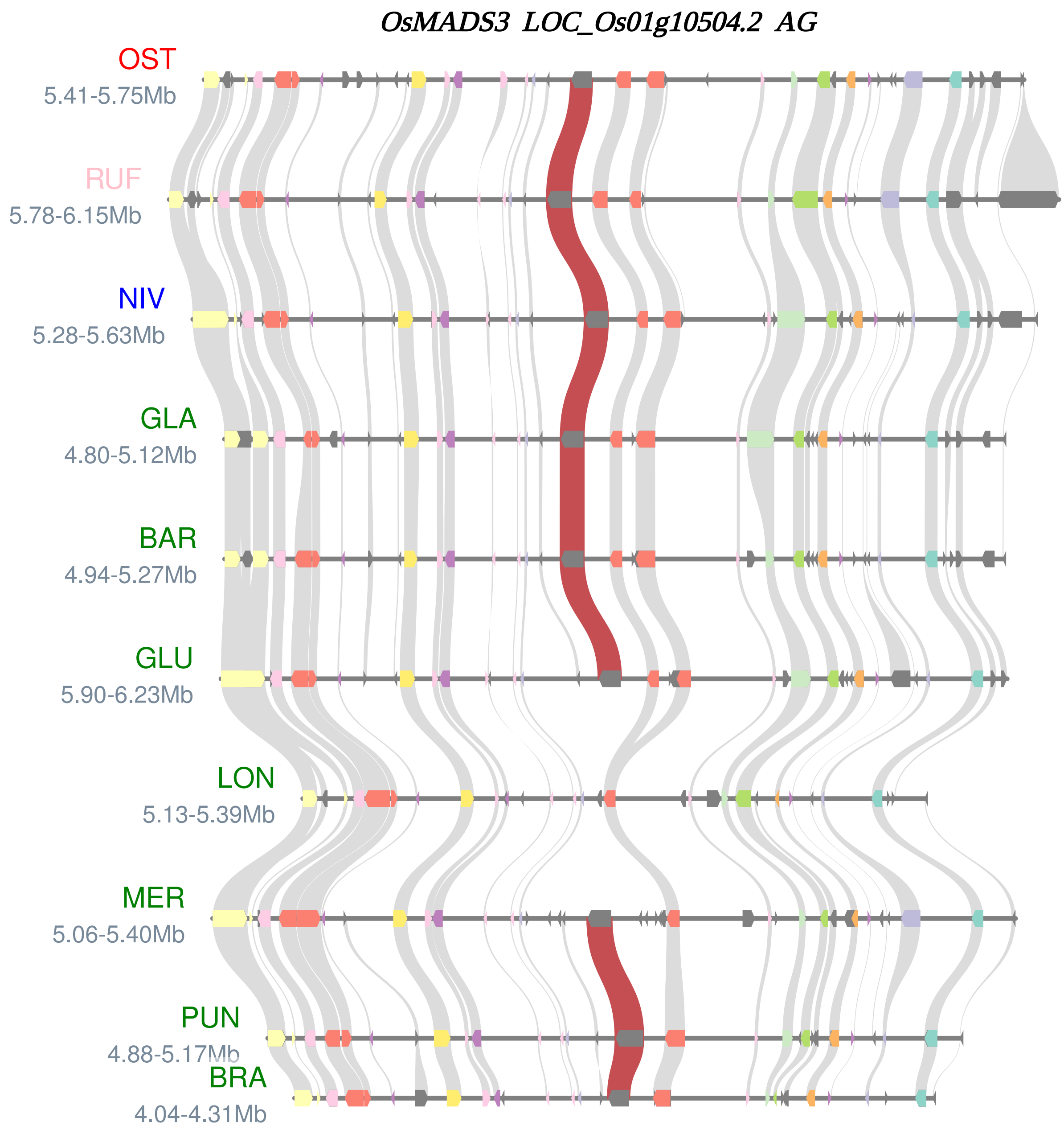
OsMADS3 LOC_Os01g10504.2 AG
OST
5.41-5.75Mb
RUF
5.78-6.15Mb
NIV
5.28-5.63Mb
GLA
4.80-5.12Mb
BAR
4.94-5.27Mb
GLU
5.90-6.23Mb
LON
5.13-5.39Mb
MER
5.06-5.40Mb
PUN
4.88-5.17Mb
BRA
4.04-4.31Mb

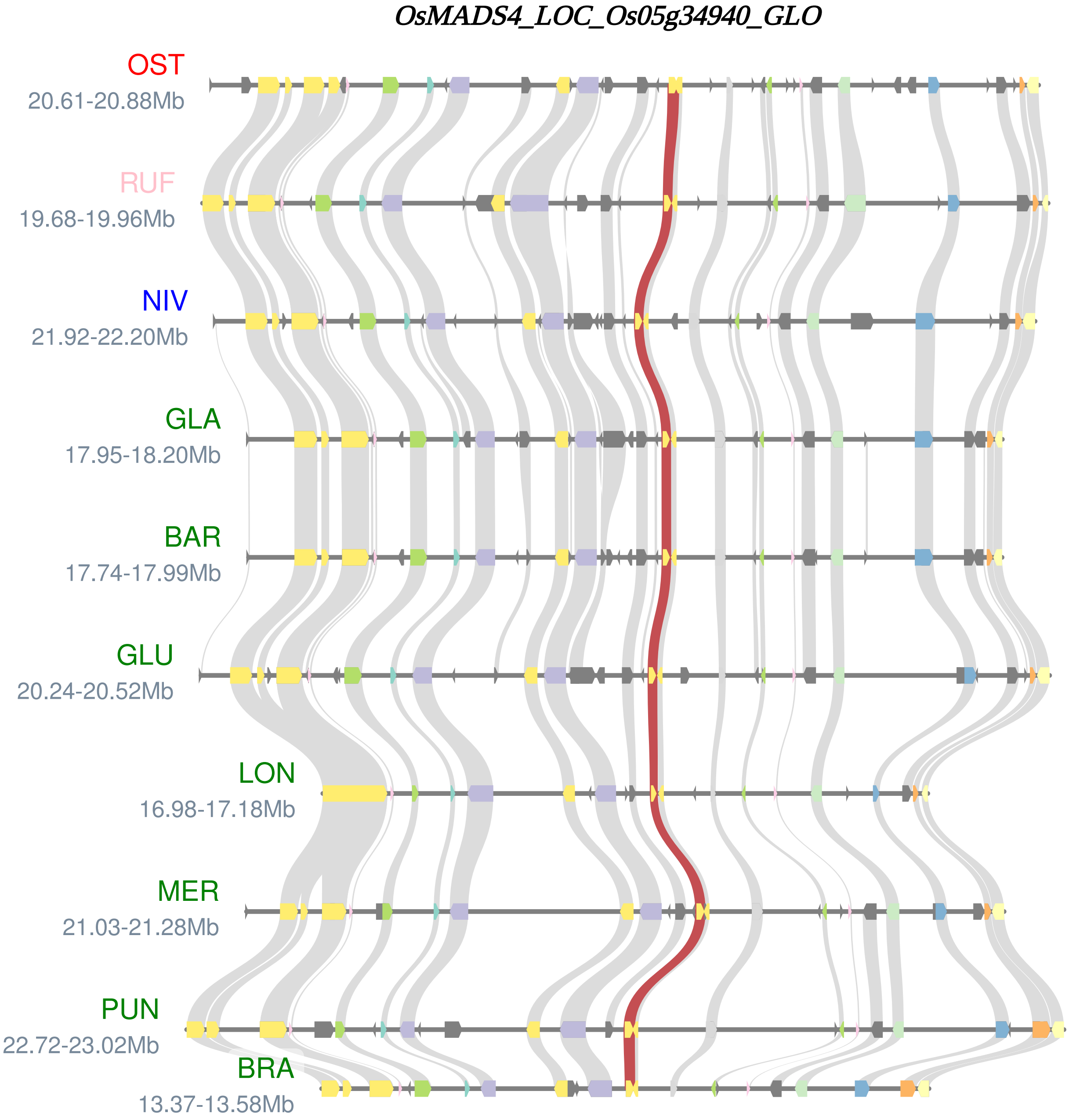
OsMADS4_LOC_Os05g34940_GLO
OST
20.61-20.88Mb
RUF
19.68-19.96Mb
NIV
21.92-22.20Mb
GLA
17.95-18.20Mb
BAR
17.74-17.99Mb
GLU
20.24-20.52Mb
LON
16.98-17.18Mb
MER
21.03-21.28Mb
PUN
22.72-23.02Mb
BRA
13.37-13.58Mb

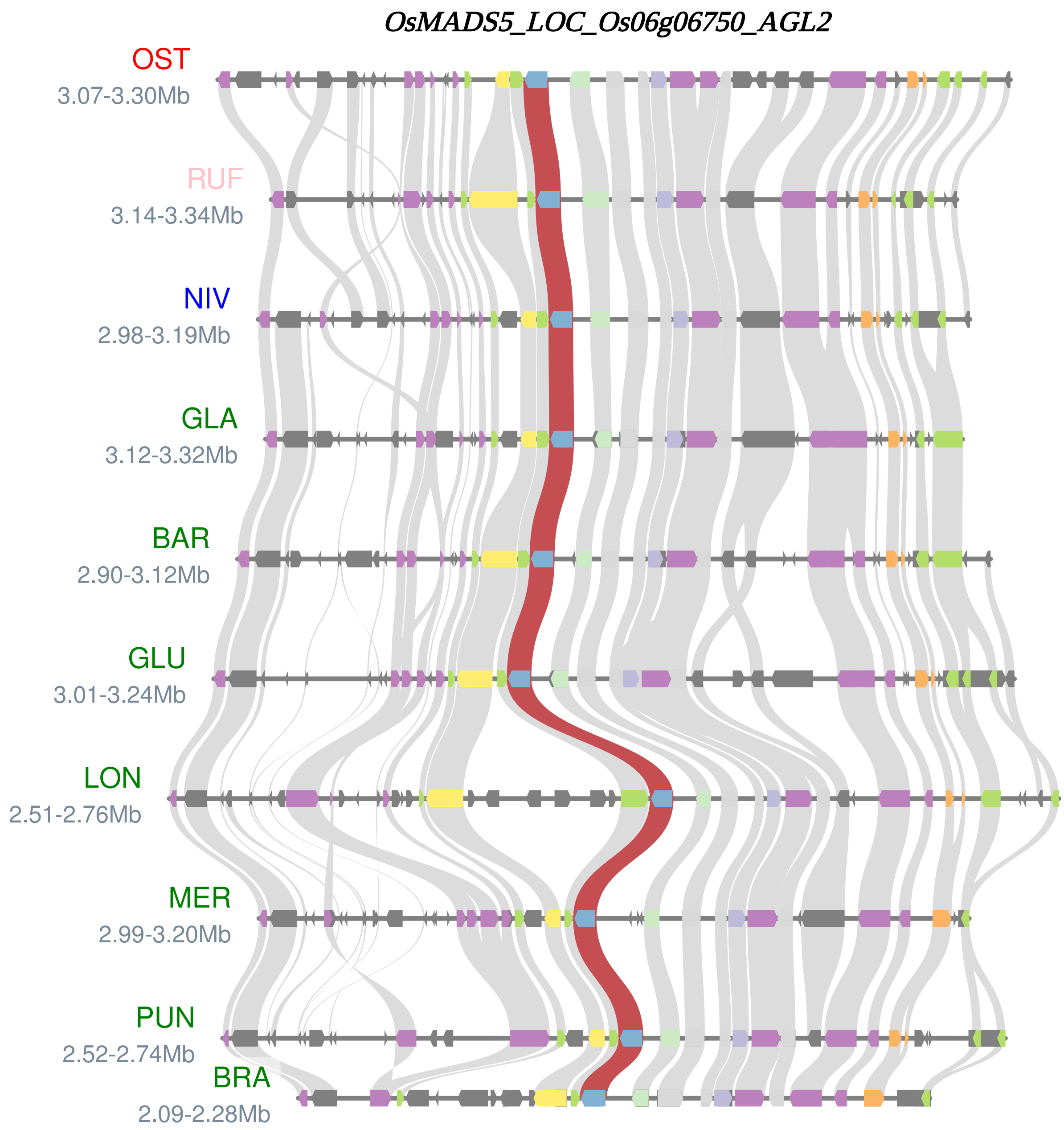

OsMADS5_LOC_Os06g06750_AGL2
OST
3.07-3.30Mb
RUF
3.14-3.34Mb
NIV
2.98-3.19Mb
GLA
3.12-3.32Mb
BAR
2.90-3.12Mb
GLU
3.01-3.24Mb
LON
2.51-2.76Mb
MER
2.99-3.20Mb
PUN
2.52-2.74Mb
BRA
2.09-2.28Mb

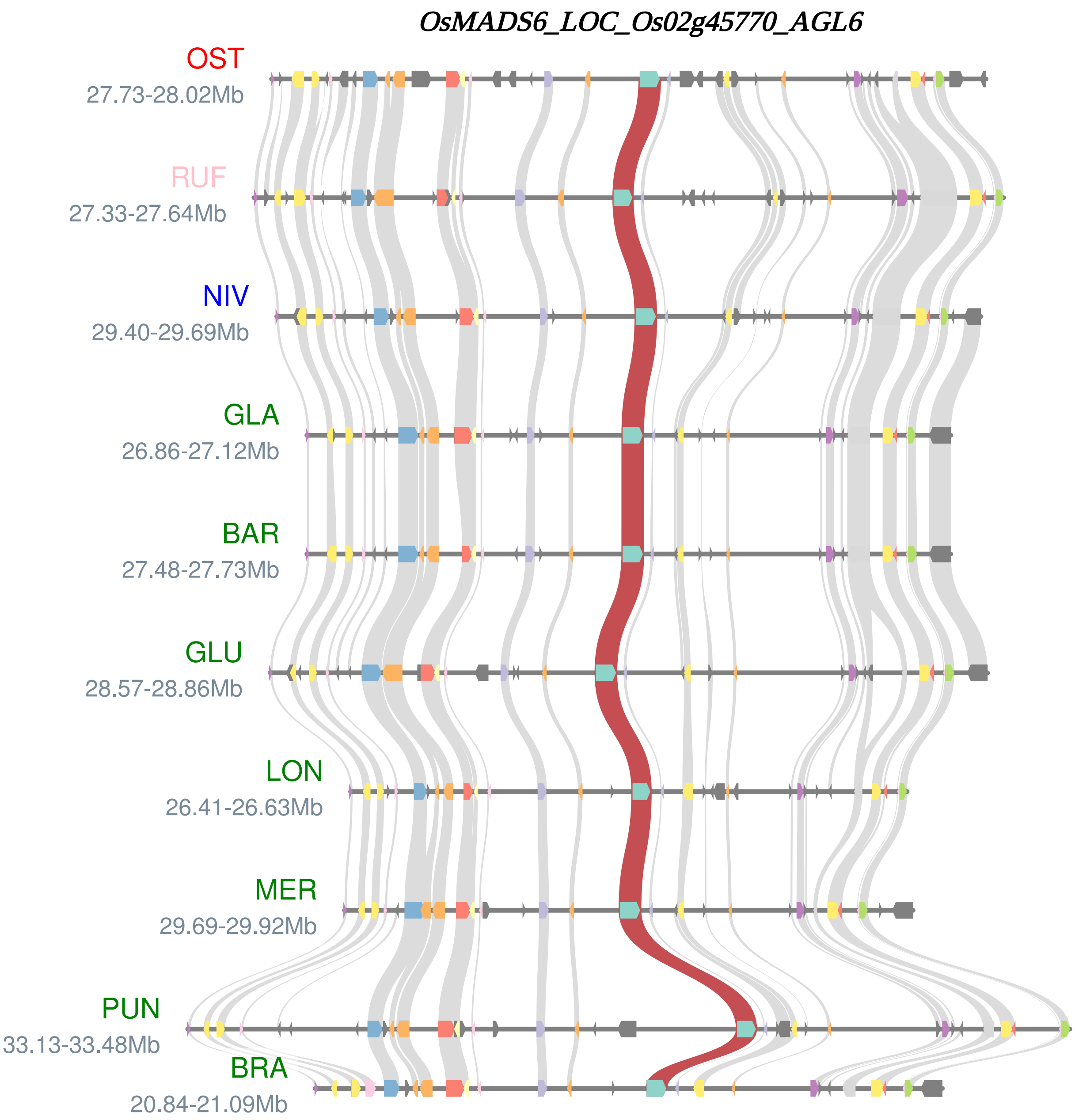
OsMADS6_LOC_Os02g45770_AGL6
OST
27.73-28.02Mb
RUF
27.33-27.64Mb
NIV
29.40-29.69Mb
GLA
26.86-27.12Mb
BAR
27.48-27.73Mb
GLU
28.57-28.86Mb
LON
26.41-26.63Mb
MER
29.69-29.92Mb
PUN
33.13-33.48Mb
BRA
20.84-21.09Mb

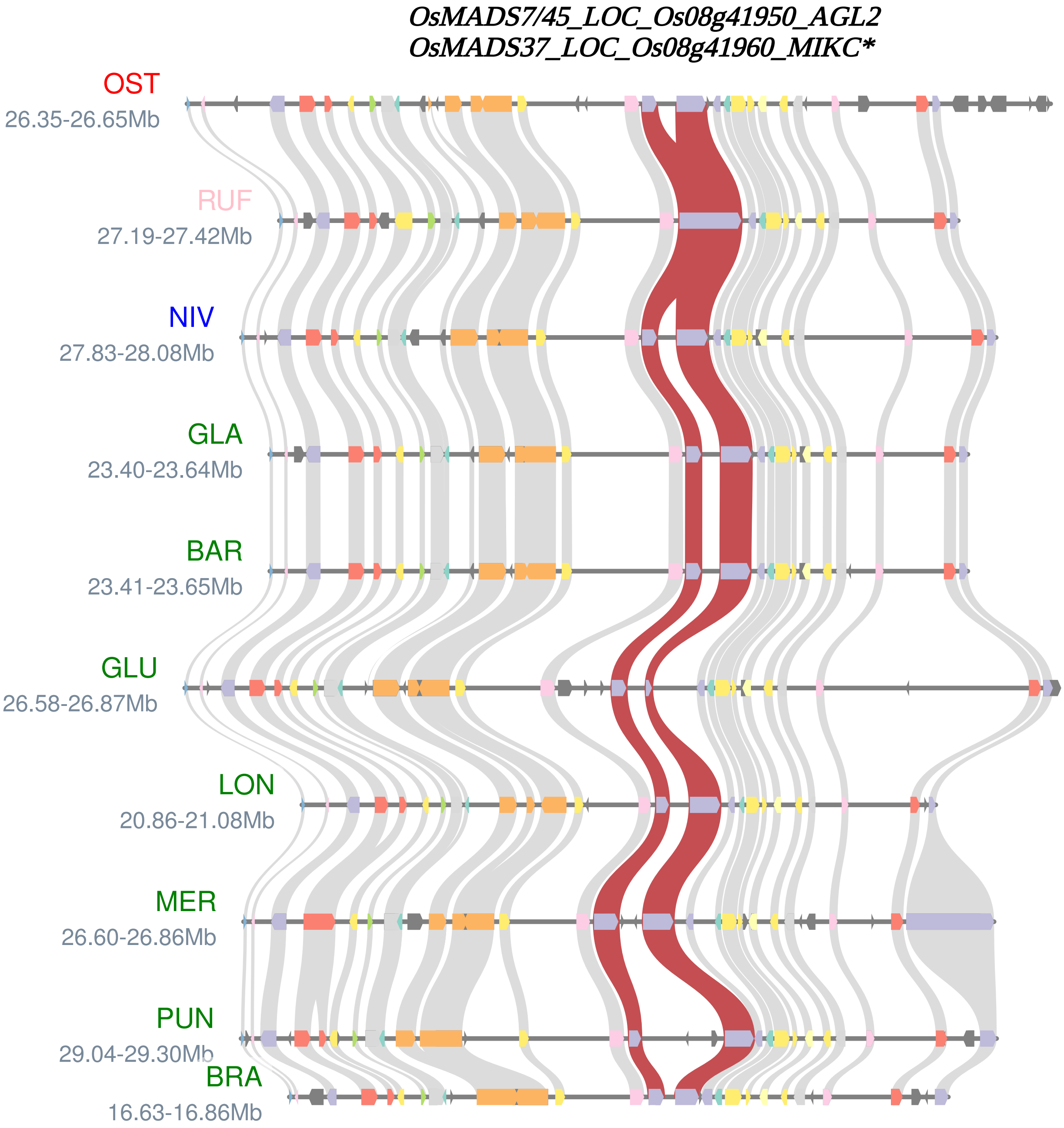
OsMADS7/45_LOC_Os08g41950_AGL2
OsMADS37_LOC_Os08g41960_MIKC*
OST
26.35-26.65Mb
RUF
27.19-27.42Mb
NIV
27.83-28.08Mb
GLA
23.40-23.64Mb
BAR
23.41-23.65Mb
GLU
26.58-26.87Mb
LON
20.86-21.08Mb
MER
26.60-26.86Mb
PUN
29.04-29.30Mb
BRA
16.63-16.86Mb

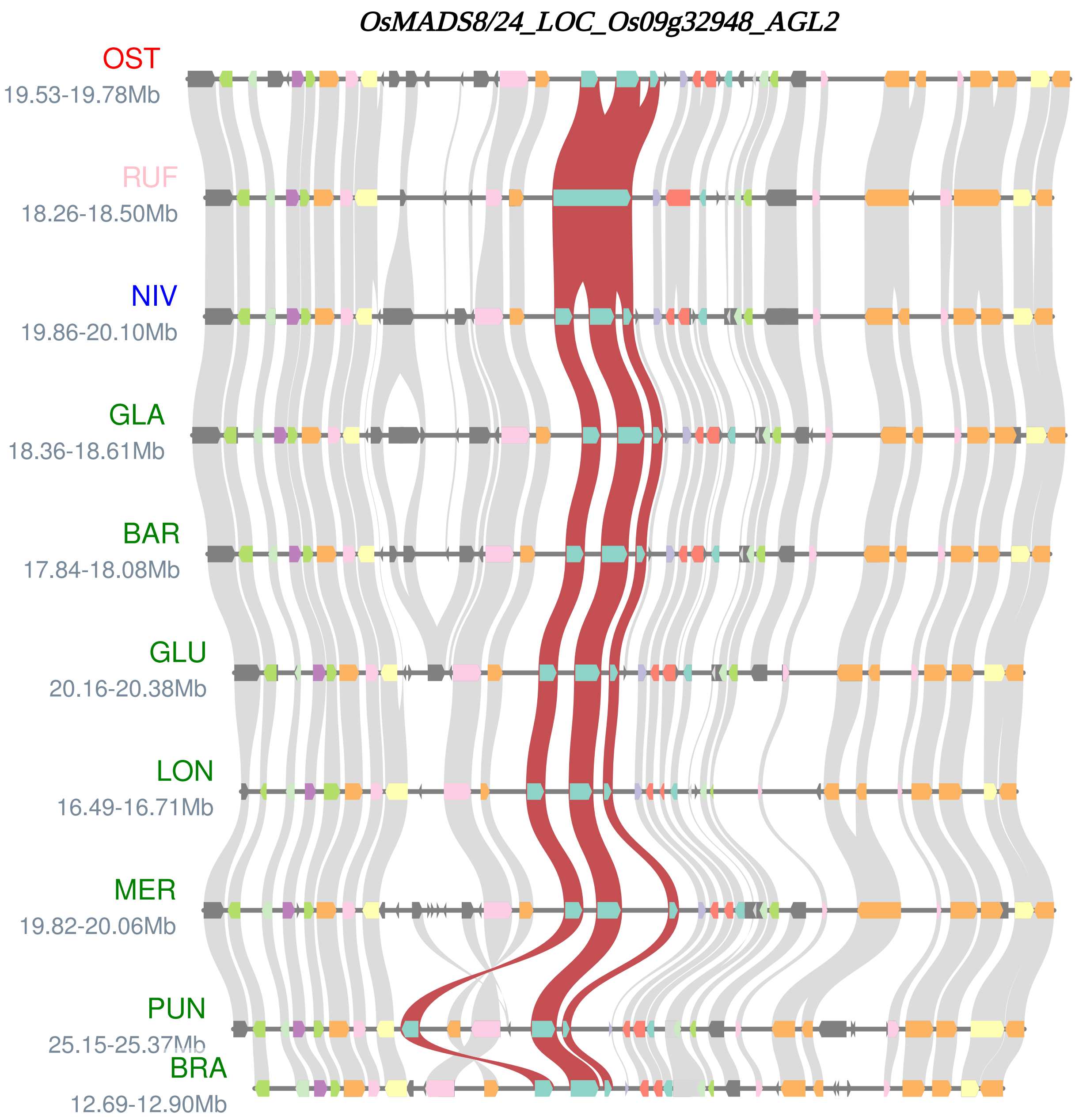
OsMADS8/24_LOC_Os09g32948_AGL2
OST
19.53-19.78Mb
RUF
18.26-18.50Mb
NIV
19.86-20.10Mb
GLA
18.36-18.61Mb
BAR
17.84-18.08Mb
GLU
20.16-20.38Mb
LON
16.49-16.71Mb
MER
19.82-20.06Mb
PUN
25.15-25.37Mb
BRA
12.69-12.90Mb

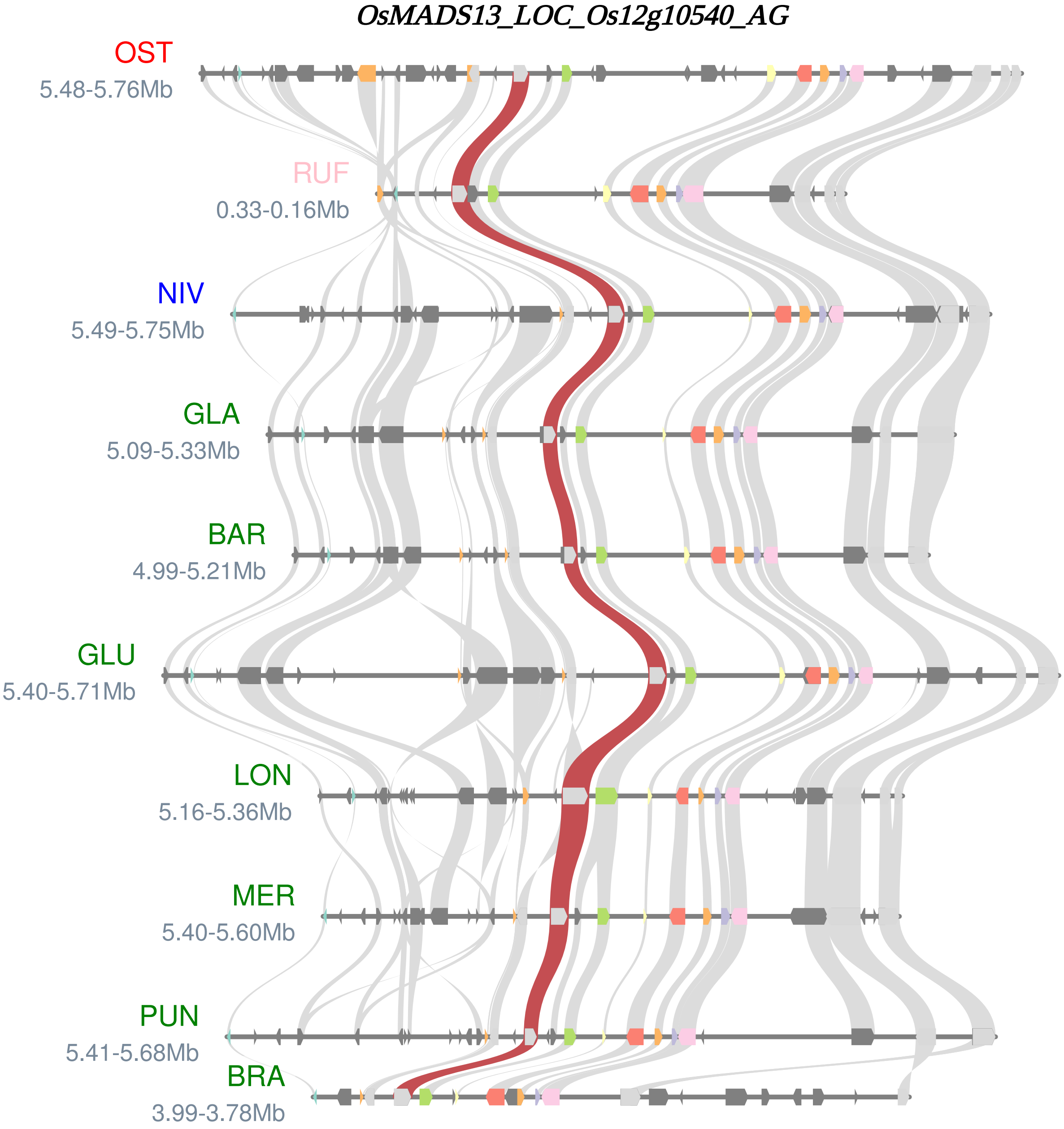

OsMADS13_LOC_Os12g10540_AG
OST
5.48-5.76Mb
RUF
0.33-0.16Mb
NIV
5.49-5.75Mb
GLA
5.09-5.33Mb
BAR
4.99-5.21Mb
GLU
5.40-5.71Mb
LON
5.16-5.36Mb
MER
5.40-5.60Mb
PUN
5.41-5.68Mb
BRA
3.99-3.78Mb

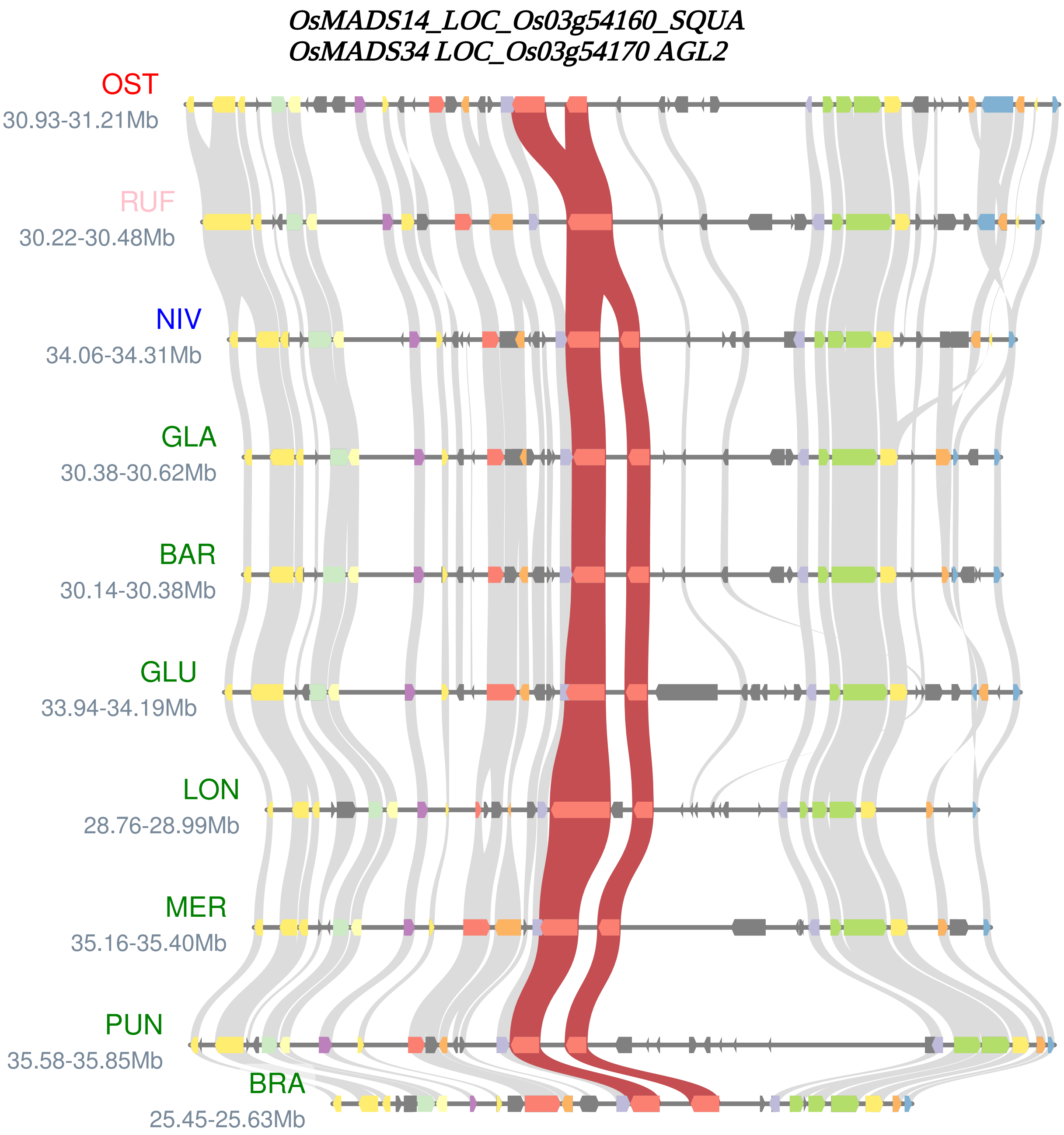
OsMADS14_LOC_Os03g54160_SQUA
OsMADS34 LOC_Os03g54170 AGL2
OST
30.93-31.21Mb
RUF
30.22-30.48Mb
NIV
34.06-34.31Mb
GLA
30.38-30.62Mb
BAR
30.14-30.38Mb
GLU
33.94-34.19Mb
LON
28.76-28.99Mb
MER
35.16-35.40Mb
PUN
35.58-35.85Mb
BRA
25.45-25.63Mb

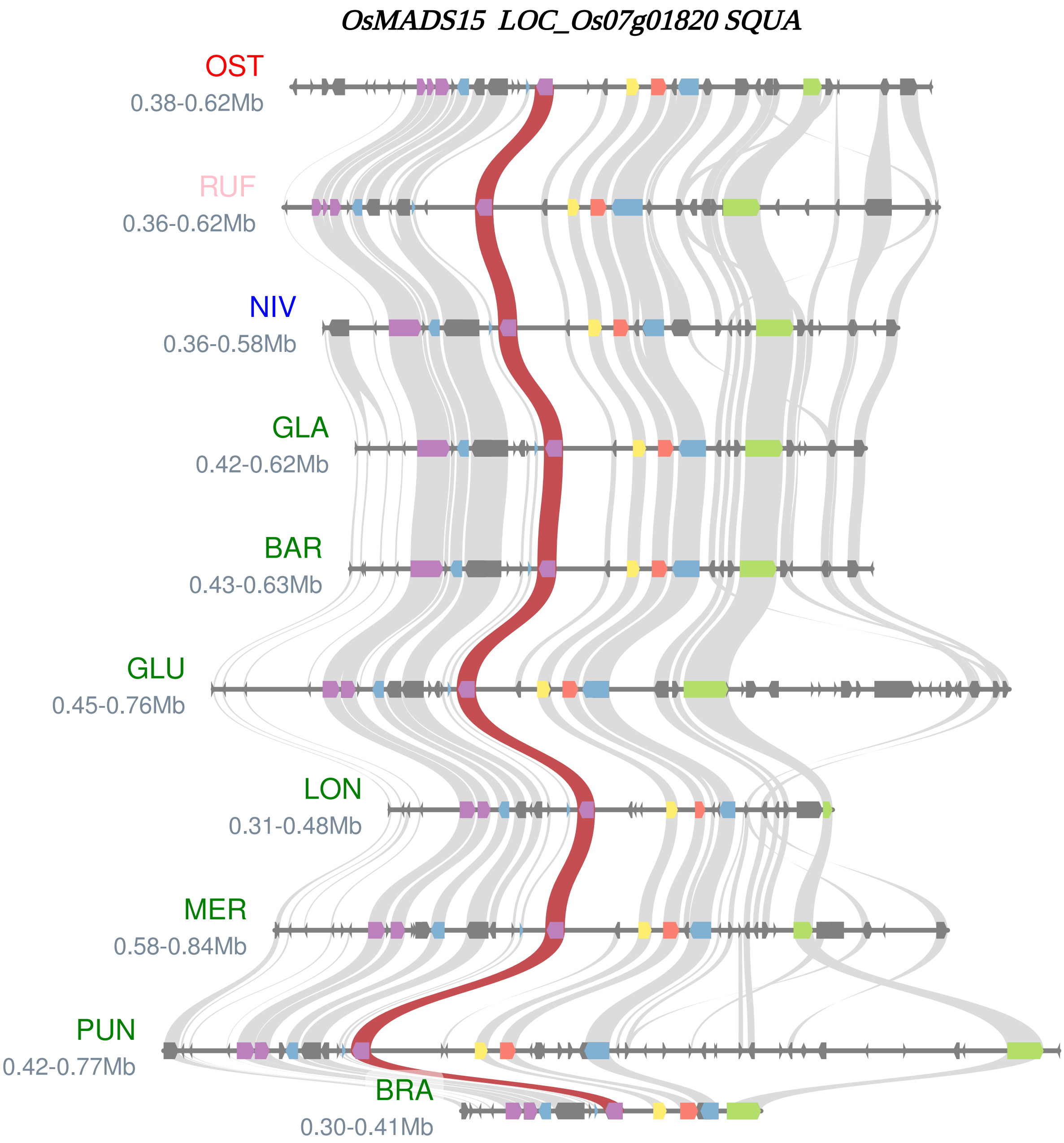

OsMADS15 LOC_Os07g01820 SQUA
OST
0.38-0.62Mb
RUF
0.36-0.62Mb
NIV
0.36-0.58Mb
GLA
0.42-0.62Mb
BAR
0.43-0.63Mb
GLU
0.45-0.76Mb
LON
0.31-0.48Mb
MER
0.58-0.84Mb
PUN
0.42-0.77Mb
BRA
0.30-0.41Mb

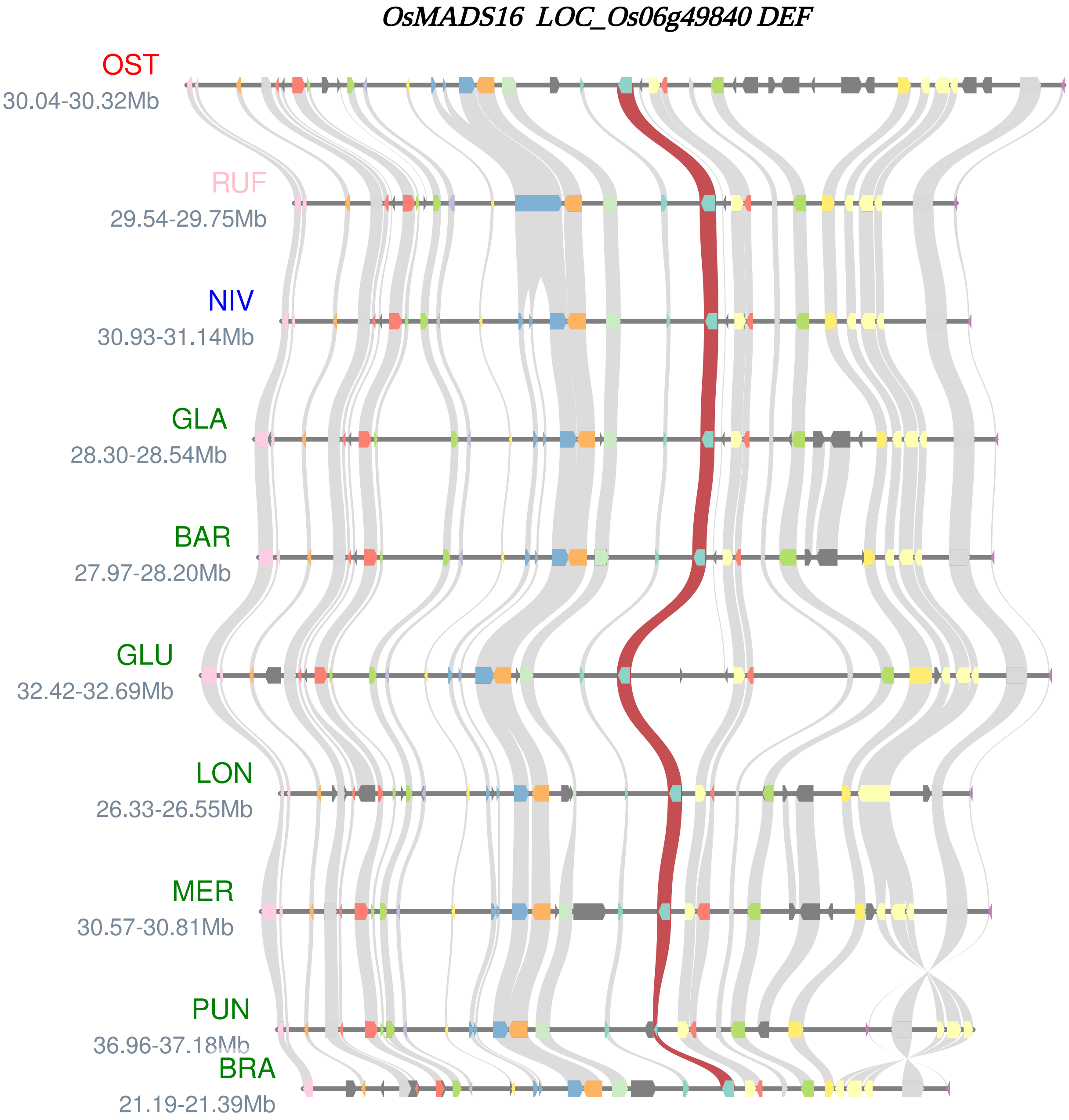
OsMADS16 LOC_Os06g49840 DEF
OST
30.04-30.32Mb
RUF
29.54-29.75Mb
NIV
30.93-31.14Mb
GLA
28.30-28.54Mb
BAR
27.97-28.20Mb
GLU
32.42-32.69Mb
LON
26.33-26.55Mb
MER
30.57-30.81Mb
PUN
36.96-37.18Mb
BRA
21.19-21.39Mb

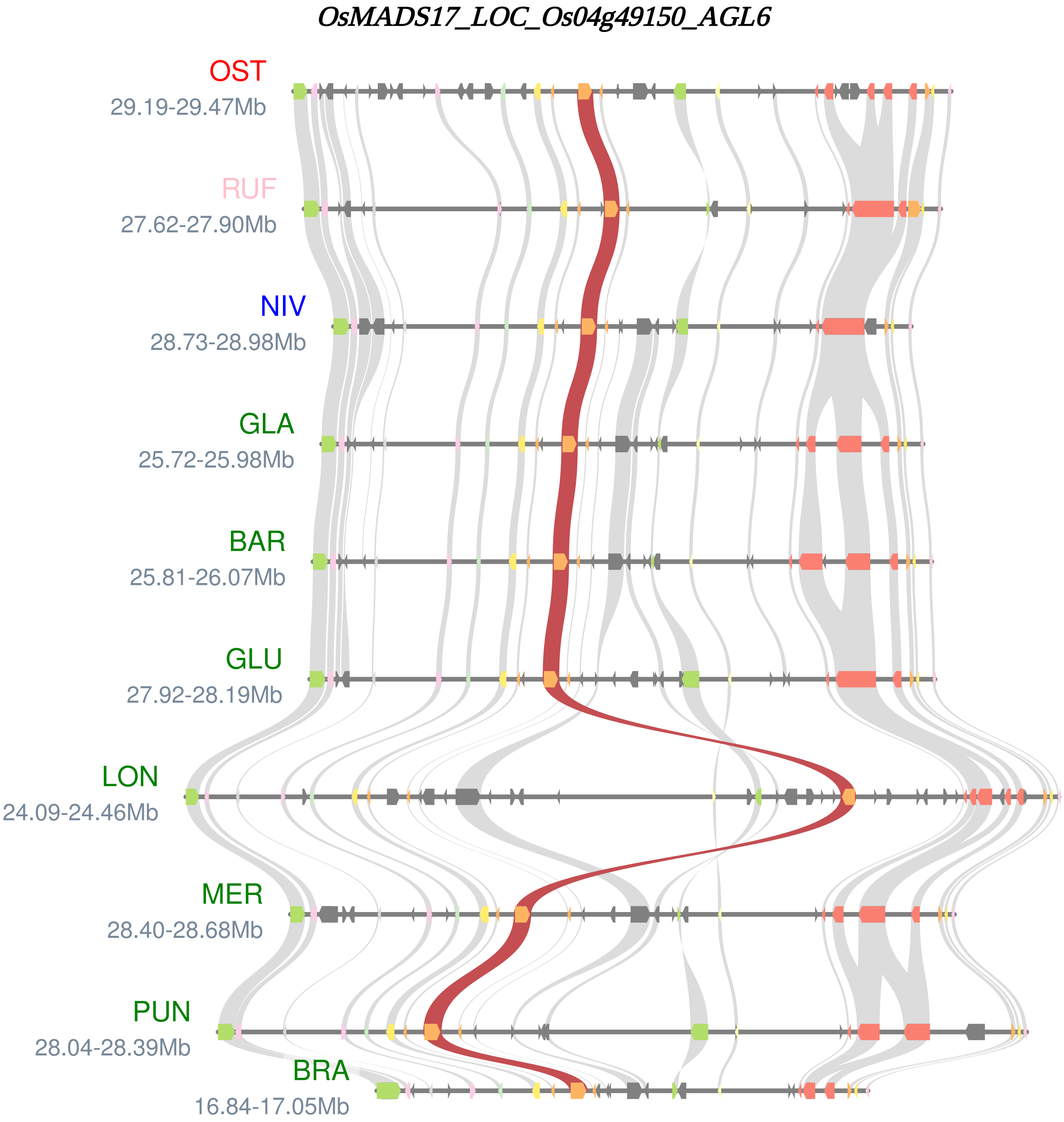
OsMADS17_LOC_Os04g49150_AGL6
OST
29.19-29.47Mb
RUF
27.62-27.90Mb
NIV
28.73-28.98Mb
GLA
25.72-25.98Mb
BAR
25.81-26.07Mb
GLU
27.92-28.19Mb
LON
24.09-24.46Mb
MER
28.40-28.68Mb
PUN
28.04-28.39Mb
BRA
16.84-17.05Mb

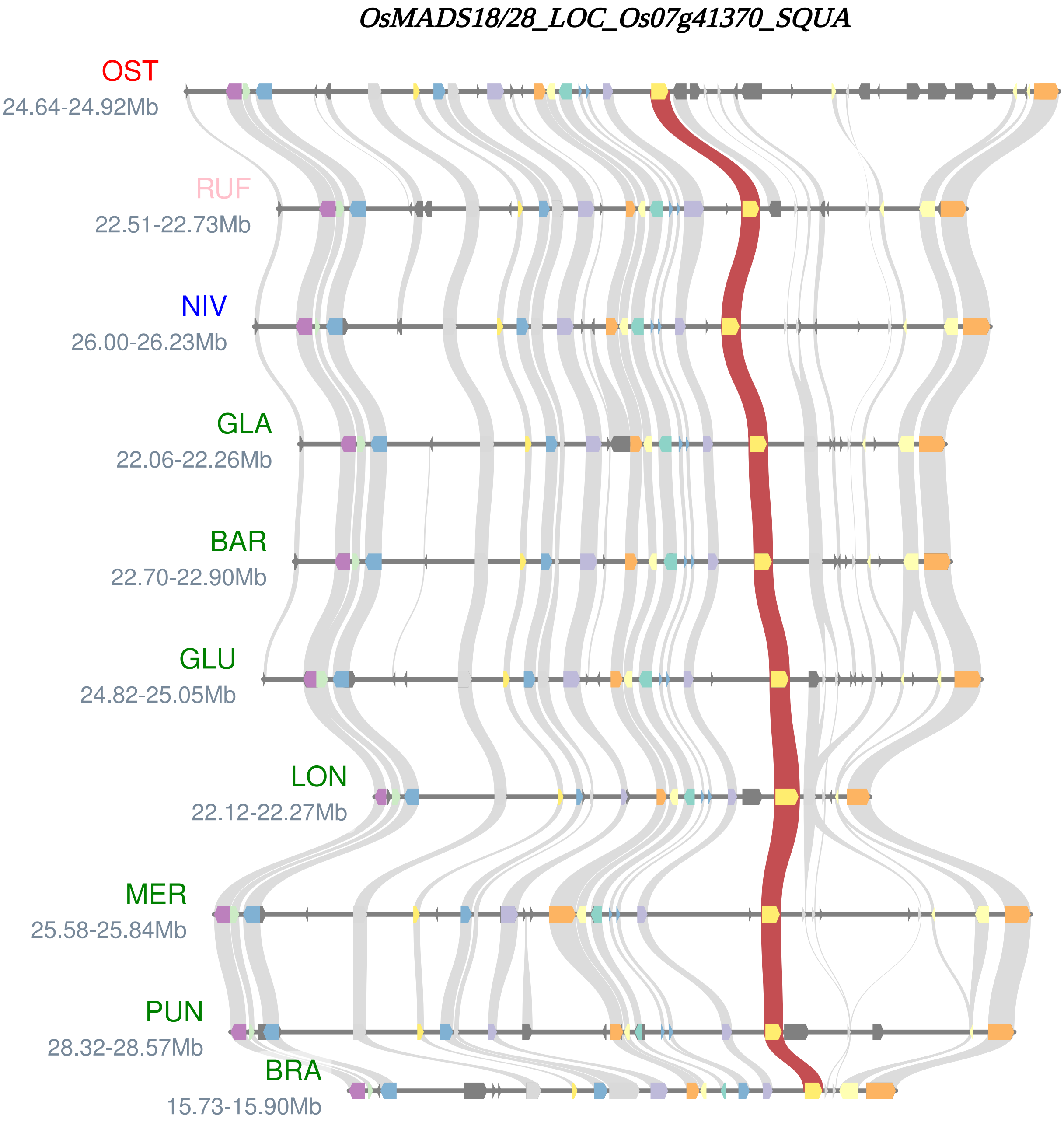

OsMADS18/28_LOC_Os07g41370_SQUA
OST
24.64-24.92Mb
RUF
22.51-22.73Mb
NIV
26.00-26.23Mb
GLA
22.06-22.26Mb
BAR
22.70-22.90Mb
GLU
24.82-25.05Mb
LON
22.12-22.27Mb
MER
25.58-25.84Mb
PUN
28.32-28.57Mb
BRA
15.73-15.90Mb

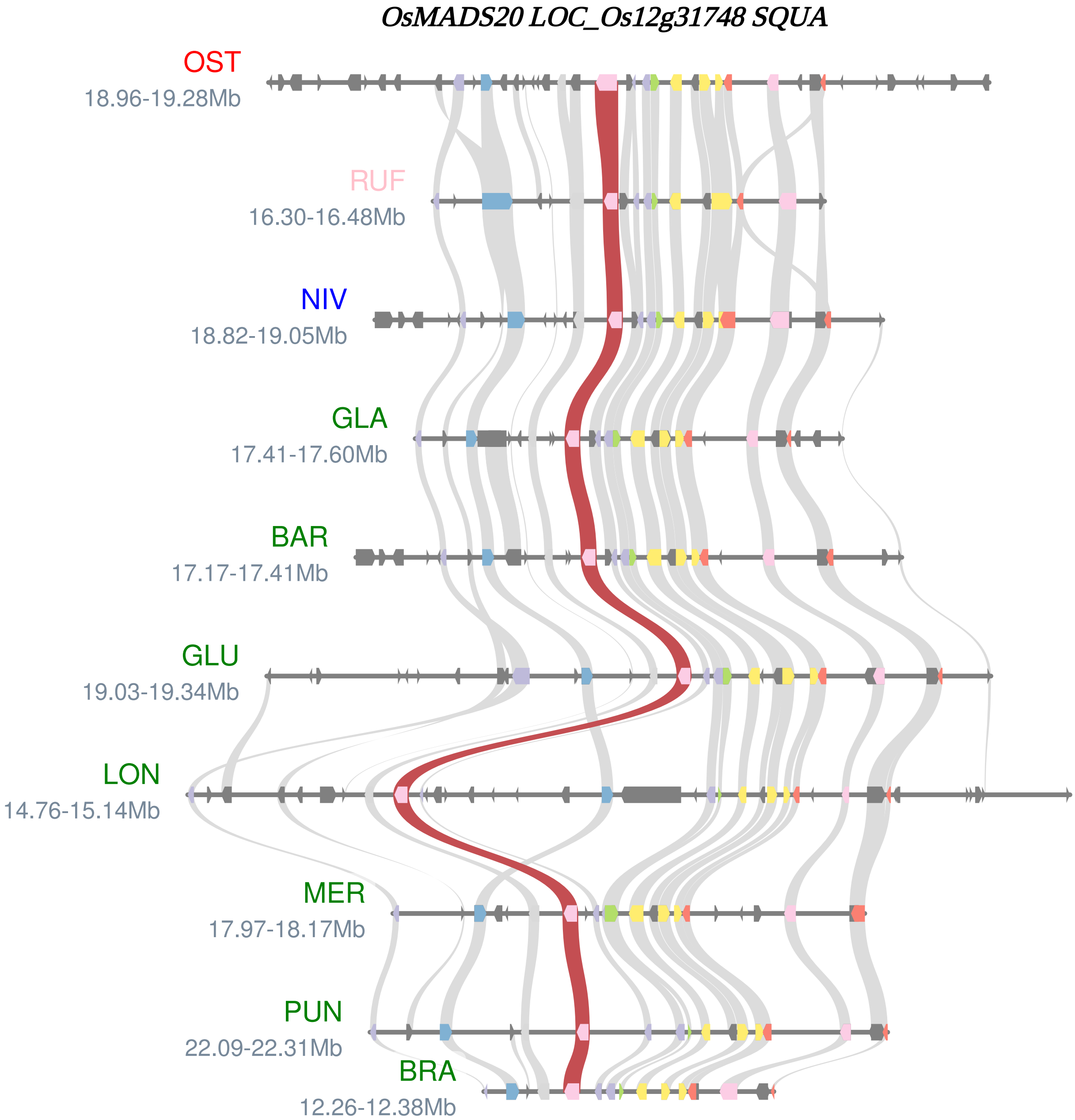
OsMADS20 LOC_Os12g31748 SQUA
OST
18.96-19.28Mb
RUF
16.30-16.48Mb
NIV
18.82-19.05Mb
GLA
17.41-17.60Mb
BAR
17.17-17.41Mb
GLU
19.03-19.34Mb
LON
14.76-15.14Mb
MER
17.97-18.17Mb
PUN
22.09-22.31Mb
BRA
12.26-12.38Mb

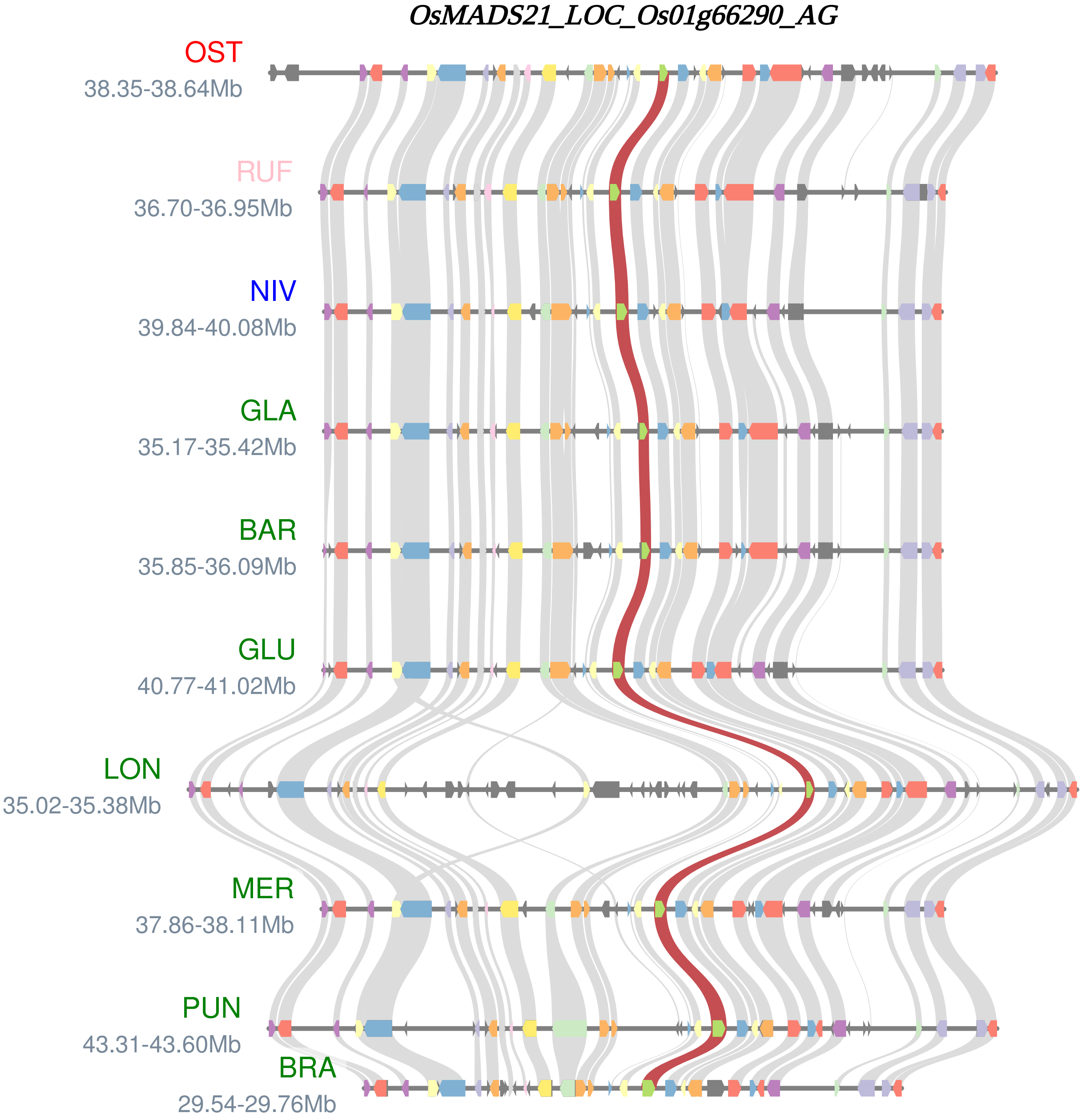

OsMADS21_LOC_Os01g66290_AG
OST
38.35-38.64Mb
RUF
36.70-36.95Mb
NIV
39.84-40.08Mb
GLA
35.17-35.42Mb
BAR
35.85-36.09Mb
GLU
40.77-41.02Mb
LON
35.02-35.38Mb
MER
37.86-38.11Mb
PUN
43.31-43.60Mb
BRA
29.54-29.76Mb

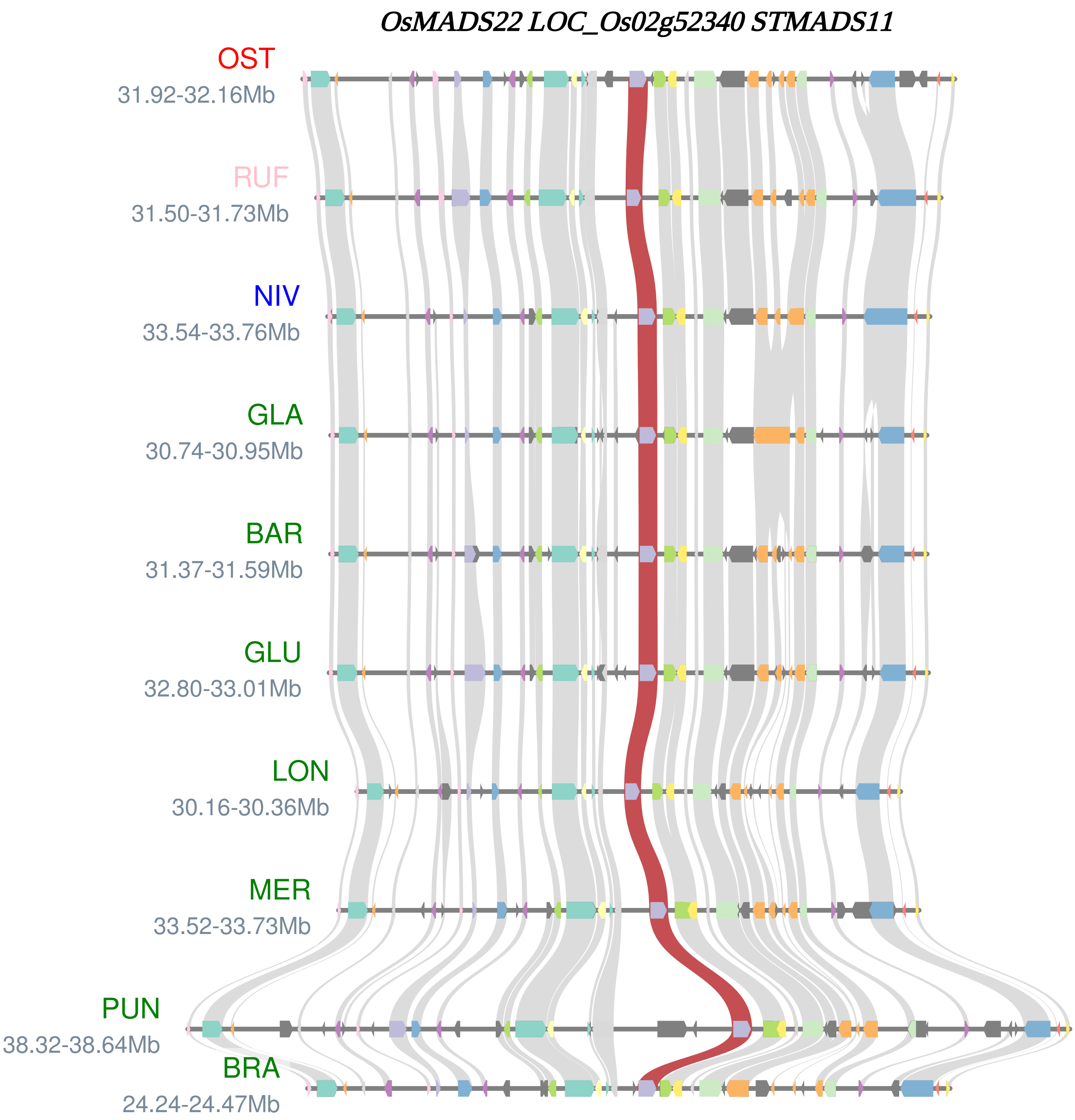
OsMADS22 LOC_Os02g52340 STMADS11
OST
31.92-32.16Mb
RUF
31.50-31.73Mb
NIV
33.54-33.76Mb
GLA
30.74-30.95Mb
BAR
31.37-31.59Mb
GLU
32.80-33.01Mb
LON
30.16-30.36Mb
MER
33.52-33.73Mb
PUN
38.32-38.64Mb
BRA
24.24-24.47Mb

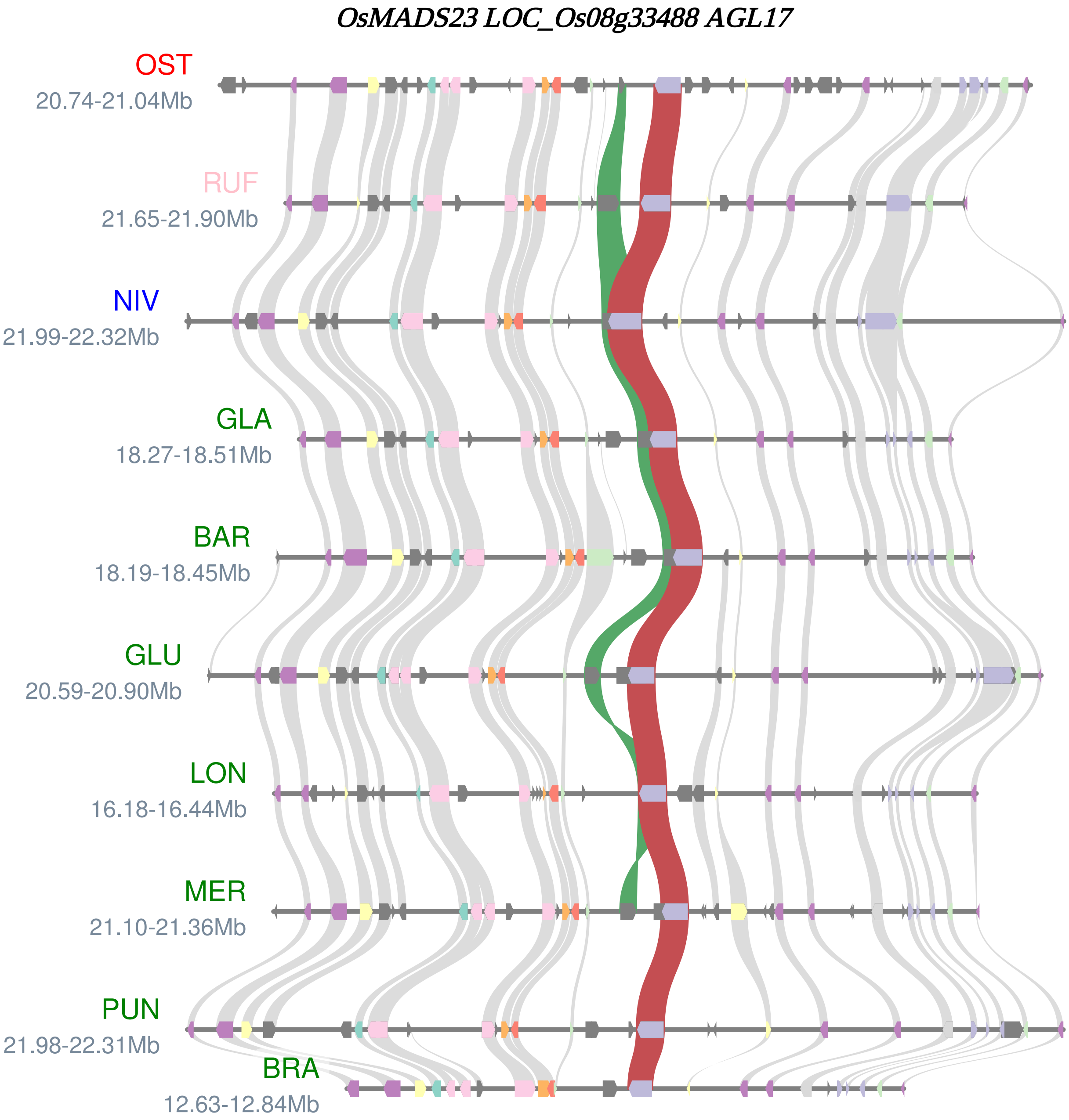

OsMADS23 LOC_Os08g33488 AGL17
OST
20.74-21.04Mb
RUF
21.65-21.90Mb
NIV
21.99-22.32Mb
GLA
18.27-18.51Mb
BAR
18.19-18.45Mb
GLU
20.59-20.90Mb
LON
16.18-16.44Mb
MER
21.10-21.36Mb
PUN
21.98-22.31Mb
BRA
12.63-12.84Mb

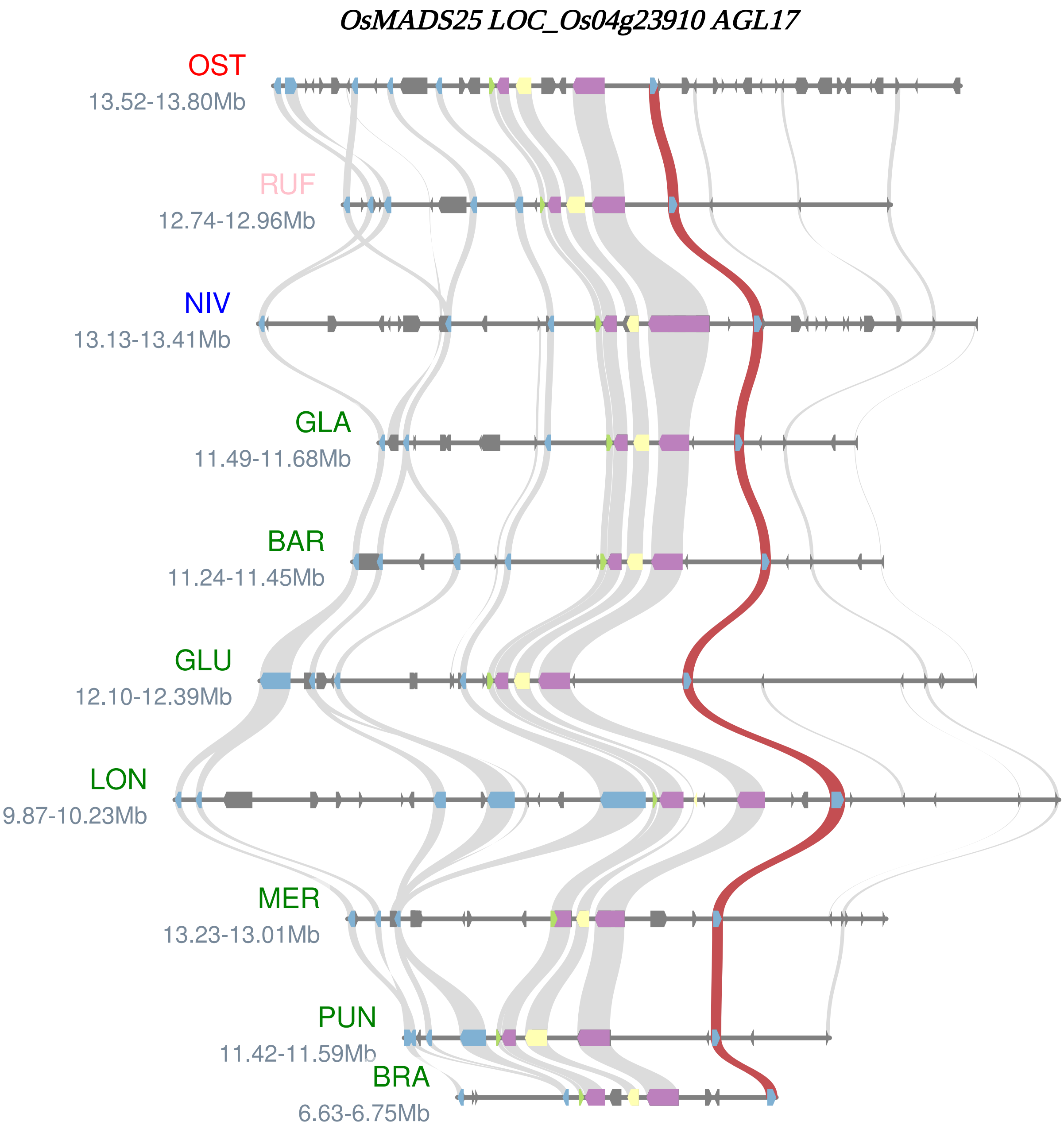

OsMADS25 LOC_Os04g23910 AGL17
OST
13.52-13.80Mb
RUF
12.74-12.96Mb
NIV
13.13-13.41Mb
GLA
11.49-11.68Mb
BAR
11.24-11.45Mb
GLU
12.10-12.39Mb
LON
9.87-10.23Mb
MER
13.23-13.01Mb
PUN
11.42-11.59Mb
BRA
6.63-6.75Mb

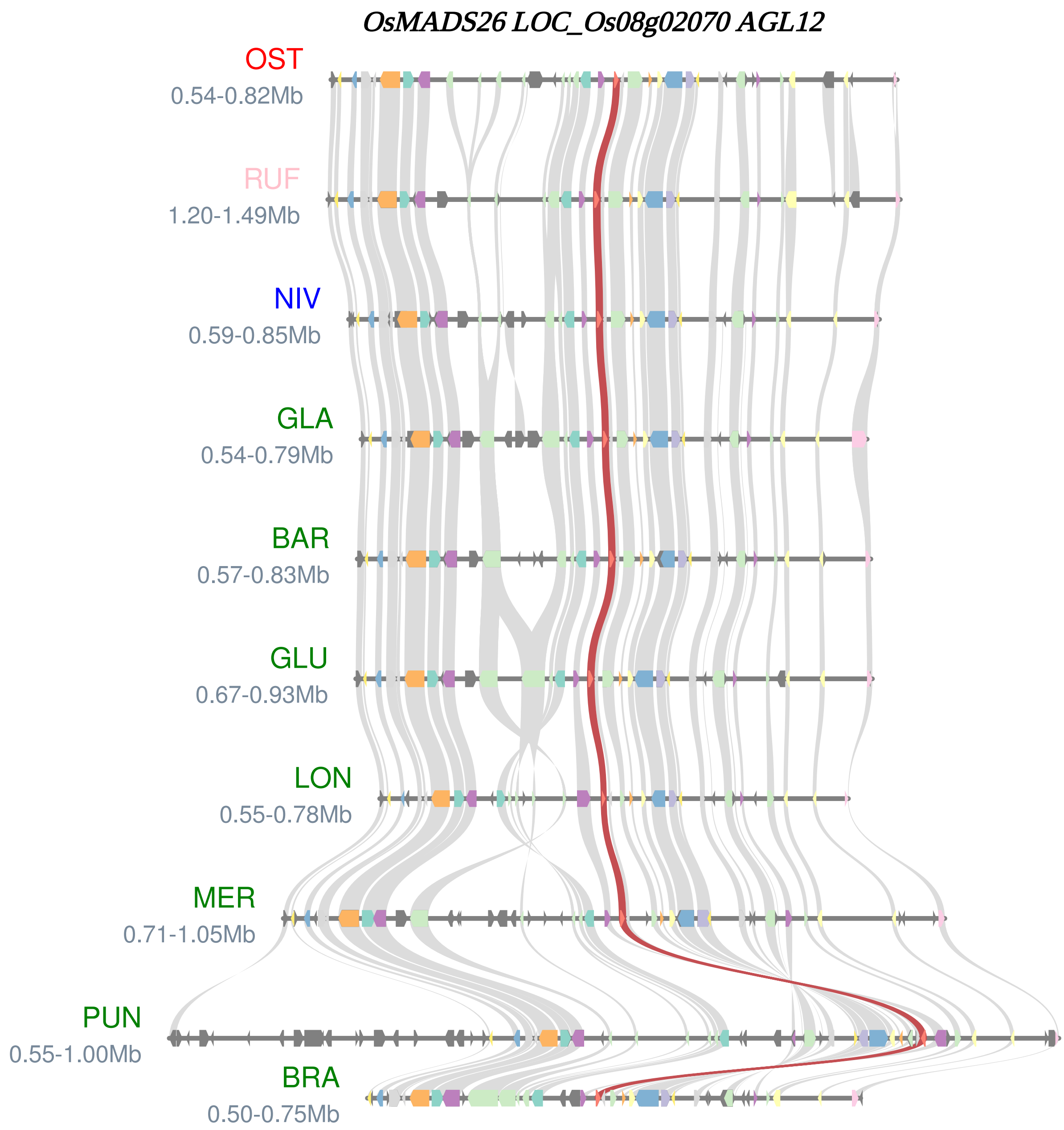
OsMADS26 LOC_Os08g02070 AGL12
OST
0.54-0.82Mb
RUF
1.20-1.49Mb
NIV
0.59-0.85Mb
GLA
0.54-0.79Mb
BAR
0.57-0.83Mb
GLU
0.67-0.93Mb
LON
0.55-0.78Mb
MER
0.71-1.05Mb
PUN
0.55-1.00Mb
BRA
0.50-0.75Mb

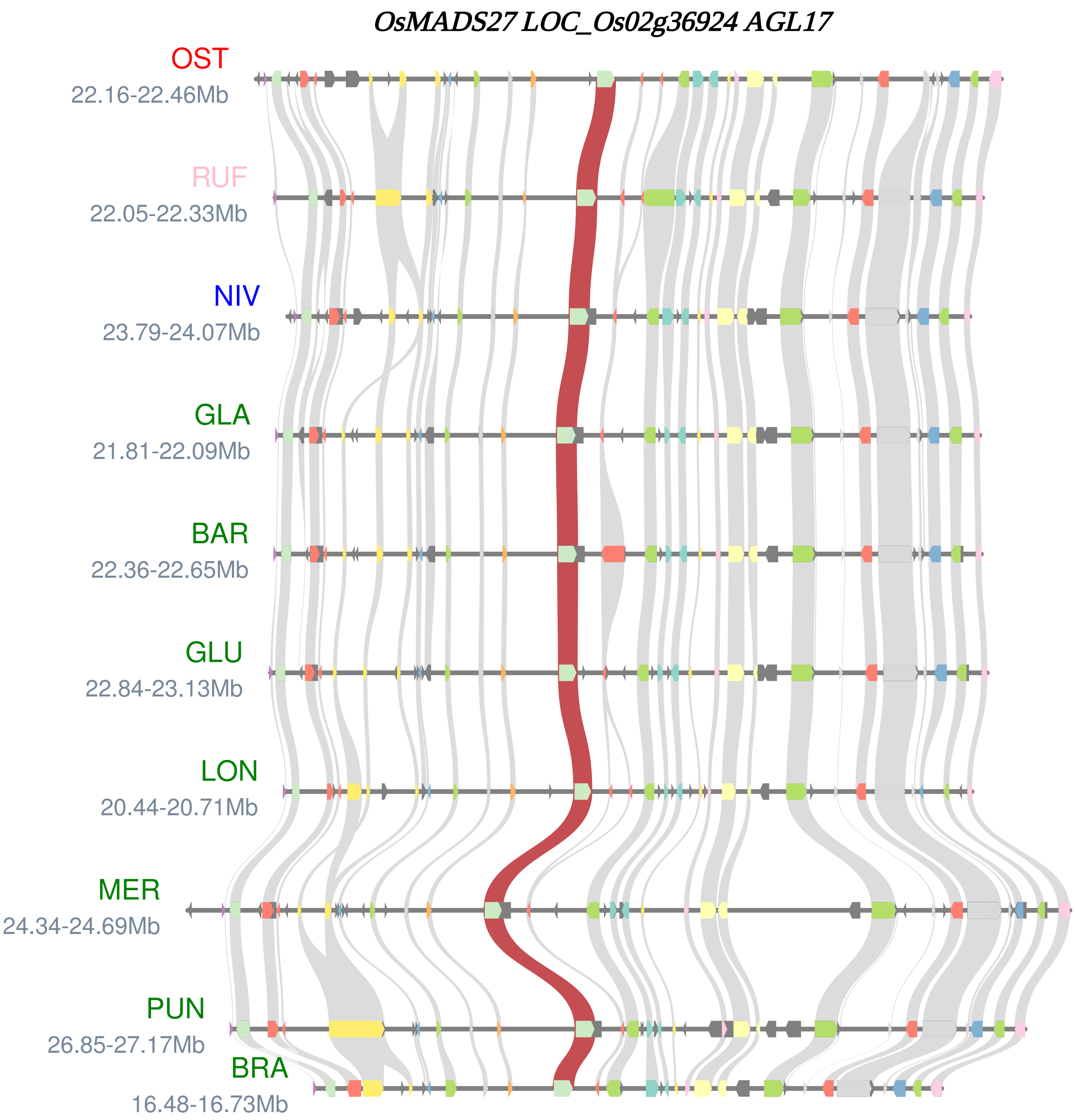
OsMADS27 LOC_Os02g36924 AGL17
OST
22.16-22.46Mb
RUF
22.05-22.33Mb
NIV
23.79-24.07Mb
GLA
21.81-22.09Mb
BAR
22.36-22.65Mb
GLU
22.84-23.13Mb
LON
20.44-20.71Mb
MER
24.34-24.69Mb
PUN
26.85-27.17Mb
BRA
16.48-16.73Mb

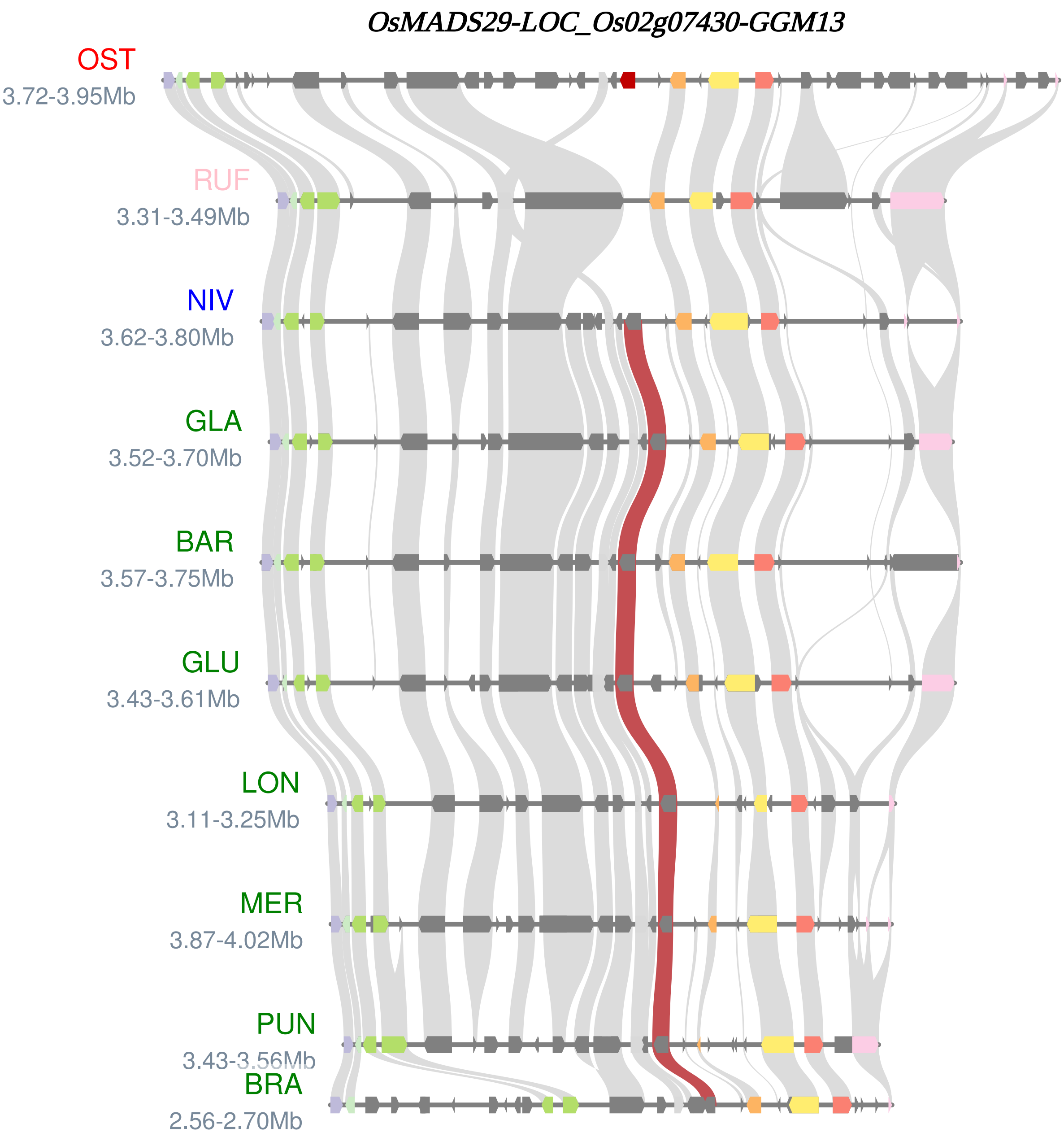
OsMADS29-LOC_Os02g07430-GGM13
OST
3.72-3.95Mb
RUF
3.31-3.49Mb
NIV
3.62-3.80Mb
GLA
3.52-3.70Mb
BAR
3.57-3.75Mb
GLU
3.43-3.61Mb
LON
3.11-3.25Mb
MER
3.87-4.02Mb
PUN
3.43-3.56Mb
BRA
2.56-2.70Mb

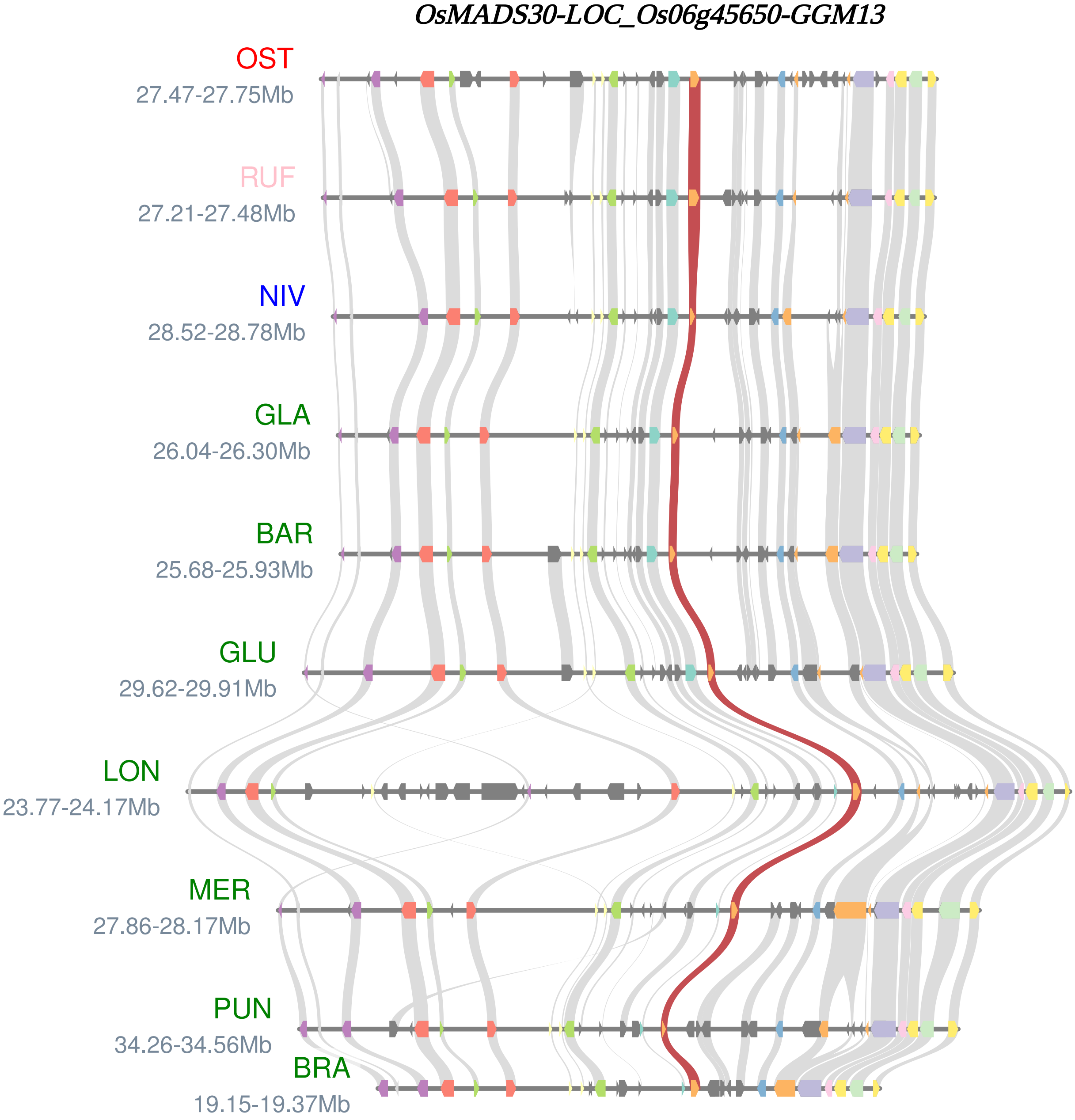

OsMADS30-LOC_Os06g45650-GGM13
OST
27.47-27.75Mb
RUF
27.21-27.48Mb
NIV
28.52-28.78Mb
GLA
26.04-26.30Mb
BAR
25.68-25.93Mb
GLU
29.62-29.91Mb
LON
23.77-24.17Mb
MER
27.86-28.17Mb
PUN
34.26-34.56Mb
BRA
19.15-19.37Mb

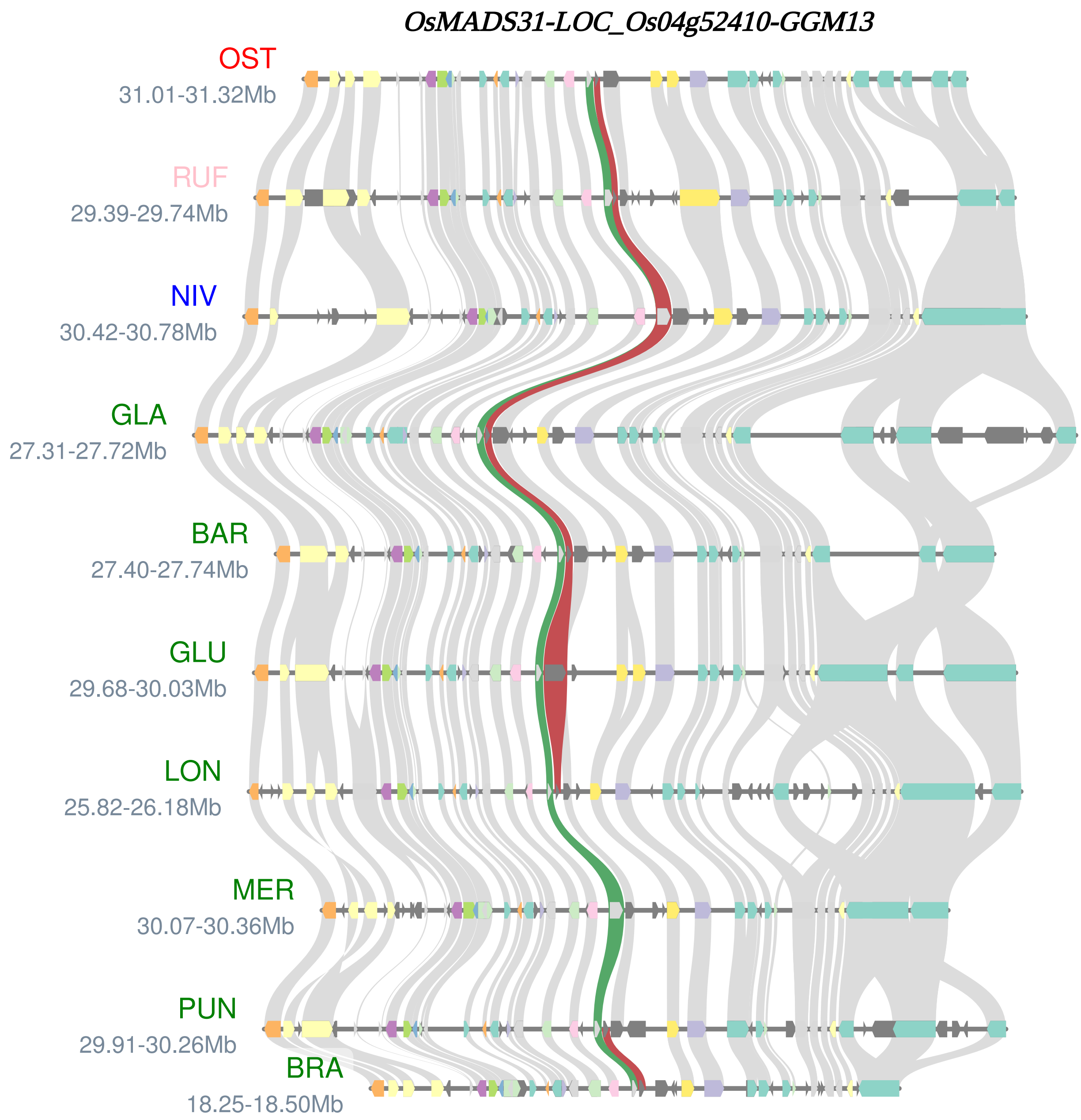
OsMADS31-LOC_Os04g52410-GGM13
OST
31.01-31.32Mb
RUF
29.39-29.74Mb
NIV
30.42-30.78Mb
GLA
27.31-27.72Mb
BAR
27.40-27.74Mb
GLU
29.68-30.03Mb
LON
25.82-26.18Mb
MER
30.07-30.36Mb
PUN
29.91-30.26Mb
BRA
18.25-18.50Mb

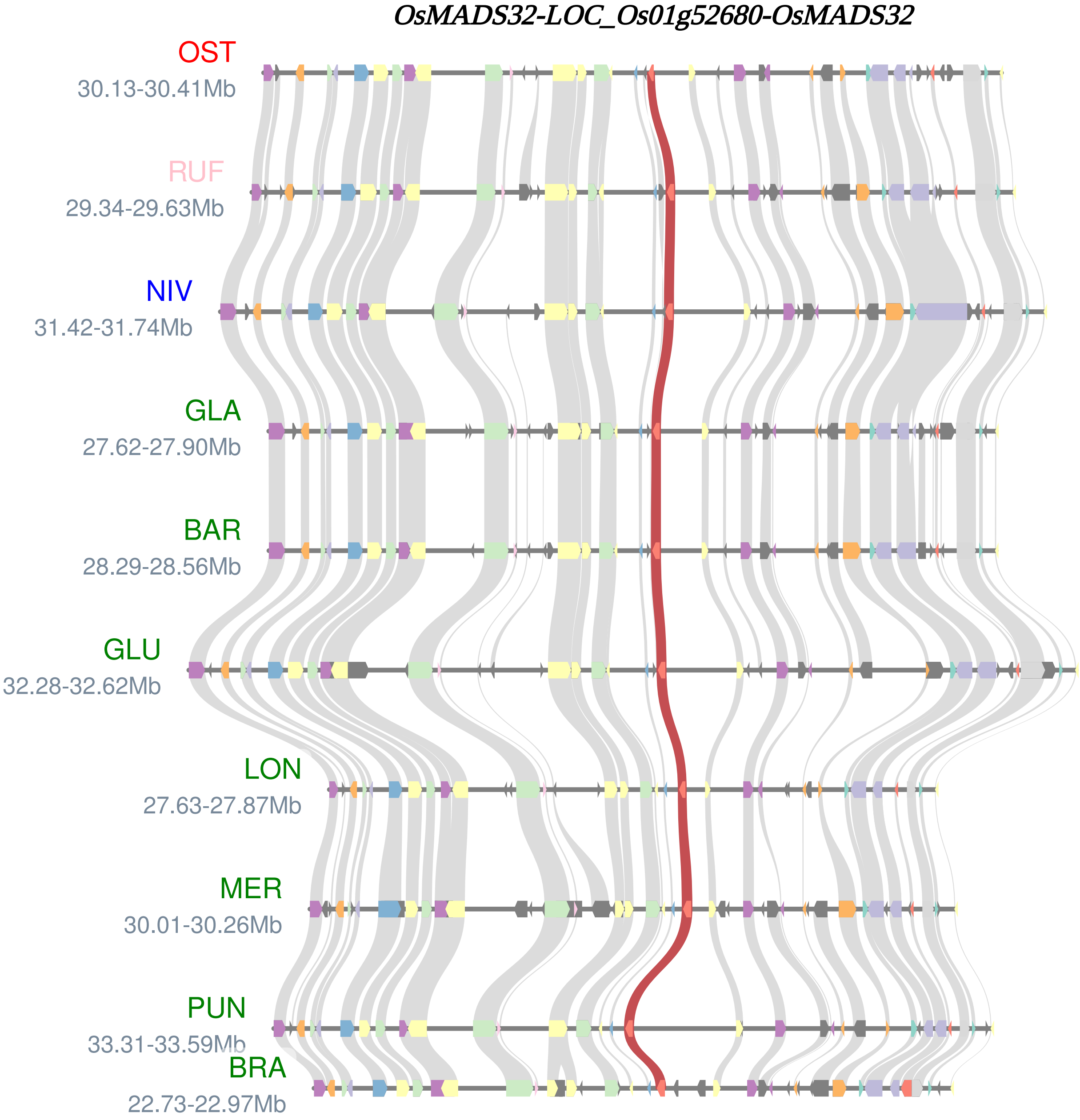
OsMADS32-LOC_Os01g52680-OsMADS32
OST
30.13-30.41Mb
RUF
29.34-29.63Mb
NIV
31.42-31.74Mb
GLA
27.62-27.90Mb
BAR
28.29-28.56Mb
GLU
32.28-32.62Mb
LON
27.63-27.87Mb
MER
30.01-30.26Mb
PUN
33.31-33.59Mb
BRA
22.73-22.97Mb

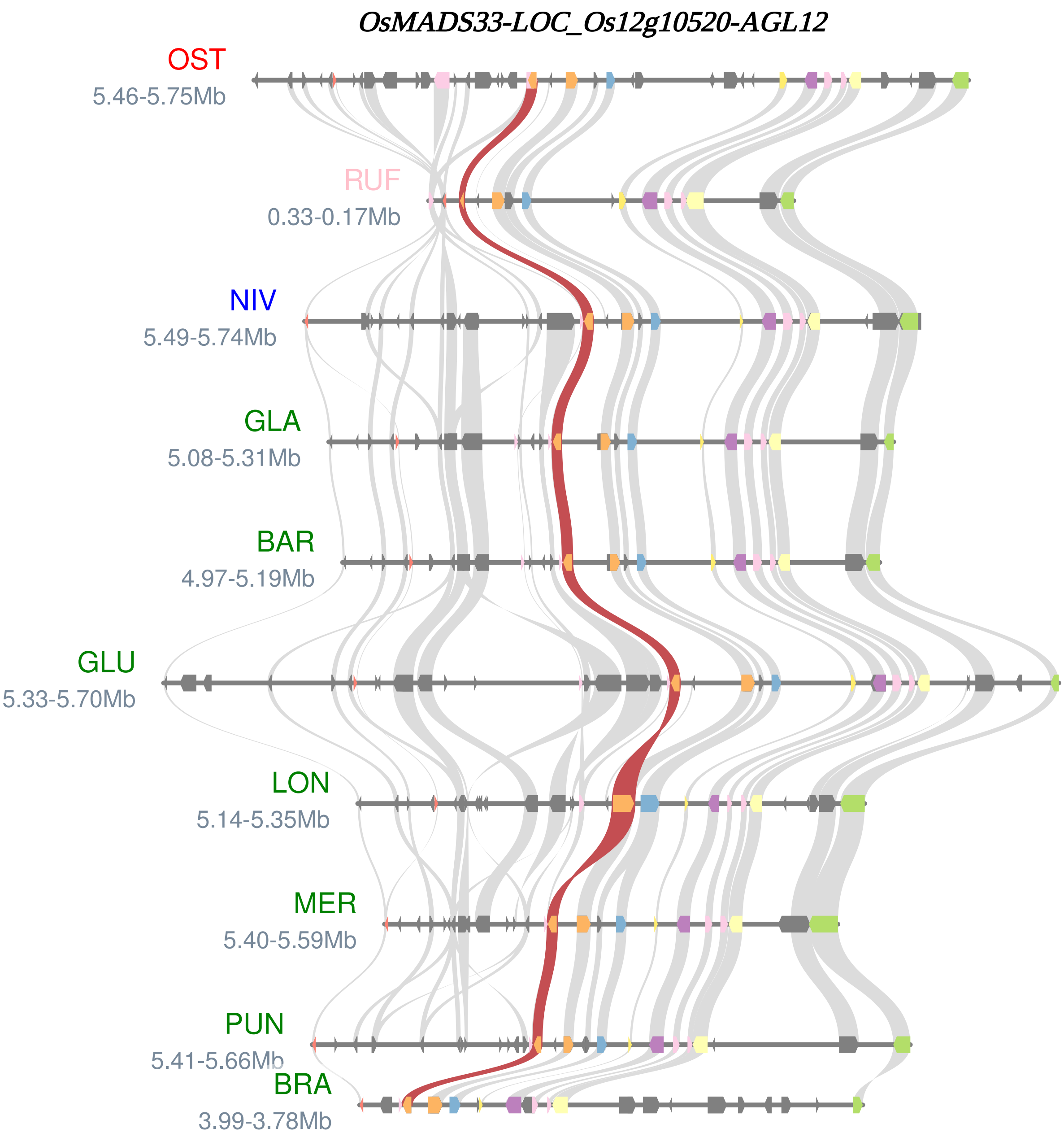

OsMADS33-LOC_Os12g10520-AGL12
OST
5.46-5.75Mb
RUF
0.33-0.17Mb
NIV
5.49-5.74Mb
GLA
5.08-5.31Mb
BAR
4.97-5.19Mb
GLU
5.33-5.70Mb
LON
5.14-5.35Mb
MER
5.40-5.59Mb
PUN
5.41-5.66Mb
BRA
3.99-3.78Mb

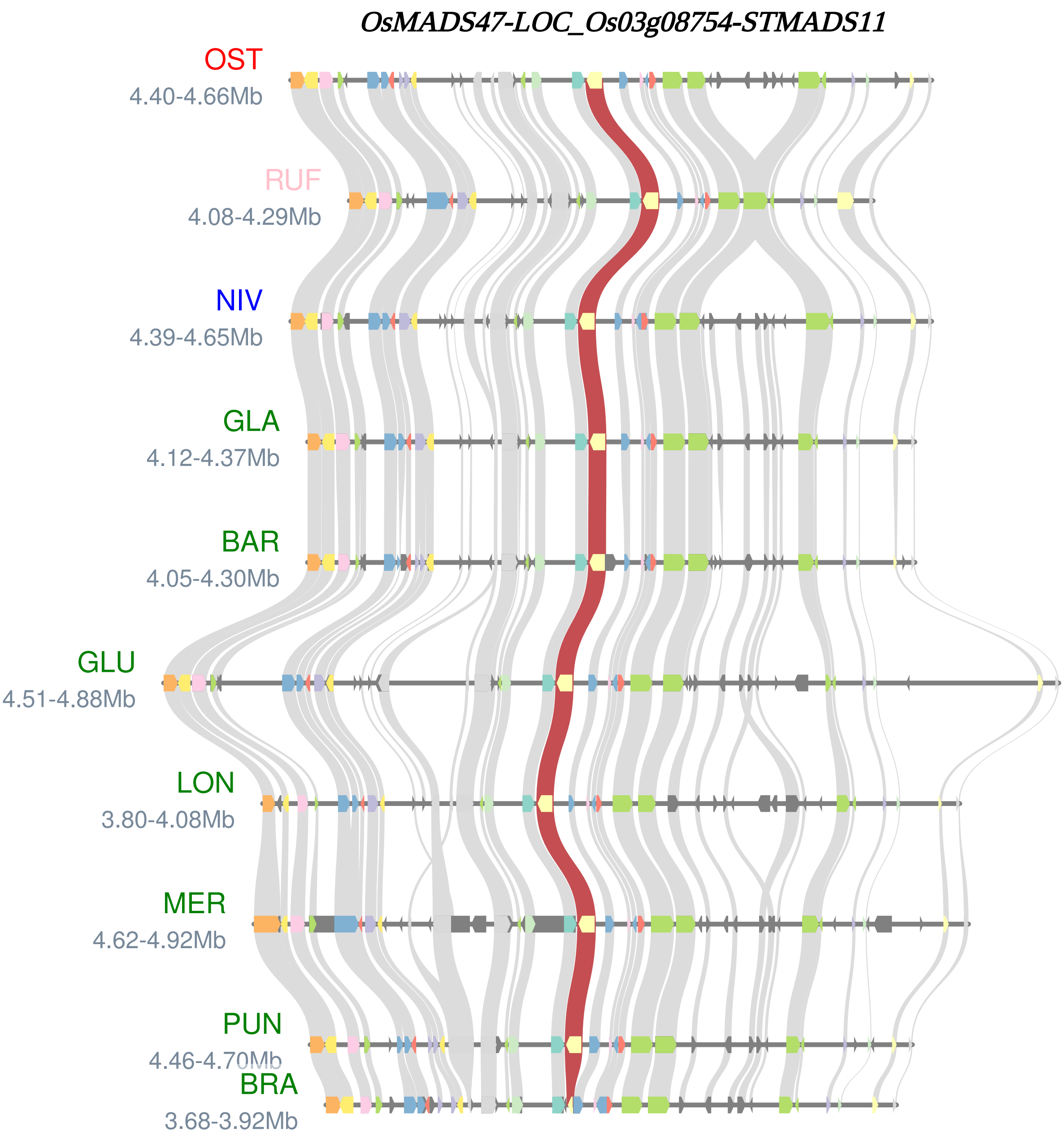

OsMADS47-LOC_Os03g08754-STMADS11
OST
4.40-4.66Mb
RUF
4.08-4.29Mb
NIV
4.39-4.65Mb
GLA
4.12-4.37Mb
BAR
4.05-4.30Mb
GLU
4.51-4.88Mb
LON
3.80-4.08Mb
MER
4.62-4.92Mb
PUN
4.46-4.70Mb
BRA
3.68-3.92Mb

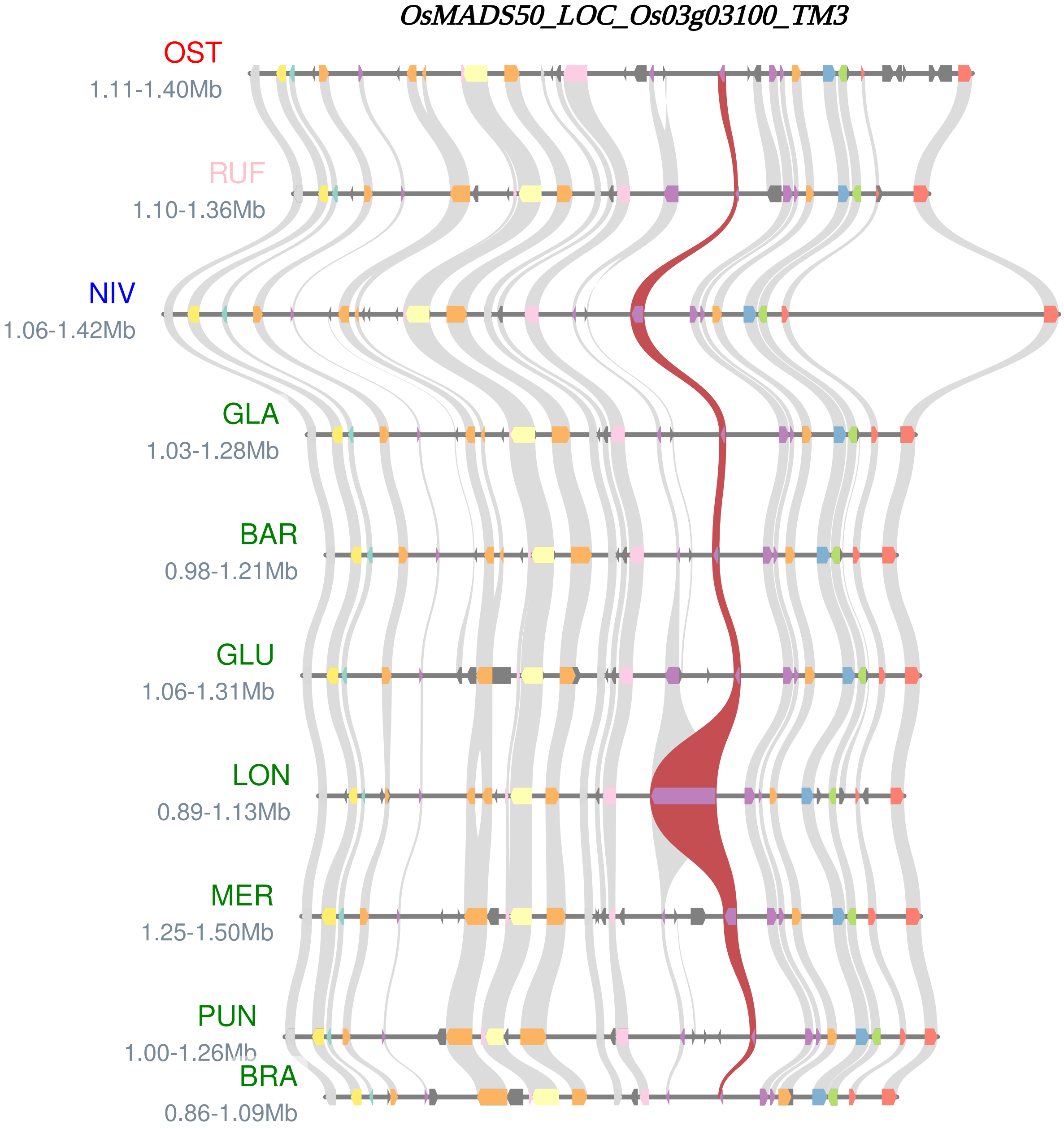

OsMADS50_LOC_Os03g03100_TM3
OST
1.11-1.40Mb
RUF
1.10-1.36Mb
NIV
1.06-1.42Mb
GLA
1.03-1.28Mb
BAR
0.98-1.21Mb
GLU
1.06-1.31Mb
LON
0.89-1.13Mb
MER
1.25-1.50Mb
PUN
1.00-1.26Mb
BRA
0.86-1.09Mb

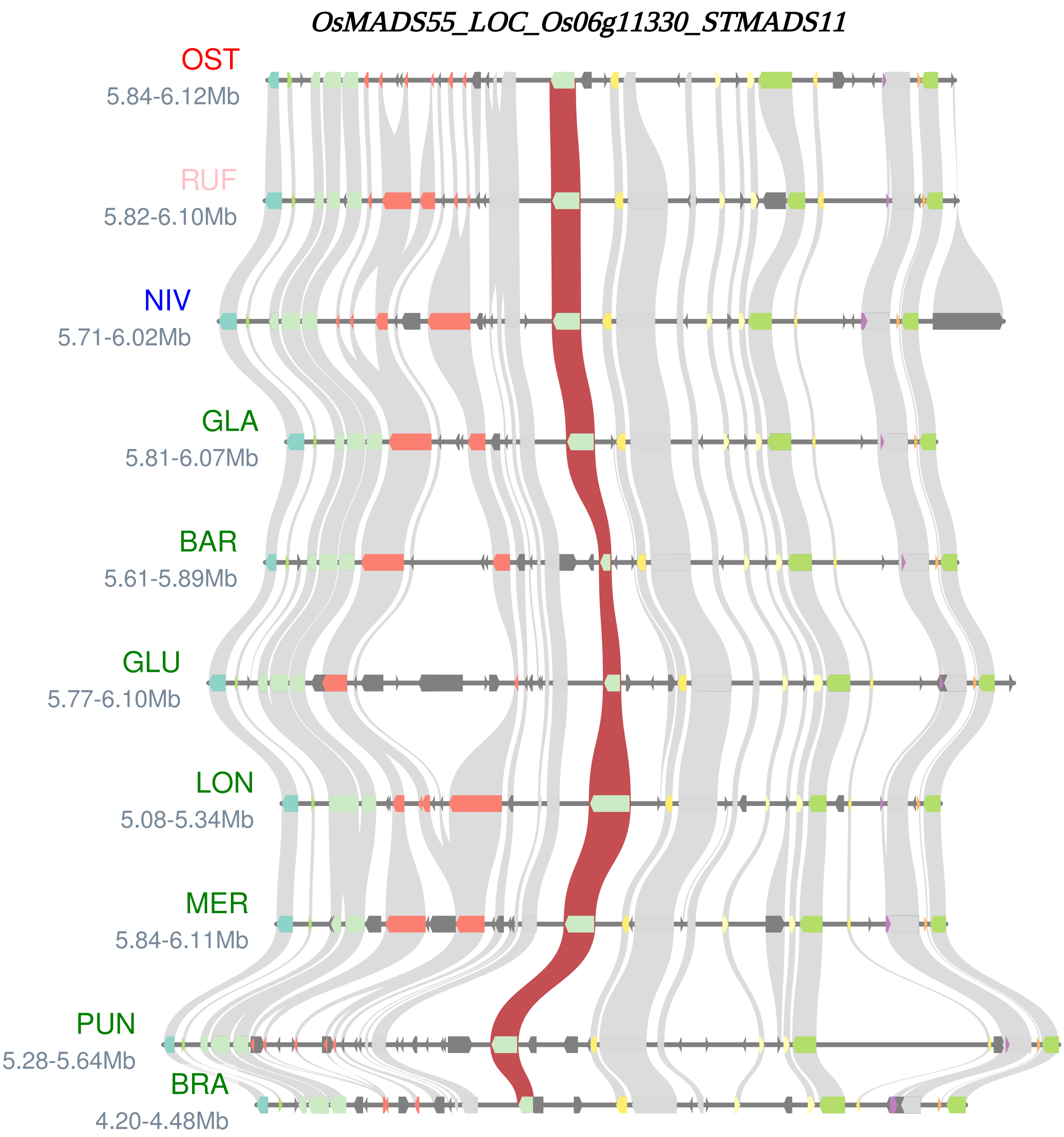

OsMADS55_LOC_Os06g11330_STMADS11
OST
5.84-6.12Mb
RUF
5.82-6.10Mb
NIV
5.71-6.02Mb
GLA
5.81-6.07Mb
BAR
5.61-5.89Mb
GLU
5.77-6.10Mb
LON
5.08-5.34Mb
MER
5.84-6.11Mb
PUN
5.28-5.64Mb
BRA
4.20-4.48Mb

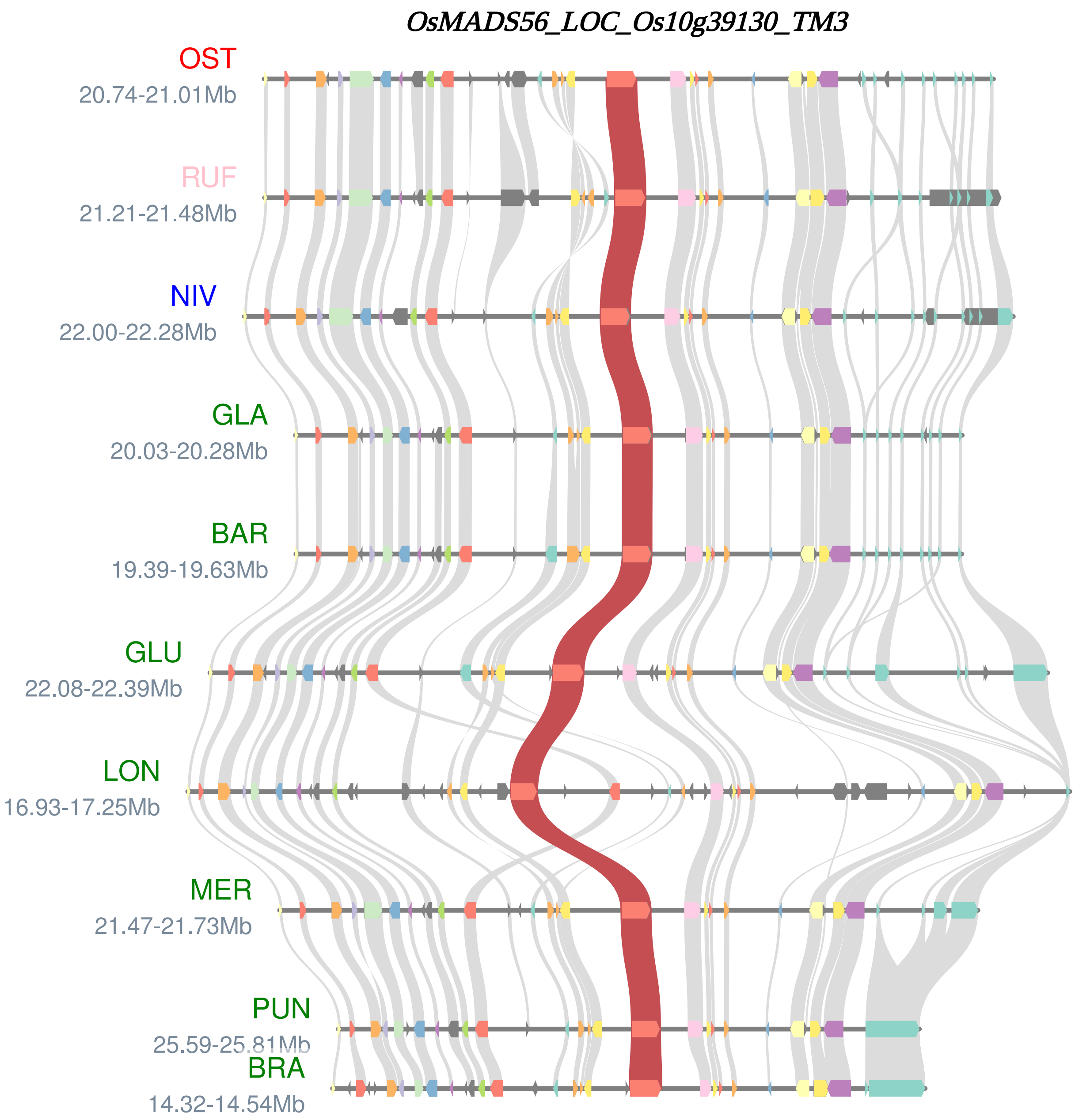

OsMADS56_LOC_Os10g39130_TM3
OST
20.74-21.01Mb
RUF
21.21-21.48Mb
NIV
22.00-22.28Mb
GLA
20.03-20.28Mb
BAR
19.39-19.63Mb
GLU
22.08-22.39Mb
LON
16.93-17.25Mb
MER
21.47-21.73Mb
PUN
25.59-25.81Mb
BRA
14.32-14.54Mb

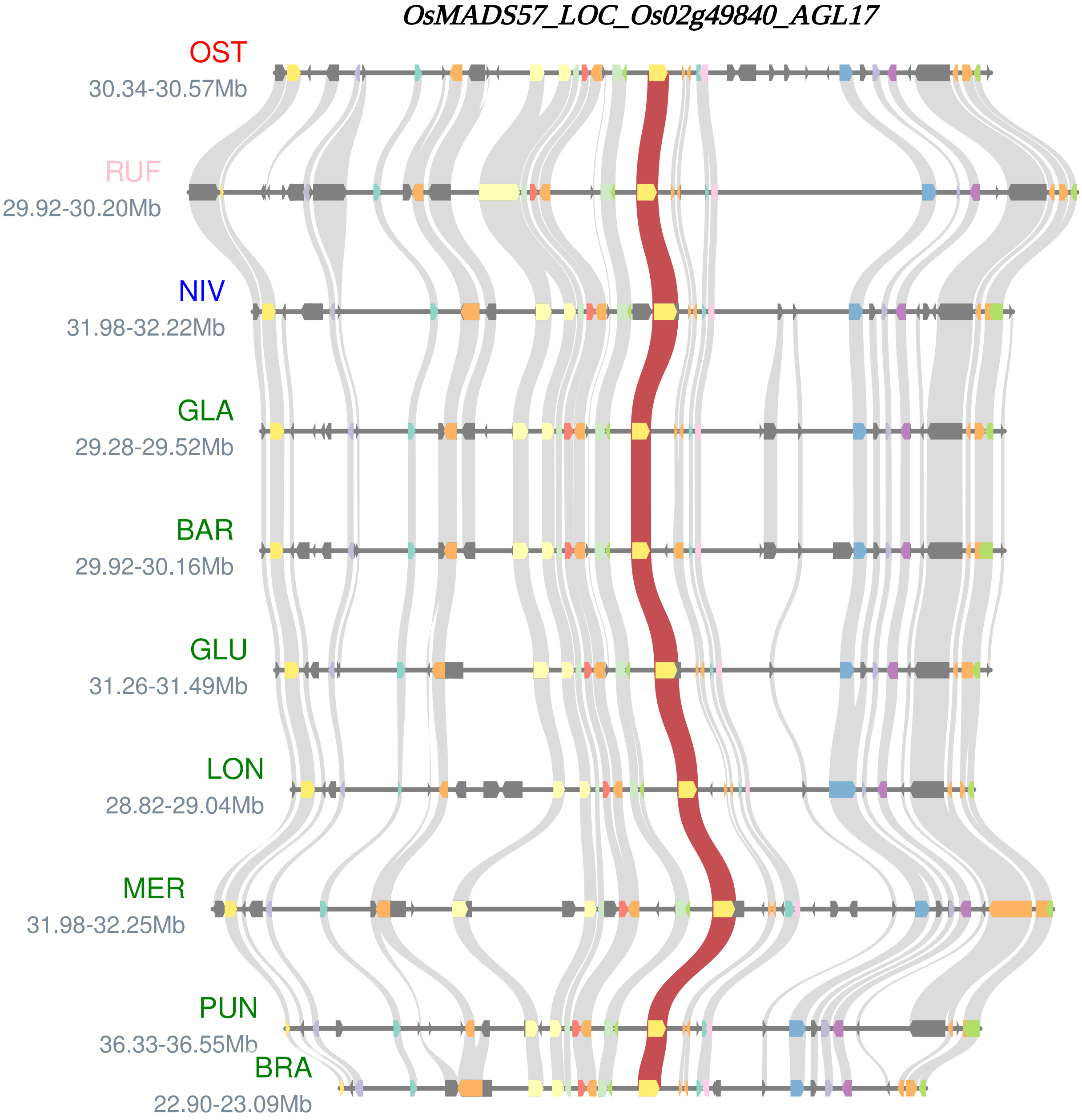

OsMADS57_LOC_Os02g49840_AGL17
OST
30.34-30.57Mb
RUF
29.92-30.20Mb
NIV
31.98-32.22Mb
GLA
29.28-29.52Mb
BAR
29.92-30.16Mb
GLU
31.26-31.49Mb
LON
28.82-29.04Mb
MER
31.98-32.25Mb
PUN
36.33-36.55Mb
BRA
22.90-23.09Mb

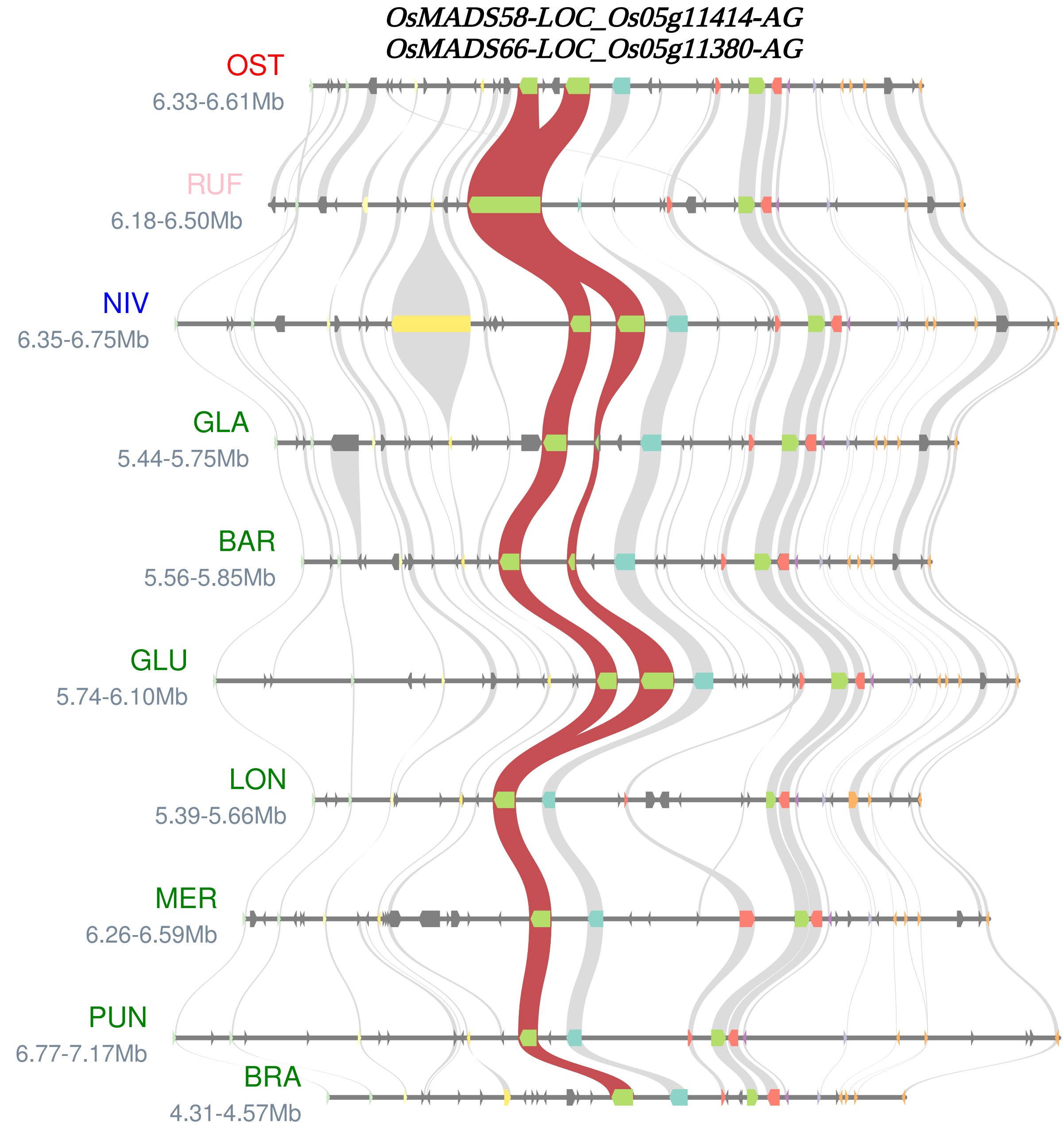

OsMADS58-LOC_Os05g11414-AG
OsMADS66-LOC_Os05g11380-AG
OST
6.33-6.61Mb
RUF
6.18-6.50Mb
NIV
6.35-6.75Mb
GLA
5.44-5.75Mb
BAR
5.56-5.85Mb
GLU
5.74-6.10Mb
LON
5.39-5.66Mb
MER
6.26-6.59Mb
PUN
6.77-7.17Mb
BRA
4.31-4.57Mb

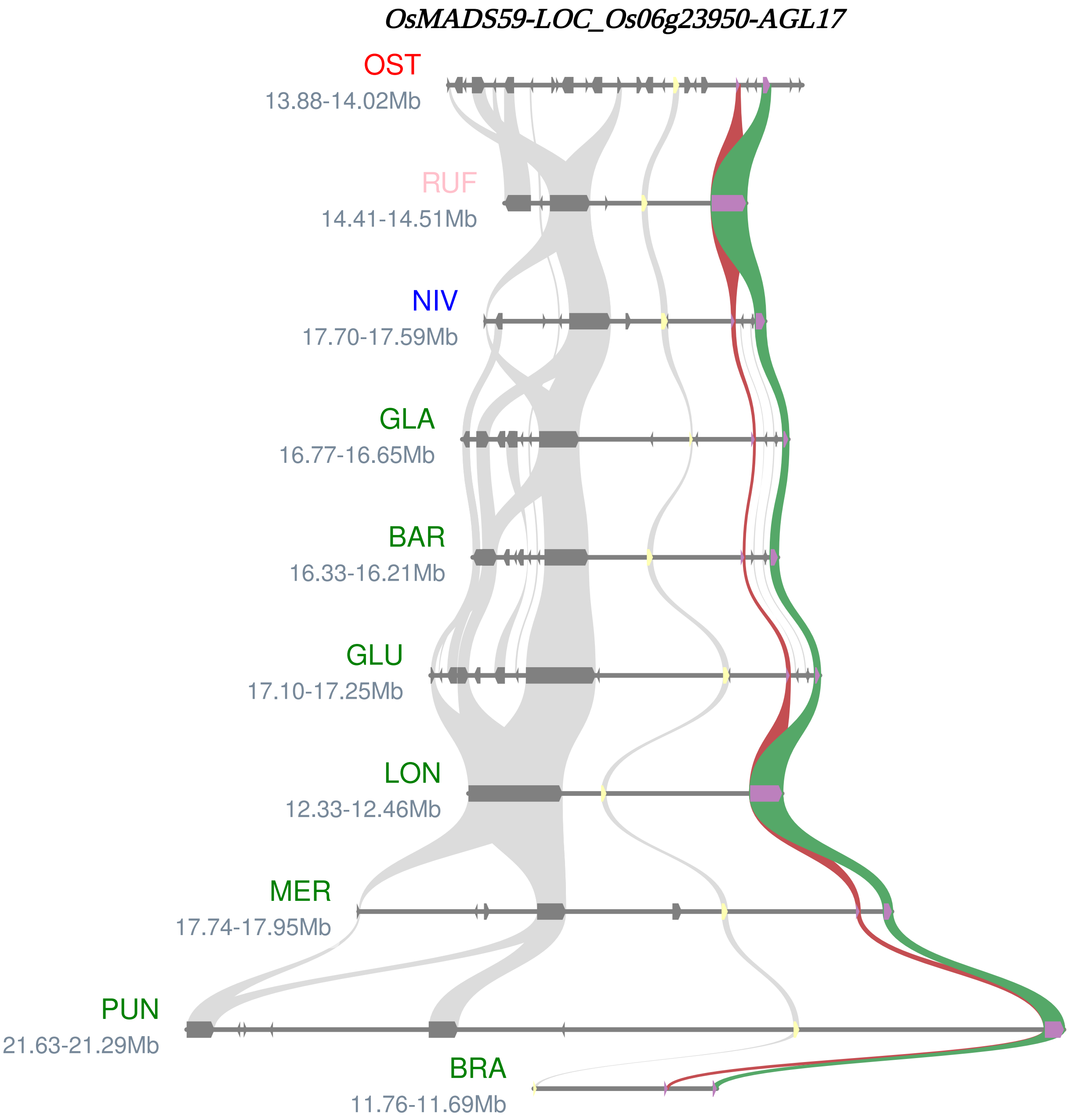
OsMADS59-LOC_Os06g23950-AGL17
OST
13.88-14.02Mb
RUF
14.41-14.51Mb
NIV
17.70-17.59Mb
GLA
16.77-16.65Mb
BAR
16.33-16.21Mb
GLU
17.10-17.25Mb
LON
12.33-12.46Mb
MER
17.74-17.95Mb
PUN
21.63-21.29Mb
BRA
11.76-11.69Mb

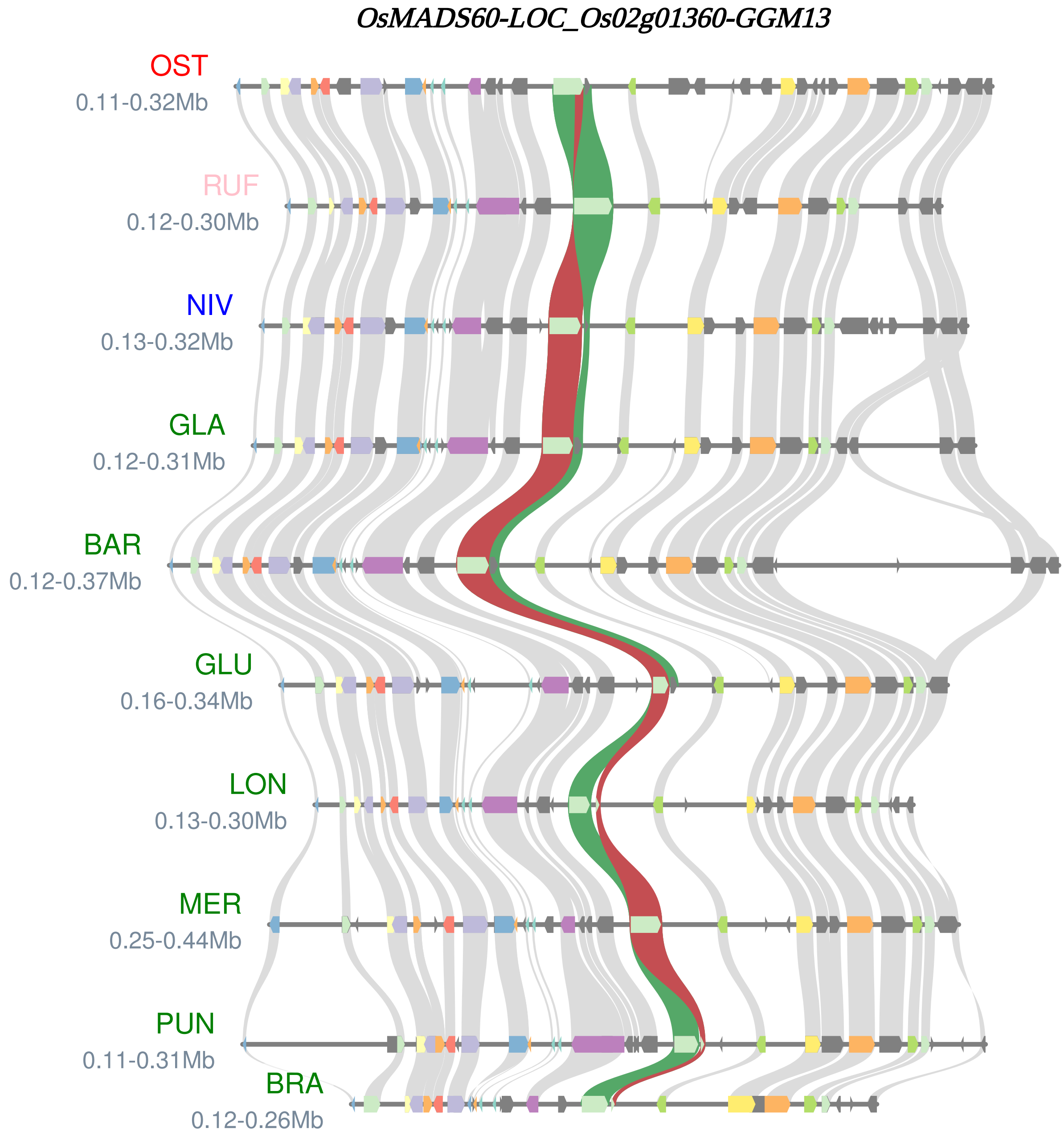
OsMADS60-LOC_Os02g01360-GGM13
OST
0.11-0.32Mb
RUF
0.12-0.30Mb
NIV
0.13-0.32Mb
GLA
0.12-0.31Mb
BAR
0.12-0.37Mb
GLU
0.16-0.34Mb
LON
0.13-0.30Mb
MER
0.25-0.44Mb
PUN
0.11-0.31Mb
BRA
0.12-0.26Mb

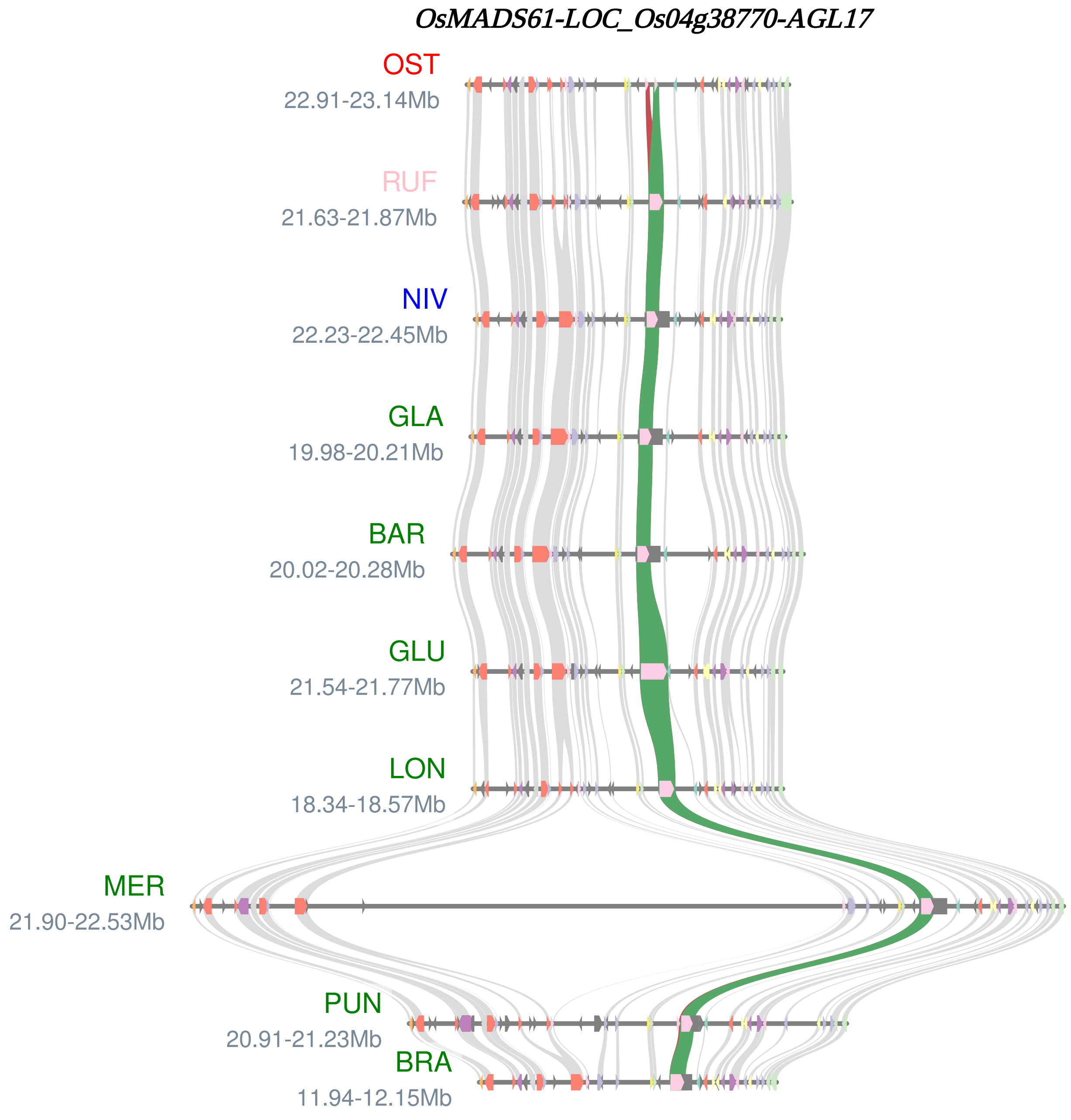
OsMADS61-LOC_Os04g38770-AGL17
OST
22.91-23.14Mb
RUF
21.63-21.87Mb
NIV
22.23-22.45Mb
GLA
19.98-20.21Mb
BAR
20.02-20.28Mb
GLU
21.54-21.77Mb
LON
18.34-18.57Mb
MER
21.90-22.53Mb
PUN
20.91-21.23Mb
BRA
11.94-12.15Mb

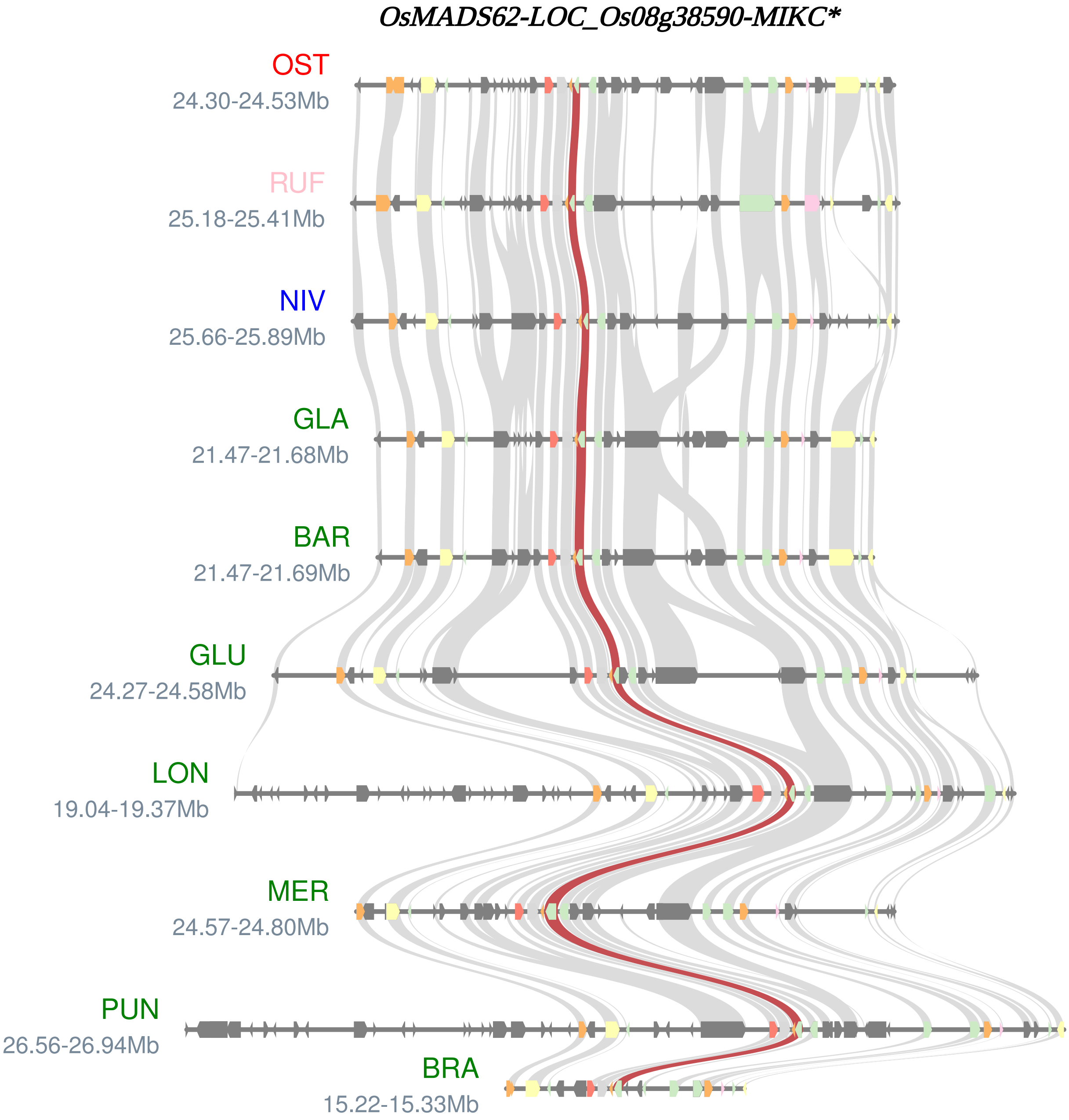

OsMADS62-LOC_Os08g38590-MIKC*
OST
24.30-24.53Mb
RUF
25.18-25.41Mb
NIV
25.66-25.89Mb
GLA
21.47-21.68Mb
BAR
21.47-21.69Mb
GLU
24.27-24.58Mb
LON
19.04-19.37Mb
MER
24.57-24.80Mb
PUN
26.56-26.94Mb
BRA
15.22-15.33Mb

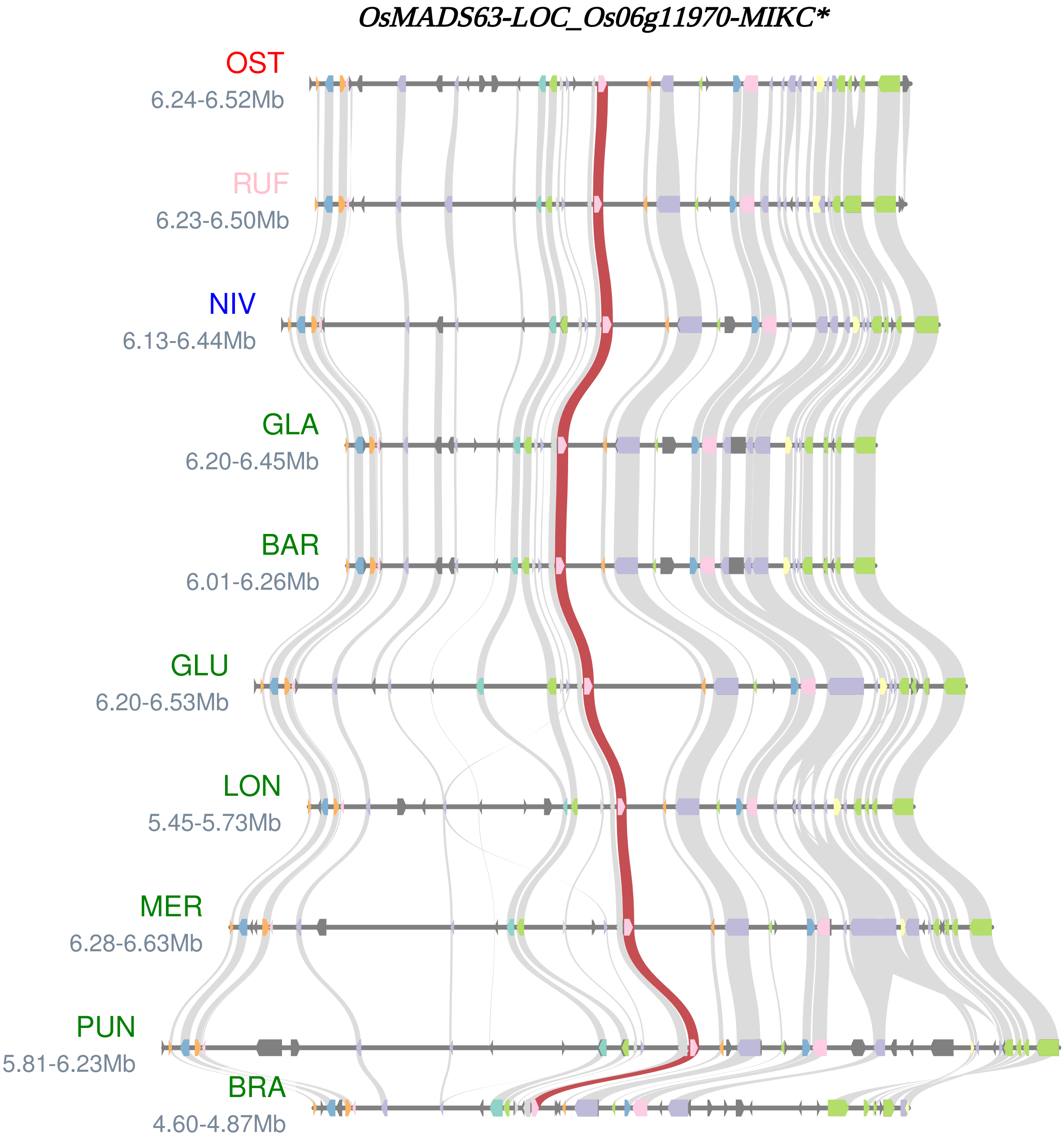
OsMADS63-LOC_Os06g11970-MIKC*
OST
6.24-6.52Mb
RUF
6.23-6.50Mb
NIV
6.13-6.44Mb
GLA
6.20-6.45Mb
BAR
6.01-6.26Mb
GLU
6.20-6.53Mb
LON
5.45-5.73Mb
MER
6.28-6.63Mb
PUN
5.81-6.23Mb
BRA
4.60-4.87Mb

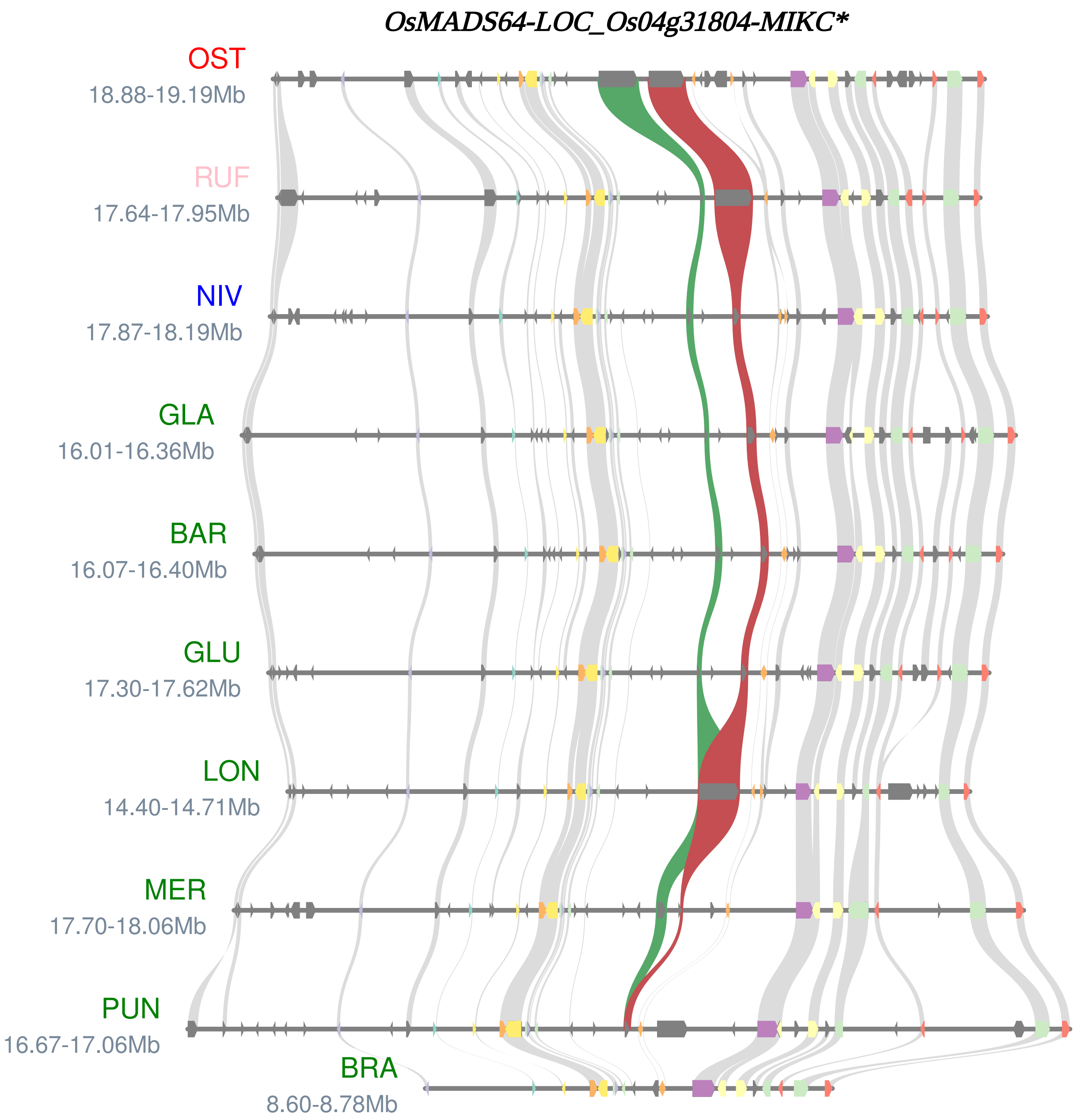

OsMADS64-LOC_Os04g31804-MIKC*
OST
18.88-19.19Mb
RUF
17.64-17.95Mb
NIV
17.87-18.19Mb
GLA
16.01-16.36Mb
BAR
16.07-16.40Mb
GLU
17.30-17.62Mb
LON
14.40-14.71Mb
MER
17.70-18.06Mb
PUN
16.67-17.06Mb
BRA
8.60-8.78Mb

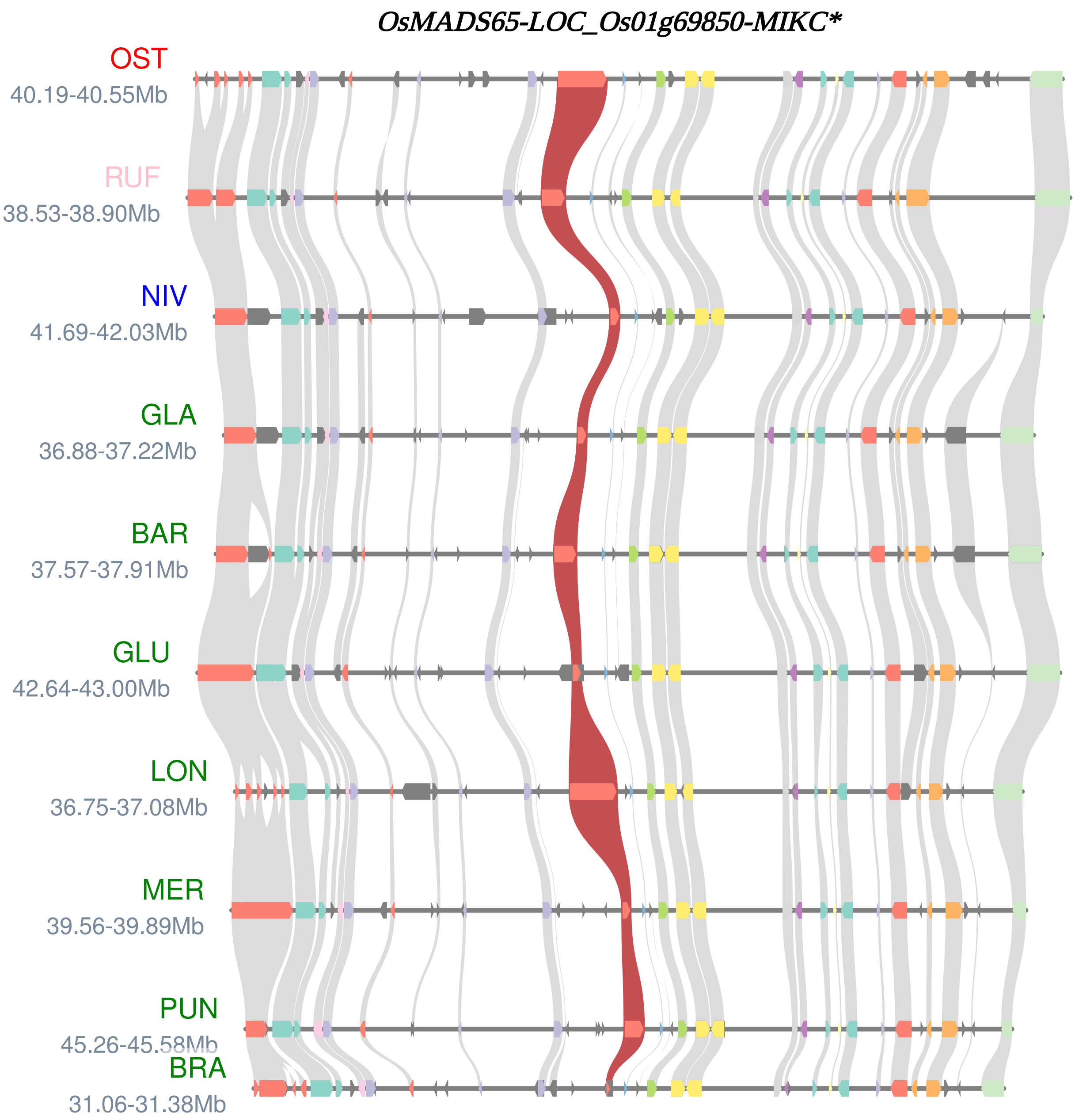

OsMADS65-LOC_Os01g69850-MIKC*
OST
40.19-40.55Mb
RUF
38.53-38.90Mb
NIV
41.69-42.03Mb
GLA
36.88-37.22Mb
BAR
37.57-37.91Mb
GLU
42.64-43.00Mb
LON
36.75-37.08Mb
MER
39.56-39.89Mb
PUN
45.26-45.58Mb
BRA
31.06-31.38Mb

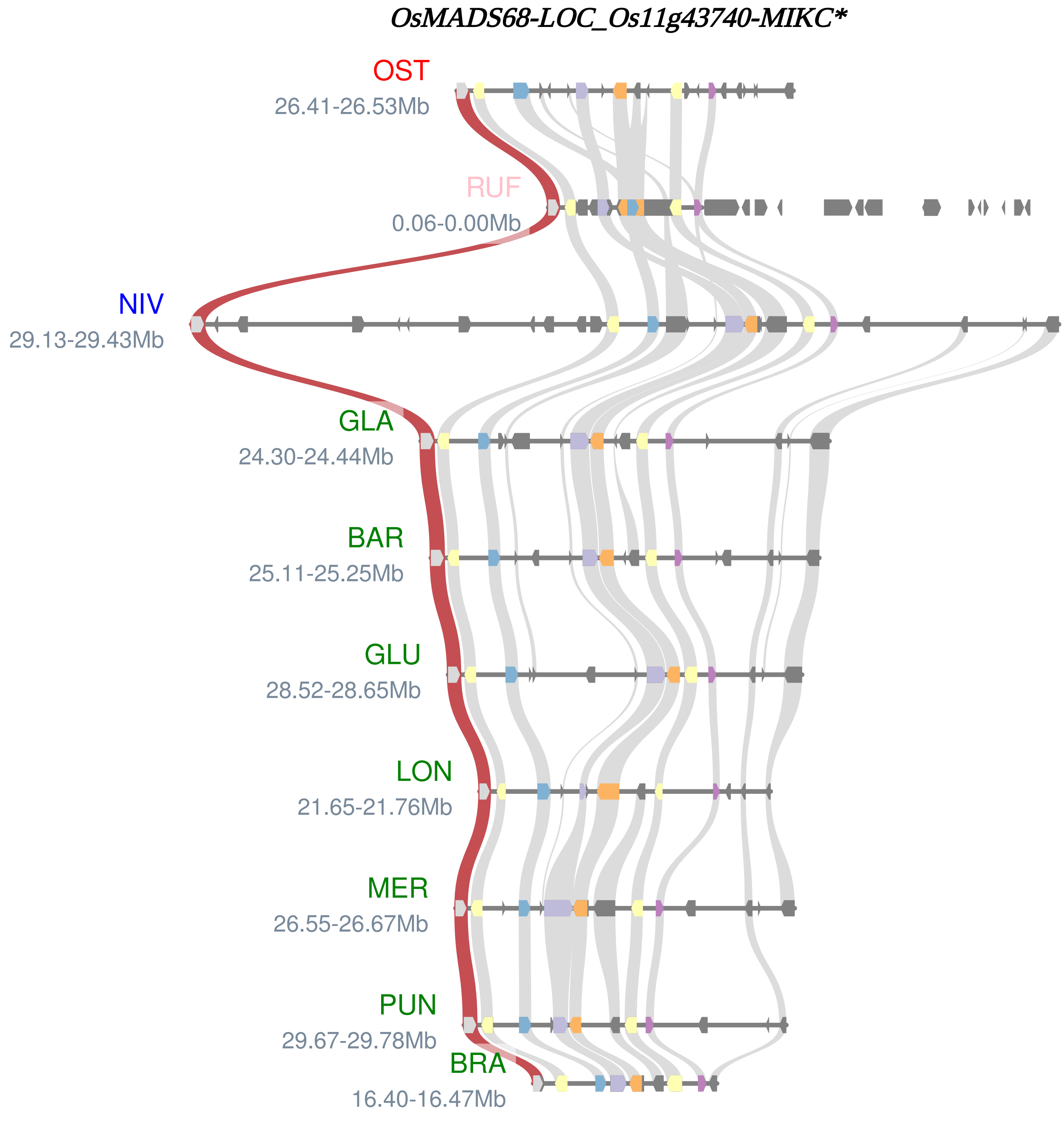

OsMADS68-LOC_Os11g43740-MIKC*
OST
26.41-26.53Mb
RUF
0.06-0.00Mb
NIV
29.13-29.43Mb
GLA
24.30-24.44Mb
BAR
25.11-25.25Mb
GLU
28.52-28.65Mb
LON
21.65-21.76Mb
MER
26.55-26.67Mb
PUN
29.67-29.78Mb
BRA
16.40-16.47Mb

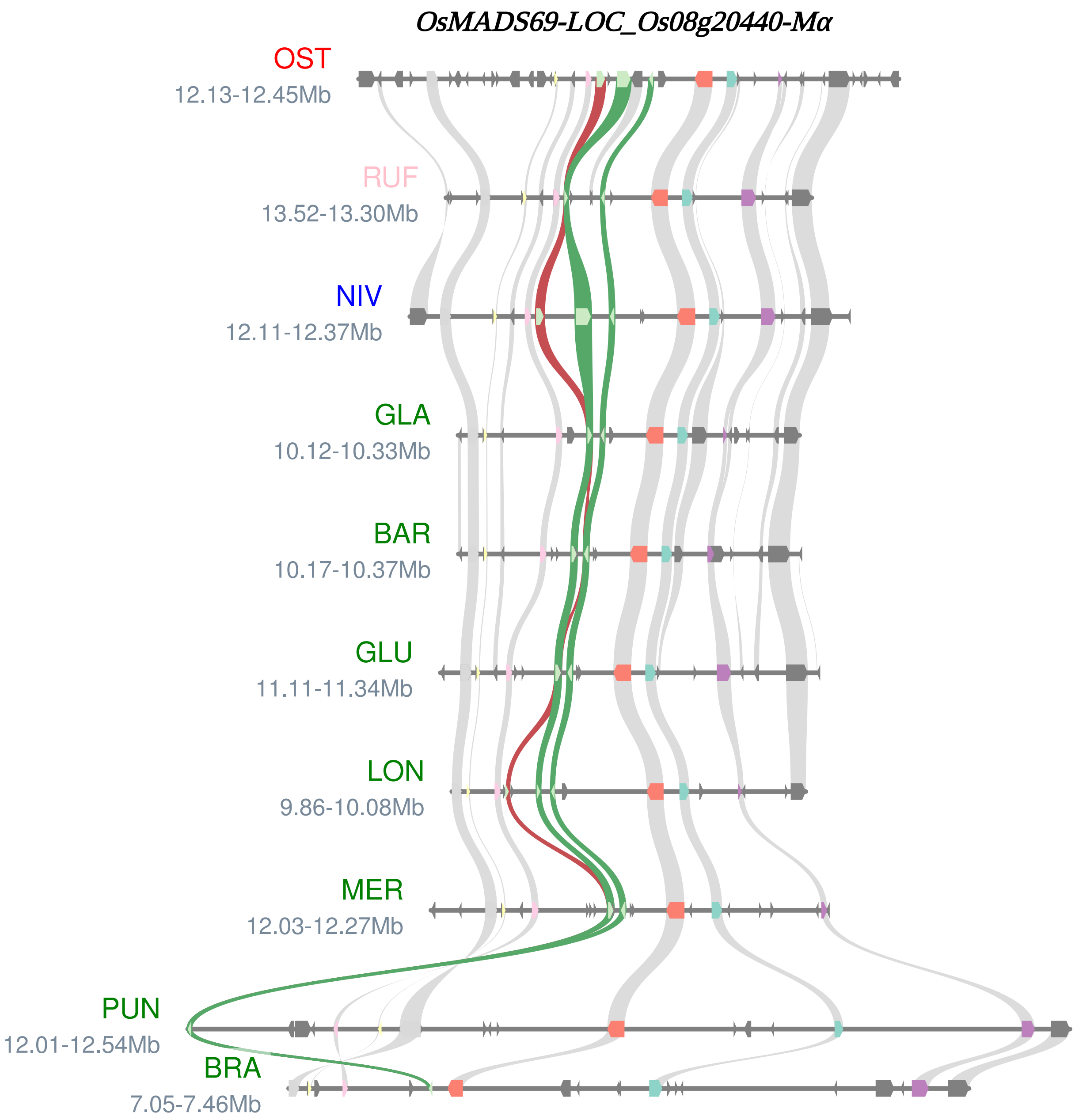
OsMADS69-LOC_Os08g20440-Mα
OST
12.13-12.45Mb
RUF
13.52-13.30Mb
NIV
12.11-12.37Mb
GLA
10.12-10.33Mb
BAR
10.17-10.37Mb
GLU
11.11-11.34Mb
LON
9.86-10.08Mb
MER
12.03-12.27Mb
PUN
12.01-12.54Mb
BRA
7.05-7.46Mb

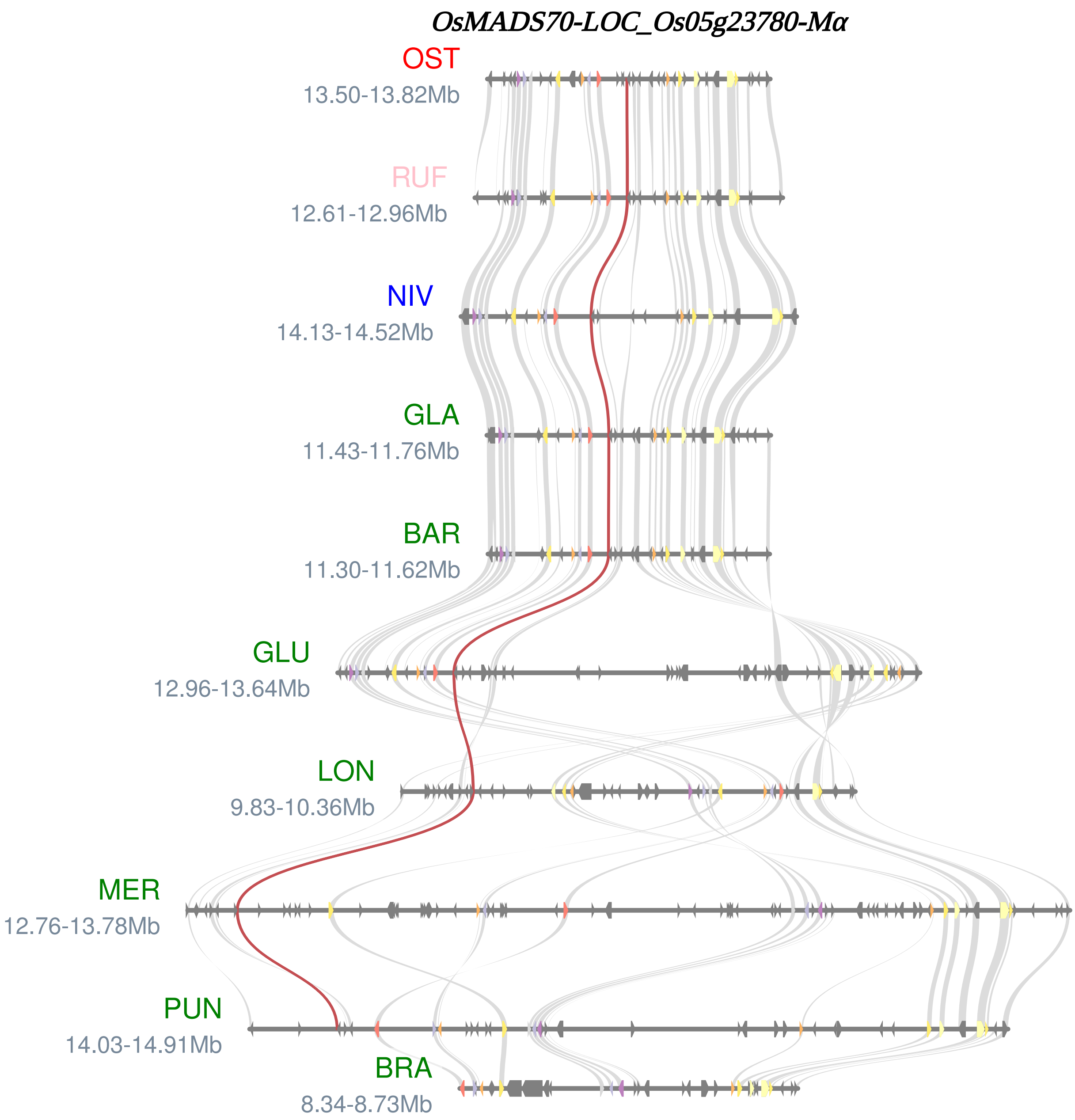

OsMADS70-LOC_Os05g23780-Mα
OST
13.50-13.82Mb
RUF
12.61-12.96Mb
NIV
14.13-14.52Mb
GLA
11.43-11.76Mb
BAR
11.30-11.62Mb
GLU
12.96-13.64Mb
LON
9.83-10.36Mb
MER
12.76-13.78Mb
PUN
14.03-14.91Mb
BRA
8.34-8.73Mb

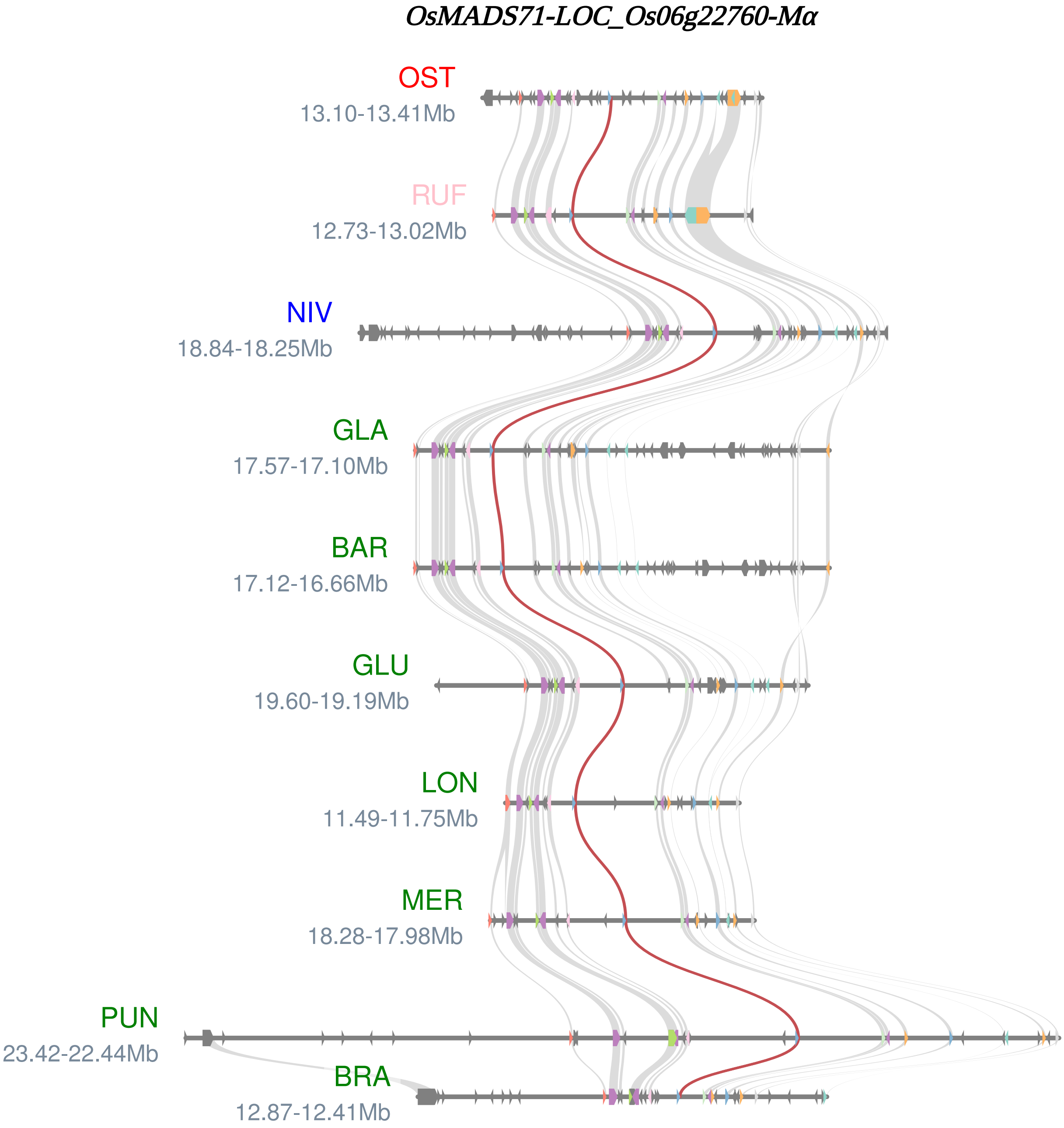

OsMADS71-LOC_Os06g22760-Mα
OST
13.10-13.41Mb
RUF
12.73-13.02Mb
NIV
18.84-18.25Mb
GLA
17.57-17.10Mb
BAR
17.12-16.66Mb
GLU
19.60-19.19Mb
LON
11.49-11.75Mb
MER
18.28-17.98Mb
PUN
23.42-22.44Mb
BRA
12.87-12.41Mb

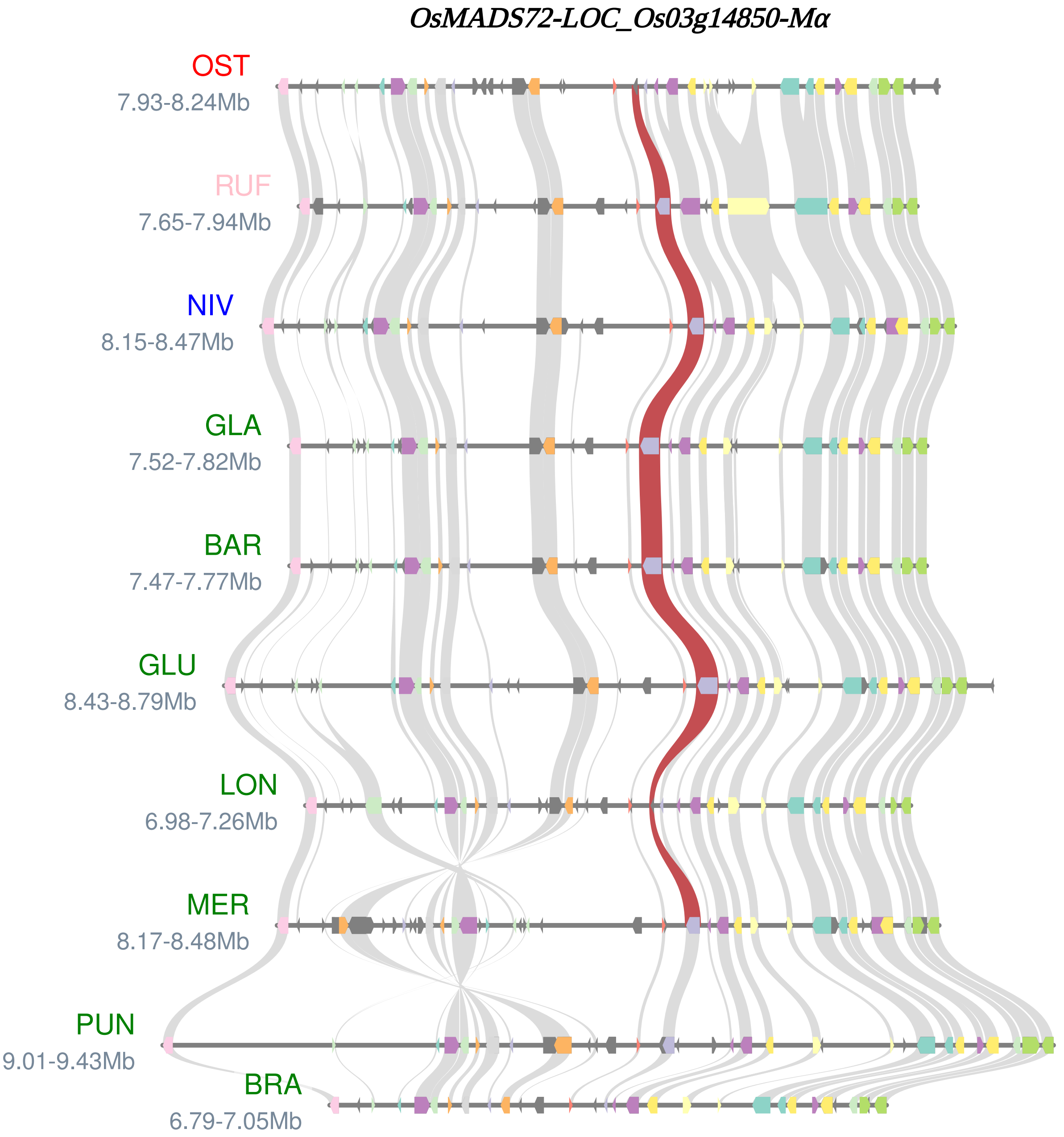

OsMADS72-LOC_Os03g14850-Mα
OST
7.93-8.24Mb
RUF
7.65-7.94Mb
NIV
8.15-8.47Mb
GLA
7.52-7.82Mb
BAR
7.47-7.77Mb
GLU
8.43-8.79Mb
LON
6.98-7.26Mb
MER
8.17-8.48Mb
PUN
9.01-9.43Mb
BRA
6.79-7.05Mb

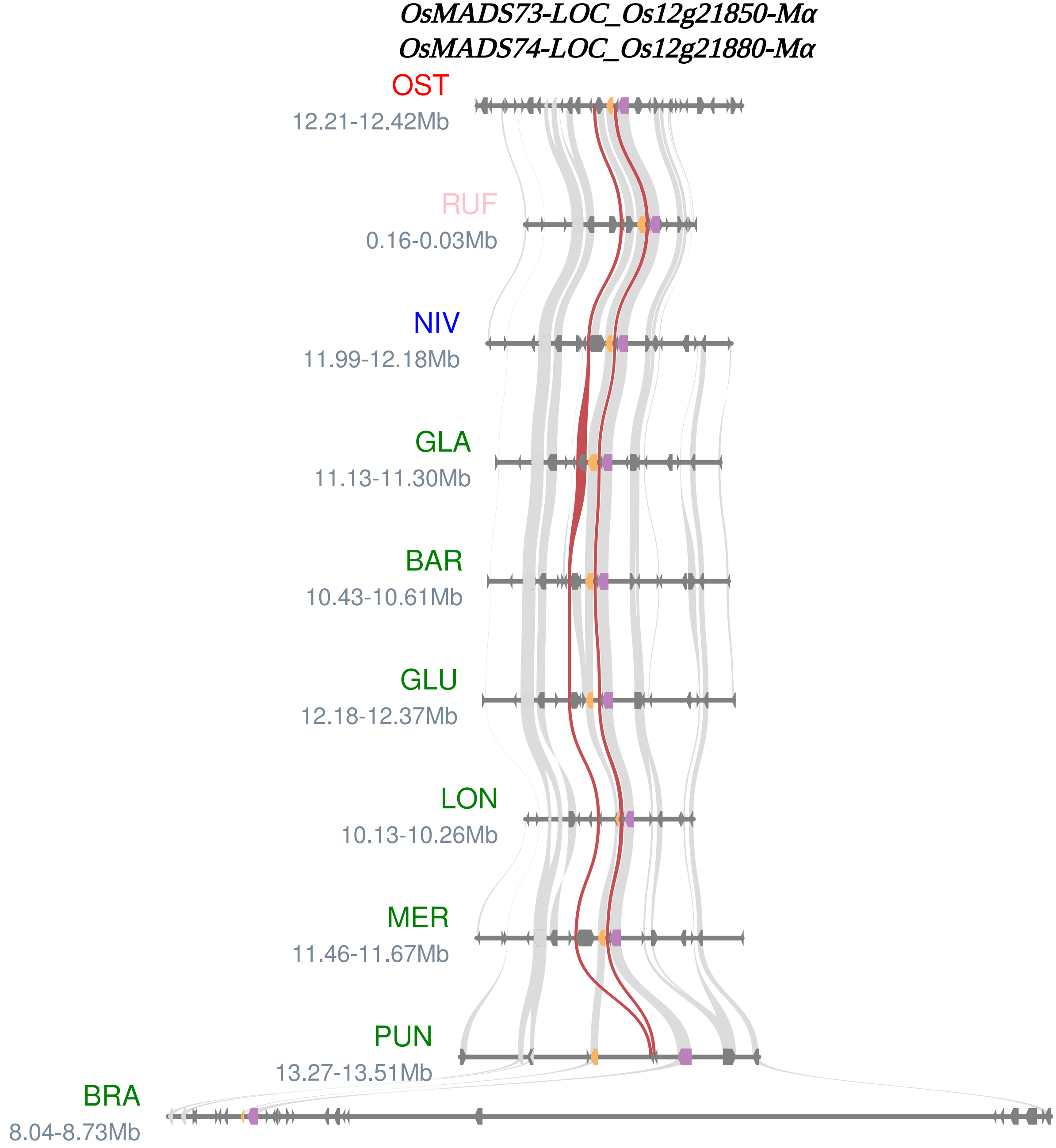

OsMADS73-LOC_Os12g21850-Mα
OsMADS74-LOC_Os12g21880-Mα
OST
12.21-12.42Mb
RUF
0.16-0.03Mb
NIV
11.99-12.18Mb
GLA
11.13-11.30Mb
BAR
10.43-10.61Mb
GLU
12.18-12.37Mb
LON
10.13-10.26Mb
MER
11.46-11.67Mb
PUN
13.27-13.51Mb
BRA
8.04-8.73Mb

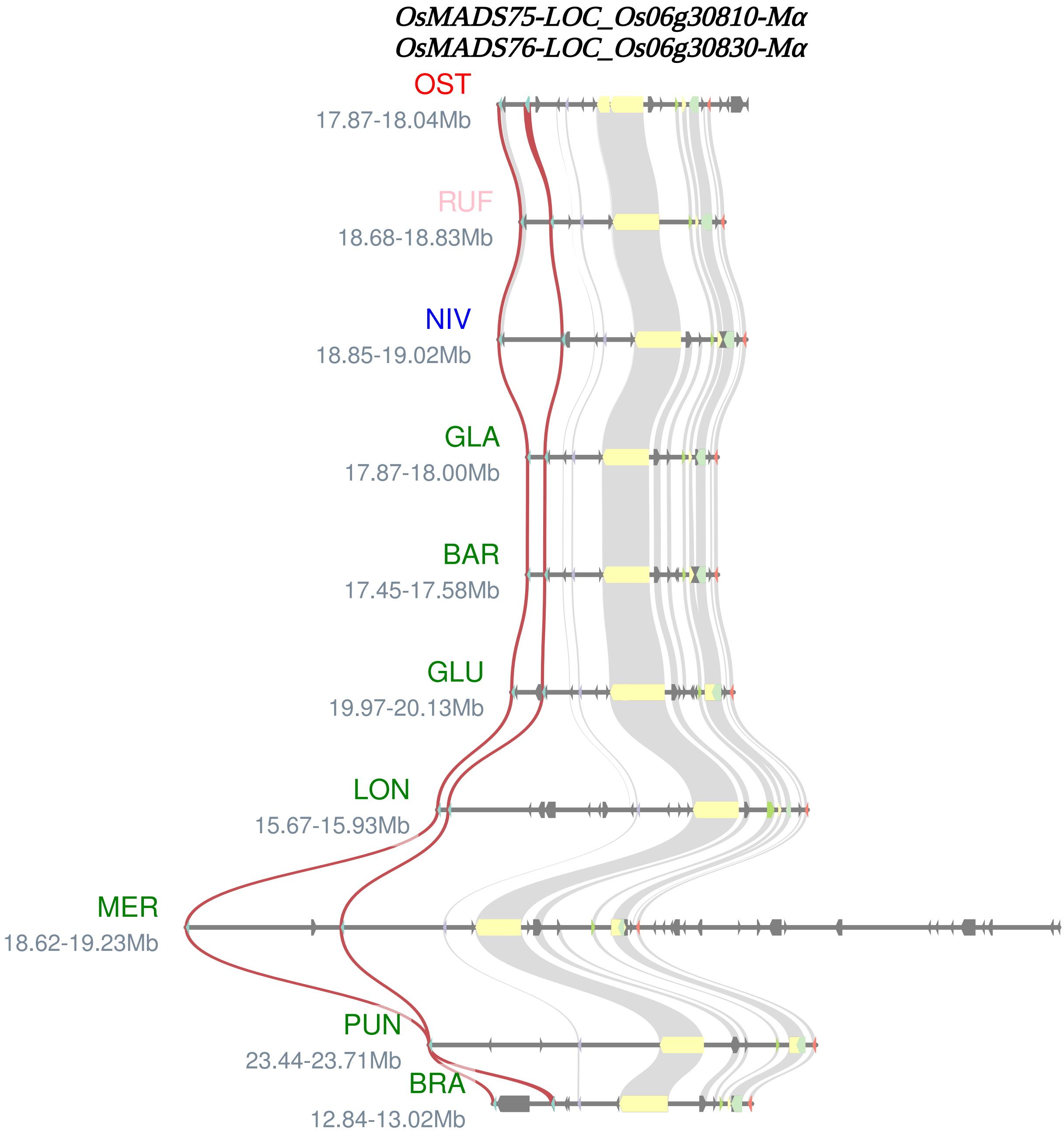

OsMADS75-LOC_Os06g30810-Mα
OsMADS76-LOC_Os06g30830-Mα
OST
17.87-18.04Mb
RUF
18.68-18.83Mb
NIV
18.85-19.02Mb
GLA
17.87-18.00Mb
BAR
17.45-17.58Mb
GLU
19.97-20.13Mb
LON
15.67-15.93Mb
MER
18.62-19.23Mb
PUN
23.44-23.71Mb
BRA
12.84-13.02Mb

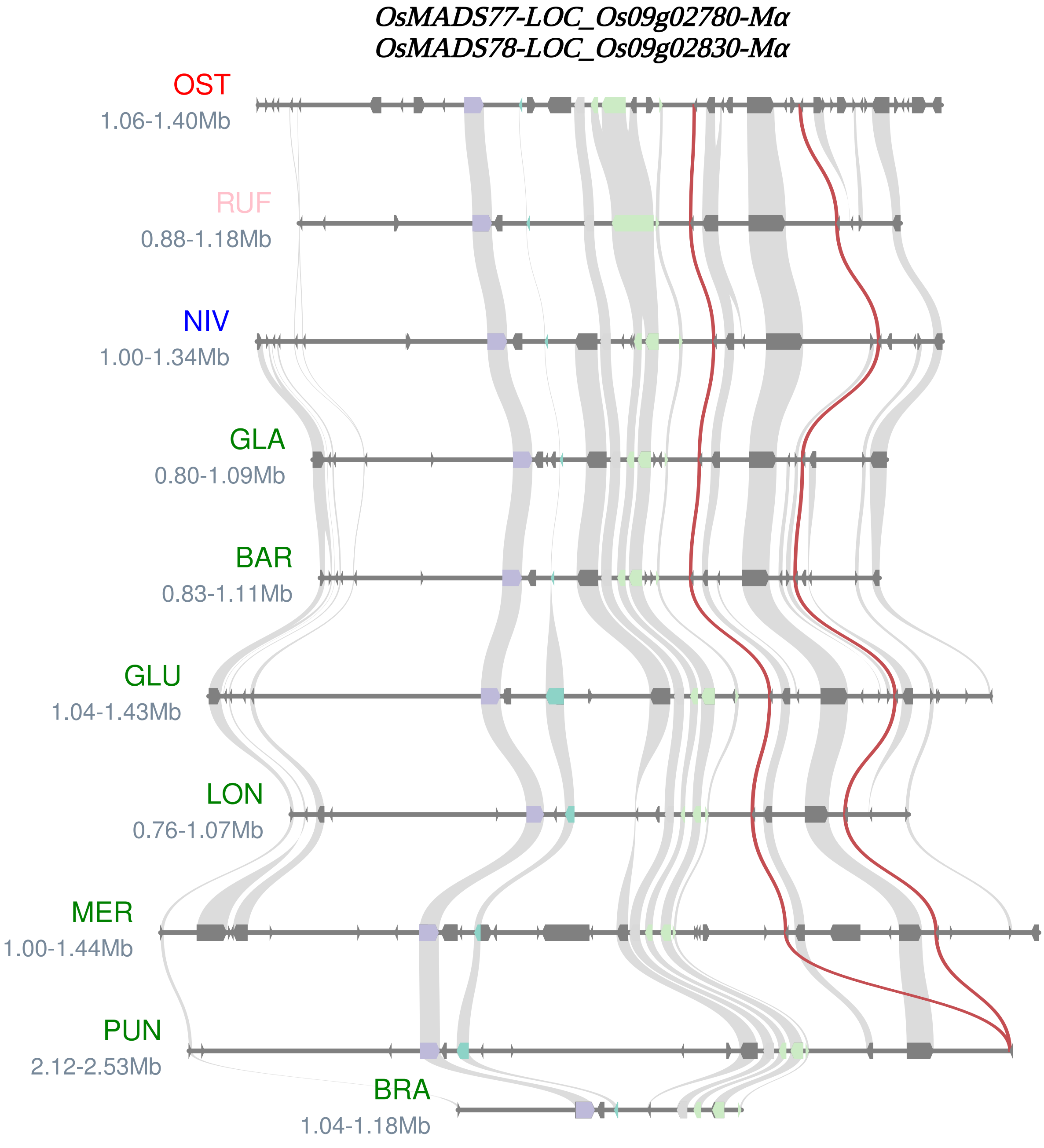

OsMADS77-LOC_Os09g02780-Mα
OsMADS78-LOC_Os09g02830-Mα
OST
1.06-1.40Mb
RUF
0.88-1.18Mb
NIV
1.00-1.34Mb
GLA
0.80-1.09Mb
BAR
0.83-1.11Mb
GLU
1.04-1.43Mb
LON
0.76-1.07Mb
MER
1.00-1.44Mb
PUN
2.12-2.53Mb
BRA
1.04-1.18Mb

OsMADS79-LOC_Os01g74440-Mα
(The chromosomal segment in the MER lacks any detected syntenic genes.)

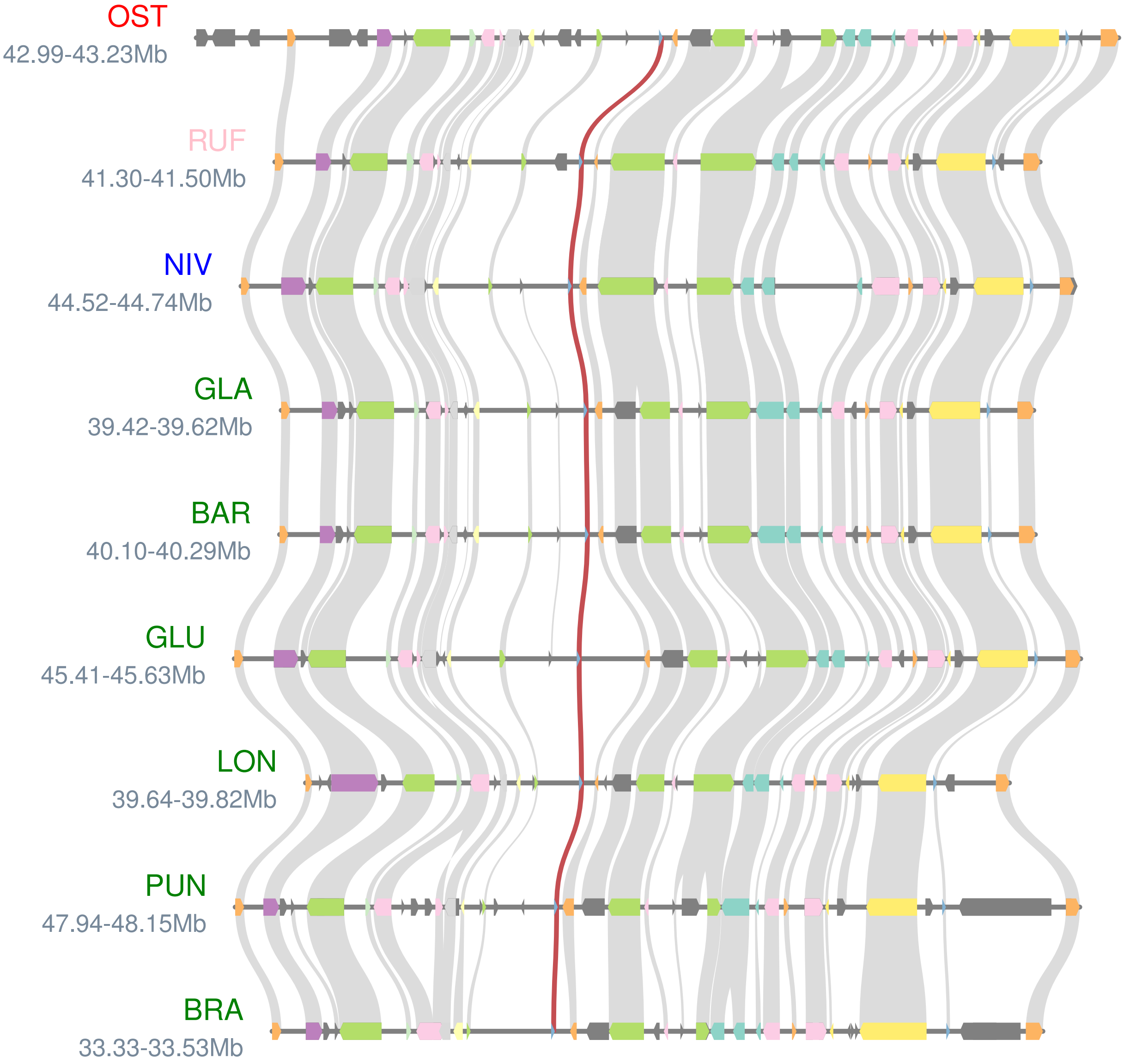

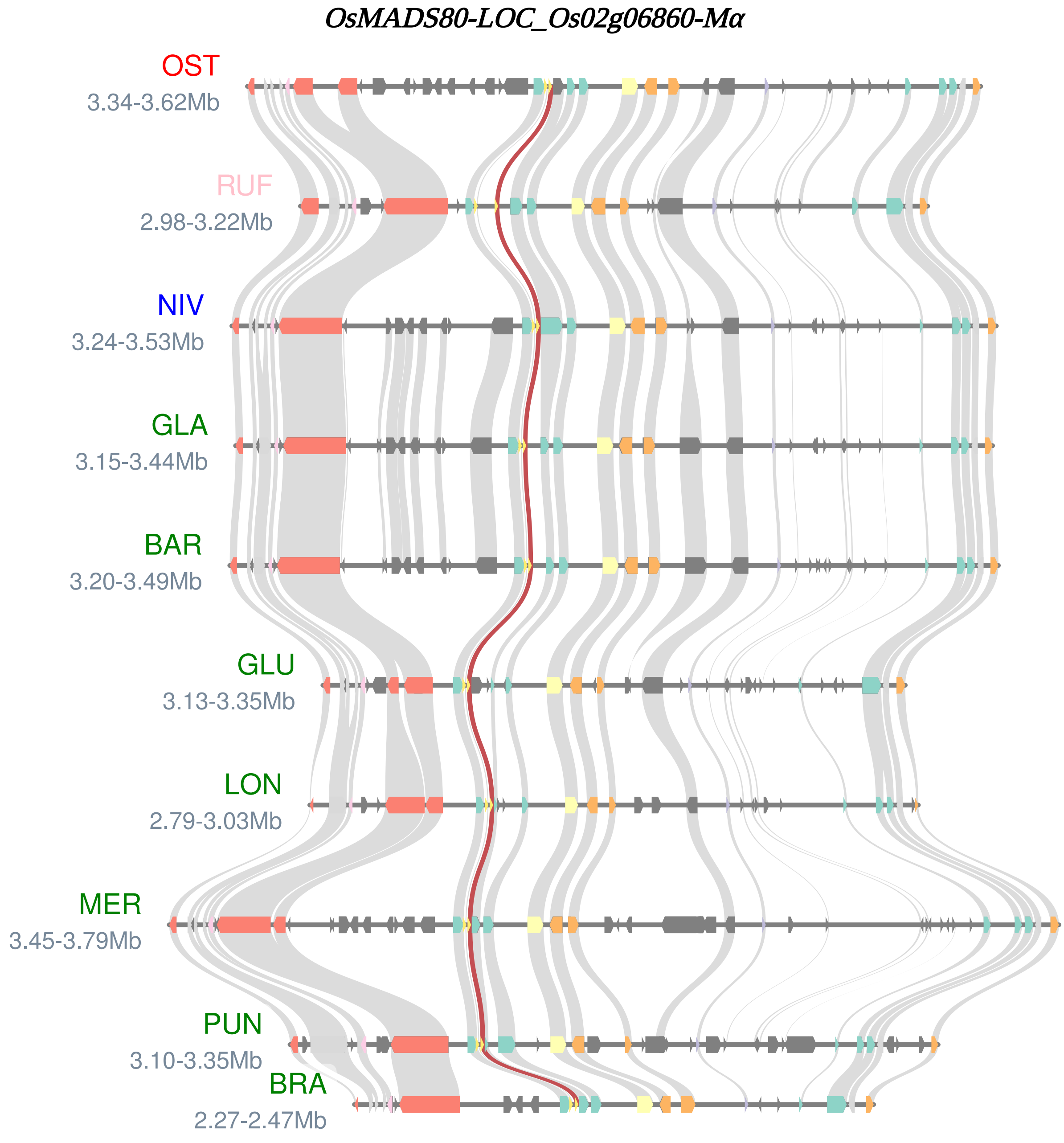
OsMADS80-LOC_Os02g06860-Mα
OST
3.34-3.62Mb
RUF
2.98-3.22Mb
NIV
3.24-3.53Mb
GLA
3.15-3.44Mb
BAR
3.20-3.49Mb
GLU
3.13-3.35Mb
LON
2.79-3.03Mb
MER
3.45-3.79Mb
PUN
3.10-3.35Mb
BRA
2.27-2.47Mb

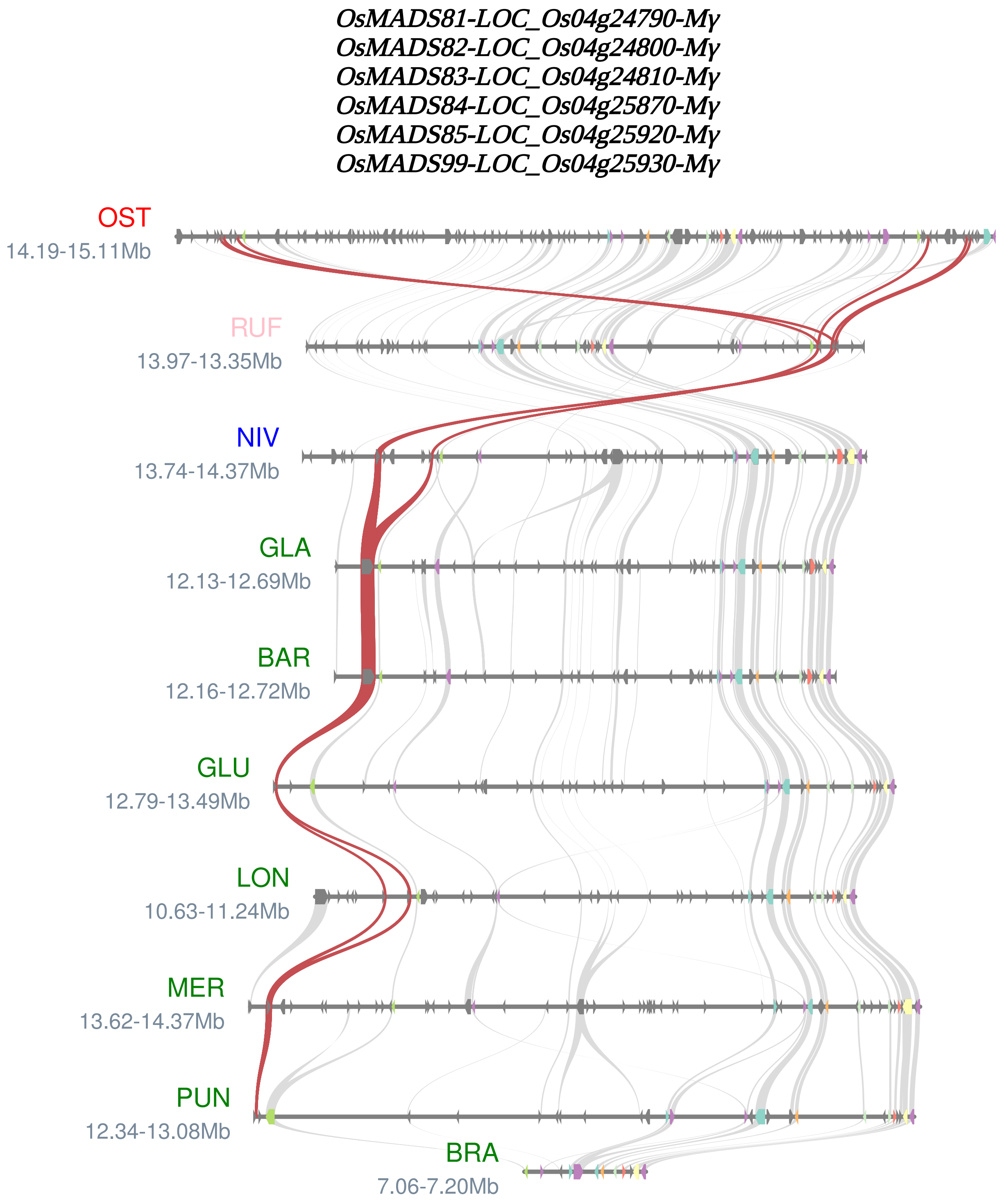

OsMADS81-LOC_Os04g24790-Mγ
OsMADS82-LOC_Os04g24800-Mγ
OsMADS83-LOC_Os04g24810-Mγ
OsMADS84-LOC_Os04g25870-Mγ
OsMADS85-LOC_Os04g25920-Mγ
OsMADS99-LOC_Os04g25930-Mγ
OST
14.19-15.11Mb
RUF
13.97-13.35Mb
NIV
13.74-14.37Mb
GLA
12.13-12.69Mb
BAR
12.16-12.72Mb
GLU
12.79-13.49Mb
LON
10.63-11.24Mb
MER
13.62-14.37Mb
PUN
12.34-13.08Mb
BRA
7.06-7.20Mb

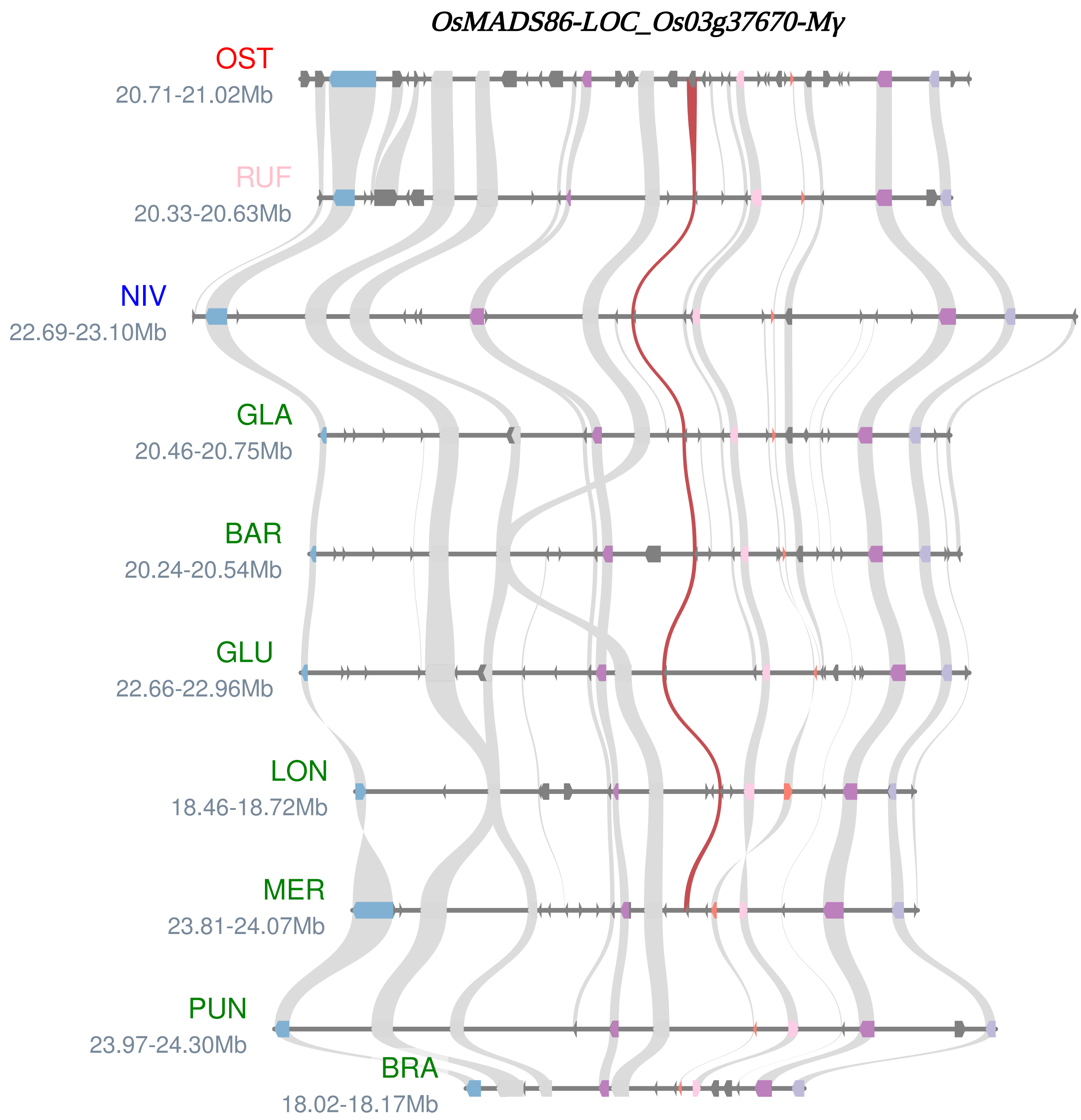

OsMADS86-LOC_Os03g37670-Mγ
OST
20.71-21.02Mb
RUF
20.33-20.63Mb
NIV
22.69-23.10Mb
GLA
20.46-20.75Mb
BAR
20.24-20.54Mb
GLU
22.66-22.96Mb
LON
18.46-18.72Mb
MER
23.81-24.07Mb
PUN
23.97-24.30Mb
BRA
18.02-18.17Mb

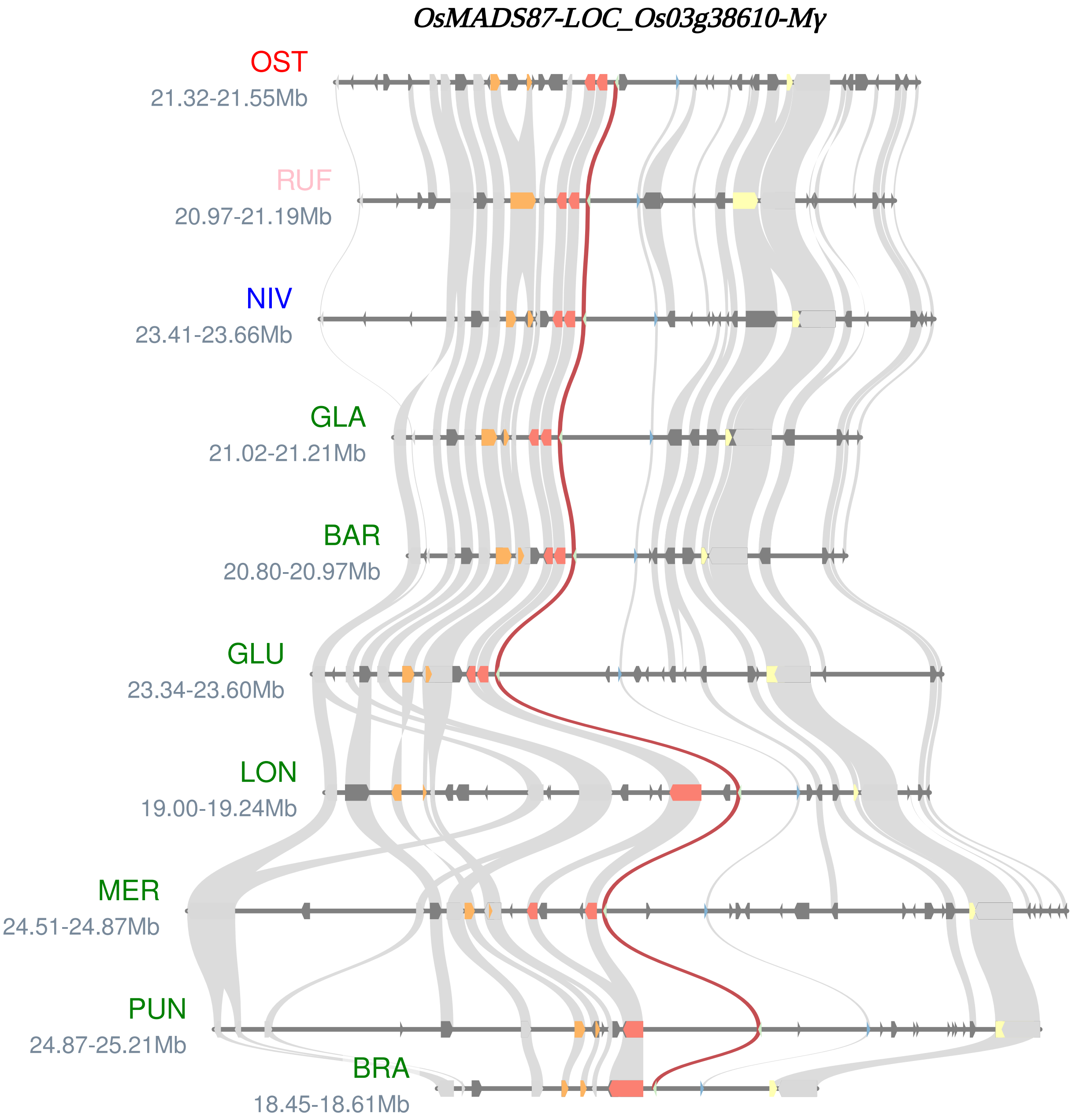

OsMADS87-LOC_Os03g38610-Mγ
OST
21.32-21.55Mb
RUF
20.97-21.19Mb
NIV
23.41-23.66Mb
GLA
21.02-21.21Mb
BAR
20.80-20.97Mb
GLU
23.34-23.60Mb
LON
19.00-19.24Mb
MER
24.51-24.87Mb
PUN
24.87-25.21Mb
BRA
18.45-18.61Mb

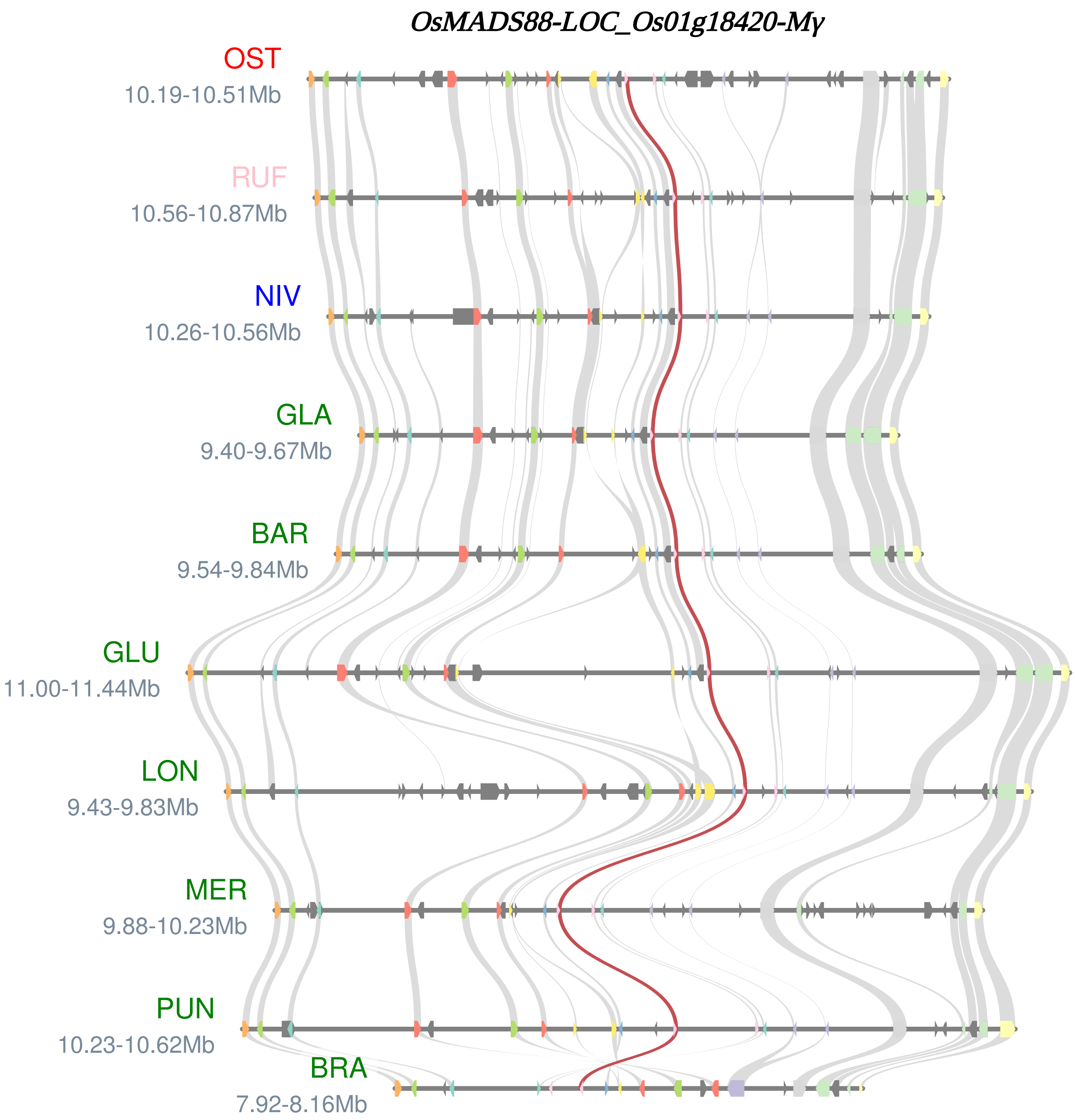
OsMADS88-LOC_Os01g18420-Mγ
OST
10.19-10.51Mb
RUF
10.56-10.87Mb
NIV
10.26-10.56Mb
GLA
9.40-9.67Mb
BAR
9.54-9.84Mb
GLU
11.00-11.44Mb
LON
9.43-9.83Mb
MER
9.88-10.23Mb
PUN
10.23-10.62Mb
BRA
7.92-8.16Mb

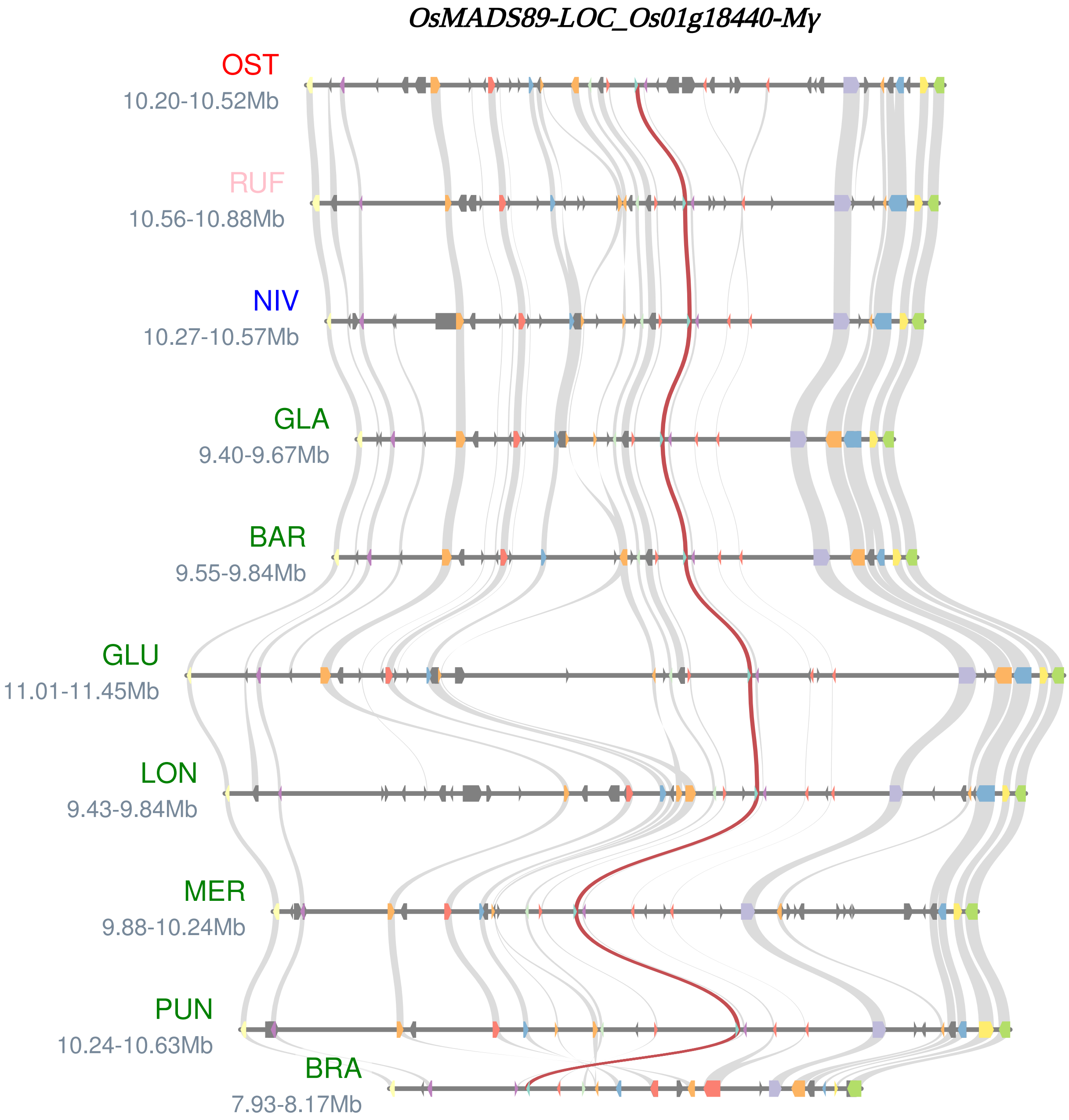

OsMADS89-LOC_Os01g18440-Mγ
OST
10.20-10.52Mb
RUF
10.56-10.88Mb
NIV
10.27-10.57Mb
GLA
9.40-9.67Mb
BAR
9.55-9.84Mb
GLU
11.01-11.45Mb
LON
9.43-9.84Mb
MER
9.88-10.24Mb
PUN
10.24-10.63Mb
BRA
7.93-8.17Mb

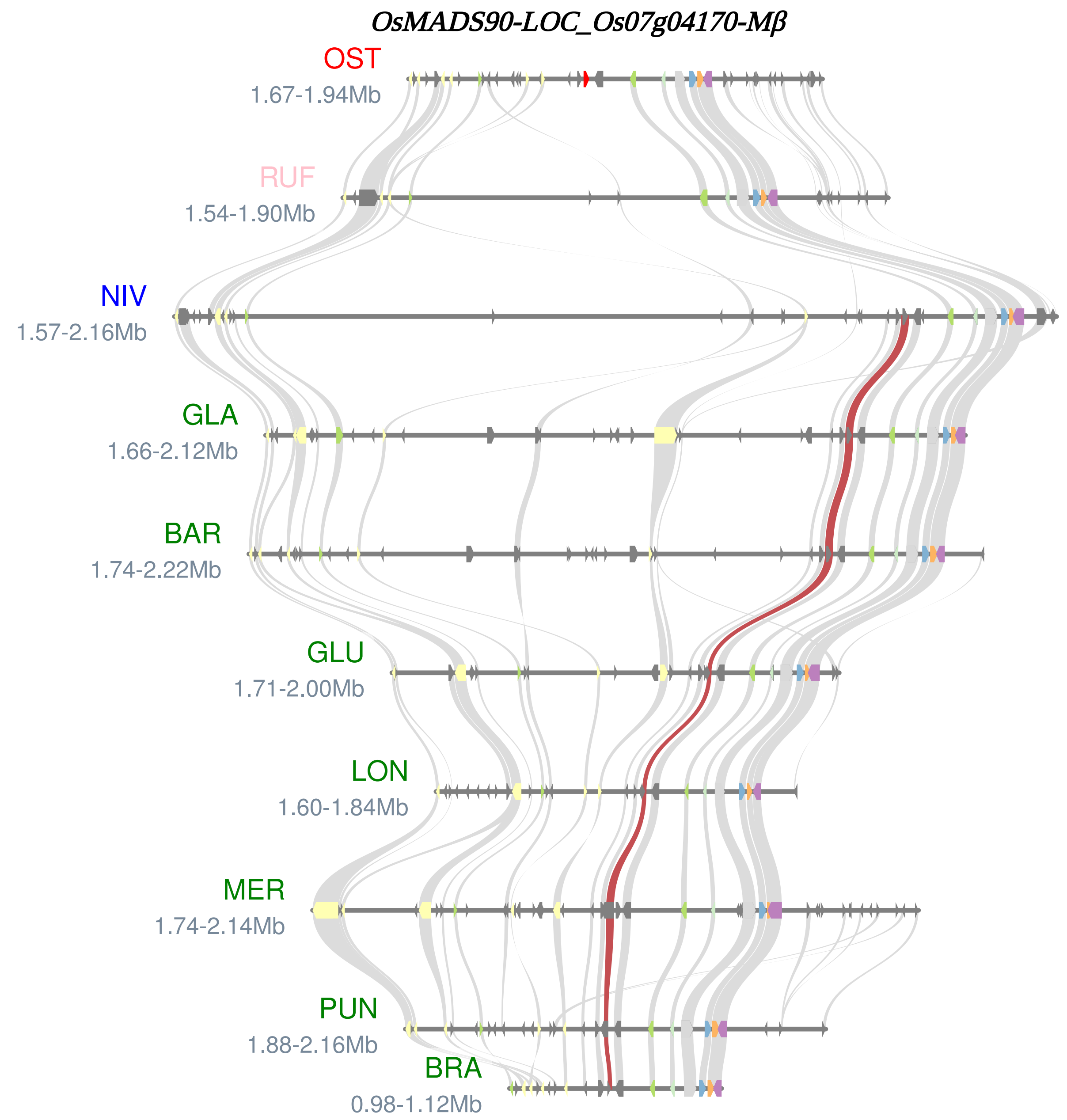

OsMADS90-LOC_Os07g04170-Mβ
OST
1.67-1.94Mb
RUF
1.54-1.90Mb
NIV
1.57-2.16Mb
GLA
1.66-2.12Mb
BAR
1.74-2.22Mb
GLU
1.71-2.00Mb
LON
1.60-1.84Mb
MER
1.74-2.14Mb
PUN
1.88-2.16Mb
BRA
0.98-1.12Mb

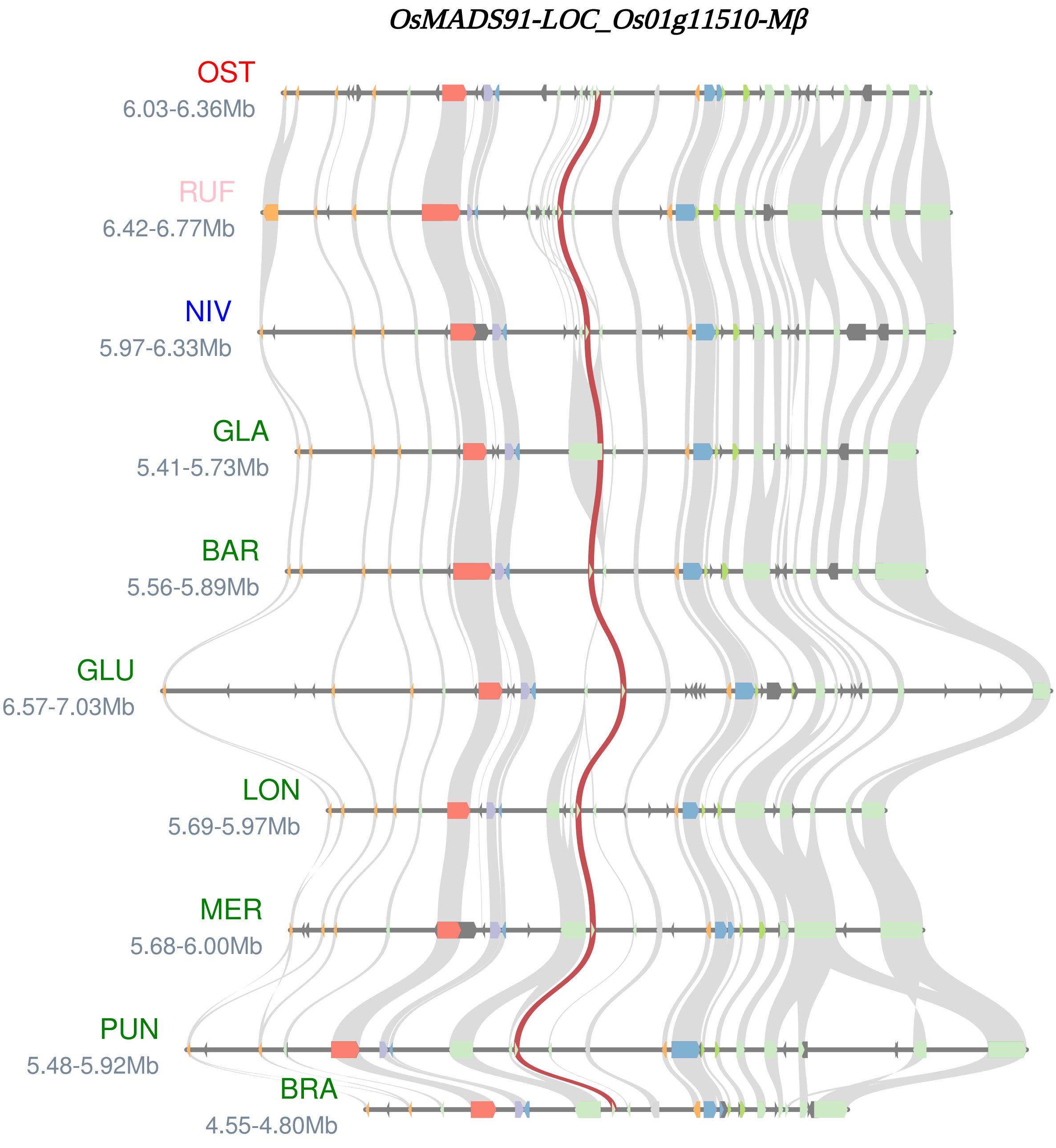
OsMADS91-LOC_Os01g11510-Mβ
OST
6.03-6.36Mb
RUF
6.42-6.77Mb
NIV
5.97-6.33Mb
GLA
5.41-5.73Mb
BAR
5.56-5.89Mb
GLU
6.57-7.03Mb
LON
5.69-5.97Mb
MER
5.68-6.00Mb
PUN
5.48-5.92Mb
BRA
4.55-4.80Mb

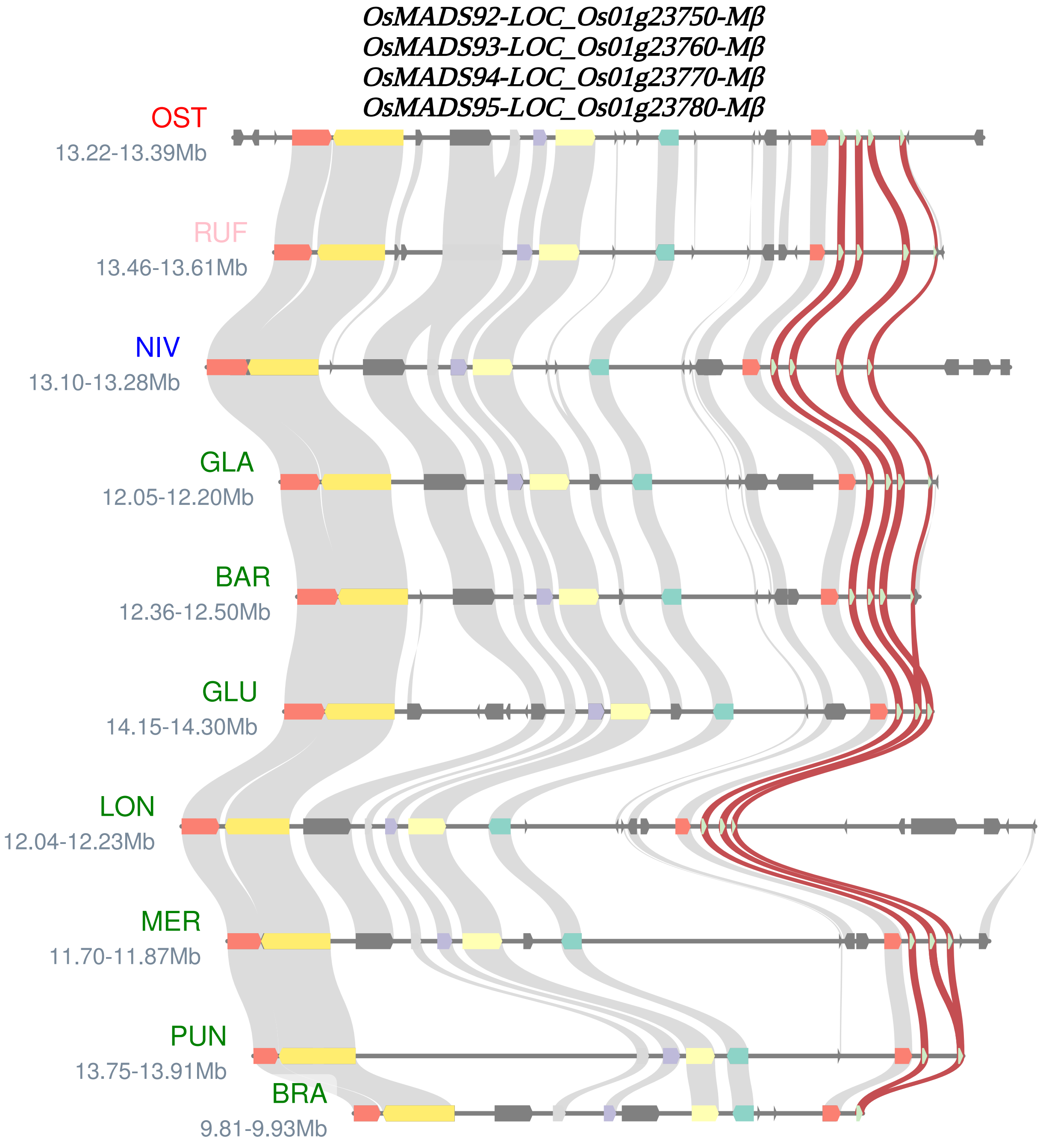

OsMADS92-LOC_Os01g23750-Mβ
OsMADS93-LOC_Os01g23760-Mβ
OsMADS94-LOC_Os01g23770-Mβ
OsMADS95-LOC_Os01g23780-Mβ
OST
13.22-13.39Mb
RUF
13.46-13.61Mb
NIV
13.10-13.28Mb
GLA
12.05-12.20Mb
BAR
12.36-12.50Mb
GLU
14.15-14.30Mb
LON
12.04-12.23Mb
MER
11.70-11.87Mb
PUN
13.75-13.91Mb
BRA
9.81-9.93Mb

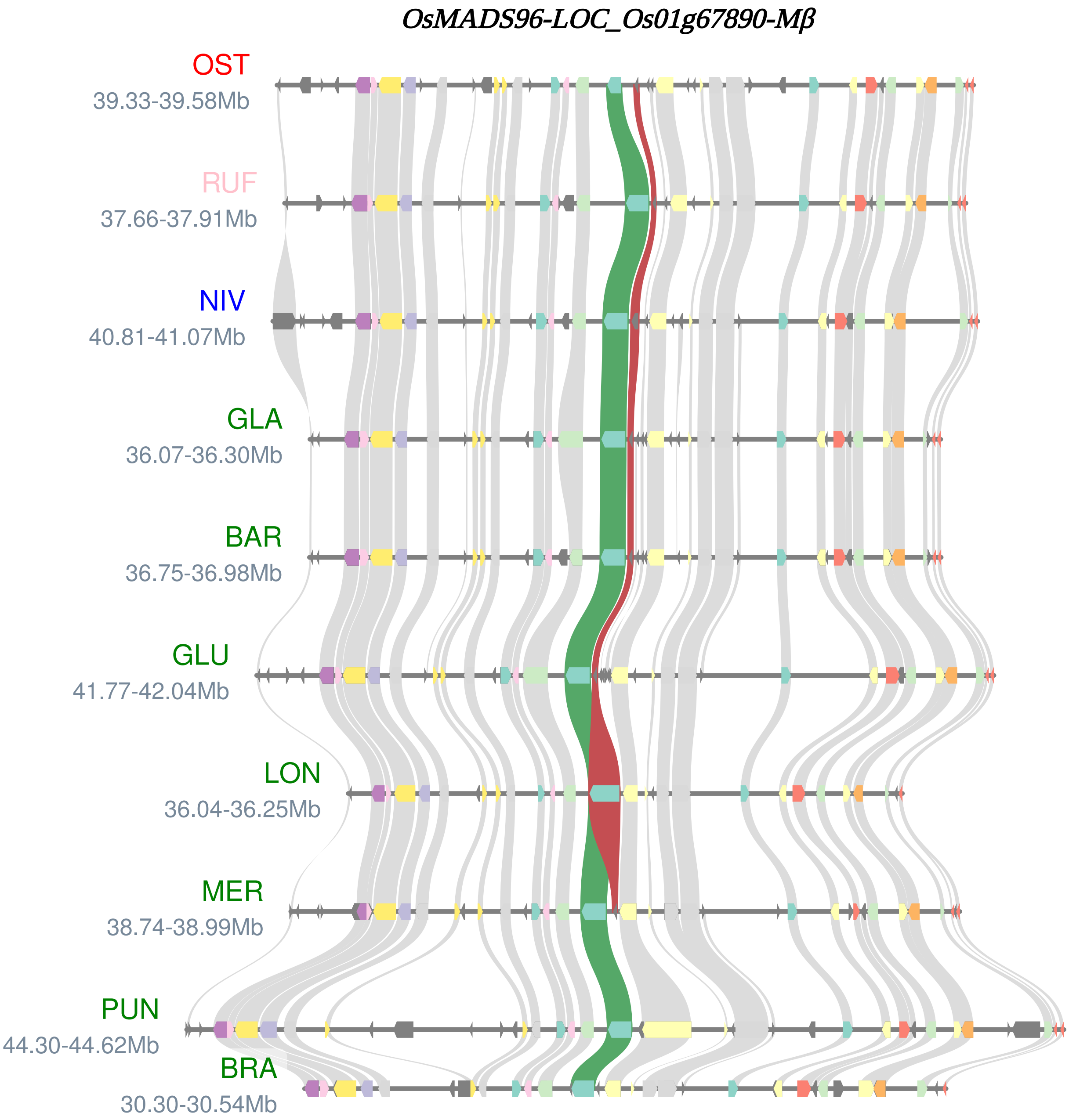

OsMADS96-LOC_Os01g67890-Mβ
OST
39.33-39.58Mb
RUF
37.66-37.91Mb
NIV
40.81-41.07Mb
GLA
36.07-36.30Mb
BAR
36.75-36.98Mb
GLU
41.77-42.04Mb
LON
36.04-36.25Mb
MER
38.74-38.99Mb
PUN
44.30-44.62Mb
BRA
30.30-30.54Mb

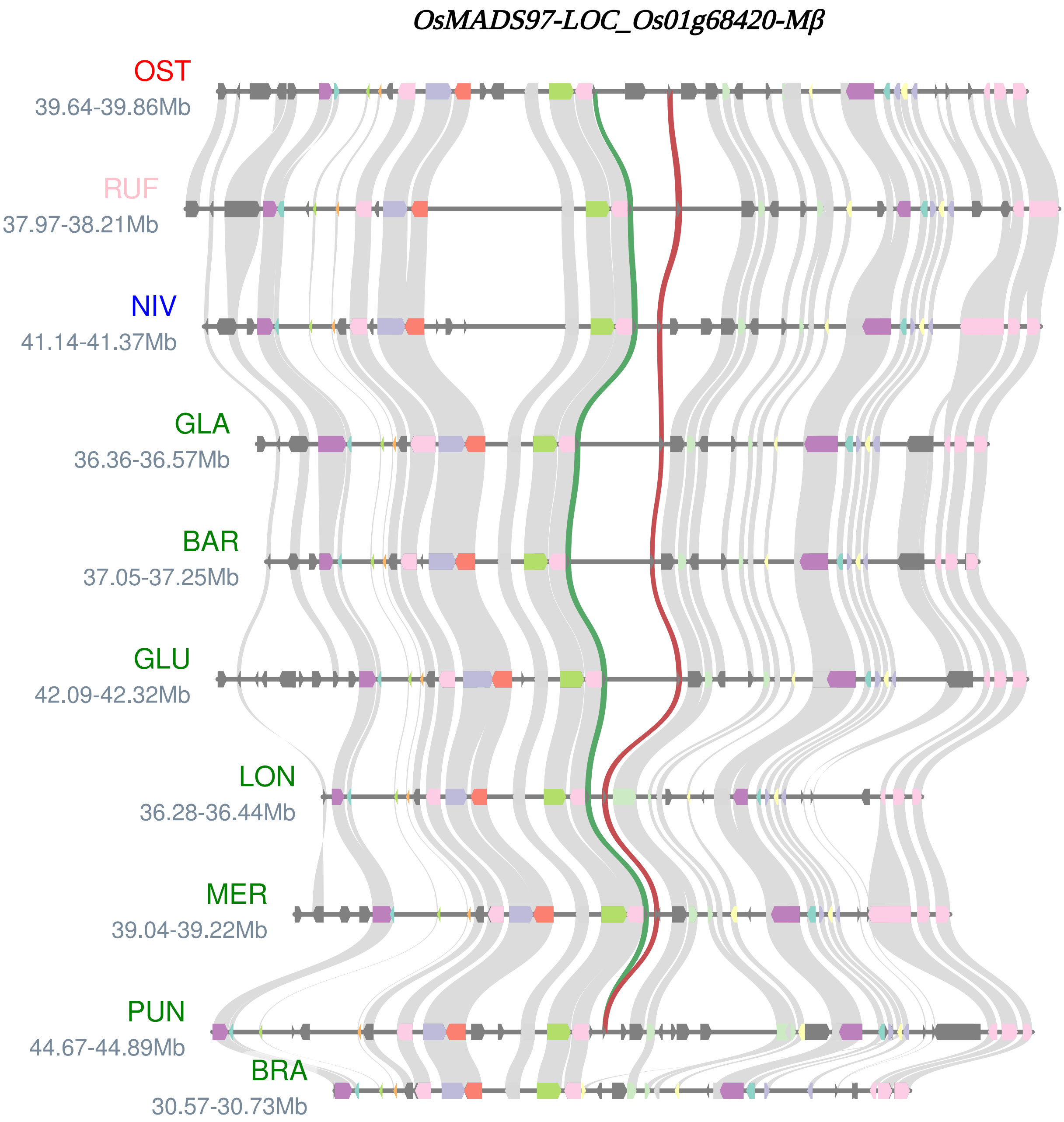

OsMADS97-LOC_Os01g68420-Mβ
OST
39.64-39.86Mb
RUF
37.97-38.21Mb
NIV
41.14-41.37Mb
GLA
36.36-36.57Mb
BAR
37.05-37.25Mb
GLU
42.09-42.32Mb
LON
36.28-36.44Mb
MER
39.04-39.22Mb
PUN
44.67-44.89Mb
BRA
30.57-30.73Mb

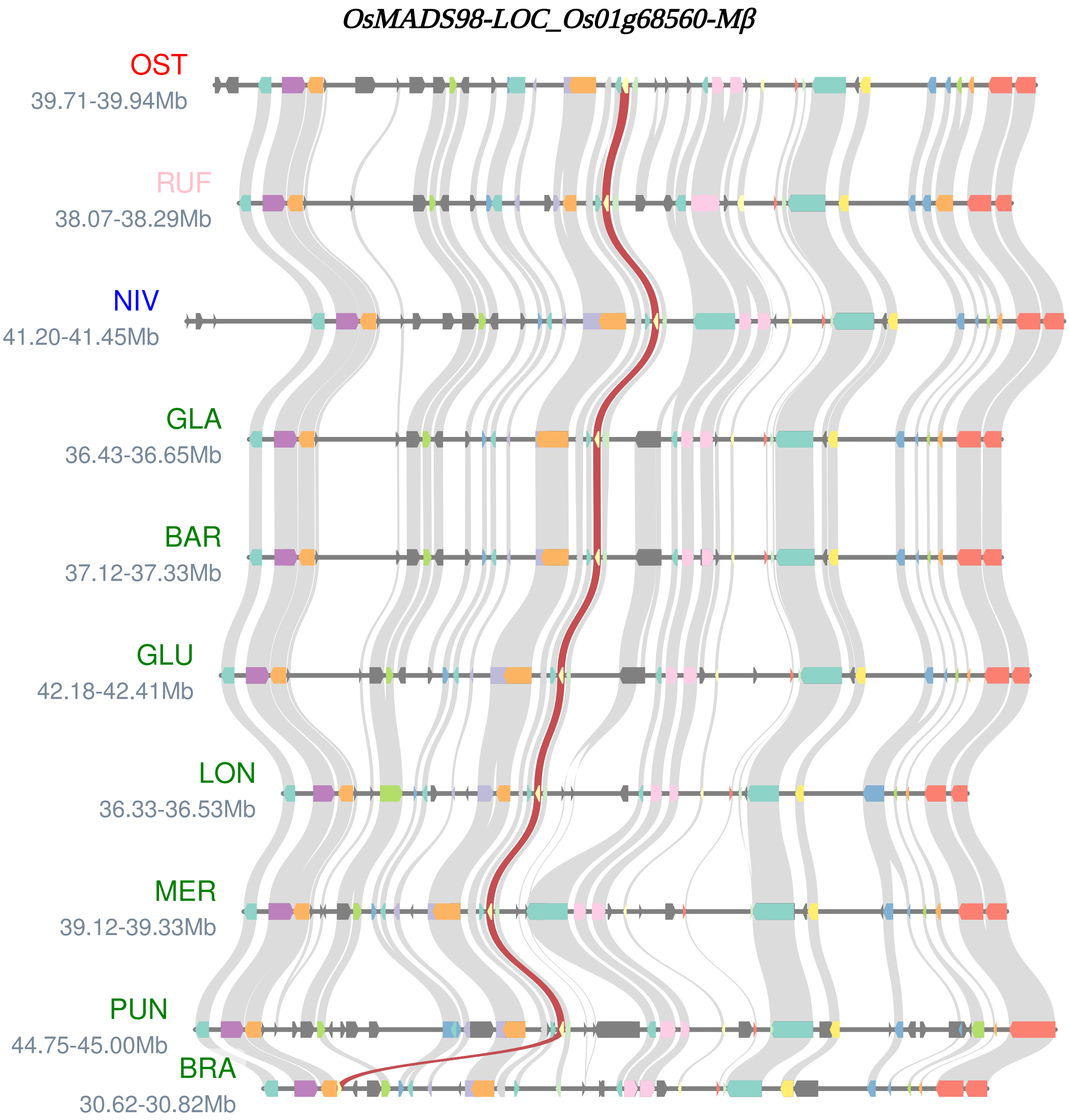

OsMADS98-LOC_Os01g68560-Mβ
OST
39.71-39.94Mb
RUF
38.07-38.29Mb
NIV
41.20-41.45Mb
GLA
36.43-36.65Mb
BAR
37.12-37.33Mb
GLU
42.18-42.41Mb
LON
36.33-36.53Mb
MER
39.12-39.33Mb
PUN
44.75-45.00Mb
BRA
30.62-30.82Mb

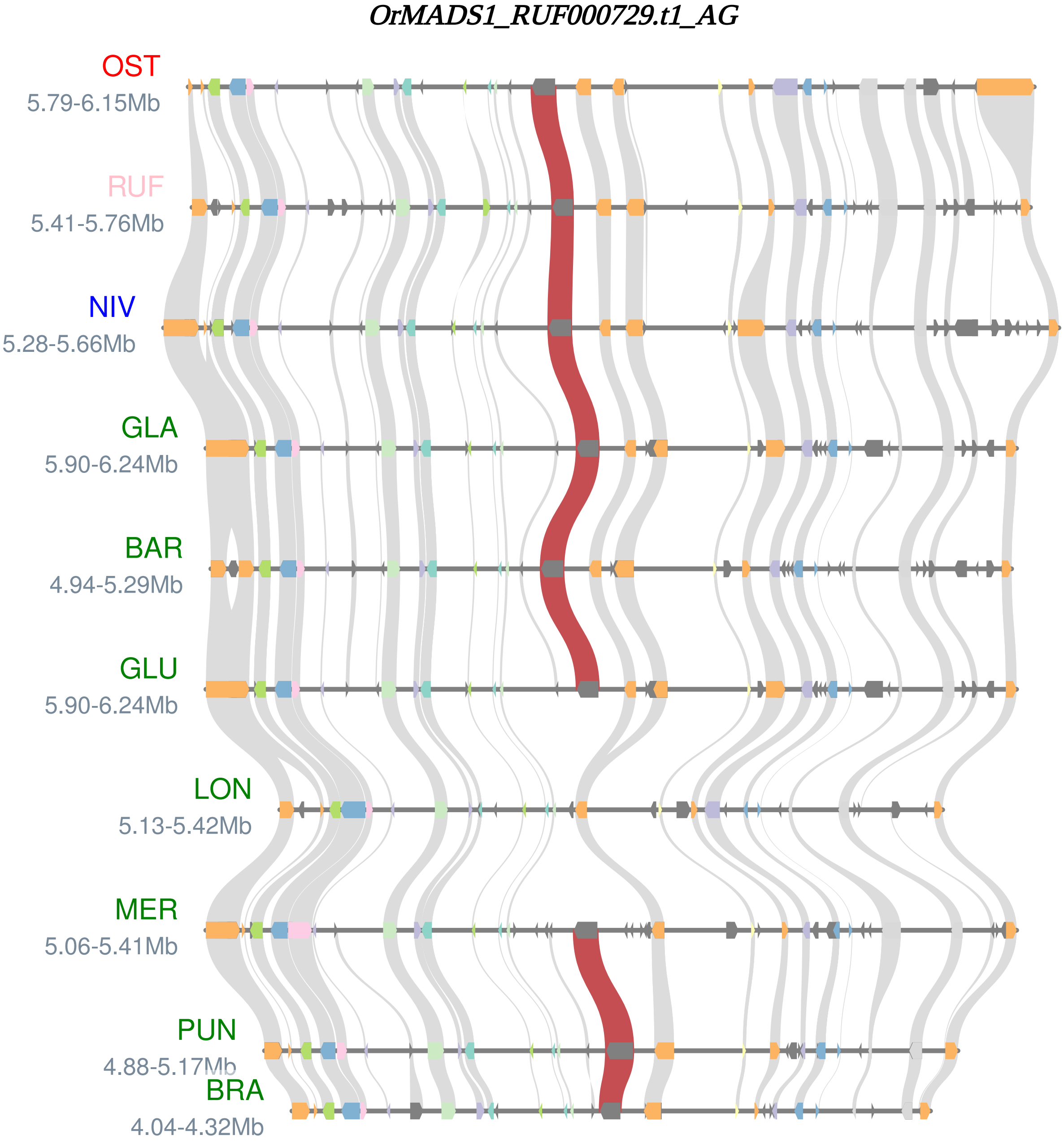
OrMADS1_RUF000729.t1_AG
OST
5.79-6.15Mb
RUF
5.41-5.76Mb
NIV
5.28-5.66Mb
GLA
5.90-6.24Mb
BAR
4.94-5.29Mb
GLU
5.90-6.24Mb
LON
5.13-5.42Mb
MER
5.06-5.41Mb
PUN
4.88-5.17Mb
BRA
4.04-4.32Mb

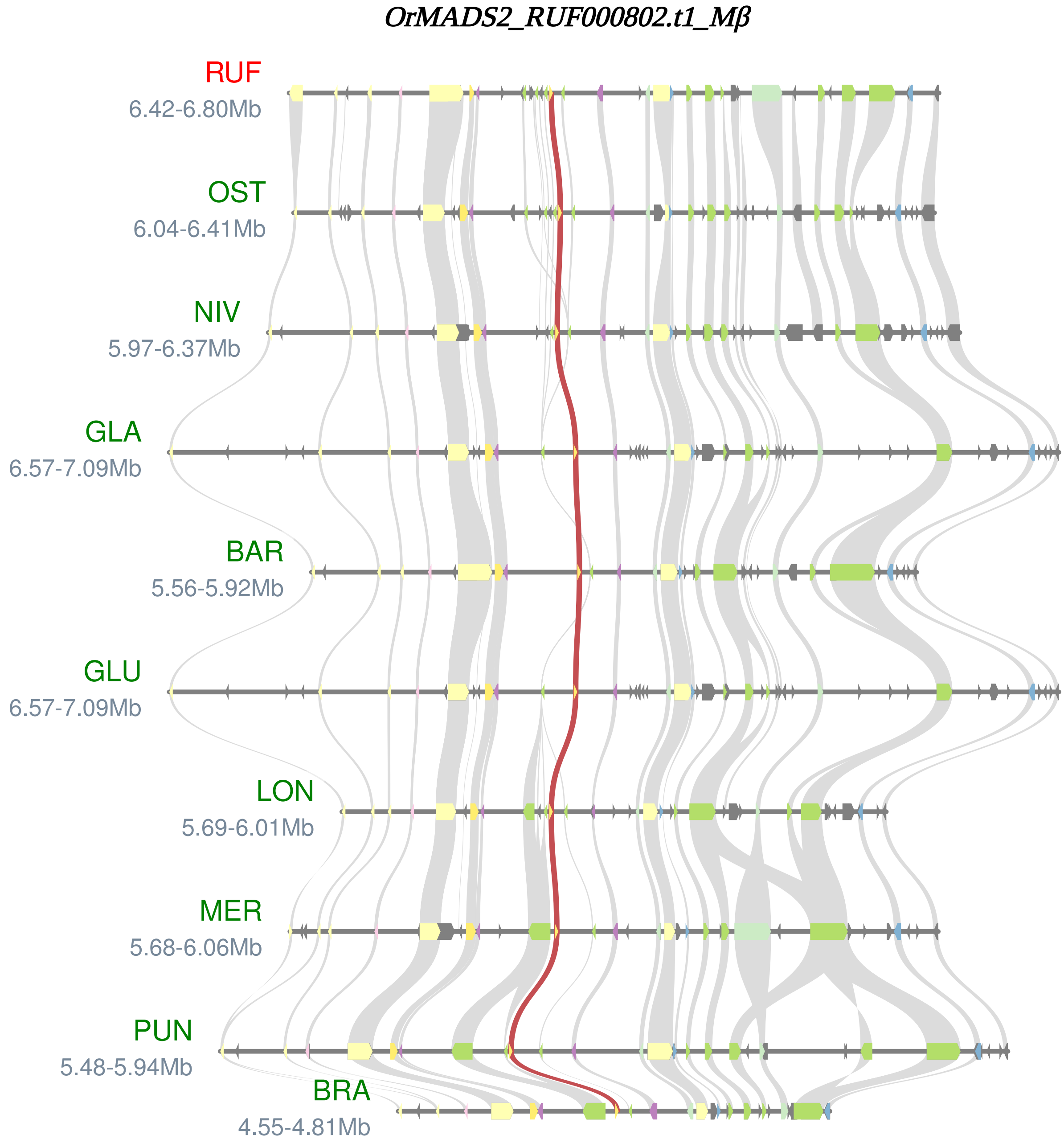

OrMADS2_RUF000802.t1_Mβ
RUF
6.42-6.80Mb
OST
6.04-6.41Mb
NIV
5.97-6.37Mb
GLA
6.57-7.09Mb
BAR
5.56-5.92Mb
GLU
6.57-7.09Mb
LON
5.69-6.01Mb
MER
5.68-6.06Mb
PUN
5.48-5.94Mb
BRA
4.55-4.81Mb

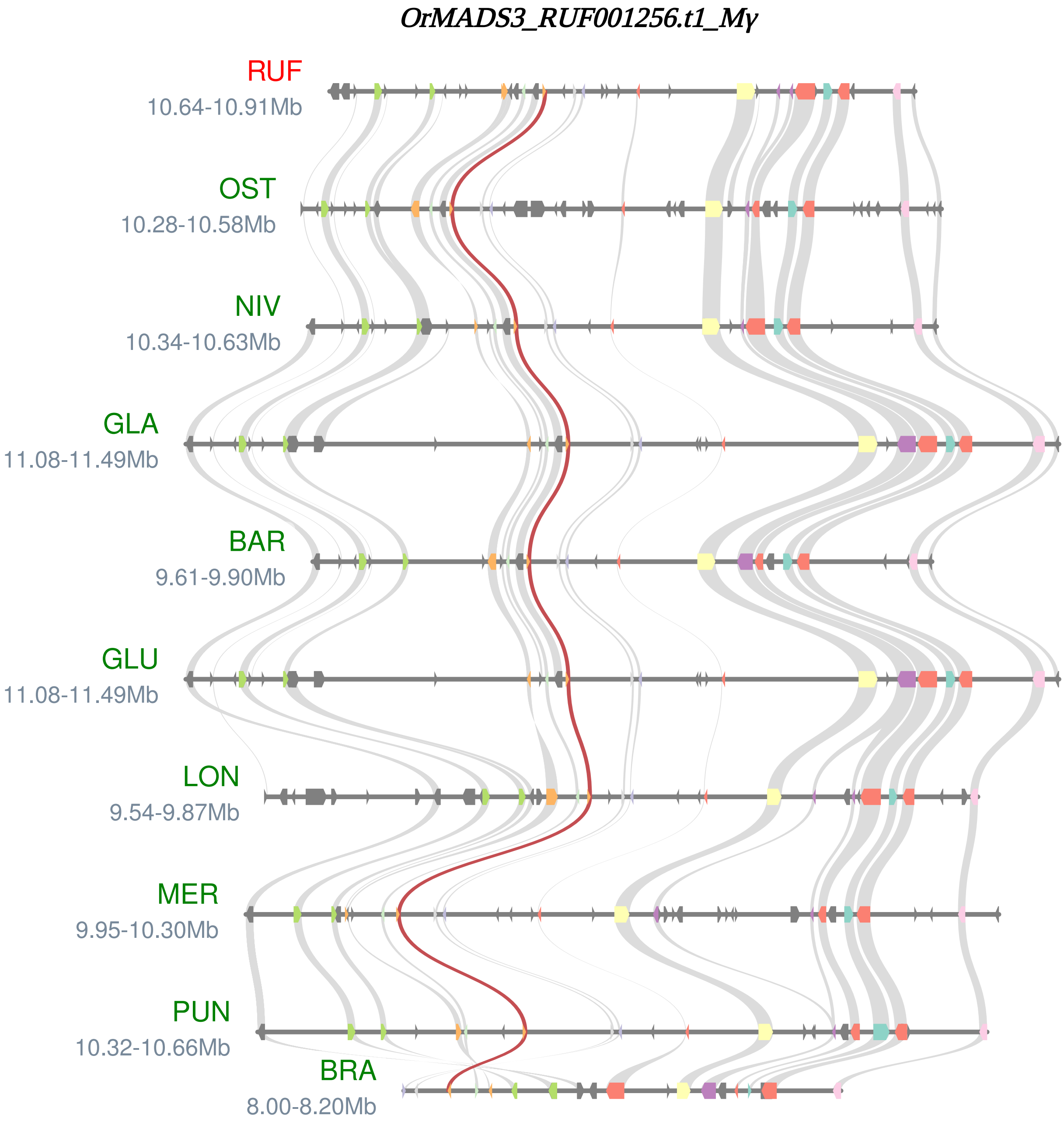

OrMADS3_RUF001256.t1_Mγ
RUF
10.64-10.91Mb
OST
10.28-10.58Mb
NIV
10.34-10.63Mb
GLA
11.08-11.49Mb
BAR
9.61-9.90Mb
GLU
11.08-11.49Mb
LON
9.54-9.87Mb
MER
9.95-10.30Mb
PUN
10.32-10.66Mb
BRA
8.00-8.20Mb

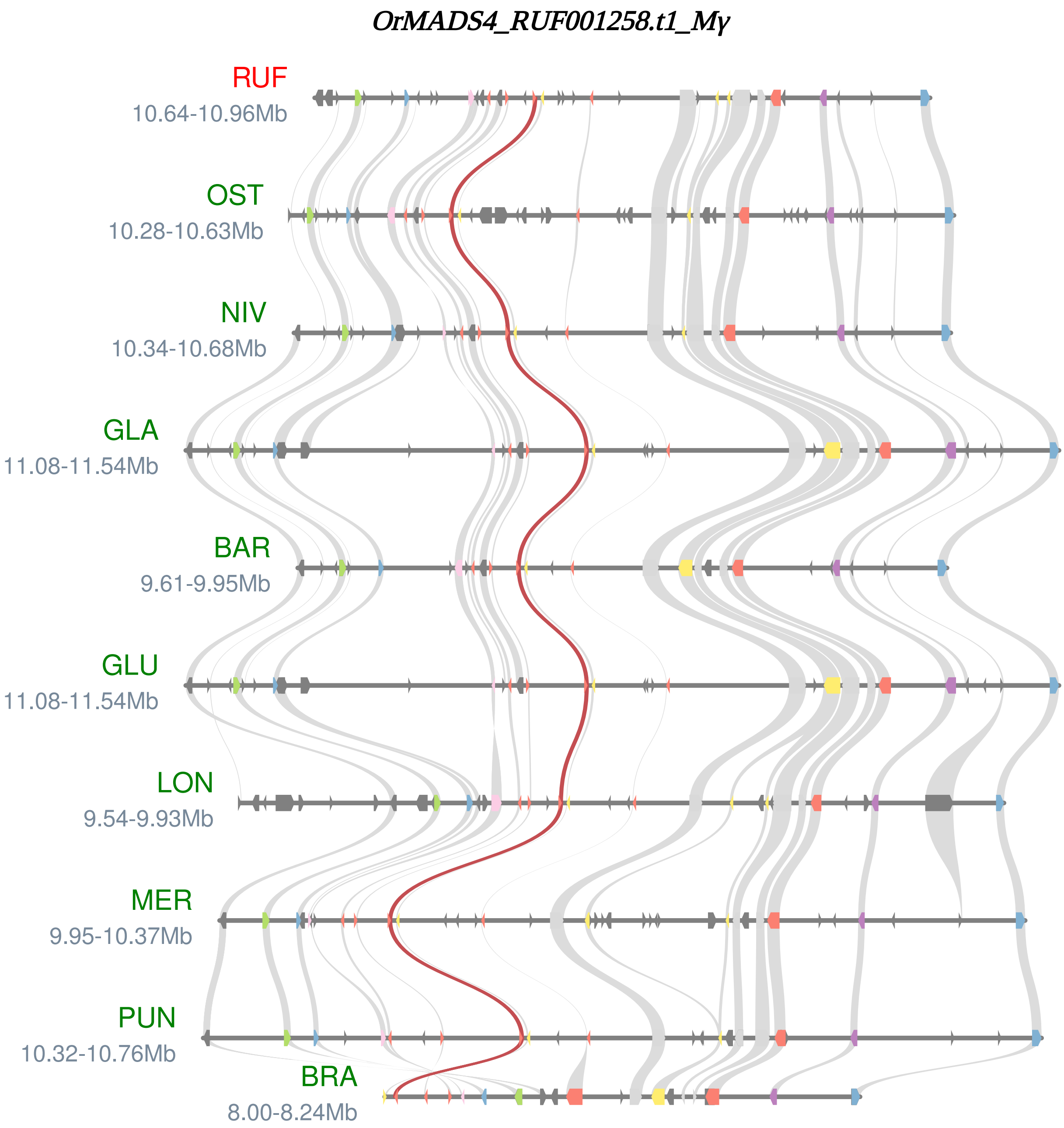
OrMADS4_RUF001258.t1_Mγ
RUF
10.64-10.96Mb
OST
10.28-10.63Mb
NIV
10.34-10.68Mb
GLA
11.08-11.54Mb
BAR
9.61-9.95Mb
GLU
11.08-11.54Mb
LON
9.54-9.93Mb
MER
9.95-10.37Mb
PUN
10.32-10.76Mb
BRA
8.00-8.24Mb

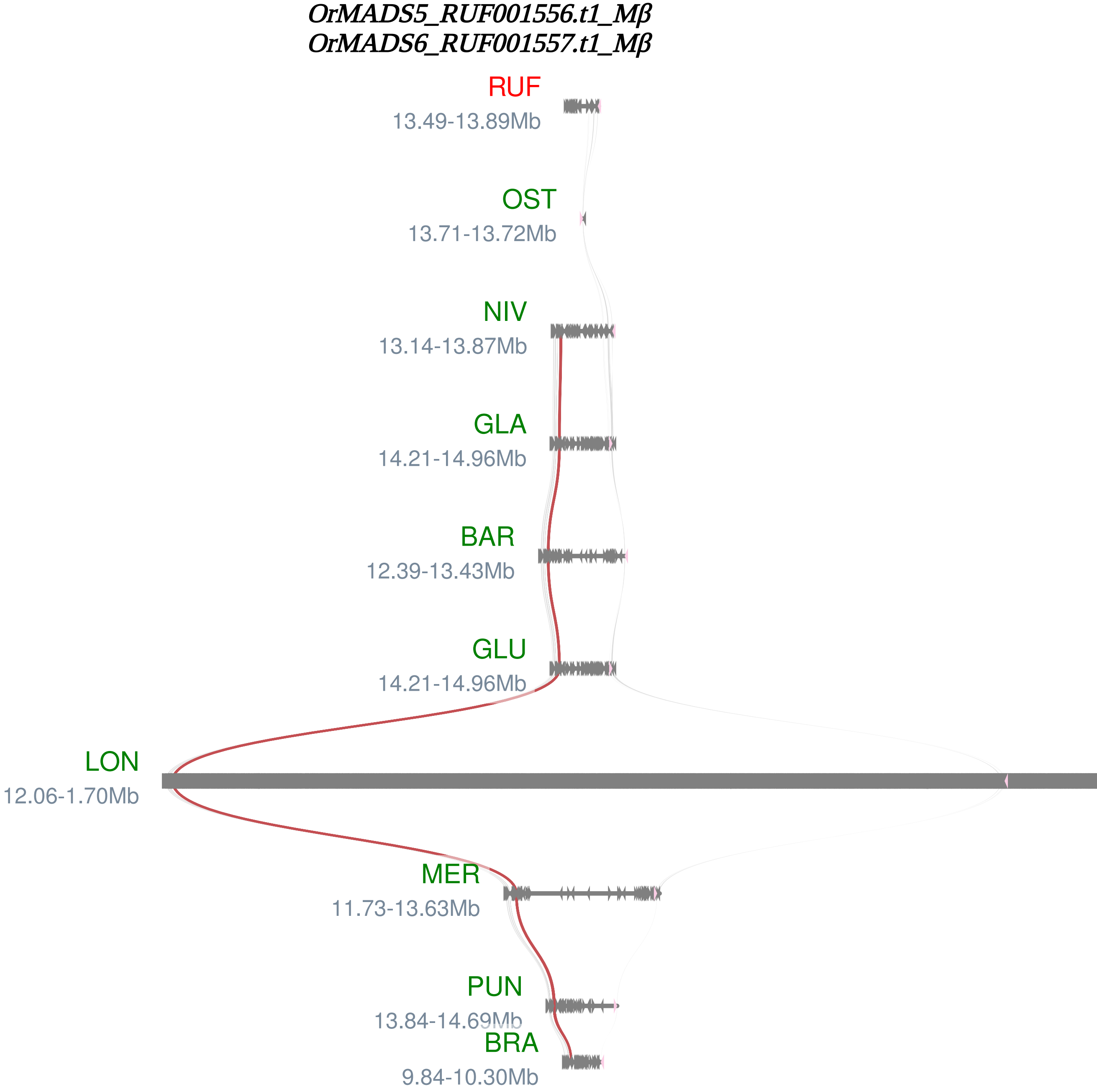
OrMADS5_RUF001556.t1_Mβ
OrMADS6_RUF001557.t1_Mβ
RUF
13.49-13.89Mb
OST
13.71-13.72Mb
NIV
13.14-13.87Mb
GLA
14.21-14.96Mb
BAR
12.39-13.43Mb
GLU
14.21-14.96Mb
LON
12.06-1.70Mb
MER
11.73-13.63Mb
PUN
13.84-14.69Mb
BRA
9.84-10.30Mb

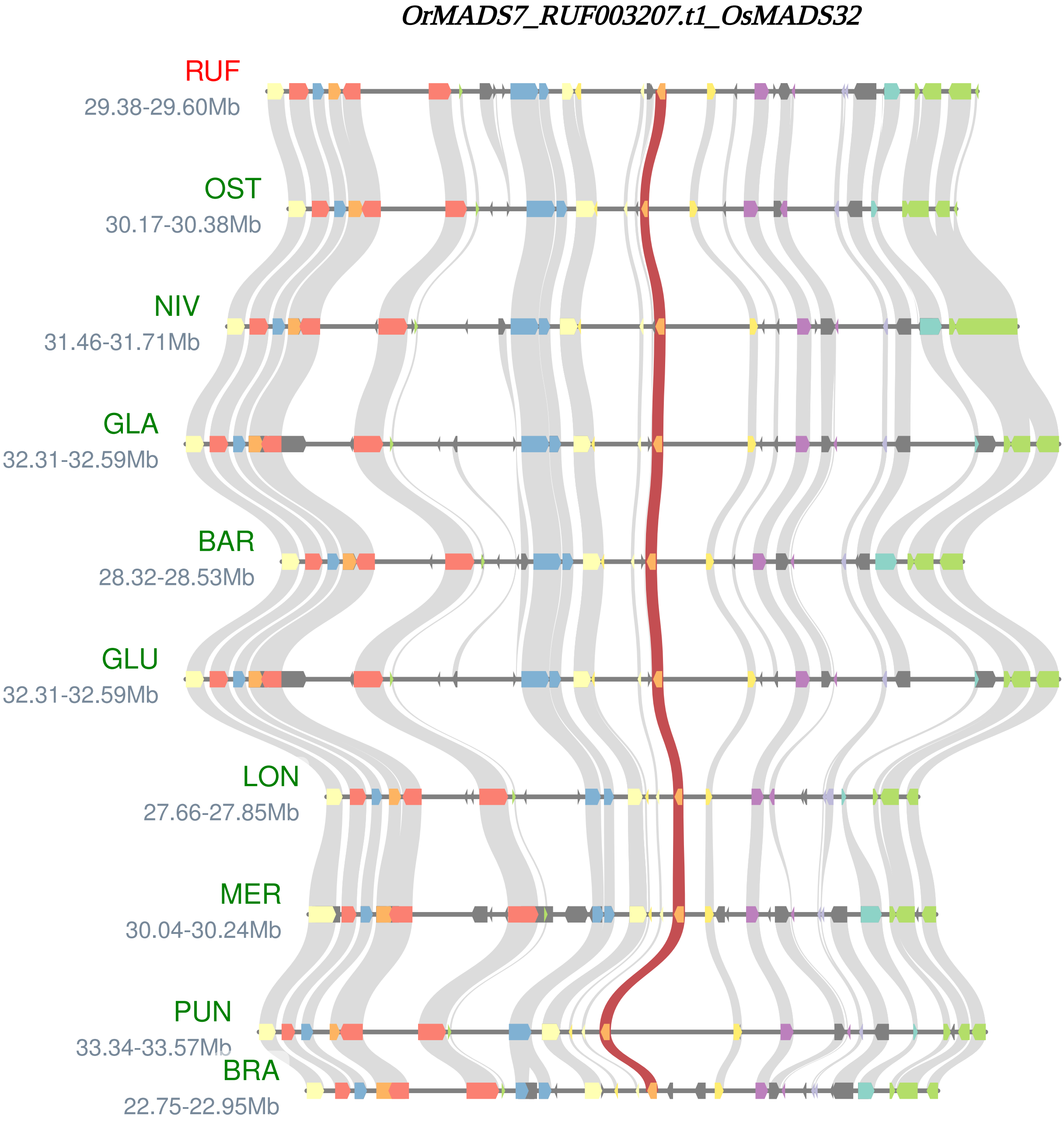
OrMADS7_RUF003207.t1_OsMADS32
RUF
29.38-29.60Mb
OST
30.17-30.38Mb
NIV
31.46-31.71Mb
GLA
32.31-32.59Mb
BAR
28.32-28.53Mb
GLU
32.31-32.59Mb
LON
27.66-27.85Mb
MER
30.04-30.24Mb
PUN
33.34-33.57Mb
BRA
22.75-22.95Mb

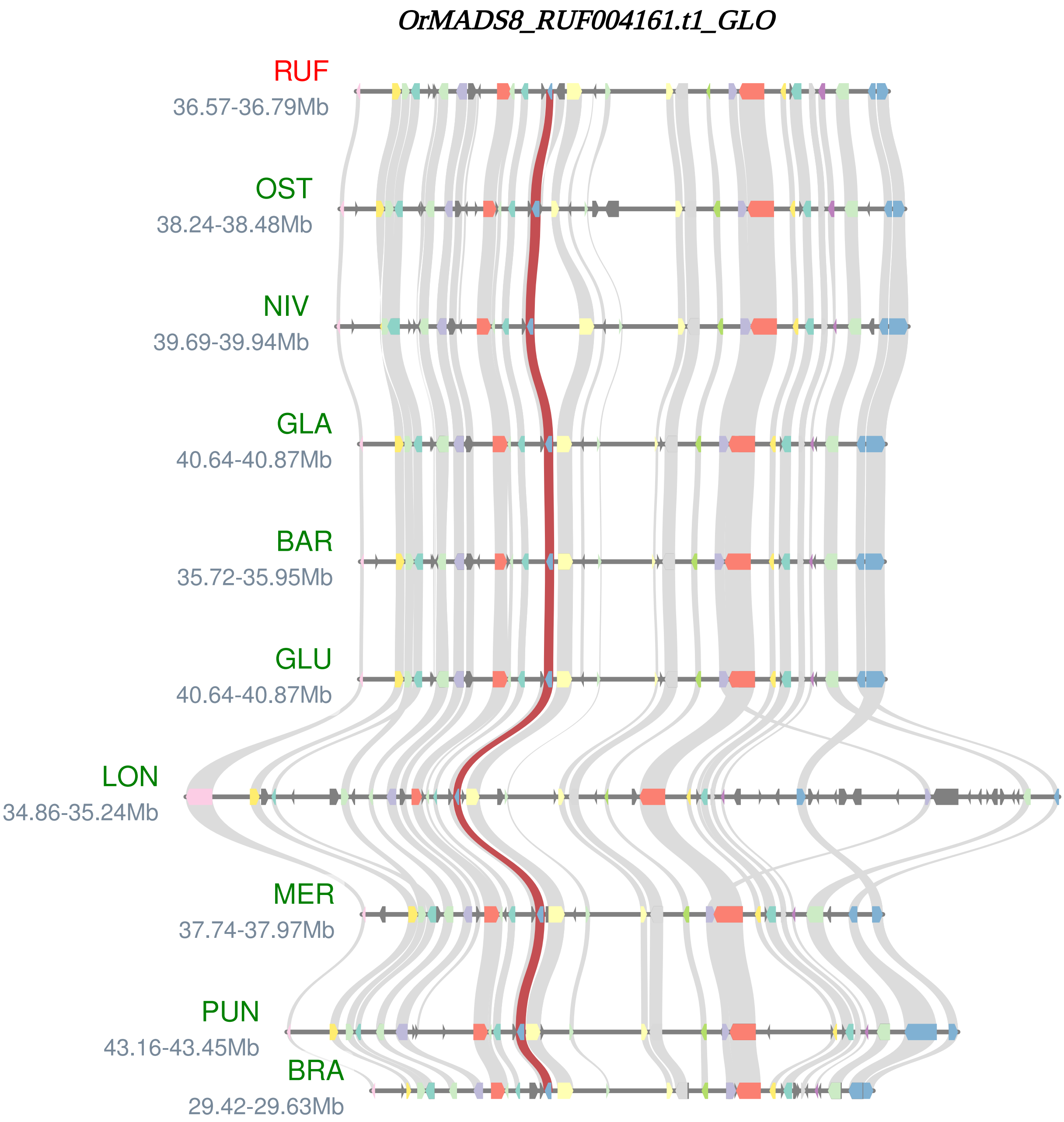
OrMADS8_RUF004161.t1_GLO
RUF
36.57-36.79Mb
OST
38.24-38.48Mb
NIV
39.69-39.94Mb
GLA
40.64-40.87Mb
BAR
35.72-35.95Mb
GLU
40.64-40.87Mb
LON
34.86-35.24Mb
MER
37.74-37.97Mb
PUN
43.16-43.45Mb
BRA
29.42-29.63Mb

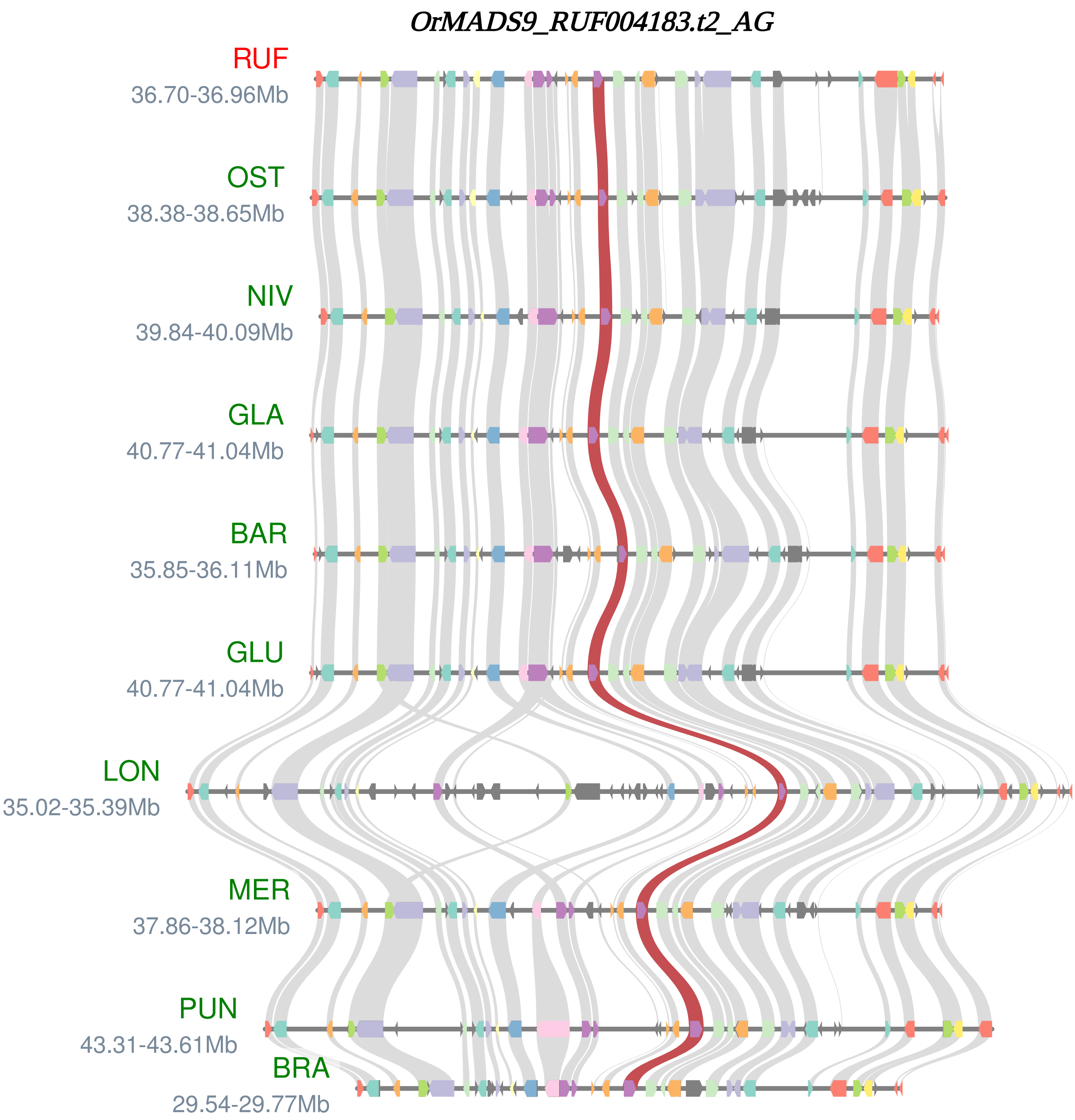

OrMADS9_RUF004183.t2_AG
RUF
36.70-36.96Mb
OST
38.38-38.65Mb
NIV
39.84-40.09Mb
GLA
40.77-41.04Mb
BAR
35.85-36.11Mb
GLU
40.77-41.04Mb
LON
35.02-35.39Mb
MER
37.86-38.12Mb
PUN
43.31-43.61Mb
BRA
29.54-29.77Mb

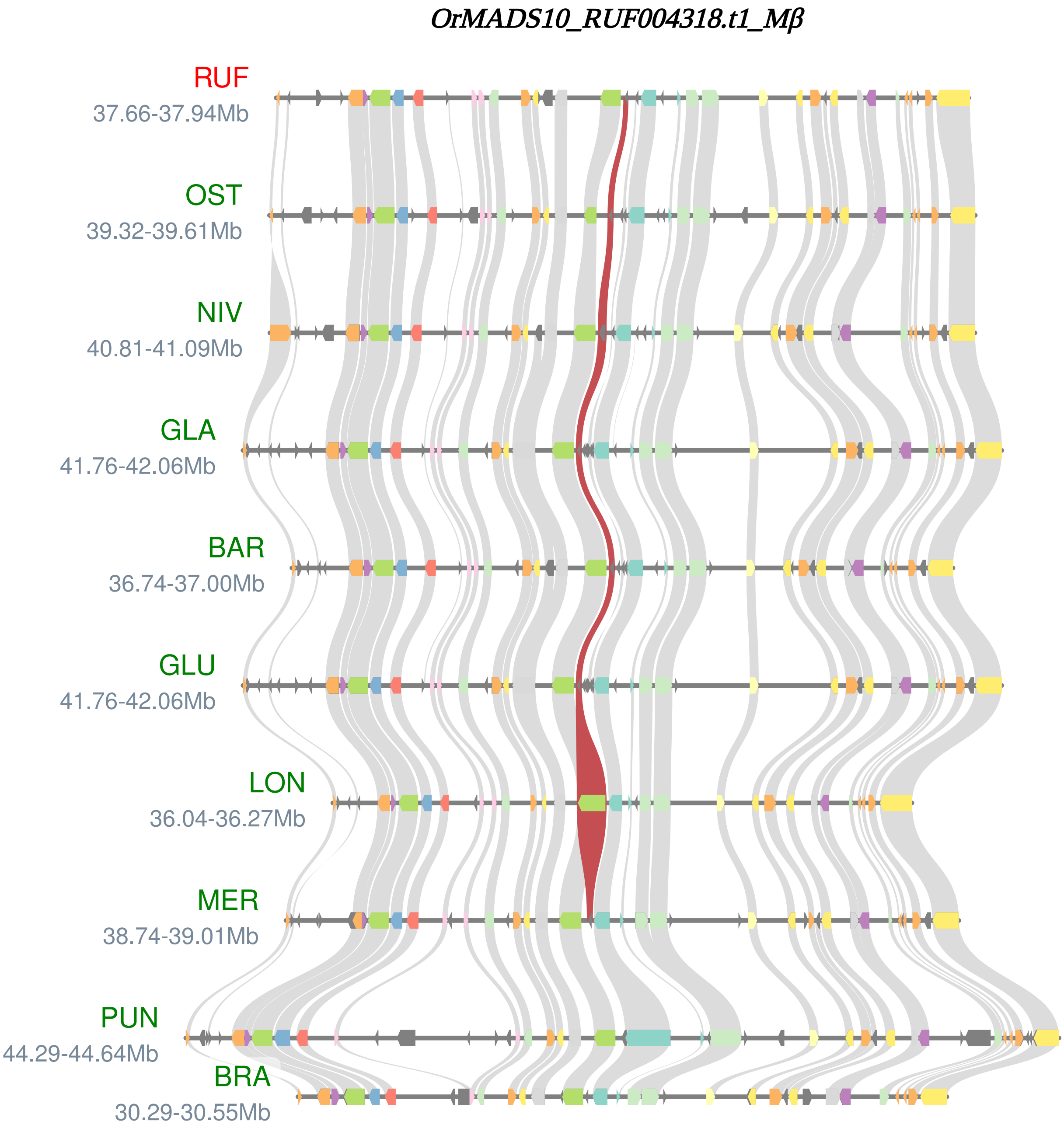
OrMADS10_RUF004318.t1_Mβ
RUF
37.66-37.94Mb
OST
39.32-39.61Mb
NIV
40.81-41.09Mb
GLA
41.76-42.06Mb
BAR
36.74-37.00Mb
GLU
41.76-42.06Mb
LON
36.04-36.27Mb
MER
38.74-39.01Mb
PUN
44.29-44.64Mb
BRA
30.29-30.55Mb

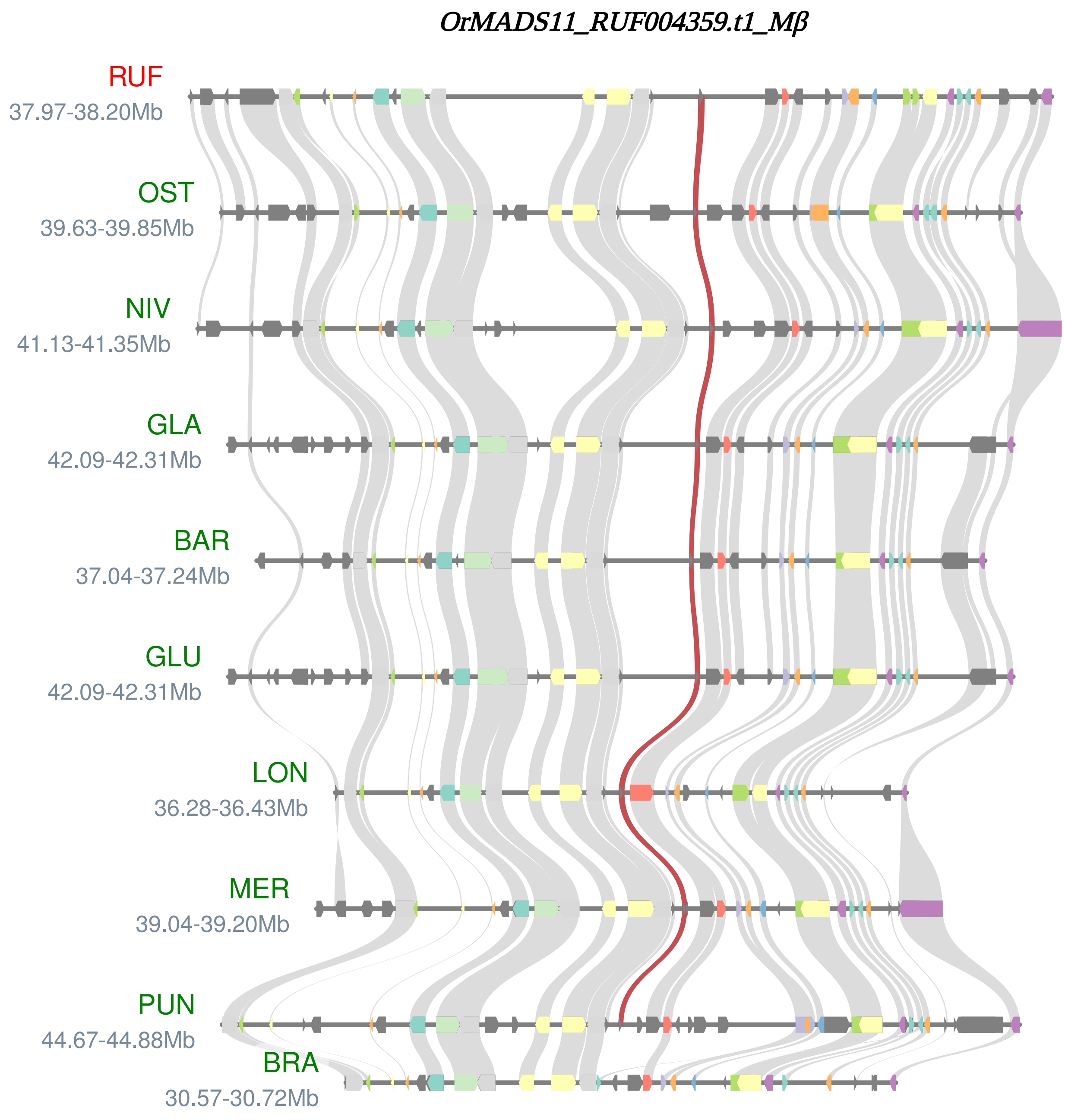
OrMADS11_RUF004359.t1_Mβ
RUF
37.97-38.20Mb
OST
39.63-39.85Mb
NIV
41.13-41.35Mb
GLA
42.09-42.31Mb
BAR
37.04-37.24Mb
GLU
42.09-42.31Mb
LON
36.28-36.43Mb
MER
39.04-39.20Mb
PUN
44.67-44.88Mb
BRA
30.57-30.72Mb

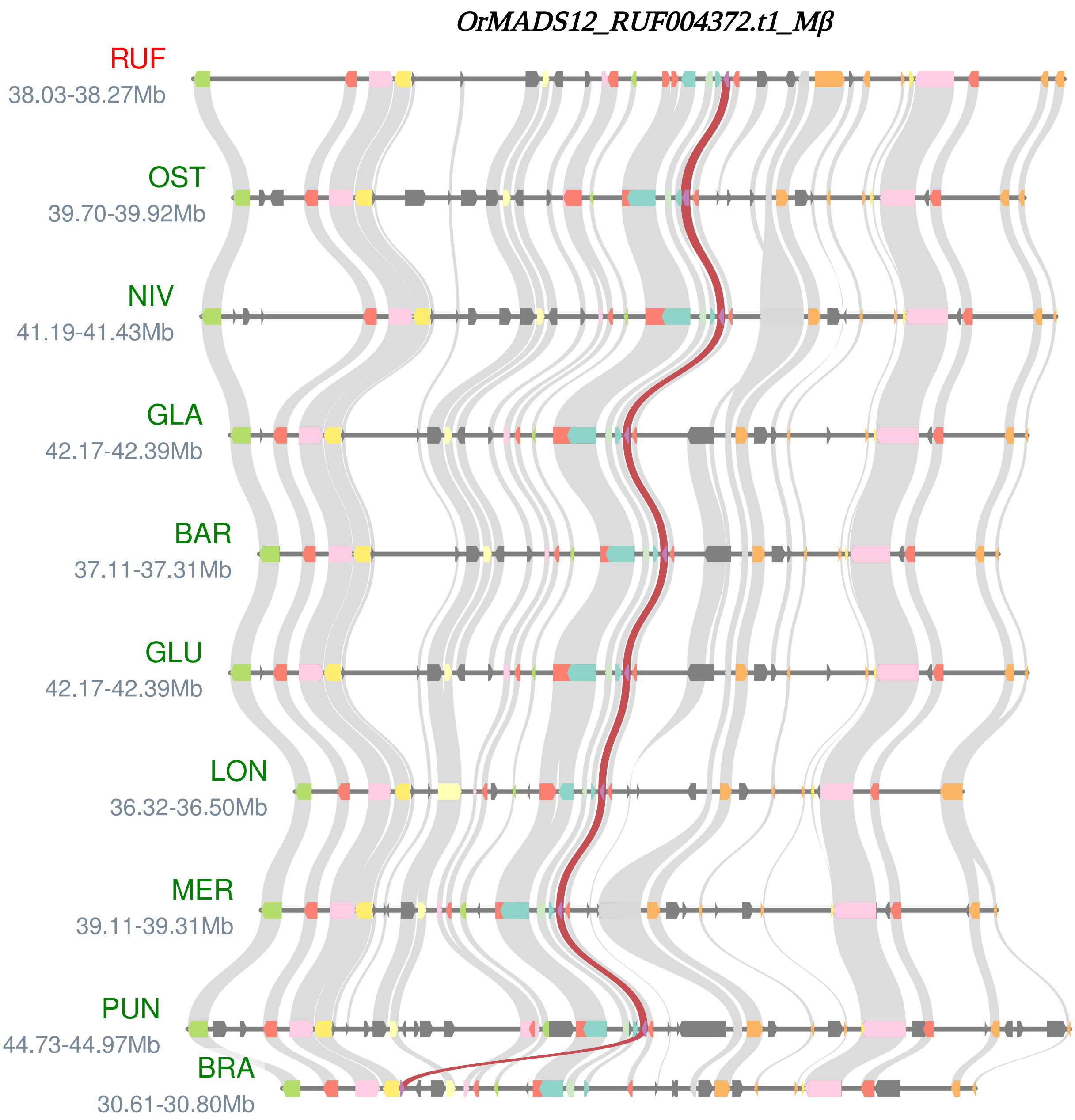
OrMADS12_RUF004372.t1_Mβ
RUF
38.03-38.27Mb
OST
39.70-39.92Mb
NIV
41.19-41.43Mb
GLA
42.17-42.39Mb
BAR
37.11-37.31Mb
GLU
42.17-42.39Mb
LON
36.32-36.50Mb
MER
39.11-39.31Mb
PUN
44.73-44.97Mb
BRA
30.61-30.80Mb

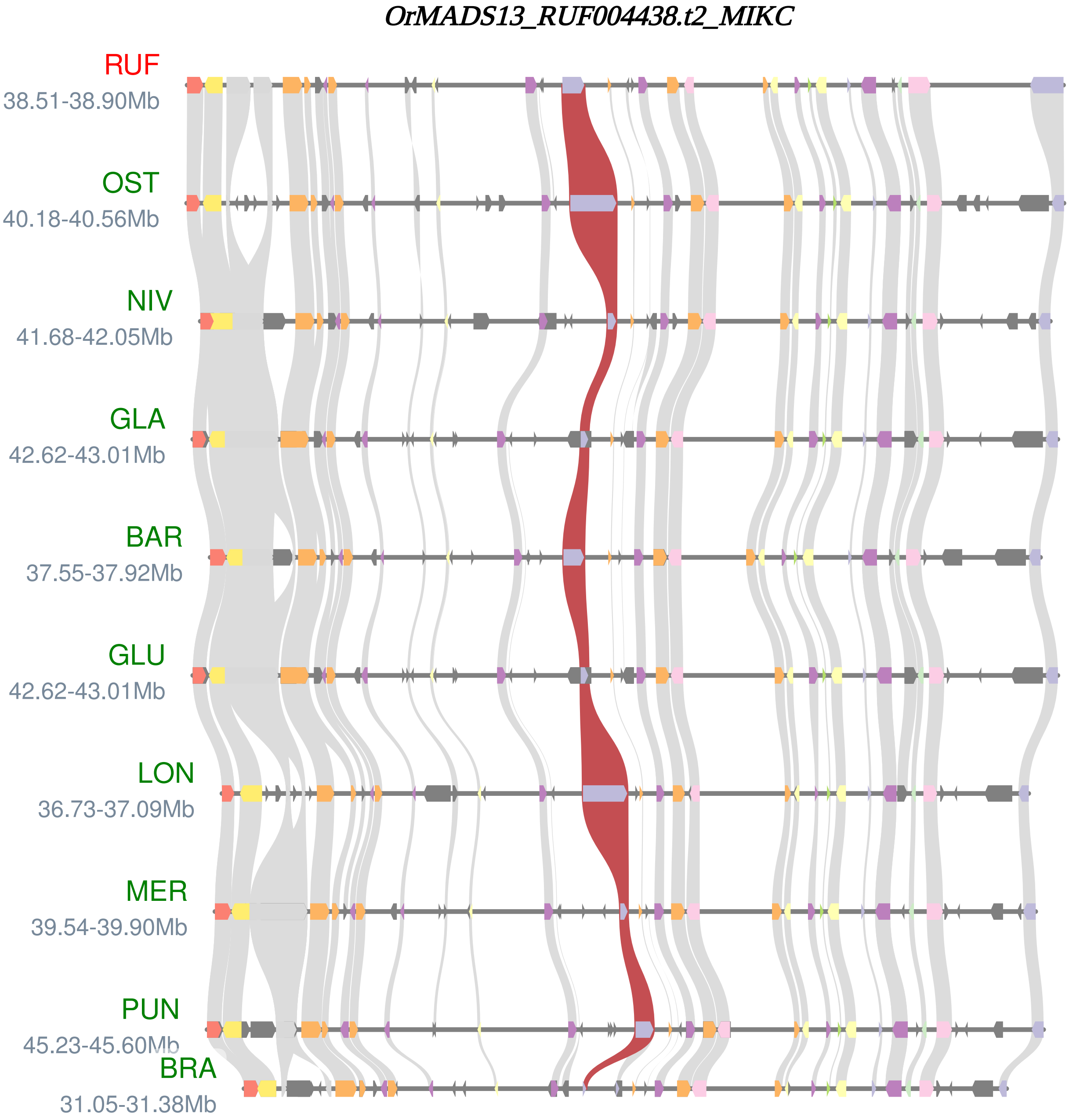
OrMADS13_RUF004438.t2_MIKC
RUF
38.51-38.90Mb
OST
40.18-40.56Mb
NIV
41.68-42.05Mb
GLA
42.62-43.01Mb
BAR
37.55-37.92Mb
GLU
42.62-43.01Mb
LON
36.73-37.09Mb
MER
39.54-39.90Mb
PUN
45.23-45.60Mb
BRA
31.05-31.38Mb

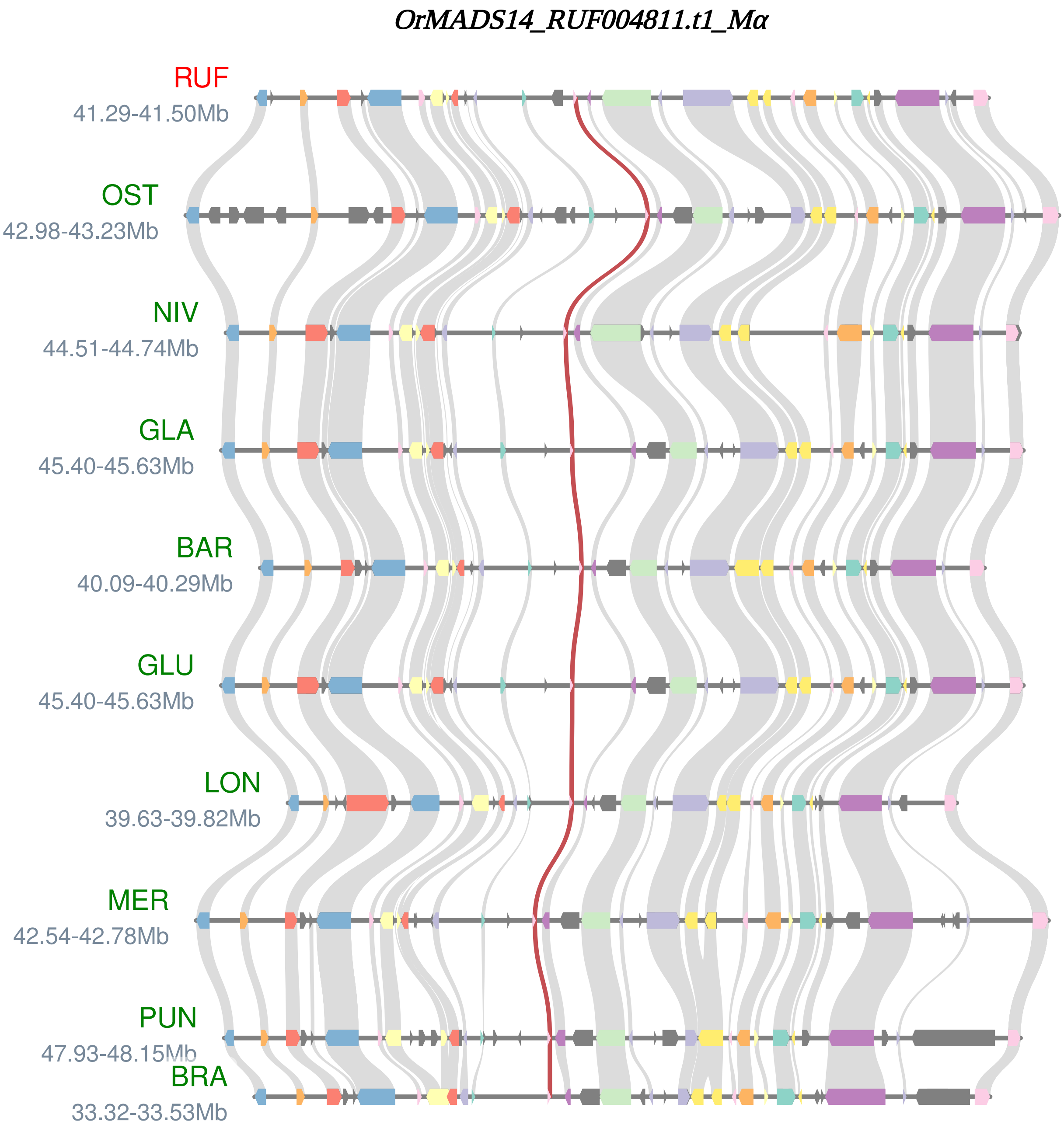
OrMADS14_RUF004811.t1_Mα
RUF
41.29-41.50Mb
OST
42.98-43.23Mb
NIV
44.51-44.74Mb
GLA
45.40-45.63Mb
BAR
40.09-40.29Mb
GLU
45.40-45.63Mb
LON
39.63-39.82Mb
MER
42.54-42.78Mb
PUN
47.93-48.15Mb
BRA
33.32-33.53Mb

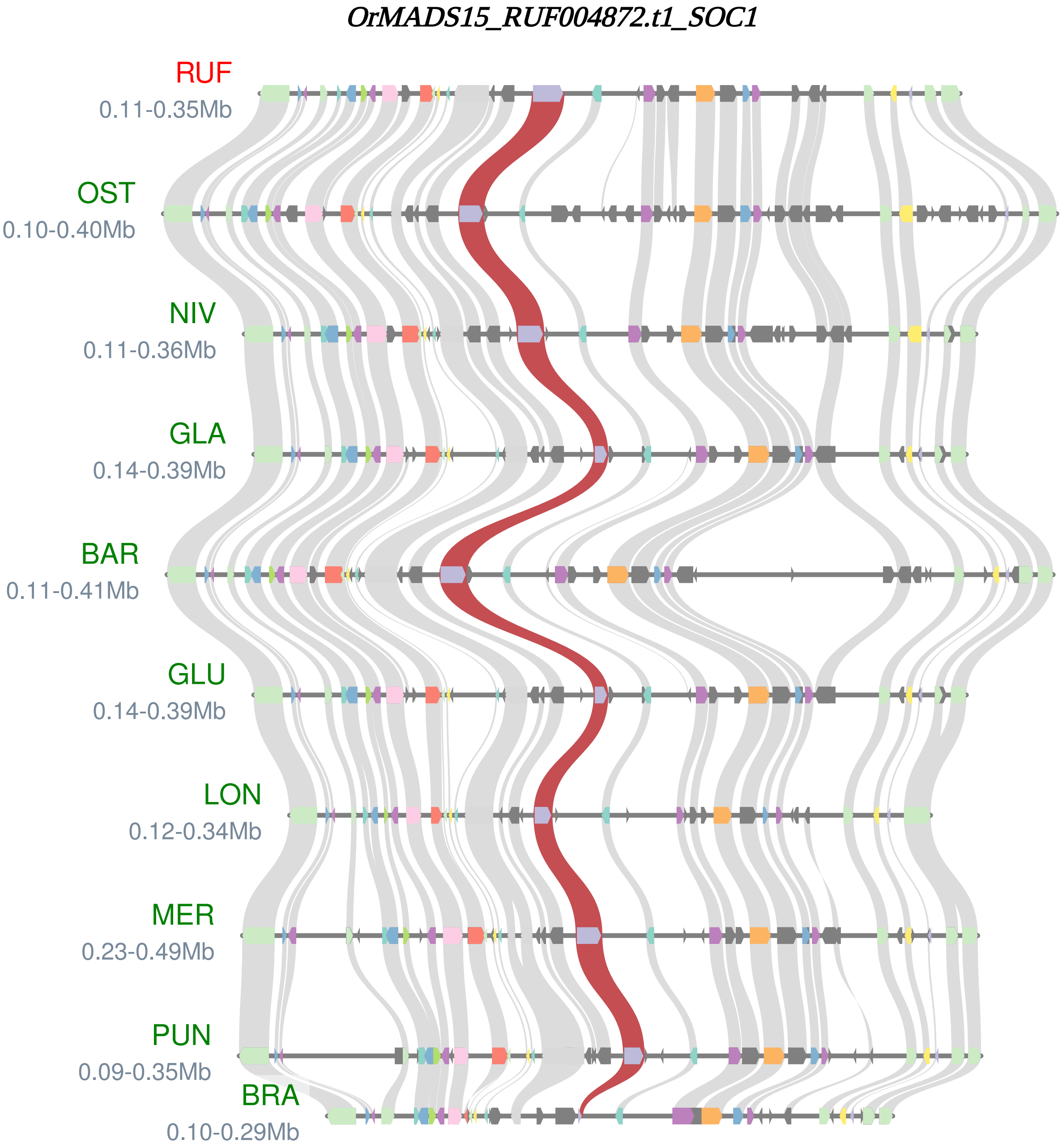
OrMADS15_RUF004872.t1_SOC1
RUF
0.11-0.35Mb
OST
0.10-0.40Mb
NIV
0.11-0.36Mb
GLA
0.14-0.39Mb
BAR
0.11-0.41Mb
GLU
0.14-0.39Mb
LON
0.12-0.34Mb
MER
0.23-0.49Mb
PUN
0.09-0.35Mb
BRA
0.10-0.29Mb

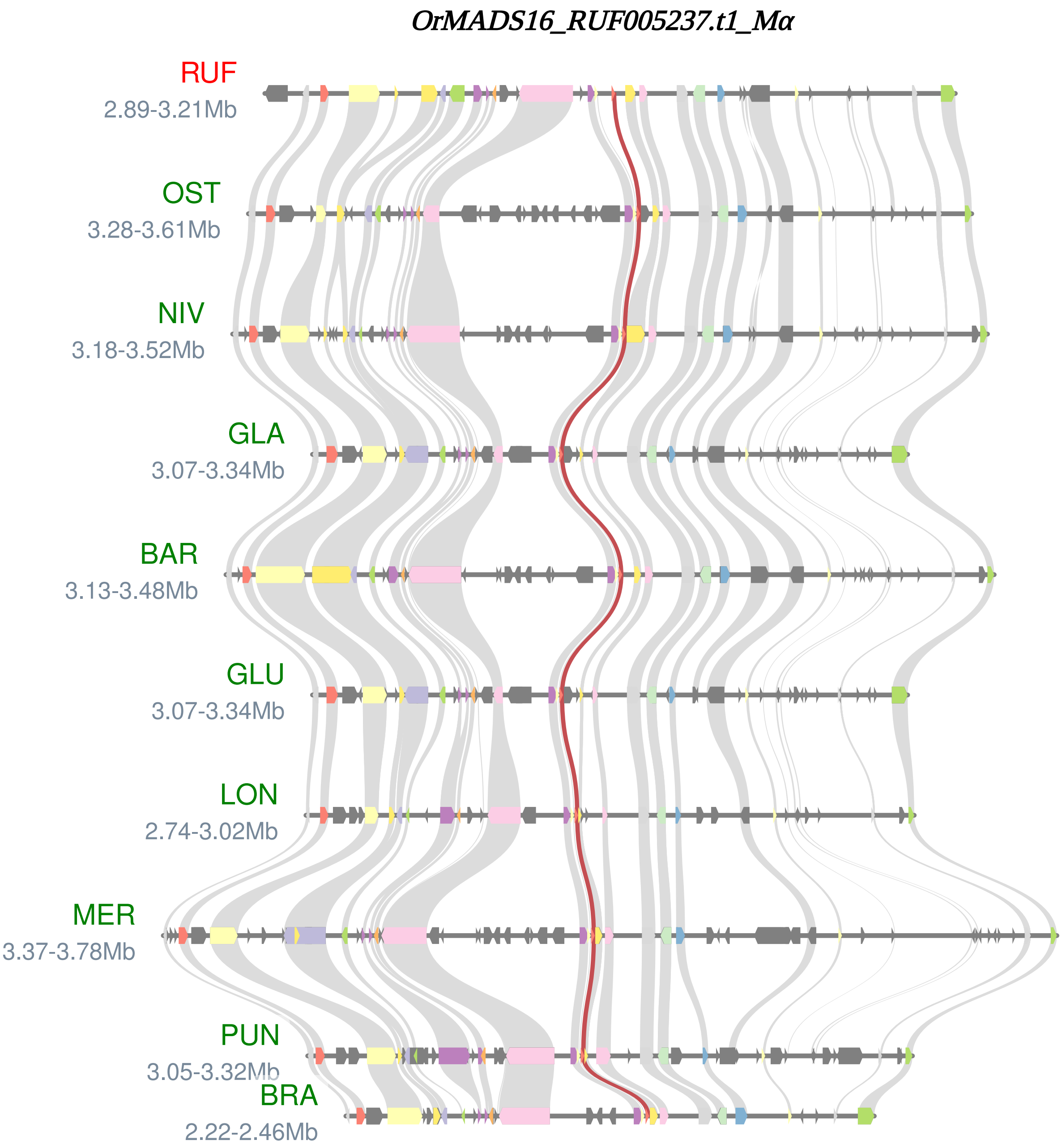

OrMADS16_RUF005237.t1_Mα
RUF
2.89-3.21Mb
OST
3.28-3.61Mb
NIV
3.18-3.52Mb
GLA
3.07-3.34Mb
BAR
3.13-3.48Mb
GLU
3.07-3.34Mb
LON
2.74-3.02Mb
MER
3.37-3.78Mb
PUN
3.05-3.32Mb
BRA
2.22-2.46Mb

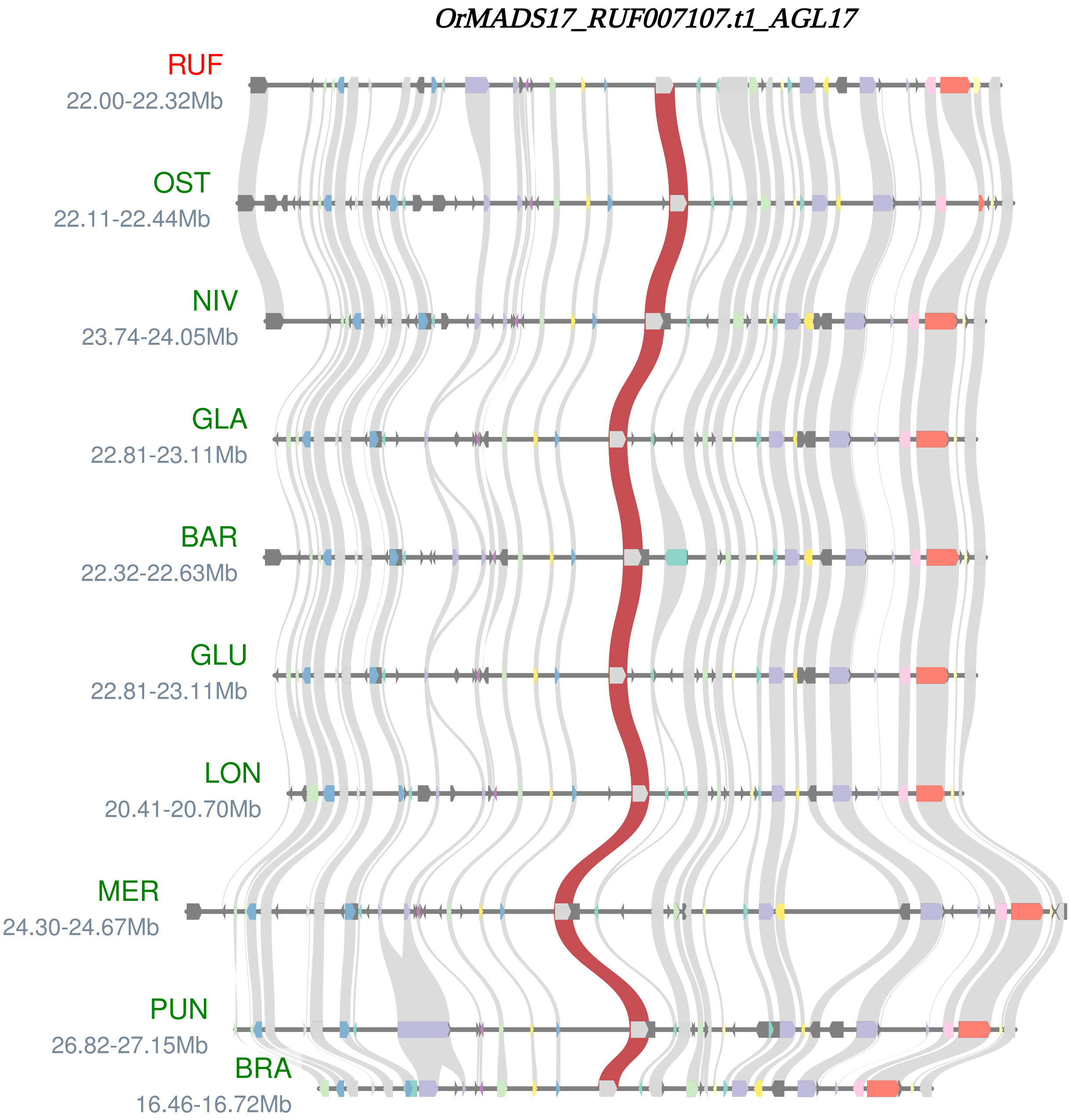

OrMADS17_RUF007107.t1_AGL17
RUF
22.00-22.32Mb
OST
22.11-22.44Mb
NIV
23.74-24.05Mb
GLA
22.81-23.11Mb
BAR
22.32-22.63Mb
GLU
22.81-23.11Mb
LON
20.41-20.70Mb
MER
24.30-24.67Mb
PUN
26.82-27.15Mb
BRA
16.46-16.72Mb

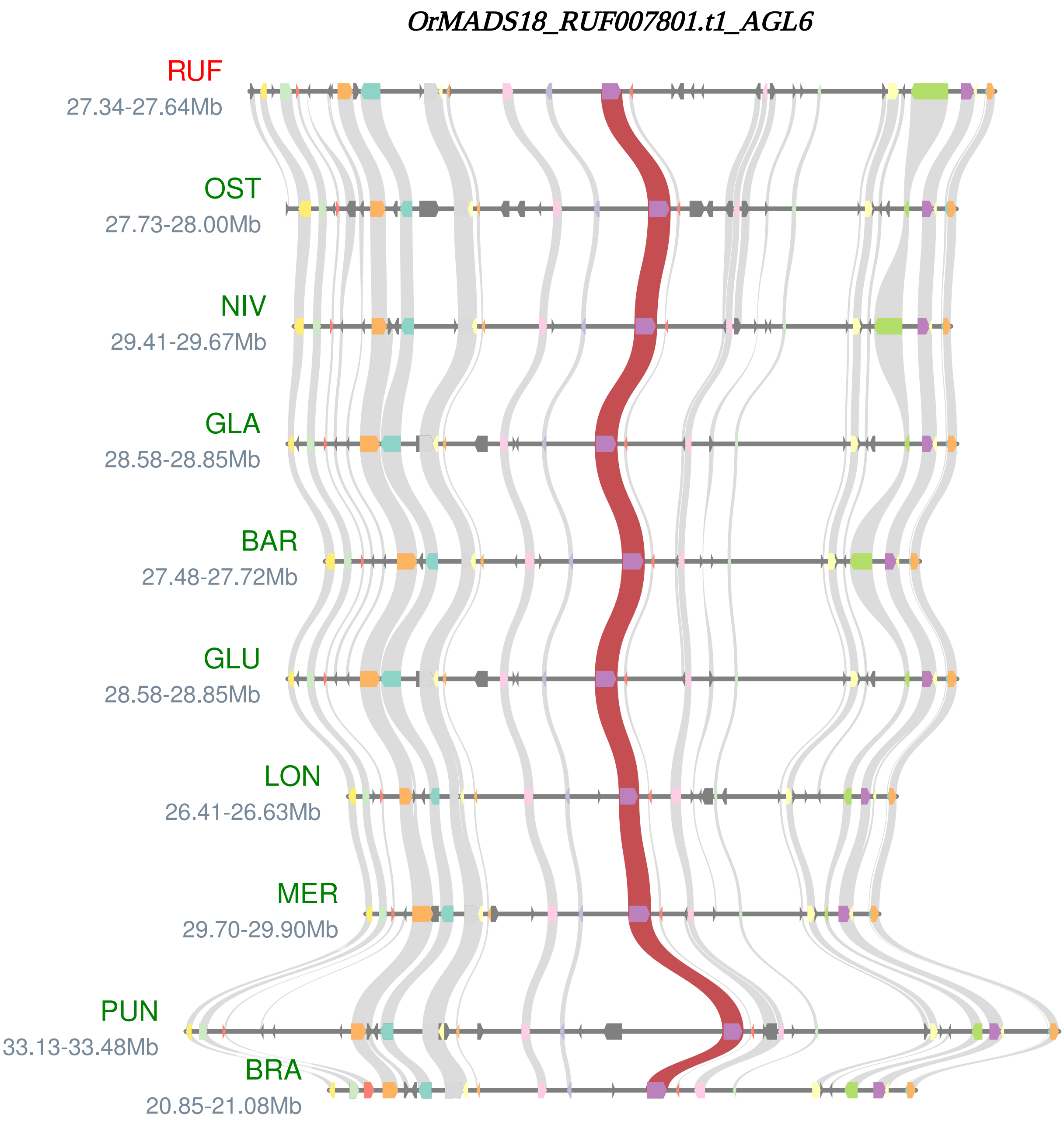
OrMADS18_RUF007801.t1_AGL6
RUF
27.34-27.64Mb
OST
27.73-28.00Mb
NIV
29.41-29.67Mb
GLA
28.58-28.85Mb
BAR
27.48-27.72Mb
GLU
28.58-28.85Mb
LON
26.41-26.63Mb
MER
29.70-29.90Mb
PUN
33.13-33.48Mb
BRA
20.85-21.08Mb

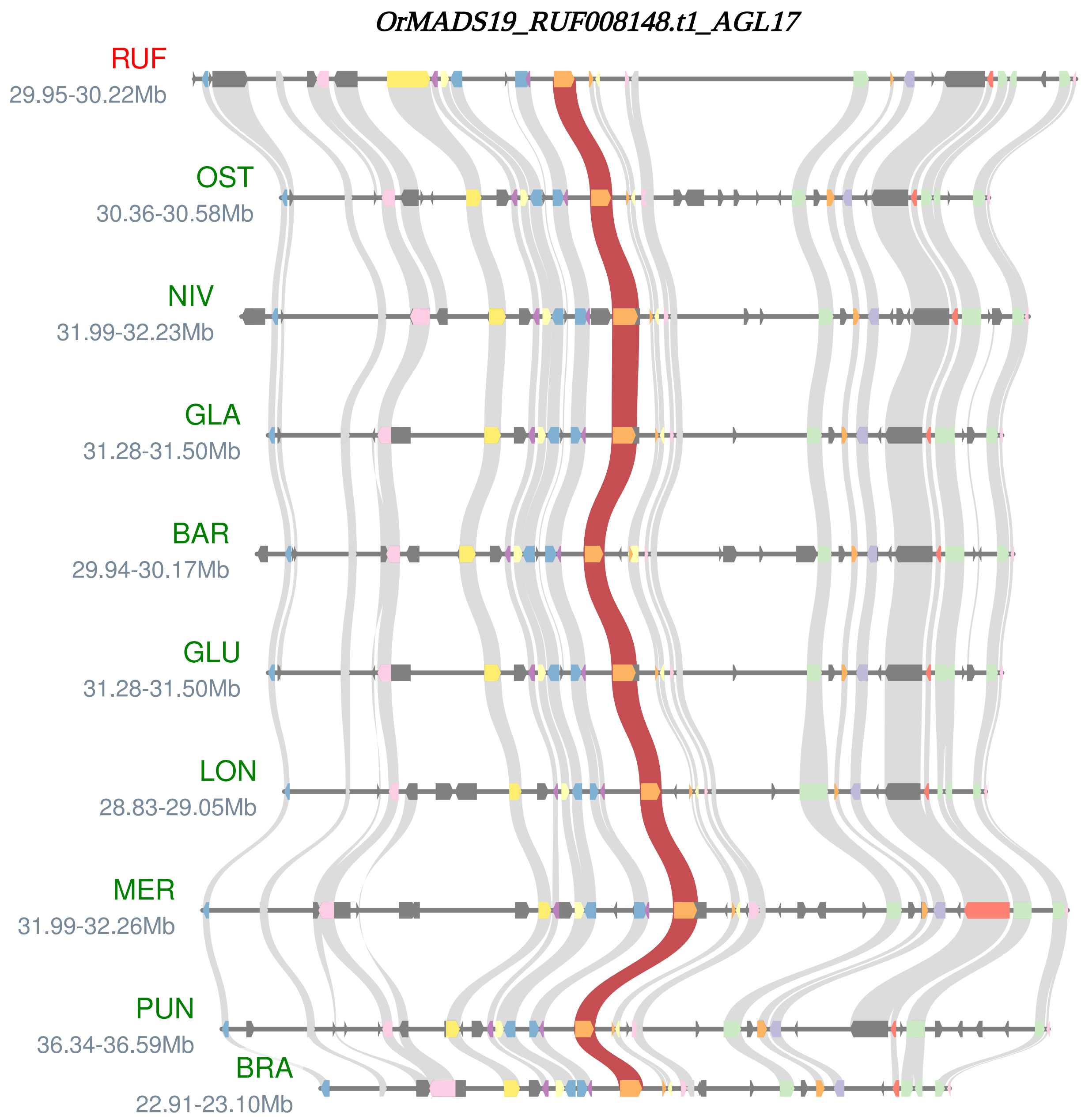
OrMADS19_RUF008148.t1_AGL17
RUF
29.95-30.22Mb
OST
30.36-30.58Mb
NIV
31.99-32.23Mb
GLA
31.28-31.50Mb
BAR
29.94-30.17Mb
GLU
31.28-31.50Mb
LON
28.83-29.05Mb
MER
31.99-32.26Mb
PUN
36.34-36.59Mb
BRA
22.91-23.10Mb

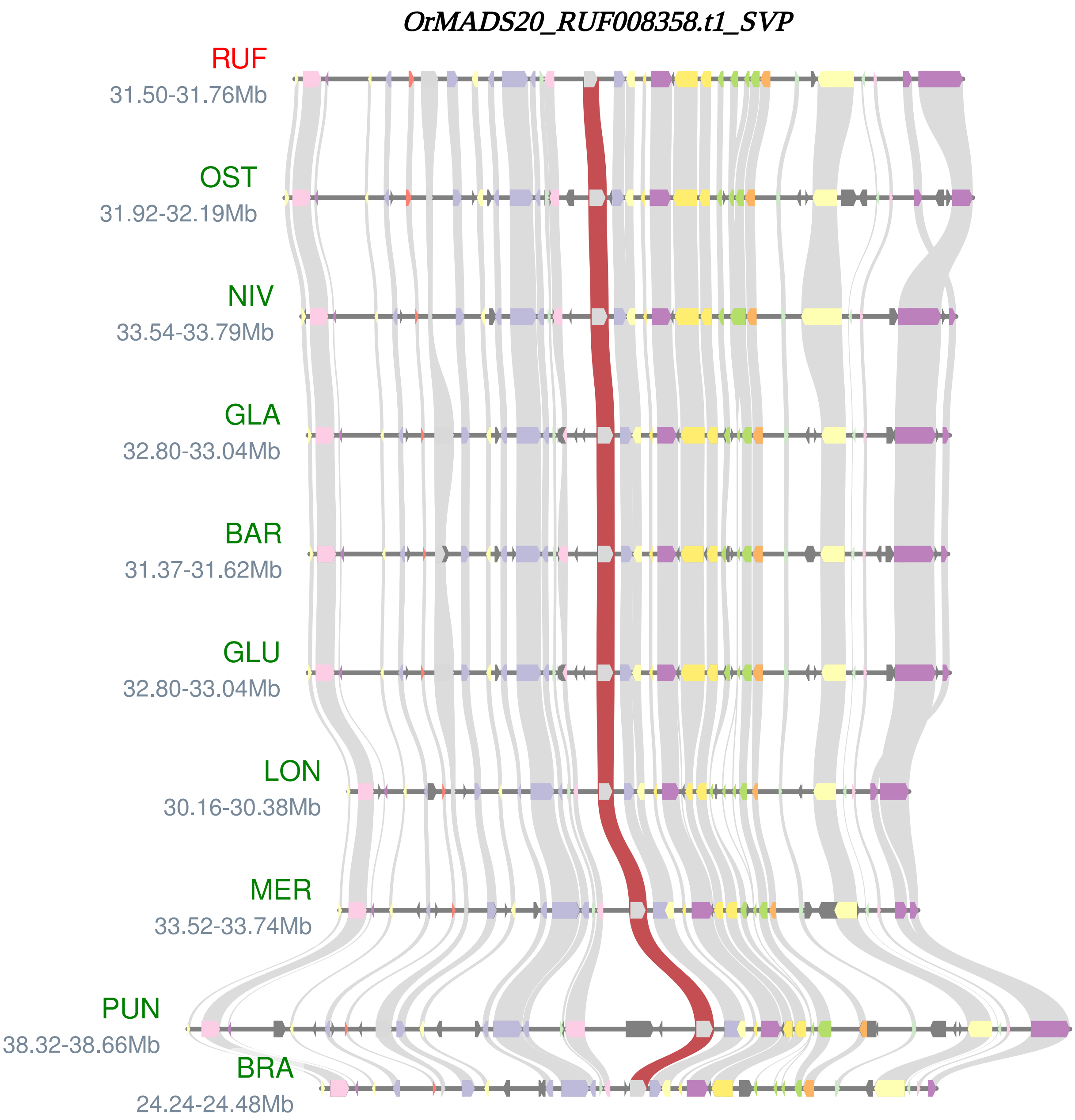

OrMADS20_RUF008358.t1_SVP
RUF
31.50-31.76Mb
OST
31.92-32.19Mb
NIV
33.54-33.79Mb
GLA
32.80-33.04Mb
BAR
31.37-31.62Mb
GLU
32.80-33.04Mb
LON
30.16-30.38Mb
MER
33.52-33.74Mb
PUN
38.32-38.66Mb
BRA
24.24-24.48Mb

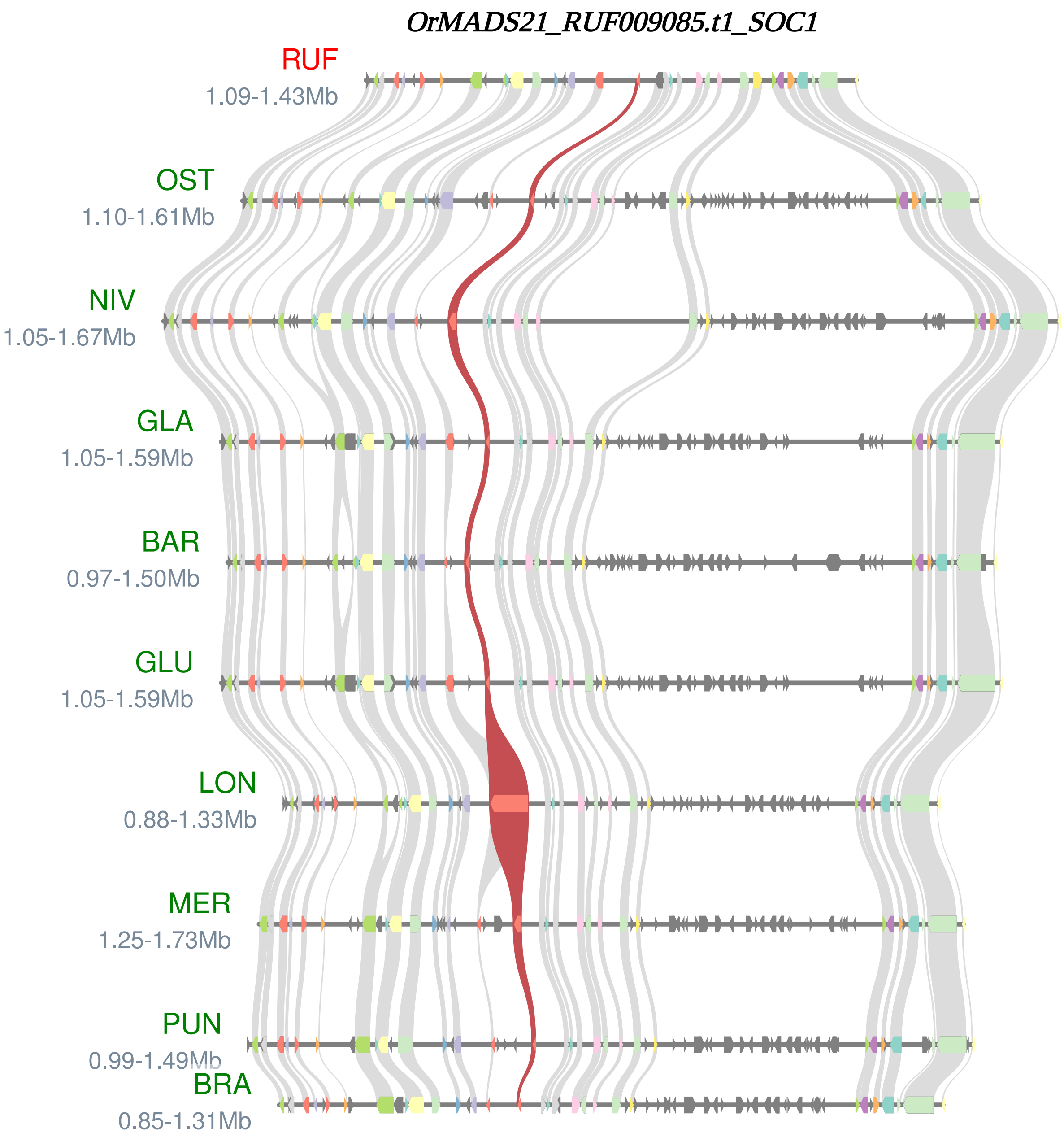
OrMADS21_RUF009085.t1_SOC1
RUF
1.09-1.43Mb
OST
1.10-1.61Mb
NIV
1.05-1.67Mb
GLA
1.05-1.59Mb
BAR
0.97-1.50Mb
GLU
1.05-1.59Mb
LON
0.88-1.33Mb
MER
1.25-1.73Mb
PUN
0.99-1.49Mb
BRA
0.85-1.31Mb

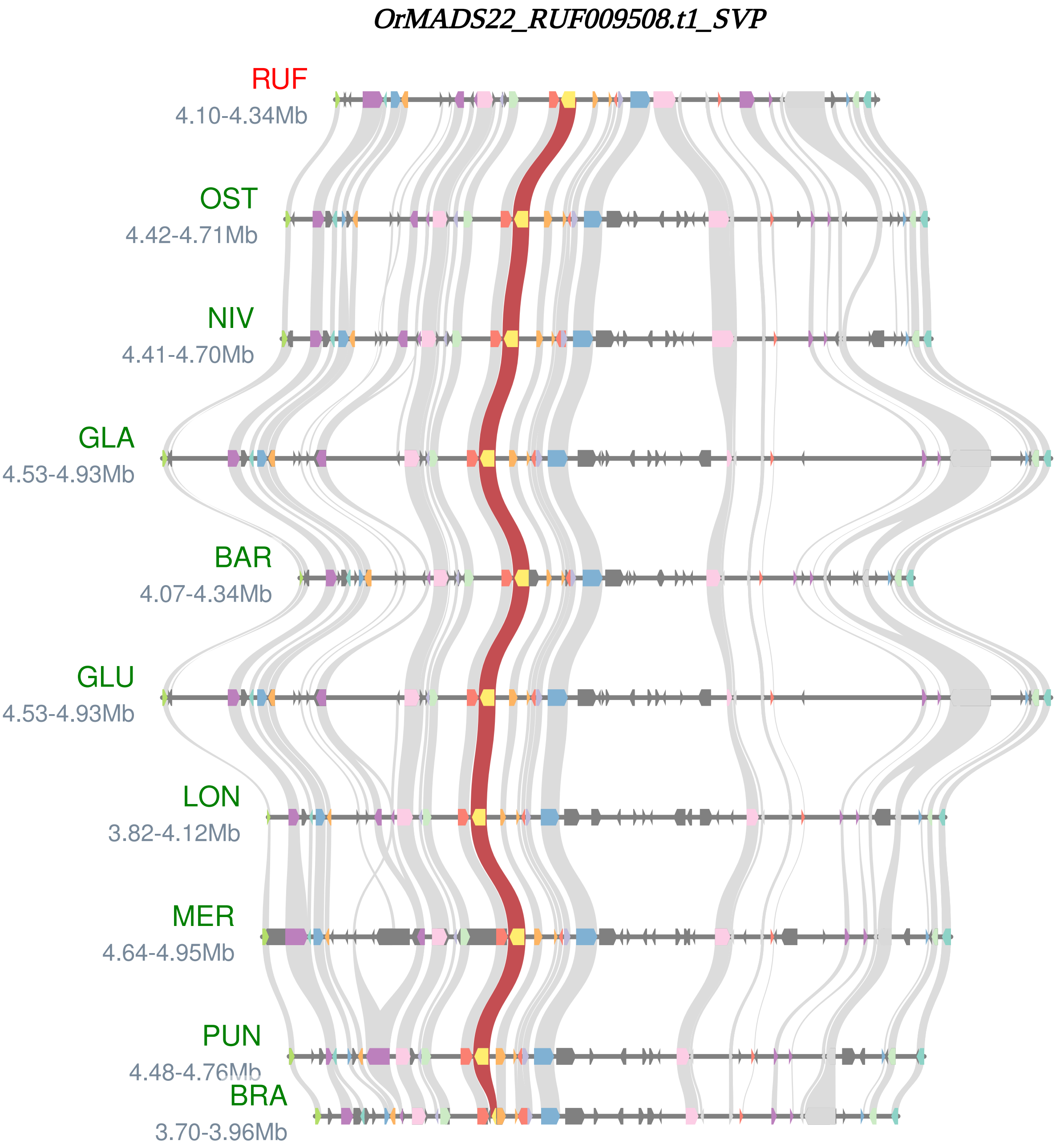

OrMADS22_RUF009508.t1_SVP
RUF
4.10-4.34Mb
OST
4.42-4.71Mb
NIV
4.41-4.70Mb
GLA
4.53-4.93Mb
BAR
4.07-4.34Mb
GLU
4.53-4.93Mb
LON
3.82-4.12Mb
MER
4.64-4.95Mb
PUN
4.48-4.76Mb
BRA
3.70-3.96Mb

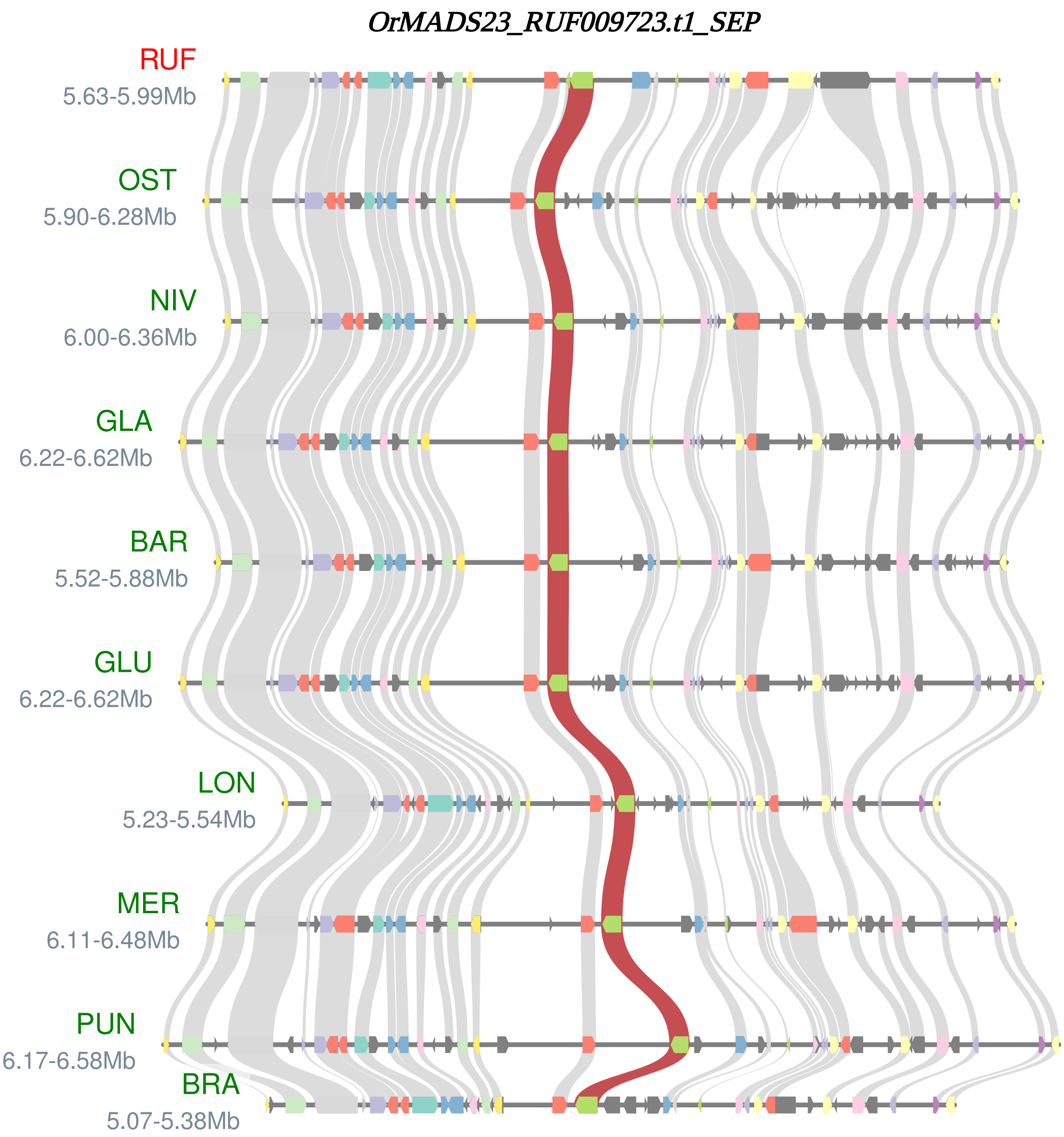

OrMADS23_RUF009723.t1_SEP
RUF
5.63-5.99Mb
OST
5.90-6.28Mb
NIV
6.00-6.36Mb
GLA
6.22-6.62Mb
BAR
5.52-5.88Mb
GLU
6.22-6.62Mb
LON
5.23-5.54Mb
MER
6.11-6.48Mb
PUN
6.17-6.58Mb
BRA
5.07-5.38Mb

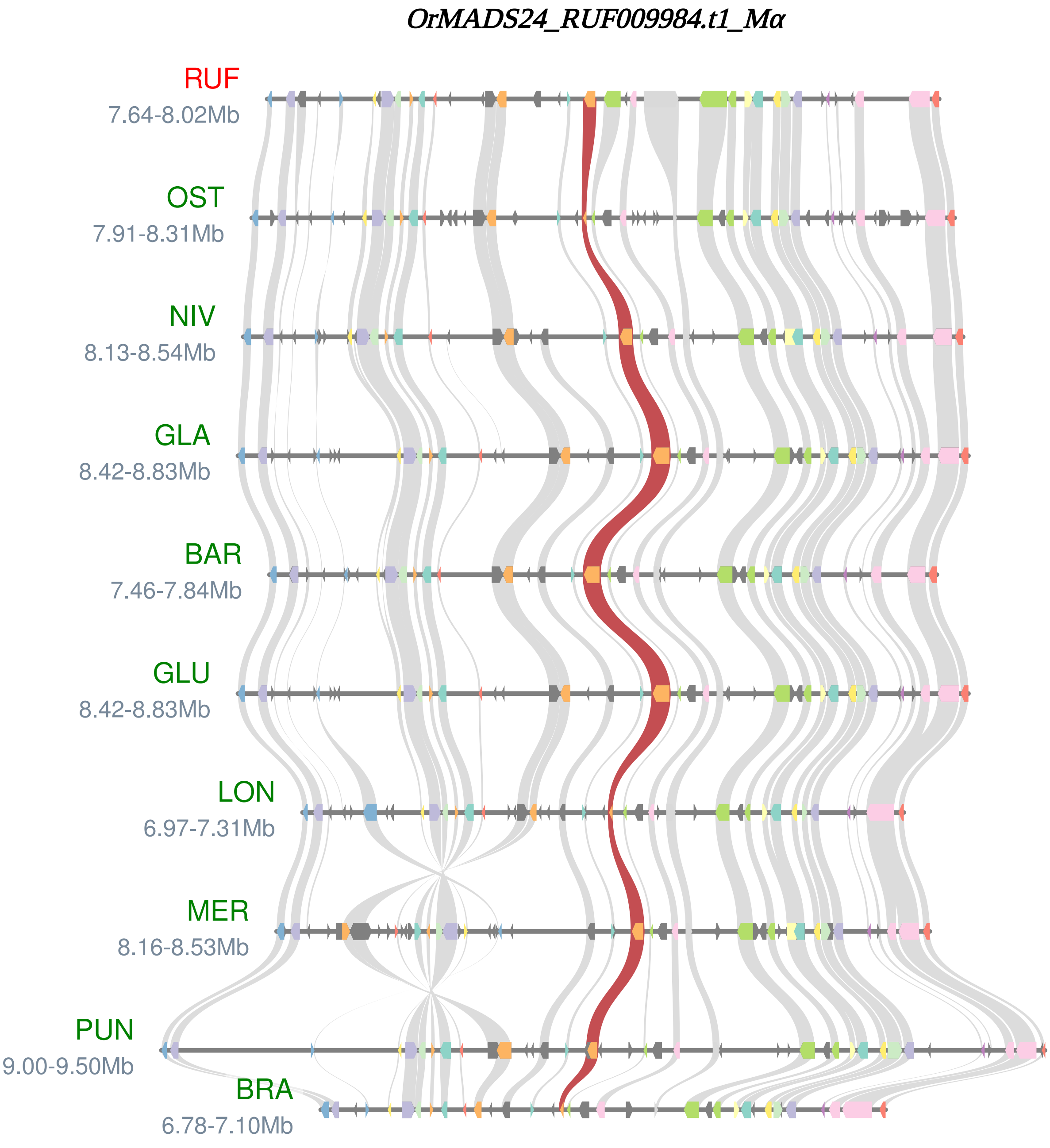
OrMADS24_RUF009984.t1_Mα
RUF
7.64-8.02Mb
OST
7.91-8.31Mb
NIV
8.13-8.54Mb
GLA
8.42-8.83Mb
BAR
7.46-7.84Mb
GLU
8.42-8.83Mb
LON
6.97-7.31Mb
MER
8.16-8.53Mb
PUN
9.00-9.50Mb
BRA
6.78-7.10Mb

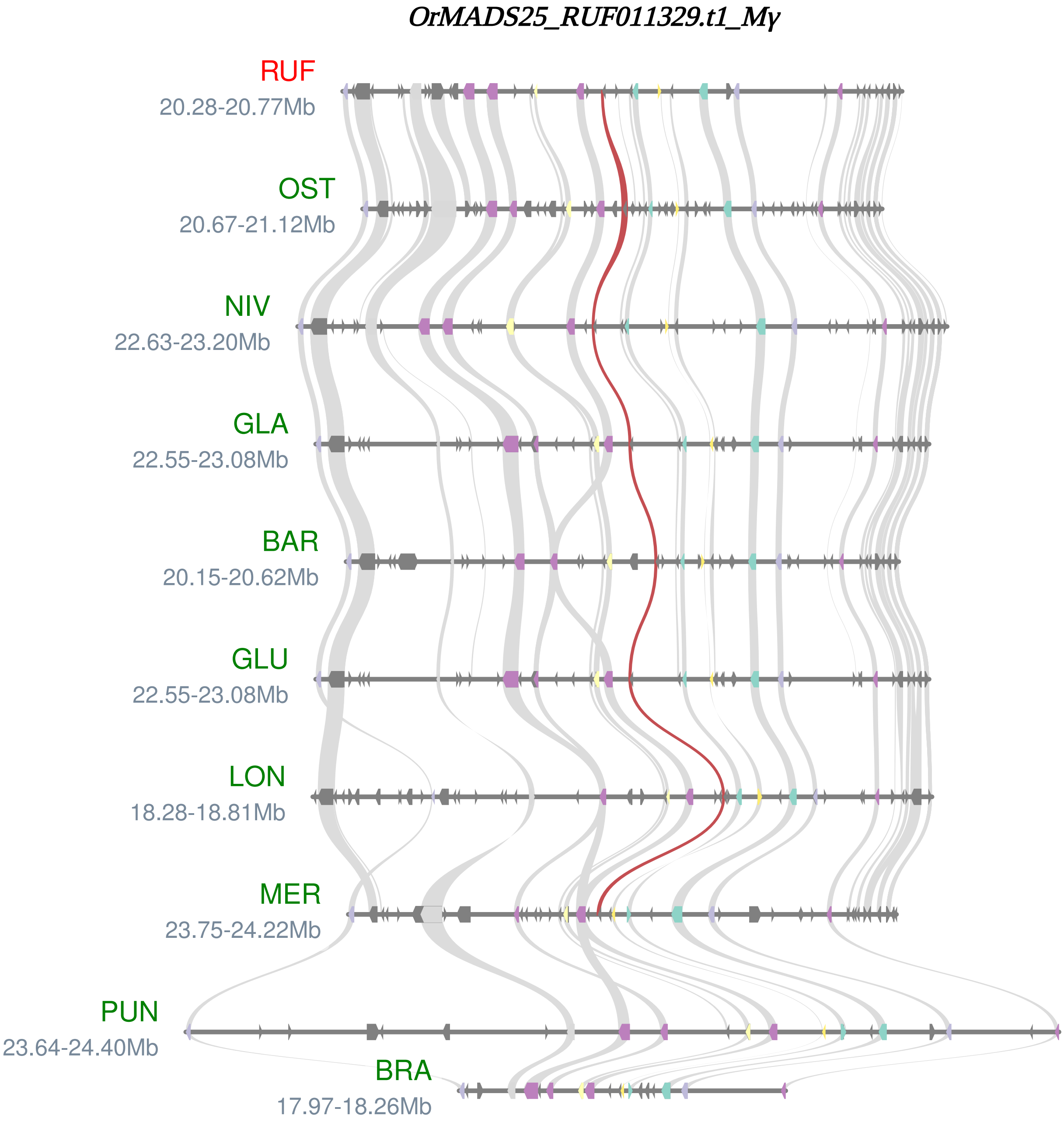
OrMADS25_RUF011329.t1_Mγ
RUF
20.28-20.77Mb
OST
20.67-21.12Mb
NIV
22.63-23.20Mb
GLA
22.55-23.08Mb
BAR
20.15-20.62Mb
GLU
22.55-23.08Mb
LON
18.28-18.81Mb
MER
23.75-24.22Mb
PUN
23.64-24.40Mb
BRA
17.97-18.26Mb

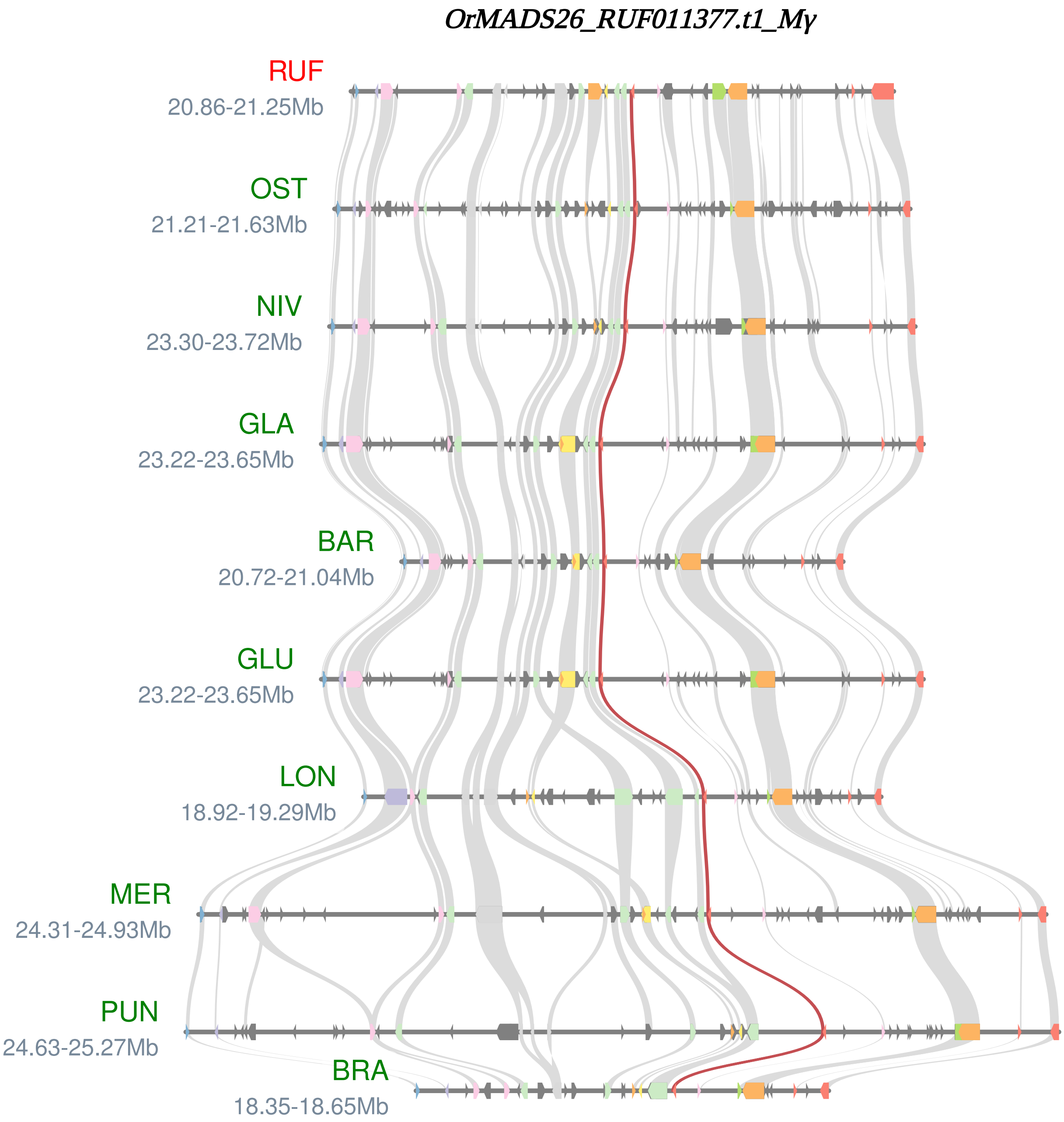
OrMADS26_RUF011377.t1_Mγ
RUF
20.86-21.25Mb
OST
21.21-21.63Mb
NIV
23.30-23.72Mb
GLA
23.22-23.65Mb
BAR
20.72-21.04Mb
GLU
23.22-23.65Mb
LON
18.92-19.29Mb
MER
24.31-24.93Mb
PUN
24.63-25.27Mb
BRA
18.35-18.65Mb

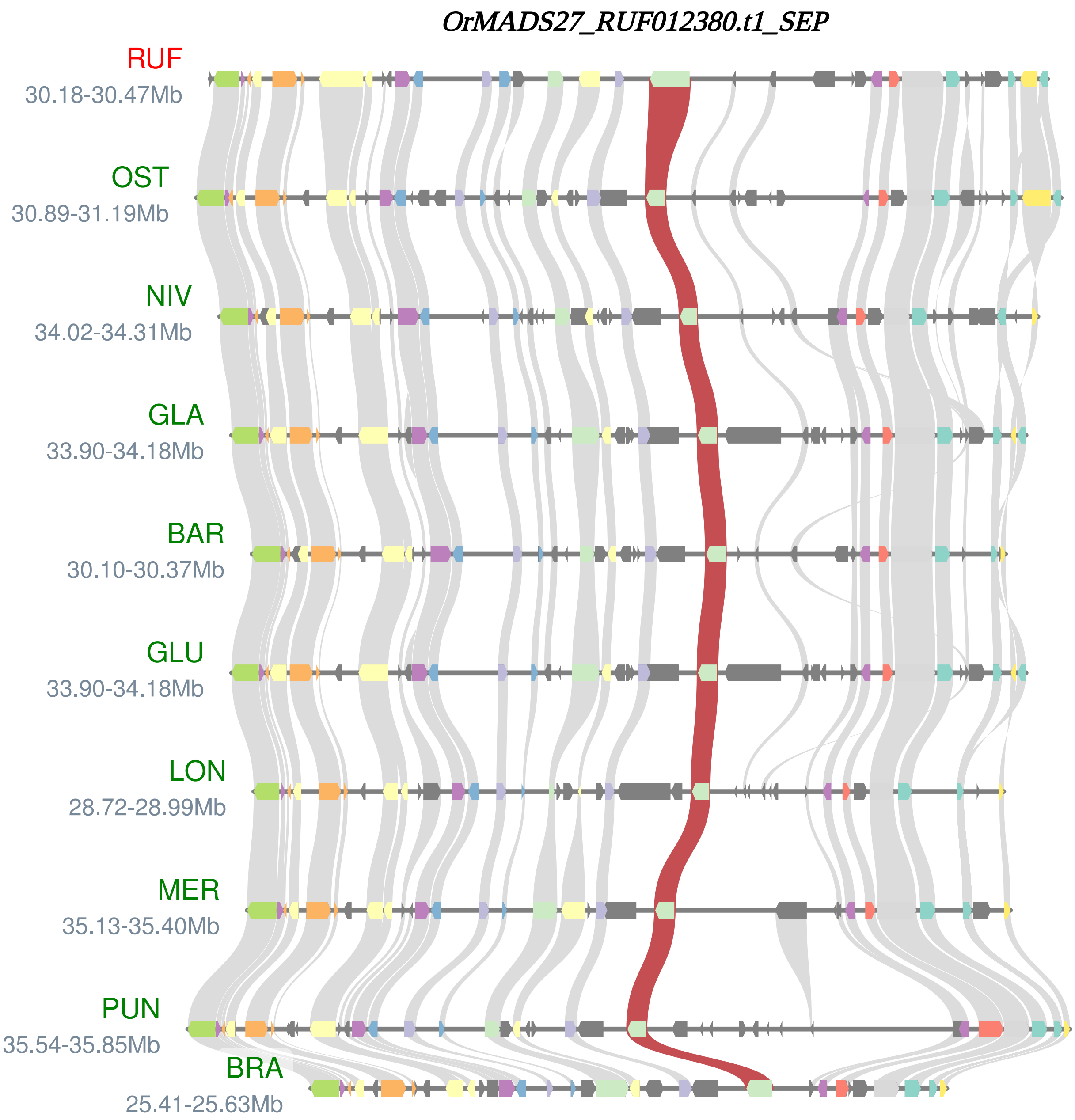
OrMADS27_RUF012380.t1_SEP
RUF
30.18-30.47Mb
OST
30.89-31.19Mb
NIV
34.02-34.31Mb
GLA
33.90-34.18Mb
BAR
30.10-30.37Mb
GLU
33.90-34.18Mb
LON
28.72-28.99Mb
MER
35.13-35.40Mb
PUN
35.54-35.85Mb
BRA
25.41-25.63Mb

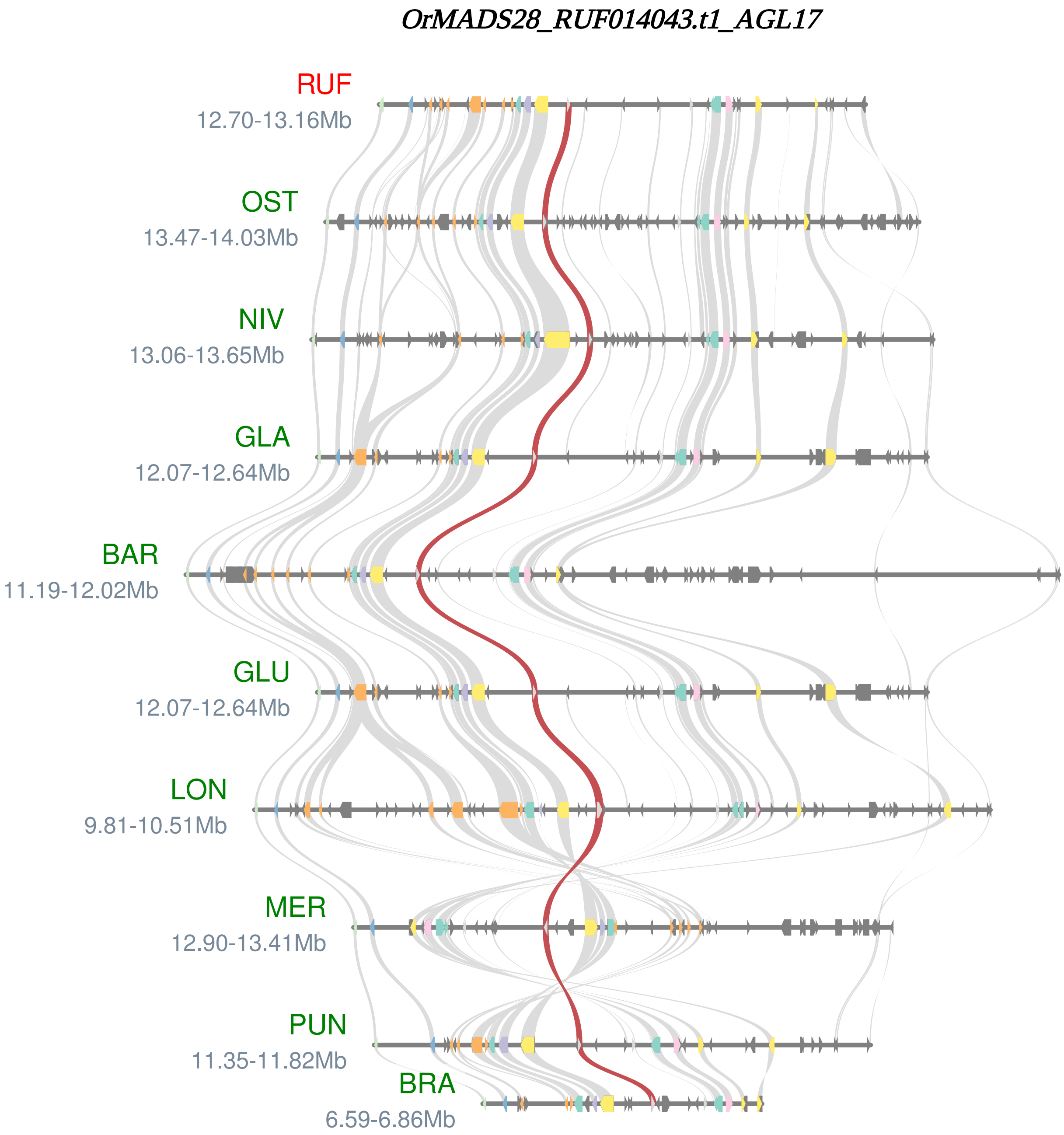

OrMADS28_RUF014043.t1_AGL17
RUF
12.70-13.16Mb
OST
13.47-14.03Mb
NIV
13.06-13.65Mb
GLA
12.07-12.64Mb
BAR
11.19-12.02Mb
GLU
12.07-12.64Mb
LON
9.81-10.51Mb
MER
12.90-13.41Mb
PUN
11.35-11.82Mb
BRA
6.59-6.86Mb

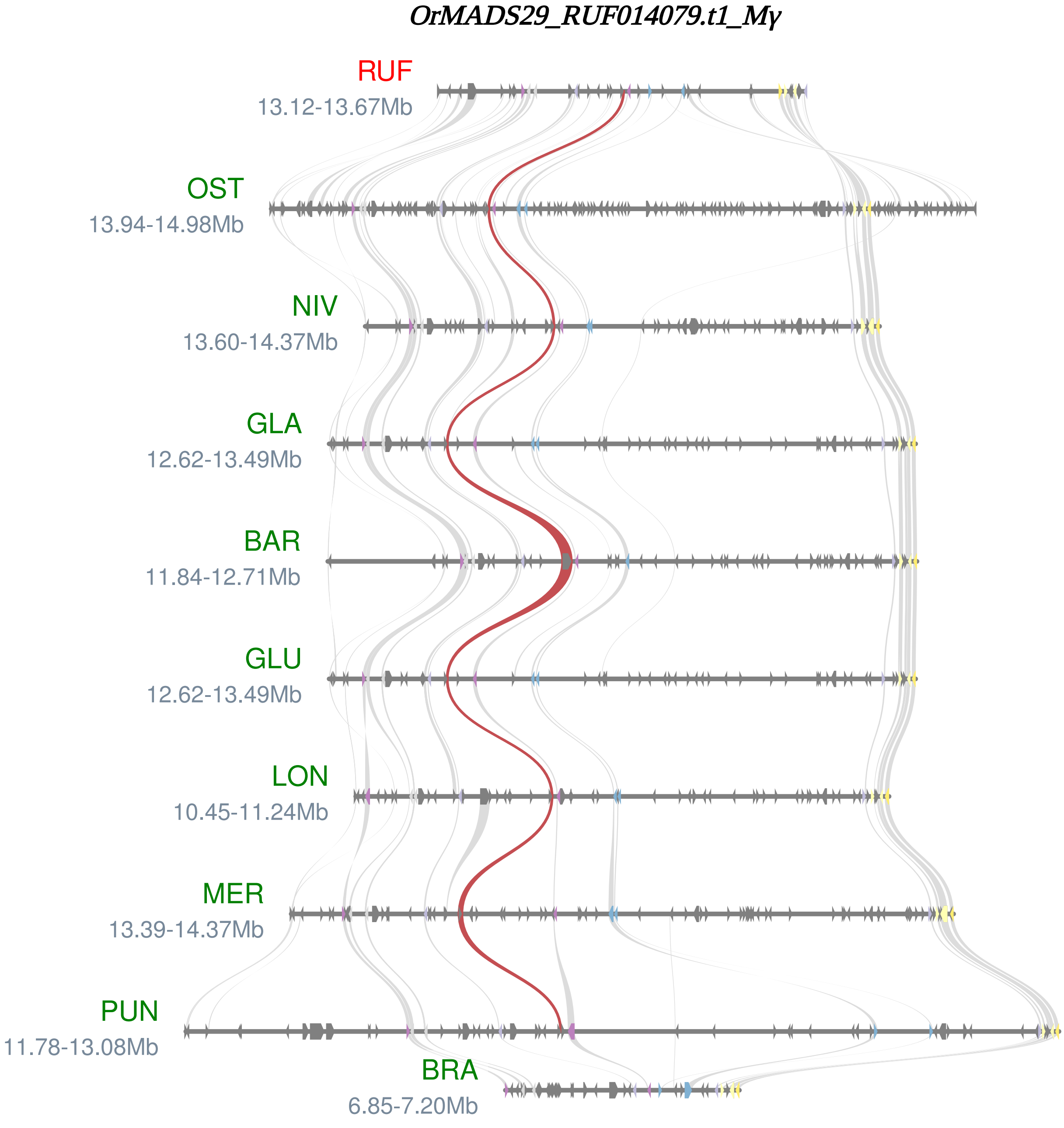
OrMADS29_RUF014079.t1_Mγ
RUF
13.12-13.67Mb
OST
13.94-14.98Mb
NIV
13.60-14.37Mb
GLA
12.62-13.49Mb
BAR
11.84-12.71Mb
GLU
12.62-13.49Mb
LON
10.45-11.24Mb
MER
13.39-14.37Mb
PUN
11.78-13.08Mb
BRA
6.85-7.20Mb

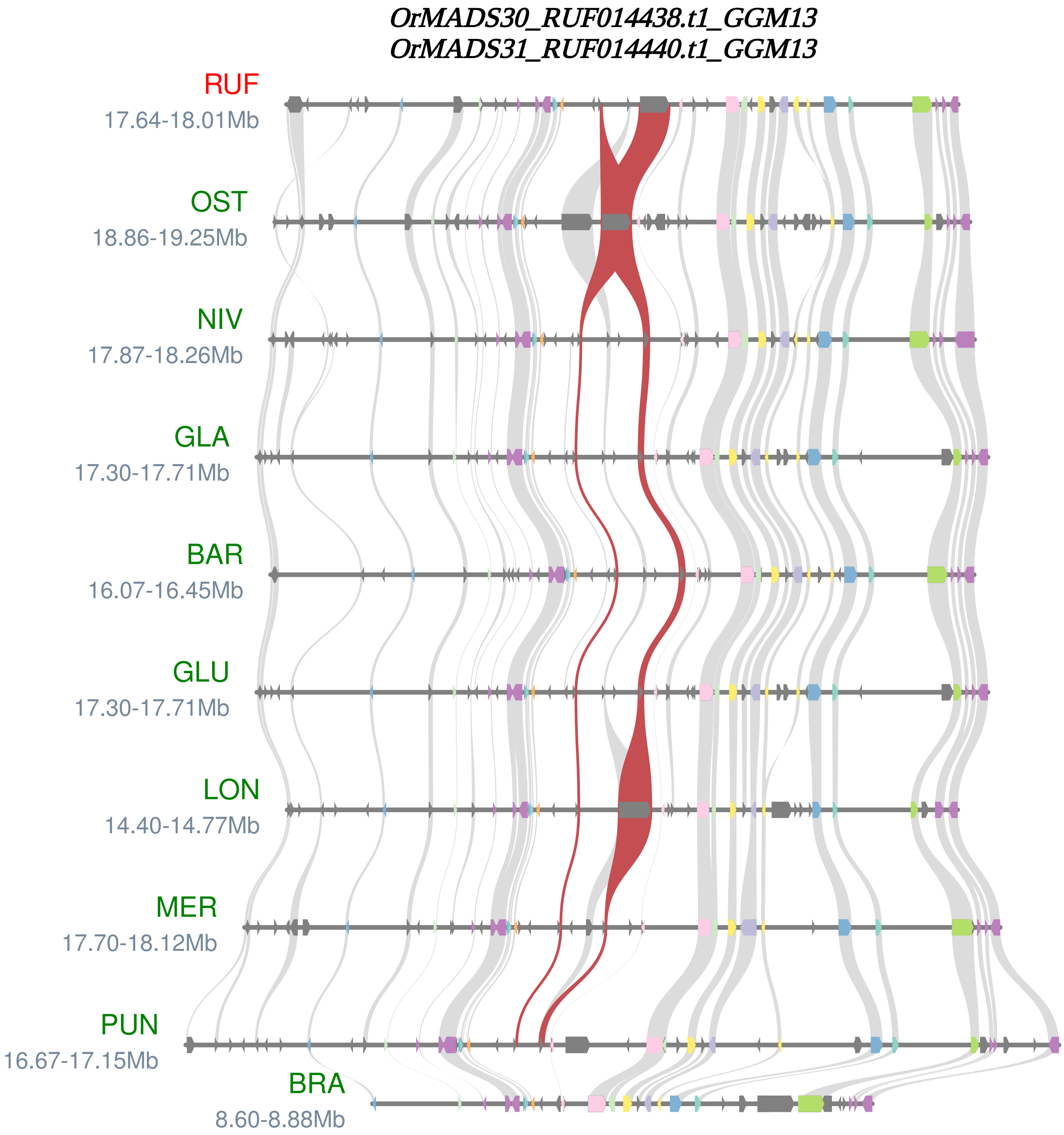

OrMADS30_RUF014438.t1_GGM13
OrMADS31_RUF014440.t1_GGM13
RUF
17.64-18.01Mb
OST
18.86-19.25Mb
NIV
17.87-18.26Mb
GLA
17.30-17.71Mb
BAR
16.07-16.45Mb
GLU
17.30-17.71Mb
LON
14.40-14.77Mb
MER
17.70-18.12Mb
PUN
16.67-17.15Mb
BRA
8.60-8.88Mb

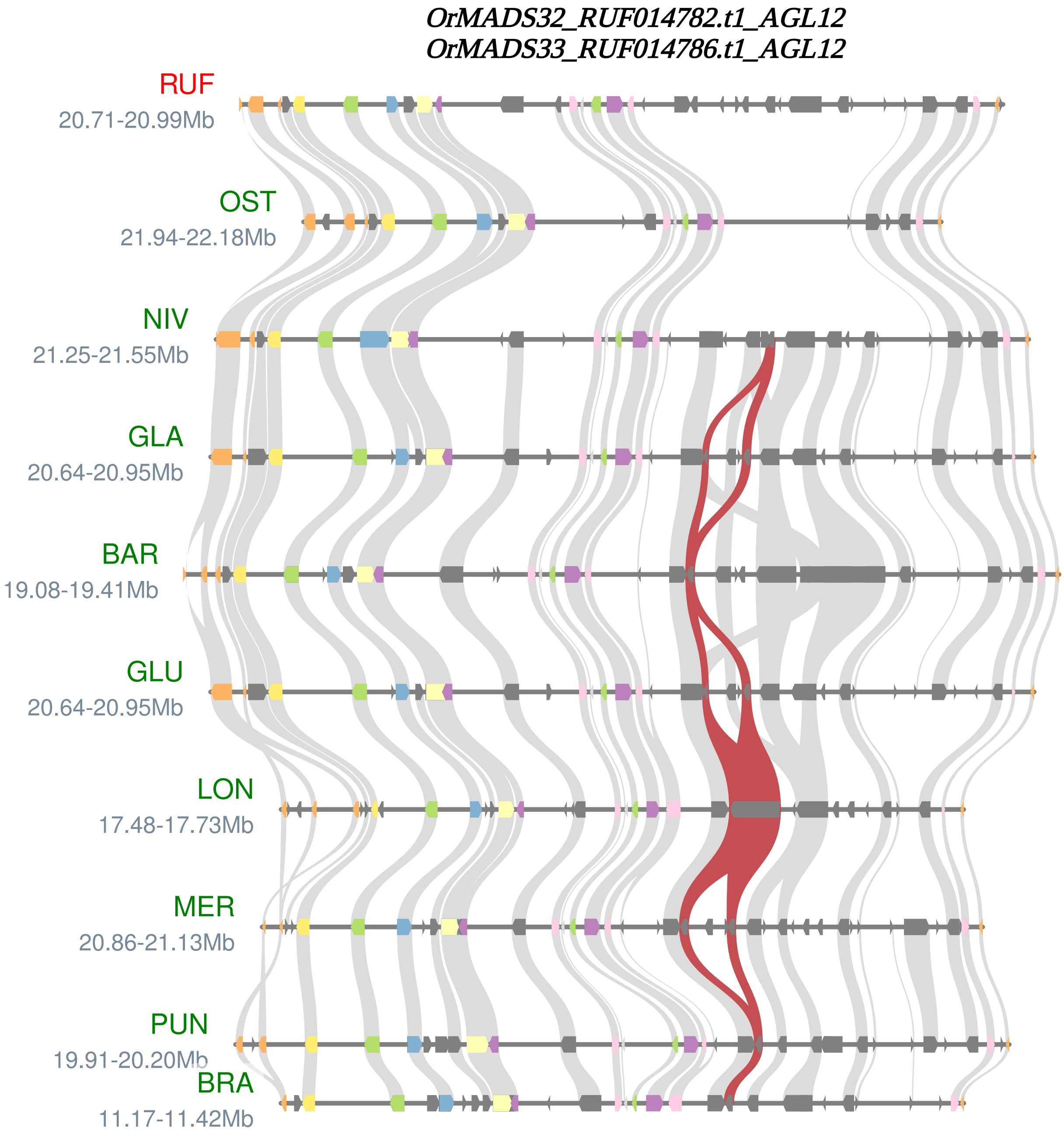

OrMADS32_RUF014782.t1_AGL12
OrMADS33_RUF014786.t1_AGL12
RUF
20.71-20.99Mb
OST
21.94-22.18Mb
NIV
21.25-21.55Mb
GLA
20.64-20.95Mb
BAR
19.08-19.41Mb
GLU
20.64-20.95Mb
LON
17.48-17.73Mb
MER
20.86-21.13Mb
PUN
19.91-20.20Mb
BRA
11.17-11.42Mb

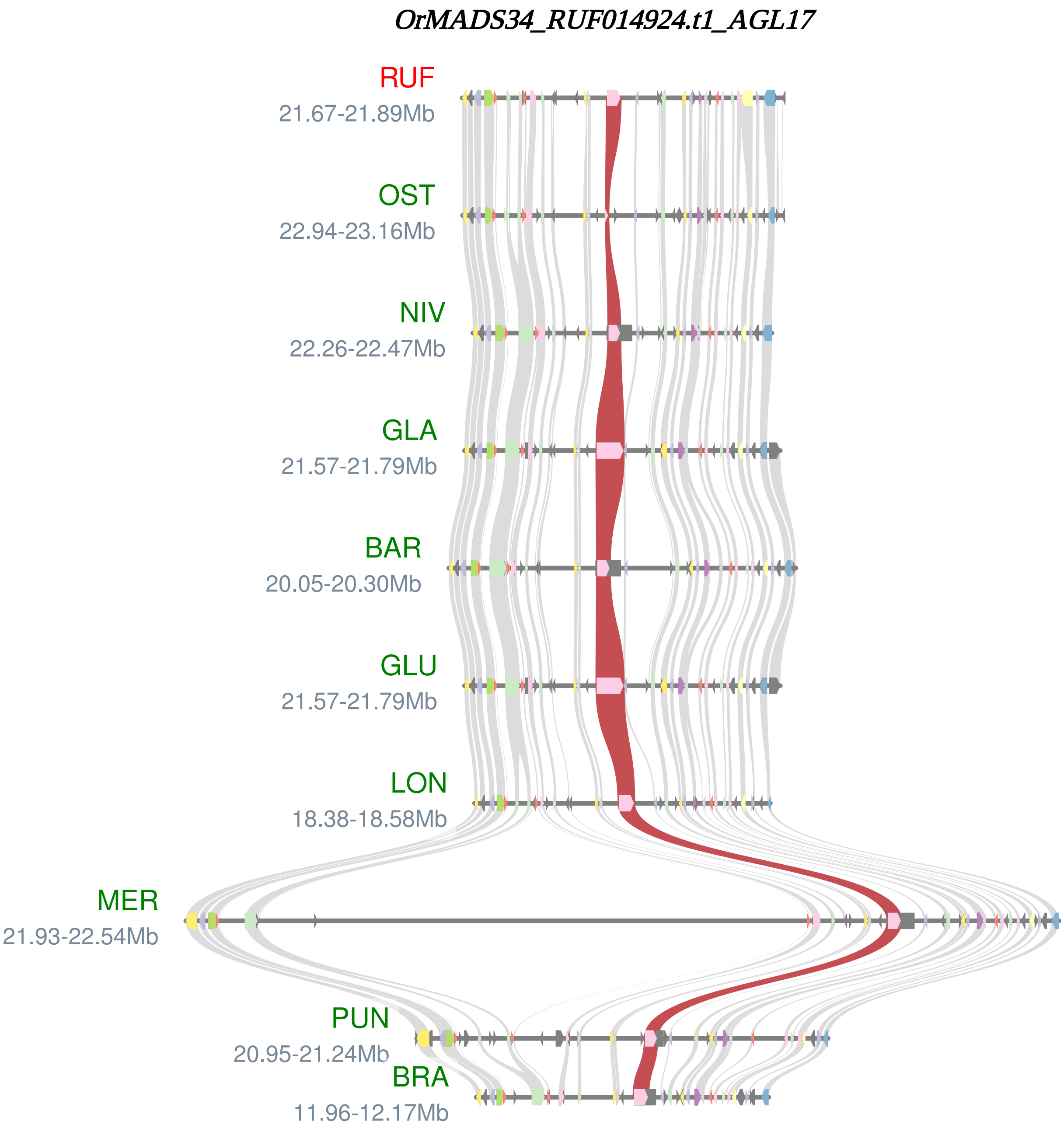

OrMADS34_RUF014924.t1_AGL17
RUF
21.67-21.89Mb
OST
22.94-23.16Mb
NIV
22.26-22.47Mb
GLA
21.57-21.79Mb
BAR
20.05-20.30Mb
GLU
21.57-21.79Mb
LON
18.38-18.58Mb
MER
21.93-22.54Mb
PUN
20.95-21.24Mb
BRA
11.96-12.17Mb

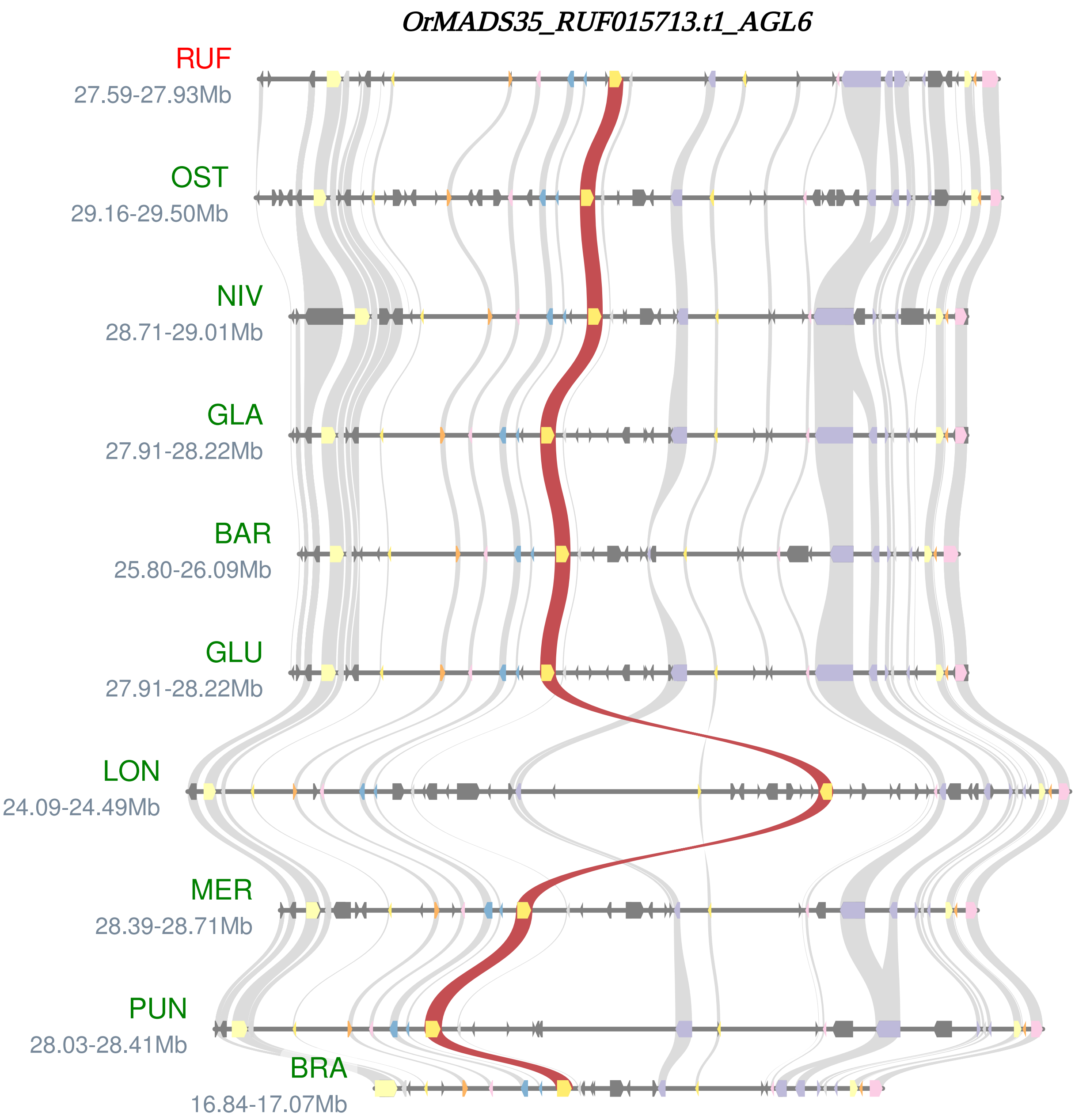
OrMADS35_RUF015713.t1_AGL6
RUF
27.59-27.93Mb
OST
29.16-29.50Mb
NIV
28.71-29.01Mb
GLA
27.91-28.22Mb
BAR
25.80-26.09Mb
GLU
27.91-28.22Mb
LON
24.09-24.49Mb
MER
28.39-28.71Mb
PUN
28.03-28.41Mb
BRA
16.84-17.07Mb

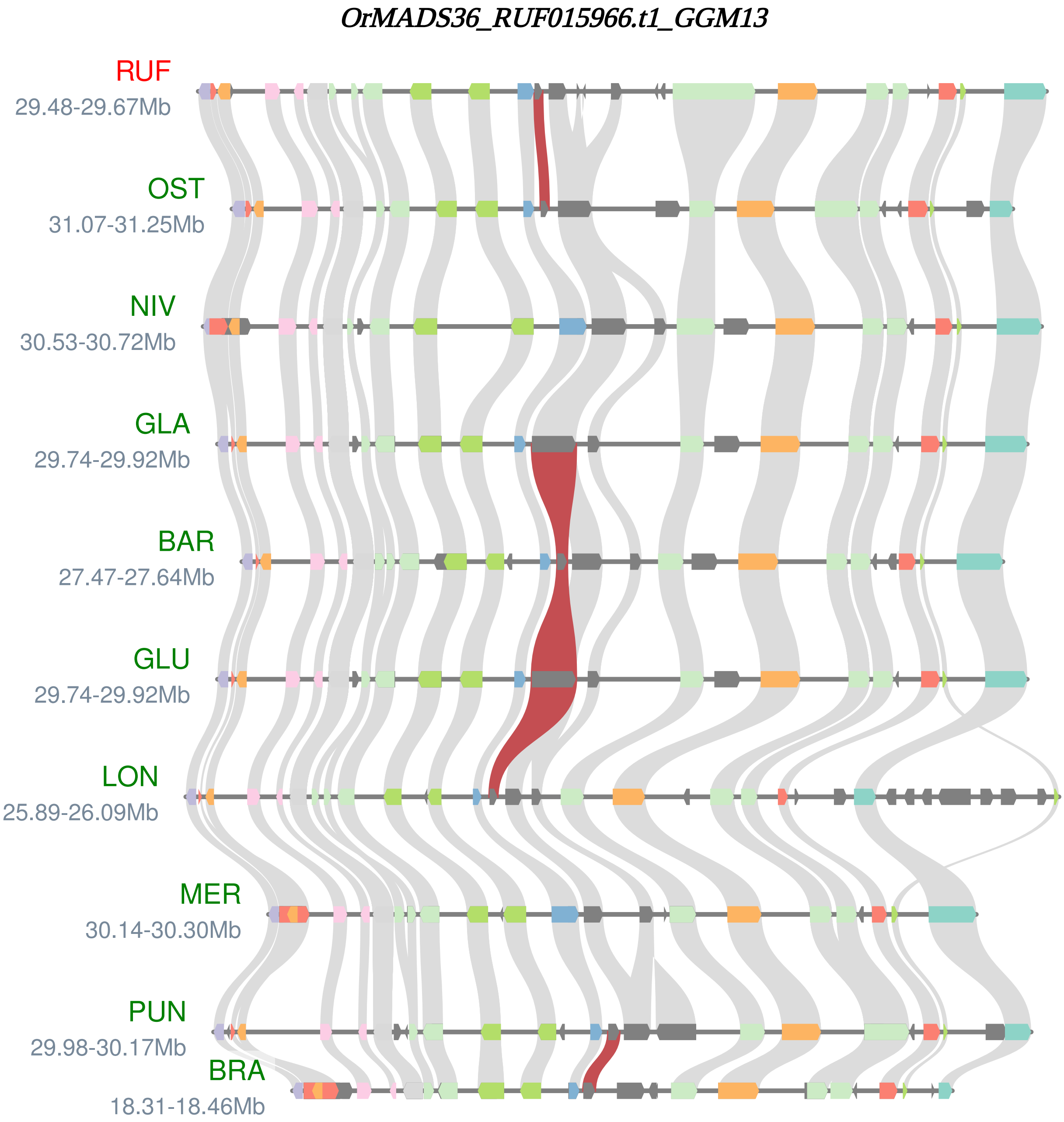
OrMADS36_RUF015966.t1_GGM13
RUF
29.48-29.67Mb
OST
31.07-31.25Mb
NIV
30.53-30.72Mb
GLA
29.74-29.92Mb
BAR
27.47-27.64Mb
GLU
29.74-29.92Mb
LON
25.89-26.09Mb
MER
30.14-30.30Mb
PUN
29.98-30.17Mb
BRA
18.31-18.46Mb

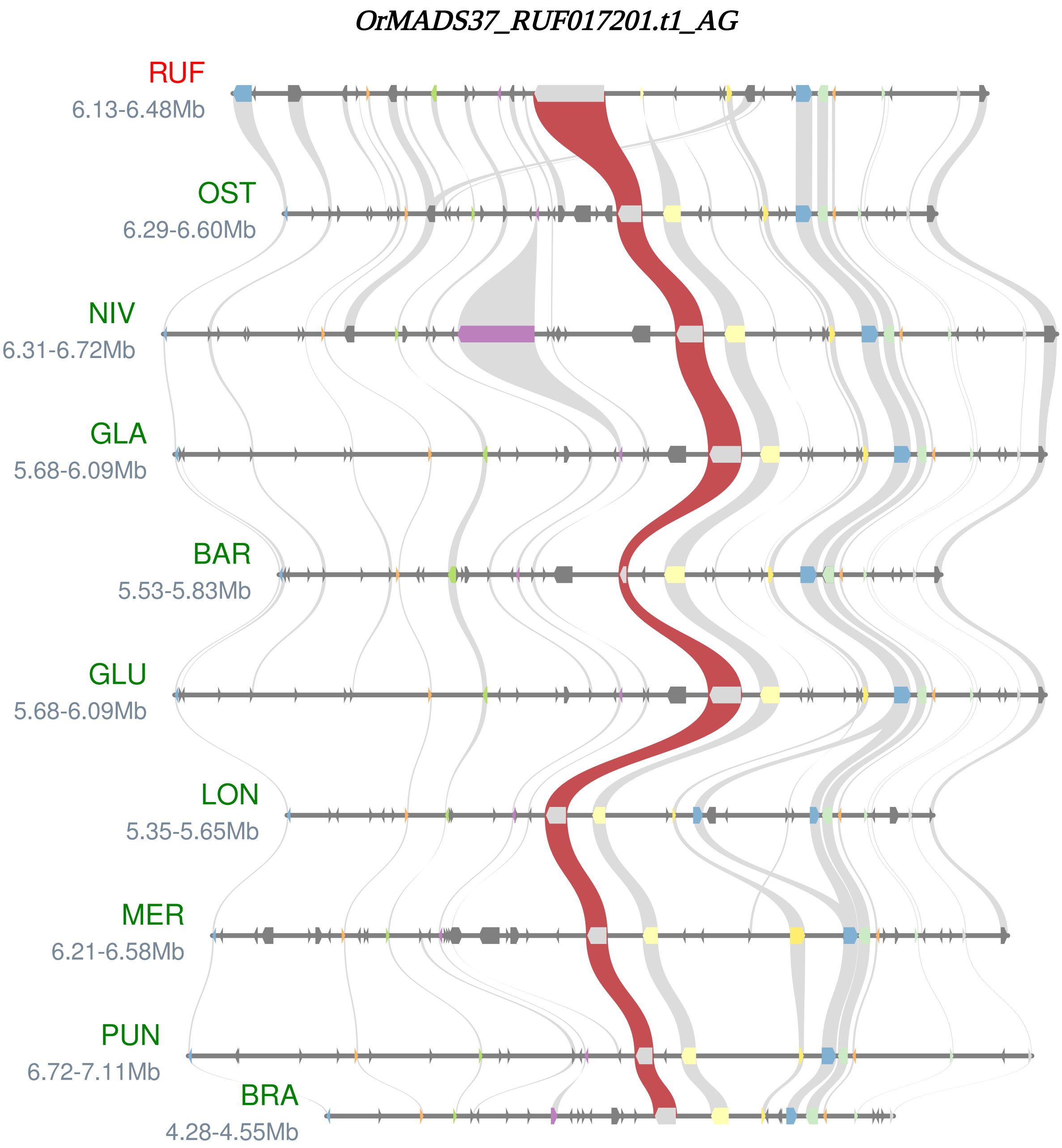
OrMADS37_RUF017201.t1_AG
RUF
6.13-6.48Mb
OST
6.29-6.60Mb
NIV
6.31-6.72Mb
GLA
5.68-6.09Mb
BAR
5.53-5.83Mb
GLU
5.68-6.09Mb
LON
5.35-5.65Mb
MER
6.21-6.58Mb
PUN
6.72-7.11Mb
BRA
4.28-4.55Mb

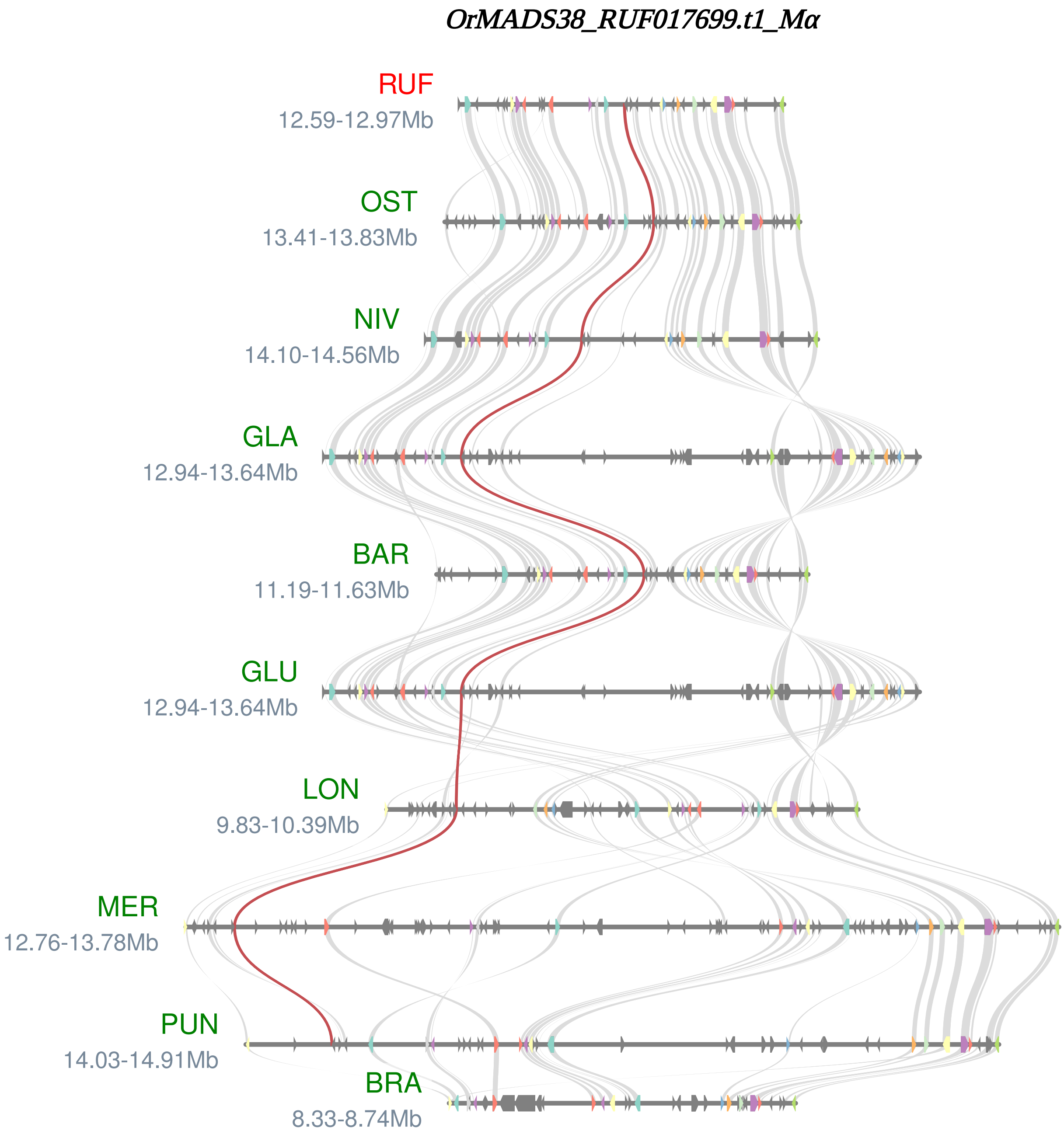

OrMADS38_RUF017699.t1_Mα
RUF
12.59-12.97Mb
OST
13.41-13.83Mb
NIV
14.10-14.56Mb
GLA
12.94-13.64Mb
BAR
11.19-11.63Mb
GLU
12.94-13.64Mb
LON
9.83-10.39Mb
MER
12.76-13.78Mb
PUN
14.03-14.91Mb
BRA
8.33-8.74Mb

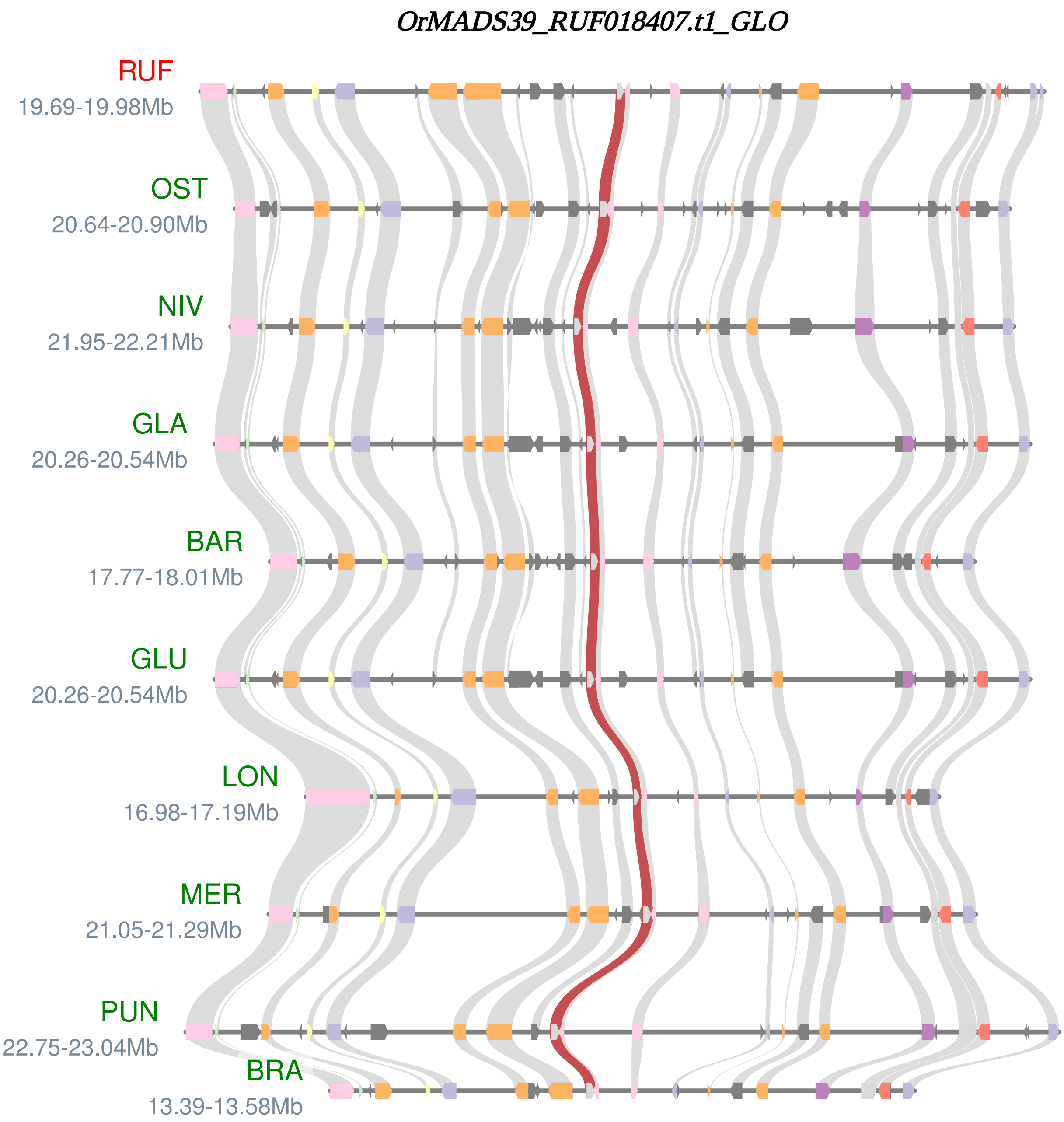
OrMADS39_RUF018407.t1_GLO
RUF
19.69-19.98Mb
OST
20.64-20.90Mb
NIV
21.95-22.21Mb
GLA
20.26-20.54Mb
BAR
17.77-18.01Mb
GLU
20.26-20.54Mb
LON
16.98-17.19Mb
MER
21.05-21.29Mb
PUN
22.75-23.04Mb
BRA
13.39-13.58Mb

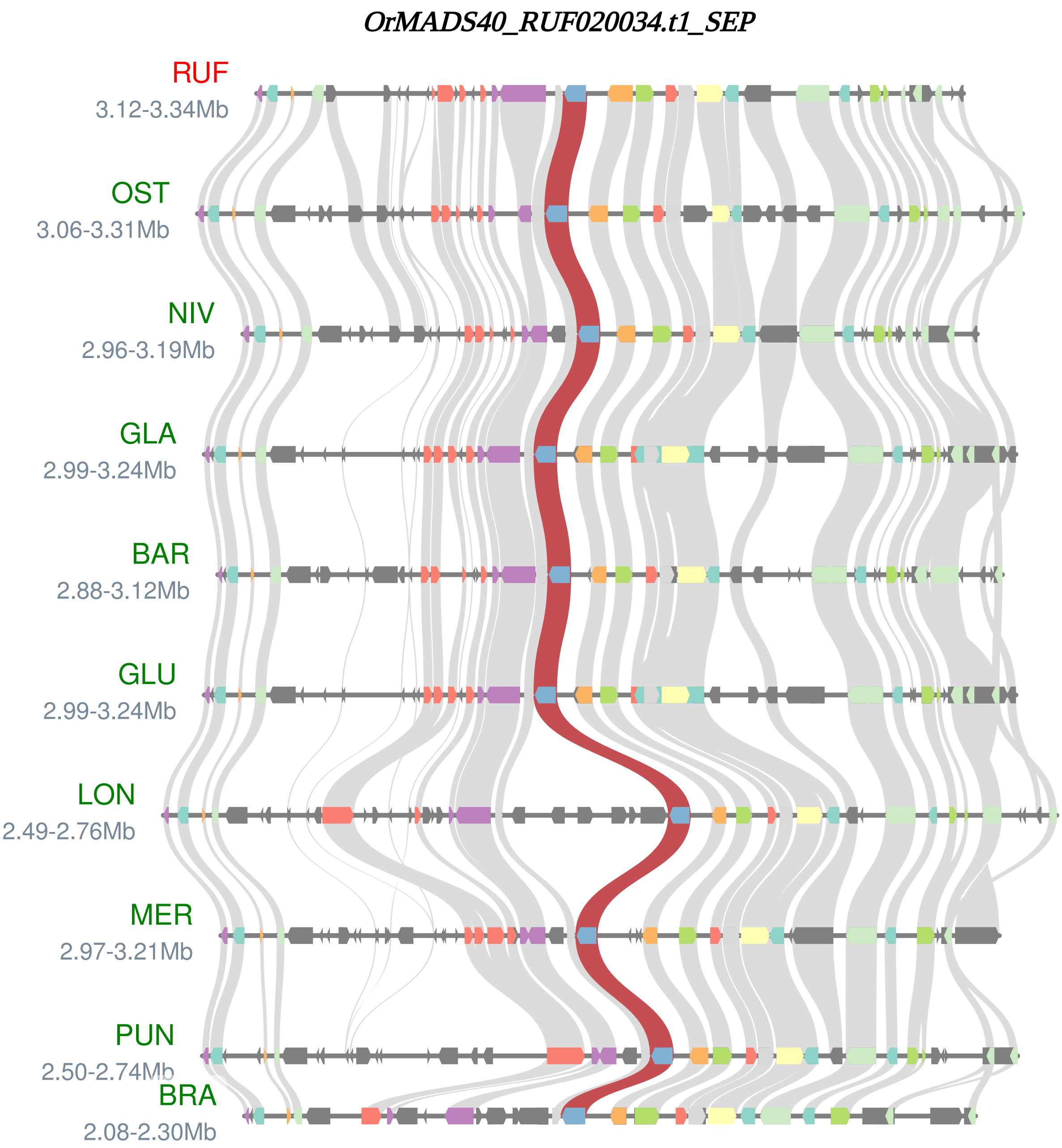
OrMADS40_RUF020034.t1_SEP
RUF
3.12-3.34Mb
OST
3.06-3.31Mb
NIV
2.96-3.19Mb
GLA
2.99-3.24Mb
BAR
2.88-3.12Mb
GLU
2.99-3.24Mb
LON
2.49-2.76Mb
MER
2.97-3.21Mb
PUN
2.50-2.74Mb
BRA
2.08-2.30Mb

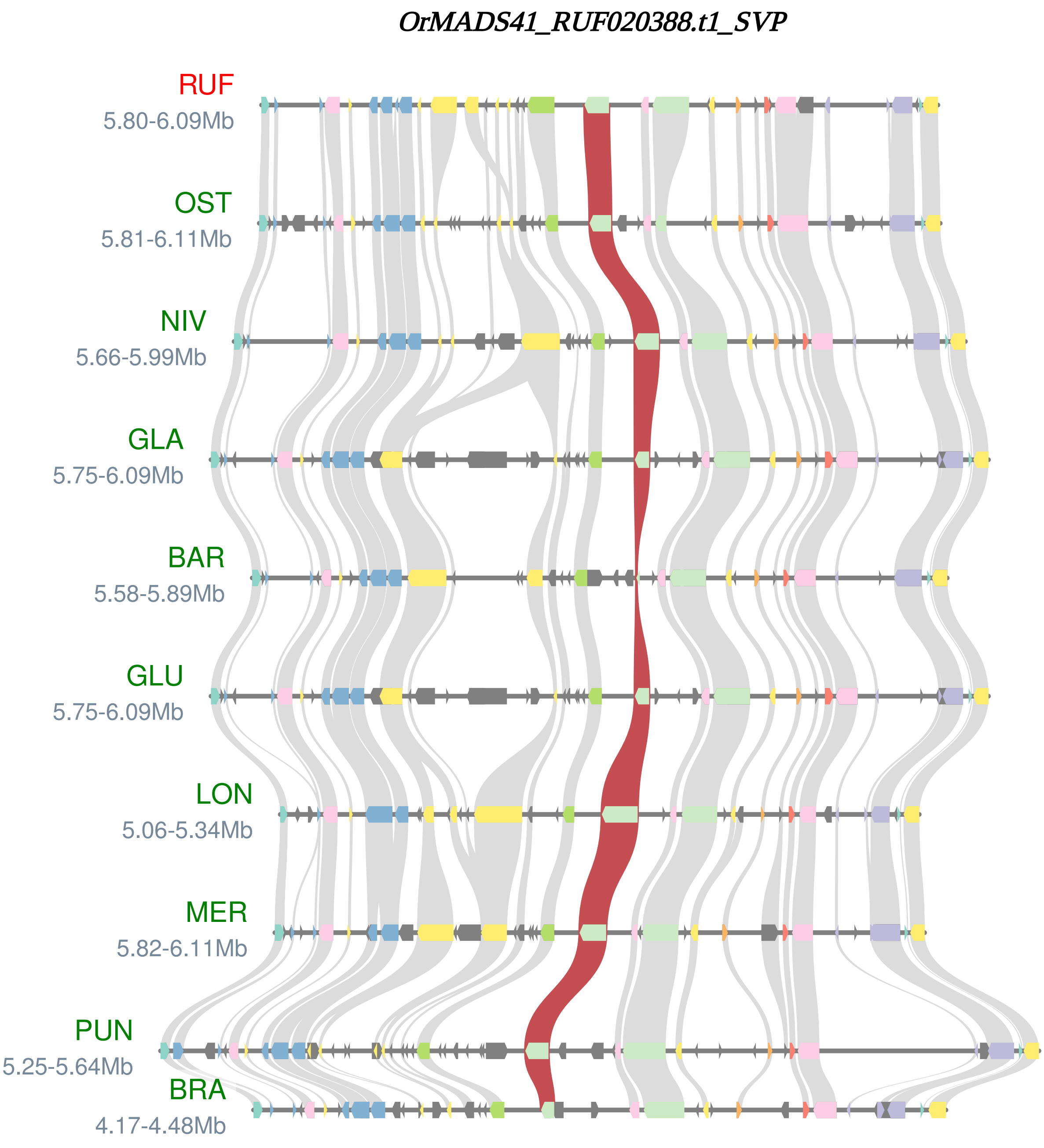

OrMADS41_RUF020388.t1_SVP
RUF
5.80-6.09Mb
OST
5.81-6.11Mb
NIV
5.66-5.99Mb
GLA
5.75-6.09Mb
BAR
5.58-5.89Mb
GLU
5.75-6.09Mb
LON
5.06-5.34Mb
MER
5.82-6.11Mb
PUN
5.25-5.64Mb
BRA
4.17-4.48Mb

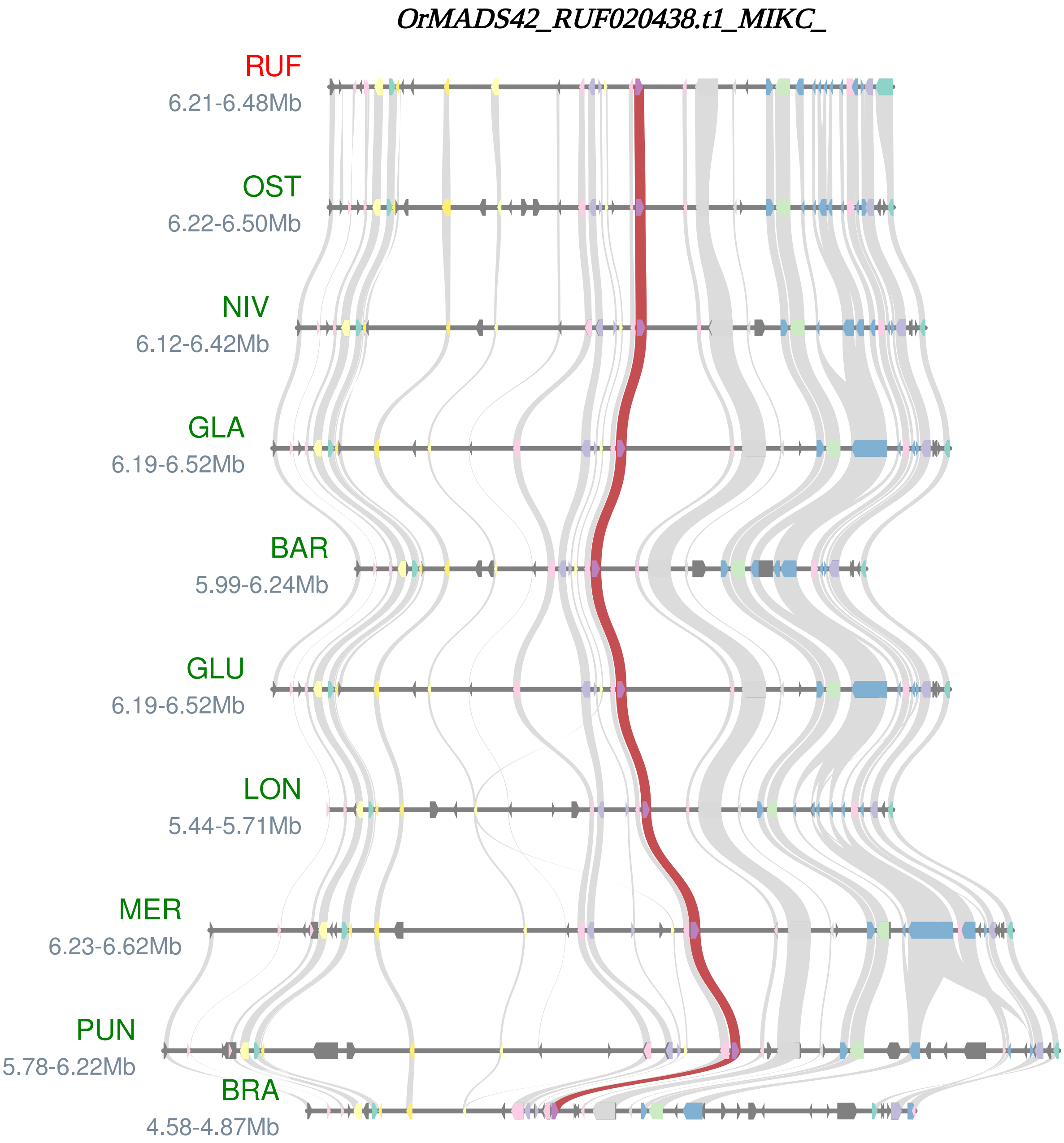

OrMADS42_RUF020438.t1_MIKC_
RUF
6.21-6.48Mb
OST
6.22-6.50Mb
NIV
6.12-6.42Mb
GLA
6.19-6.52Mb
BAR
5.99-6.24Mb
GLU
6.19-6.52Mb
LON
5.44-5.71Mb
MER
6.23-6.62Mb
PUN
5.78-6.22Mb
BRA
4.58-4.87Mb

OrMADS43_RUF021060.t1_Mα

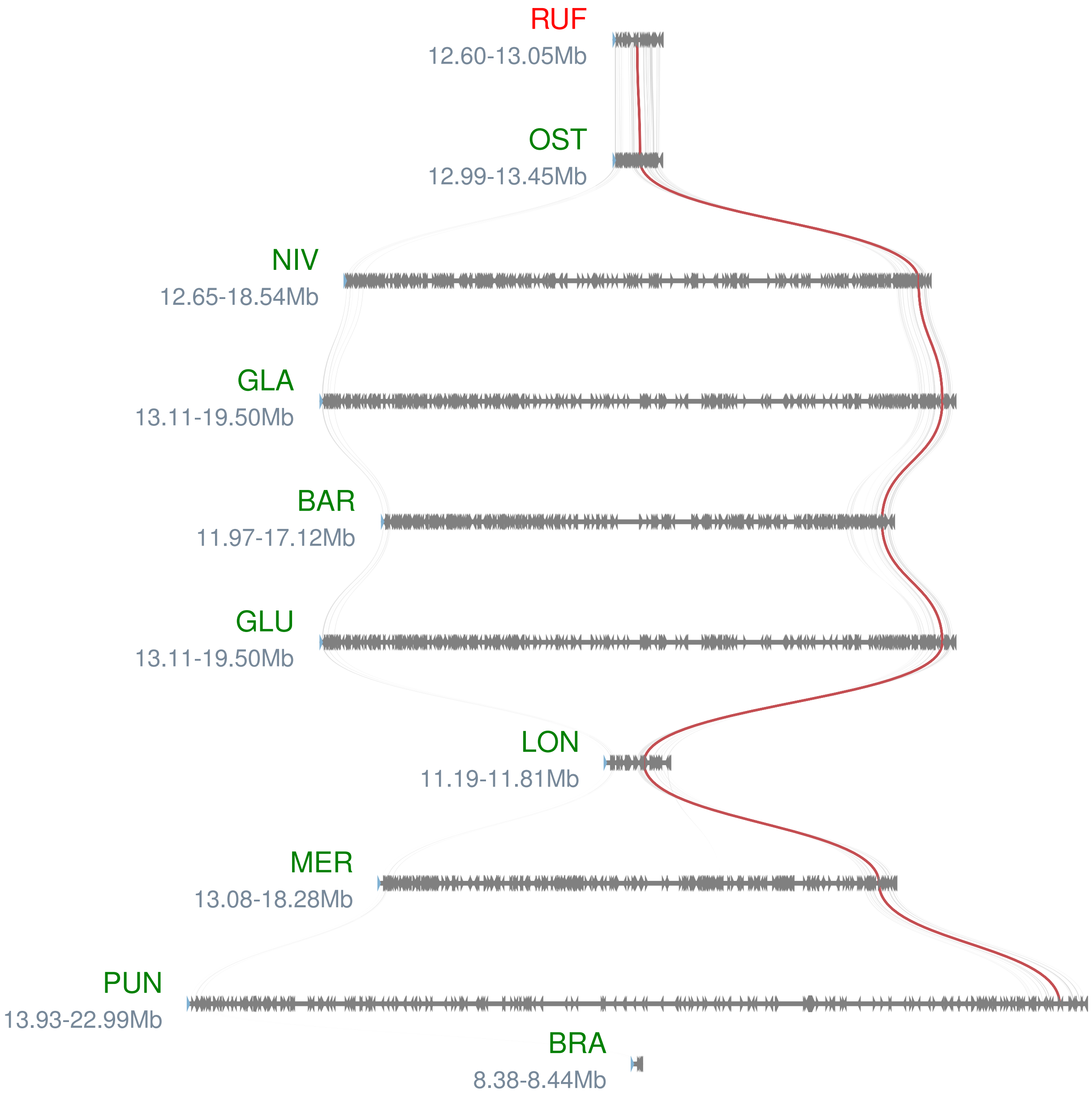

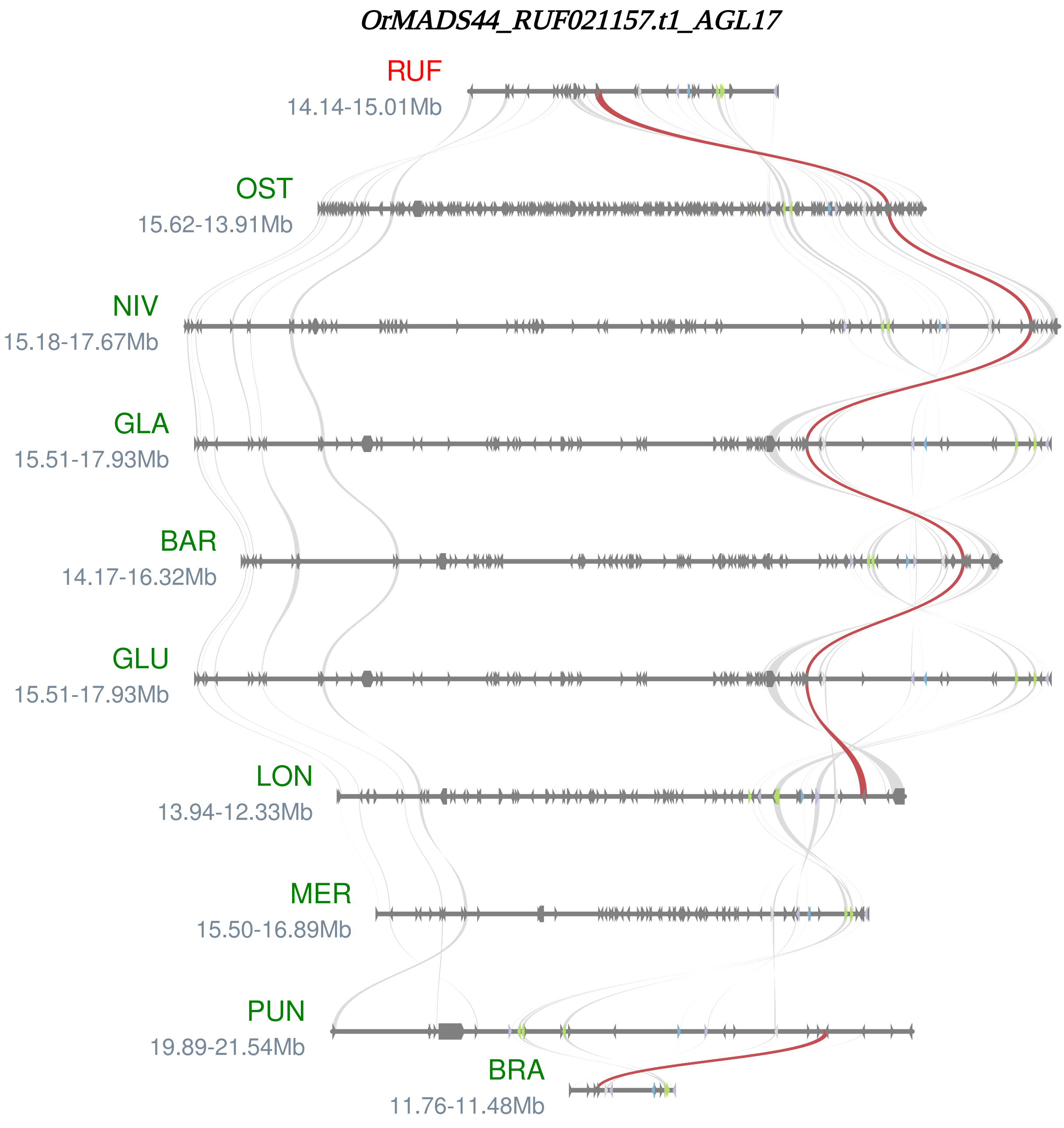
OrMADS44_RUF021157.t1_AGL17
RUF
14.14-15.01Mb
OST
15.62-13.91Mb
NIV
15.18-17.67Mb
GLA
15.51-17.93Mb
BAR
14.17-16.32Mb
GLU
15.51-17.93Mb
LON
13.94-12.33Mb
MER
15.50-16.89Mb
PUN
19.89-21.54Mb
BRA
11.76-11.48Mb

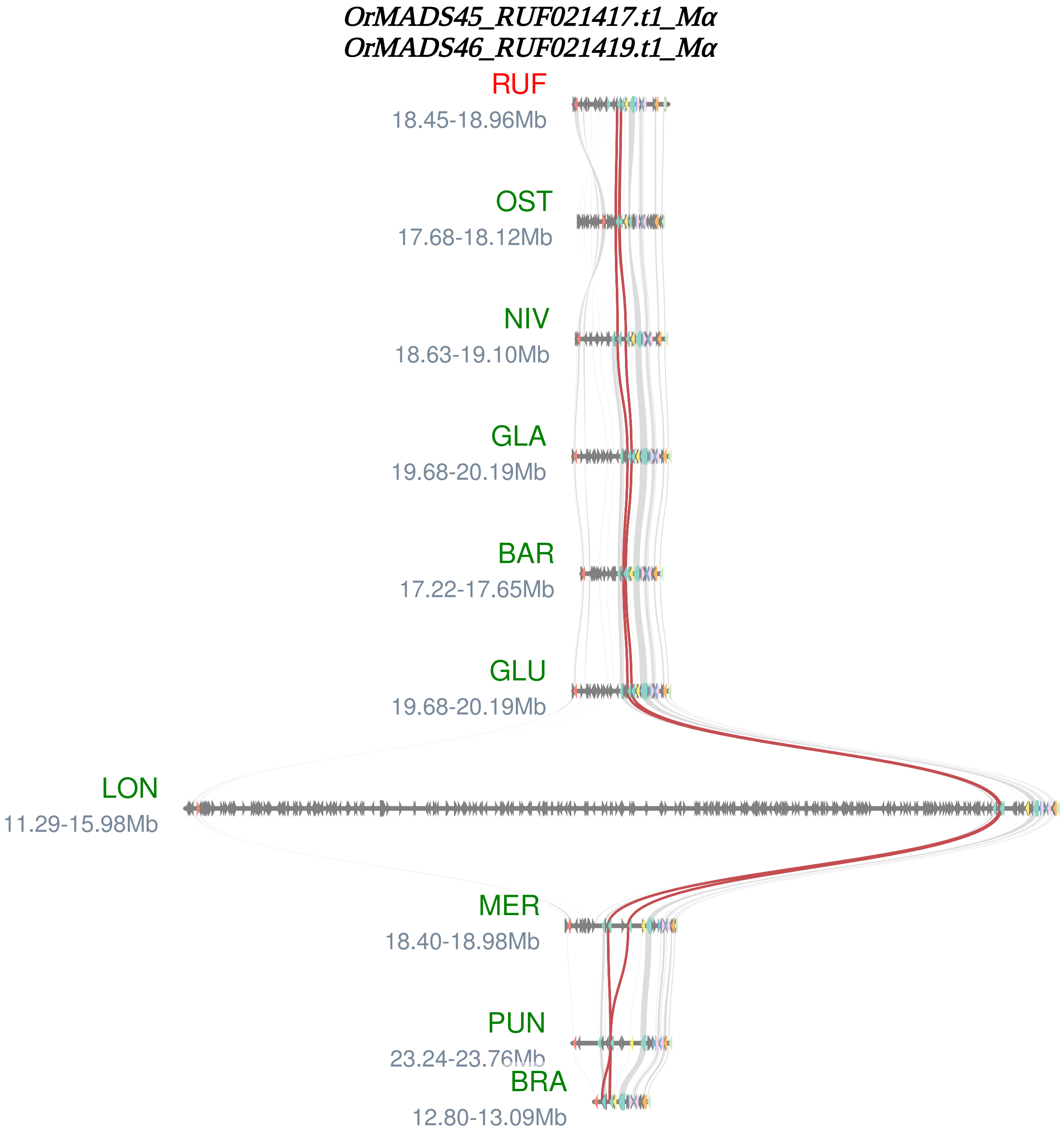
OrMADS45_RUF021417.t1_Mα
OrMADS46_RUF021419.t1_Mα
RUF
18.45-18.96Mb
OST
17.68-18.12Mb
NIV
18.63-19.10Mb
GLA
19.68-20.19Mb
BAR
17.22-17.65Mb
GLU
19.68-20.19Mb
LON
11.29-15.98Mb
MER
18.40-18.98Mb
PUN
23.24-23.76Mb
BRA
12.80-13.09Mb

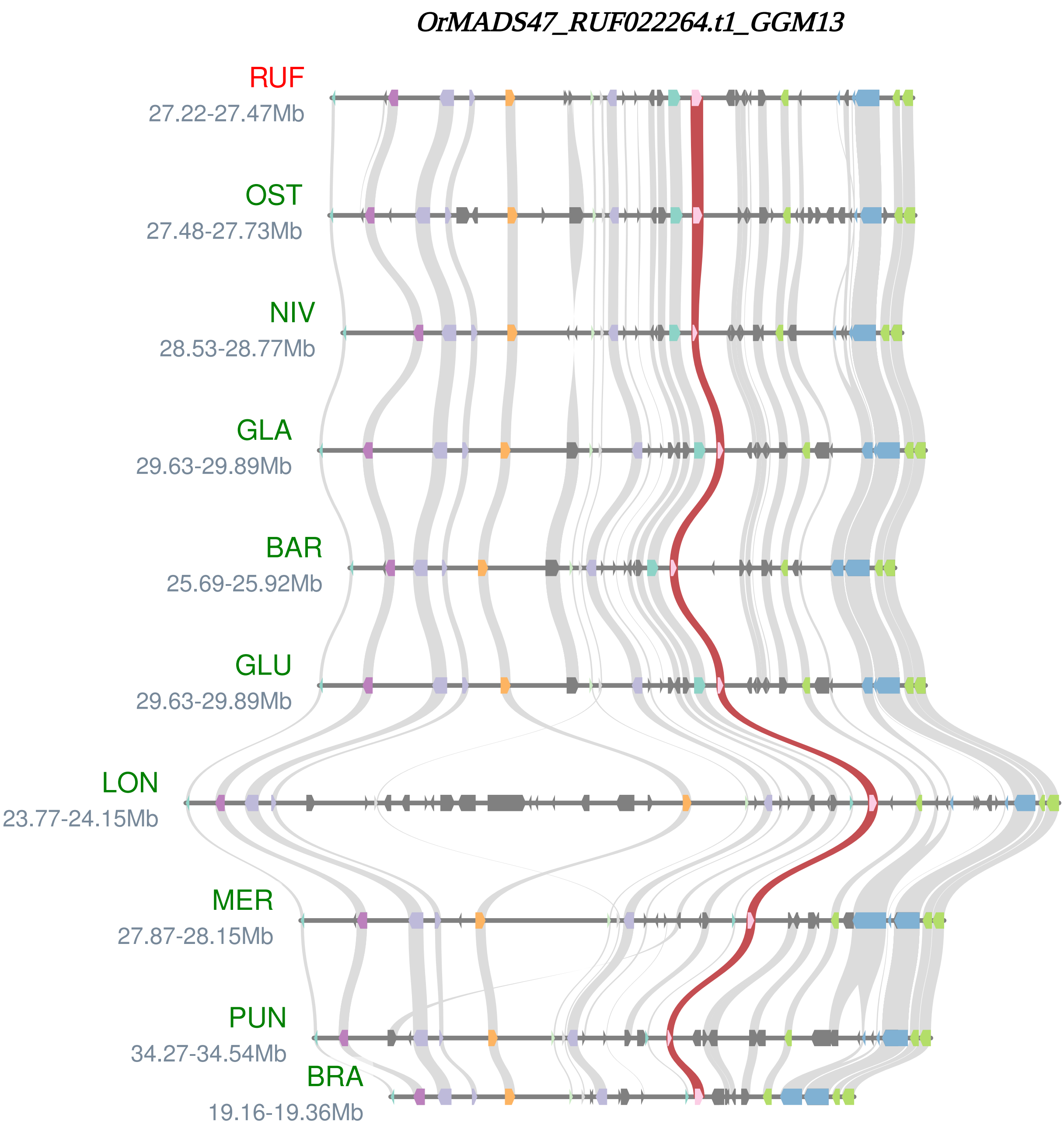

OrMADS47_RUF022264.t1_GGM13
RUF
27.22-27.47Mb
OST
27.48-27.73Mb
NIV
28.53-28.77Mb
GLA
29.63-29.89Mb
BAR
25.69-25.92Mb
GLU
29.63-29.89Mb
LON
23.77-24.15Mb
MER
27.87-28.15Mb
PUN
34.27-34.54Mb
BRA
19.16-19.36Mb

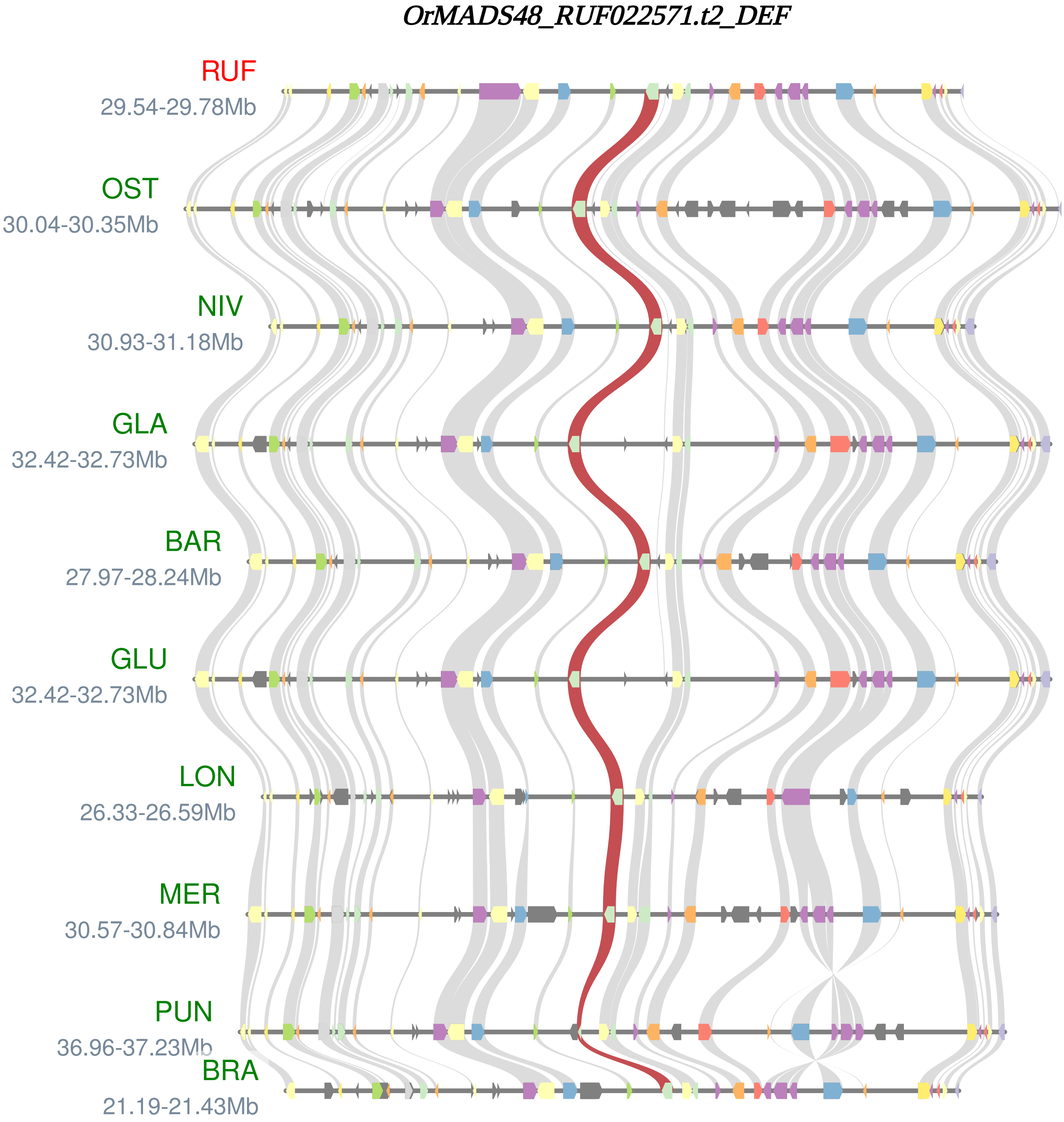

OrMADS48_RUF022571.t2_DEF
RUF
29.54-29.78Mb
OST
30.04-30.35Mb
NIV
30.93-31.18Mb
GLA
32.42-32.73Mb
BAR
27.97-28.24Mb
GLU
32.42-32.73Mb
LON
26.33-26.59Mb
MER
30.57-30.84Mb
PUN
36.96-37.23Mb
BRA
21.19-21.43Mb

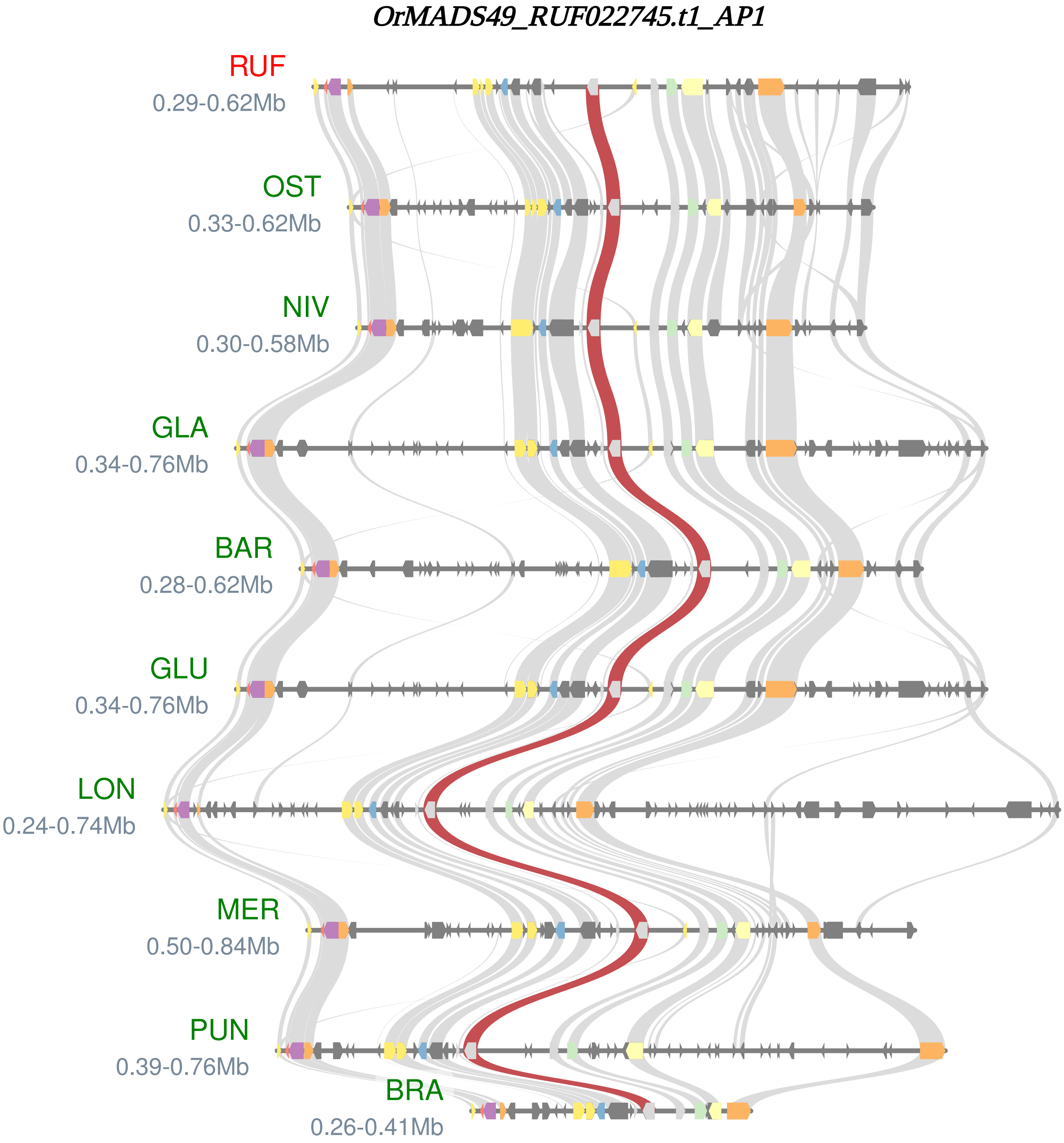

OrMADS49_RUF022745.t1_AP1
RUF
0.29-0.62Mb
OST
0.33-0.62Mb
NIV
0.30-0.58Mb
GLA
0.34-0.76Mb
BAR
0.28-0.62Mb
GLU
0.34-0.76Mb
LON
0.24-0.74Mb
MER
0.50-0.84Mb
PUN
0.39-0.76Mb
BRA
0.26-0.41Mb

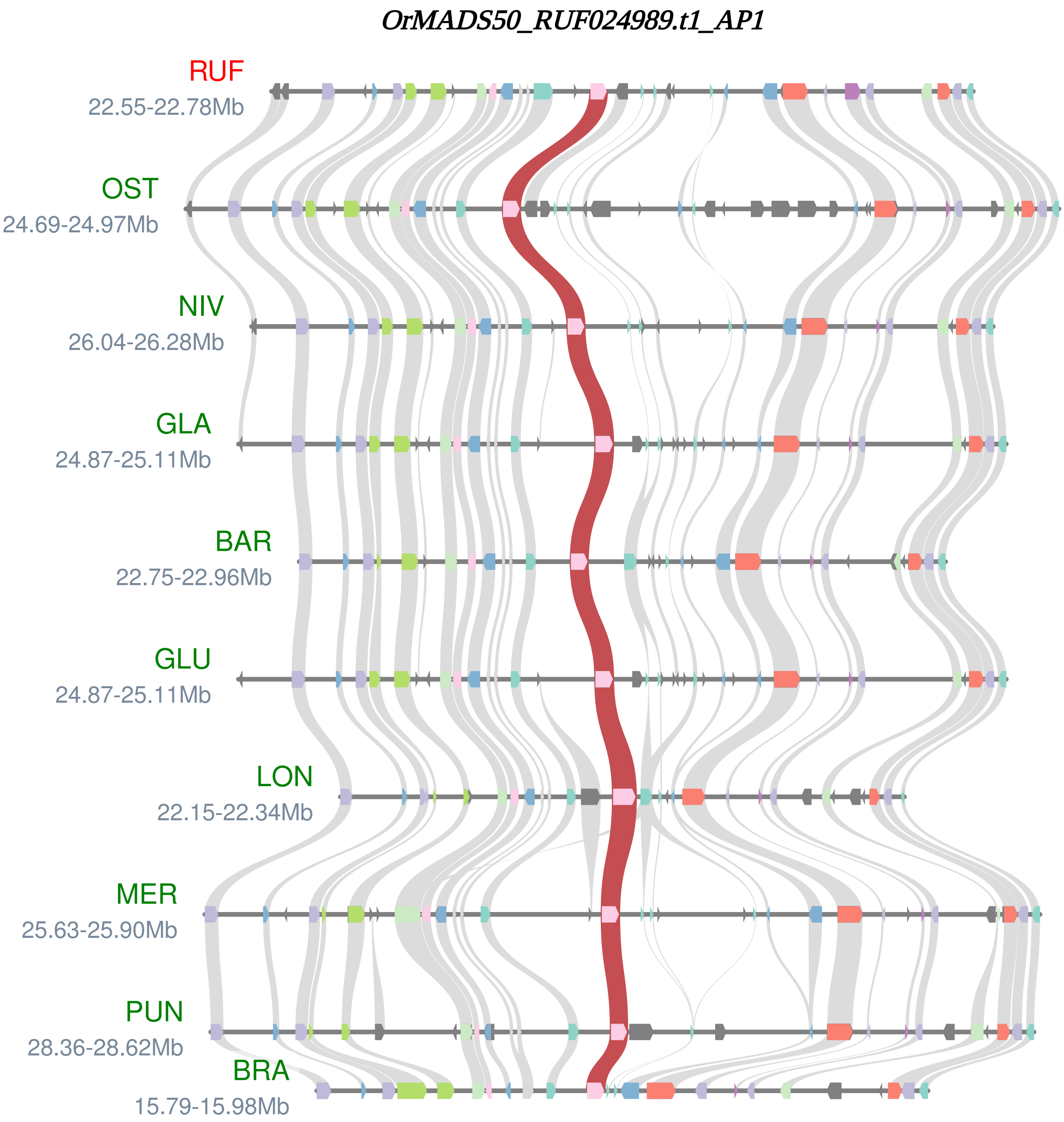
OrMADS50_RUF024989.t1_AP1
RUF
22.55-22.78Mb
OST
24.69-24.97Mb
NIV
26.04-26.28Mb
GLA
24.87-25.11Mb
BAR
22.75-22.96Mb
GLU
24.87-25.11Mb
LON
22.15-22.34Mb
MER
25.63-25.90Mb
PUN
28.36-28.62Mb
BRA
15.79-15.98Mb

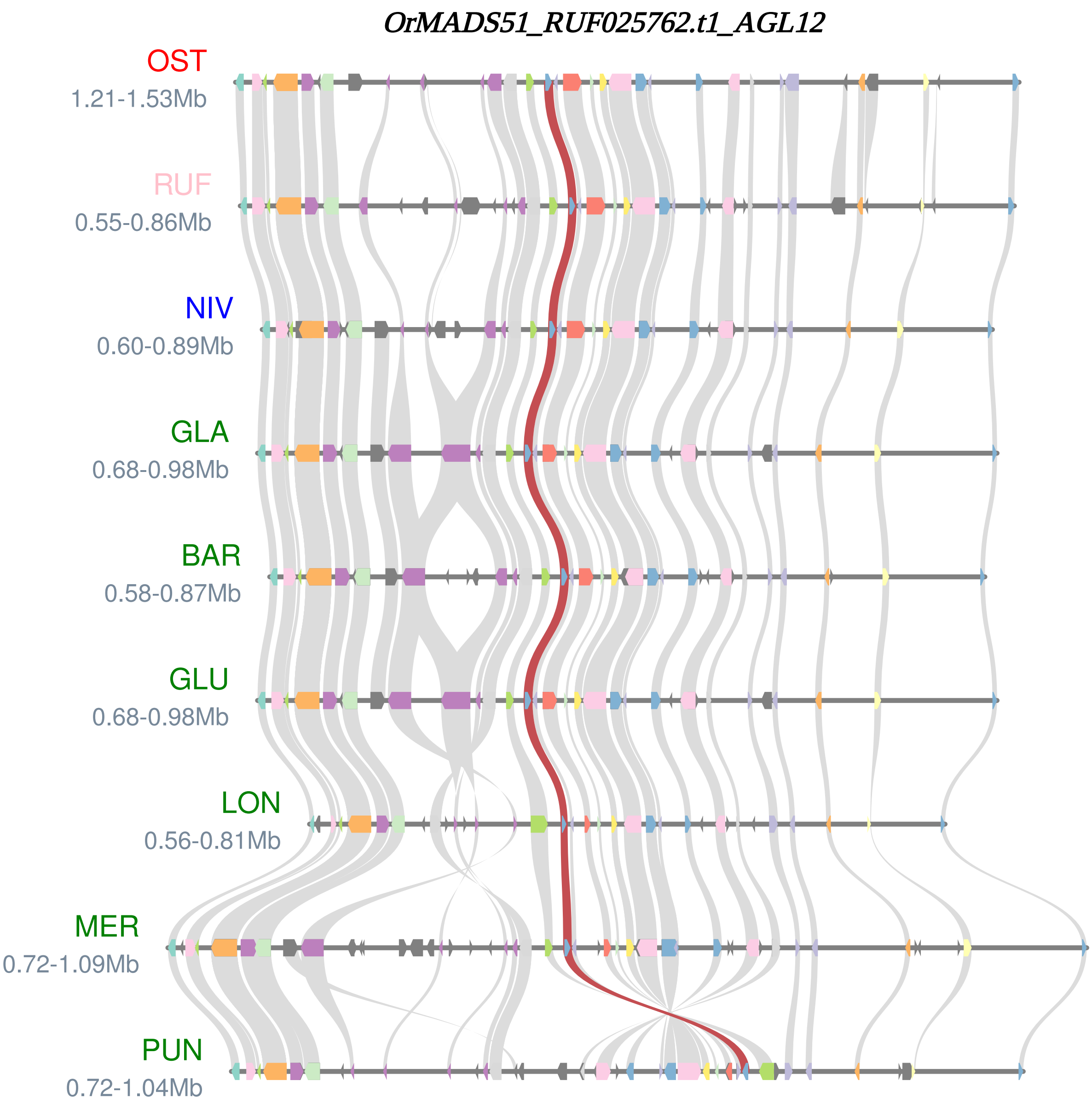

OrMADS51_RUF025762.t1_AGL12
OST
1.21-1.53Mb
RUF
0.55-0.86Mb
NIV
0.60-0.89Mb
GLA
0.68-0.98Mb
BAR
0.58-0.87Mb
GLU
0.68-0.98Mb
LON
0.56-0.81Mb
MER
0.72-1.09Mb
PUN
0.72-1.04Mb

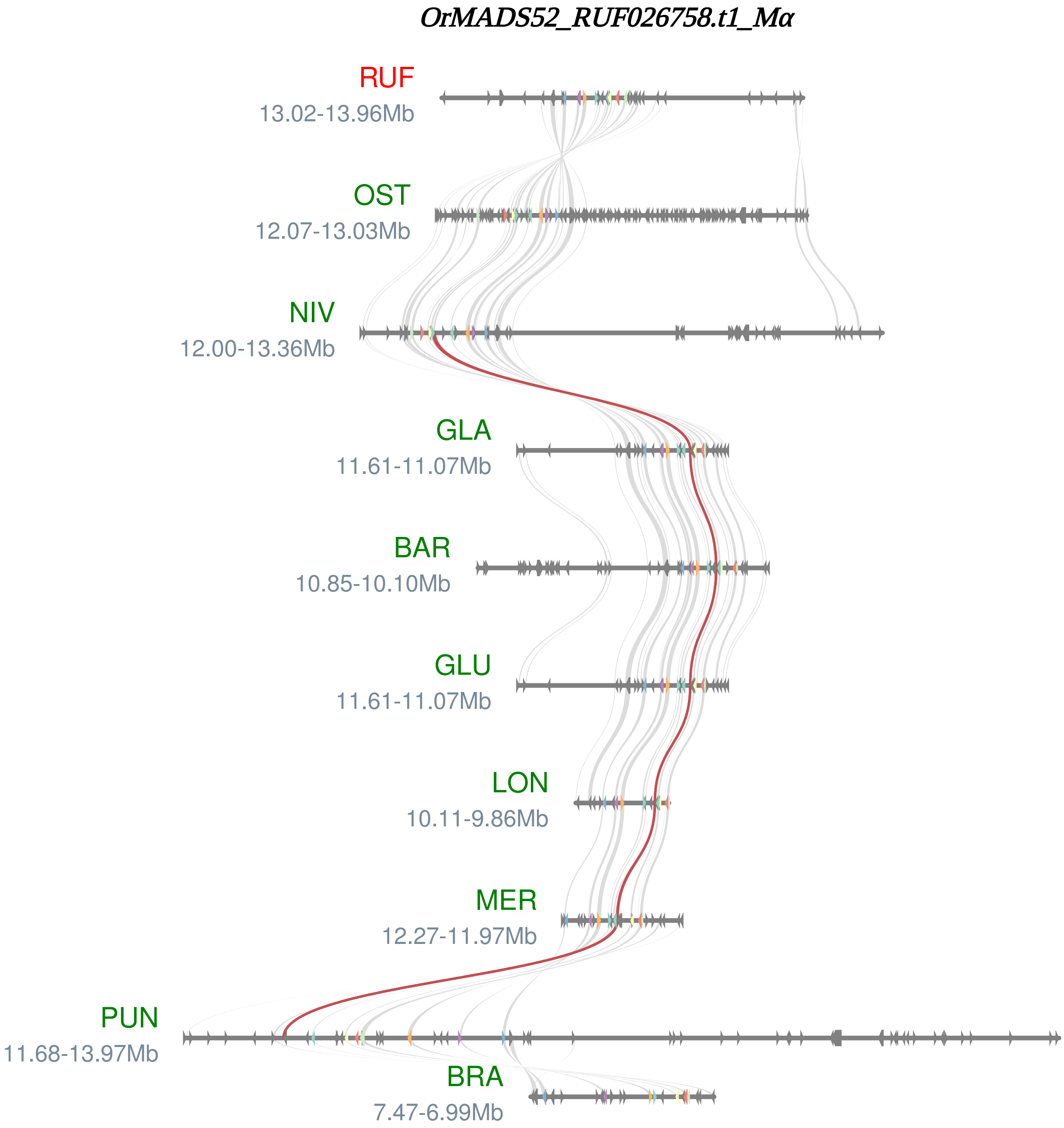

OrMADS52_RUF026758.t1_Mα
RUF
13.02-13.96Mb
OST
12.07-13.03Mb
NIV
12.00-13.36Mb
GLA
11.61-11.07Mb
BAR
10.85-10.10Mb
GLU
11.61-11.07Mb
LON
10.11-9.86Mb
MER
12.27-11.97Mb
PUN
11.68-13.97Mb
BRA
7.47-6.99Mb

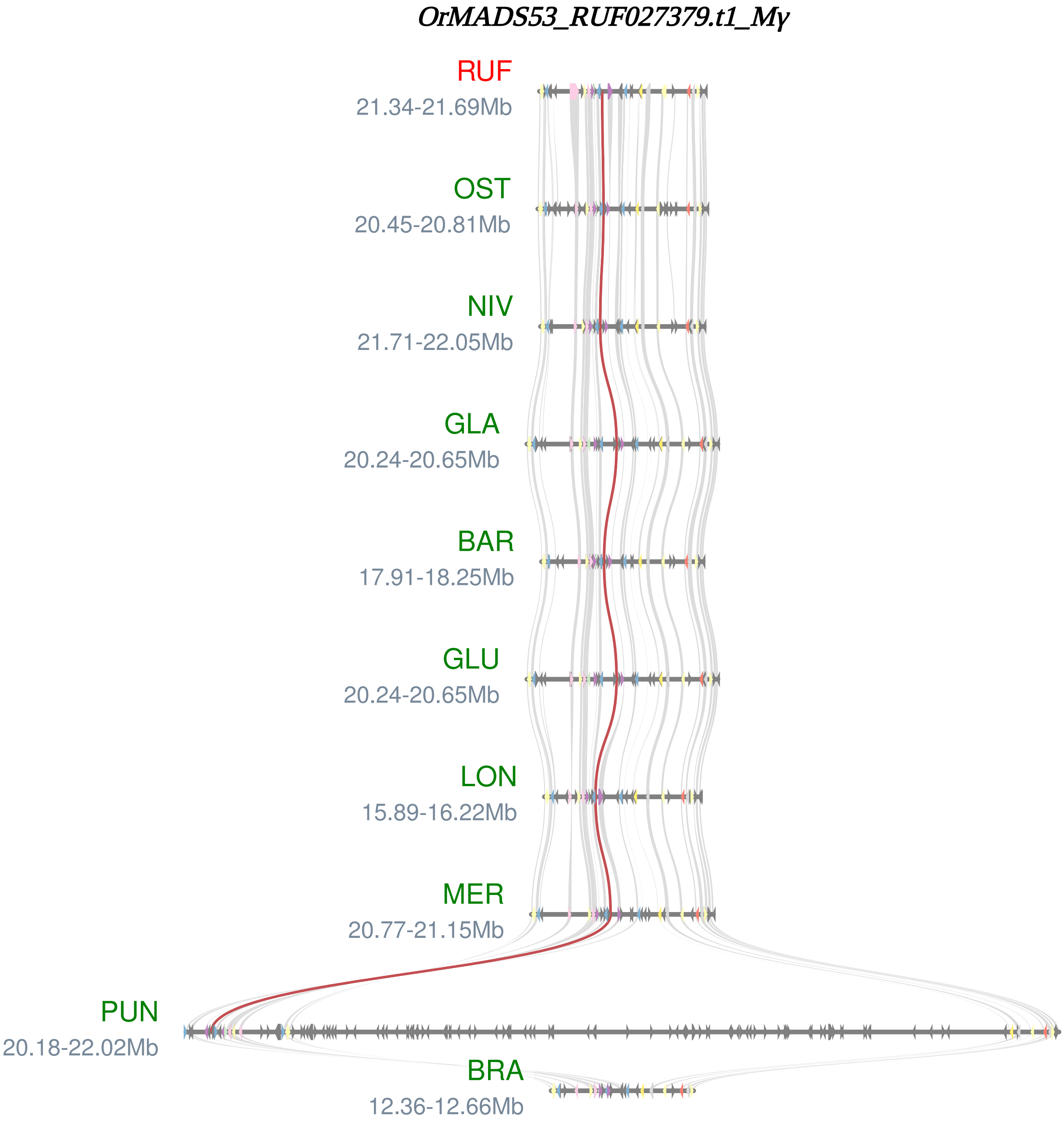
OrMADS53_RUF027379.t1_Mγ
RUF
21.34-21.69Mb
OST
20.45-20.81Mb
NIV
21.71-22.05Mb
GLA
20.24-20.65Mb
BAR
17.91-18.25Mb
GLU
20.24-20.65Mb
LON
15.89-16.22Mb
MER
20.77-21.15Mb
PUN
20.18-22.02Mb
BRA
12.36-12.66Mb

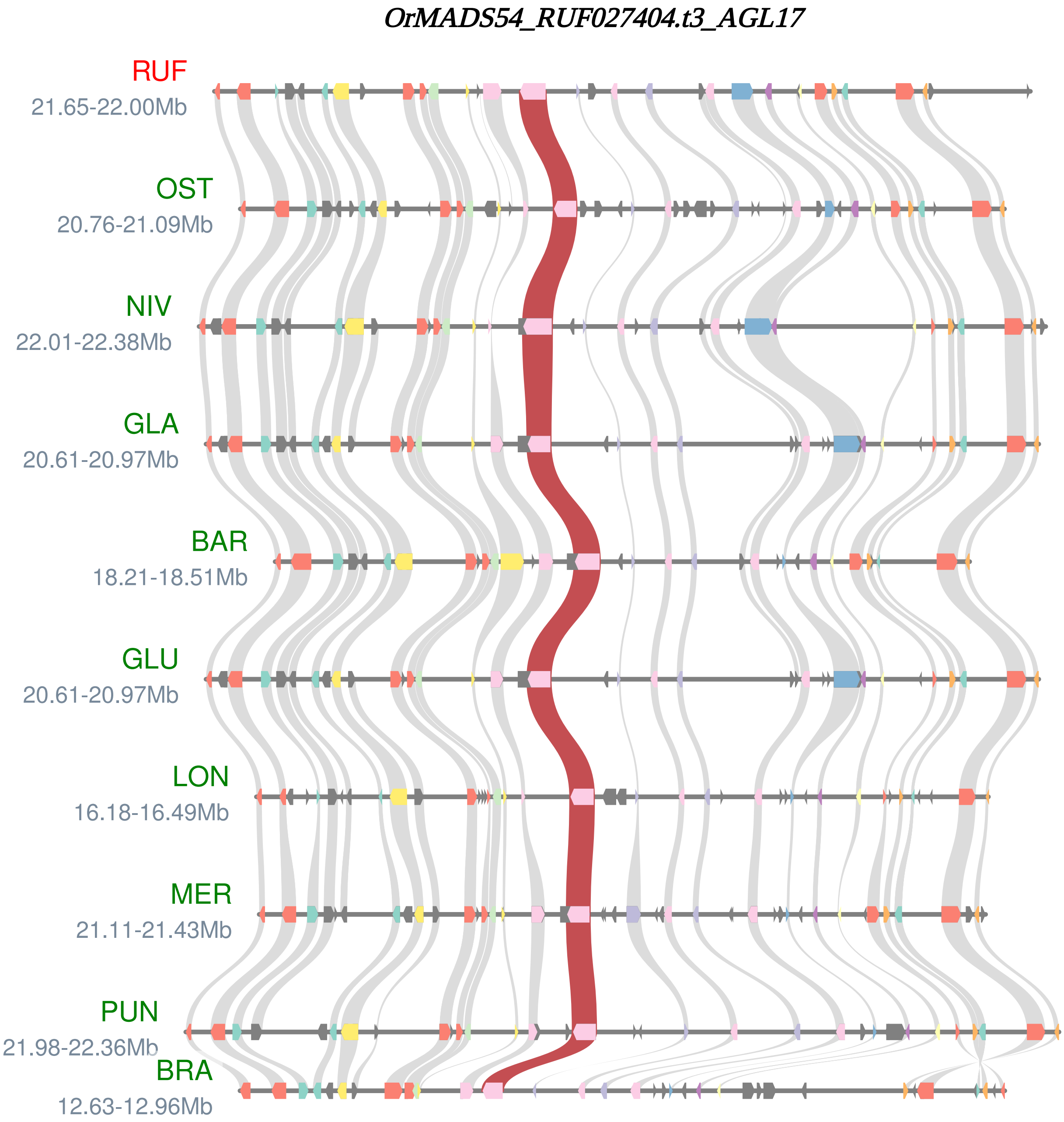
OrMADS54_RUF027404.t3_AGL17
RUF
21.65-22.00Mb
OST
20.76-21.09Mb
NIV
22.01-22.38Mb
GLA
20.61-20.97Mb
BAR
18.21-18.51Mb
GLU
20.61-20.97Mb
LON
16.18-16.49Mb
MER
21.11-21.43Mb
PUN
21.98-22.36Mb
BRA
12.63-12.96Mb

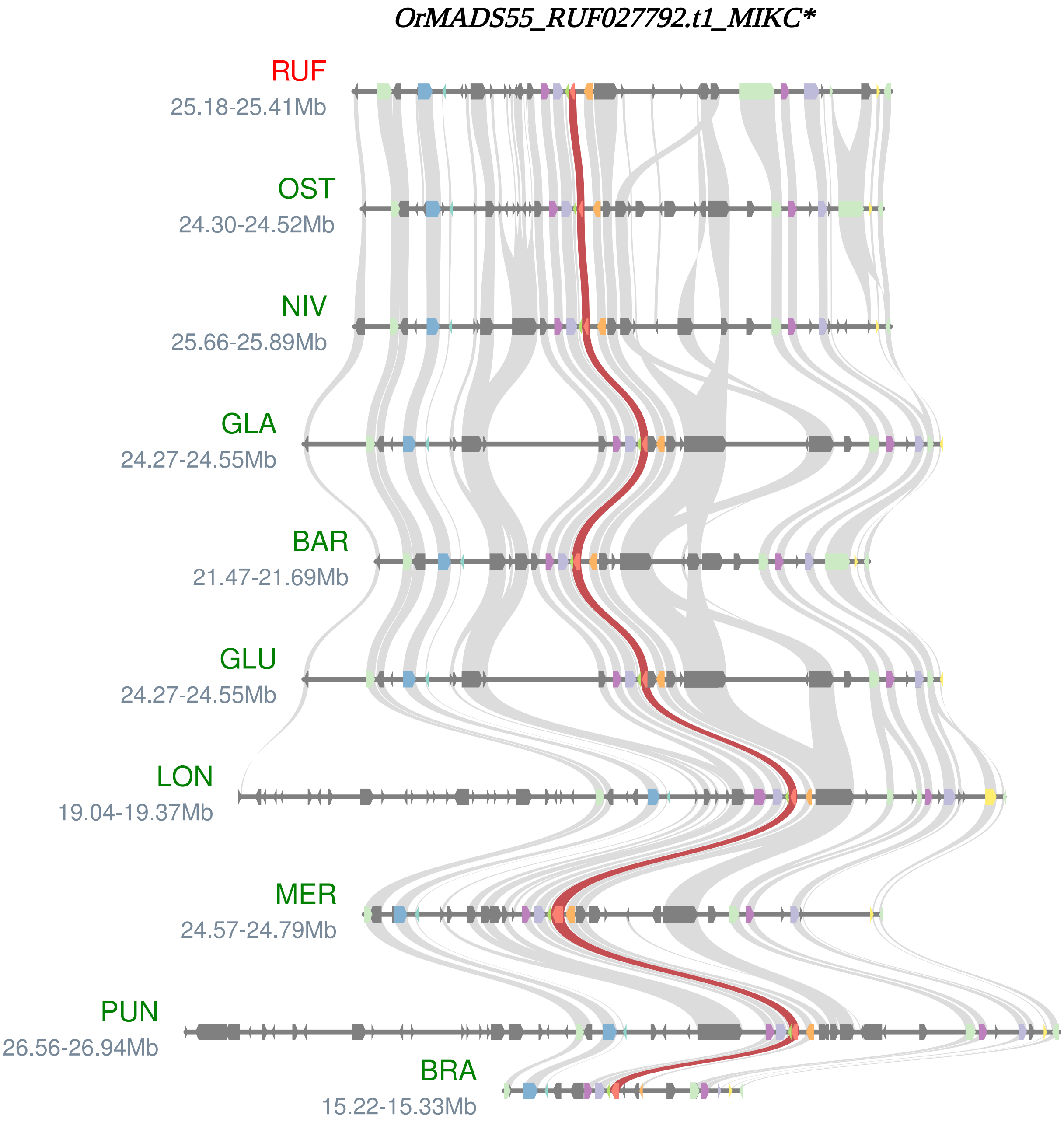
OrMADS55_RUF027792.t1_MIKC*
RUF
25.18-25.41Mb
OST
24.30-24.52Mb
NIV
25.66-25.89Mb
GLA
24.27-24.55Mb
BAR
21.47-21.69Mb
GLU
24.27-24.55Mb
LON
19.04-19.37Mb
MER
24.57-24.79Mb
PUN
26.56-26.94Mb
BRA
15.22-15.33Mb

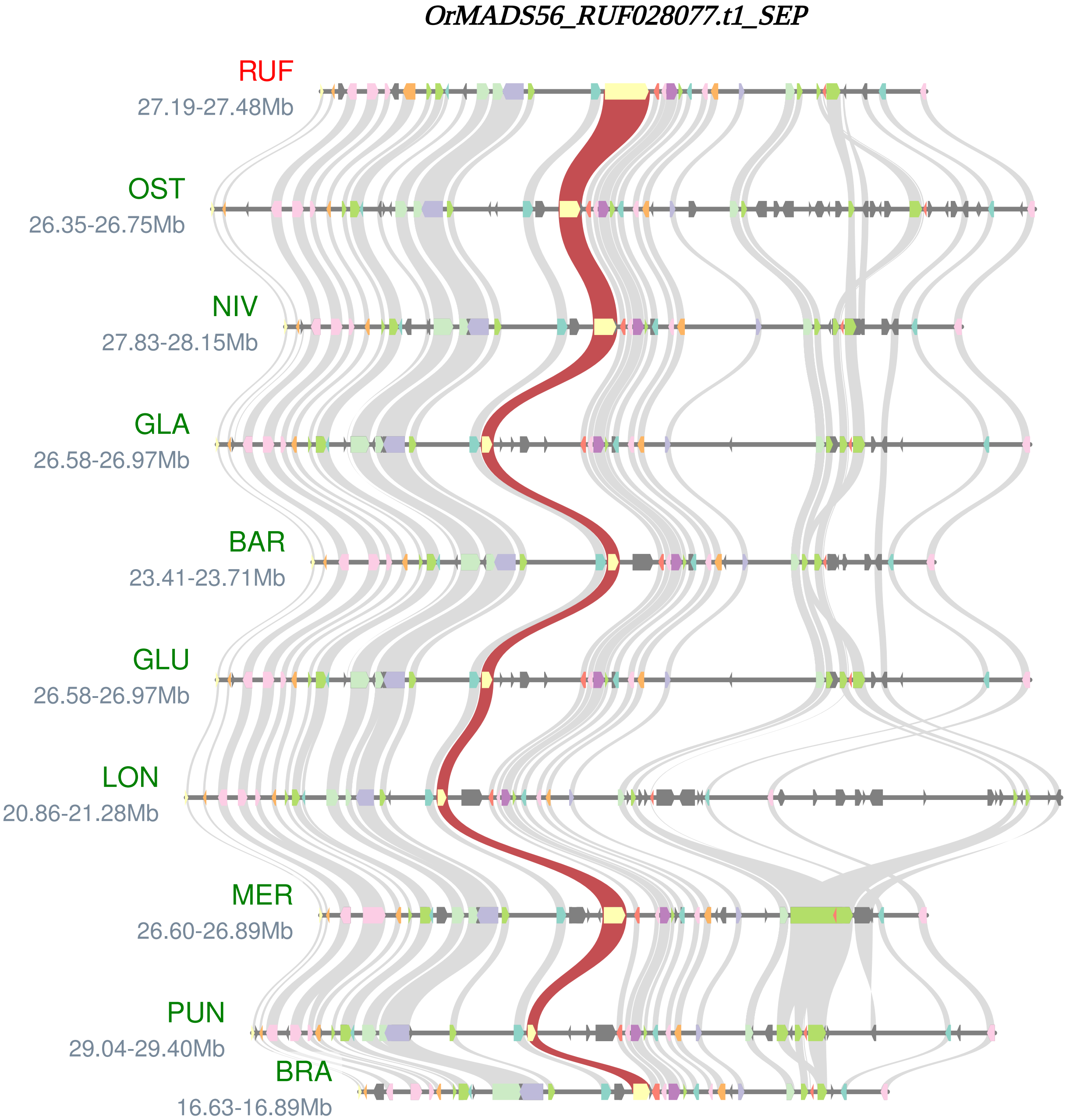

OrMADS56_RUF028077.t1_SEP
RUF
27.19-27.48Mb
OST
26.35-26.75Mb
NIV
27.83-28.15Mb
GLA
26.58-26.97Mb
BAR
23.41-23.71Mb
GLU
26.58-26.97Mb
LON
20.86-21.28Mb
MER
26.60-26.89Mb
PUN
29.04-29.40Mb
BRA
16.63-16.89Mb

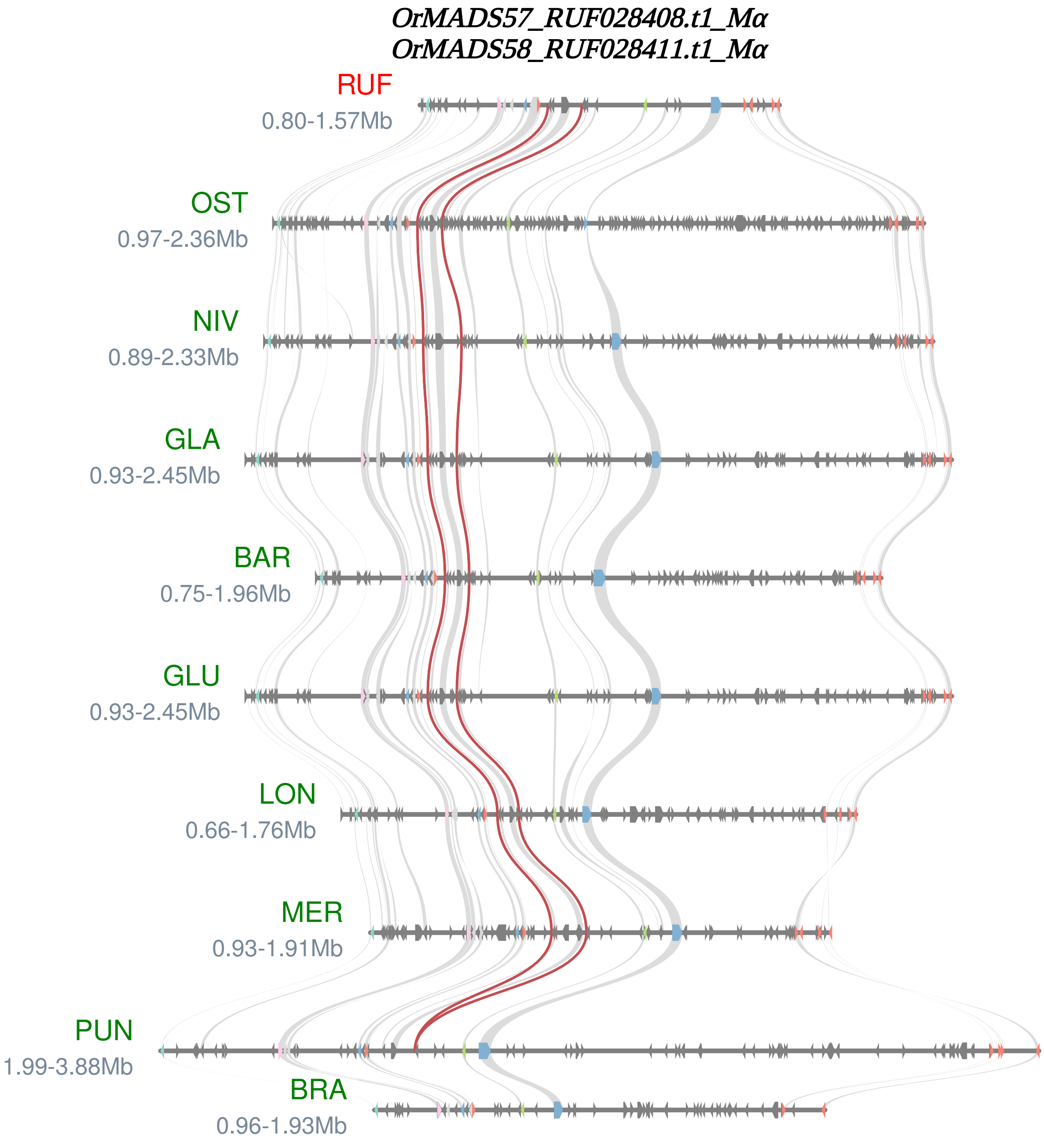

OrMADS57_RUF028408.t1_Mα
OrMADS58_RUF028411.t1_Mα
RUF
0.80-1.57Mb
OST
0.97-2.36Mb
NIV
0.89-2.33Mb
GLA
0.93-2.45Mb
BAR
0.75-1.96Mb
GLU
0.93-2.45Mb
LON
0.66-1.76Mb
MER
0.93-1.91Mb
PUN
1.99-3.88Mb
BRA
0.96-1.93Mb

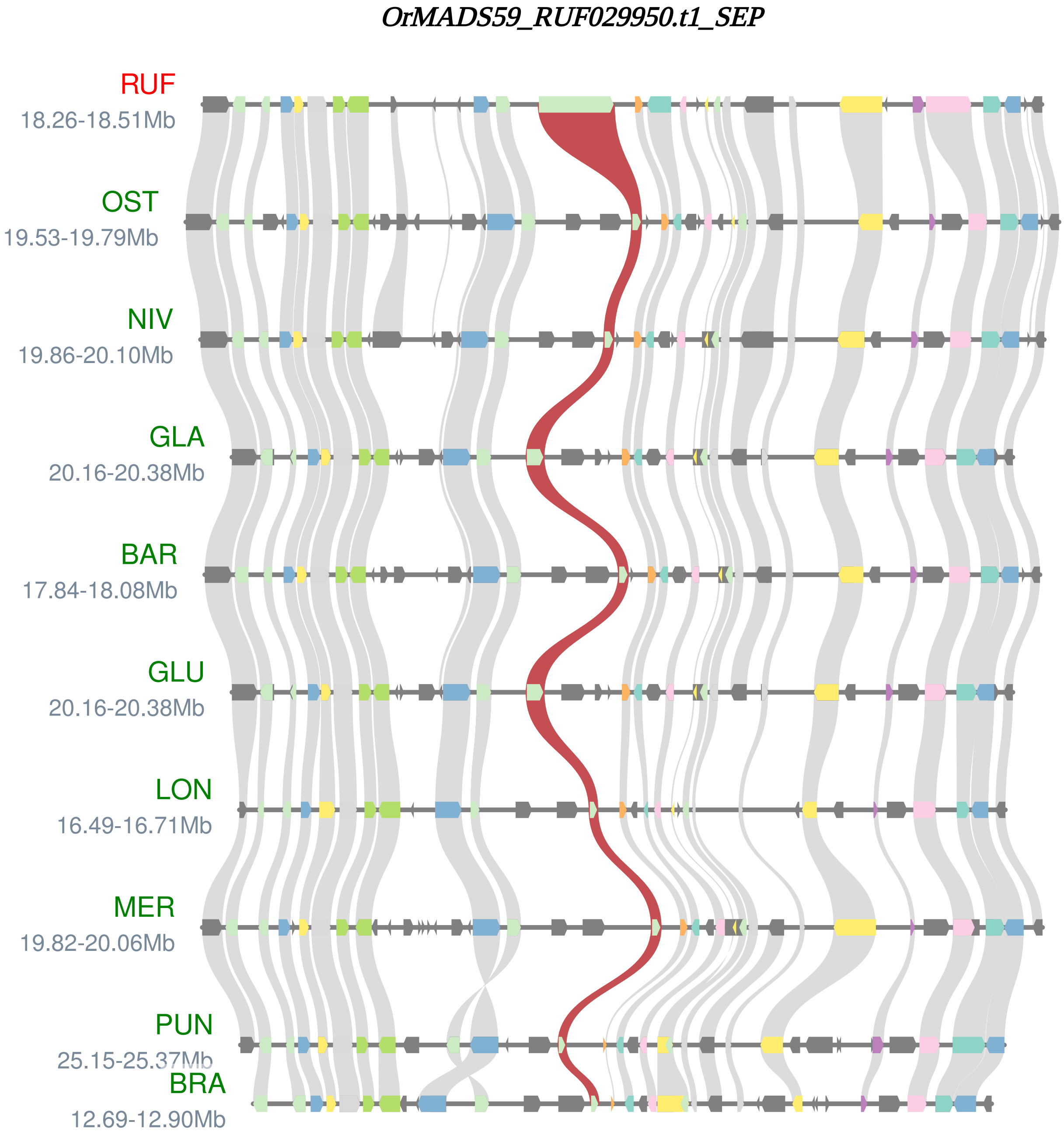

OrMADS59_RUF029950.t1_SEP
RUF
18.26-18.51Mb
OST
19.53-19.79Mb
NIV
19.86-20.10Mb
GLA
20.16-20.38Mb
BAR
17.84-18.08Mb
GLU
20.16-20.38Mb
LON
16.49-16.71Mb
MER
19.82-20.06Mb
PUN
25.15-25.37Mb
BRA
12.69-12.90Mb

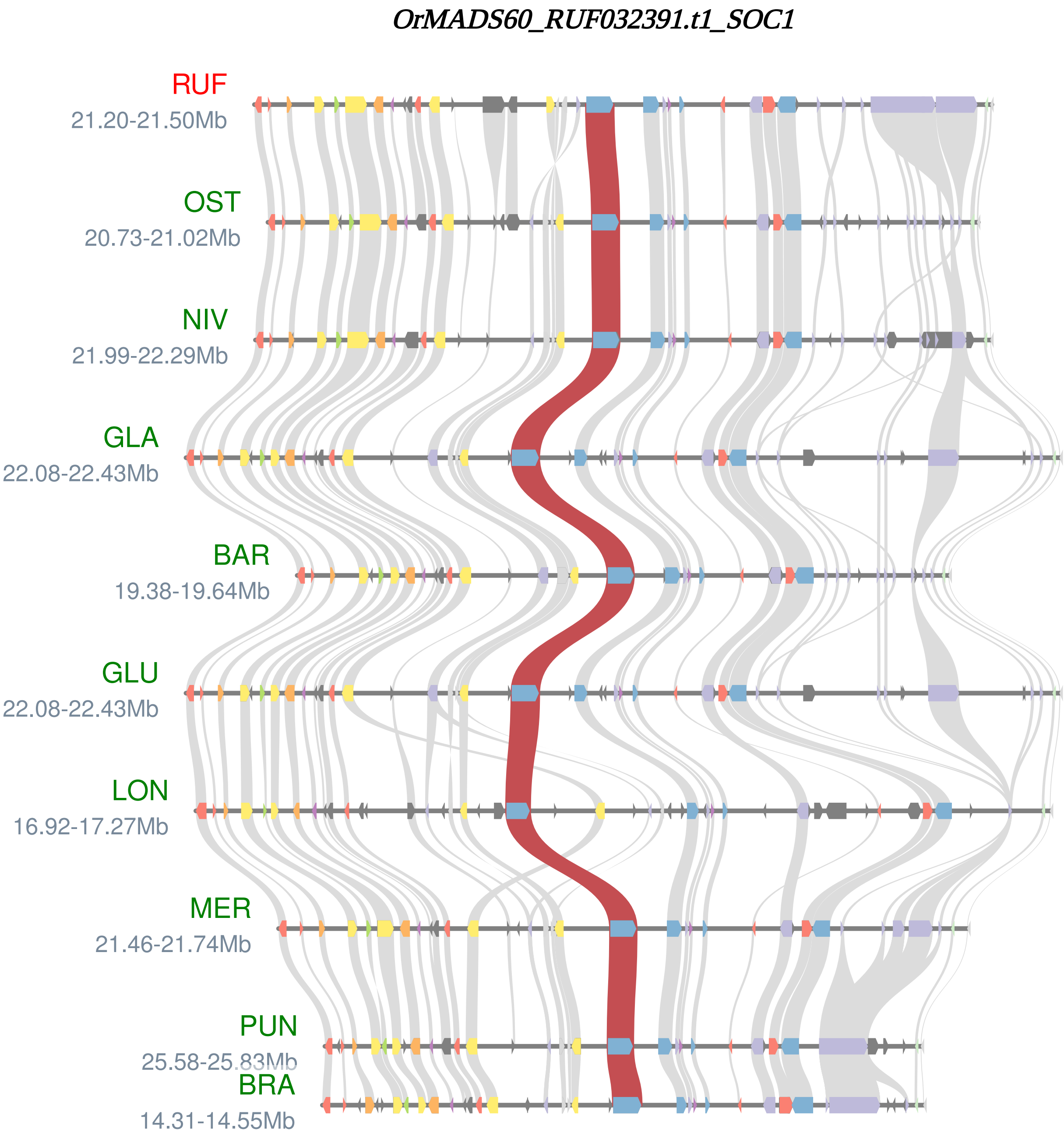

OrMADS60_RUF032391.t1_SOC1
RUF
21.20-21.50Mb
OST
20.73-21.02Mb
NIV
21.99-22.29Mb
GLA
22.08-22.43Mb
BAR
19.38-19.64Mb
GLU
22.08-22.43Mb
LON
16.92-17.27Mb
MER
21.46-21.74Mb
PUN
25.58-25.83Mb
BRA
14.31-14.55Mb

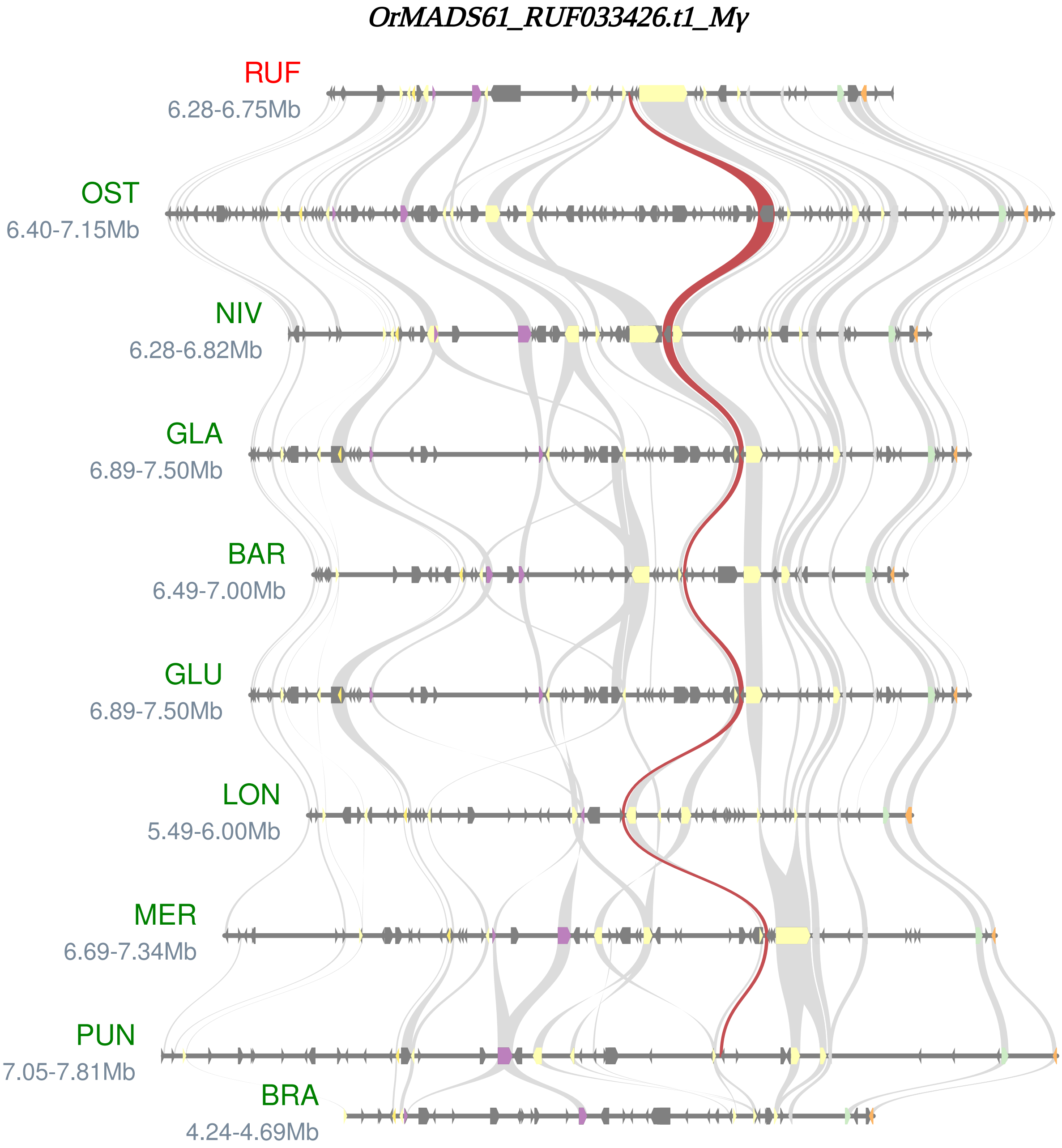
OrMADS61_RUF033426.t1_Mγ
RUF
6.28-6.75Mb
OST
6.40-7.15Mb
NIV
6.28-6.82Mb
GLA
6.89-7.50Mb
BAR
6.49-7.00Mb
GLU
6.89-7.50Mb
LON
5.49-6.00Mb
MER
6.69-7.34Mb
PUN
7.05-7.81Mb
BRA
4.24-4.69Mb

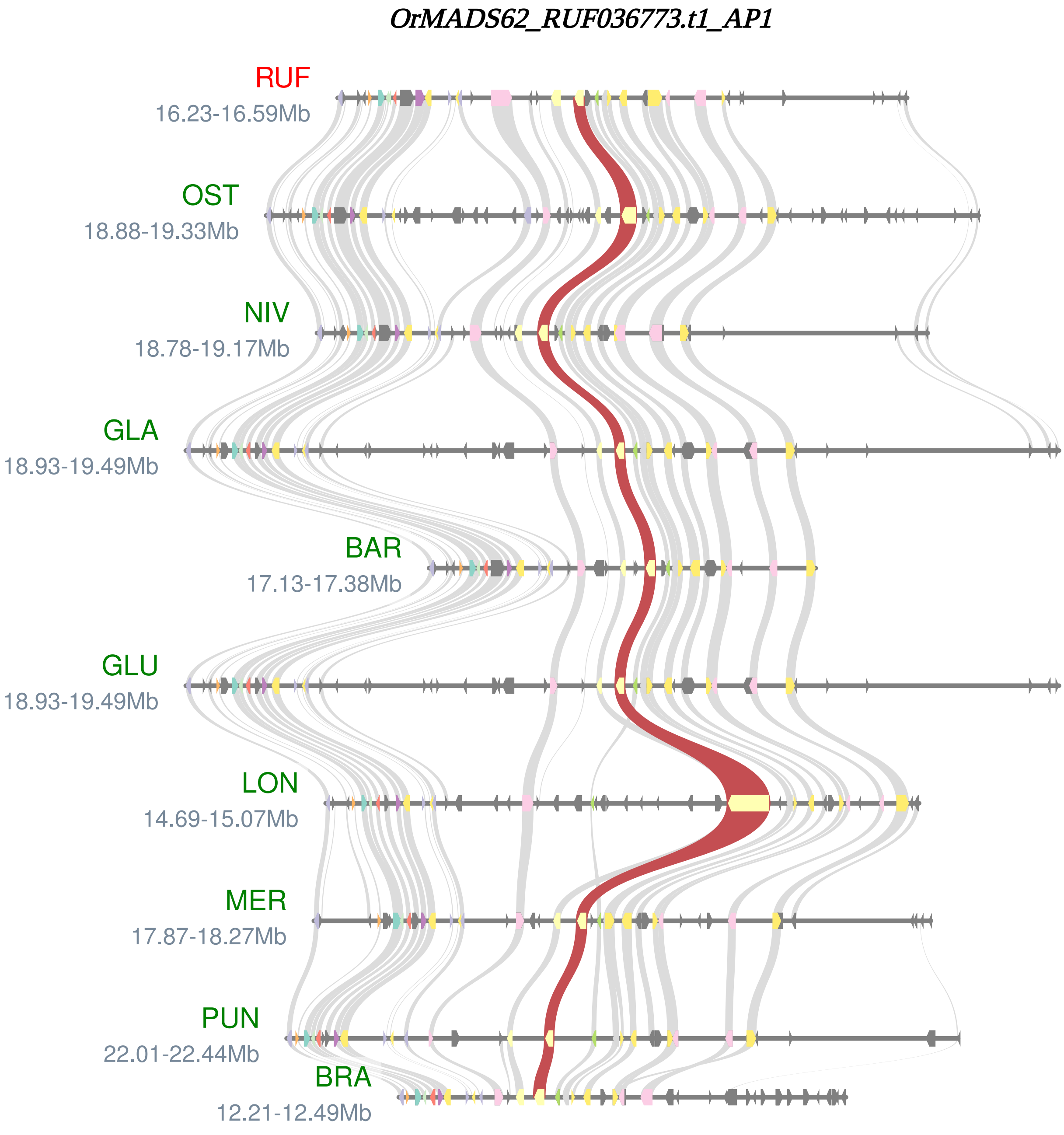

OrMADS62_RUF036773.t1_AP1
RUF
16.23-16.59Mb
OST
18.88-19.33Mb
NIV
18.78-19.17Mb
GLA
18.93-19.49Mb
BAR
17.13-17.38Mb
GLU
18.93-19.49Mb
LON
14.69-15.07Mb
MER
17.87-18.27Mb
PUN
22.01-22.44Mb
BRA
12.21-12.49Mb

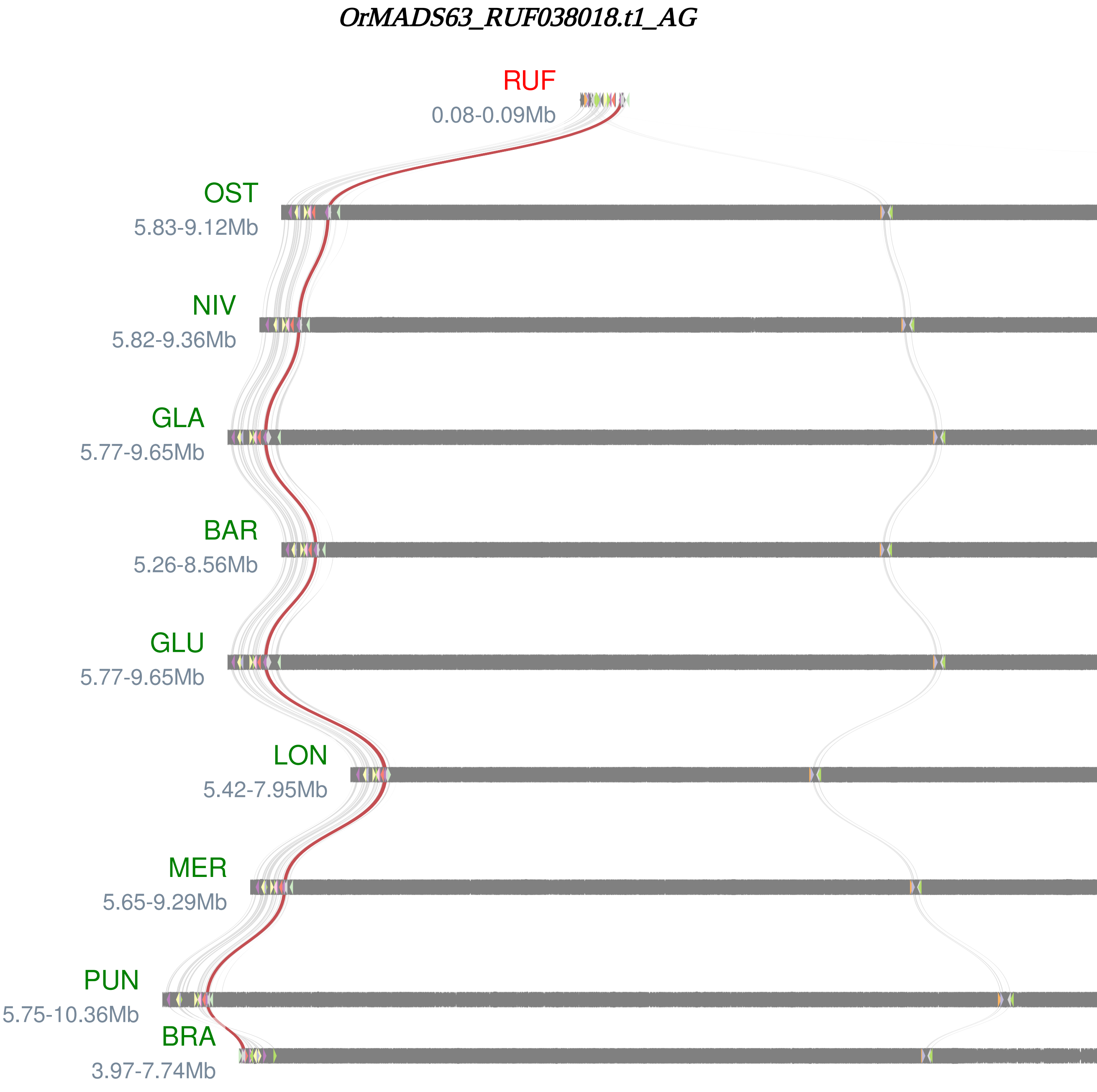
OrMADS63_RUF038018.t1_AG
RUF
0.08-0.09Mb
OST
5.83-9.12Mb
NIV
5.82-9.36Mb
GLA
5.77-9.65Mb
BAR
5.26-8.56Mb
GLU
5.77-9.65Mb
LON
5.42-7.95Mb
MER
5.65-9.29Mb
PUN
5.75-10.36Mb
BRA
3.97-7.74Mb

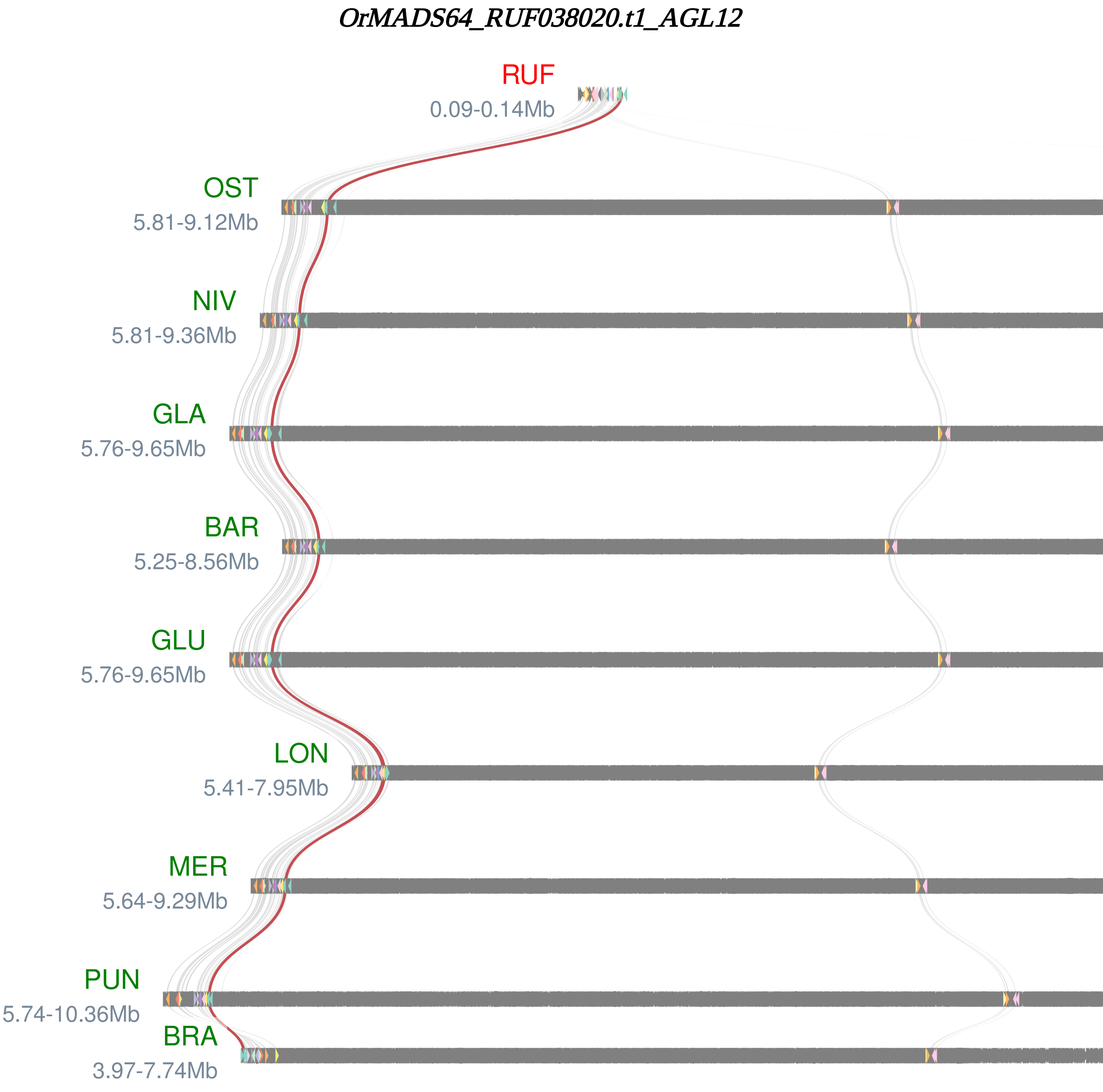
OrMADS64_RUF038020.t1_AGL12
RUF
0.09-0.14Mb
OST
5.81-9.12Mb
NIV
5.81-9.36Mb
GLA
5.76-9.65Mb
BAR
5.25-8.56Mb
GLU
5.76-9.65Mb
LON
5.41-7.95Mb
MER
5.64-9.29Mb
PUN
5.74-10.36Mb
BRA
3.97-7.74Mb

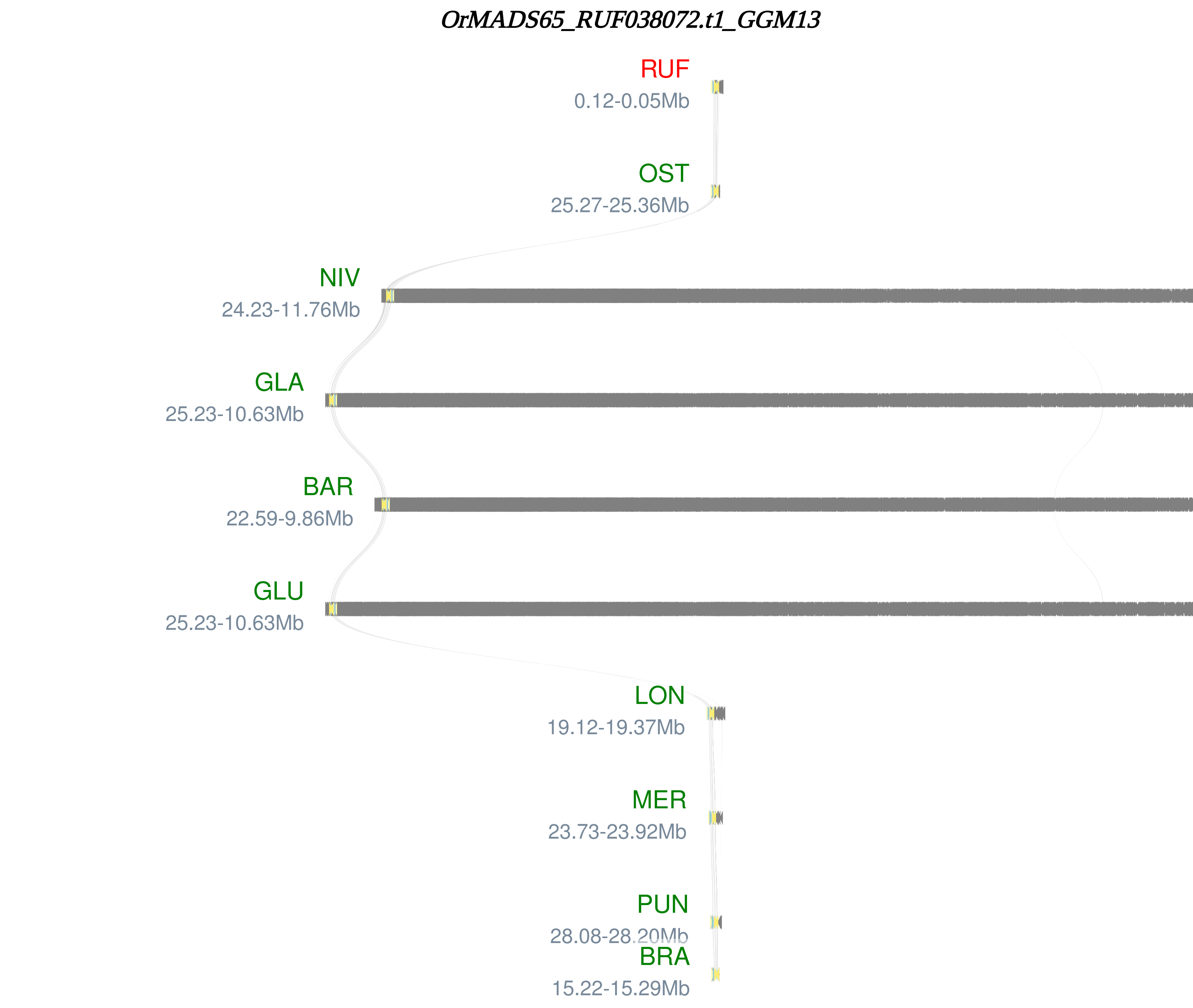
OrMADS65_RUF038072.t1_GGM13
RUF
0.12-0.05Mb
OST
25.27-25.36Mb
NIV
24.23-11.76Mb
GLA
25.23-10.63Mb
BAR
22.59-9.86Mb
GLU
25.23-10.63Mb
LON
19.12-19.37Mb
MER
23.73-23.92Mb
PUN
28.08-28.20Mb
BRA
15.22-15.29Mb

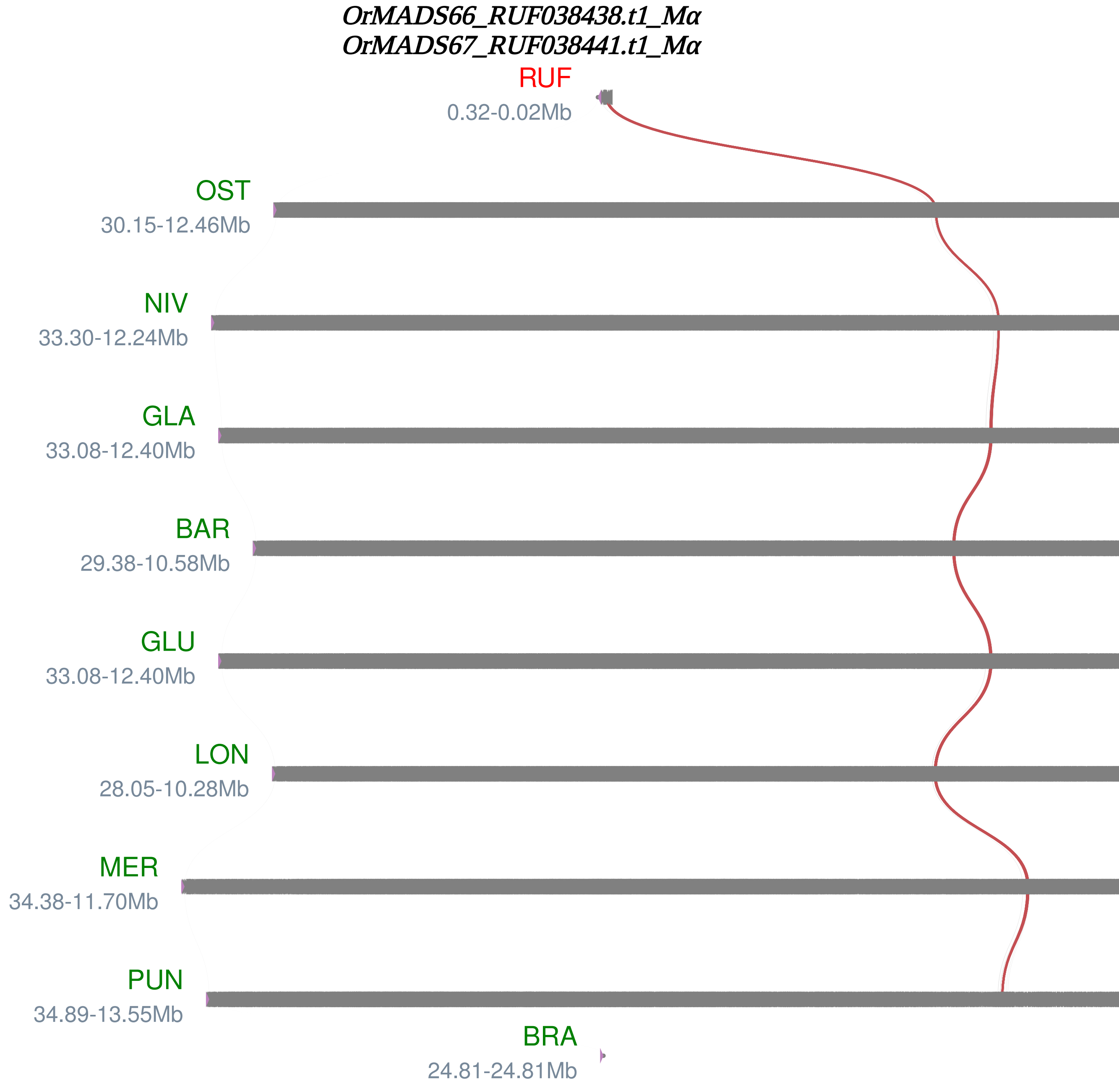
OrMADS66_RUF038438.t1_Mα
OrMADS67_RUF038441.t1_Mα
RUF
0.32-0.02Mb
OST
30.15-12.46Mb
NIV
33.30-12.24Mb
GLA
33.08-12.40Mb
BAR
29.38-10.58Mb
GLU
33.08-12.40Mb
LON
28.05-10.28Mb
MER
34.38-11.70Mb
PUN
34.89-13.55Mb
BRA
24.81-24.81Mb

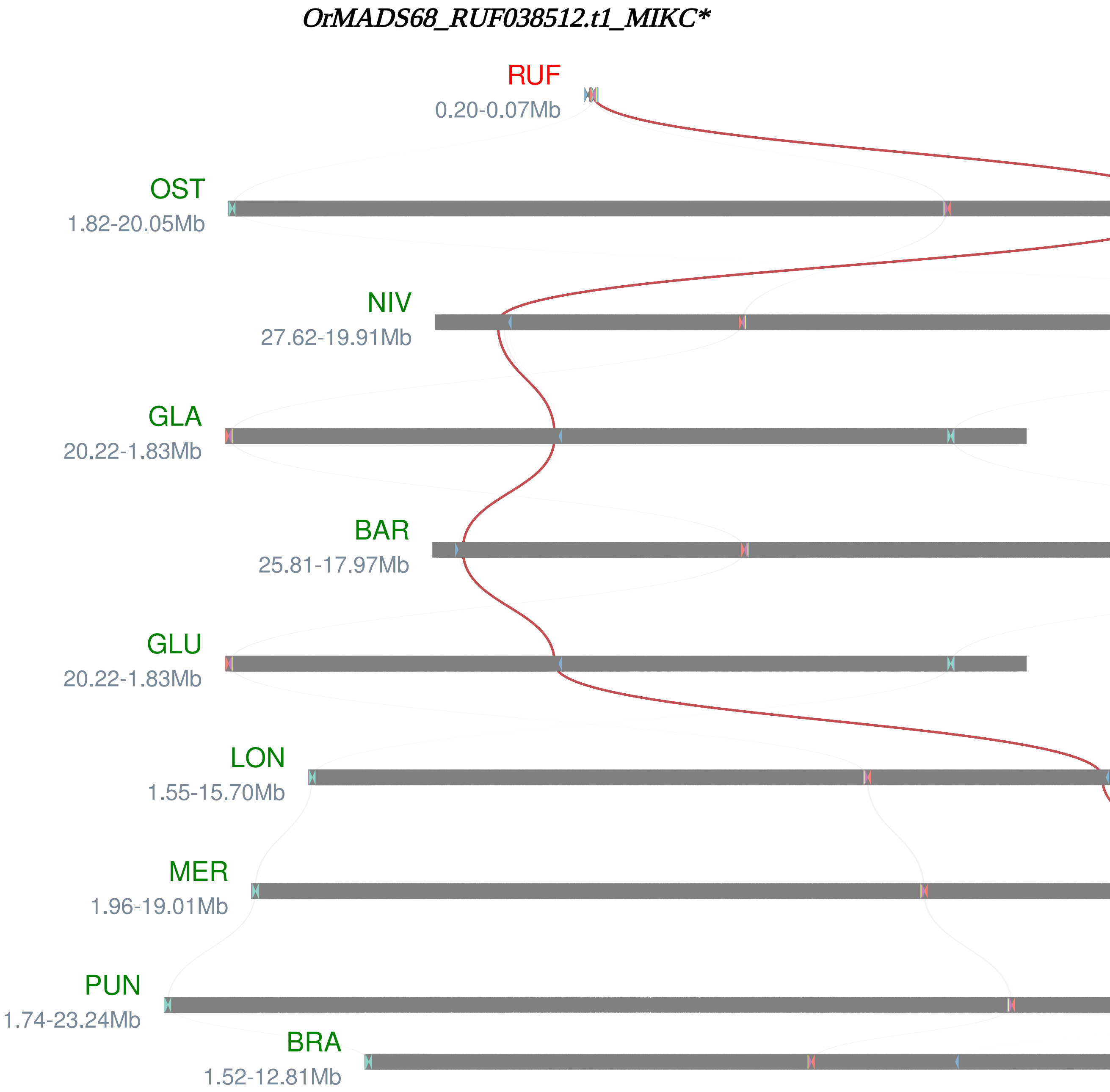
OrMADS68_RUF038512.t1_MIKC*
RUF
0.20-0.07Mb
OST
1.82-20.05Mb
NIV
27.62-19.91Mb
GLA
20.22-1.83Mb
BAR
25.81-17.97Mb
GLU
20.22-1.83Mb
LON
1.55-15.70Mb
MER
1.96-19.01Mb
PUN
1.74-23.24Mb
BRA
1.52-12.81Mb

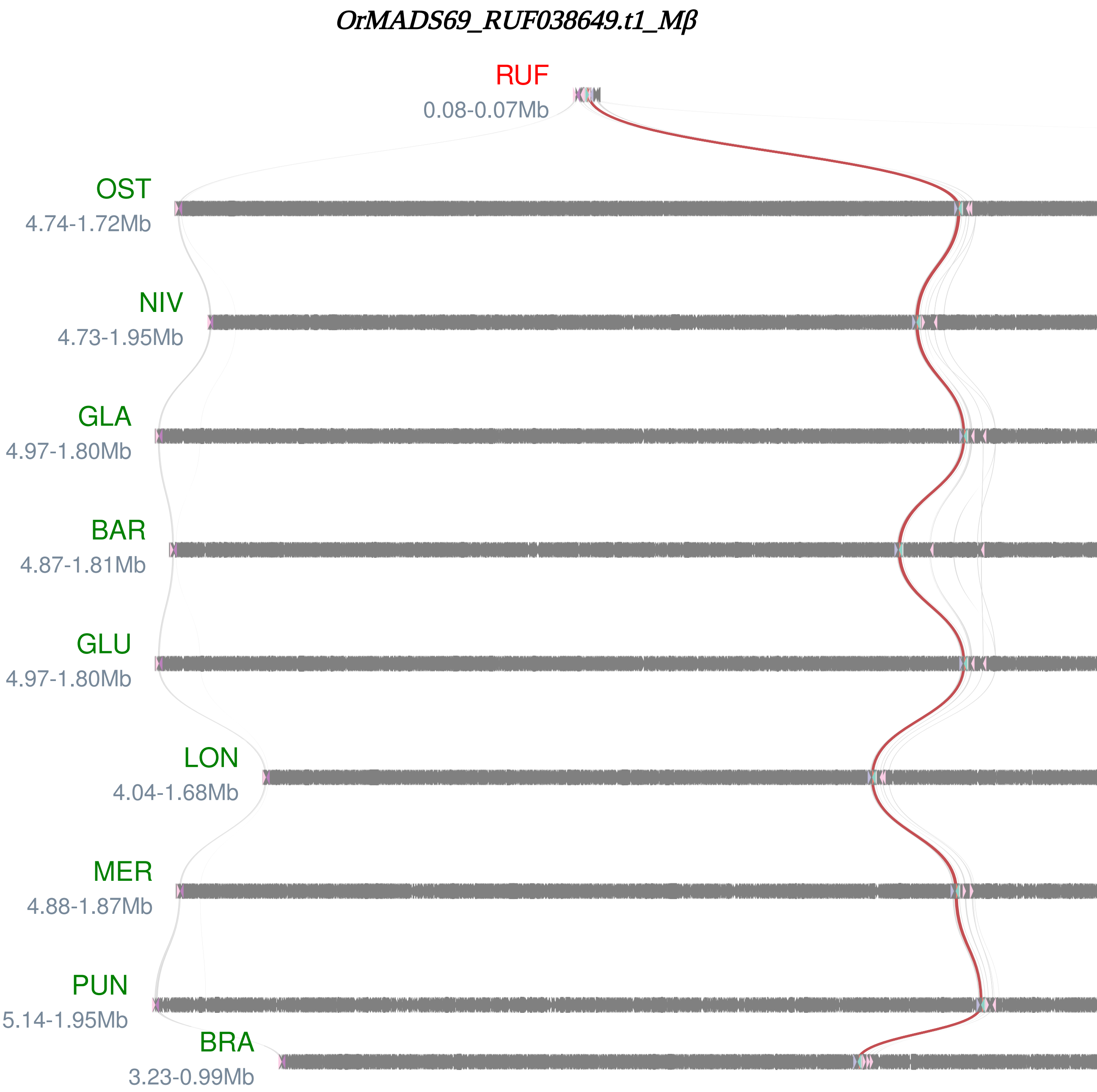
OrMADS69_RUF038649.t1_Mβ
RUF
0.08-0.07Mb
OST
4.74-1.72Mb
NIV
4.73-1.95Mb
GLA
4.97-1.80Mb
BAR
4.87-1.81Mb
GLU
4.97-1.80Mb
LON
4.04-1.68Mb
MER
4.88-1.87Mb
PUN
5.14-1.95Mb
BRA
3.23-0.99Mb

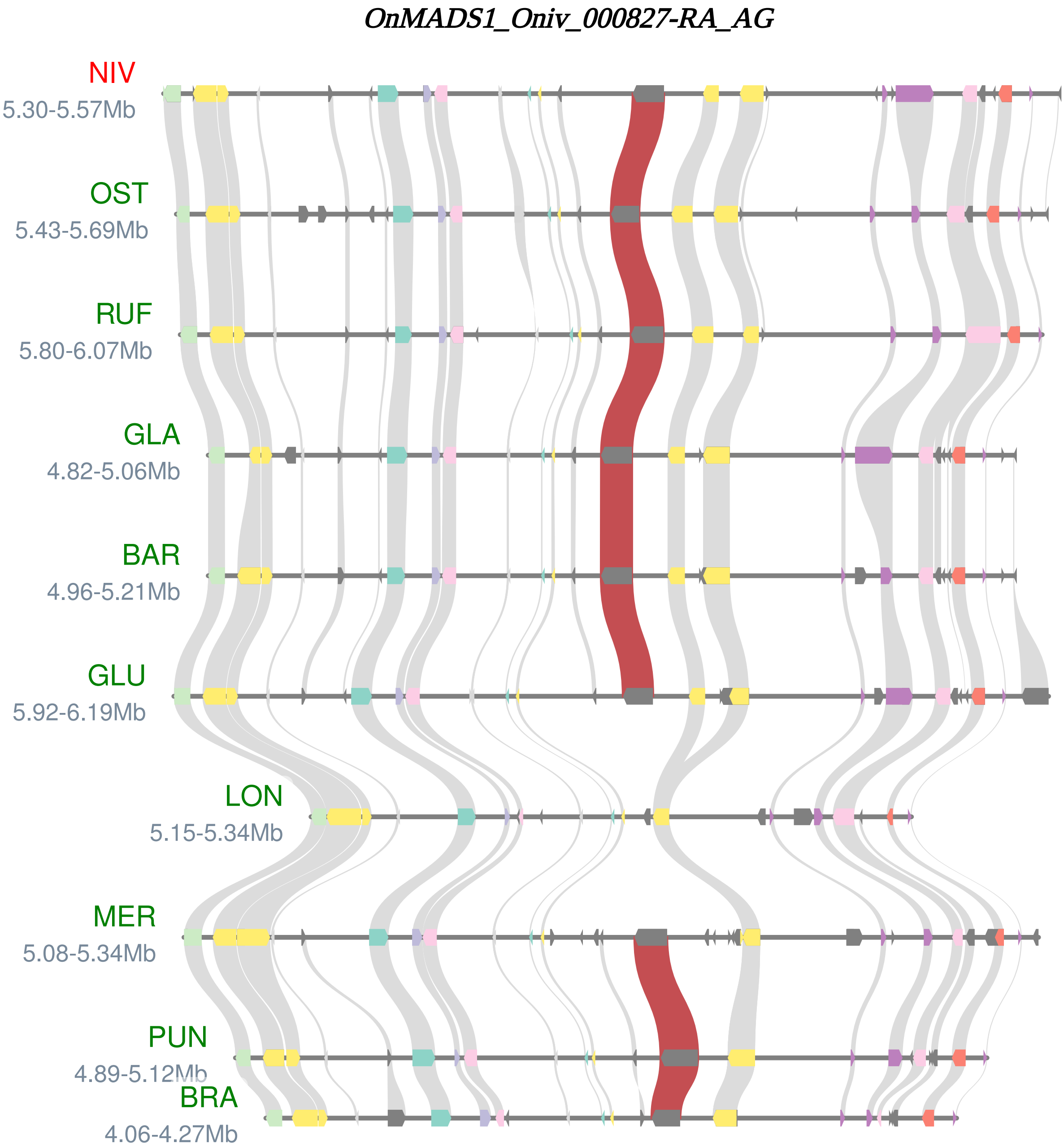
OnMADS1_Oniv_000827-RA_AG
NIV
5.30-5.57Mb
OST
5.43-5.69Mb
RUF
5.80-6.07Mb
GLA
4.82-5.06Mb
BAR
4.96-5.21Mb
GLU
5.92-6.19Mb
LON
5.15-5.34Mb
MER
5.08-5.34Mb
PUN
4.89-5.12Mb
BRA
4.06-4.27Mb

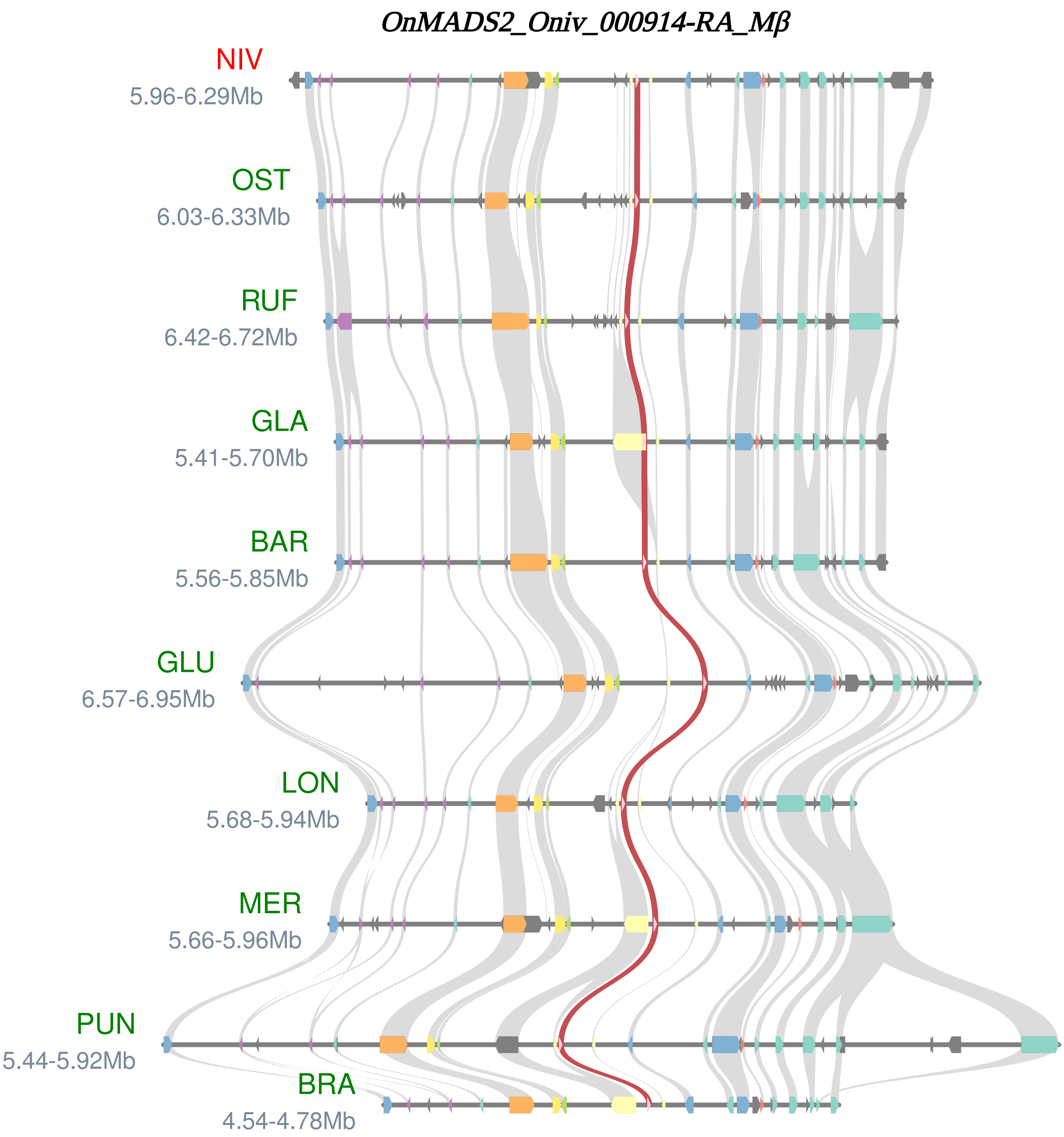

OnMADS2_Oniv_000914-RA_Mβ
NIV
5.96-6.29Mb
OST
6.03-6.33Mb
RUF
6.42-6.72Mb
GLA
5.41-5.70Mb
BAR
5.56-5.85Mb
GLU
6.57-6.95Mb
LON
5.68-5.94Mb
MER
5.66-5.96Mb
PUN
5.44-5.92Mb
BRA
4.54-4.78Mb

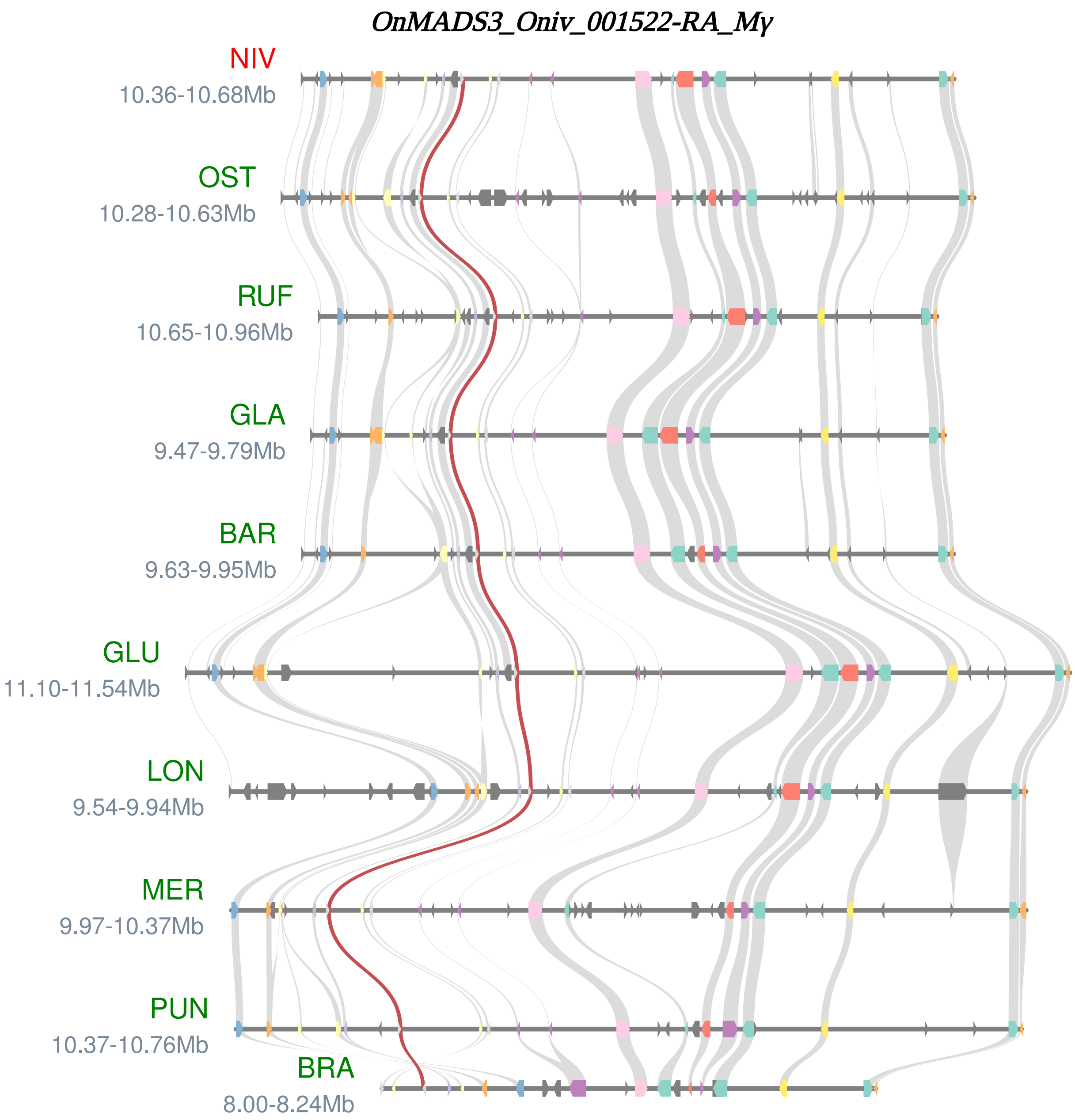
OnMADS3_Oniv_001522-RA_Mγ
NIV
10.36-10.68Mb
OST
10.28-10.63Mb
RUF
10.65-10.96Mb
GLA
9.47-9.79Mb
BAR
9.63-9.95Mb
GLU
11.10-11.54Mb
LON
9.54-9.94Mb
MER
9.97-10.37Mb
PUN
10.37-10.76Mb
BRA
8.00-8.24Mb

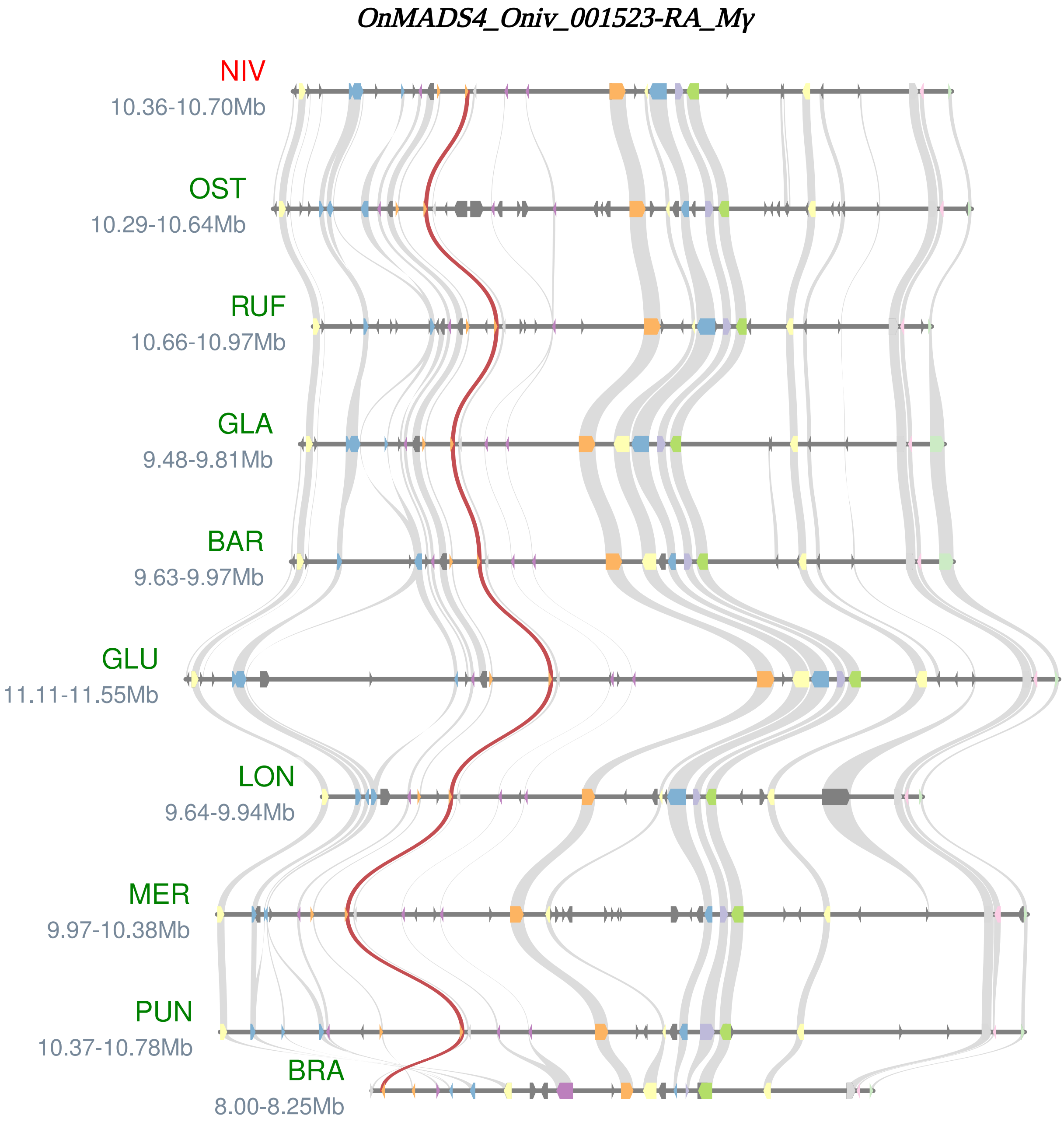
OnMADS4_Oniv_001523-RA_Mγ
NIV
10.36-10.70Mb
OST
10.29-10.64Mb
RUF
10.66-10.97Mb
GLA
9.48-9.81Mb
BAR
9.63-9.97Mb
GLU
11.11-11.55Mb
LON
9.64-9.94Mb
MER
9.97-10.38Mb
PUN
10.37-10.78Mb
BRA
8.00-8.25Mb

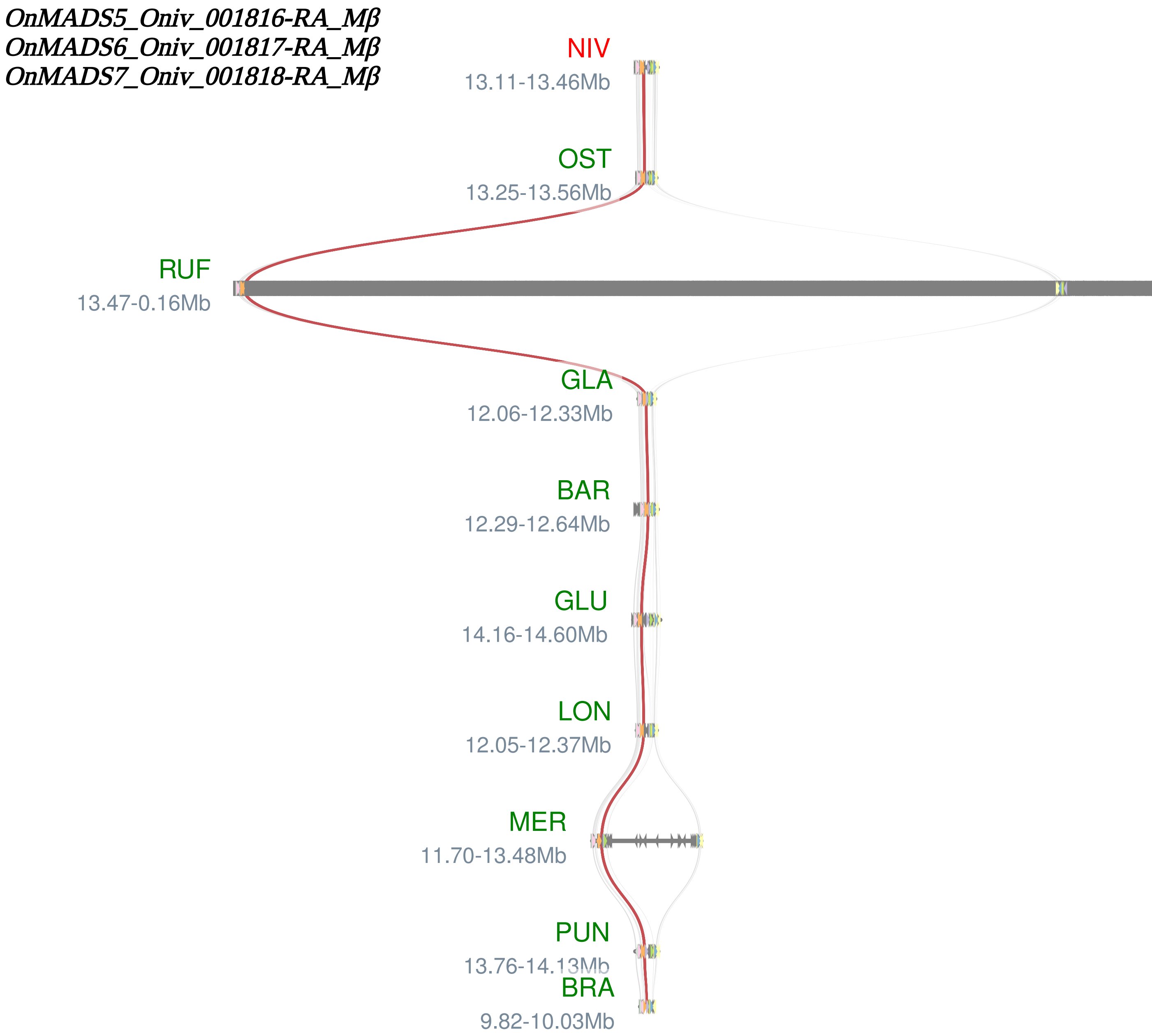

OnMADS5_Oniv_001816-RA_Mβ
OnMADS6_Oniv_001817-RA_Mβ
OnMADS7_Oniv_001818-RA_Mβ
NIV
13.11-13.46Mb
OST
13.25-13.56Mb
RUF
13.47-0.16Mb
GLA
12.06-12.33Mb
BAR
12.29-12.64Mb
GLU
14.16-14.60Mb
LON
12.05-12.37Mb
MER
11.70-13.48Mb
PUN
13.76-14.13Mb
BRA
9.82-10.03Mb

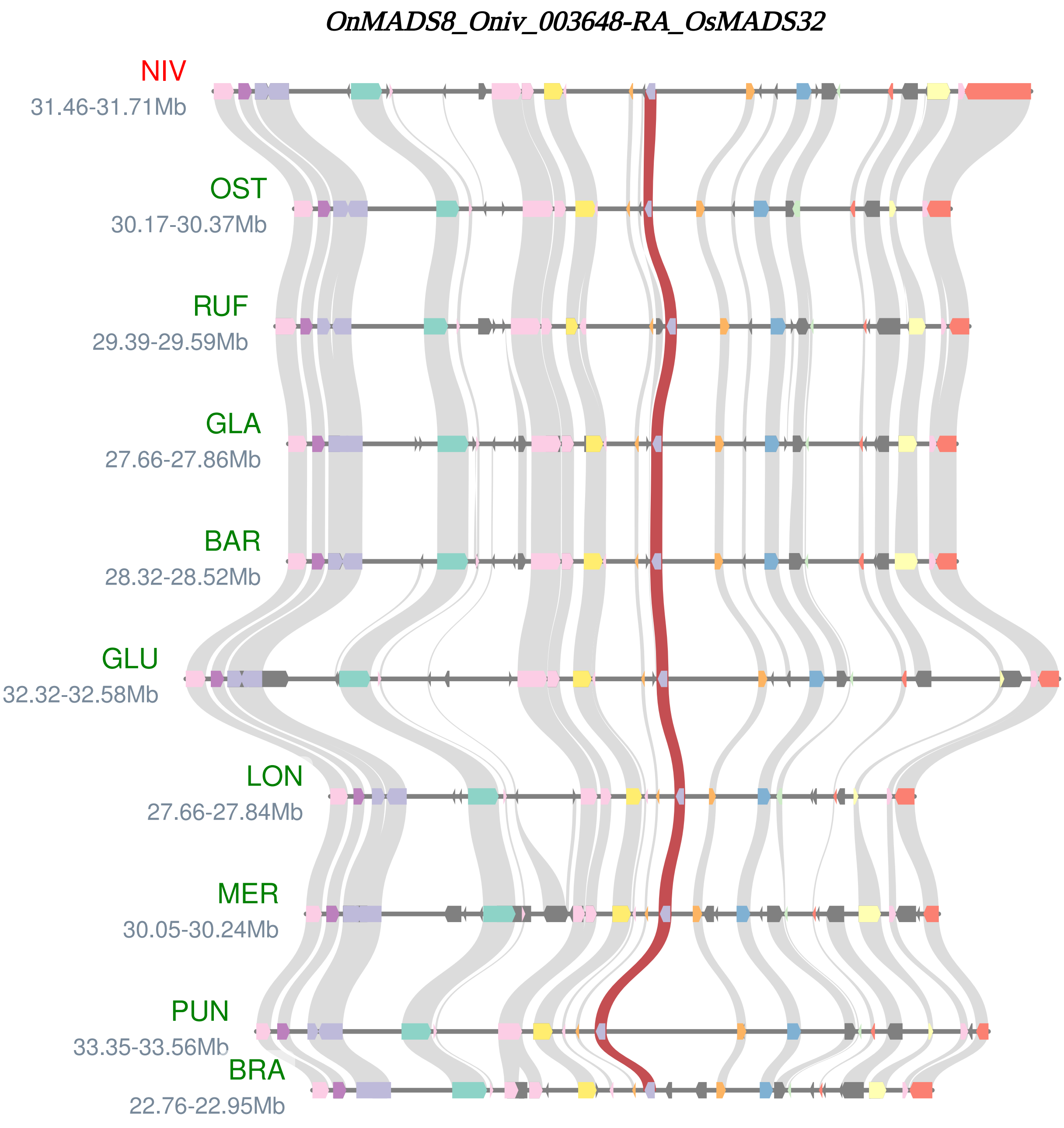
OnMADS8_Oniv_003648-RA_OsMADS32
NIV
31.46-31.71Mb
OST
30.17-30.37Mb
RUF
29.39-29.59Mb
GLA
27.66-27.86Mb
BAR
28.32-28.52Mb
GLU
32.32-32.58Mb
LON
27.66-27.84Mb
MER
30.05-30.24Mb
PUN
33.35-33.56Mb
BRA
22.76-22.95Mb

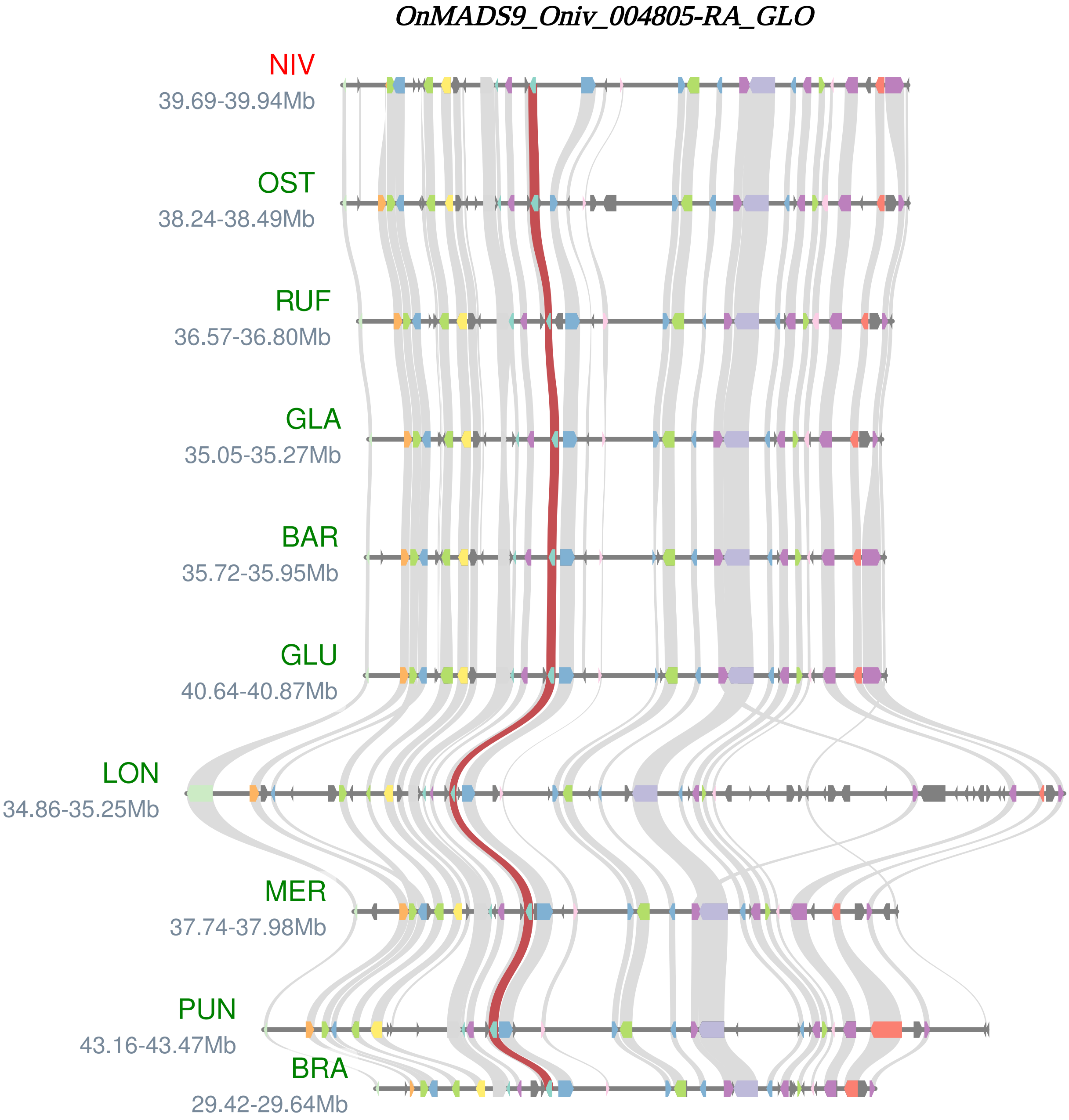
OnMADS9_Oniv_004805-RA_GLO
NIV
39.69-39.94Mb
OST
38.24-38.49Mb
RUF
36.57-36.80Mb
GLA
35.05-35.27Mb
BAR
35.72-35.95Mb
GLU
40.64-40.87Mb
LON
34.86-35.25Mb
MER
37.74-37.98Mb
PUN
43.16-43.47Mb
BRA
29.42-29.64Mb

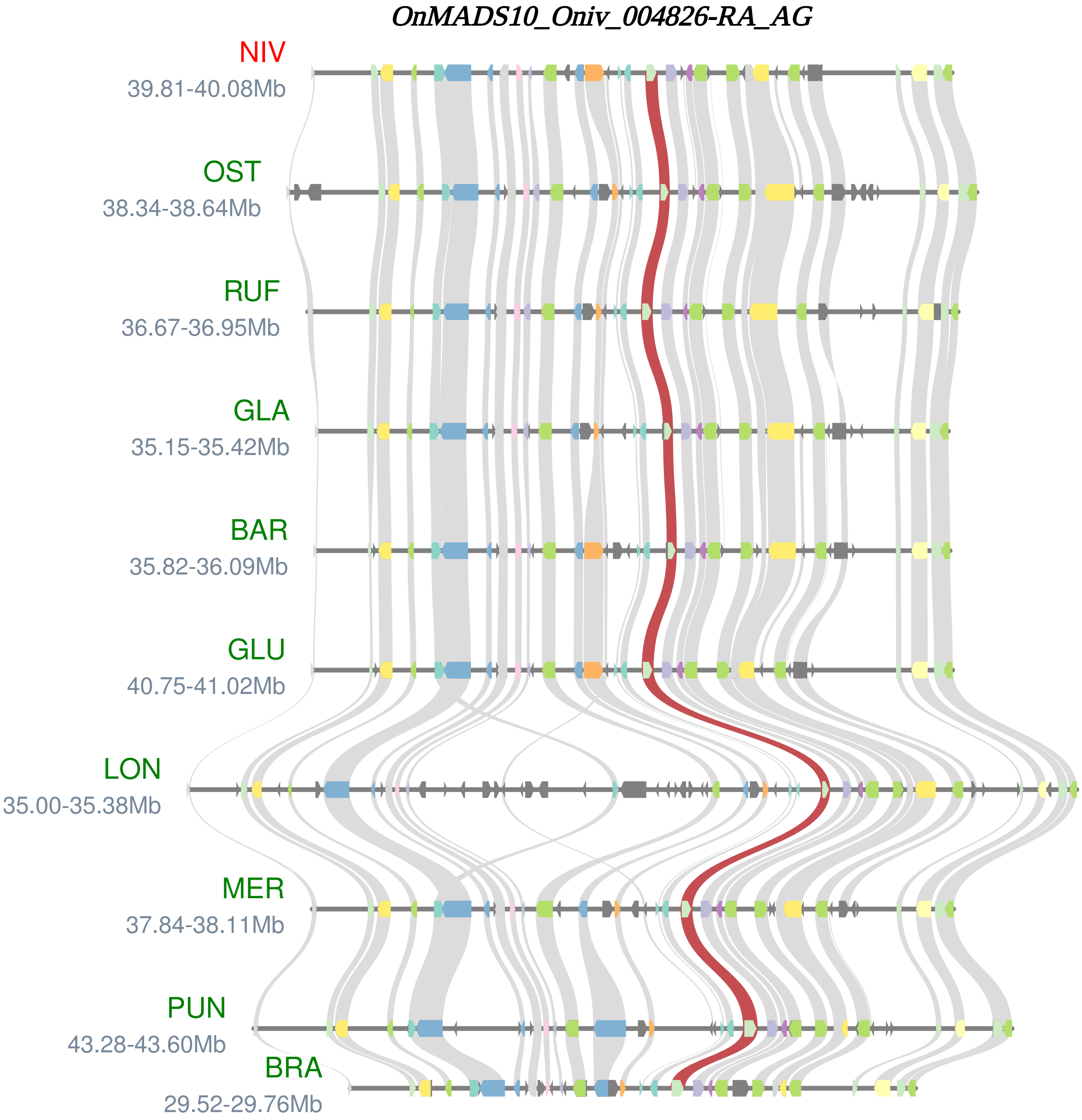
OnMADS10_Oniv_004826-RA_AG
NIV
39.81-40.08Mb
OST
38.34-38.64Mb
RUF
36.67-36.95Mb
GLA
35.15-35.42Mb
BAR
35.82-36.09Mb
GLU
40.75-41.02Mb
LON
35.00-35.38Mb
MER
37.84-38.11Mb
PUN
43.28-43.60Mb
BRA
29.52-29.76Mb

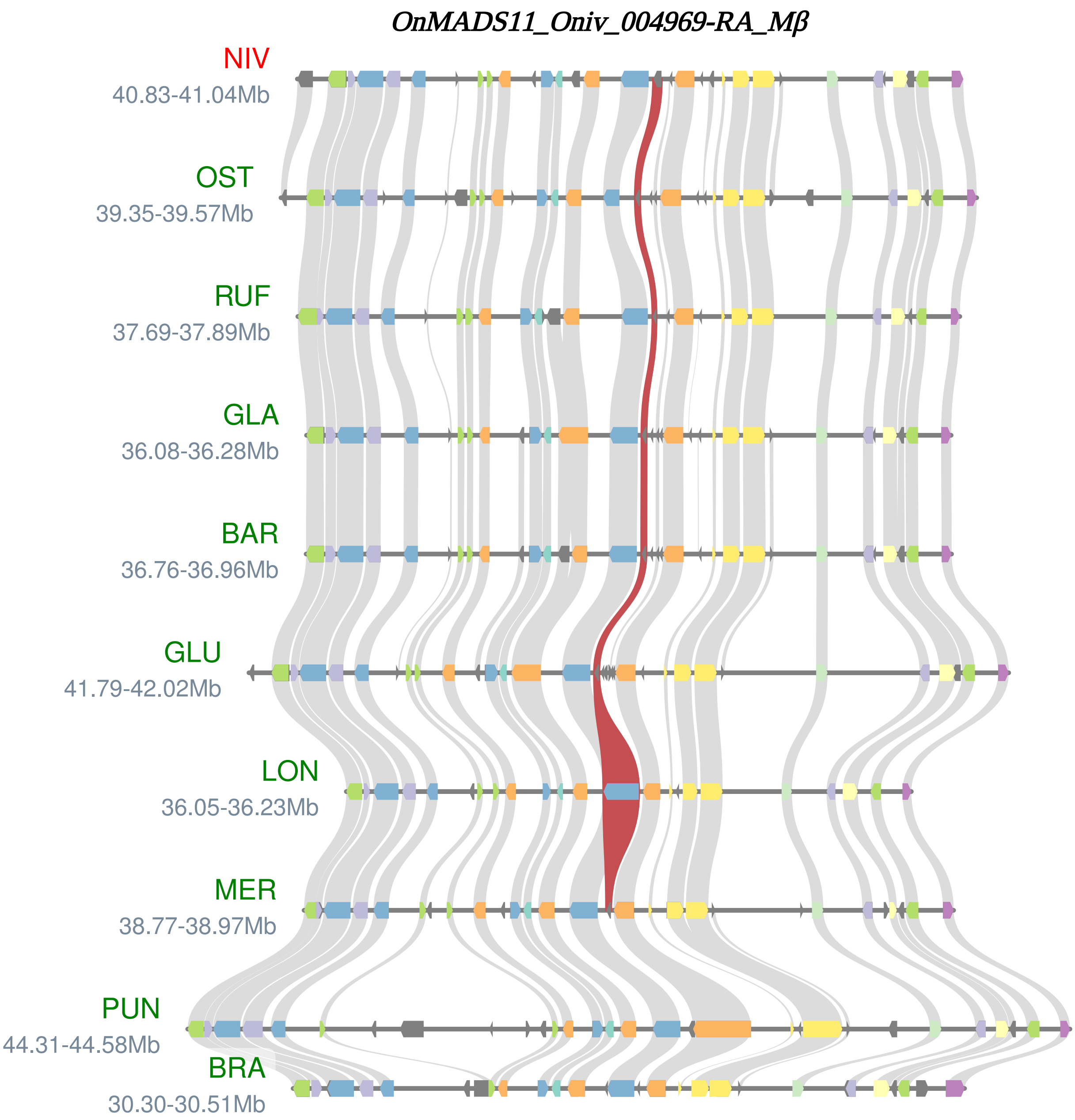
OnMADS11_Oniv_004969-RA_Mβ
NIV
40.83-41.04Mb
OST
39.35-39.57Mb
RUF
37.69-37.89Mb
GLA
36.08-36.28Mb
BAR
36.76-36.96Mb
GLU
41.79-42.02Mb
LON
36.05-36.23Mb
MER
38.77-38.97Mb
PUN
44.31-44.58Mb
BRA
30.30-30.51Mb

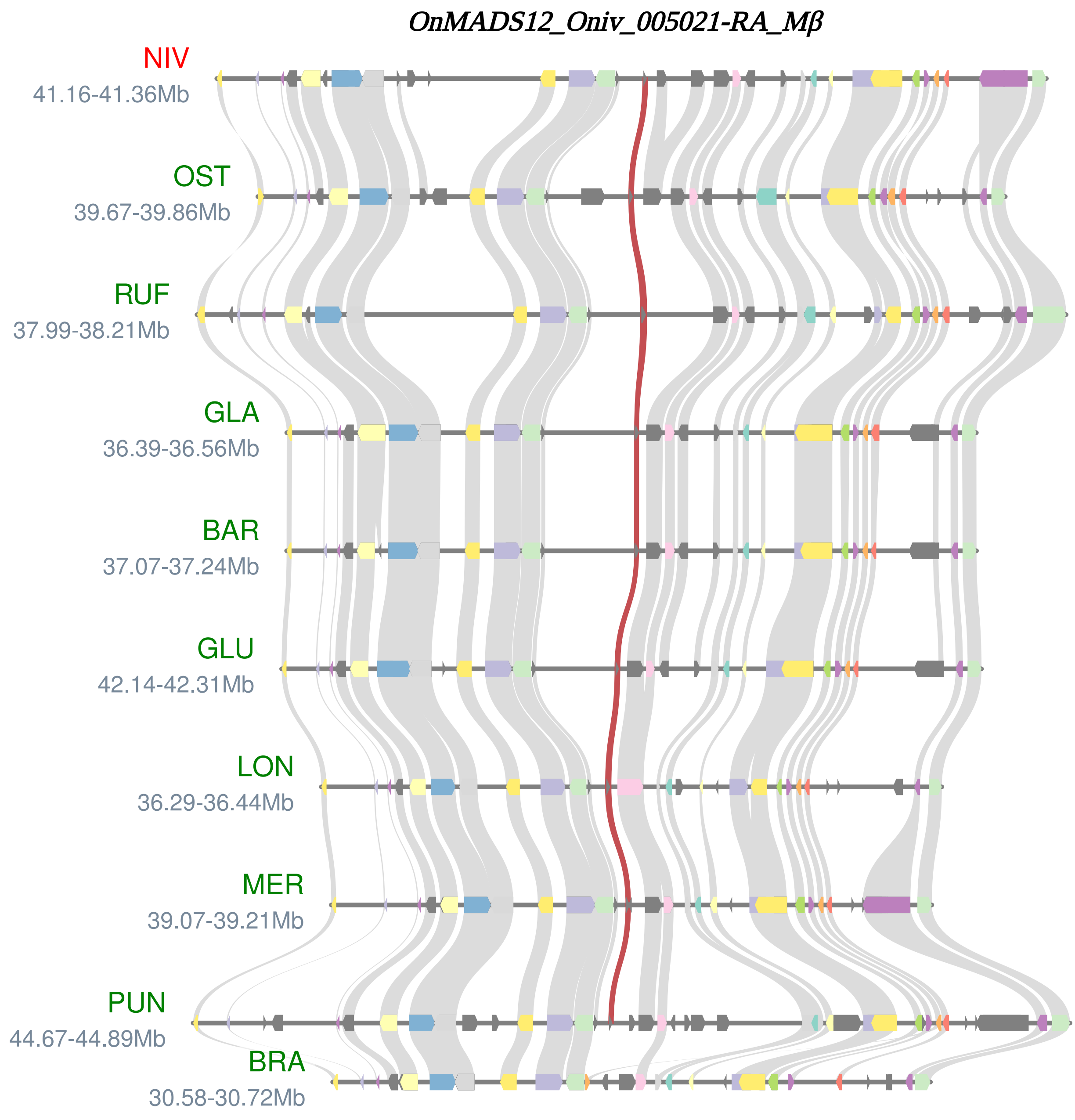

OnMADS12_Oniv_005021-RA_Mβ
NIV
41.16-41.36Mb
OST
39.67-39.86Mb
RUF
37.99-38.21Mb
GLA
36.39-36.56Mb
BAR
37.07-37.24Mb
GLU
42.14-42.31Mb
LON
36.29-36.44Mb
MER
39.07-39.21Mb
PUN
44.67-44.89Mb
BRA
30.58-30.72Mb

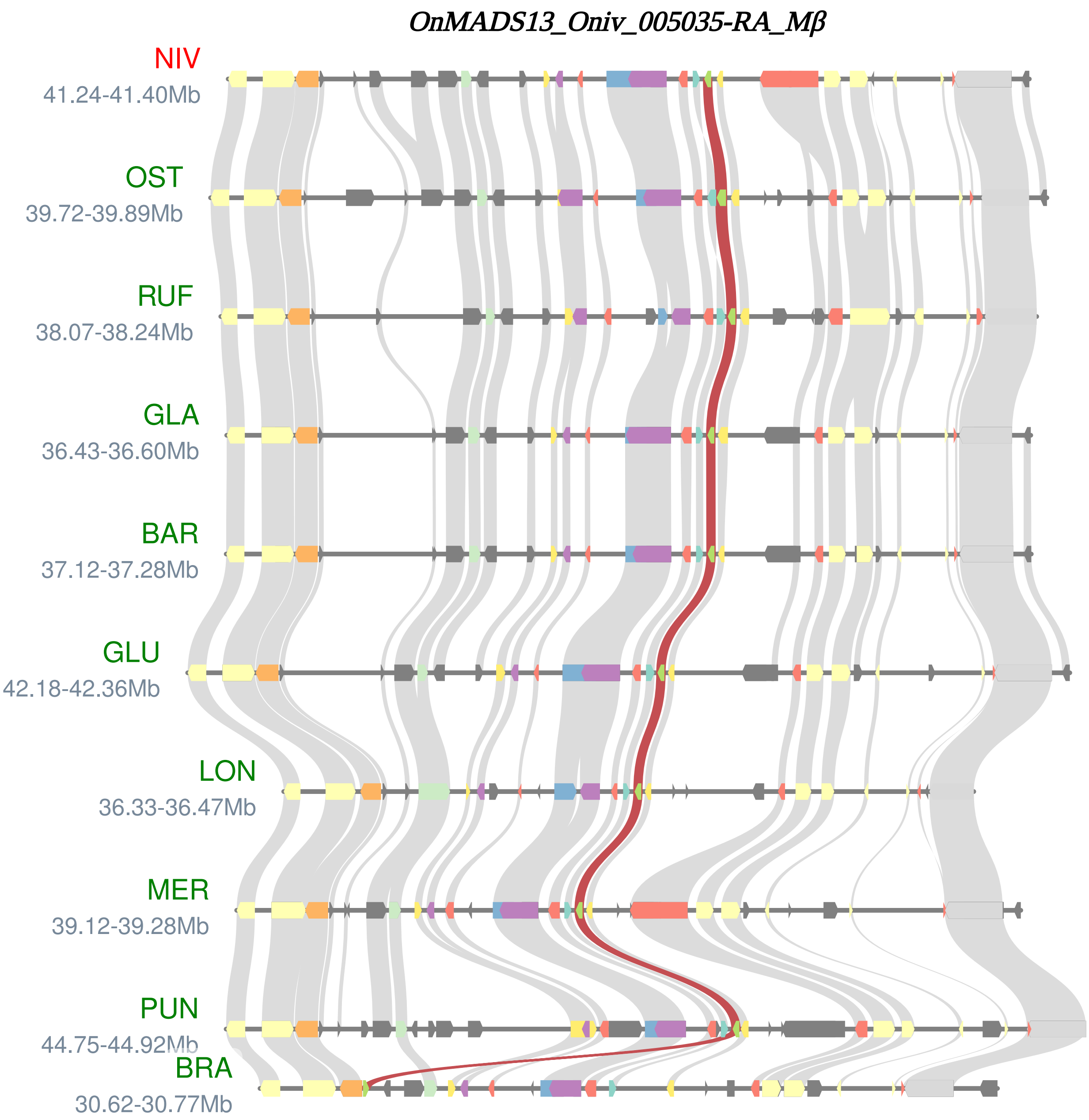

OnMADS13_Oniv_005035-RA_Mβ
NIV
41.24-41.40Mb
OST
39.72-39.89Mb
RUF
38.07-38.24Mb
GLA
36.43-36.60Mb
BAR
37.12-37.28Mb
GLU
42.18-42.36Mb
LON
36.33-36.47Mb
MER
39.12-39.28Mb
PUN
44.75-44.92Mb
BRA
30.62-30.77Mb

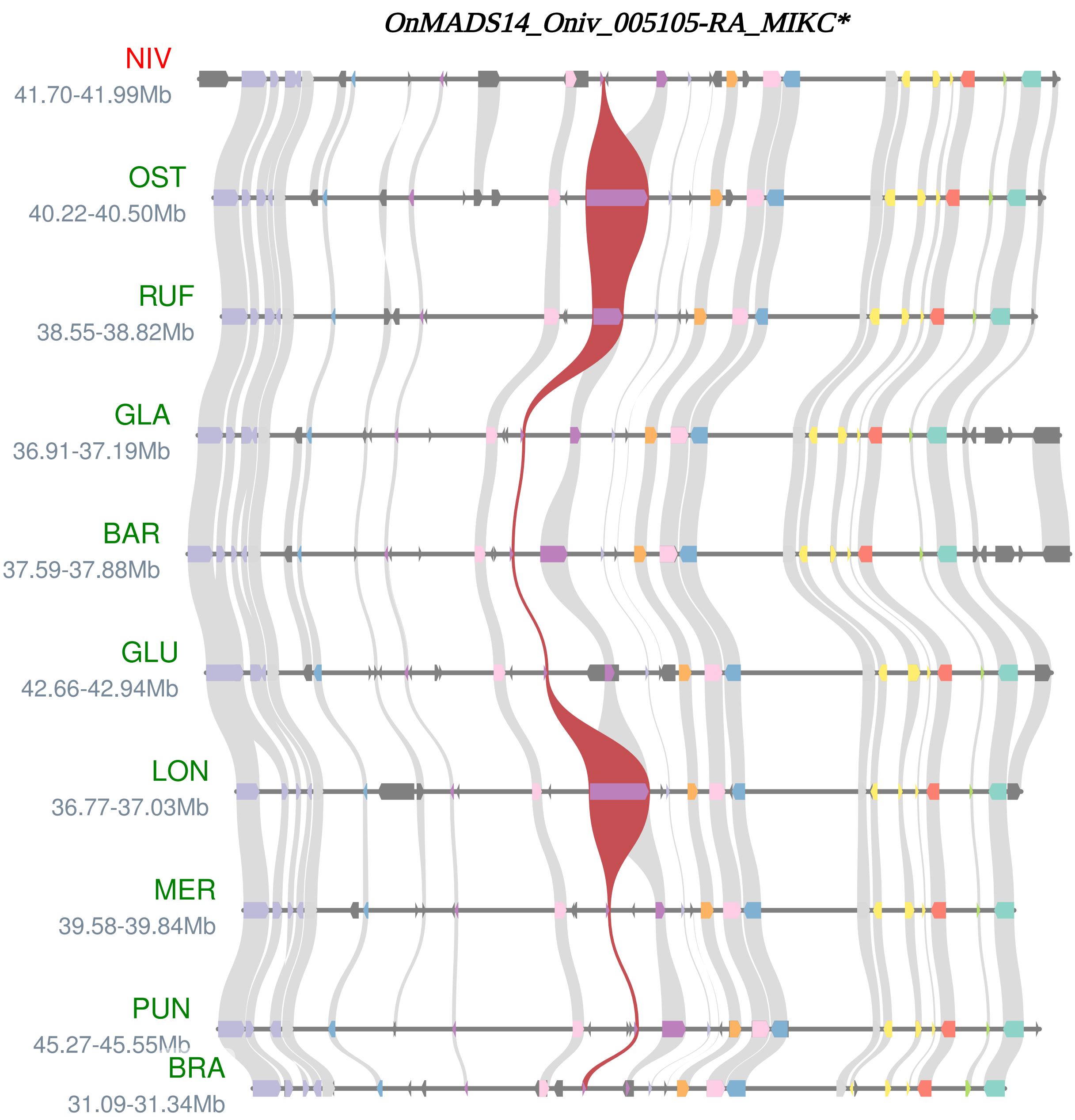

OnMADS14_Oniv_005105-RA_MIKC*
NIV
41.70-41.99Mb
OST
40.22-40.50Mb
RUF
38.55-38.82Mb
GLA
36.91-37.19Mb
BAR
37.59-37.88Mb
GLU
42.66-42.94Mb
LON
36.77-37.03Mb
MER
39.58-39.84Mb
PUN
45.27-45.55Mb
BRA
31.09-31.34Mb

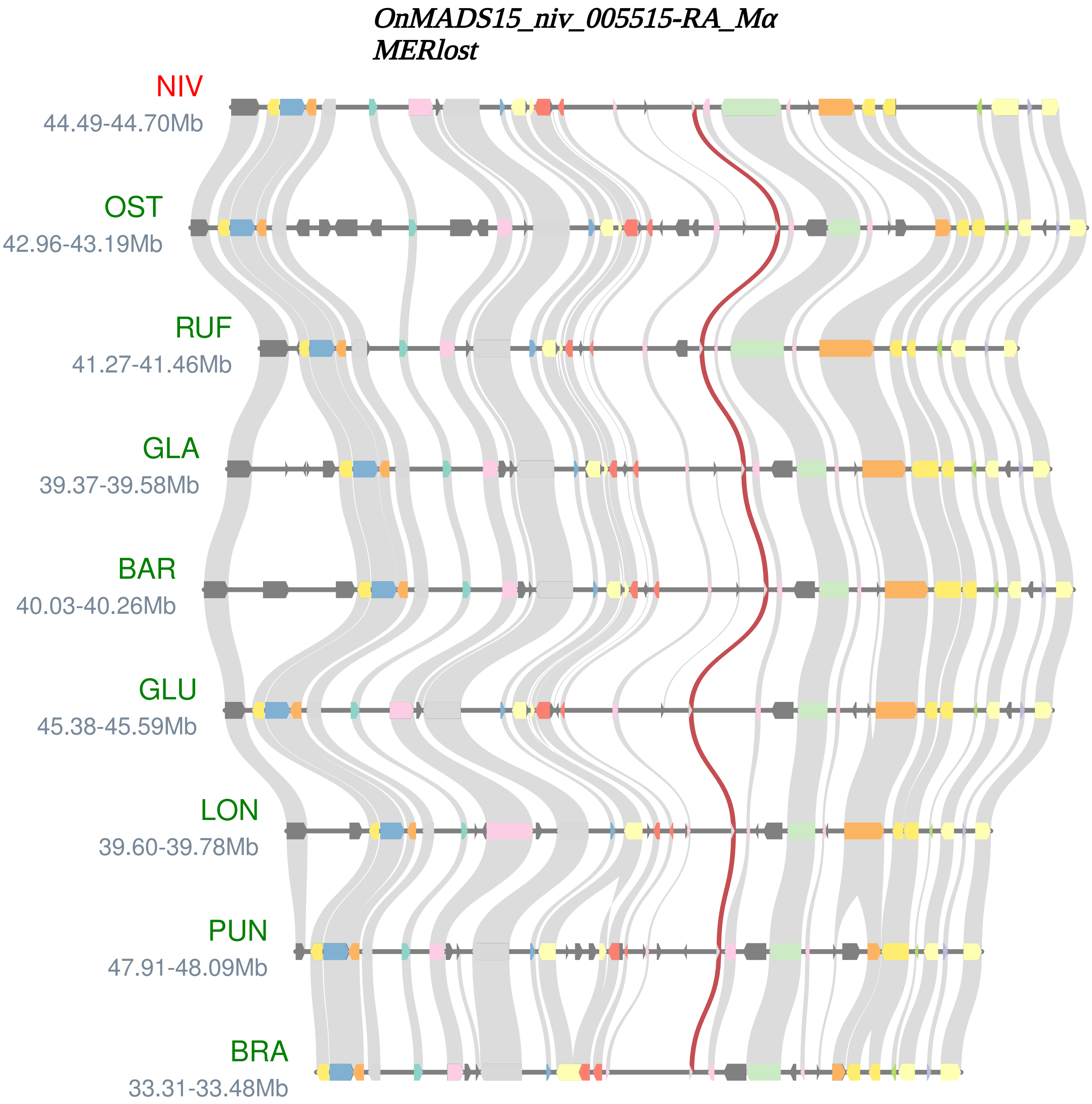
OnMADS15_niv_005515-RA_Mα
MERlost
NIV
44.49-44.70Mb
OST
42.96-43.19Mb
RUF
41.27-41.46Mb
GLA
39.37-39.58Mb
BAR
40.03-40.26Mb
GLU
45.38-45.59Mb
LON
39.60-39.78Mb
PUN
47.91-48.09Mb
BRA
33.31-33.48Mb

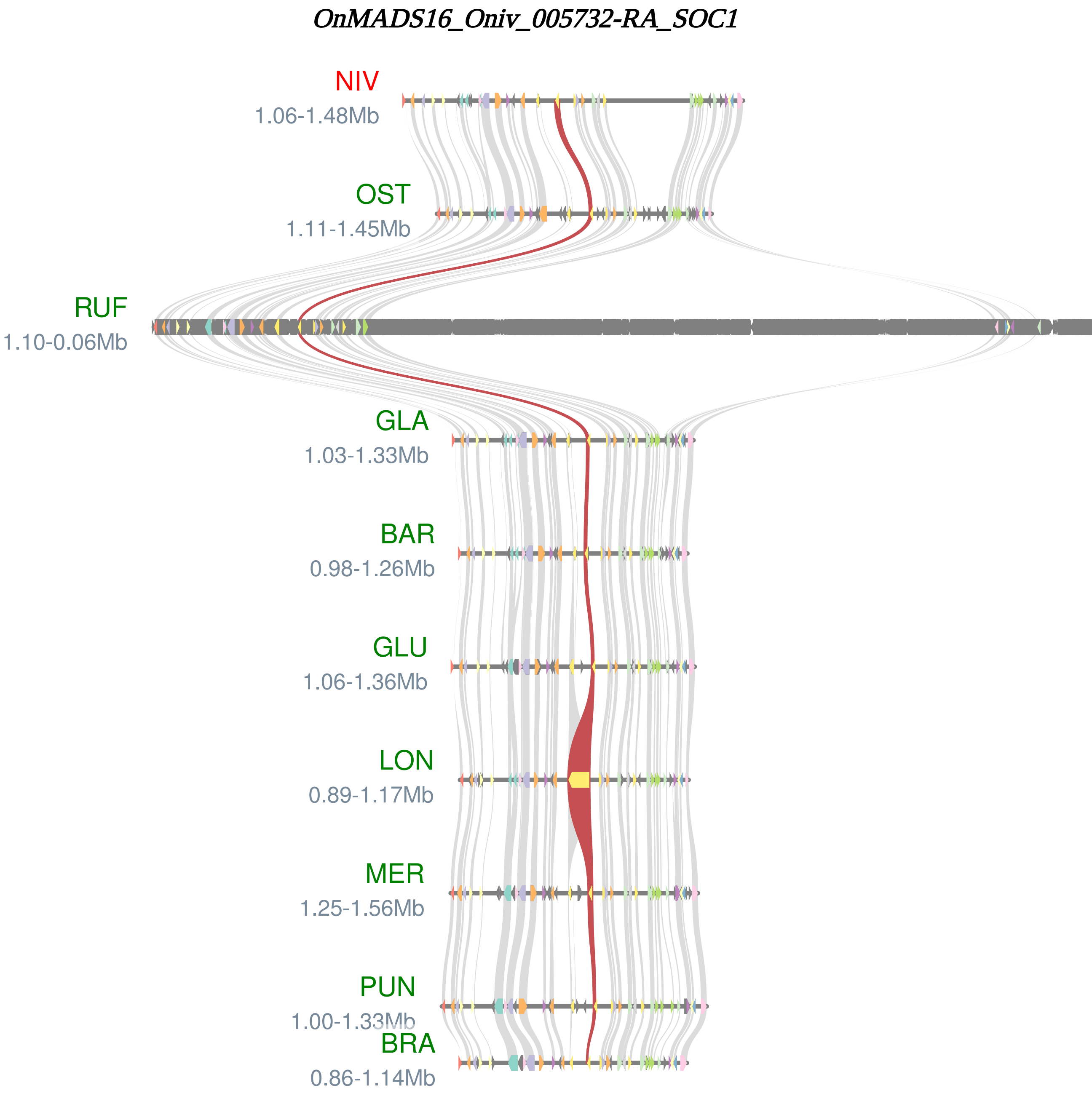
OnMADS16_Oniv_005732-RA_SOC1
NIV
1.06-1.48Mb
OST
1.11-1.45Mb
RUF
1.10-0.06Mb
GLA
1.03-1.33Mb
BAR
0.98-1.26Mb
GLU
1.06-1.36Mb
LON
0.89-1.17Mb
MER
1.25-1.56Mb
PUN
1.00-1.33Mb
BRA
0.86-1.14Mb

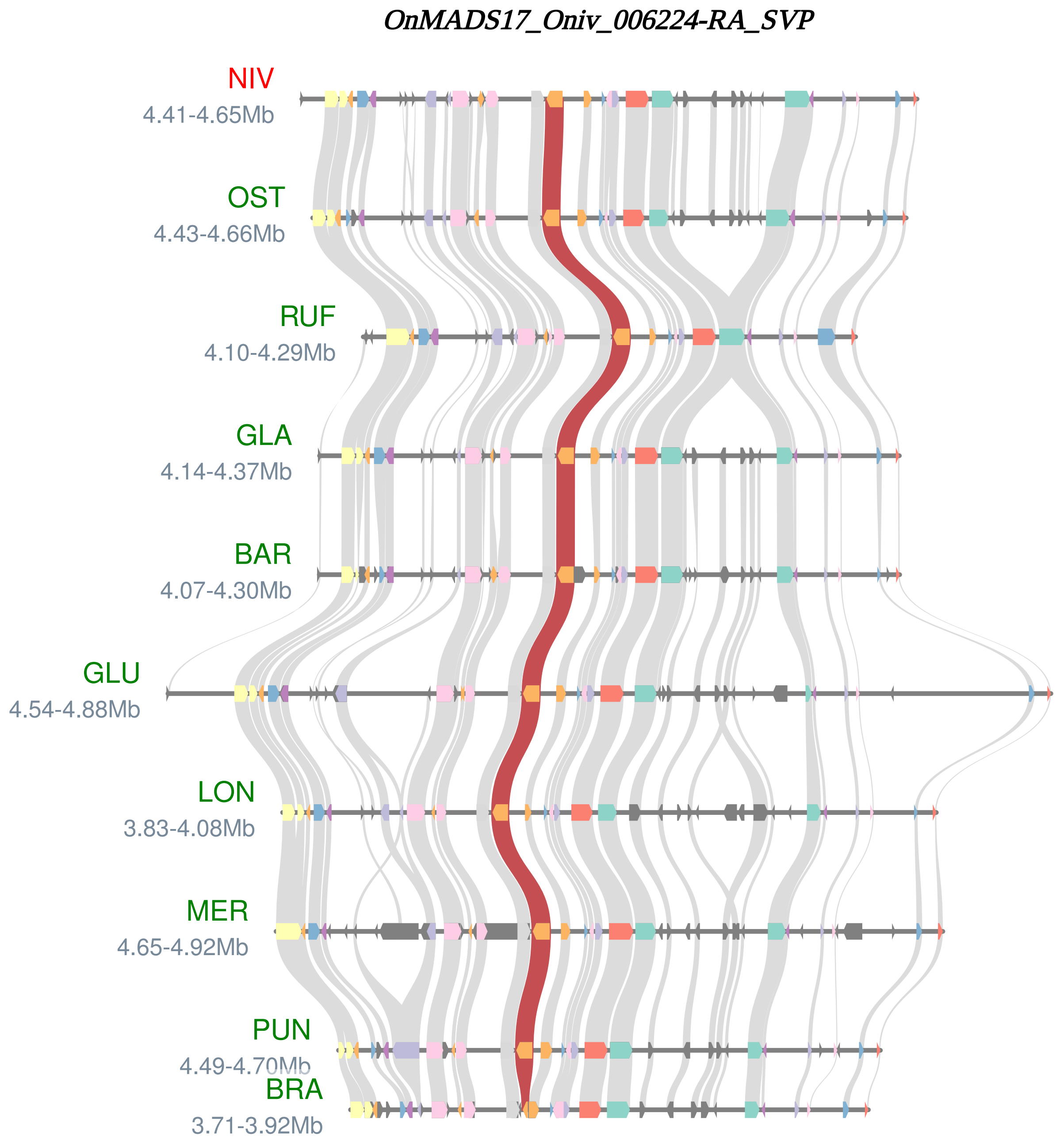
OnMADS17_Oniv_006224-RA_SVP
NIV
4.41-4.65Mb
OST
4.43-4.66Mb
RUF
4.10-4.29Mb
GLA
4.14-4.37Mb
BAR
4.07-4.30Mb
GLU
4.54-4.88Mb
LON
3.83-4.08Mb
MER
4.65-4.92Mb
PUN
4.49-4.70Mb
BRA
3.71-3.92Mb

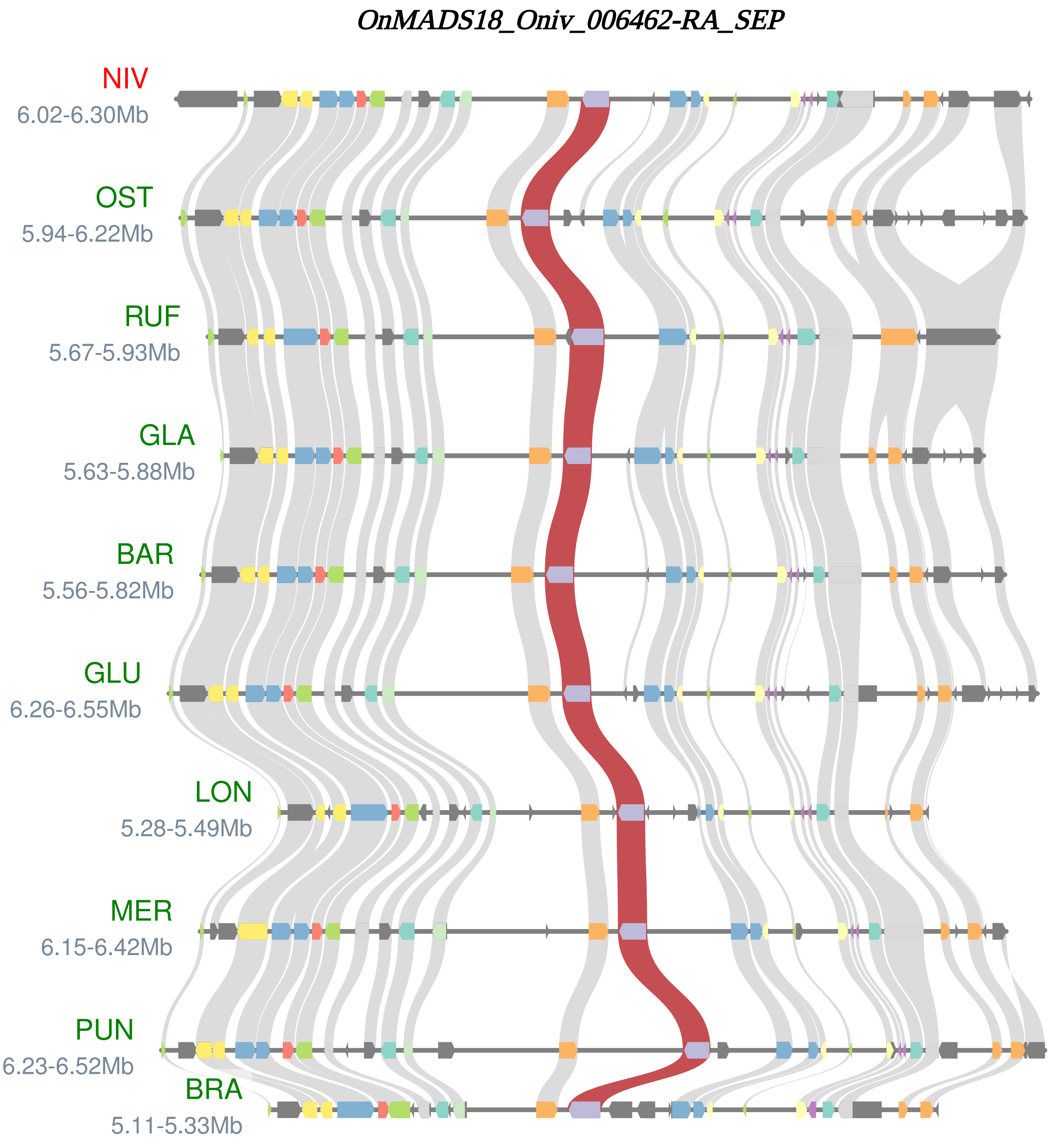
OnMADS18_Oniv_006462-RA_SEP
NIV
6.02-6.30Mb
OST
5.94-6.22Mb
RUF
5.67-5.93Mb
GLA
5.63-5.88Mb
BAR
5.56-5.82Mb
GLU
6.26-6.55Mb
LON
5.28-5.49Mb
MER
6.15-6.42Mb
PUN
6.23-6.52Mb
BRA
5.11-5.33Mb

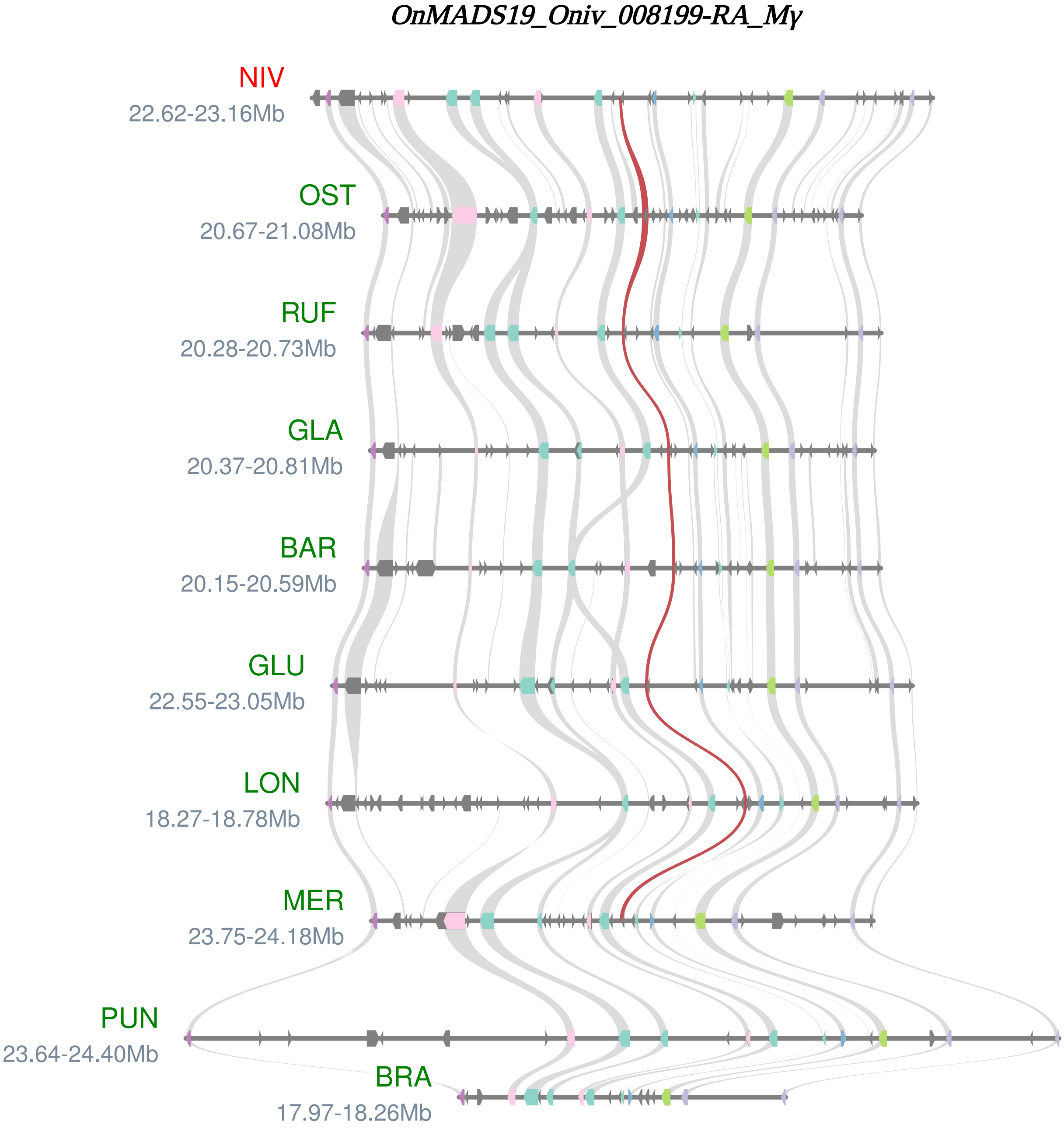
OnMADS19_Oniv_008199-RA_Mγ
NIV
22.62-23.16Mb
OST
20.67-21.08Mb
RUF
20.28-20.73Mb
GLA
20.37-20.81Mb
BAR
20.15-20.59Mb
GLU
22.55-23.05Mb
LON
18.27-18.78Mb
MER
23.75-24.18Mb
PUN
23.64-24.40Mb
BRA
17.97-18.26Mb

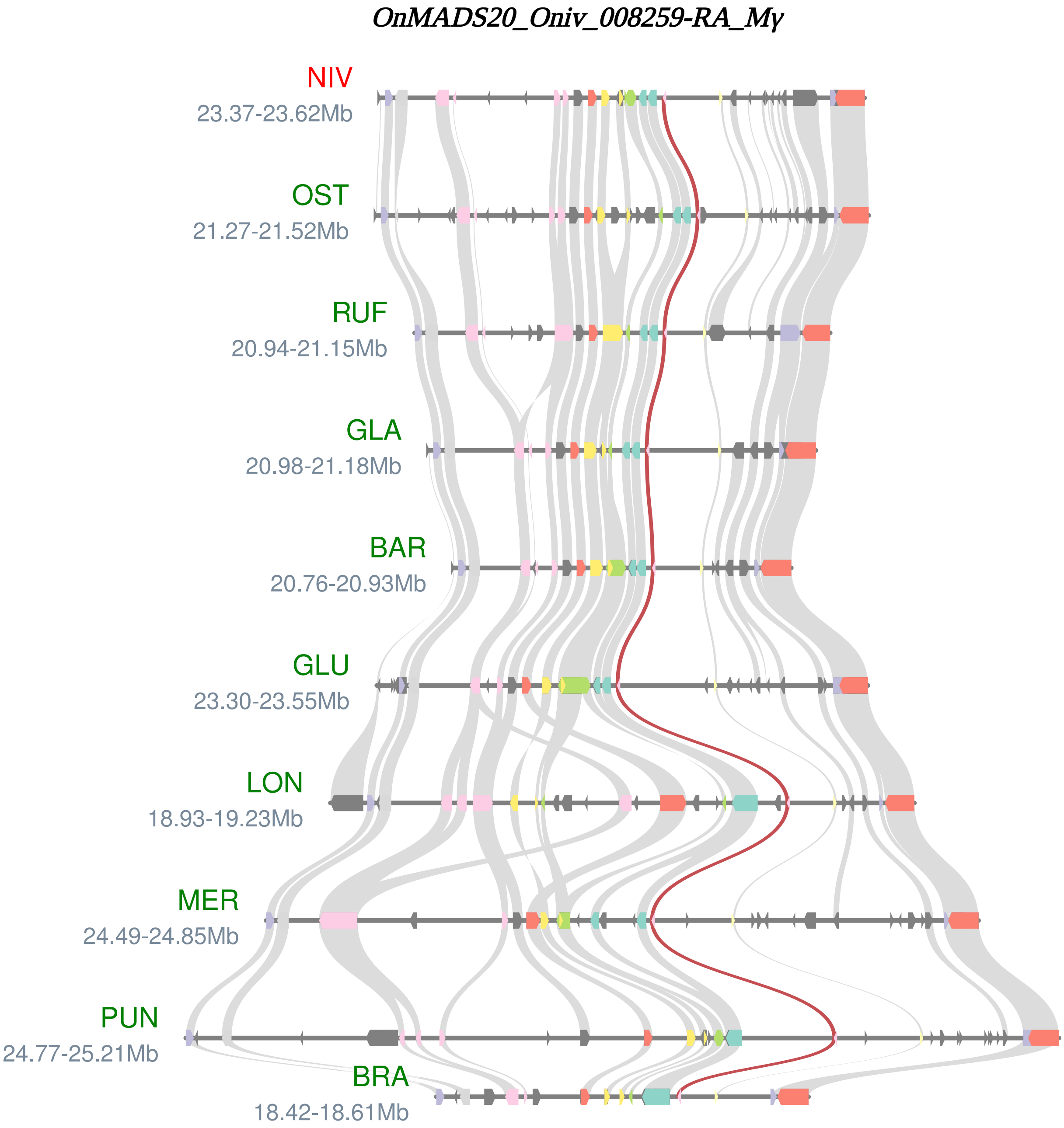

OnMADS20_Oniv_008259-RA_Mγ
NIV
23.37-23.62Mb
OST
21.27-21.52Mb
RUF
20.94-21.15Mb
GLA
20.98-21.18Mb
BAR
20.76-20.93Mb
GLU
23.30-23.55Mb
LON
18.93-19.23Mb
MER
24.49-24.85Mb
PUN
24.77-25.21Mb
BRA
18.42-18.61Mb

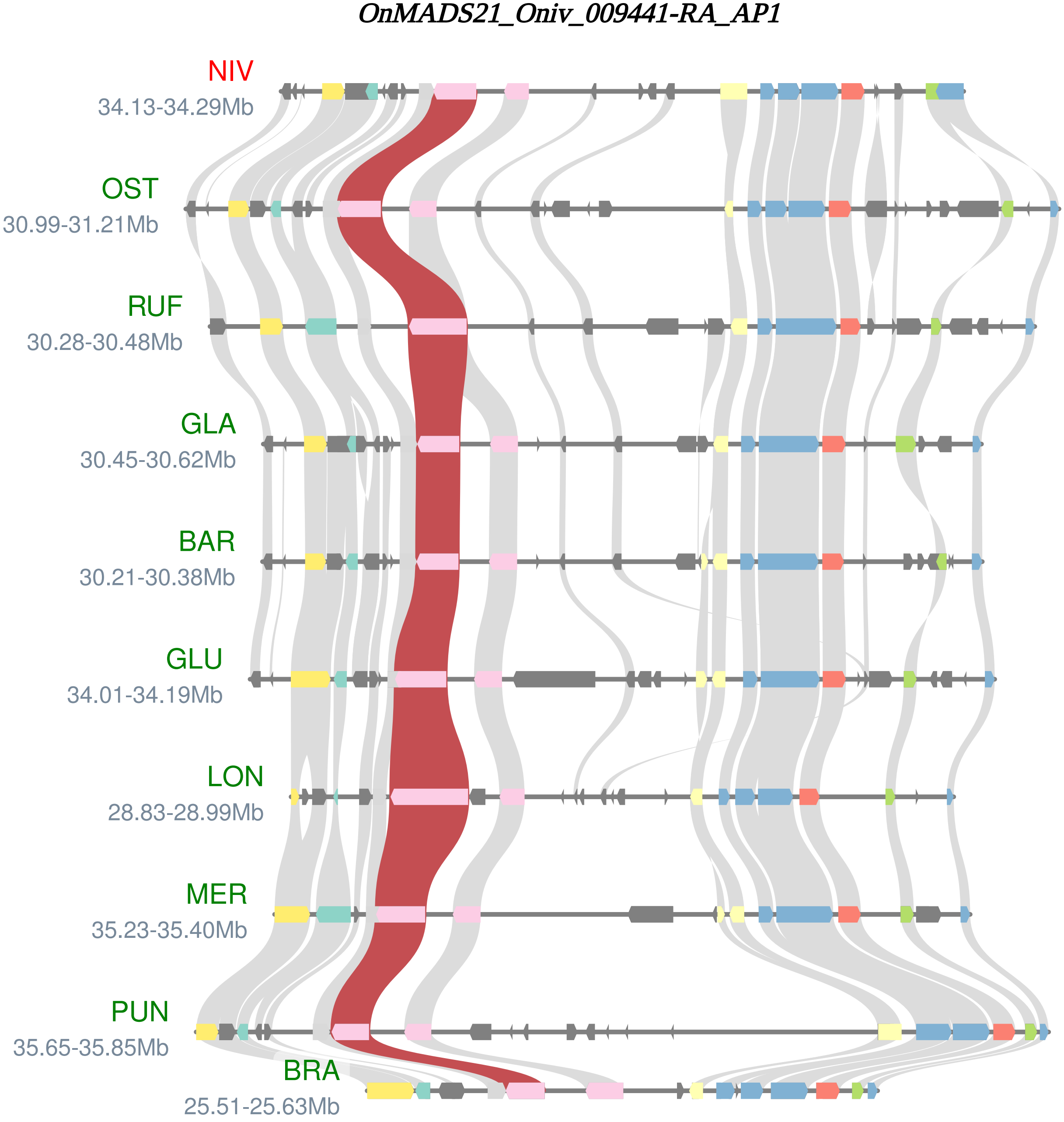
OnMADS21_Oniv_009441-RA_AP1
NIV
34.13-34.29Mb
OST
30.99-31.21Mb
RUF
30.28-30.48Mb
GLA
30.45-30.62Mb
BAR
30.21-30.38Mb
GLU
34.01-34.19Mb
LON
28.83-28.99Mb
MER
35.23-35.40Mb
PUN
35.65-35.85Mb
BRA
25.51-25.63Mb

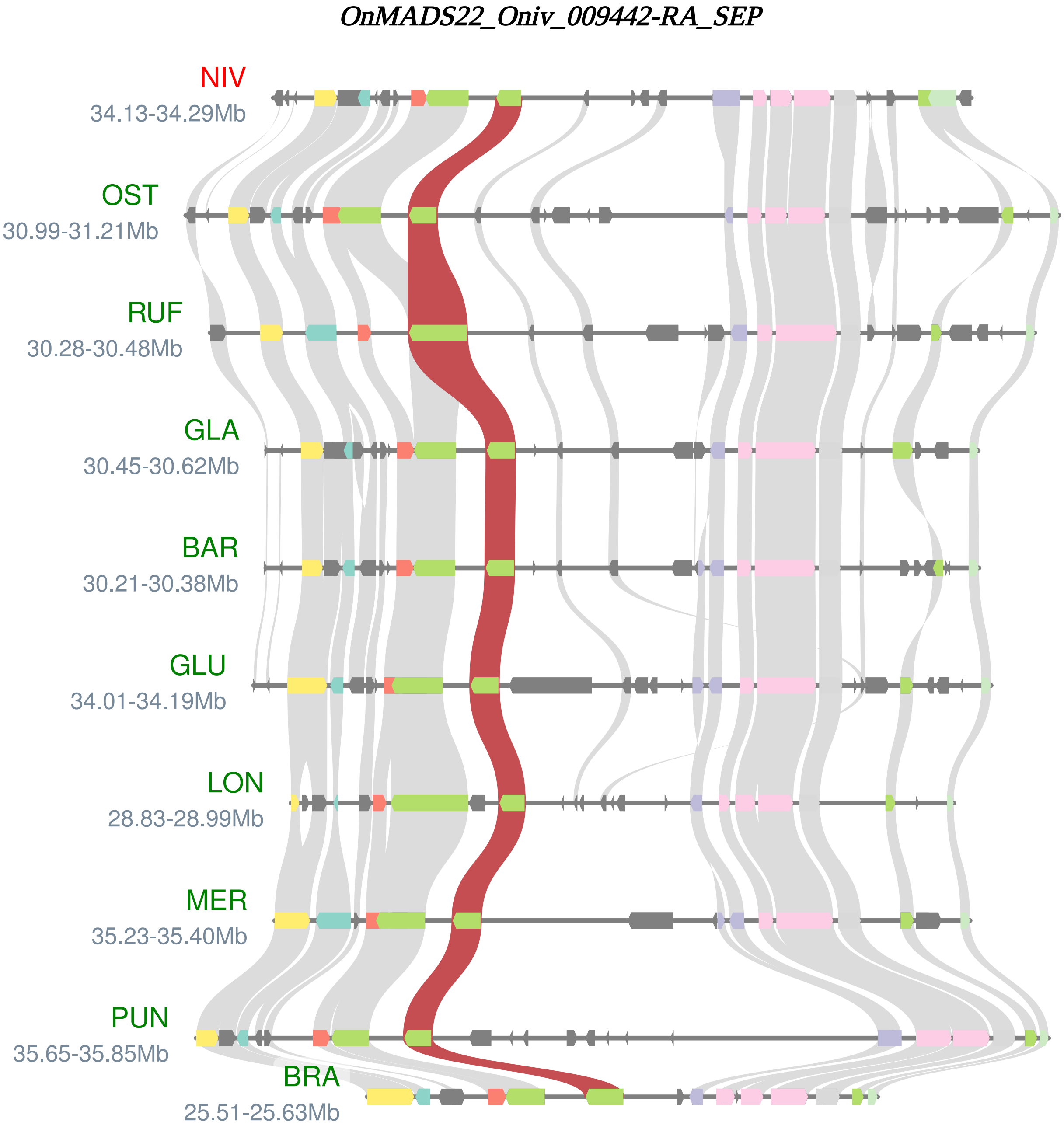

OnMADS22_Oniv_009442-RA_SEP
NIV
34.13-34.29Mb
OST
30.99-31.21Mb
RUF
30.28-30.48Mb
GLA
30.45-30.62Mb
BAR
30.21-30.38Mb
GLU
34.01-34.19Mb
LON
28.83-28.99Mb
MER
35.23-35.40Mb
PUN
35.65-35.85Mb
BRA
25.51-25.63Mb

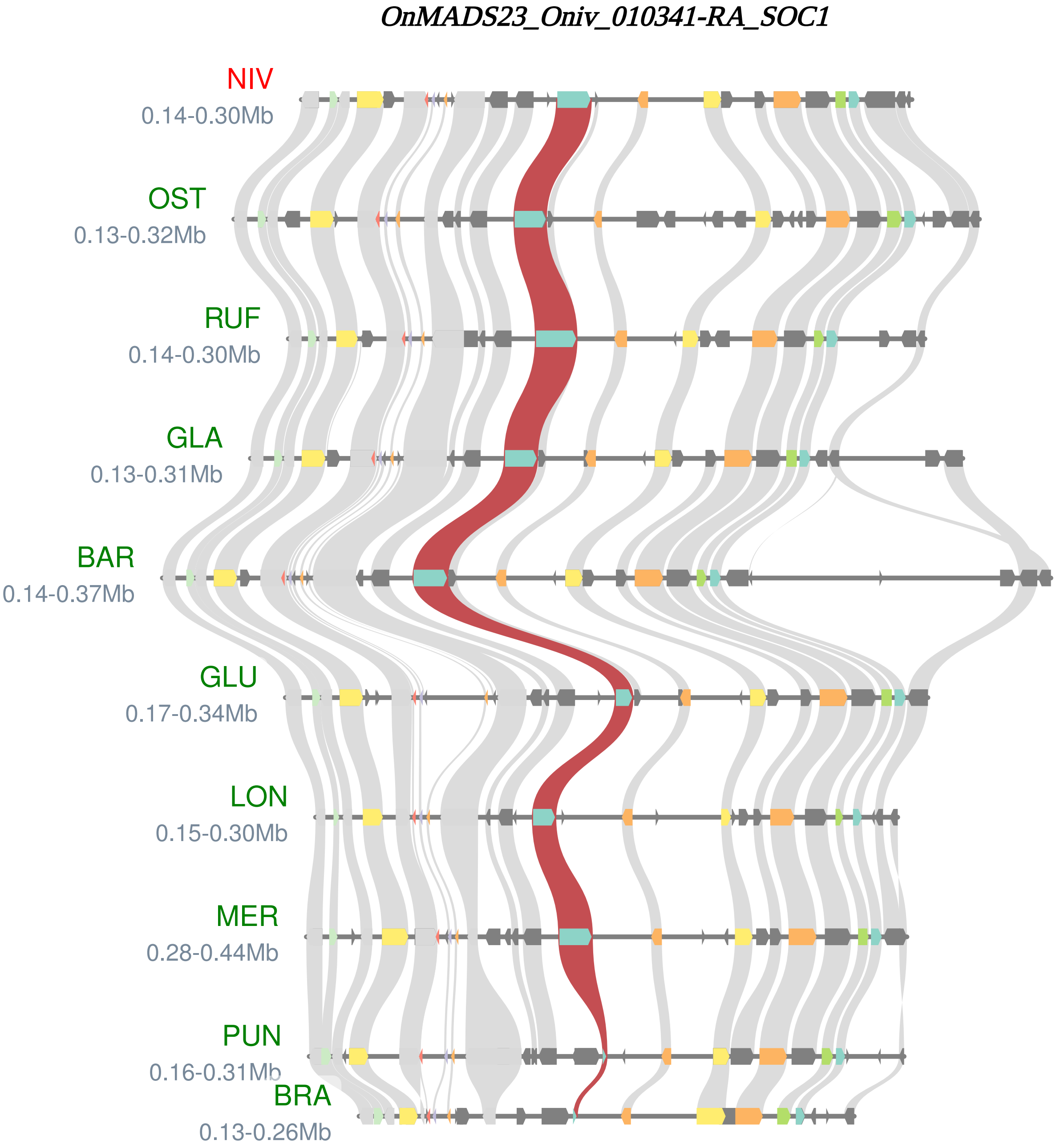
OnMADS23_Oniv_010341-RA_SOC1
NIV
0.14-0.30Mb
OST
0.13-0.32Mb
RUF
0.14-0.30Mb
GLA
0.13-0.31Mb
BAR
0.14-0.37Mb
GLU
0.17-0.34Mb
LON
0.15-0.30Mb
MER
0.28-0.44Mb
PUN
0.16-0.31Mb
BRA
0.13-0.26Mb

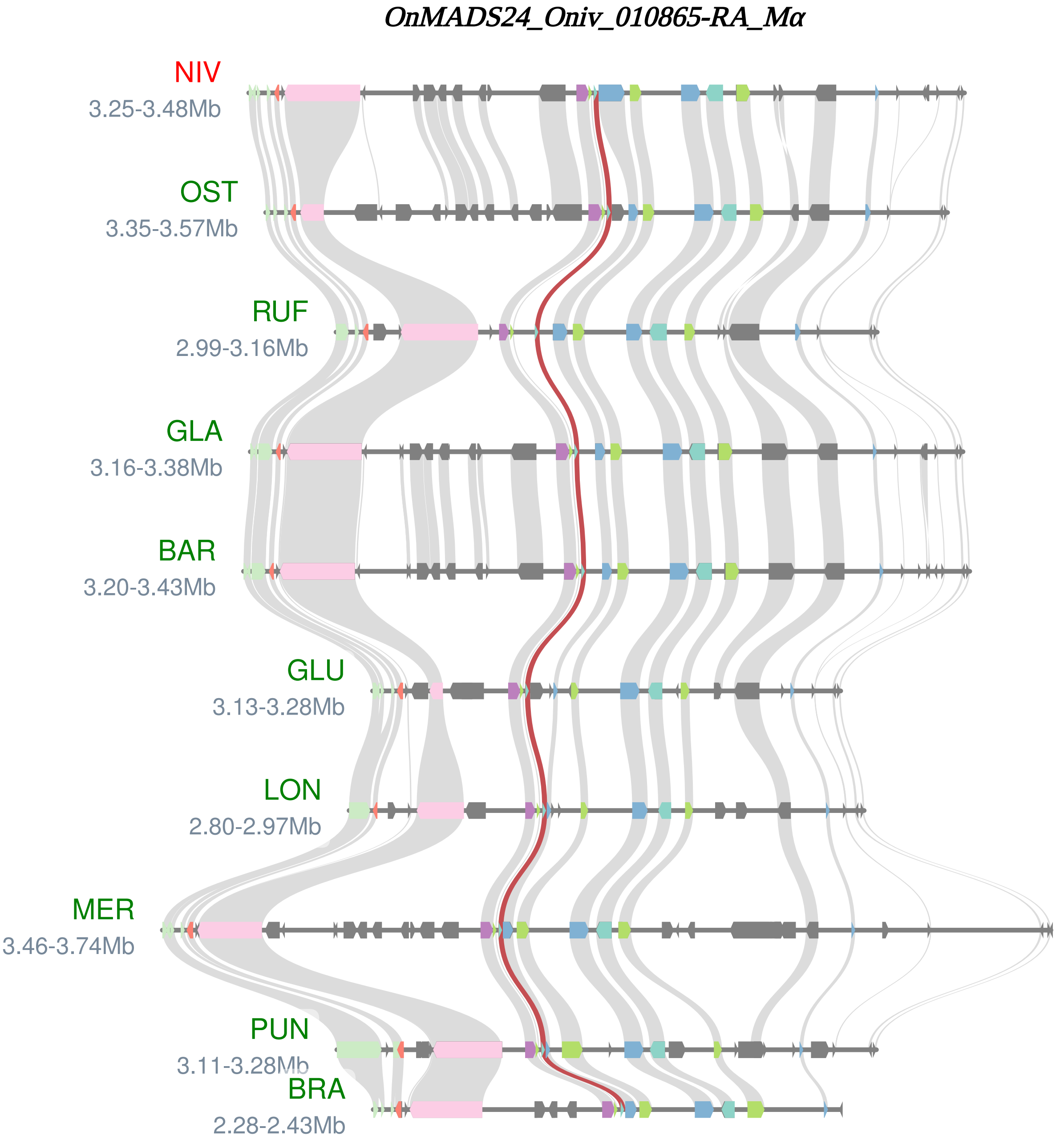
OnMADS24_Oniv_010865-RA_Mα
NIV
3.25-3.48Mb
OST
3.35-3.57Mb
RUF
2.99-3.16Mb
GLA
3.16-3.38Mb
BAR
3.20-3.43Mb
GLU
3.13-3.28Mb
LON
2.80-2.97Mb
MER
3.46-3.74Mb
PUN
3.11-3.28Mb
BRA
2.28-2.43Mb

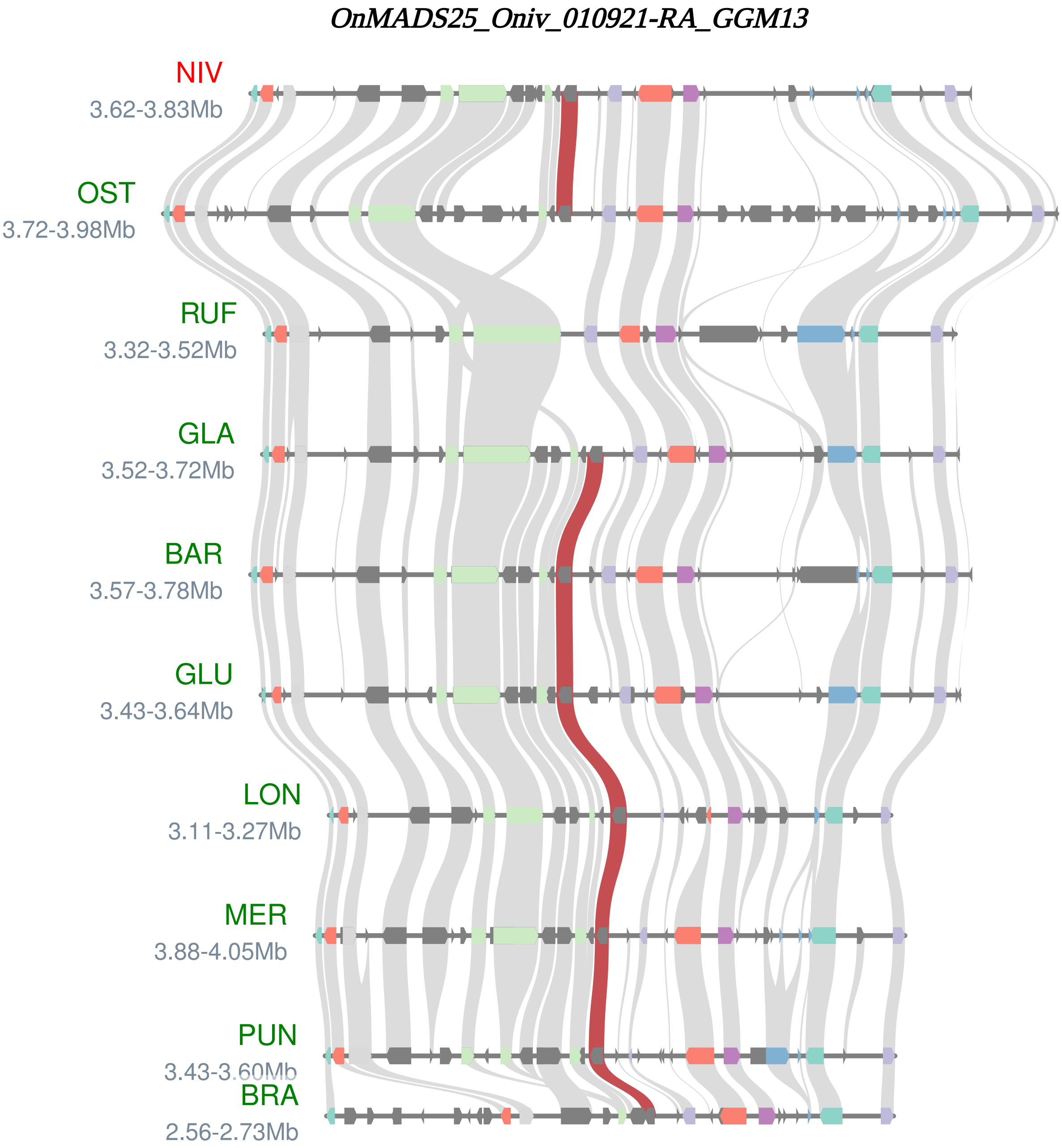
OnMADS25_Oniv_010921-RA_GGM13
NIV
3.62-3.83Mb
OST
3.72-3.98Mb
RUF
3.32-3.52Mb
GLA
3.52-3.72Mb
BAR
3.57-3.78Mb
GLU
3.43-3.64Mb
LON
3.11-3.27Mb
MER
3.88-4.05Mb
PUN
3.43-3.60Mb
BRA
2.56-2.73Mb

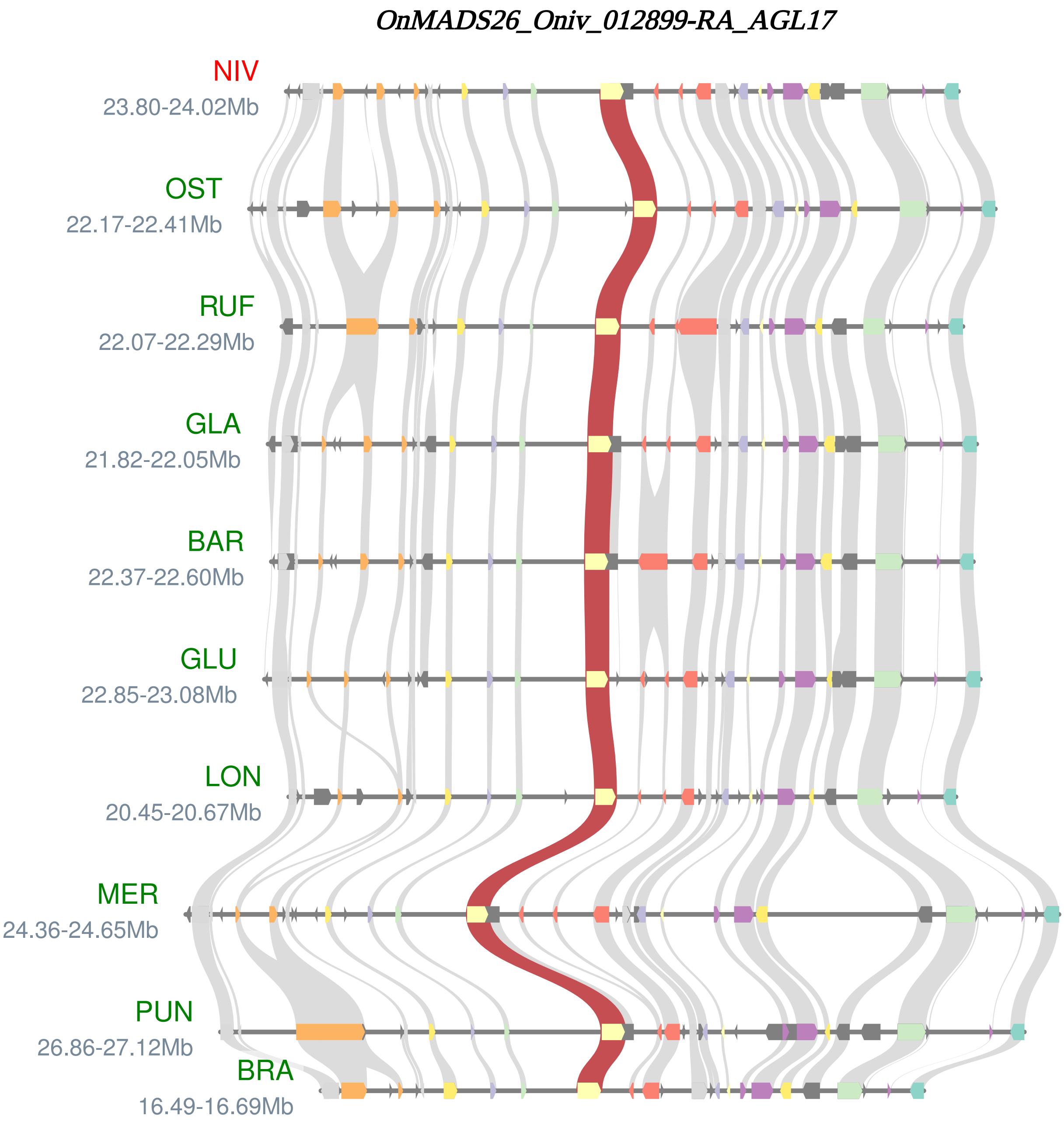
OnMADS26_Oniv_012899-RA_AGL17
NIV
23.80-24.02Mb
OST
22.17-22.41Mb
RUF
22.07-22.29Mb
GLA
21.82-22.05Mb
BAR
22.37-22.60Mb
GLU
22.85-23.08Mb
LON
20.45-20.67Mb
MER
24.36-24.65Mb
PUN
26.86-27.12Mb
BRA
16.49-16.69Mb

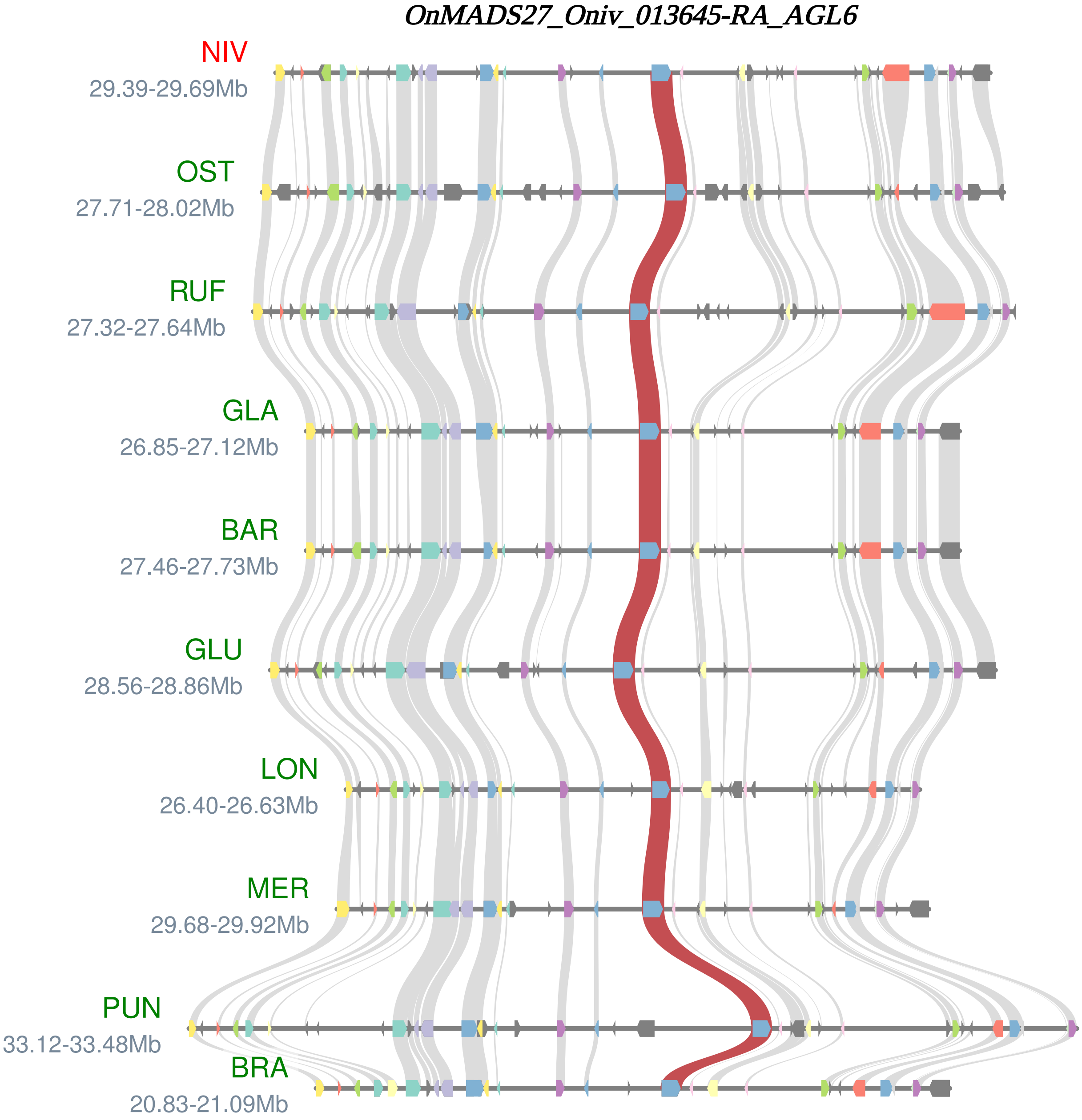

OnMADS27_Oniv_013645-RA_AGL6
NIV
29.39-29.69Mb
OST
27.71-28.02Mb
RUF
27.32-27.64Mb
GLA
26.85-27.12Mb
BAR
27.46-27.73Mb
GLU
28.56-28.86Mb
LON
26.40-26.63Mb
MER
29.68-29.92Mb
PUN
33.12-33.48Mb
BRA
20.83-21.09Mb

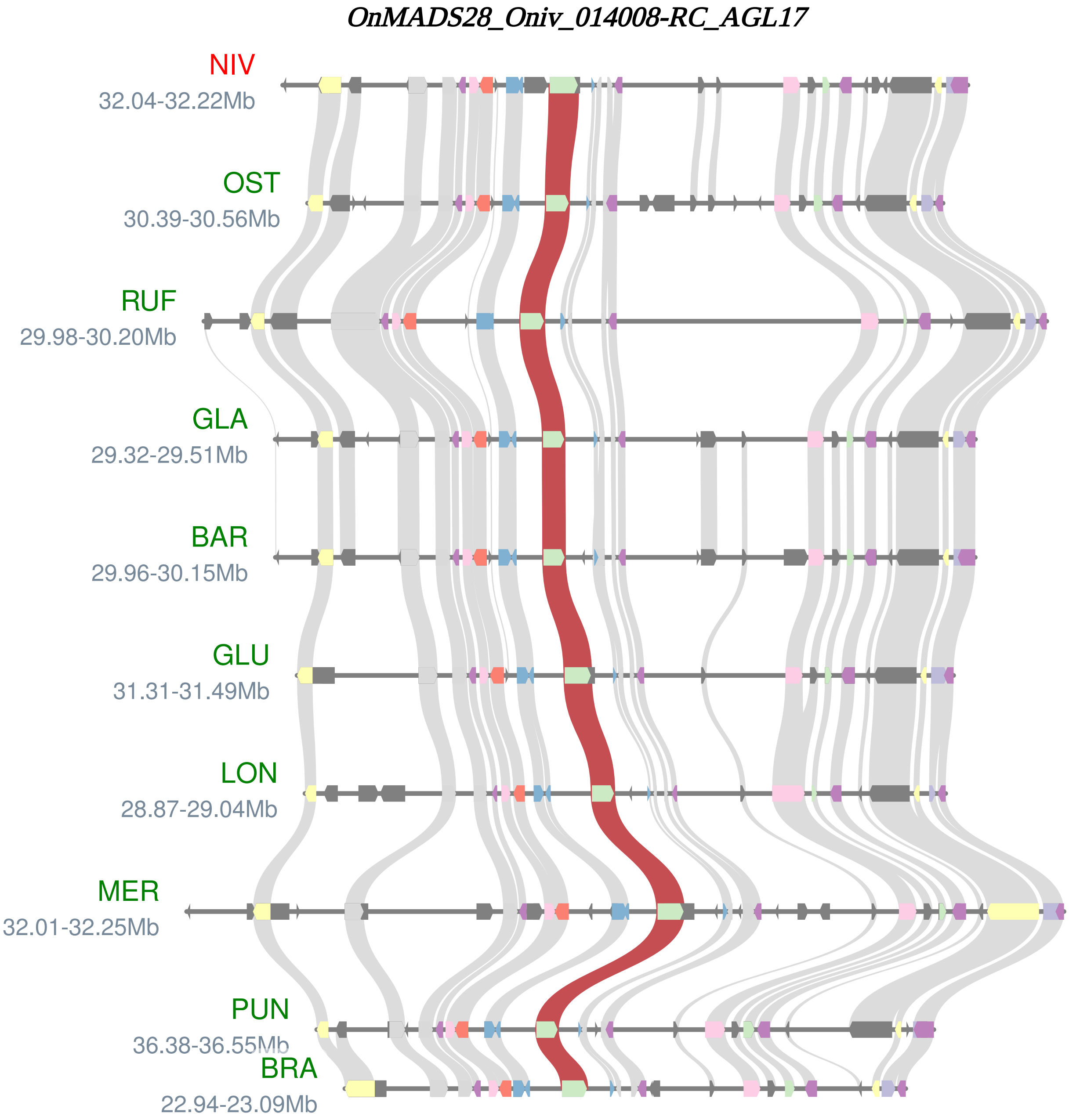

OnMADS28_Oniv_014008-RC_AGL17
NIV
32.04-32.22Mb
OST
30.39-30.56Mb
RUF
29.98-30.20Mb
GLA
29.32-29.51Mb
BAR
29.96-30.15Mb
GLU
31.31-31.49Mb
LON
28.87-29.04Mb
MER
32.01-32.25Mb
PUN
36.38-36.55Mb
BRA
22.94-23.09Mb

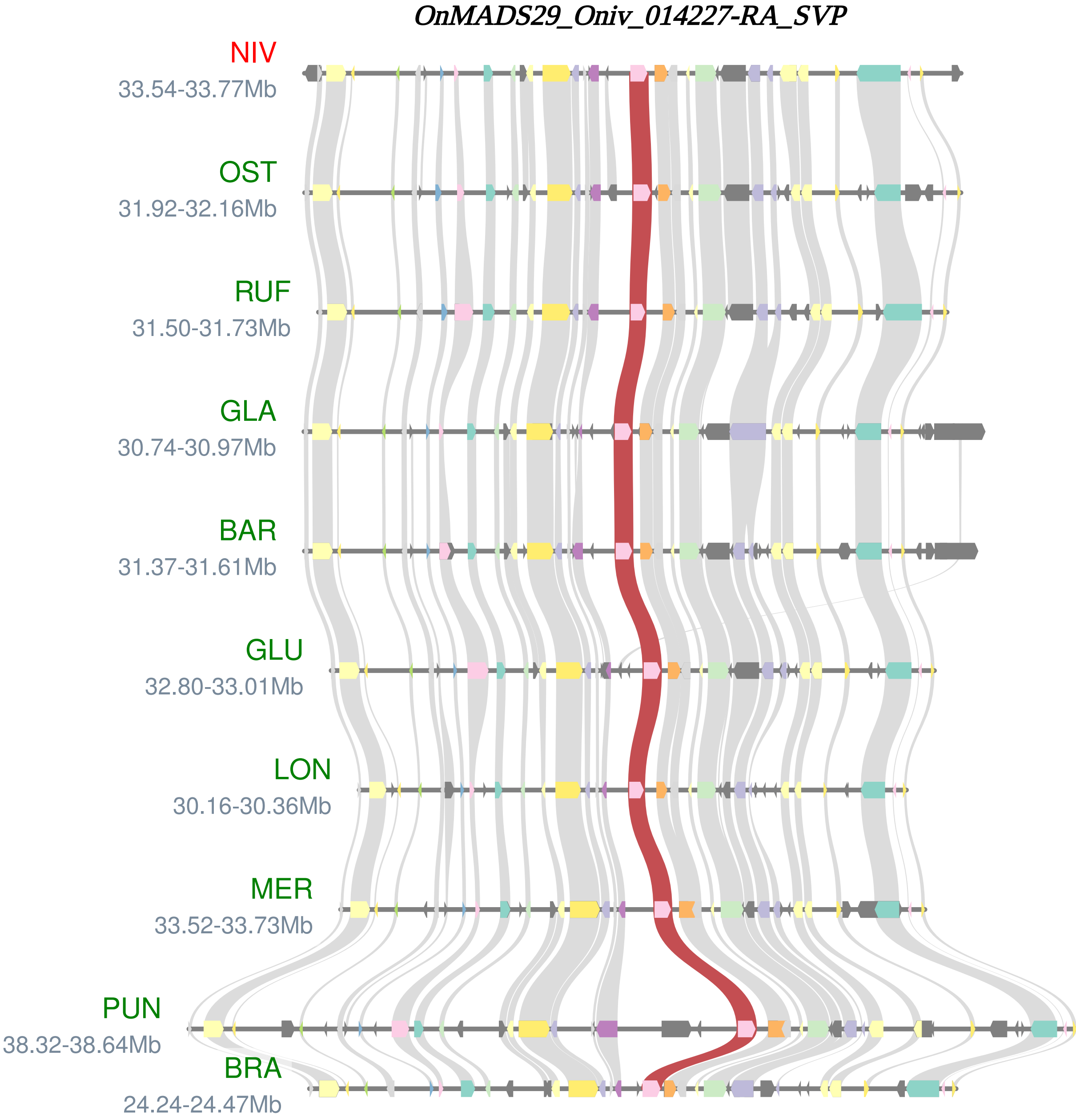

OnMADS29_Oniv_014227-RA_SVP
NIV
33.54-33.77Mb
OST
31.92-32.16Mb
RUF
31.50-31.73Mb
GLA
30.74-30.97Mb
BAR
31.37-31.61Mb
GLU
32.80-33.01Mb
LON
30.16-30.36Mb
MER
33.52-33.73Mb
PUN
38.32-38.64Mb
BRA
24.24-24.47Mb

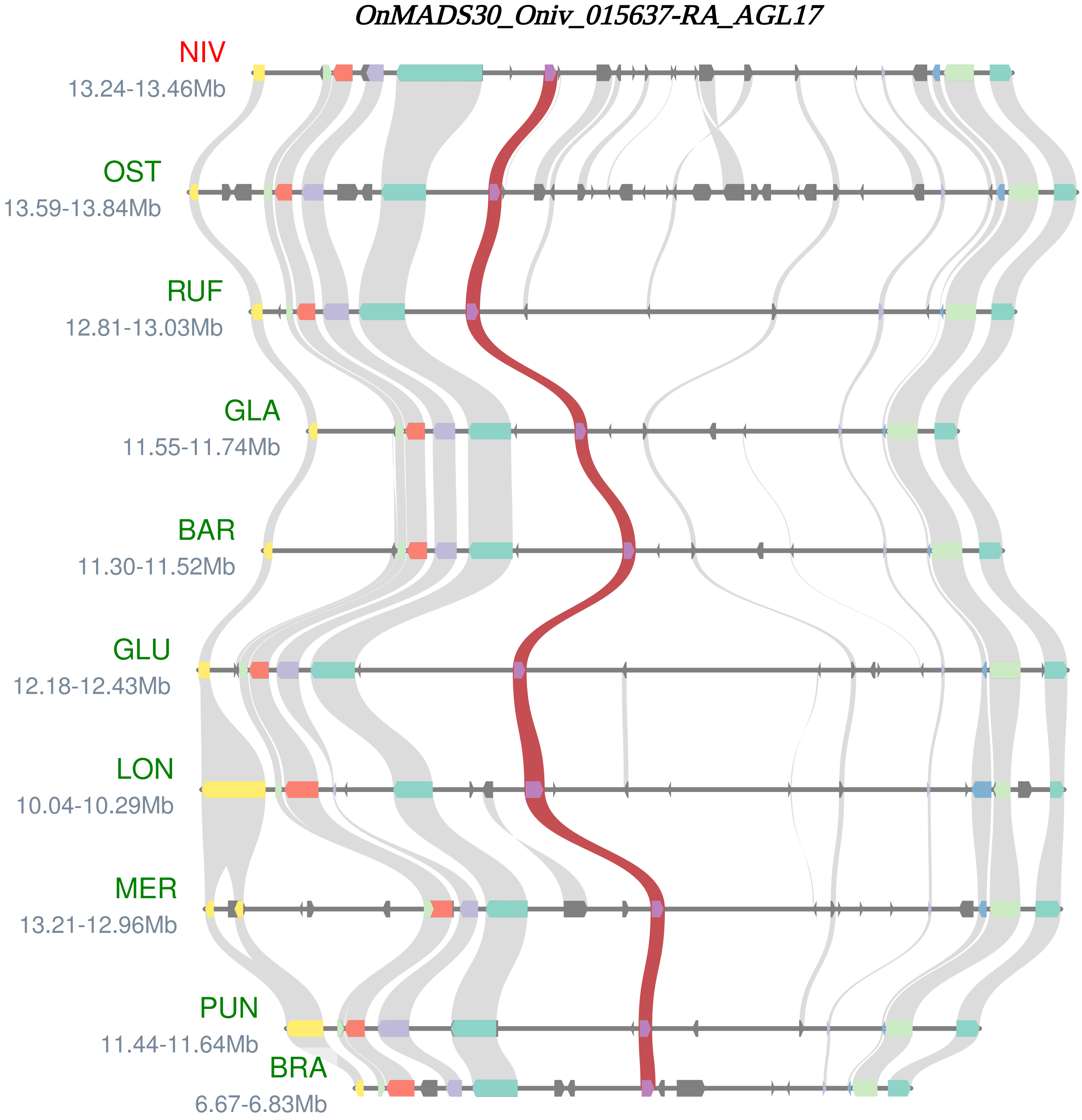
OnMADS30_Oniv_015637-RA_AGL17
NIV
13.24-13.46Mb
OST
13.59-13.84Mb
RUF
12.81-13.03Mb
GLA
11.55-11.74Mb
BAR
11.30-11.52Mb
GLU
12.18-12.43Mb
LON
10.04-10.29Mb
MER
13.21-12.96Mb
PUN
11.44-11.64Mb
BRA
6.67-6.83Mb

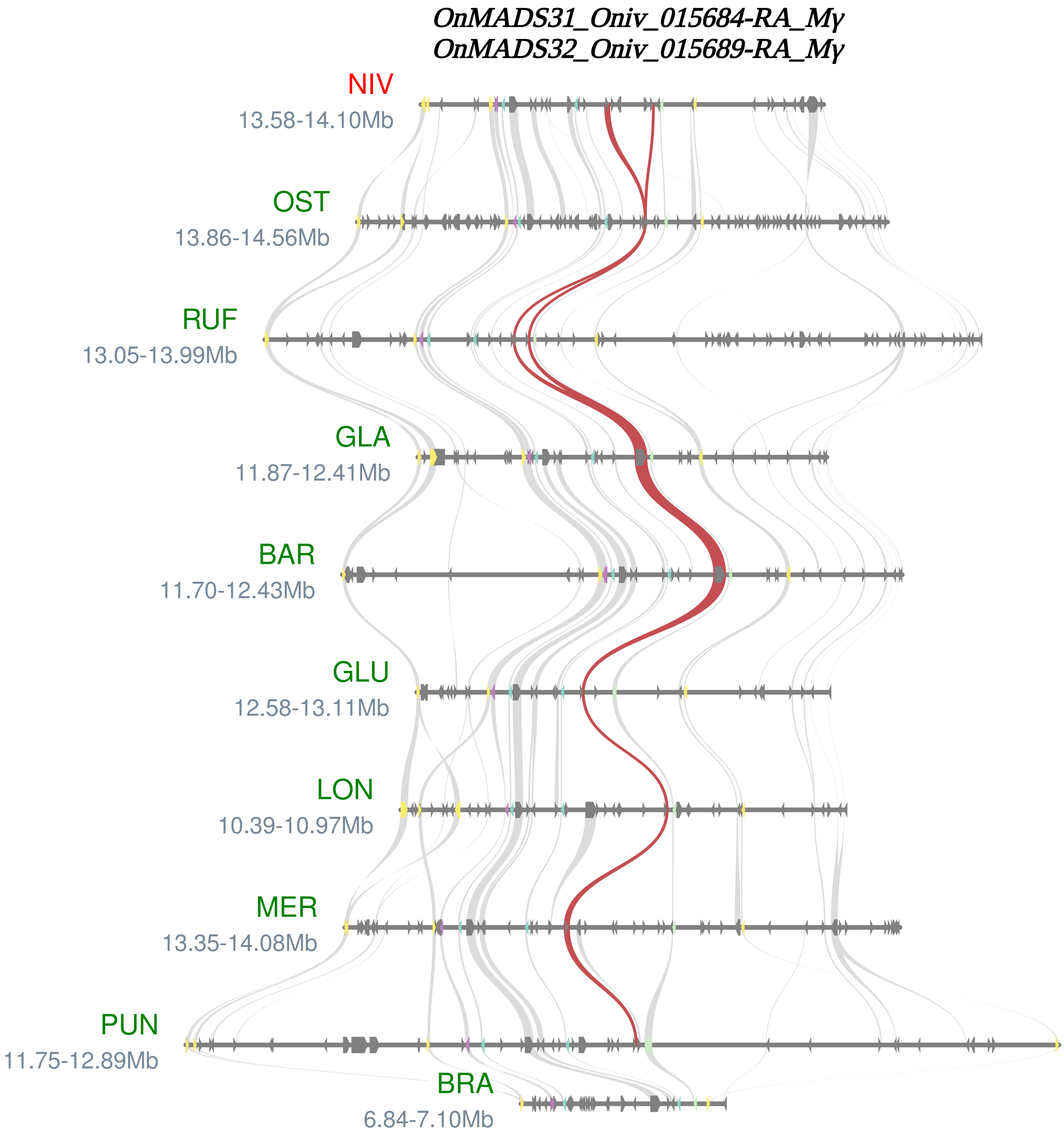

OnMADS31_Oniv_015684-RA_Mγ
OnMADS32_Oniv_015689-RA_Mγ
NIV
13.58-14.10Mb
OST
13.86-14.56Mb
RUF
13.05-13.99Mb
GLA
11.87-12.41Mb
BAR
11.70-12.43Mb
GLU
12.58-13.11Mb
LON
10.39-10.97Mb
MER
13.35-14.08Mb
PUN
11.75-12.89Mb
BRA
6.84-7.10Mb

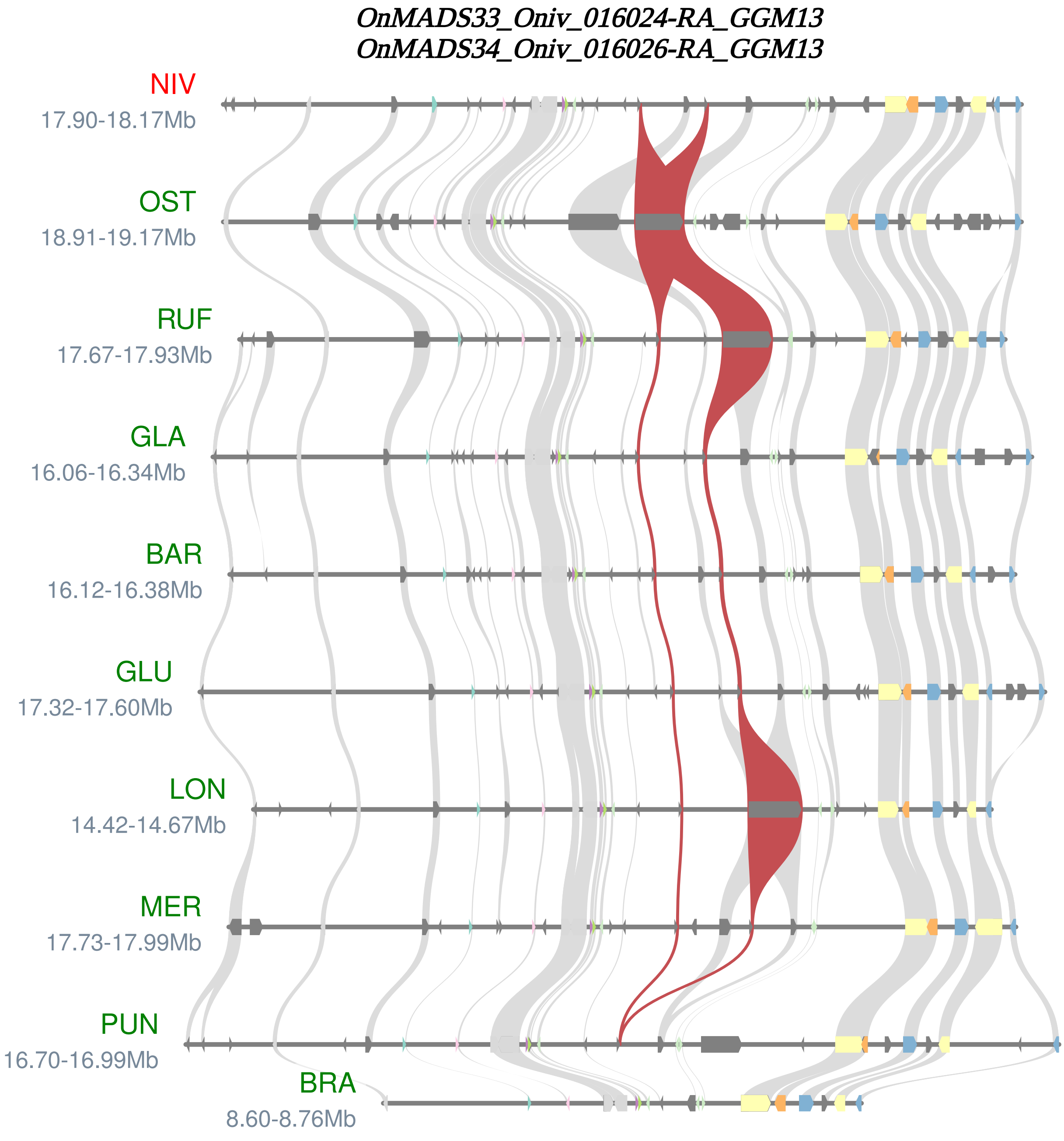
OnMADS33_Oniv_016024-RA_GGM13
OnMADS34_Oniv_016026-RA_GGM13
NIV
17.90-18.17Mb
OST
18.91-19.17Mb
RUF
17.67-17.93Mb
GLA
16.06-16.34Mb
BAR
16.12-16.38Mb
GLU
17.32-17.60Mb
LON
14.42-14.67Mb
MER
17.73-17.99Mb
PUN
16.70-16.99Mb
BRA
8.60-8.76Mb

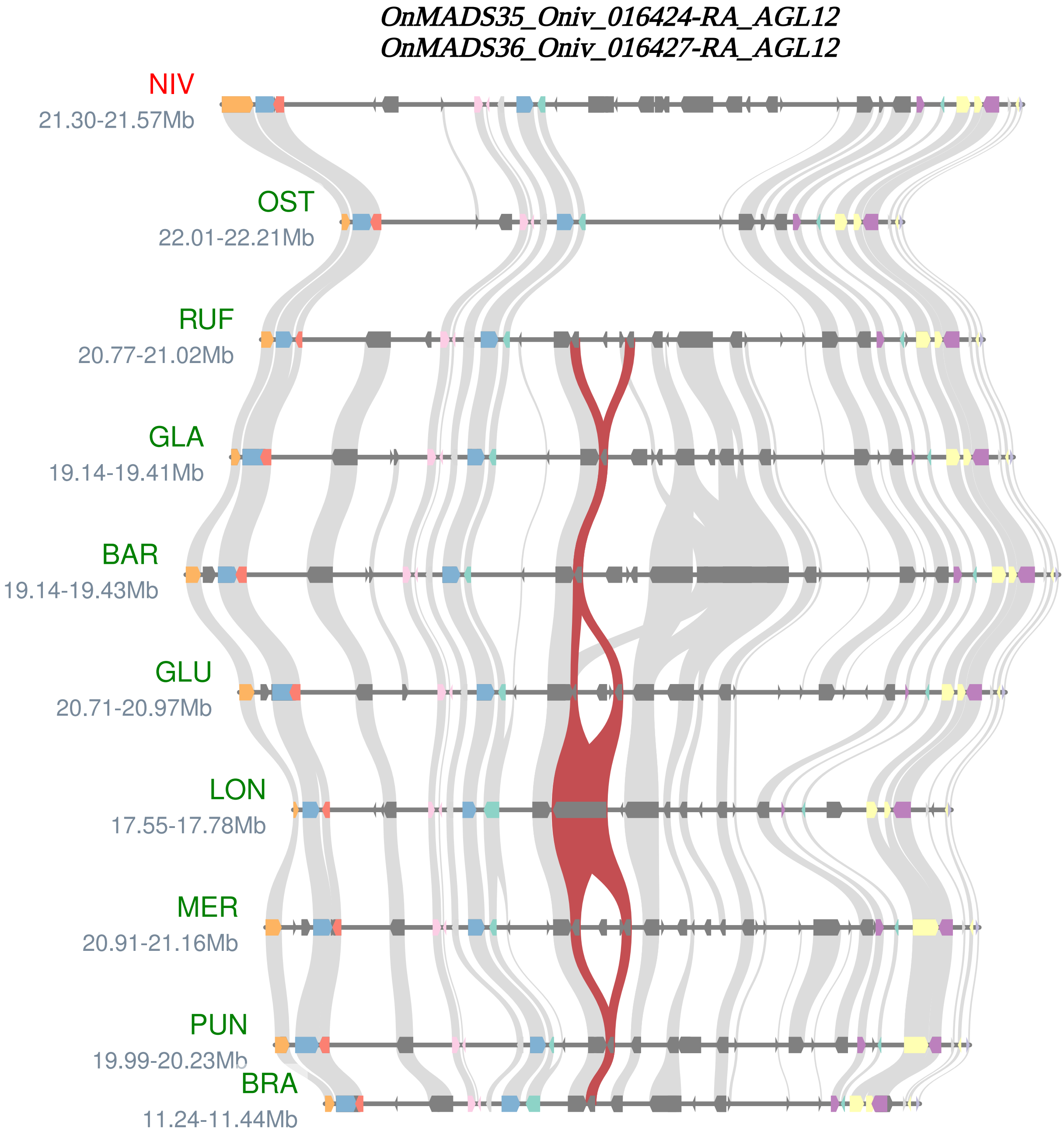
OnMADS35_Oniv_016424-RA_AGL12
OnMADS36_Oniv_016427-RA_AGL12
NIV
21.30-21.57Mb
OST
22.01-22.21Mb
RUF
20.77-21.02Mb
GLA
19.14-19.41Mb
BAR
19.14-19.43Mb
GLU
20.71-20.97Mb
LON
17.55-17.78Mb
MER
20.91-21.16Mb
PUN
19.99-20.23Mb
BRA
11.24-11.44Mb

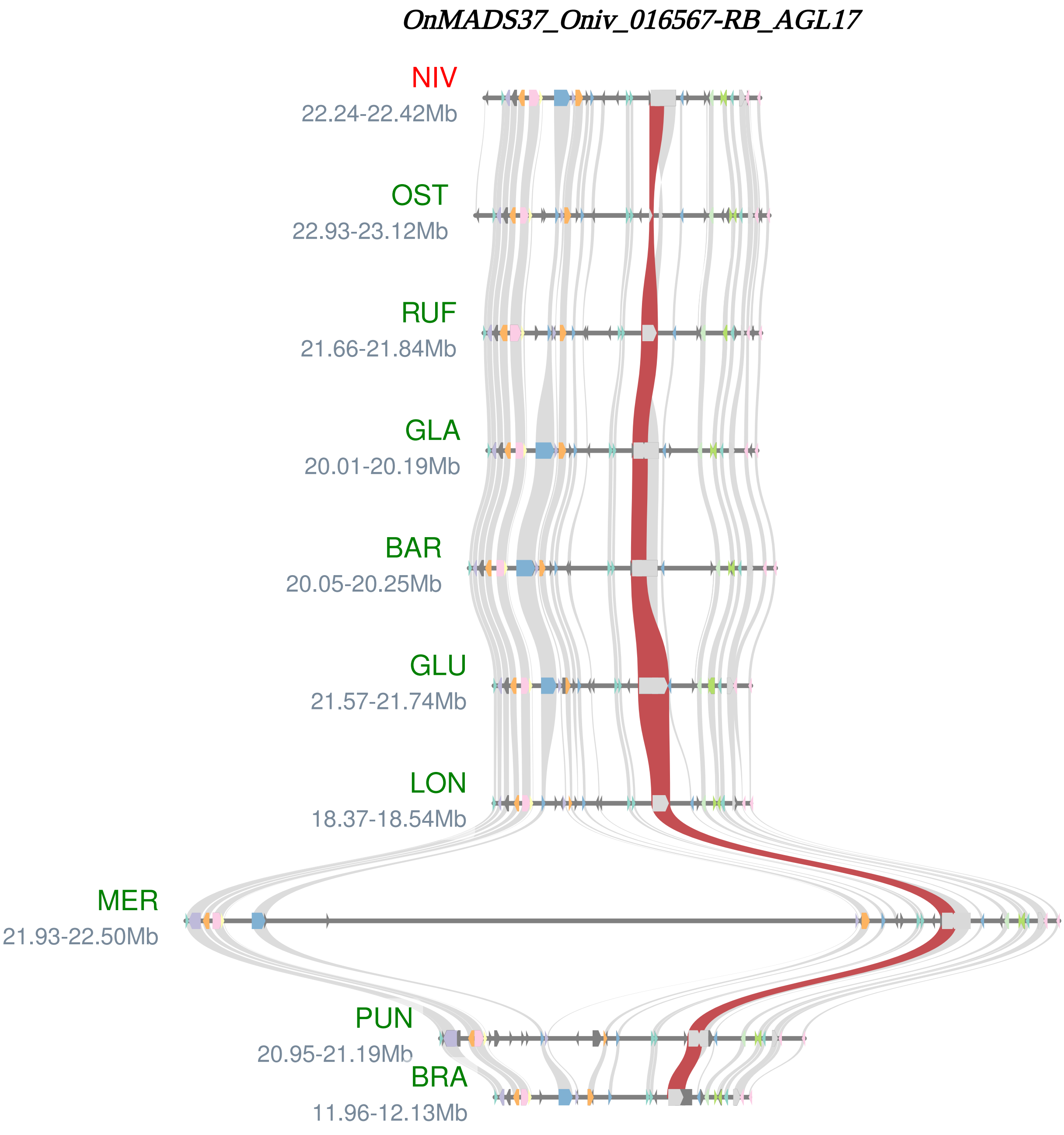
OnMADS37_Oniv_016567-RB_AGL17
NIV
22.24-22.42Mb
OST
22.93-23.12Mb
RUF
21.66-21.84Mb
GLA
20.01-20.19Mb
BAR
20.05-20.25Mb
GLU
21.57-21.74Mb
LON
18.37-18.54Mb
MER
21.93-22.50Mb
PUN
20.95-21.19Mb
BRA
11.96-12.13Mb

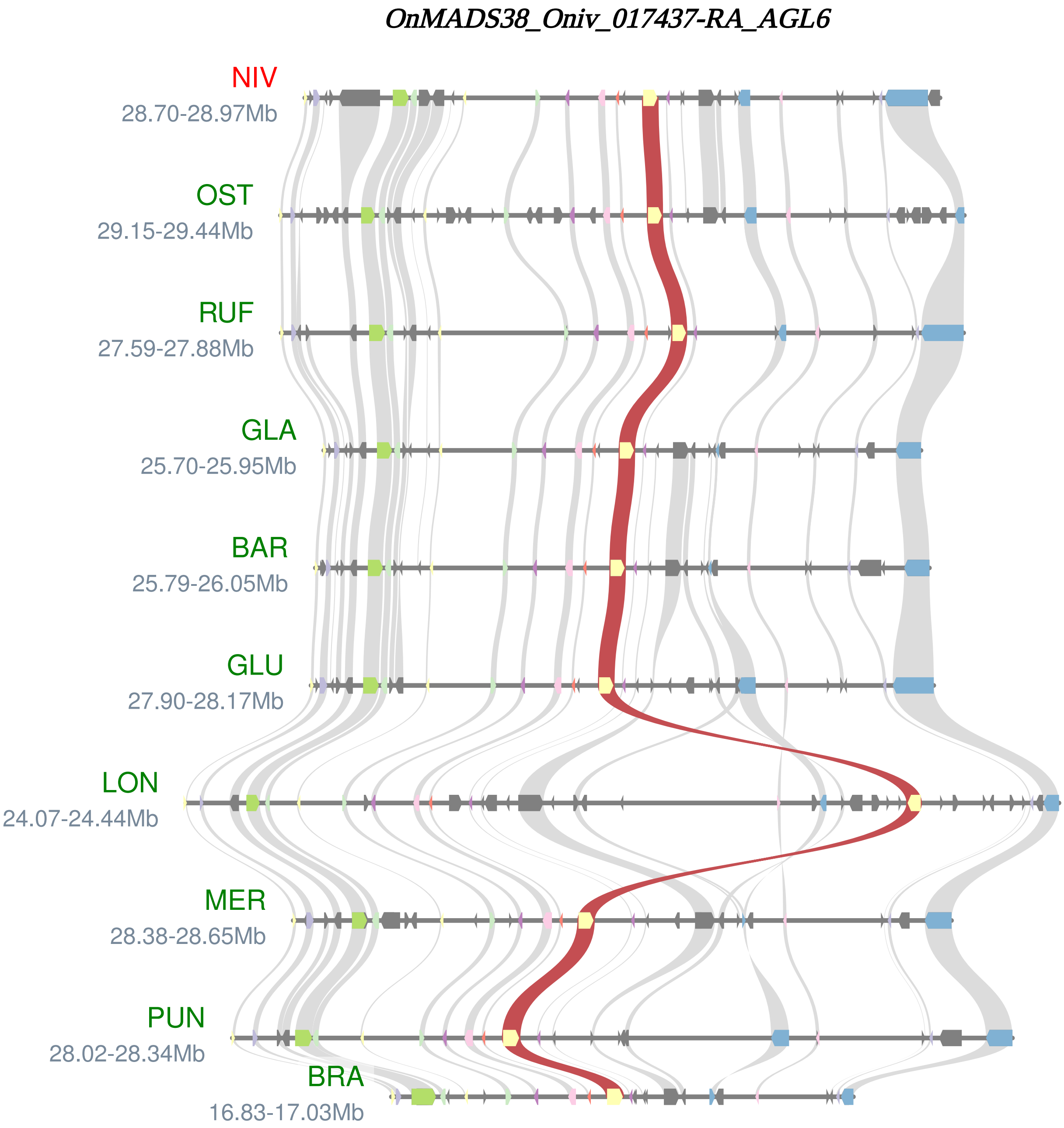
OnMADS38_Oniv_017437-RA_AGL6
NIV
28.70-28.97Mb
OST
29.15-29.44Mb
RUF
27.59-27.88Mb
GLA
25.70-25.95Mb
BAR
25.79-26.05Mb
GLU
27.90-28.17Mb
LON
24.07-24.44Mb
MER
28.38-28.65Mb
PUN
28.02-28.34Mb
BRA
16.83-17.03Mb

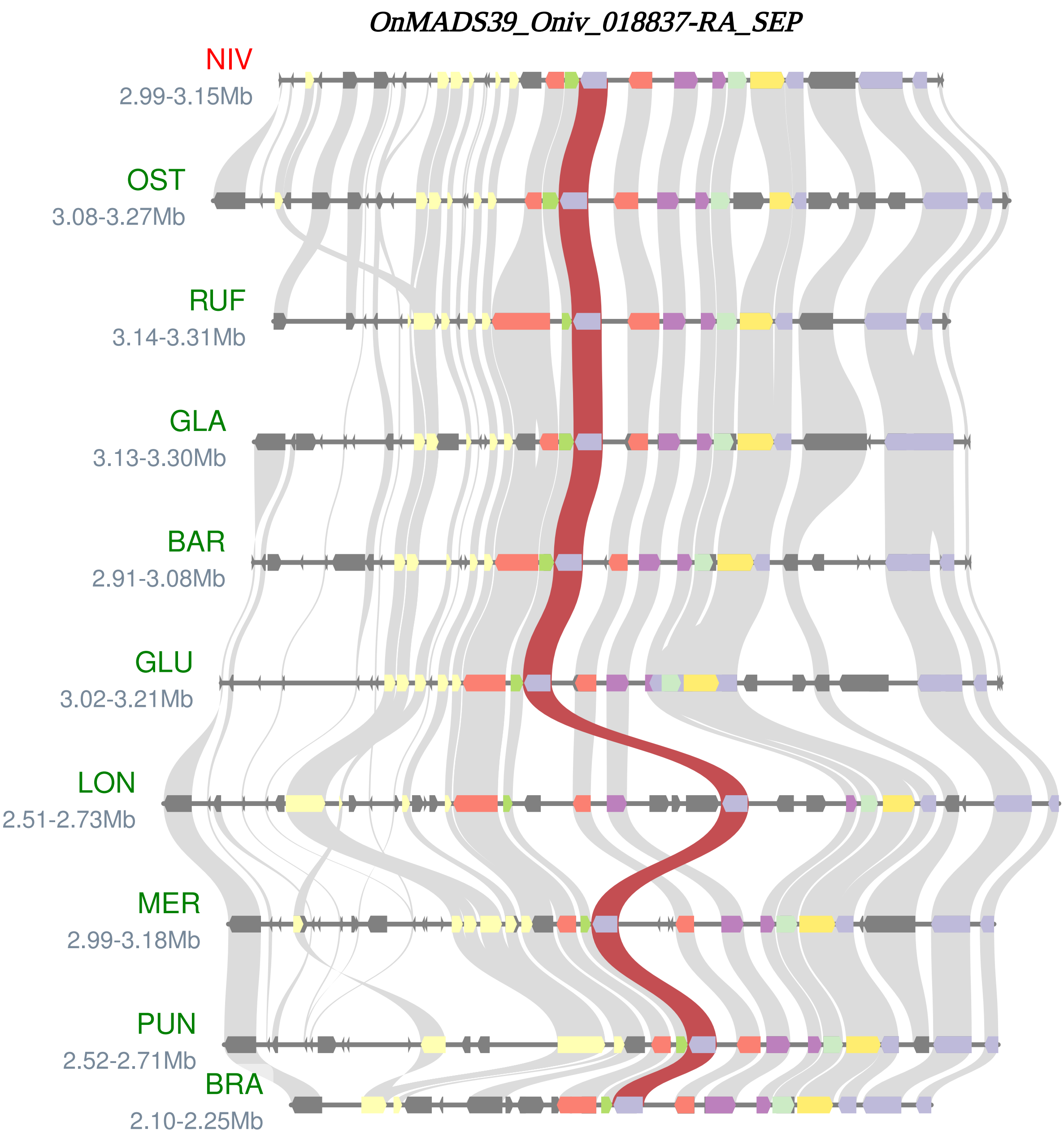
OnMADS39_Oniv_018837-RA_SEP
NIV
2.99-3.15Mb
OST
3.08-3.27Mb
RUF
3.14-3.31Mb
GLA
3.13-3.30Mb
BAR
2.91-3.08Mb
GLU
3.02-3.21Mb
LON
2.51-2.73Mb
MER
2.99-3.18Mb
PUN
2.52-2.71Mb
BRA
2.10-2.25Mb

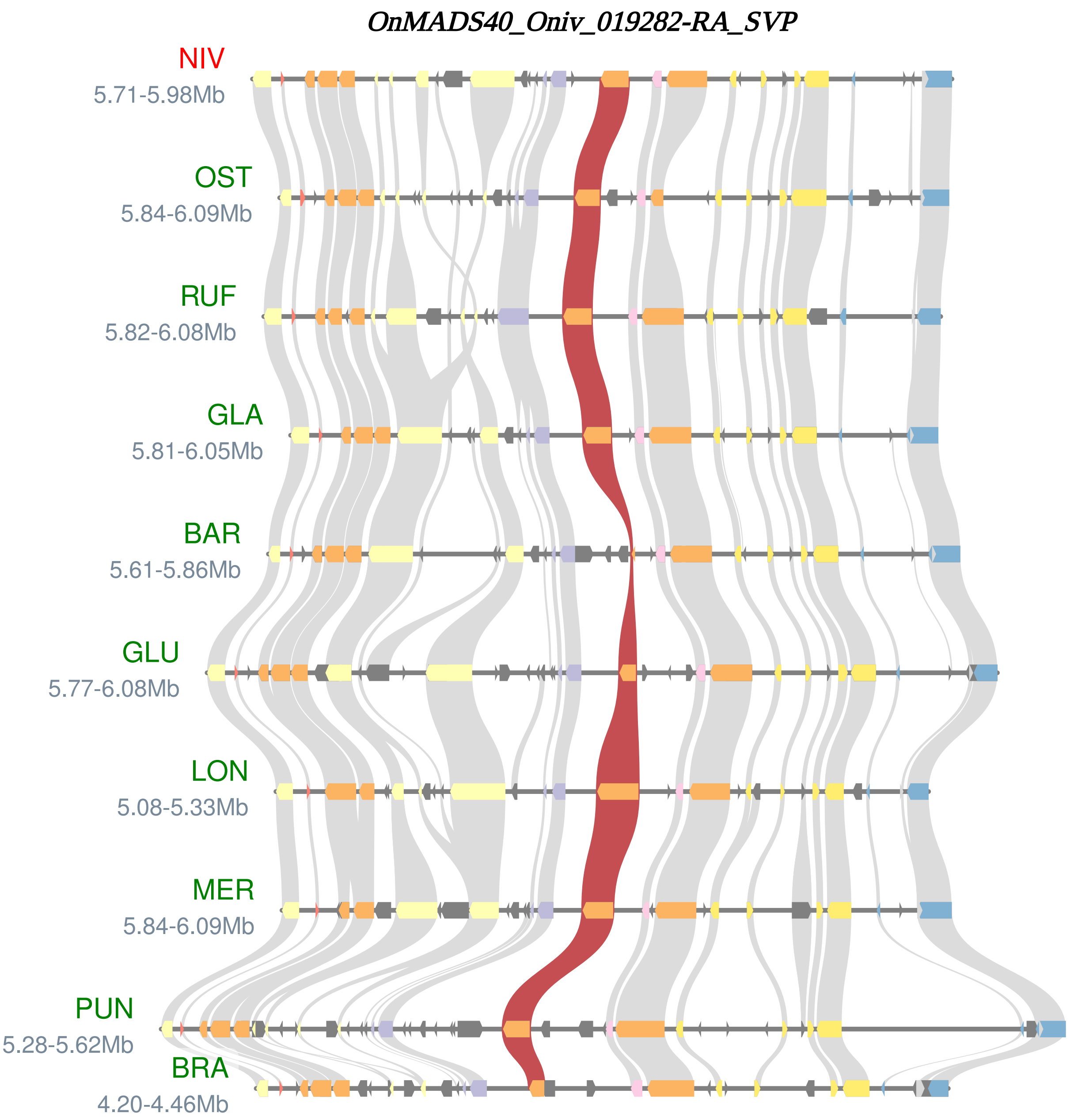

OnMADS40_Oniv_019282-RA_SVP
NIV
5.71-5.98Mb
OST
5.84-6.09Mb
RUF
5.82-6.08Mb
GLA
5.81-6.05Mb
BAR
5.61-5.86Mb
GLU
5.77-6.08Mb
LON
5.08-5.33Mb
MER
5.84-6.09Mb
PUN
5.28-5.62Mb
BRA
4.20-4.46Mb

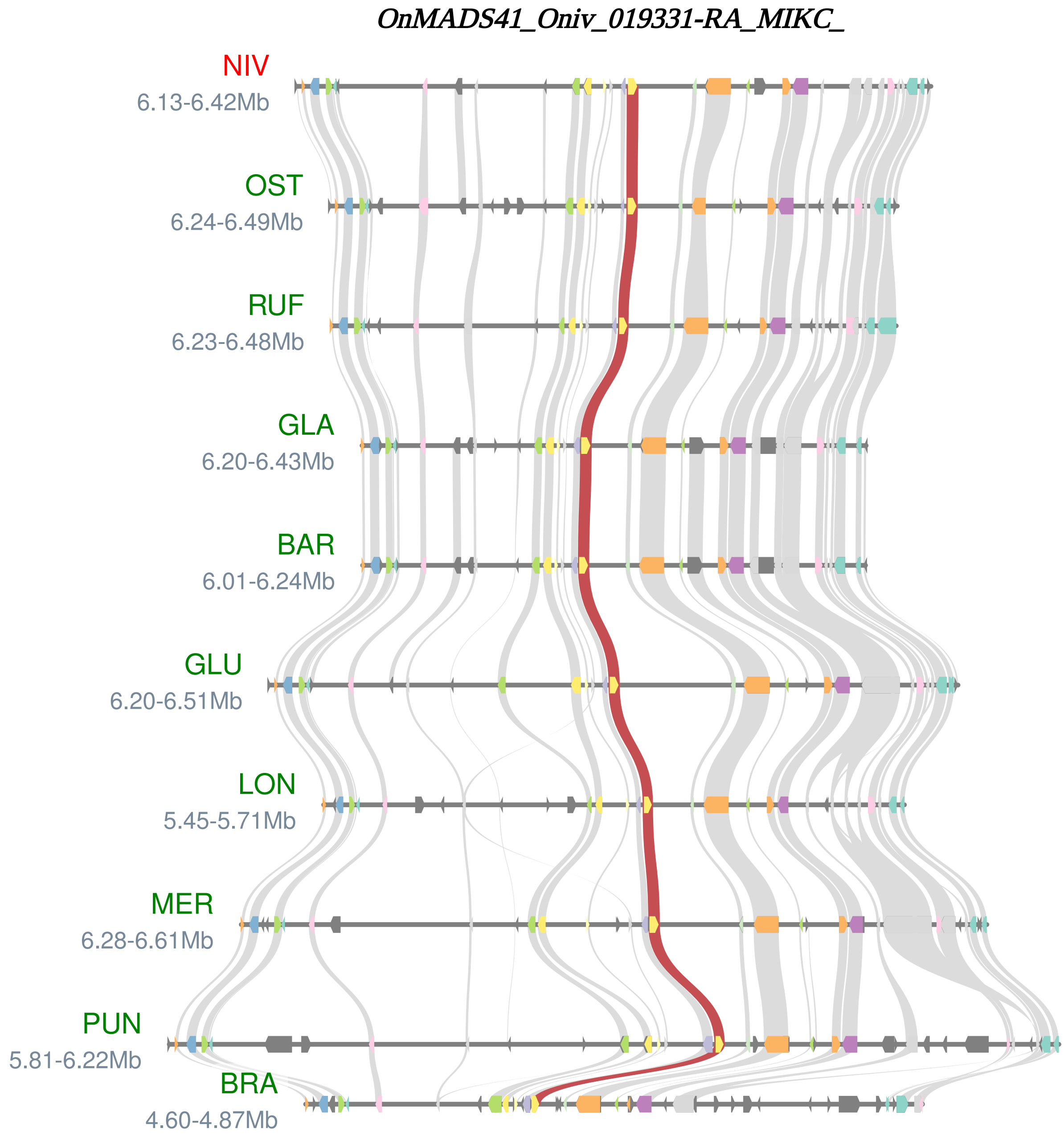
OnMADS41_Oniv_019331-RA_MIKC_
NIV
6.13-6.42Mb
OST
6.24-6.49Mb
RUF
6.23-6.48Mb
GLA
6.20-6.43Mb
BAR
6.01-6.24Mb
GLU
6.20-6.51Mb
LON
5.45-5.71Mb
MER
6.28-6.61Mb
PUN
5.81-6.22Mb
BRA
4.60-4.87Mb

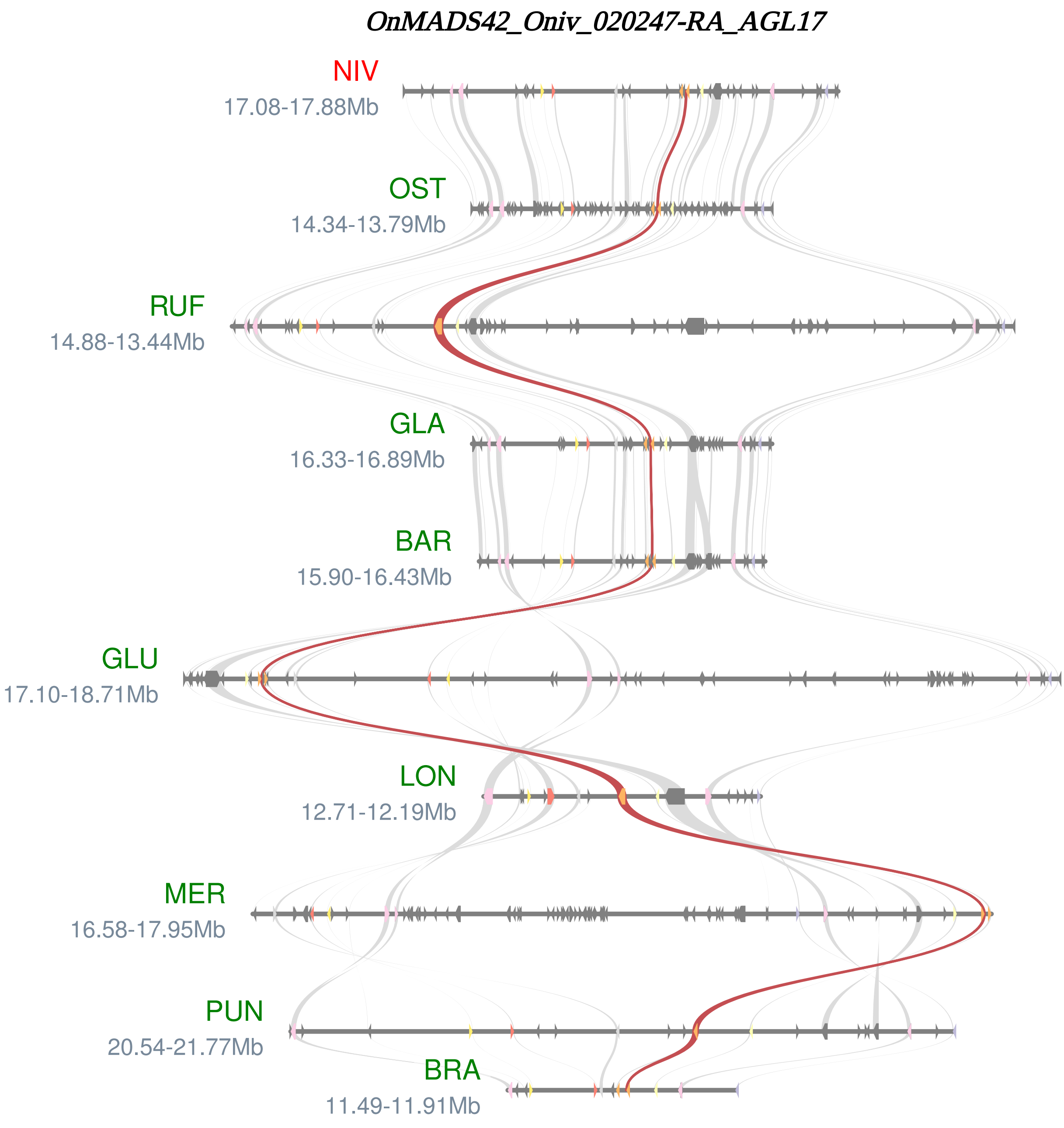
OnMADS42_Oniv_020247-RA_AGL17
NIV
17.08-17.88Mb
OST
14.34-13.79Mb
RUF
14.88-13.44Mb
GLA
16.33-16.89Mb
BAR
15.90-16.43Mb
GLU
17.10-18.71Mb
LON
12.71-12.19Mb
MER
16.58-17.95Mb
PUN
20.54-21.77Mb
BRA
11.49-11.91Mb

OnMADS43_Oniv_020329-RA_Mα

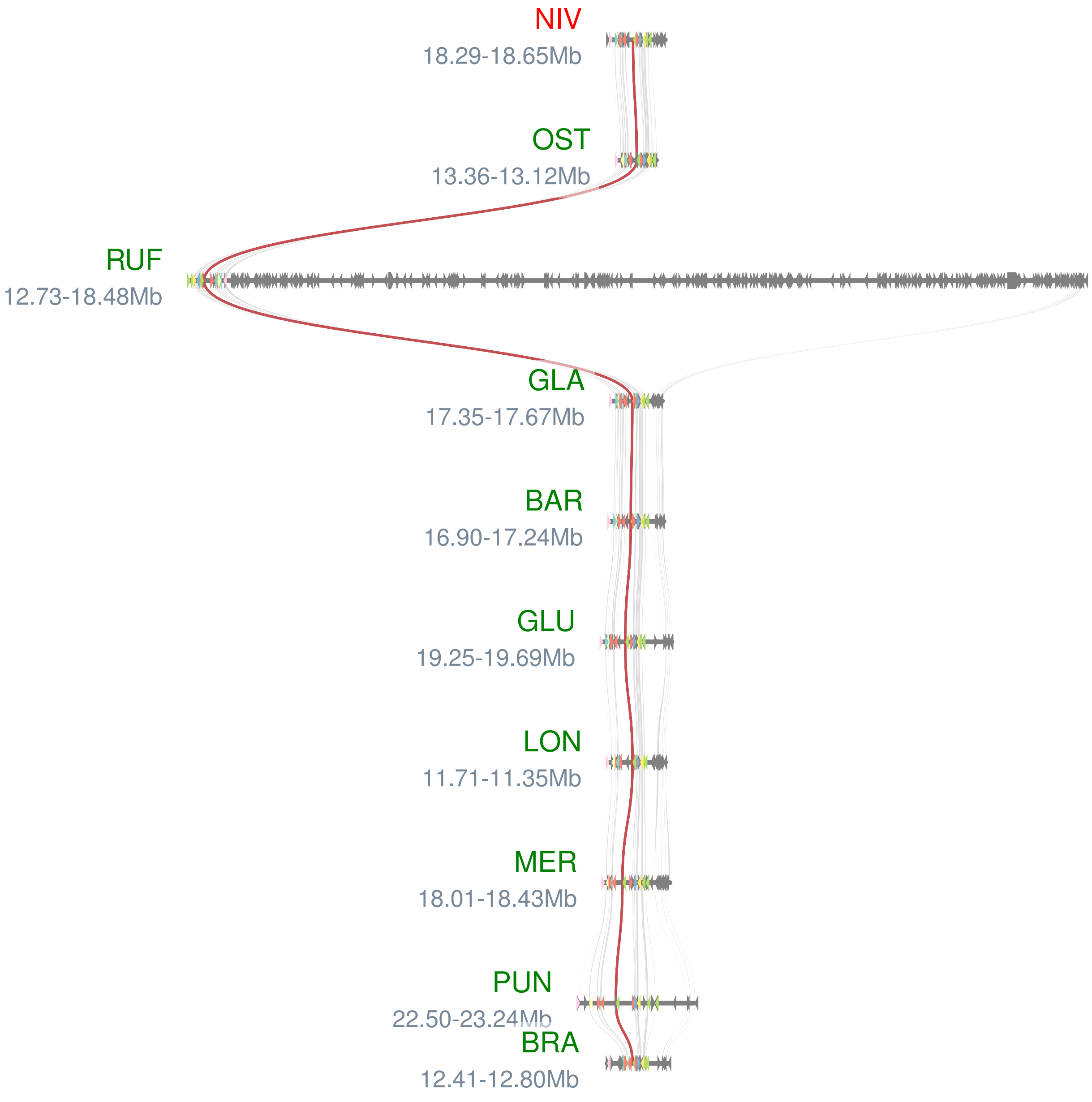

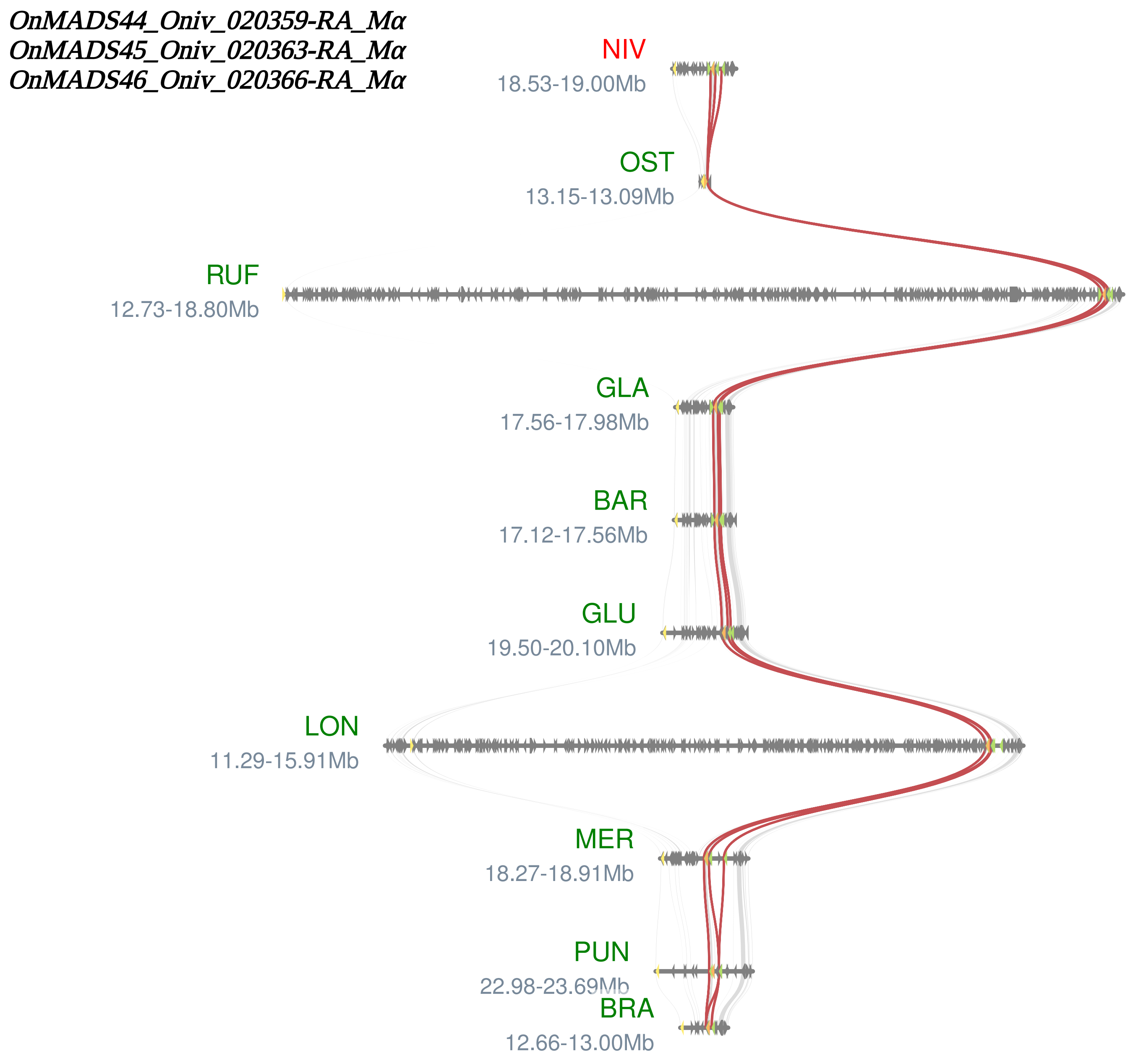
OnMADS44_Oniv_020359-RA_Mα
OnMADS45_Oniv_020363-RA_Mα
OnMADS46_Oniv_020366-RA_Mα
NIV
18.53-19.00Mb
OST
13.15-13.09Mb
RUF
12.73-18.80Mb
GLA
17.56-17.98Mb
BAR
17.12-17.56Mb
GLU
19.50-20.10Mb
LON
11.29-15.91Mb
MER
18.27-18.91Mb
PUN
22.98-23.69Mb
BRA
12.66-13.00Mb

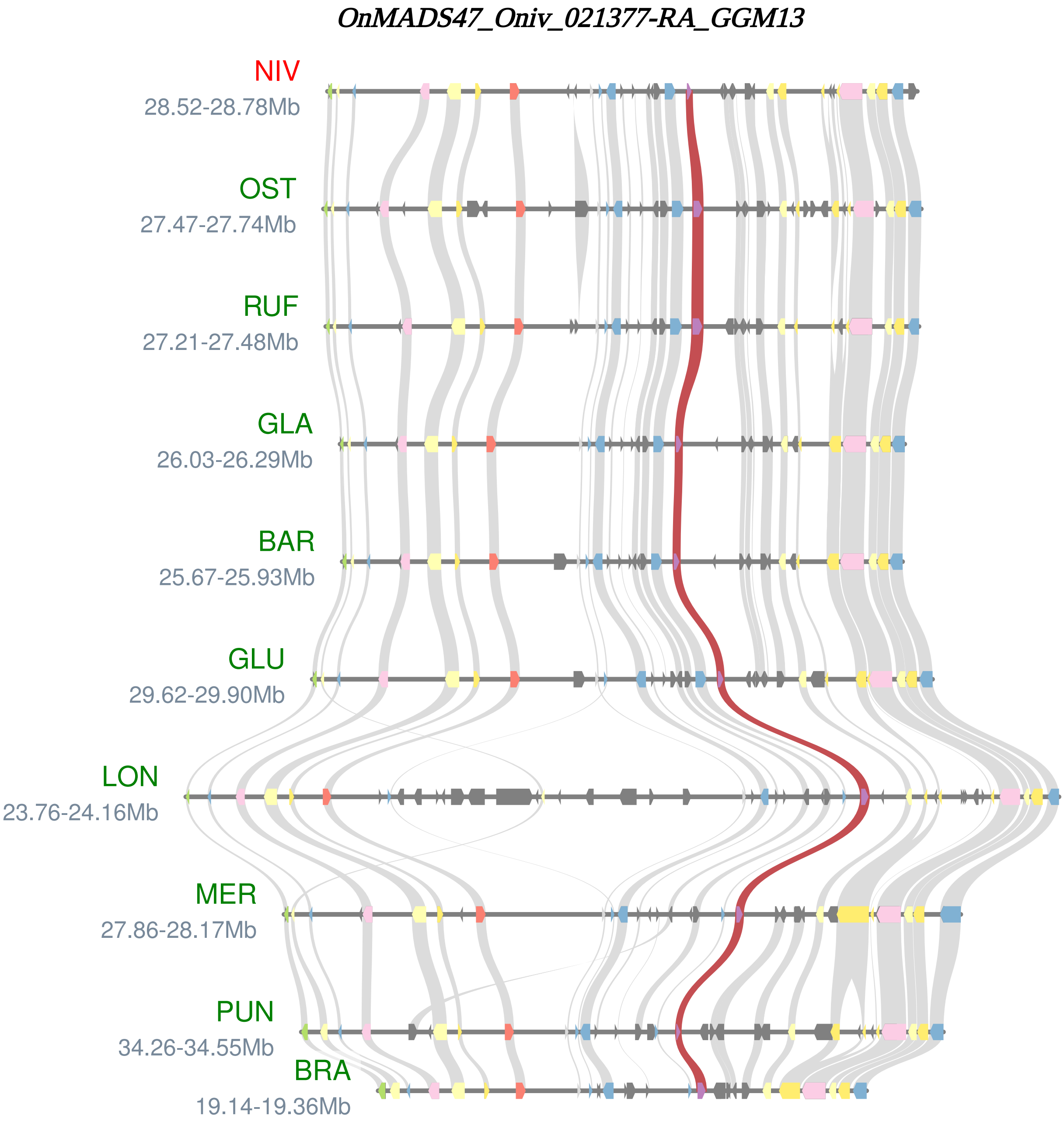
OnMADS47_Oniv_021377-RA_GGM13
NIV
28.52-28.78Mb
OST
27.47-27.74Mb
RUF
27.21-27.48Mb
GLA
26.03-26.29Mb
BAR
25.67-25.93Mb
GLU
29.62-29.90Mb
LON
23.76-24.16Mb
MER
27.86-28.17Mb
PUN
34.26-34.55Mb
BRA
19.14-19.36Mb

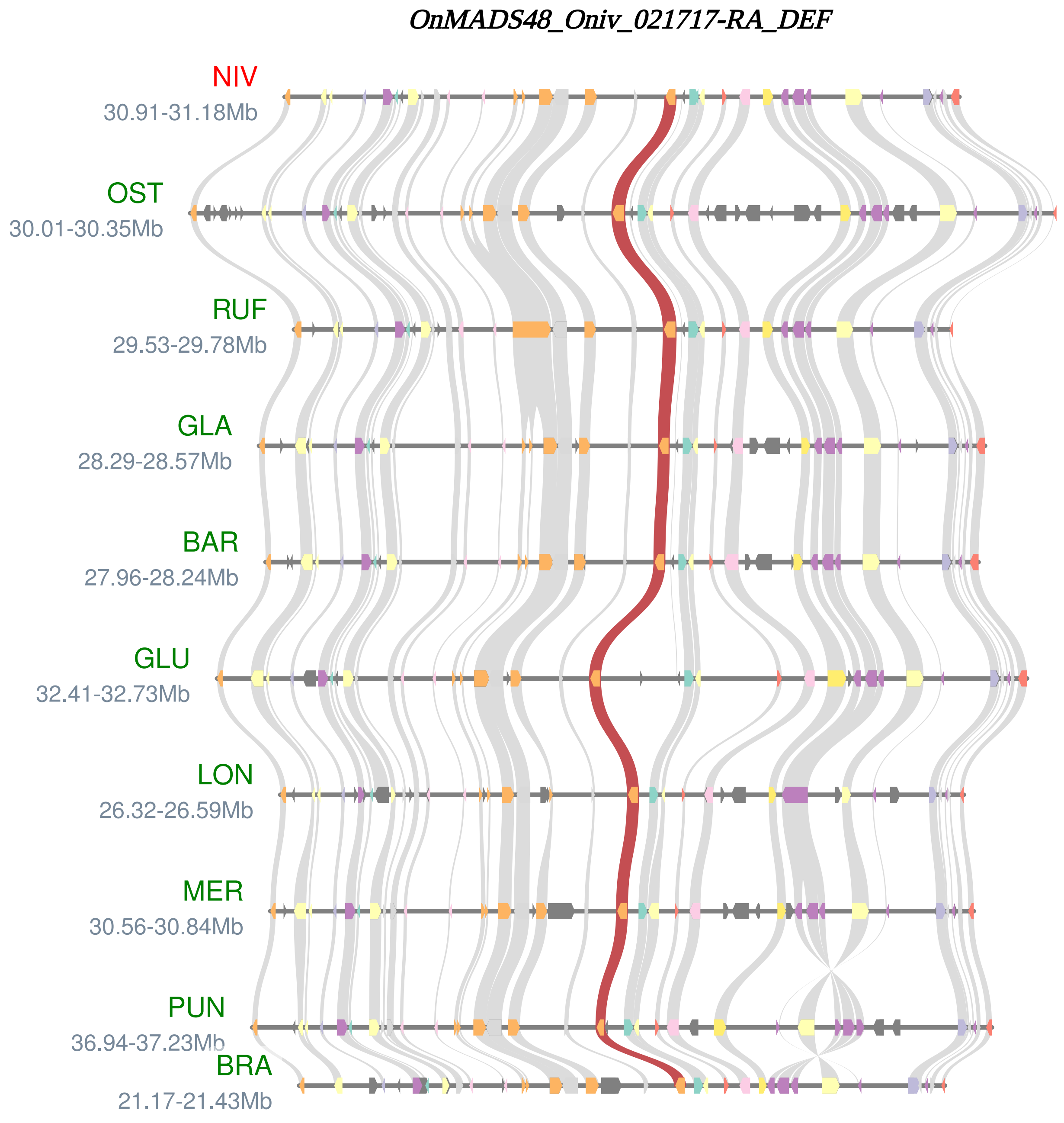
OnMADS48_Oniv_021717-RA_DEF
NIV
30.91-31.18Mb
OST
30.01-30.35Mb
RUF
29.53-29.78Mb
GLA
28.29-28.57Mb
BAR
27.96-28.24Mb
GLU
32.41-32.73Mb
LON
26.32-26.59Mb
MER
30.56-30.84Mb
PUN
36.94-37.23Mb
BRA
21.17-21.43Mb

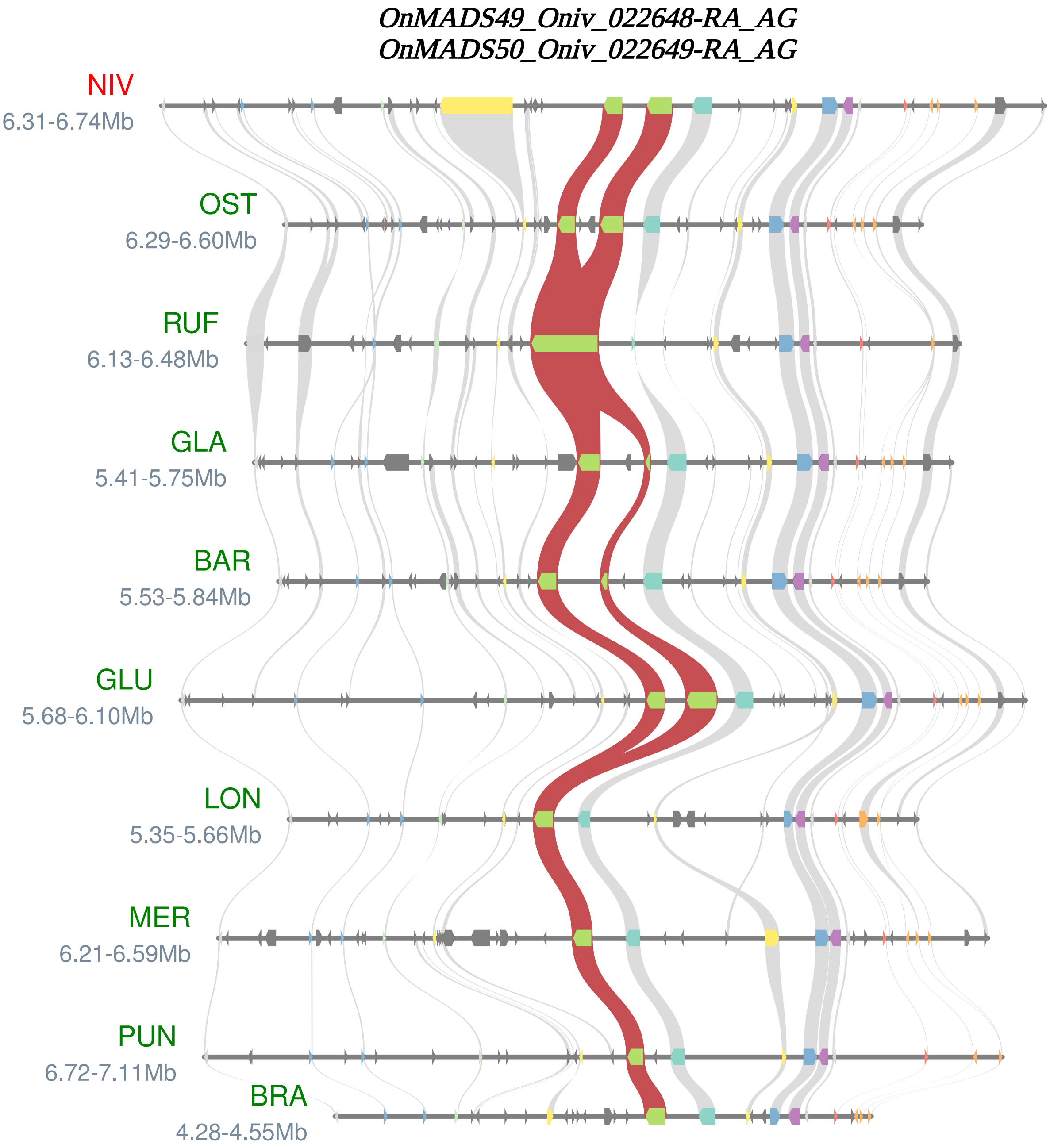
OnMADS49_Oniv_022648-RA_AG
OnMADS50_Oniv_022649-RA_AG
NIV
6.31-6.74Mb
OST
6.29-6.60Mb
RUF
6.13-6.48Mb
GLA
5.41-5.75Mb
BAR
5.53-5.84Mb
GLU
5.68-6.10Mb
LON
5.35-5.66Mb
MER
6.21-6.59Mb
PUN
6.72-7.11Mb
BRA
4.28-4.55Mb

OnMADS51_Oniv_023123-RA_Mα
(The chromosomal segment in the LON lacks any detected syntenic genes.)

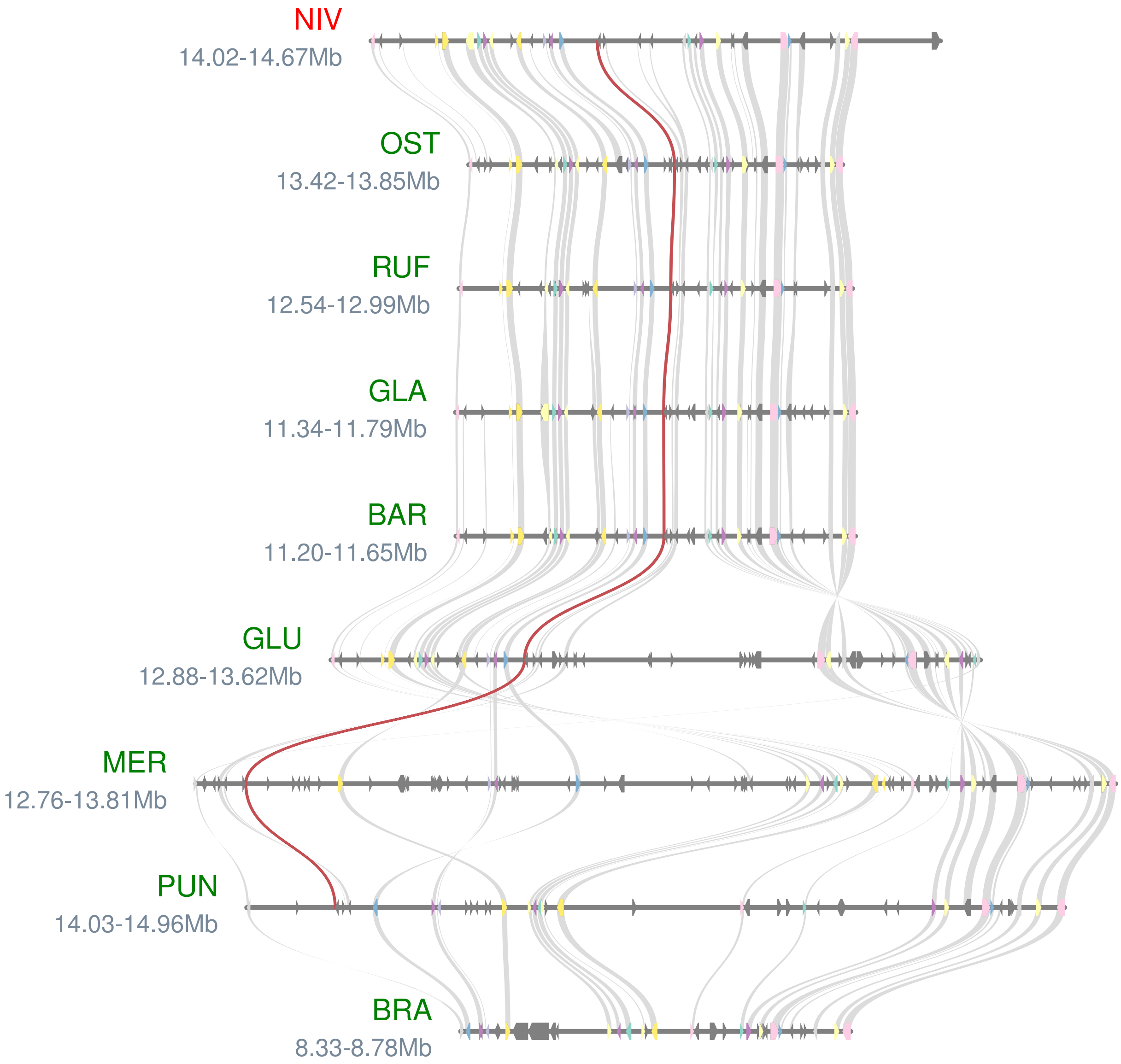

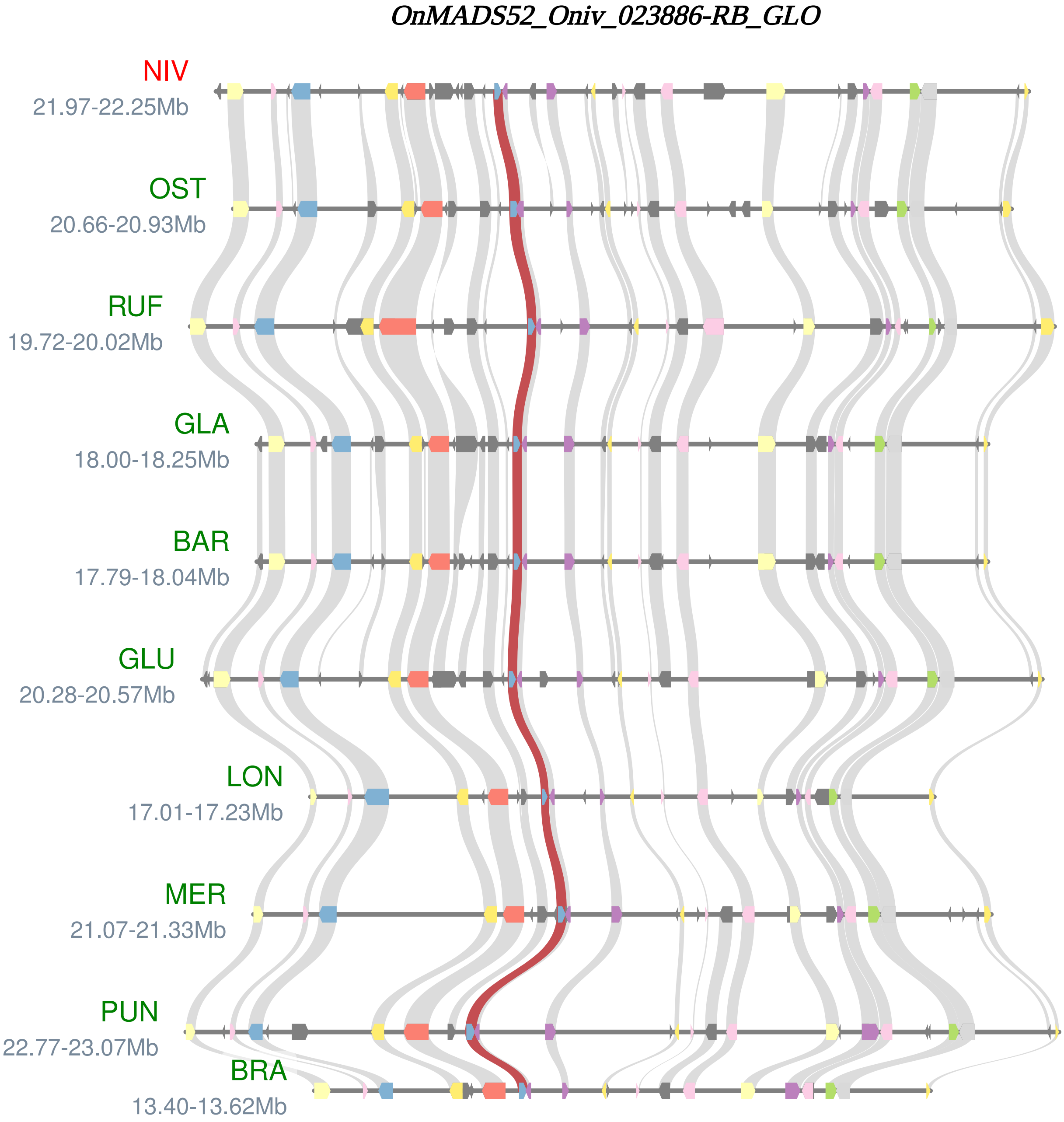

OnMADS52_Oniv_023886-RB_GLO
NIV
21.97-22.25Mb
OST
20.66-20.93Mb
RUF
19.72-20.02Mb
GLA
18.00-18.25Mb
BAR
17.79-18.04Mb
GLU
20.28-20.57Mb
LON
17.01-17.23Mb
MER
21.07-21.33Mb
PUN
22.77-23.07Mb
BRA
13.40-13.62Mb

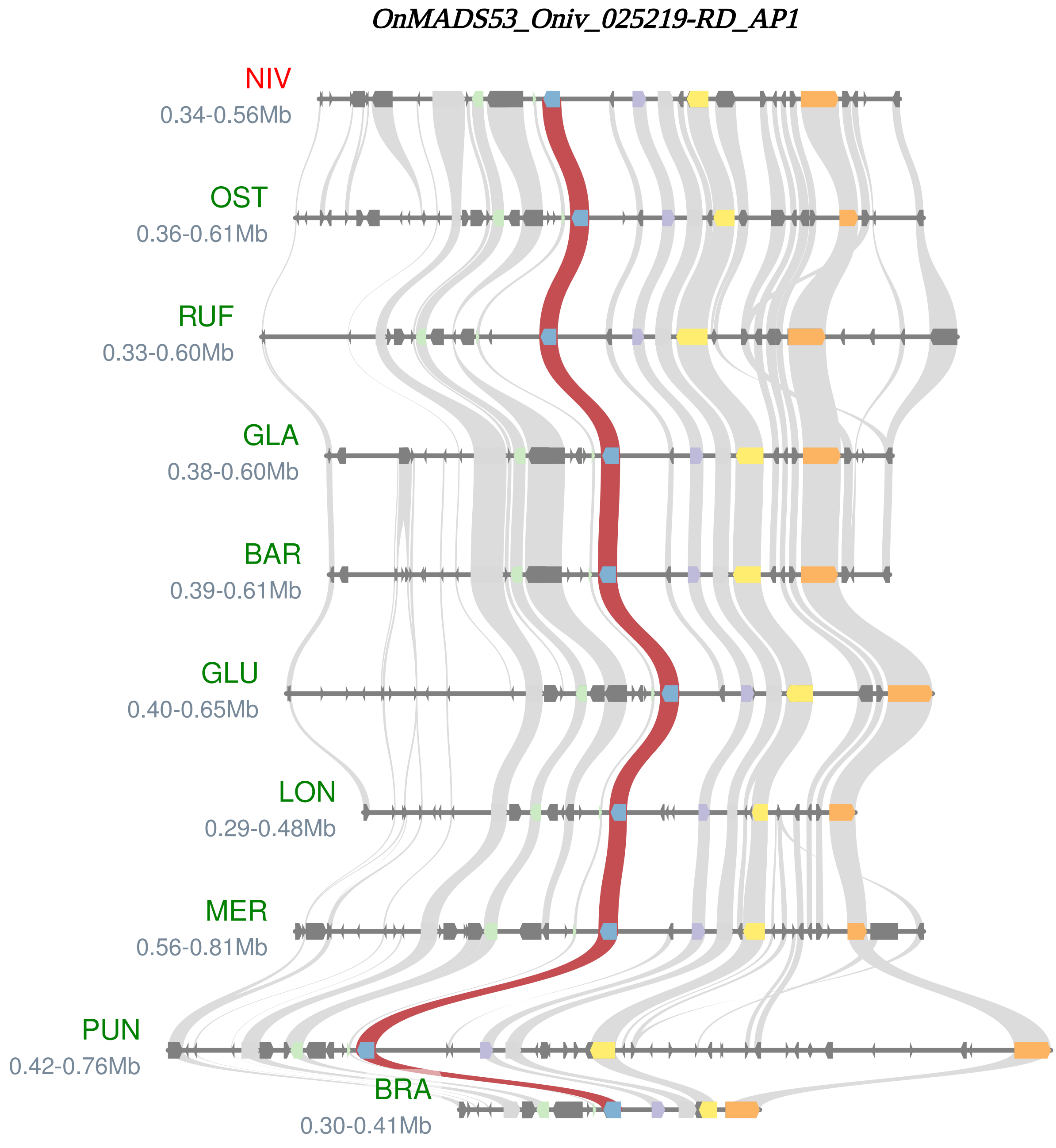

OnMADS53_Oniv_025219-RD_AP1
NIV
0.34-0.56Mb
OST
0.36-0.61Mb
RUF
0.33-0.60Mb
GLA
0.38-0.60Mb
BAR
0.39-0.61Mb
GLU
0.40-0.65Mb
LON
0.29-0.48Mb
MER
0.56-0.81Mb
PUN
0.42-0.76Mb
BRA
0.30-0.41Mb

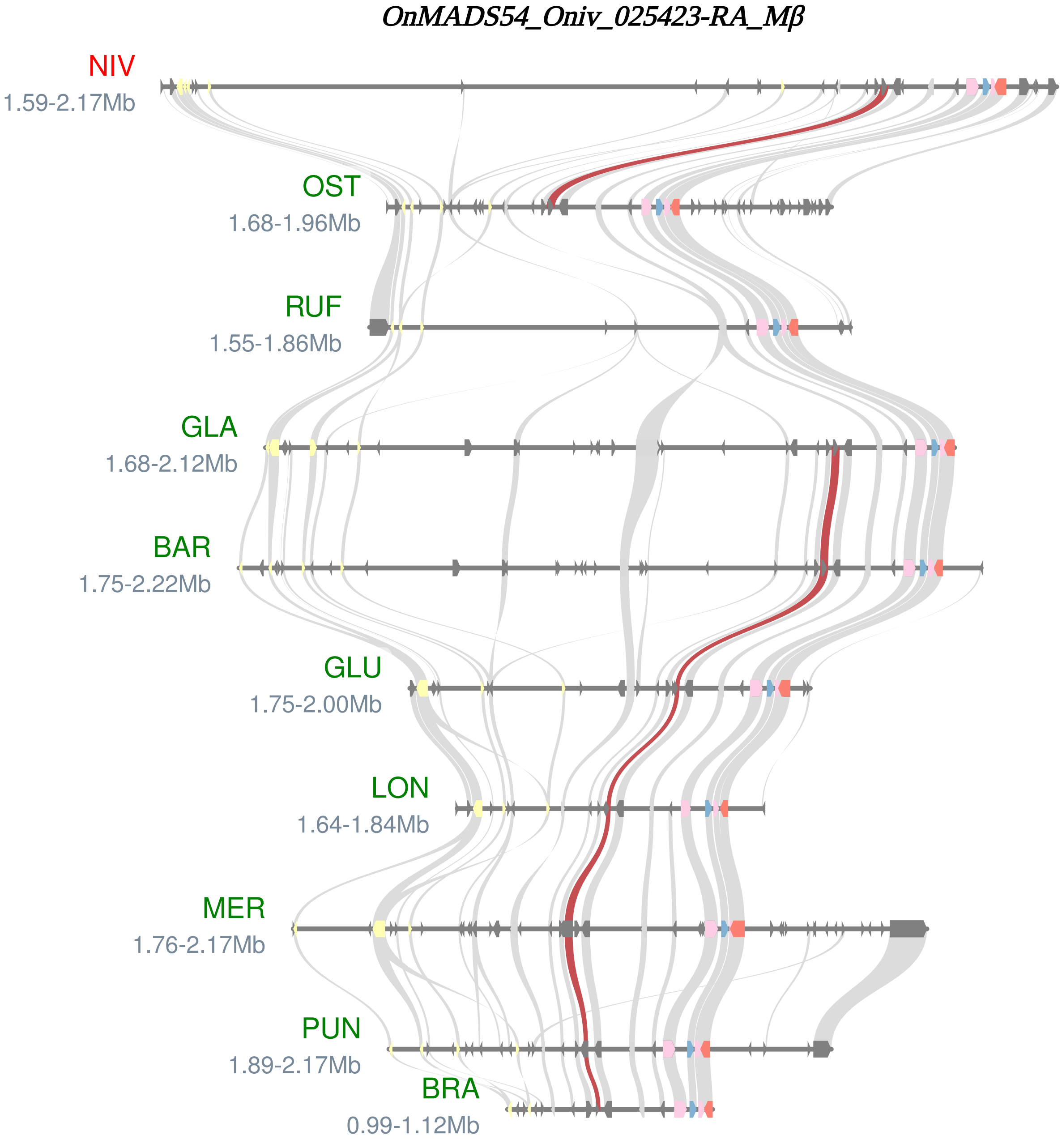
OnMADS54_Oniv_025423-RA_Mβ
NIV
1.59-2.17Mb
OST
1.68-1.96Mb
RUF
1.55-1.86Mb
GLA
1.68-2.12Mb
BAR
1.75-2.22Mb
GLU
1.75-2.00Mb
LON
1.64-1.84Mb
MER
1.76-2.17Mb
PUN
1.89-2.17Mb
BRA
0.99-1.12Mb

OnMADS55_Oniv_027738-RA_AP1
(The chromosomal segment in the LON lacks any detected syntenic genes.)

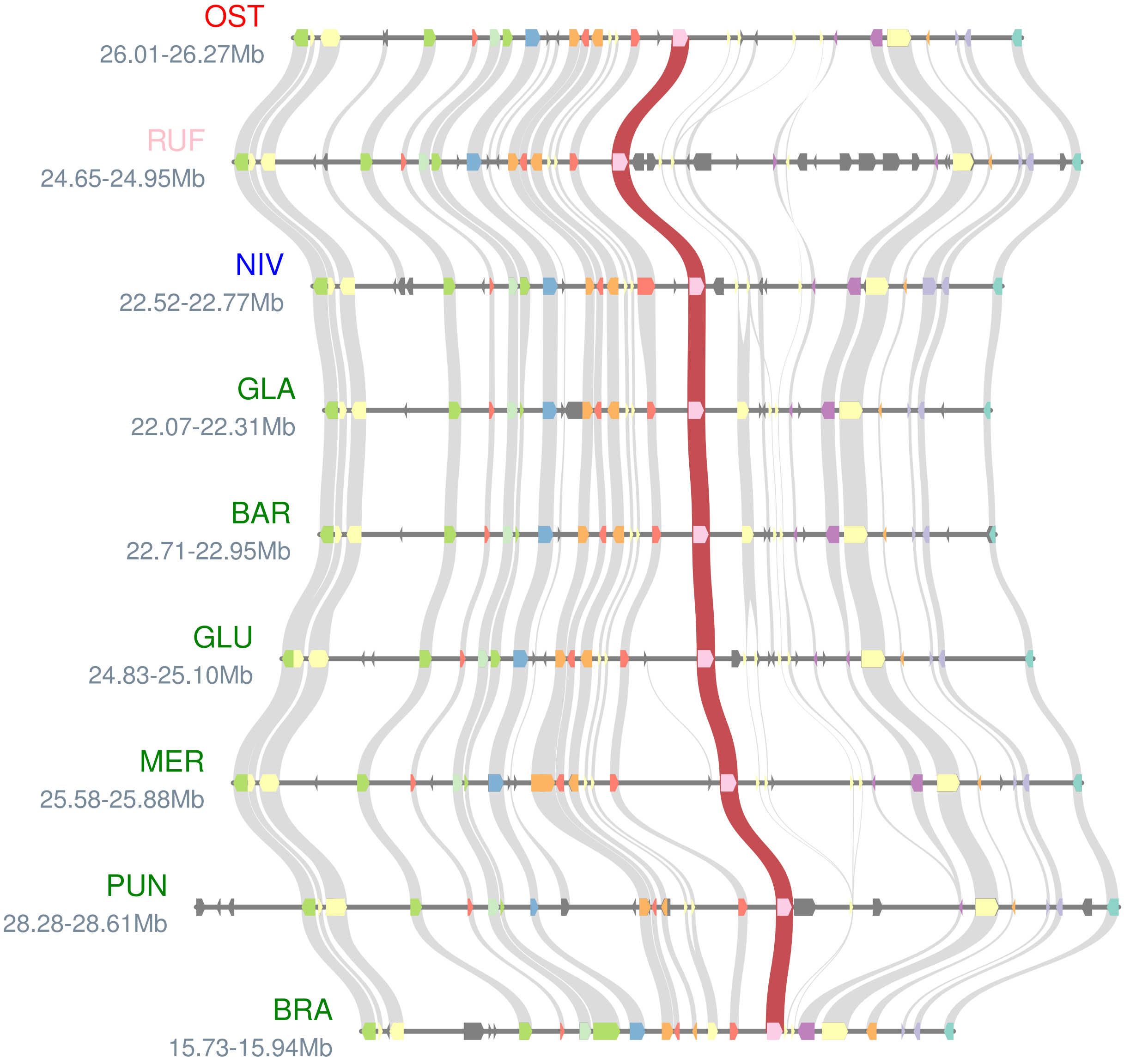

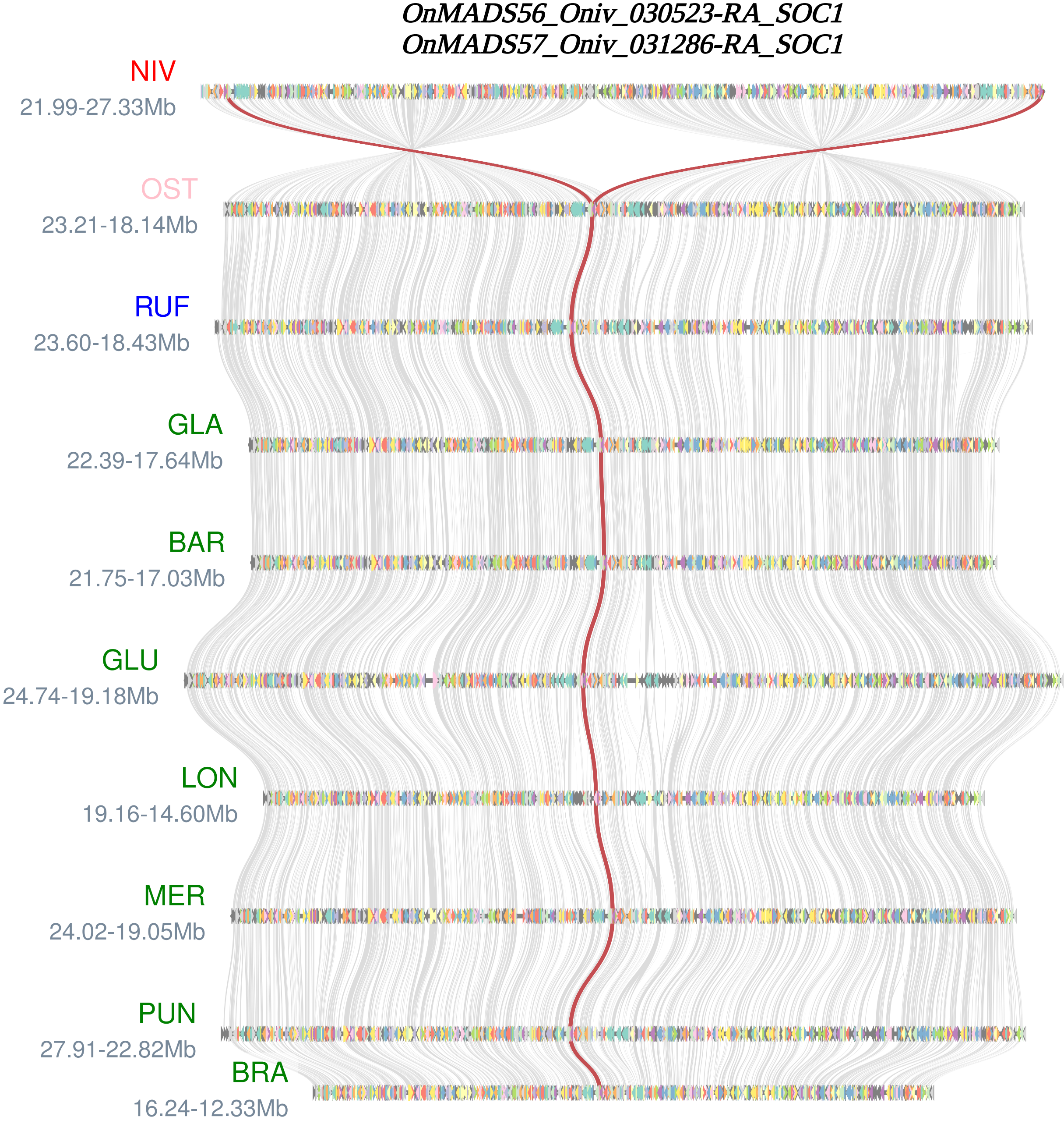
OnMADS56_Oniv_030523-RA_SOC1
OnMADS57_Oniv_031286-RA_SOC1
NIV
21.99-27.33Mb
OST
23.21-18.14Mb
RUF
23.60-18.43Mb
GLA
22.39-17.64Mb
BAR
21.75-17.03Mb
GLU
24.74-19.18Mb
LON
19.16-14.60Mb
MER
24.02-19.05Mb
PUN
27.91-22.82Mb
BRA
16.24-12.33Mb

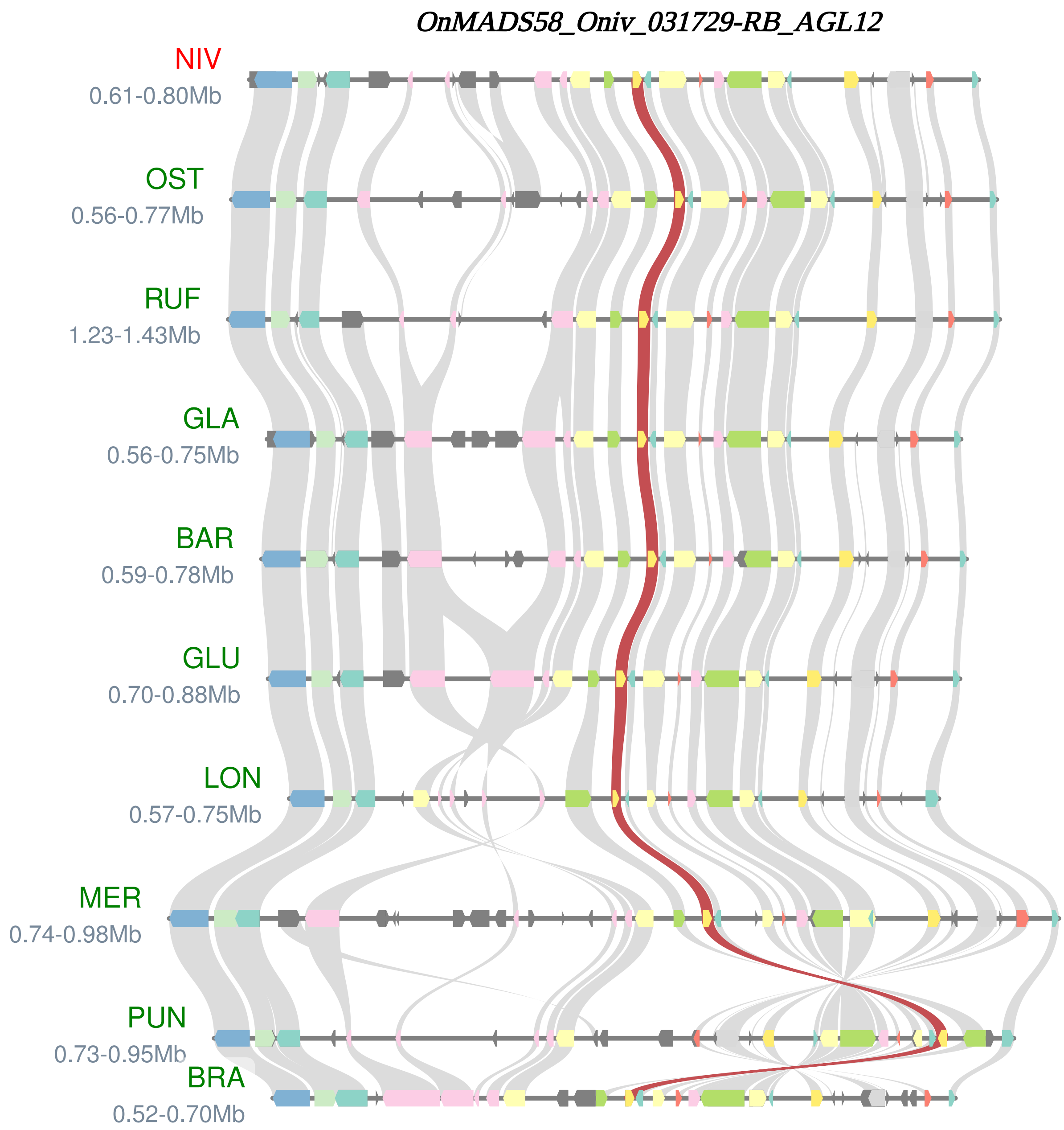
OnMADS58_Oniv_031729-RB_AGL12
NIV
0.61-0.80Mb
OST
0.56-0.77Mb
RUF
1.23-1.43Mb
GLA
0.56-0.75Mb
BAR
0.59-0.78Mb
GLU
0.70-0.88Mb
LON
0.57-0.75Mb
MER
0.74-0.98Mb
PUN
0.73-0.95Mb
BRA
0.52-0.70Mb

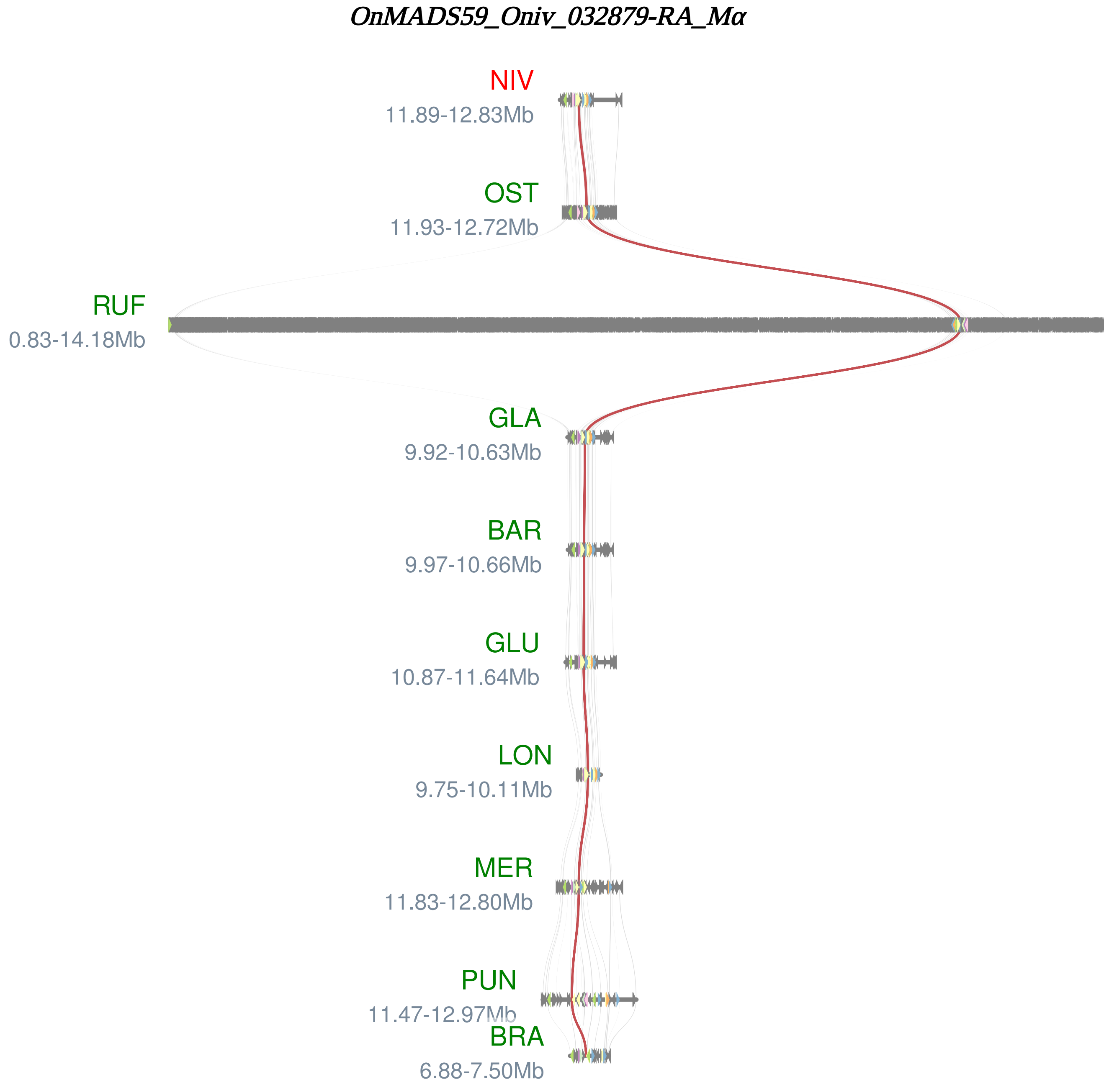
OnMADS59_Oniv_032879-RA_Mα
NIV
11.89-12.83Mb
OST
11.93-12.72Mb
RUF
0.83-14.18Mb
GLA
9.92-10.63Mb
BAR
9.97-10.66Mb
GLU
10.87-11.64Mb
LON
9.75-10.11Mb
MER
11.83-12.80Mb
PUN
11.47-12.97Mb
BRA
6.88-7.50Mb

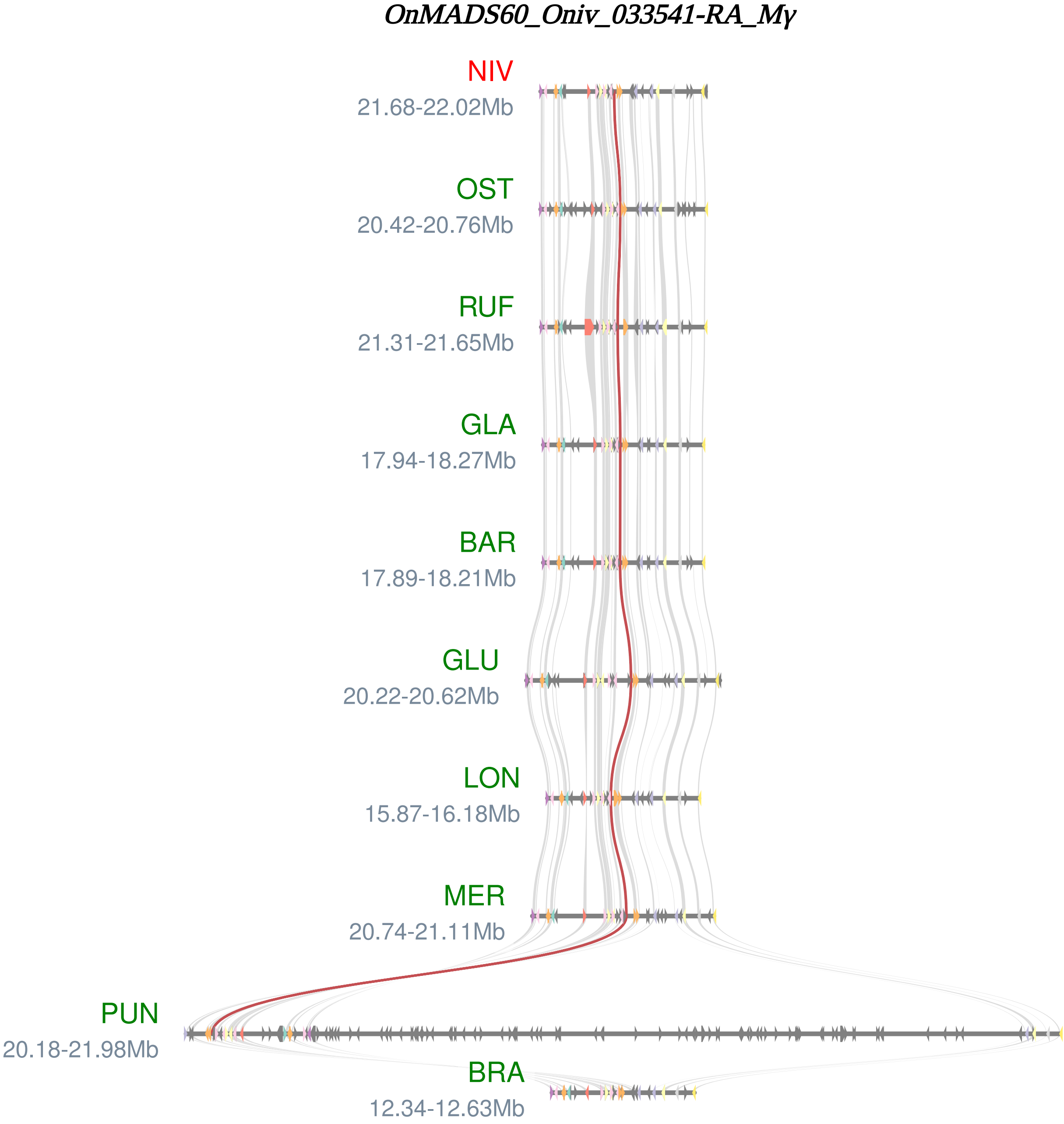
OnMADS60_Oniv_033541-RA_Mγ
NIV
21.68-22.02Mb
OST
20.42-20.76Mb
RUF
21.31-21.65Mb
GLA
17.94-18.27Mb
BAR
17.89-18.21Mb
GLU
20.22-20.62Mb
LON
15.87-16.18Mb
MER
20.74-21.11Mb
PUN
20.18-21.98Mb
BRA
12.34-12.63Mb

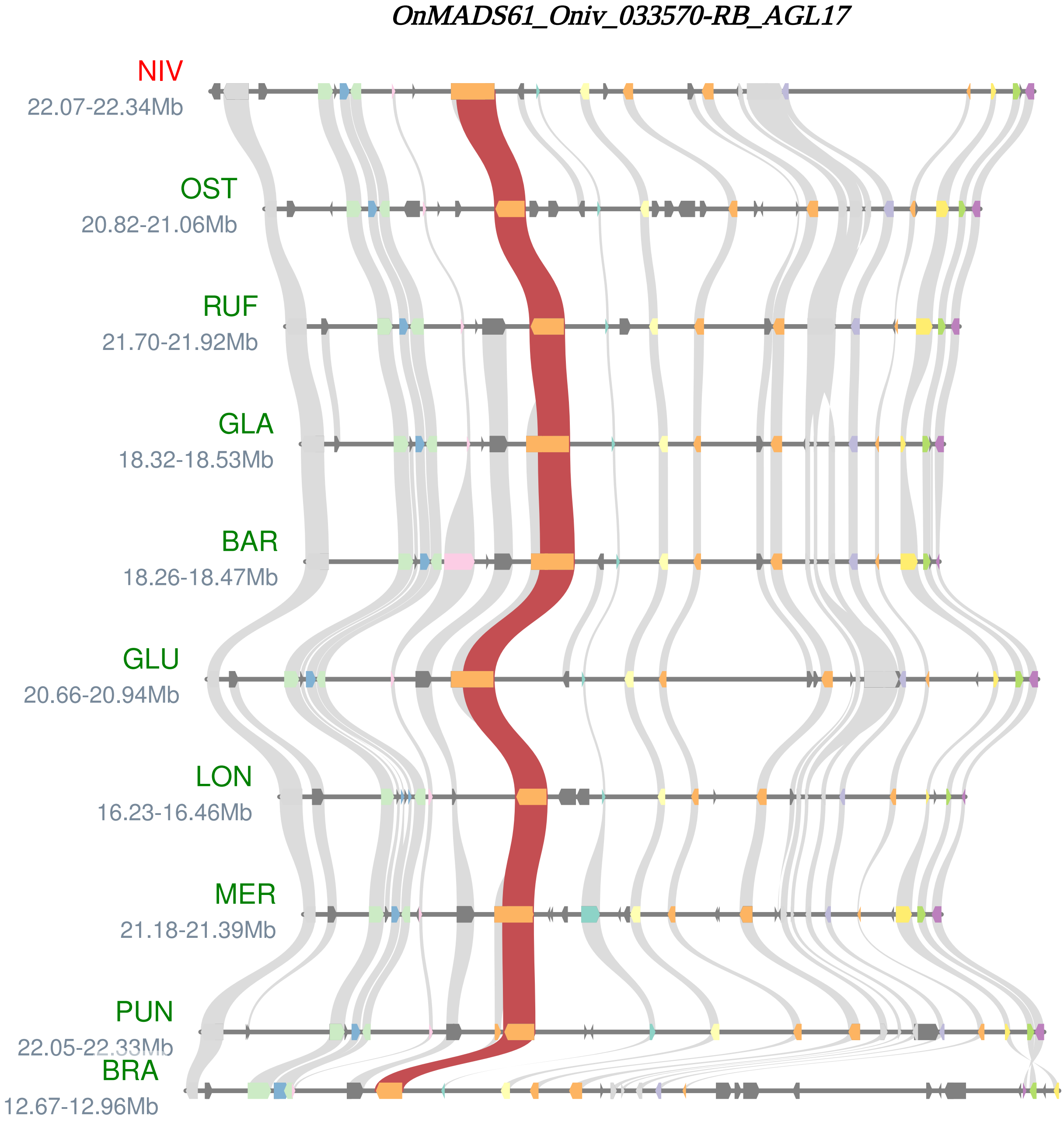

OnMADS61_Oniv_033570-RB_AGL17
NIV
22.07-22.34Mb
OST
20.82-21.06Mb
RUF
21.70-21.92Mb
GLA
18.32-18.53Mb
BAR
18.26-18.47Mb
GLU
20.66-20.94Mb
LON
16.23-16.46Mb
MER
21.18-21.39Mb
PUN
22.05-22.33Mb
BRA
12.67-12.96Mb

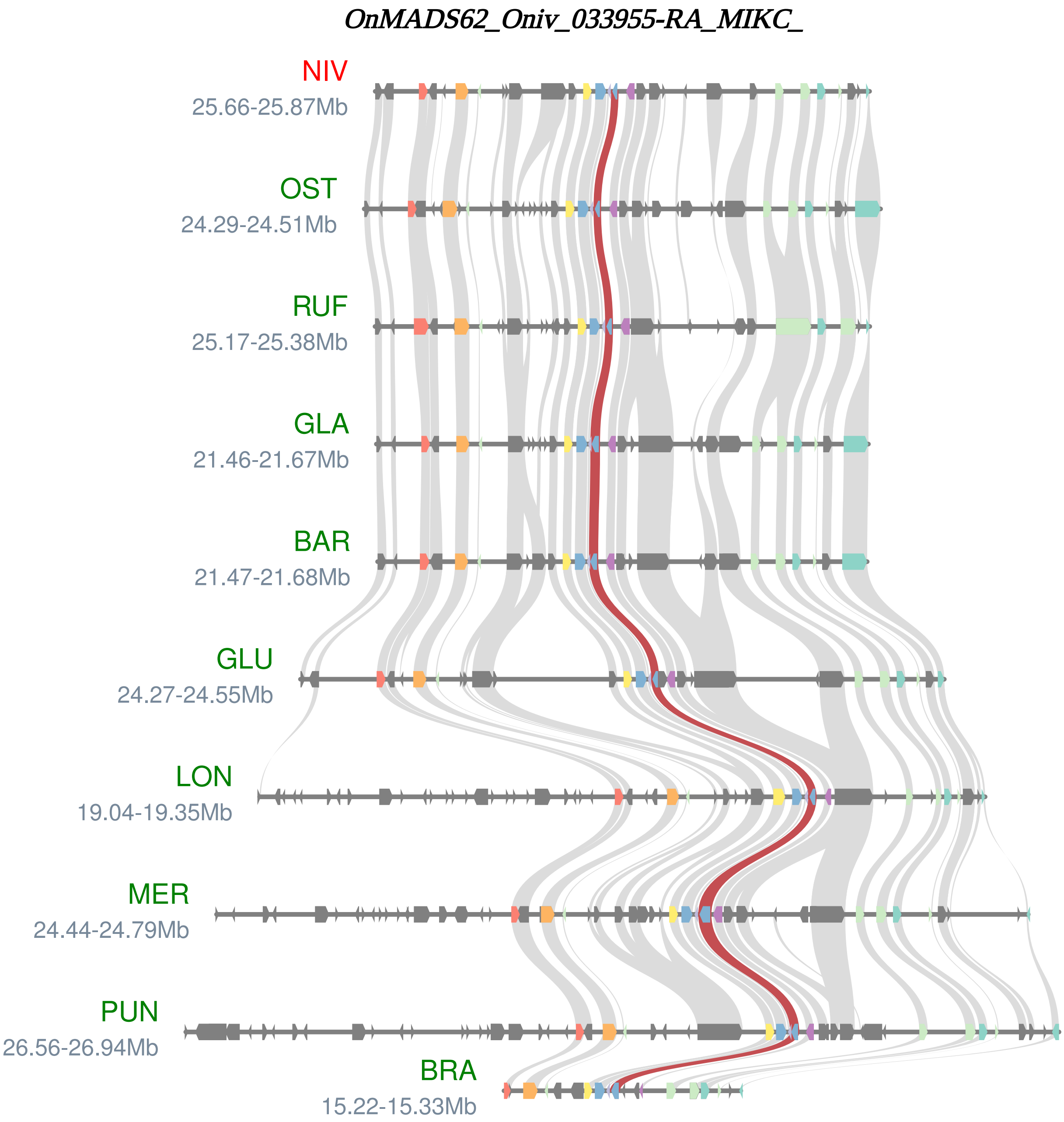

OnMADS62_Oniv_033955-RA_MIKC_
NIV
25.66-25.87Mb
OST
24.29-24.51Mb
RUF
25.17-25.38Mb
GLA
21.46-21.67Mb
BAR
21.47-21.68Mb
GLU
24.27-24.55Mb
LON
19.04-19.35Mb
MER
24.44-24.79Mb
PUN
26.56-26.94Mb
BRA
15.22-15.33Mb

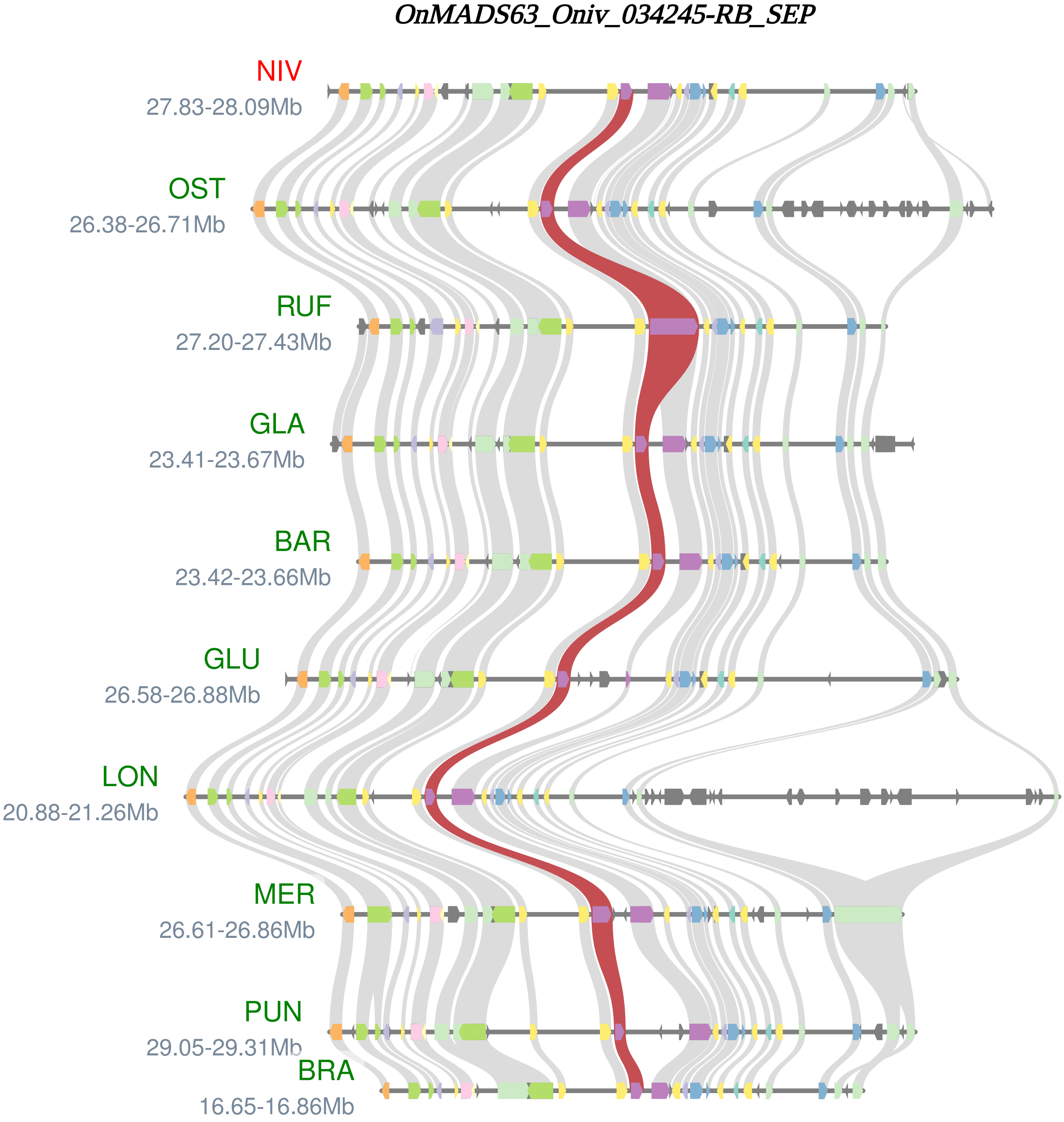

OnMADS63_Oniv_034245-RB_SEP
NIV
27.83-28.09Mb
OST
26.38-26.71Mb
RUF
27.20-27.43Mb
GLA
23.41-23.67Mb
BAR
23.42-23.66Mb
GLU
26.58-26.88Mb
LON
20.88-21.26Mb
MER
26.61-26.86Mb
PUN
29.05-29.31Mb
BRA
16.65-16.86Mb

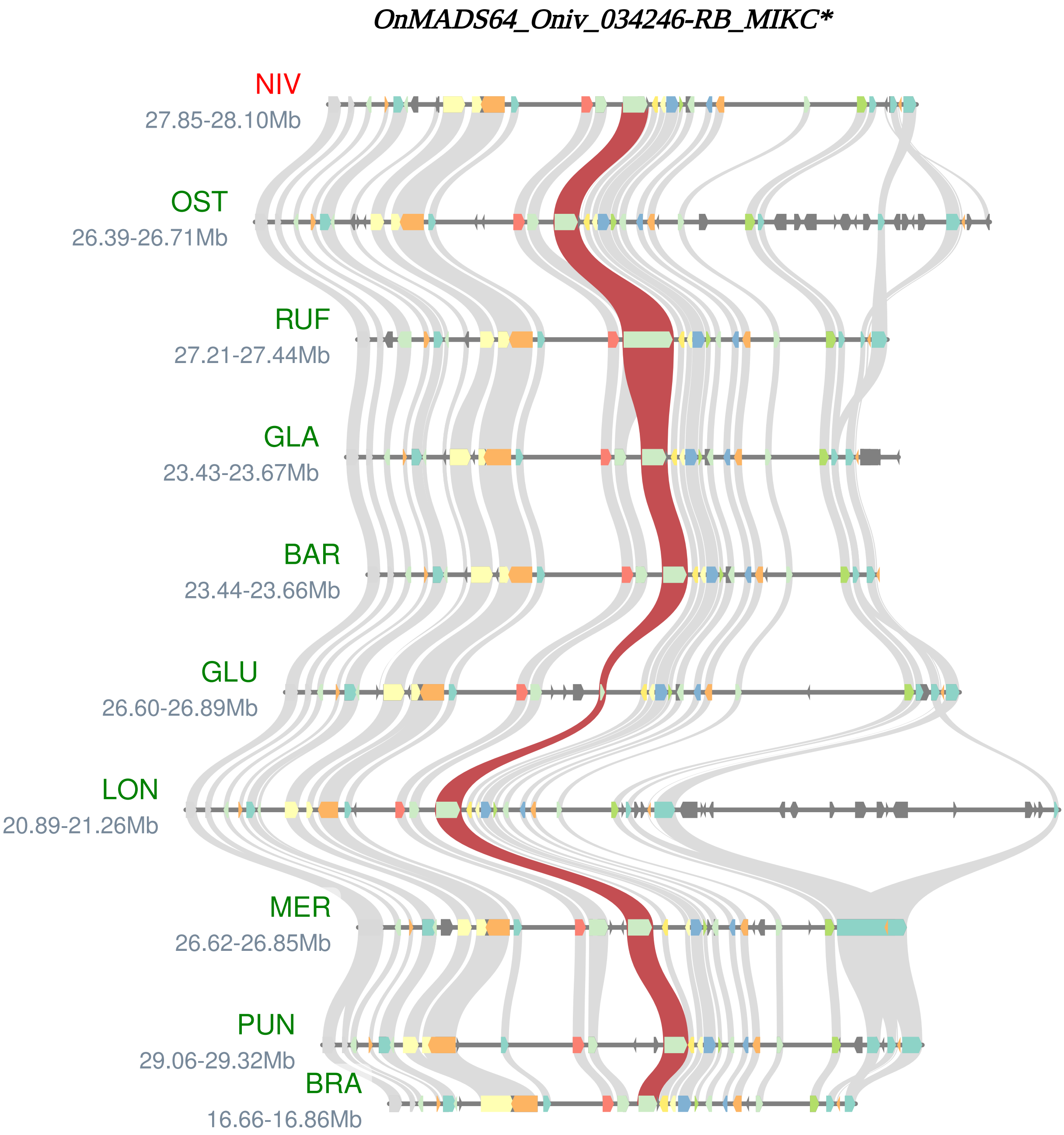
OnMADS64_Oniv_034246-RB_MIKC*
NIV
27.85-28.10Mb
OST
26.39-26.71Mb
RUF
27.21-27.44Mb
GLA
23.43-23.67Mb
BAR
23.44-23.66Mb
GLU
26.60-26.89Mb
LON
20.89-21.26Mb
MER
26.62-26.85Mb
PUN
29.06-29.32Mb
BRA
16.66-16.86Mb

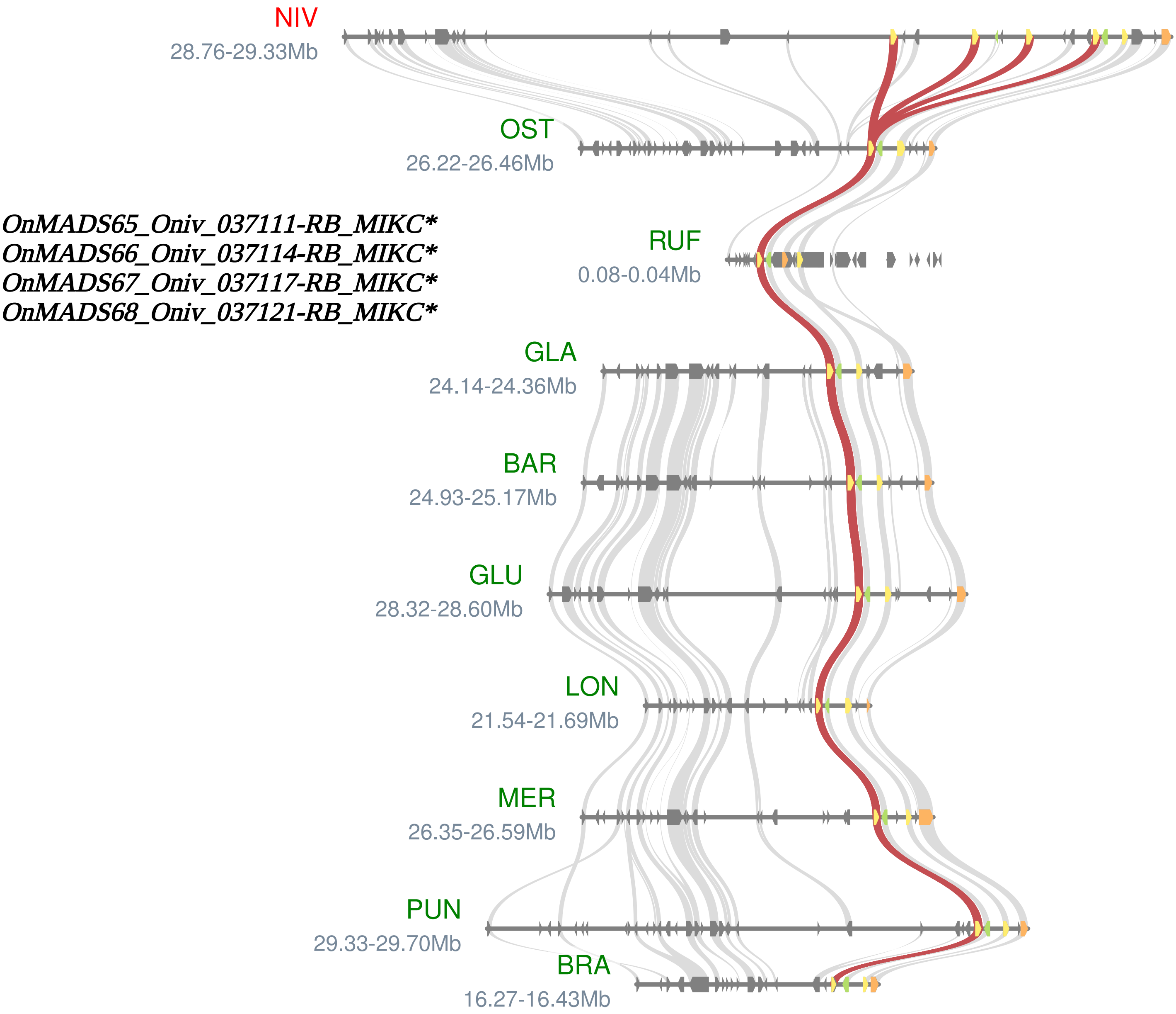
NIV
28.76-29.33Mb
OST
26.22-26.46Mb
OnMADS65_Oniv_037111-RB_MIKC*
OnMADS66_Oniv_037114-RB_MIKC*
OnMADS67_Oniv_037117-RB_MIKC*
OnMADS68_Oniv_037121-RB_MIKC*
RUF
0.08-0.04Mb
GLA
24.14-24.36Mb
BAR
24.93-25.17Mb
GLU
28.32-28.60Mb
LON
21.54-21.69Mb
MER
26.35-26.59Mb
PUN
29.33-29.70Mb
BRA
16.27-16.43Mb

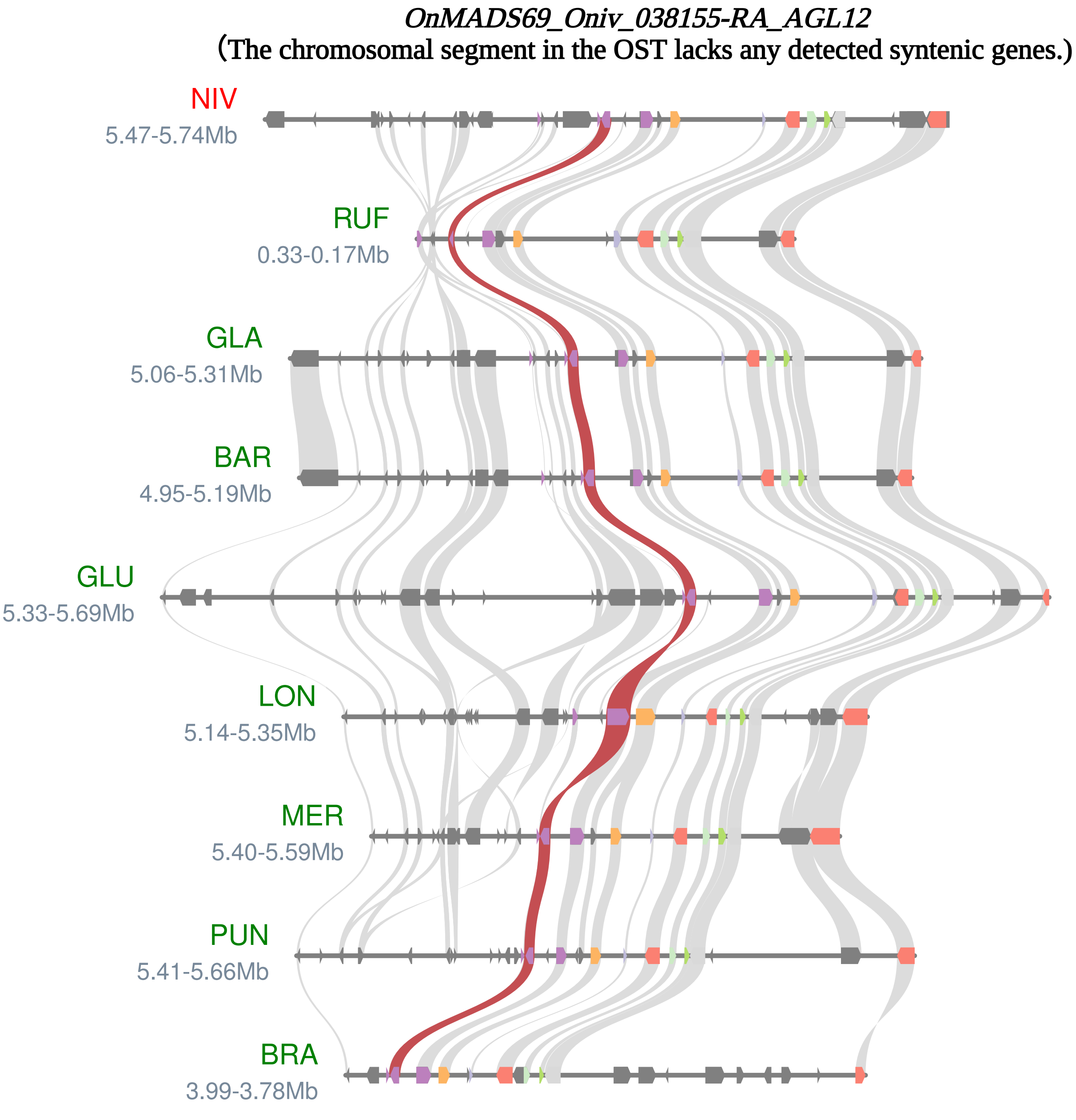

OnMADS69_Oniv_038155-RA_AGL12
(The chromosomal segment in the OST lacks any detected syntenic genes.)
NIV
5.47-5.74Mb
RUF
0.33-0.17Mb
GLA
5.06-5.31Mb
BAR
4.95-5.19Mb
GLU
5.33-5.69Mb
LON
5.14-5.35Mb
MER
5.40-5.59Mb
PUN
5.41-5.66Mb
BRA
3.99-3.78Mb

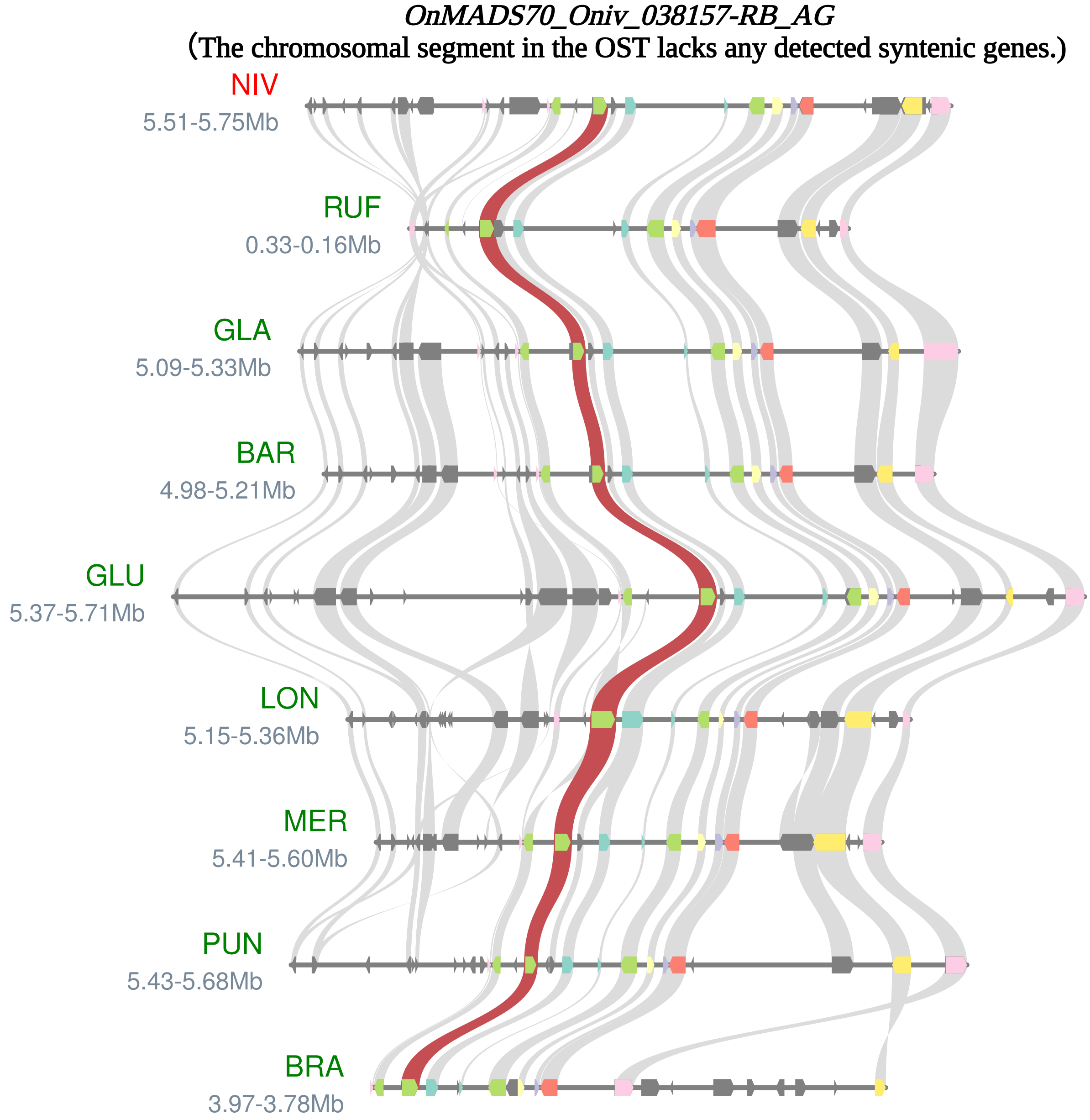

OnMADS70_Oniv_038157-RB_AG
(The chromosomal segment in the OST lacks any detected syntenic genes.)
NIV
5.51-5.75Mb
RUF
0.33-0.16Mb
GLA
5.09-5.33Mb
BAR
4.98-5.21Mb
GLU
5.37-5.71Mb
LON
5.15-5.36Mb
MER
5.41-5.60Mb
PUN
5.43-5.68Mb
BRA
3.97-3.78Mb

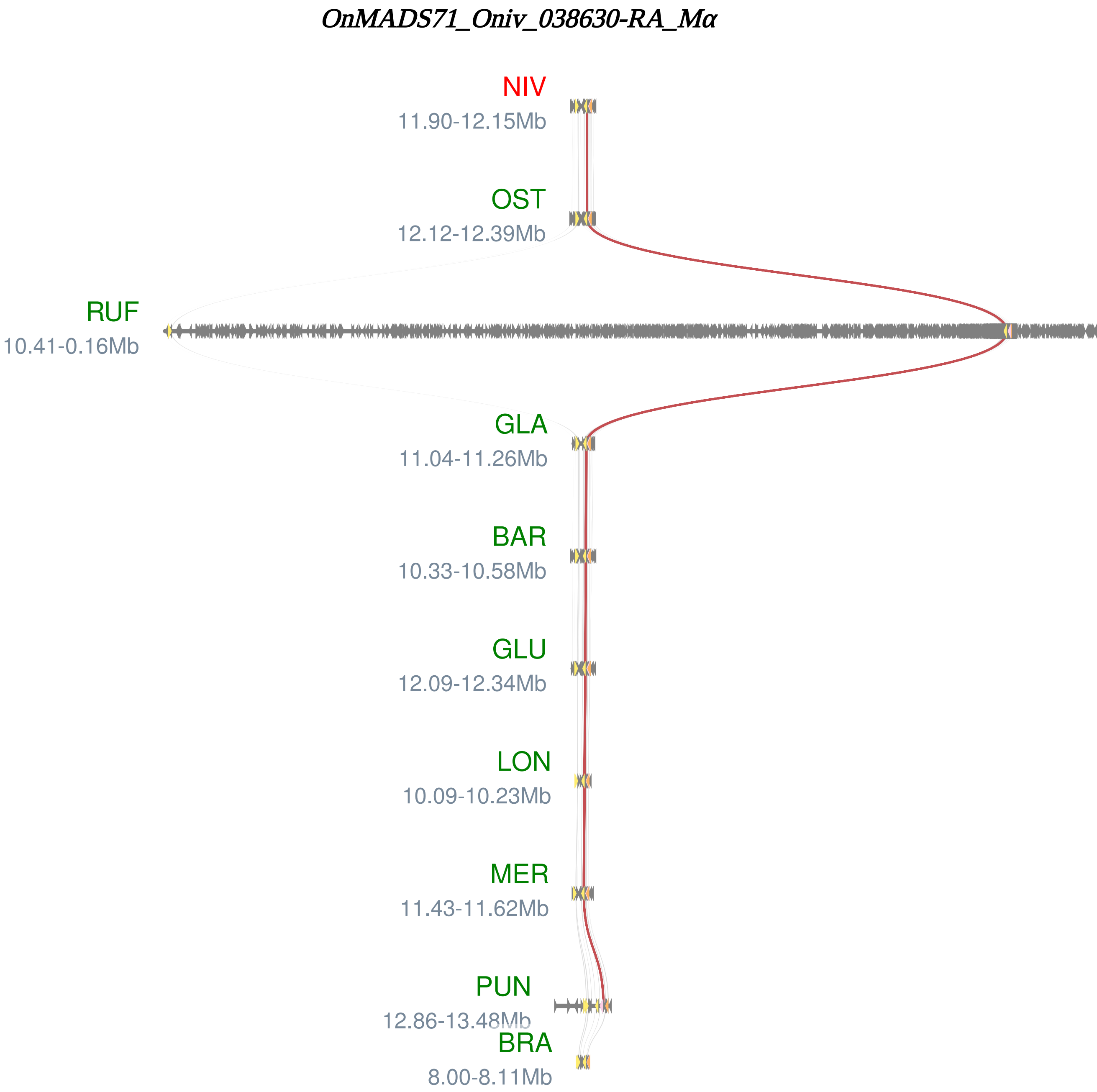

OnMADS71_Oniv_038630-RA_Mα
NIV
11.90-12.15Mb
OST
12.12-12.39Mb
RUF
10.41-0.16Mb
GLA
11.04-11.26Mb
BAR
10.33-10.58Mb
GLU
12.09-12.34Mb
LON
10.09-10.23Mb
MER
11.43-11.62Mb
PUN
12.86-13.48Mb
BRA
8.00-8.11Mb

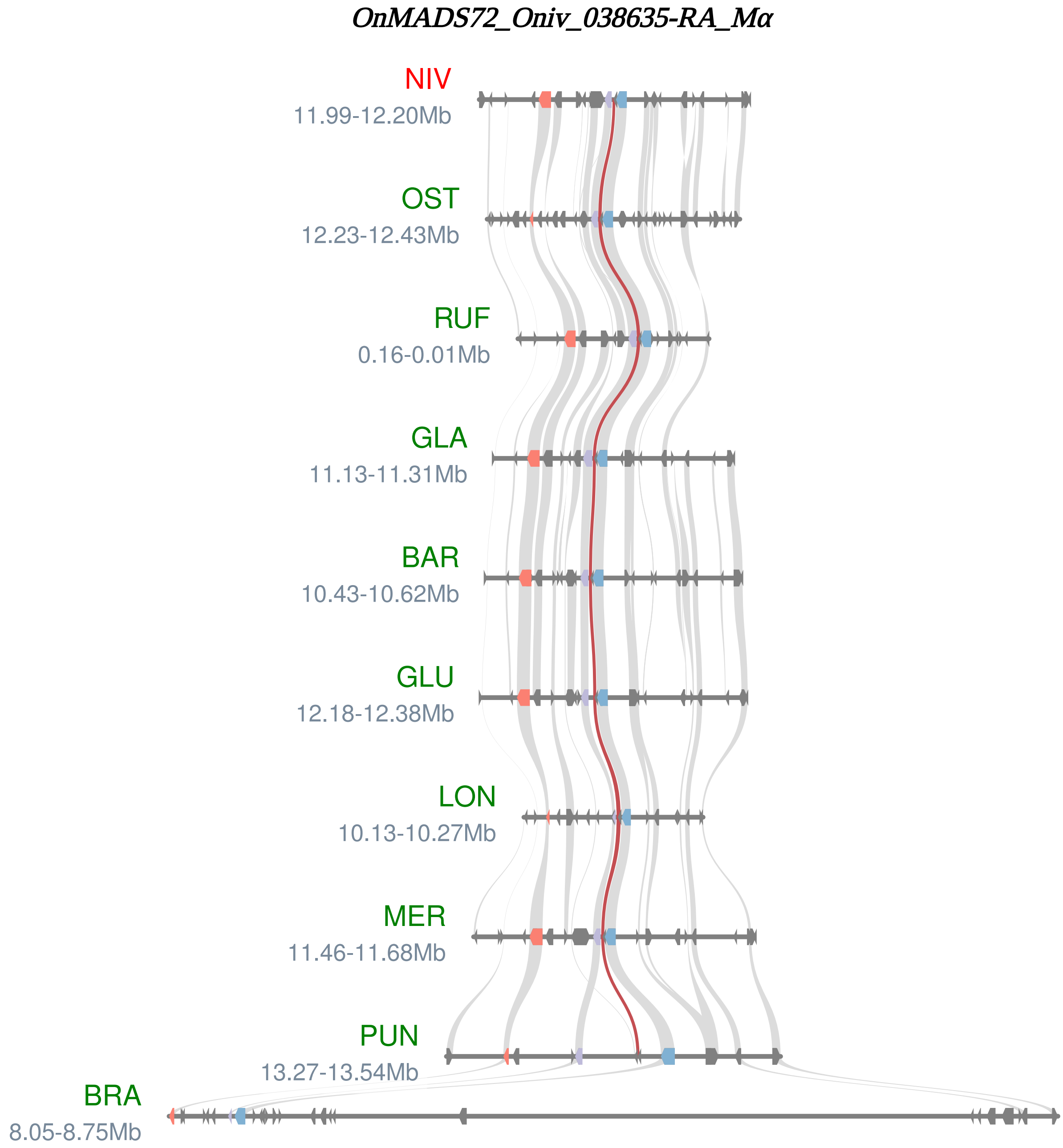
OnMADS72_Oniv_038635-RA_Mα
NIV
11.99-12.20Mb
OST
12.23-12.43Mb
RUF
0.16-0.01Mb
GLA
11.13-11.31Mb
BAR
10.43-10.62Mb
GLU
12.18-12.38Mb
LON
10.13-10.27Mb
MER
11.46-11.68Mb
PUN
13.27-13.54Mb
BRA
8.05-8.75Mb

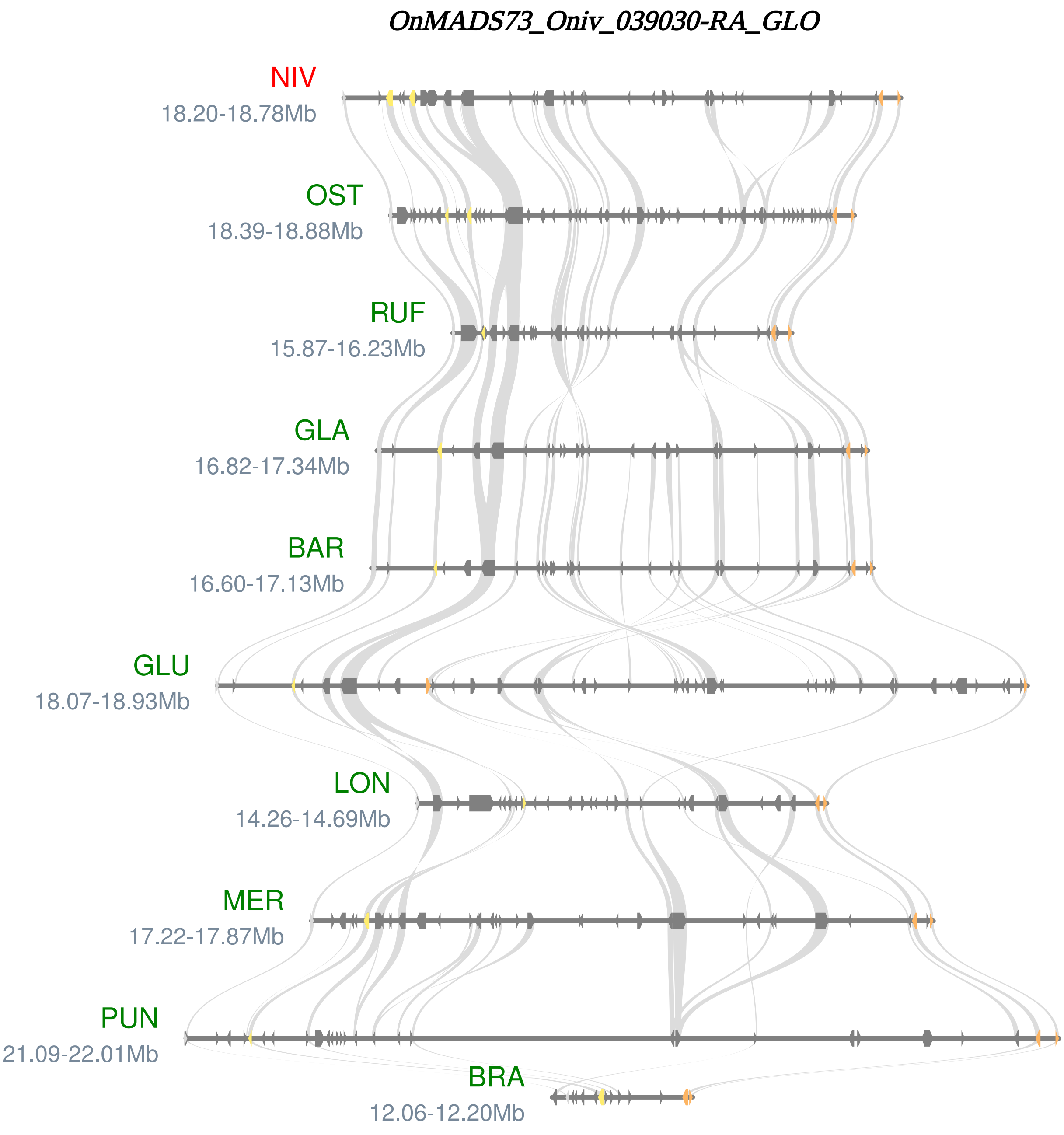
OnMADS73_Oniv_039030-RA_GLO
NIV
18.20-18.78Mb
OST
18.39-18.88Mb
RUF
15.87-16.23Mb
GLA
16.82-17.34Mb
BAR
16.60-17.13Mb
GLU
18.07-18.93Mb
LON
14.26-14.69Mb
MER
17.22-17.87Mb
PUN
21.09-22.01Mb
BRA
12.06-12.20Mb

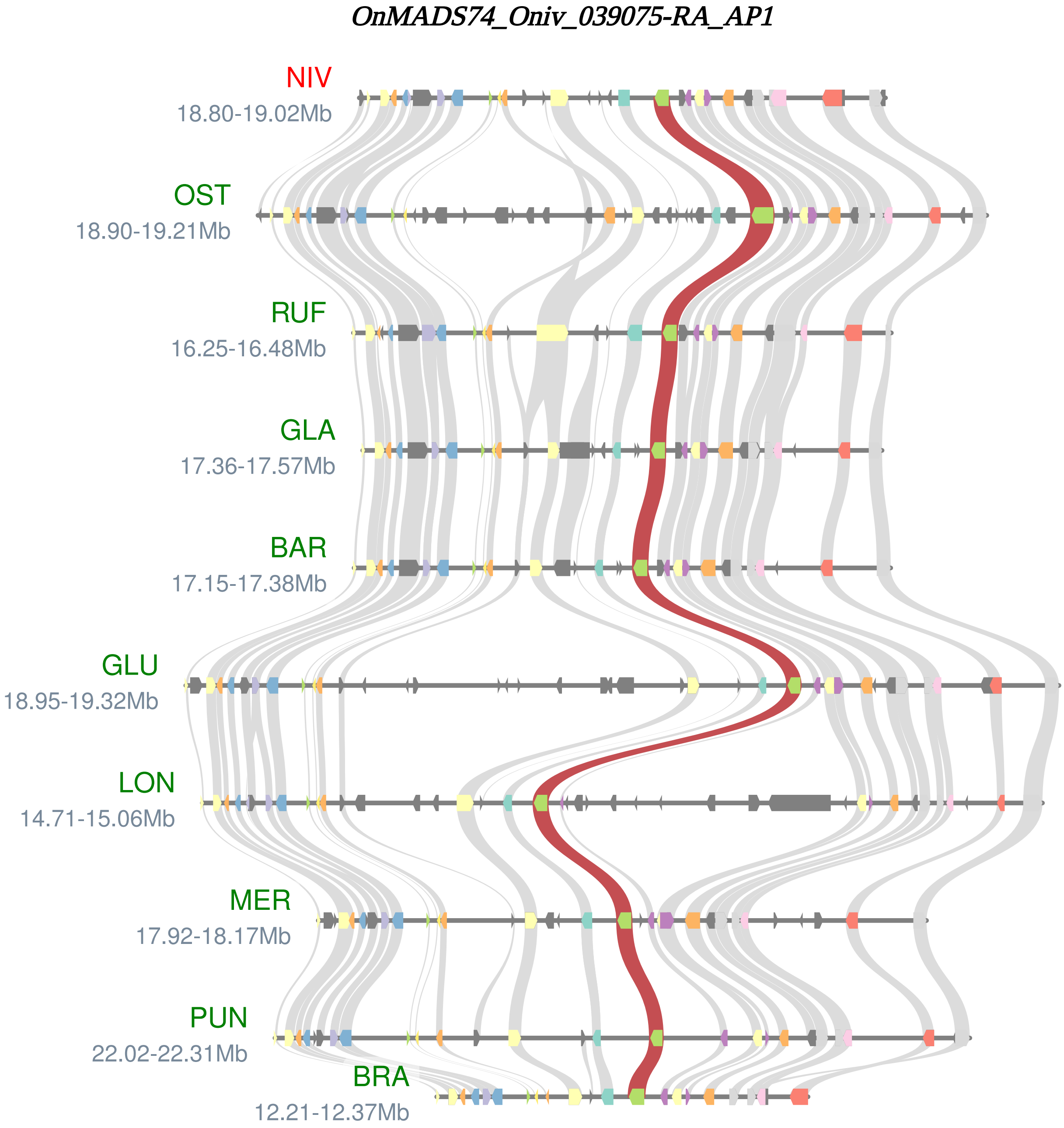
OnMADS74_Oniv_039075-RA_AP1
NIV
18.80-19.02Mb
OST
18.90-19.21Mb
RUF
16.25-16.48Mb
GLA
17.36-17.57Mb
BAR
17.15-17.38Mb
GLU
18.95-19.32Mb
LON
14.71-15.06Mb
MER
17.92-18.17Mb
PUN
22.02-22.31Mb
BRA
12.21-12.37Mb

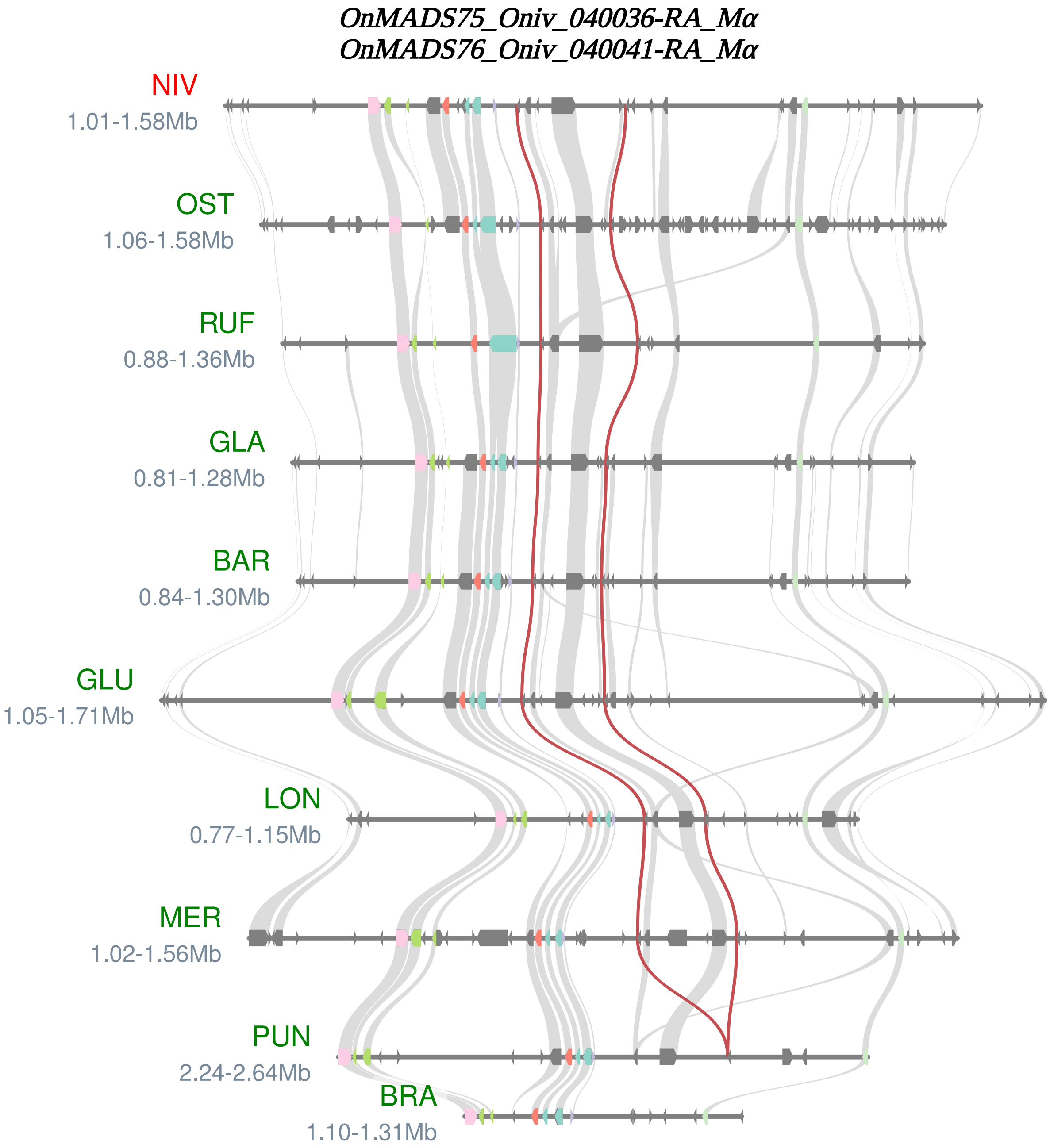

OnMADS75_Oniv_040036-RA_Mα
OnMADS76_Oniv_040041-RA_Mα
NIV
1.01-1.58Mb
OST
1.06-1.58Mb
RUF
0.88-1.36Mb
GLA
0.81-1.28Mb
BAR
0.84-1.30Mb
GLU
1.05-1.71Mb
LON
0.77-1.15Mb
MER
1.02-1.56Mb
PUN
2.24-2.64Mb
BRA
1.10-1.31Mb

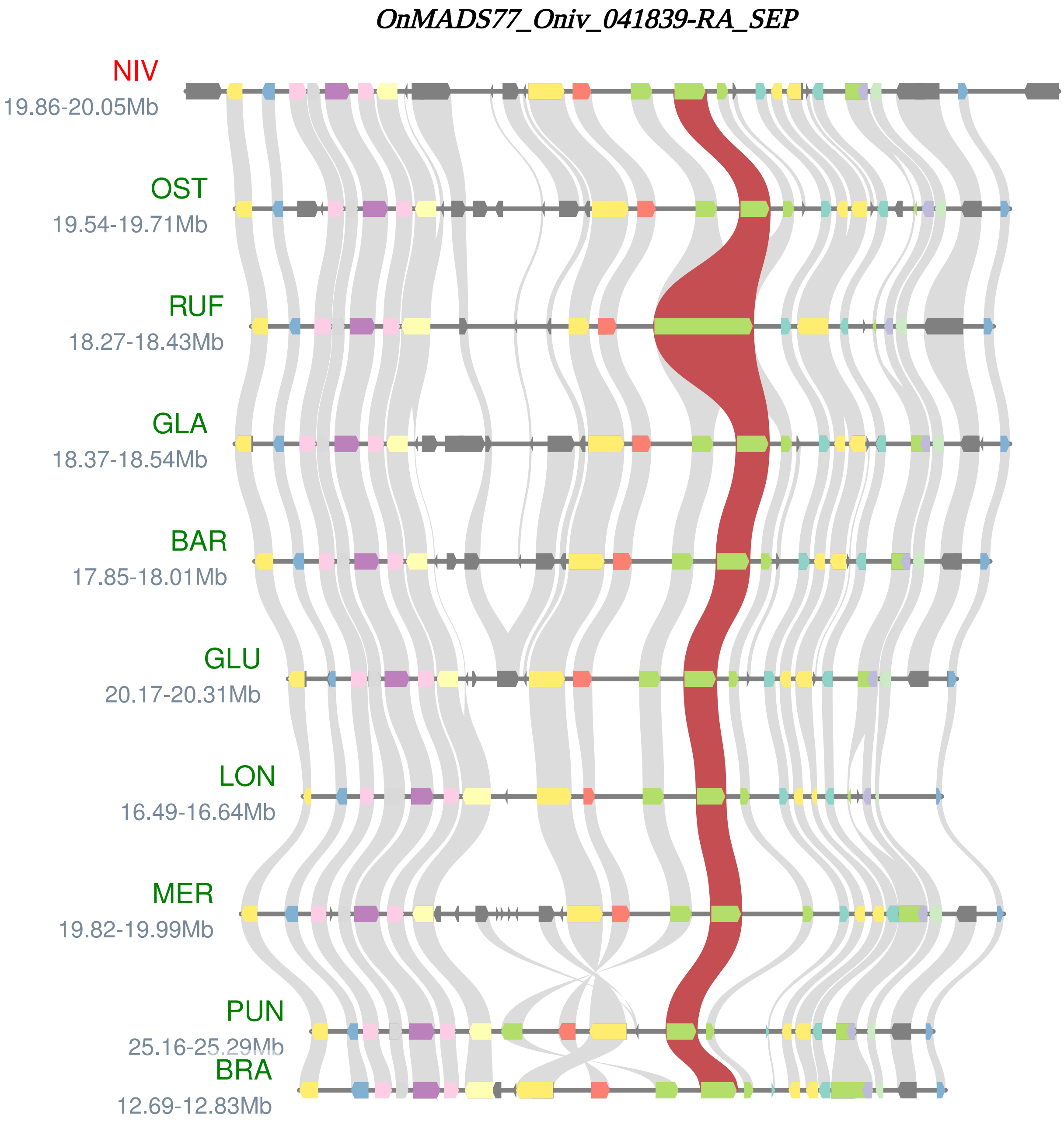
OnMADS77_Oniv_041839-RA_SEP
NIV
19.86-20.05Mb
OST
19.54-19.71Mb
RUF
18.27-18.43Mb
GLA
18.37-18.54Mb
BAR
17.85-18.01Mb
GLU
20.17-20.31Mb
LON
16.49-16.64Mb
MER
19.82-19.99Mb
PUN
25.16-25.29Mb
BRA
12.69-12.83Mb

*OnMADS78_Oniv_042424-RB_MIKC**
(The chromosomal segment in the LON& MER&PUN&BRA lacks any detected syntenic genes.)

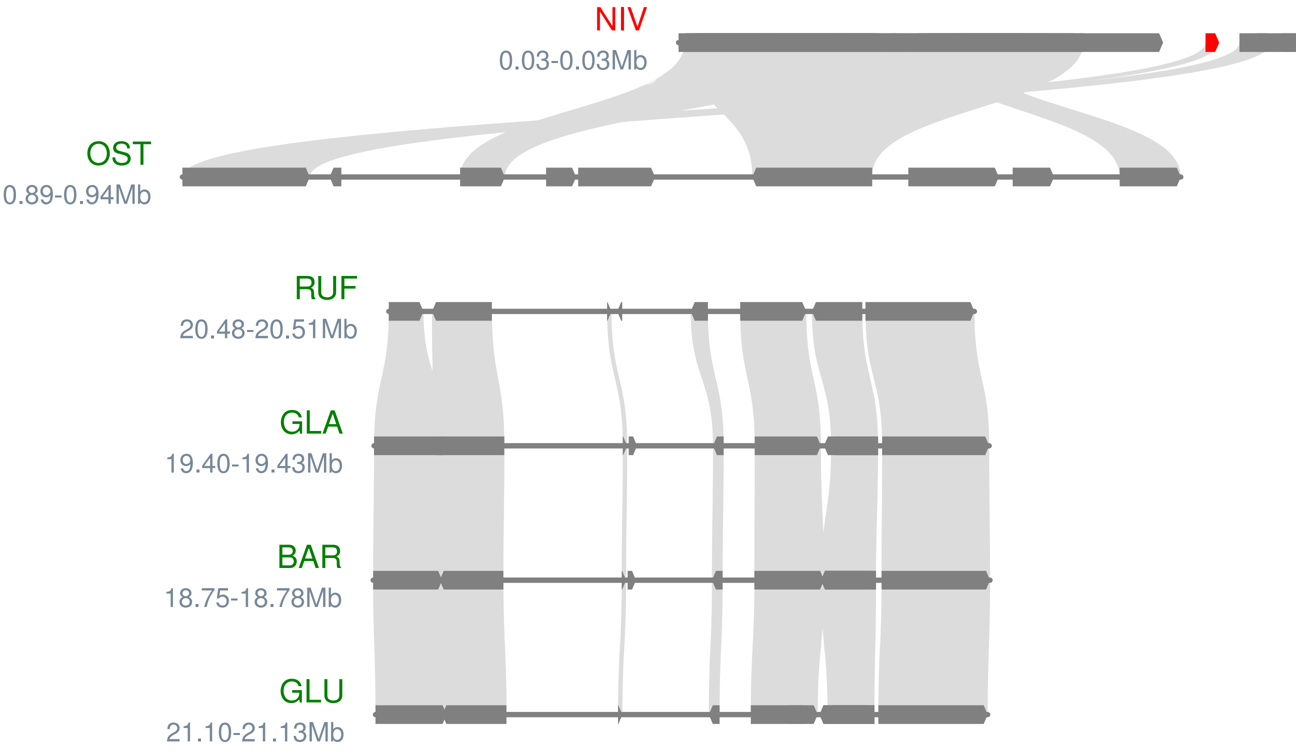

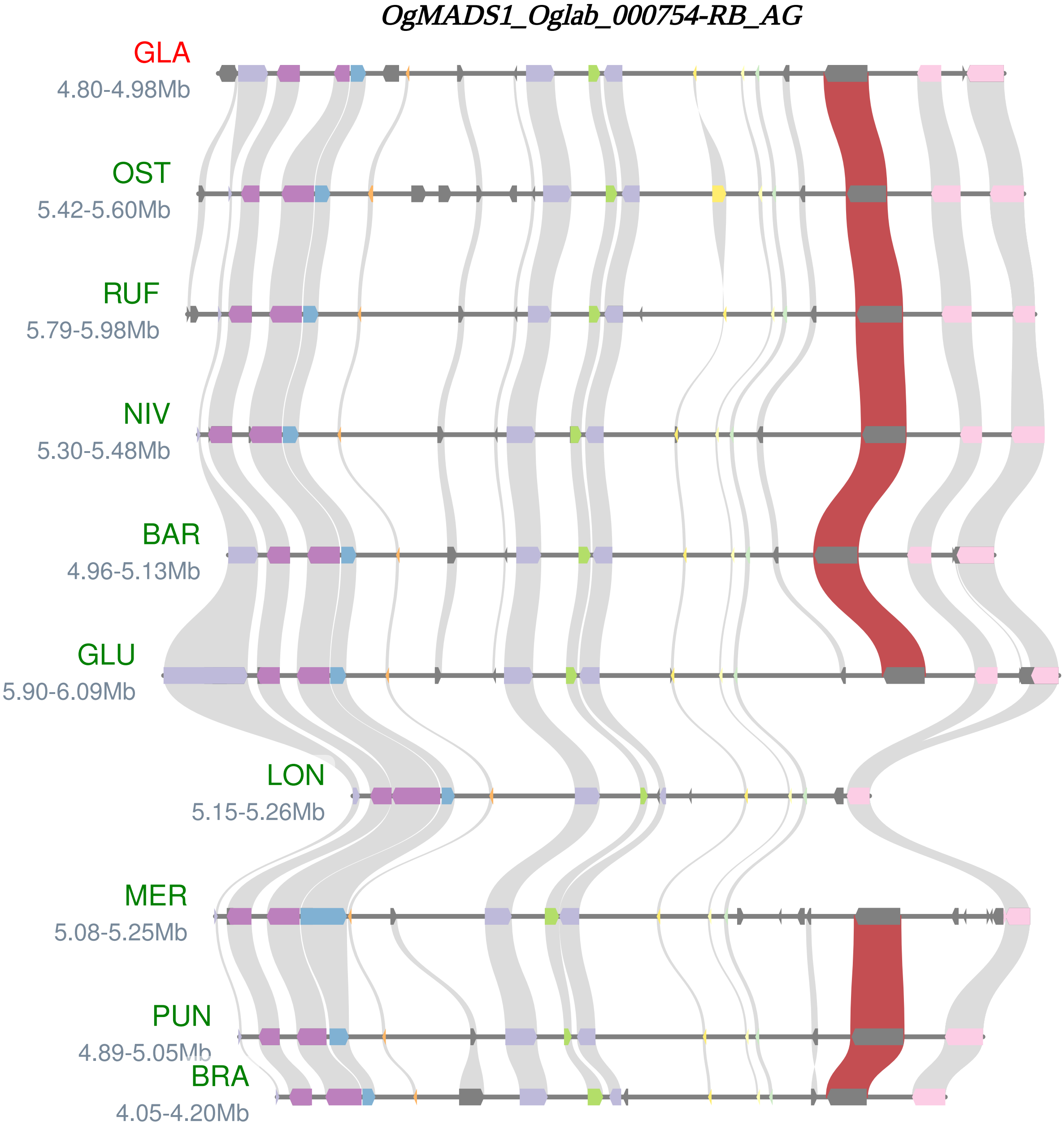

OgMADS1_Oglab_000754-RB_AG
GLA
4.80-4.98Mb
OST
5.42-5.60Mb
RUF
5.79-5.98Mb
NIV
5.30-5.48Mb
BAR
4.96-5.13Mb
GLU
5.90-6.09Mb
LON
5.15-5.26Mb
MER
5.08-5.25Mb
PUN
4.89-5.05Mb
BRA
4.05-4.20Mb

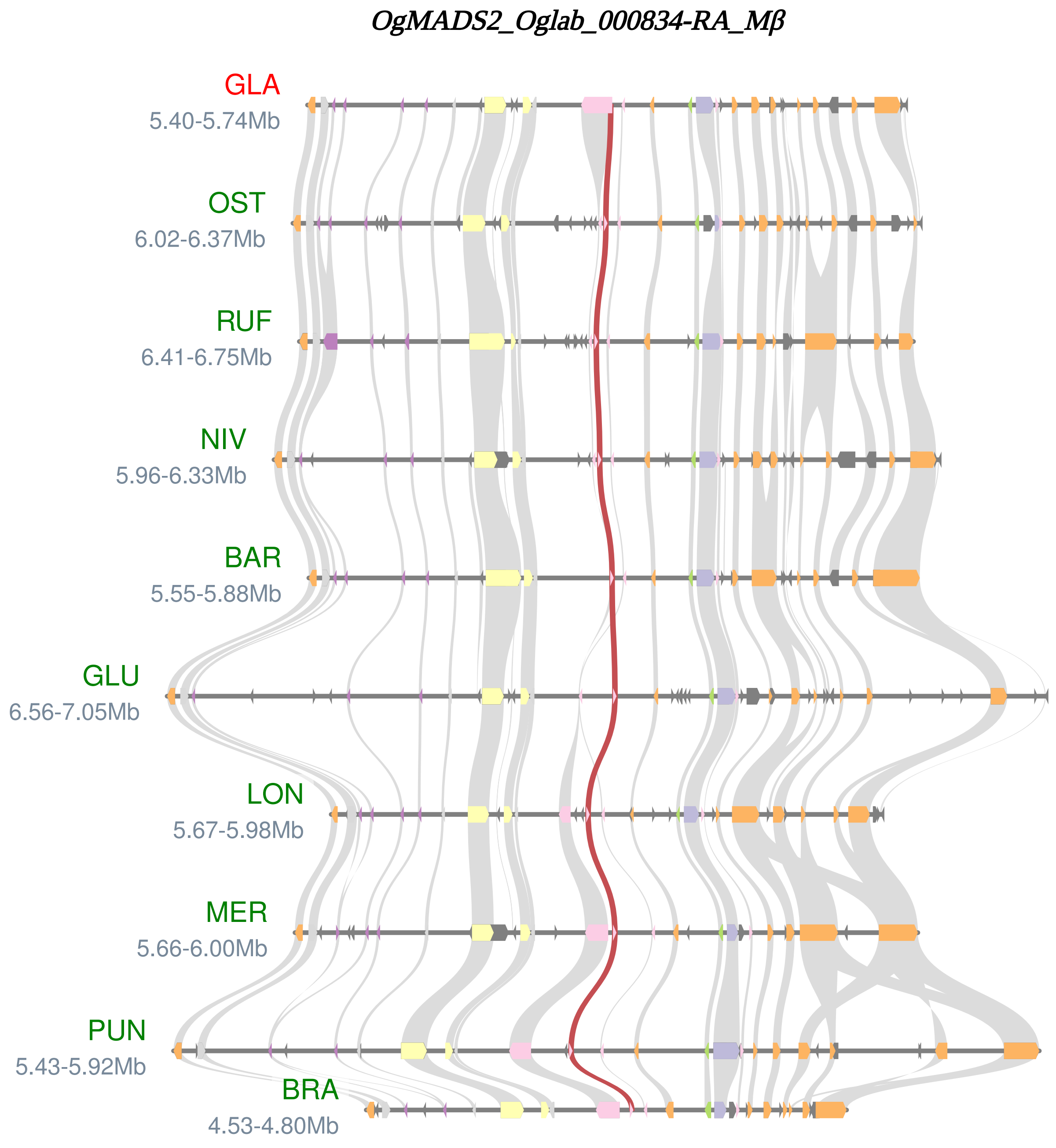
OgMADS2_Oglab_000834-RA_Mβ
GLA
5.40-5.74Mb
OST
6.02-6.37Mb
RUF
6.41-6.75Mb
NIV
5.96-6.33Mb
BAR
5.55-5.88Mb
GLU
6.56-7.05Mb
LON
5.67-5.98Mb
MER
5.66-6.00Mb
PUN
5.43-5.92Mb
BRA
4.53-4.80Mb

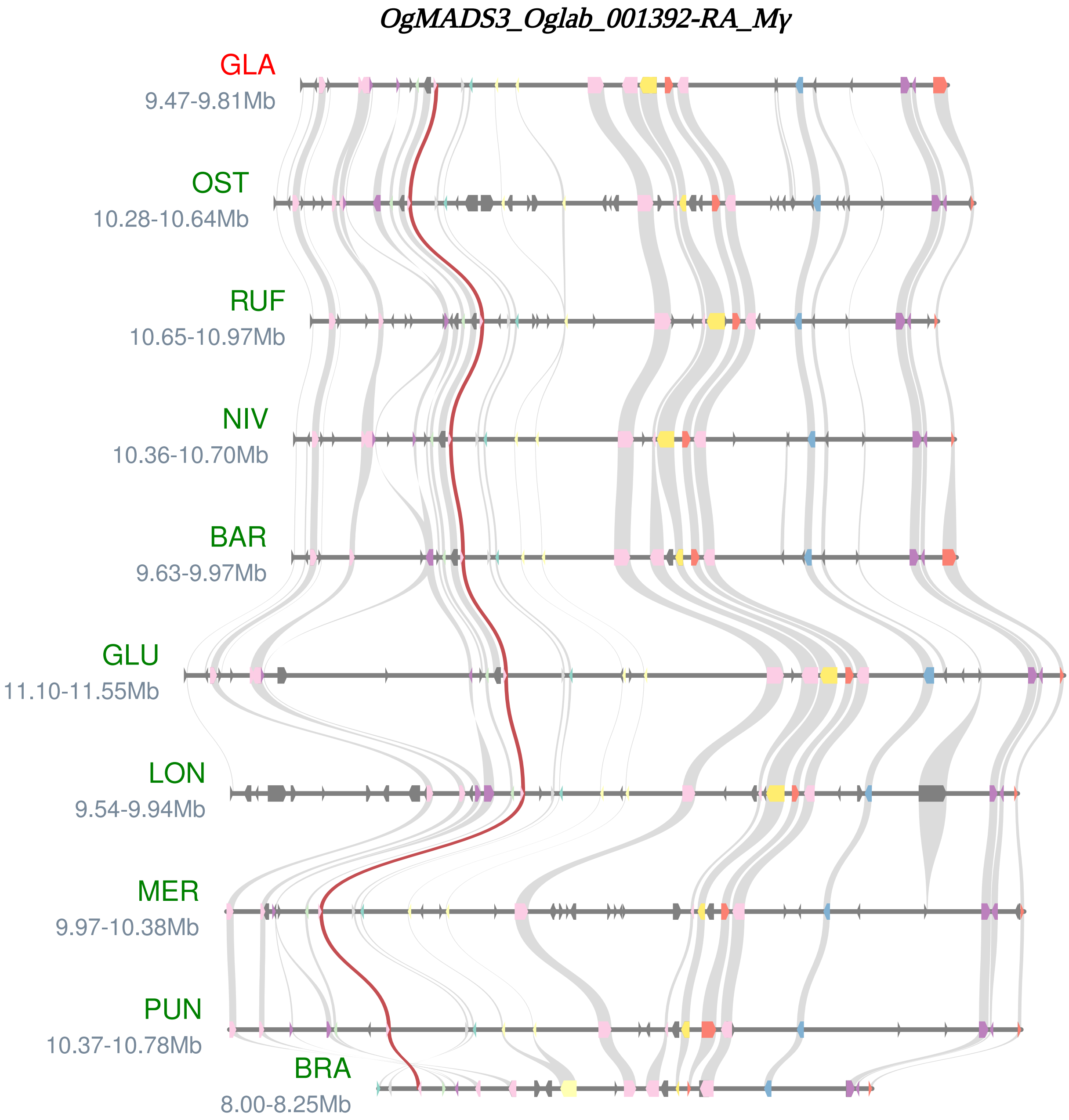

OgMADS3_Oglab_001392-RA_Mγ
GLA
9.47-9.81Mb
OST
10.28-10.64Mb
RUF
10.65-10.97Mb
NIV
10.36-10.70Mb
BAR
9.63-9.97Mb
GLU
11.10-11.55Mb
LON
9.54-9.94Mb
MER
9.97-10.38Mb
PUN
10.37-10.78Mb
BRA
8.00-8.25Mb

OgMADS4_Oglab_001393-RA_Mγ

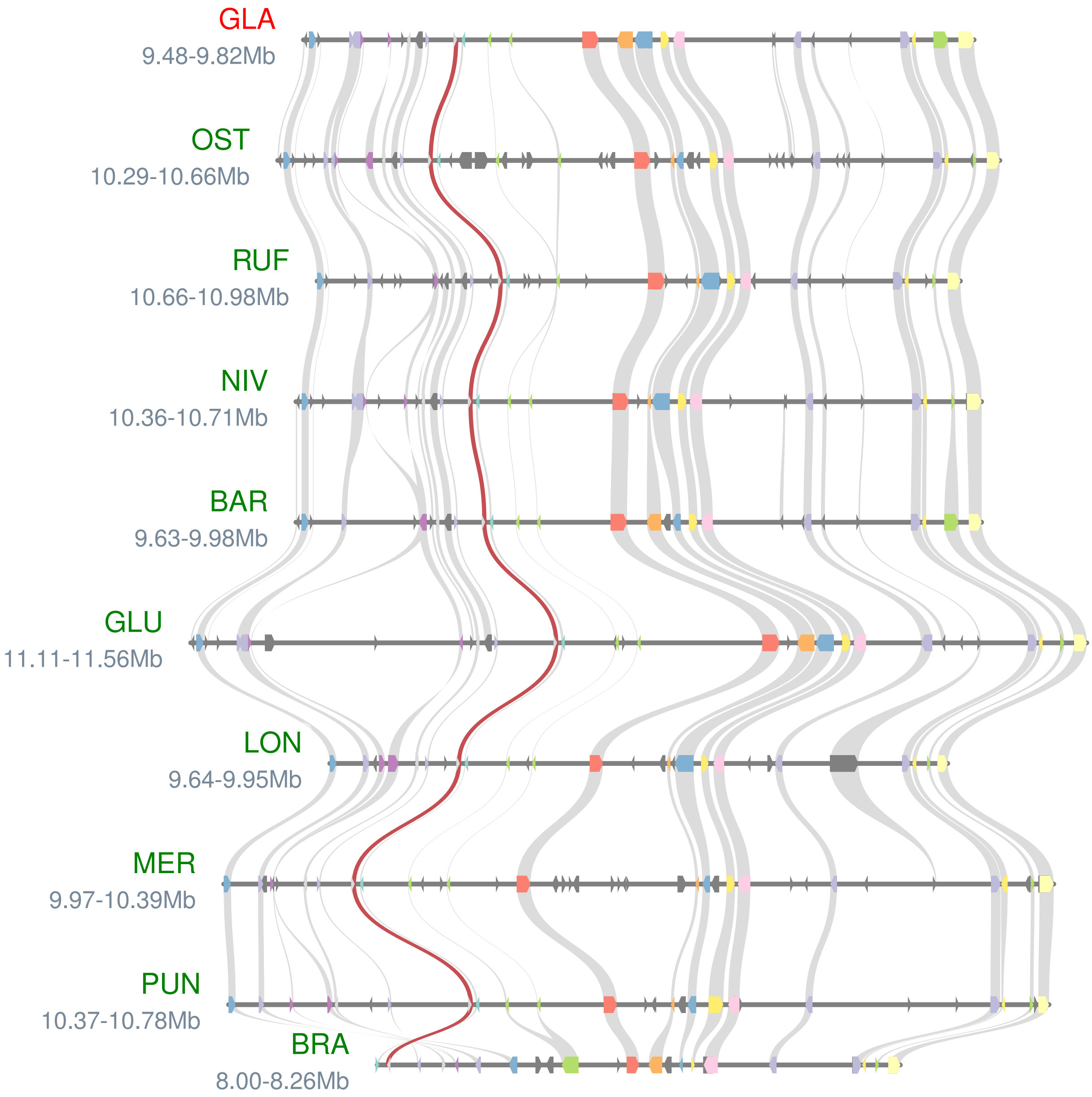

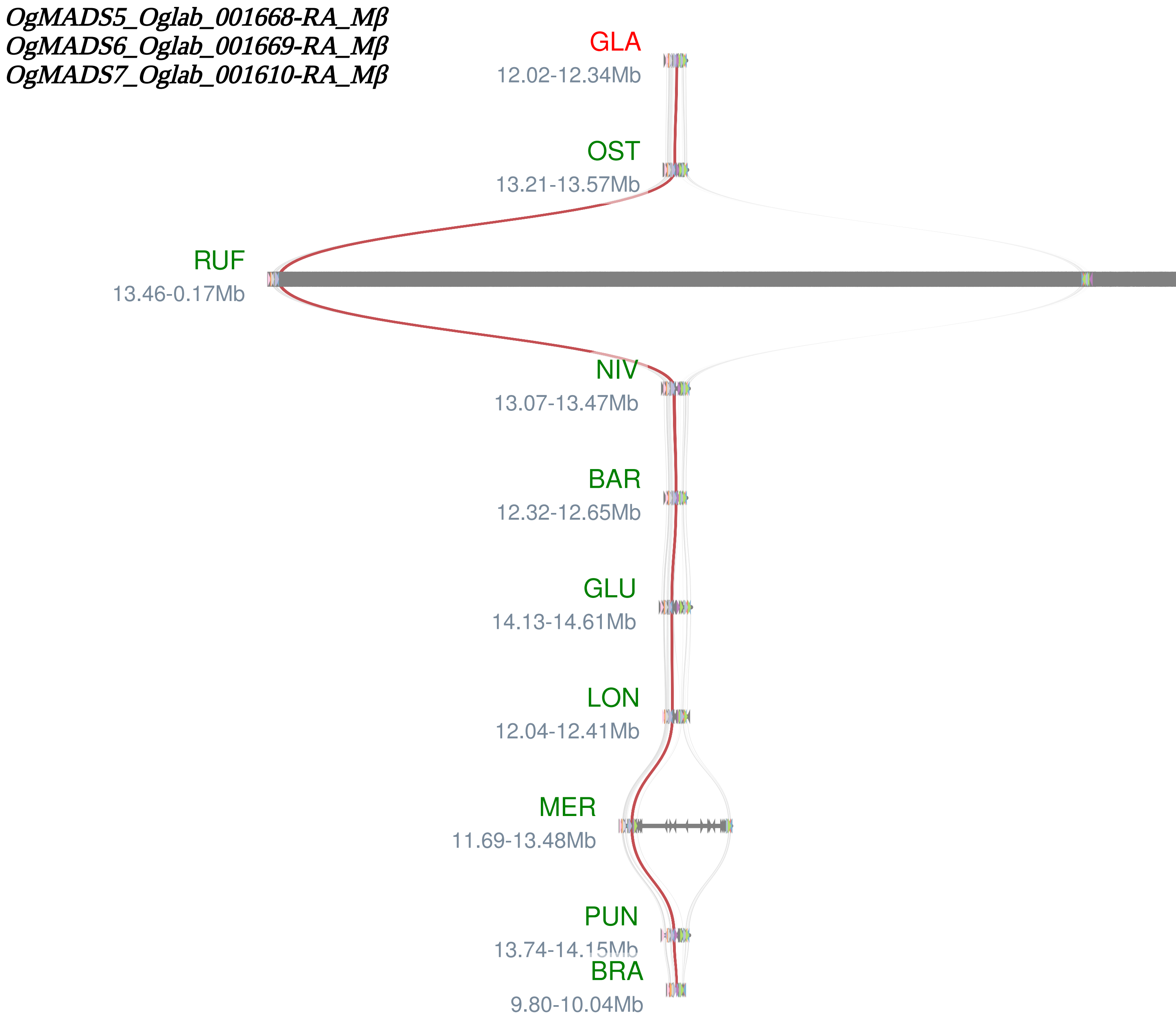

OgMADS5_Oglab_001668-RA_Mβ
OgMADS6_Oglab_001669-RA_Mβ
OgMADS7_Oglab_001610-RA_Mβ
GLA
12.02-12.34Mb
OST
13.21-13.57Mb
RUF
13.46-0.17Mb
NIV
13.07-13.47Mb
BAR
12.32-12.65Mb
GLU
14.13-14.61Mb
LON
12.04-12.41Mb
MER
11.69-13.48Mb
PUN
13.74-14.15Mb
BRA
9.80-10.04Mb

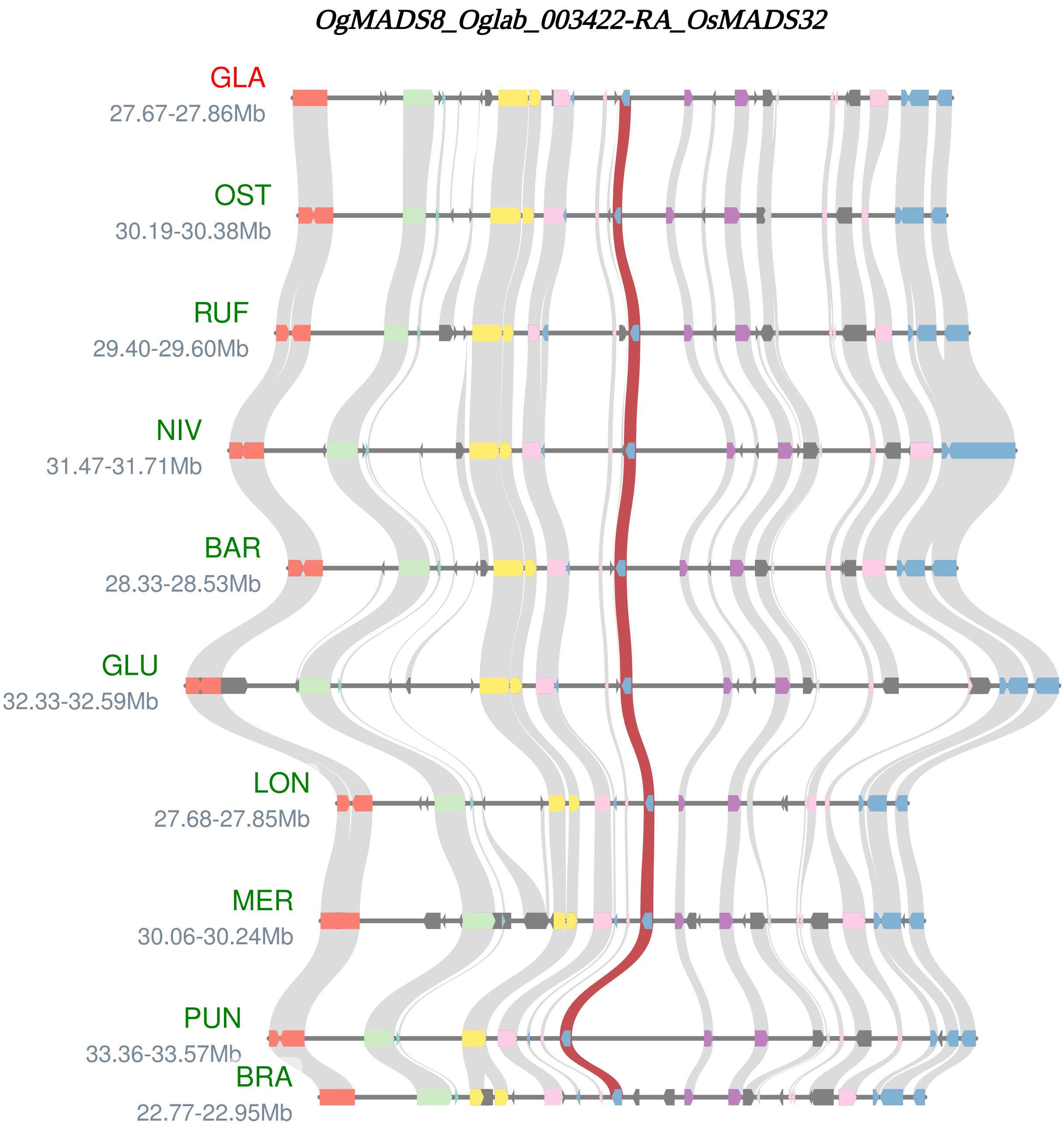

OgMADS8_Oglab_003422-RA_OsMADS32
GLA
27.67-27.86Mb
OST
30.19-30.38Mb
RUF
29.40-29.60Mb
NIV
31.47-31.71Mb
BAR
28.33-28.53Mb
GLU
32.33-32.59Mb
LON
27.68-27.85Mb
MER
30.06-30.24Mb
PUN
33.36-33.57Mb
BRA
22.77-22.95Mb

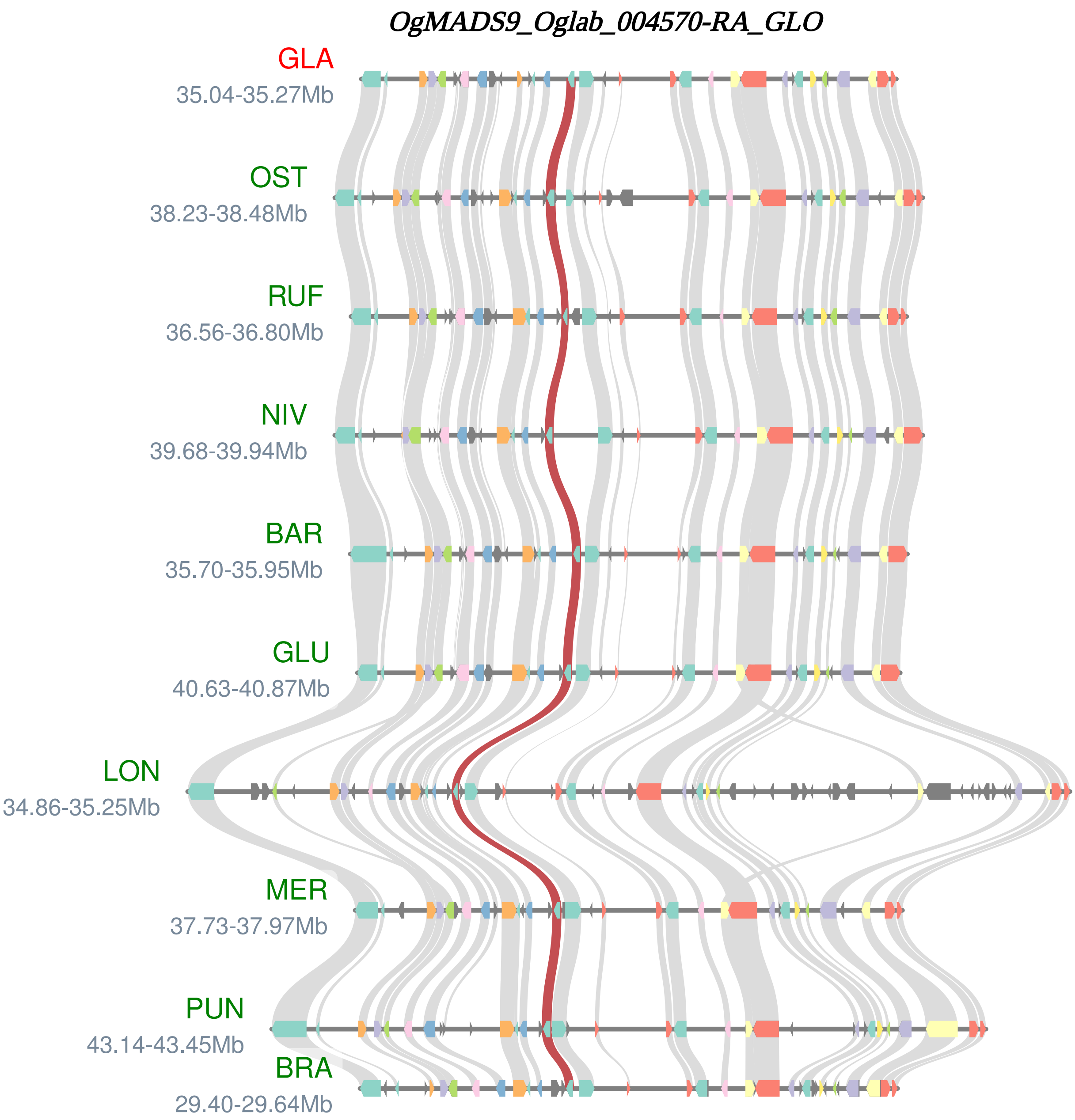
OgMADS9_Oglab_004570-RA_GLO
GLA
35.04-35.27Mb
OST
38.23-38.48Mb
RUF
36.56-36.80Mb
NIV
39.68-39.94Mb
BAR
35.70-35.95Mb
GLU
40.63-40.87Mb
LON
34.86-35.25Mb
MER
37.73-37.97Mb
PUN
43.14-43.45Mb
BRA
29.40-29.64Mb

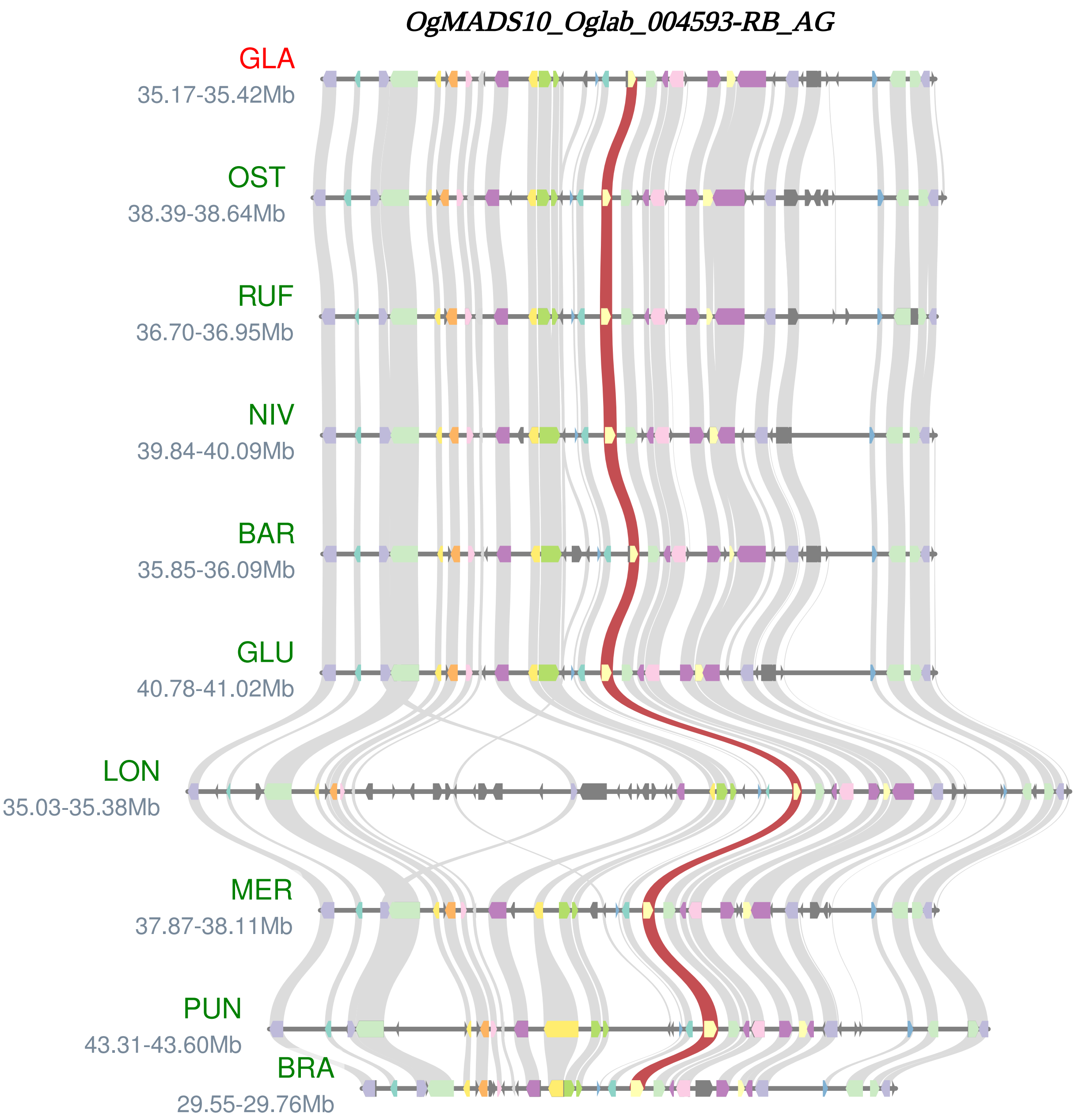
OgMADS10_Oglab_004593-RB_AG
GLA
35.17-35.42Mb
OST
38.39-38.64Mb
RUF
36.70-36.95Mb
NIV
39.84-40.09Mb
BAR
35.85-36.09Mb
GLU
40.78-41.02Mb
LON
35.03-35.38Mb
MER
37.87-38.11Mb
PUN
43.31-43.60Mb
BRA
29.55-29.76Mb

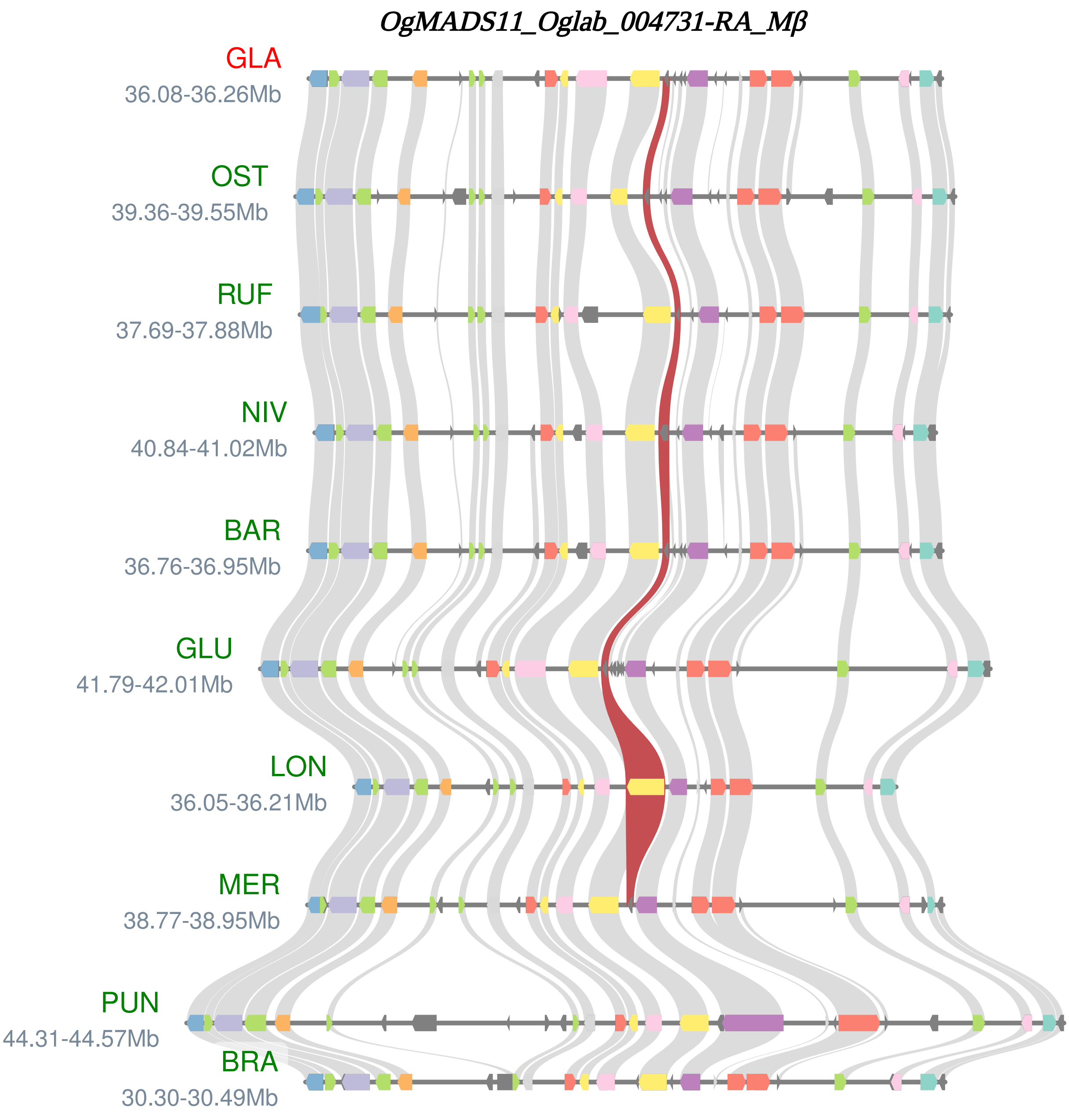
OgMADS11_Oglab_004731-RA_Mβ
GLA
36.08-36.26Mb
OST
39.36-39.55Mb
RUF
37.69-37.88Mb
NIV
40.84-41.02Mb
BAR
36.76-36.95Mb
GLU
41.79-42.01Mb
LON
36.05-36.21Mb
MER
38.77-38.95Mb
PUN
44.31-44.57Mb
BRA
30.30-30.49Mb

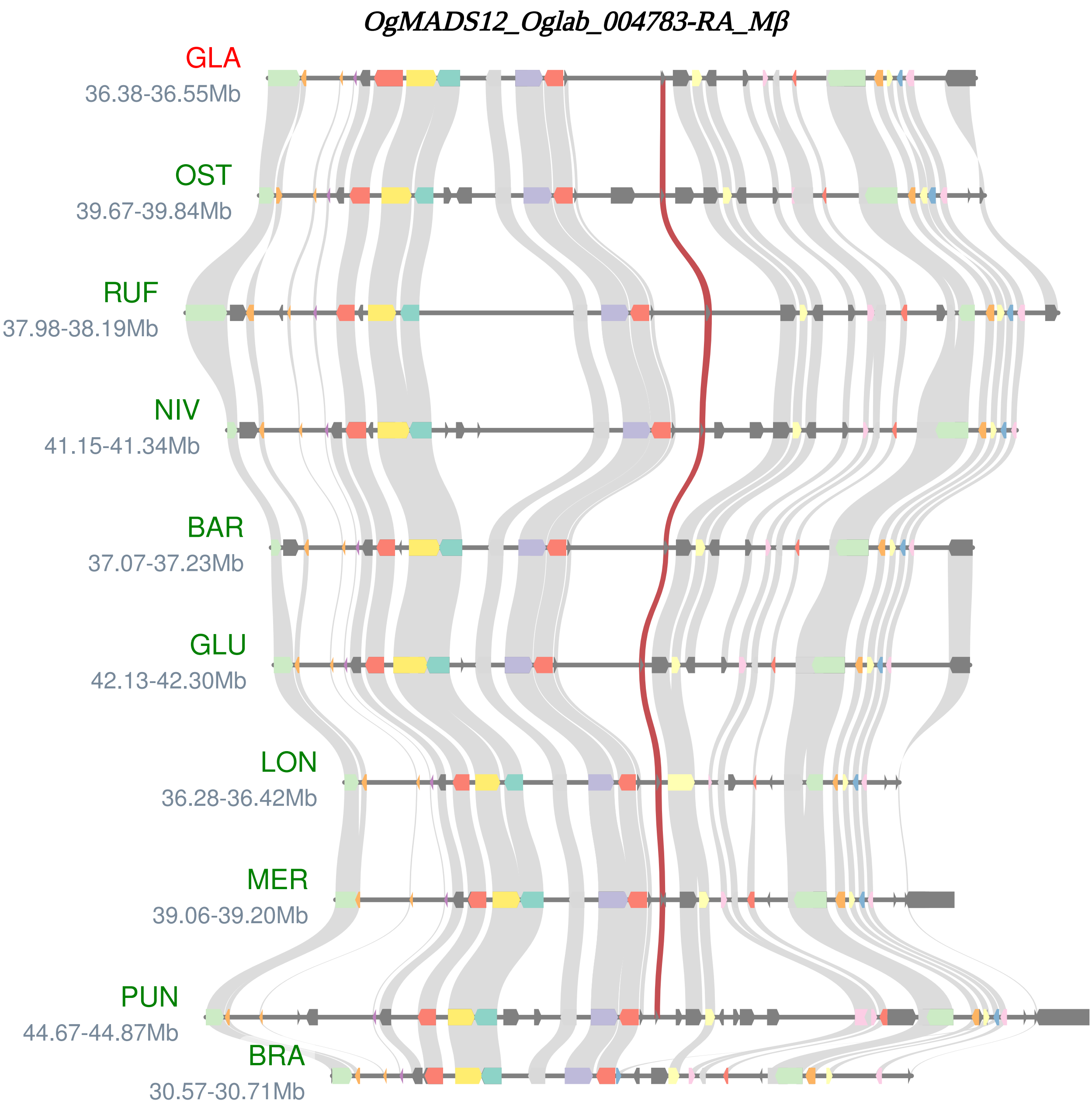

OgMADS12_Oglab_004783-RA_Mβ
GLA
36.38-36.55Mb
OST
39.67-39.84Mb
RUF
37.98-38.19Mb
NIV
41.15-41.34Mb
BAR
37.07-37.23Mb
GLU
42.13-42.30Mb
LON
36.28-36.42Mb
MER
39.06-39.20Mb
PUN
44.67-44.87Mb
BRA
30.57-30.71Mb

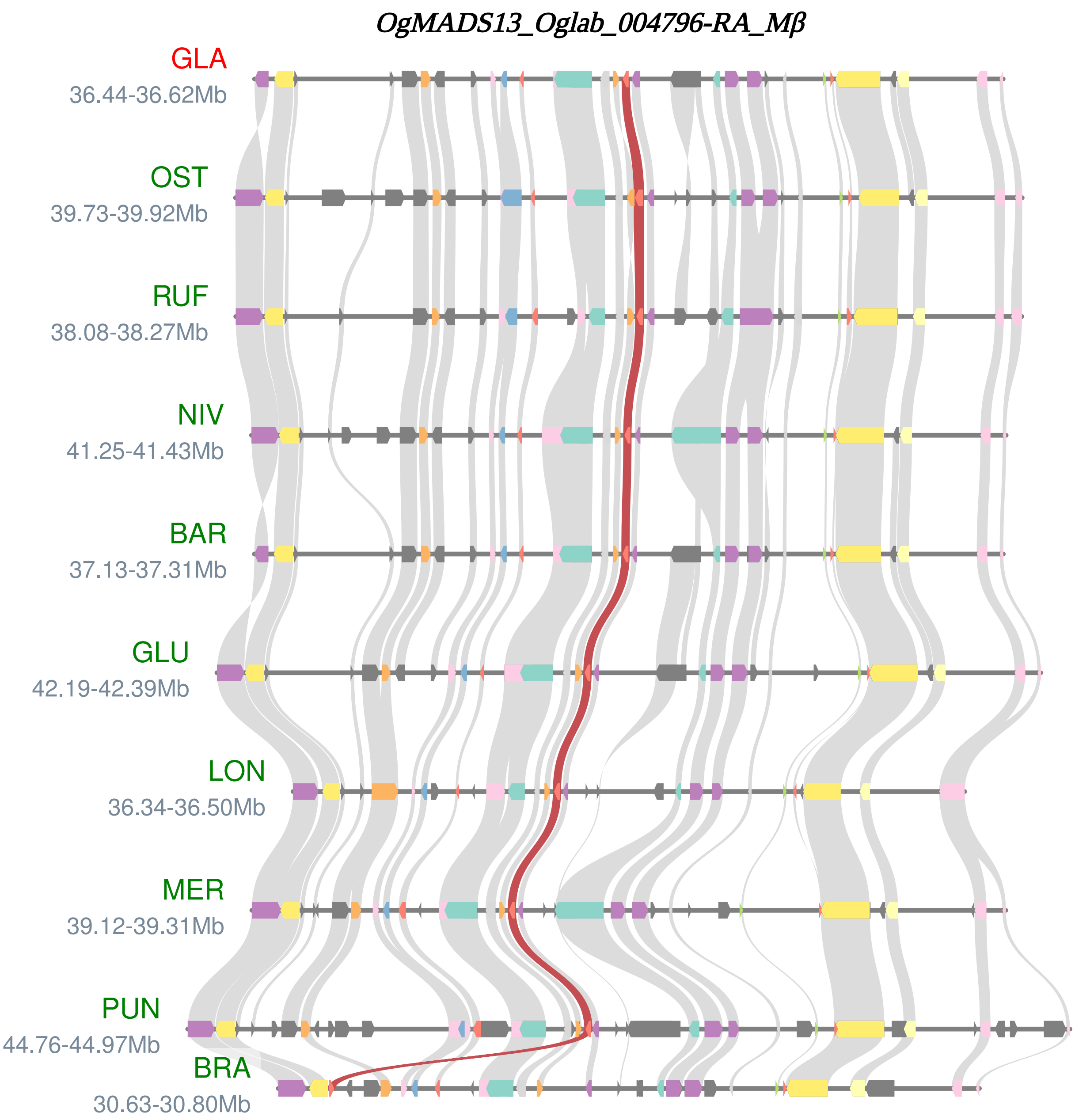
OgMADS13_Oglab_004796-RA_Mβ
GLA
36.44-36.62Mb
OST
39.73-39.92Mb
RUF
38.08-38.27Mb
NIV
41.25-41.43Mb
BAR
37.13-37.31Mb
GLU
42.19-42.39Mb
LON
36.34-36.50Mb
MER
39.12-39.31Mb
PUN
44.76-44.97Mb
BRA
30.63-30.80Mb

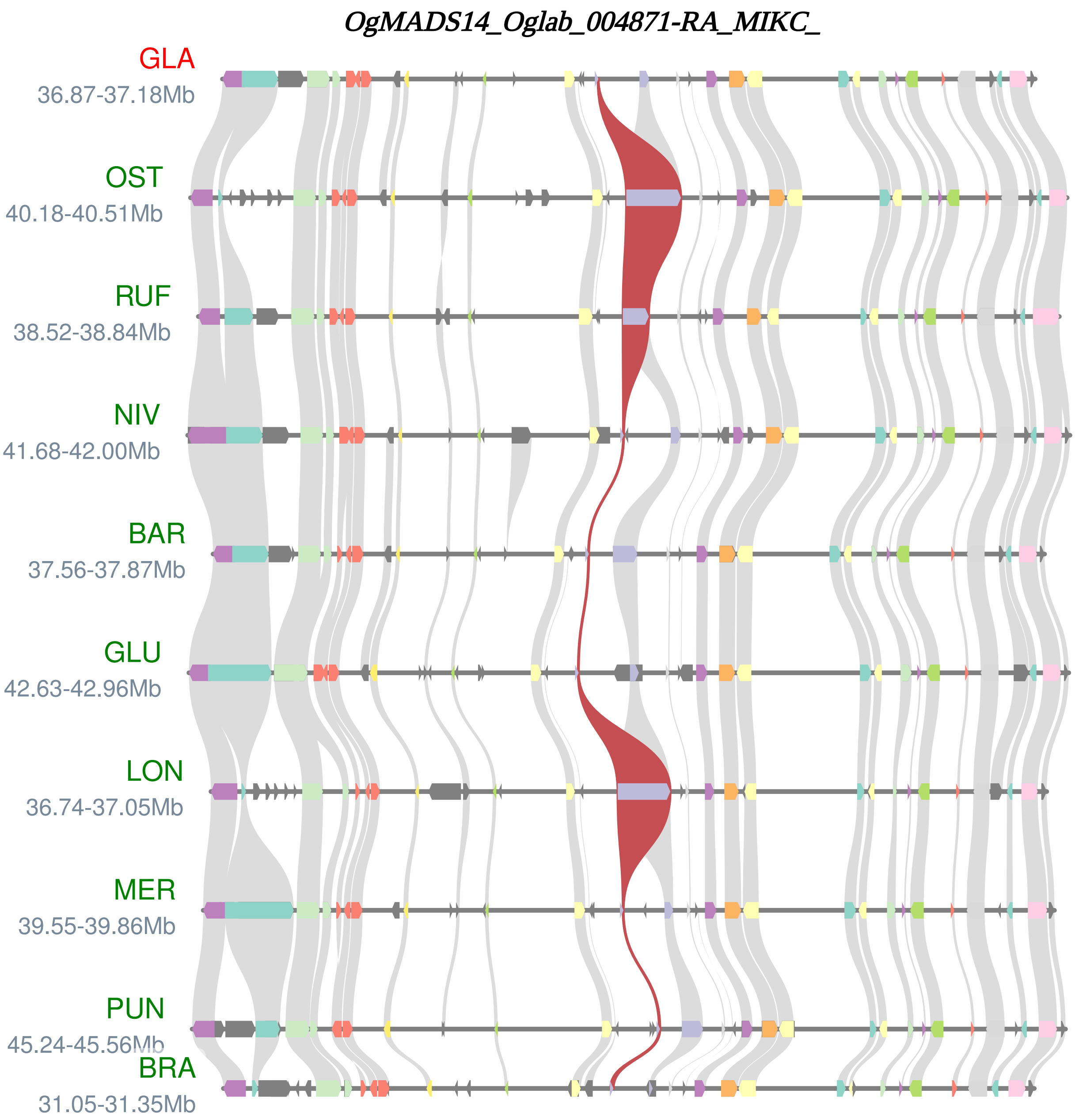

OgMADS14_Oglab_004871-RA_MIKC_
GLA
36.87-37.18Mb
OST
40.18-40.51Mb
RUF
38.52-38.84Mb
NIV
41.68-42.00Mb
BAR
37.56-37.87Mb
GLU
42.63-42.96Mb
LON
36.74-37.05Mb
MER
39.55-39.86Mb
PUN
45.24-45.56Mb
BRA
31.05-31.35Mb

OgMADS15_Oglab_005261-RA_Mα
(The chromosomal segment in the MER lacks any detected syntenic genes.)

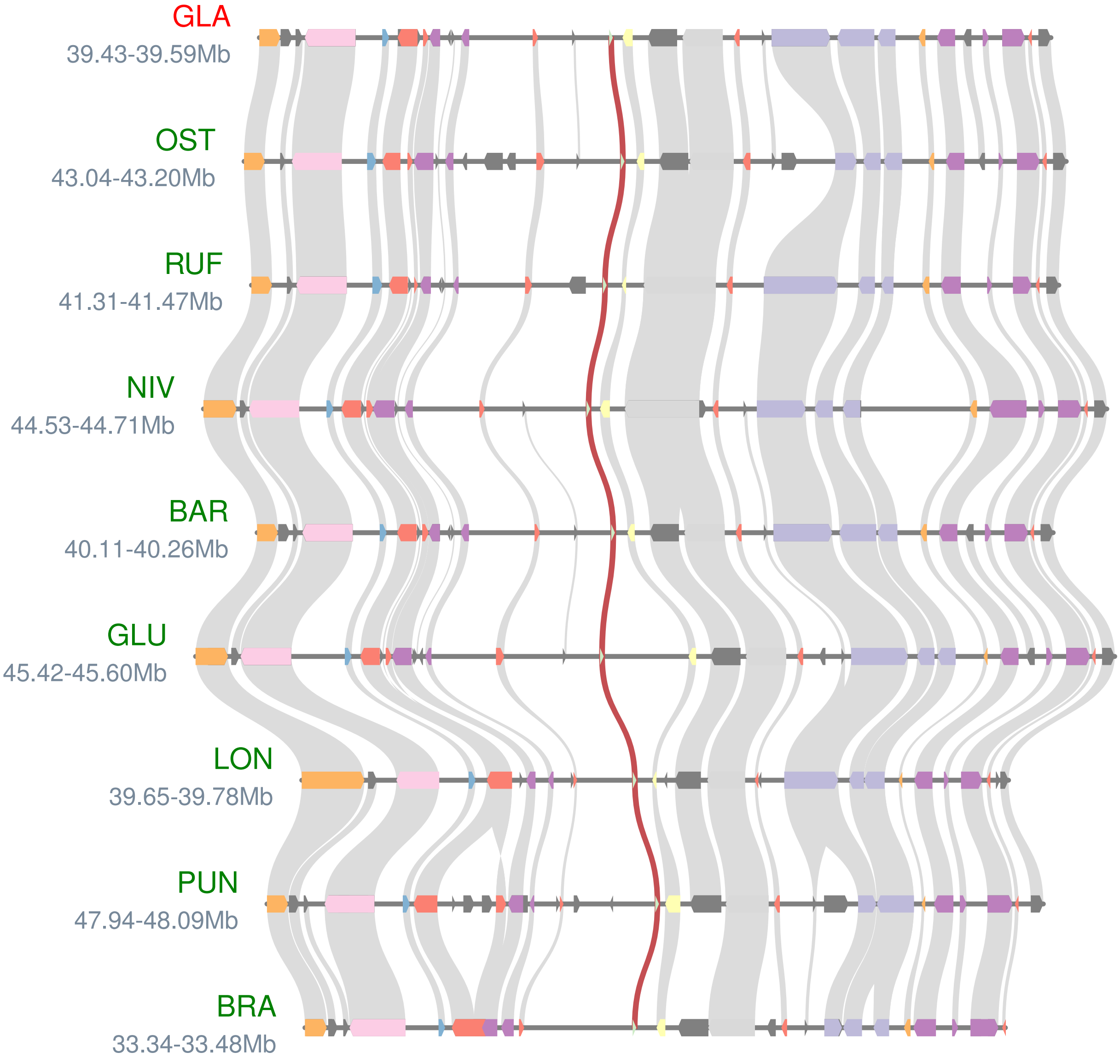

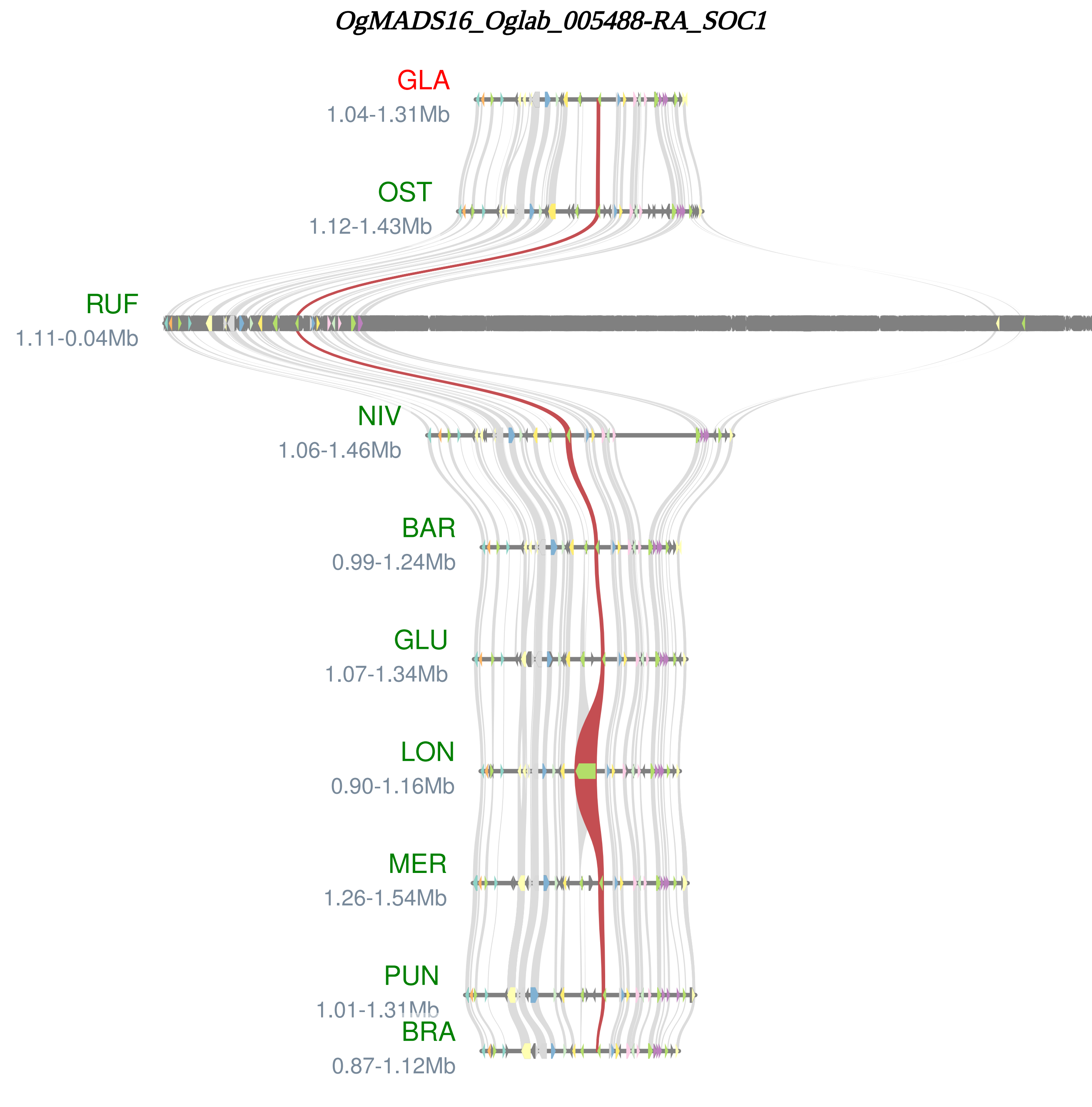
OgMADS16_Oglab_005488-RA_SOC1
GLA
1.04-1.31Mb
OST
1.12-1.43Mb
RUF
1.11-0.04Mb
NIV
1.06-1.46Mb
BAR
0.99-1.24Mb
GLU
1.07-1.34Mb
LON
0.90-1.16Mb
MER
1.26-1.54Mb
PUN
1.01-1.31Mb
BRA
0.87-1.12Mb

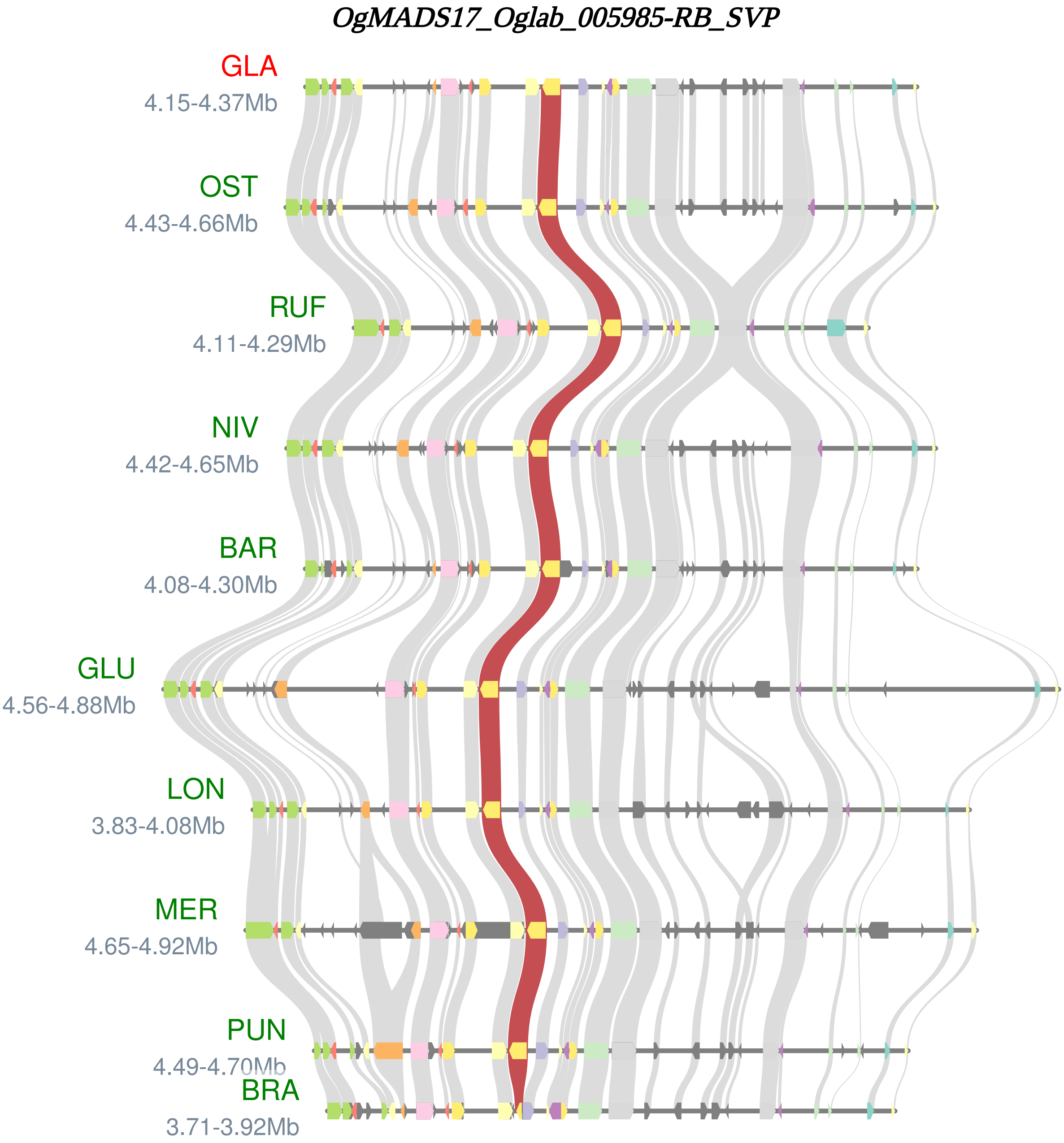
OgMADS17_Oglab_005985-RB_SVP
GLA
4.15-4.37Mb
OST
4.43-4.66Mb
RUF
4.11-4.29Mb
NIV
4.42-4.65Mb
BAR
4.08-4.30Mb
GLU
4.56-4.88Mb
LON
3.83-4.08Mb
MER
4.65-4.92Mb
PUN
4.49-4.70Mb
BRA
3.71-3.92Mb

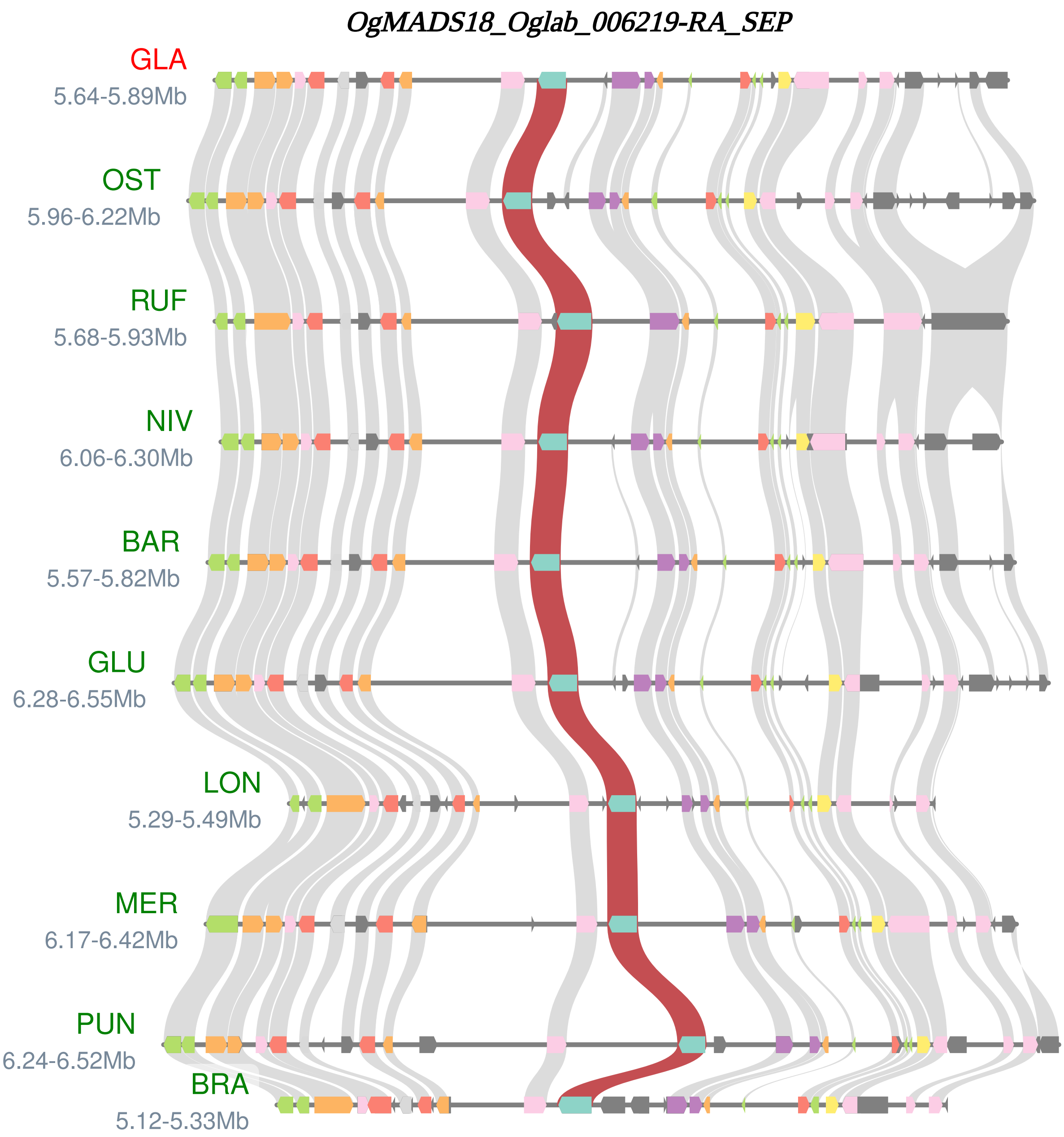
OgMADS18_Oglab_006219-RA_SEP
GLA
5.64-5.89Mb
OST
5.96-6.22Mb
RUF
5.68-5.93Mb
NIV
6.06-6.30Mb
BAR
5.57-5.82Mb
GLU
6.28-6.55Mb
LON
5.29-5.49Mb
MER
6.17-6.42Mb
PUN
6.24-6.52Mb
BRA
5.12-5.33Mb

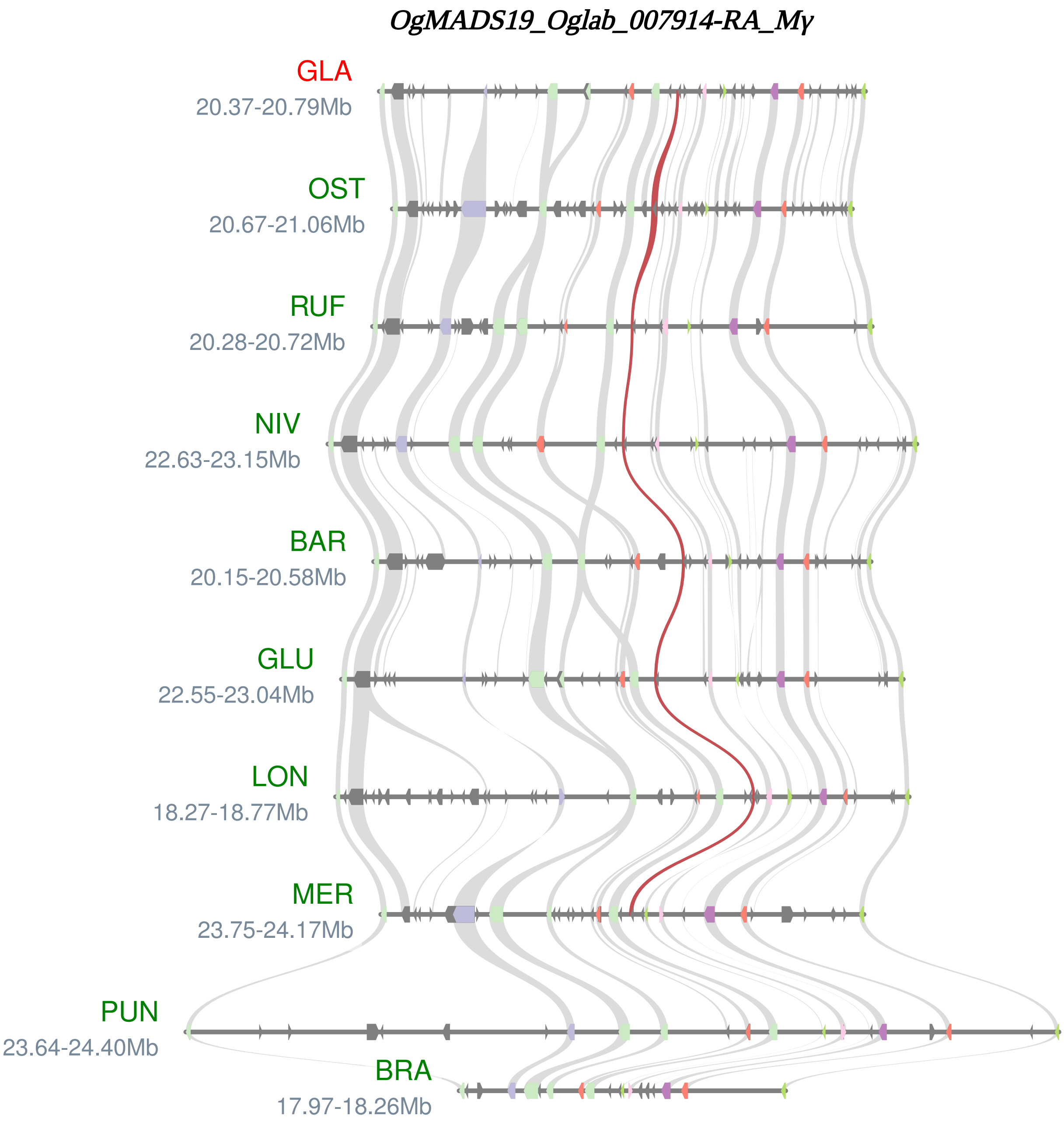
OgMADS19_Oglab_007914-RA_Mγ
GLA
20.37-20.79Mb
OST
20.67-21.06Mb
RUF
20.28-20.72Mb
NIV
22.63-23.15Mb
BAR
20.15-20.58Mb
GLU
22.55-23.04Mb
LON
18.27-18.77Mb
MER
23.75-24.17Mb
PUN
23.64-24.40Mb
BRA
17.97-18.26Mb

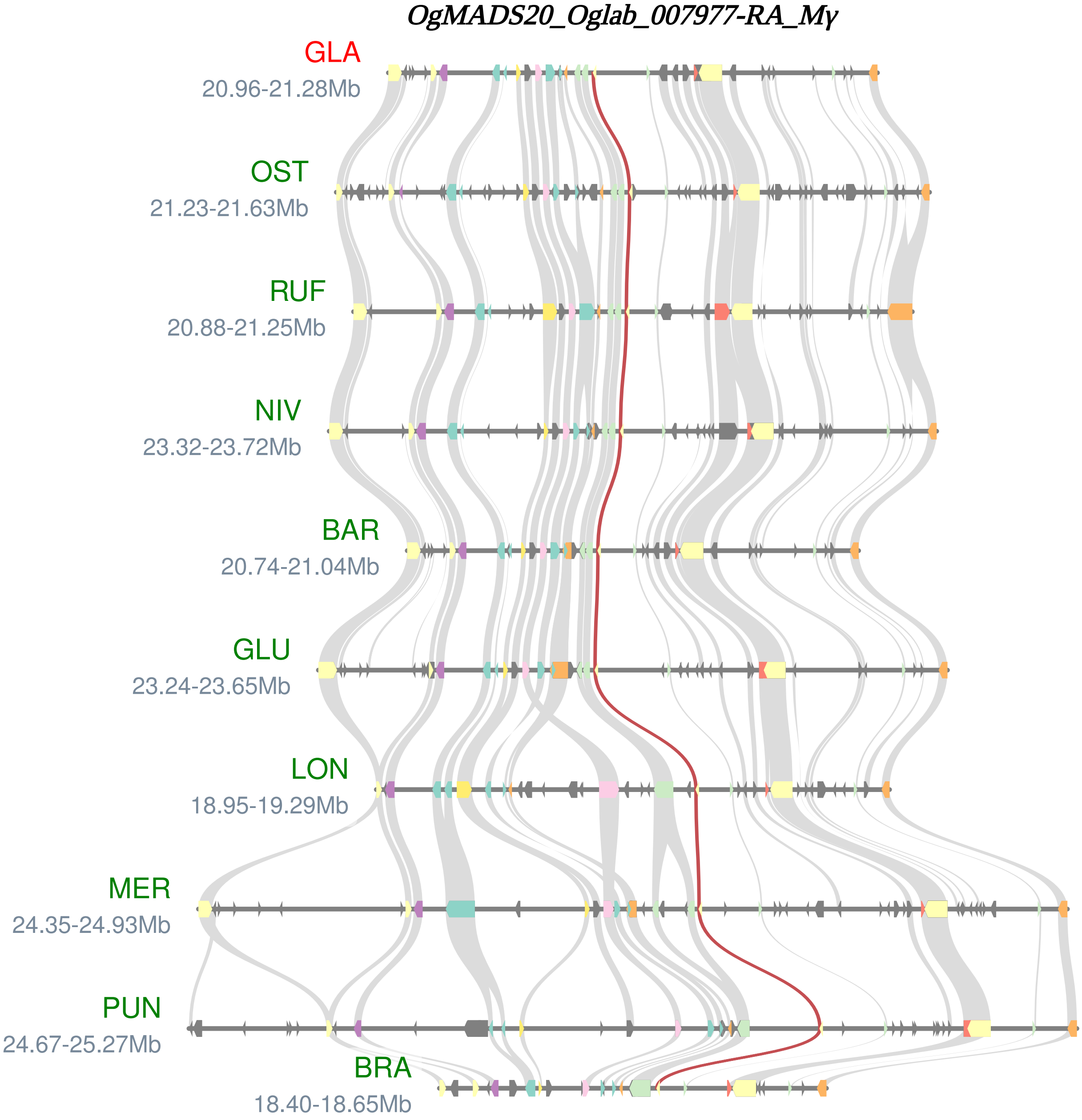

OgMADS20_Oglab_007977-RA_Mγ
GLA
20.96-21.28Mb
OST
21.23-21.63Mb
RUF
20.88-21.25Mb
NIV
23.32-23.72Mb
BAR
20.74-21.04Mb
GLU
23.24-23.65Mb
LON
18.95-19.29Mb
MER
24.35-24.93Mb
PUN
24.67-25.27Mb
BRA
18.40-18.65Mb

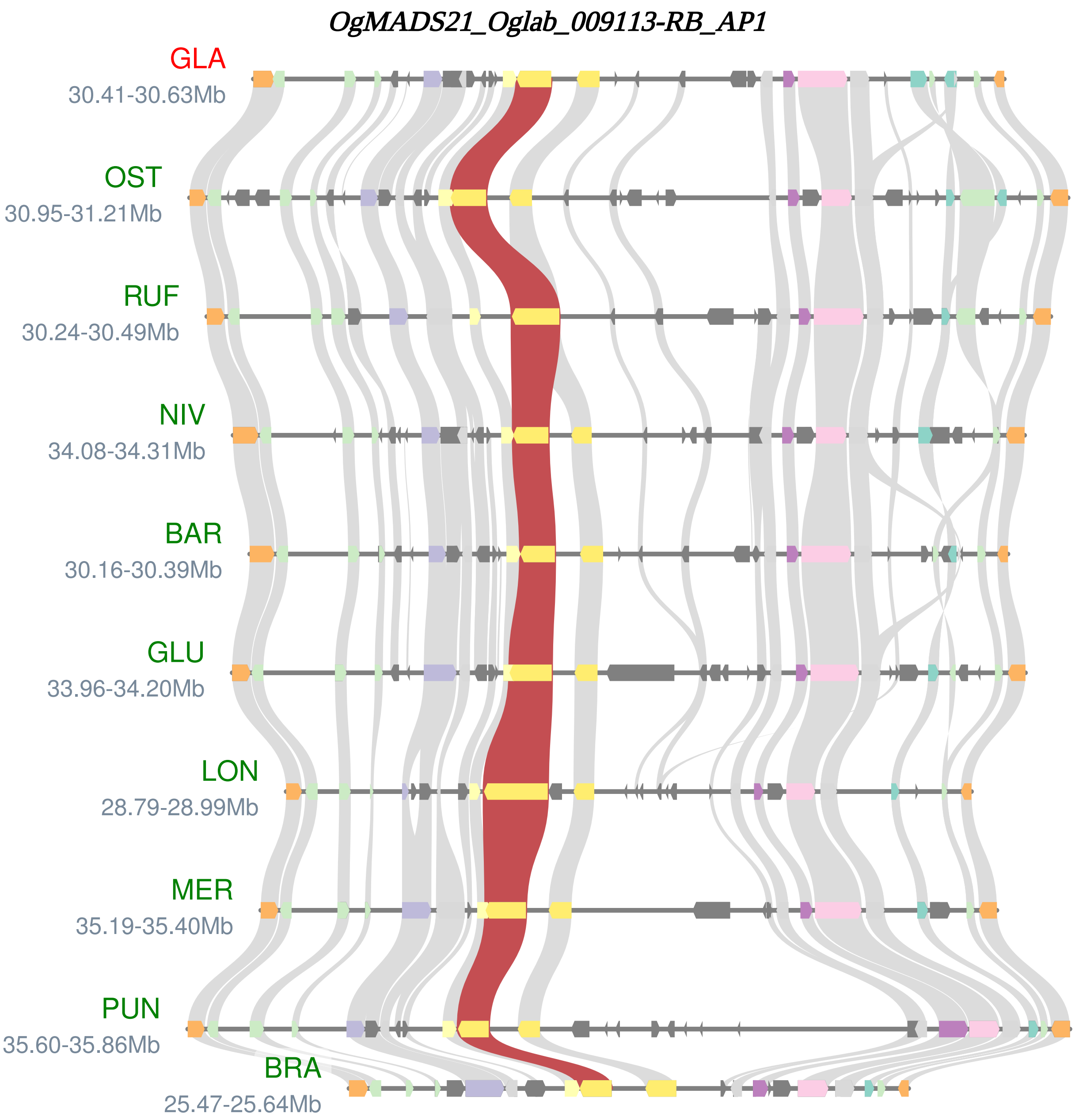

OgMADS21_Oglab_009113-RB_AP1
GLA
30.41-30.63Mb
OST
30.95-31.21Mb
RUF
30.24-30.49Mb
NIV
34.08-34.31Mb
BAR
30.16-30.39Mb
GLU
33.96-34.20Mb
LON
28.79-28.99Mb
MER
35.19-35.40Mb
PUN
35.60-35.86Mb
BRA
25.47-25.64Mb

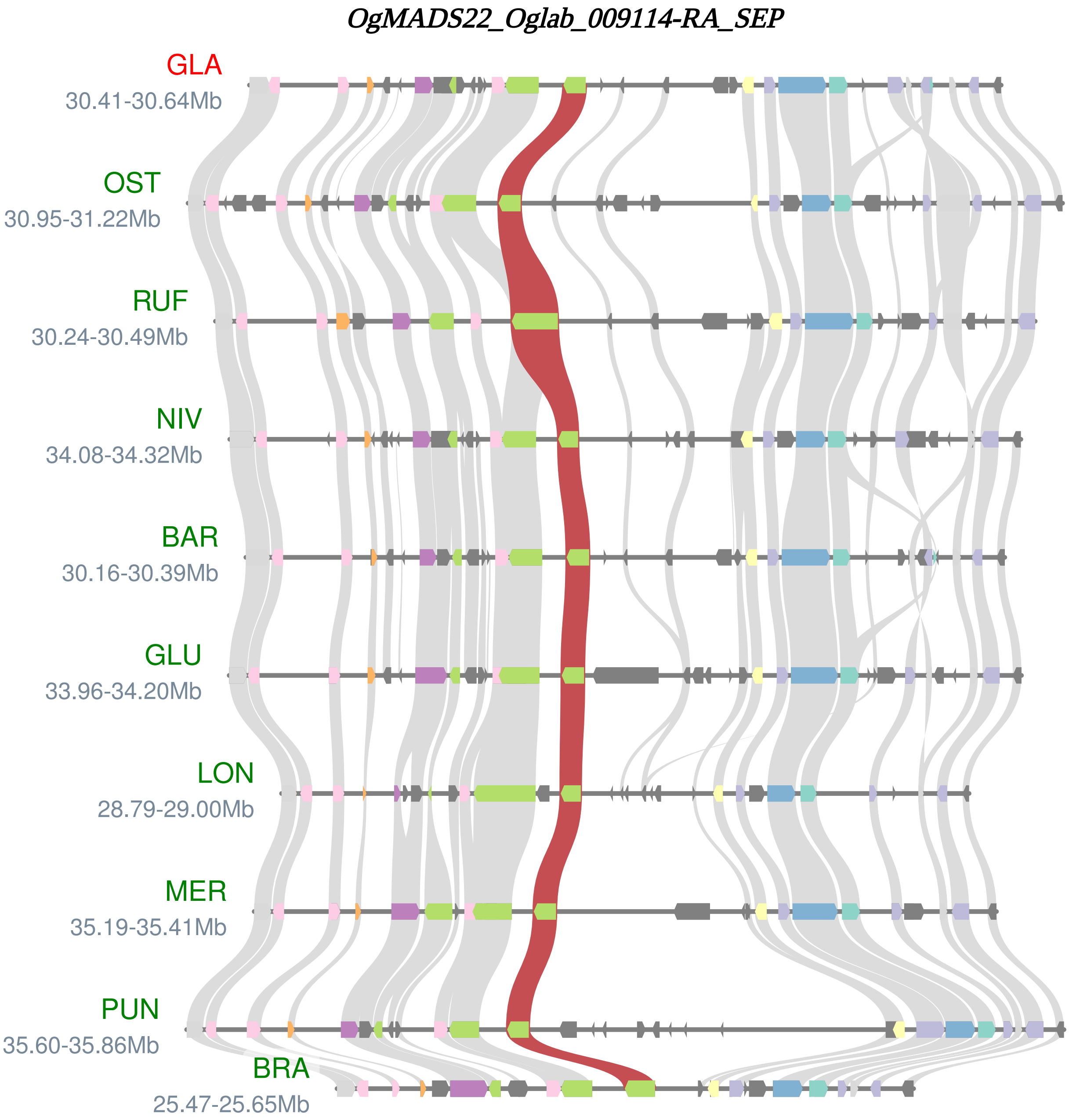

OgMADS22_Oglab_009114-RA_SEP
GLA
30.41-30.64Mb
OST
30.95-31.22Mb
RUF
30.24-30.49Mb
NIV
34.08-34.32Mb
BAR
30.16-30.39Mb
GLU
33.96-34.20Mb
LON
28.79-29.00Mb
MER
35.19-35.41Mb
PUN
35.60-35.86Mb
BRA
25.47-25.65Mb

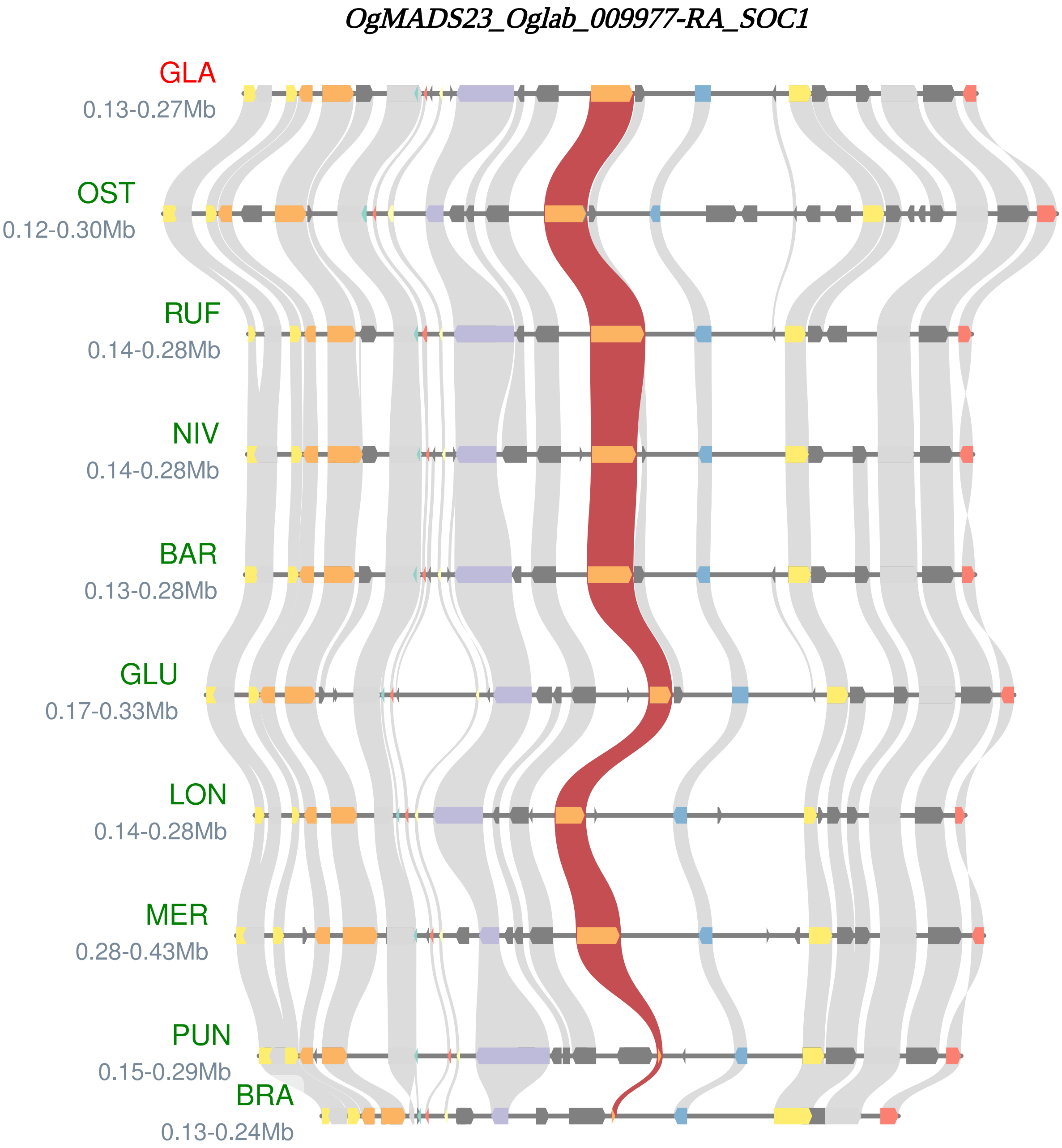
OgMADS23_Oglab_009977-RA_SOC1
GLA
0.13-0.27Mb
OST
0.12-0.30Mb
RUF
0.14-0.28Mb
NIV
0.14-0.28Mb
BAR
0.13-0.28Mb
GLU
0.17-0.33Mb
LON
0.14-0.28Mb
MER
0.28-0.43Mb
PUN
0.15-0.29Mb
BRA
0.13-0.24Mb

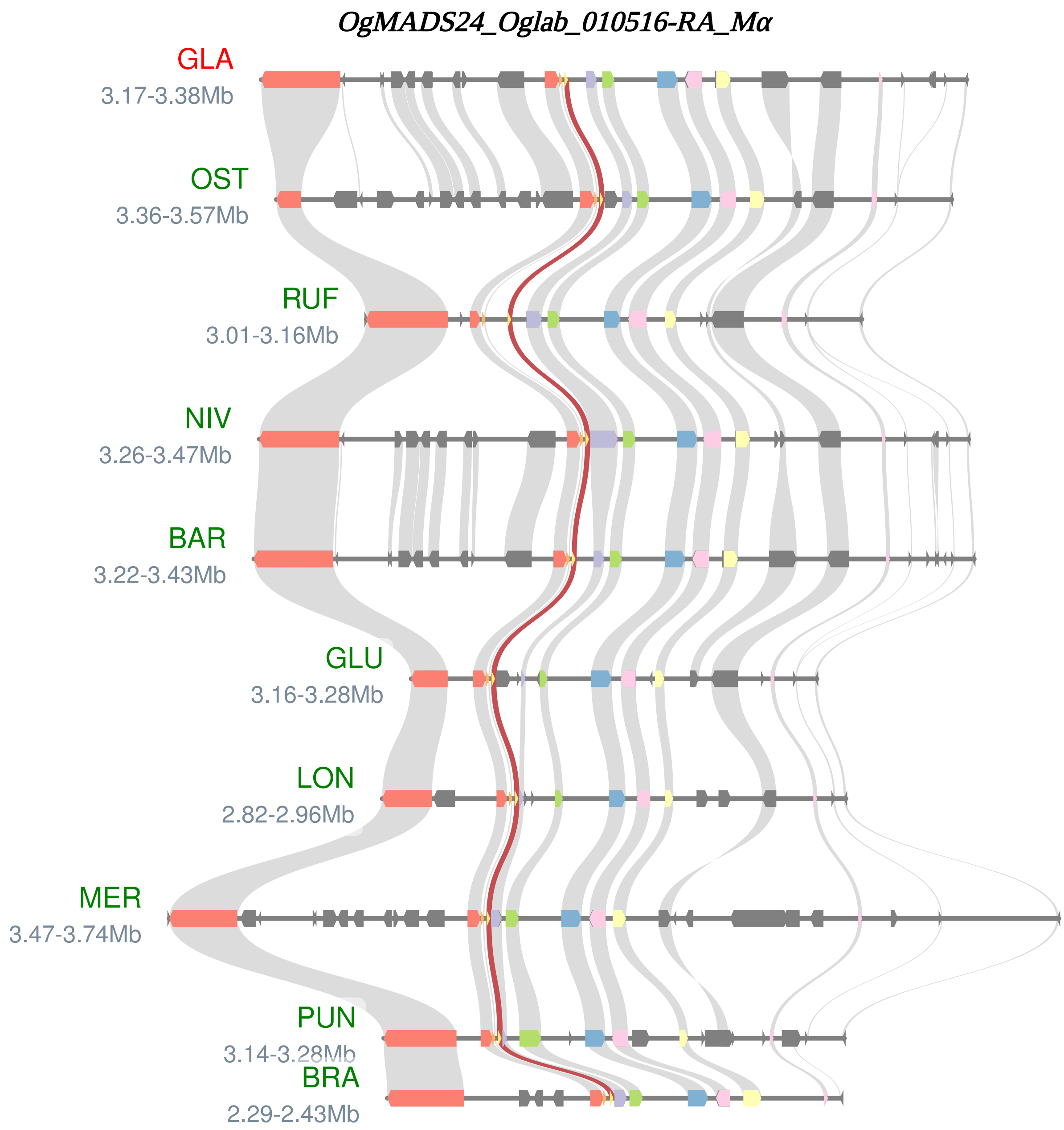
OgMADS24_Oglab_010516-RA_Mα
GLA
3.17-3.38Mb
OST
3.36-3.57Mb
RUF
3.01-3.16Mb
NIV
3.26-3.47Mb
BAR
3.22-3.43Mb
GLU
3.16-3.28Mb
LON
2.82-2.96Mb
MER
3.47-3.74Mb
PUN
3.14-3.28Mb
BRA
2.29-2.43Mb

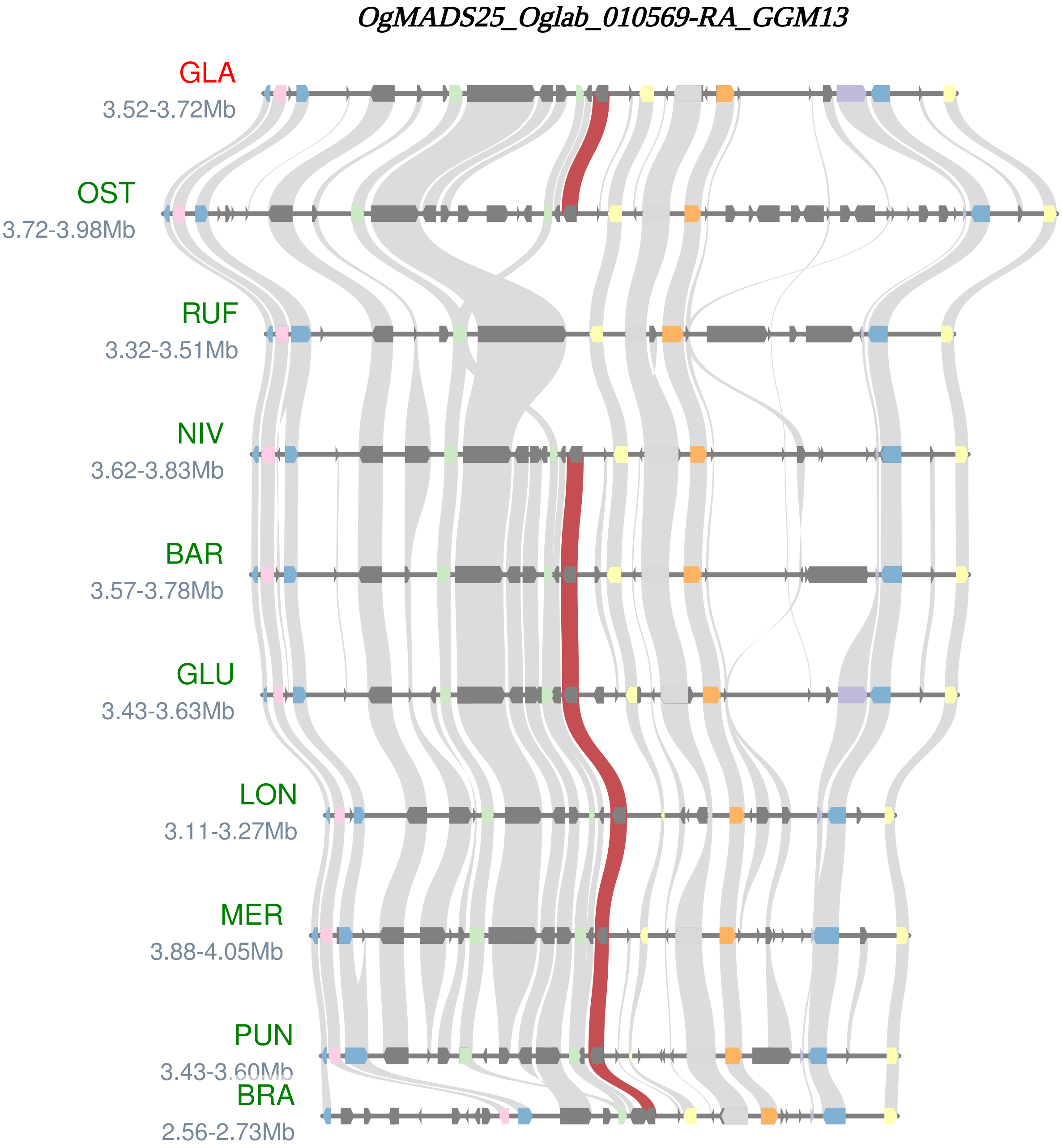
OgMADS25_Oglab_010569-RA_GGM13
GLA
3.52-3.72Mb
OST
3.72-3.98Mb
RUF
3.32-3.51Mb
NIV
3.62-3.83Mb
BAR
3.57-3.78Mb
GLU
3.43-3.63Mb
LON
3.11-3.27Mb
MER
3.88-4.05Mb
PUN
3.43-3.60Mb
BRA
2.56-2.73Mb

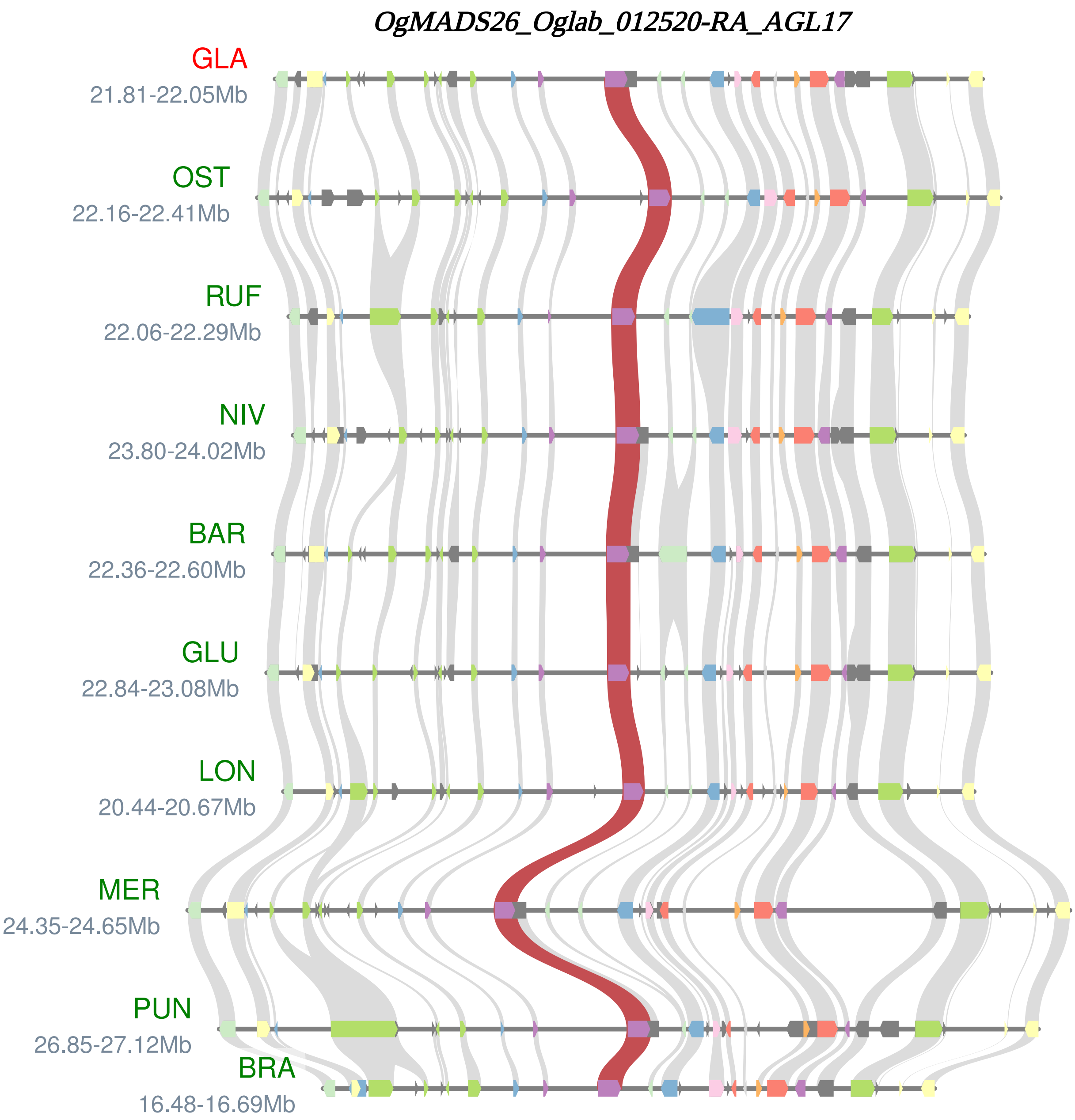

OgMADS26_Oglab_012520-RA_AGL17
GLA
21.81-22.05Mb
OST
22.16-22.41Mb
RUF
22.06-22.29Mb
NIV
23.80-24.02Mb
BAR
22.36-22.60Mb
GLU
22.84-23.08Mb
LON
20.44-20.67Mb
MER
24.35-24.65Mb
PUN
26.85-27.12Mb
BRA
16.48-16.69Mb

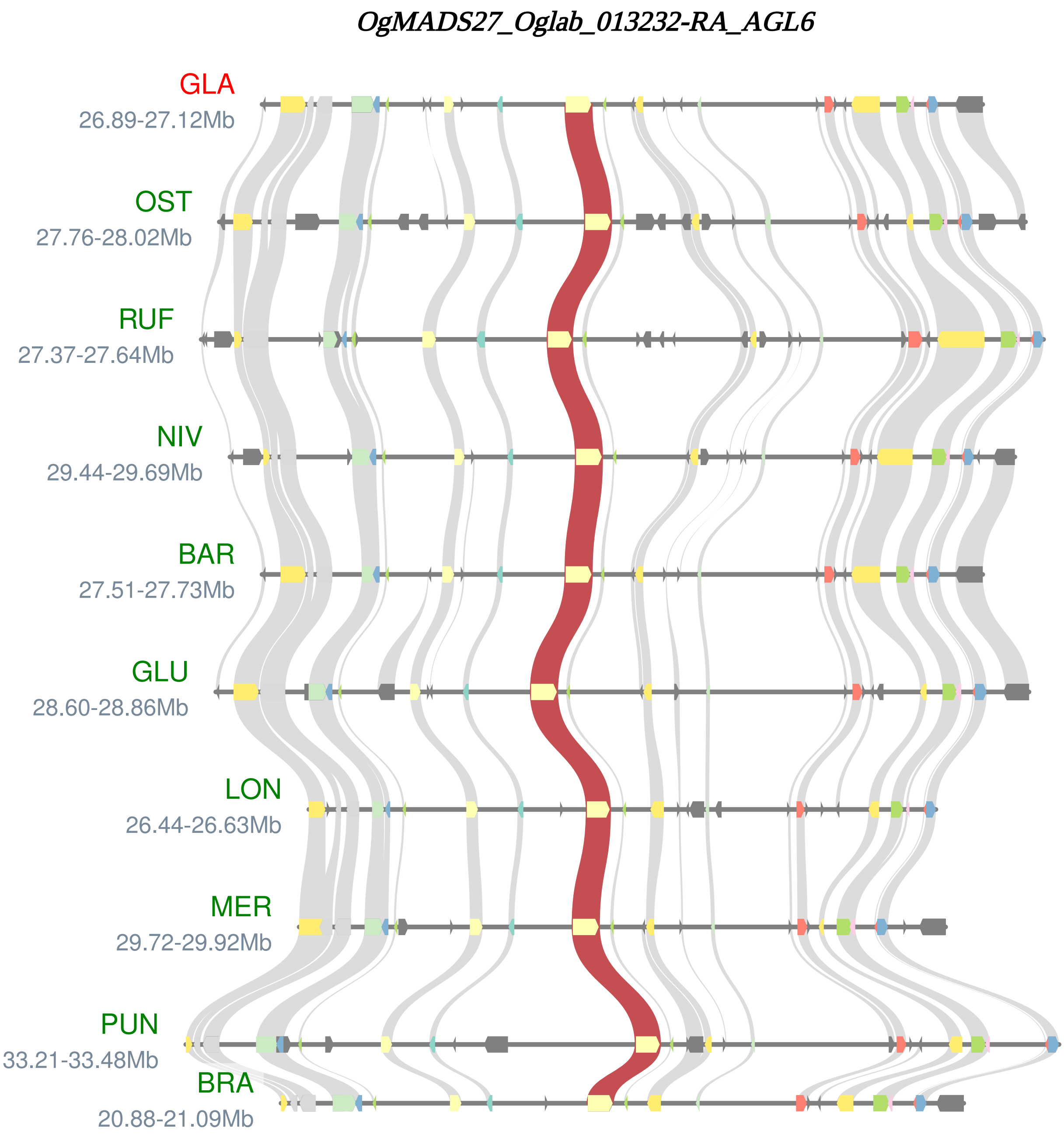
OgMADS27_Oglab_013232-RA_AGL6
GLA
26.89-27.12Mb
OST
27.76-28.02Mb
RUF
27.37-27.64Mb
NIV
29.44-29.69Mb
BAR
27.51-27.73Mb
GLU
28.60-28.86Mb
LON
26.44-26.63Mb
MER
29.72-29.92Mb
PUN
33.21-33.48Mb
BRA
20.88-21.09Mb

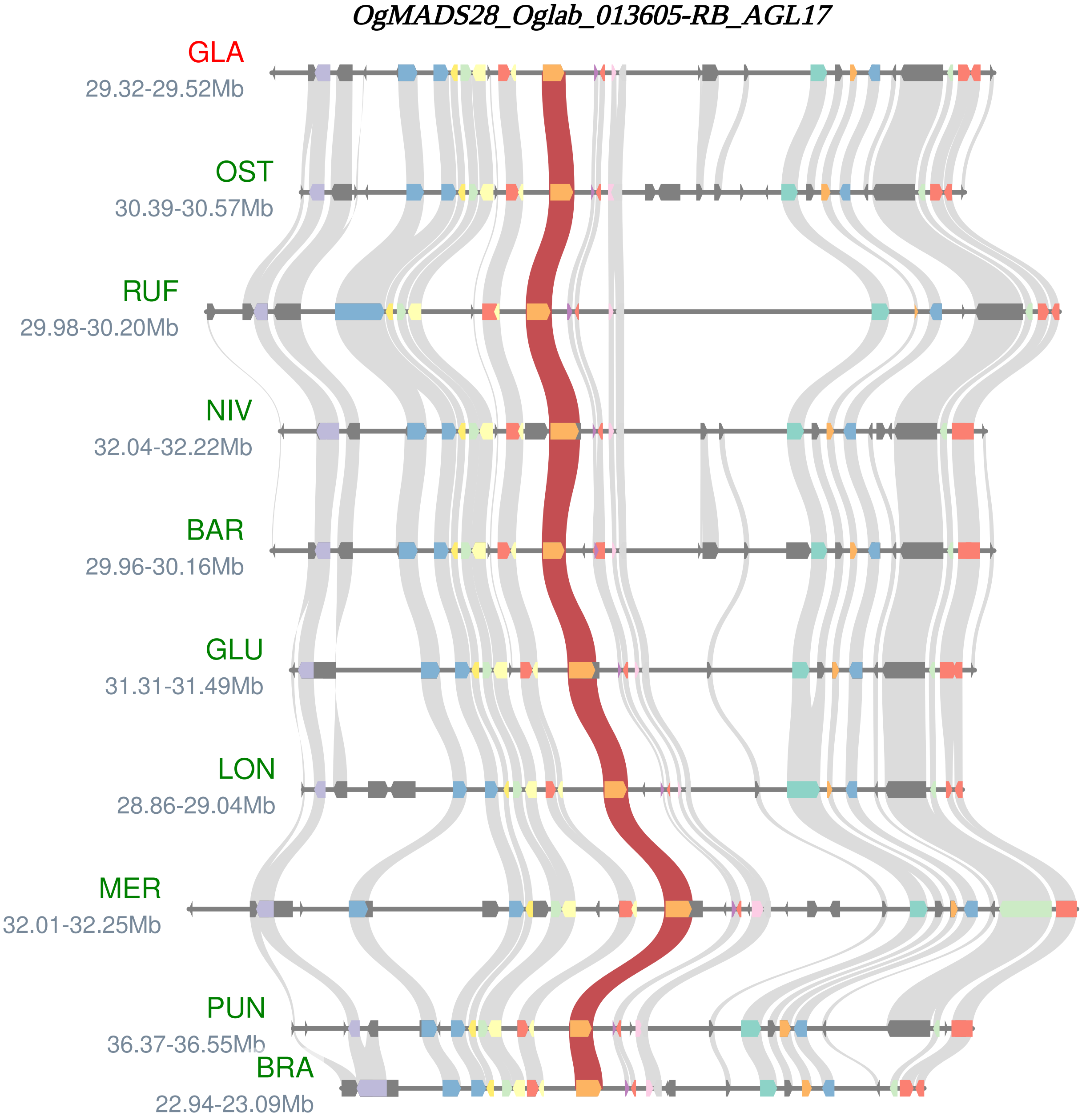
OgMADS28_Oglab_013605-RB_AGL17
GLA
29.32-29.52Mb
OST
30.39-30.57Mb
RUF
29.98-30.20Mb
NIV
32.04-32.22Mb
BAR
29.96-30.16Mb
GLU
31.31-31.49Mb
LON
28.86-29.04Mb
MER
32.01-32.25Mb
PUN
36.37-36.55Mb
BRA
22.94-23.09Mb

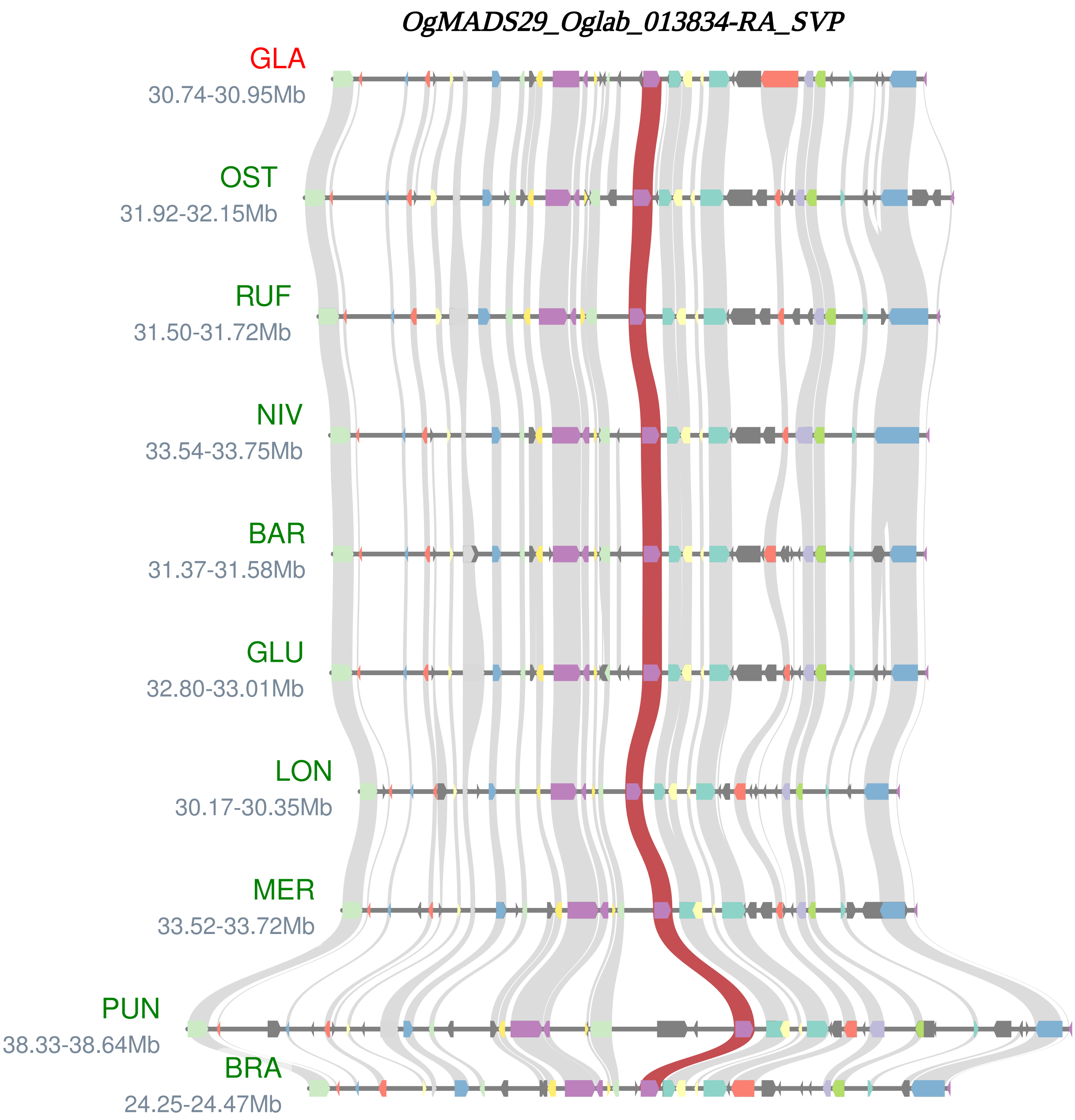
OgMADS29_Oglab_013834-RA_SVP
GLA
30.74-30.95Mb
OST
31.92-32.15Mb
RUF
31.50-31.72Mb
NIV
33.54-33.75Mb
BAR
31.37-31.58Mb
GLU
32.80-33.01Mb
LON
30.17-30.35Mb
MER
33.52-33.72Mb
PUN
38.33-38.64Mb
BRA
24.25-24.47Mb

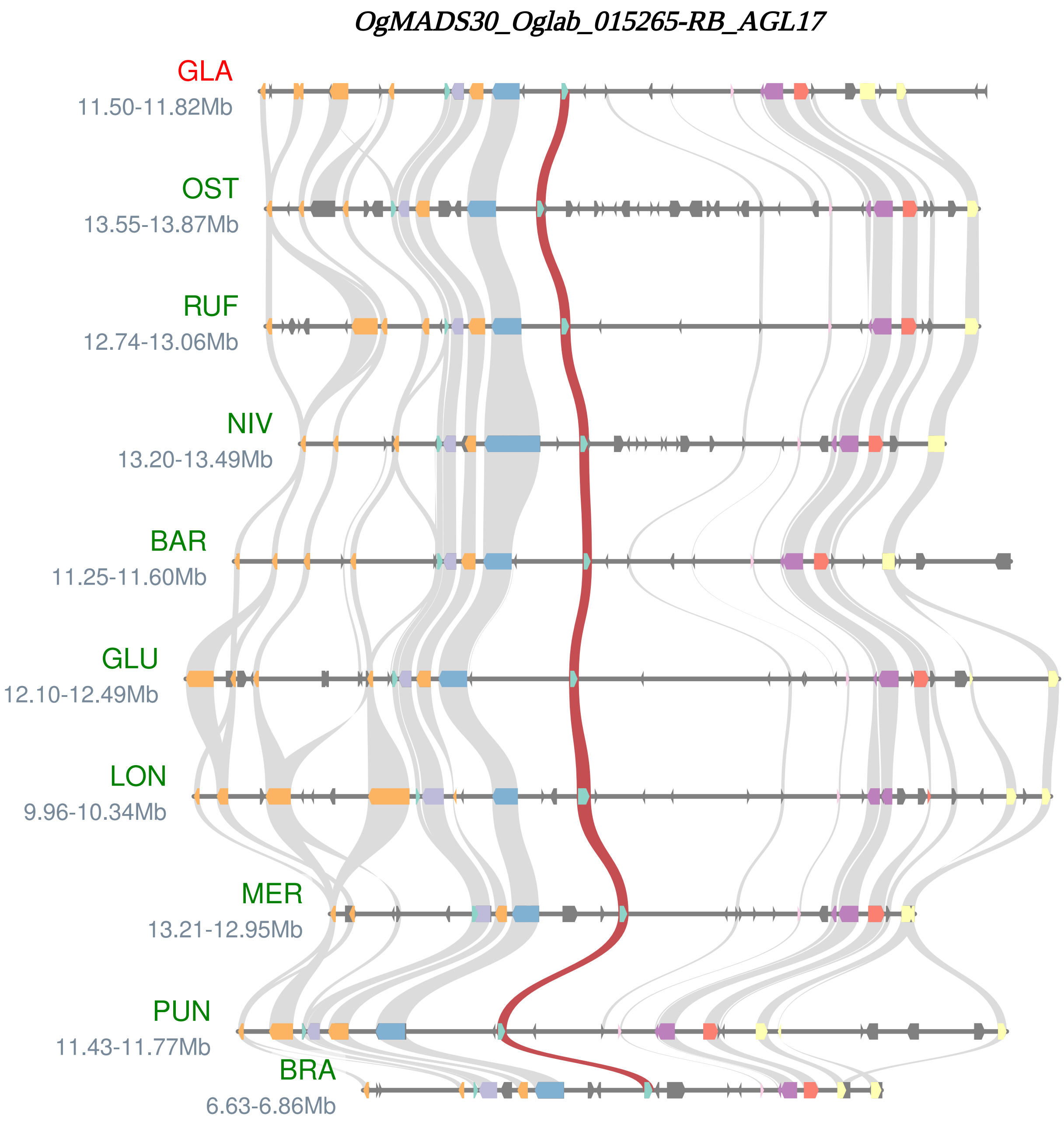
OgMADS30_Oglab_015265-RB_AGL17
GLA
11.50-11.82Mb
OST
13.55-13.87Mb
RUF
12.74-13.06Mb
NIV
13.20-13.49Mb
BAR
11.25-11.60Mb
GLU
12.10-12.49Mb
LON
9.96-10.34Mb
MER
13.21-12.95Mb
PUN
11.43-11.77Mb
BRA
6.63-6.86Mb

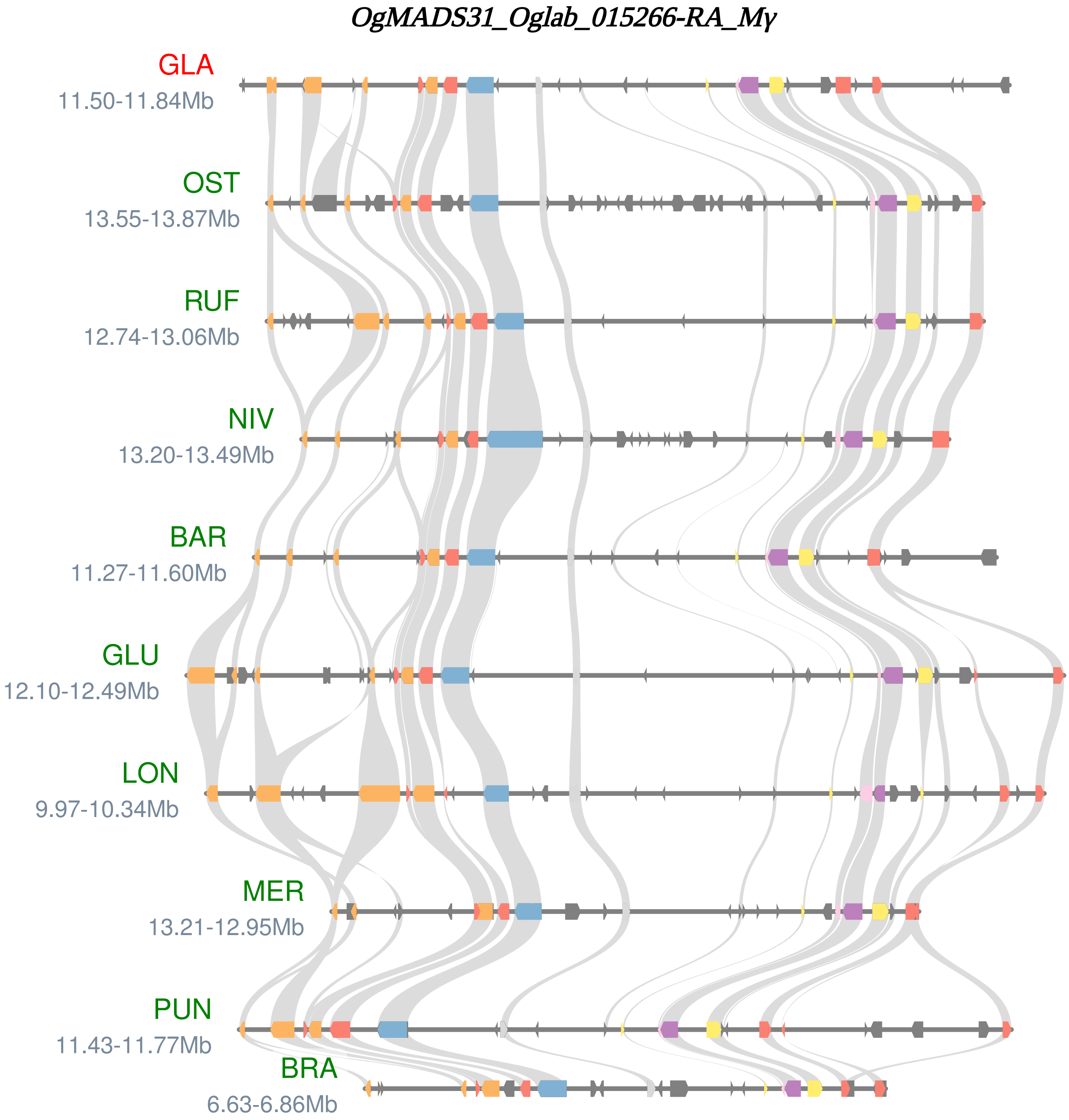
OgMADS31_Oglab_015266-RA_Mγ
GLA
11.50-11.84Mb
OST
13.55-13.87Mb
RUF
12.74-13.06Mb
NIV
13.20-13.49Mb
BAR
11.27-11.60Mb
GLU
12.10-12.49Mb
LON
9.97-10.34Mb
MER
13.21-12.95Mb
PUN
11.43-11.77Mb
BRA
6.63-6.86Mb

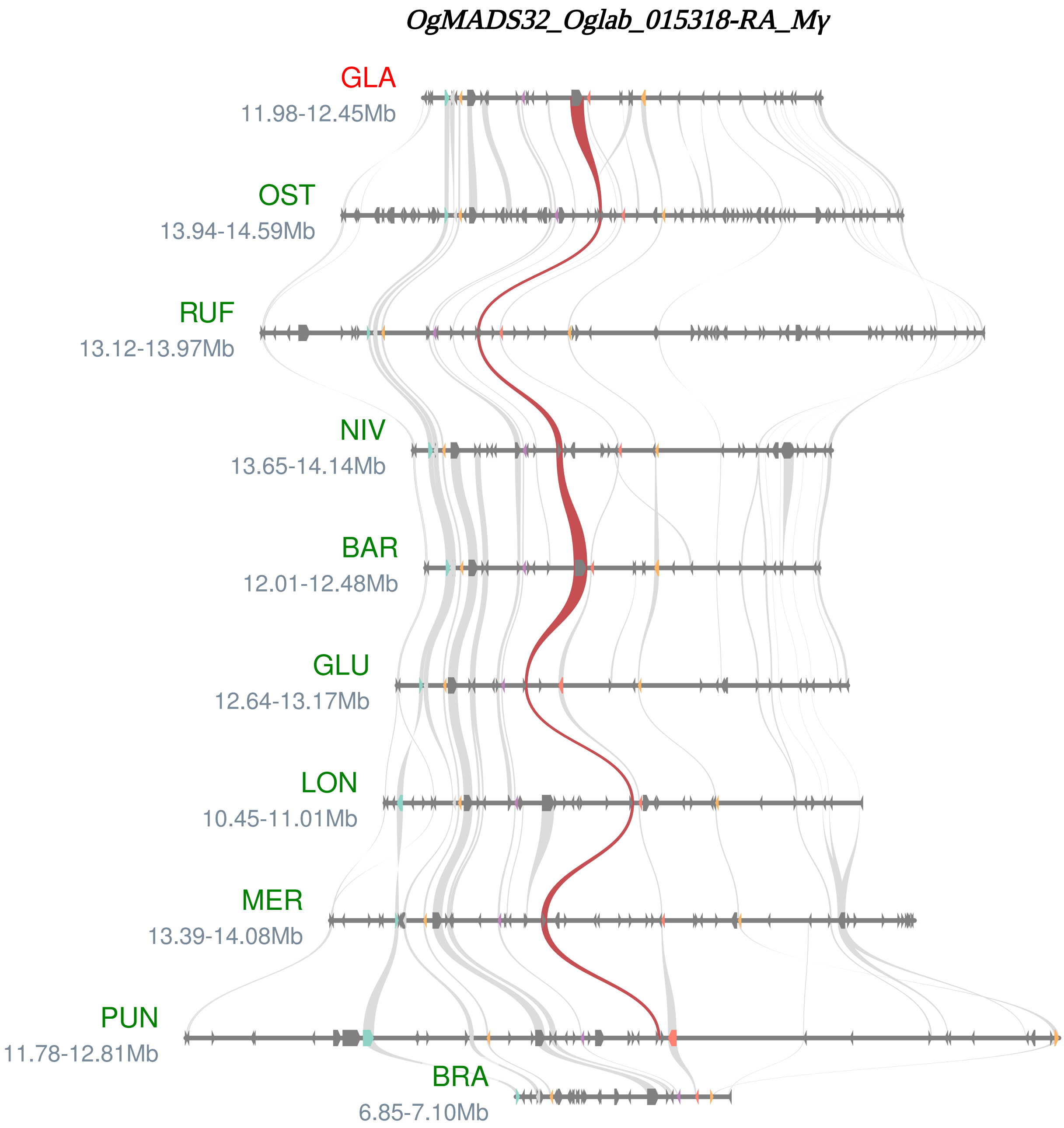
OgMADS32_Oglab_015318-RA_Mγ
GLA
11.98-12.45Mb
OST
13.94-14.59Mb
RUF
13.12-13.97Mb
NIV
13.65-14.14Mb
BAR
12.01-12.48Mb
GLU
12.64-13.17Mb
LON
10.45-11.01Mb
MER
13.39-14.08Mb
PUN
11.78-12.81Mb
BRA
6.85-7.10Mb

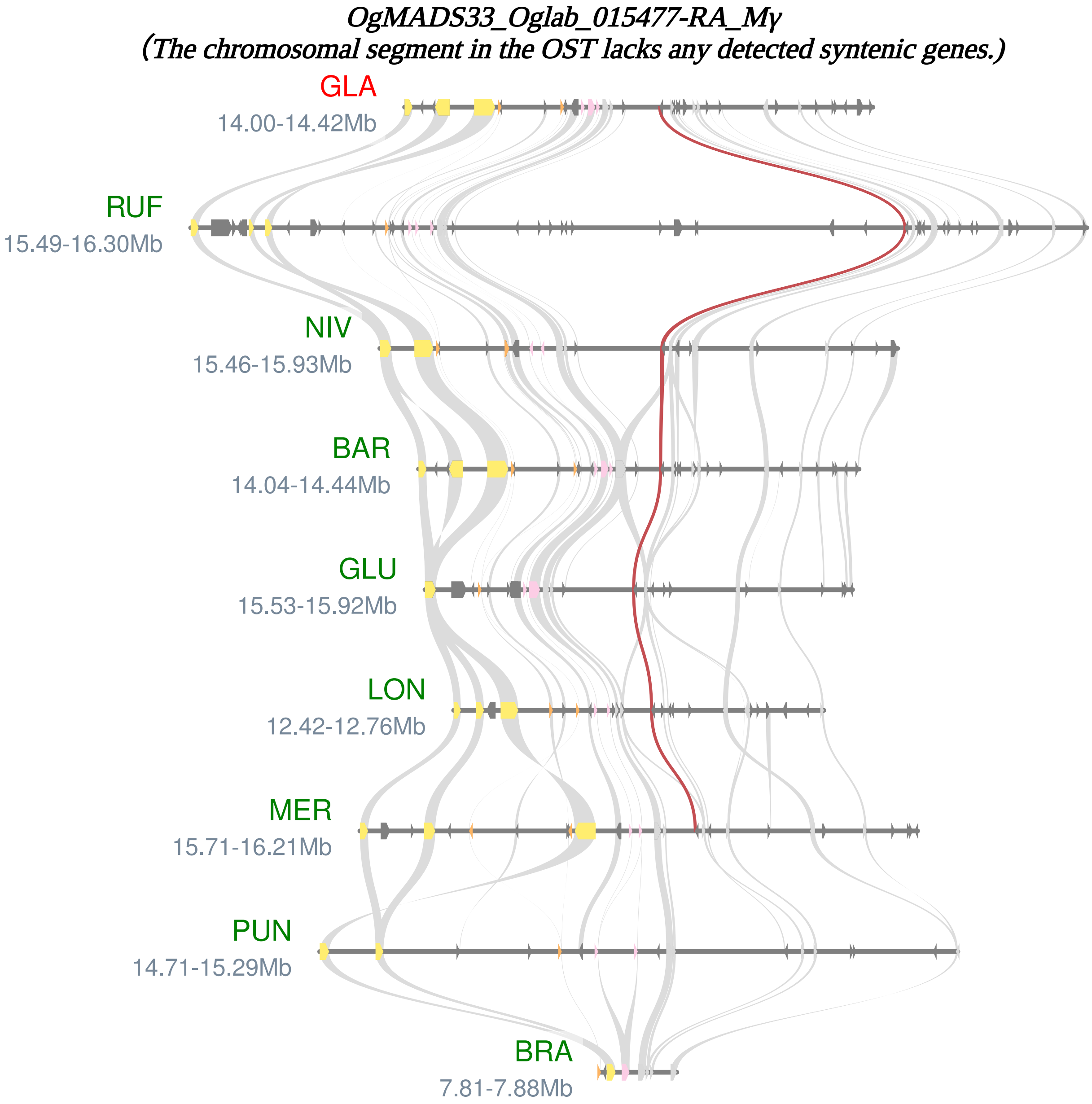

OgMADS33_Oglab_015477-RA_Mγ
(The chromosomal segment in the OST lacks any detected syntenic genes.)
GLA
14.00-14.42Mb
RUF
15.49-16.30Mb
NIV
15.46-15.93Mb
BAR
14.04-14.44Mb
GLU
15.53-15.92Mb
LON
12.42-12.76Mb
MER
15.71-16.21Mb
PUN
14.71-15.29Mb
BRA
7.81-7.88Mb

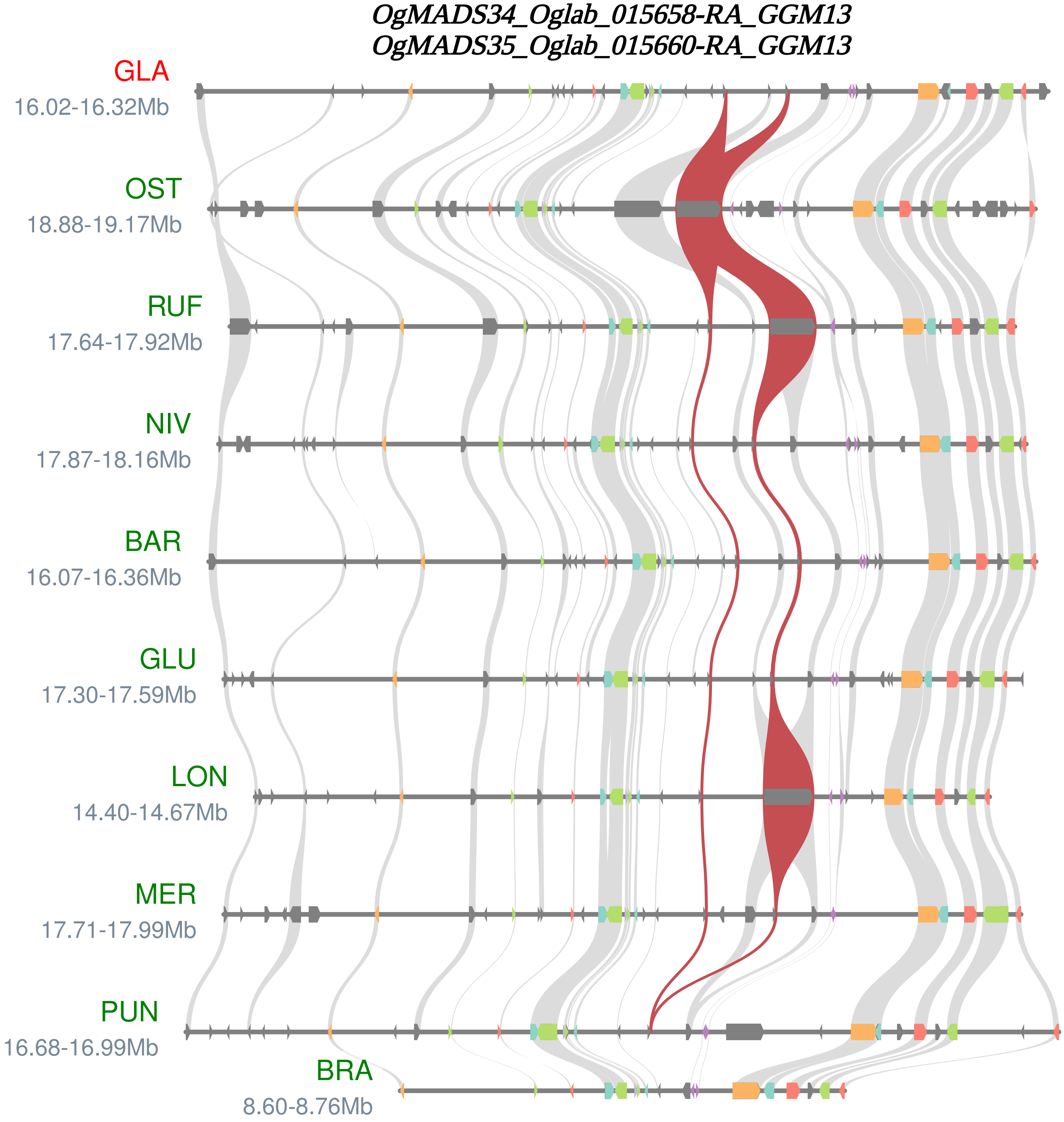
OgMADS34_Oglab_015658-RA_GGM13
OgMADS35_Oglab_015660-RA_GGM13
GLA
16.02-16.32Mb
OST
18.88-19.17Mb
RUF
17.64-17.92Mb
NIV
17.87-18.16Mb
BAR
16.07-16.36Mb
GLU
17.30-17.59Mb
LON
14.40-14.67Mb
MER
17.71-17.99Mb
PUN
16.68-16.99Mb
BRA
8.60-8.76Mb

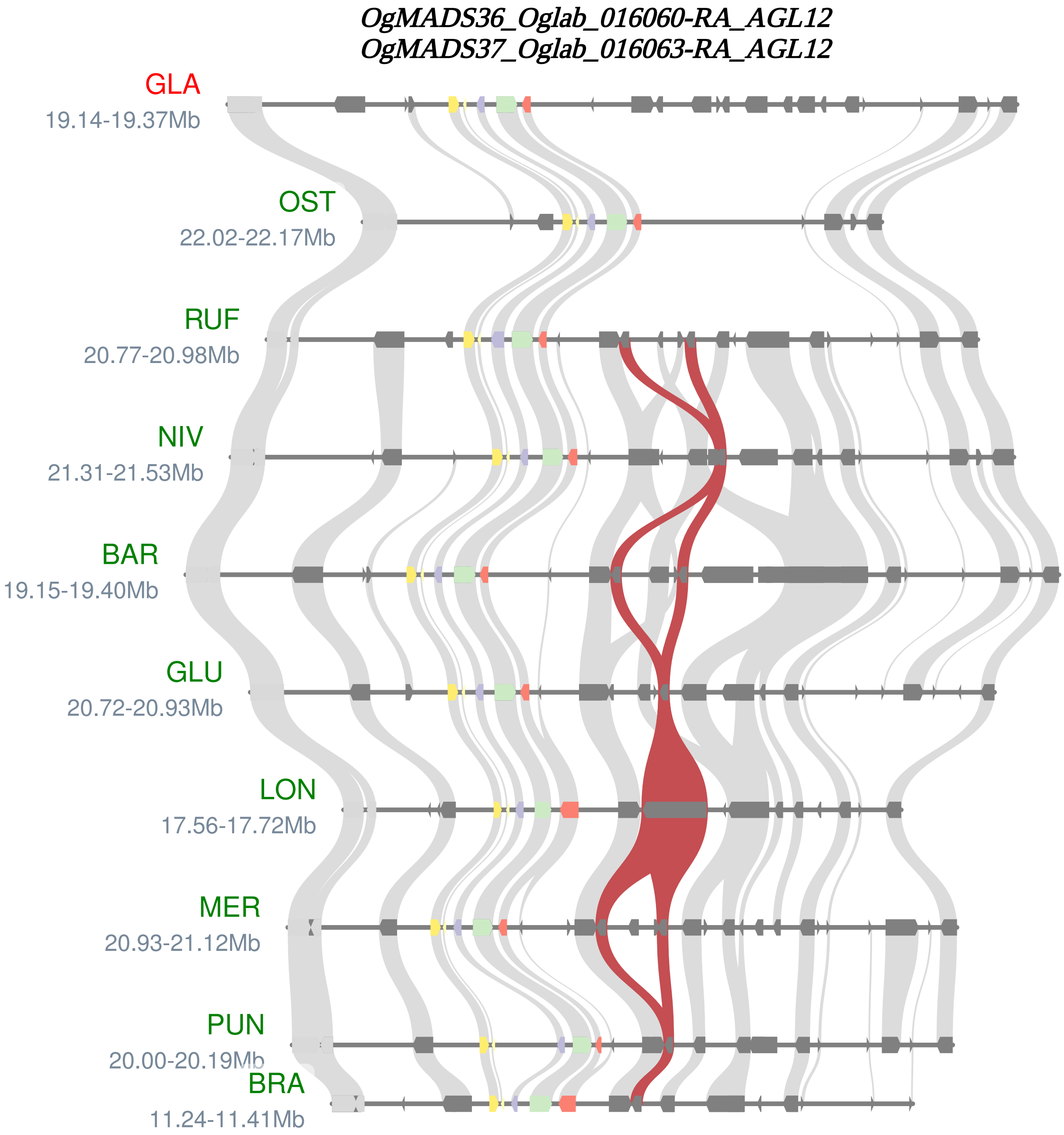
OgMADS36_Oglab_016060-RA_AGL12
OgMADS37_Oglab_016063-RA_AGL12
GLA
19.14-19.37Mb
OST
22.02-22.17Mb
RUF
20.77-20.98Mb
NIV
21.31-21.53Mb
BAR
19.15-19.40Mb
GLU
20.72-20.93Mb
LON
17.56-17.72Mb
MER
20.93-21.12Mb
PUN
20.00-20.19Mb
BRA
11.24-11.41Mb

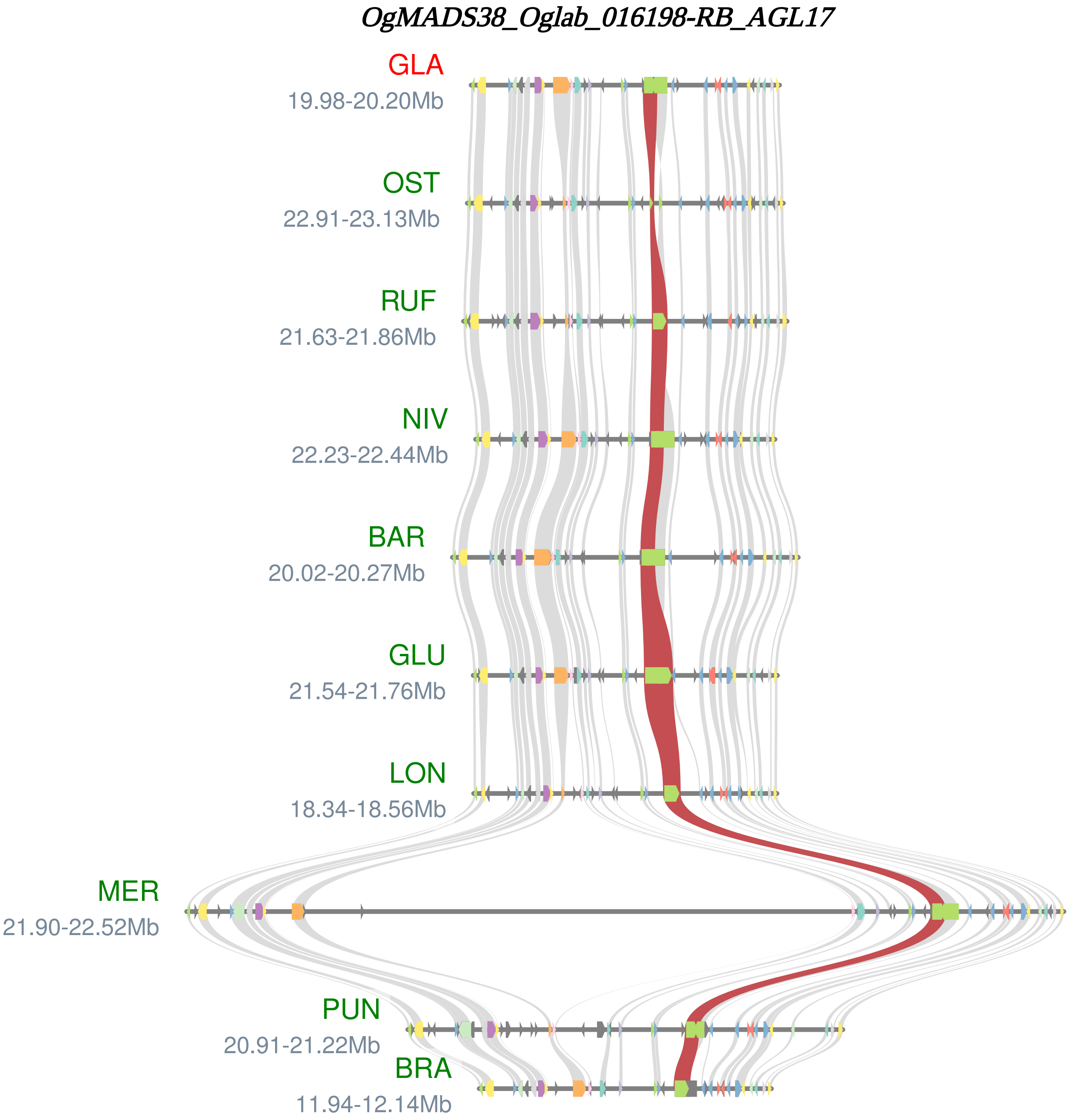
OgMADS38_Oglab_016198-RB_AGL17
GLA
19.98-20.20Mb
OST
22.91-23.13Mb
RUF
21.63-21.86Mb
NIV
22.23-22.44Mb
BAR
20.02-20.27Mb
GLU
21.54-21.76Mb
LON
18.34-18.56Mb
MER
21.90-22.52Mb
PUN
20.91-21.22Mb
BRA
11.94-12.14Mb

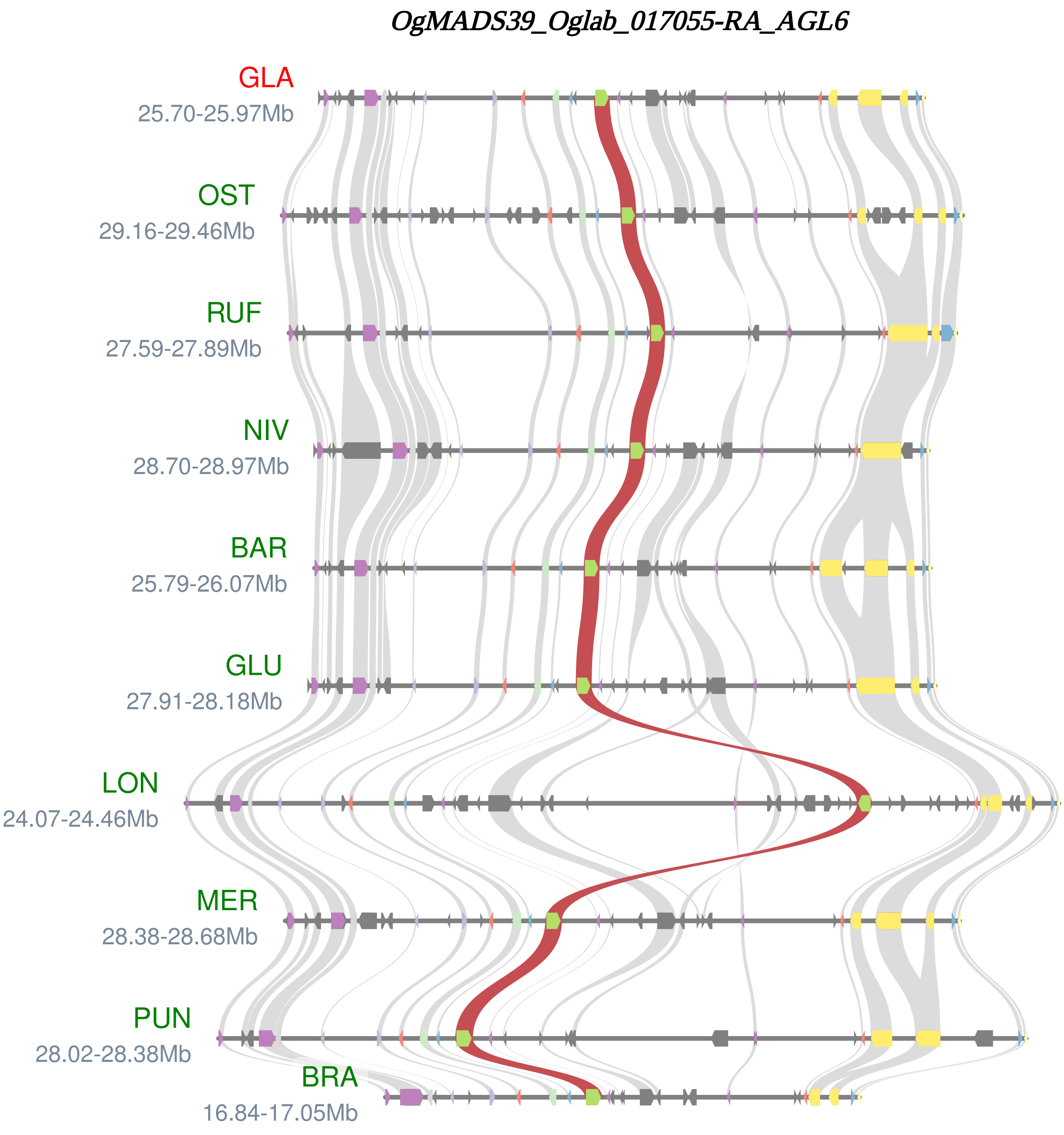

OgMADS39_Oglab_017055-RA_AGL6
GLA
25.70-25.97Mb
OST
29.16-29.46Mb
RUF
27.59-27.89Mb
NIV
28.70-28.97Mb
BAR
25.79-26.07Mb
GLU
27.91-28.18Mb
LON
24.07-24.46Mb
MER
28.38-28.68Mb
PUN
28.02-28.38Mb
BRA
16.84-17.05Mb

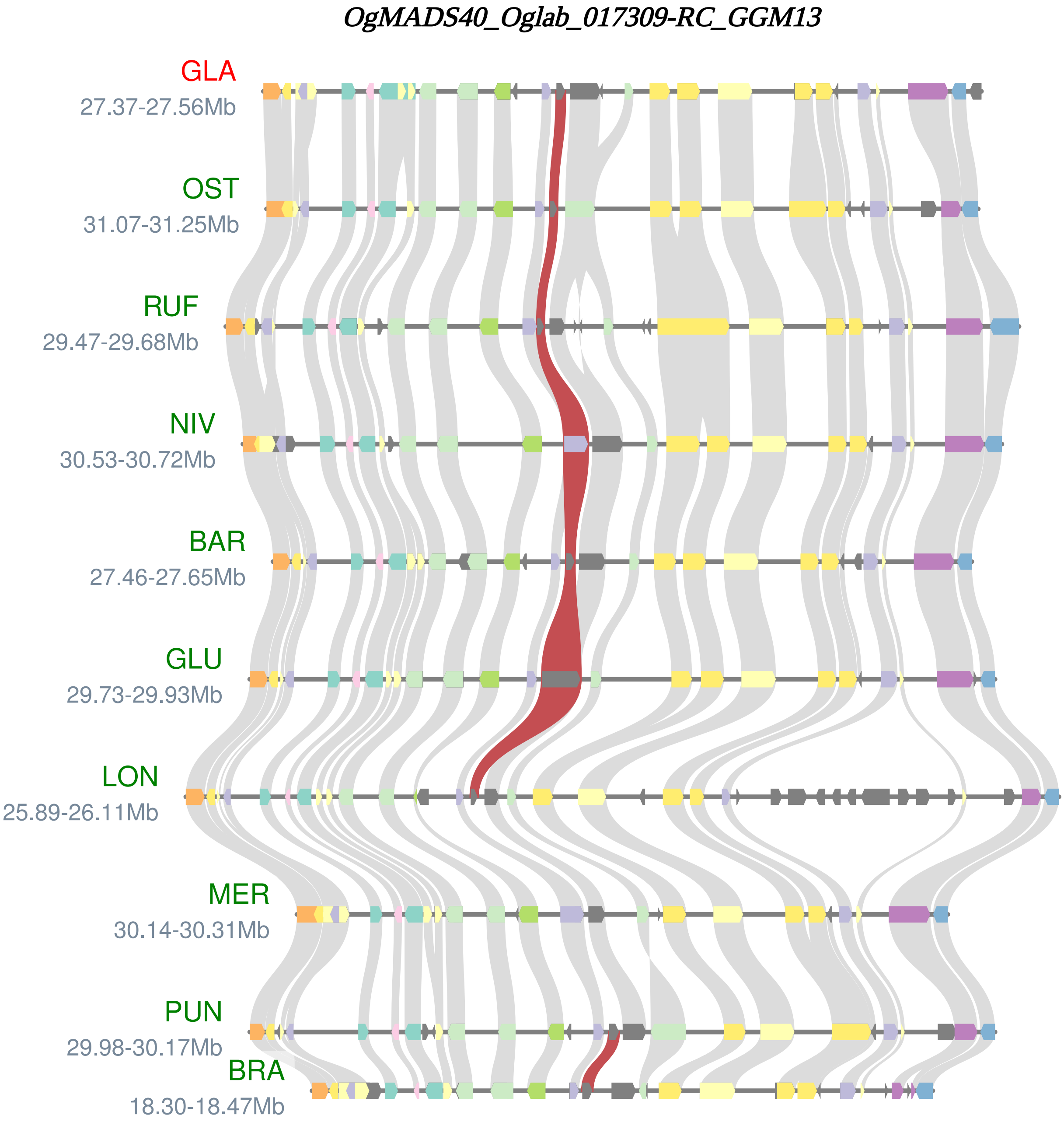
OgMADS40_Oglab_017309-RC_GGM13
GLA
27.37-27.56Mb
OST
31.07-31.25Mb
RUF
29.47-29.68Mb
NIV
30.53-30.72Mb
BAR
27.46-27.65Mb
GLU
29.73-29.93Mb
LON
25.89-26.11Mb
MER
30.14-30.31Mb
PUN
29.98-30.17Mb
BRA
18.30-18.47Mb

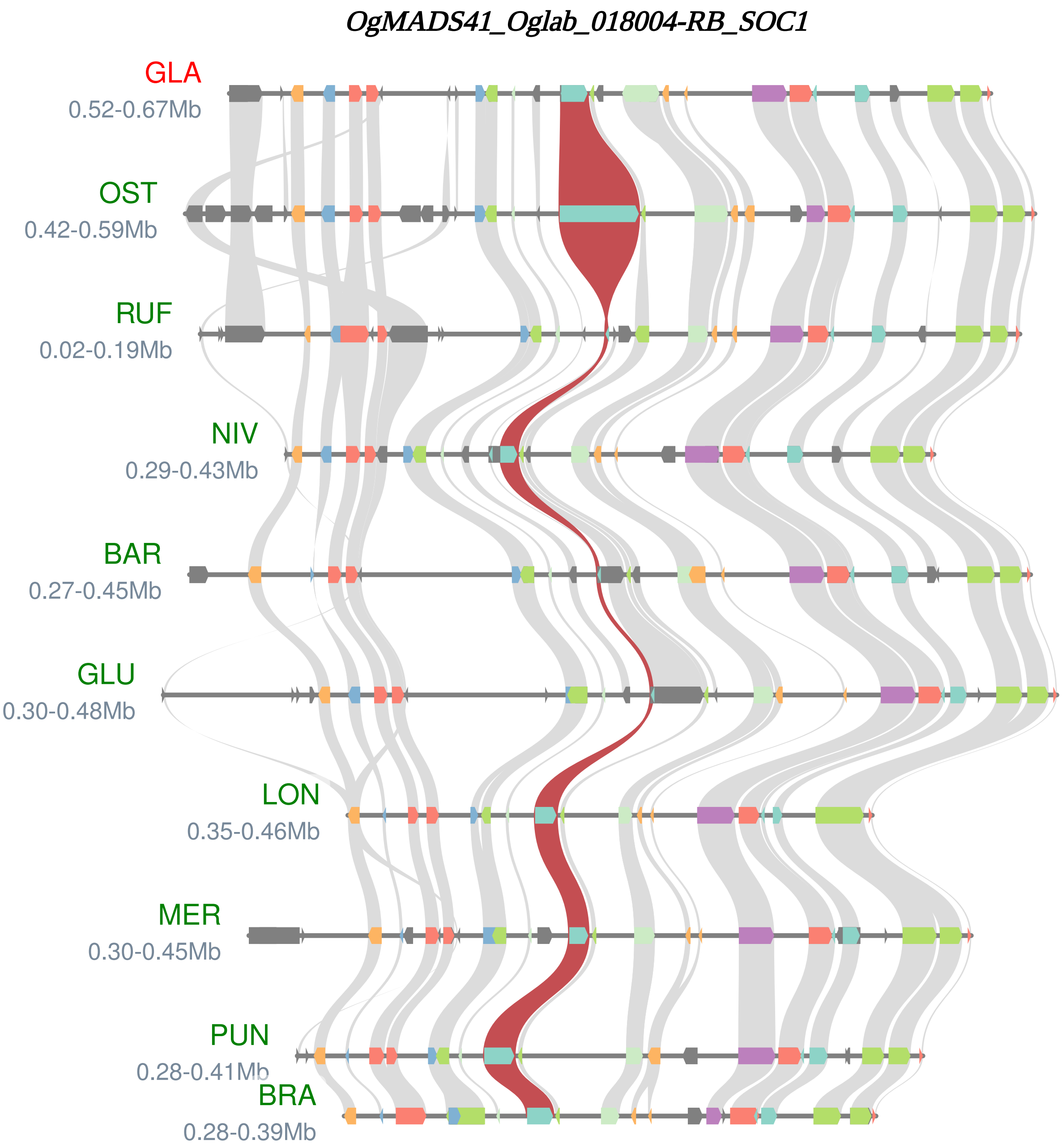
OgMADS41_Oglab_018004-RB_SOC1
GLA
0.52-0.67Mb
OST
0.42-0.59Mb
RUF
0.02-0.19Mb
NIV
0.29-0.43Mb
BAR
0.27-0.45Mb
GLU
0.30-0.48Mb
LON
0.35-0.46Mb
MER
0.30-0.45Mb
PUN
0.28-0.41Mb
BRA
0.28-0.39Mb

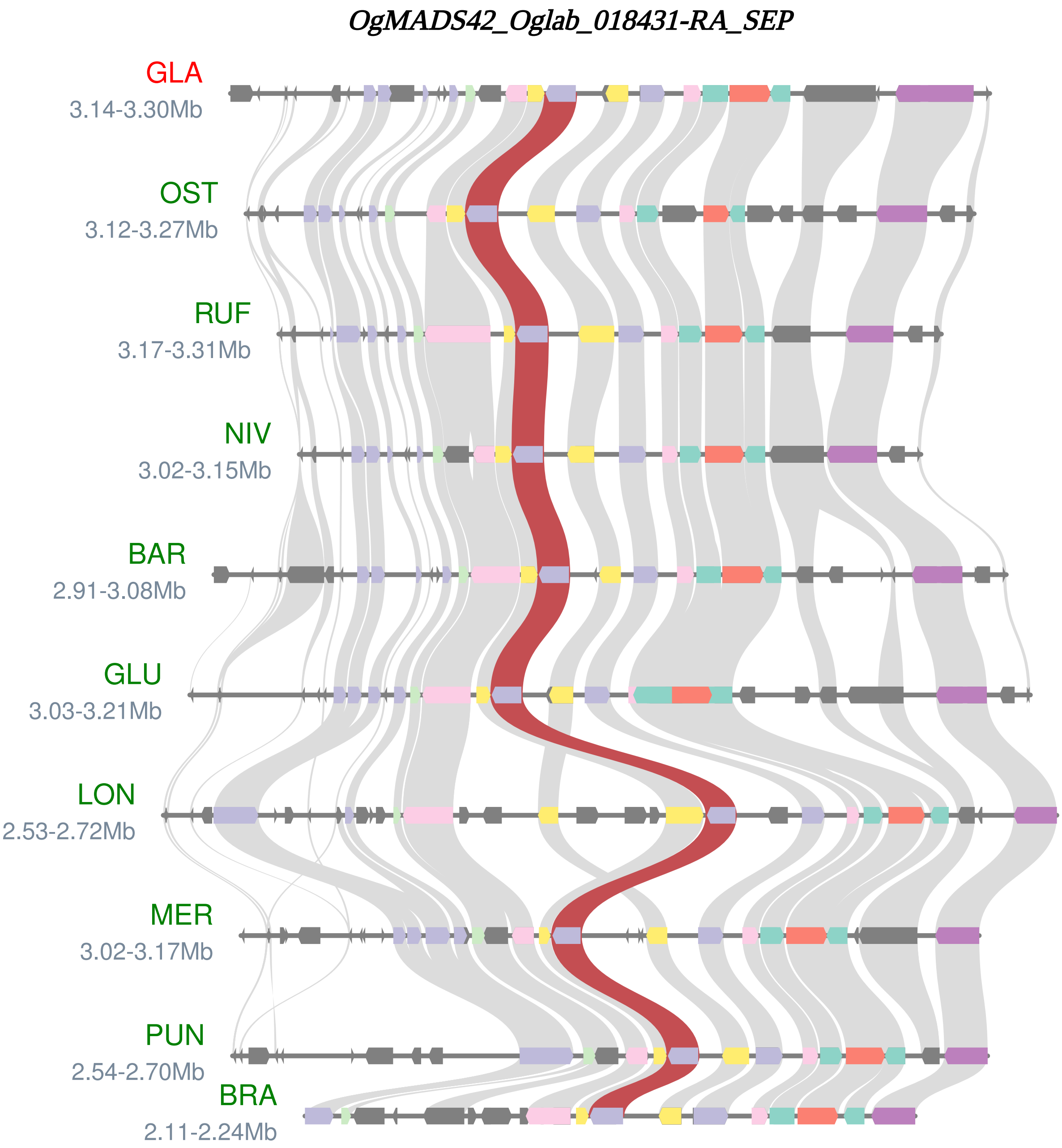
OgMADS42_Oglab_018431-RA_SEP
GLA
3.14-3.30Mb
OST
3.12-3.27Mb
RUF
3.17-3.31Mb
NIV
3.02-3.15Mb
BAR
2.91-3.08Mb
GLU
3.03-3.21Mb
LON
2.53-2.72Mb
MER
3.02-3.17Mb
PUN
2.54-2.70Mb
BRA
2.11-2.24Mb

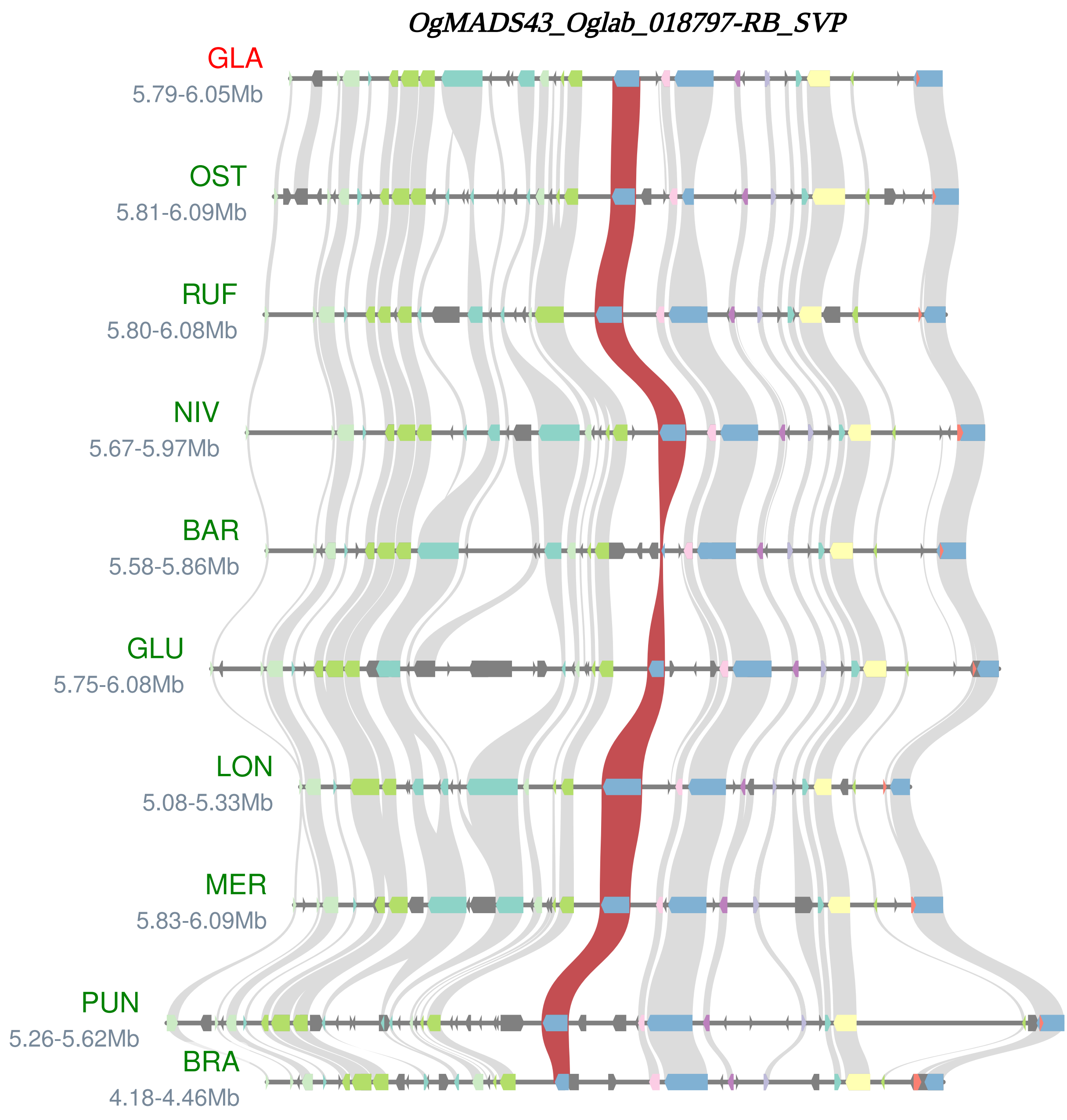

OgMADS43_Oglab_018797-RB_SVP
GLA
5.79-6.05Mb
OST
5.81-6.09Mb
RUF
5.80-6.08Mb
NIV
5.67-5.97Mb
BAR
5.58-5.86Mb
GLU
5.75-6.08Mb
LON
5.08-5.33Mb
MER
5.83-6.09Mb
PUN
5.26-5.62Mb
BRA
4.18-4.46Mb

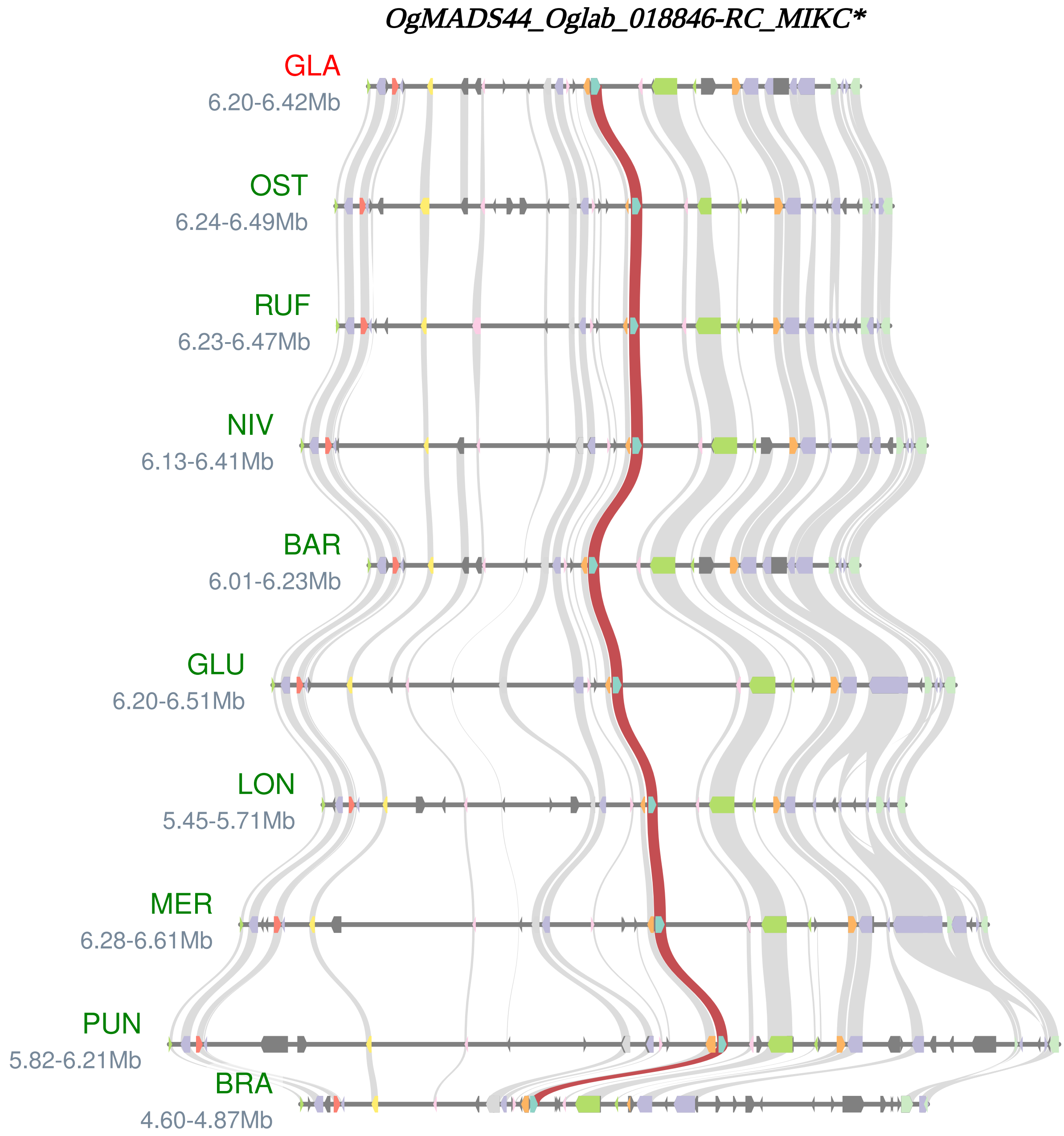

OgMADS44_Oglab_018846-RC_MIKC*
GLA
6.20-6.42Mb
OST
6.24-6.49Mb
RUF
6.23-6.47Mb
NIV
6.13-6.41Mb
BAR
6.01-6.23Mb
GLU
6.20-6.51Mb
LON
5.45-5.71Mb
MER
6.28-6.61Mb
PUN
5.82-6.21Mb
BRA
4.60-4.87Mb

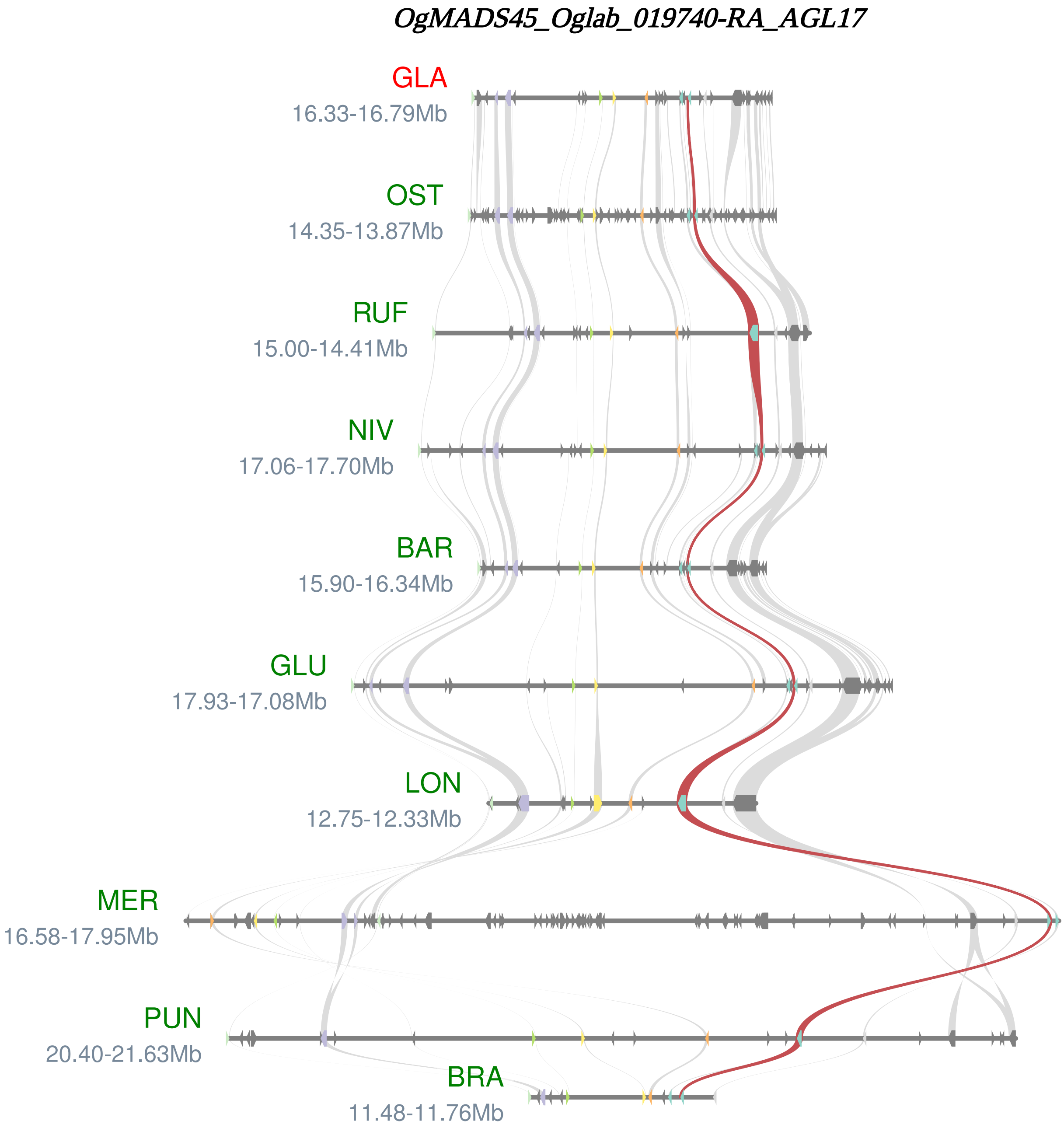

OgMADS45_Oglab_019740-RA_AGL17
GLA
16.33-16.79Mb
OST
14.35-13.87Mb
RUF
15.00-14.41Mb
NIV
17.06-17.70Mb
BAR
15.90-16.34Mb
GLU
17.93-17.08Mb
LON
12.75-12.33Mb
MER
16.58-17.95Mb
PUN
20.40-21.63Mb
BRA
11.48-11.76Mb

OgMADS46_Oglab_019820-RA_Mα

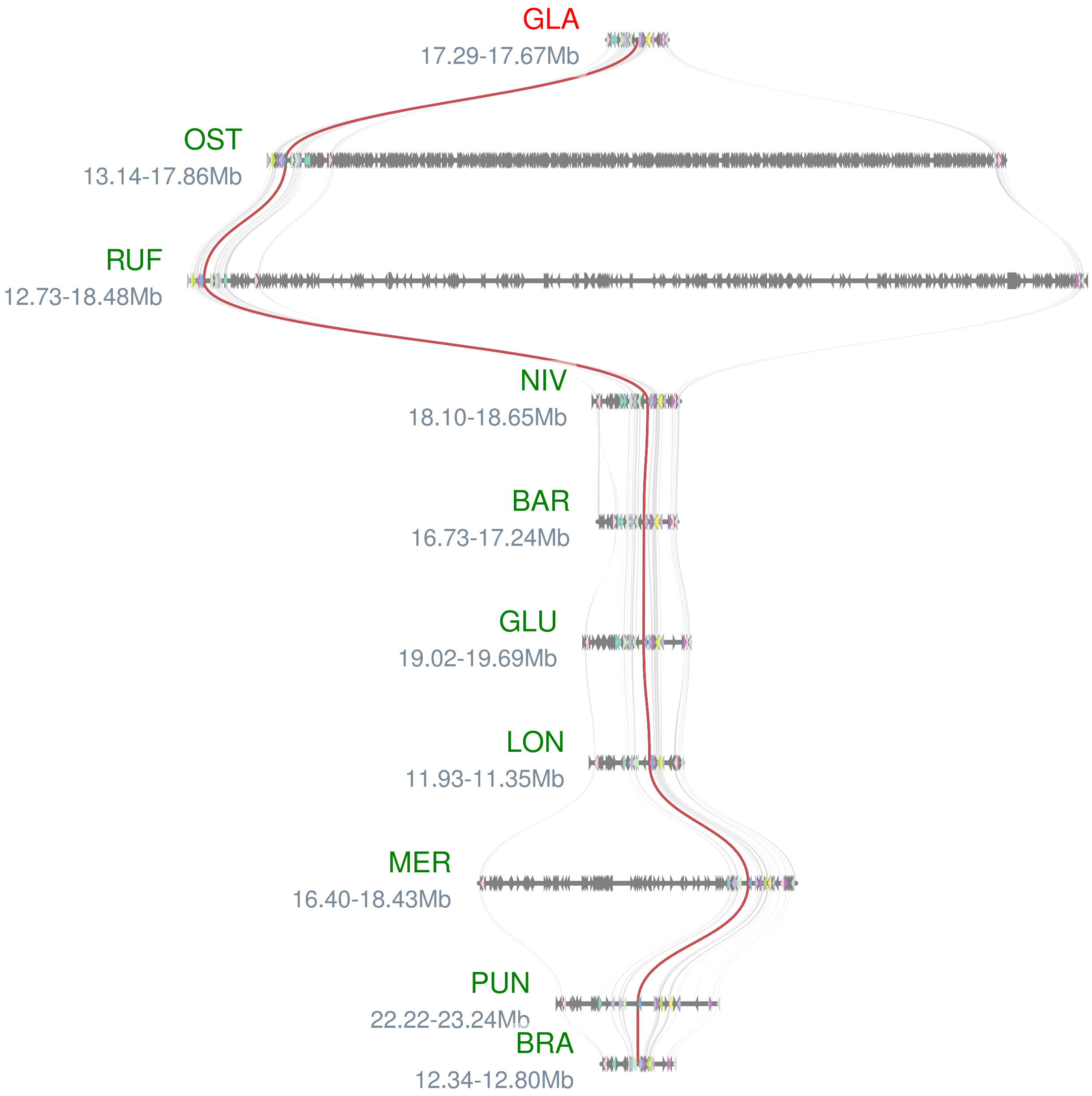

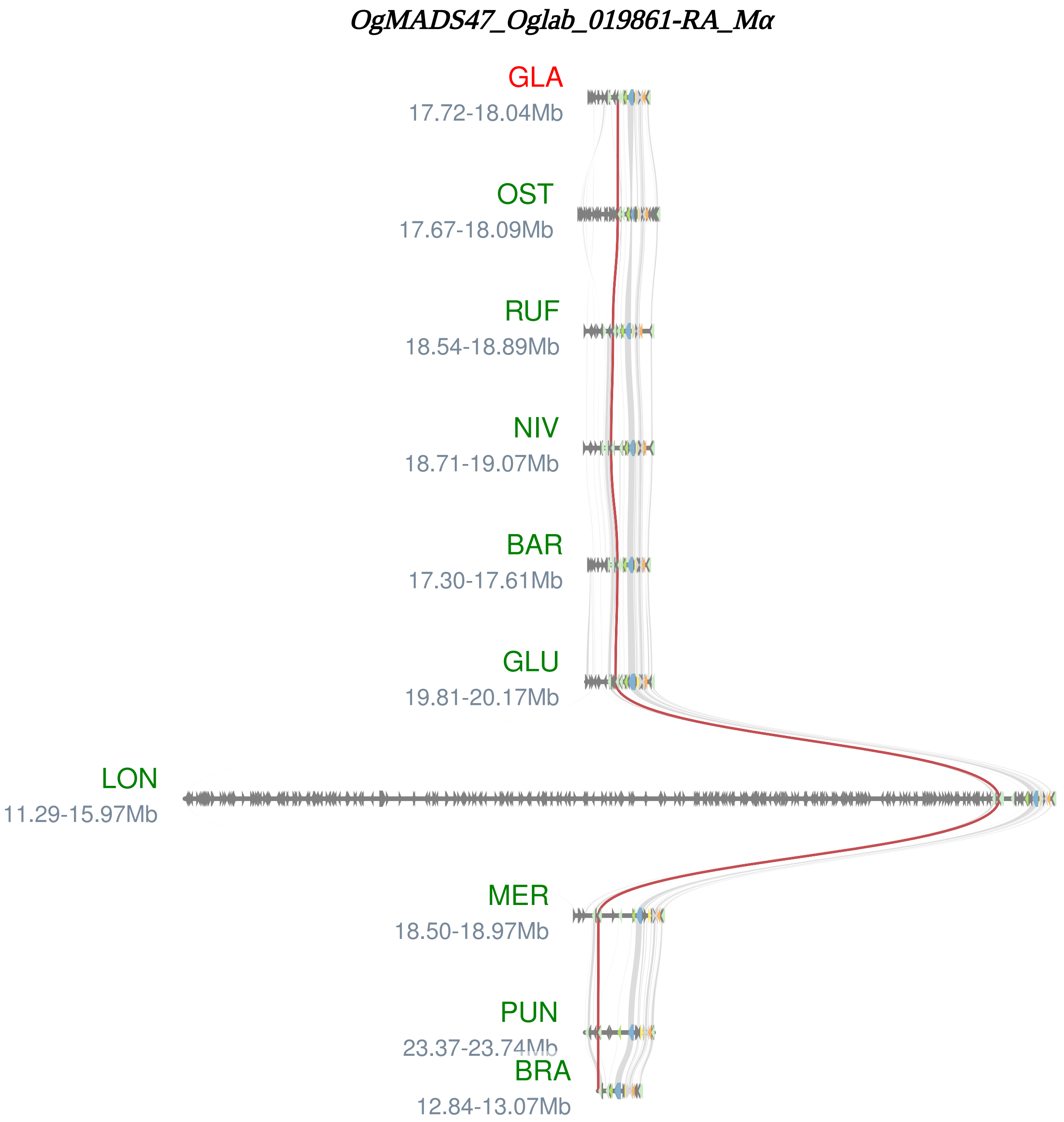

OgMADS47_Oglab_019861-RA_Mα
GLA
17.72-18.04Mb
OST
17.67-18.09Mb
RUF
18.54-18.89Mb
NIV
18.71-19.07Mb
BAR
17.30-17.61Mb
GLU
19.81-20.17Mb
LON
11.29-15.97Mb
MER
18.50-18.97Mb
PUN
23.37-23.74Mb
BRA
12.84-13.07Mb

OgMADS48_Oglab_019862-RA_Mα

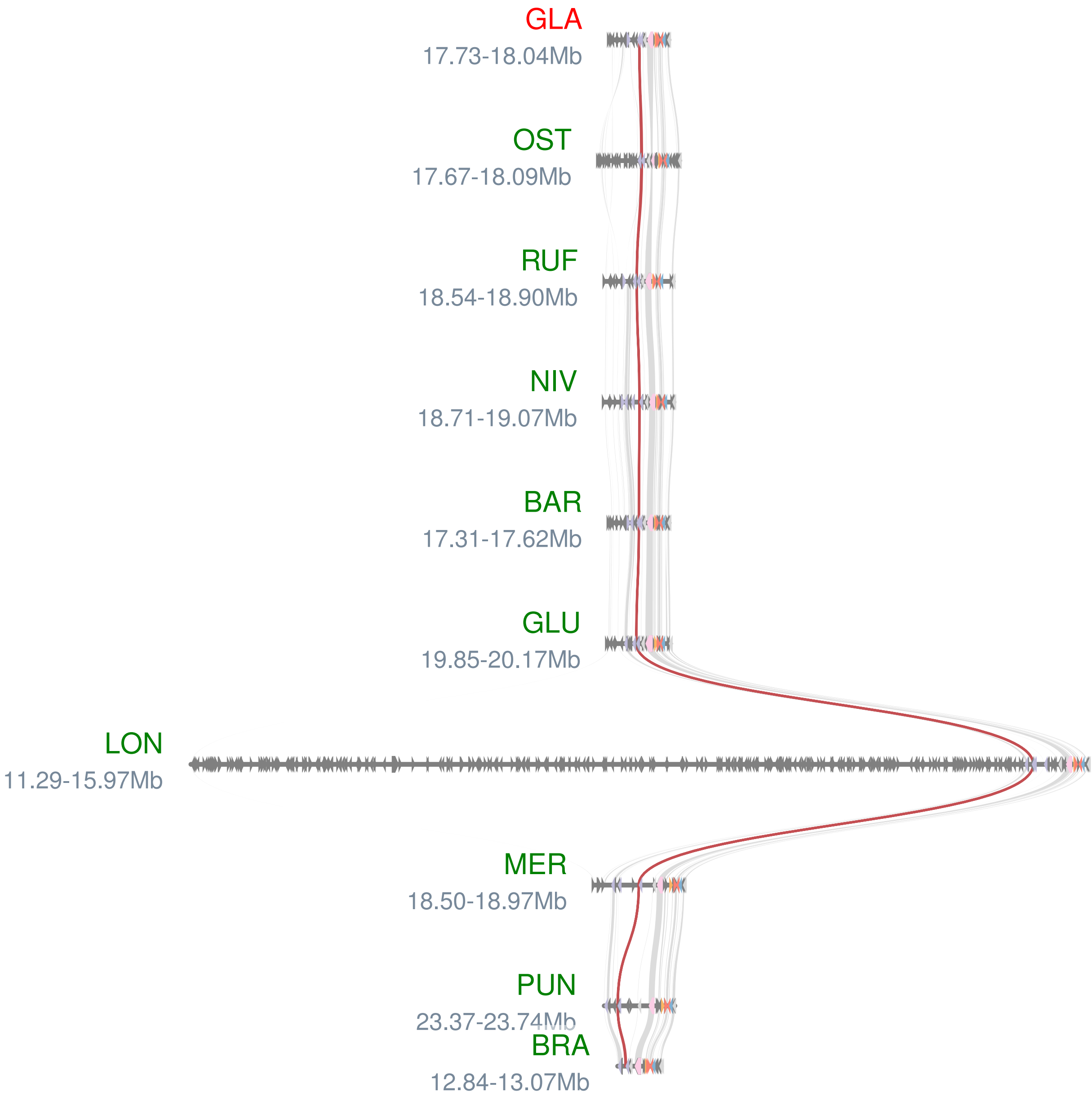

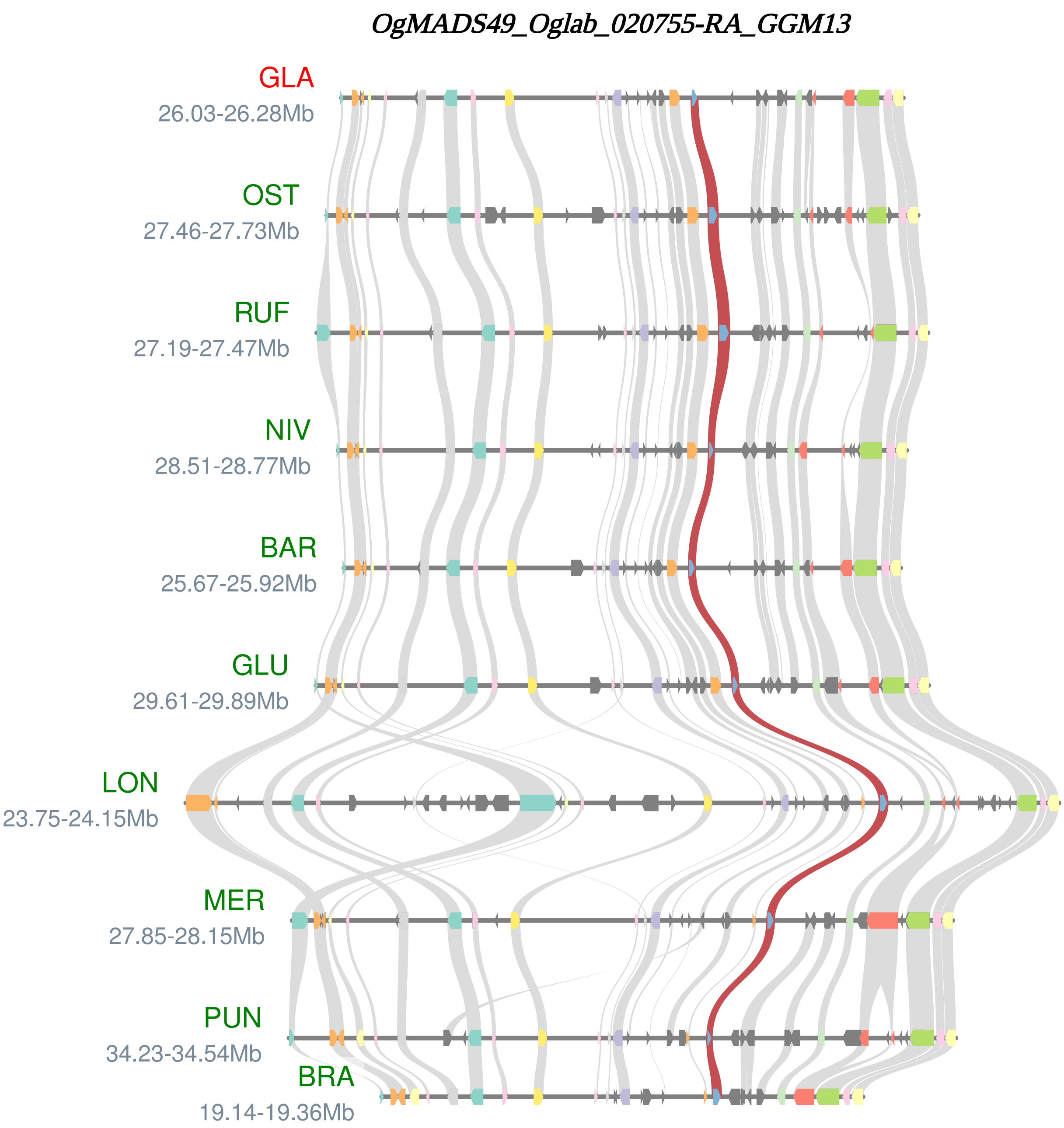
OgMADS49_Oglab_020755-RA_GGM13
GLA
26.03-26.28Mb
OST
27.46-27.73Mb
RUF
27.19-27.47Mb
NIV
28.51-28.77Mb
BAR
25.67-25.92Mb
GLU
29.61-29.89Mb
LON
23.75-24.15Mb
MER
27.85-28.15Mb
PUN
34.23-34.54Mb
BRA
19.14-19.36Mb

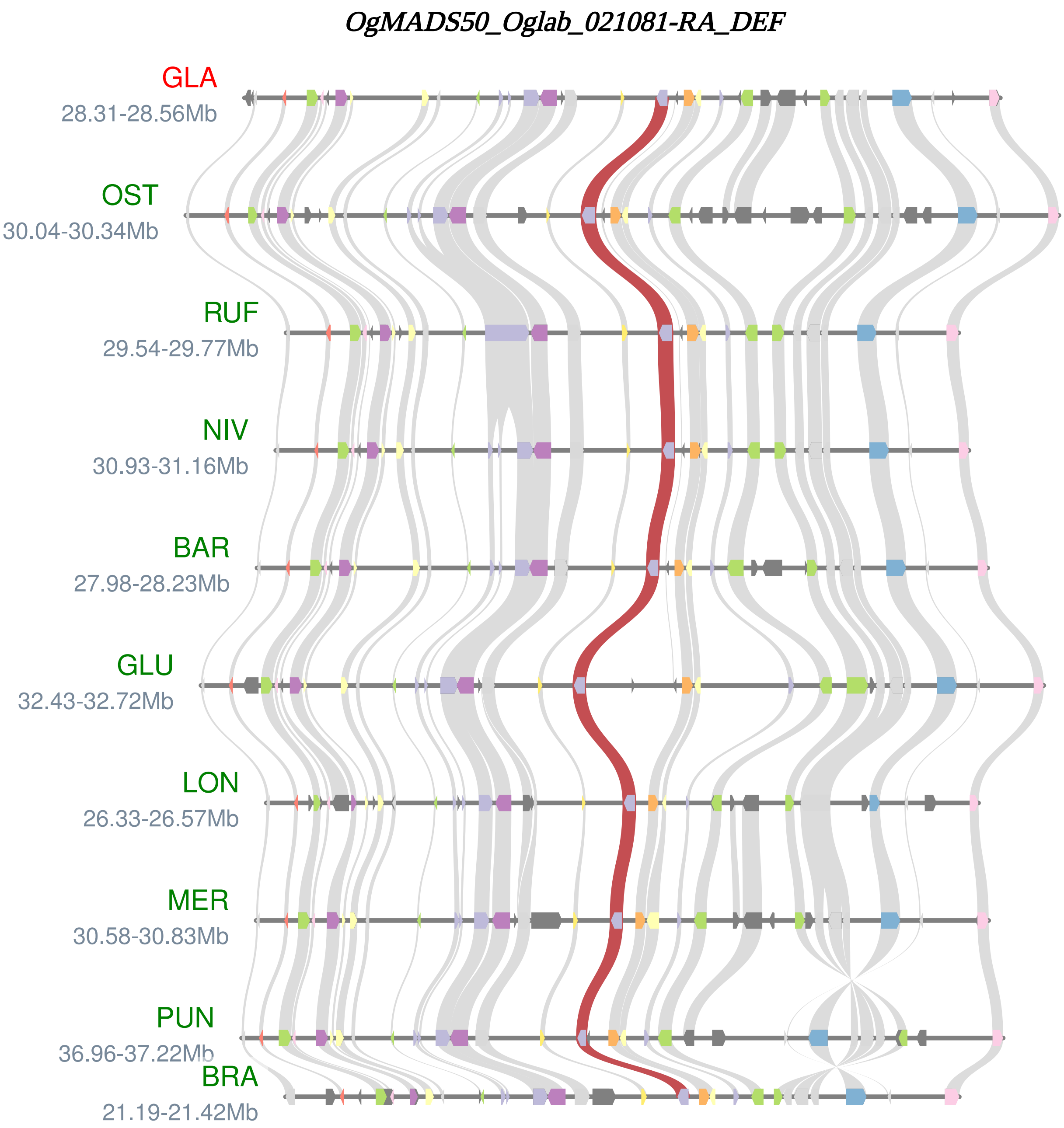
OgMADS50_Oglab_021081-RA_DEF
GLA
28.31-28.56Mb
OST
30.04-30.34Mb
RUF
29.54-29.77Mb
NIV
30.93-31.16Mb
BAR
27.98-28.23Mb
GLU
32.43-32.72Mb
LON
26.33-26.57Mb
MER
30.58-30.83Mb
PUN
36.96-37.22Mb
BRA
21.19-21.42Mb

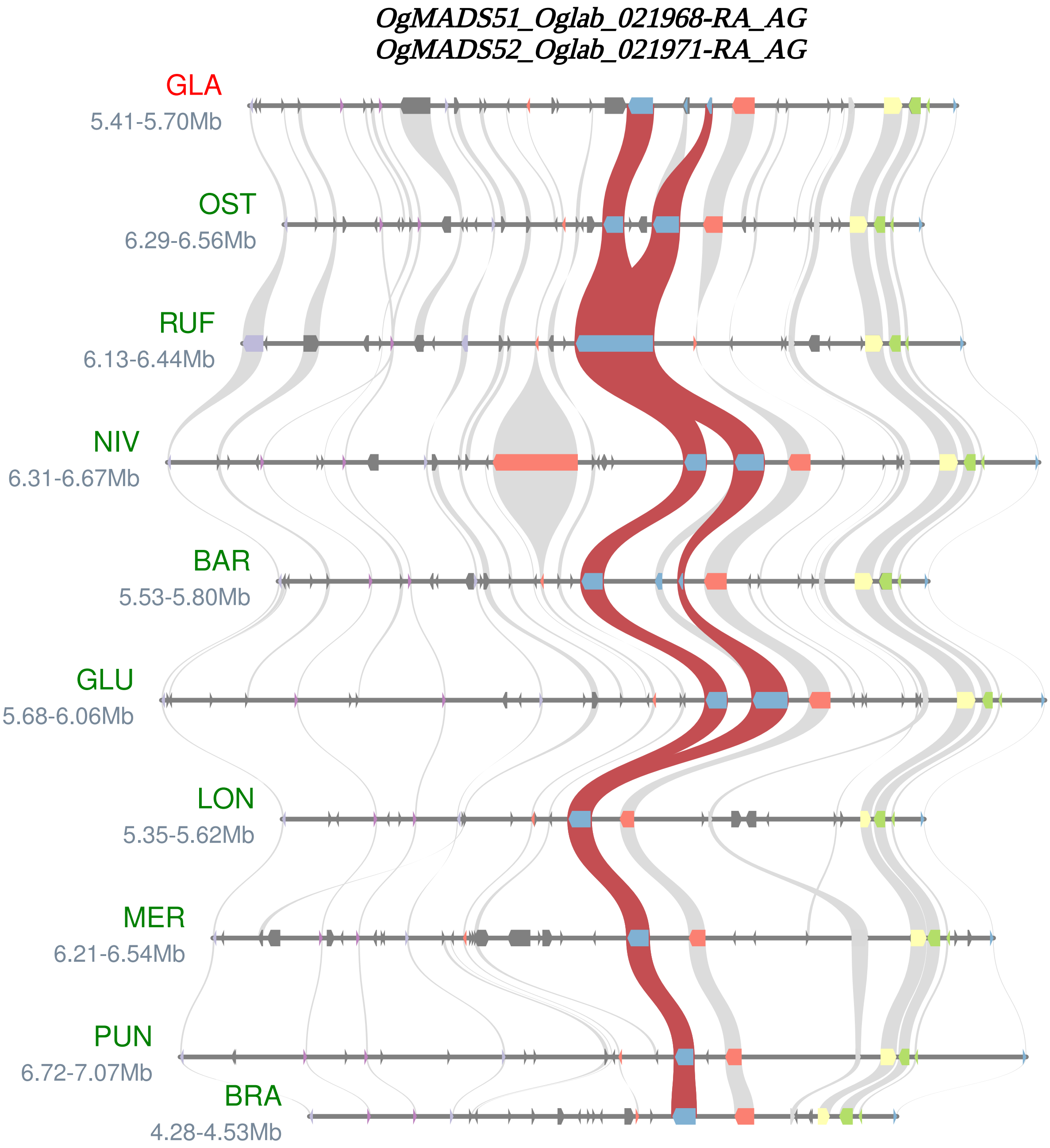

OgMADS51_Oglab_021968-RA_AG
OgMADS52_Oglab_021971-RA_AG
GLA
5.41-5.70Mb
OST
6.29-6.56Mb
RUF
6.13-6.44Mb
NIV
6.31-6.67Mb
BAR
5.53-5.80Mb
GLU
5.68-6.06Mb
LON
5.35-5.62Mb
MER
6.21-6.54Mb
PUN
6.72-7.07Mb
BRA
4.28-4.53Mb

OgMADS53_Oglab_022433-RA_Mα

(The chromosomal segment in the LON lacks any detected syntenic genes.)

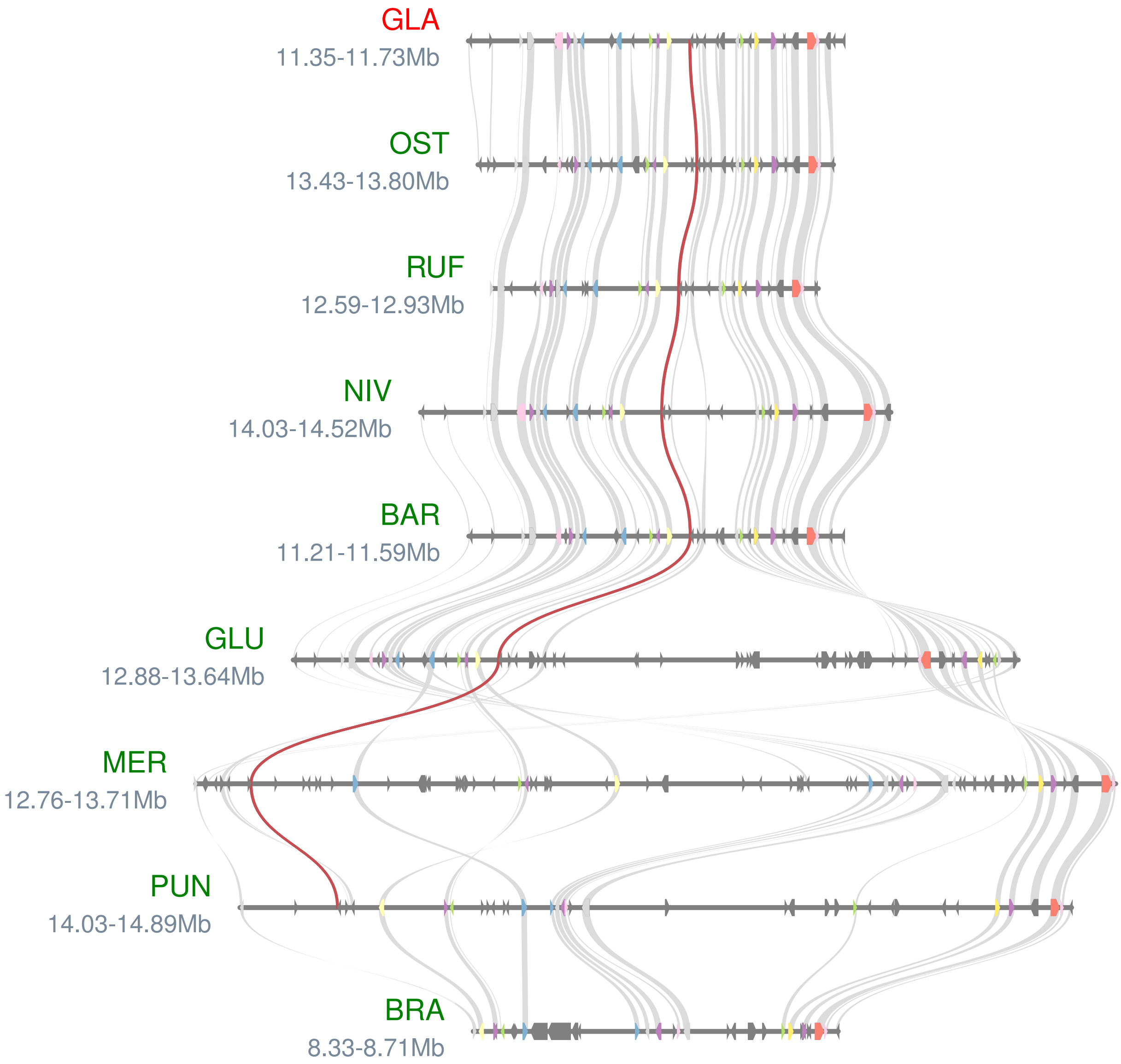

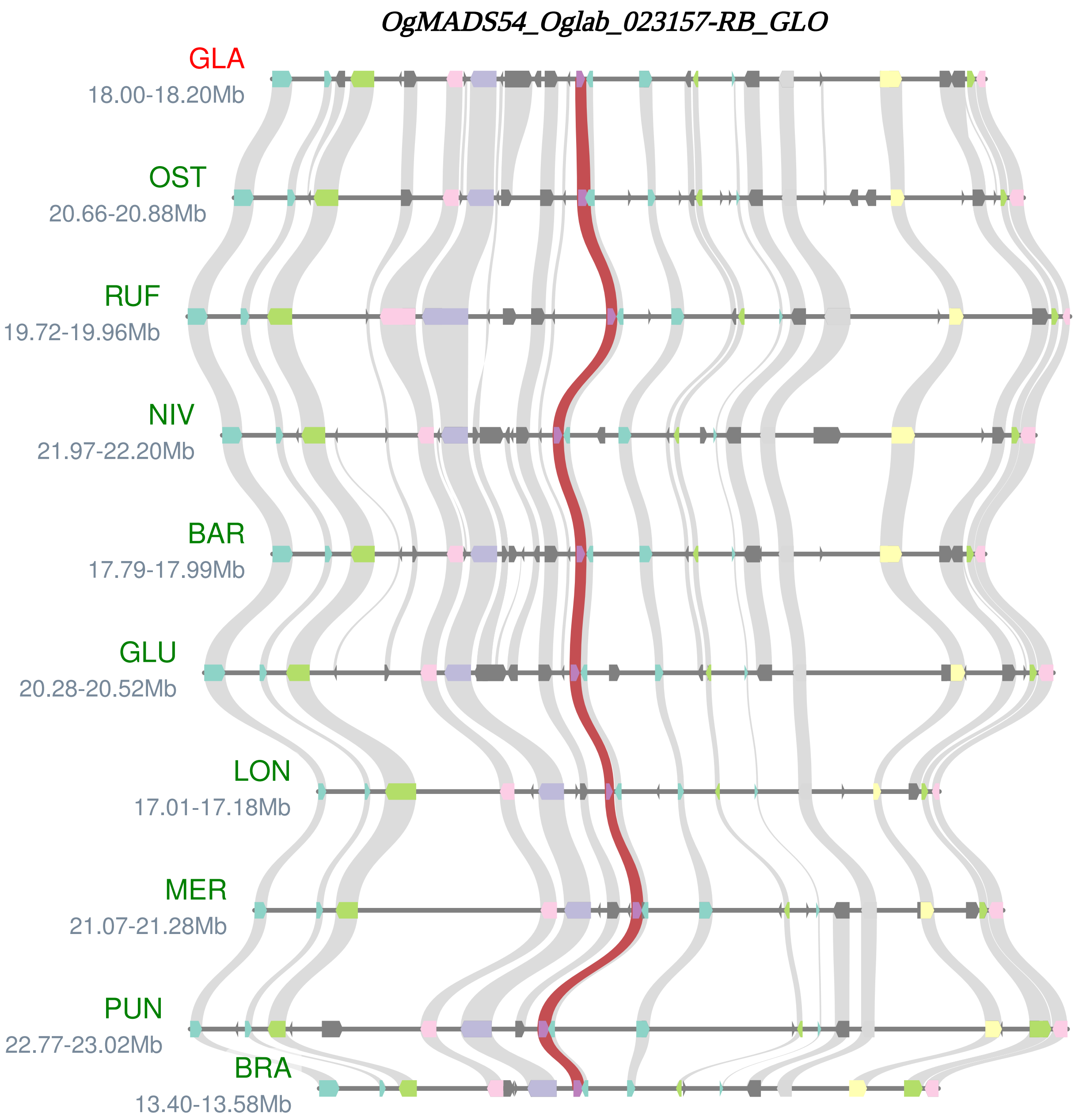

OgMADS54_Oglab_023157-RB_GLO
GLA
18.00-18.20Mb
OST
20.66-20.88Mb
RUF
19.72-19.96Mb
NIV
21.97-22.20Mb
BAR
17.79-17.99Mb
GLU
20.28-20.52Mb
LON
17.01-17.18Mb
MER
21.07-21.28Mb
PUN
22.77-23.02Mb
BRA
13.40-13.58Mb

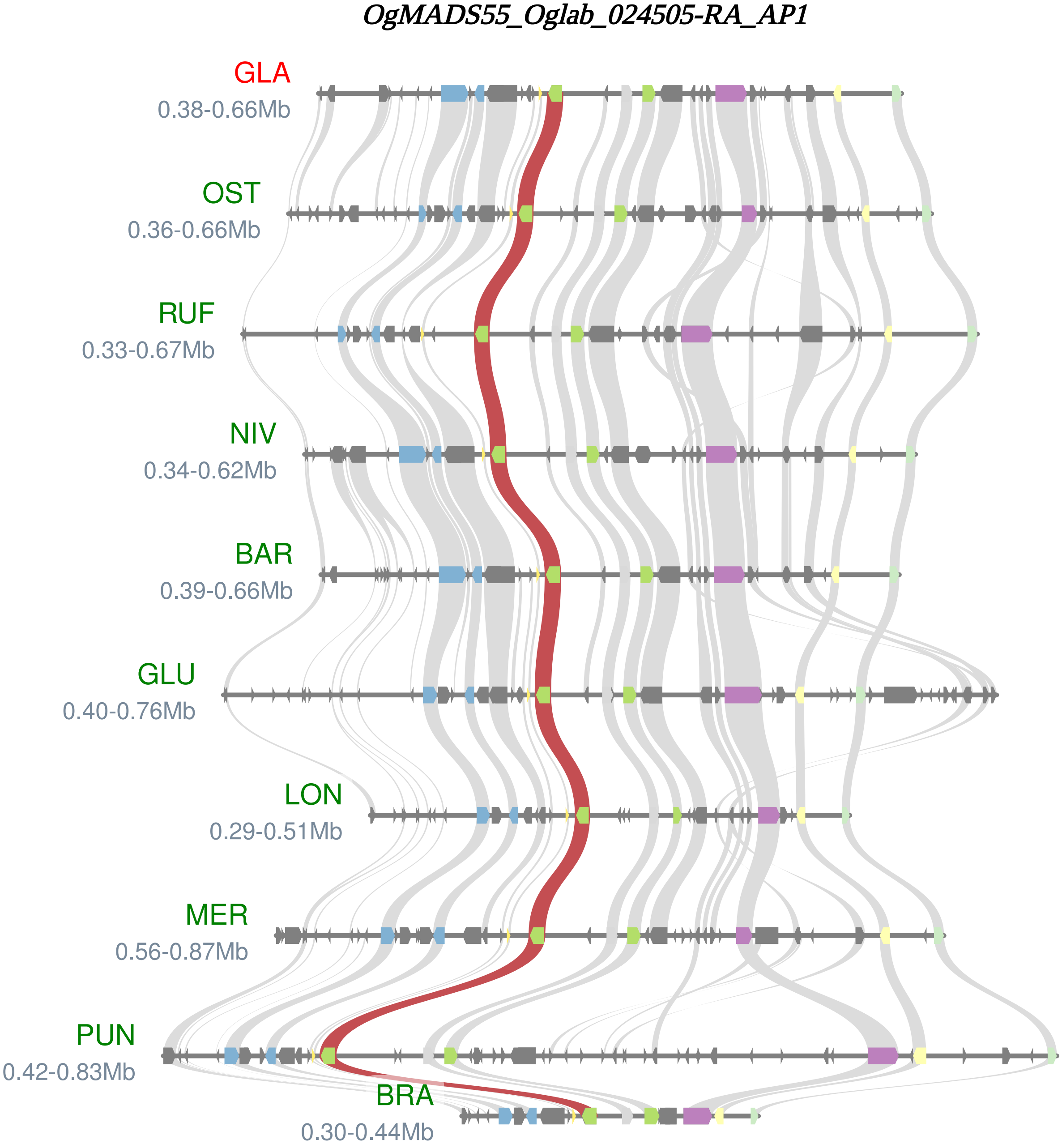
OgMADS55_Oglab_024505-RA_AP1
GLA
0.38-0.66Mb
OST
0.36-0.66Mb
RUF
0.33-0.67Mb
NIV
0.34-0.62Mb
BAR
0.39-0.66Mb
GLU
0.40-0.76Mb
LON
0.29-0.51Mb
MER
0.56-0.87Mb
PUN
0.42-0.83Mb
BRA
0.30-0.44Mb

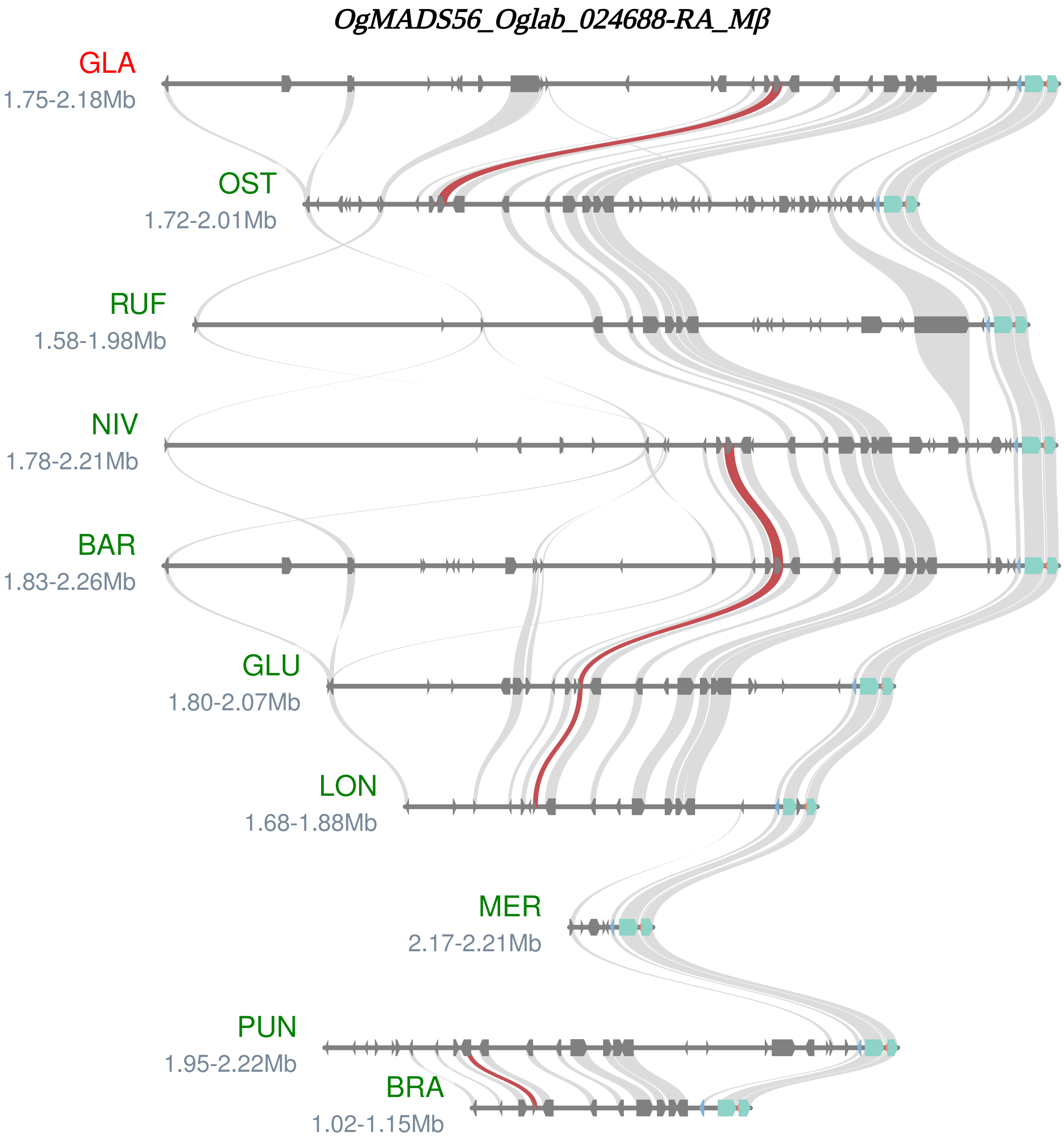
OgMADS56_Oglab_024688-RA_Mβ
GLA
1.75-2.18Mb
OST
1.72-2.01Mb
RUF
1.58-1.98Mb
NIV
1.78-2.21Mb
BAR
1.83-2.26Mb
GLU
1.80-2.07Mb
LON
1.68-1.88Mb
MER
2.17-2.21Mb
PUN
1.95-2.22Mb
BRA
1.02-1.15Mb

OgMADS57_Oglab_026973-RB_AP1
(The chromosomal segment in the NIV&LON lacks any detected syntenic genes.)

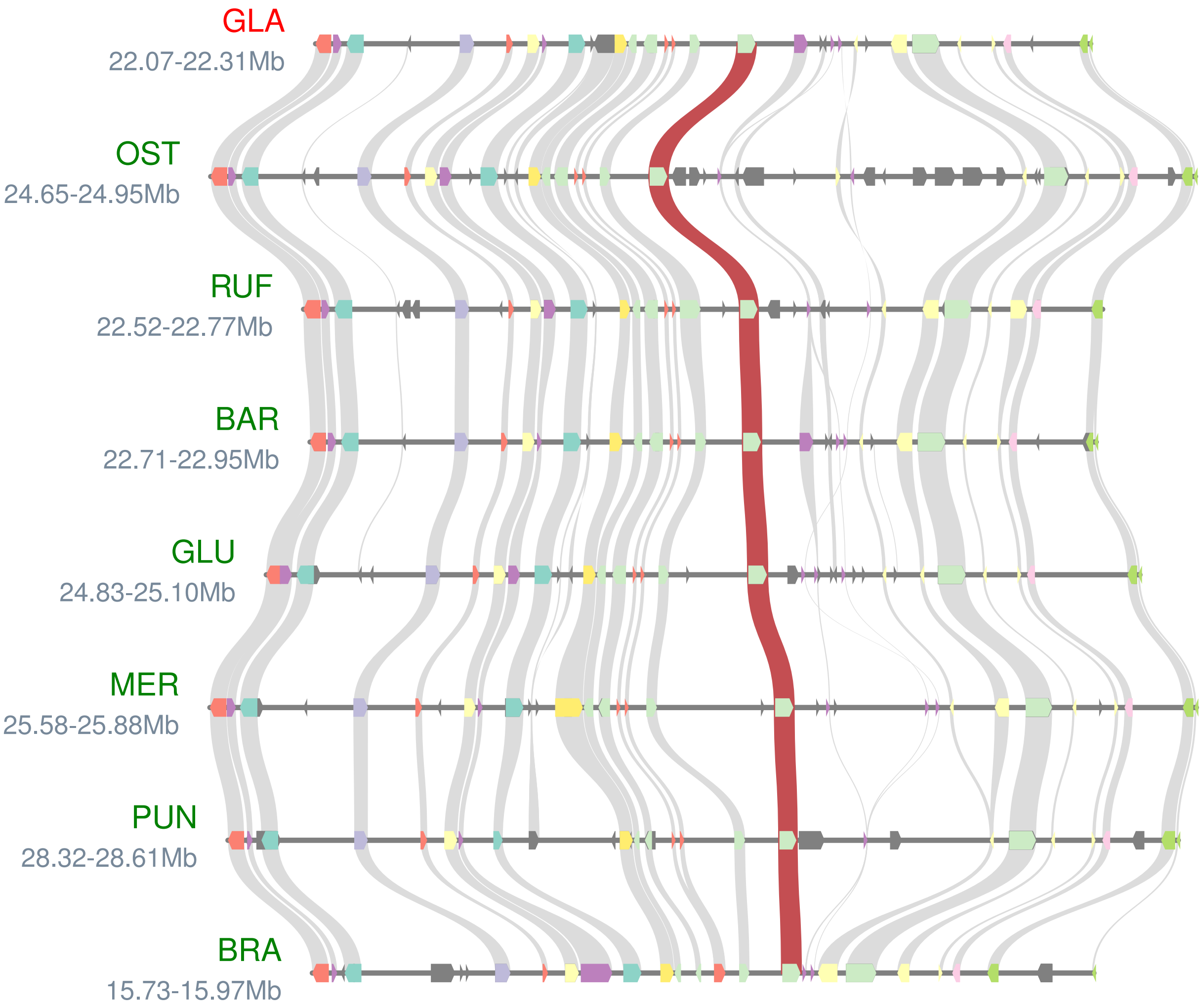

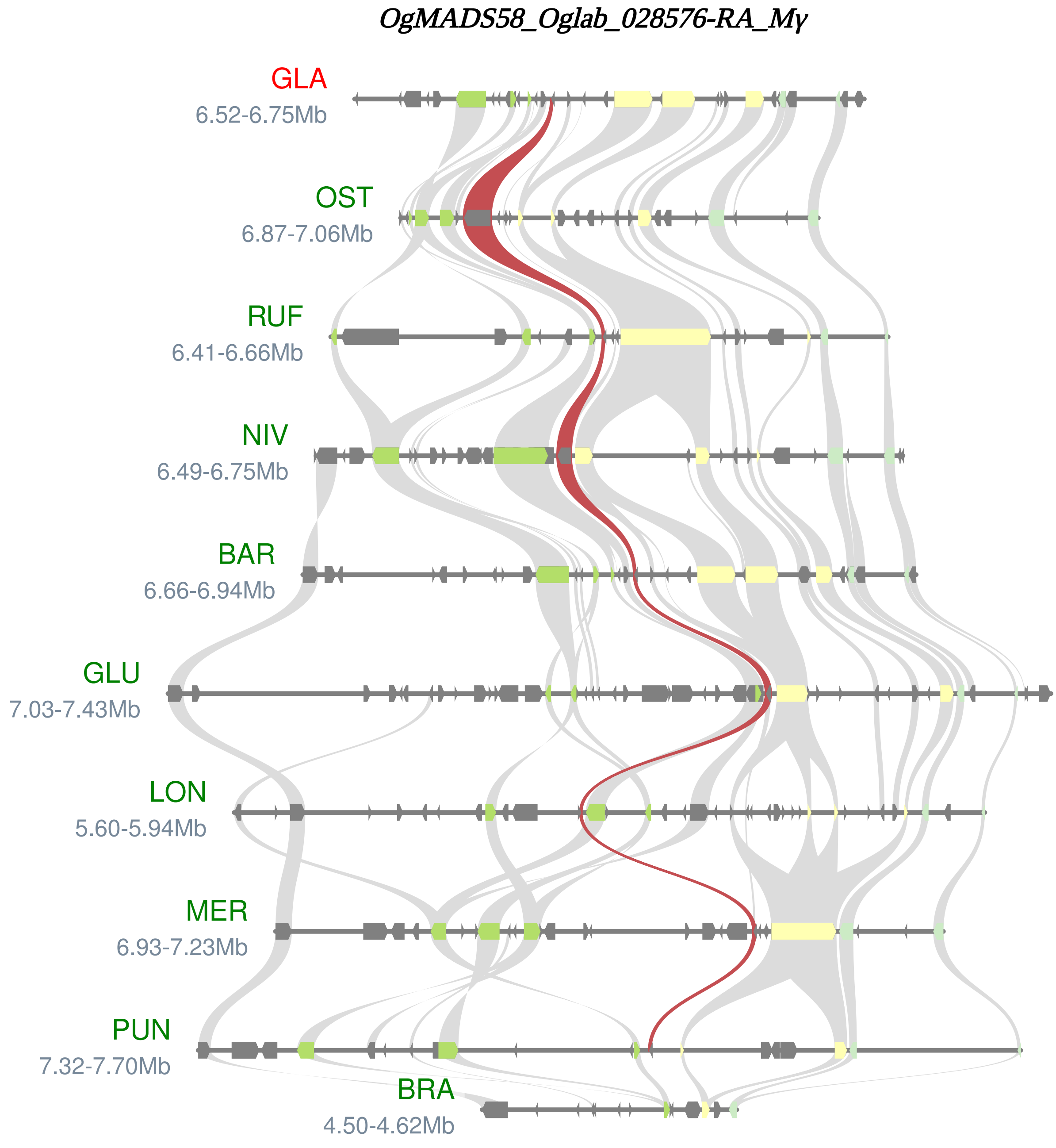
OgMADS58_Oglab_028576-RA_Mγ
GLA
6.52-6.75Mb
OST
6.87-7.06Mb
RUF
6.41-6.66Mb
NIV
6.49-6.75Mb
BAR
6.66-6.94Mb
GLU
7.03-7.43Mb
LON
5.60-5.94Mb
MER
6.93-7.23Mb
PUN
7.32-7.70Mb
BRA
4.50-4.62Mb

OgMADS59_Oglab_030185-RB_MIKC
(The chromosomal segment in the GLU&LON lacks any detected syntenic genes.)

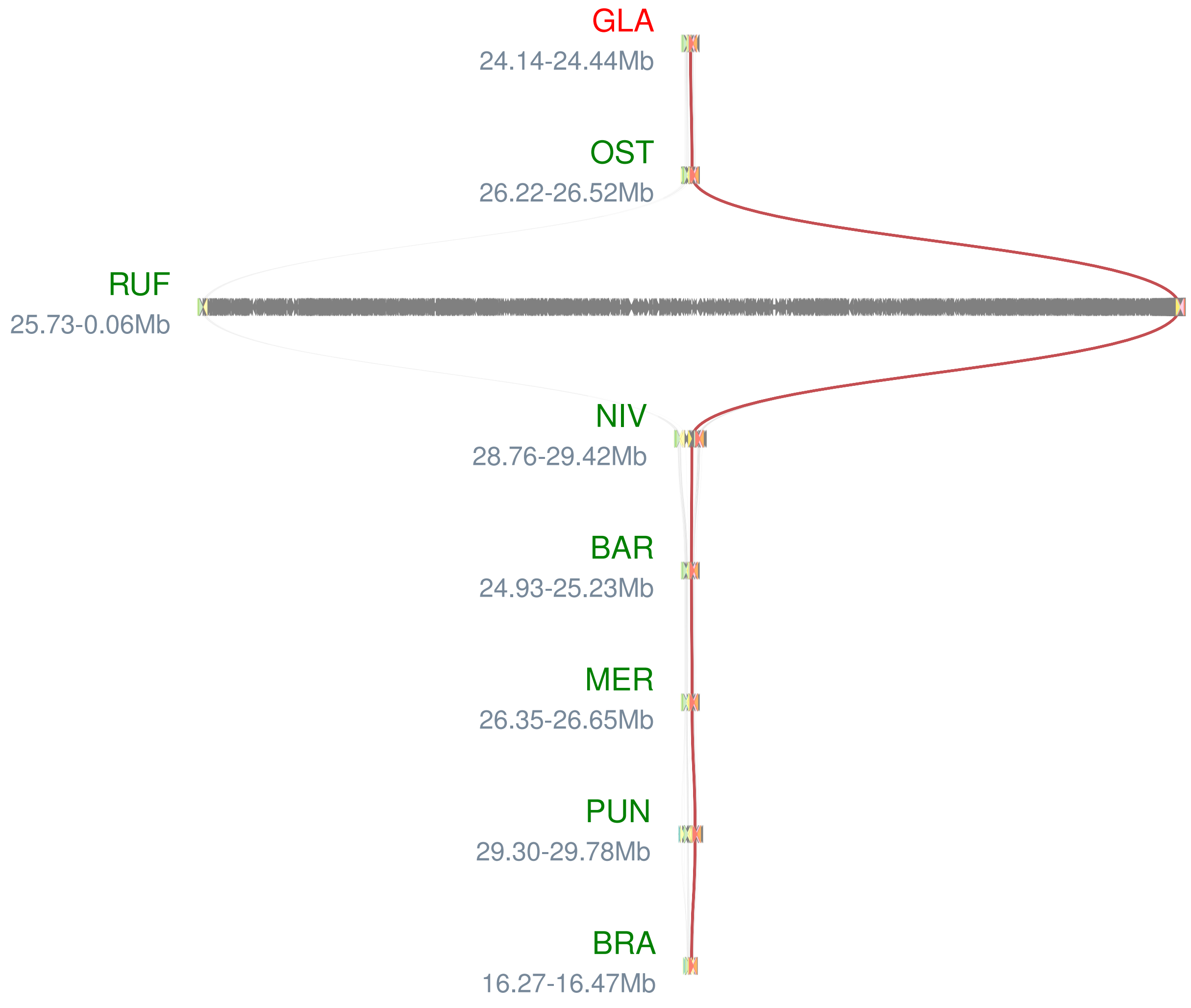

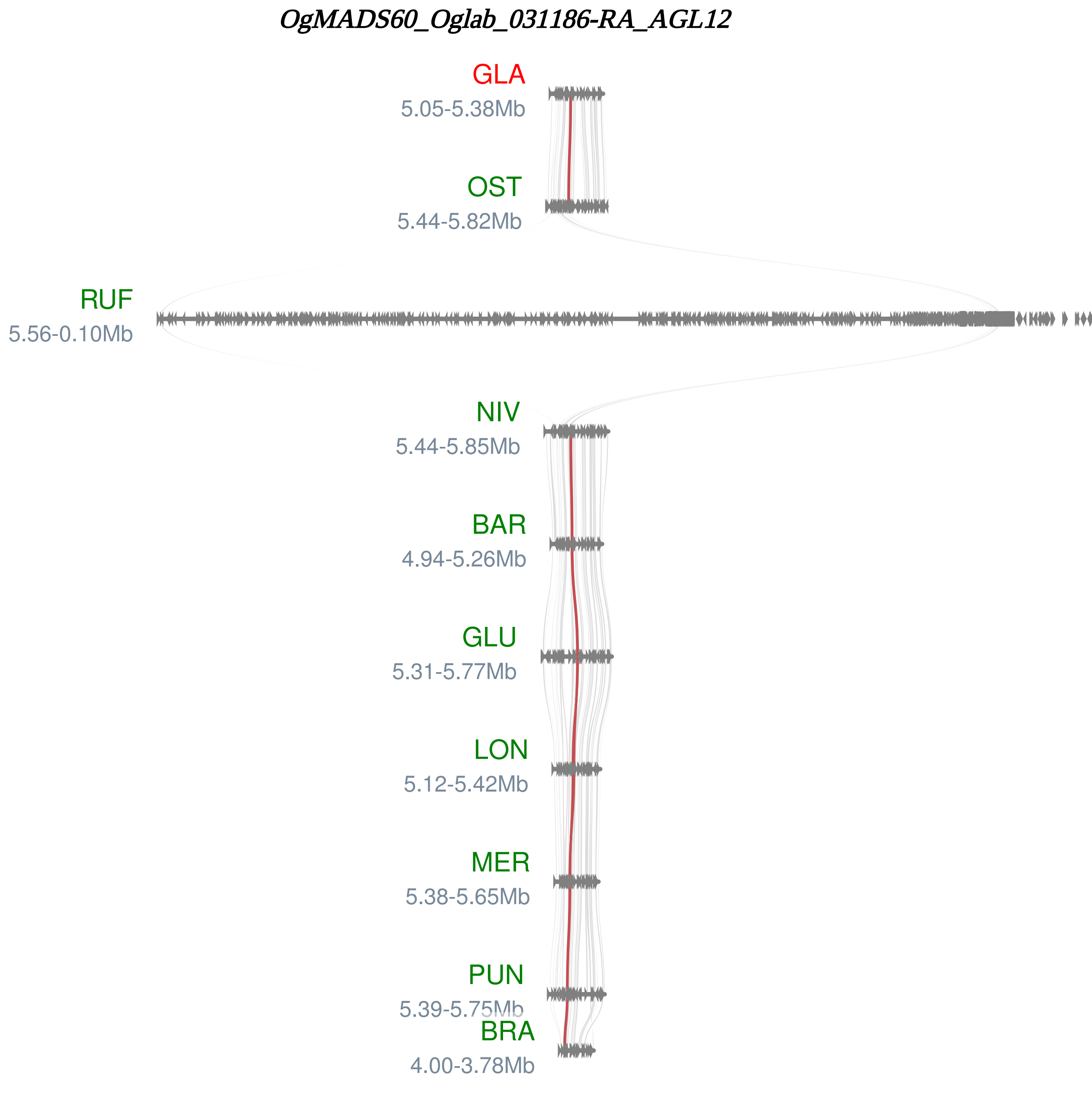

OgMADS60_Oglab_031186-RA_AGL12
GLA
5.05-5.38Mb
OST
5.44-5.82Mb
RUF
5.56-0.10Mb
NIV
5.44-5.85Mb
BAR
4.94-5.26Mb
GLU
5.31-5.77Mb
LON
5.12-5.42Mb
MER
5.38-5.65Mb
PUN
5.39-5.75Mb
BRA
4.00-3.78Mb

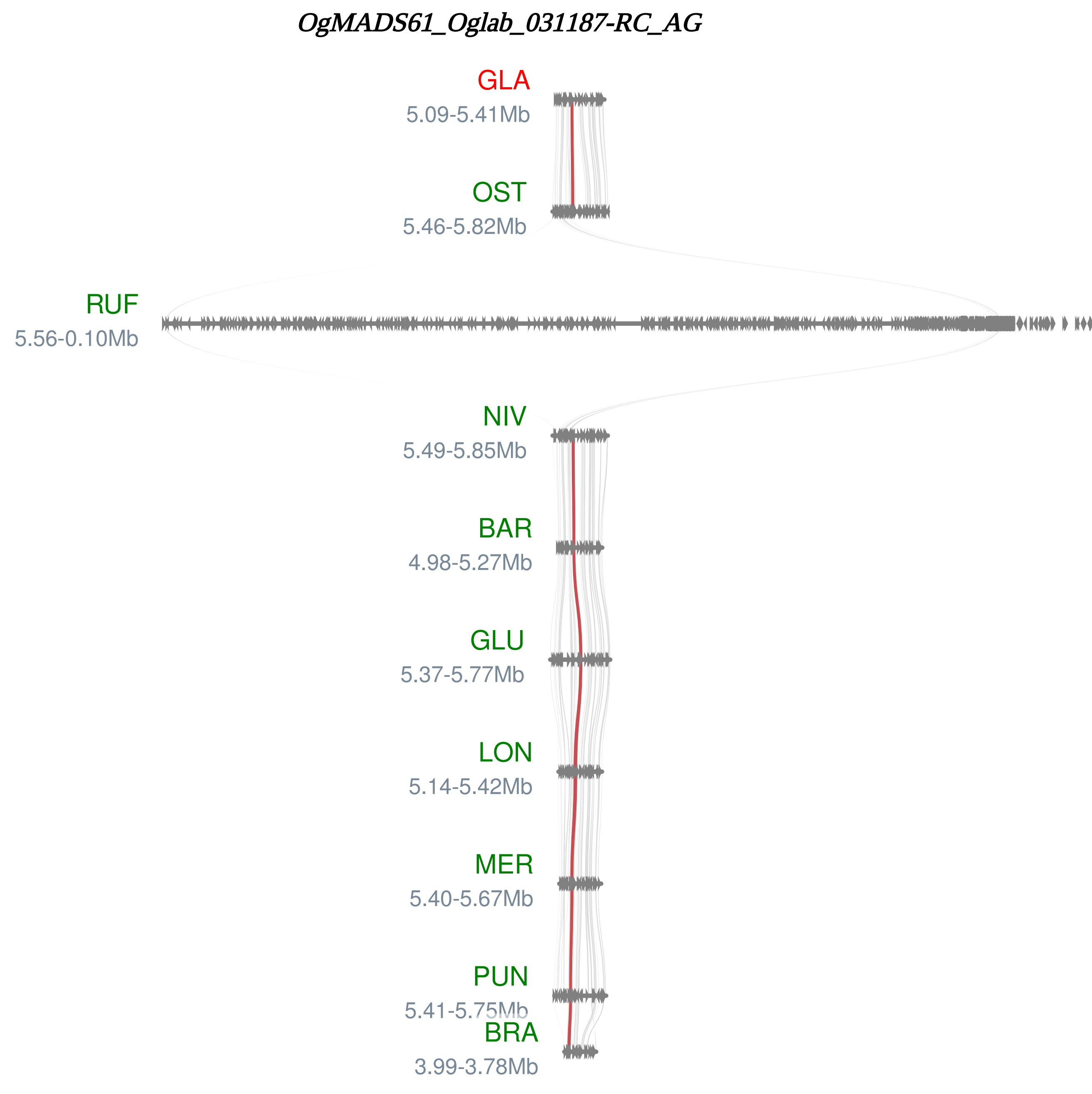
OgMADS61_Oglab_031187-RC_AG
GLA
5.09-5.41Mb
OST
5.46-5.82Mb
RUF
5.56-0.10Mb
NIV
5.49-5.85Mb
BAR
4.98-5.27Mb
GLU
5.37-5.77Mb
LON
5.14-5.42Mb
MER
5.40-5.67Mb
PUN
5.41-5.75Mb
BRA
3.99-3.78Mb

OgMADS62_Oglab_031627-RA_Mα
OgMADS63_Oglab_031629-RA_Mα
(The chromosomal segment in the PUN lacks any detected syntenic genes.)

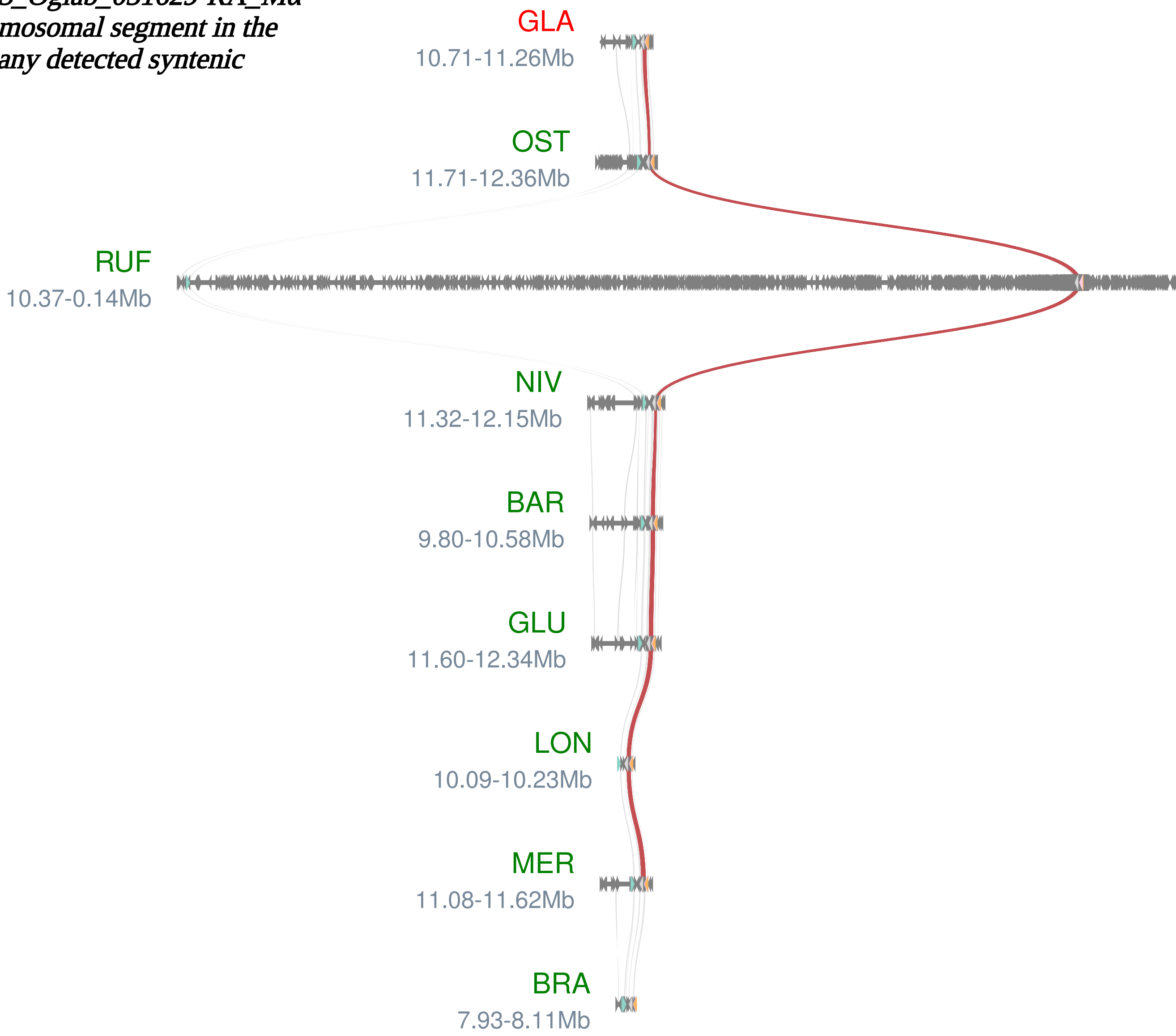

OgMADS64_Oglab_032046-RA_AP1
(The chromosomal segment in the OST lacks any detected syntenic genes.)

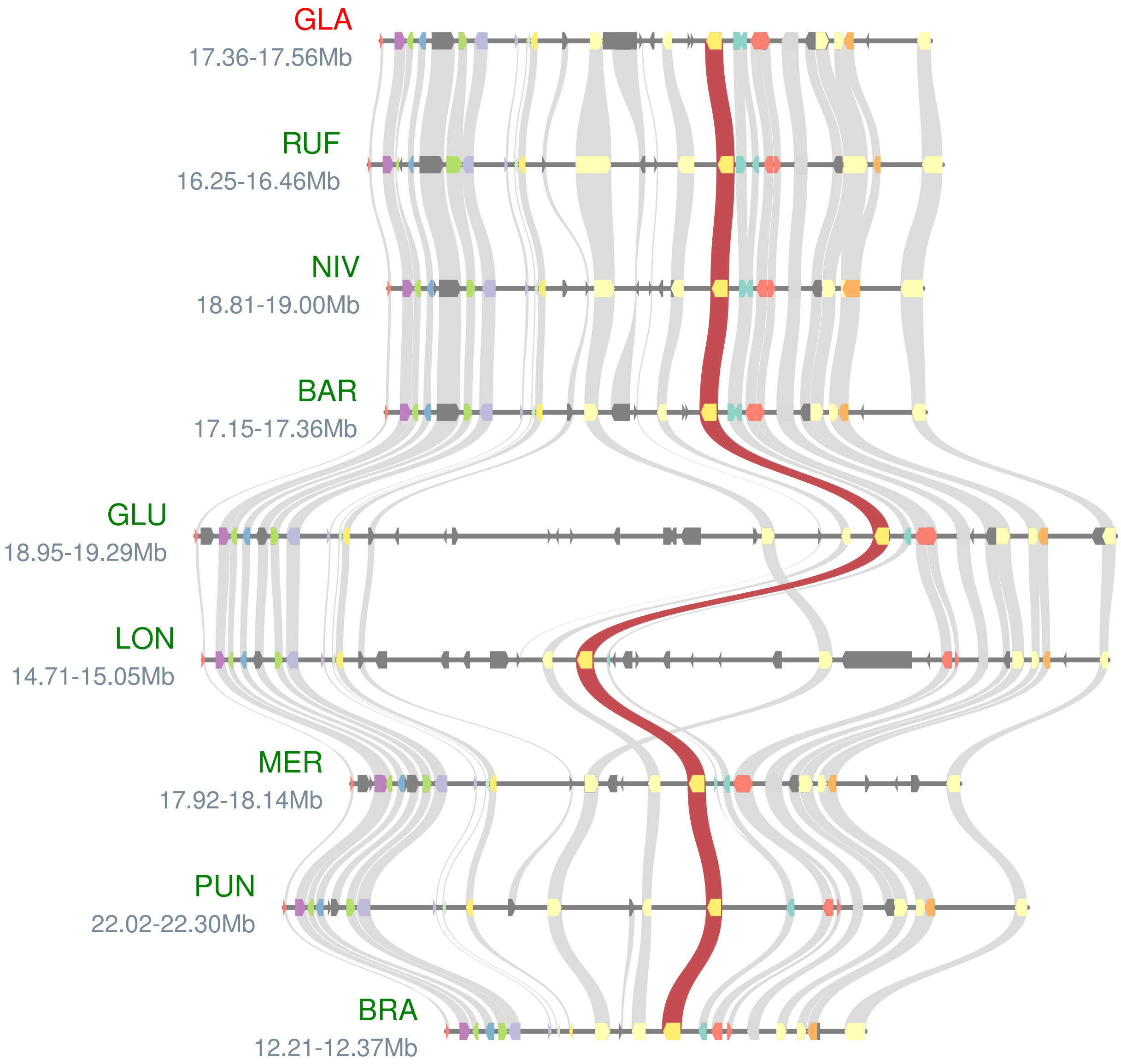

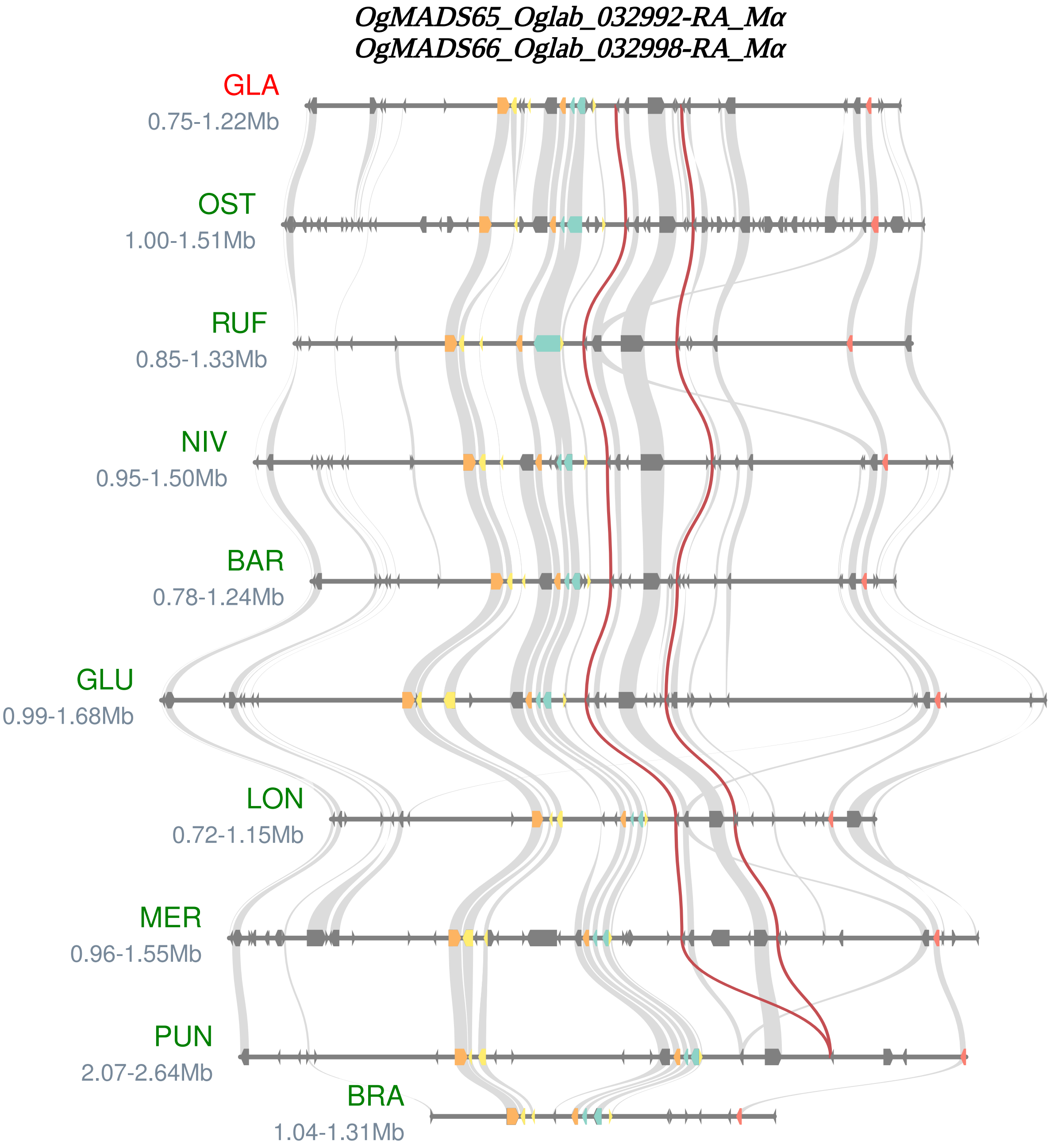

OgMADS65_Oglab_032992-RA_Mα
OgMADS66_Oglab_032998-RA_Mα
GLA
0.75-1.22Mb
OST
1.00-1.51Mb
RUF
0.85-1.33Mb
NIV
0.95-1.50Mb
BAR
0.78-1.24Mb
GLU
0.99-1.68Mb
LON
0.72-1.15Mb
MER
0.96-1.55Mb
PUN
2.07-2.64Mb
BRA
1.04-1.31Mb

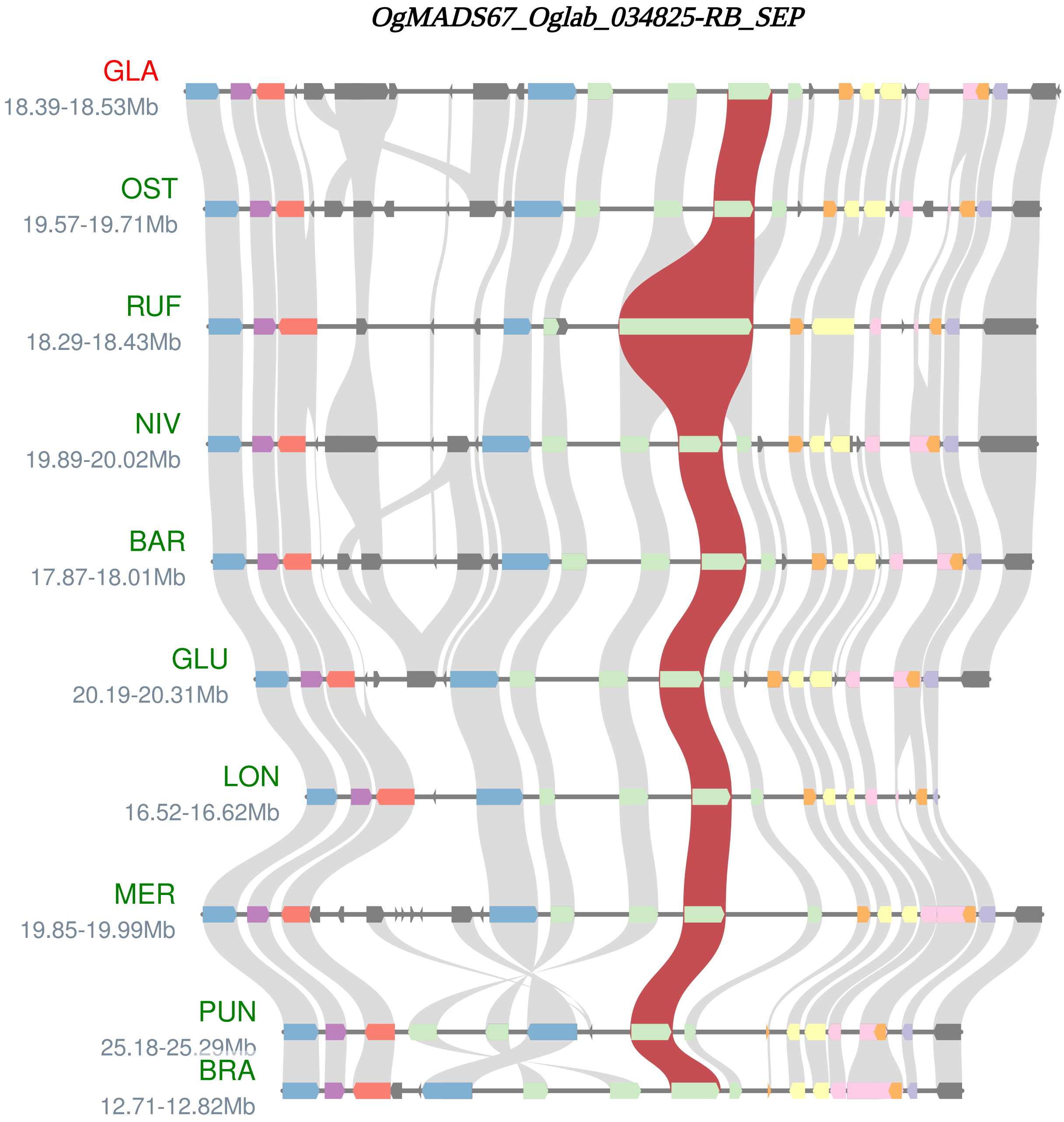

OgMADS67_Oglab_034825-RB_SEP
GLA
18.39-18.53Mb
OST
19.57-19.71Mb
RUF
18.29-18.43Mb
NIV
19.89-20.02Mb
BAR
17.87-18.01Mb
GLU
20.19-20.31Mb
LON
16.52-16.62Mb
MER
19.85-19.99Mb
PUN
25.18-25.29Mb
BRA
12.71-12.82Mb

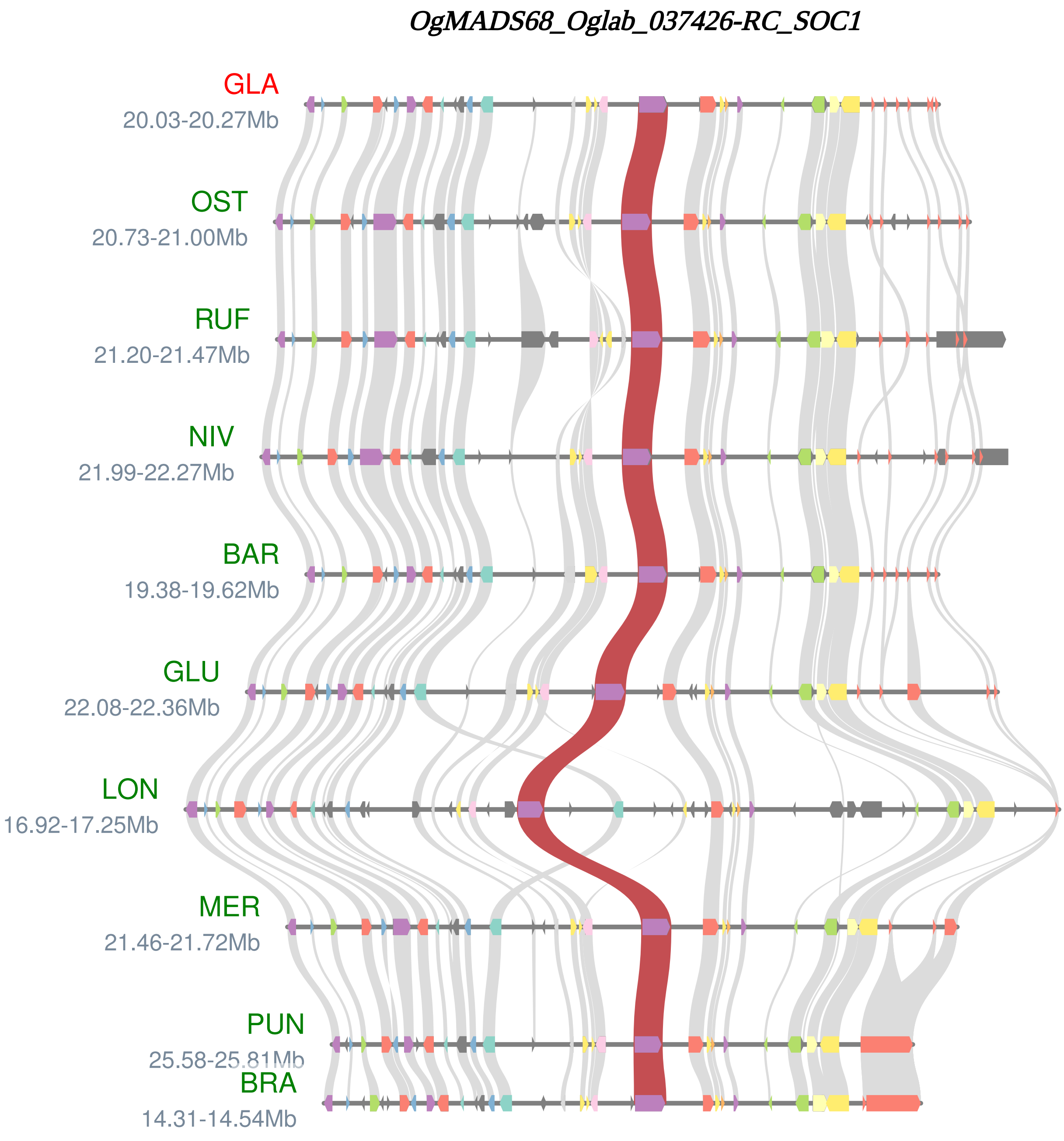
OgMADS68_Oglab_037426-RC_SOC1
GLA
20.03-20.27Mb
OST
20.73-21.00Mb
RUF
21.20-21.47Mb
NIV
21.99-22.27Mb
BAR
19.38-19.62Mb
GLU
22.08-22.36Mb
LON
16.92-17.25Mb
MER
21.46-21.72Mb
PUN
25.58-25.81Mb
BRA
14.31-14.54Mb

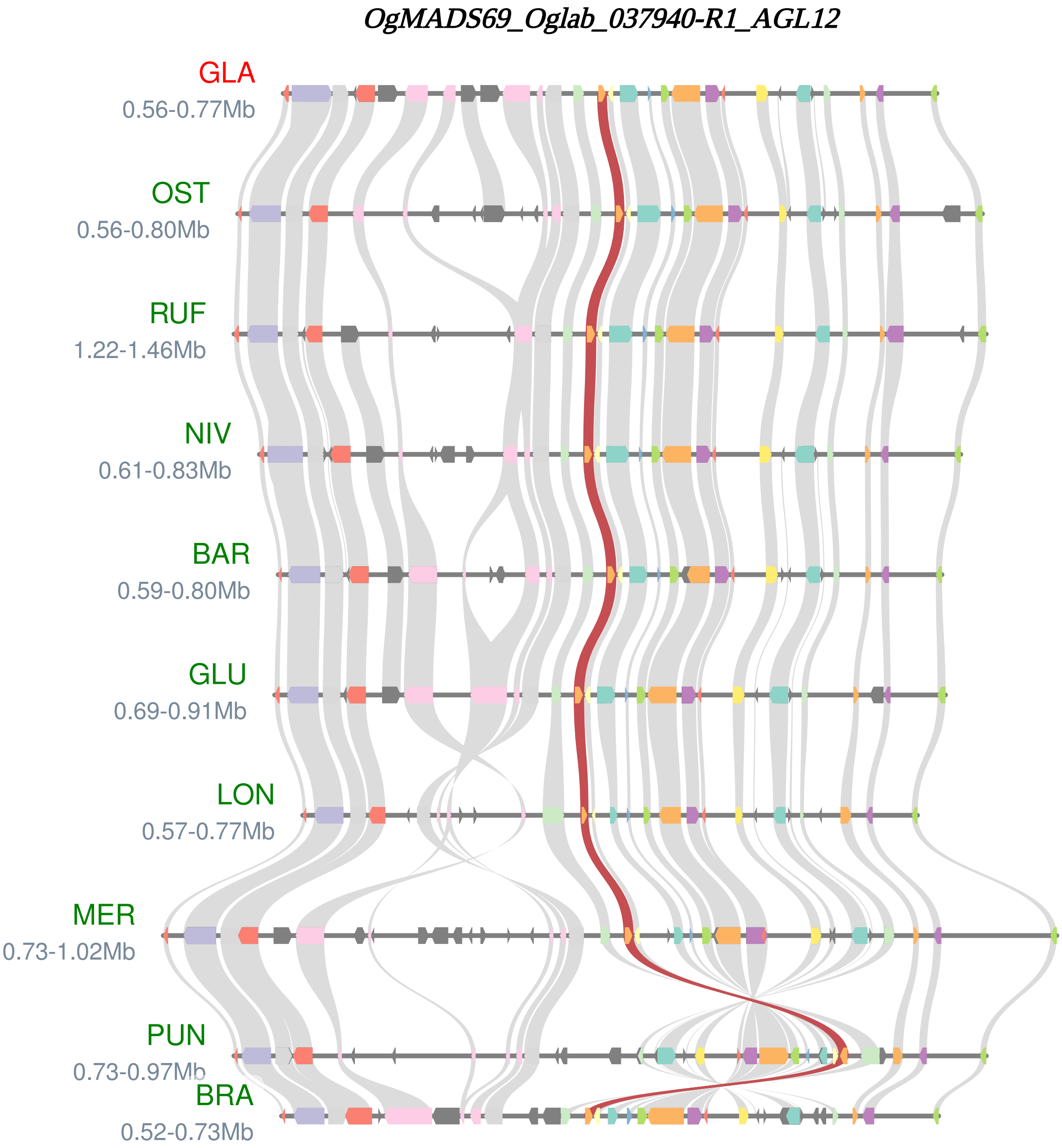
OgMADS69_Oglab_037940-R1_AGL12
GLA
0.56-0.77Mb
OST
0.56-0.80Mb
RUF
1.22-1.46Mb
NIV
0.61-0.83Mb
BAR
0.59-0.80Mb
GLU
0.69-0.91Mb
LON
0.57-0.77Mb
MER
0.73-1.02Mb
PUN
0.73-0.97Mb
BRA
0.52-0.73Mb

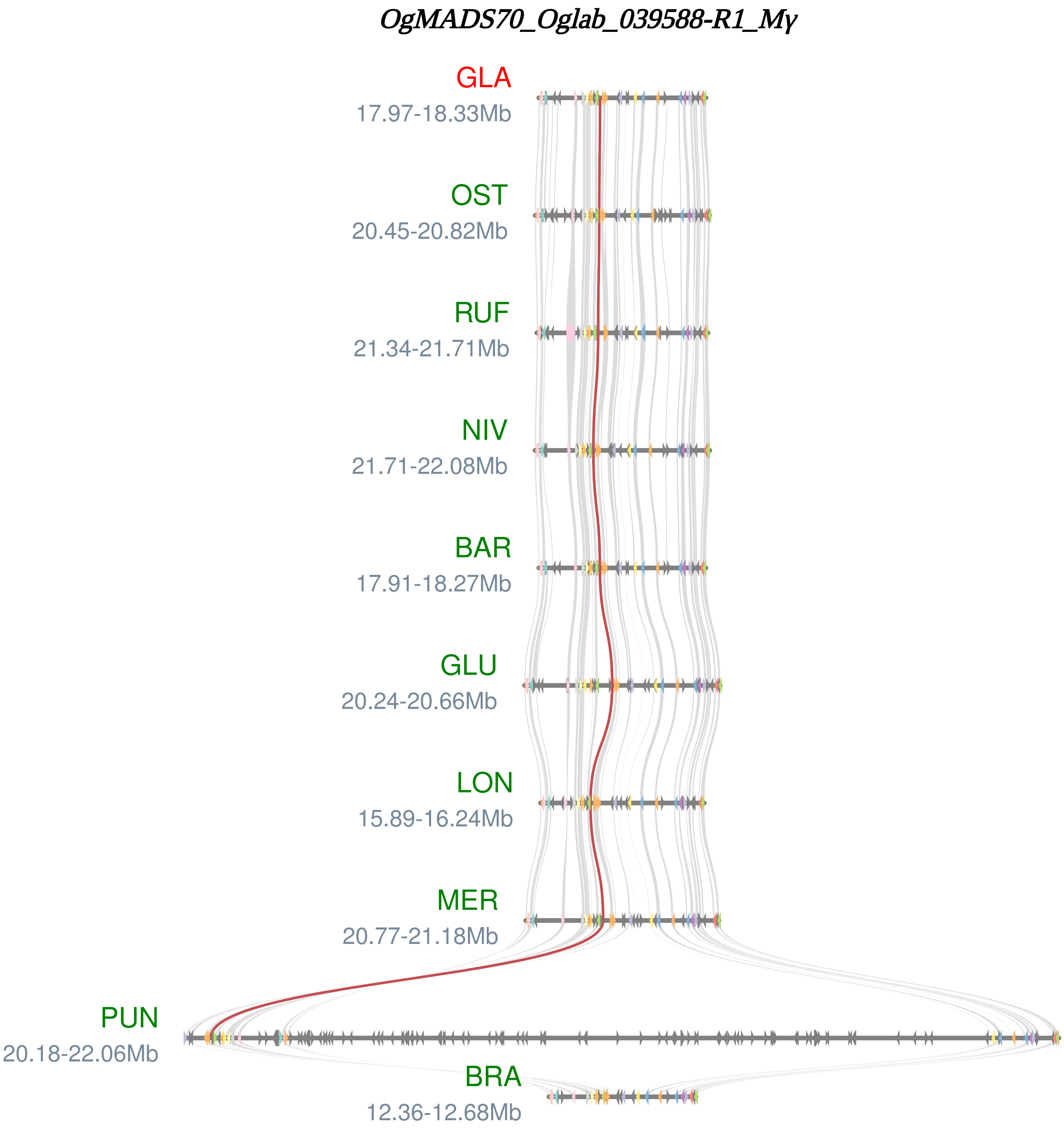

OgMADS70_Oglab_039588-R1_Mγ
GLA
17.97-18.33Mb
OST
20.45-20.82Mb
RUF
21.34-21.71Mb
NIV
21.71-22.08Mb
BAR
17.91-18.27Mb
GLU
20.24-20.66Mb
LON
15.89-16.24Mb
MER
20.77-21.18Mb
PUN
20.18-22.06Mb
BRA
12.36-12.68Mb

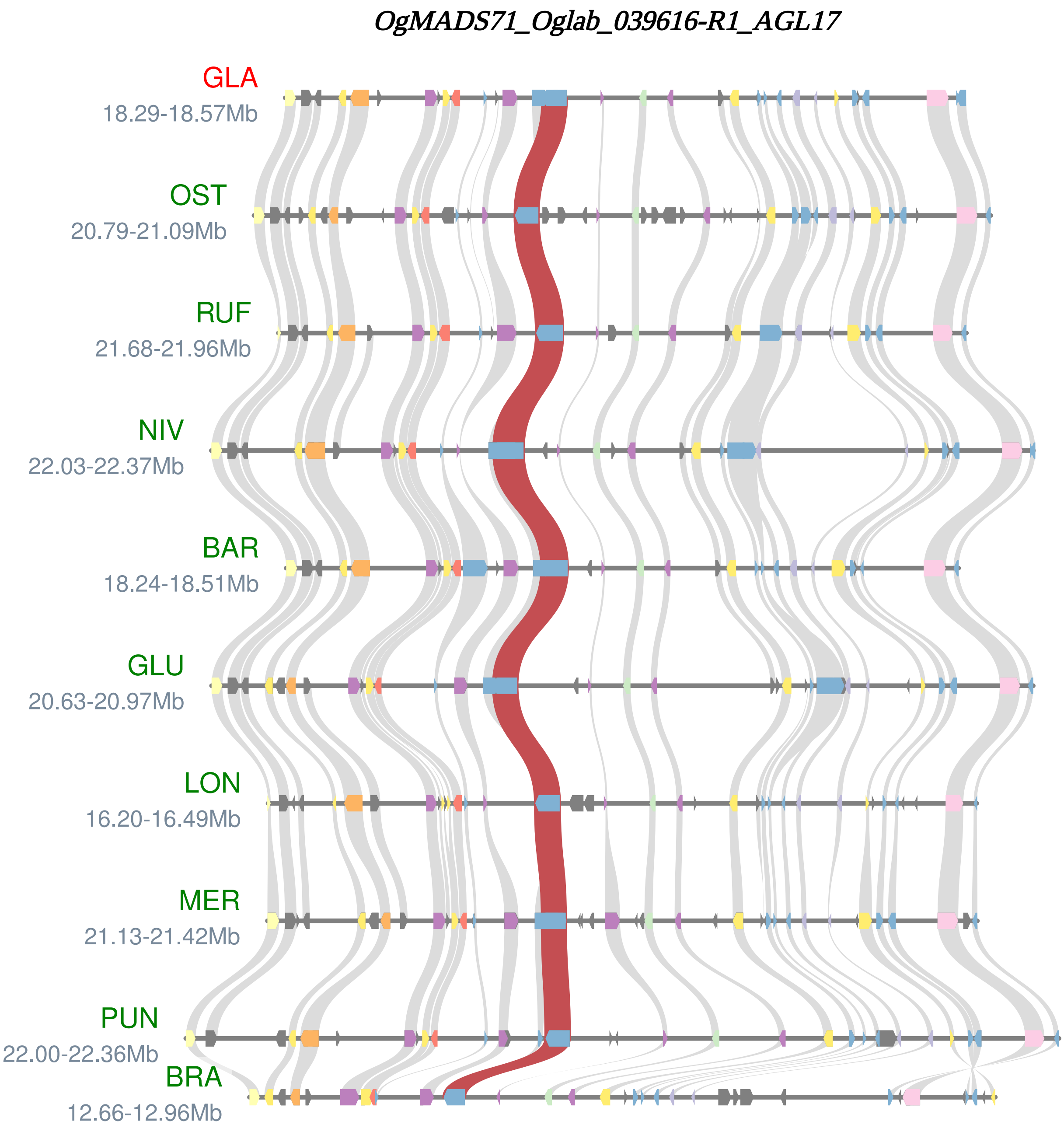
OgMADS71_Oglab_039616-R1_AGL17
GLA
18.29-18.57Mb
OST
20.79-21.09Mb
RUF
21.68-21.96Mb
NIV
22.03-22.37Mb
BAR
18.24-18.51Mb
GLU
20.63-20.97Mb
LON
16.20-16.49Mb
MER
21.13-21.42Mb
PUN
22.00-22.36Mb
BRA
12.66-12.96Mb

*OgMADS72_Oglab_039996-R1_MIKC_*

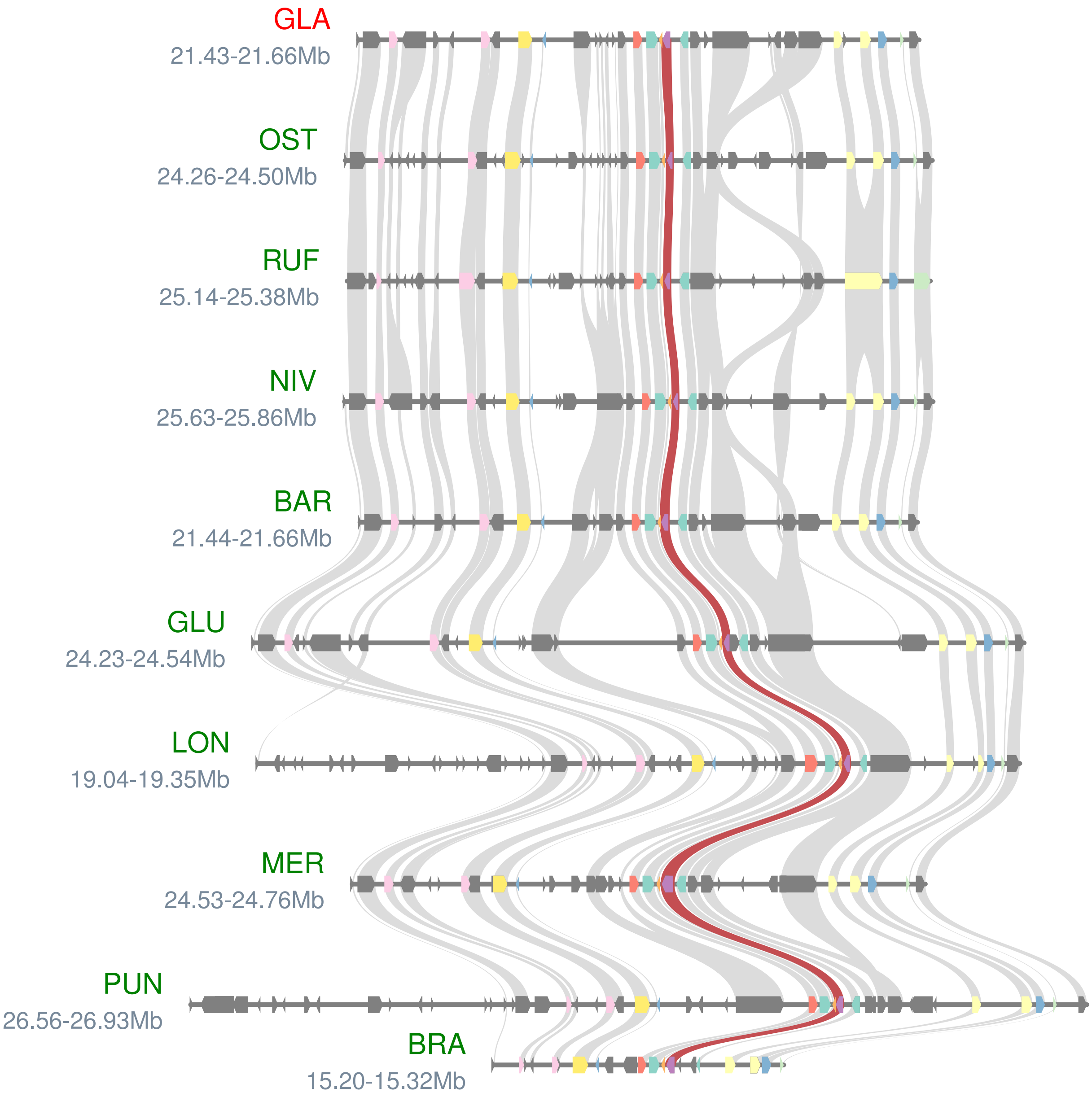

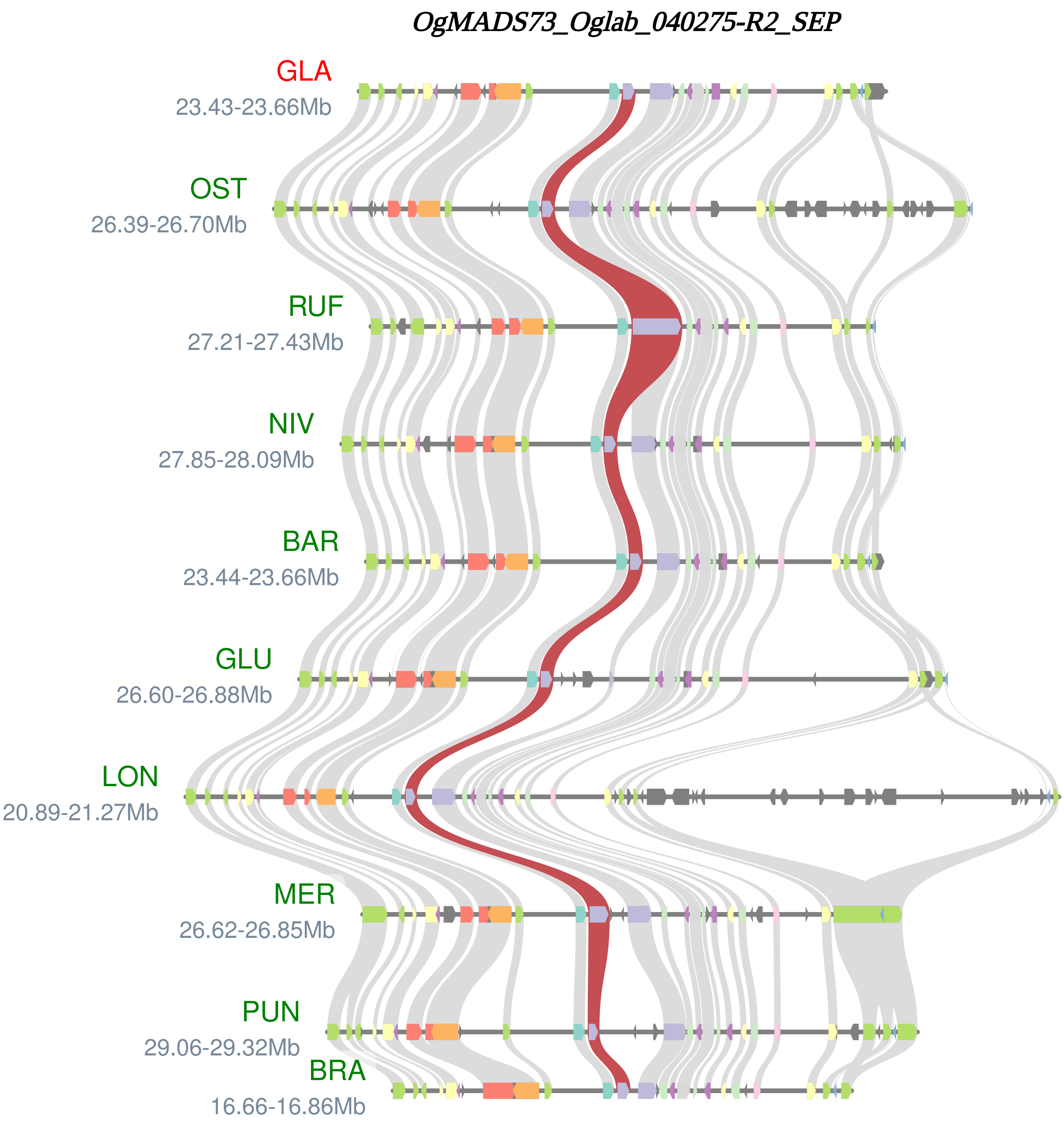

OgMADS73_Oglab_040275-R2_SEP
GLA
23.43-23.66Mb
OST
26.39-26.70Mb
RUF
27.21-27.43Mb
NIV
27.85-28.09Mb
BAR
23.44-23.66Mb
GLU
26.60-26.88Mb
LON
20.89-21.27Mb
MER
26.62-26.85Mb
PUN
29.06-29.32Mb
BRA
16.66-16.86Mb

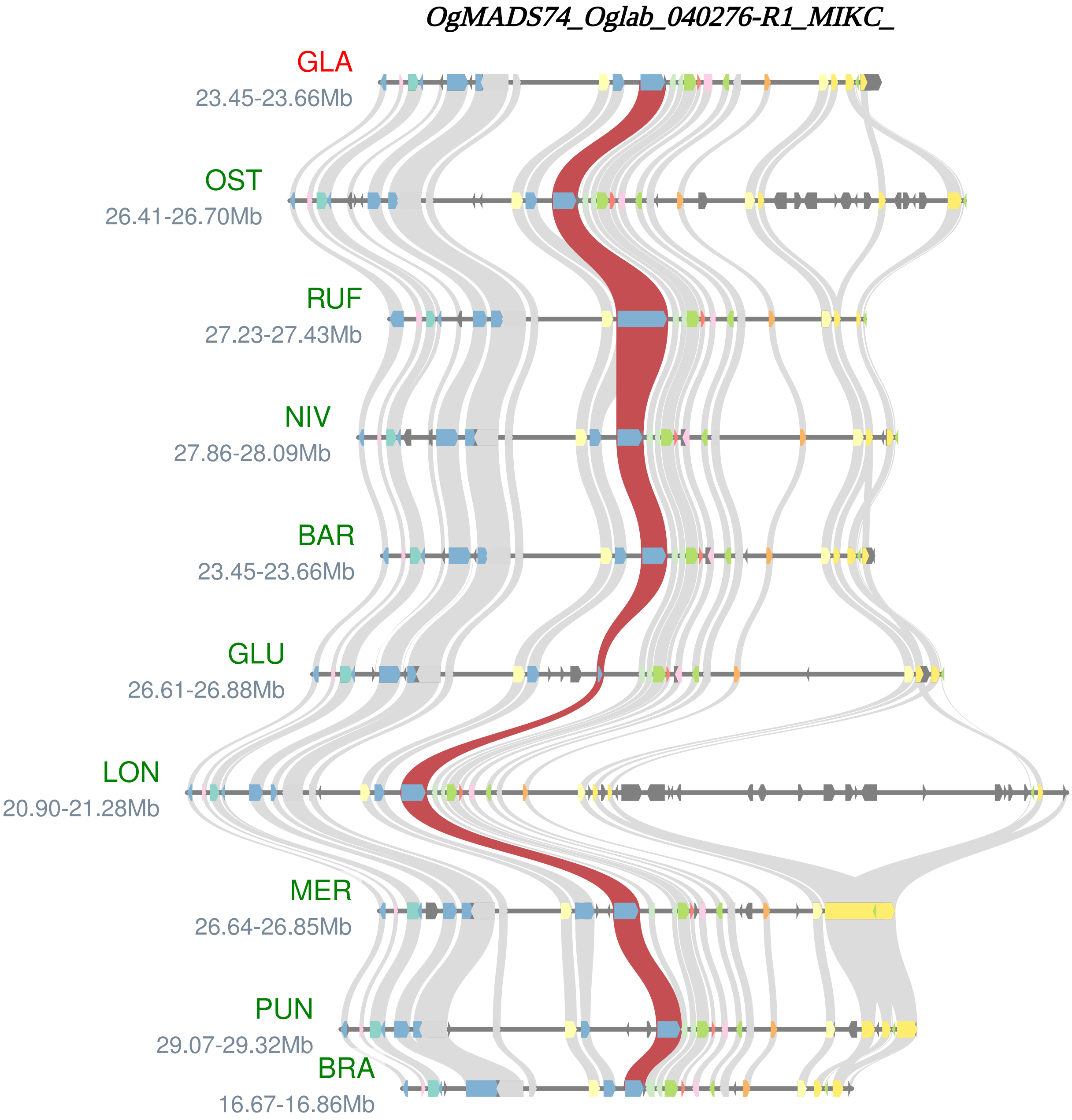
OgMADS74_Oglab_040276-R1_MIKC_
GLA
23.45-23.66Mb
OST
26.41-26.70Mb
RUF
27.23-27.43Mb
NIV
27.86-28.09Mb
BAR
23.45-23.66Mb
GLU
26.61-26.88Mb
LON
20.90-21.28Mb
MER
26.64-26.85Mb
PUN
29.07-29.32Mb
BRA
16.67-16.86Mb

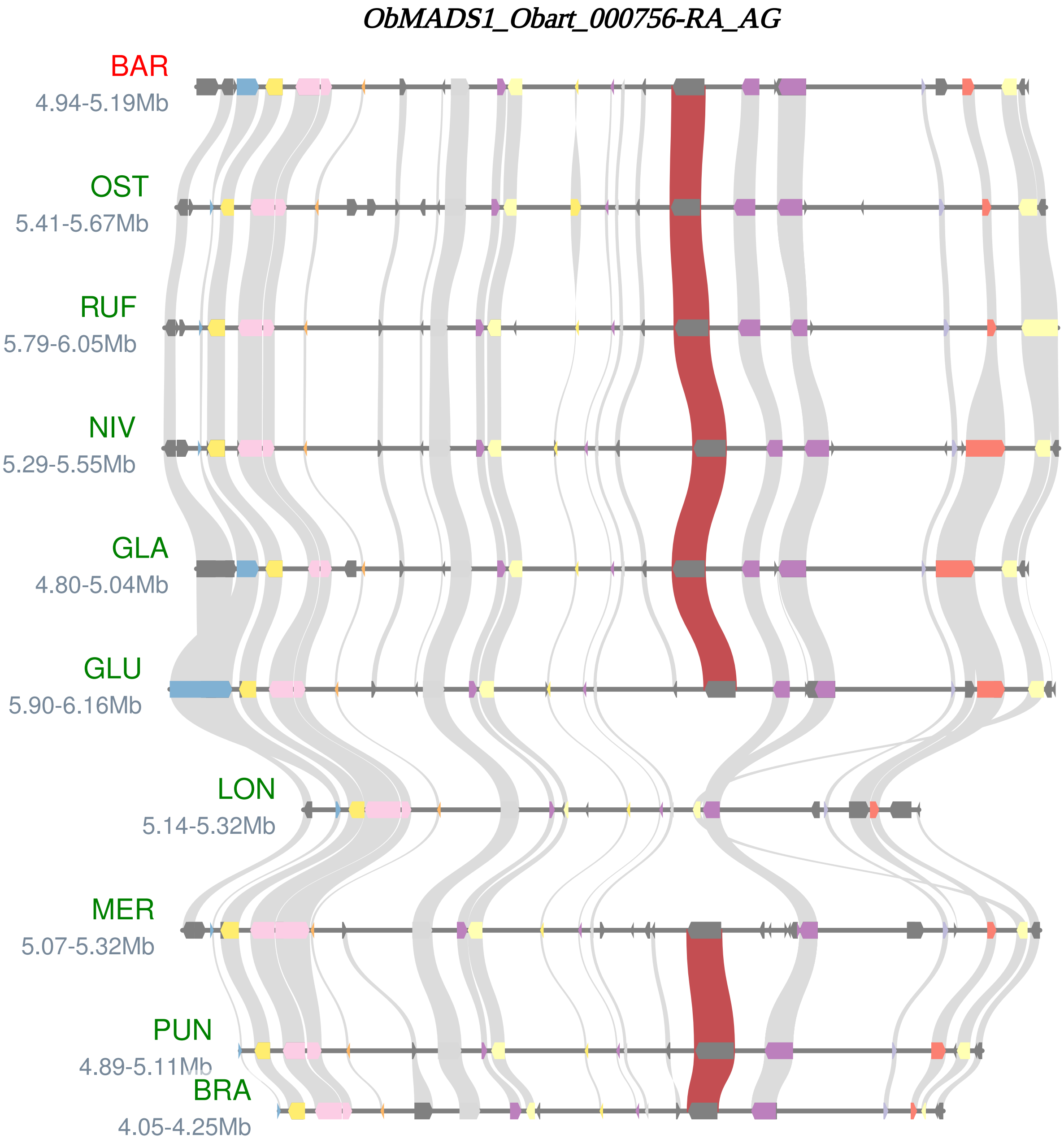
ObMADS1_Obart_000756-RA_AG
BAR
4.94-5.19Mb
OST
5.41-5.67Mb
RUF
5.79-6.05Mb
NIV
5.29-5.55Mb
GLA
4.80-5.04Mb
GLU
5.90-6.16Mb
LON
5.14-5.32Mb
MER
5.07-5.32Mb
PUN
4.89-5.11Mb
BRA
4.05-4.25Mb

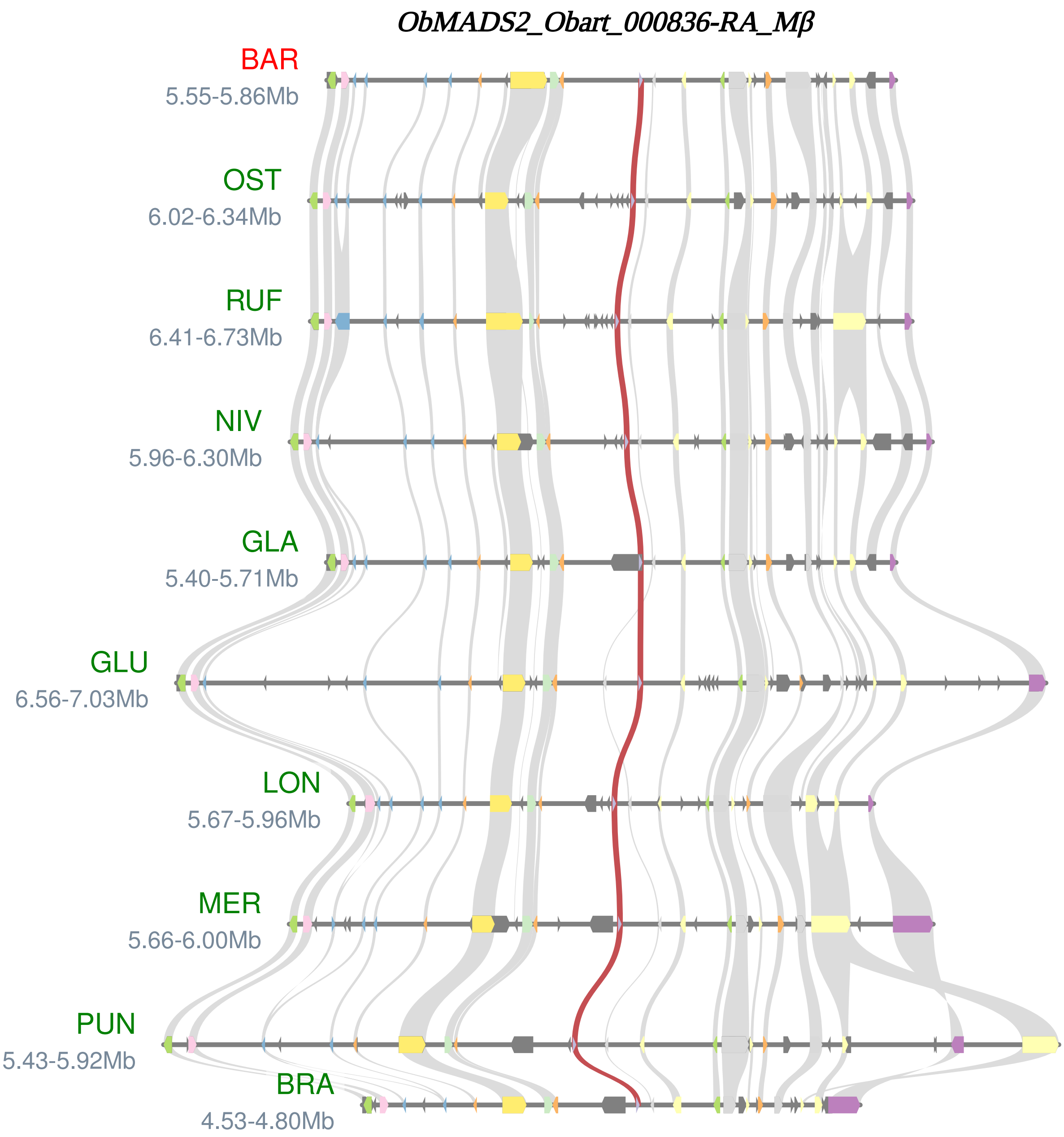
ObMADS2_Obart_000836-RA_Mβ
BAR
5.55-5.86Mb
OST
6.02-6.34Mb
RUF
6.41-6.73Mb
NIV
5.96-6.30Mb
GLA
5.40-5.71Mb
GLU
6.56-7.03Mb
LON
5.67-5.96Mb
MER
5.66-6.00Mb
PUN
5.43-5.92Mb
BRA
4.53-4.80Mb

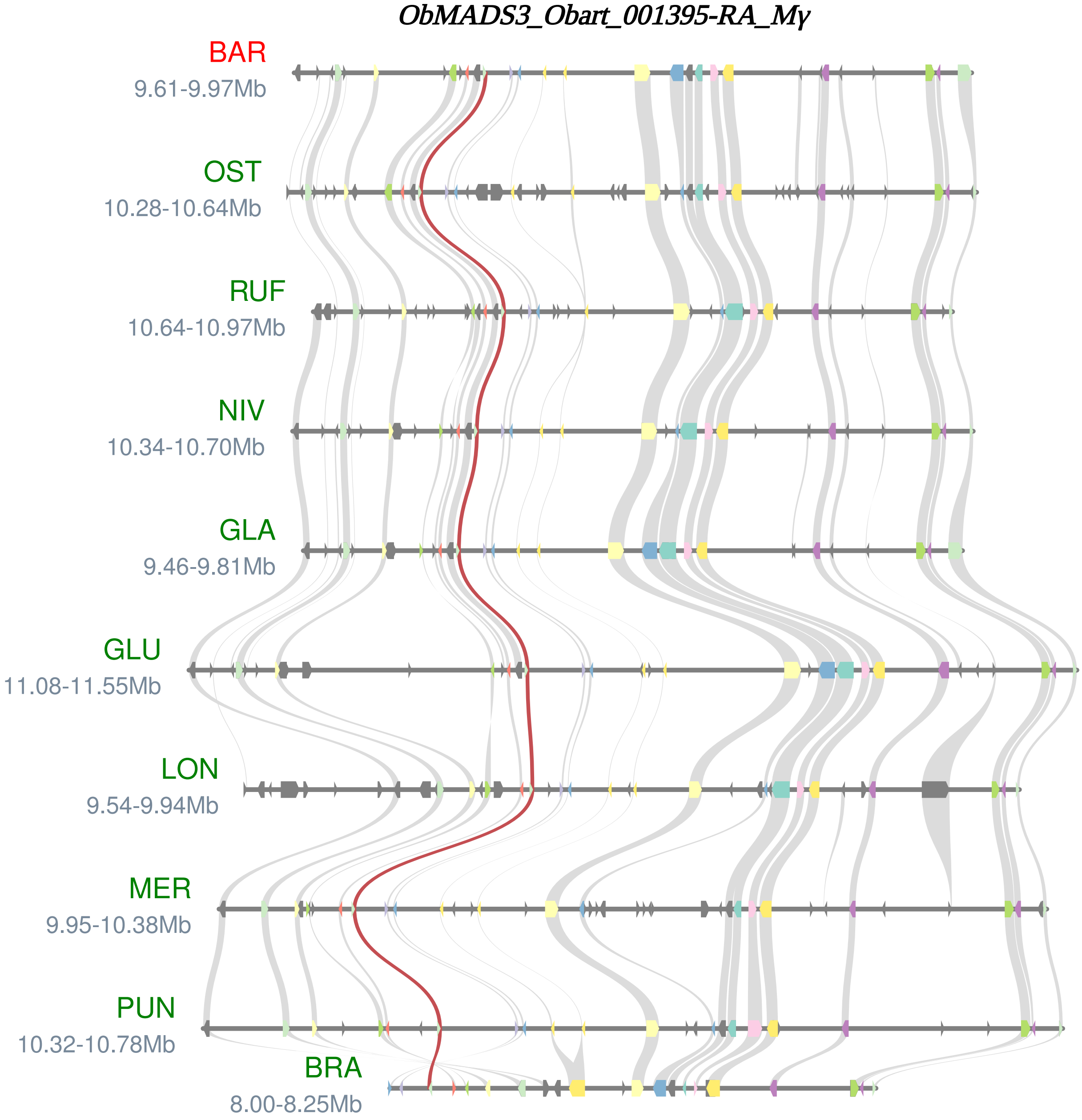

ObMADS3_Obart_001395-RA_Mγ
BAR
9.61-9.97Mb
OST
10.28-10.64Mb
RUF
10.64-10.97Mb
NIV
10.34-10.70Mb
GLA
9.46-9.81Mb
GLU
11.08-11.55Mb
LON
9.54-9.94Mb
MER
9.95-10.38Mb
PUN
10.32-10.78Mb
BRA
8.00-8.25Mb

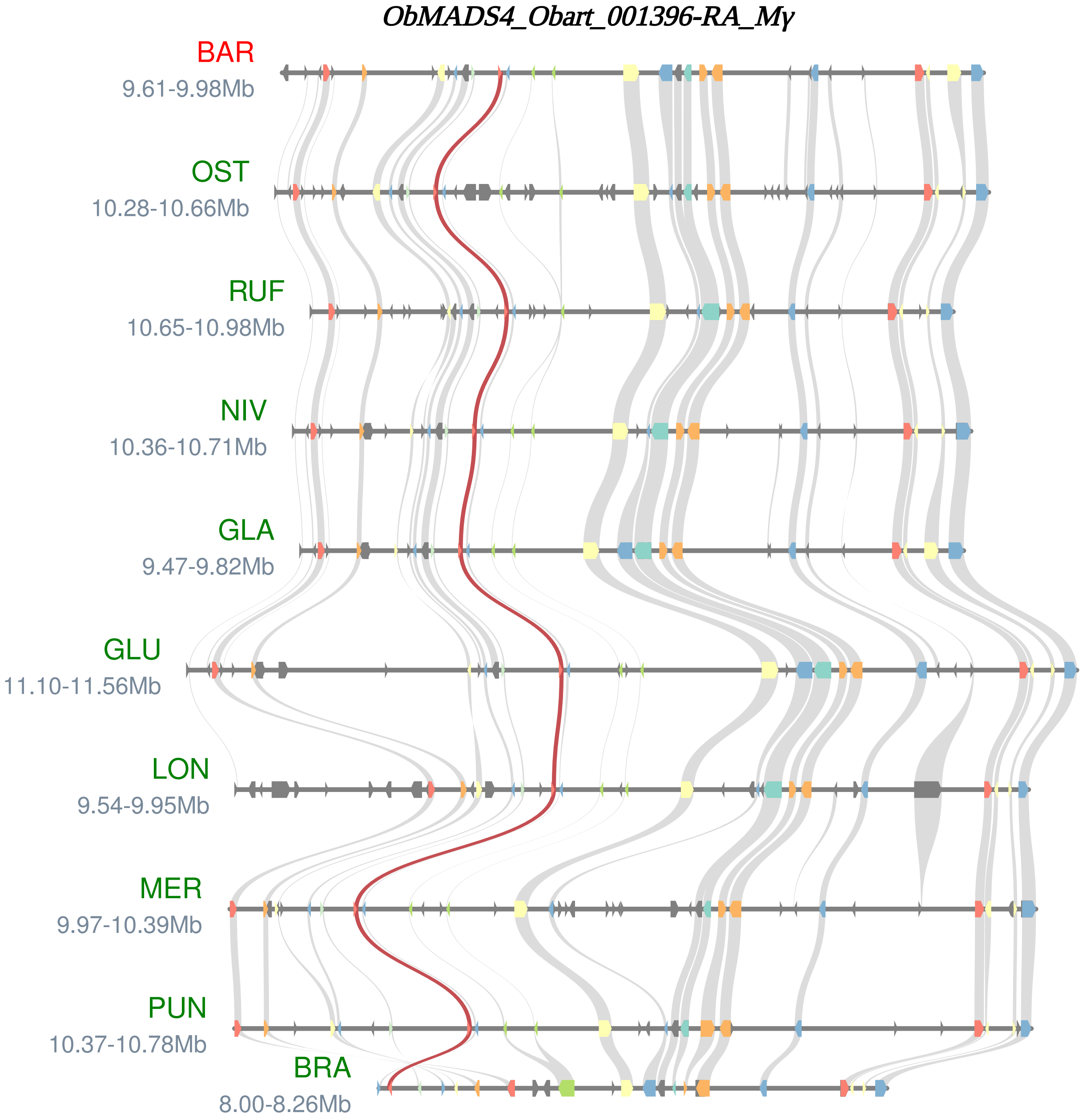
ObMADS4_Obart_001396-RA_Mγ
BAR
9.61-9.98Mb
OST
10.28-10.66Mb
RUF
10.65-10.98Mb
NIV
10.36-10.71Mb
GLA
9.47-9.82Mb
GLU
11.10-11.56Mb
LON
9.54-9.95Mb
MER
9.97-10.39Mb
PUN
10.37-10.78Mb
BRA
8.00-8.26Mb

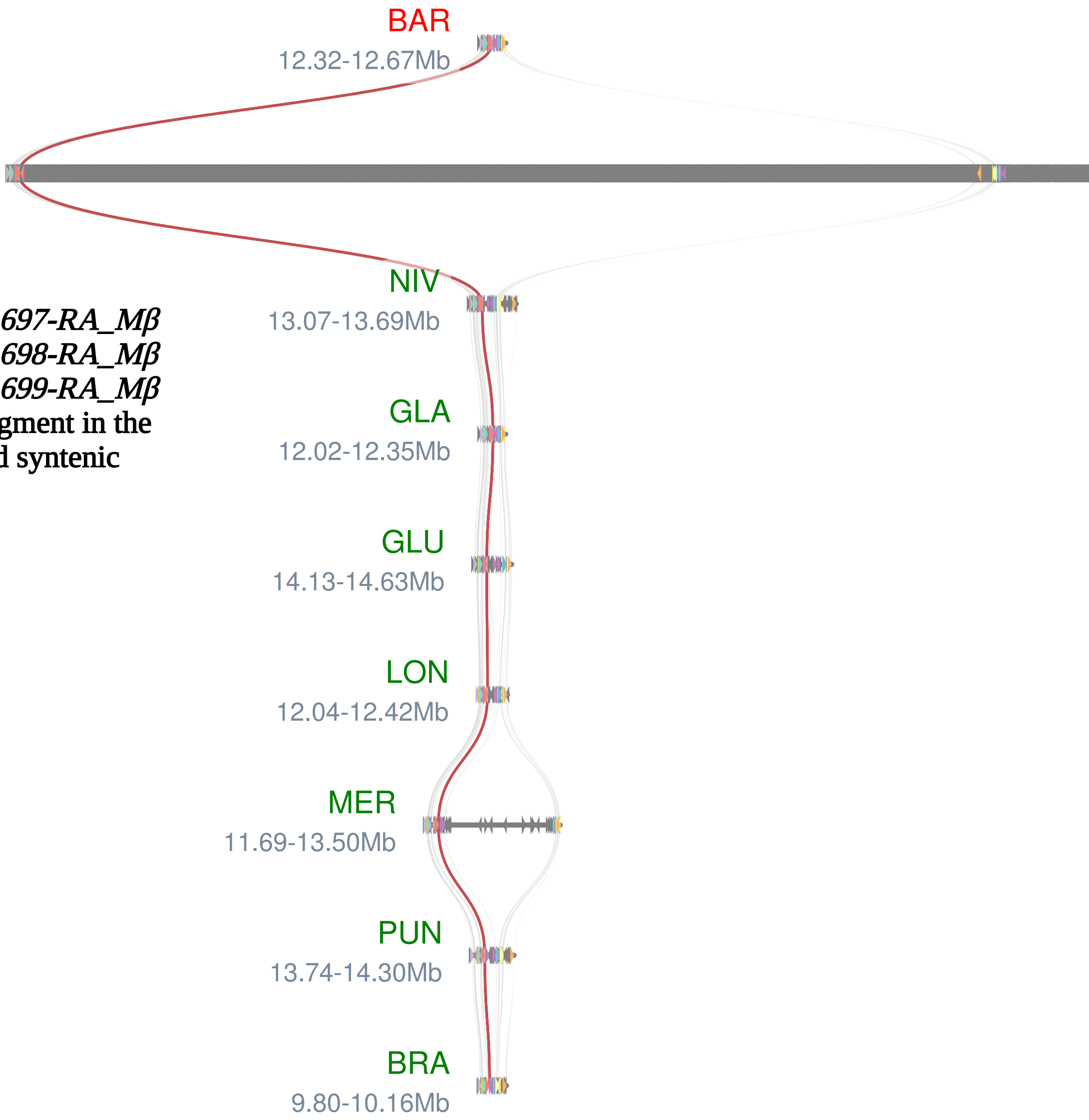

ObMADS5_Obart_001697-RA_Mβ
ObMADS6_Obart_001698-RA_Mβ
ObMADS7_Obart_001699-RA_Mβ
（The chromosomal segment in the OST lacks any detected syntenic genes.)

ObMADS8_Obart_003478-RA_OsMADS32

(The chromosomal segment in the RUF lacks any detected syntenic genes.)

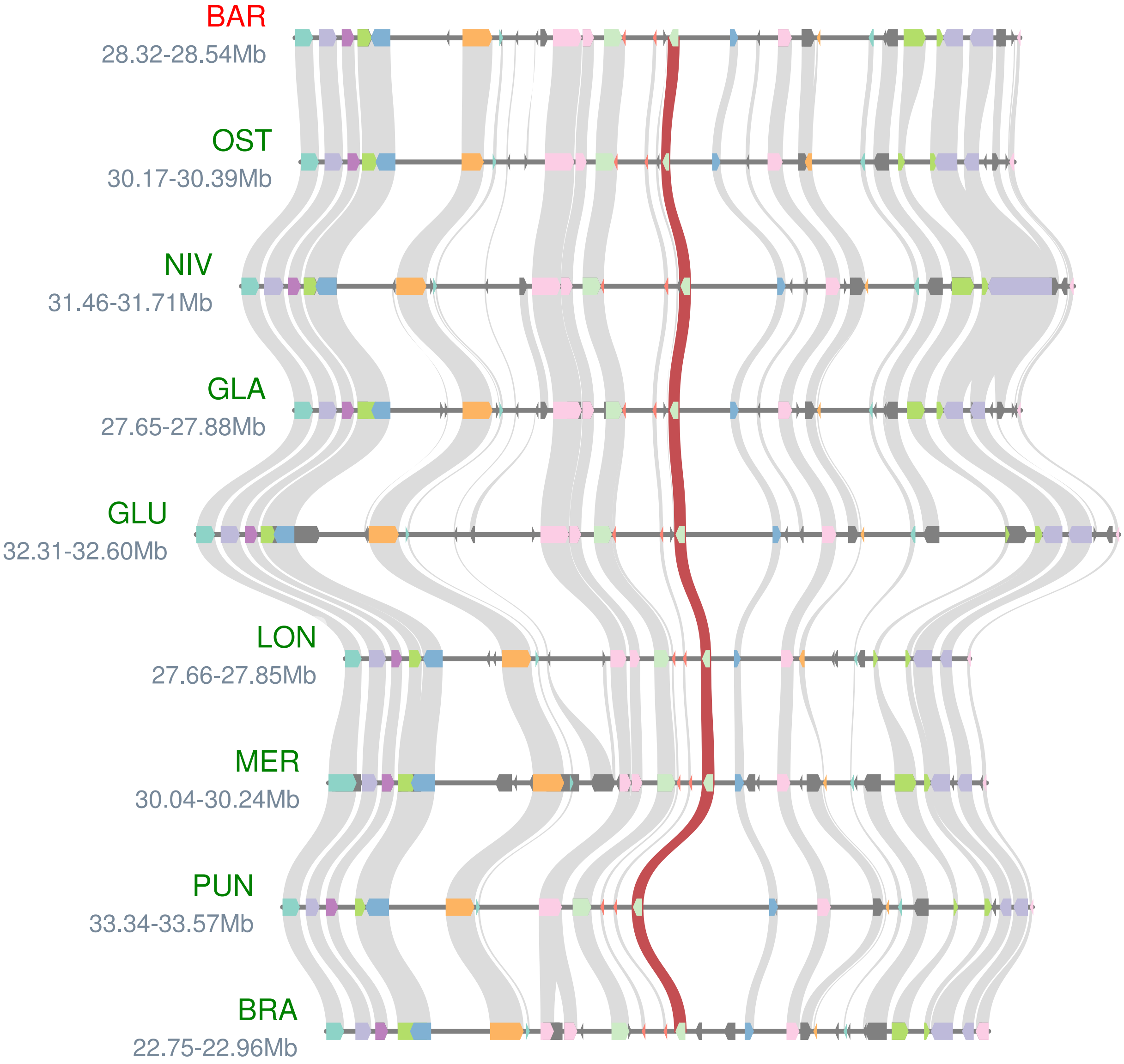

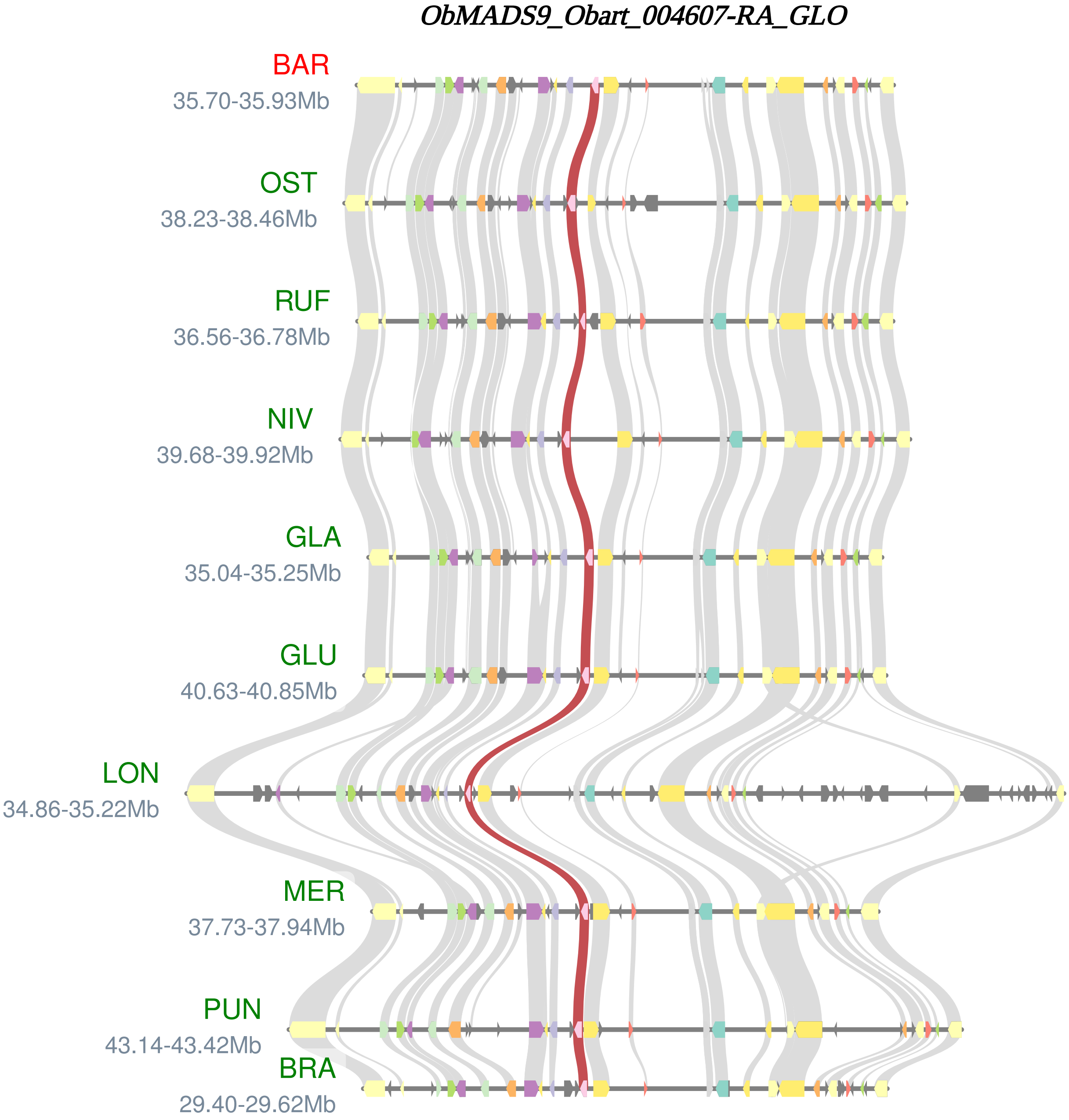
ObMADS9_Obart_004607-RA_GLO
BAR
35.70-35.93Mb
OST
38.23-38.46Mb
RUF
36.56-36.78Mb
NIV
39.68-39.92Mb
GLA
35.04-35.25Mb
GLU
40.63-40.85Mb
LON
34.86-35.22Mb
MER
37.73-37.94Mb
PUN
43.14-43.42Mb
BRA
29.40-29.62Mb

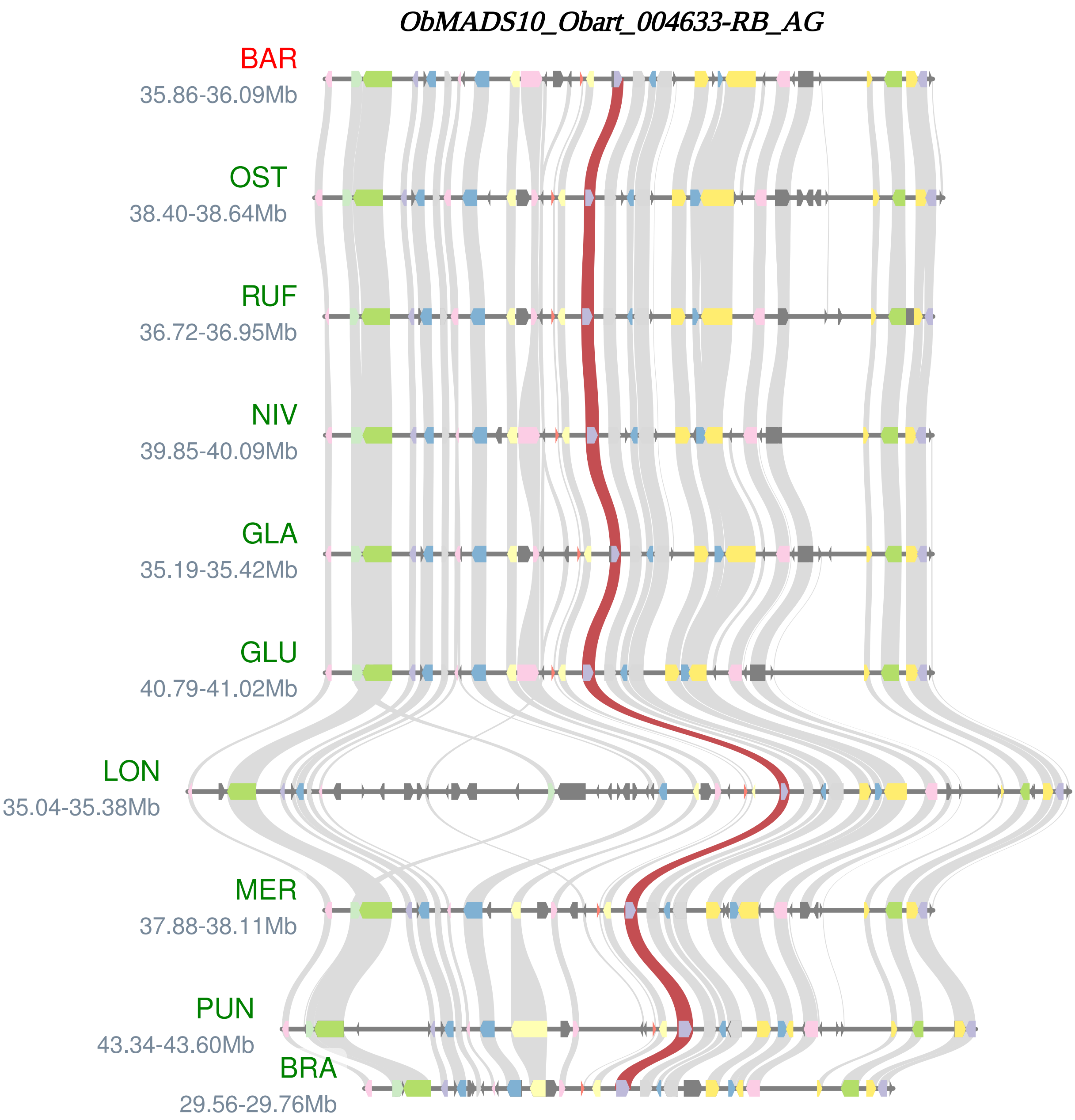
ObMADS10_Obart_004633-RB_AG
BAR
35.86-36.09Mb
OST
38.40-38.64Mb
RUF
36.72-36.95Mb
NIV
39.85-40.09Mb
GLA
35.19-35.42Mb
GLU
40.79-41.02Mb
LON
35.04-35.38Mb
MER
37.88-38.11Mb
PUN
43.34-43.60Mb
BRA
29.56-29.76Mb

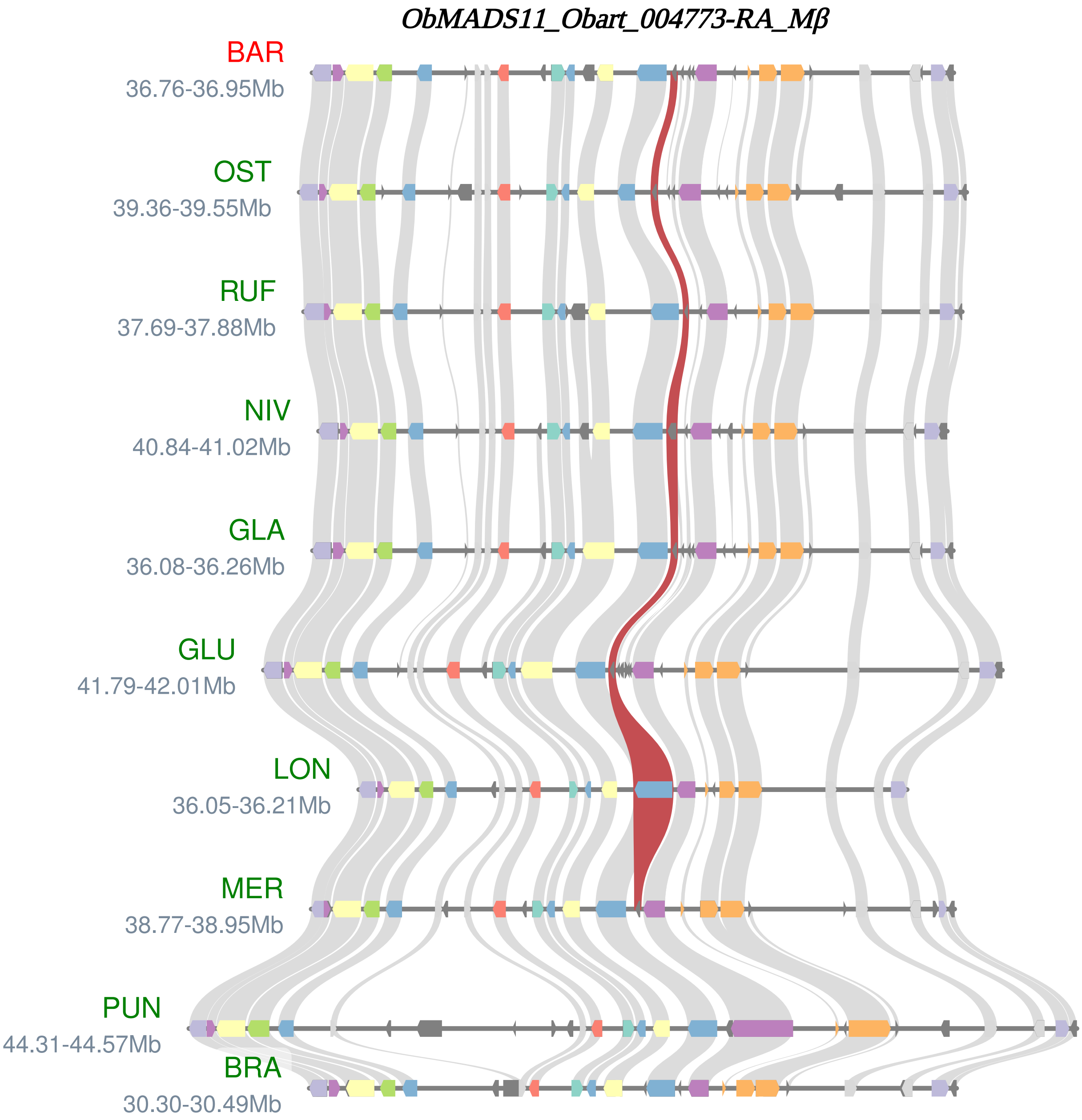

ObMADS11_Obart_004773-RA_Mβ
BAR
36.76-36.95Mb
OST
39.36-39.55Mb
RUF
37.69-37.88Mb
NIV
40.84-41.02Mb
GLA
36.08-36.26Mb
GLU
41.79-42.01Mb
LON
36.05-36.21Mb
MER
38.77-38.95Mb
PUN
44.31-44.57Mb
BRA
30.30-30.49Mb

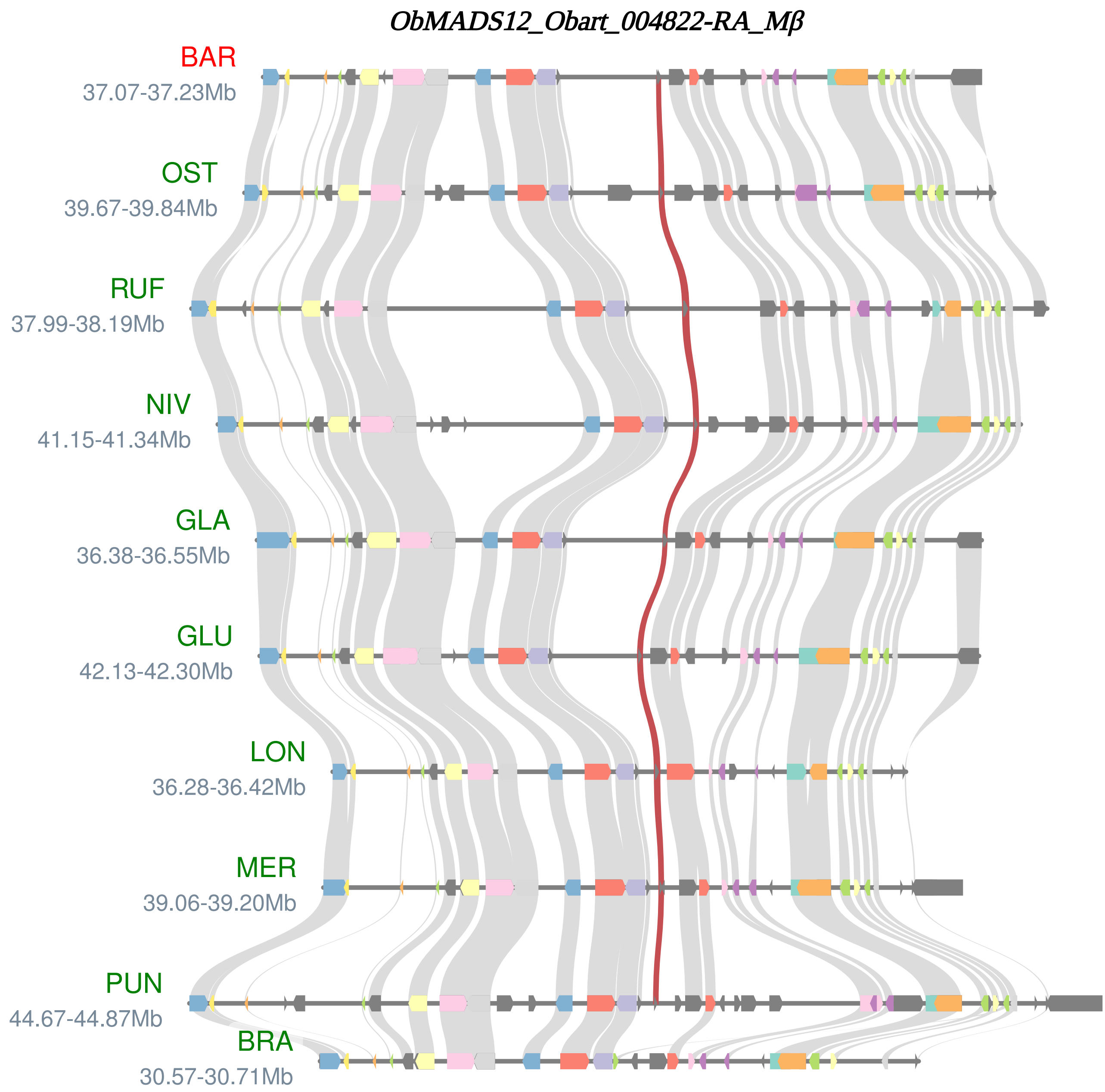

ObMADS12_Obart_004822-RA_Mβ
BAR
37.07-37.23Mb
OST
39.67-39.84Mb
RUF
37.99-38.19Mb
NIV
41.15-41.34Mb
GLA
36.38-36.55Mb
GLU
42.13-42.30Mb
LON
36.28-36.42Mb
MER
39.06-39.20Mb
PUN
44.67-44.87Mb
BRA
30.57-30.71Mb

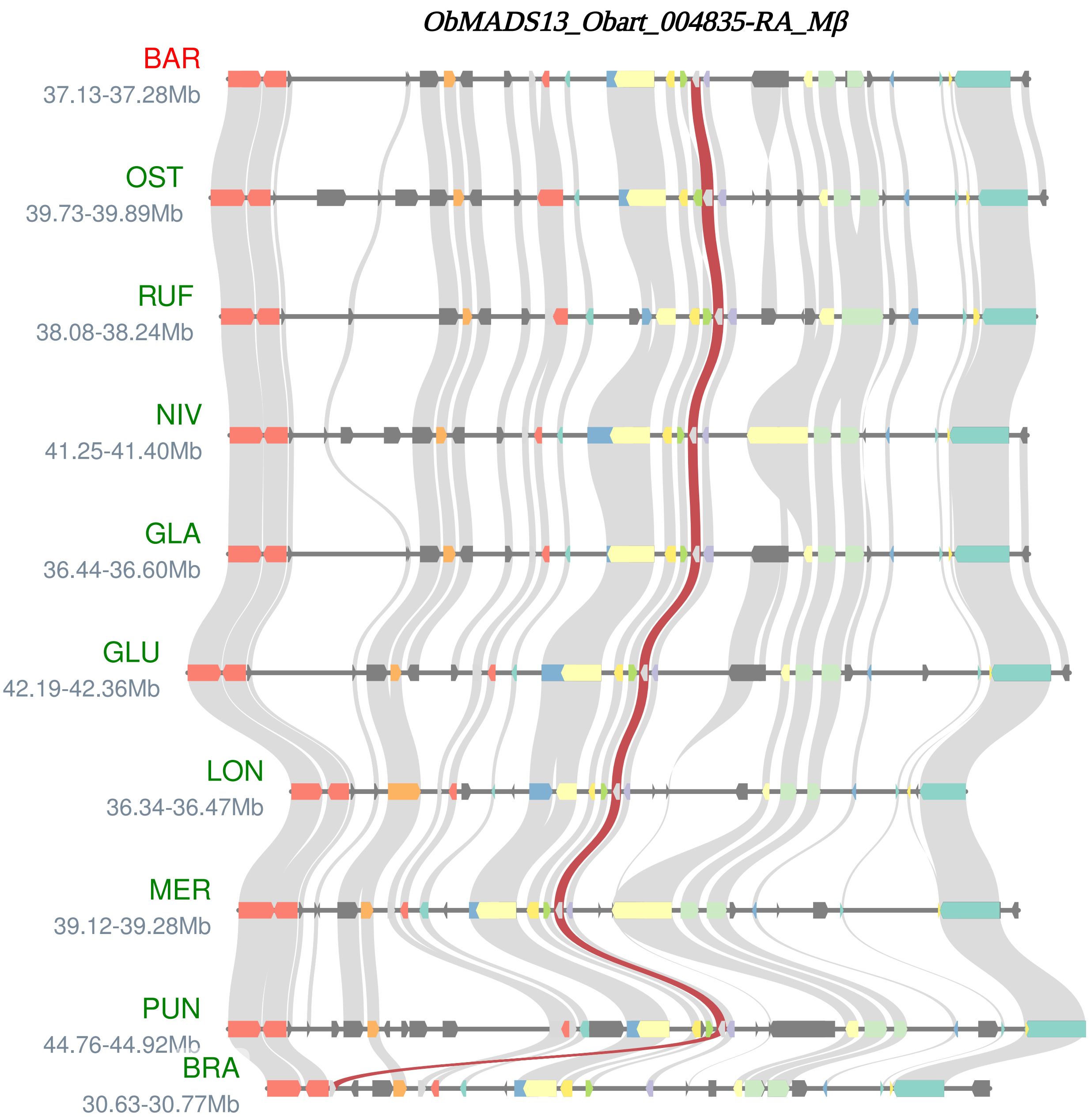
ObMADS13_Obart_004835-RA_Mβ
BAR
37.13-37.28Mb
OST
39.73-39.89Mb
RUF
38.08-38.24Mb
NIV
41.25-41.40Mb
GLA
36.44-36.60Mb
GLU
42.19-42.36Mb
LON
36.34-36.47Mb
MER
39.12-39.28Mb
PUN
44.76-44.92Mb
BRA
30.63-30.77Mb

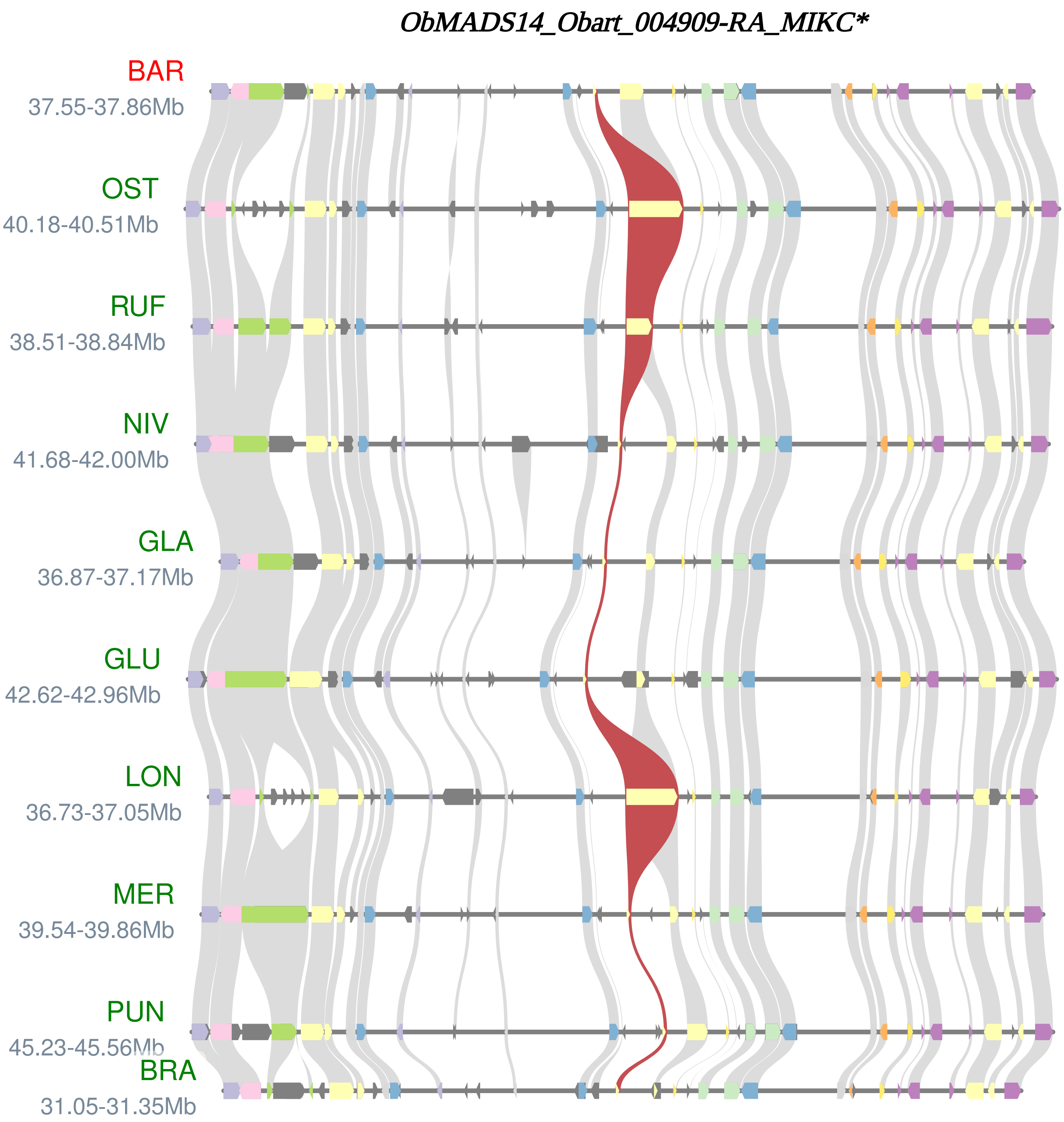

ObMADS14_Obart_004909-RA_MIKC*
BAR
37.55-37.86Mb
OST
40.18-40.51Mb
RUF
38.51-38.84Mb
NIV
41.68-42.00Mb
GLA
36.87-37.17Mb
GLU
42.62-42.96Mb
LON
36.73-37.05Mb
MER
39.54-39.86Mb
PUN
45.23-45.56Mb
BRA
31.05-31.35Mb

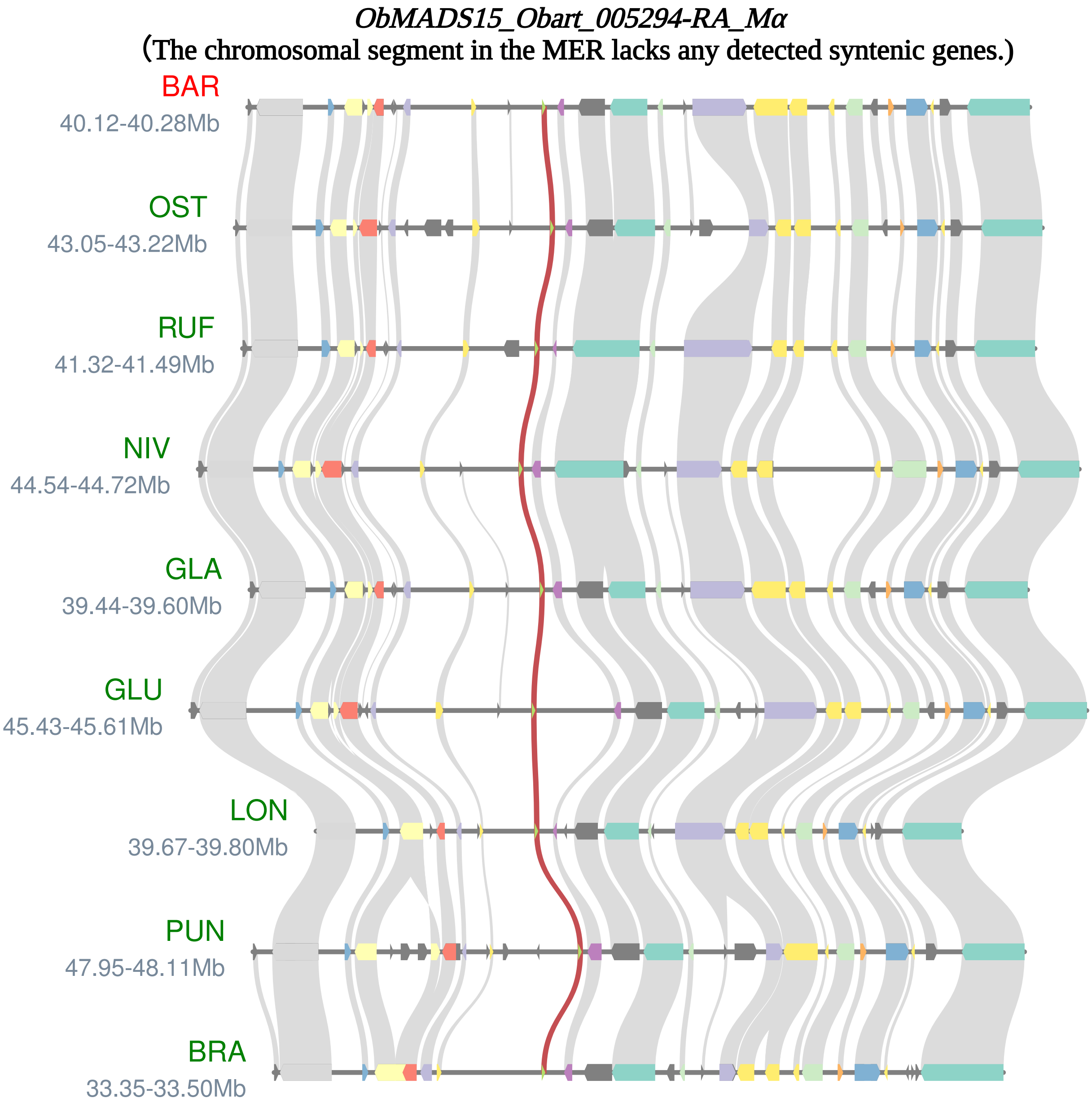
ObMADS15_Obart_005294-RA_Mα
(The chromosomal segment in the MER lacks any detected syntenic genes.)
BAR
40.12-40.28Mb
OST
43.05-43.22Mb
RUF
41.32-41.49Mb
NIV
44.54-44.72Mb
GLA
39.44-39.60Mb
GLU
45.43-45.61Mb
LON
39.67-39.80Mb
PUN
47.95-48.11Mb
BRA
33.35-33.50Mb

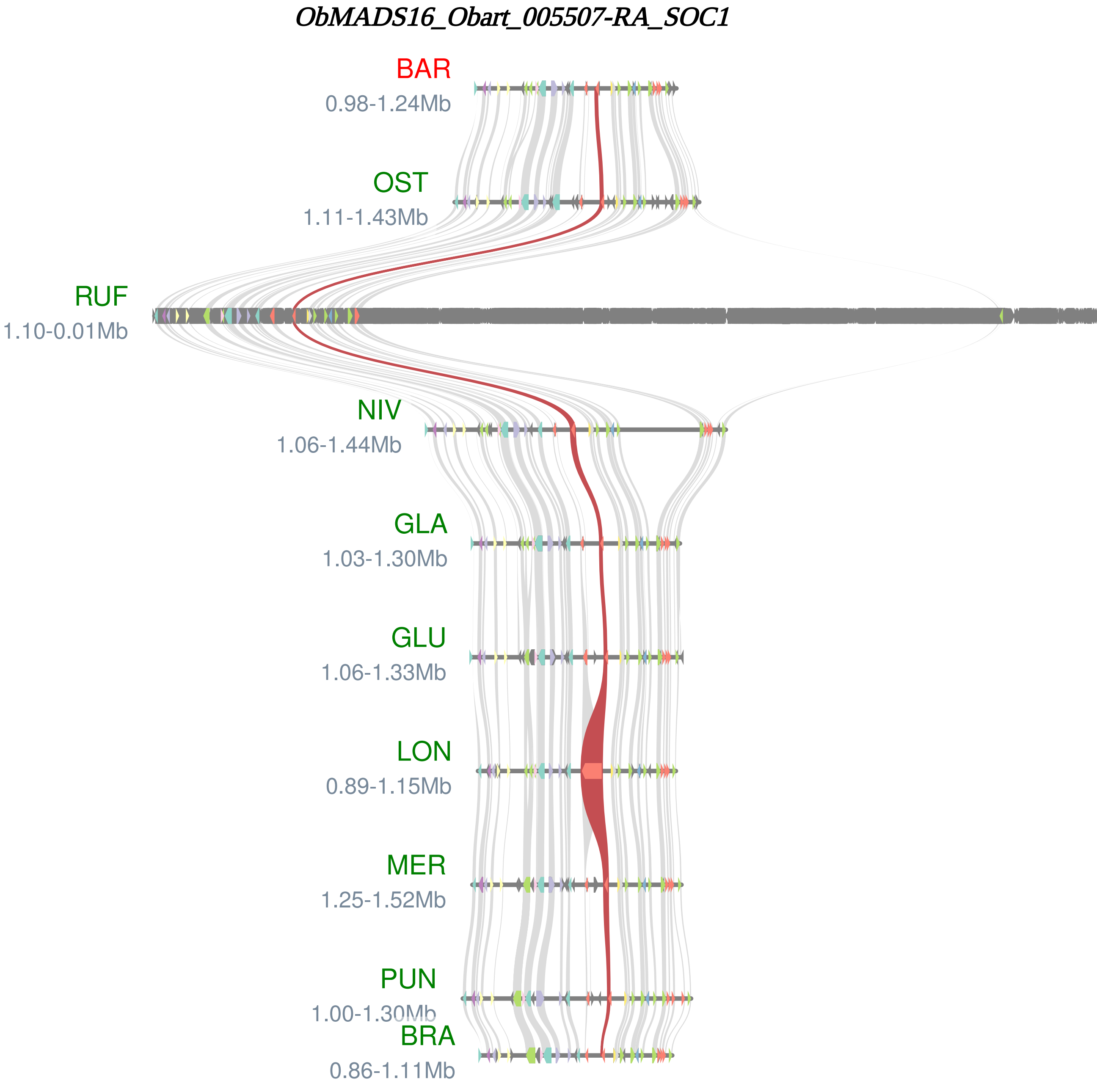

ObMADS16_Obart_005507-RA_SOC1
BAR
0.98-1.24Mb
OST
1.11-1.43Mb
RUF
1.10-0.01Mb
NIV
1.06-1.44Mb
GLA
1.03-1.30Mb
GLU
1.06-1.33Mb
LON
0.89-1.15Mb
MER
1.25-1.52Mb
PUN
1.00-1.30Mb
BRA
0.86-1.11Mb

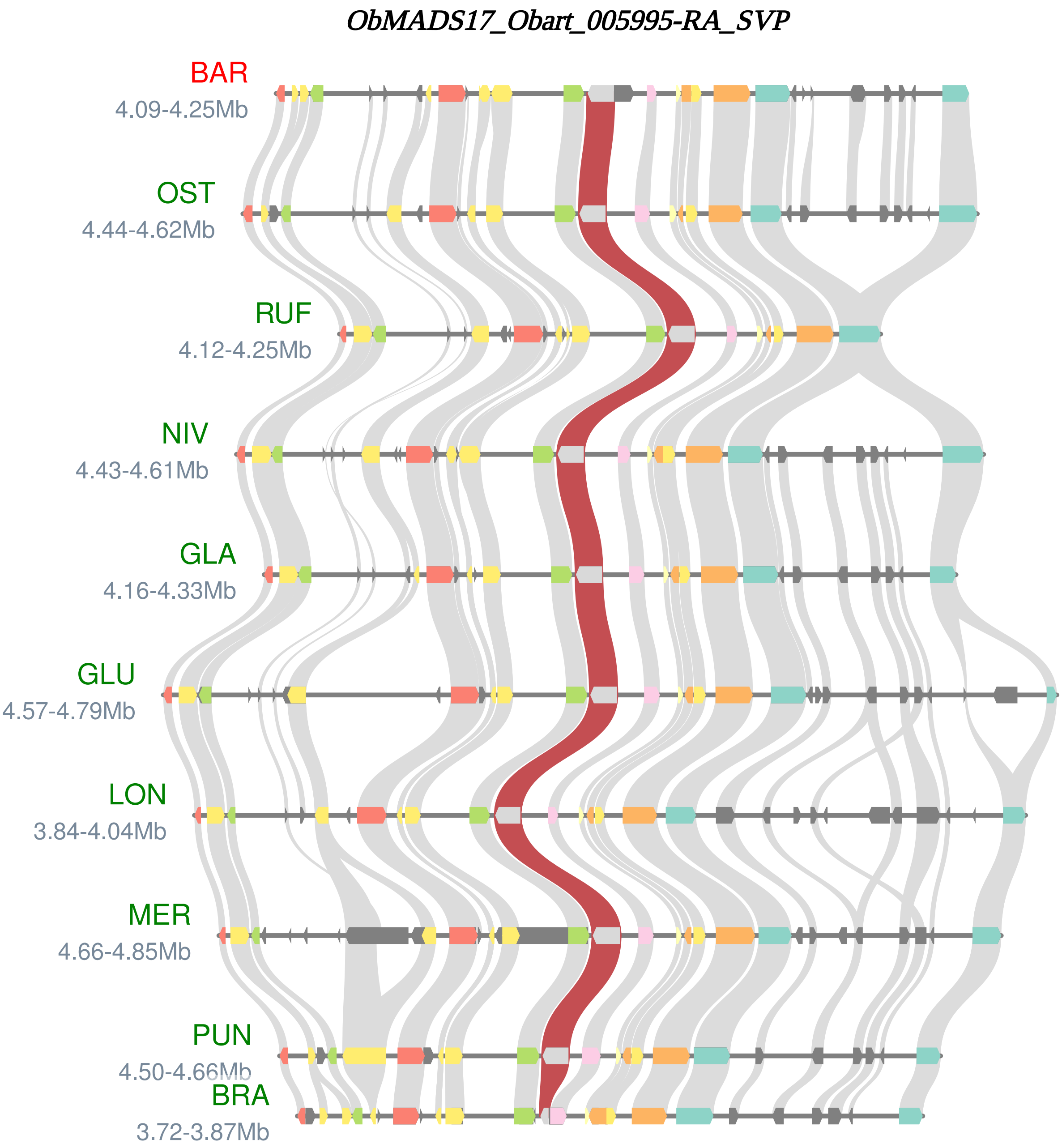

ObMADS17_Obart_005995-RA_SVP
BAR
4.09-4.25Mb
OST
4.44-4.62Mb
RUF
4.12-4.25Mb
NIV
4.43-4.61Mb
GLA
4.16-4.33Mb
GLU
4.57-4.79Mb
LON
3.84-4.04Mb
MER
4.66-4.85Mb
PUN
4.50-4.66Mb
BRA
3.72-3.87Mb

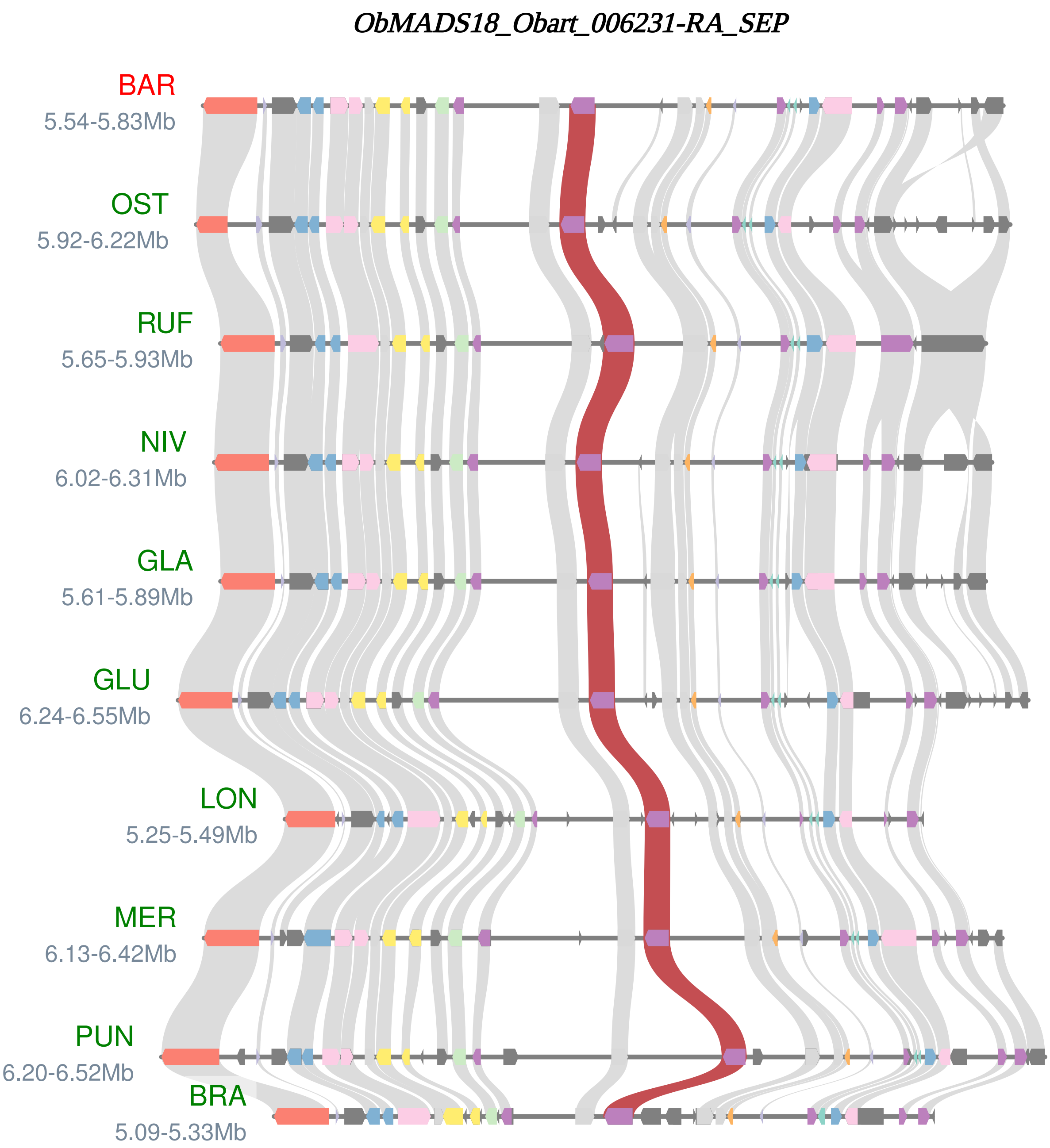
ObMADS18_Obart_006231-RA_SEP
BAR
5.54-5.83Mb
OST
5.92-6.22Mb
RUF
5.65-5.93Mb
NIV
6.02-6.31Mb
GLA
5.61-5.89Mb
GLU
6.24-6.55Mb
LON
5.25-5.49Mb
MER
6.13-6.42Mb
PUN
6.20-6.52Mb
BRA
5.09-5.33Mb

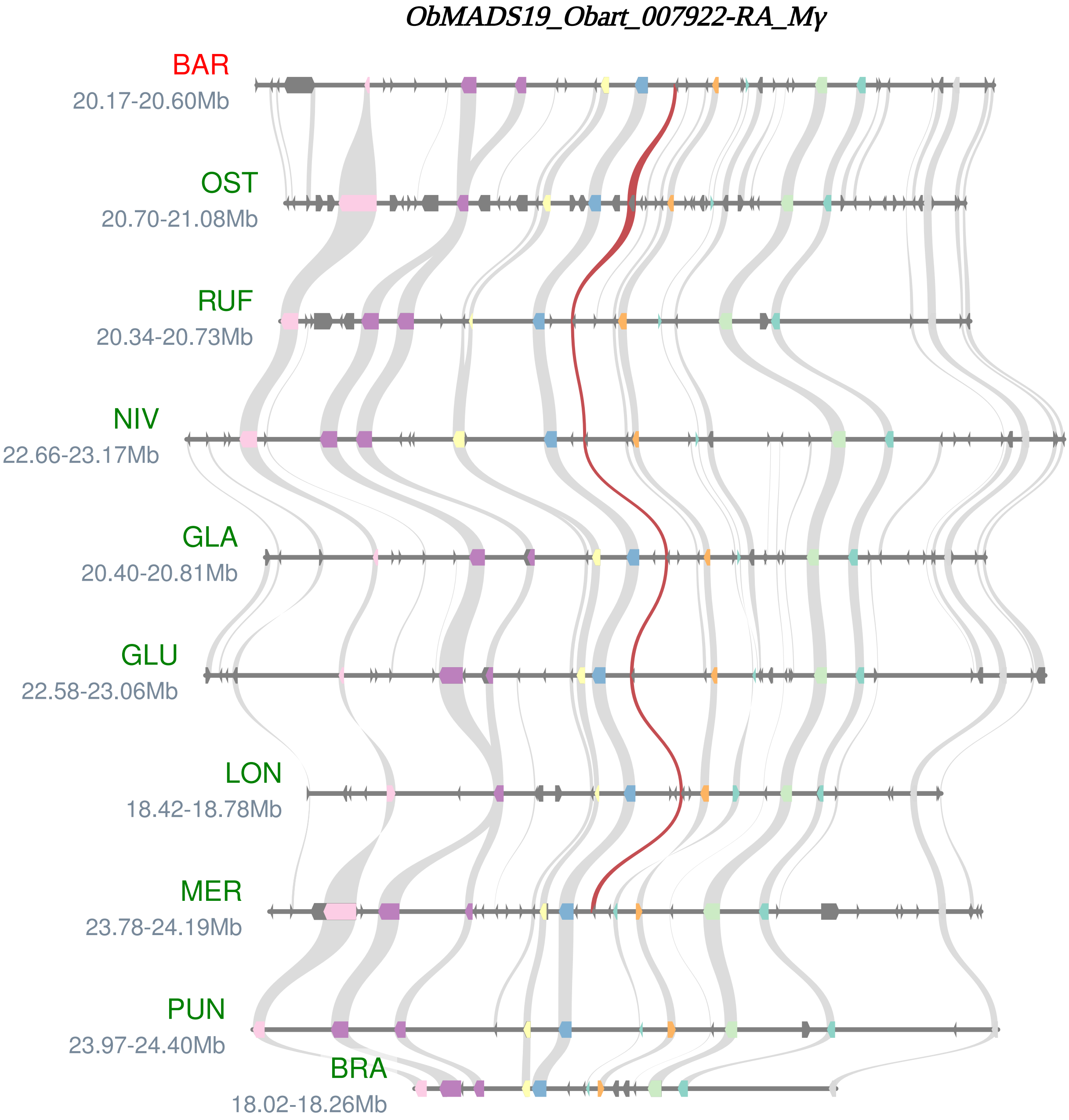

ObMADS19_Obart_007922-RA_Mγ
BAR
20.17-20.60Mb
OST
20.70-21.08Mb
RUF
20.34-20.73Mb
NIV
22.66-23.17Mb
GLA
20.40-20.81Mb
GLU
22.58-23.06Mb
LON
18.42-18.78Mb
MER
23.78-24.19Mb
PUN
23.97-24.40Mb
BRA
18.02-18.26Mb

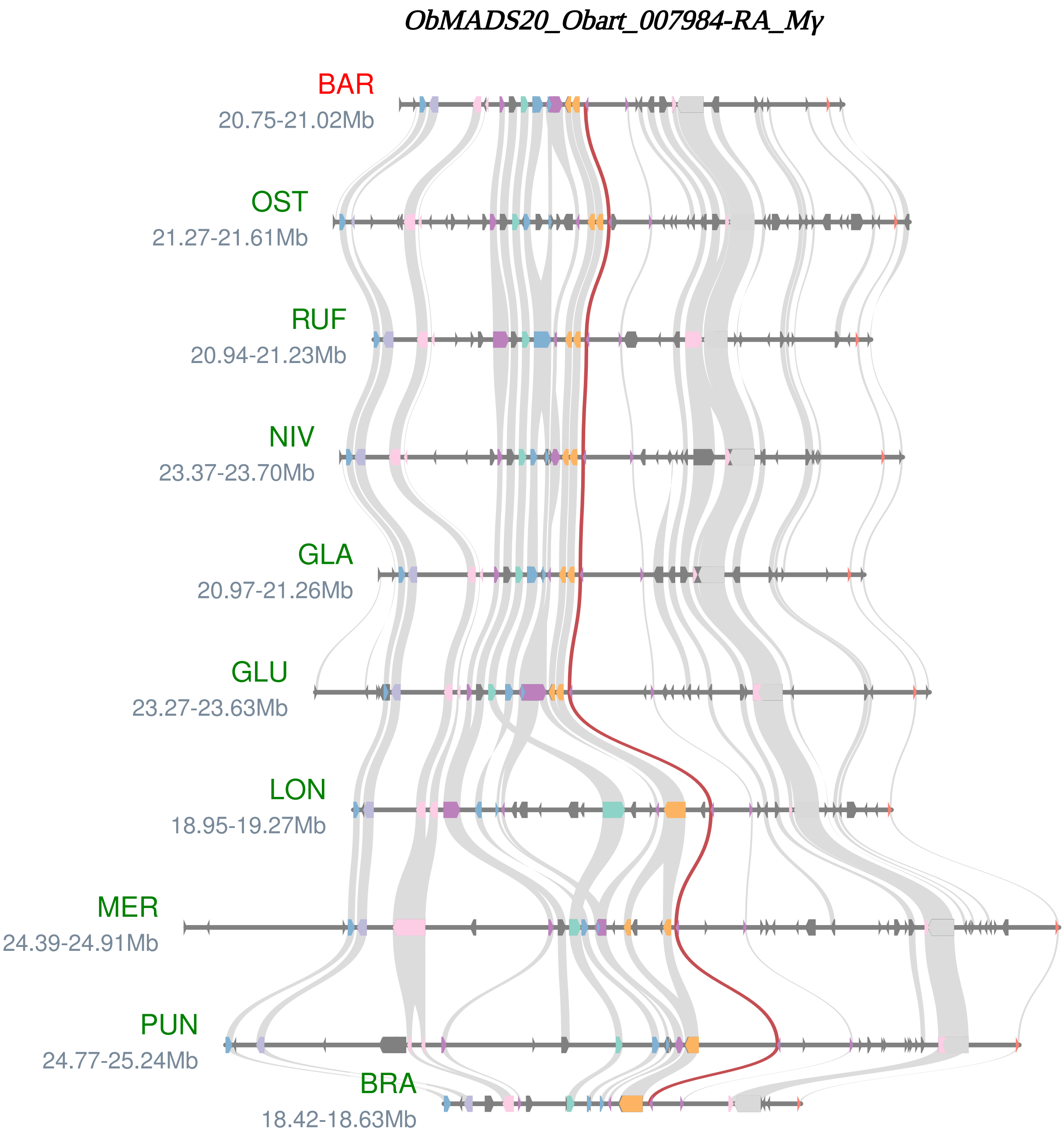

ObMADS20_Obart_007984-RA_Mγ
BAR
20.75-21.02Mb
OST
21.27-21.61Mb
RUF
20.94-21.23Mb
NIV
23.37-23.70Mb
GLA
20.97-21.26Mb
GLU
23.27-23.63Mb
LON
18.95-19.27Mb
MER
24.39-24.91Mb
PUN
24.77-25.24Mb
BRA
18.42-18.63Mb

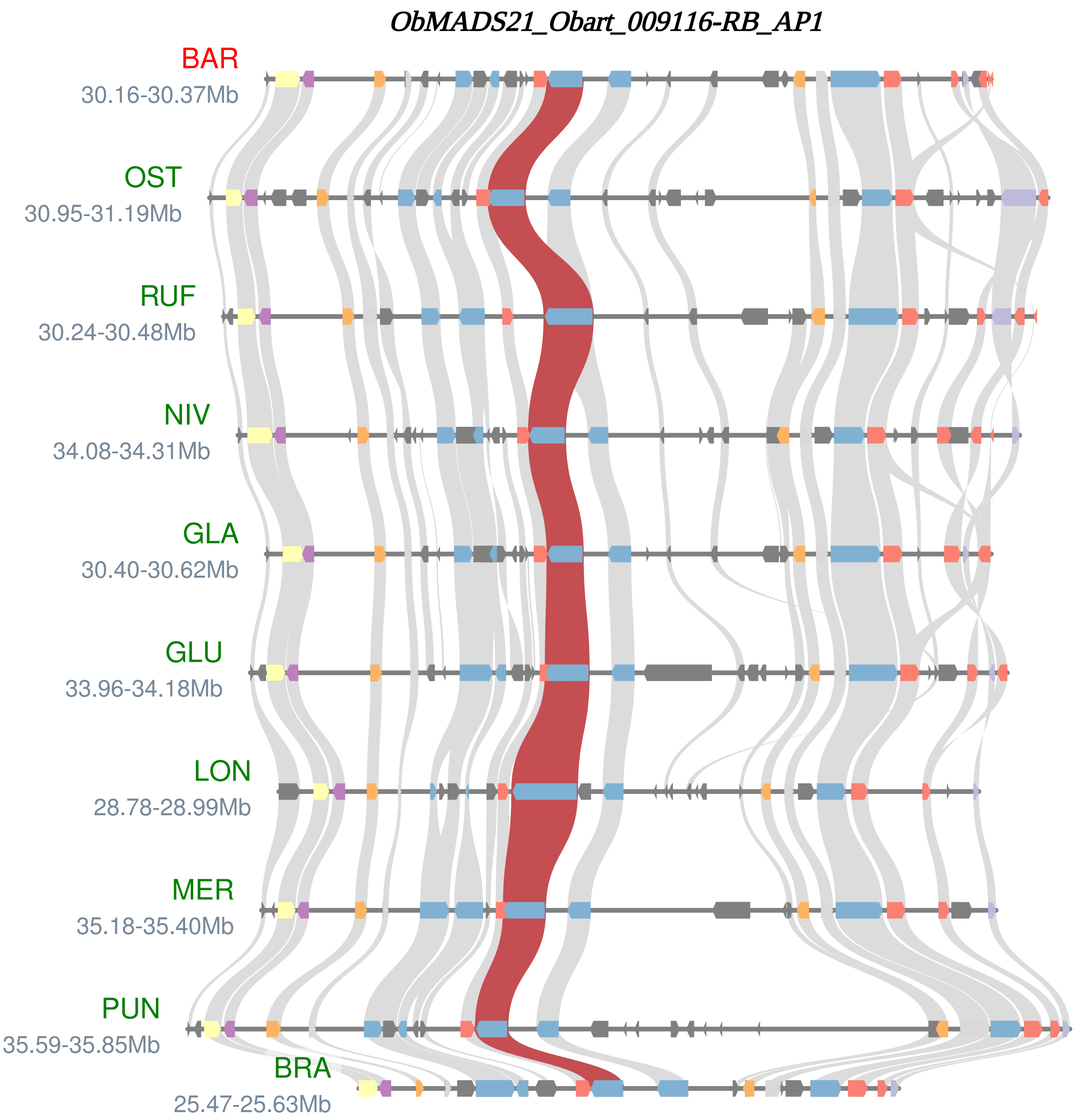
ObMADS21_Obart_009116-RB_AP1
BAR
30.16-30.37Mb
OST
30.95-31.19Mb
RUF
30.24-30.48Mb
NIV
34.08-34.31Mb
GLA
30.40-30.62Mb
GLU
33.96-34.18Mb
LON
28.78-28.99Mb
MER
35.18-35.40Mb
PUN
35.59-35.85Mb
BRA
25.47-25.63Mb

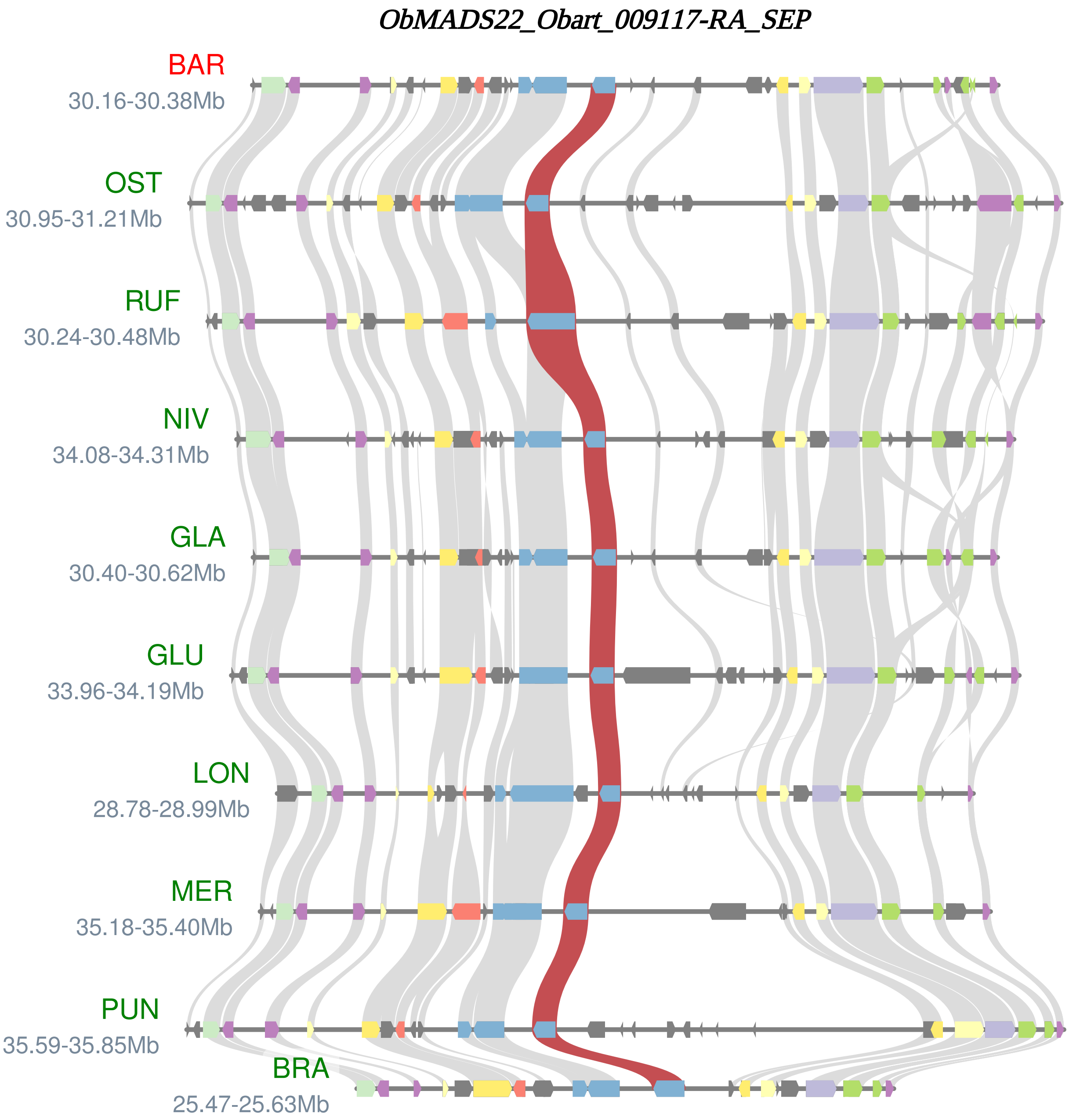
ObMADS22_Obart_009117-RA_SEP
BAR
30.16-30.38Mb
OST
30.95-31.21Mb
RUF
30.24-30.48Mb
NIV
34.08-34.31Mb
GLA
30.40-30.62Mb
GLU
33.96-34.19Mb
LON
28.78-28.99Mb
MER
35.18-35.40Mb
PUN
35.59-35.85Mb
BRA
25.47-25.63Mb

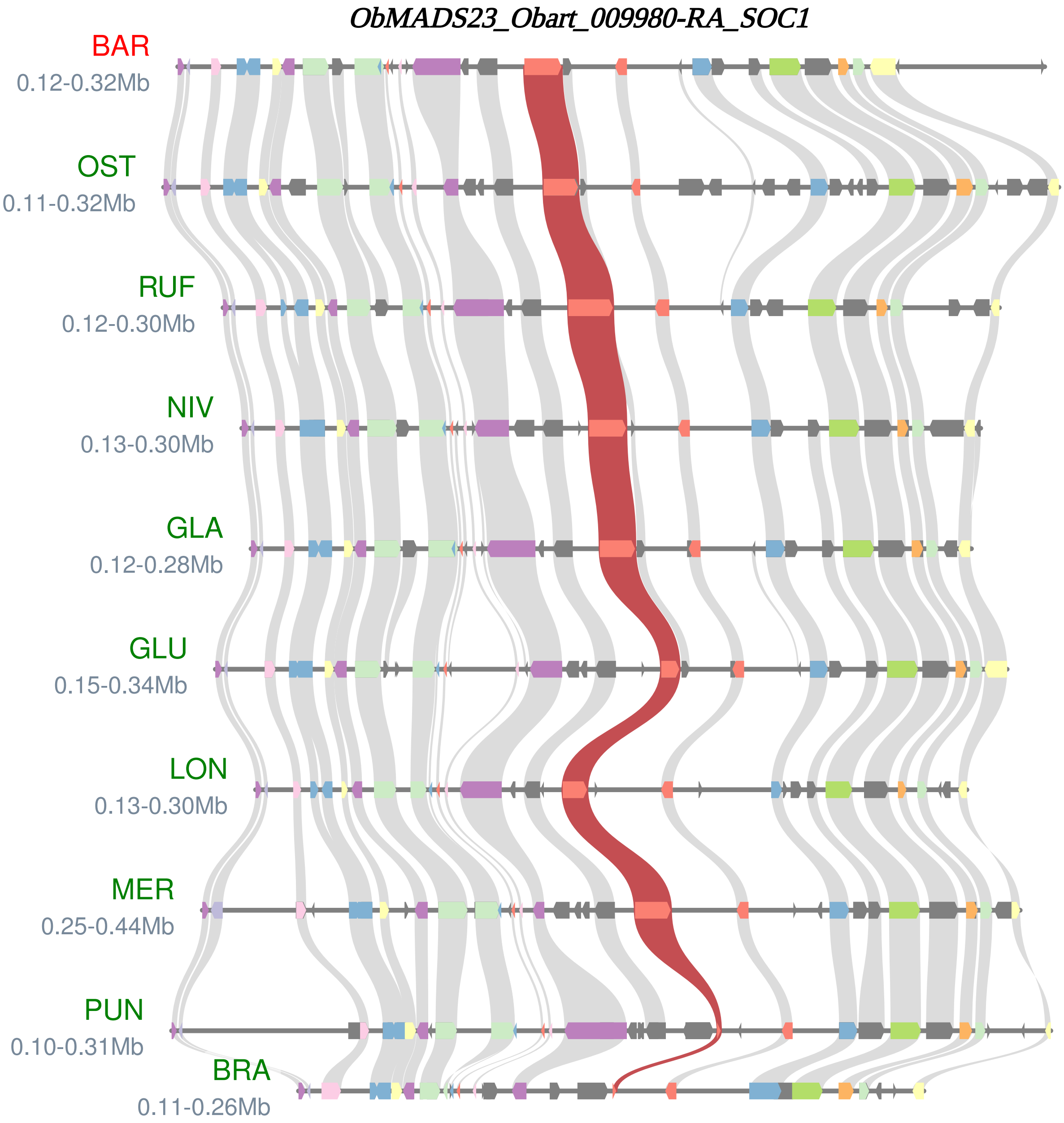

ObMADS23_Obart_009980-RA_SOC1
BAR
0.12-0.32Mb
OST
0.11-0.32Mb
RUF
0.12-0.30Mb
NIV
0.13-0.30Mb
GLA
0.12-0.28Mb
GLU
0.15-0.34Mb
LON
0.13-0.30Mb
MER
0.25-0.44Mb
PUN
0.10-0.31Mb
BRA
0.11-0.26Mb

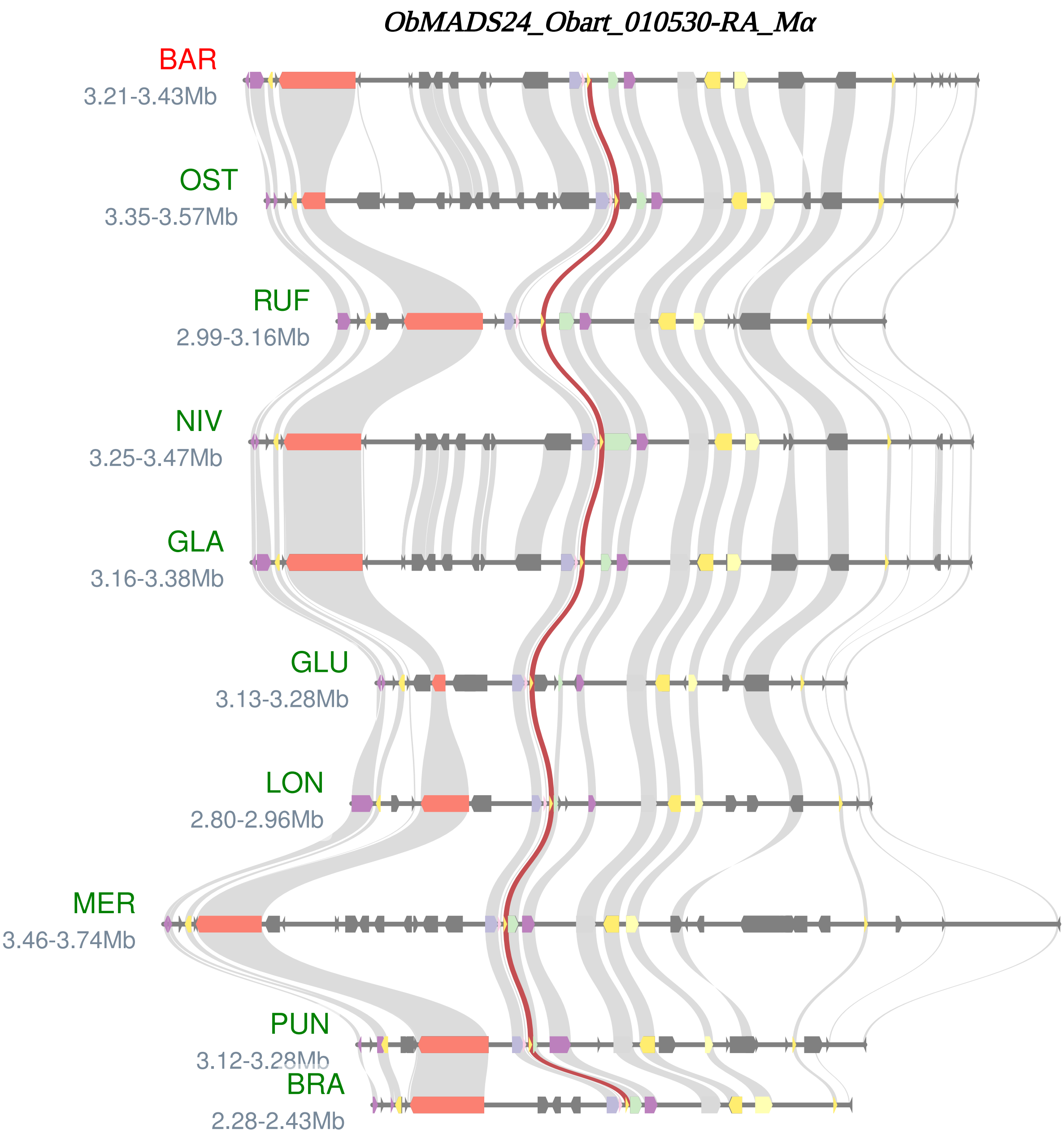
ObMADS24_Obart_010530-RA_Mα
BAR
3.21-3.43Mb
OST
3.35-3.57Mb
RUF
2.99-3.16Mb
NIV
3.25-3.47Mb
GLA
3.16-3.38Mb
GLU
3.13-3.28Mb
LON
2.80-2.96Mb
MER
3.46-3.74Mb
PUN
3.12-3.28Mb
BRA
2.28-2.43Mb

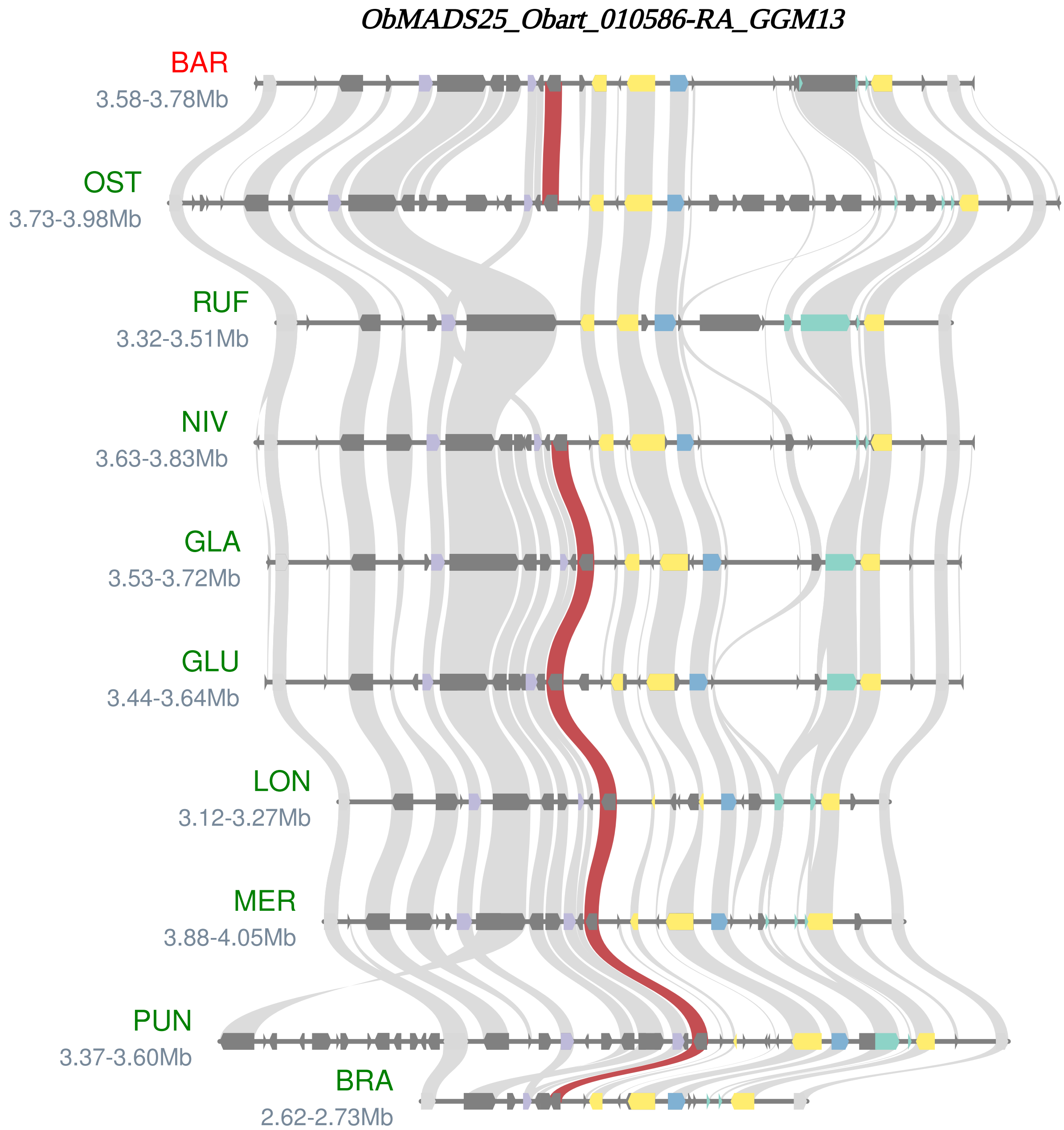
ObMADS25_Obart_010586-RA_GGM13
BAR
3.58-3.78Mb
OST
3.73-3.98Mb
RUF
3.32-3.51Mb
NIV
3.63-3.83Mb
GLA
3.53-3.72Mb
GLU
3.44-3.64Mb
LON
3.12-3.27Mb
MER
3.88-4.05Mb
PUN
3.37-3.60Mb
BRA
2.62-2.73Mb

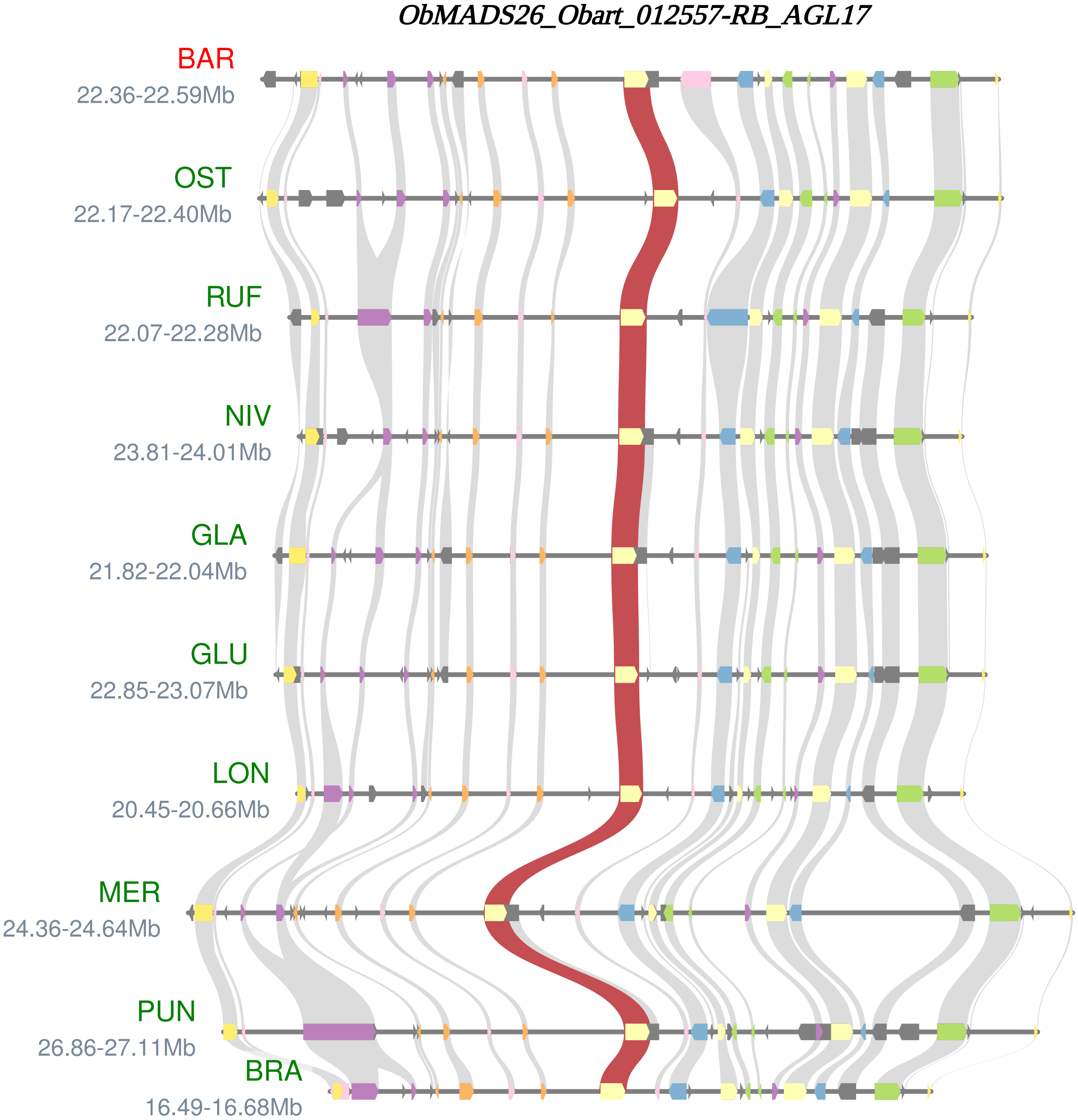

ObMADS26_Obart_012557-RB_AGL17
BAR
22.36-22.59Mb
OST
22.17-22.40Mb
RUF
22.07-22.28Mb
NIV
23.81-24.01Mb
GLA
21.82-22.04Mb
GLU
22.85-23.07Mb
LON
20.45-20.66Mb
MER
24.36-24.64Mb
PUN
26.86-27.11Mb
BRA
16.49-16.68Mb

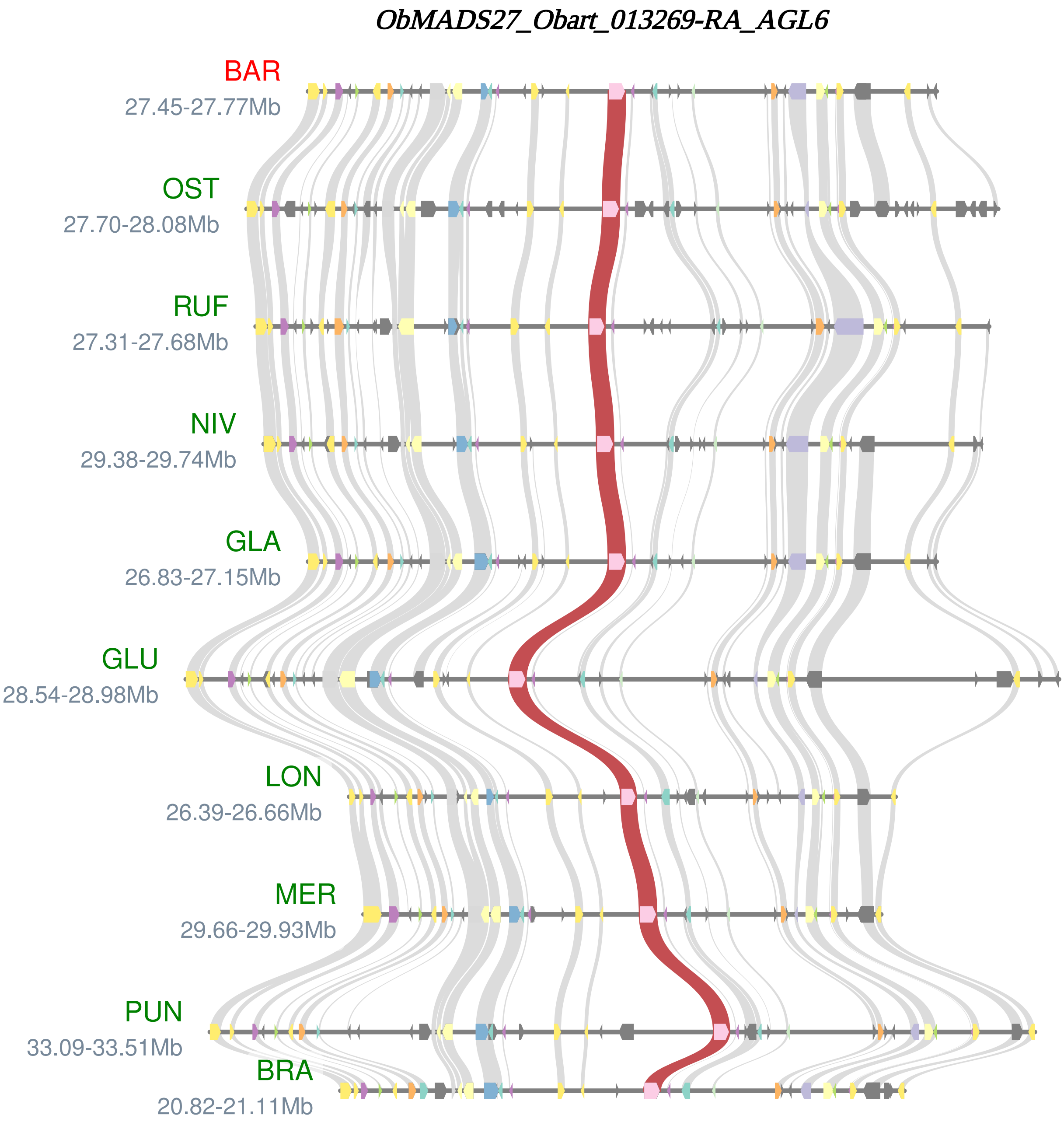
ObMADS27_Obart_013269-RA_AGL6
BAR
27.45-27.77Mb
OST
27.70-28.08Mb
RUF
27.31-27.68Mb
NIV
29.38-29.74Mb
GLA
26.83-27.15Mb
GLU
28.54-28.98Mb
LON
26.39-26.66Mb
MER
29.66-29.93Mb
PUN
33.09-33.51Mb
BRA
20.82-21.11Mb

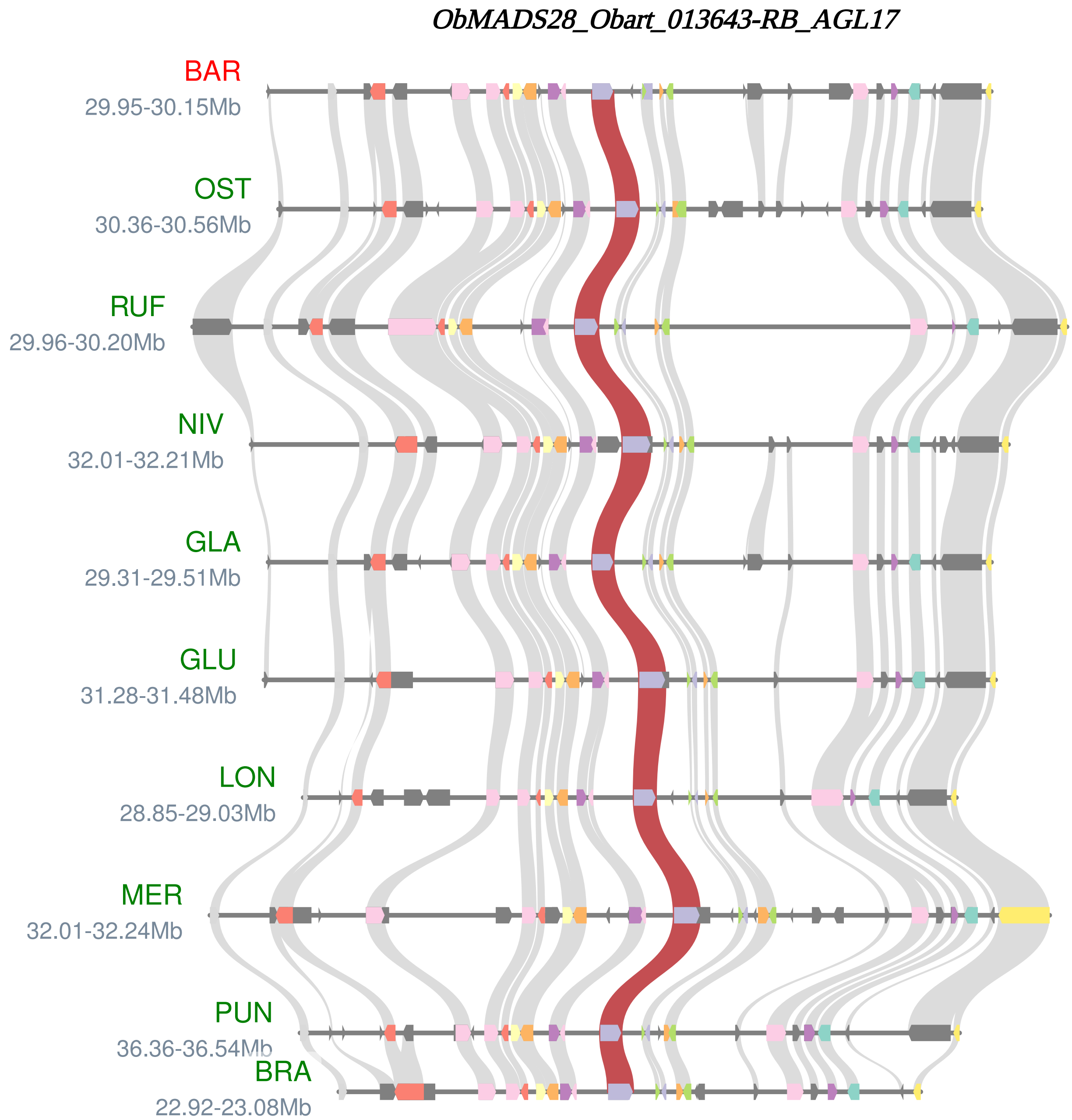

ObMADS28_Obart_013643-RB_AGL17
BAR
29.95-30.15Mb
OST
30.36-30.56Mb
RUF
29.96-30.20Mb
NIV
32.01-32.21Mb
GLA
29.31-29.51Mb
GLU
31.28-31.48Mb
LON
28.85-29.03Mb
MER
32.01-32.24Mb
PUN
36.36-36.54Mb
BRA
22.92-23.08Mb

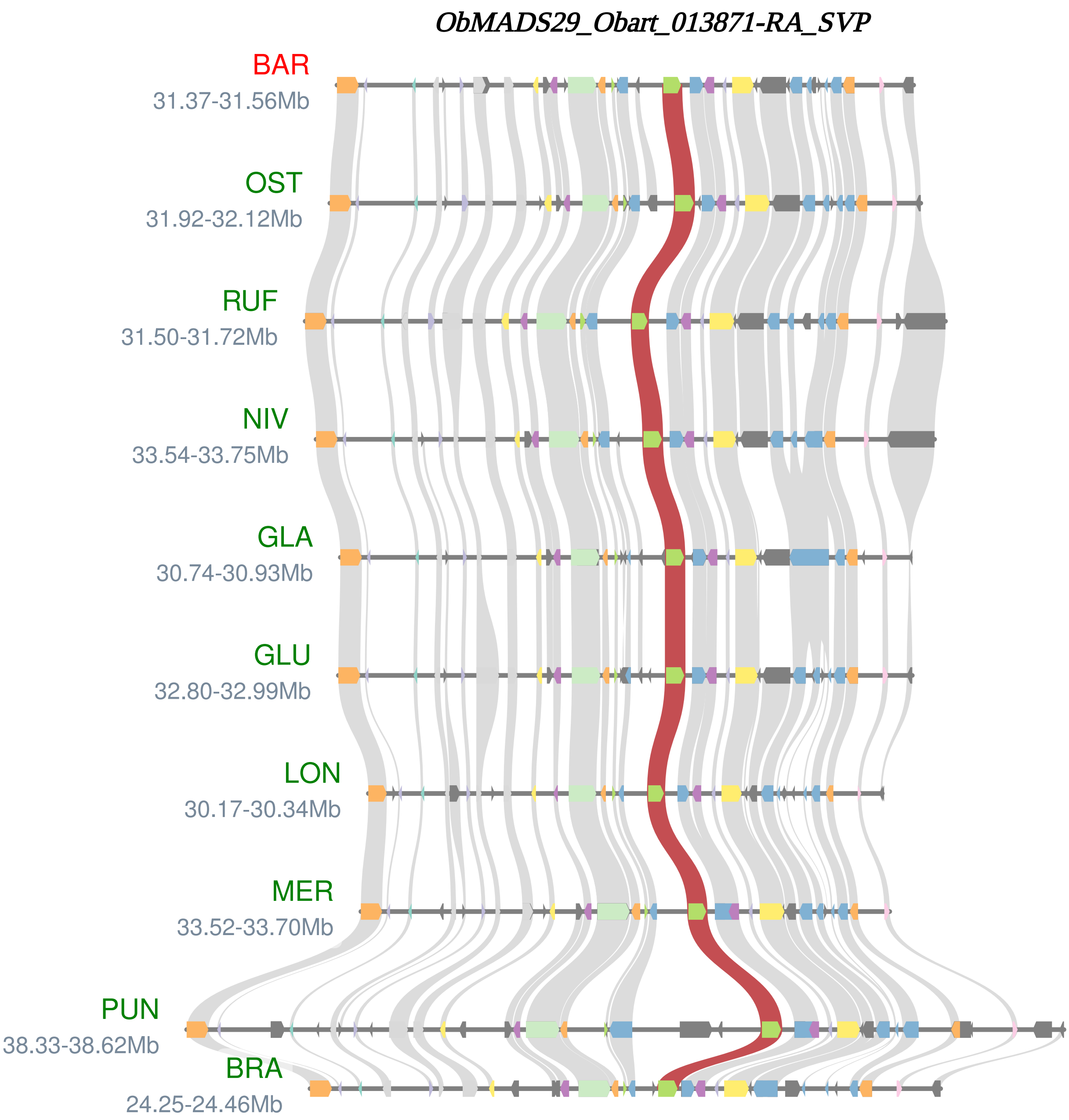

ObMADS29_Obart_013871-RA_SVP
BAR
31.37-31.56Mb
OST
31.92-32.12Mb
RUF
31.50-31.72Mb
NIV
33.54-33.75Mb
GLA
30.74-30.93Mb
GLU
32.80-32.99Mb
LON
30.17-30.34Mb
MER
33.52-33.70Mb
PUN
38.33-38.62Mb
BRA
24.25-24.46Mb

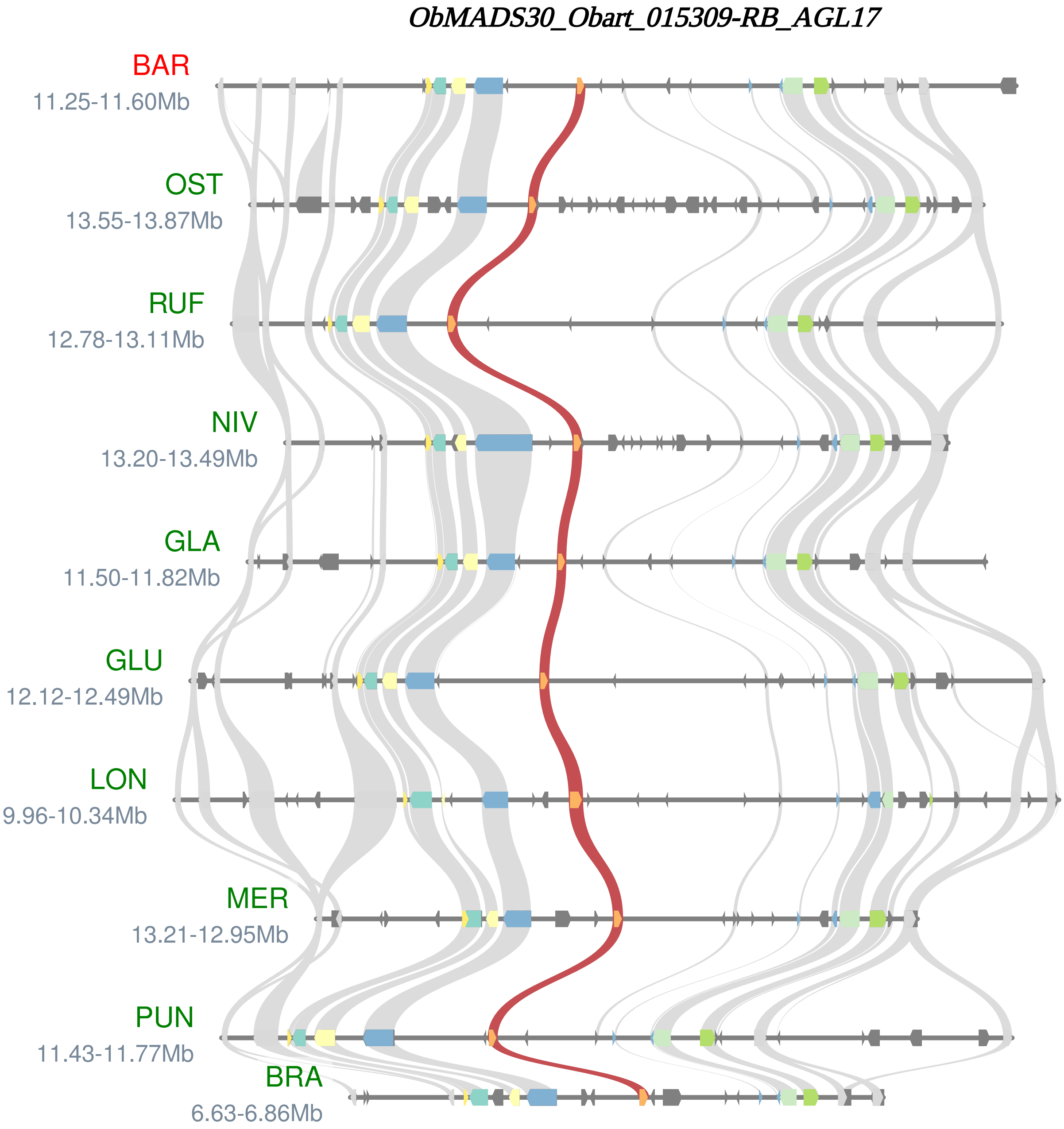

ObMADS30_Obart_015309-RB_AGL17
BAR
11.25-11.60Mb
OST
13.55-13.87Mb
RUF
12.78-13.11Mb
NIV
13.20-13.49Mb
GLA
11.50-11.82Mb
GLU
12.12-12.49Mb
LON
9.96-10.34Mb
MER
13.21-12.95Mb
PUN
11.43-11.77Mb
BRA
6.63-6.86Mb

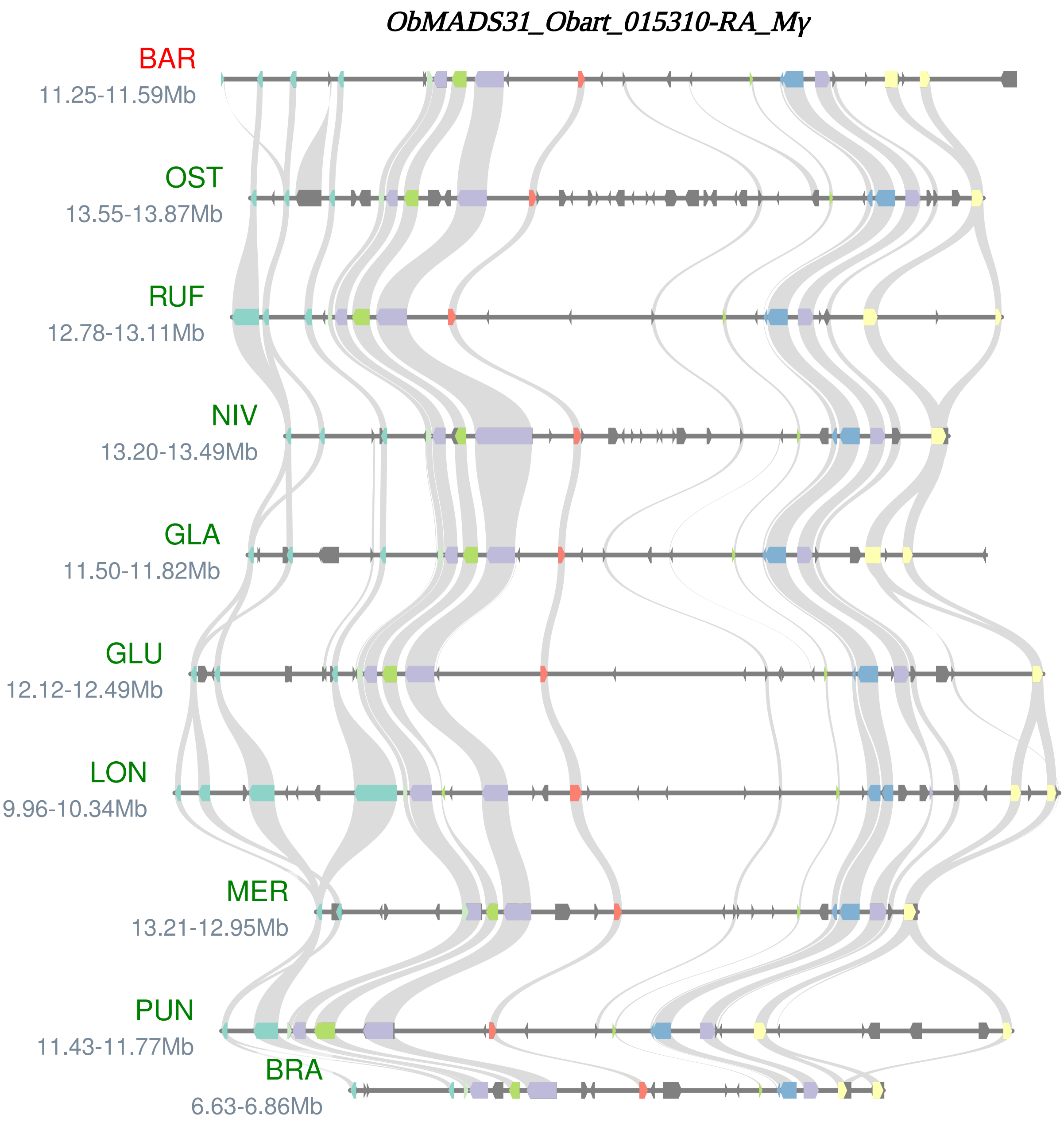

ObMADS31_Obart_015310-RA_Mγ
BAR
11.25-11.59Mb
OST
13.55-13.87Mb
RUF
12.78-13.11Mb
NIV
13.20-13.49Mb
GLA
11.50-11.82Mb
GLU
12.12-12.49Mb
LON
9.96-10.34Mb
MER
13.21-12.95Mb
PUN
11.43-11.77Mb
BRA
6.63-6.86Mb

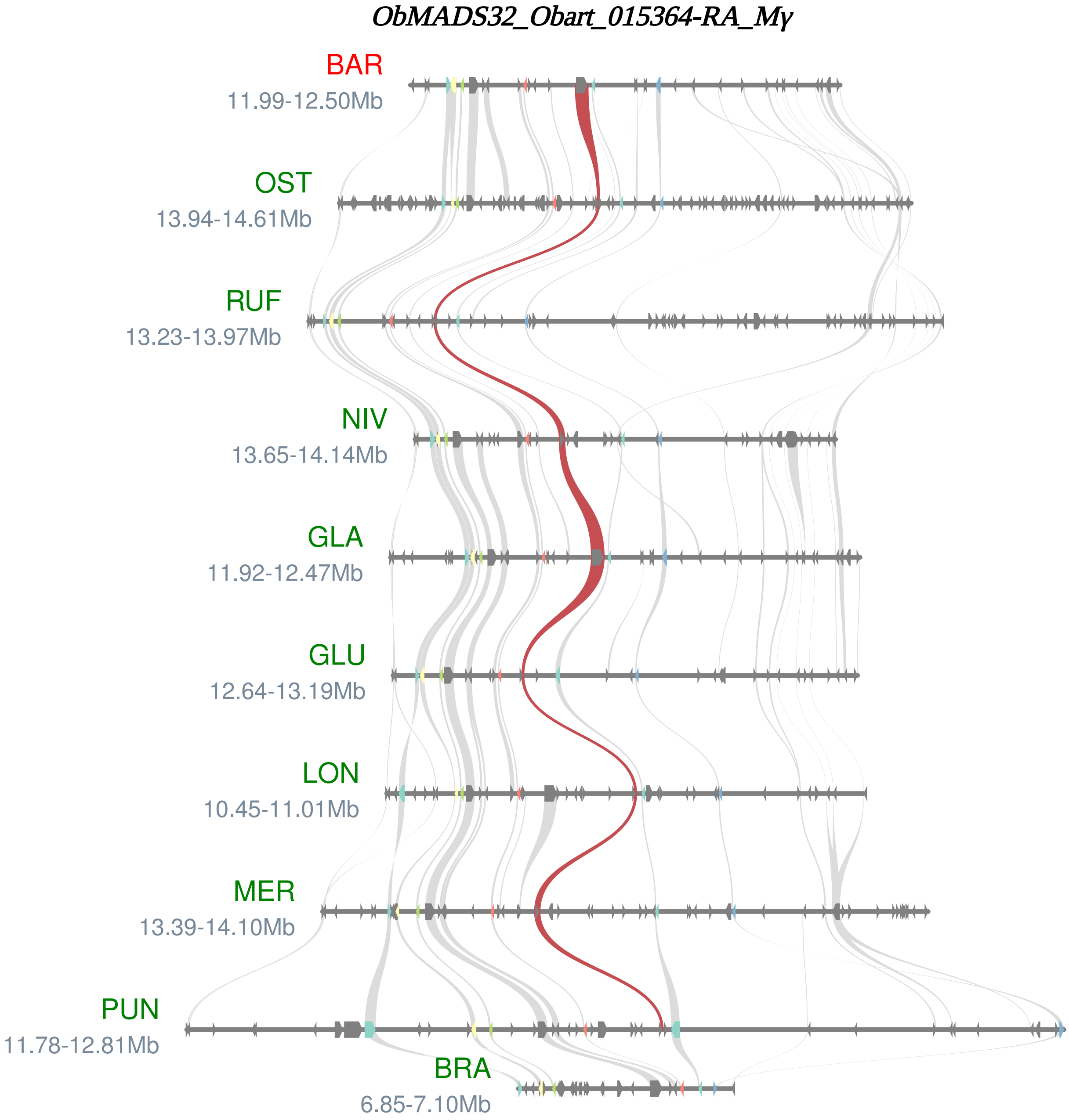
ObMADS32_Obart_015364-RA_Mγ
BAR
11.99-12.50Mb
OST
13.94-14.61Mb
RUF
13.23-13.97Mb
NIV
13.65-14.14Mb
GLA
11.92-12.47Mb
GLU
12.64-13.19Mb
LON
10.45-11.01Mb
MER
13.39-14.10Mb
PUN
11.78-12.81Mb
BRA
6.85-7.10Mb

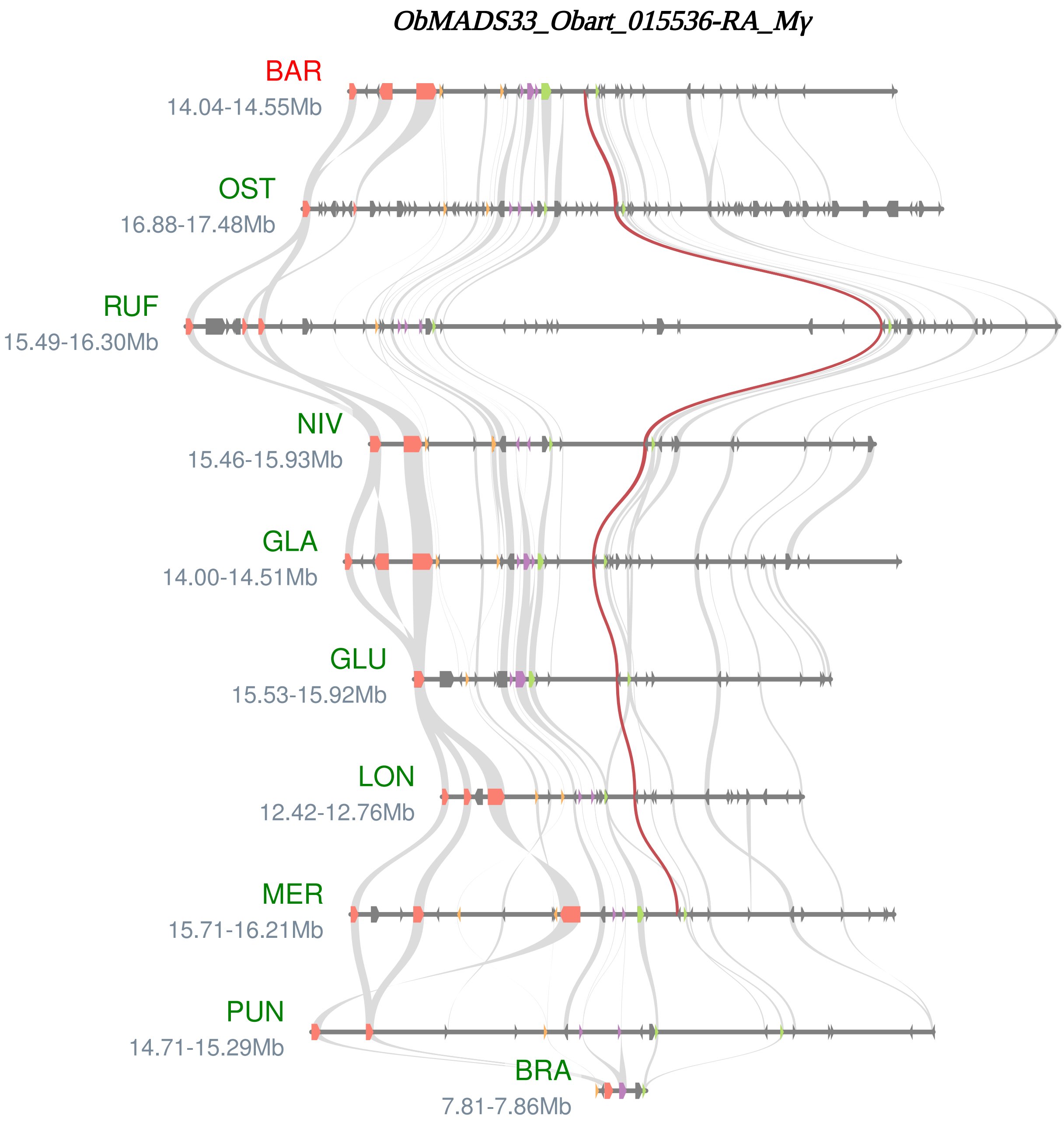

ObMADS33_Obart_015536-RA_Mγ
BAR
14.04-14.55Mb
OST
16.88-17.48Mb
RUF
15.49-16.30Mb
NIV
15.46-15.93Mb
GLA
14.00-14.51Mb
GLU
15.53-15.92Mb
LON
12.42-12.76Mb
MER
15.71-16.21Mb
PUN
14.71-15.29Mb
BRA
7.81-7.86Mb

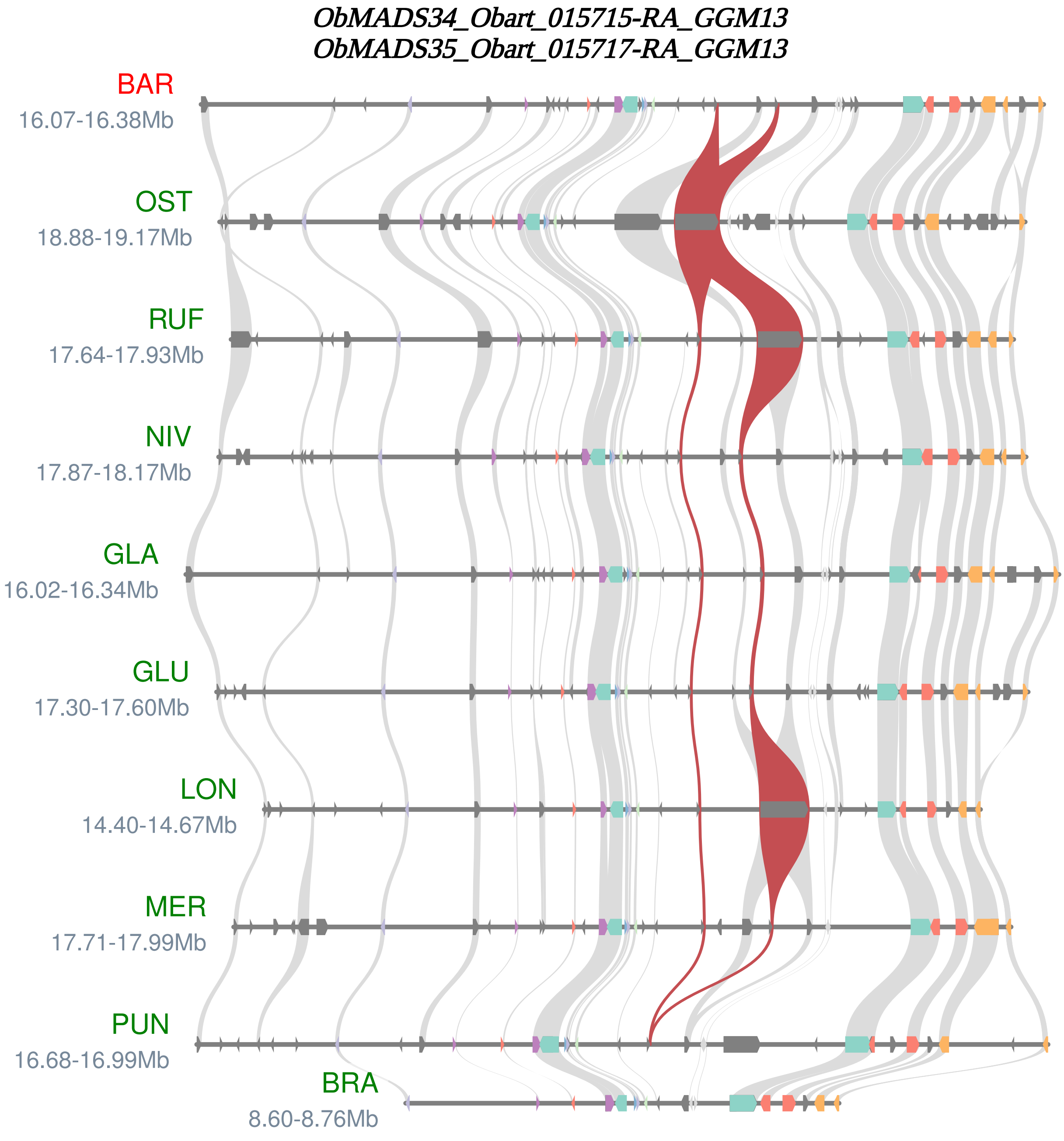
ObMADS34_Obart_015715-RA_GGM13
ObMADS35_Obart_015717-RA_GGM13
BAR
16.07-16.38Mb
OST
18.88-19.17Mb
RUF
17.64-17.93Mb
NIV
17.87-18.17Mb
GLA
16.02-16.34Mb
GLU
17.30-17.60Mb
LON
14.40-14.67Mb
MER
17.71-17.99Mb
PUN
16.68-16.99Mb
BRA
8.60-8.76Mb

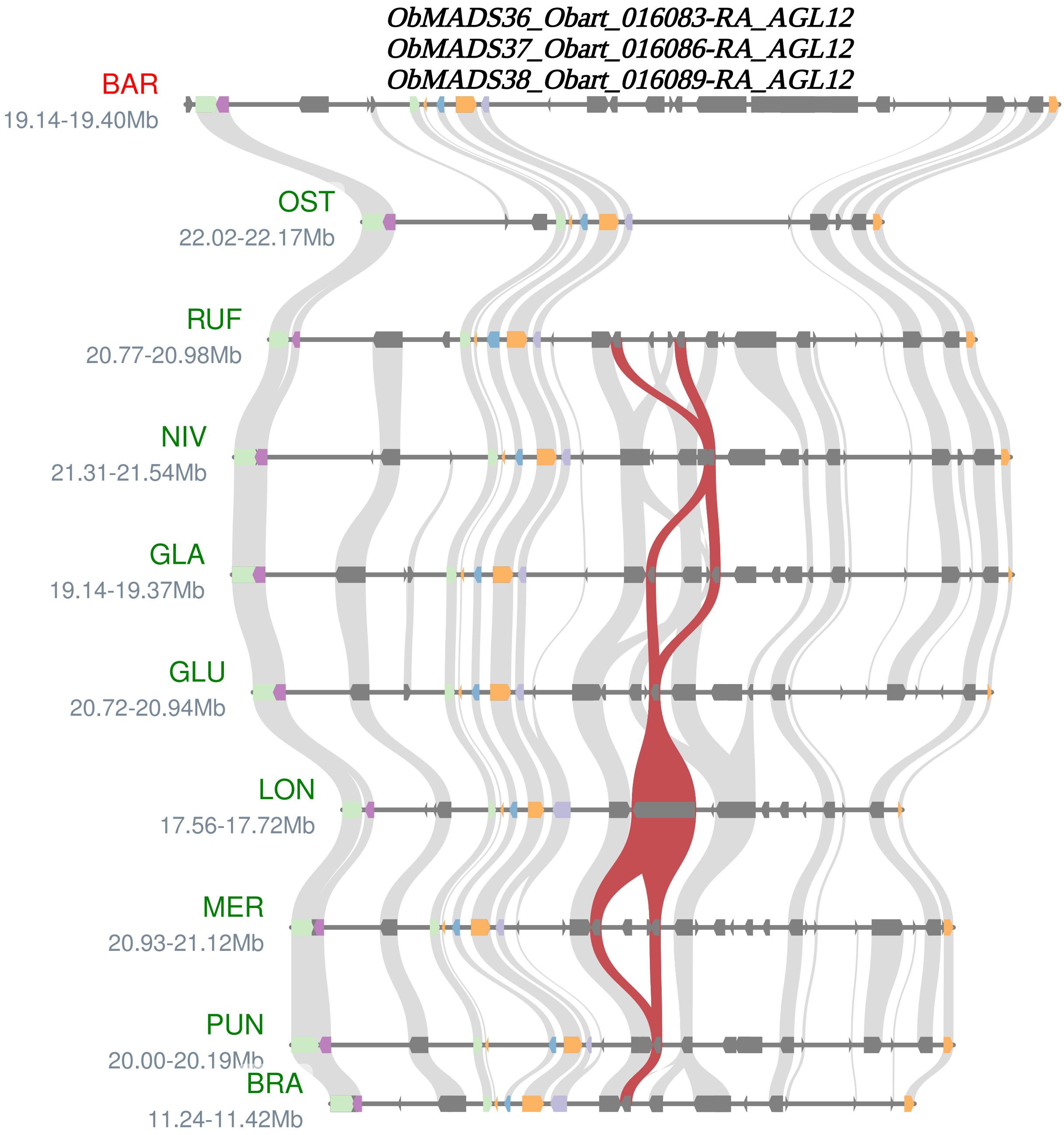

ObMADS36_Obart_016083-RA_AGL12
ObMADS37_Obart_016086-RA_AGL12
ObMADS38_Obart_016089-RA_AGL12
BAR
19.14-19.40Mb
OST
22.02-22.17Mb
RUF
20.77-20.98Mb
NIV
21.31-21.54Mb
GLA
19.14-19.37Mb
GLU
20.72-20.94Mb
LON
17.56-17.72Mb
MER
20.93-21.12Mb
PUN
20.00-20.19Mb
BRA
11.24-11.42Mb

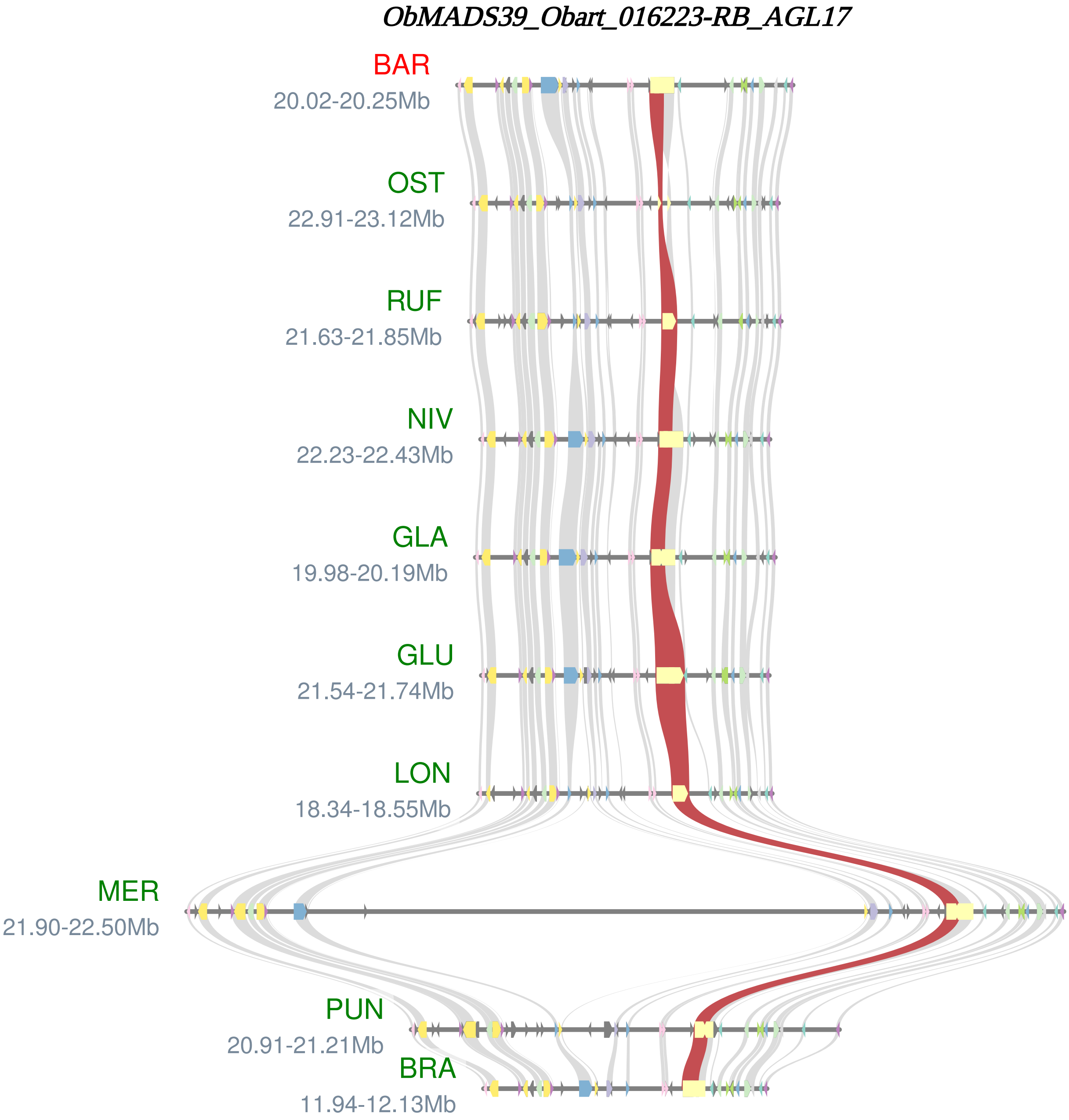
ObMADS39_Obart_016223-RB_AGL17
BAR
20.02-20.25Mb
OST
22.91-23.12Mb
RUF
21.63-21.85Mb
NIV
22.23-22.43Mb
GLA
19.98-20.19Mb
GLU
21.54-21.74Mb
LON
18.34-18.55Mb
MER
21.90-22.50Mb
PUN
20.91-21.21Mb
BRA
11.94-12.13Mb

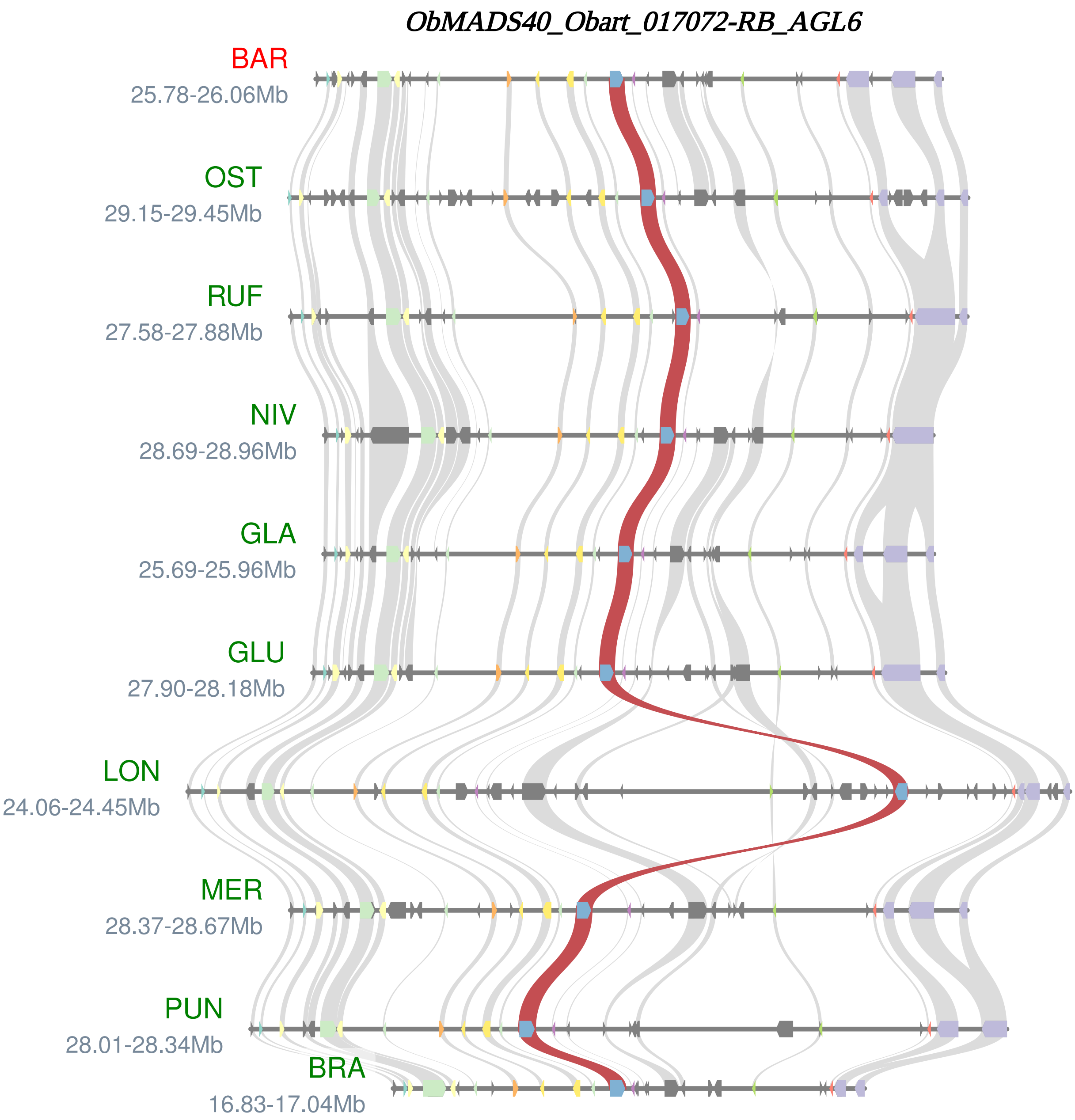

ObMADS40_Obart_017072-RB_AGL6
BAR
25.78-26.06Mb
OST
29.15-29.45Mb
RUF
27.58-27.88Mb
NIV
28.69-28.96Mb
GLA
25.69-25.96Mb
GLU
27.90-28.18Mb
LON
24.06-24.45Mb
MER
28.37-28.67Mb
PUN
28.01-28.34Mb
BRA
16.83-17.04Mb

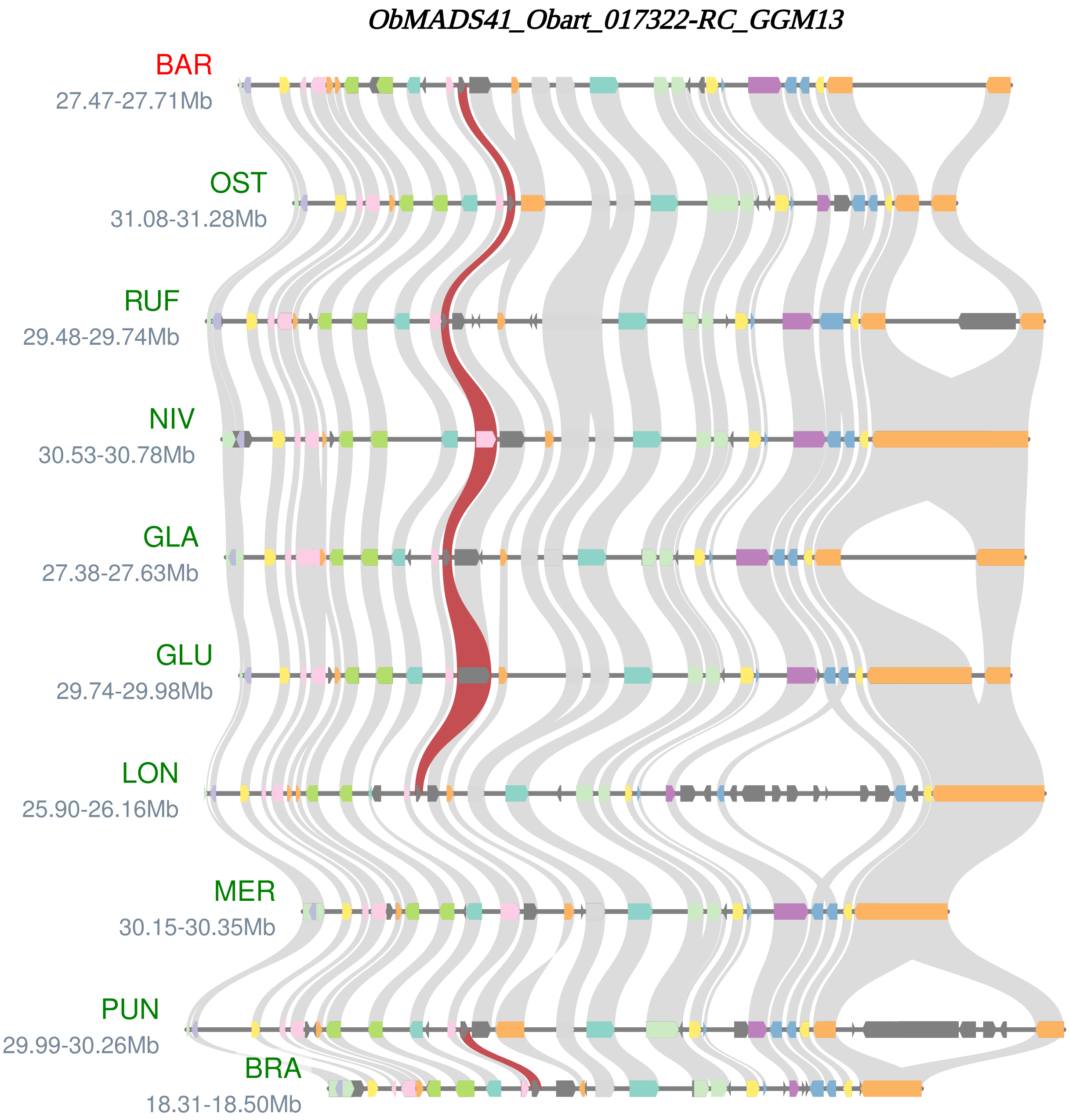
ObMADS41_Obart_017322-RC_GGM13
BAR
27.47-27.71Mb
OST
31.08-31.28Mb
RUF
29.48-29.74Mb
NIV
30.53-30.78Mb
GLA
27.38-27.63Mb
GLU
29.74-29.98Mb
LON
25.90-26.16Mb
MER
30.15-30.35Mb
PUN
29.99-30.26Mb
BRA
18.31-18.50Mb

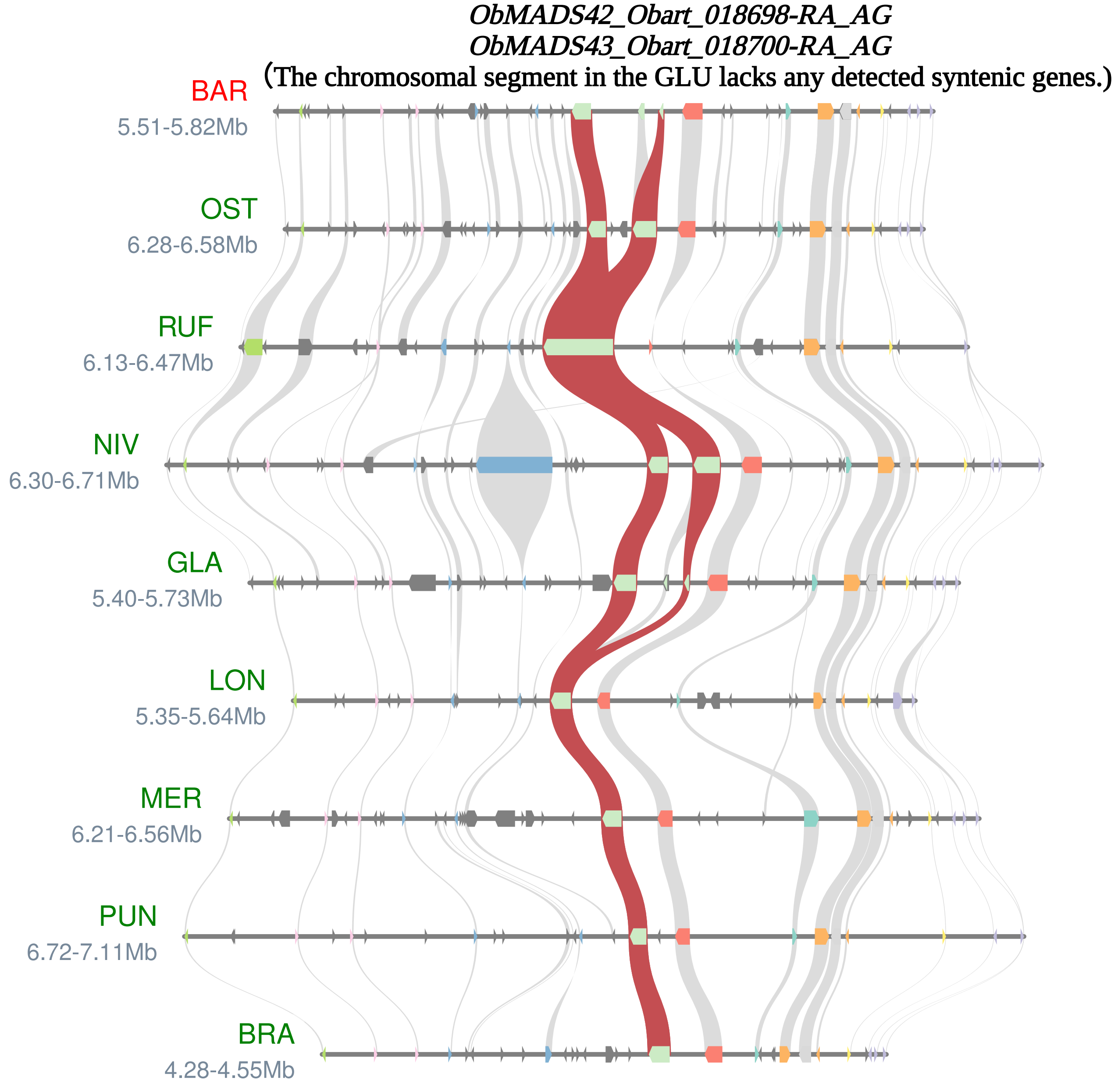
ObMADS42_Obart_018698-RA_AG
ObMADS43_Obart_018700-RA_AG
(The chromosomal segment in the GLU lacks any detected syntenic genes.)
BAR
5.51-5.82Mb
OST
6.28-6.58Mb
RUF
6.13-6.47Mb
NIV
6.30-6.71Mb
GLA
5.40-5.73Mb
LON
5.35-5.64Mb
MER
6.21-6.56Mb
PUN
6.72-7.11Mb
BRA
4.28-4.55Mb

ObMADS44_Obart_019174-RA_Mα
(The chromosomal segment in the GLU&LON lacks any detected syntenic genes.)

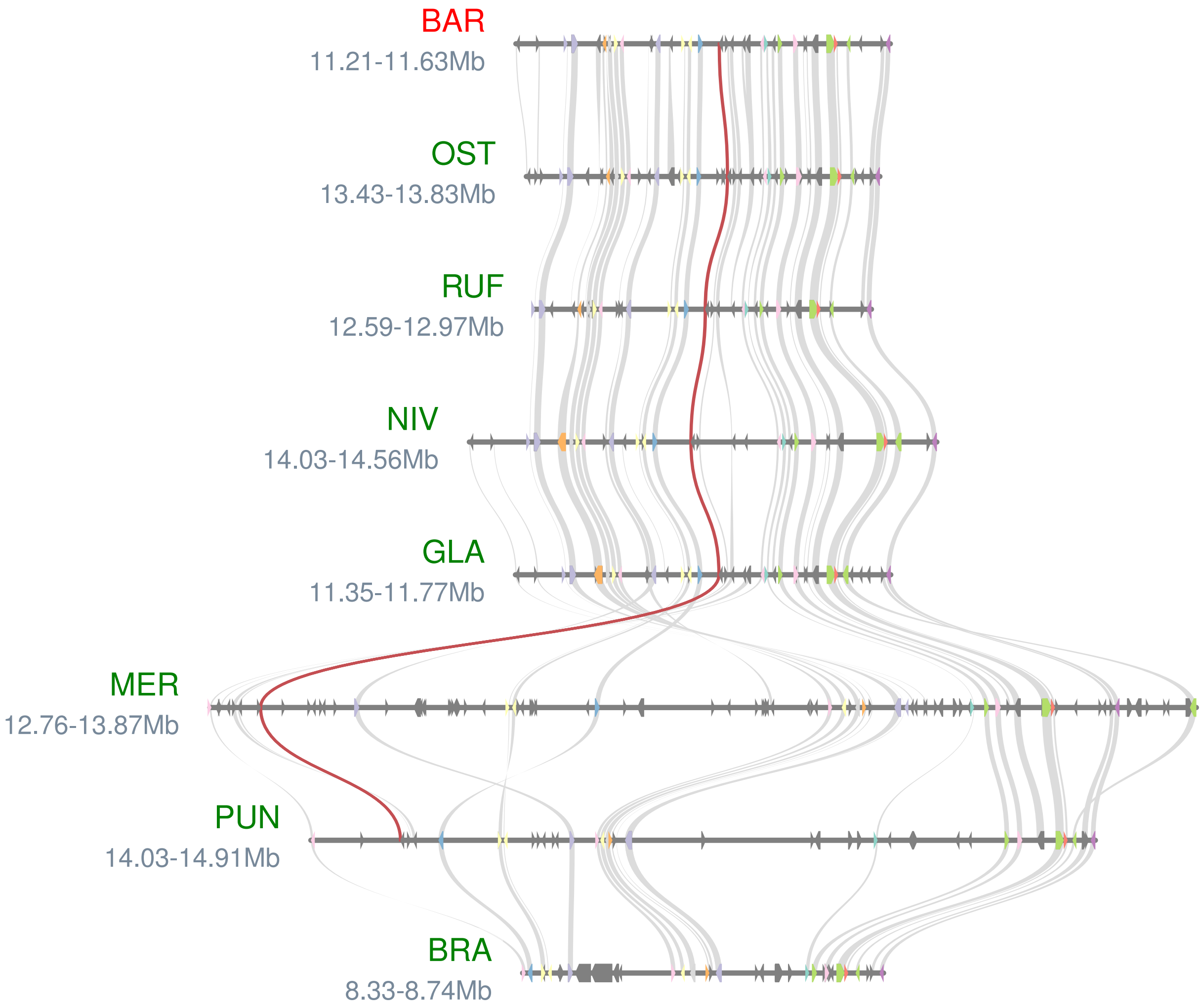

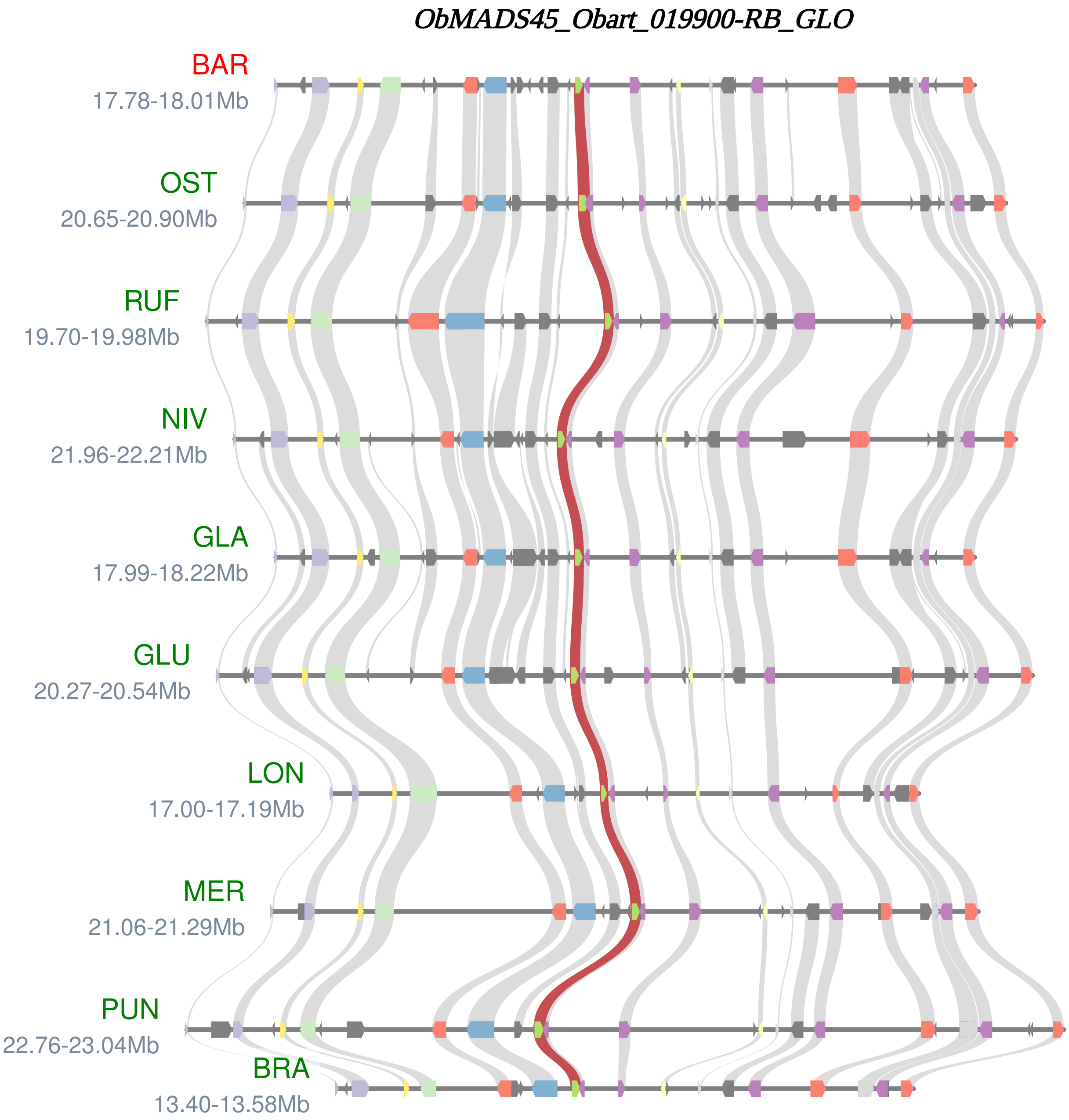

ObMADS45_Obart_019900-RB_GLO
BAR
17.78-18.01Mb
OST
20.65-20.90Mb
RUF
19.70-19.98Mb
NIV
21.96-22.21Mb
GLA
17.99-18.22Mb
GLU
20.27-20.54Mb
LON
17.00-17.19Mb
MER
21.06-21.29Mb
PUN
22.76-23.04Mb
BRA
13.40-13.58Mb

ObMADS46_Obart_021647-RA_SEP
(The chromosomal segment in the MER lacks any detected syntenic genes.)

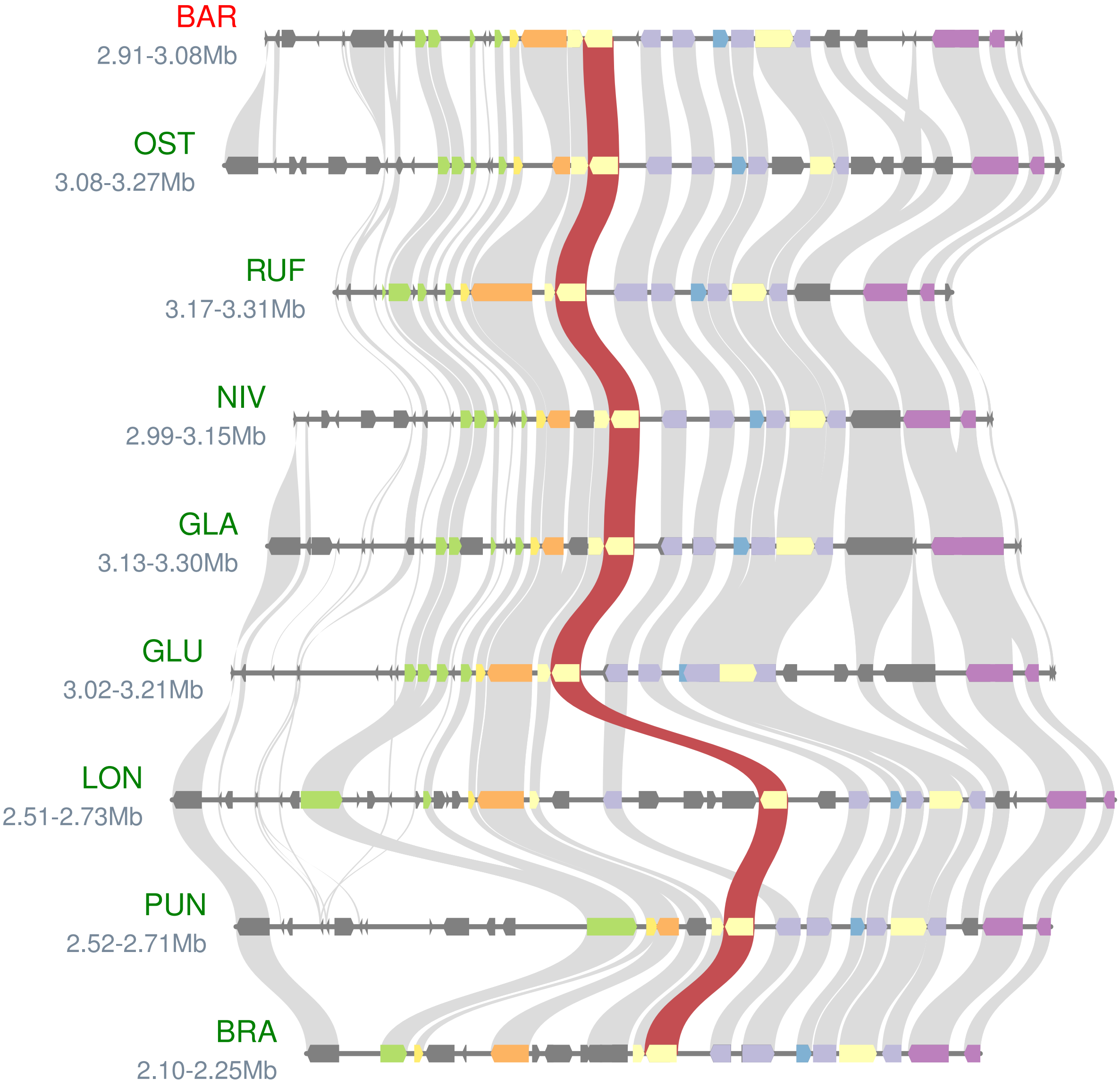

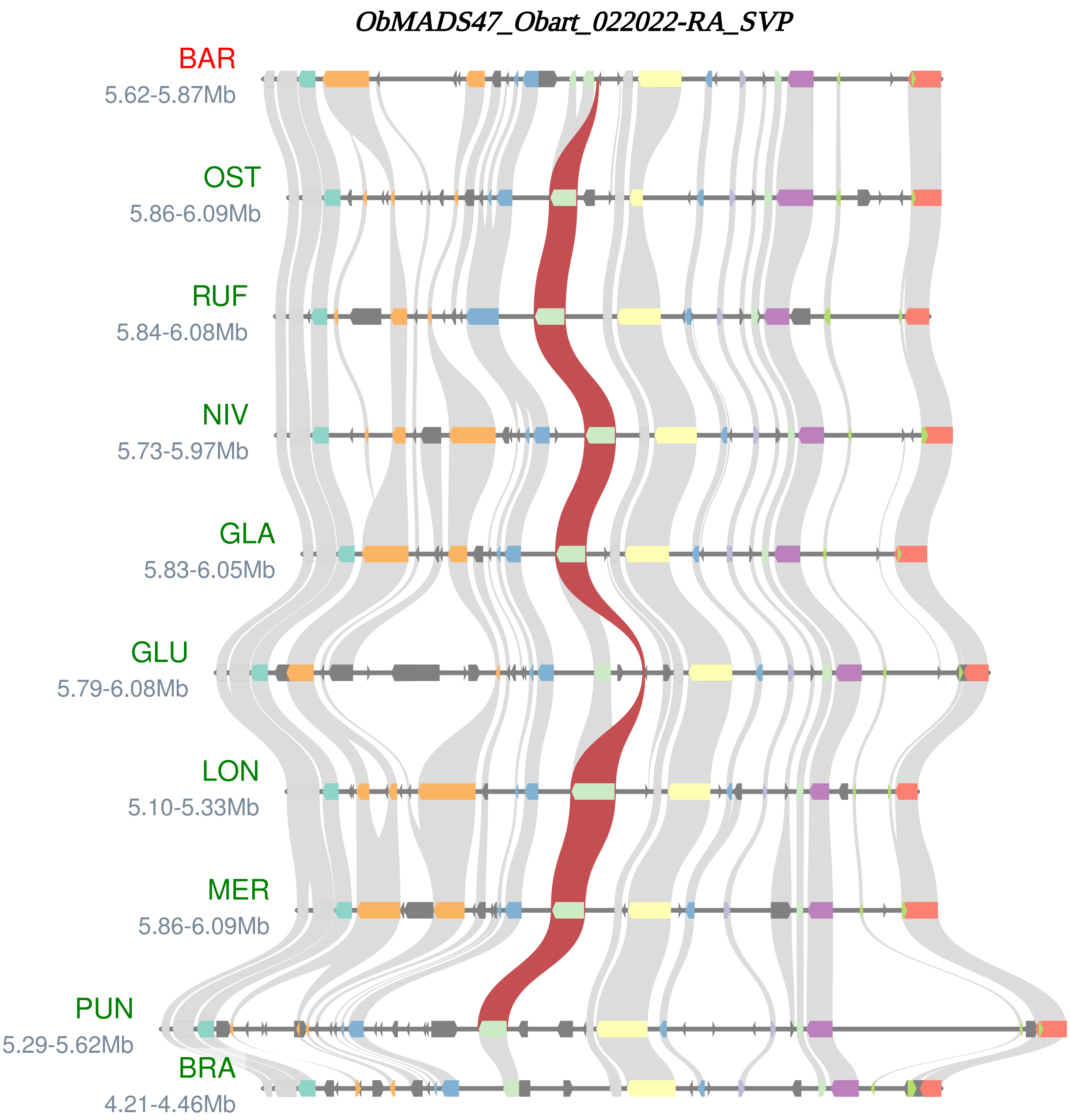
ObMADS47_Obart_022022-RA_SVP
BAR
5.62-5.87Mb
OST
5.86-6.09Mb
RUF
5.84-6.08Mb
NIV
5.73-5.97Mb
GLA
5.83-6.05Mb
GLU
5.79-6.08Mb
LON
5.10-5.33Mb
MER
5.86-6.09Mb
PUN
5.29-5.62Mb
BRA
4.21-4.46Mb

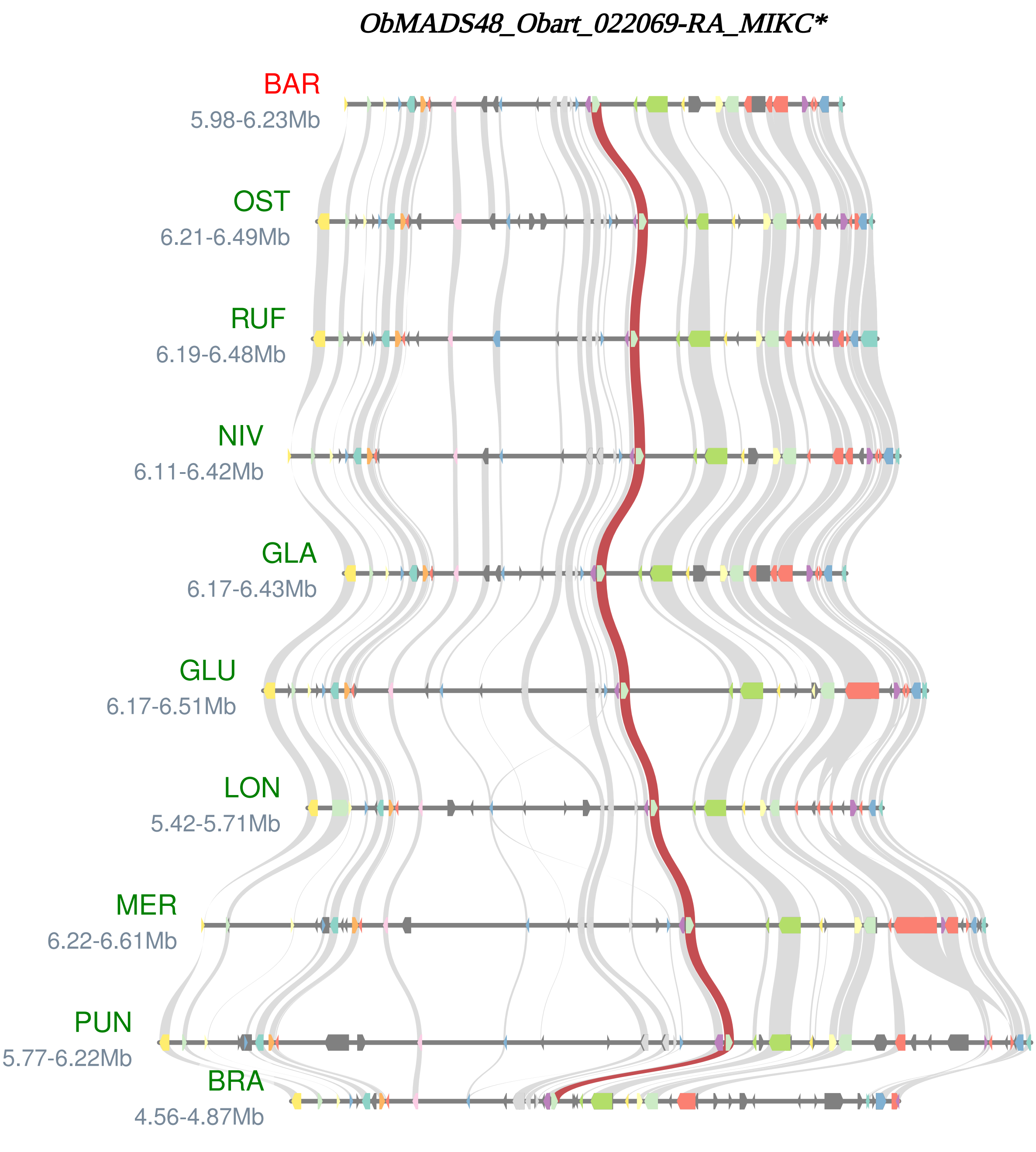
ObMADS48_Obart_022069-RA_MIKC*
BAR
5.98-6.23Mb
OST
6.21-6.49Mb
RUF
6.19-6.48Mb
NIV
6.11-6.42Mb
GLA
6.17-6.43Mb
GLU
6.17-6.51Mb
LON
5.42-5.71Mb
MER
6.22-6.61Mb
PUN
5.77-6.22Mb
BRA
4.56-4.87Mb

ObMADS49_Obart_022960-RA_AGL17

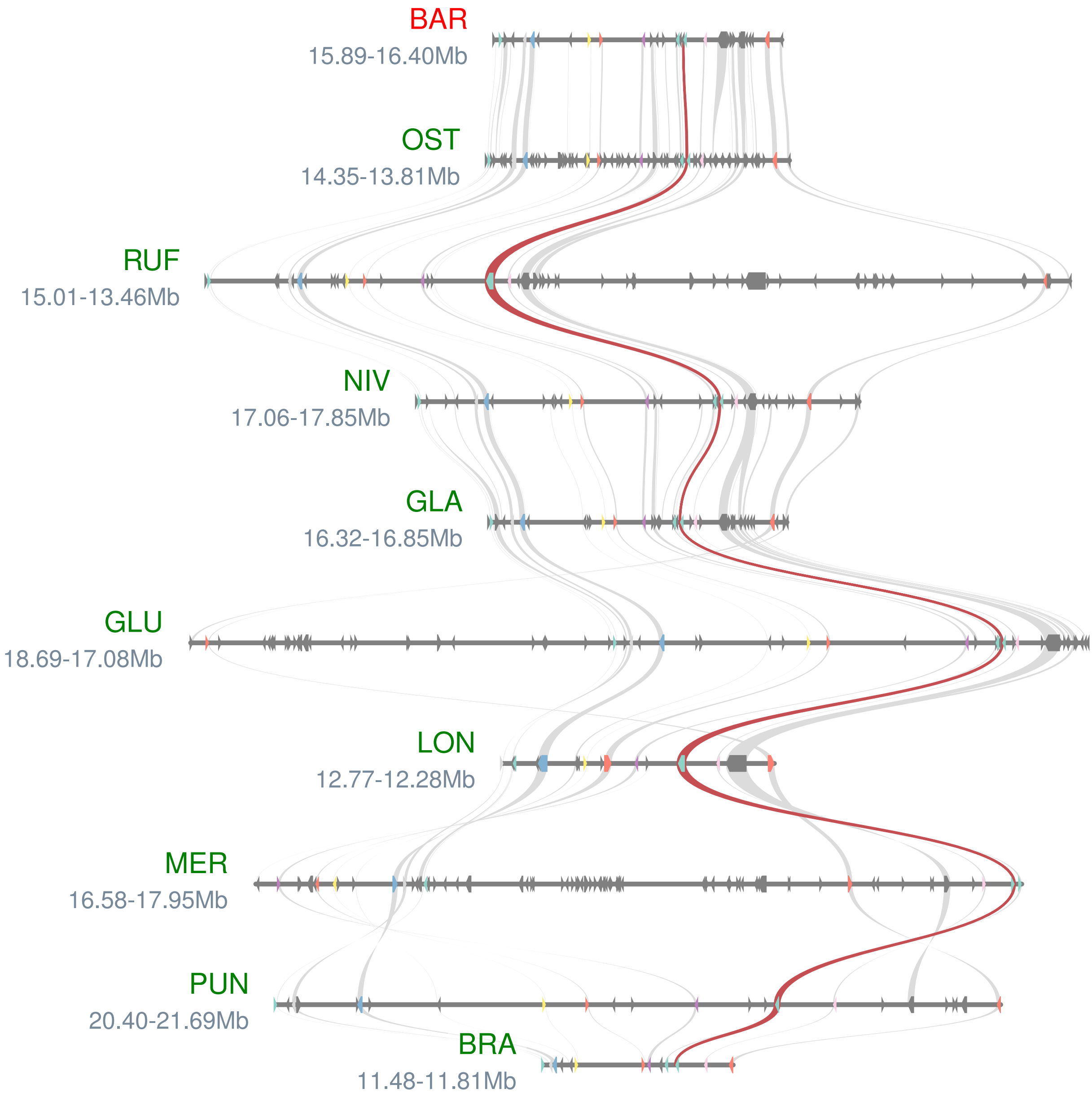

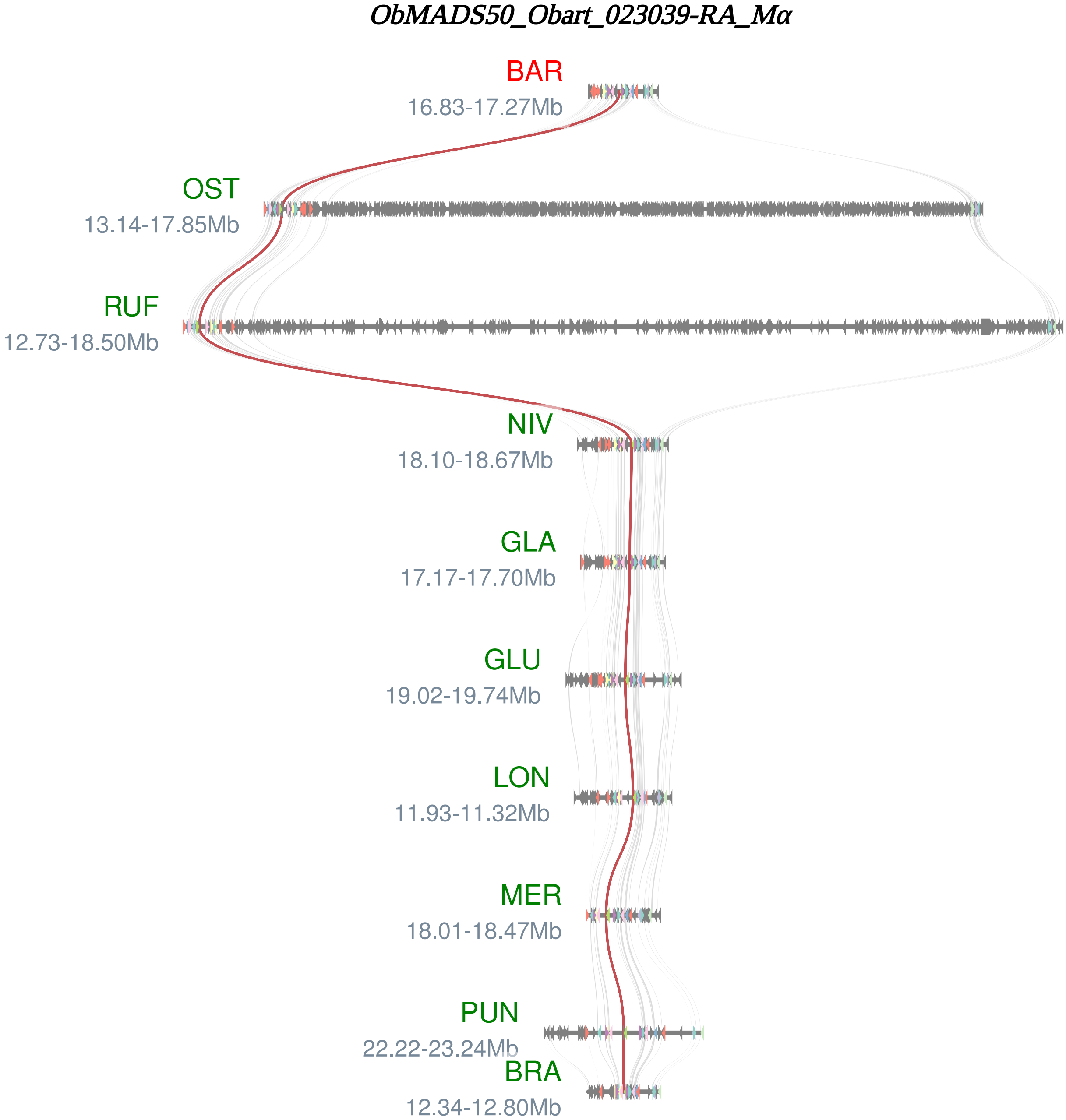

ObMADS50_Obart_023039-RA_Mα
BAR
16.83-17.27Mb
OST
13.14-17.85Mb
RUF
12.73-18.50Mb
NIV
18.10-18.67Mb
GLA
17.17-17.70Mb
GLU
19.02-19.74Mb
LON
11.93-11.32Mb
MER
18.01-18.47Mb
PUN
22.22-23.24Mb
BRA
12.34-12.80Mb

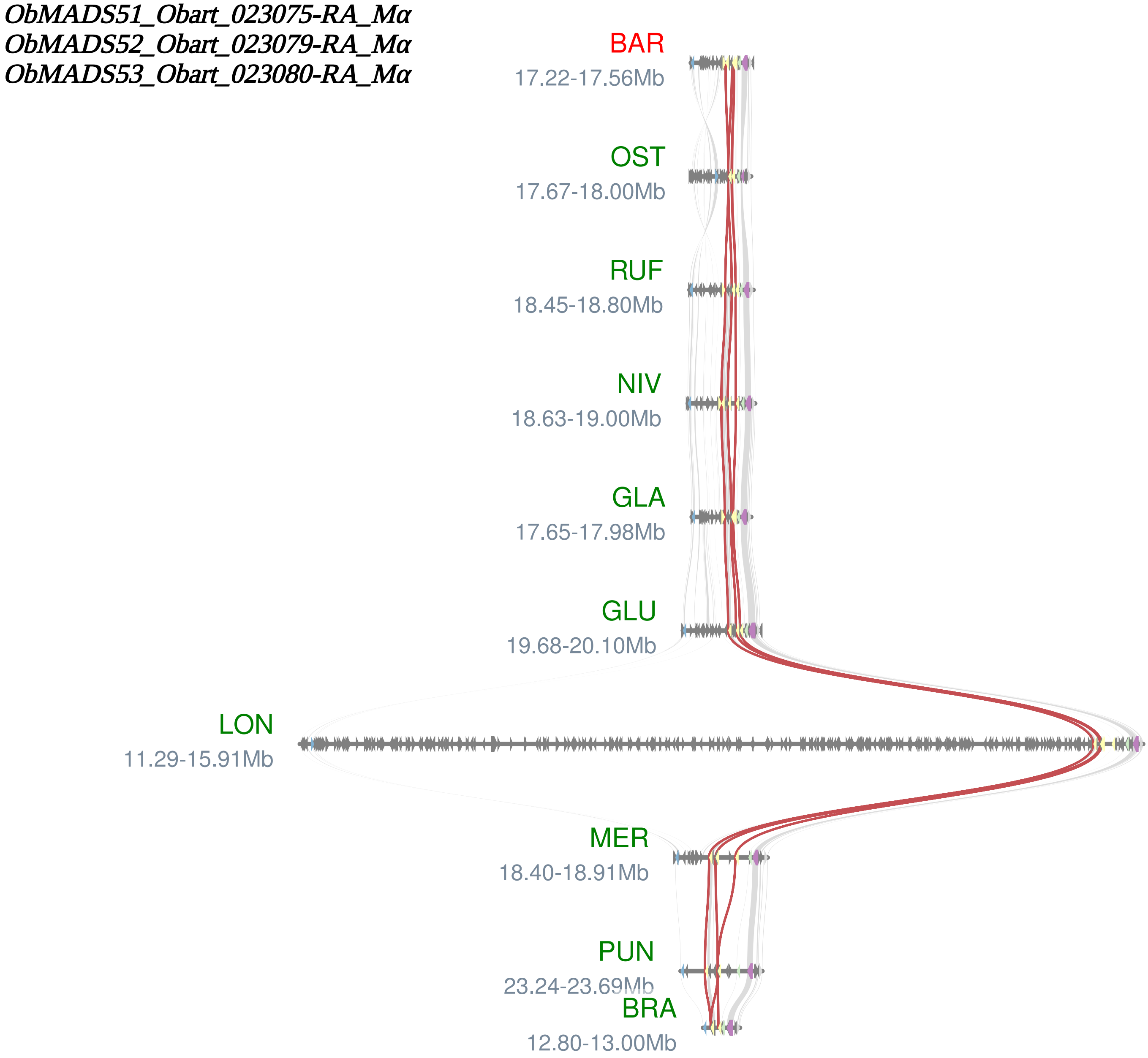

ObMADS51_Obart_023075-RA_Mα
ObMADS52_Obart_023079-RA_Mα
ObMADS53_Obart_023080-RA_Mα
BAR
17.22-17.56Mb
OST
17.67-18.00Mb
RUF
18.45-18.80Mb
NIV
18.63-19.00Mb
GLA
17.65-17.98Mb
GLU
19.68-20.10Mb
LON
11.29-15.91Mb
MER
18.40-18.91Mb
PUN
23.24-23.69Mb
BRA
12.80-13.00Mb

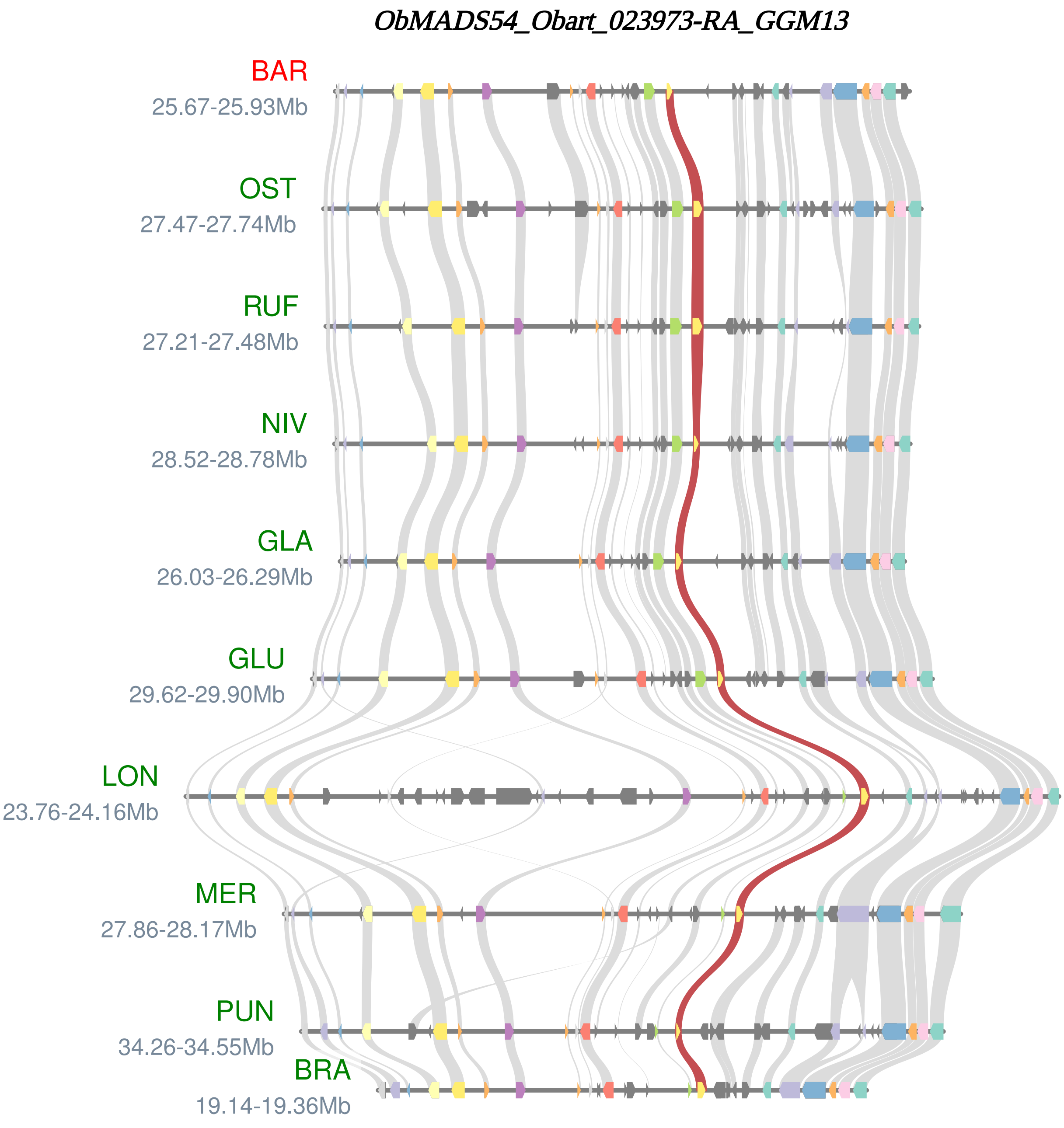
ObMADS54_Obart_023973-RA_GGM13
BAR
25.67-25.93Mb
OST
27.47-27.74Mb
RUF
27.21-27.48Mb
NIV
28.52-28.78Mb
GLA
26.03-26.29Mb
GLU
29.62-29.90Mb
LON
23.76-24.16Mb
MER
27.86-28.17Mb
PUN
34.26-34.55Mb
BRA
19.14-19.36Mb

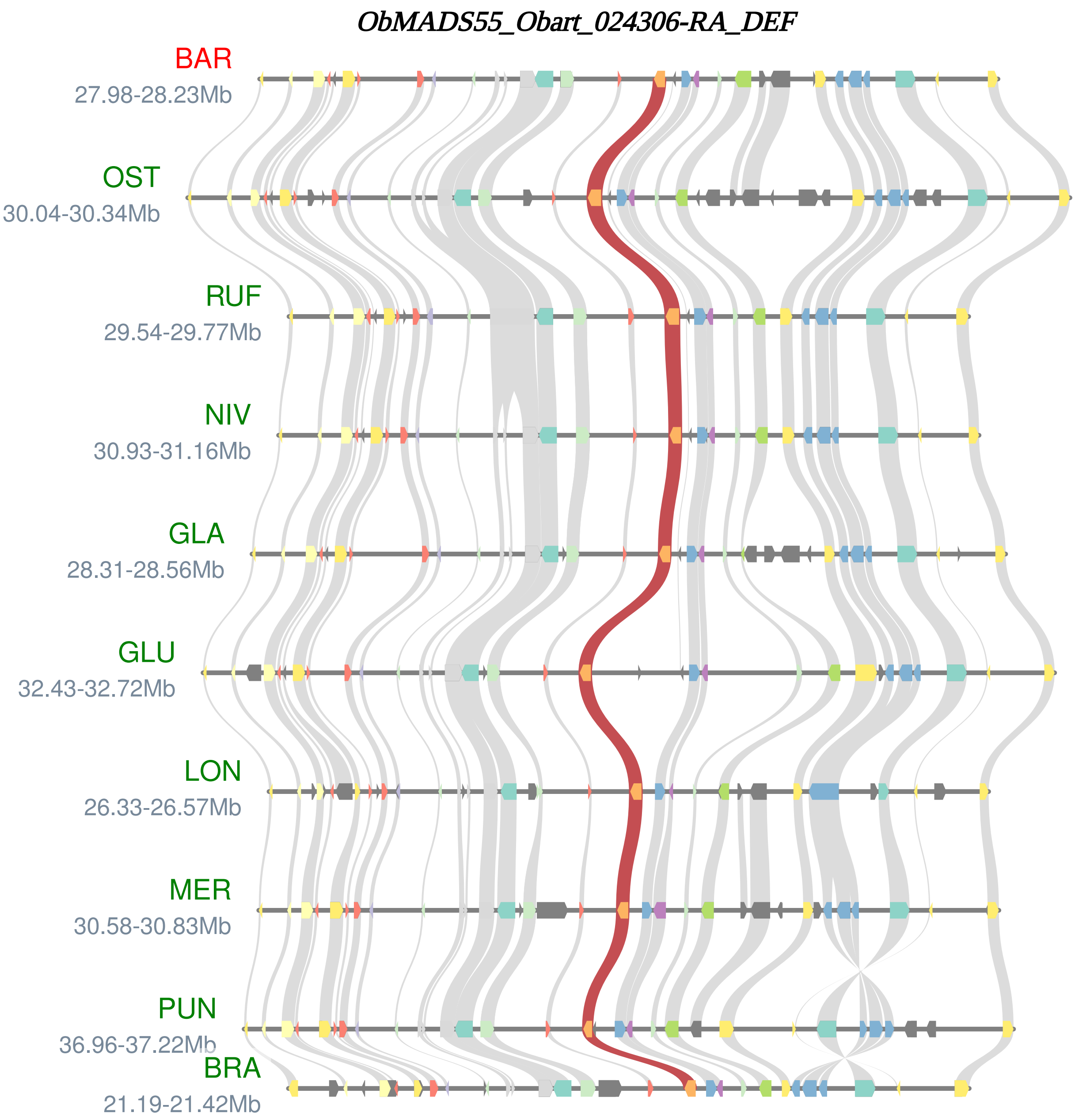
ObMADS55_Obart_024306-RA_DEF
BAR
27.98-28.23Mb
OST
30.04-30.34Mb
RUF
29.54-29.77Mb
NIV
30.93-31.16Mb
GLA
28.31-28.56Mb
GLU
32.43-32.72Mb
LON
26.33-26.57Mb
MER
30.58-30.83Mb
PUN
36.96-37.22Mb
BRA
21.19-21.42Mb

ObMADS56_Obart_024519-RA_AP1
(The chromosomal segment in the MER lacks any detected syntenic genes.)

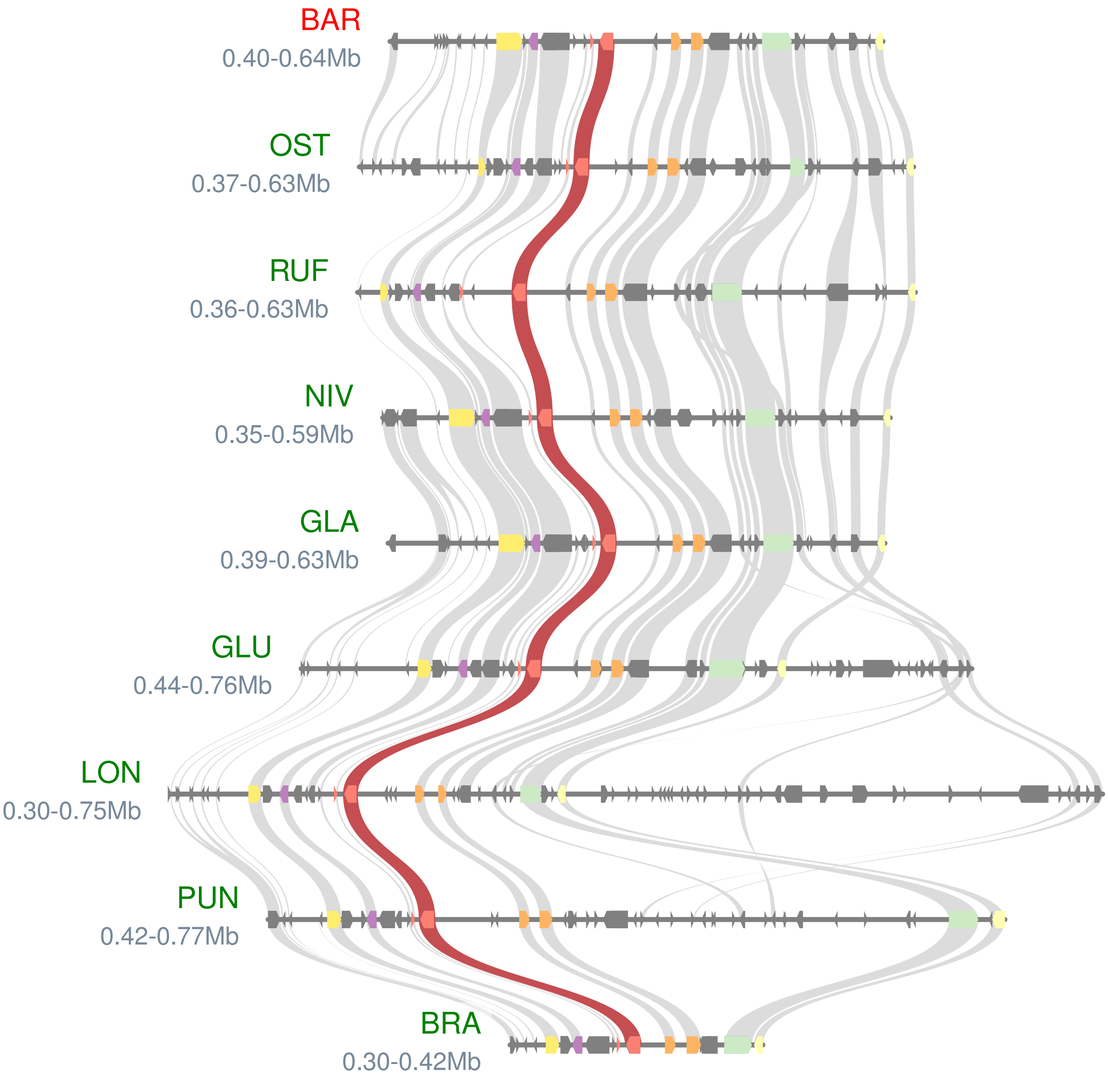

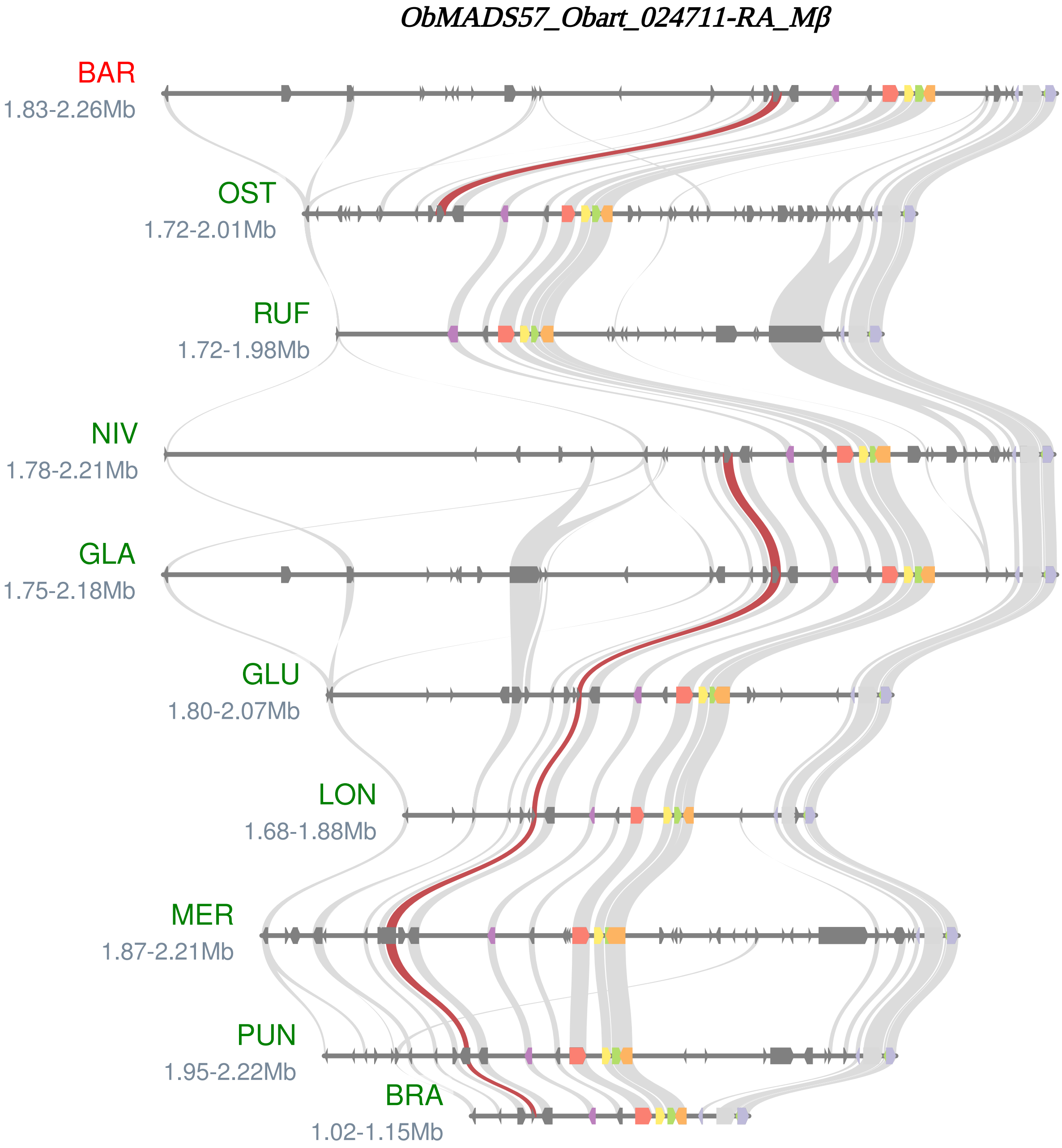

ObMADS57_Obart_024711-RA_Mβ
BAR
1.83-2.26Mb
OST
1.72-2.01Mb
RUF
1.72-1.98Mb
NIV
1.78-2.21Mb
GLA
1.75-2.18Mb
GLU
1.80-2.07Mb
LON
1.68-1.88Mb
MER
1.87-2.21Mb
PUN
1.95-2.22Mb
BRA
1.02-1.15Mb

ObMADS58_Obart_026965-RB_AP1

(The chromosomal segment in the LON lacks any detected syntenic genes.)

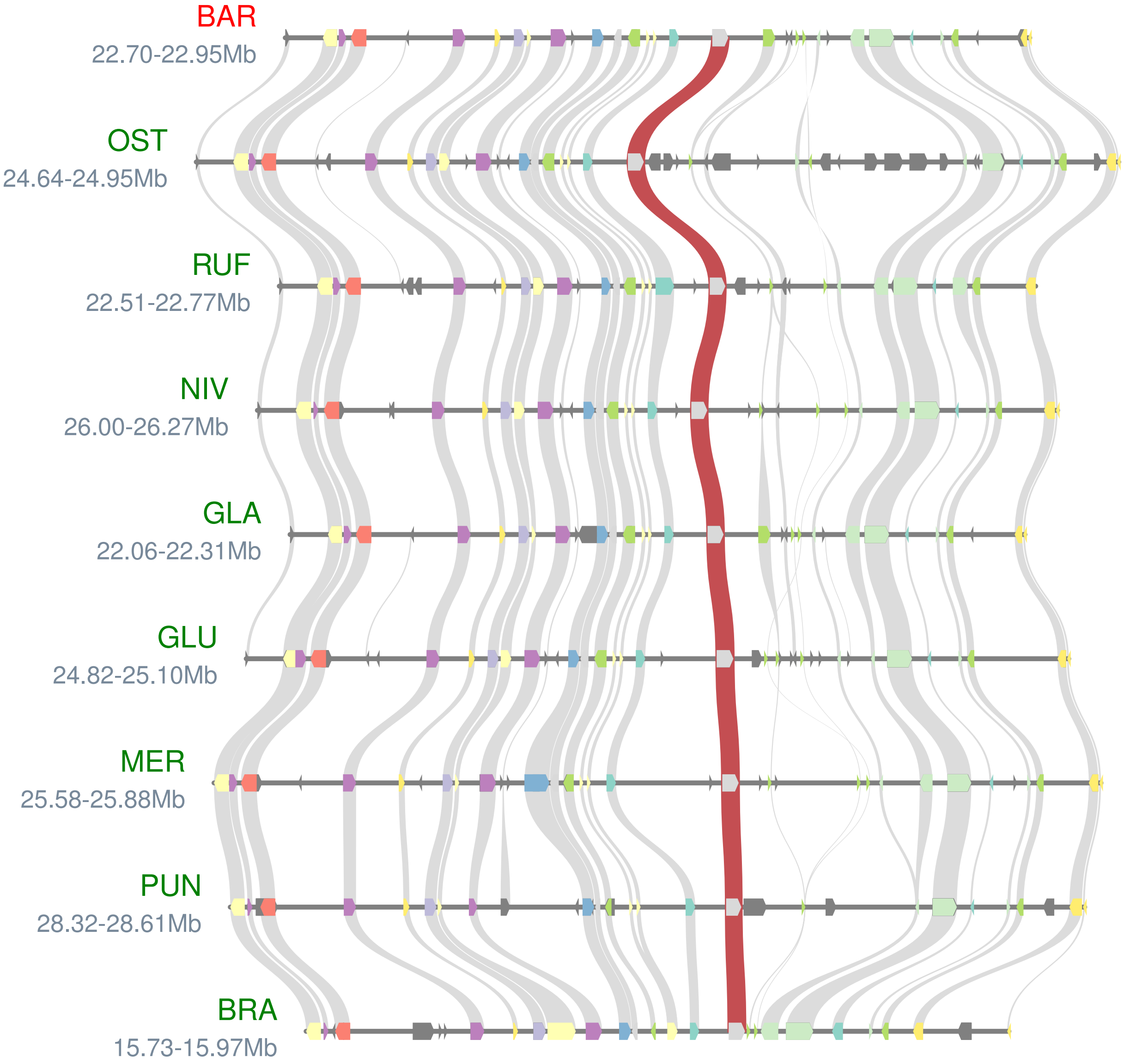

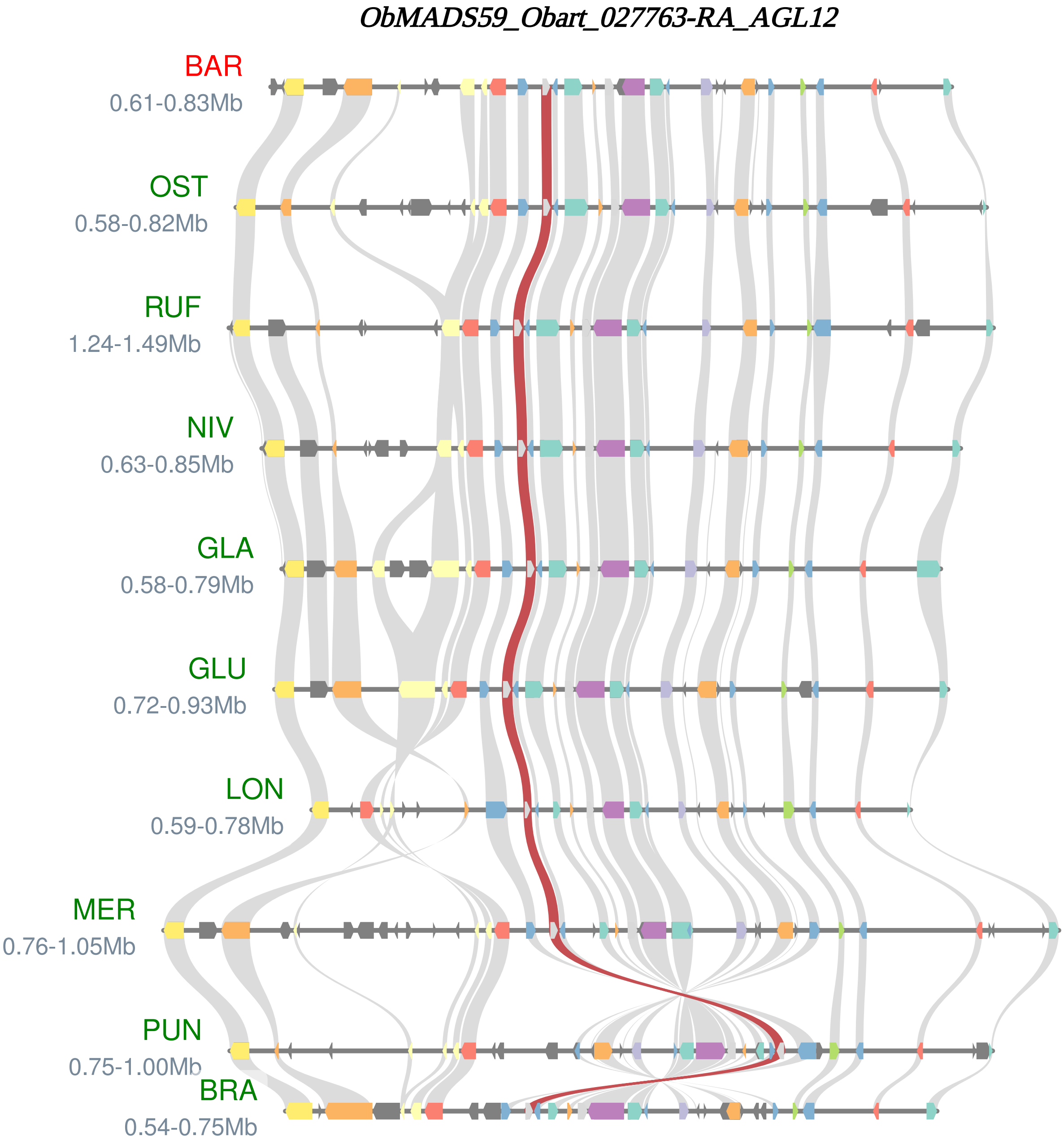

ObMADS59_Obart_027763-RA_AGL12
BAR
0.61-0.83Mb
OST
0.58-0.82Mb
RUF
1.24-1.49Mb
NIV
0.63-0.85Mb
GLA
0.58-0.79Mb
GLU
0.72-0.93Mb
LON
0.59-0.78Mb
MER
0.76-1.05Mb
PUN
0.75-1.00Mb
BRA
0.54-0.75Mb

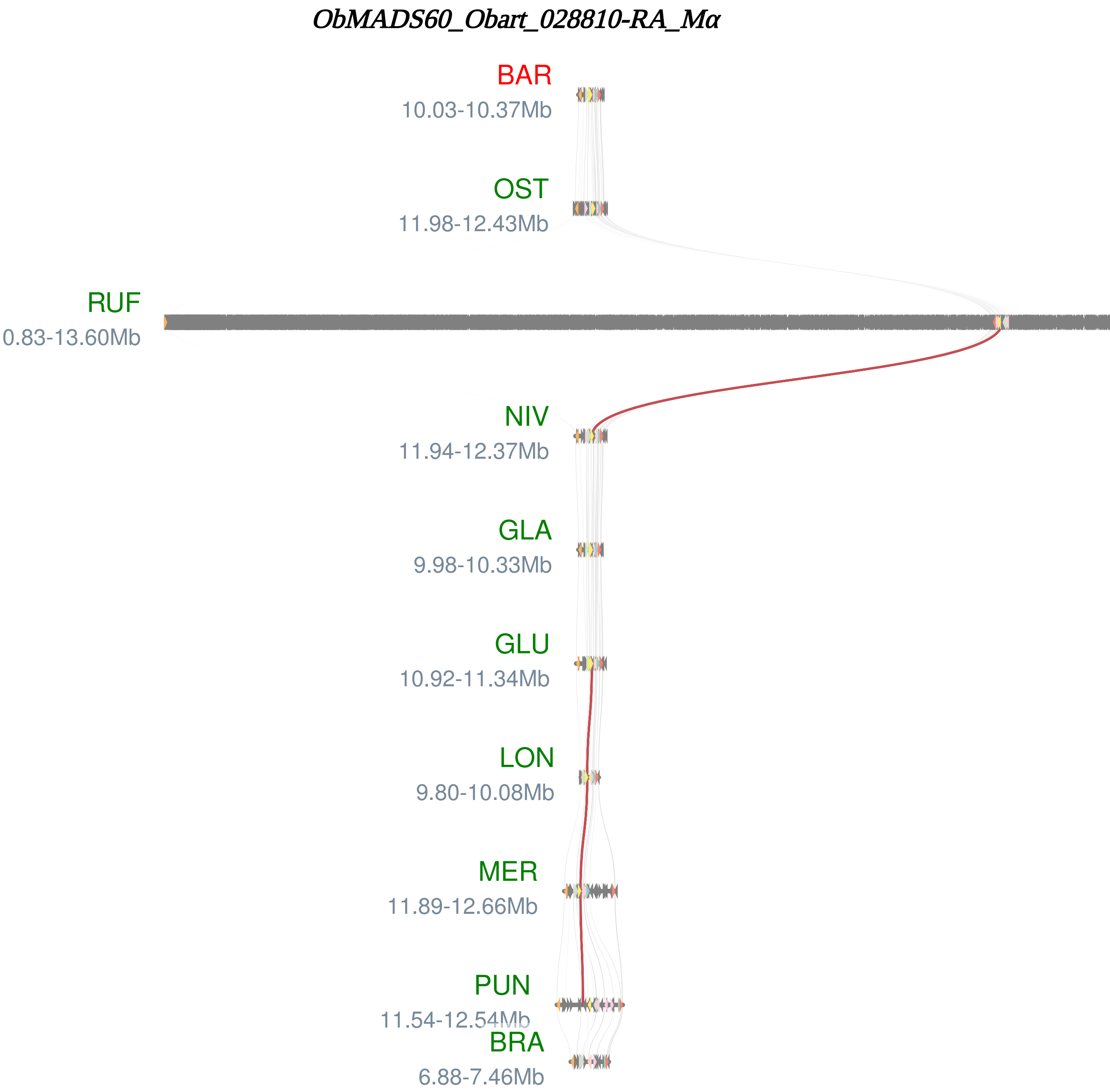
ObMADS60_Obart_028810-RA_Mα
BAR
10.03-10.37Mb
OST
11.98-12.43Mb
RUF
0.83-13.60Mb
NIV
11.94-12.37Mb
GLA
9.98-10.33Mb
GLU
10.92-11.34Mb
LON
9.80-10.08Mb
MER
11.89-12.66Mb
PUN
11.54-12.54Mb
BRA
6.88-7.46Mb

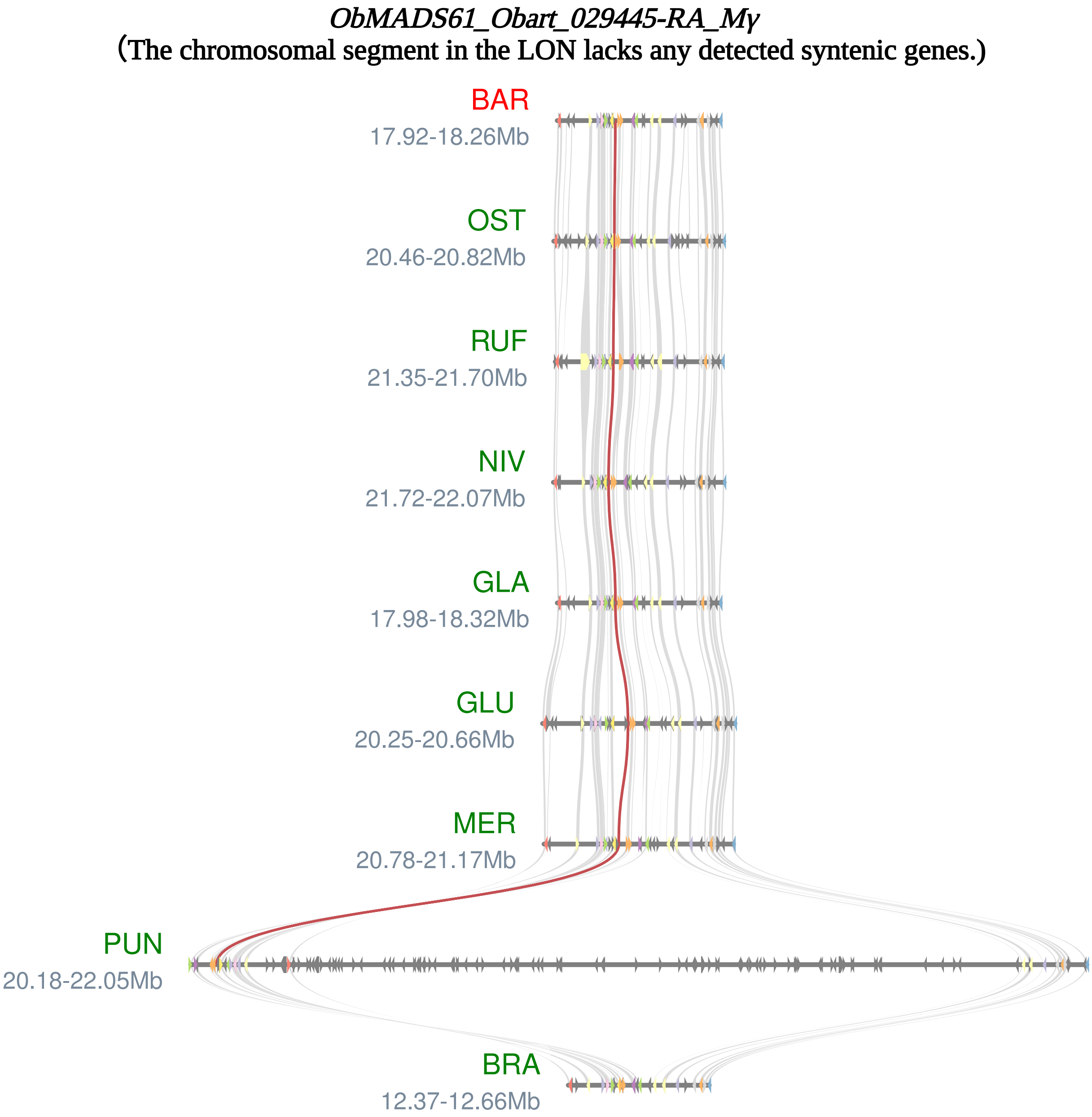
ObMADS61_Obart_029445-RA_Mγ
(The chromosomal segment in the LON lacks any detected syntenic genes.)
BAR
17.92-18.26Mb
OST
20.46-20.82Mb
RUF
21.35-21.70Mb
NIV
21.72-22.07Mb
GLA
17.98-18.32Mb
GLU
20.25-20.66Mb
MER
20.78-21.17Mb
PUN
20.18-22.05Mb
BRA
12.37-12.66Mb

ObMADS62_Obart_029473-RC_AGL17
(The chromosomal segment in the LON lacks any detected syntenic genes.)

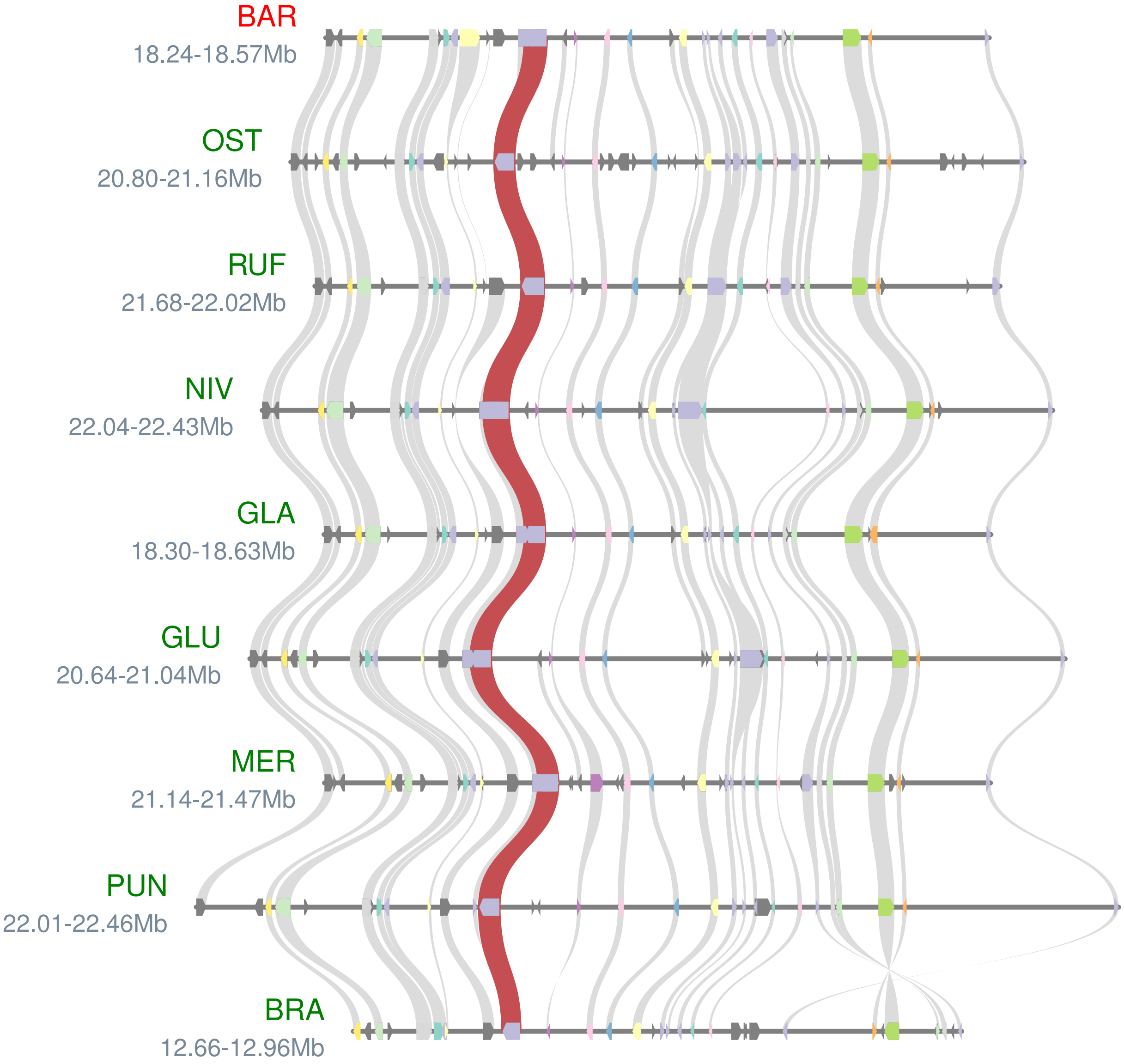

*ObMADS63_Obart_029890-RA_MIKC**

(The chromosomal segment in the LON lacks any detected syntenic genes.)

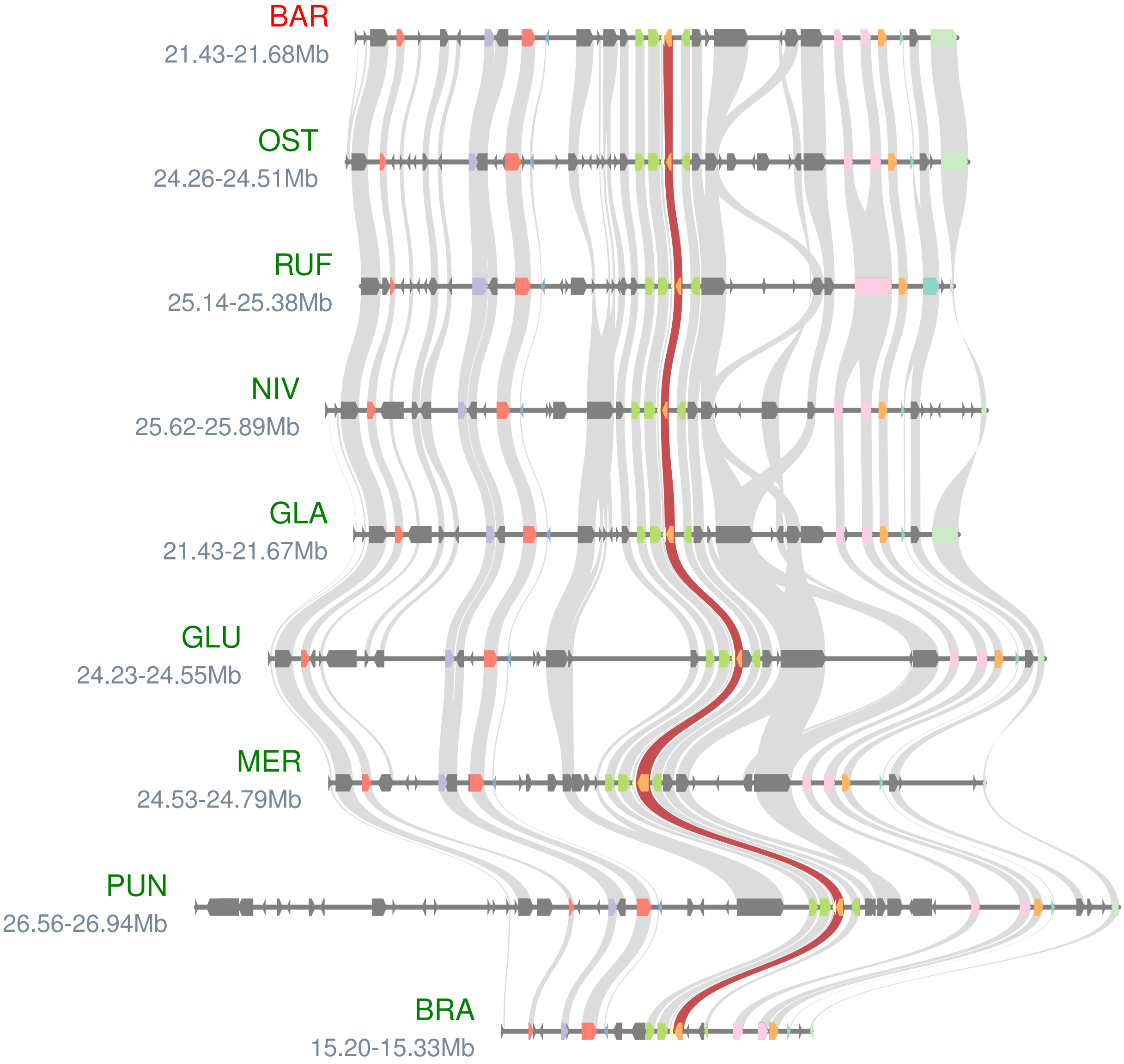

ObMADS64_Obart_030174-RB_SEP
(The chromosomal segment in the LON lacks any detected syntenic genes.)

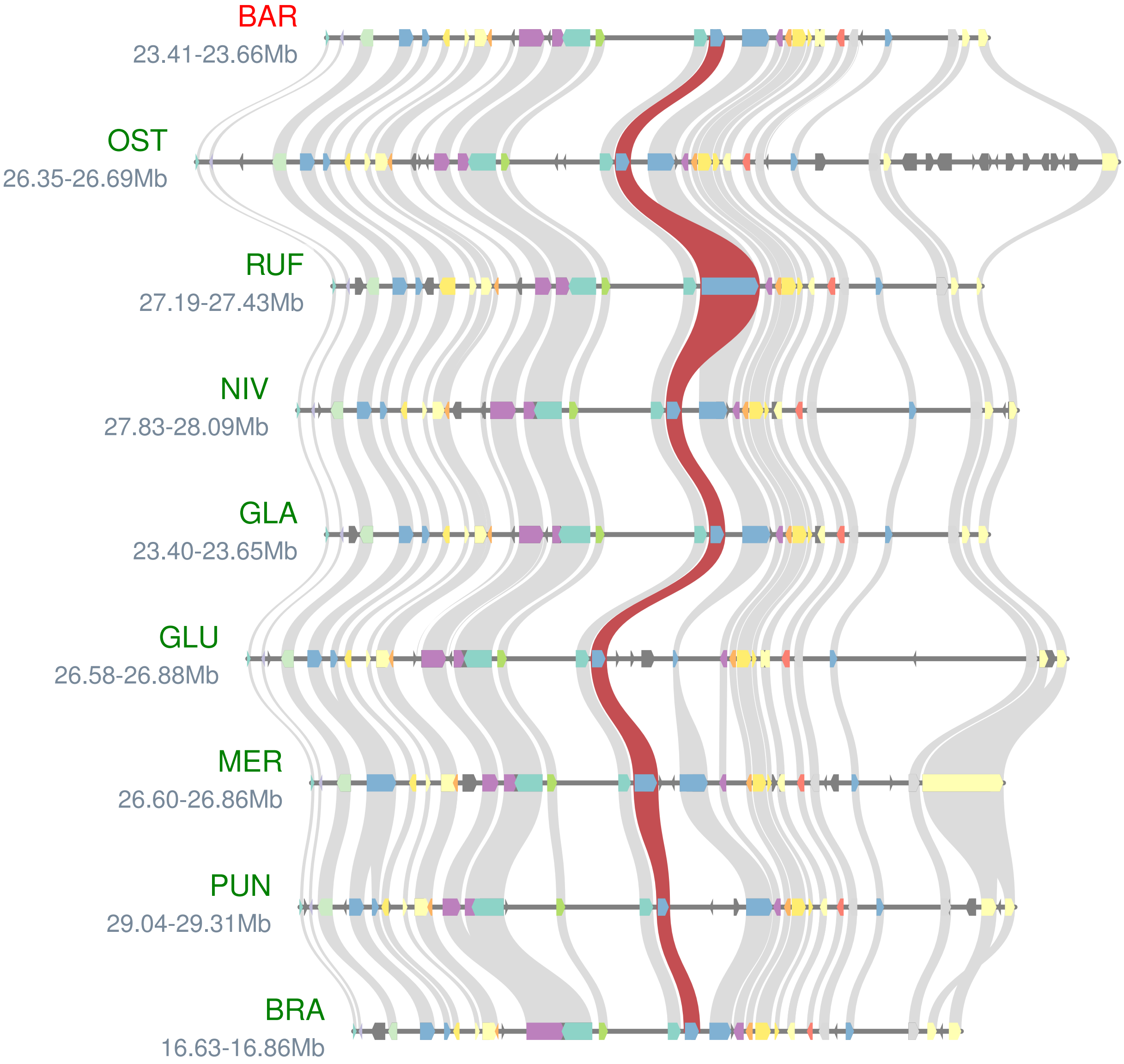

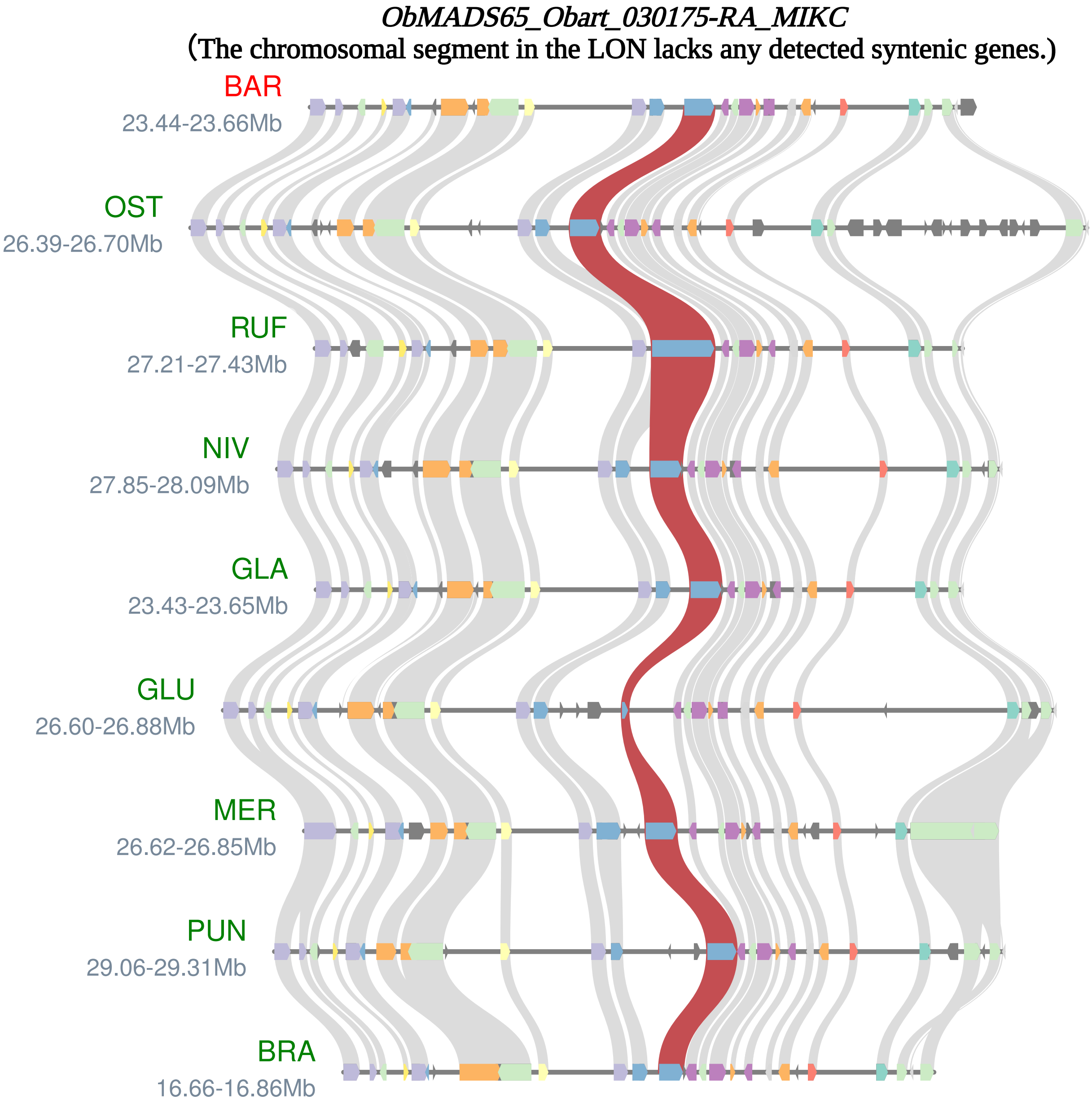

ObMADS65_Obart_030175-RA_MIKC
(The chromosomal segment in the LON lacks any detected syntenic genes.)
BAR
23.44-23.66Mb
OST
26.39-26.70Mb
RUF
27.21-27.43Mb
NIV
27.85-28.09Mb
GLA
23.43-23.65Mb
GLU
26.60-26.88Mb
MER
26.62-26.85Mb
PUN
29.06-29.31Mb
BRA
16.66-16.86Mb

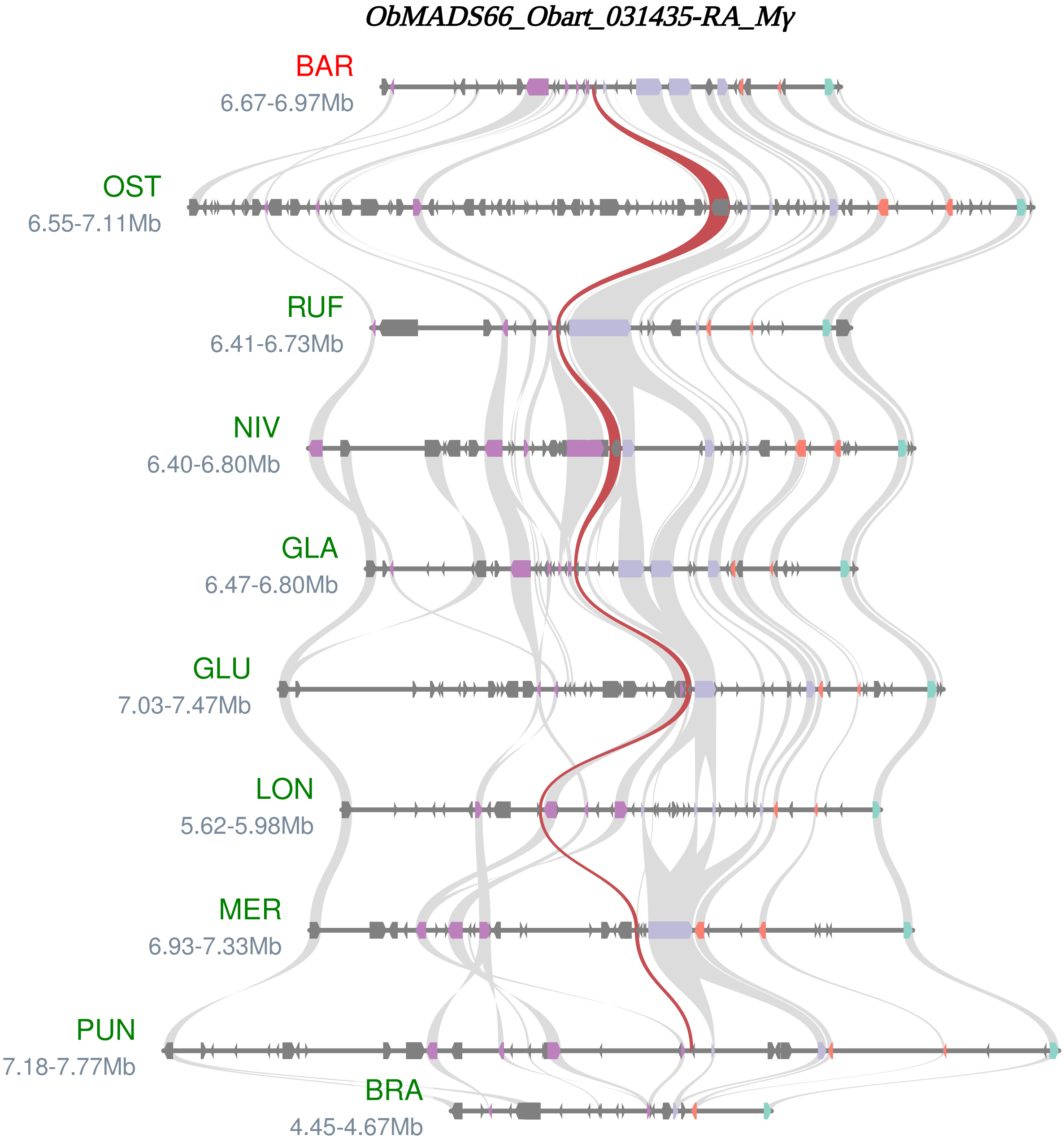
ObMADS66_Obart_031435-RA_Mγ
BAR
6.67-6.97Mb
OST
6.55-7.11Mb
RUF
6.41-6.73Mb
NIV
6.40-6.80Mb
GLA
6.47-6.80Mb
GLU
7.03-7.47Mb
LON
5.62-5.98Mb
MER
6.93-7.33Mb
PUN
7.18-7.77Mb
BRA
4.45-4.67Mb

ObMADS67_Obart_033046-RB_MIKC
(The chromosomal segment in the LON lacks any detected syntenic genes.)

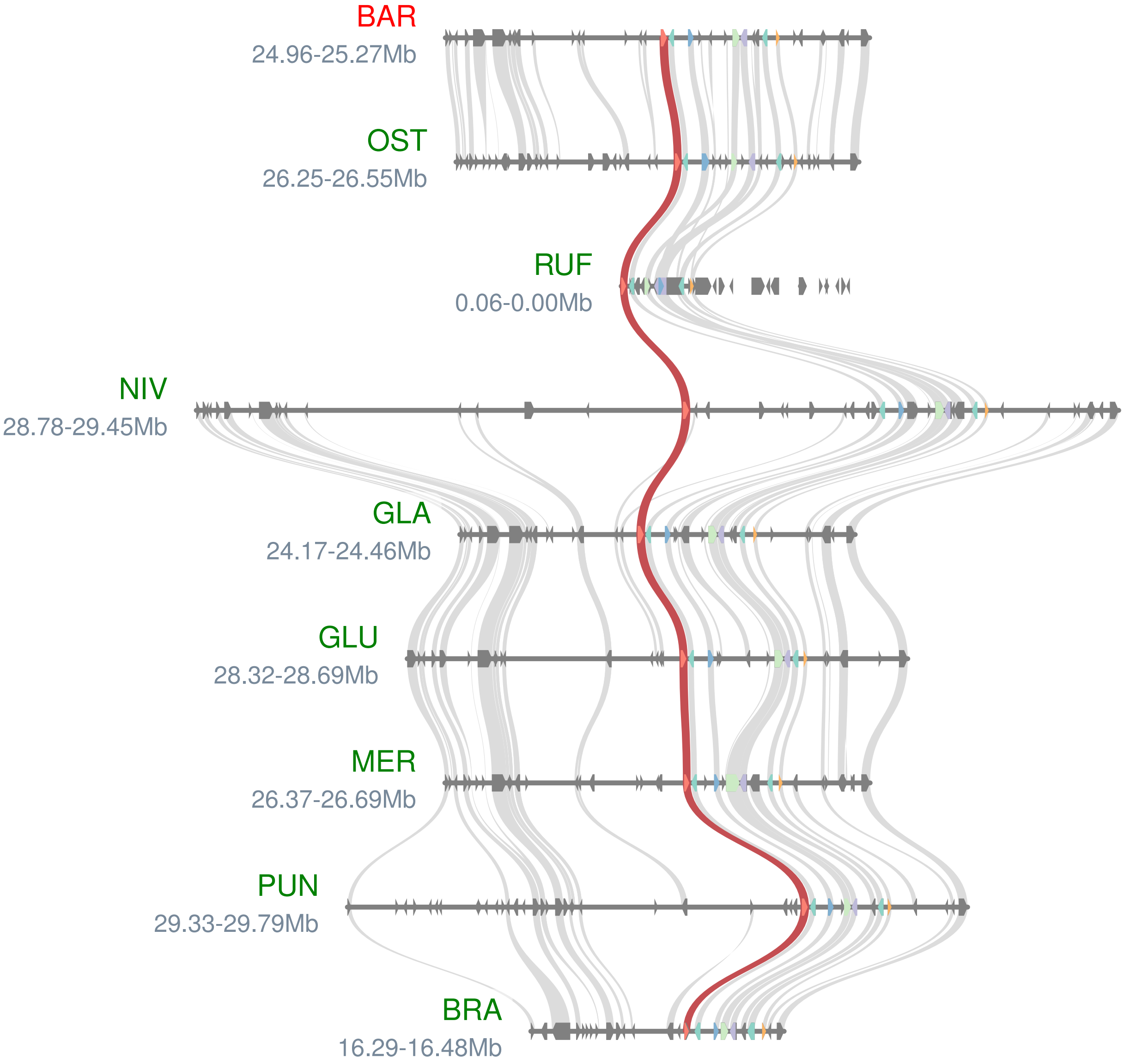

ObMADS68_Obart_034012-RA_AGL12
(The chromosomal segment in the OST lacks any detected syntenic genes.)

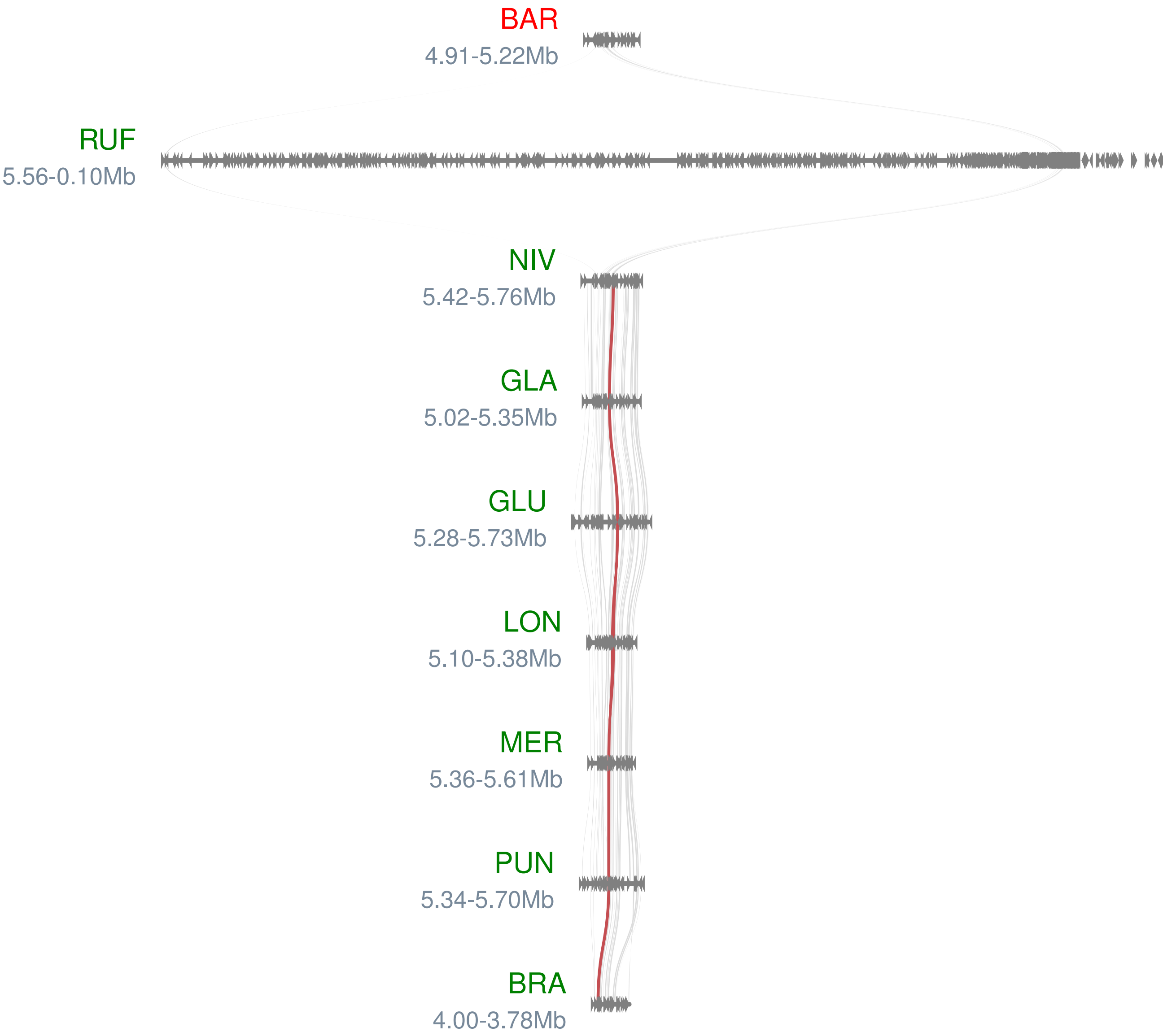

ObMADS69_Obart_034013-RC_AG
(The chromosomal segment in the OST lacks any detected syntenic genes.)

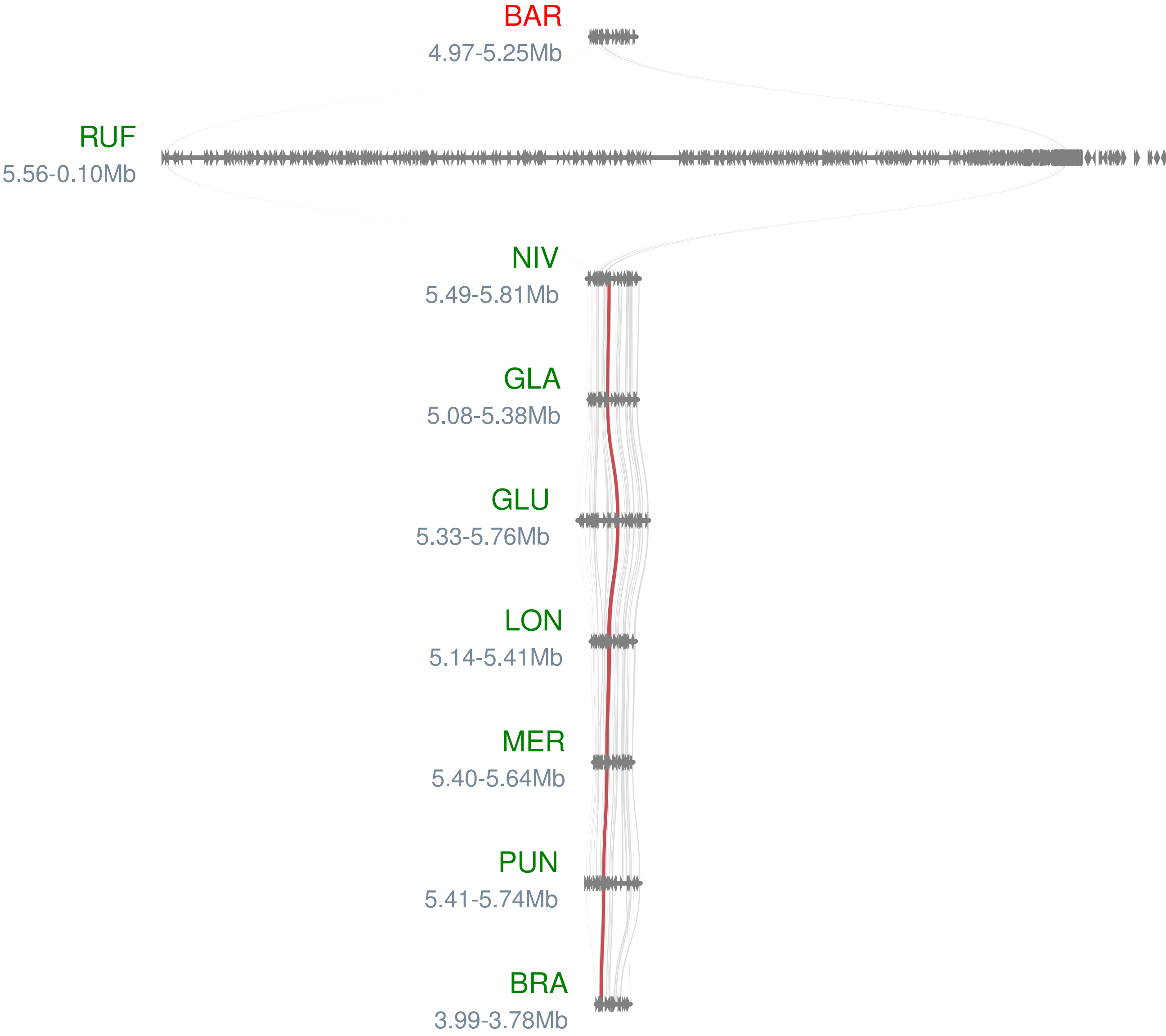

ObMADS70_Obart_034450-RA_Mα
ObMADS71_Obart_034454-RA_Mα
(The chromosomal segment in the PUN lacks any detected syntenic genes.)

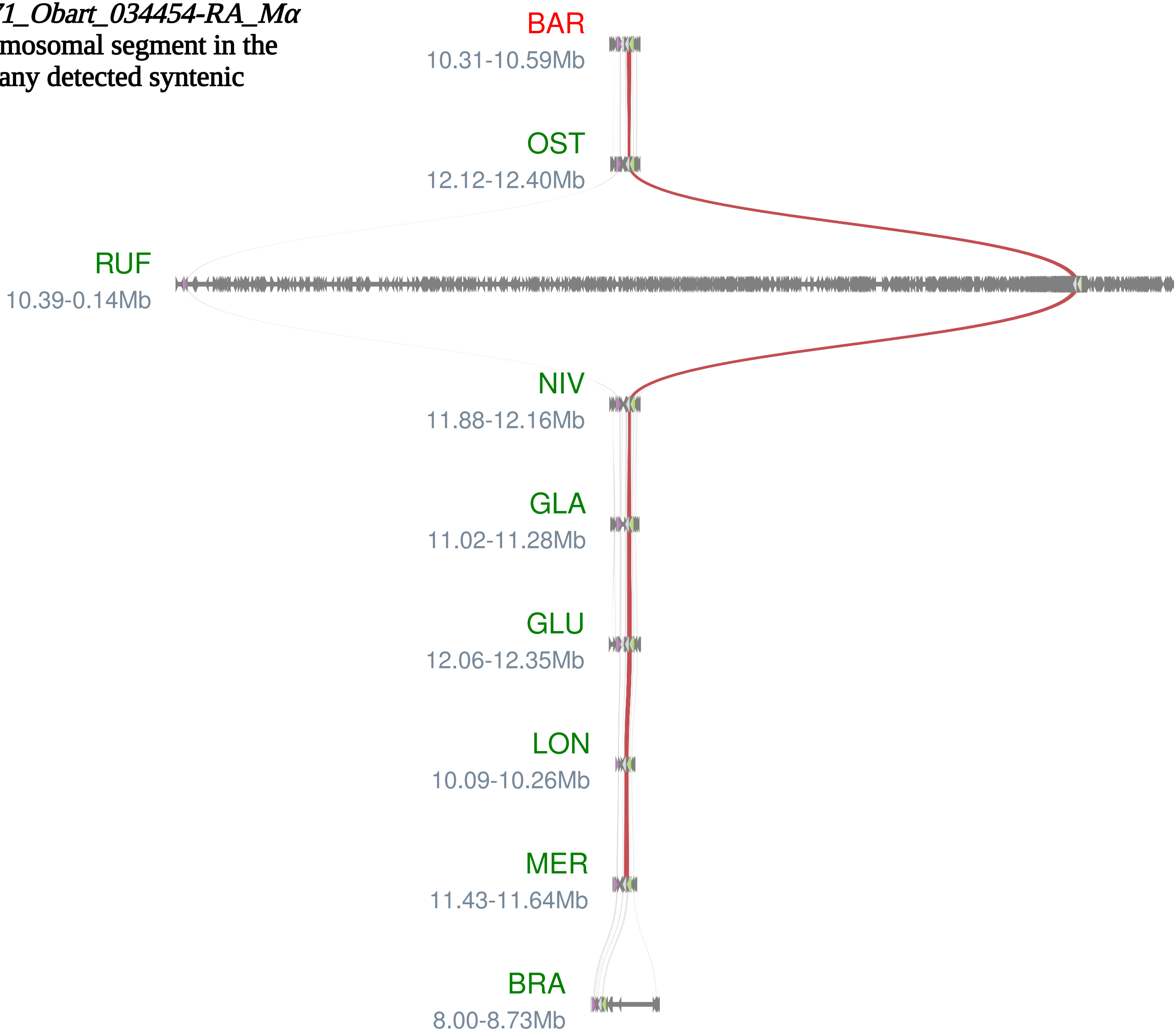

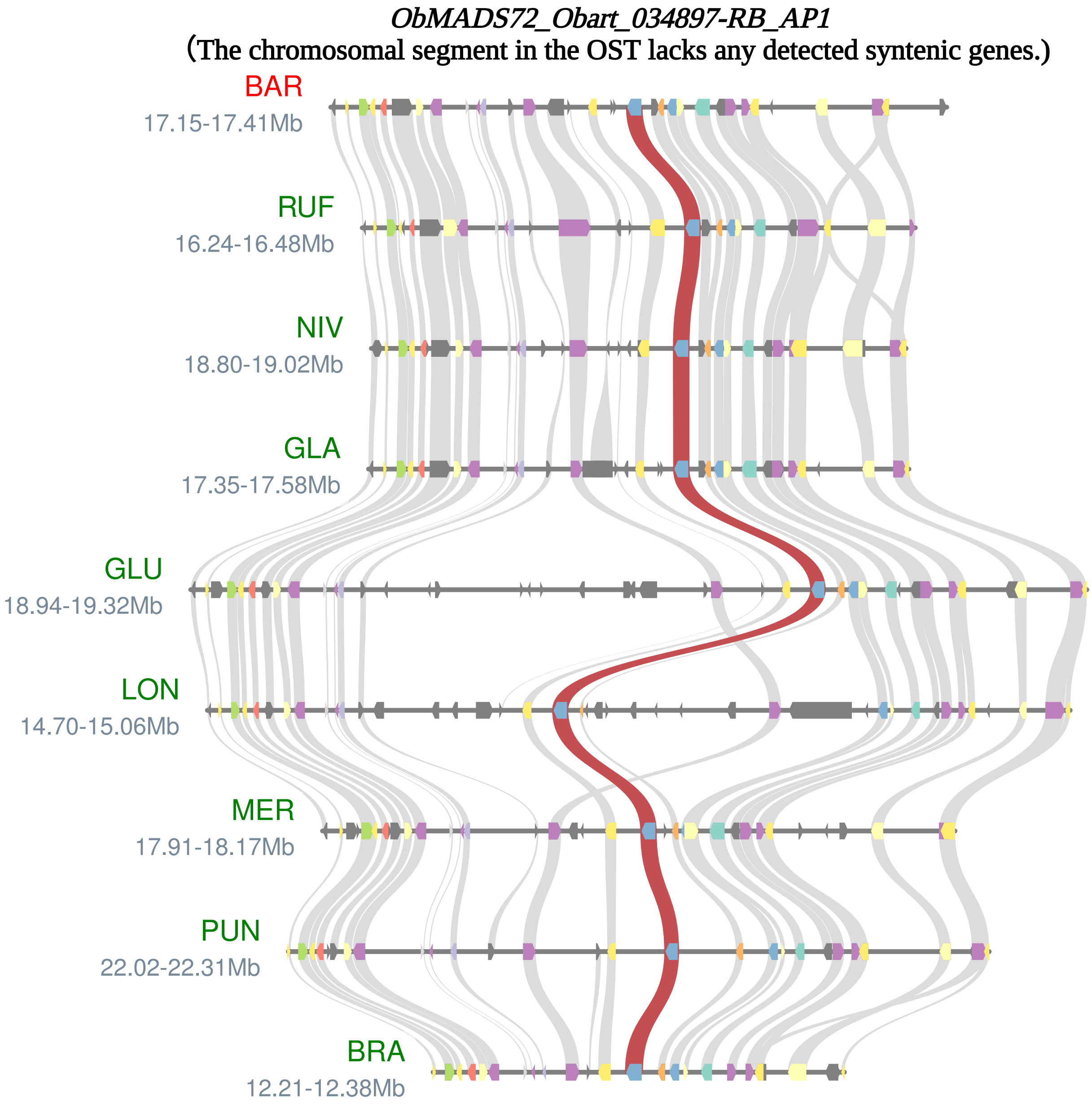
ObMADS72_Obart_034897-RB_AP1
(The chromosomal segment in the OST lacks any detected syntenic genes.)
BAR
17.15-17.41Mb
RUF
16.24-16.48Mb
NIV
18.80-19.02Mb
GLA
17.35-17.58Mb
GLU
18.94-19.32Mb
LON
14.70-15.06Mb
MER
17.91-18.17Mb
PUN
22.02-22.31Mb
BRA
12.21-12.38Mb

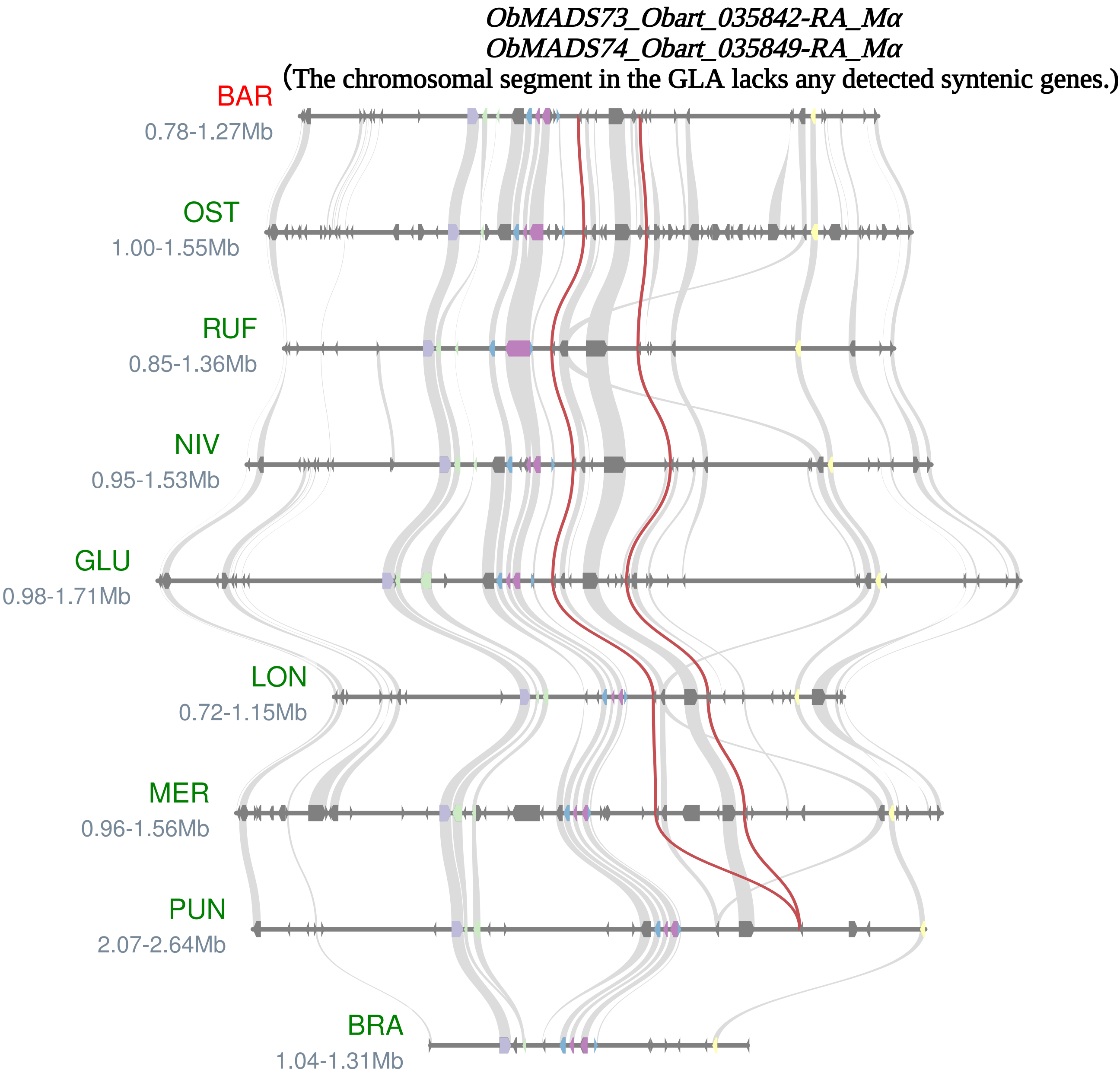
ObMADS73_Obart_035842-RA_Mα
ObMADS74_Obart_035849-RA_Mα
(The chromosomal segment in the GLA lacks any detected syntenic genes.)
BAR
0.78-1.27Mb
OST
1.00-1.55Mb
RUF
0.85-1.36Mb
NIV
0.95-1.53Mb
GLU
0.98-1.71Mb
LON
0.72-1.15Mb
MER
0.96-1.56Mb
PUN
2.07-2.64Mb
BRA
1.04-1.31Mb

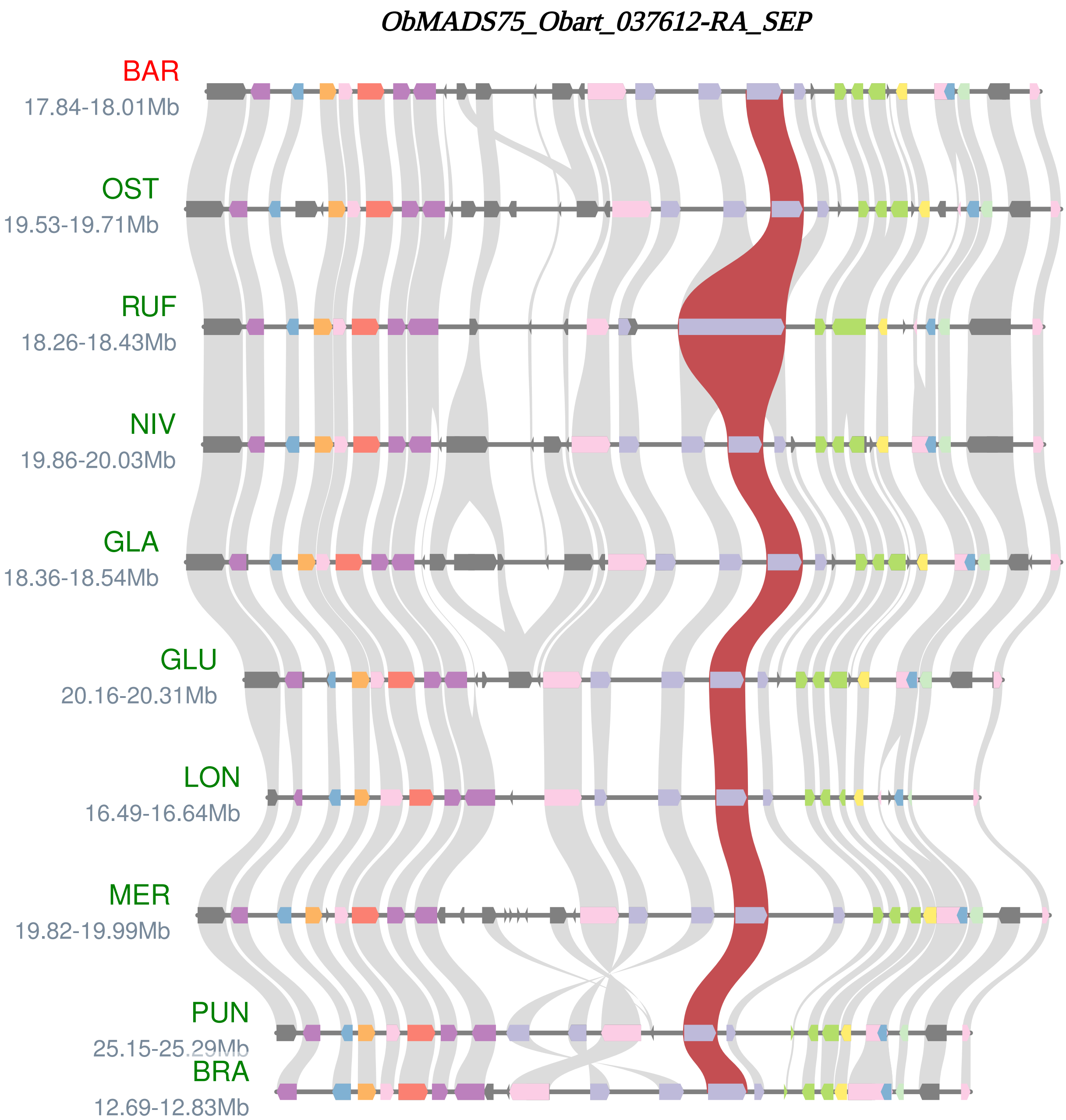
ObMADS75_Obart_037612-RA_SEP
BAR
17.84-18.01Mb
OST
19.53-19.71Mb
RUF
18.26-18.43Mb
NIV
19.86-20.03Mb
GLA
18.36-18.54Mb
GLU
20.16-20.31Mb
LON
16.49-16.64Mb
MER
19.82-19.99Mb
PUN
25.15-25.29Mb
BRA
12.69-12.83Mb

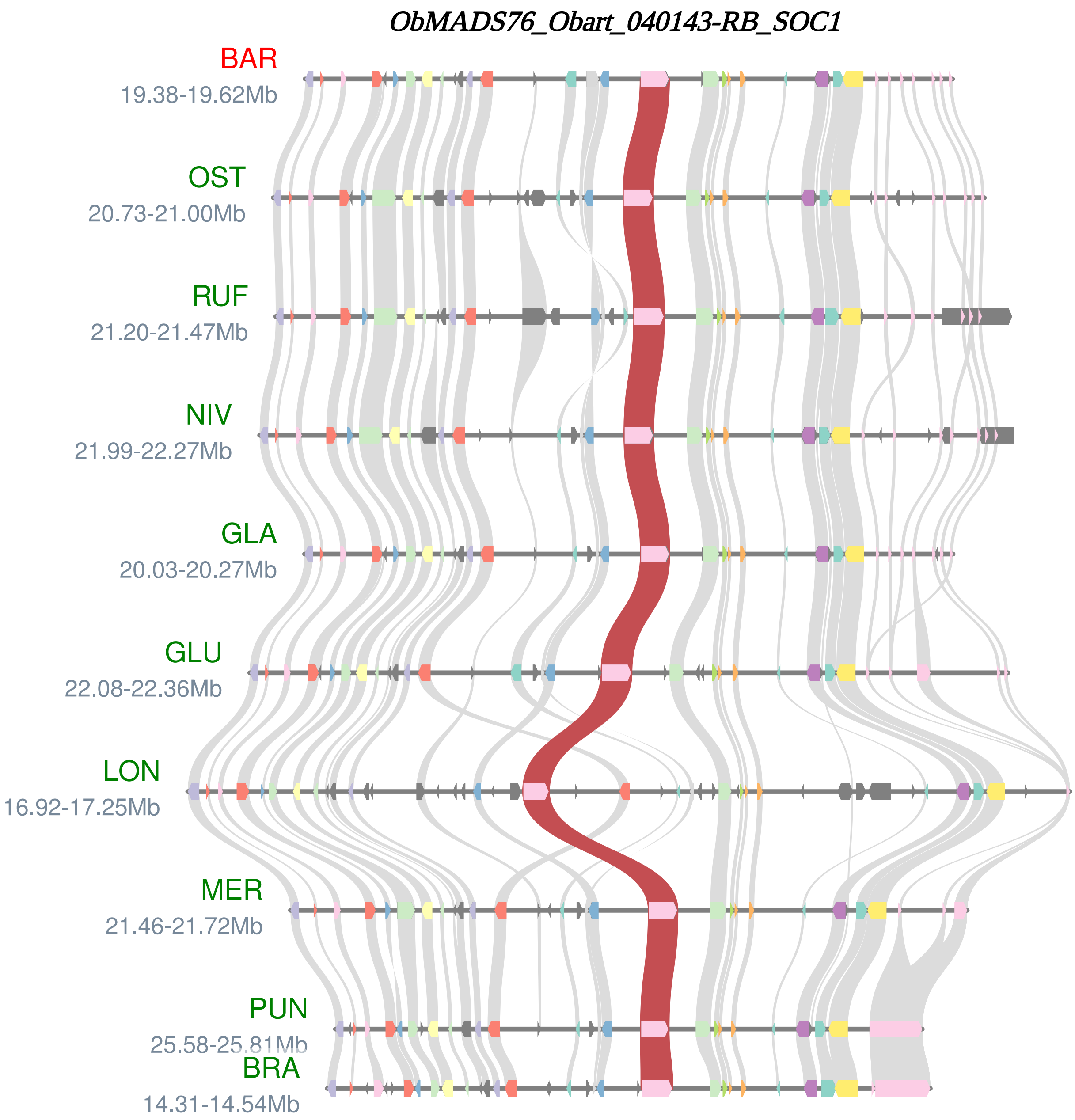

ObMADS76_Obart_040143-RB_SOC1
BAR
19.38-19.62Mb
OST
20.73-21.00Mb
RUF
21.20-21.47Mb
NIV
21.99-22.27Mb
GLA
20.03-20.27Mb
GLU
22.08-22.36Mb
LON
16.92-17.25Mb
MER
21.46-21.72Mb
PUN
25.58-25.81Mb
BRA
14.31-14.54Mb

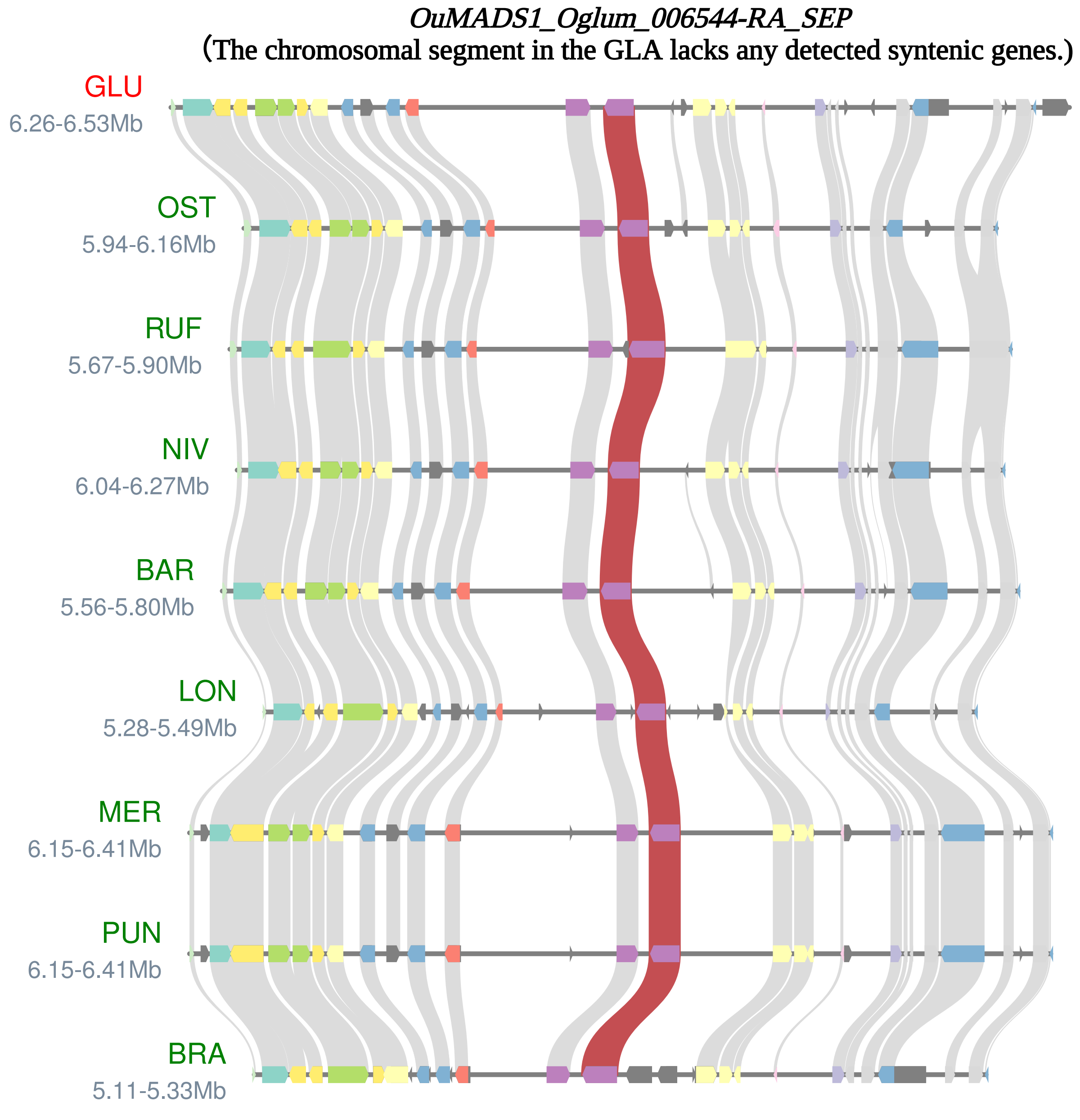
OuMADS1_Oglum_006544-RA_SEP
(The chromosomal segment in the GLA lacks any detected syntenic genes.)
GLU
6.26-6.53Mb
OST
5.94-6.16Mb
RUF
5.67-5.90Mb
NIV
6.04-6.27Mb
BAR
5.56-5.80Mb
LON
5.28-5.49Mb
MER
6.15-6.41Mb
PUN
6.15-6.41Mb
BRA
5.11-5.33Mb

OuMADS2_Oglum_035096-RA_Mα
OuMADS12_Oglum_035091-RA_Mα

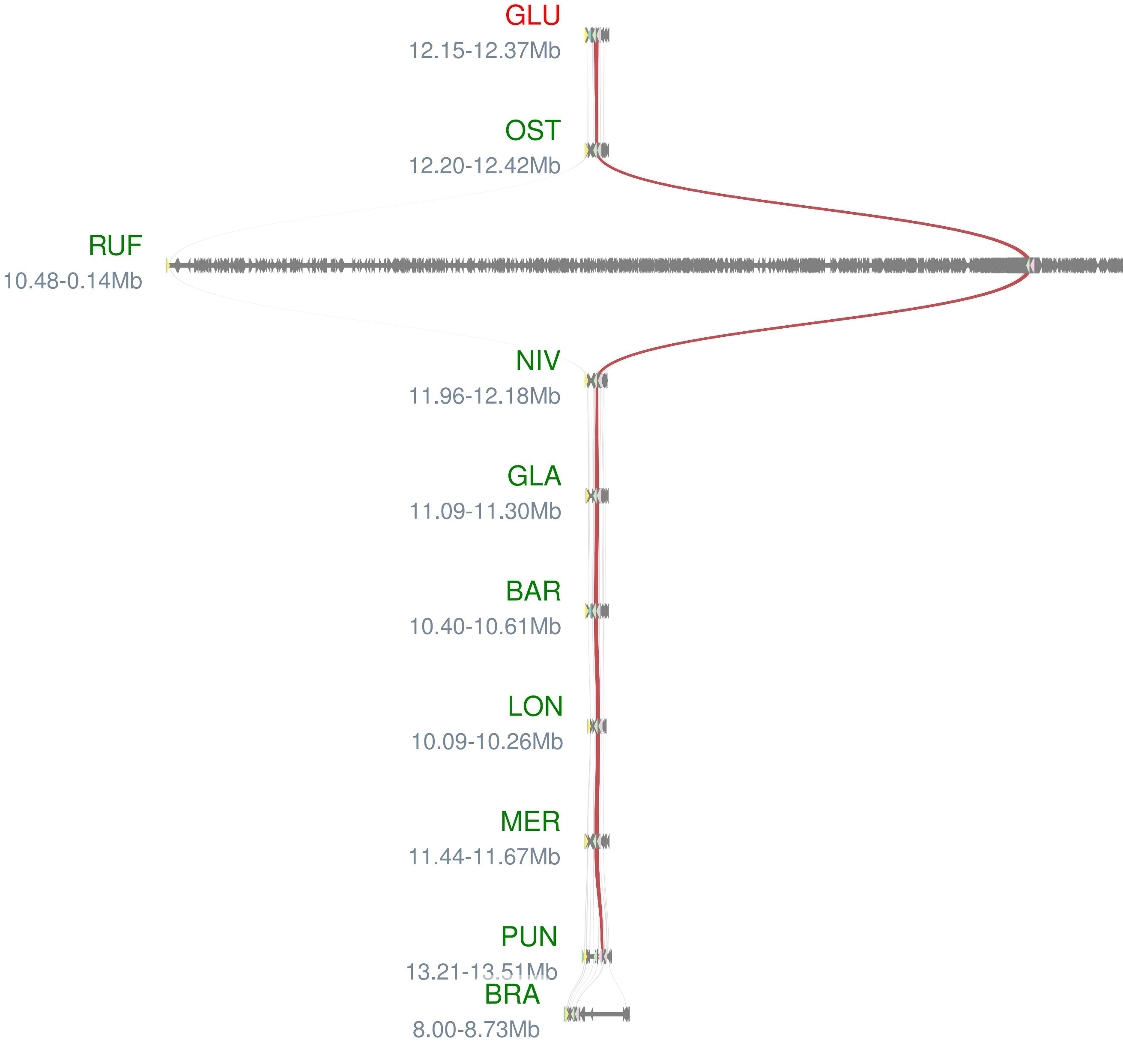

OuMADS3_Oglum_015883-RA_Mγ

(The chromosomal segment in the NIV lacks any detected syntenic genes.)

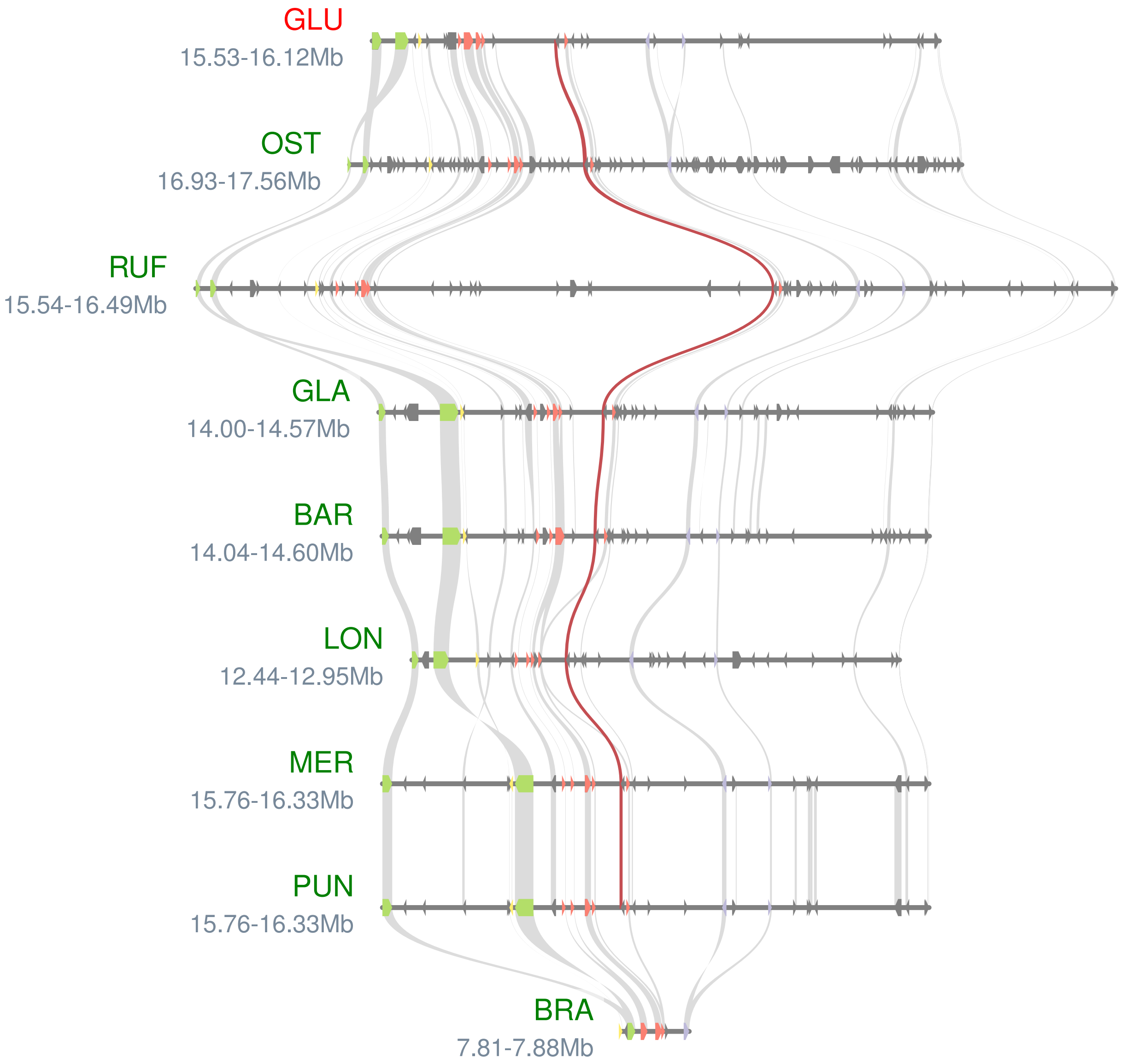

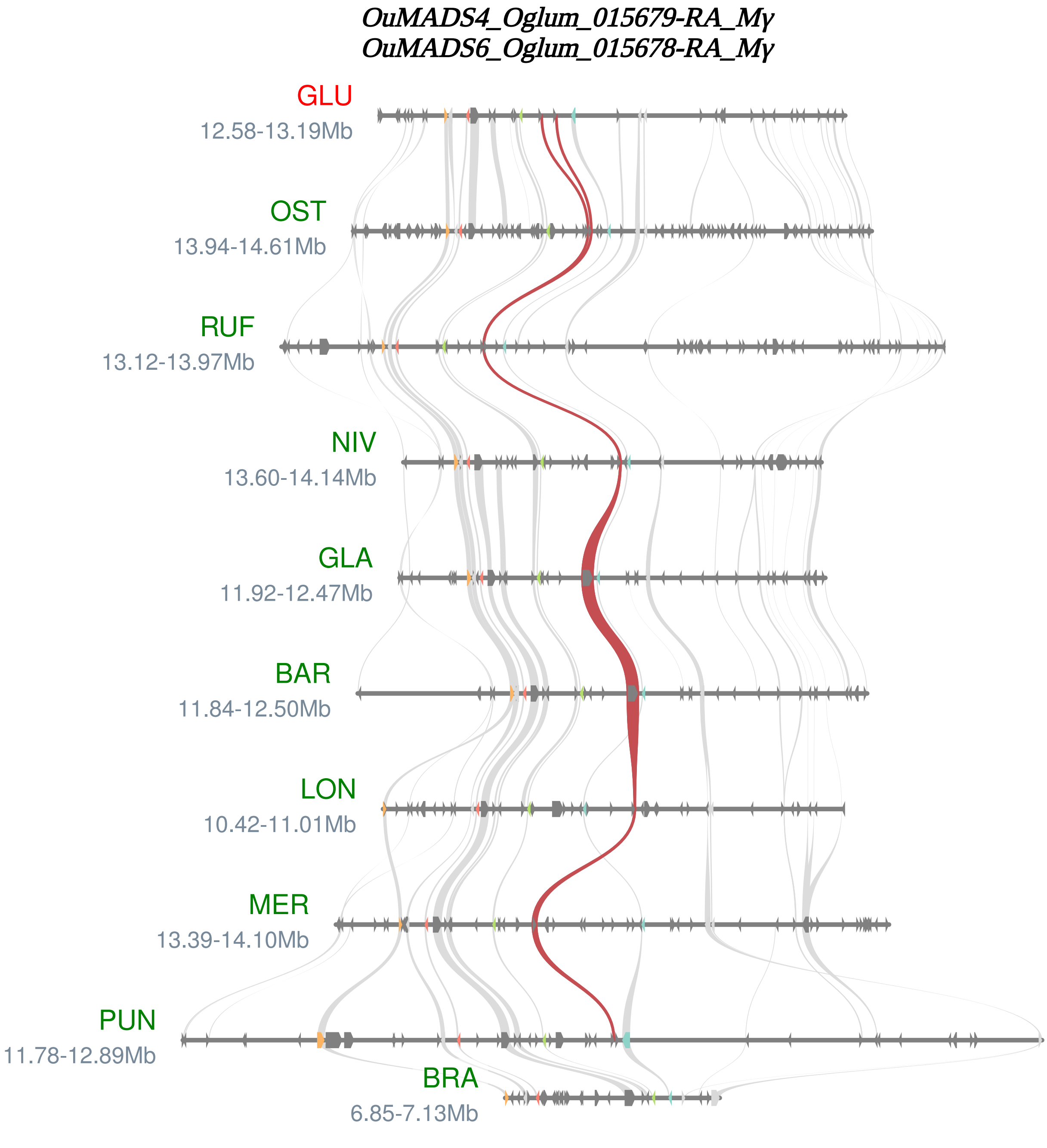

OuMADS4_Oglum_015679-RA_Mγ
OuMADS6_Oglum_015678-RA_Mγ
GLU
12.58-13.19Mb
OST
13.94-14.61Mb
RUF
13.12-13.97Mb
NIV
13.60-14.14Mb
GLA
11.92-12.47Mb
BAR
11.84-12.50Mb
LON
10.42-11.01Mb
MER
13.39-14.10Mb
PUN
11.78-12.89Mb
BRA
6.85-7.13Mb

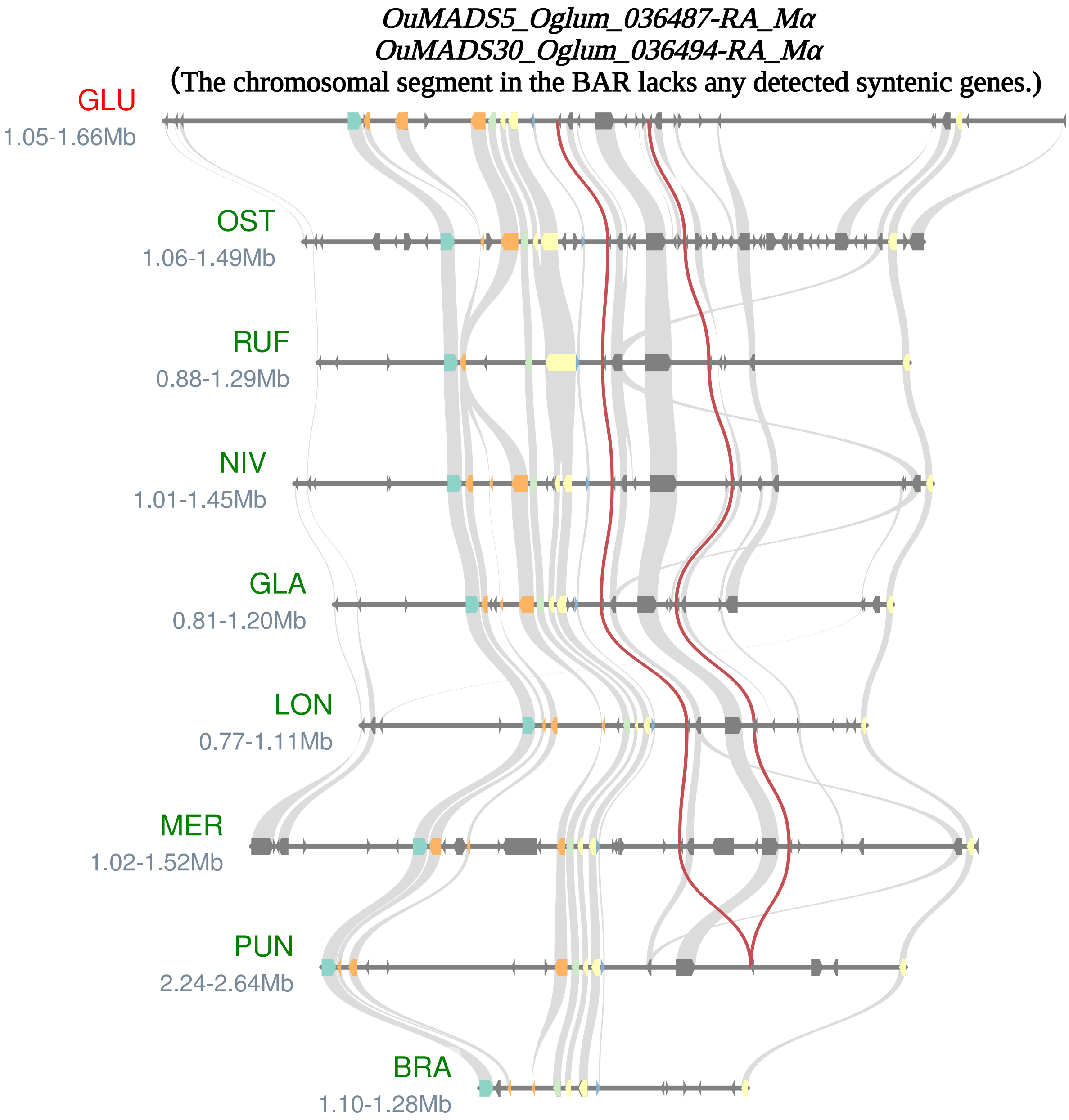
OuMADS5_Oglum_036487-RA_Mα
OuMADS30_Oglum_036494-RA_Mα
(The chromosomal segment in the BAR lacks any detected syntenic genes.)
GLU
1.05-1.66Mb
OST
1.06-1.49Mb
RUF
0.88-1.29Mb
NIV
1.01-1.45Mb
GLA
0.81-1.20Mb
LON
0.77-1.11Mb
MER
1.02-1.52Mb
PUN
2.24-2.64Mb
BRA
1.10-1.28Mb

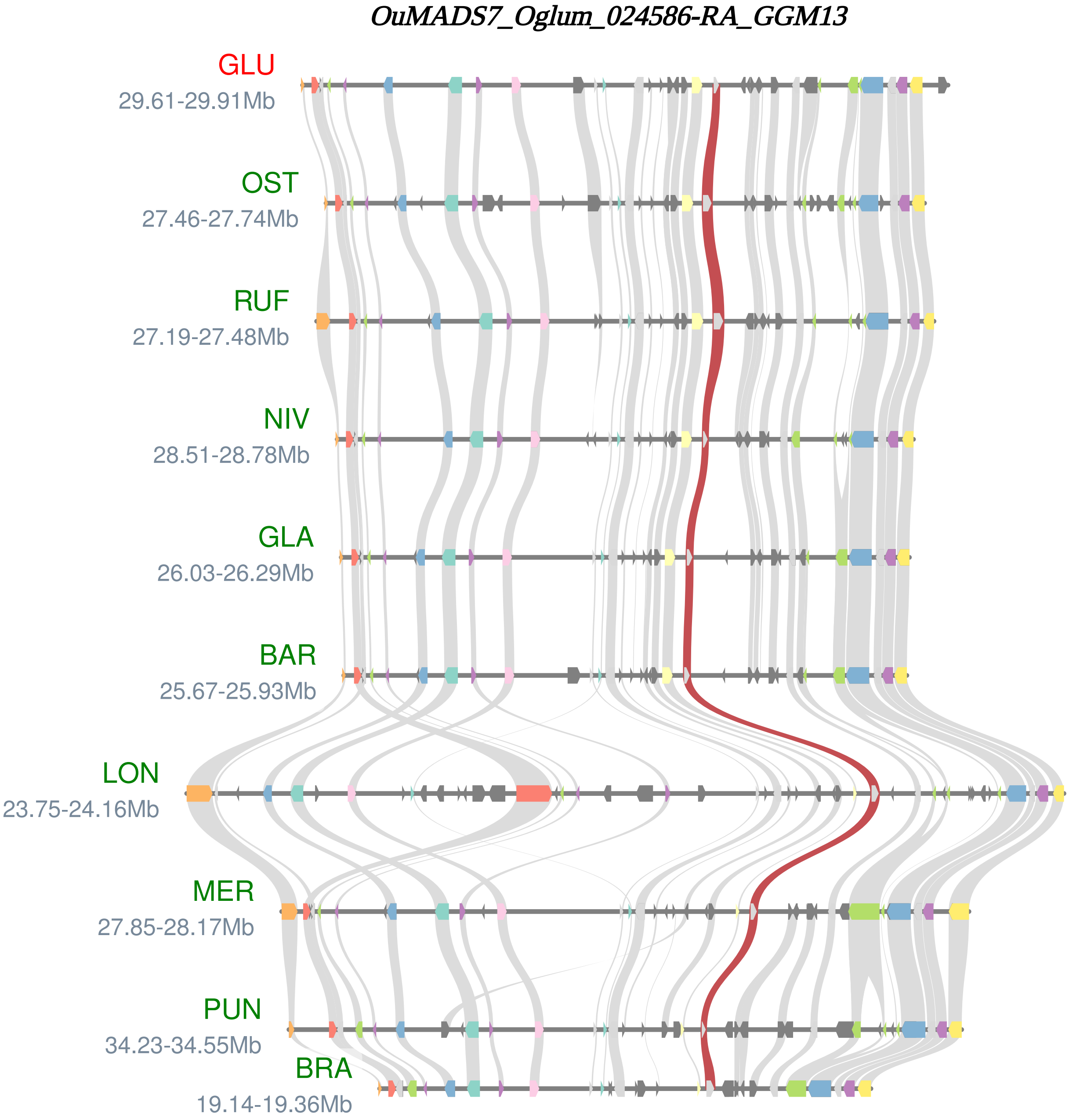

OuMADS7_Oglum_024586-RA_GGM13
GLU
29.61-29.91Mb
OST
27.46-27.74Mb
RUF
27.19-27.48Mb
NIV
28.51-28.78Mb
GLA
26.03-26.29Mb
BAR
25.67-25.93Mb
LON
23.75-24.16Mb
MER
27.85-28.17Mb
PUN
34.23-34.55Mb
BRA
19.14-19.36Mb

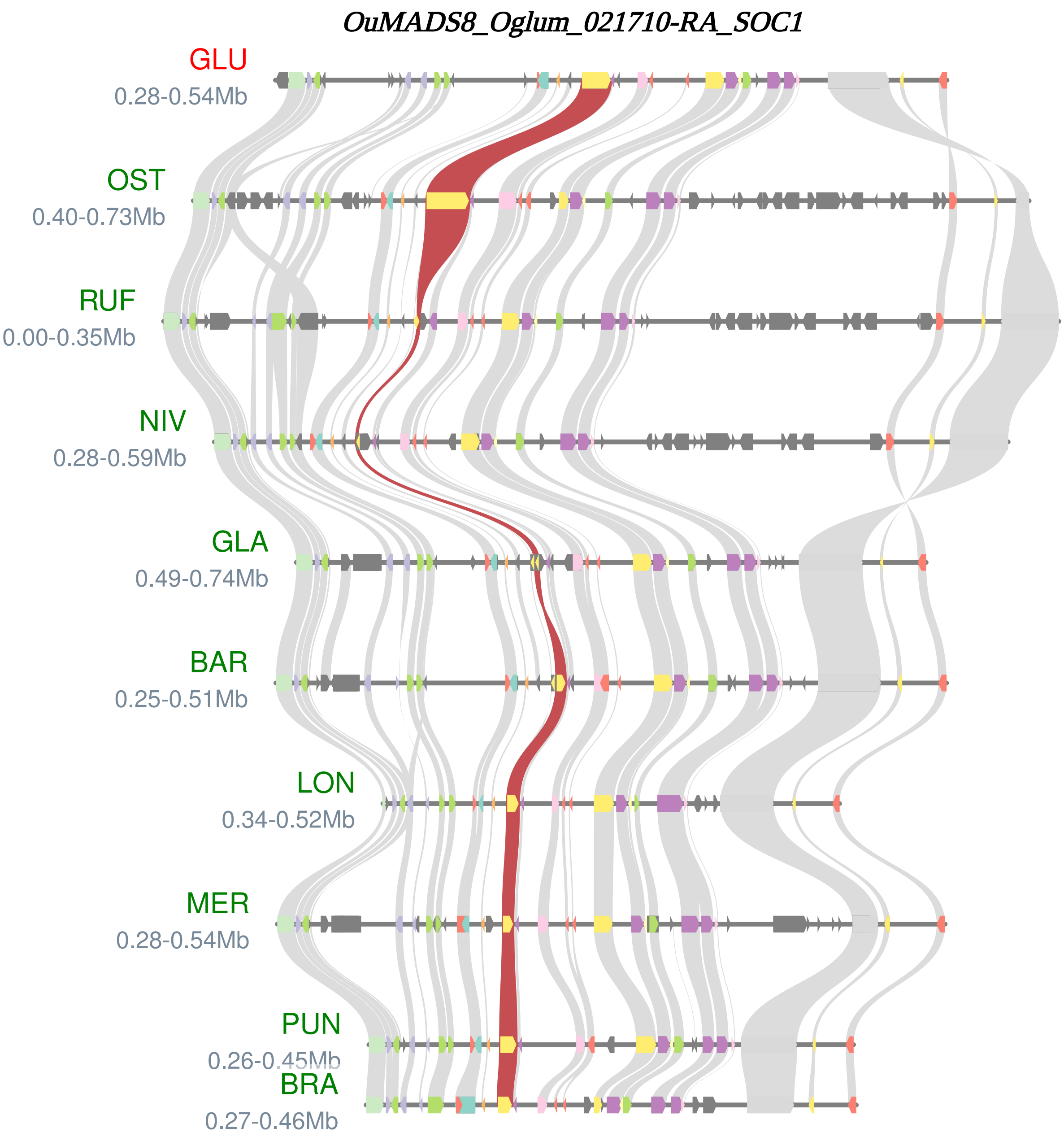

OuMADS8_Oglum_021710-RA_SOC1
GLU
0.28-0.54Mb
OST
0.40-0.73Mb
RUF
0.00-0.35Mb
NIV
0.28-0.59Mb
GLA
0.49-0.74Mb
BAR
0.25-0.51Mb
LON
0.34-0.52Mb
MER
0.28-0.54Mb
PUN
0.26-0.45Mb
BRA
0.27-0.46Mb

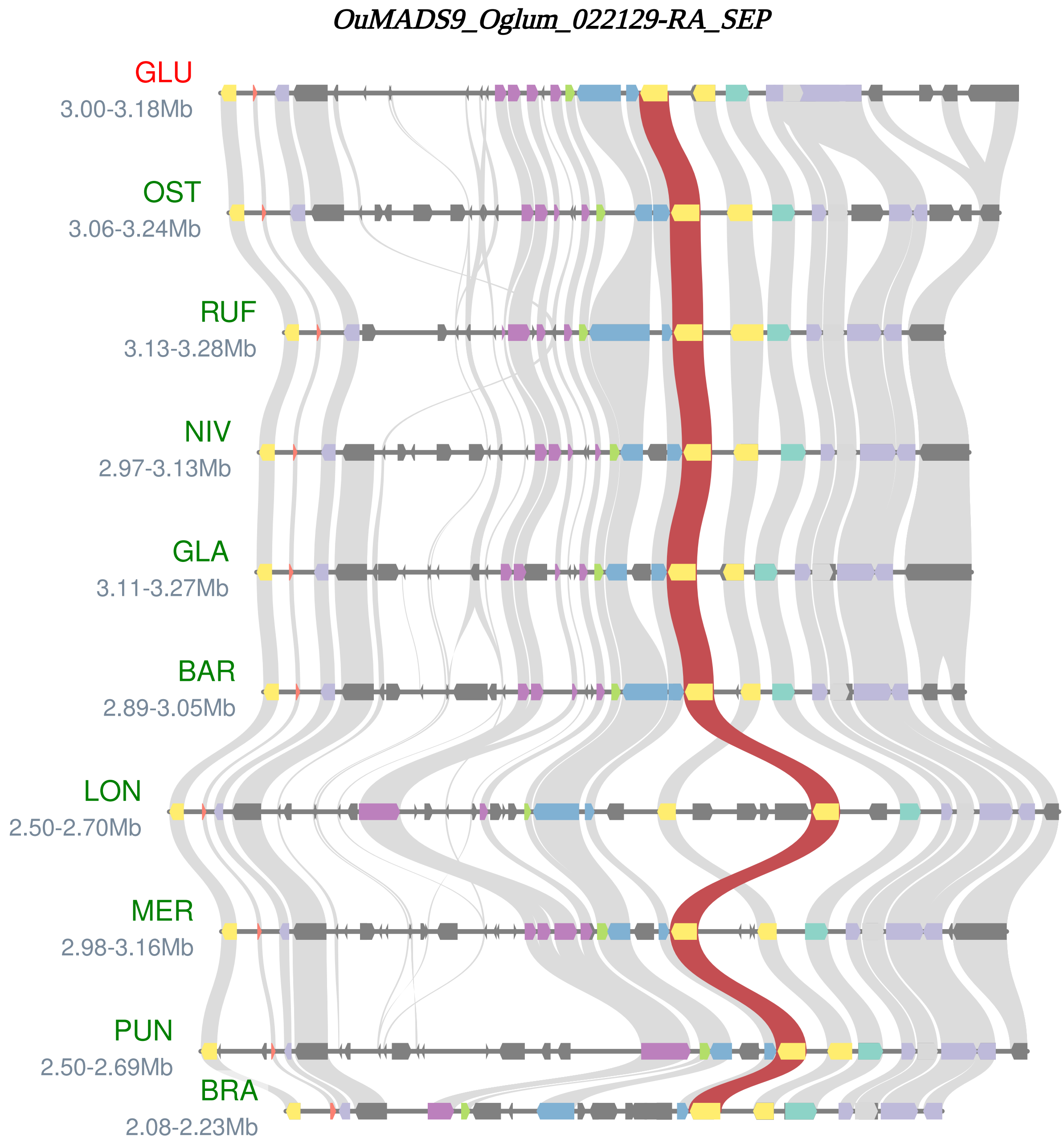

OuMADS9_Oglum_022129-RA_SEP
GLU
3.00-3.18Mb
OST
3.06-3.24Mb
RUF
3.13-3.28Mb
NIV
2.97-3.13Mb
GLA
3.11-3.27Mb
BAR
2.89-3.05Mb
LON
2.50-2.70Mb
MER
2.98-3.16Mb
PUN
2.50-2.69Mb
BRA
2.08-2.23Mb

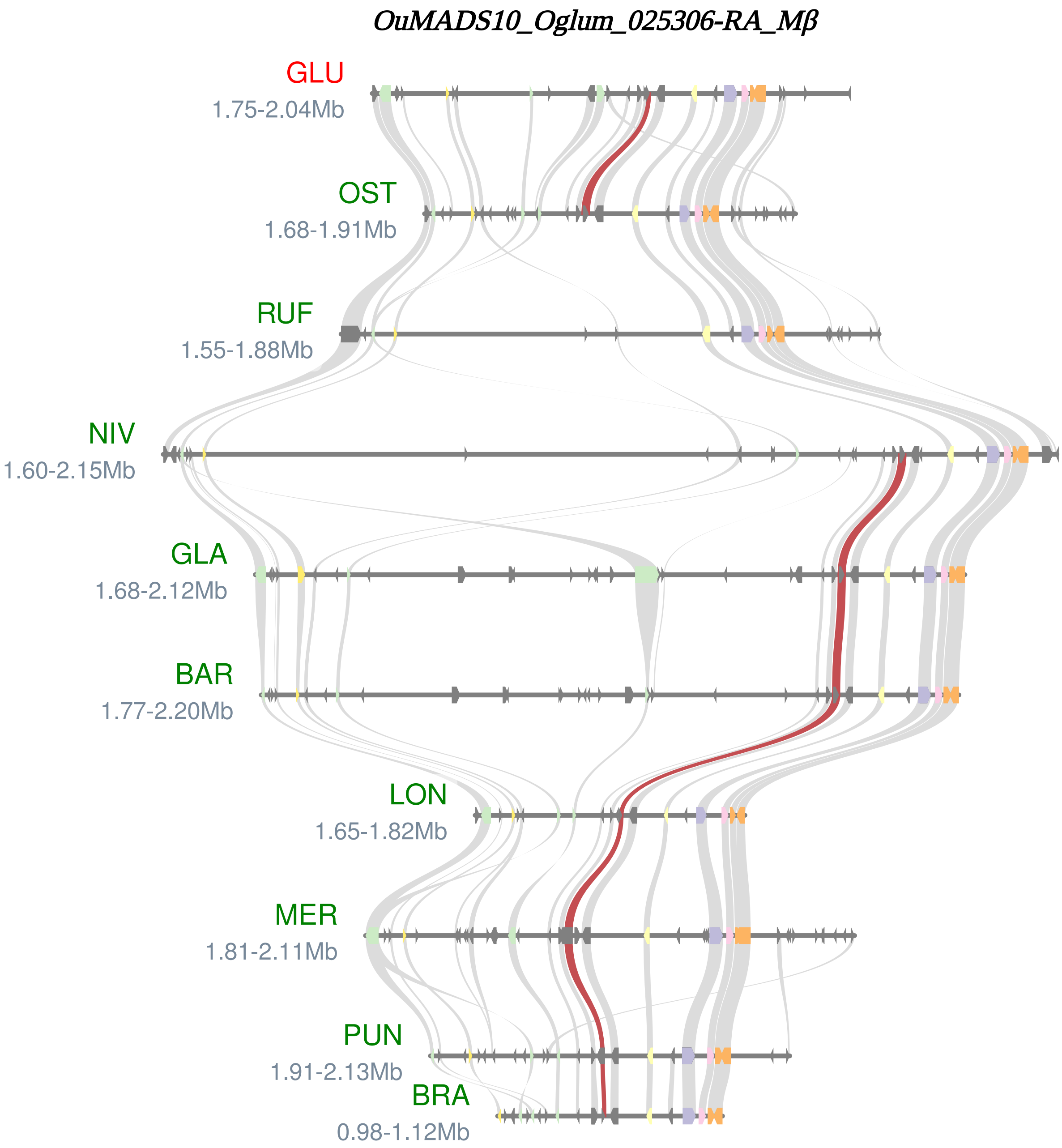
OuMADS10_Oglum_025306-RA_Mβ
GLU
1.75-2.04Mb
OST
1.68-1.91Mb
RUF
1.55-1.88Mb
NIV
1.60-2.15Mb
GLA
1.68-2.12Mb
BAR
1.77-2.20Mb
LON
1.65-1.82Mb
MER
1.81-2.11Mb
PUN
1.91-2.13Mb
BRA
0.98-1.12Mb

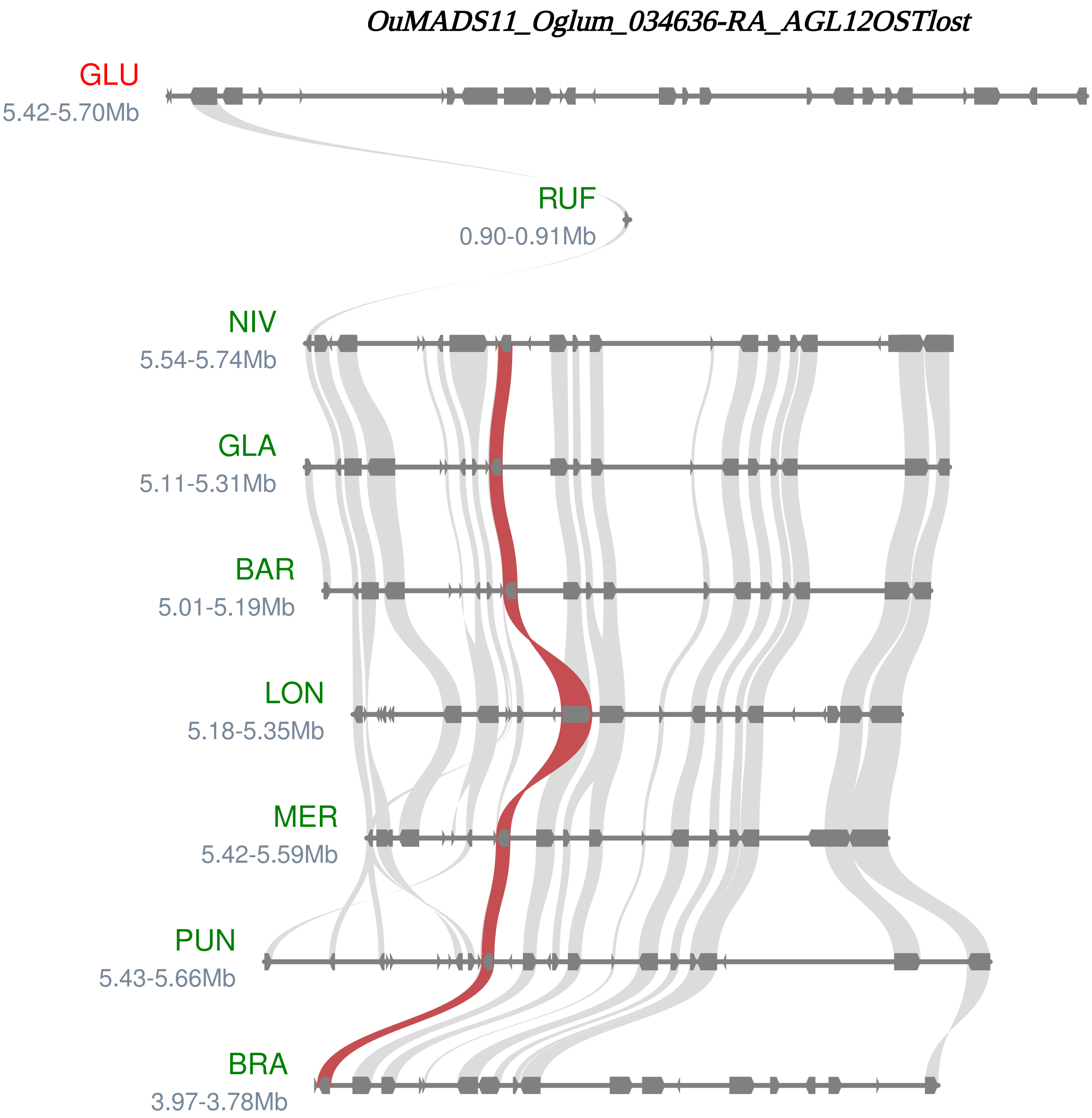

OuMADS11_Oglum_034636-RA_AGL12OSTlost
GLU
5.42-5.70Mb
RUF
0.90-0.91Mb
NIV
5.54-5.74Mb
GLA
5.11-5.31Mb
BAR
5.01-5.19Mb
LON
5.18-5.35Mb
MER
5.42-5.59Mb
PUN
5.43-5.66Mb
BRA
3.97-3.78Mb

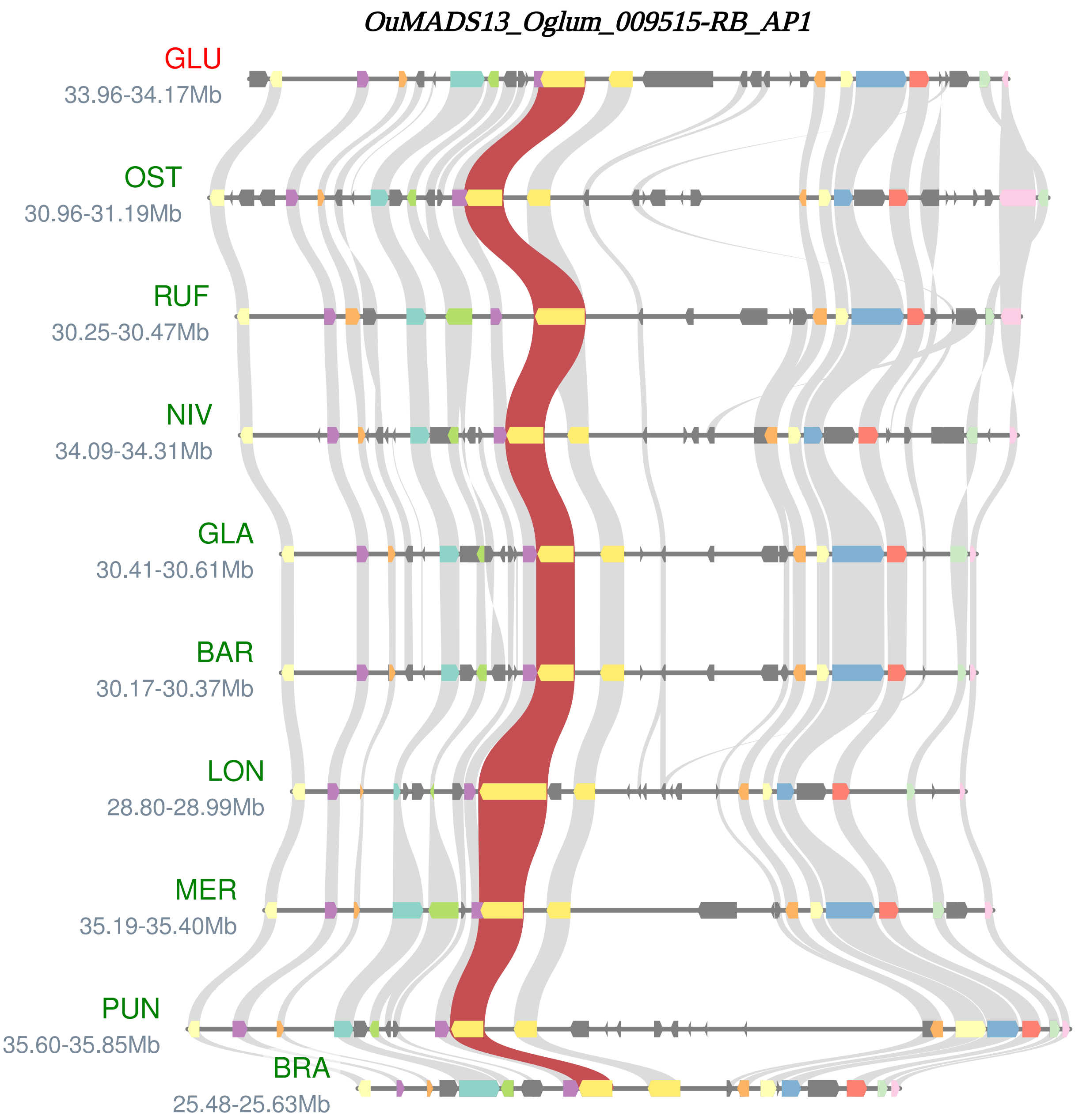

OuMADS13_Oglum_009515-RB_AP1
GLU
33.96-34.17Mb
OST
30.96-31.19Mb
RUF
30.25-30.47Mb
NIV
34.09-34.31Mb
GLA
30.41-30.61Mb
BAR
30.17-30.37Mb
LON
28.80-28.99Mb
MER
35.19-35.40Mb
PUN
35.60-35.85Mb
BRA
25.48-25.63Mb

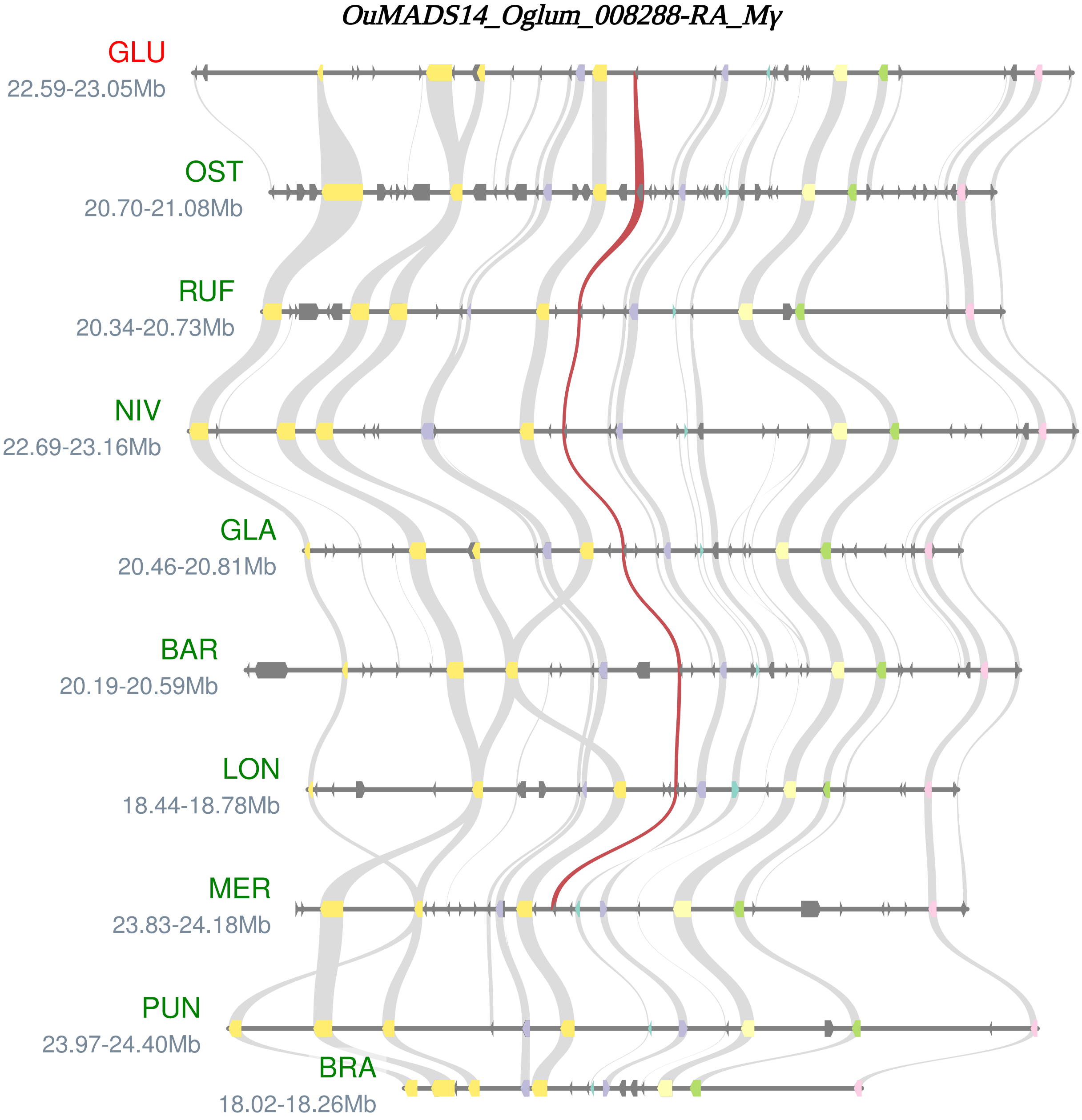
OuMADS14_Oglum_008288-RA_Mγ
GLU
22.59-23.05Mb
OST
20.70-21.08Mb
RUF
20.34-20.73Mb
NIV
22.69-23.16Mb
GLA
20.46-20.81Mb
BAR
20.19-20.59Mb
LON
18.44-18.78Mb
MER
23.83-24.18Mb
PUN
23.97-24.40Mb
BRA
18.02-18.26Mb

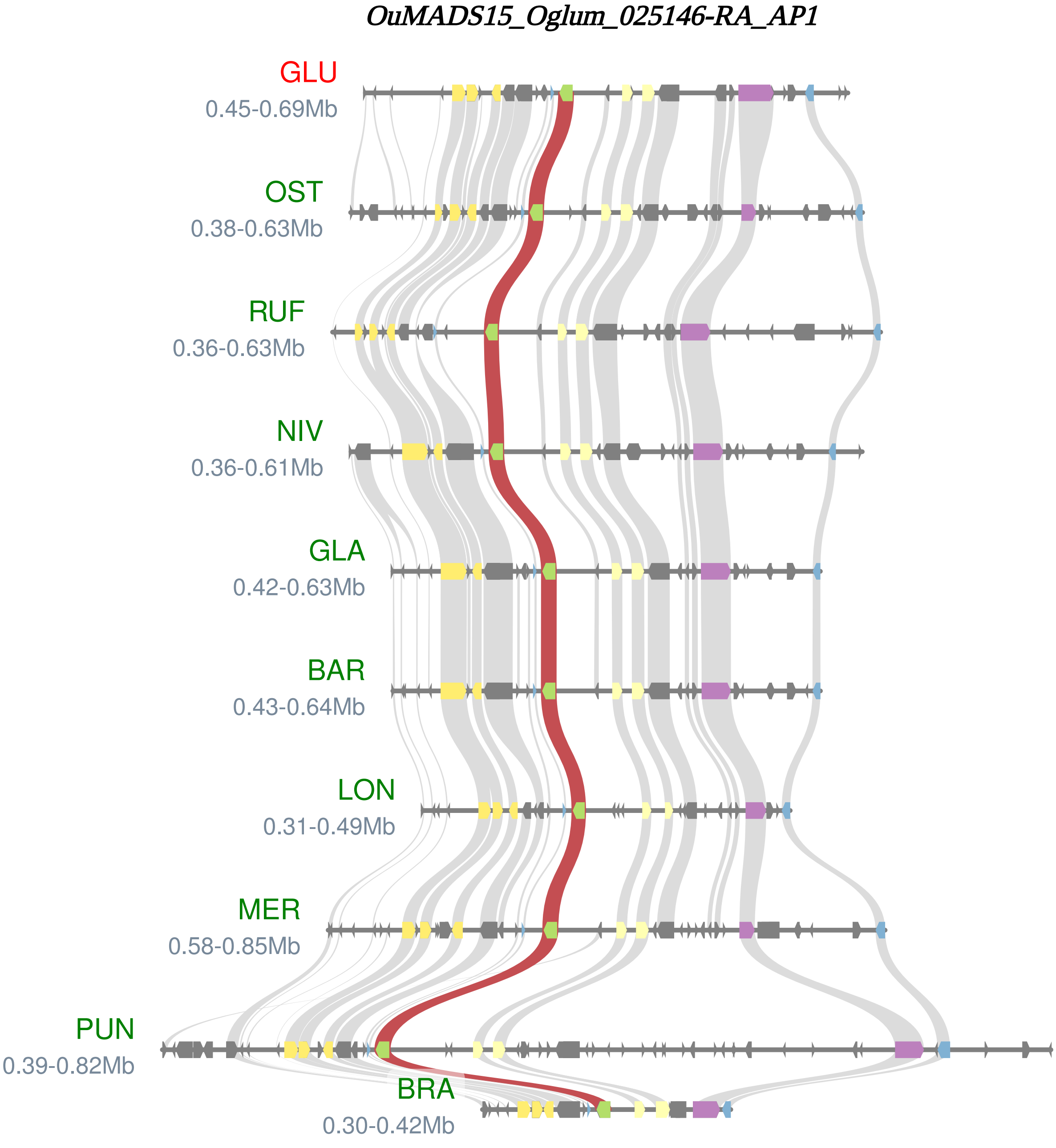
OuMADS15_Oglum_025146-RA_AP1
GLU
0.45-0.69Mb
OST
0.38-0.63Mb
RUF
0.36-0.63Mb
NIV
0.36-0.61Mb
GLA
0.42-0.63Mb
BAR
0.43-0.64Mb
LON
0.31-0.49Mb
MER
0.58-0.85Mb
PUN
0.39-0.82Mb
BRA
0.30-0.42Mb

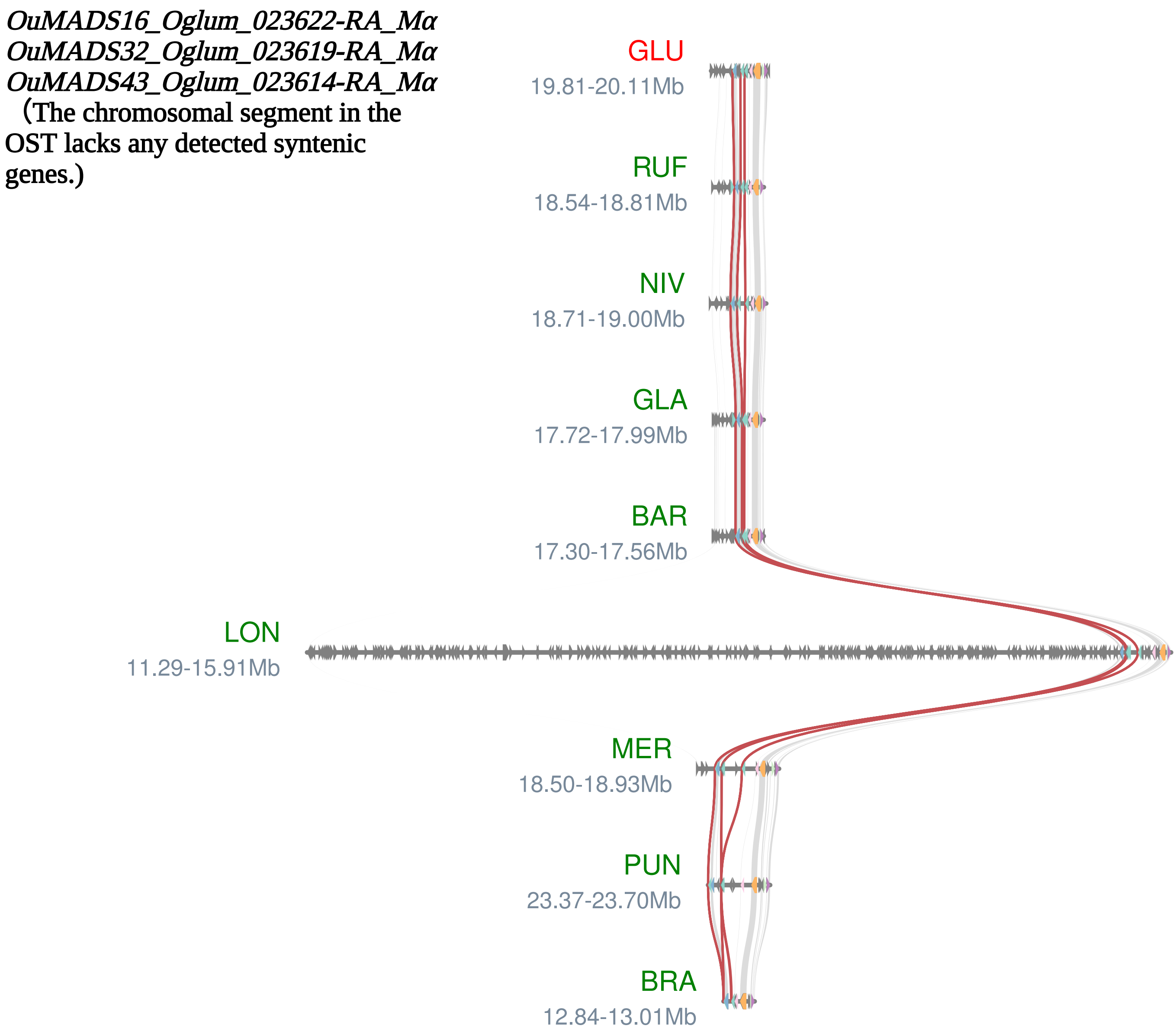

OuMADS16_Oglum_023622-RA_Mα
OuMADS32_Oglum_023619-RA_Mα
OuMADS43_Oglum_023614-RA_Mα
（The chromosomal segment in the OST lacks any detected syntenic genes.)
GLU
19.81-20.11Mb
RUF
18.54-18.81Mb
NIV
18.71-19.00Mb
GLA
17.72-17.99Mb
BAR
17.30-17.56Mb
LON
11.29-15.91Mb
MER
18.50-18.93Mb
PUN
23.37-23.70Mb
BRA
12.84-13.01Mb

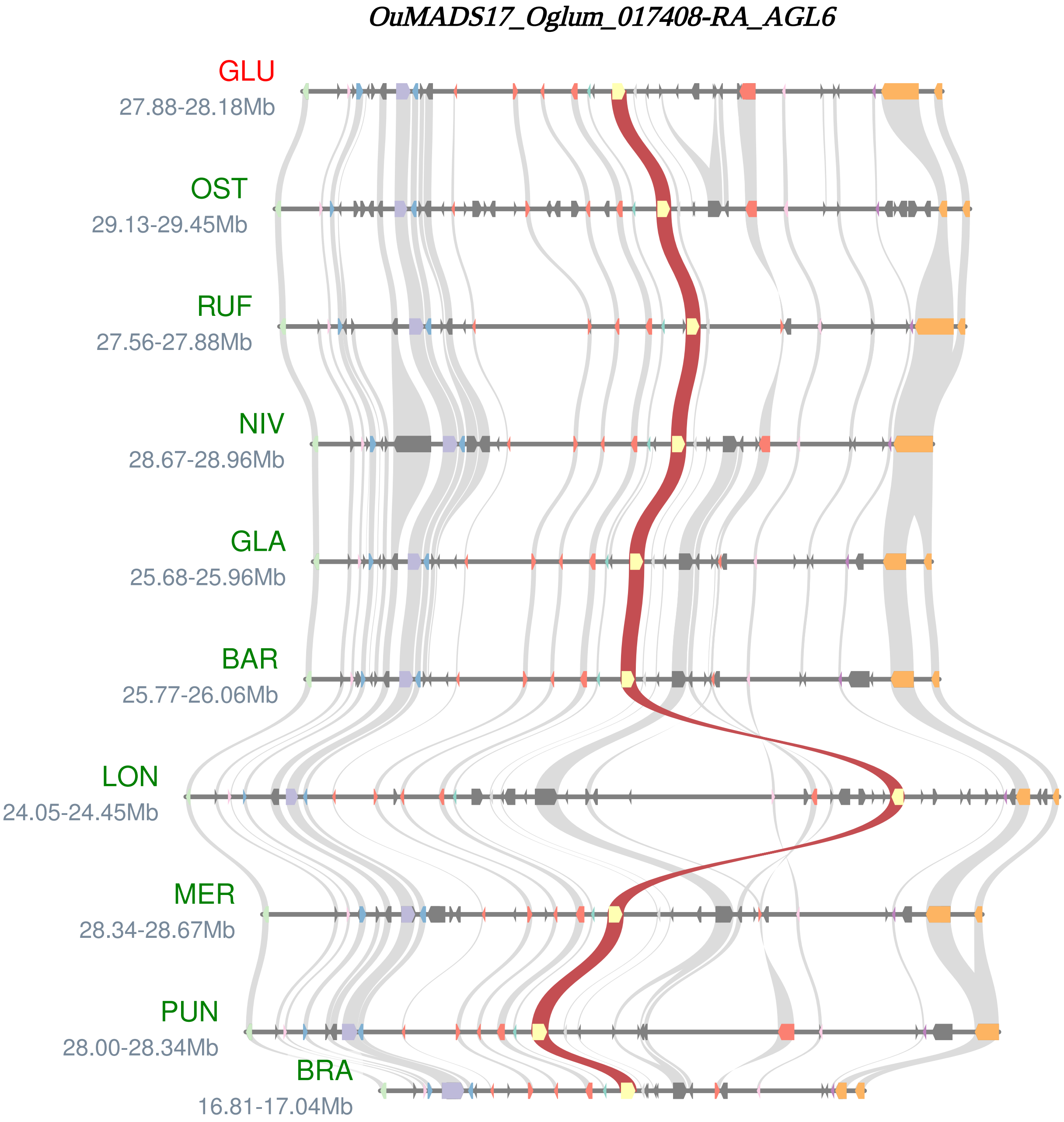
OuMADS17_Oglum_017408-RA_AGL6
GLU
27.88-28.18Mb
OST
29.13-29.45Mb
RUF
27.56-27.88Mb
NIV
28.67-28.96Mb
GLA
25.68-25.96Mb
BAR
25.77-26.06Mb
LON
24.05-24.45Mb
MER
28.34-28.67Mb
PUN
28.00-28.34Mb
BRA
16.81-17.04Mb

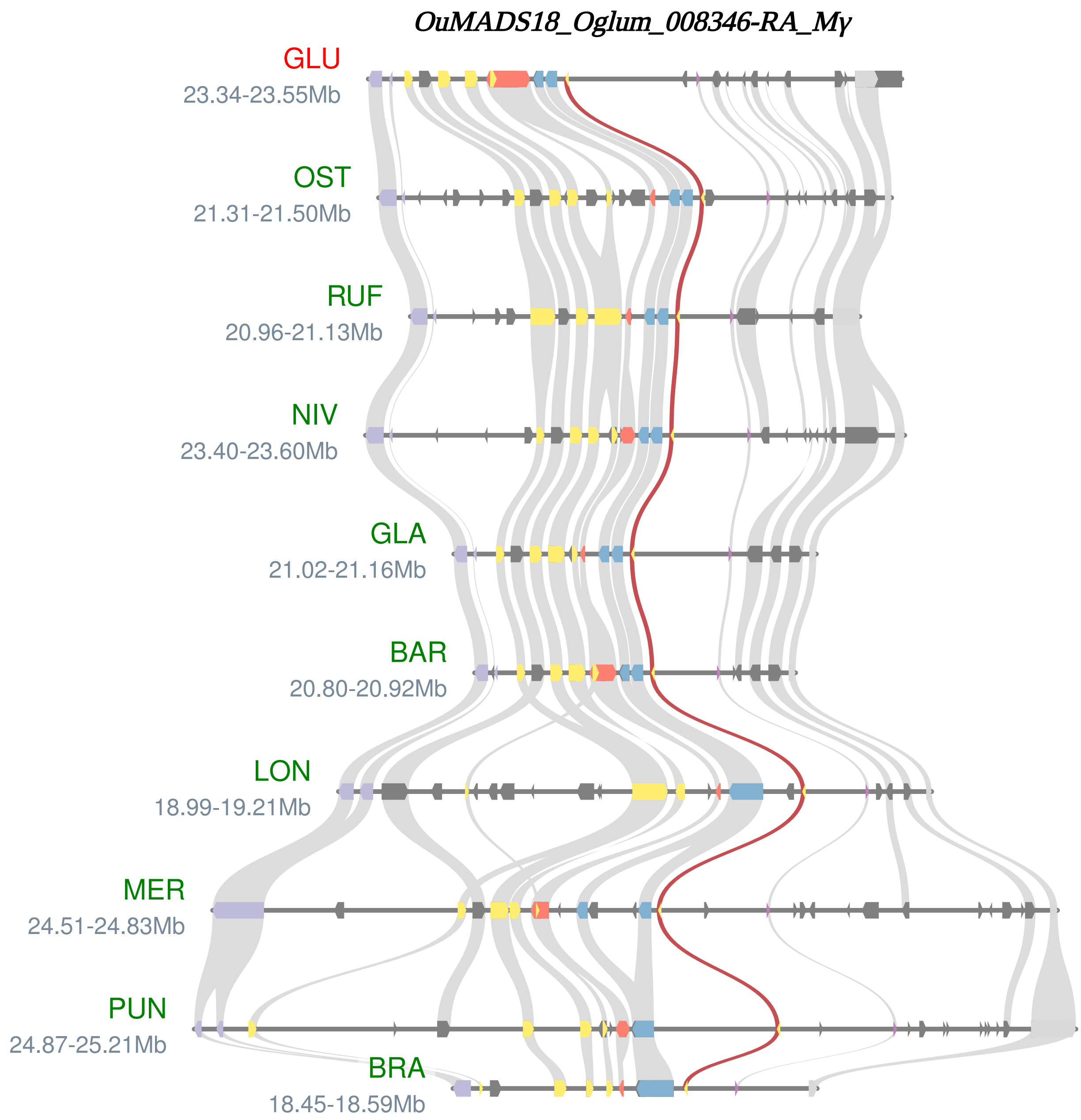

OuMADS18_Oglum_008346-RA_Mγ
GLU
23.34-23.55Mb
OST
21.31-21.50Mb
RUF
20.96-21.13Mb
NIV
23.40-23.60Mb
GLA
21.02-21.16Mb
BAR
20.80-20.92Mb
LON
18.99-19.21Mb
MER
24.51-24.83Mb
PUN
24.87-25.21Mb
BRA
18.45-18.59Mb

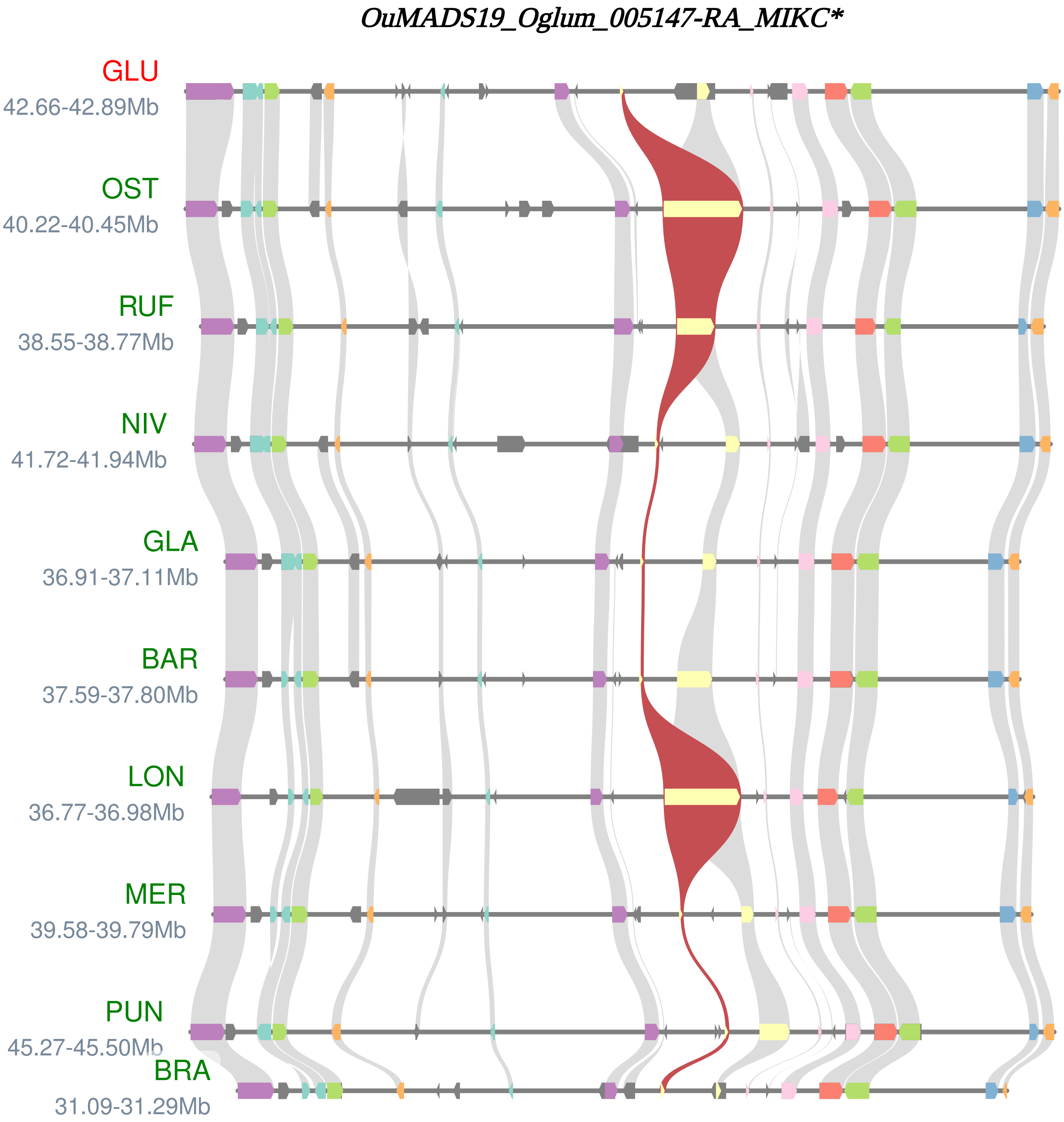
OuMADS19_Oglum_005147-RA_MIKC*
GLU
42.66-42.89Mb
OST
40.22-40.45Mb
RUF
38.55-38.77Mb
NIV
41.72-41.94Mb
GLA
36.91-37.11Mb
BAR
37.59-37.80Mb
LON
36.77-36.98Mb
MER
39.58-39.79Mb
PUN
45.27-45.50Mb
BRA
31.09-31.29Mb

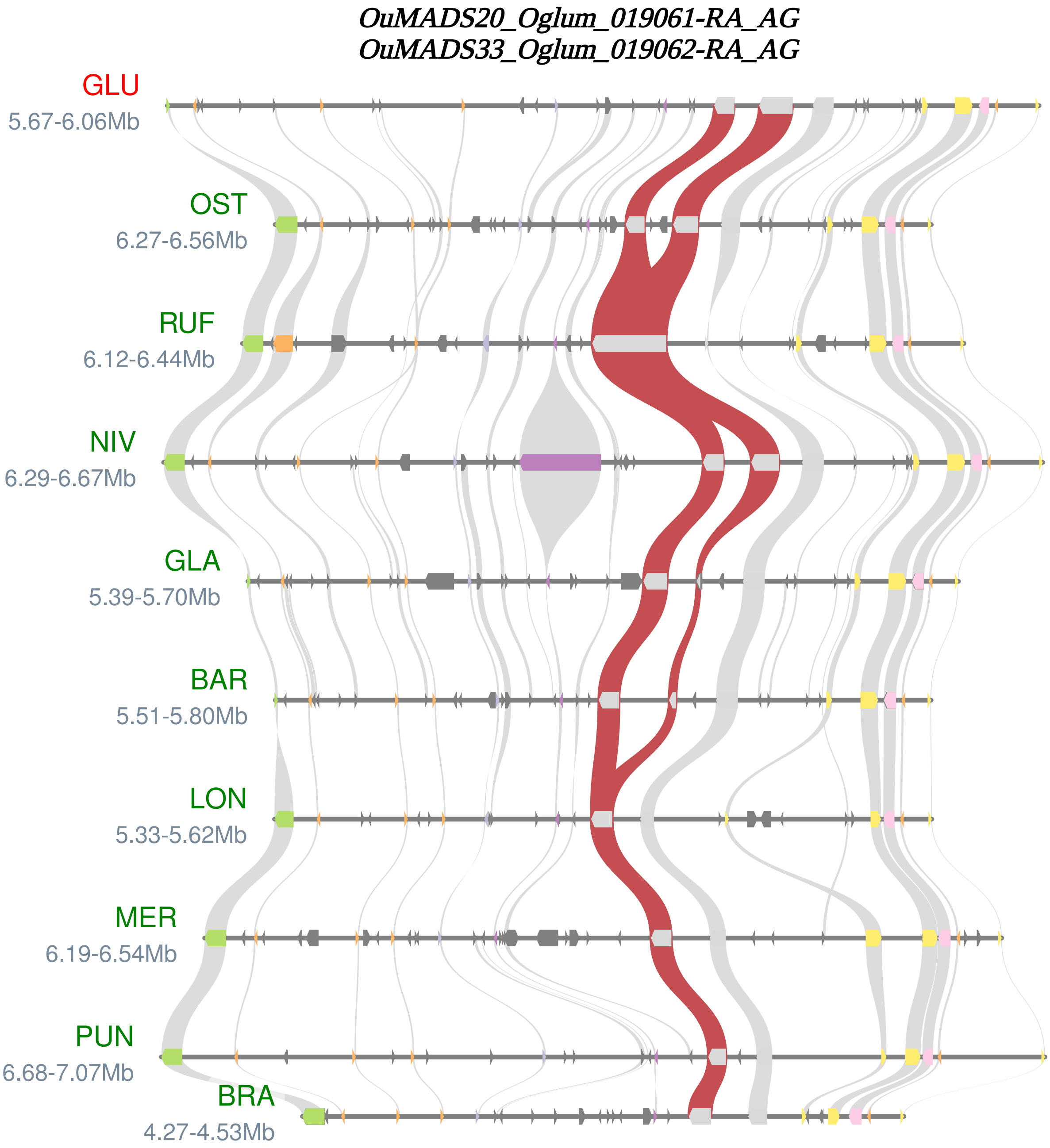
OuMADS20_Oglum_019061-RA_AG
OuMADS33_Oglum_019062-RA_AG
GLU
5.67-6.06Mb
OST
6.27-6.56Mb
RUF
6.12-6.44Mb
NIV
6.29-6.67Mb
GLA
5.39-5.70Mb
BAR
5.51-5.80Mb
LON
5.33-5.62Mb
MER
6.19-6.54Mb
PUN
6.68-7.07Mb
BRA
4.27-4.53Mb

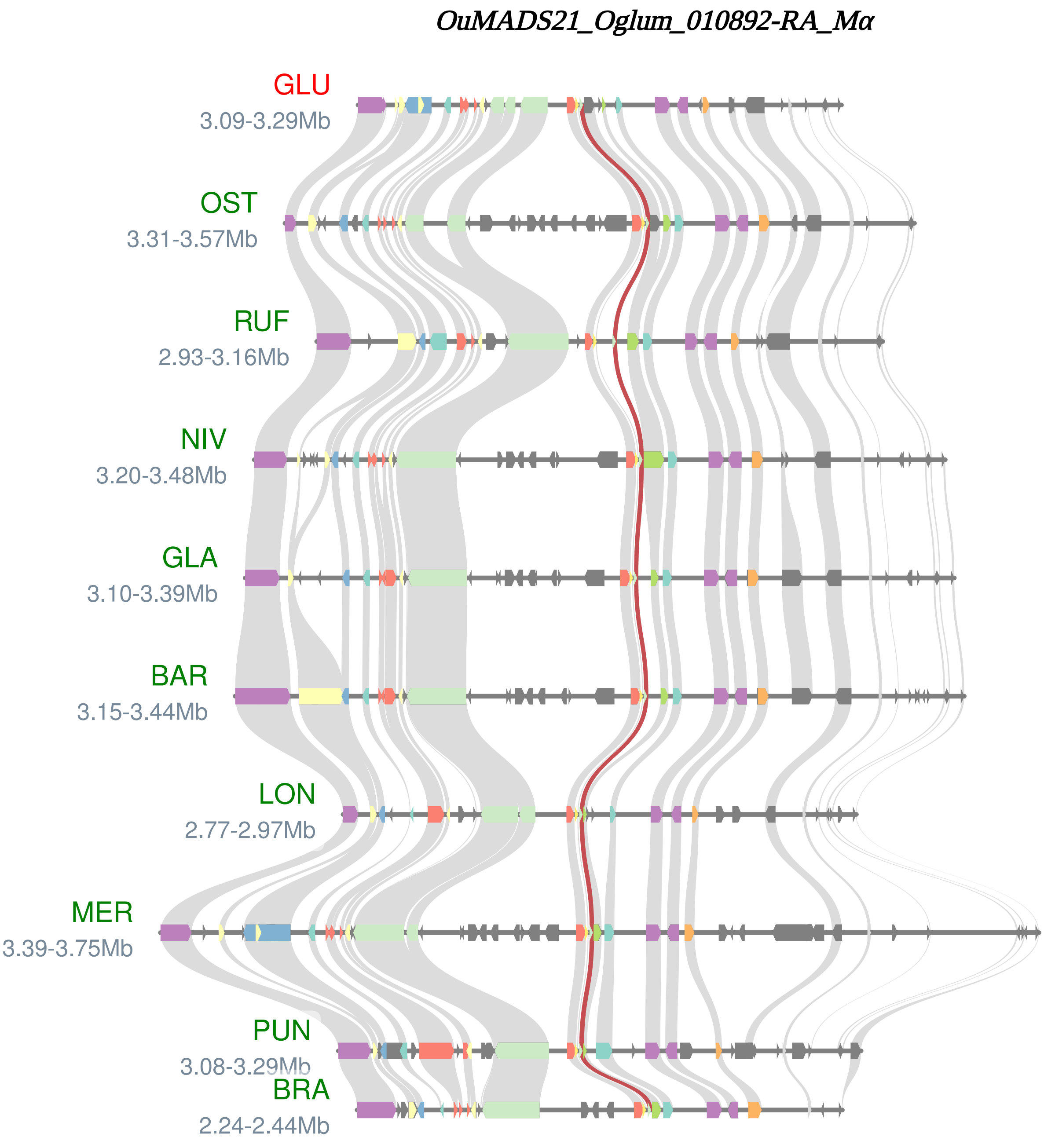

OuMADS21_Oglum_010892-RA_Mα
GLU
3.09-3.29Mb
OST
3.31-3.57Mb
RUF
2.93-3.16Mb
NIV
3.20-3.48Mb
GLA
3.10-3.39Mb
BAR
3.15-3.44Mb
LON
2.77-2.97Mb
MER
3.39-3.75Mb
PUN
3.08-3.29Mb
BRA
2.24-2.44Mb

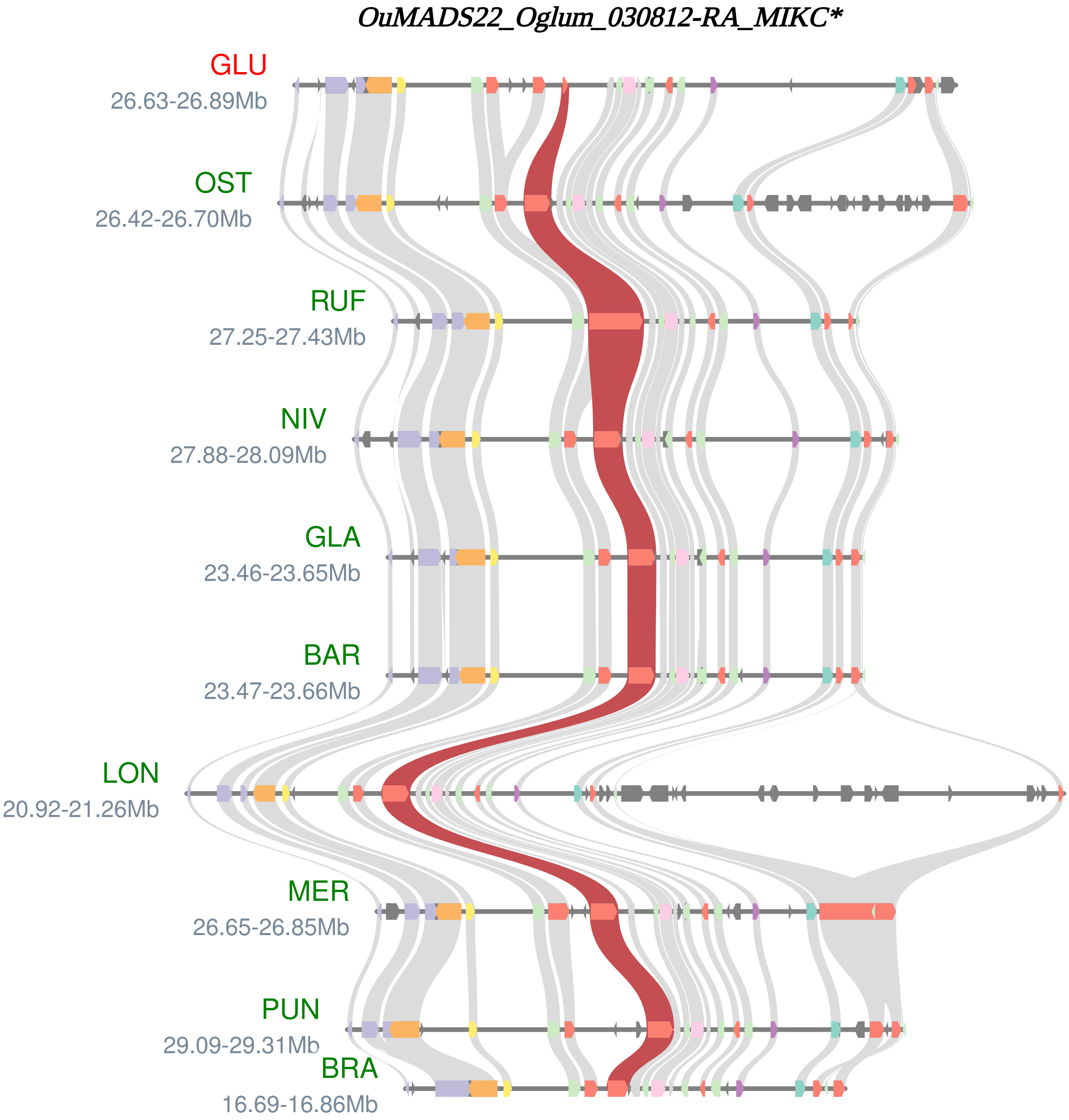
OuMADS22_Oglum_030812-RA_MIKC*
GLU
26.63-26.89Mb
OST
26.42-26.70Mb
RUF
27.25-27.43Mb
NIV
27.88-28.09Mb
GLA
23.46-23.65Mb
BAR
23.47-23.66Mb
LON
20.92-21.26Mb
MER
26.65-26.85Mb
PUN
29.09-29.31Mb
BRA
16.69-16.86Mb

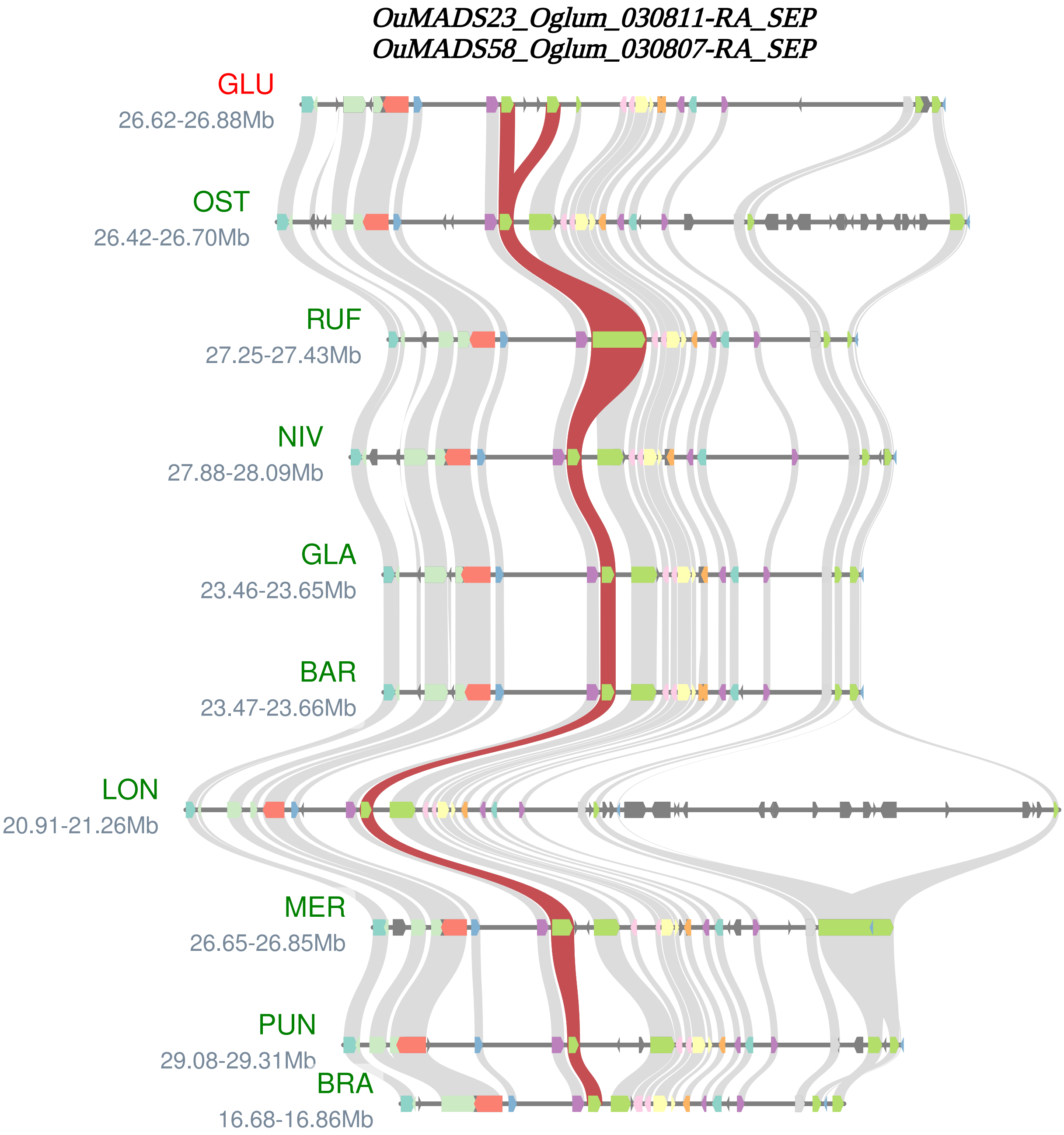
OuMADS23_Oglum_030811-RA_SEP
OuMADS58_Oglum_030807-RA_SEP
GLU
26.62-26.88Mb
OST
26.42-26.70Mb
RUF
27.25-27.43Mb
NIV
27.88-28.09Mb
GLA
23.46-23.65Mb
BAR
23.47-23.66Mb
LON
20.91-21.26Mb
MER
26.65-26.85Mb
PUN
29.08-29.31Mb
BRA
16.68-16.86Mb

OuMADS24_Oglum_034638-RA_AG
(The chromosomal segment in the OST lacks any detected syntenic genes.)

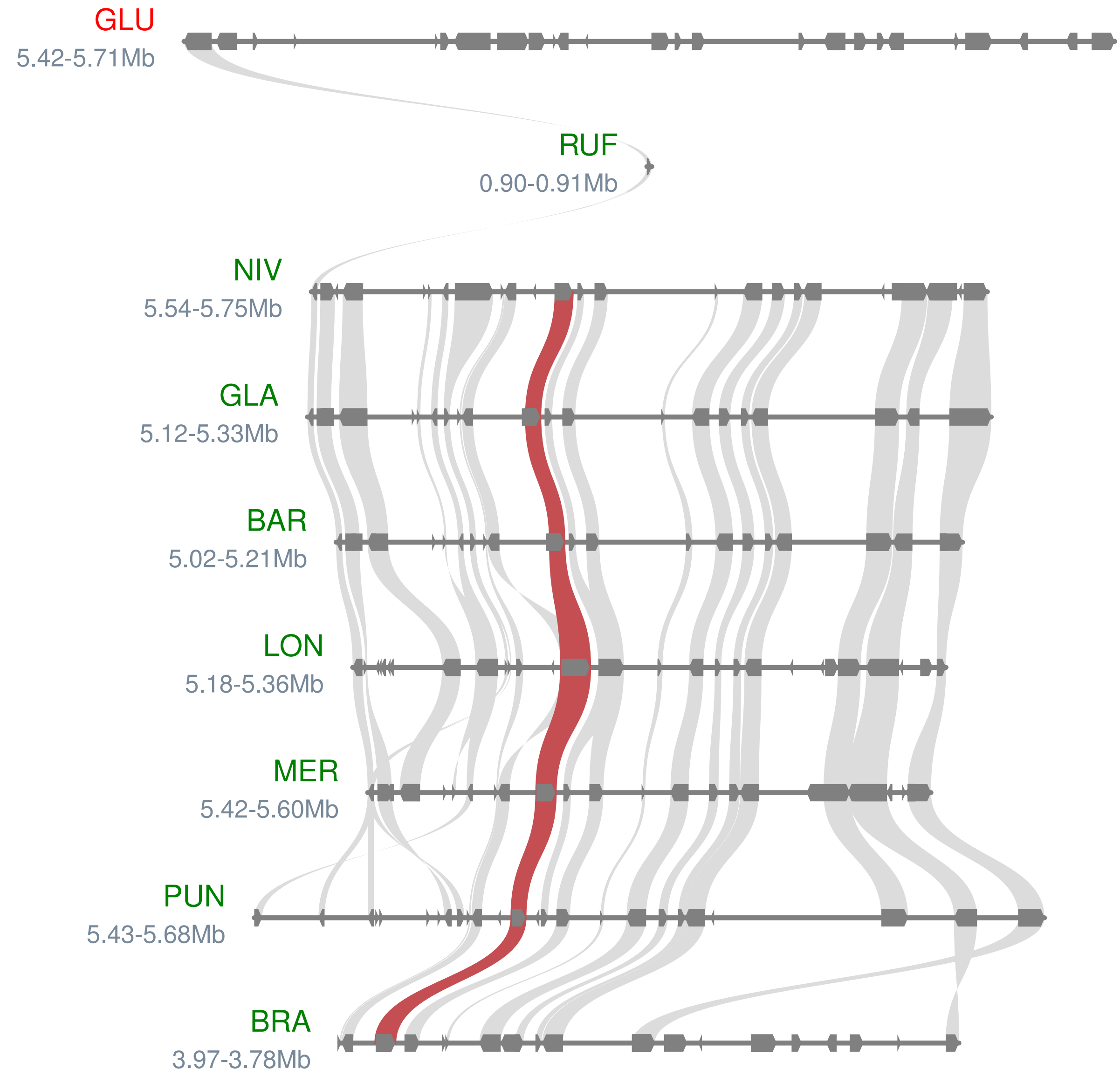

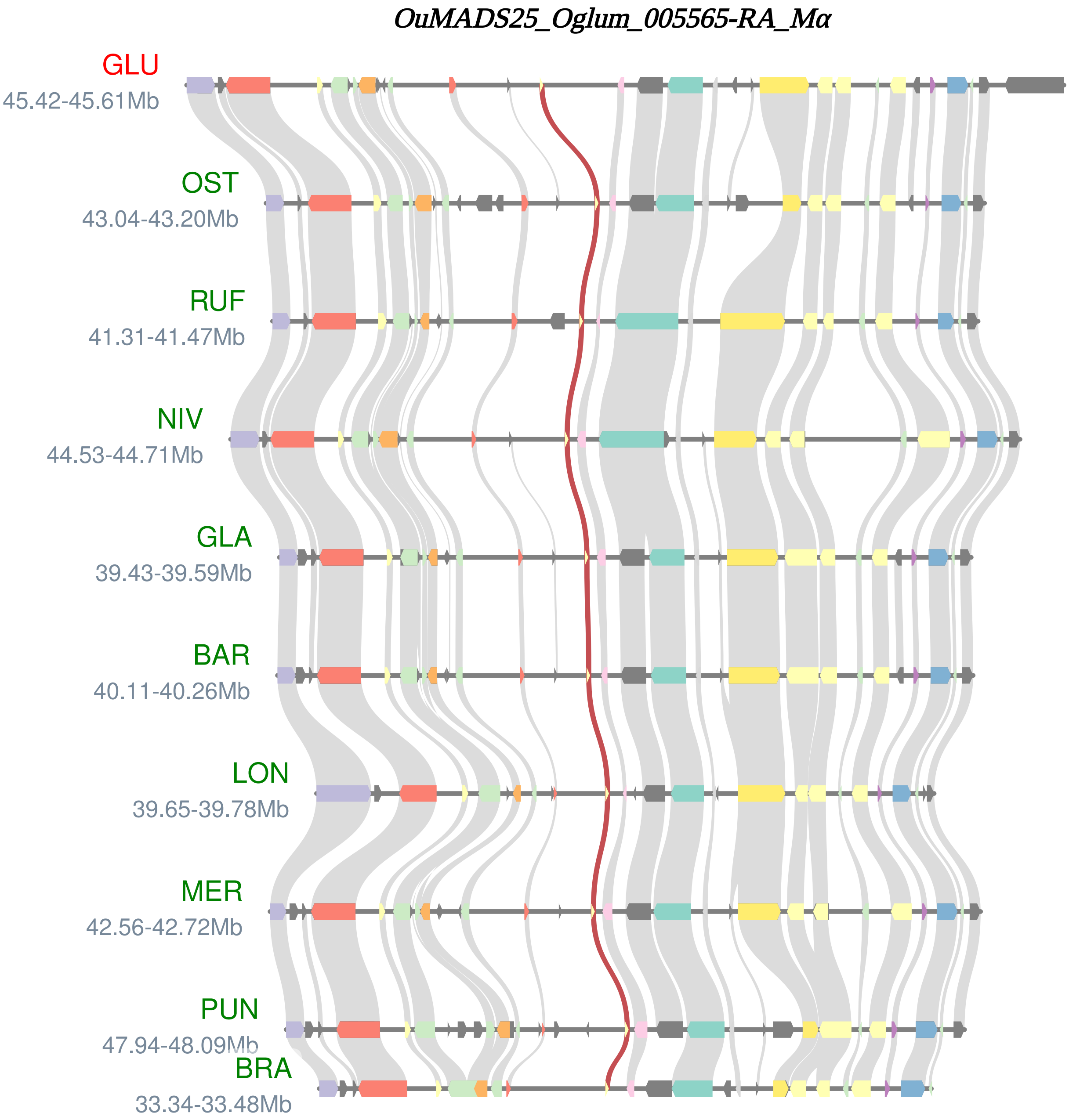

OuMADS25_Oglum_005565-RA_Mα
GLU
45.42-45.61Mb
OST
43.04-43.20Mb
RUF
41.31-41.47Mb
NIV
44.53-44.71Mb
GLA
39.43-39.59Mb
BAR
40.11-40.26Mb
LON
39.65-39.78Mb
MER
42.56-42.72Mb
PUN
47.94-48.09Mb
BRA
33.34-33.48Mb

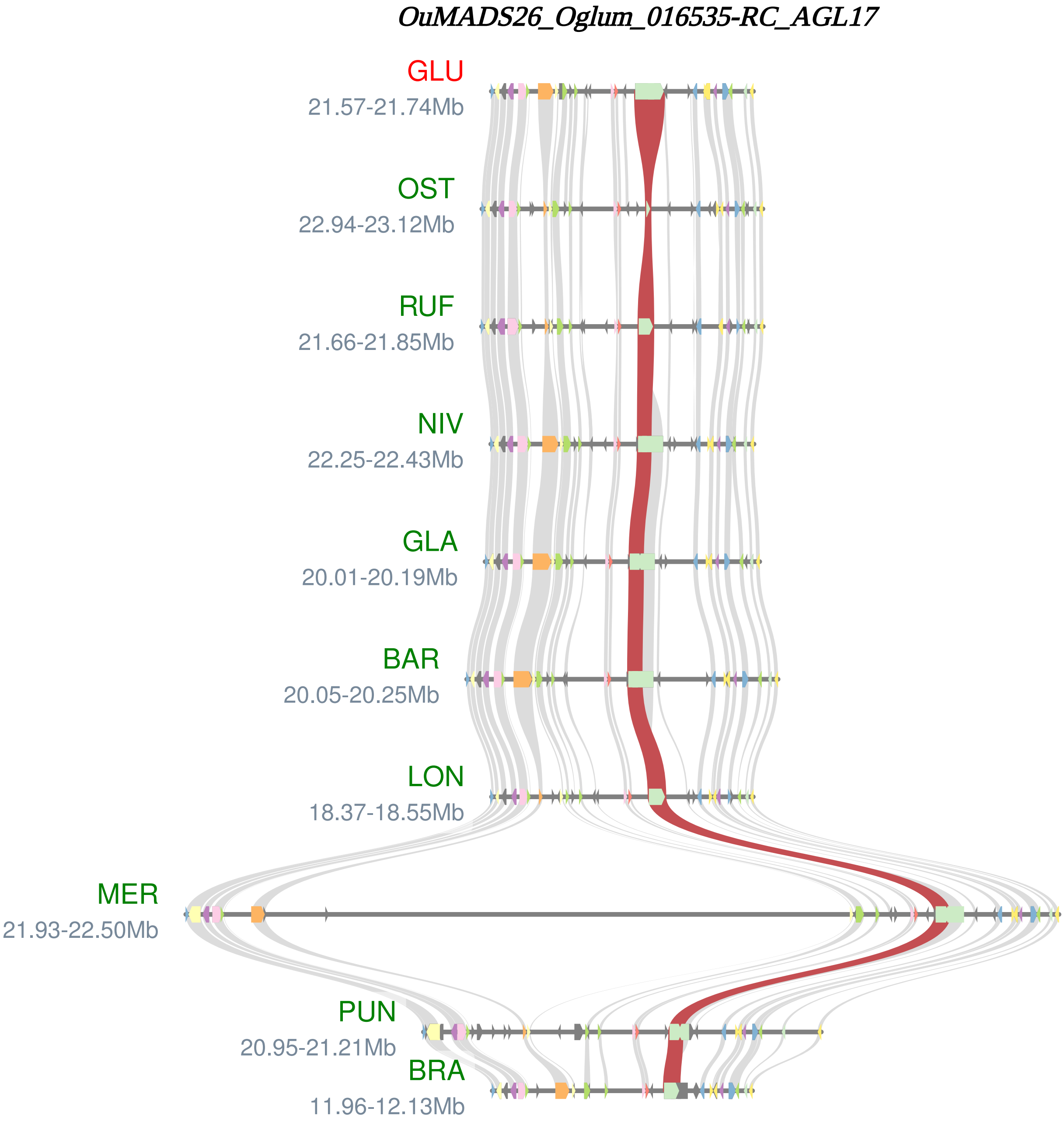

OuMADS26_Oglum_016535-RC_AGL17
GLU
21.57-21.74Mb
OST
22.94-23.12Mb
RUF
21.66-21.85Mb
NIV
22.25-22.43Mb
GLA
20.01-20.19Mb
BAR
20.05-20.25Mb
LON
18.37-18.55Mb
MER
21.93-22.50Mb
PUN
20.95-21.21Mb
BRA
11.96-12.13Mb

OuMADS27_Oglum_006280-RA_SVP
(The chromosomal segment in the GLA lacks any detected syntenic genes.)

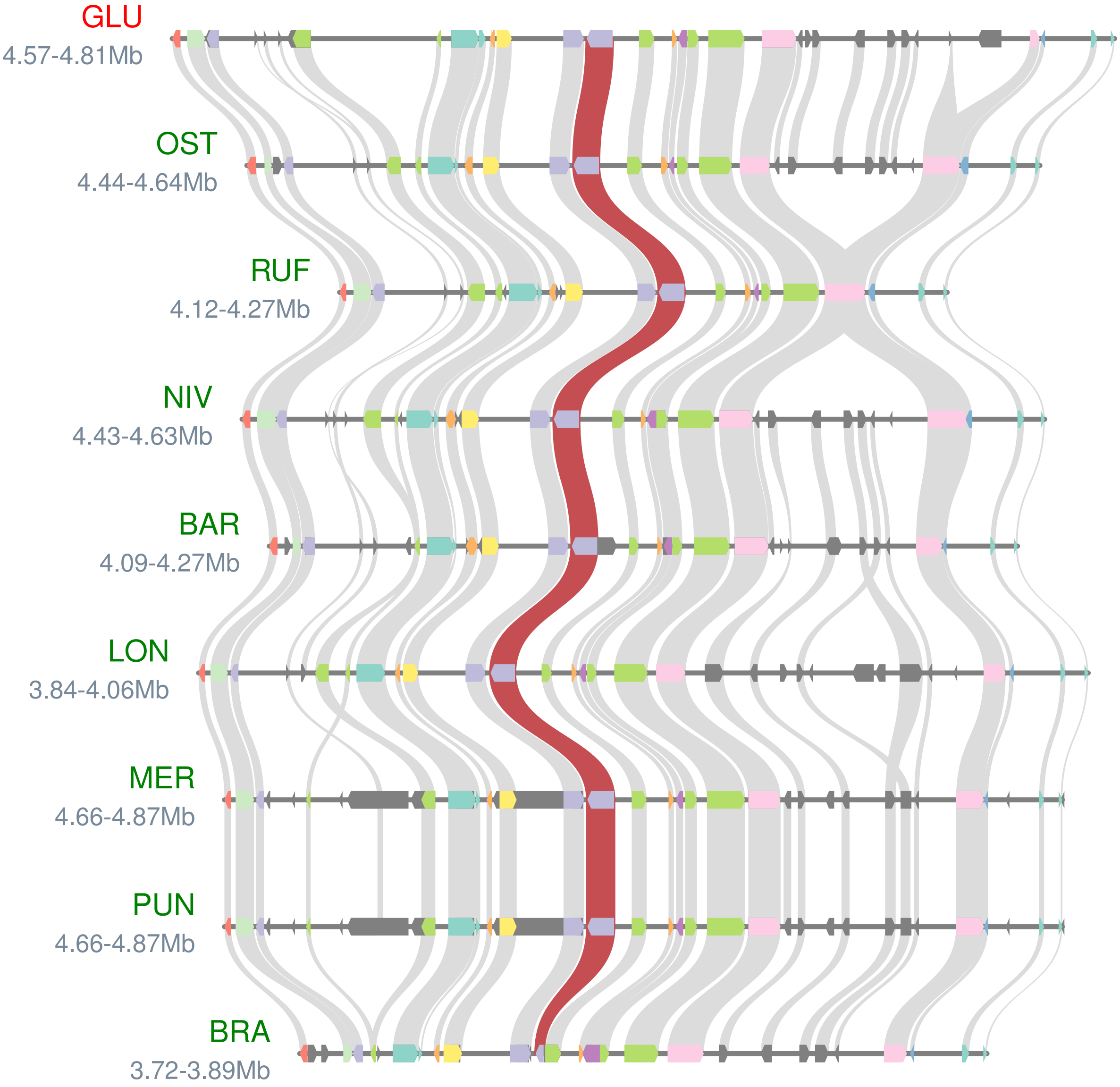

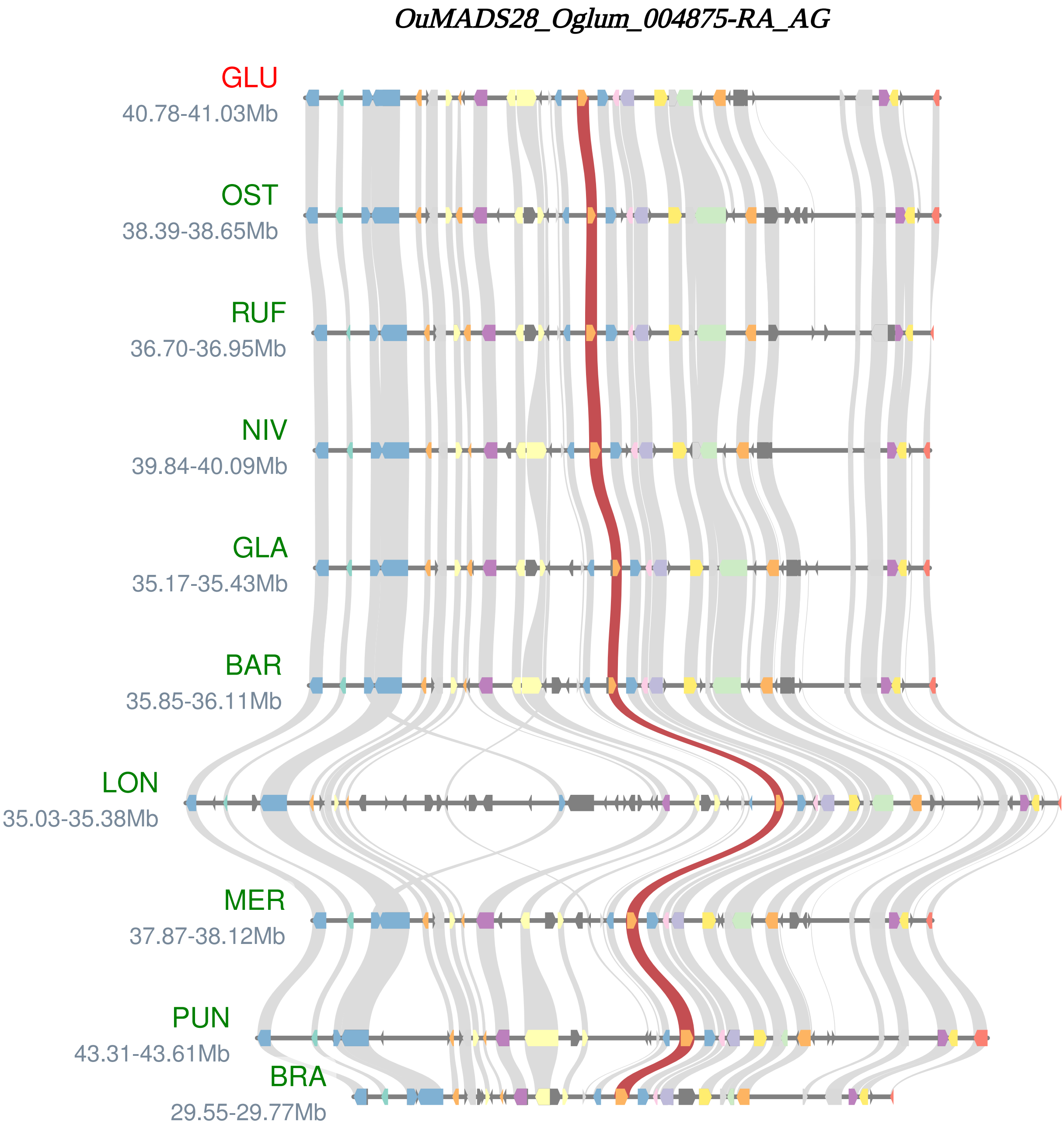

OuMADS28_Oglum_004875-RA_AG
GLU
40.78-41.03Mb
OST
38.39-38.65Mb
RUF
36.70-36.95Mb
NIV
39.84-40.09Mb
GLA
35.17-35.43Mb
BAR
35.85-36.11Mb
LON
35.03-35.38Mb
MER
37.87-38.12Mb
PUN
43.31-43.61Mb
BRA
29.55-29.77Mb

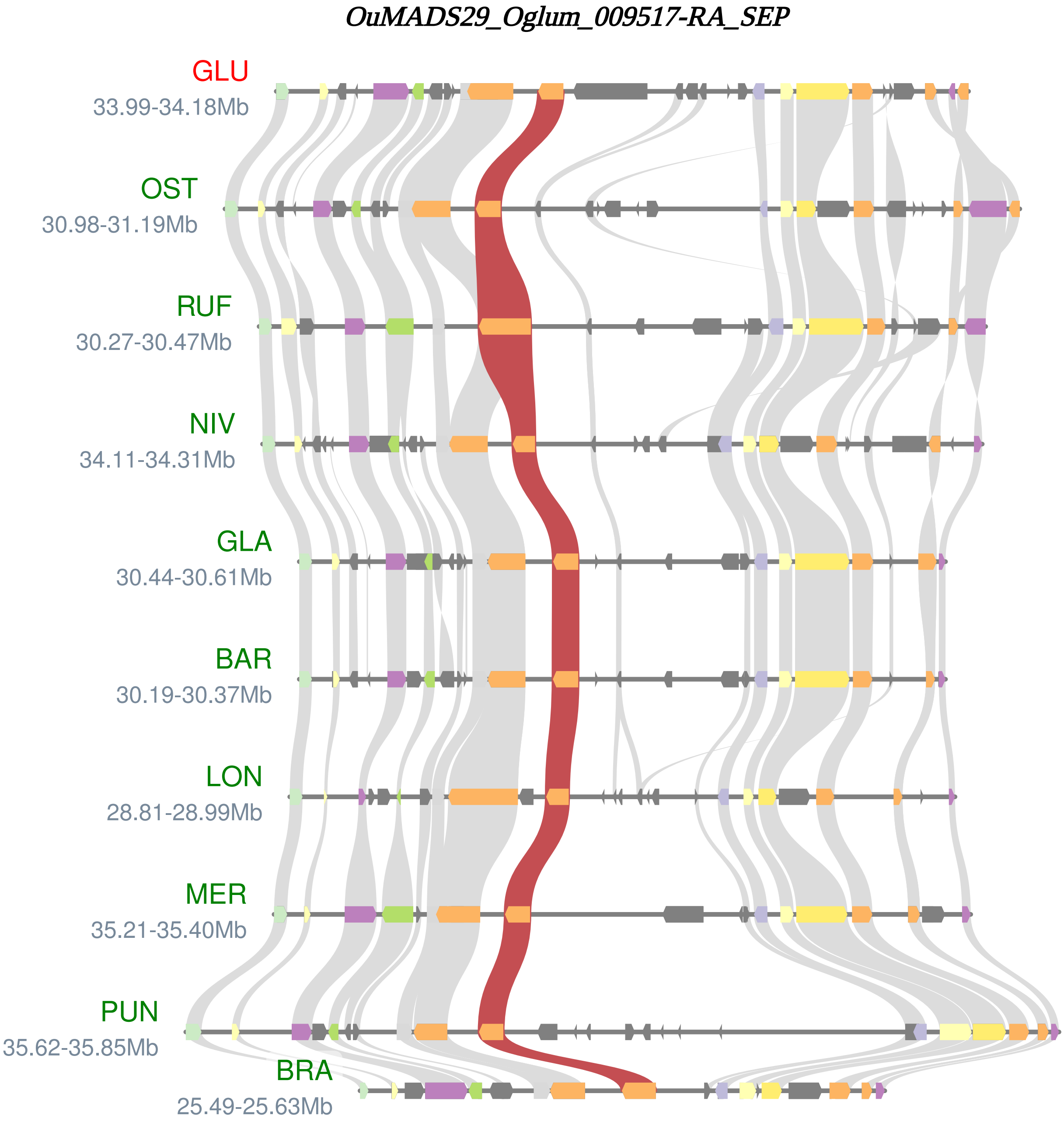
OuMADS29_Oglum_009517-RA_SEP
GLU
33.99-34.18Mb
OST
30.98-31.19Mb
RUF
30.27-30.47Mb
NIV
34.11-34.31Mb
GLA
30.44-30.61Mb
BAR
30.19-30.37Mb
LON
28.81-28.99Mb
MER
35.21-35.40Mb
PUN
35.62-35.85Mb
BRA
25.49-25.63Mb

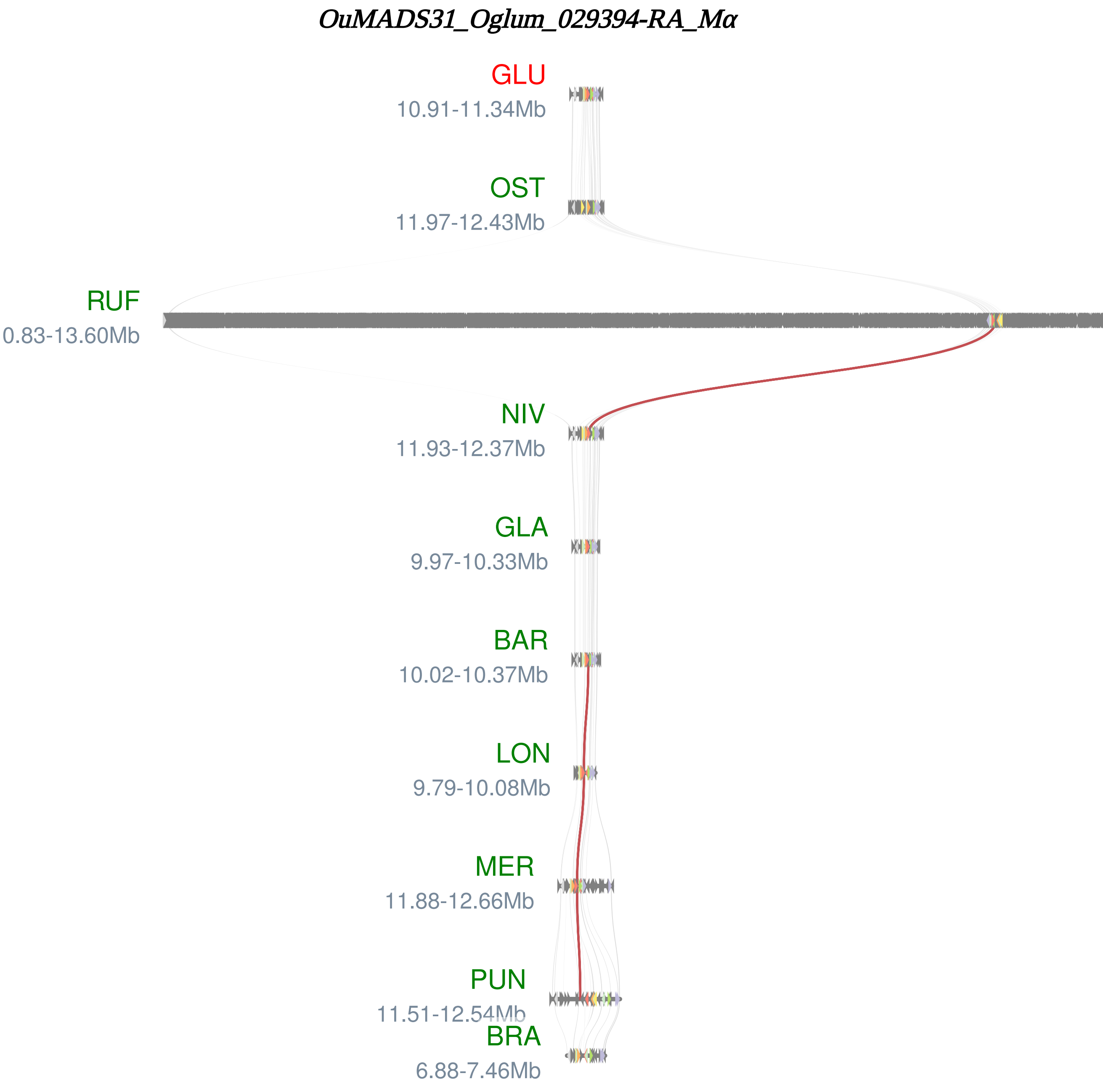
OuMADS31_Oglum_029394-RA_Mα
GLU
10.91-11.34Mb
OST
11.97-12.43Mb
RUF
0.83-13.60Mb
NIV
11.93-12.37Mb
GLA
9.97-10.33Mb
BAR
10.02-10.37Mb
LON
9.79-10.08Mb
MER
11.88-12.66Mb
PUN
11.51-12.54Mb
BRA
6.88-7.46Mb

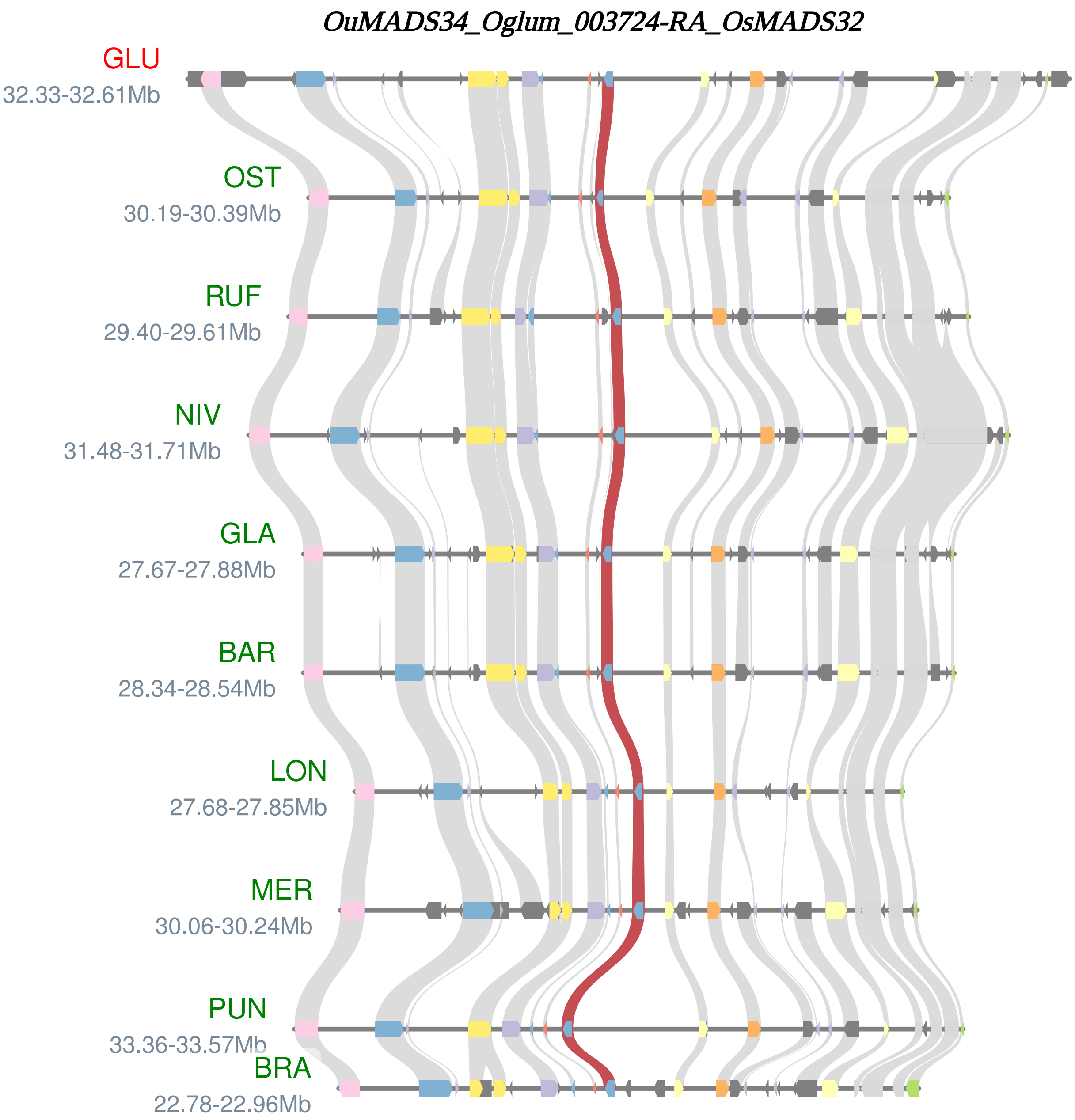

OuMADS34_Oglum_003724-RA_OsMADS32
GLU
32.33-32.61Mb
OST
30.19-30.39Mb
RUF
29.40-29.61Mb
NIV
31.48-31.71Mb
GLA
27.67-27.88Mb
BAR
28.34-28.54Mb
LON
27.68-27.85Mb
MER
30.06-30.24Mb
PUN
33.36-33.57Mb
BRA
22.78-22.96Mb

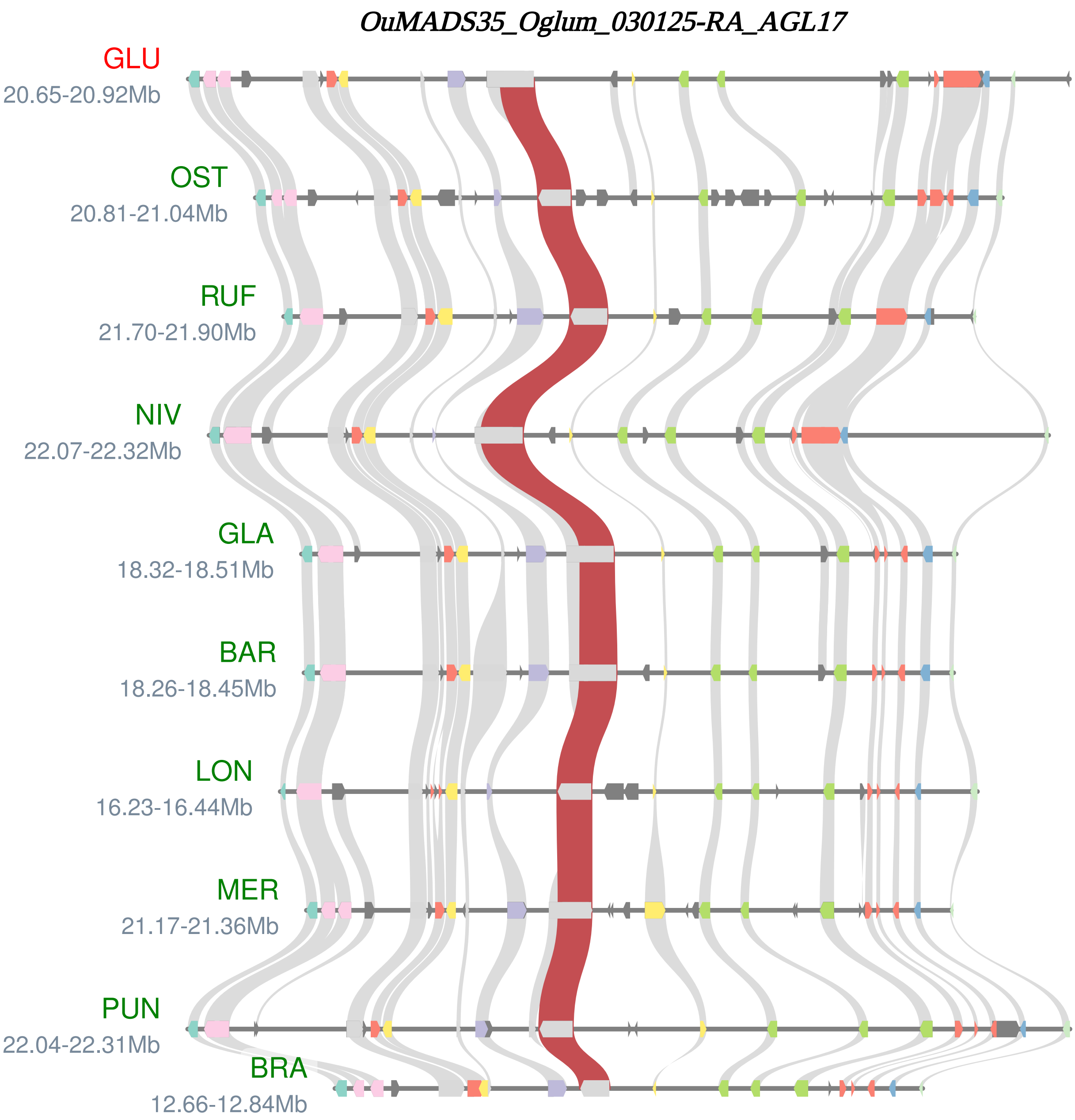

OuMADS35_Oglum_030125-RA_AGL17
GLU
20.65-20.92Mb
OST
20.81-21.04Mb
RUF
21.70-21.90Mb
NIV
22.07-22.32Mb
GLA
18.32-18.51Mb
BAR
18.26-18.45Mb
LON
16.23-16.44Mb
MER
21.17-21.36Mb
PUN
22.04-22.31Mb
BRA
12.66-12.84Mb

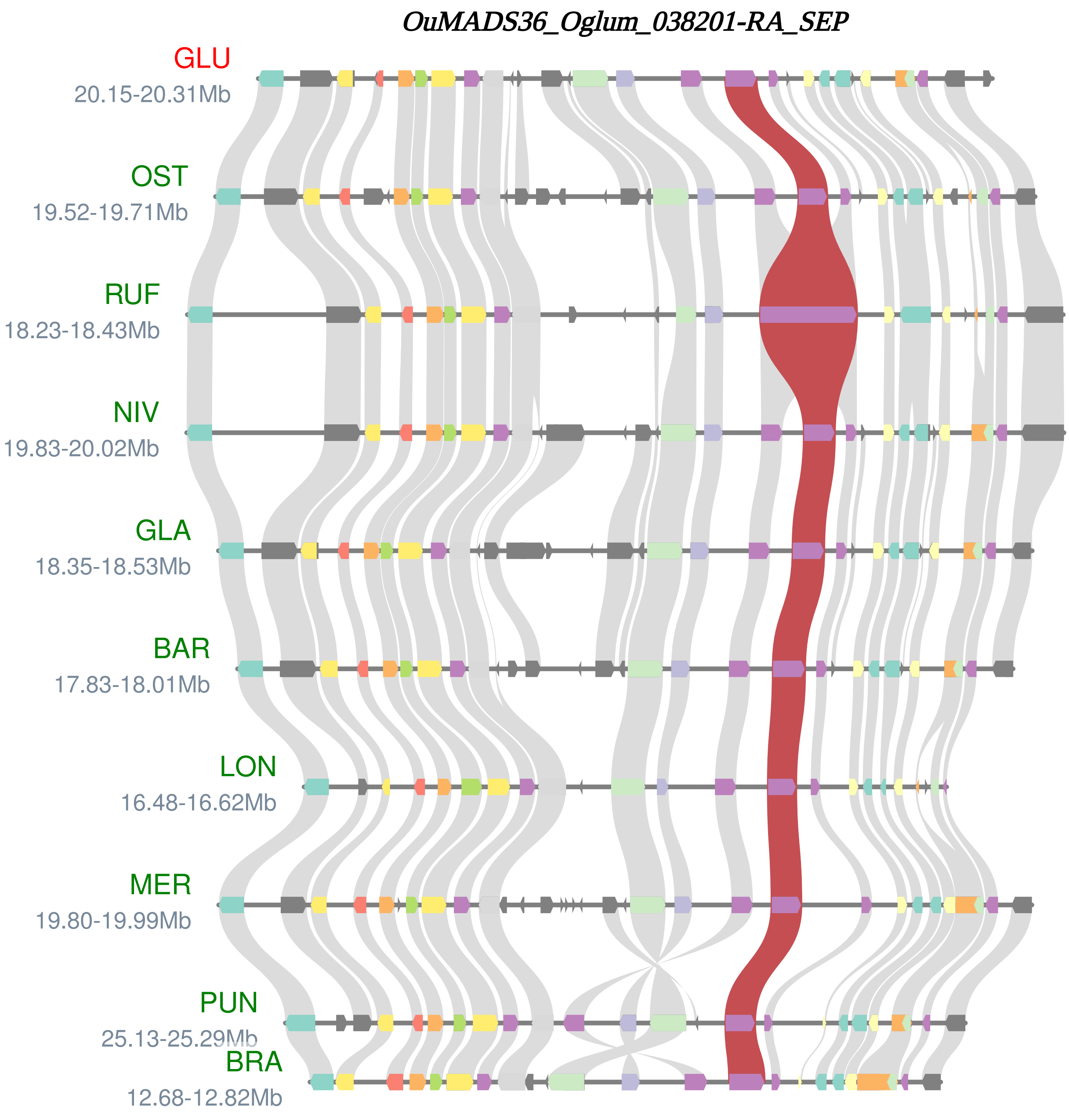

OuMADS36_Oglum_038201-RA_SEP
GLU
20.15-20.31Mb
OST
19.52-19.71Mb
RUF
18.23-18.43Mb
NIV
19.83-20.02Mb
GLA
18.35-18.53Mb
BAR
17.83-18.01Mb
LON
16.48-16.62Mb
MER
19.80-19.99Mb
PUN
25.13-25.29Mb
BRA
12.68-12.82Mb

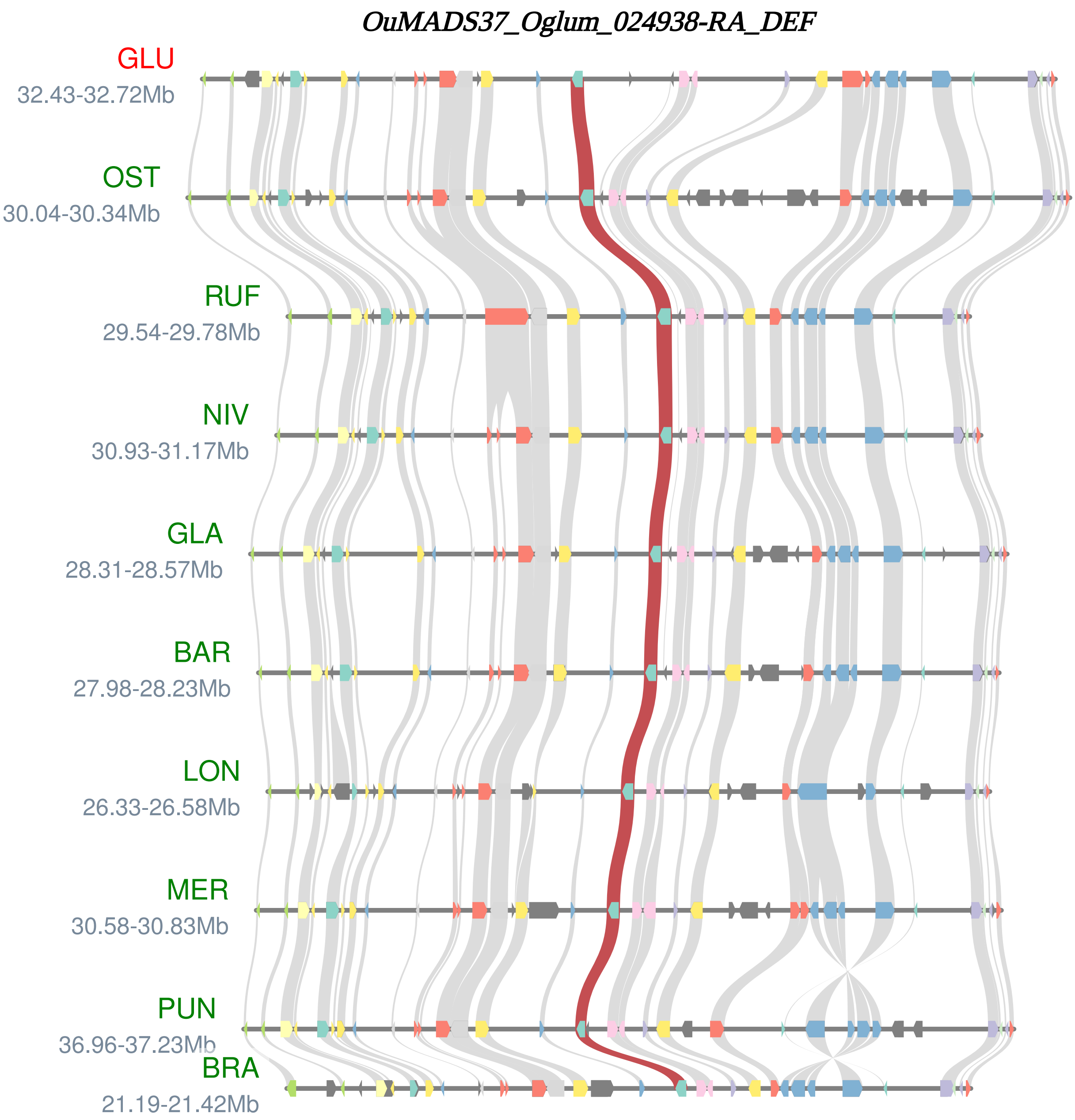

OuMADS37_Oglum_024938-RA_DEF
GLU
32.43-32.72Mb
OST
30.04-30.34Mb
RUF
29.54-29.78Mb
NIV
30.93-31.17Mb
GLA
28.31-28.57Mb
BAR
27.98-28.23Mb
LON
26.33-26.58Mb
MER
30.58-30.83Mb
PUN
36.96-37.23Mb
BRA
21.19-21.42Mb

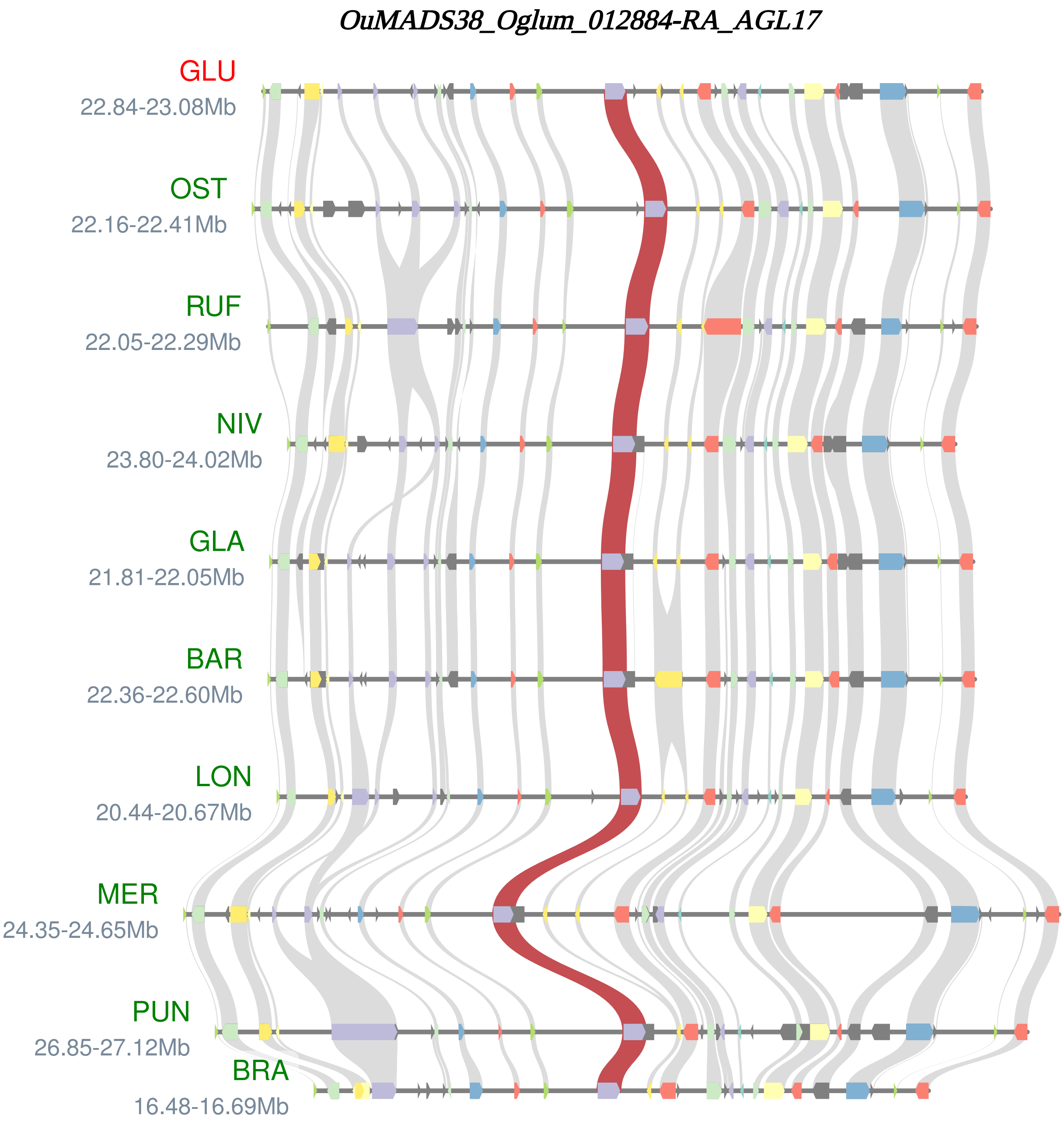

OuMADS38_Oglum_012884-RA_AGL17
GLU
22.84-23.08Mb
OST
22.16-22.41Mb
RUF
22.05-22.29Mb
NIV
23.80-24.02Mb
GLA
21.81-22.05Mb
BAR
22.36-22.60Mb
LON
20.44-20.67Mb
MER
24.35-24.65Mb
PUN
26.85-27.12Mb
BRA
16.48-16.69Mb

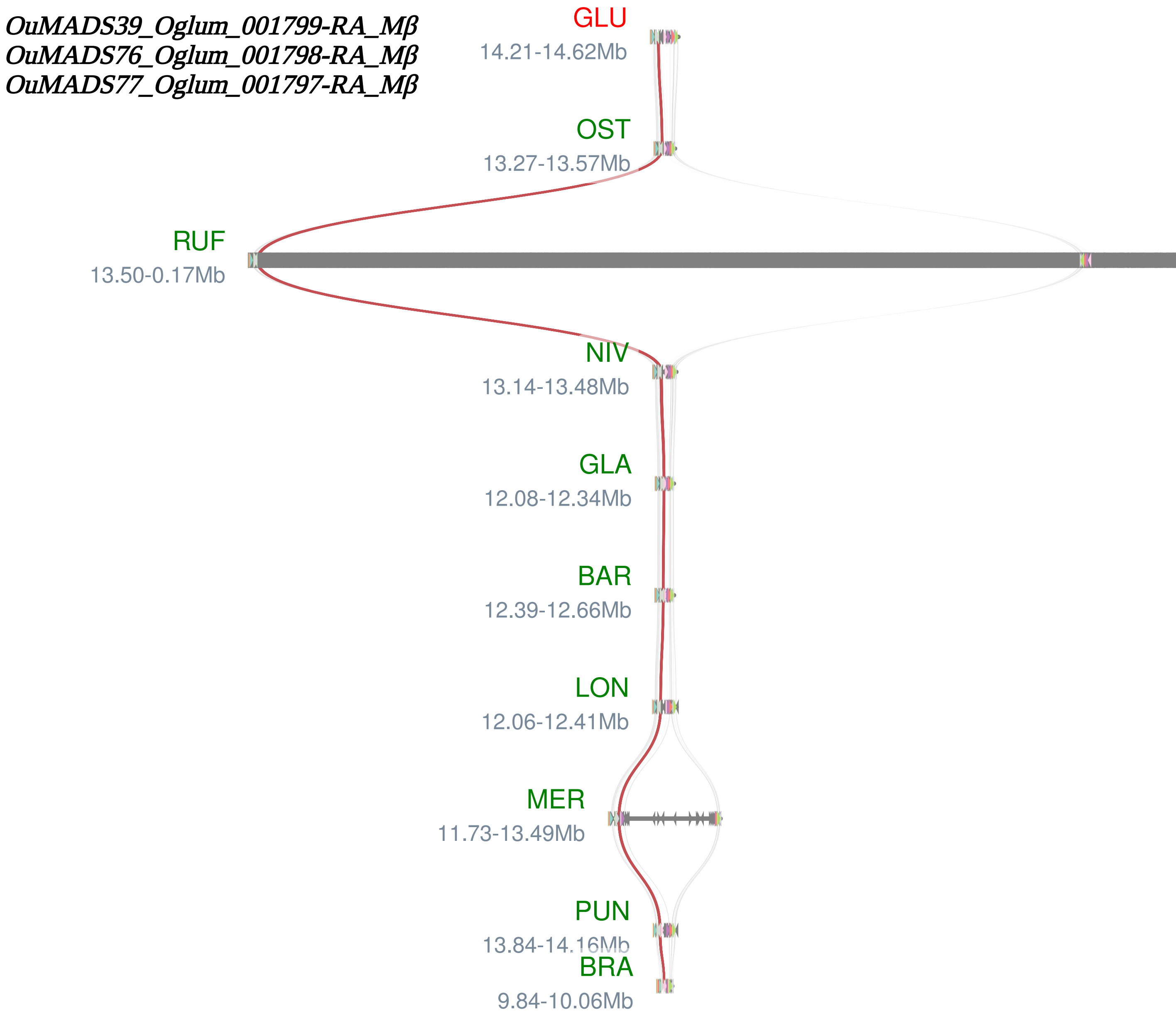

OuMADS39_Oglum_001799-RA_Mβ
OuMADS76_Oglum_001798-RA_Mβ
OuMADS77_Oglum_001797-RA_Mβ
GLU
14.21-14.62Mb
OST
13.27-13.57Mb
RUF
13.50-0.17Mb
NIV
13.14-13.48Mb
GLA
12.08-12.34Mb
BAR
12.39-12.66Mb
LON
12.06-12.41Mb
MER
11.73-13.49Mb
PUN
13.84-14.16Mb
BRA
9.84-10.06Mb

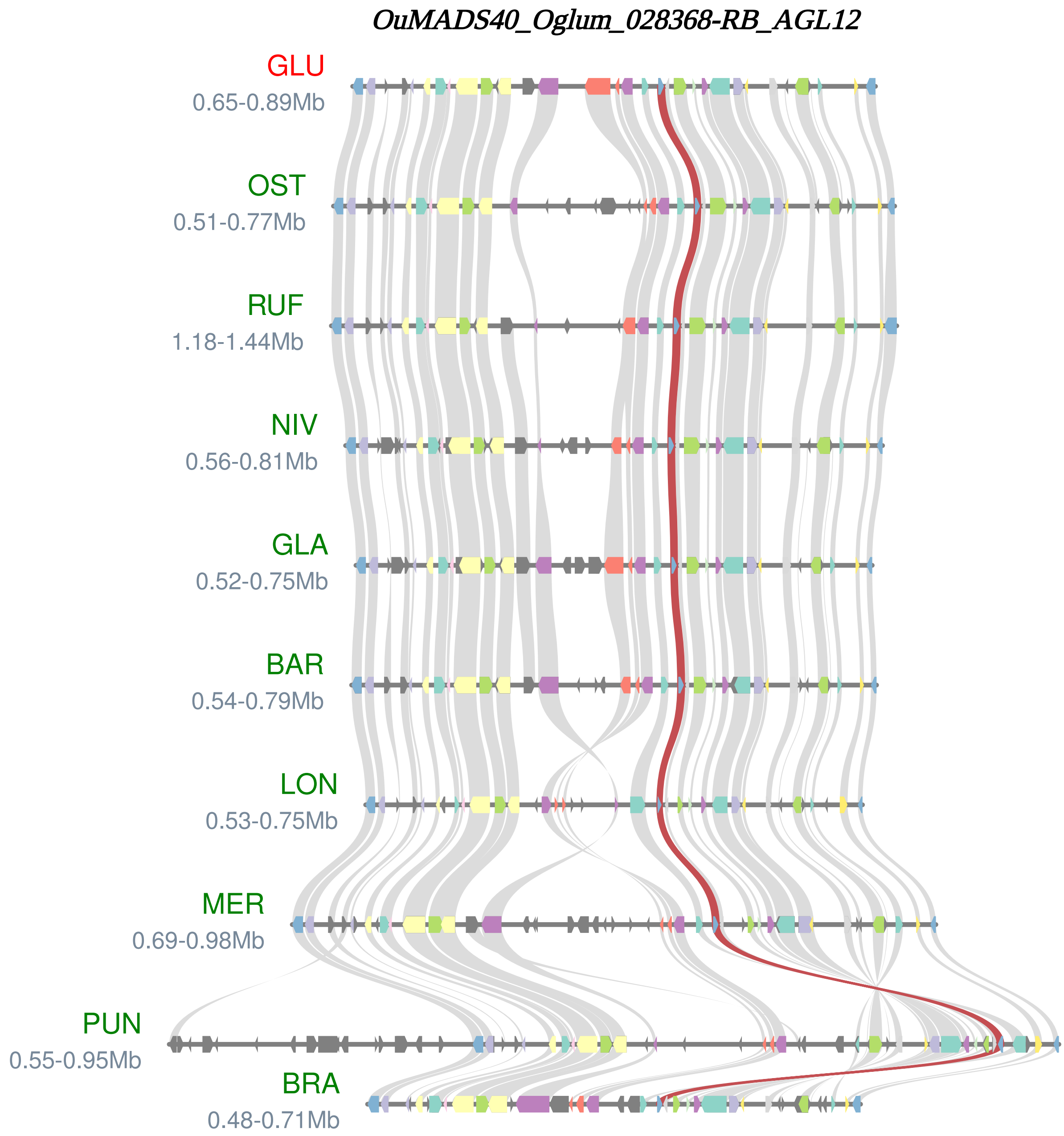
OuMADS40_Oglum_028368-RB_AGL12
GLU
0.65-0.89Mb
OST
0.51-0.77Mb
RUF
1.18-1.44Mb
NIV
0.56-0.81Mb
GLA
0.52-0.75Mb
BAR
0.54-0.79Mb
LON
0.53-0.75Mb
MER
0.69-0.98Mb
PUN
0.55-0.95Mb
BRA
0.48-0.71Mb

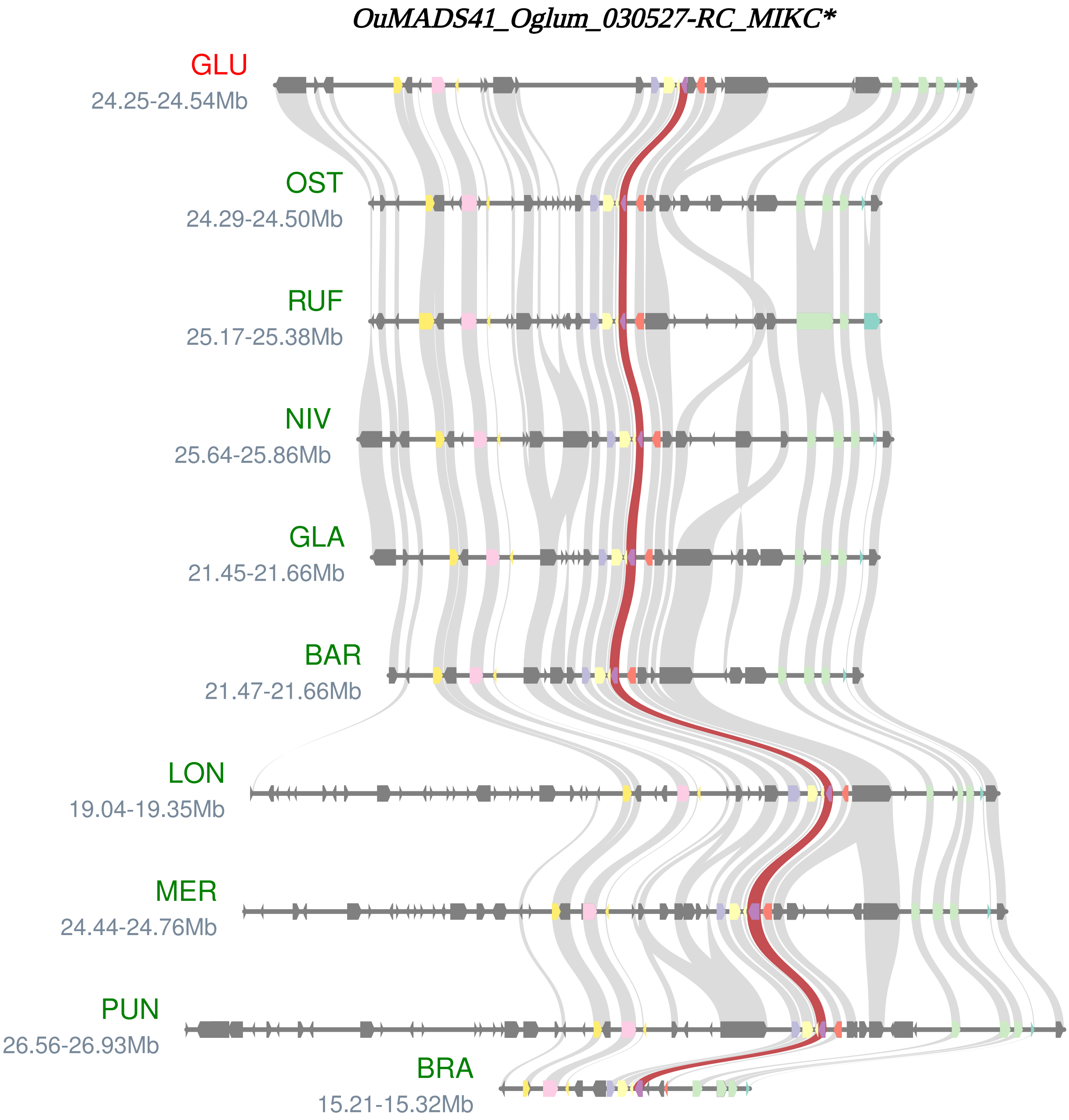
OuMADS41_Oglum_030527-RC_MIKC*
GLU
24.25-24.54Mb
OST
24.29-24.50Mb
RUF
25.17-25.38Mb
NIV
25.64-25.86Mb
GLA
21.45-21.66Mb
BAR
21.47-21.66Mb
LON
19.04-19.35Mb
MER
24.44-24.76Mb
PUN
26.56-26.93Mb
BRA
15.21-15.32Mb

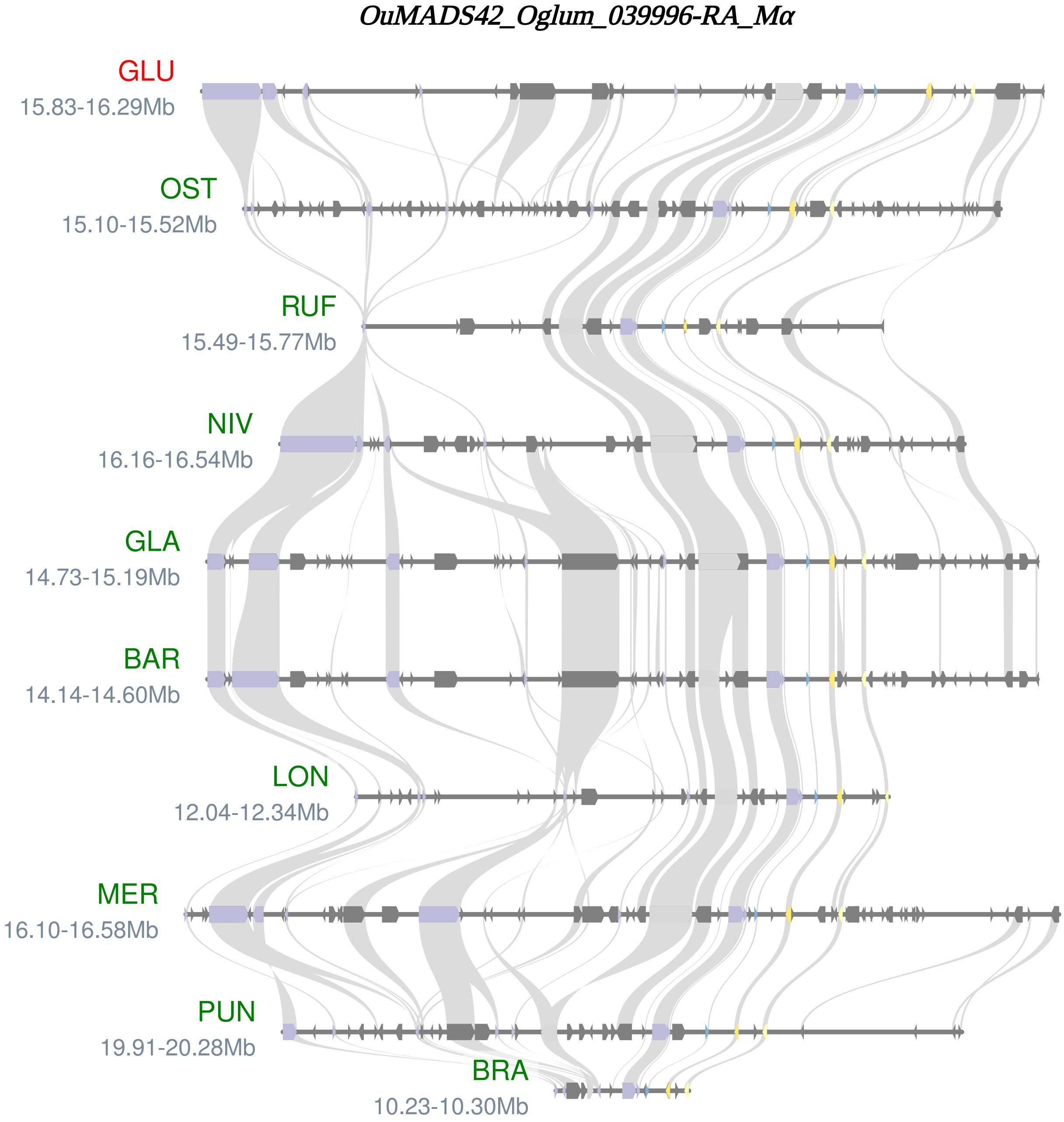
OuMADS42_Oglum_039996-RA_Mα
GLU
15.83-16.29Mb
OST
15.10-15.52Mb
RUF
15.49-15.77Mb
NIV
16.16-16.54Mb
GLA
14.73-15.19Mb
BAR
14.14-14.60Mb
LON
12.04-12.34Mb
MER
16.10-16.58Mb
PUN
19.91-20.28Mb
BRA
10.23-10.30Mb

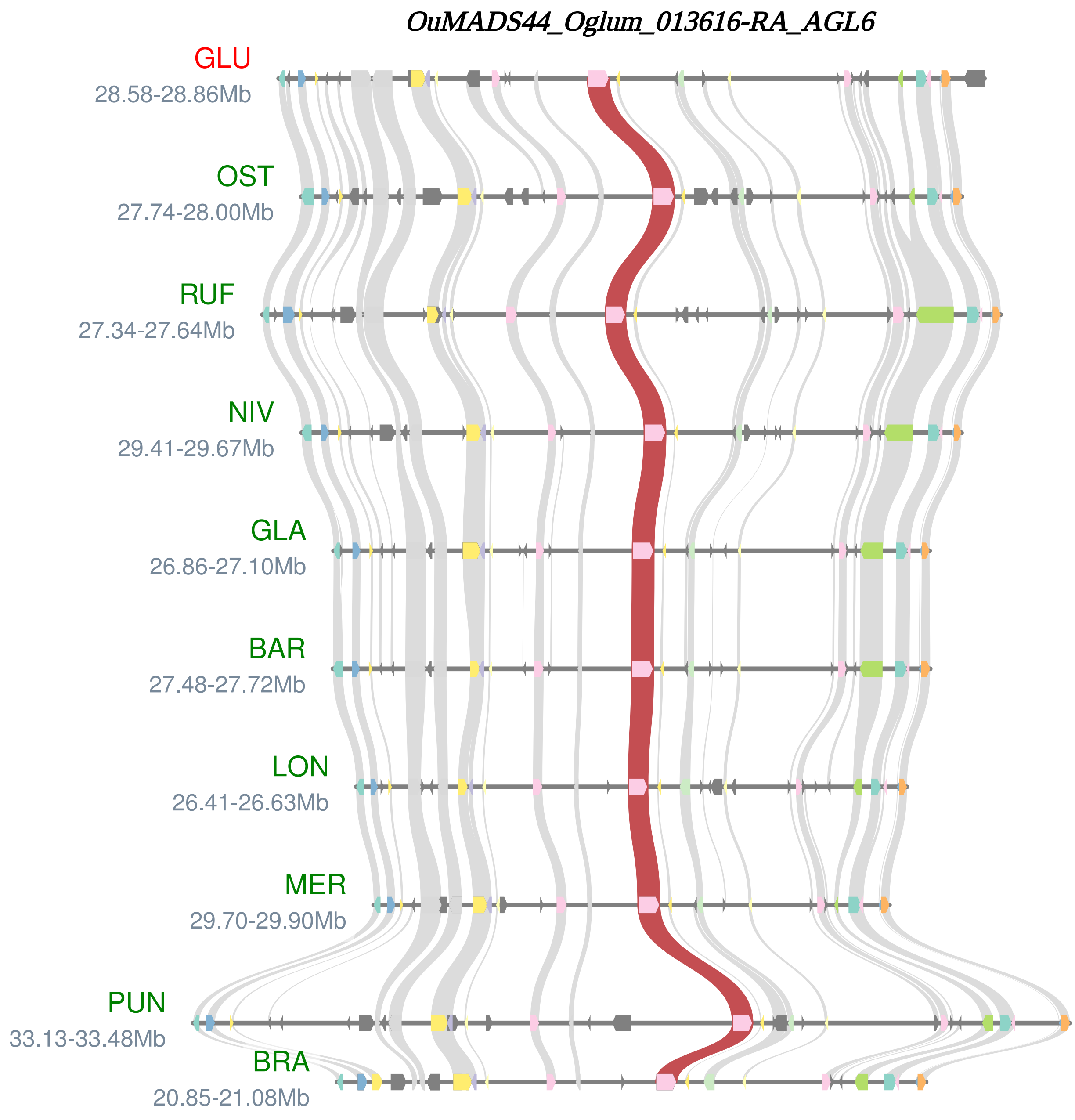
OuMADS44_Oglum_013616-RA_AGL6
GLU
28.58-28.86Mb
OST
27.74-28.00Mb
RUF
27.34-27.64Mb
NIV
29.41-29.67Mb
GLA
26.86-27.10Mb
BAR
27.48-27.72Mb
LON
26.41-26.63Mb
MER
29.70-29.90Mb
PUN
33.13-33.48Mb
BRA
20.85-21.08Mb

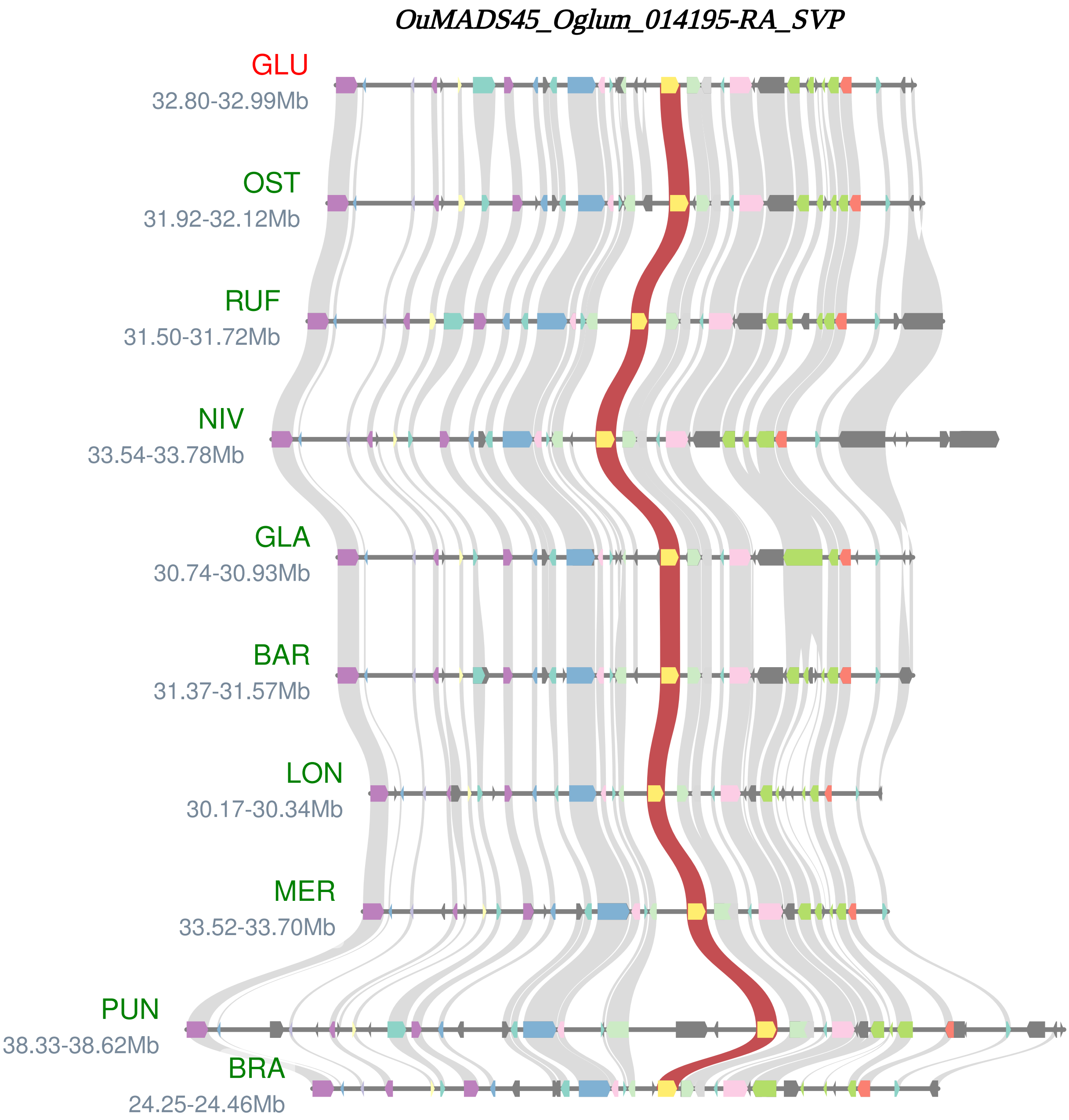
OuMADS45_Oglum_014195-RA_SVP
GLU
32.80-32.99Mb
OST
31.92-32.12Mb
RUF
31.50-31.72Mb
NIV
33.54-33.78Mb
GLA
30.74-30.93Mb
BAR
31.37-31.57Mb
LON
30.17-30.34Mb
MER
33.52-33.70Mb
PUN
38.33-38.62Mb
BRA
24.25-24.46Mb

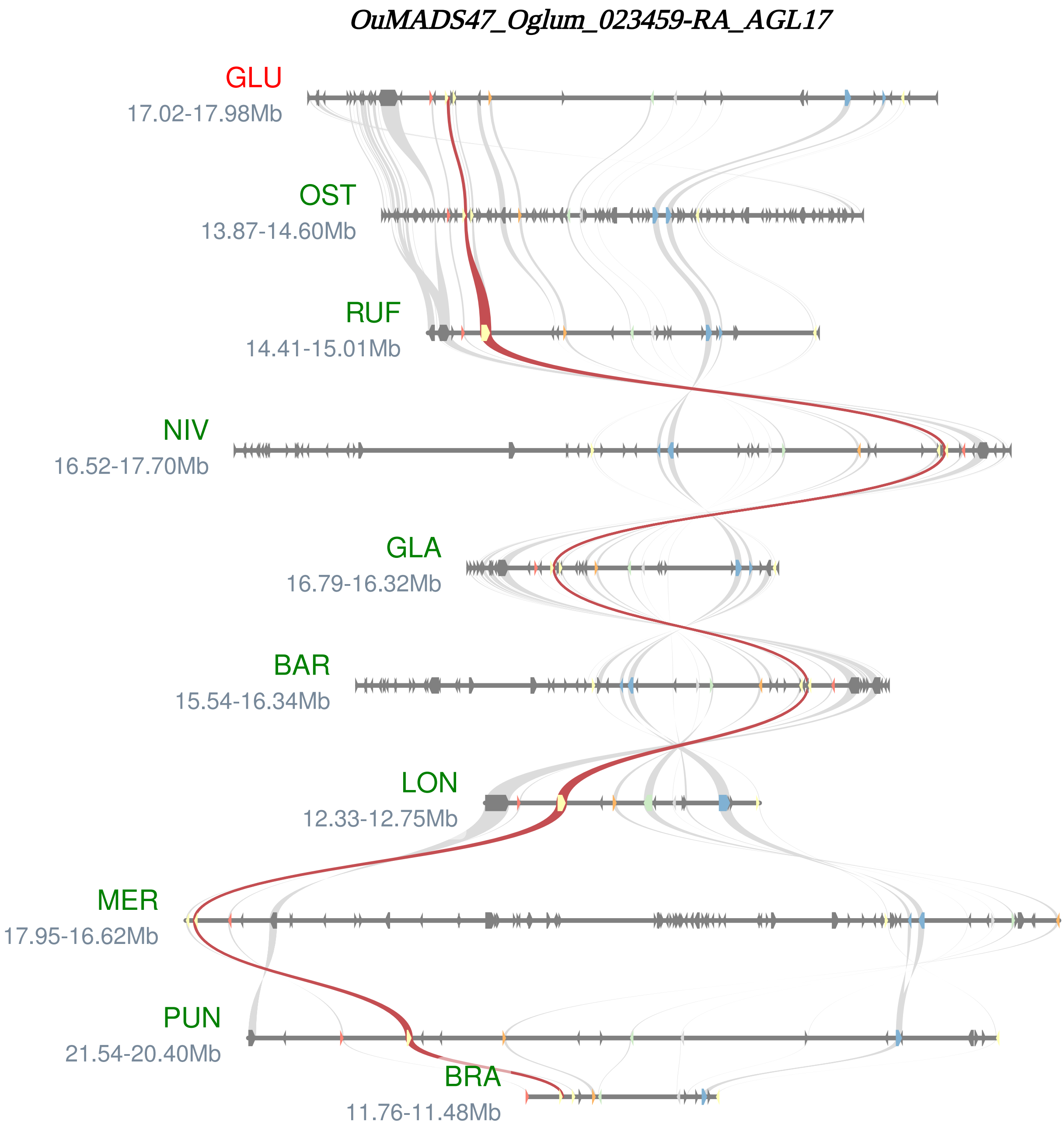
OuMADS47_Oglum_023459-RA_AGL17
GLU
17.02-17.98Mb
OST
13.87-14.60Mb
RUF
14.41-15.01Mb
NIV
16.52-17.70Mb
GLA
16.79-16.32Mb
BAR
15.54-16.34Mb
LON
12.33-12.75Mb
MER
17.95-16.62Mb
PUN
21.54-20.40Mb
BRA
11.76-11.48Mb

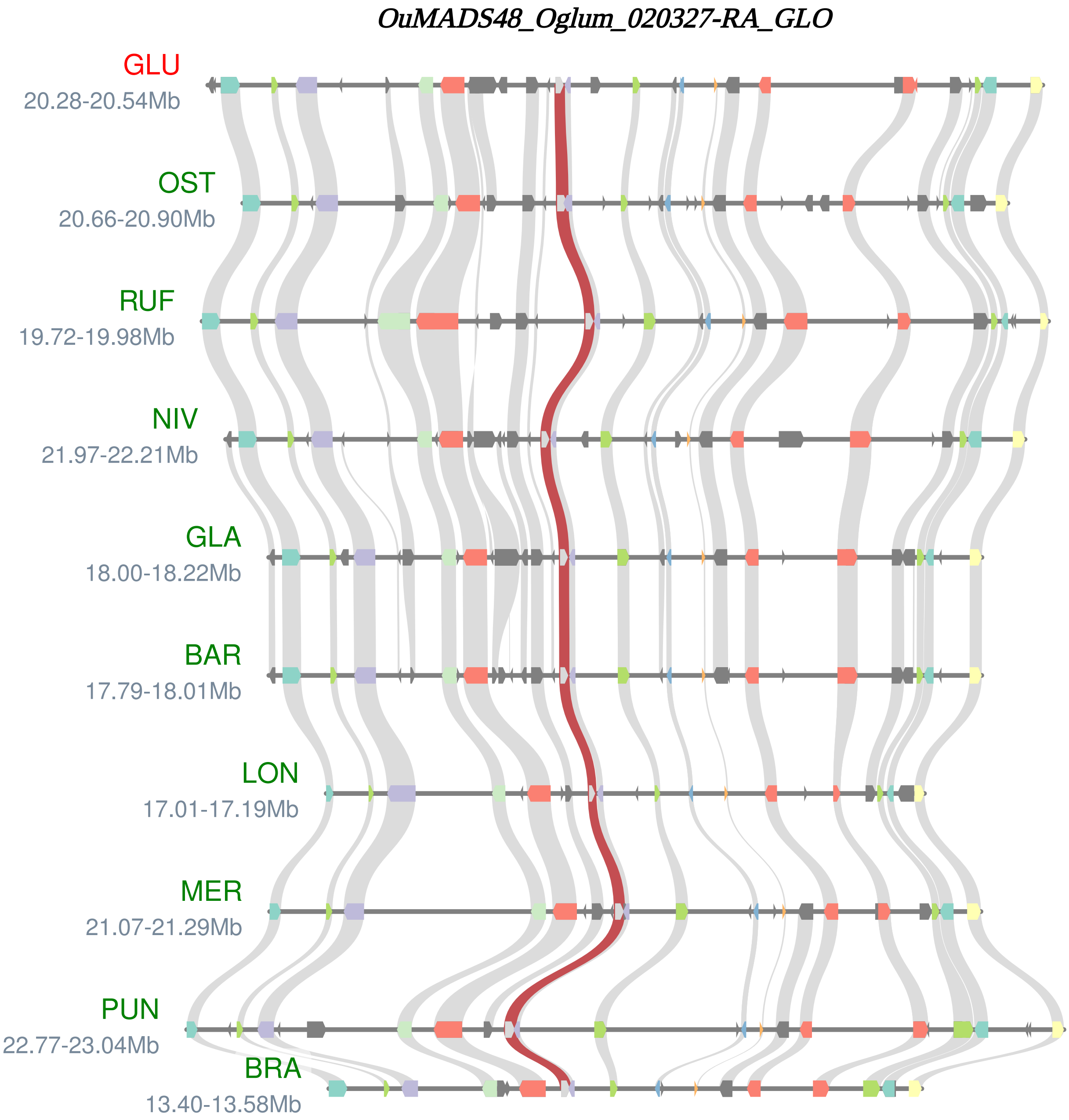
OuMADS48_Oglum_020327-RA_GLO
GLU
20.28-20.54Mb
OST
20.66-20.90Mb
RUF
19.72-19.98Mb
NIV
21.97-22.21Mb
GLA
18.00-18.22Mb
BAR
17.79-18.01Mb
LON
17.01-17.19Mb
MER
21.07-21.29Mb
PUN
22.77-23.04Mb
BRA
13.40-13.58Mb

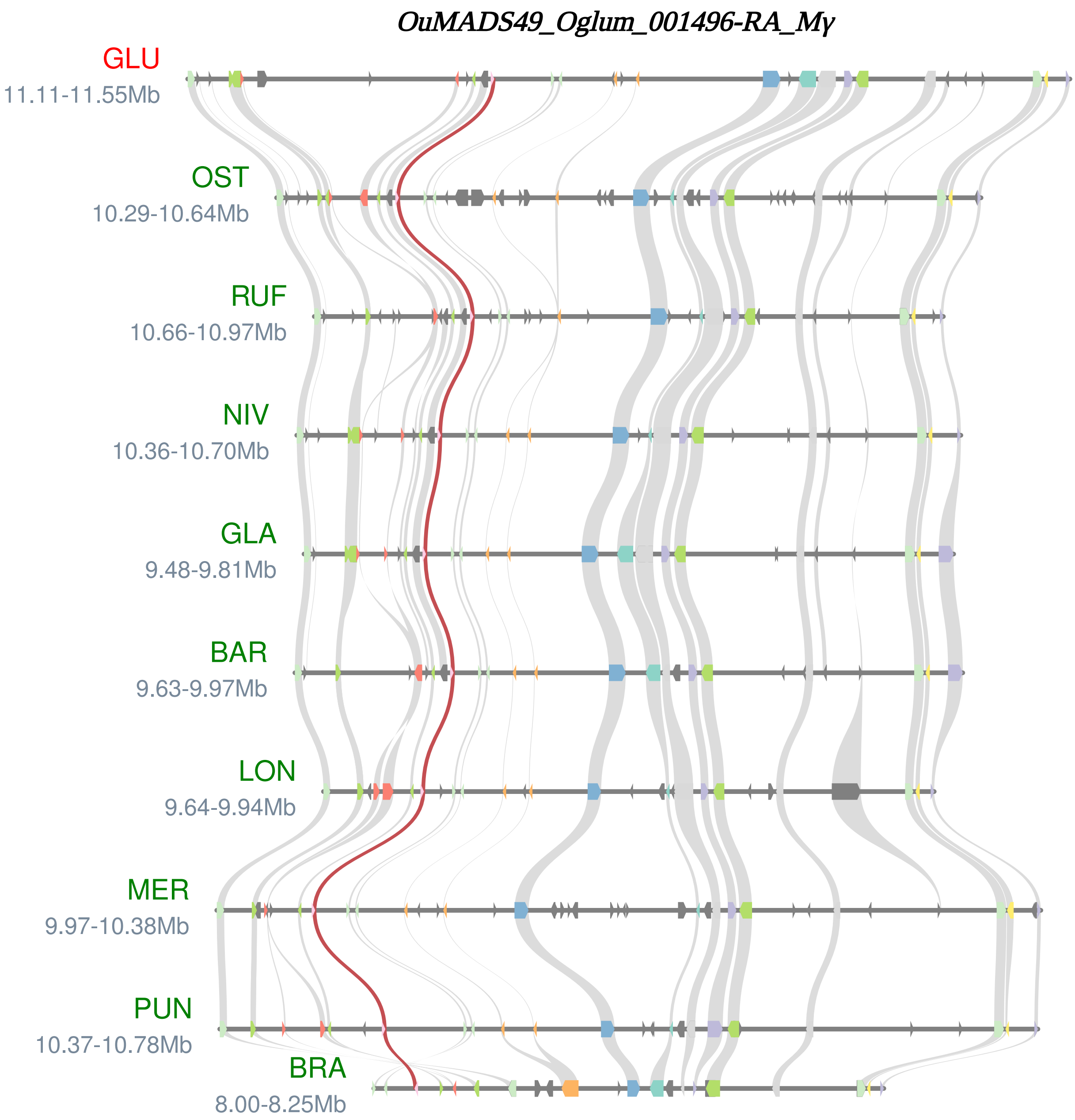

OuMADS49_Oglum_001496-RA_Mγ
GLU
11.11-11.55Mb
OST
10.29-10.64Mb
RUF
10.66-10.97Mb
NIV
10.36-10.70Mb
GLA
9.48-9.81Mb
BAR
9.63-9.97Mb
LON
9.64-9.94Mb
MER
9.97-10.38Mb
PUN
10.37-10.78Mb
BRA
8.00-8.25Mb

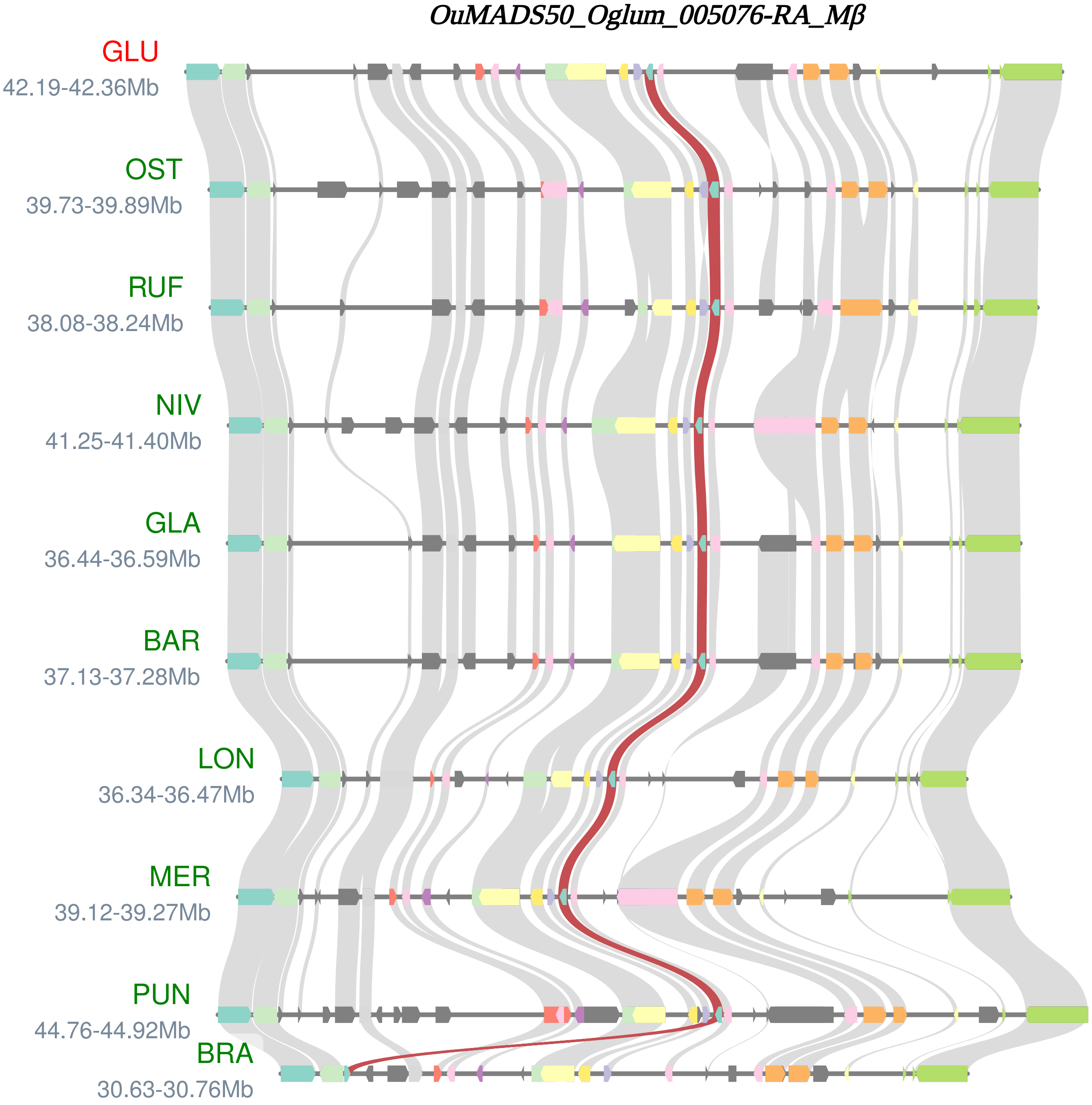
OuMADS50_Oglum_005076-RA_Mβ
GLU
42.19-42.36Mb
OST
39.73-39.89Mb
RUF
38.08-38.24Mb
NIV
41.25-41.40Mb
GLA
36.44-36.59Mb
BAR
37.13-37.28Mb
LON
36.34-36.47Mb
MER
39.12-39.27Mb
PUN
44.76-44.92Mb
BRA
30.63-30.76Mb

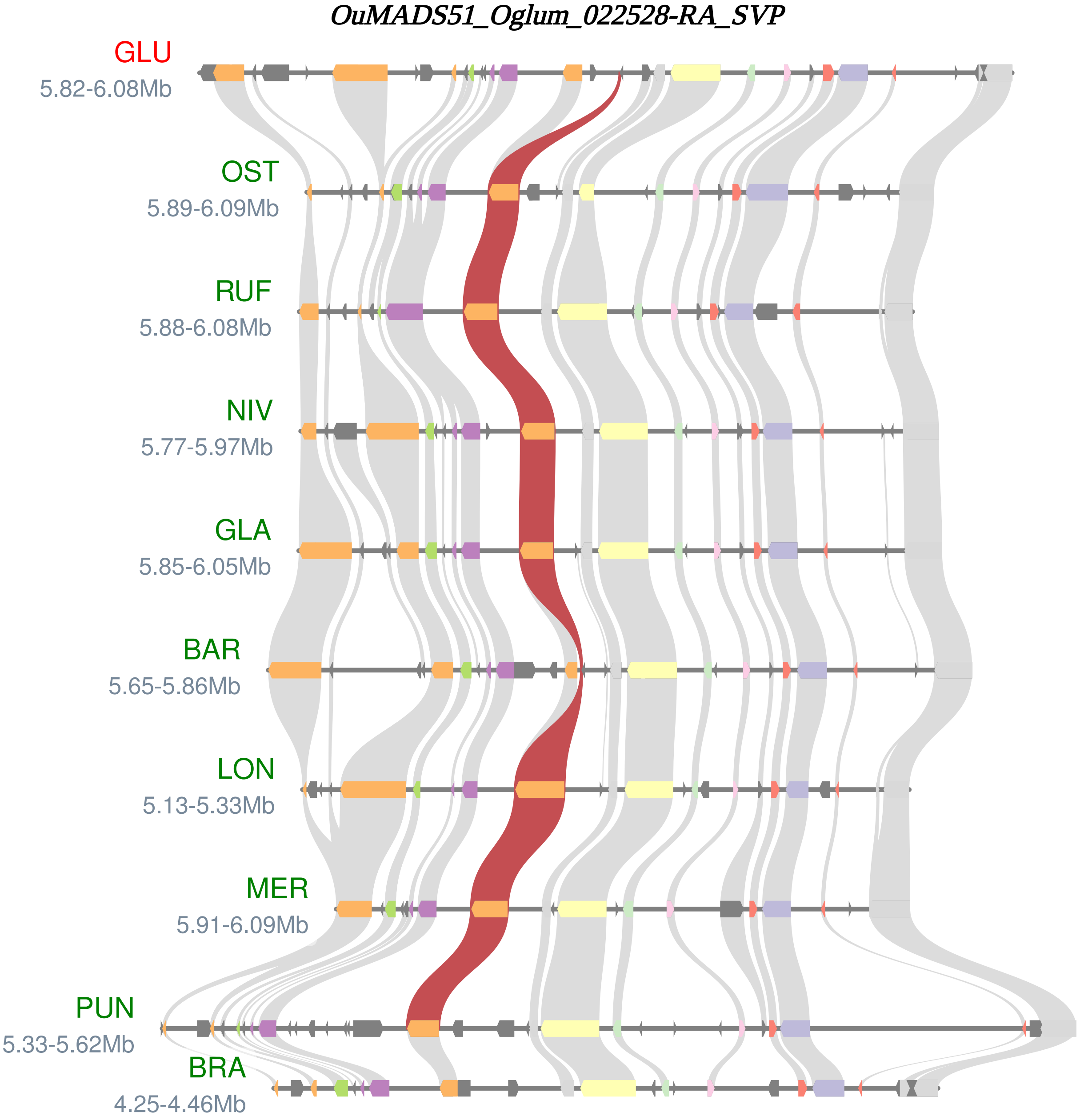
OuMADS51_Oglum_022528-RA_SVP
GLU
5.82-6.08Mb
OST
5.89-6.09Mb
RUF
5.88-6.08Mb
NIV
5.77-5.97Mb
GLA
5.85-6.05Mb
BAR
5.65-5.86Mb
LON
5.13-5.33Mb
MER
5.91-6.09Mb
PUN
5.33-5.62Mb
BRA
4.25-4.46Mb

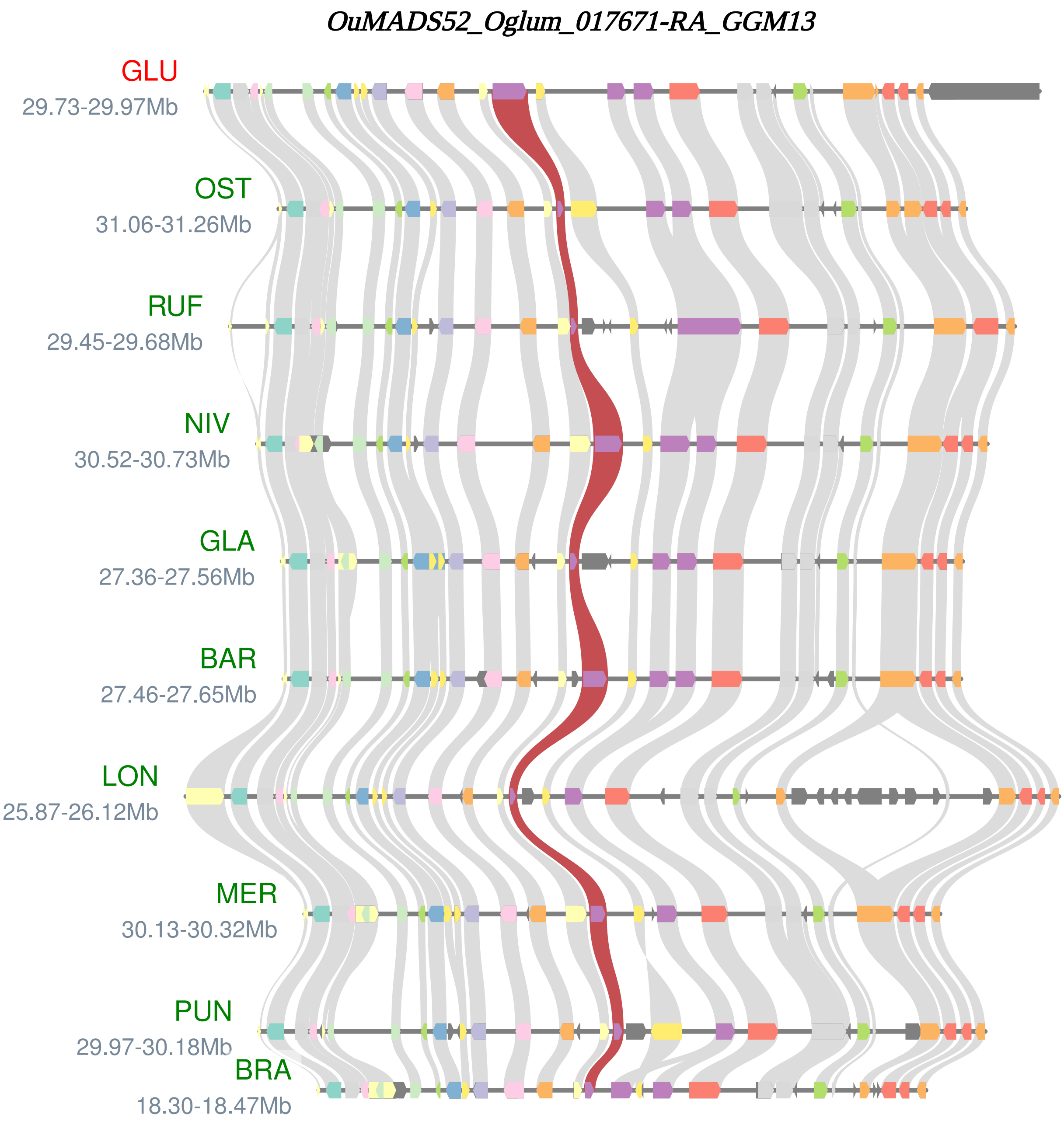
OuMADS52_Oglum_017671-RA_GGM13
GLU
29.73-29.97Mb
OST
31.06-31.26Mb
RUF
29.45-29.68Mb
NIV
30.52-30.73Mb
GLA
27.36-27.56Mb
BAR
27.46-27.65Mb
LON
25.87-26.12Mb
MER
30.13-30.32Mb
PUN
29.97-30.18Mb
BRA
18.30-18.47Mb

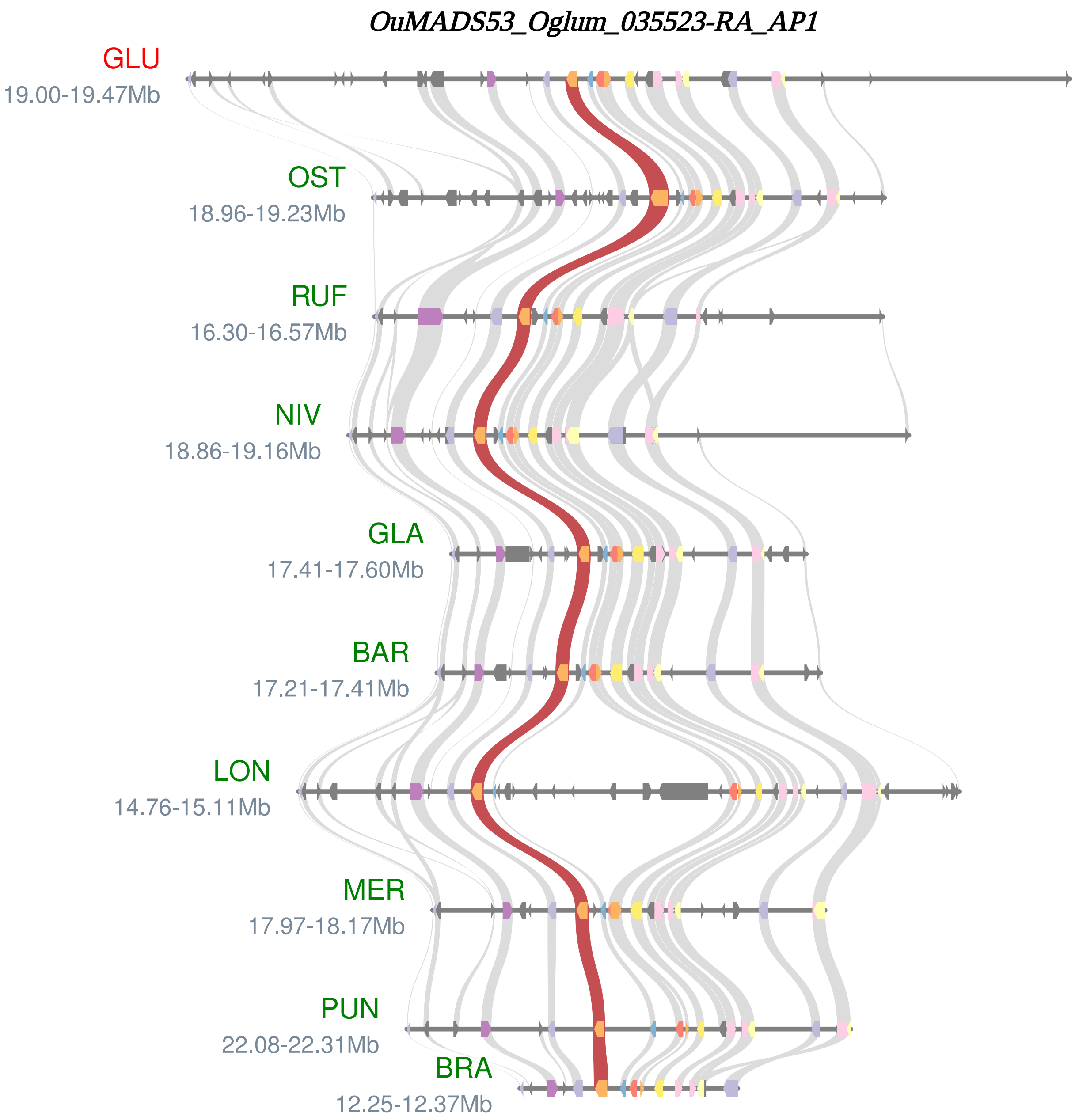
OuMADS53_Oglum_035523-RA_AP1
GLU
19.00-19.47Mb
OST
18.96-19.23Mb
RUF
16.30-16.57Mb
NIV
18.86-19.16Mb
GLA
17.41-17.60Mb
BAR
17.21-17.41Mb
LON
14.76-15.11Mb
MER
17.97-18.17Mb
PUN
22.08-22.31Mb
BRA
12.25-12.37Mb

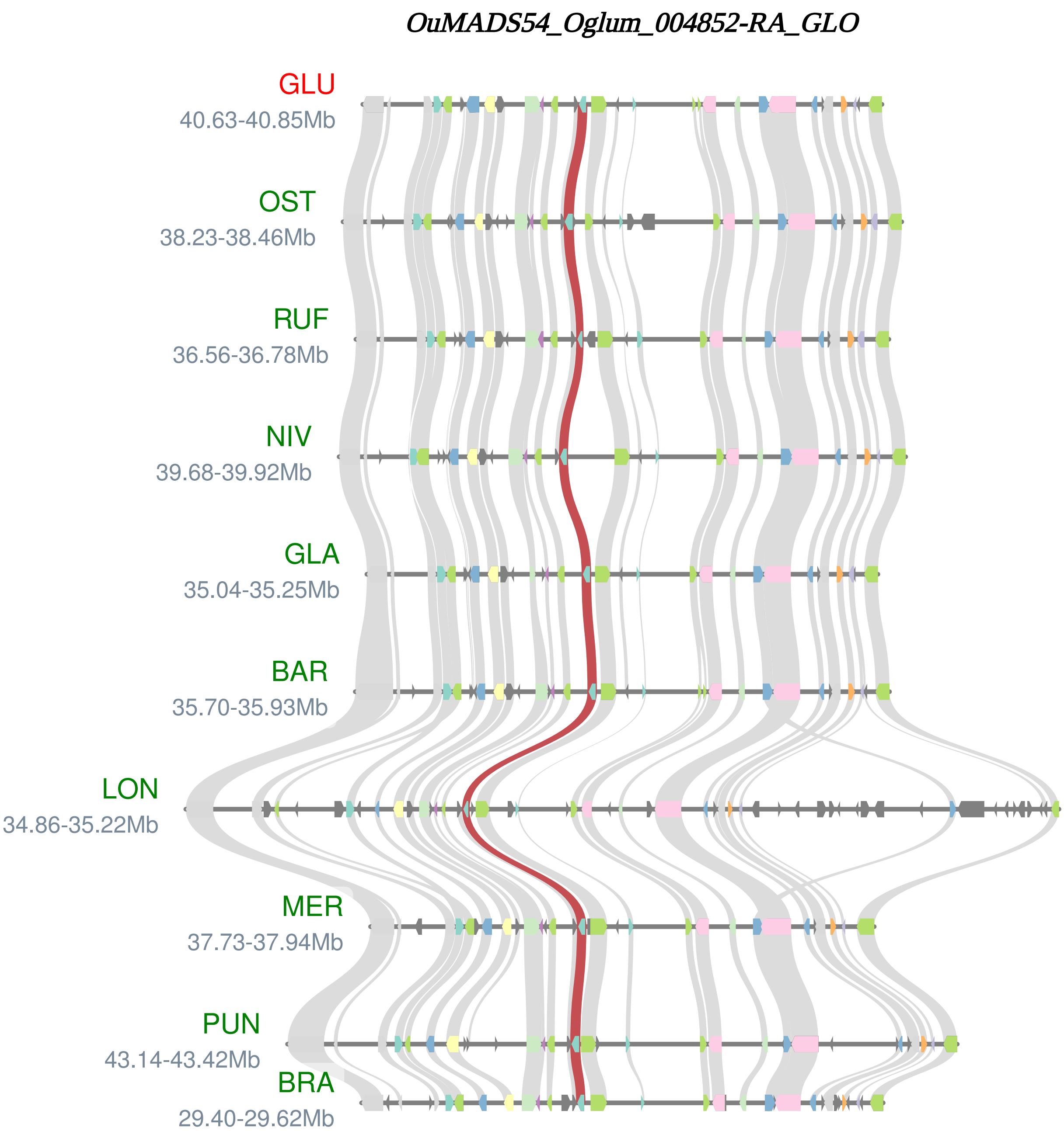

OuMADS54_Oglum_004852-RA_GLO
GLU
40.63-40.85Mb
OST
38.23-38.46Mb
RUF
36.56-36.78Mb
NIV
39.68-39.92Mb
GLA
35.04-35.25Mb
BAR
35.70-35.93Mb
LON
34.86-35.22Mb
MER
37.73-37.94Mb
PUN
43.14-43.42Mb
BRA
29.40-29.62Mb

OuMADS55_Oglum_027581-RC_AP1
(The chromosomal segment in the NIV&GLA&LON lacks any detected syntenic genes.)

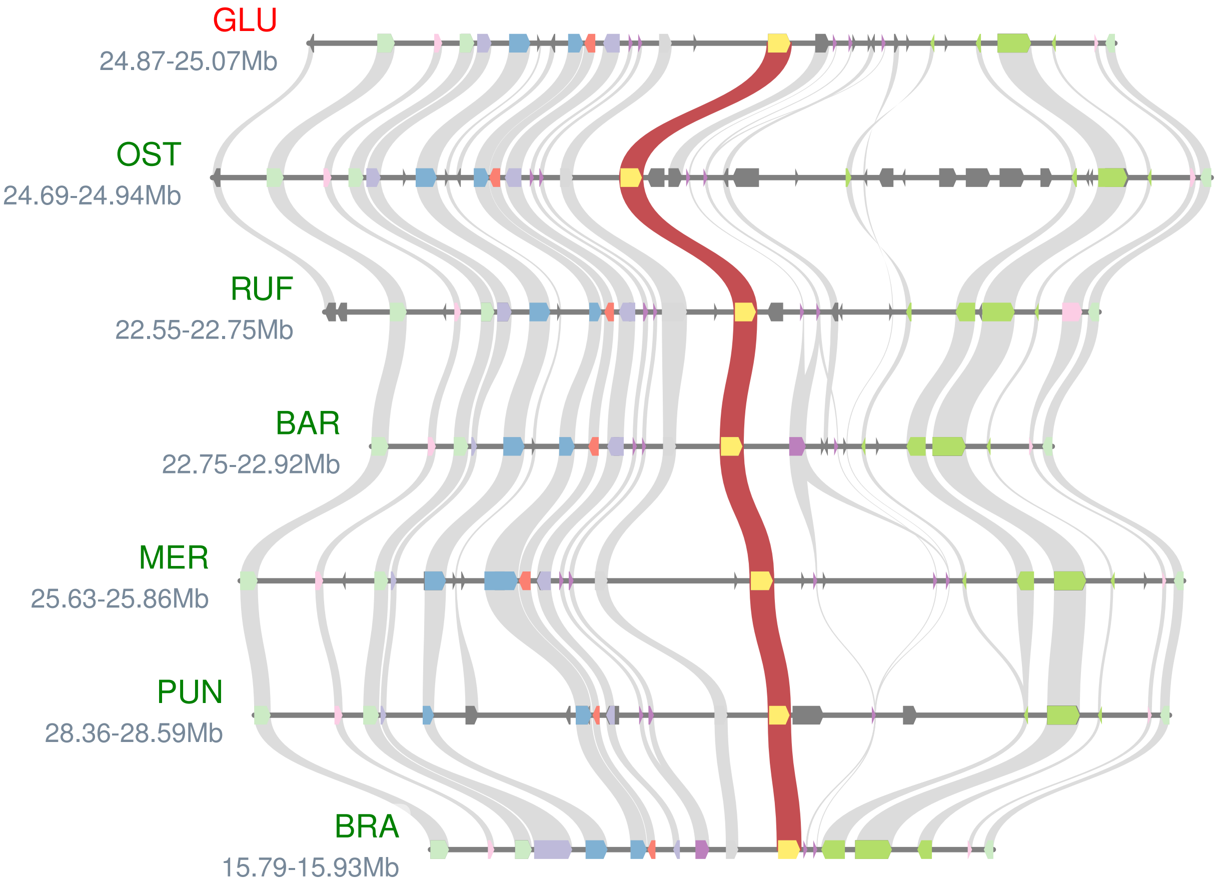

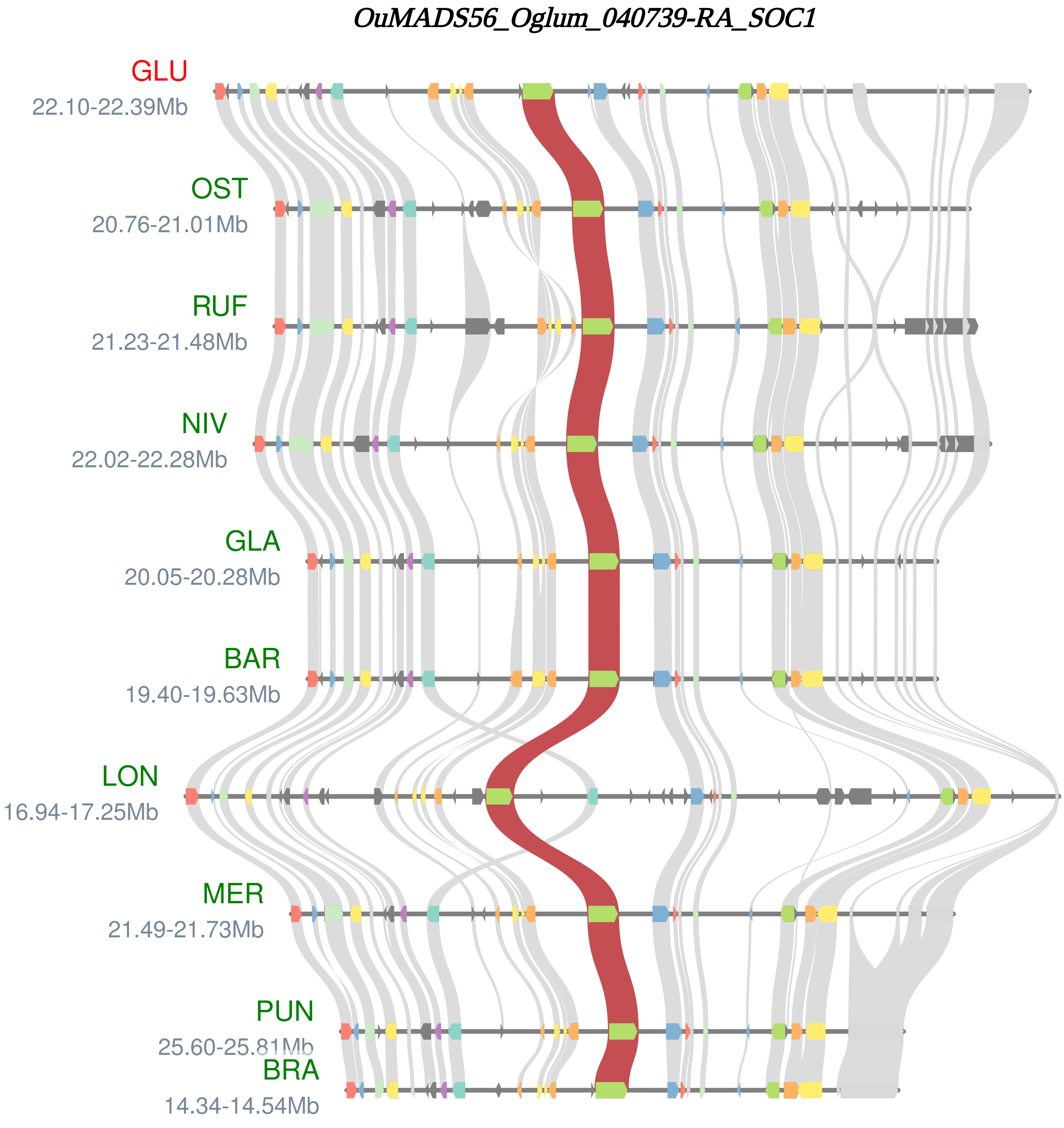
OuMADS56_Oglum_040739-RA_SOC1
GLU
22.10-22.39Mb
OST
20.76-21.01Mb
RUF
21.23-21.48Mb
NIV
22.02-22.28Mb
GLA
20.05-20.28Mb
BAR
19.40-19.63Mb
LON
16.94-17.25Mb
MER
21.49-21.73Mb
PUN
25.60-25.81Mb
BRA
14.34-14.54Mb

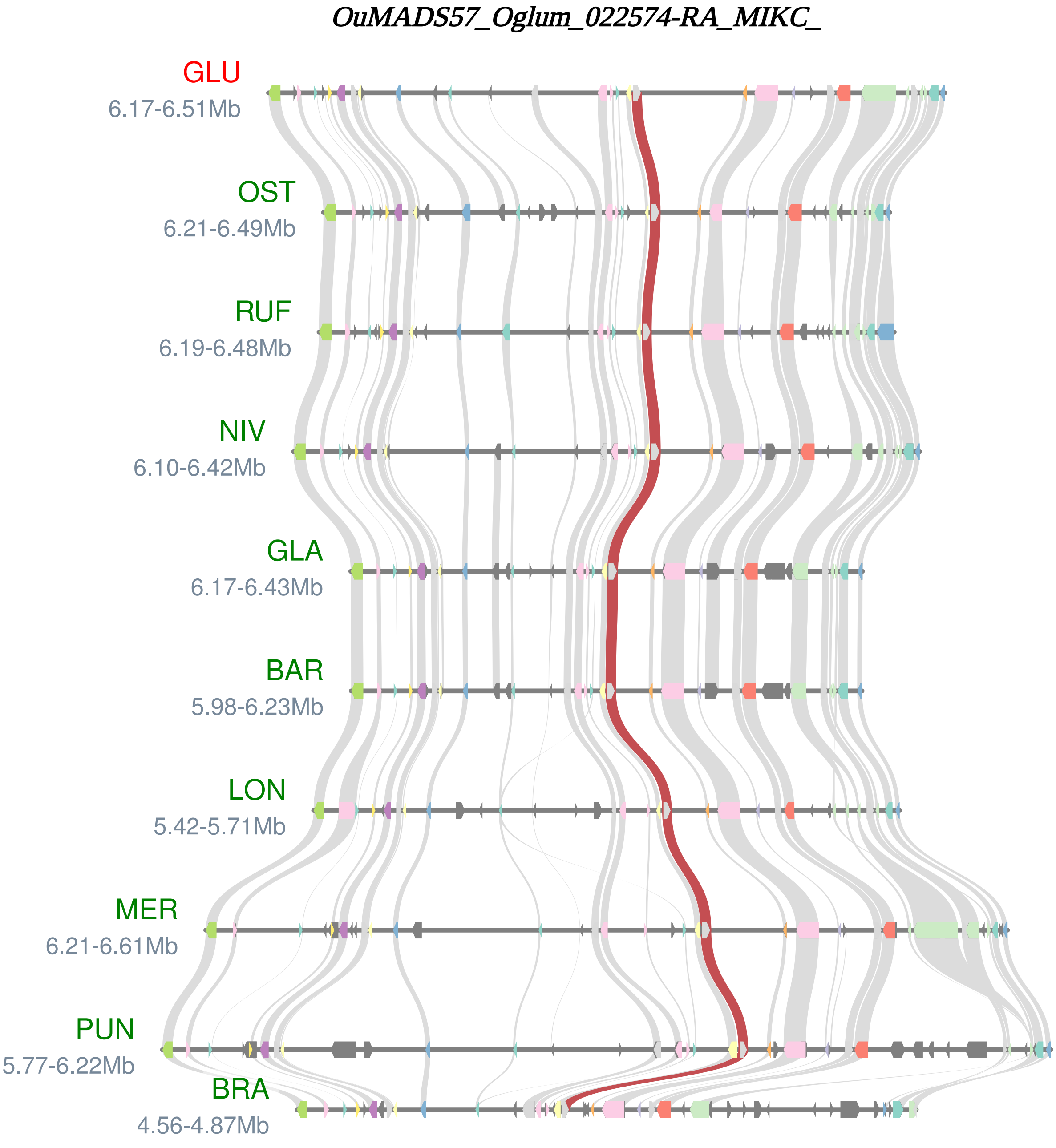
OuMADS57_Oglum_022574-RA_MIKC_
GLU
6.17-6.51Mb
OST
6.21-6.49Mb
RUF
6.19-6.48Mb
NIV
6.10-6.42Mb
GLA
6.17-6.43Mb
BAR
5.98-6.23Mb
LON
5.42-5.71Mb
MER
6.21-6.61Mb
PUN
5.77-6.22Mb
BRA
4.56-4.87Mb

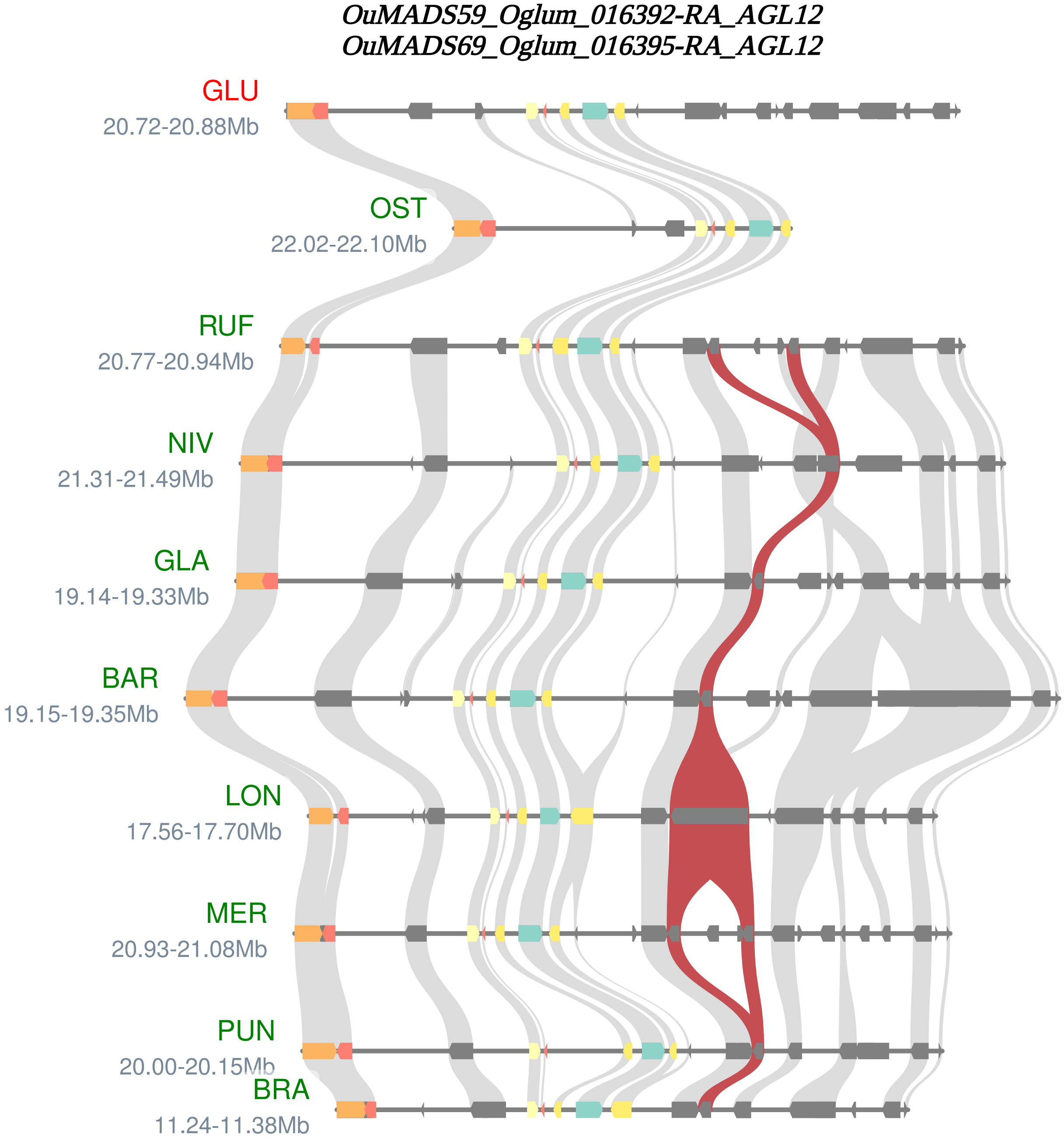

OuMADS59_Oglum_016392-RA_AGL12
OuMADS69_Oglum_016395-RA_AGL12
GLU
20.72-20.88Mb
OST
22.02-22.10Mb
RUF
20.77-20.94Mb
NIV
21.31-21.49Mb
GLA
19.14-19.33Mb
BAR
19.15-19.35Mb
LON
17.56-17.70Mb
MER
20.93-21.08Mb
PUN
20.00-20.15Mb
BRA
11.24-11.38Mb

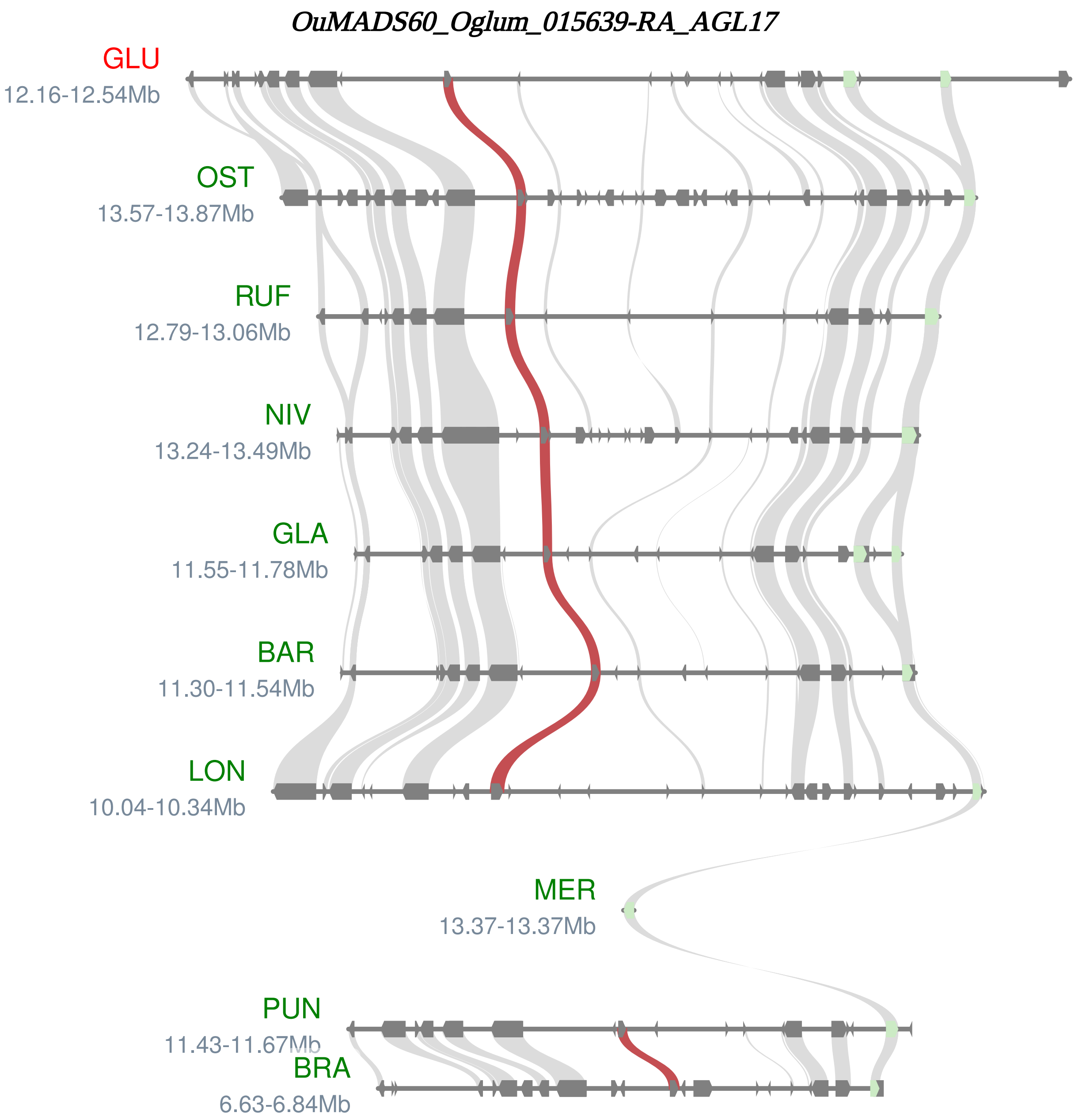

OuMADS60_Oglum_015639-RA_AGL17
GLU
12.16-12.54Mb
OST
13.57-13.87Mb
RUF
12.79-13.06Mb
NIV
13.24-13.49Mb
GLA
11.55-11.78Mb
BAR
11.30-11.54Mb
LON
10.04-10.34Mb
MER
13.37-13.37Mb
PUN
11.43-11.67Mb
BRA
6.63-6.84Mb

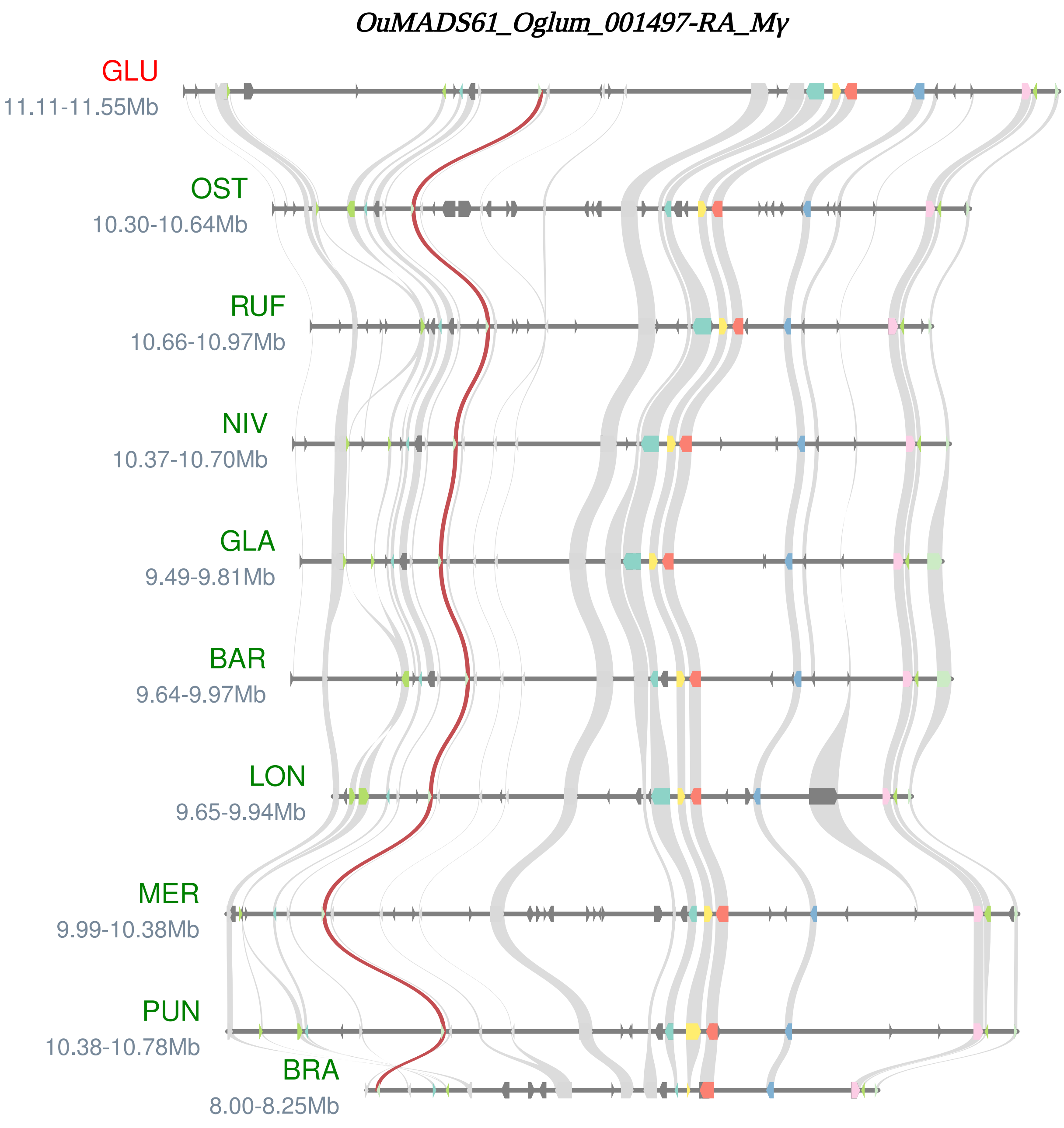

OuMADS61_Oglum_001497-RA_Mγ
GLU
11.11-11.55Mb
OST
10.30-10.64Mb
RUF
10.66-10.97Mb
NIV
10.37-10.70Mb
GLA
9.49-9.81Mb
BAR
9.64-9.97Mb
LON
9.65-9.94Mb
MER
9.99-10.38Mb
PUN
10.38-10.78Mb
BRA
8.00-8.25Mb

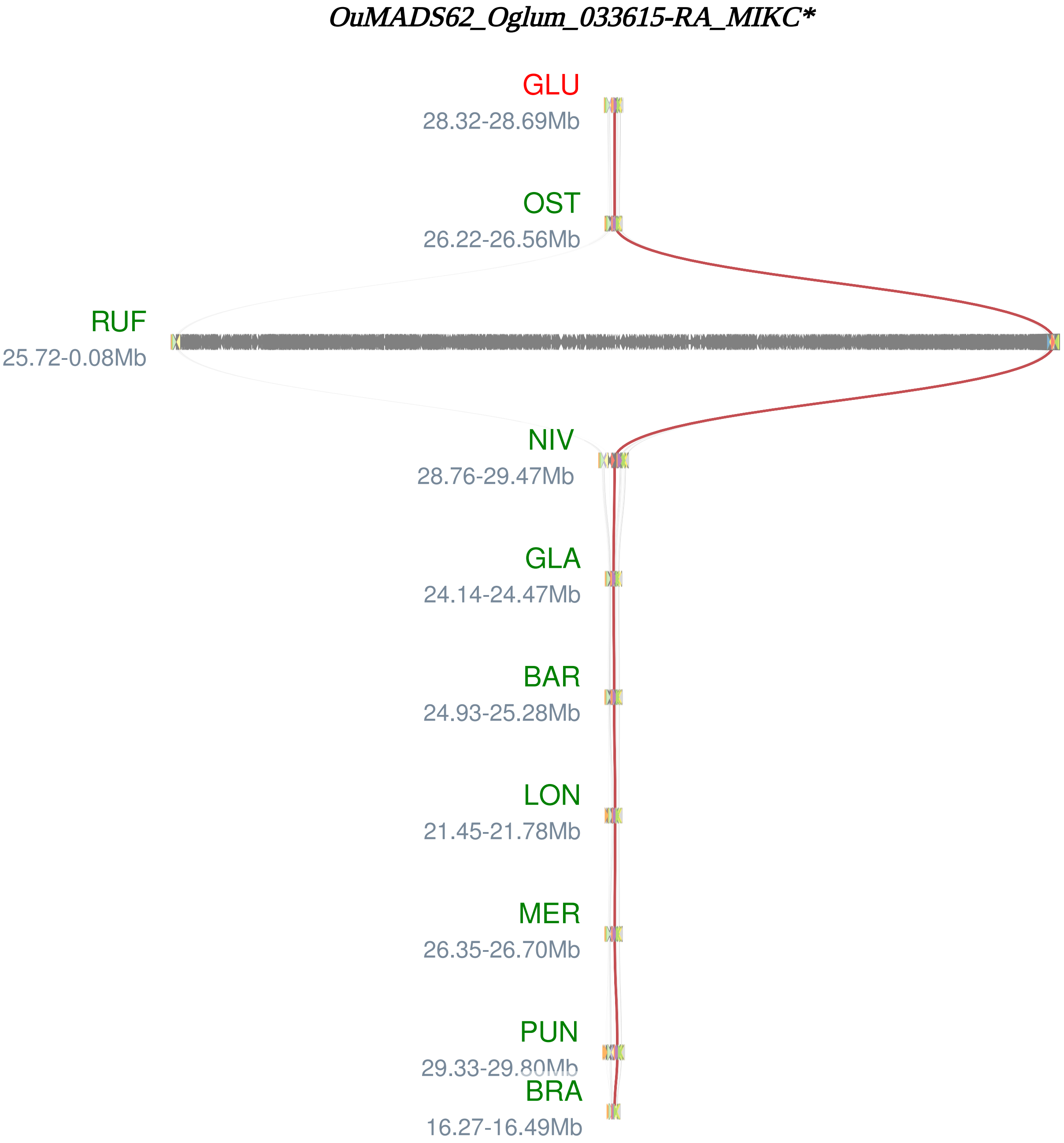
OuMADS62_Oglum_033615-RA_MIKC*
GLU
28.32-28.69Mb
OST
26.22-26.56Mb
RUF
25.72-0.08Mb
NIV
28.76-29.47Mb
GLA
24.14-24.47Mb
BAR
24.93-25.28Mb
LON
21.45-21.78Mb
MER
26.35-26.70Mb
PUN
29.33-29.80Mb
BRA
16.27-16.49Mb

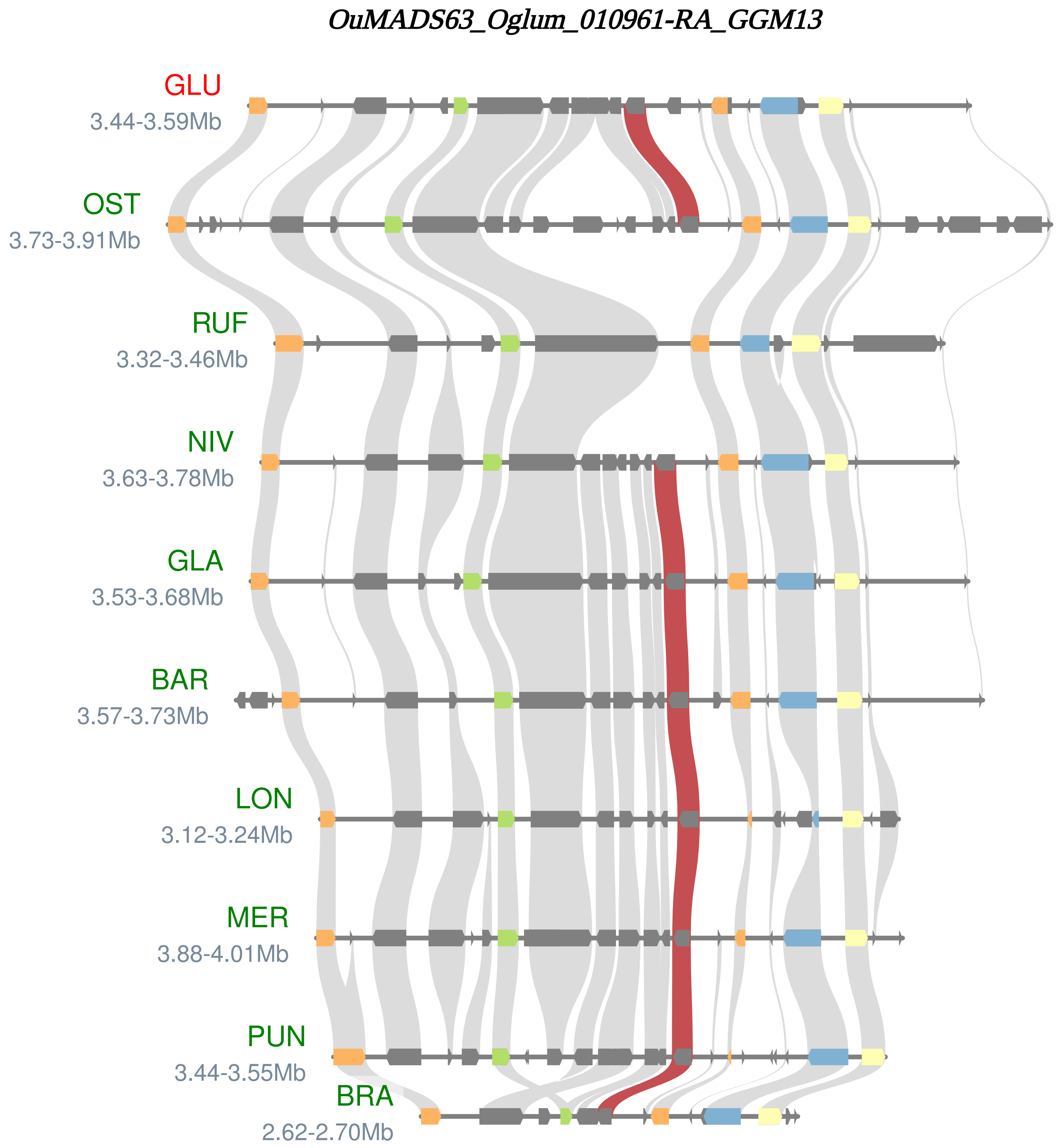

OuMADS63_Oglum_010961-RA_GGM13
GLU
3.44-3.59Mb
OST
3.73-3.91Mb
RUF
3.32-3.46Mb
NIV
3.63-3.78Mb
GLA
3.53-3.68Mb
BAR
3.57-3.73Mb
LON
3.12-3.24Mb
MER
3.88-4.01Mb
PUN
3.44-3.55Mb
BRA
2.62-2.70Mb

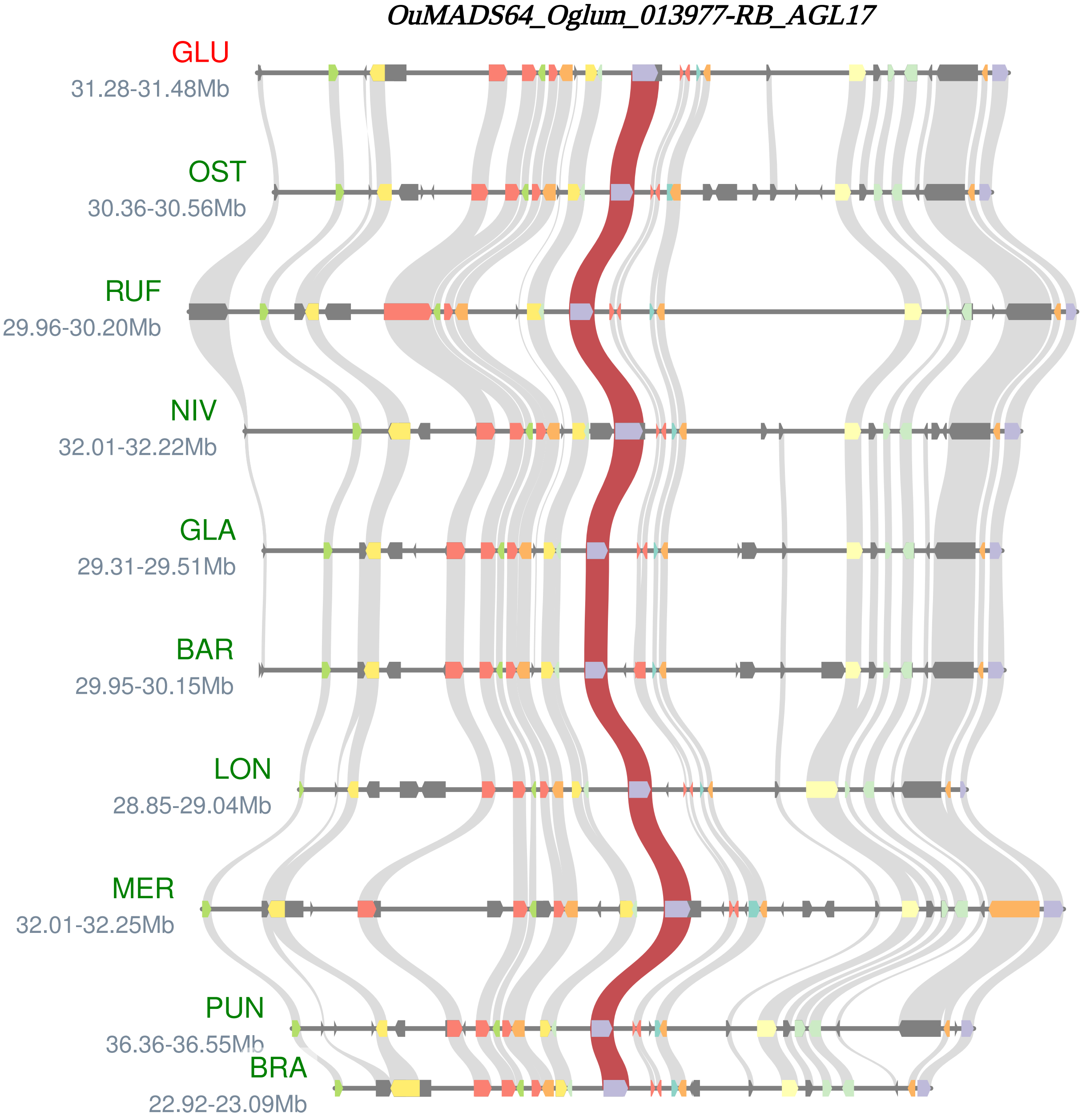
OuMADS64_Oglum_013977-RB_AGL17
GLU
31.28-31.48Mb
OST
30.36-30.56Mb
RUF
29.96-30.20Mb
NIV
32.01-32.22Mb
GLA
29.31-29.51Mb
BAR
29.95-30.15Mb
LON
28.85-29.04Mb
MER
32.01-32.25Mb
PUN
36.36-36.55Mb
BRA
22.92-23.09Mb

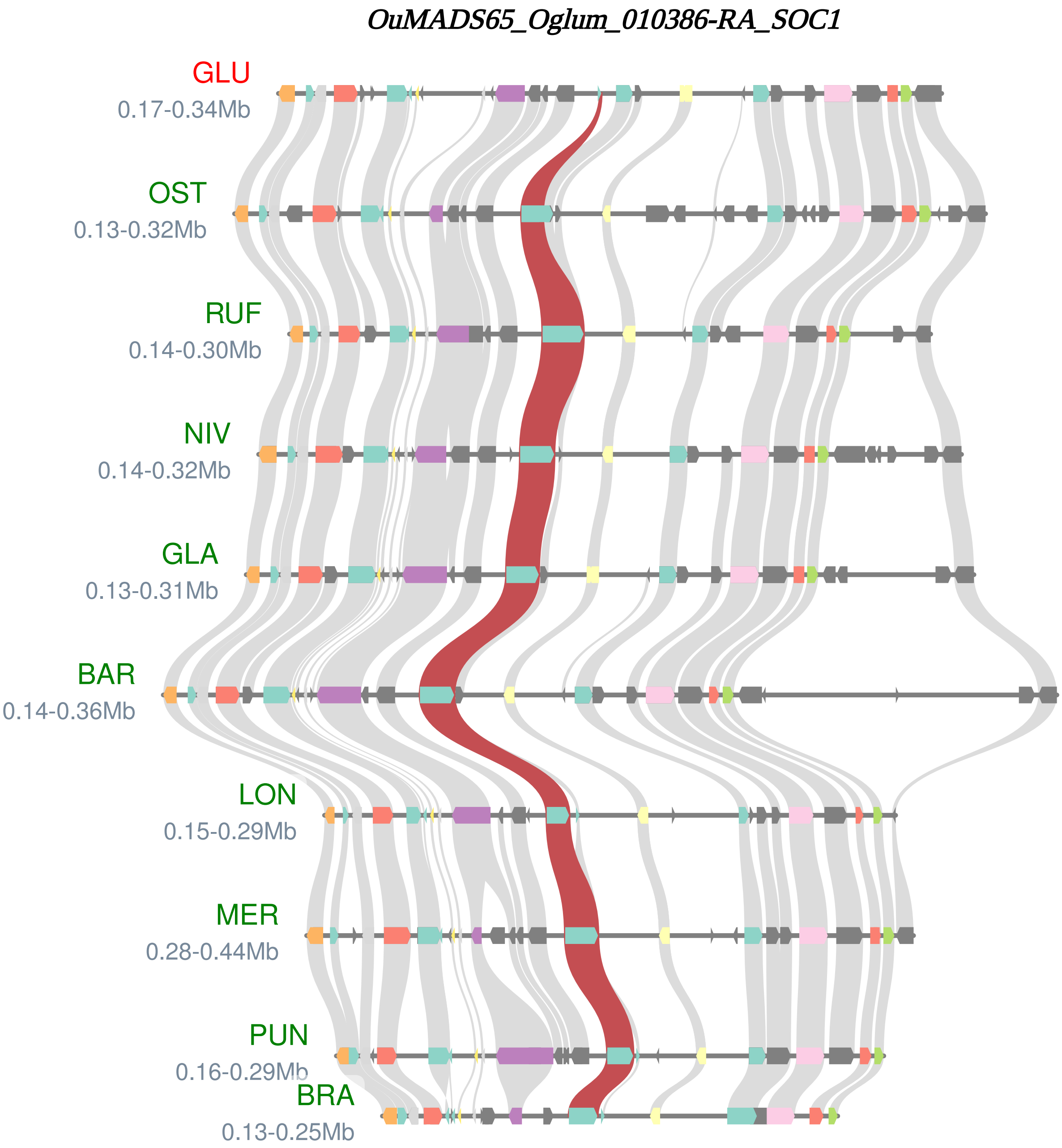

OuMADS65_Oglum_010386-RA_SOC1
GLU
0.17-0.34Mb
OST
0.13-0.32Mb
RUF
0.14-0.30Mb
NIV
0.14-0.32Mb
GLA
0.13-0.31Mb
BAR
0.14-0.36Mb
LON
0.15-0.29Mb
MER
0.28-0.44Mb
PUN
0.16-0.29Mb
BRA
0.13-0.25Mb

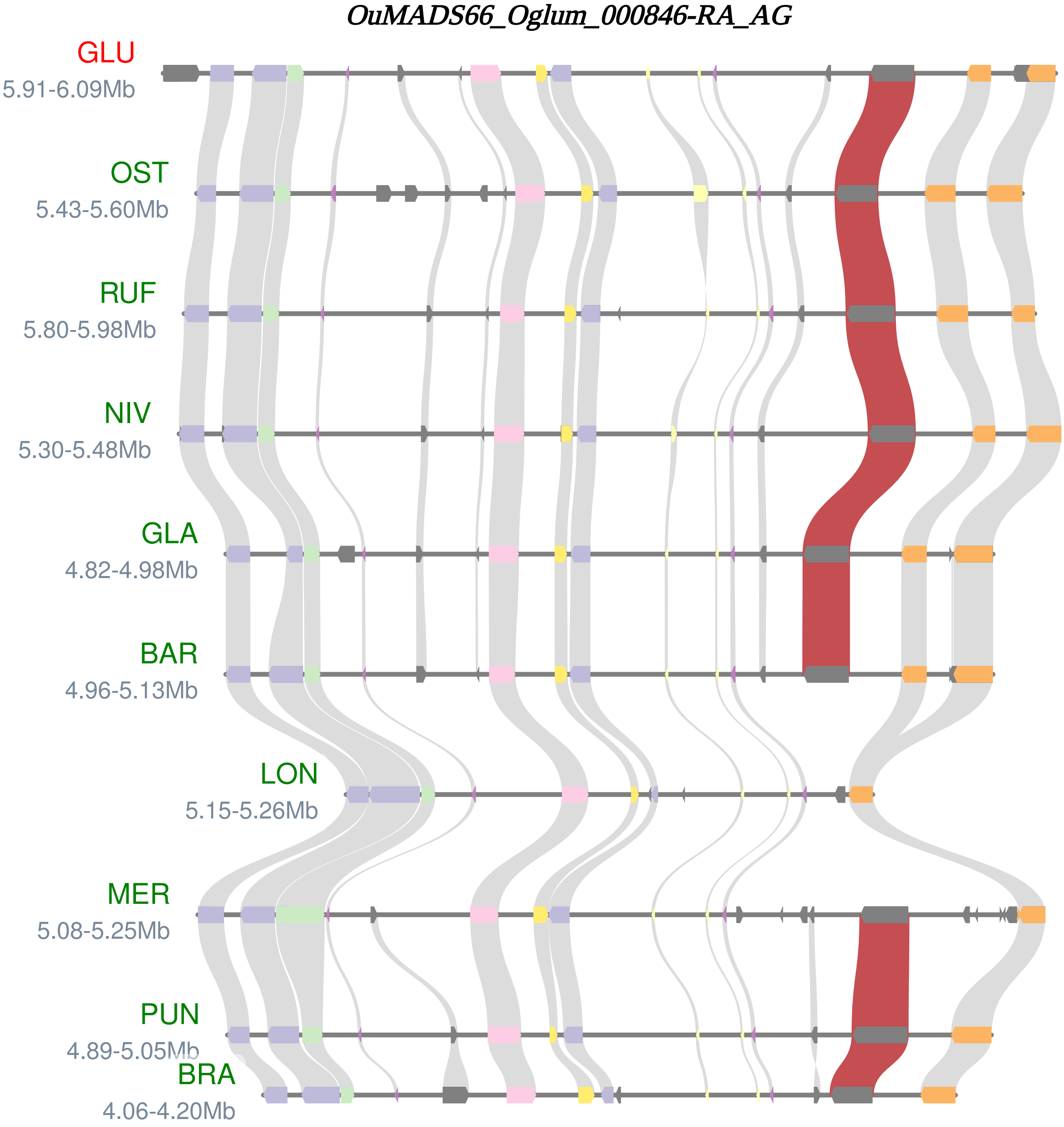
OuMADS66_Oglum_000846-RA_AG
GLU
5.91-6.09Mb
OST
5.43-5.60Mb
RUF
5.80-5.98Mb
NIV
5.30-5.48Mb
GLA
4.82-4.98Mb
BAR
4.96-5.13Mb
LON
5.15-5.26Mb
MER
5.08-5.25Mb
PUN
4.89-5.05Mb
BRA
4.06-4.20Mb

OuMADS67_Oglum_019565-RA_Mα

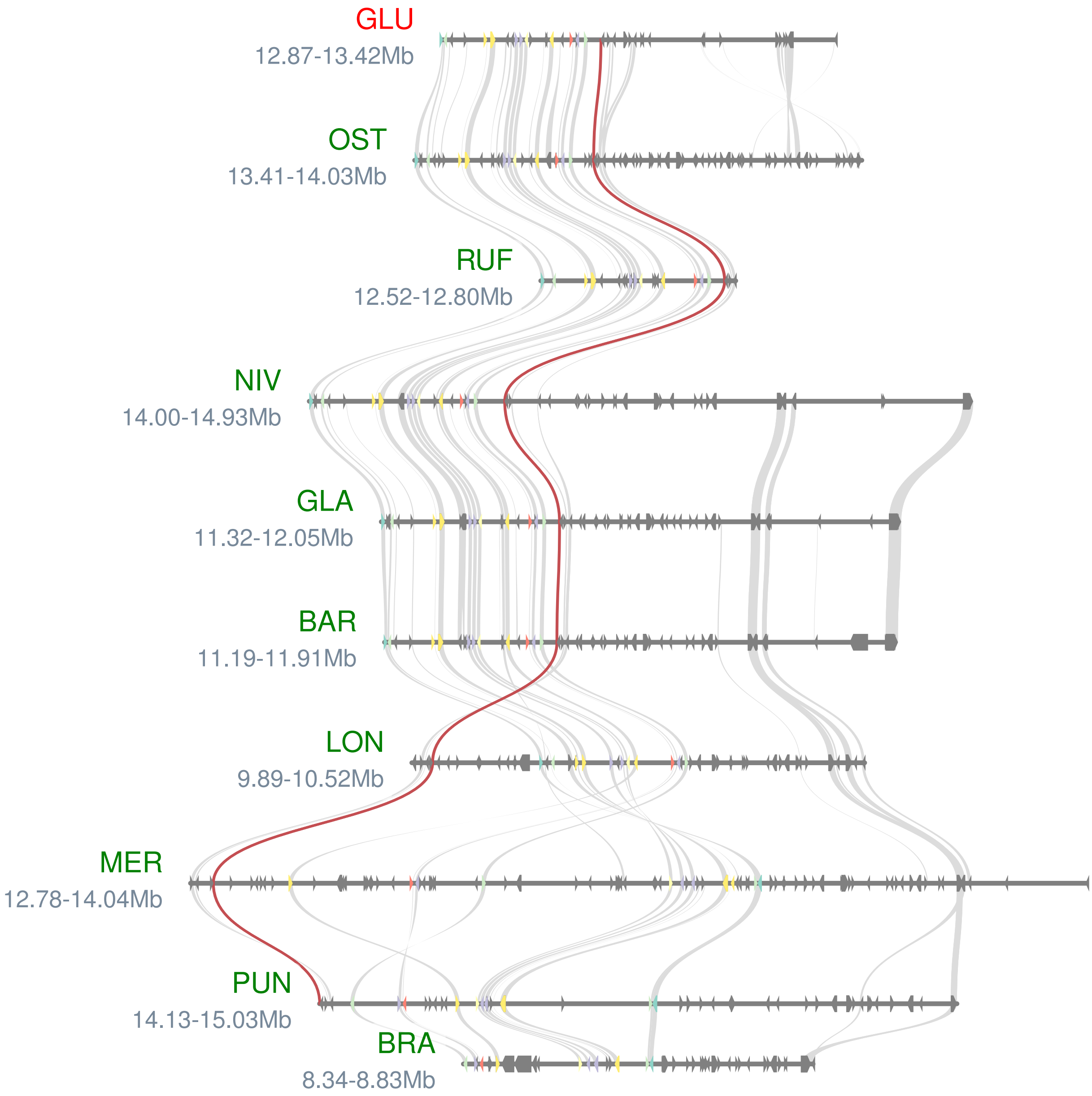

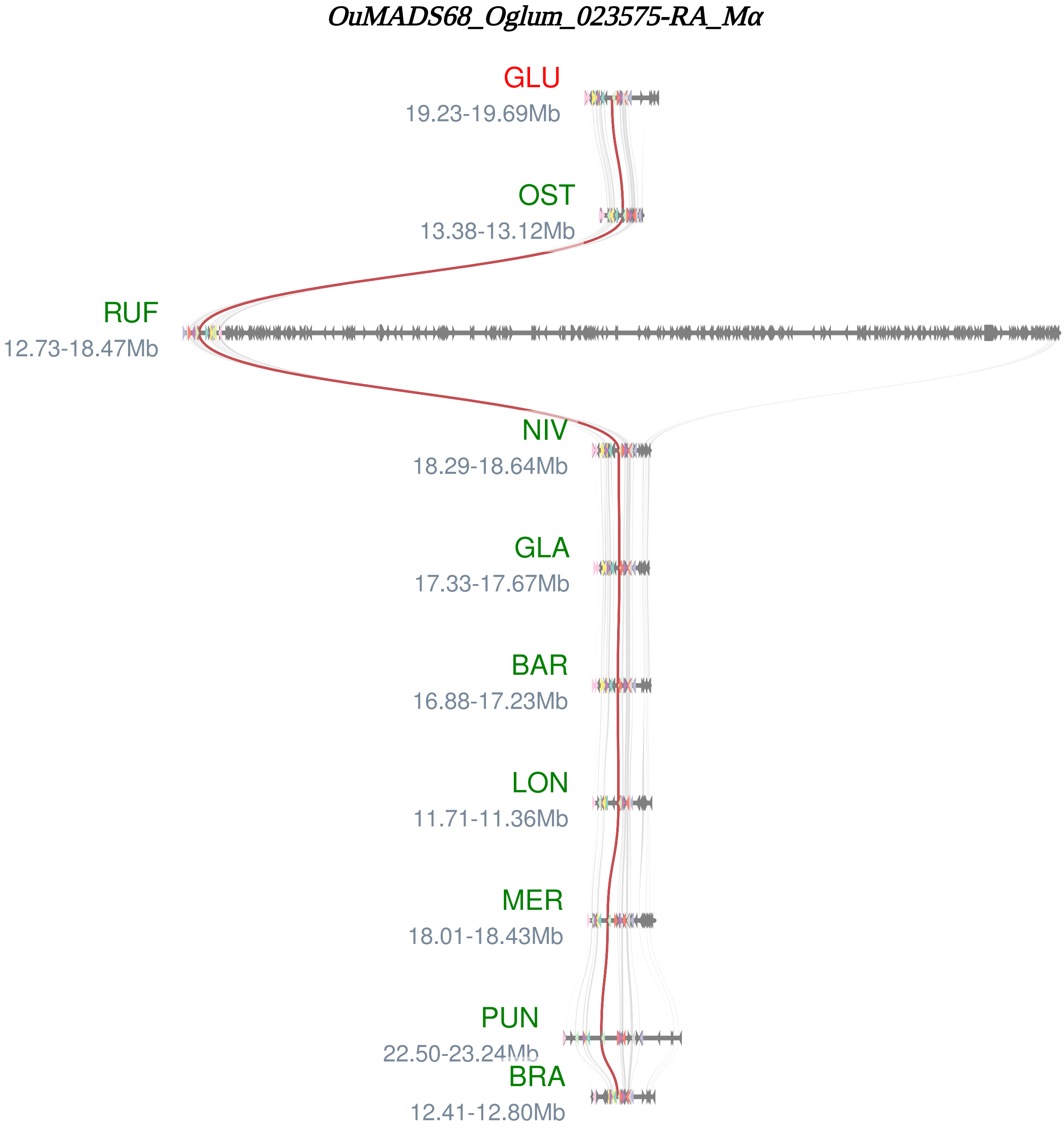
OuMADS68_Oglum_023575-RA_Mα
GLU
19.23-19.69Mb
OST
13.38-13.12Mb
RUF
12.73-18.47Mb
NIV
18.29-18.64Mb
GLA
17.33-17.67Mb
BAR
16.88-17.23Mb
LON
11.71-11.36Mb
MER
18.01-18.43Mb
PUN
22.50-23.24Mb
BRA
12.41-12.80Mb

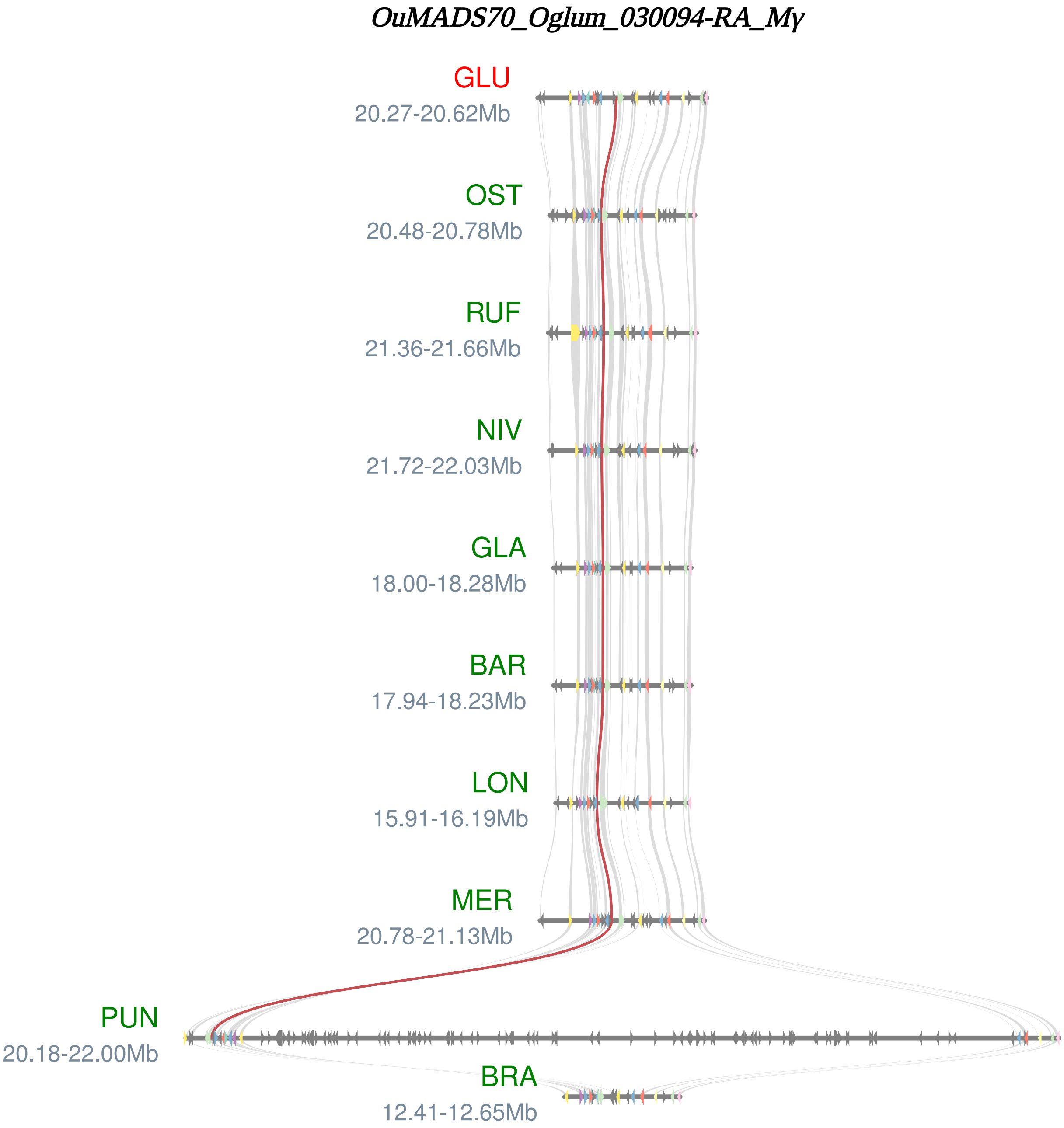

OuMADS70_Oglum_030094-RA_Mγ
GLU
20.27-20.62Mb
OST
20.48-20.78Mb
RUF
21.36-21.66Mb
NIV
21.72-22.03Mb
GLA
18.00-18.28Mb
BAR
17.94-18.23Mb
LON
15.91-16.19Mb
MER
20.78-21.13Mb
PUN
20.18-22.00Mb
BRA
12.41-12.65Mb

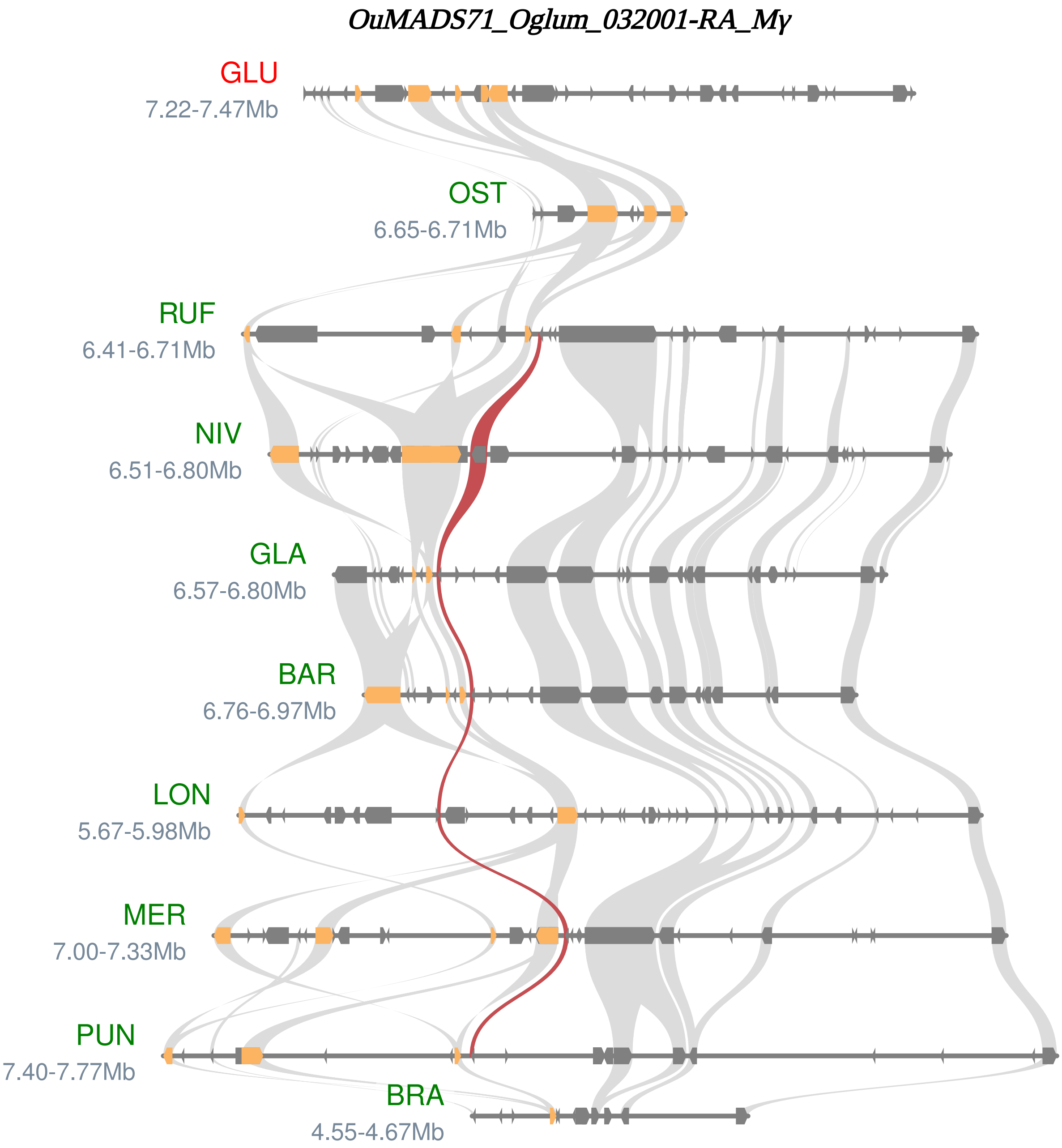
OuMADS71_Oglum_032001-RA_Mγ
GLU
7.22-7.47Mb
OST
6.65-6.71Mb
RUF
6.41-6.71Mb
NIV
6.51-6.80Mb
GLA
6.57-6.80Mb
BAR
6.76-6.97Mb
LON
5.67-5.98Mb
MER
7.00-7.33Mb
PUN
7.40-7.77Mb
BRA
4.55-4.67Mb

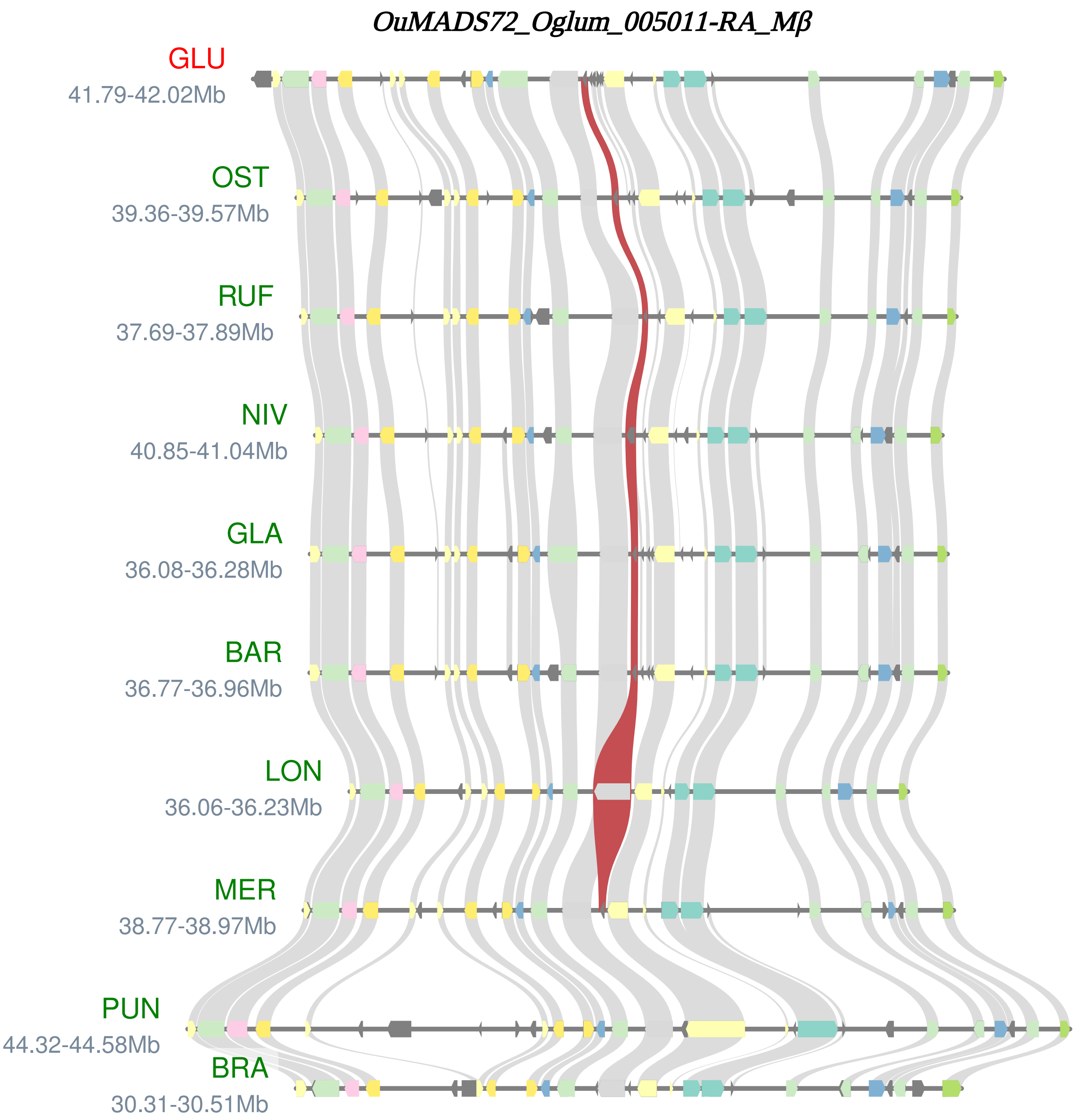
OuMADS72_Oglum_005011-RA_Mβ
GLU
41.79-42.02Mb
OST
39.36-39.57Mb
RUF
37.69-37.89Mb
NIV
40.85-41.04Mb
GLA
36.08-36.28Mb
BAR
36.77-36.96Mb
LON
36.06-36.23Mb
MER
38.77-38.97Mb
PUN
44.32-44.58Mb
BRA
30.31-30.51Mb

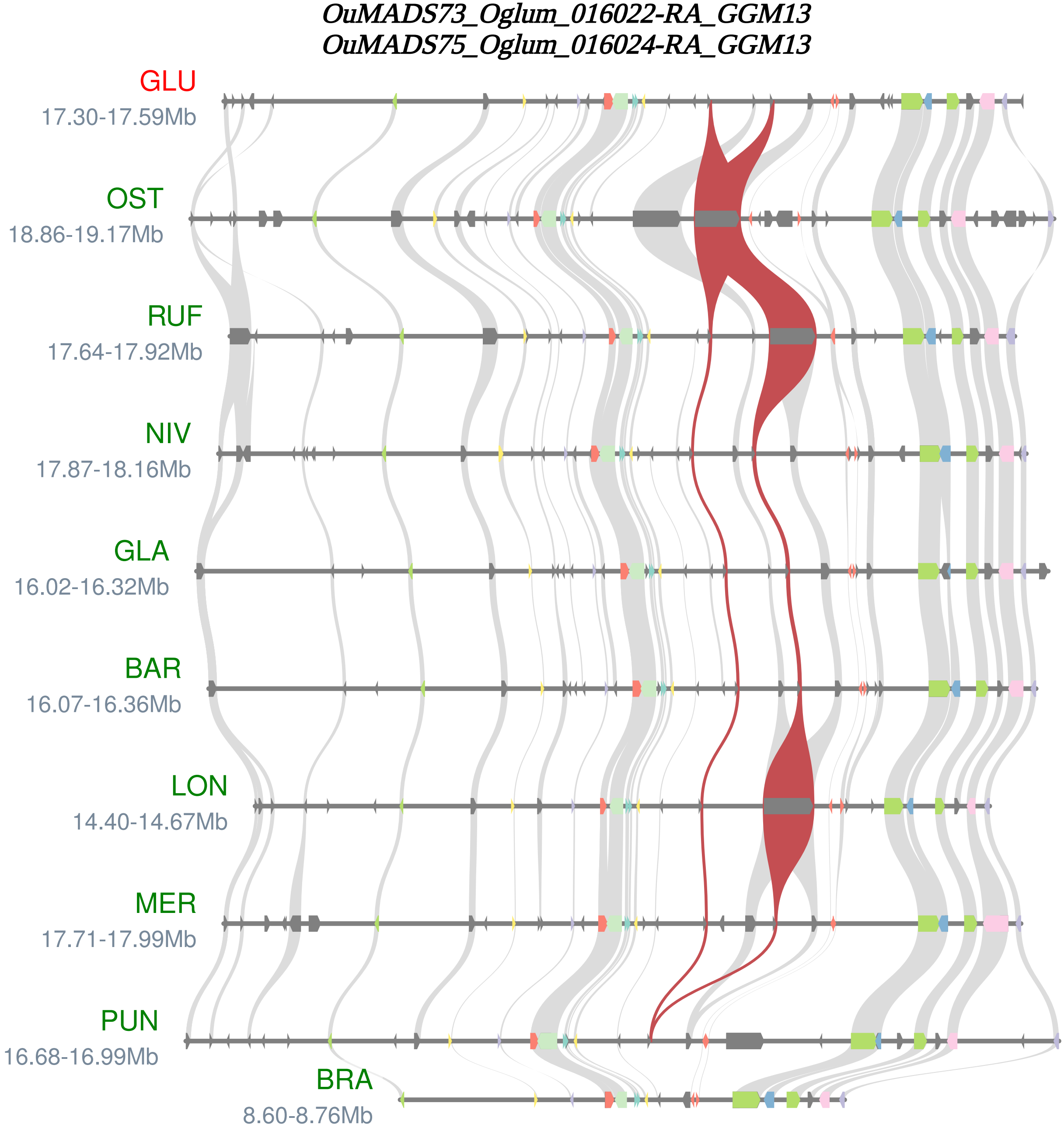
OuMADS73_Oglum_016022-RA_GGM13
OuMADS75_Oglum_016024-RA_GGM13
GLU
17.30-17.59Mb
OST
18.86-19.17Mb
RUF
17.64-17.92Mb
NIV
17.87-18.16Mb
GLA
16.02-16.32Mb
BAR
16.07-16.36Mb
LON
14.40-14.67Mb
MER
17.71-17.99Mb
PUN
16.68-16.99Mb
BRA
8.60-8.76Mb

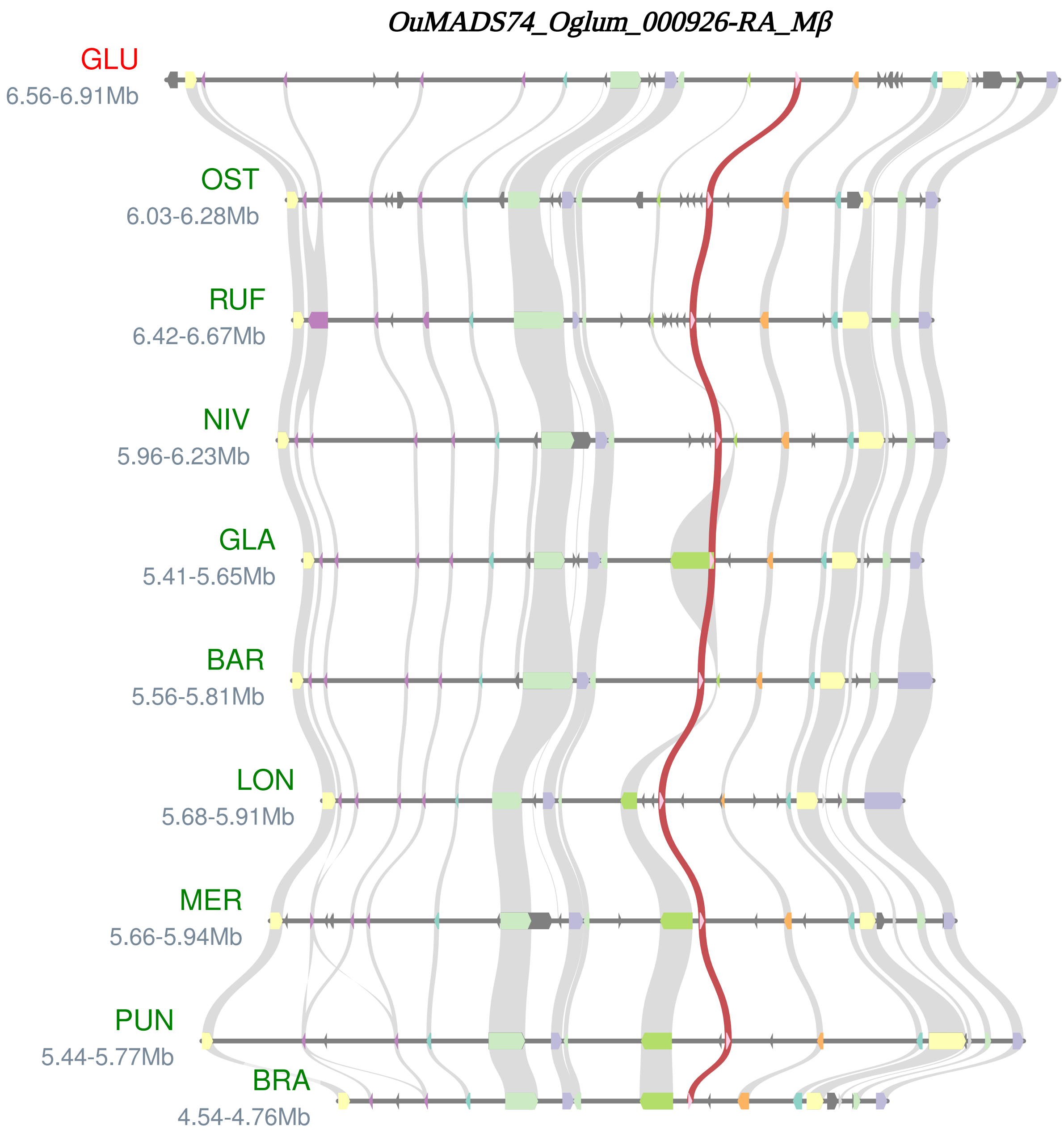
OuMADS74_Oglum_000926-RA_Mβ
GLU
6.56-6.91Mb
OST
6.03-6.28Mb
RUF
6.42-6.67Mb
NIV
5.96-6.23Mb
GLA
5.41-5.65Mb
BAR
5.56-5.81Mb
LON
5.68-5.91Mb
MER
5.66-5.94Mb
PUN
5.44-5.77Mb
BRA
4.54-4.76Mb

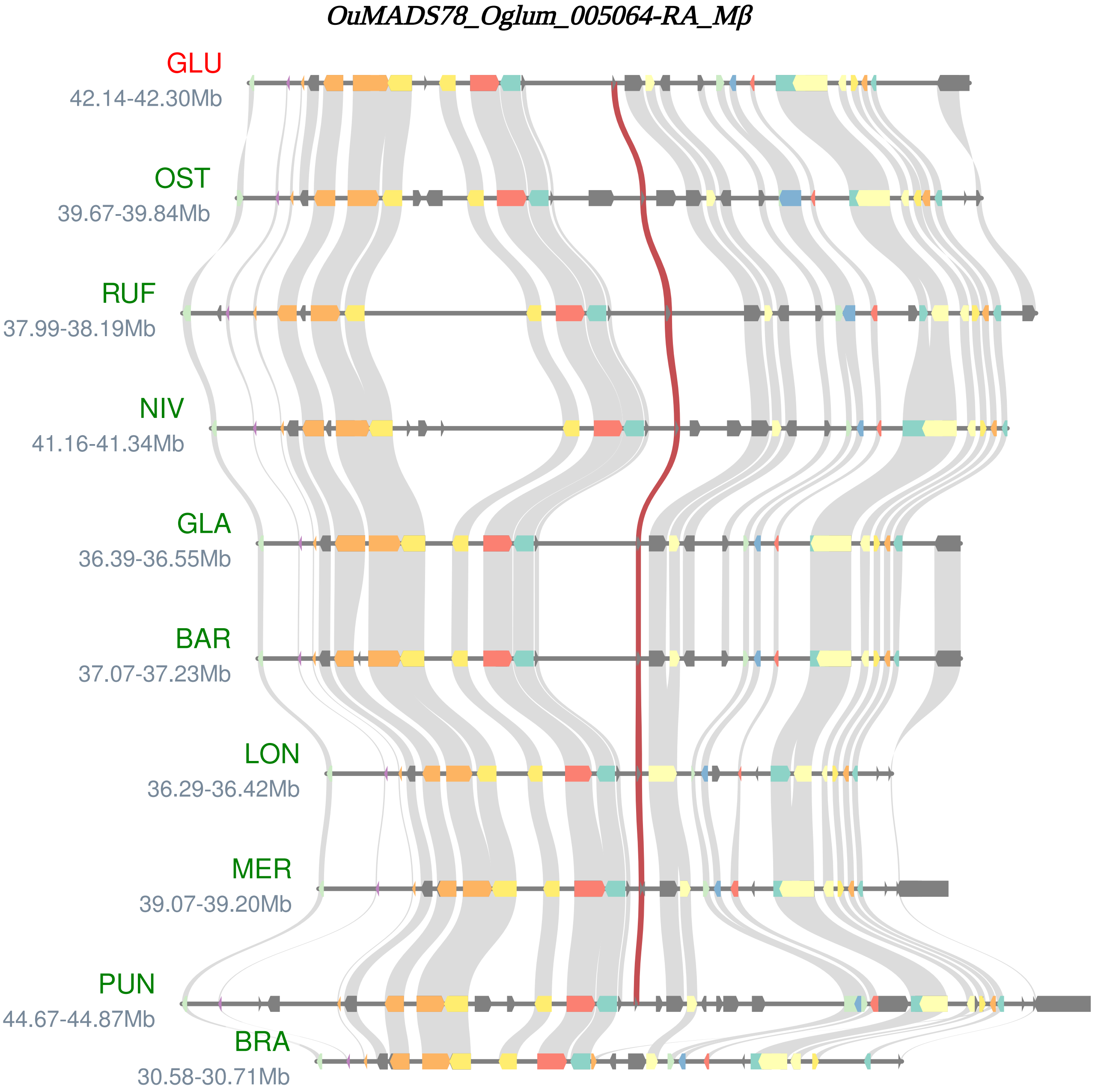
OuMADS78_Oglum_005064-RA_Mβ
GLU
42.14-42.30Mb
OST
39.67-39.84Mb
RUF
37.99-38.19Mb
NIV
41.16-41.34Mb
GLA
36.39-36.55Mb
BAR
37.07-37.23Mb
LON
36.29-36.42Mb
MER
39.07-39.20Mb
PUN
44.67-44.87Mb
BRA
30.58-30.71Mb

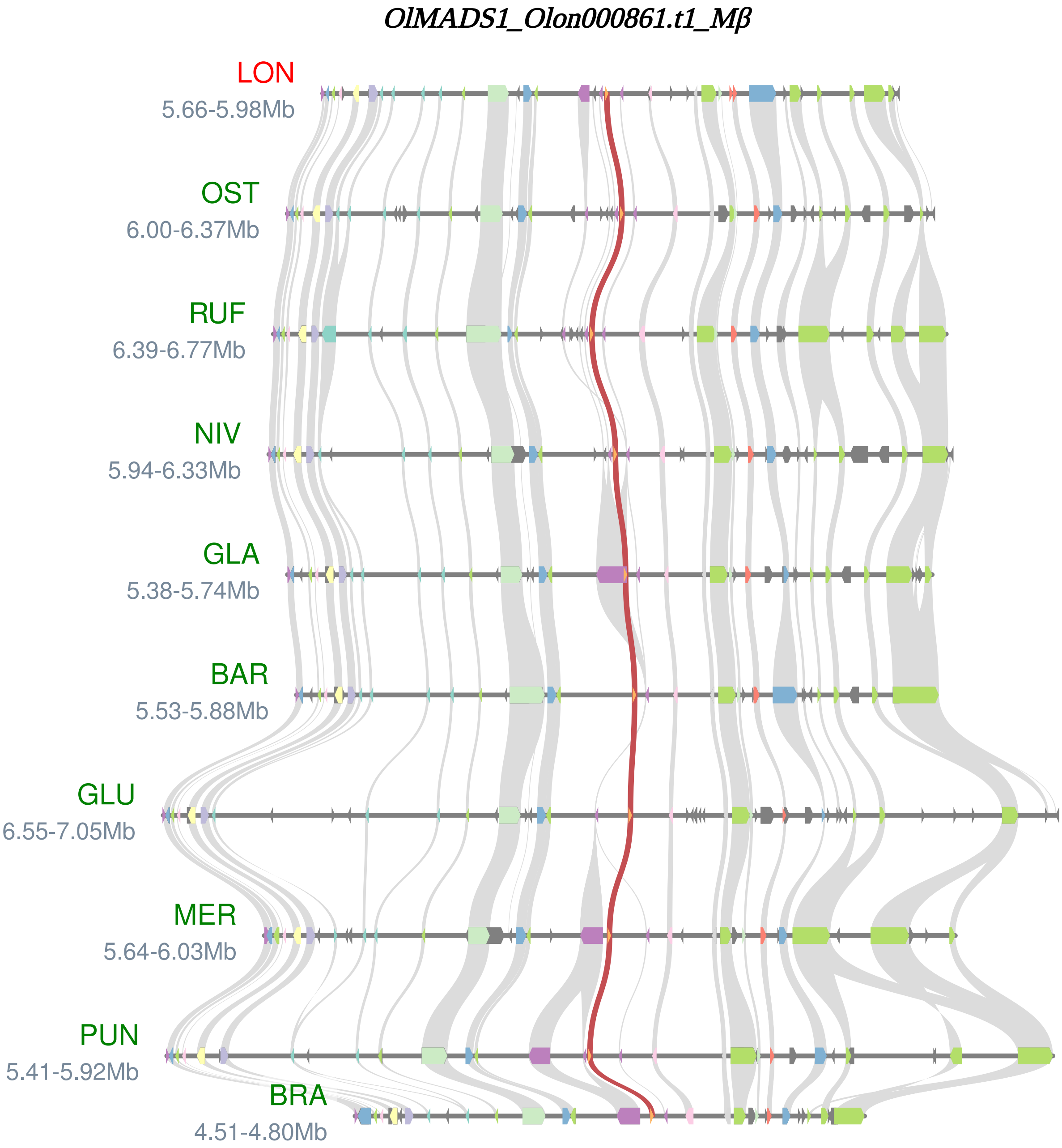

OlMADS1_Olon000861.t1_Mβ
LON
5.66-5.98Mb
OST
6.00-6.37Mb
RUF
6.39-6.77Mb
NIV
5.94-6.33Mb
GLA
5.38-5.74Mb
BAR
5.53-5.88Mb
GLU
6.55-7.05Mb
MER
5.64-6.03Mb
PUN
5.41-5.92Mb
BRA
4.51-4.80Mb

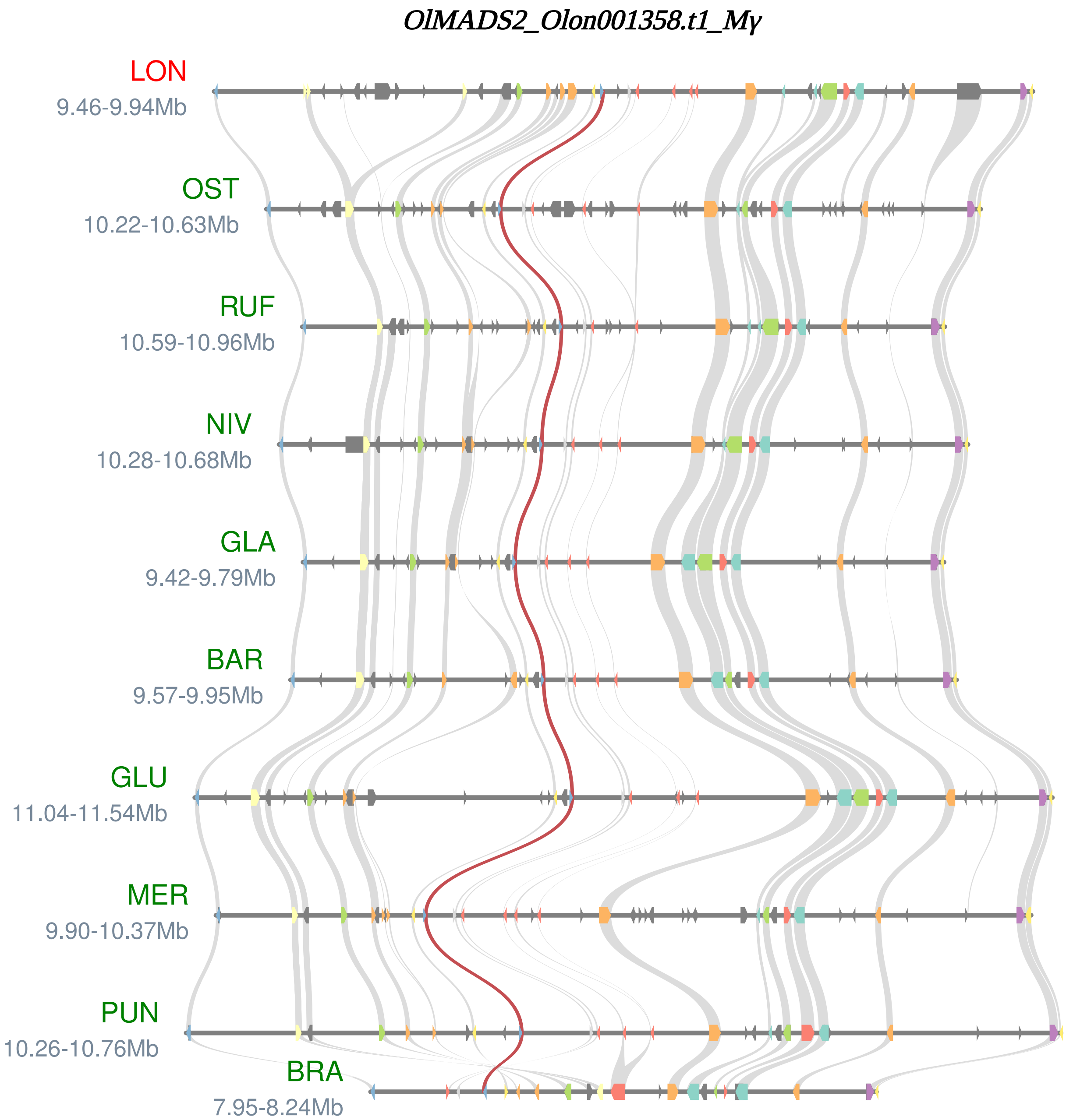

OlMADS2_Olon001358.t1_Mγ
LON
9.46-9.94Mb
OST
10.22-10.63Mb
RUF
10.59-10.96Mb
NIV
10.28-10.68Mb
GLA
9.42-9.79Mb
BAR
9.57-9.95Mb
GLU
11.04-11.54Mb
MER
9.90-10.37Mb
PUN
10.26-10.76Mb
BRA
7.95-8.24Mb

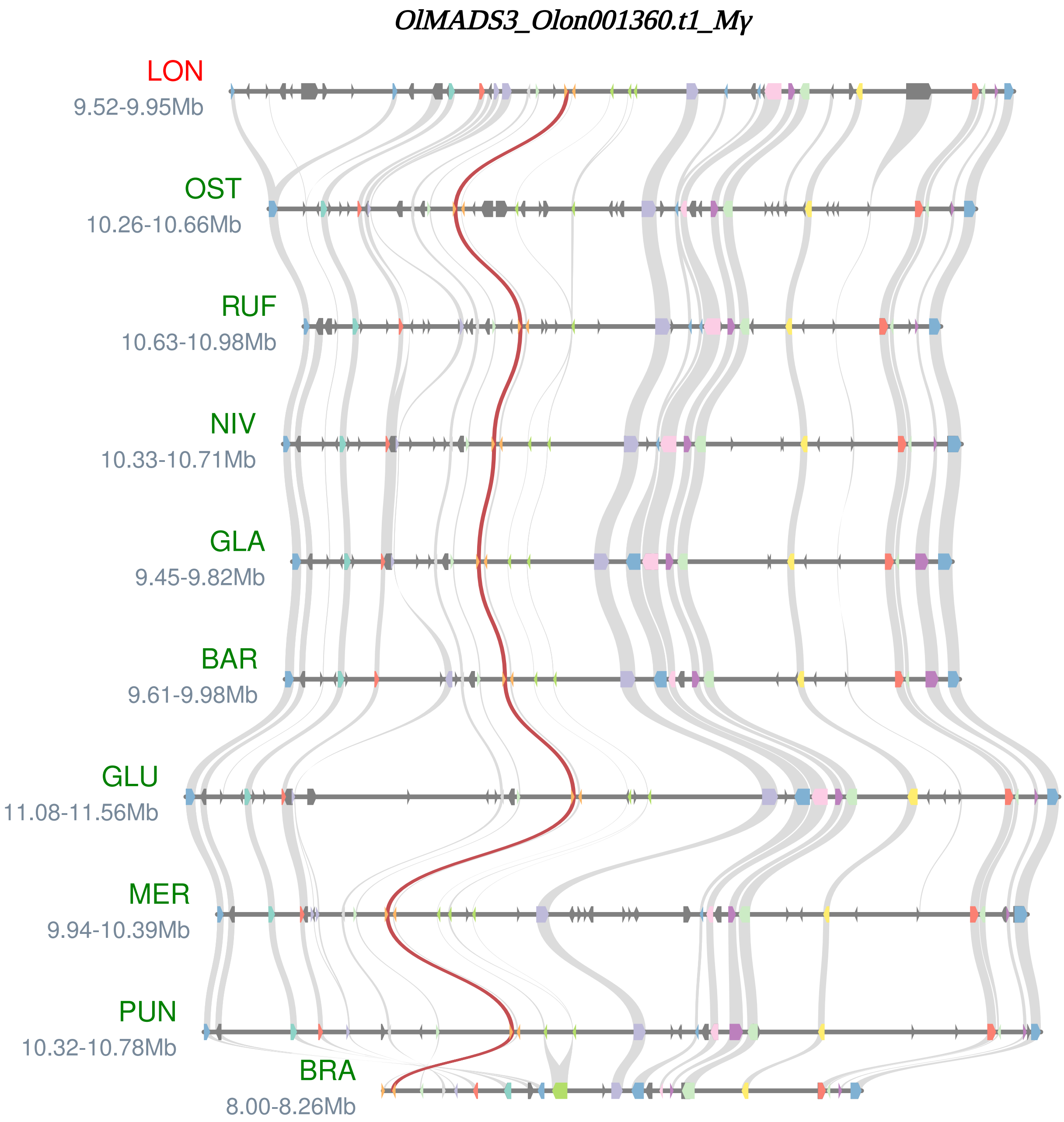

OlMADS3_Olon001360.t1_Mγ
LON
9.52-9.95Mb
OST
10.26-10.66Mb
RUF
10.63-10.98Mb
NIV
10.33-10.71Mb
GLA
9.45-9.82Mb
BAR
9.61-9.98Mb
GLU
11.08-11.56Mb
MER
9.94-10.39Mb
PUN
10.32-10.78Mb
BRA
8.00-8.26Mb

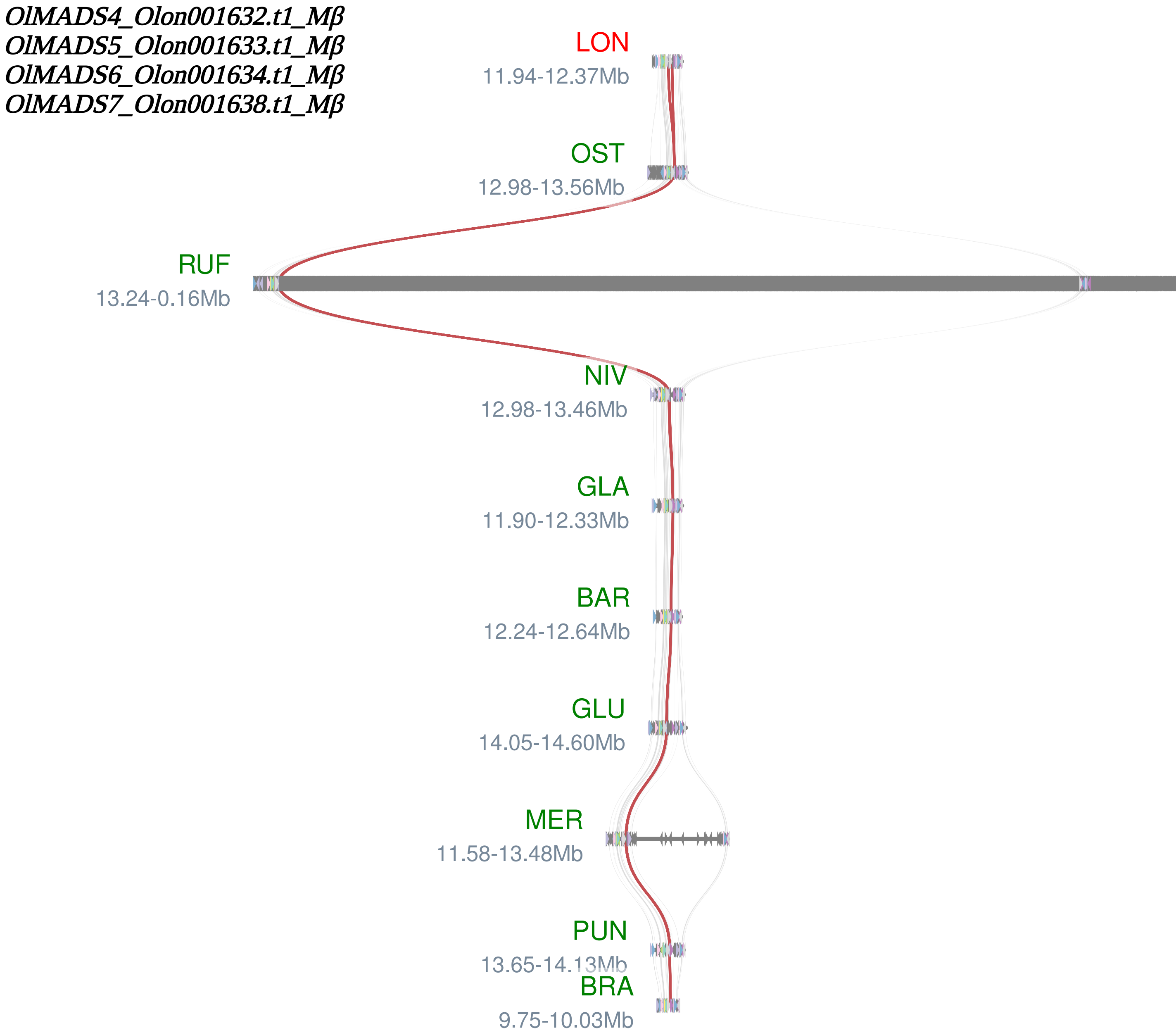
OlMADS4_Olon001632.t1_Mβ
OlMADS5_Olon001633.t1_Mβ
OlMADS6_Olon001634.t1_Mβ
OlMADS7_Olon001638.t1_Mβ
LON
11.94-12.37Mb
OST
12.98-13.56Mb
RUF
13.24-0.16Mb
NIV
12.98-13.46Mb
GLA
11.90-12.33Mb
BAR
12.24-12.64Mb
GLU
14.05-14.60Mb
MER
11.58-13.48Mb
PUN
13.65-14.13Mb
BRA
9.75-10.03Mb

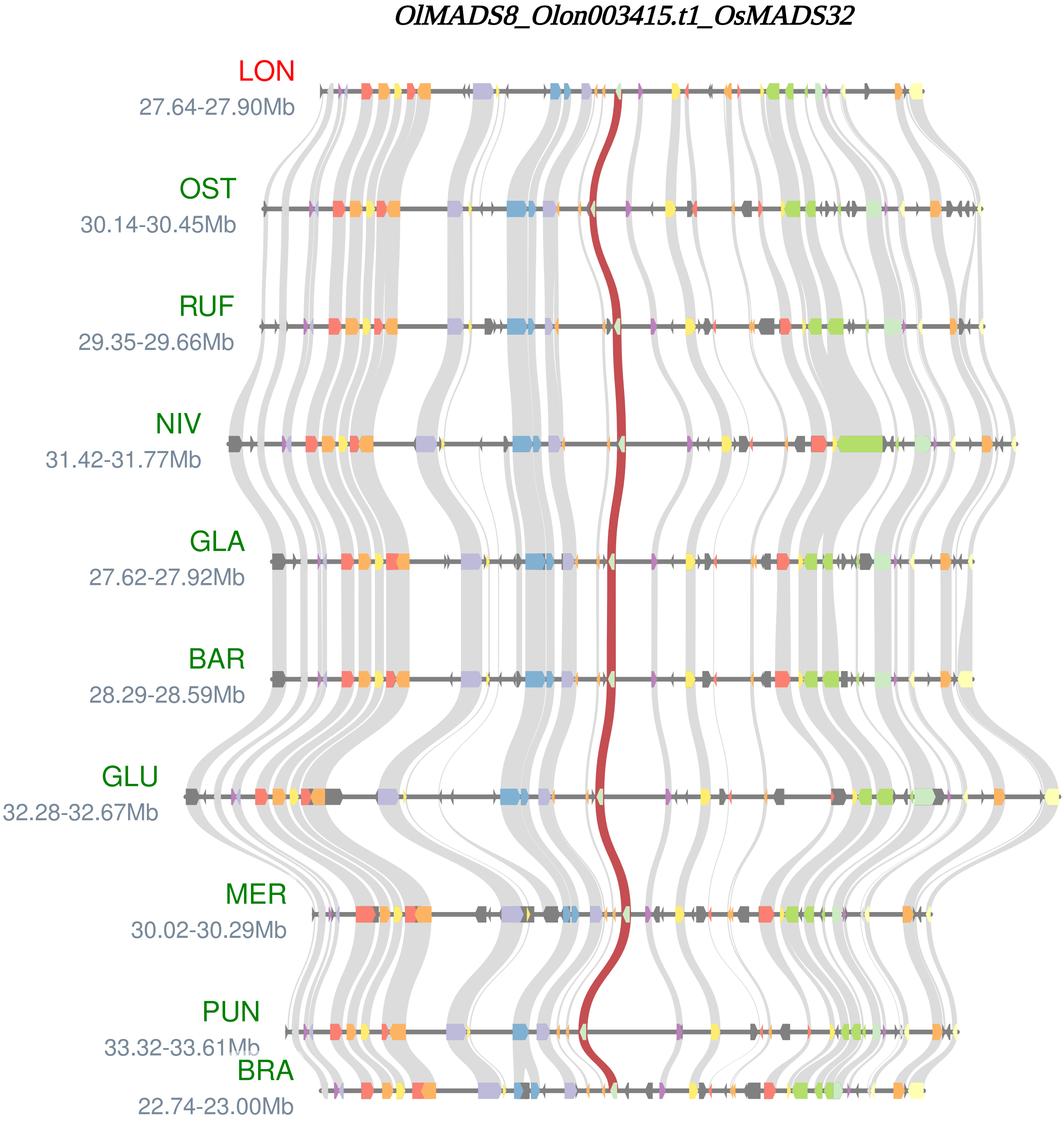
OlMADS8_Olon003415.t1_OsMADS32
LON
27.64-27.90Mb
OST
30.14-30.45Mb
RUF
29.35-29.66Mb
NIV
31.42-31.77Mb
GLA
27.62-27.92Mb
BAR
28.29-28.59Mb
GLU
32.28-32.67Mb
MER
30.02-30.29Mb
PUN
33.32-33.61Mb
BRA
22.74-23.00Mb

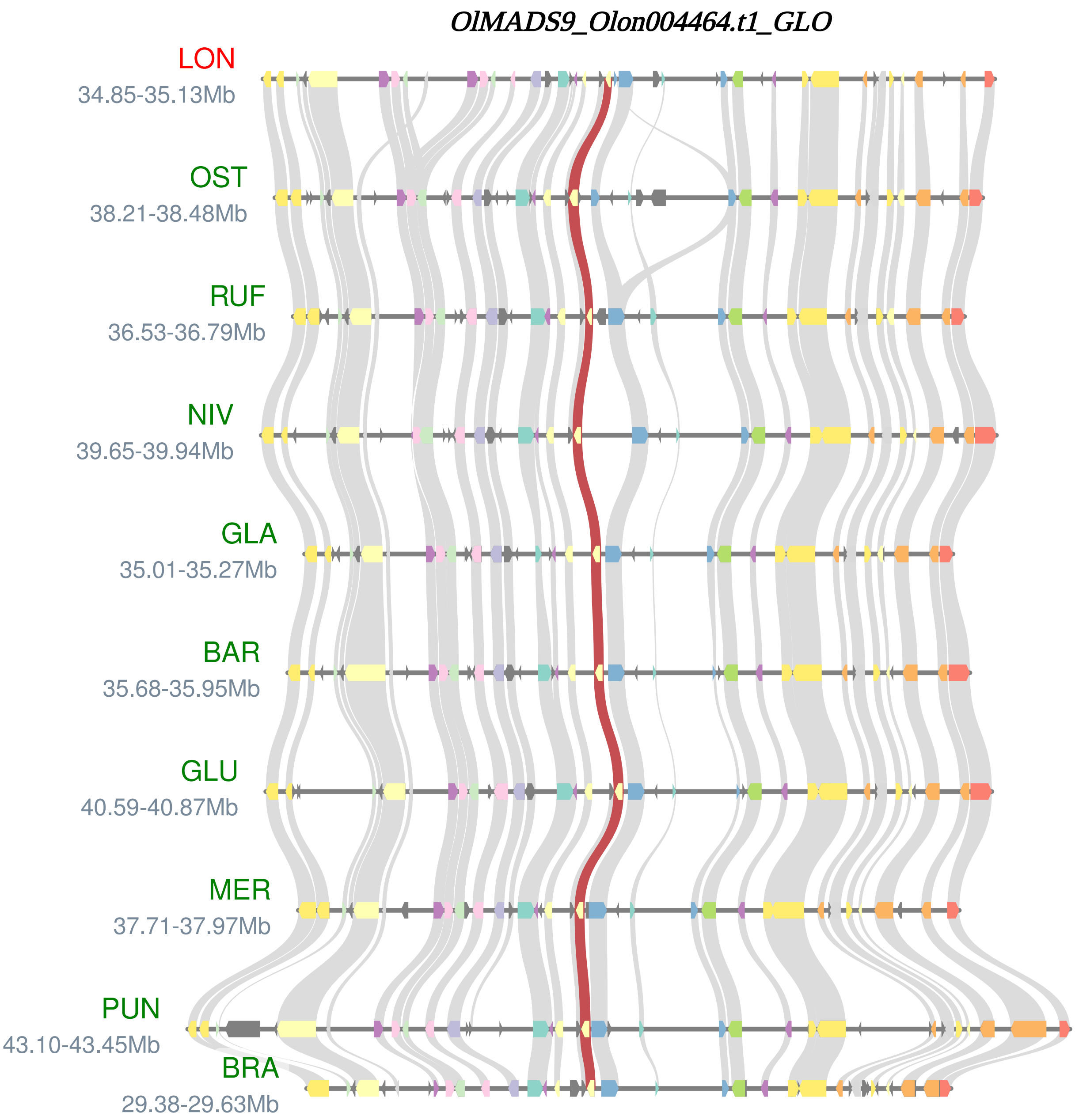
OlMADS9_Olon004464.t1_GLO
LON
34.85-35.13Mb
OST
38.21-38.48Mb
RUF
36.53-36.79Mb
NIV
39.65-39.94Mb
GLA
35.01-35.27Mb
BAR
35.68-35.95Mb
GLU
40.59-40.87Mb
MER
37.71-37.97Mb
PUN
43.10-43.45Mb
BRA
29.38-29.63Mb

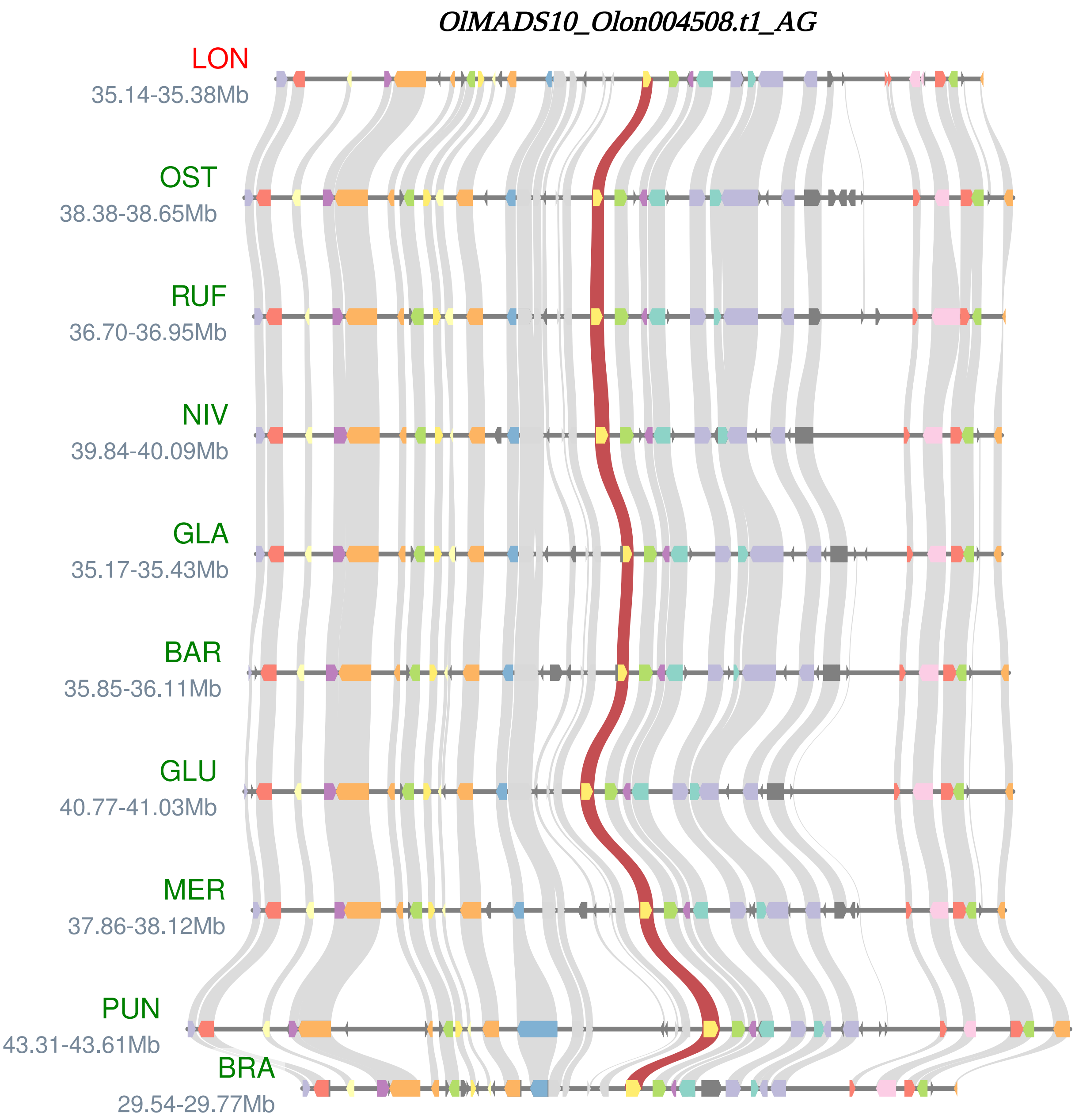

OlMADS10_Olon004508.t1_AG
LON
35.14-35.38Mb
OST
38.38-38.65Mb
RUF
36.70-36.95Mb
NIV
39.84-40.09Mb
GLA
35.17-35.43Mb
BAR
35.85-36.11Mb
GLU
40.77-41.03Mb
MER
37.86-38.12Mb
PUN
43.31-43.61Mb
BRA
29.54-29.77Mb

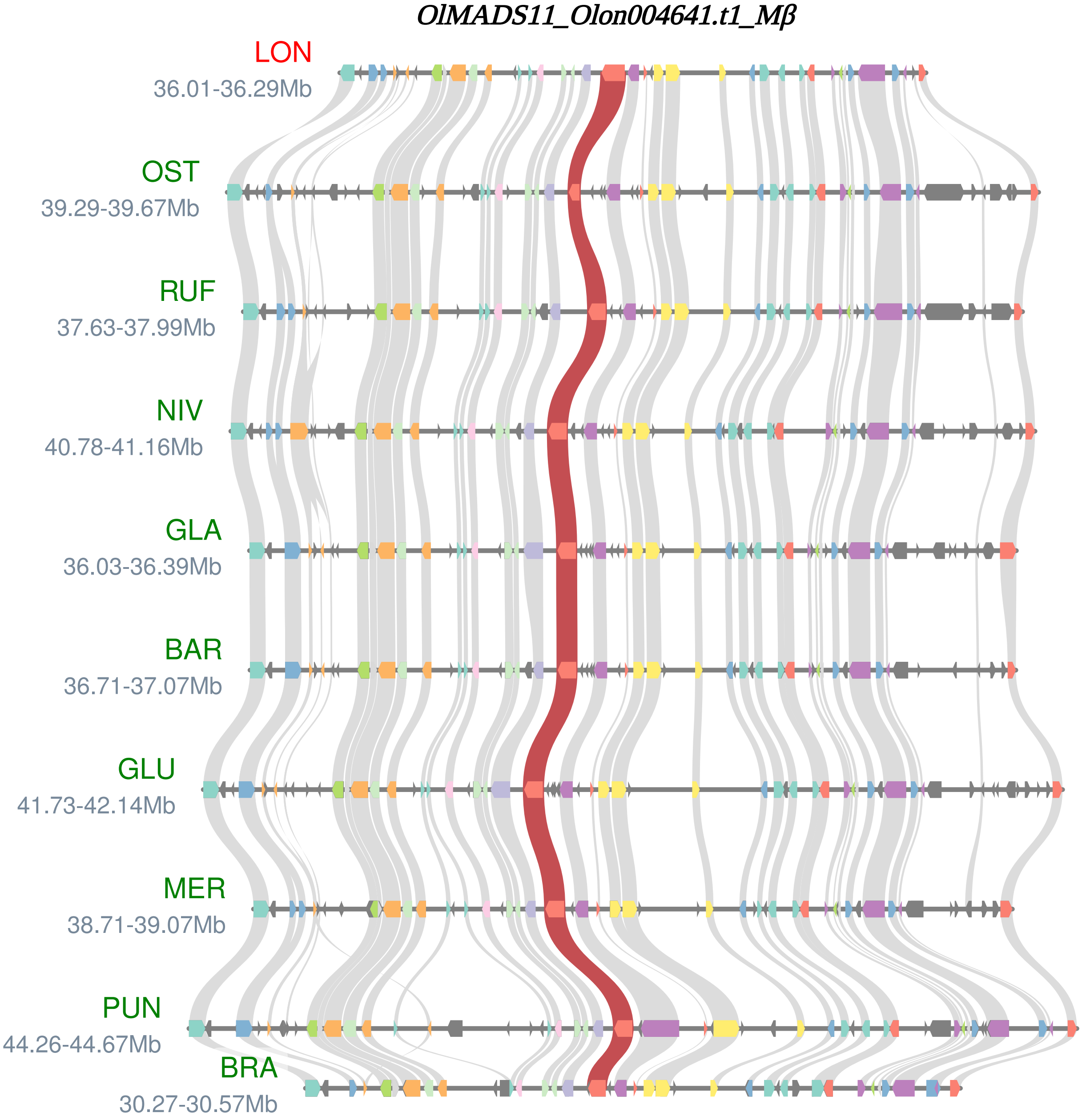

OlMADS11_Olon004641.t1_Mβ
LON
36.01-36.29Mb
OST
39.29-39.67Mb
RUF
37.63-37.99Mb
NIV
40.78-41.16Mb
GLA
36.03-36.39Mb
BAR
36.71-37.07Mb
GLU
41.73-42.14Mb
MER
38.71-39.07Mb
PUN
44.26-44.67Mb
BRA
30.27-30.57Mb

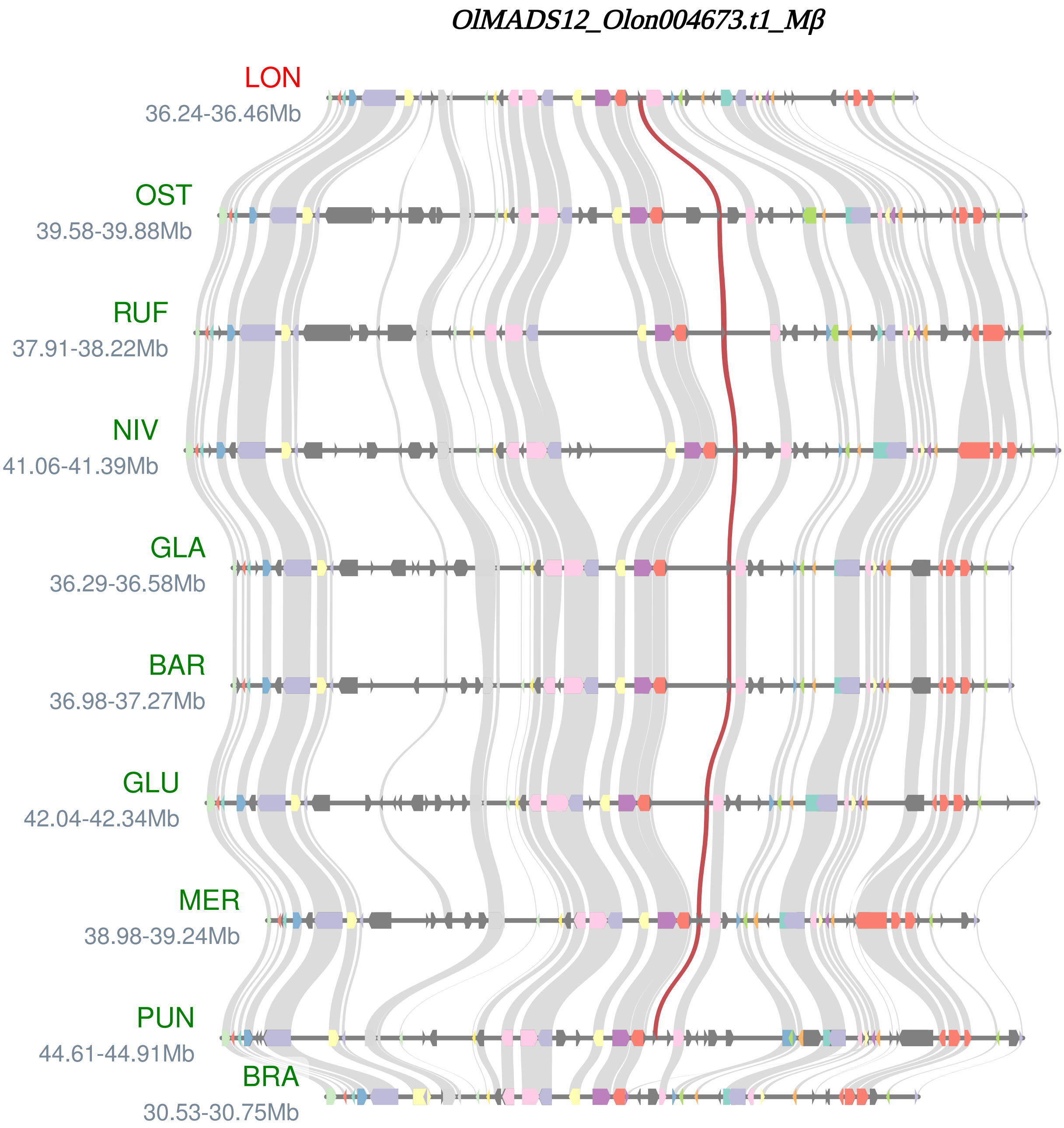
OlMADS12_Olon004673.t1_Mβ
LON
36.24-36.46Mb
OST
39.58-39.88Mb
RUF
37.91-38.22Mb
NIV
41.06-41.39Mb
GLA
36.29-36.58Mb
BAR
36.98-37.27Mb
GLU
42.04-42.34Mb
MER
38.98-39.24Mb
PUN
44.61-44.91Mb
BRA
30.53-30.75Mb

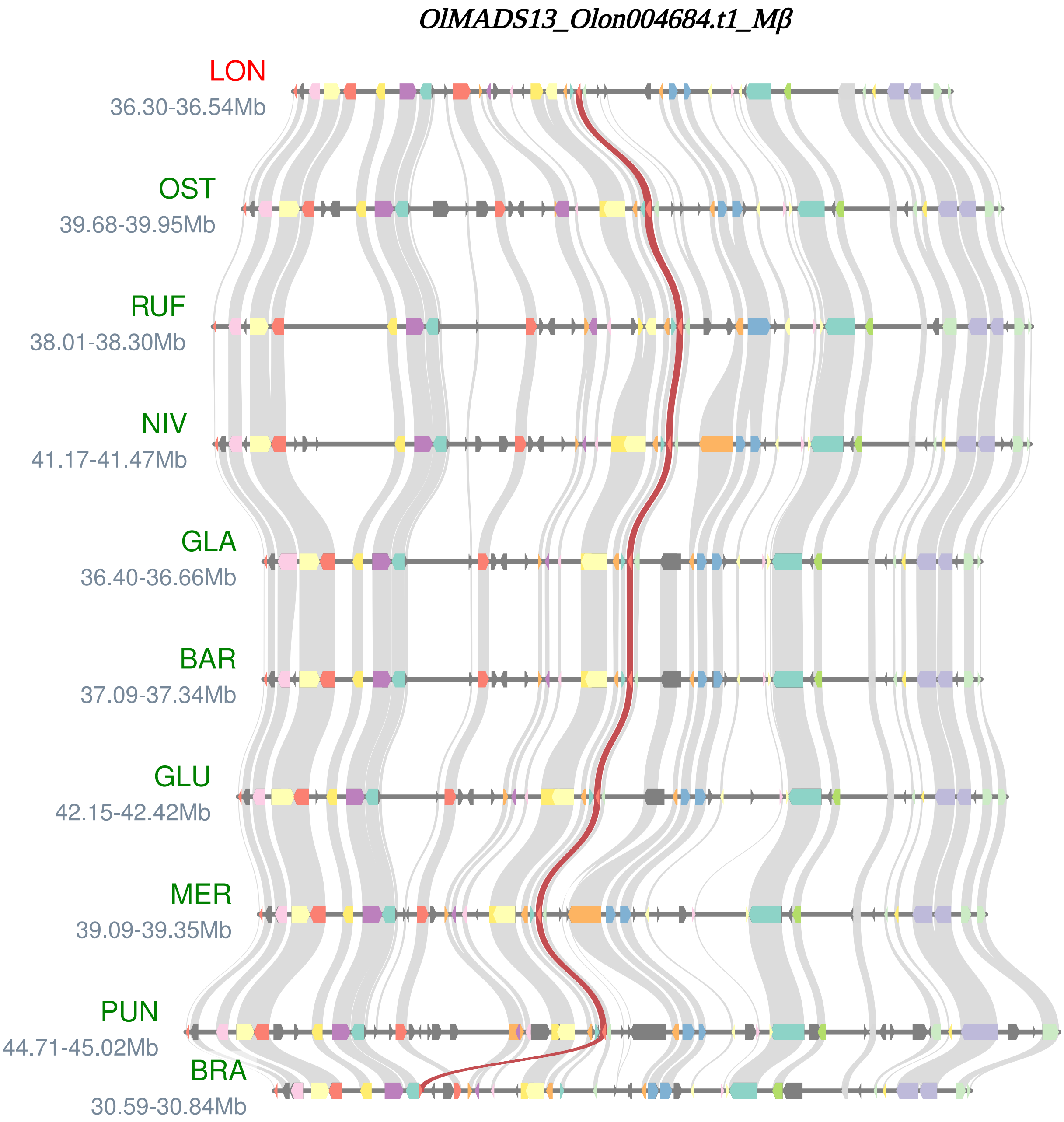

OlMADS13_Olon004684.t1_Mβ
LON
36.30-36.54Mb
OST
39.68-39.95Mb
RUF
38.01-38.30Mb
NIV
41.17-41.47Mb
GLA
36.40-36.66Mb
BAR
37.09-37.34Mb
GLU
42.15-42.42Mb
MER
39.09-39.35Mb
PUN
44.71-45.02Mb
BRA
30.59-30.84Mb

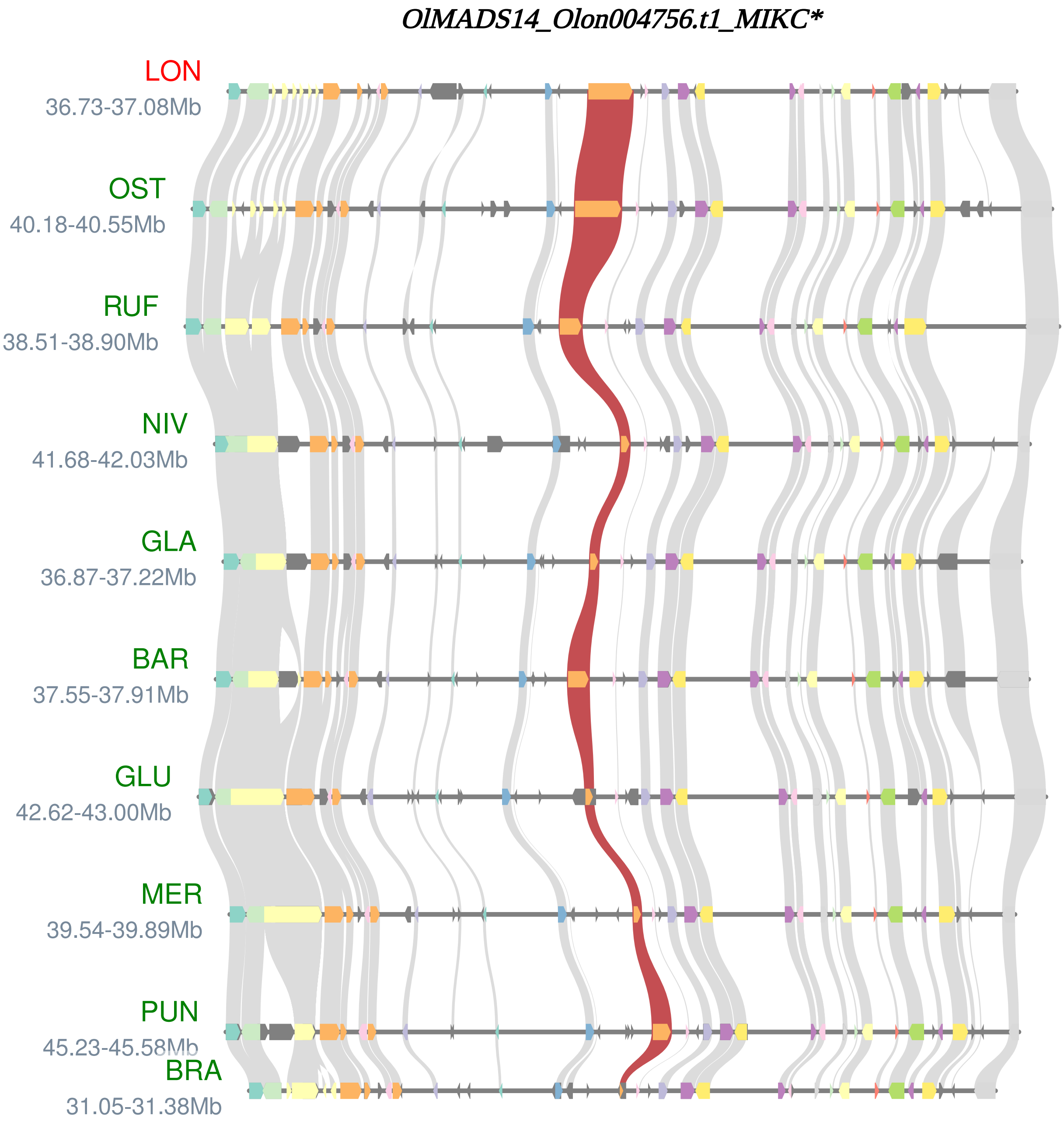
OlMADS14_Olon004756.t1_MIKC*
LON
36.73-37.08Mb
OST
40.18-40.55Mb
RUF
38.51-38.90Mb
NIV
41.68-42.03Mb
GLA
36.87-37.22Mb
BAR
37.55-37.91Mb
GLU
42.62-43.00Mb
MER
39.54-39.89Mb
PUN
45.23-45.58Mb
BRA
31.05-31.38Mb

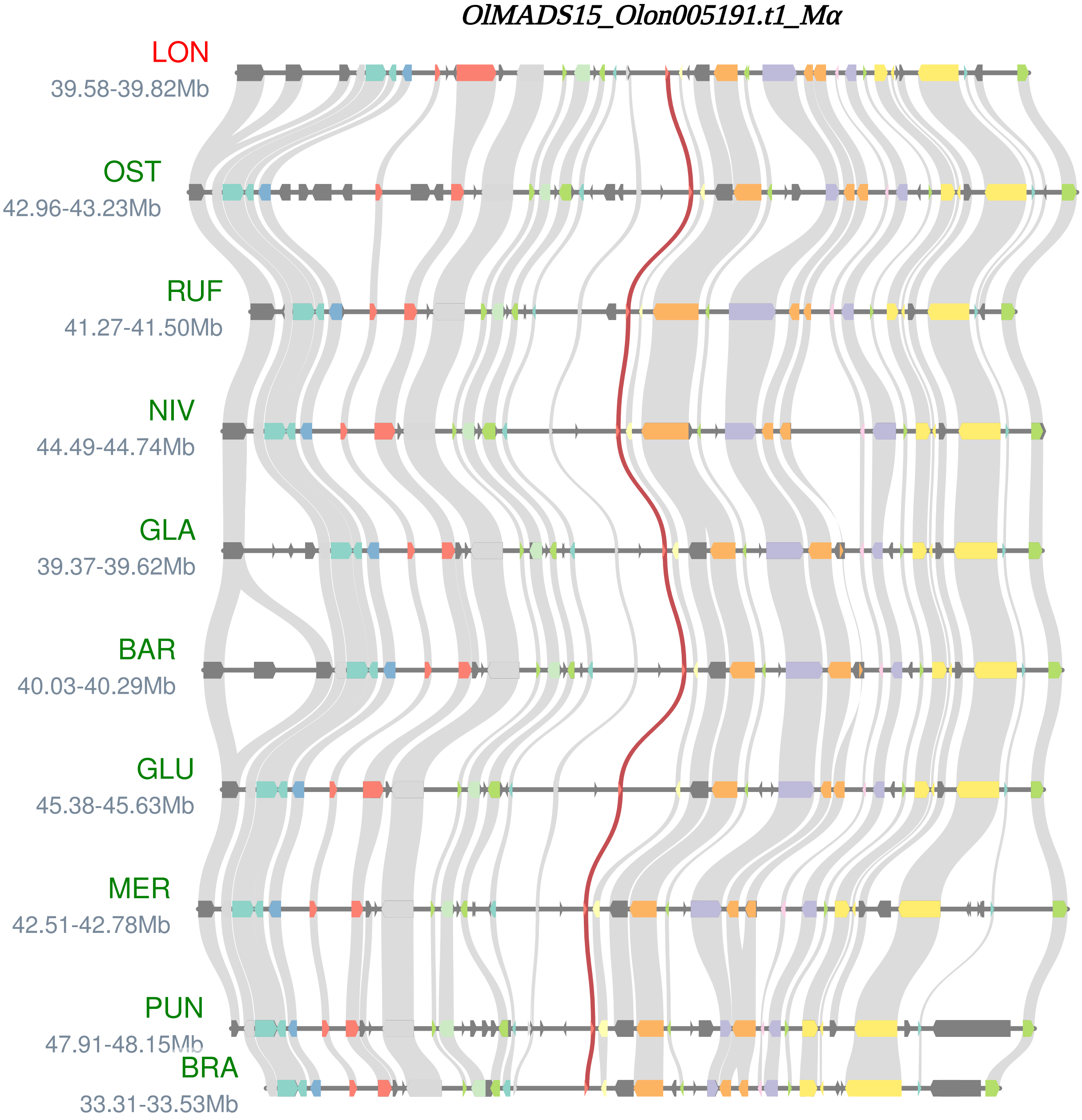
OlMADS15_Olon005191.t1_Mα
LON
39.58-39.82Mb
OST
42.96-43.23Mb
RUF
41.27-41.50Mb
NIV
44.49-44.74Mb
GLA
39.37-39.62Mb
BAR
40.03-40.29Mb
GLU
45.38-45.63Mb
MER
42.51-42.78Mb
PUN
47.91-48.15Mb
BRA
33.31-33.53Mb

OlMADS16_Olon005247.t1_SOC1
(The chromosomal segment in the GLU lacks any detected syntenic genes.)

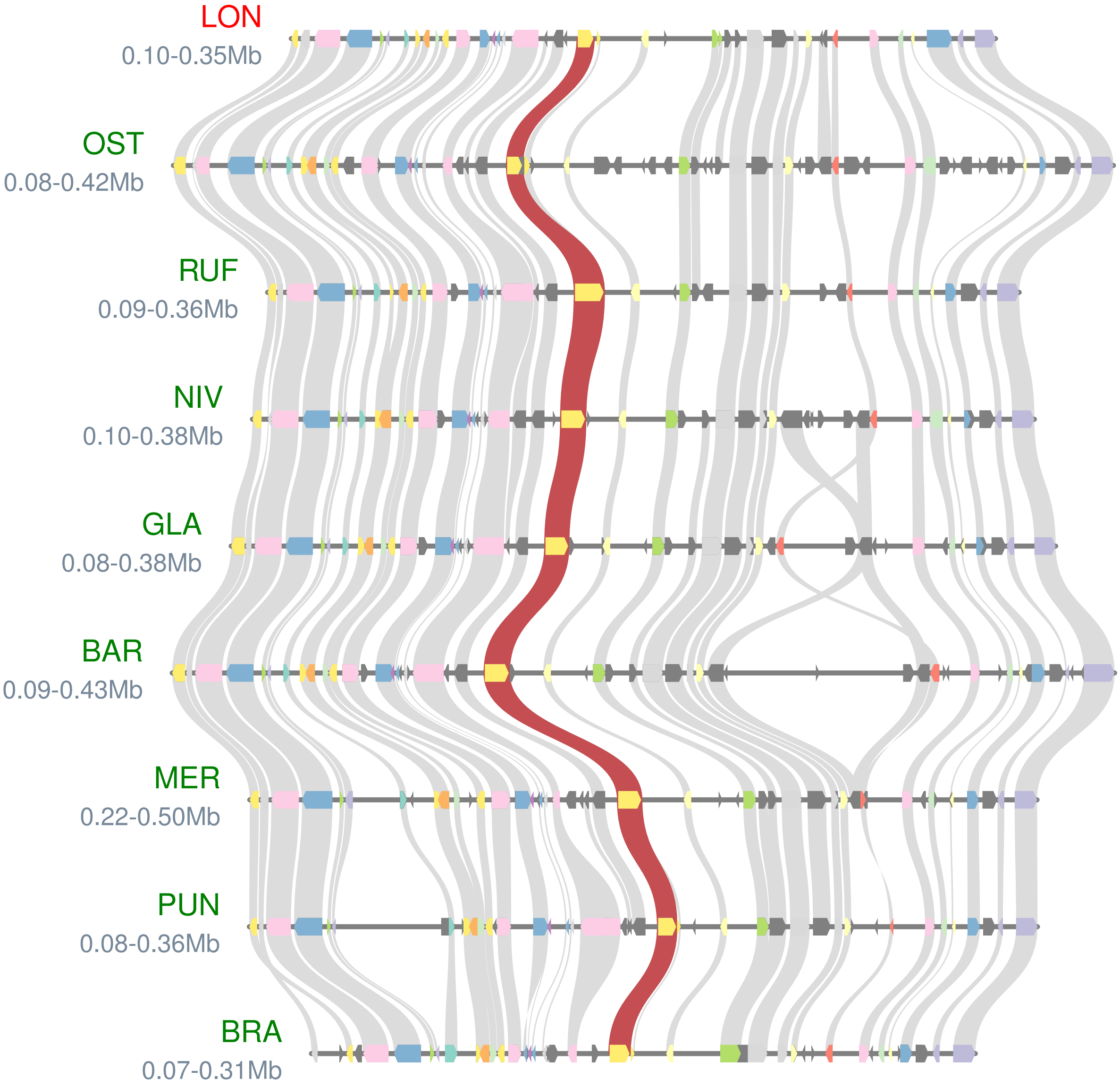

OlMADS17_Olon005641.t1_Mα

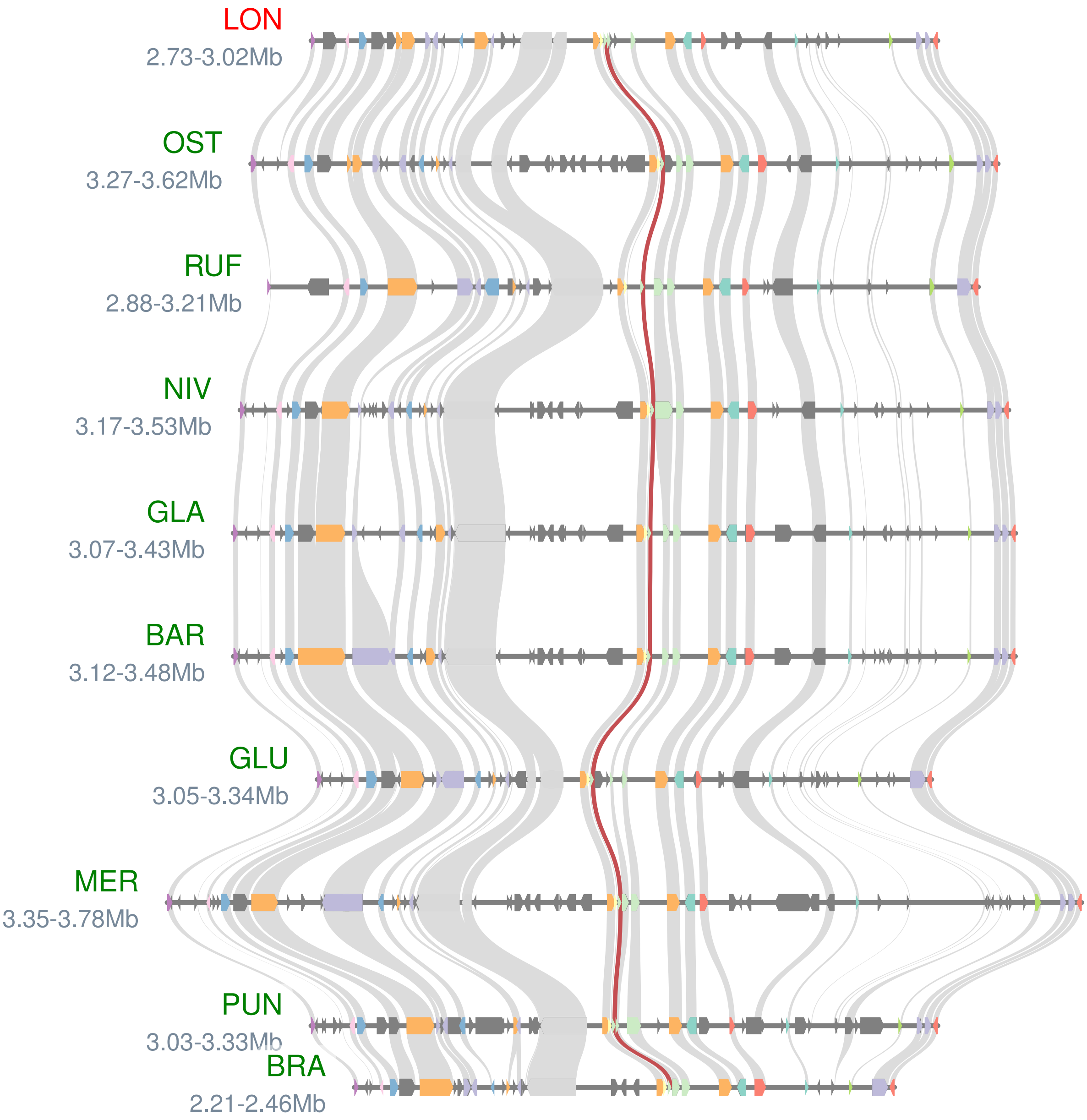

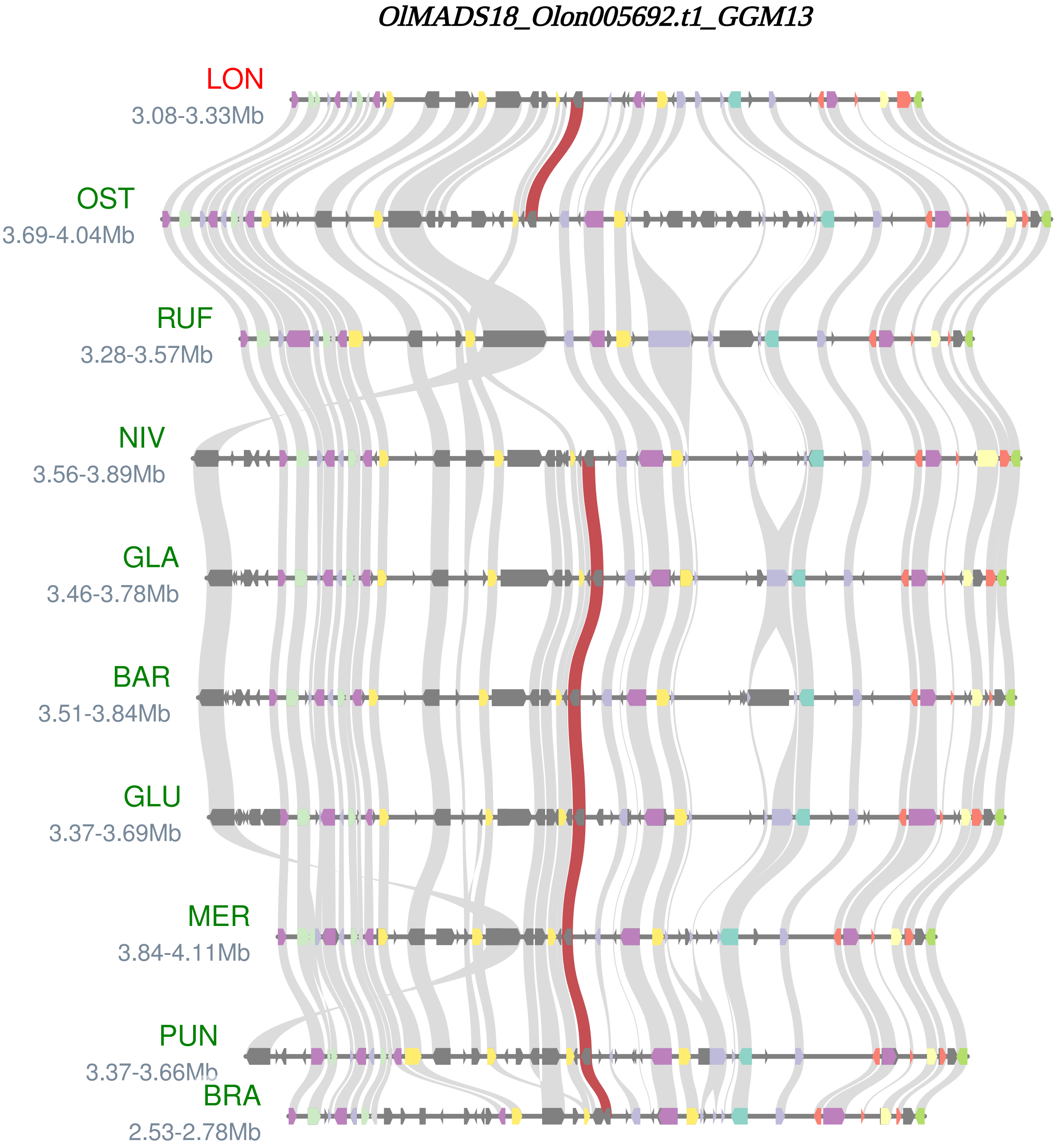

OlMADS18_Olon005692.t1_GGM13
LON
3.08-3.33Mb
OST
3.69-4.04Mb
RUF
3.28-3.57Mb
NIV
3.56-3.89Mb
GLA
3.46-3.78Mb
BAR
3.51-3.84Mb
GLU
3.37-3.69Mb
MER
3.84-4.11Mb
PUN
3.37-3.66Mb
BRA
2.53-2.78Mb

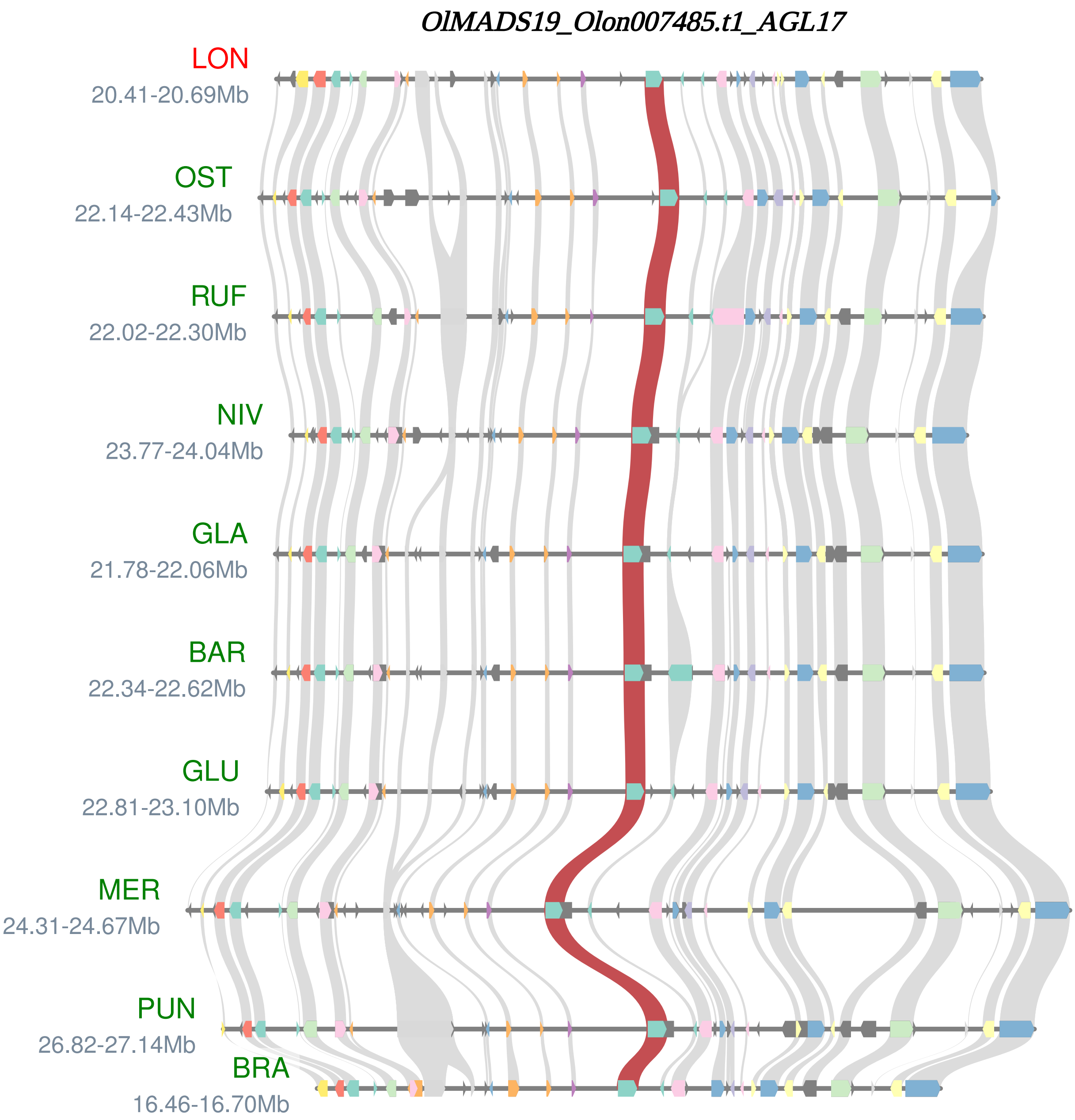
OlMADS19_Olon007485.t1_AGL17
LON
20.41-20.69Mb
OST
22.14-22.43Mb
RUF
22.02-22.30Mb
NIV
23.77-24.04Mb
GLA
21.78-22.06Mb
BAR
22.34-22.62Mb
GLU
22.81-23.10Mb
MER
24.31-24.67Mb
PUN
26.82-27.14Mb
BRA
16.46-16.70Mb

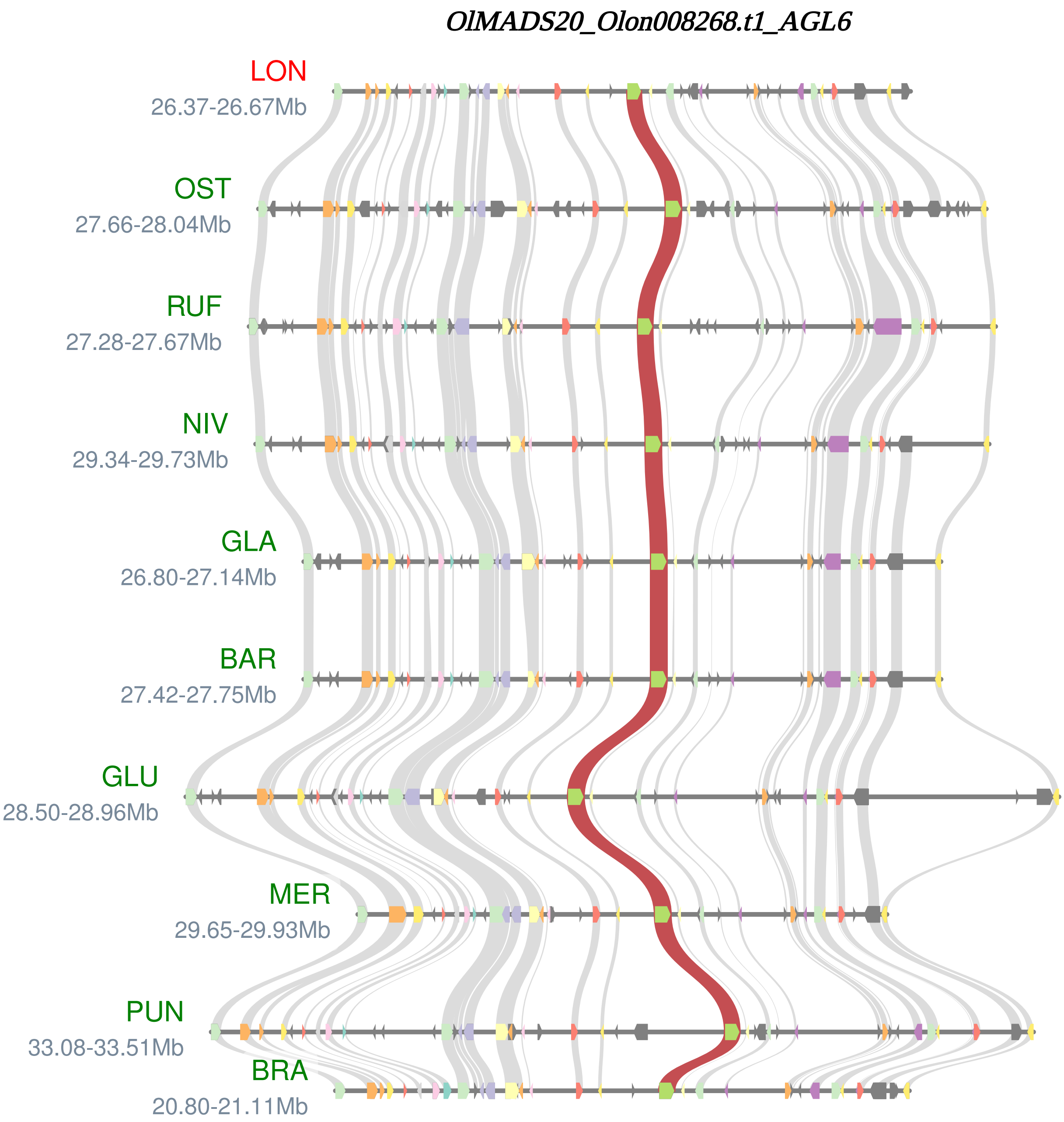
OlMADS20_Olon008268.t1_AGL6
LON
26.37-26.67Mb
OST
27.66-28.04Mb
RUF
27.28-27.67Mb
NIV
29.34-29.73Mb
GLA
26.80-27.14Mb
BAR
27.42-27.75Mb
GLU
28.50-28.96Mb
MER
29.65-29.93Mb
PUN
33.08-33.51Mb
BRA
20.80-21.11Mb

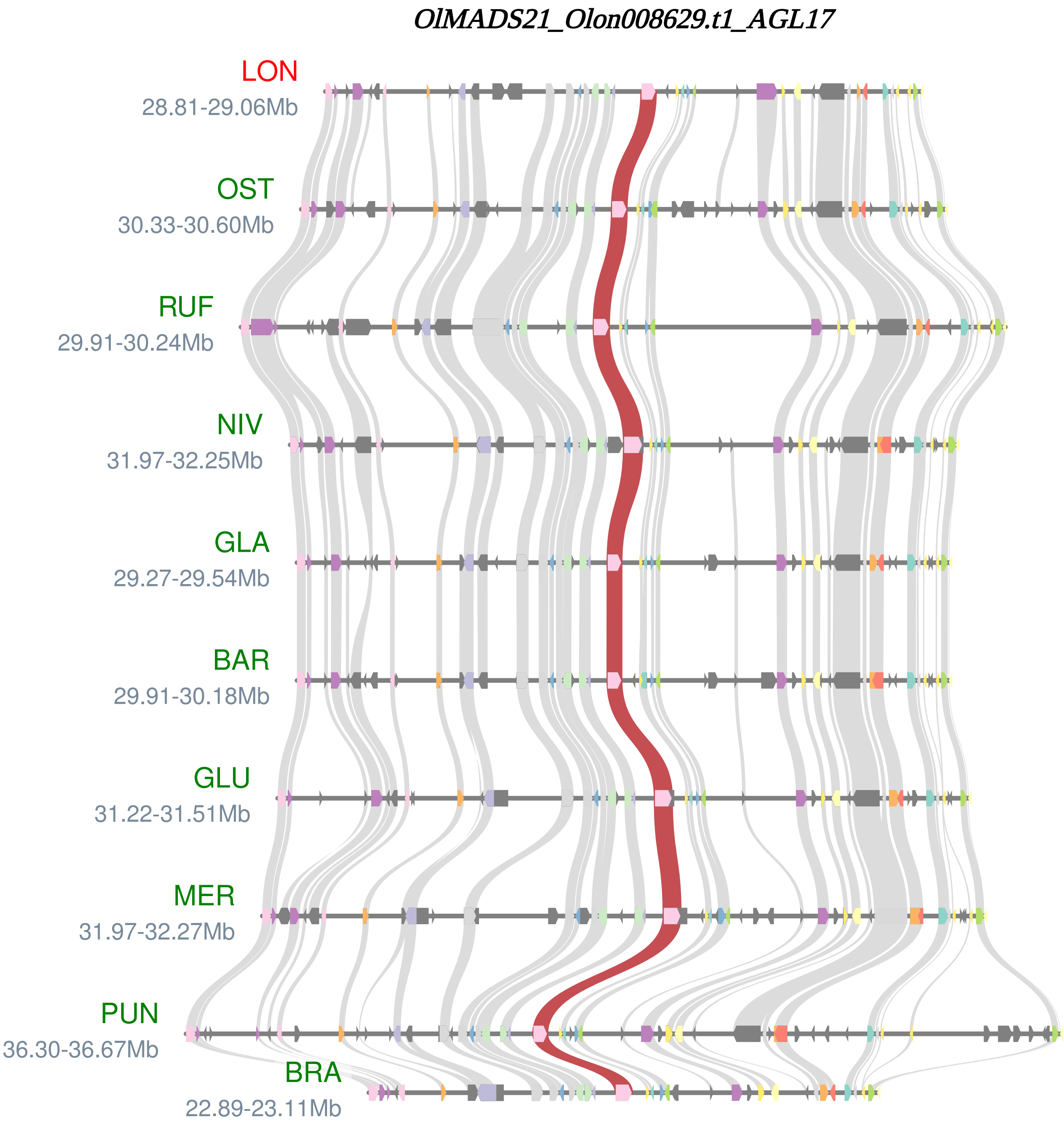

OlMADS21_Olon008629.t1_AGL17
LON
28.81-29.06Mb
OST
30.33-30.60Mb
RUF
29.91-30.24Mb
NIV
31.97-32.25Mb
GLA
29.27-29.54Mb
BAR
29.91-30.18Mb
GLU
31.22-31.51Mb
MER
31.97-32.27Mb
PUN
36.30-36.67Mb
BRA
22.89-23.11Mb

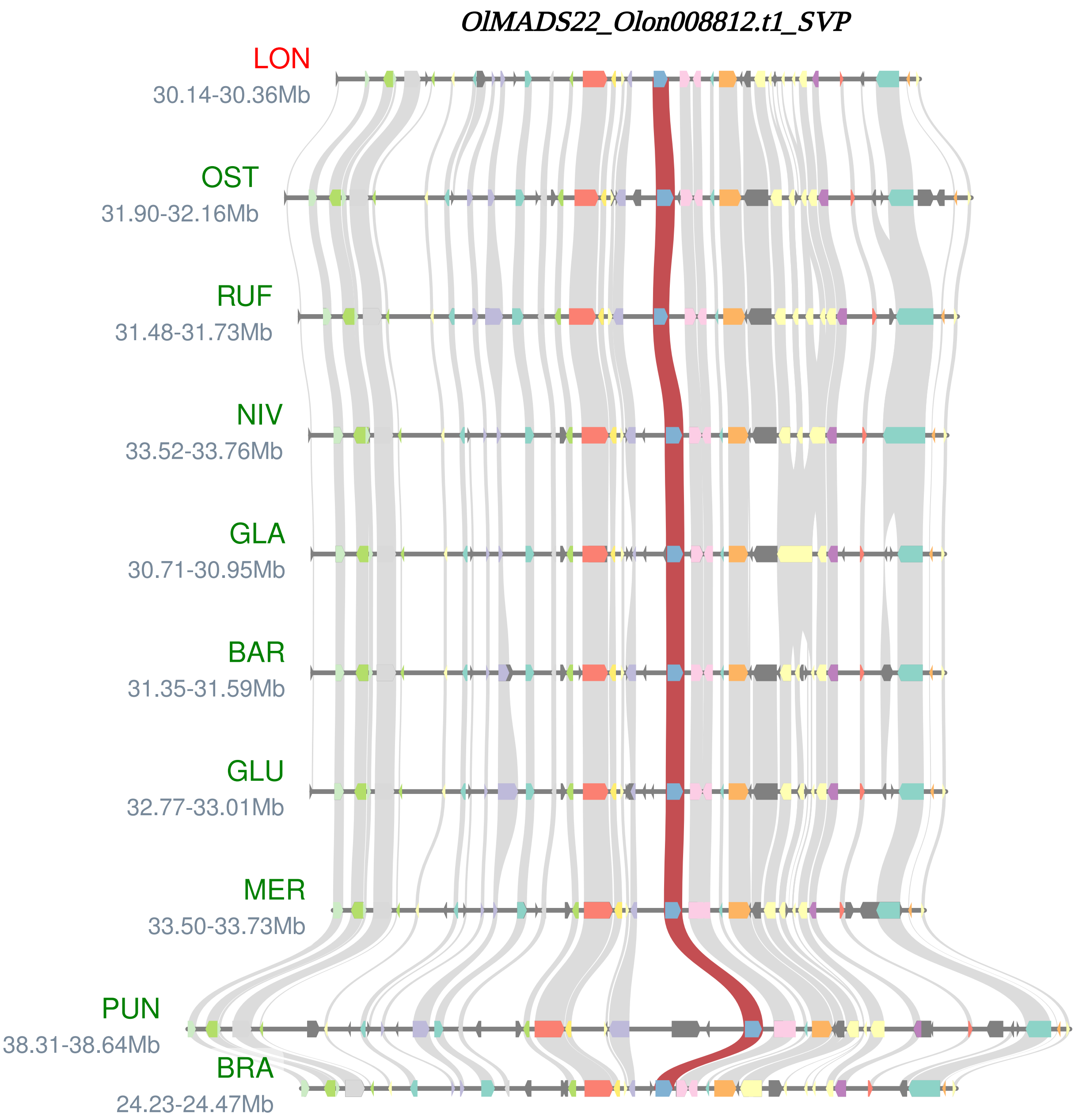
OlMADS22_Olon008812.t1_SVP
LON
30.14-30.36Mb
OST
31.90-32.16Mb
RUF
31.48-31.73Mb
NIV
33.52-33.76Mb
GLA
30.71-30.95Mb
BAR
31.35-31.59Mb
GLU
32.77-33.01Mb
MER
33.50-33.73Mb
PUN
38.31-38.64Mb
BRA
24.23-24.47Mb

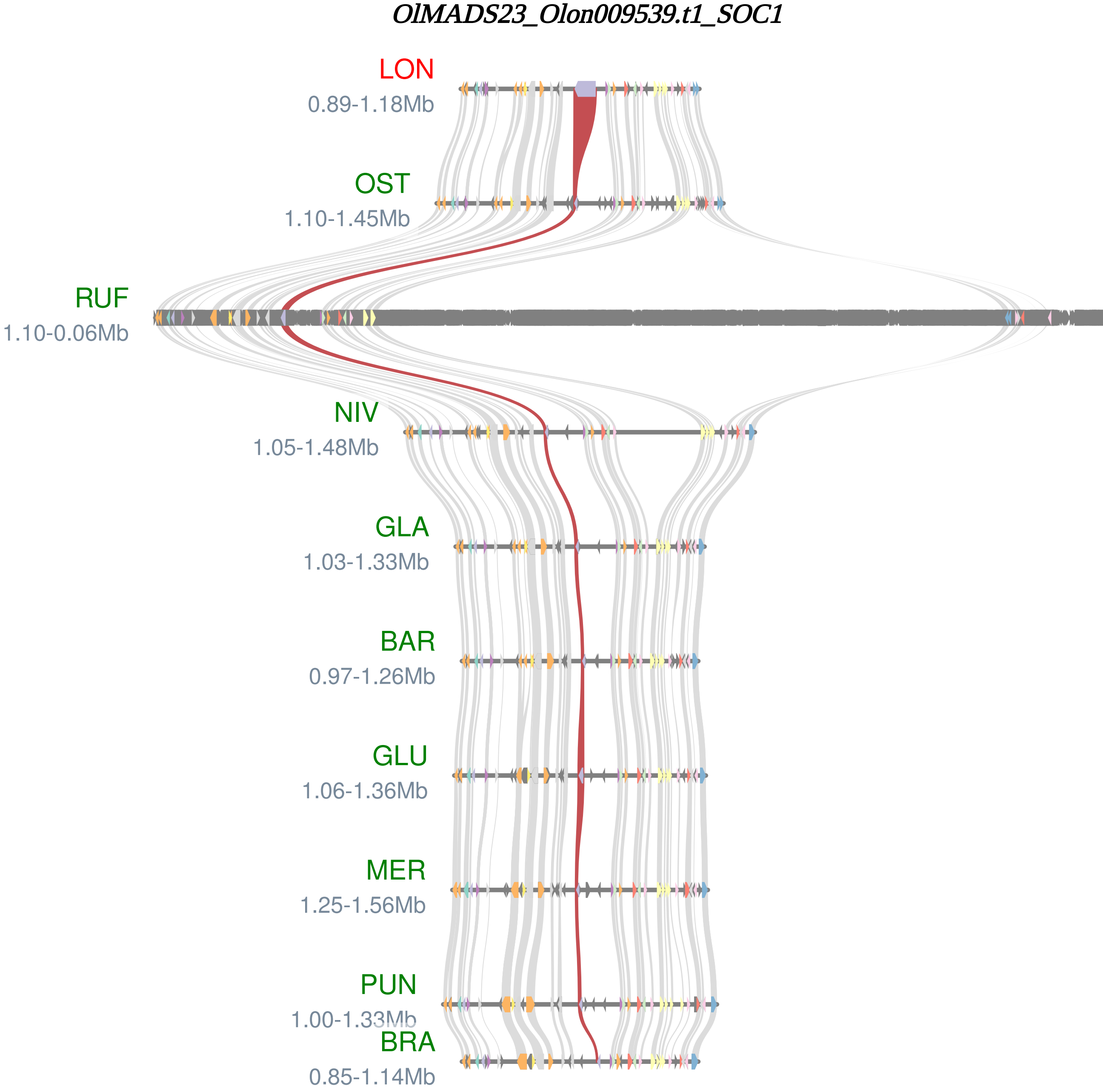

OlMADS23_Olon009539.t1_SOC1
LON
0.89-1.18Mb
OST
1.10-1.45Mb
RUF
1.10-0.06Mb
NIV
1.05-1.48Mb
GLA
1.03-1.33Mb
BAR
0.97-1.26Mb
GLU
1.06-1.36Mb
MER
1.25-1.56Mb
PUN
1.00-1.33Mb
BRA
0.85-1.14Mb

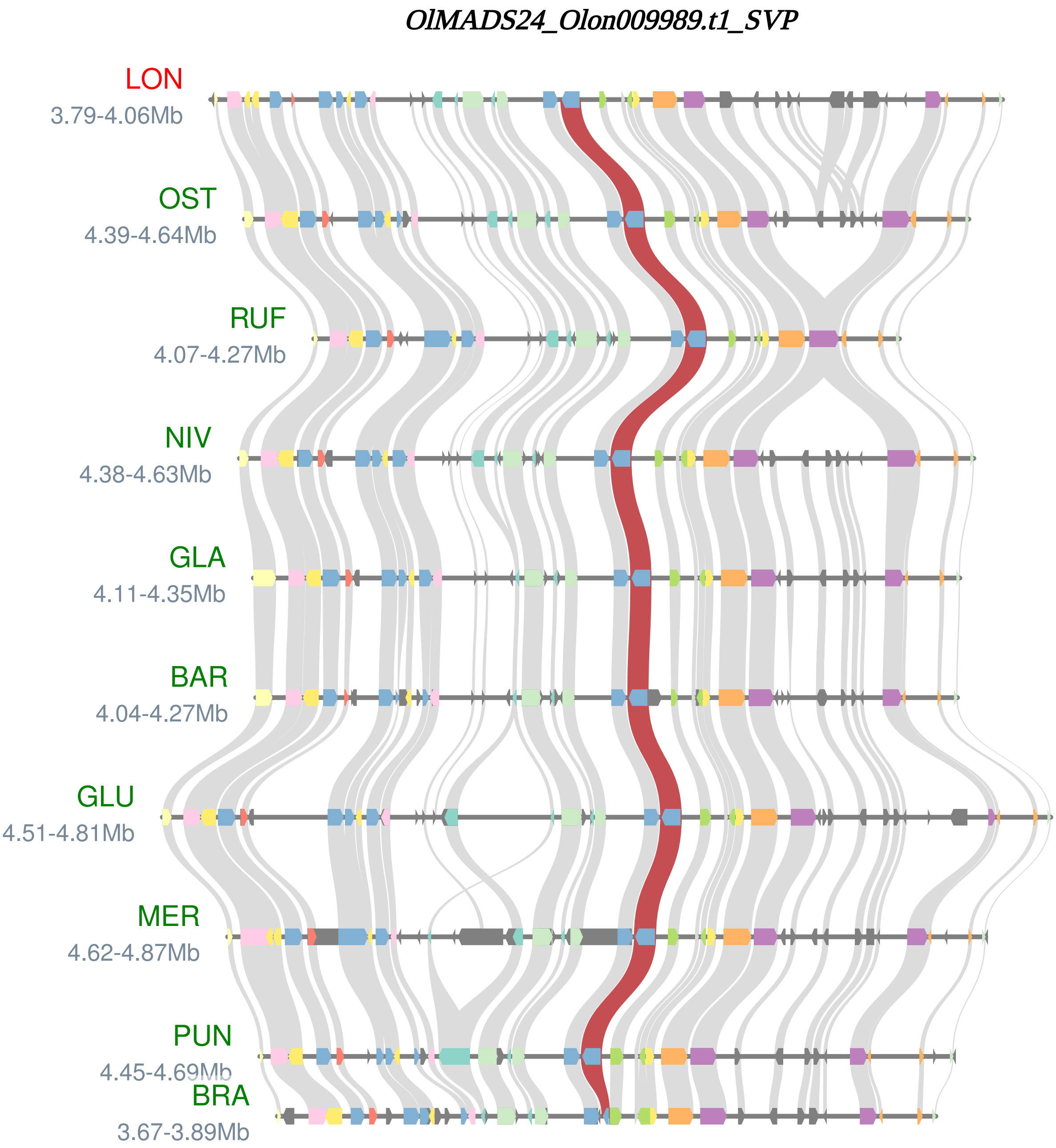
OlMADS24_Olon009989.t1_SVP
LON
3.79-4.06Mb
OST
4.39-4.64Mb
RUF
4.07-4.27Mb
NIV
4.38-4.63Mb
GLA
4.11-4.35Mb
BAR
4.04-4.27Mb
GLU
4.51-4.81Mb
MER
4.62-4.87Mb
PUN
4.45-4.69Mb
BRA
3.67-3.89Mb

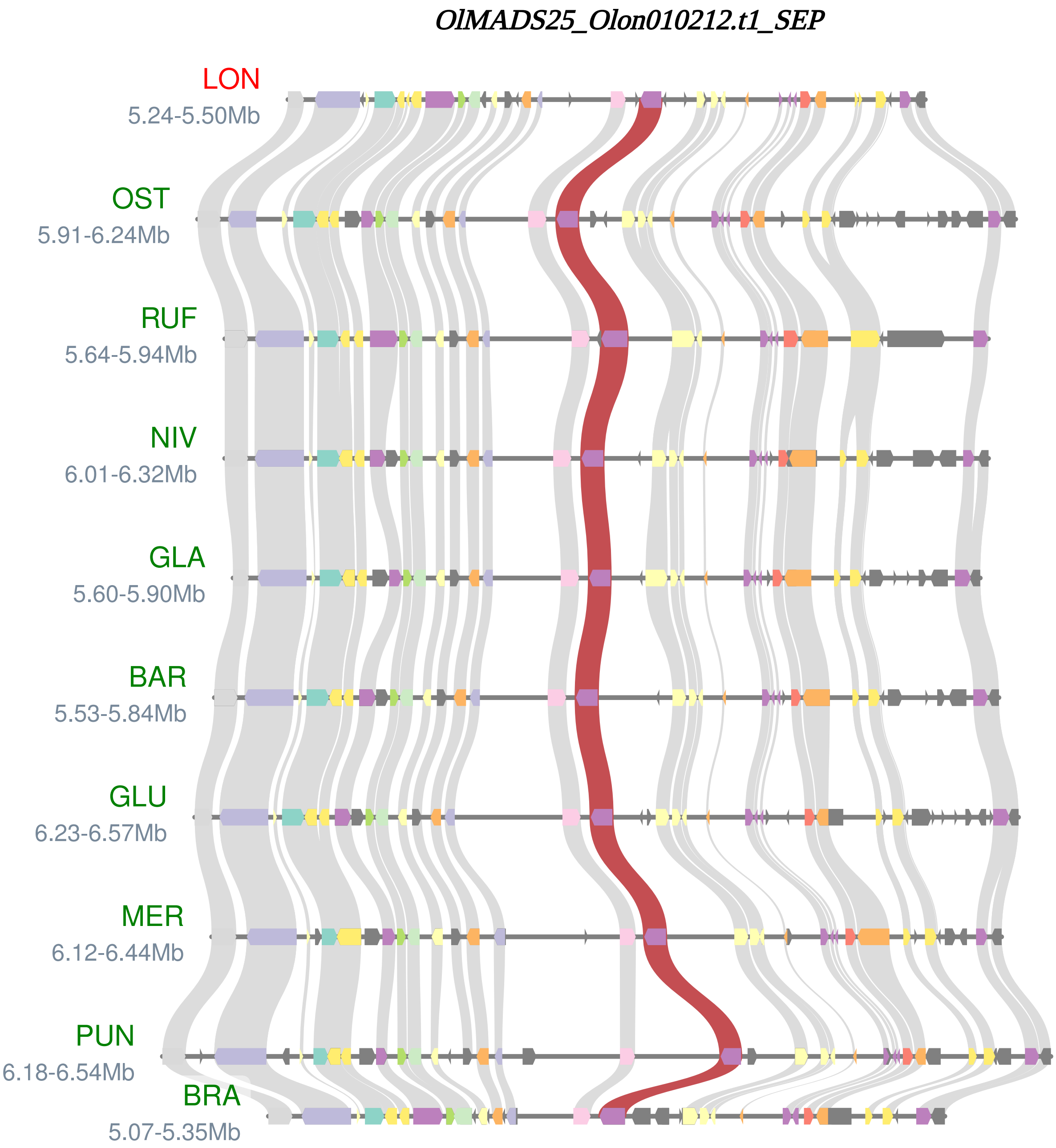
OlMADS25_Olon010212.t1_SEP
LON
5.24-5.50Mb
OST
5.91-6.24Mb
RUF
5.64-5.94Mb
NIV
6.01-6.32Mb
GLA
5.60-5.90Mb
BAR
5.53-5.84Mb
GLU
6.23-6.57Mb
MER
6.12-6.44Mb
PUN
6.18-6.54Mb
BRA
5.07-5.35Mb

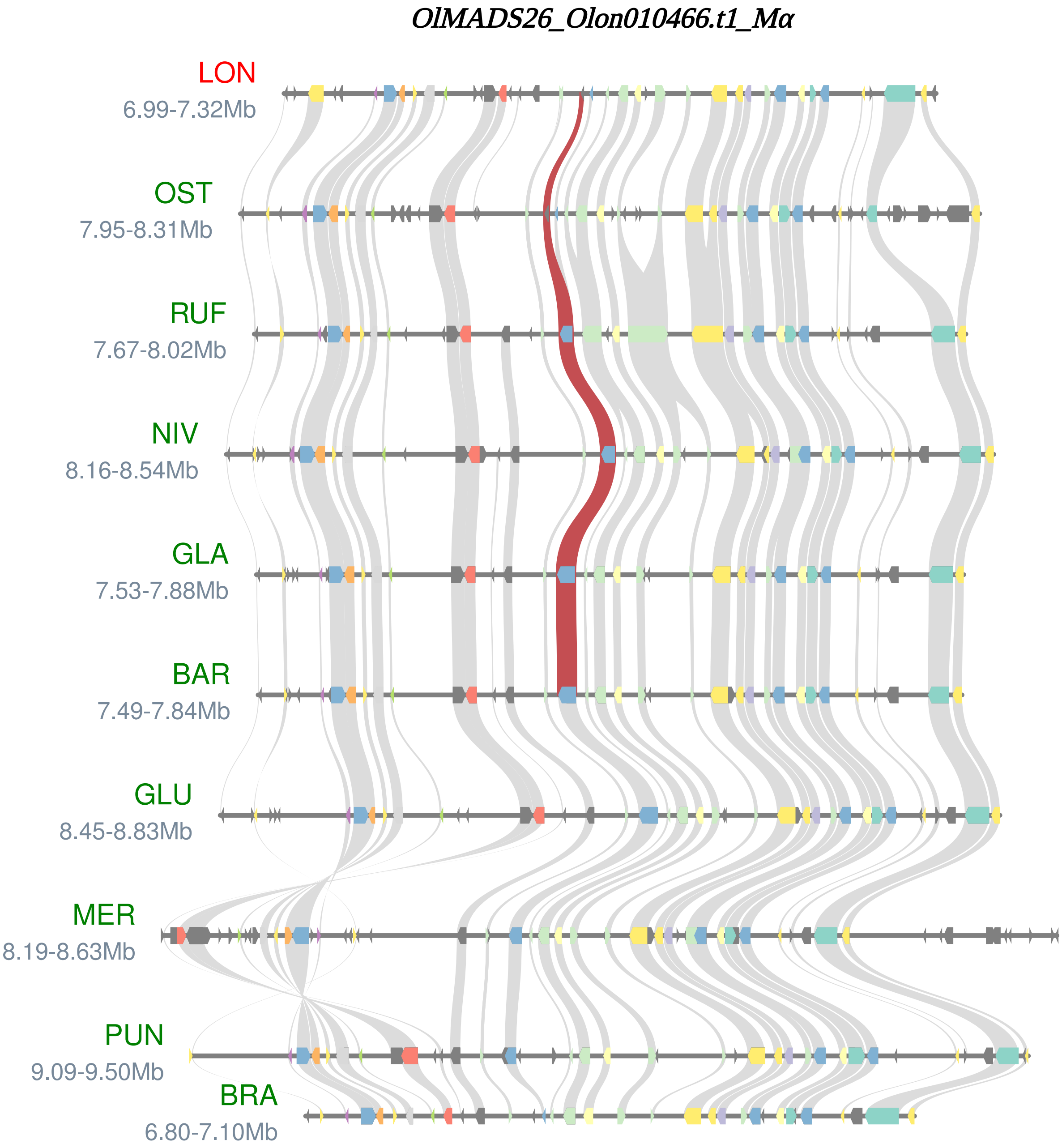
OlMADS26_Olon010466.t1_Mα
LON
6.99-7.32Mb
OST
7.95-8.31Mb
RUF
7.67-8.02Mb
NIV
8.16-8.54Mb
GLA
7.53-7.88Mb
BAR
7.49-7.84Mb
GLU
8.45-8.83Mb
MER
8.19-8.63Mb
PUN
9.09-9.50Mb
BRA
6.80-7.10Mb

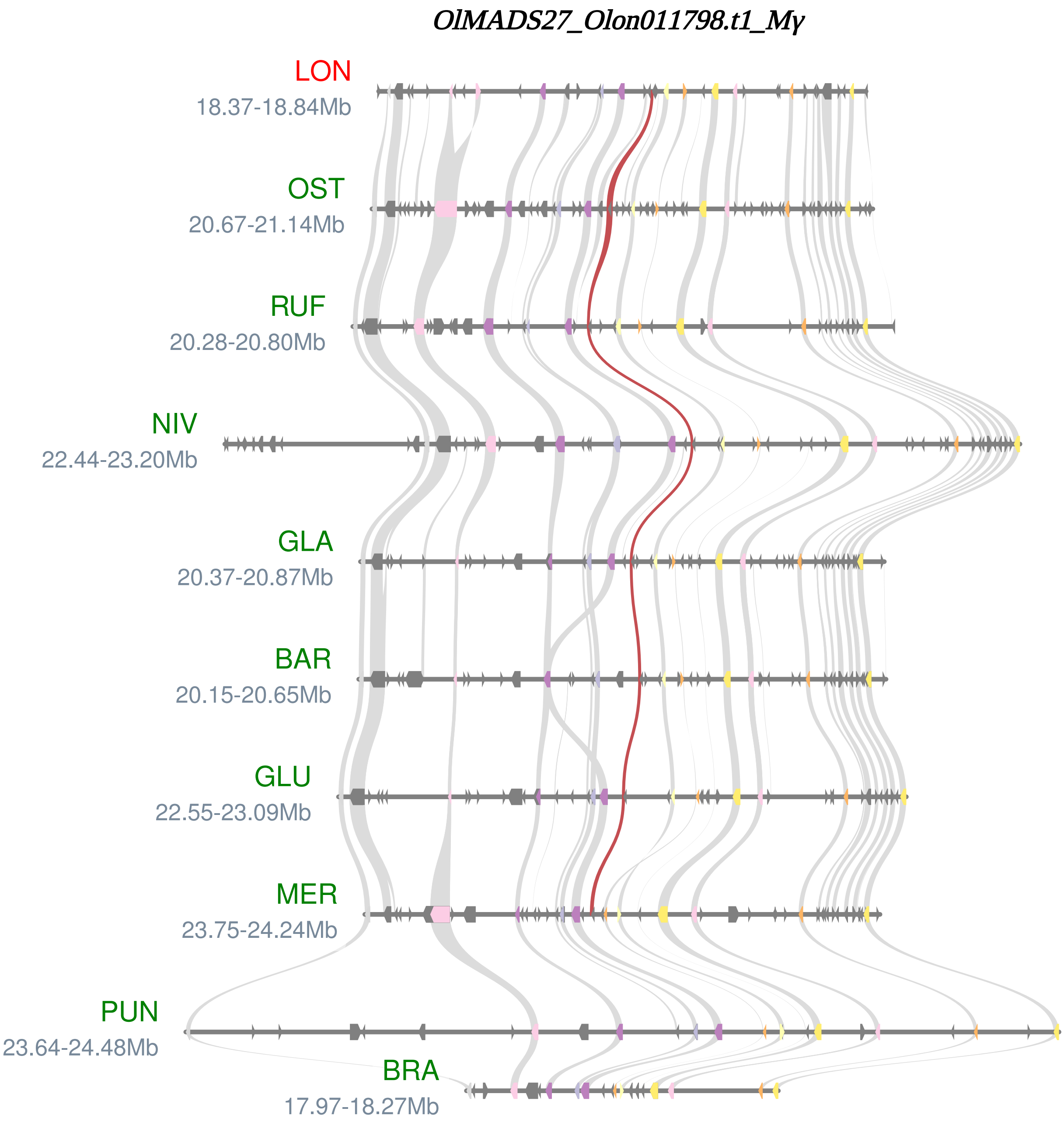

OlMADS27_Olon011798.t1_Mγ
LON
18.37-18.84Mb
OST
20.67-21.14Mb
RUF
20.28-20.80Mb
NIV
22.44-23.20Mb
GLA
20.37-20.87Mb
BAR
20.15-20.65Mb
GLU
22.55-23.09Mb
MER
23.75-24.24Mb
PUN
23.64-24.48Mb
BRA
17.97-18.27Mb

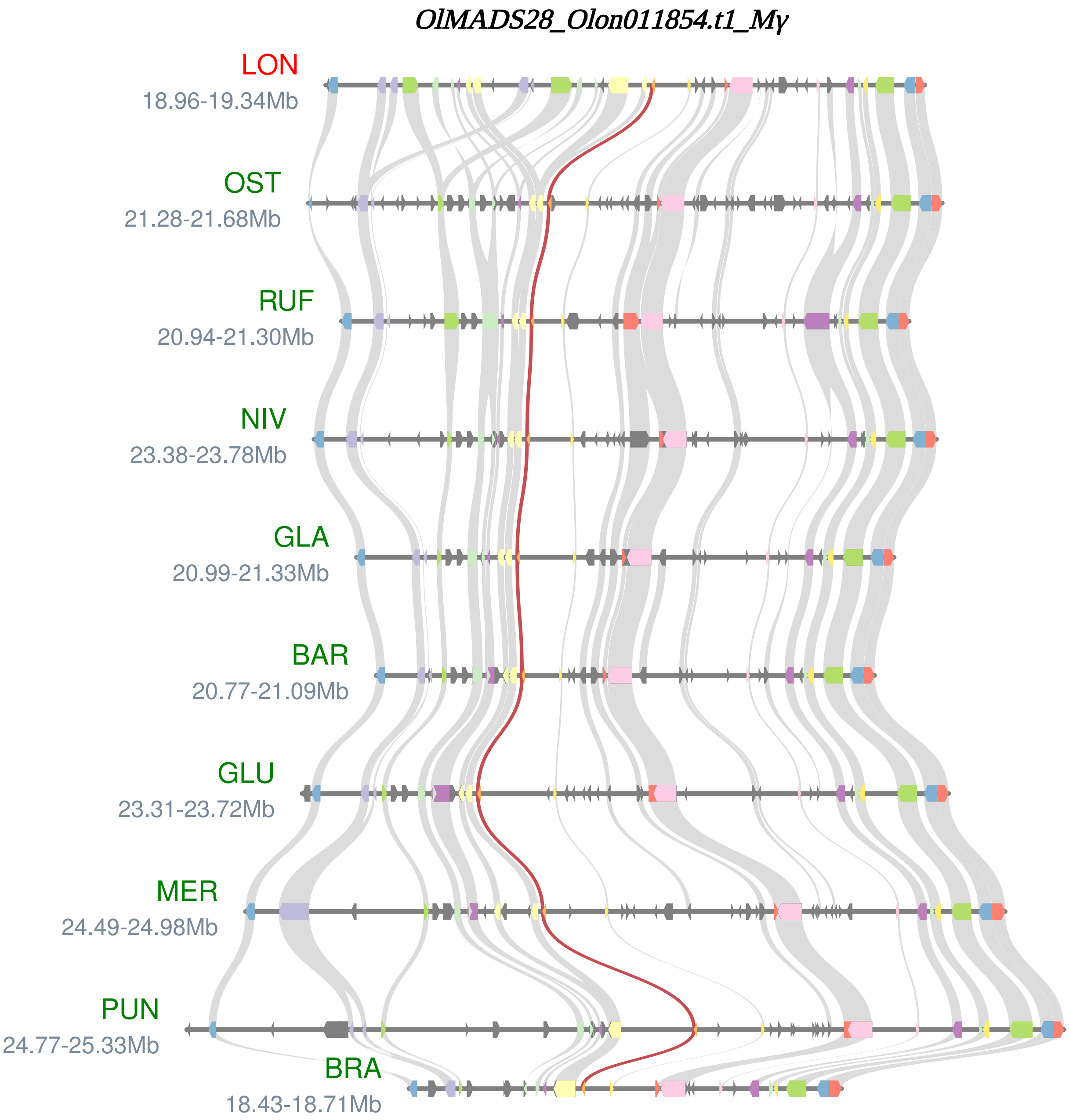

OlMADS28_Olon011854.t1_Mγ
LON
18.96-19.34Mb
OST
21.28-21.68Mb
RUF
20.94-21.30Mb
NIV
23.38-23.78Mb
GLA
20.99-21.33Mb
BAR
20.77-21.09Mb
GLU
23.31-23.72Mb
MER
24.49-24.98Mb
PUN
24.77-25.33Mb
BRA
18.43-18.71Mb

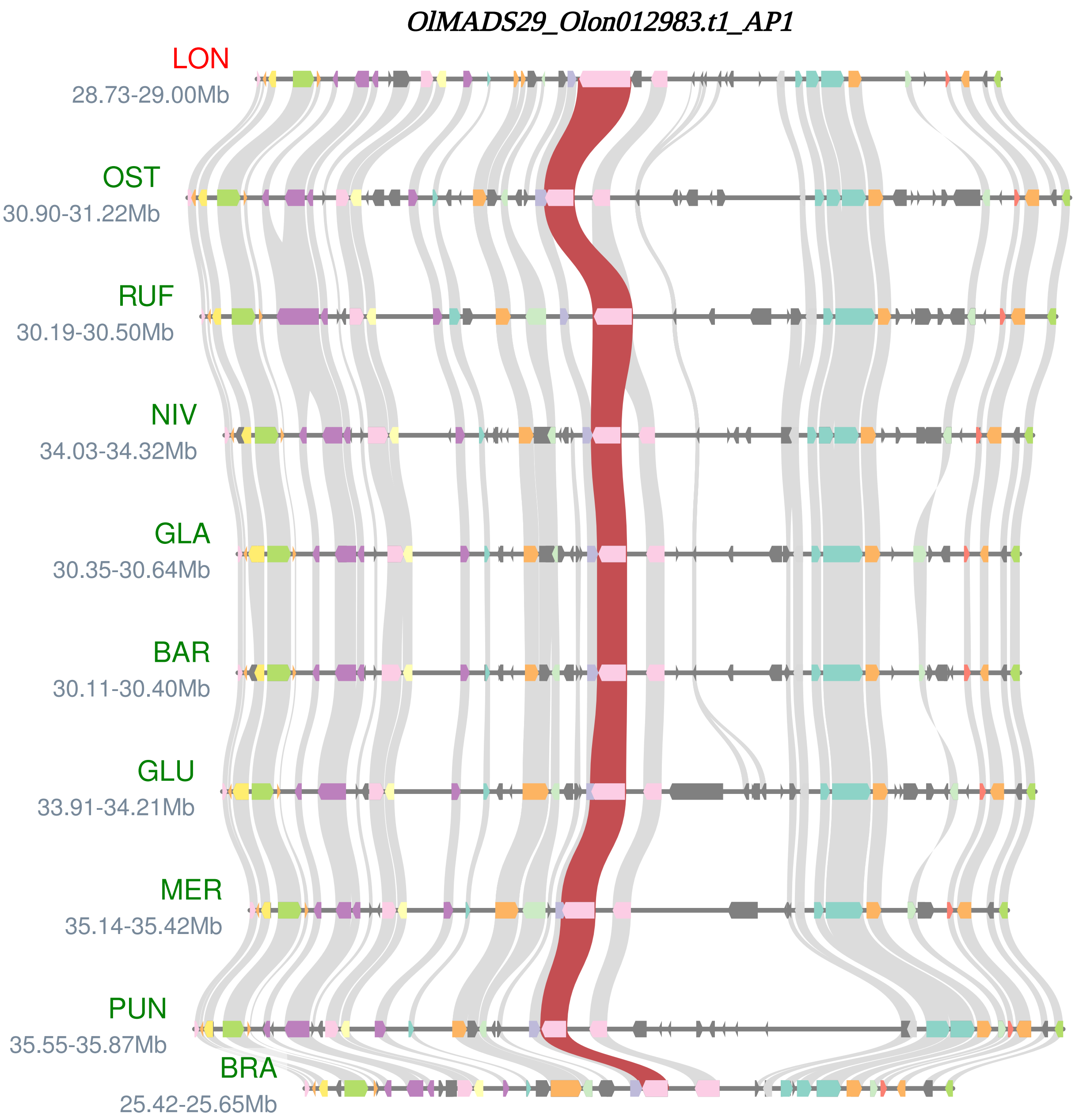
OlMADS29_Olon012983.t1_AP1
LON
28.73-29.00Mb
OST
30.90-31.22Mb
RUF
30.19-30.50Mb
NIV
34.03-34.32Mb
GLA
30.35-30.64Mb
BAR
30.11-30.40Mb
GLU
33.91-34.21Mb
MER
35.14-35.42Mb
PUN
35.55-35.87Mb
BRA
25.42-25.65Mb

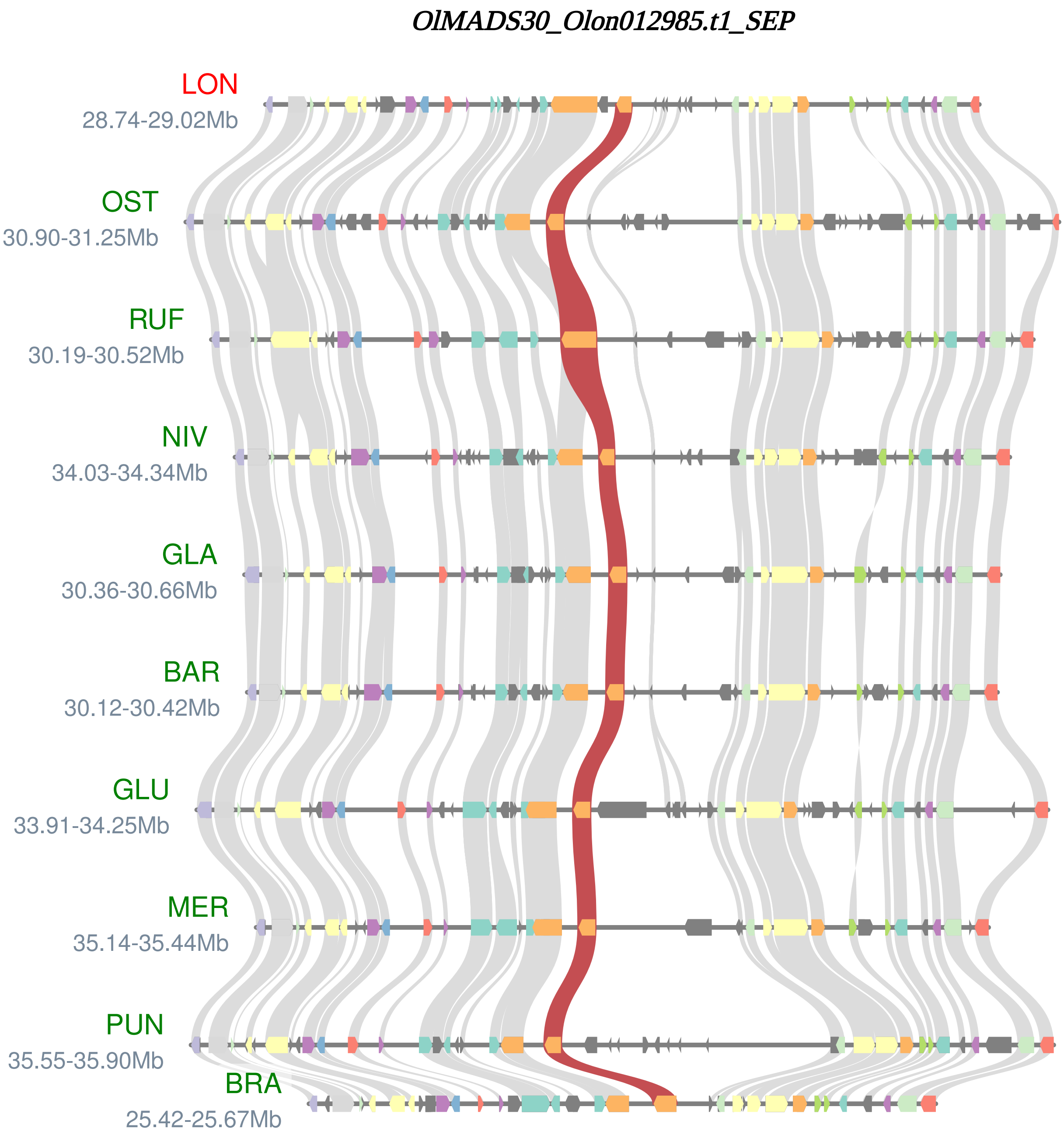

OlMADS30_Olon012985.t1_SEP
LON
28.74-29.02Mb
OST
30.90-31.25Mb
RUF
30.19-30.52Mb
NIV
34.03-34.34Mb
GLA
30.36-30.66Mb
BAR
30.12-30.42Mb
GLU
33.91-34.25Mb
MER
35.14-35.44Mb
PUN
35.55-35.90Mb
BRA
25.42-25.67Mb

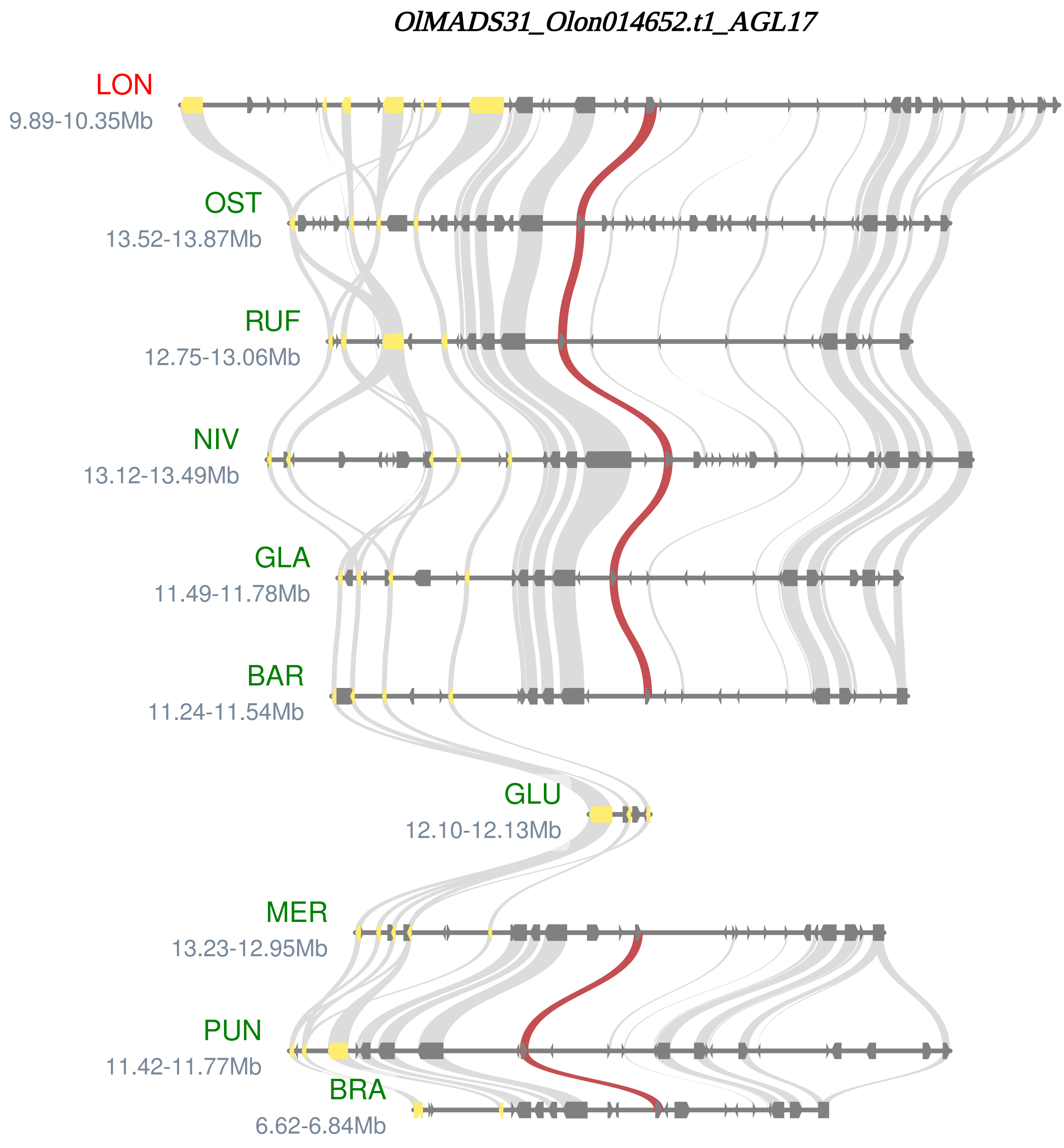
OlMADS31_Olon014652.t1_AGL17
LON
9.89-10.35Mb
OST
13.52-13.87Mb
RUF
12.75-13.06Mb
NIV
13.12-13.49Mb
GLA
11.49-11.78Mb
BAR
11.24-11.54Mb
GLU
12.10-12.13Mb
MER
13.23-12.95Mb
PUN
11.42-11.77Mb
BRA
6.62-6.84Mb

OlMADS32_Olon014703.t1_Mγ
OlMADS33_Olon014704.t1_Mγ
(The chromosomal segment in the GLU lacks any detected syntenic genes.)

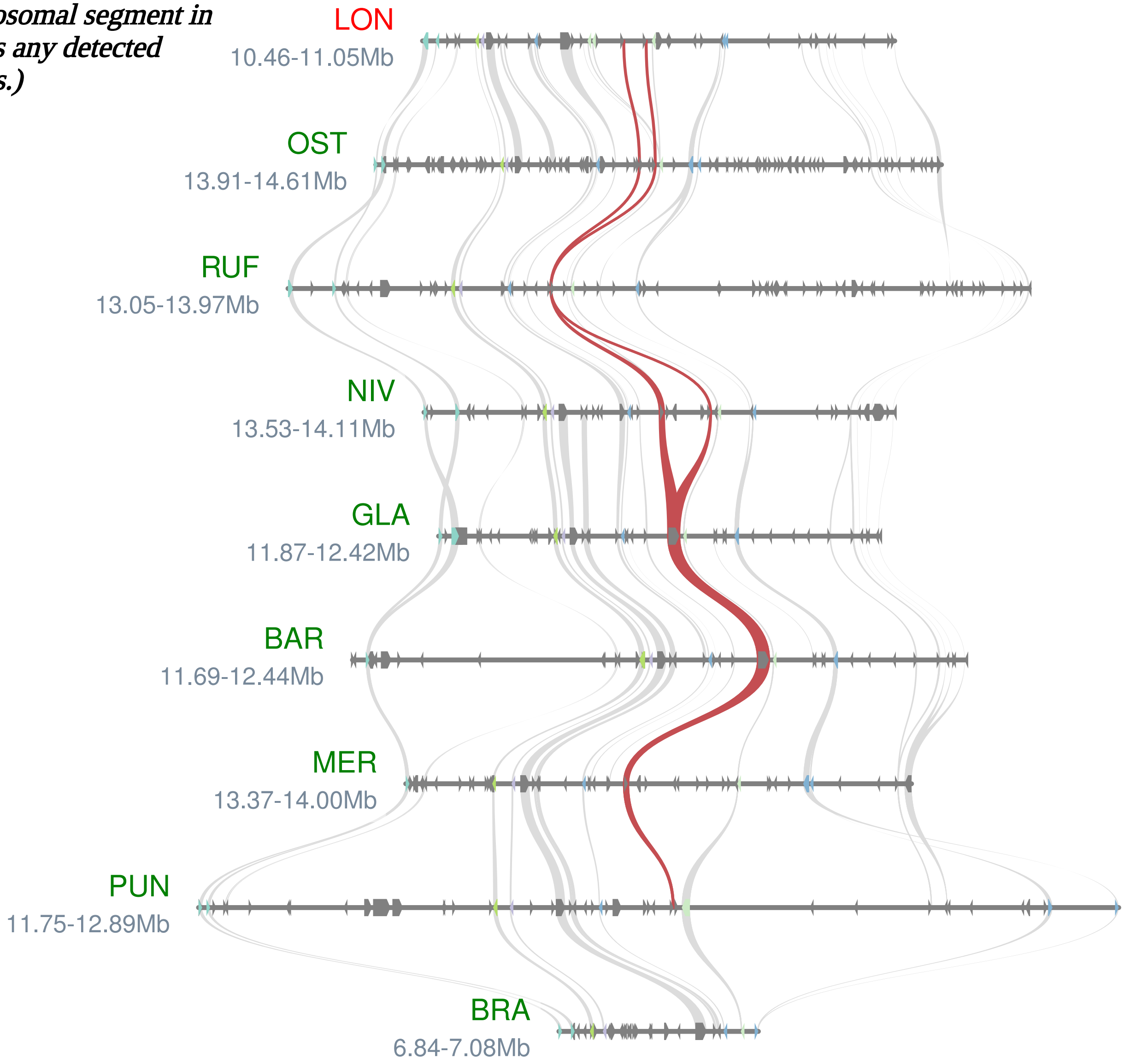

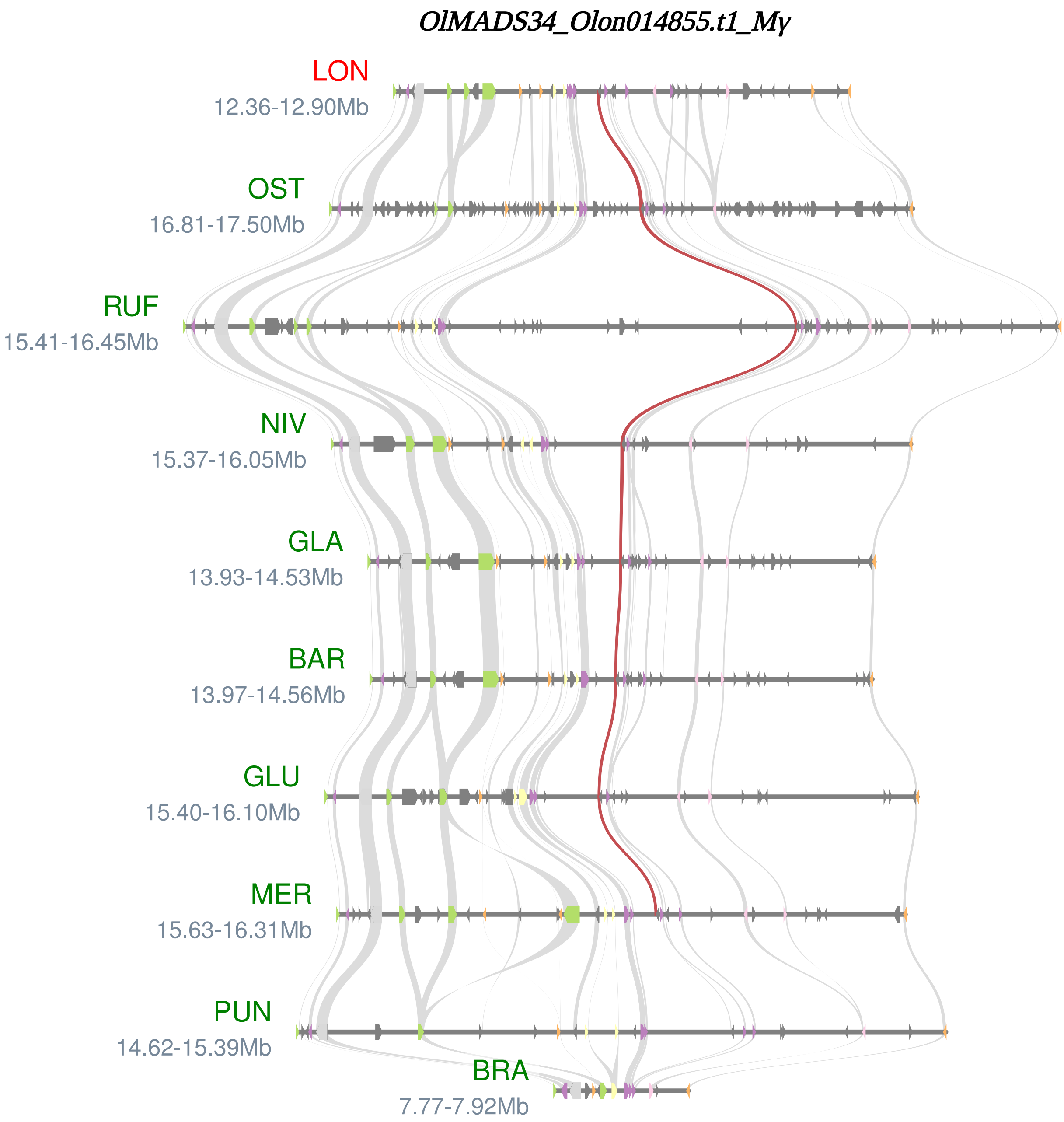

OlMADS34_Olon014855.t1_Mγ
LON
12.36-12.90Mb
OST
16.81-17.50Mb
RUF
15.41-16.45Mb
NIV
15.37-16.05Mb
GLA
13.93-14.53Mb
BAR
13.97-14.56Mb
GLU
15.40-16.10Mb
MER
15.63-16.31Mb
PUN
14.62-15.39Mb
BRA
7.77-7.92Mb

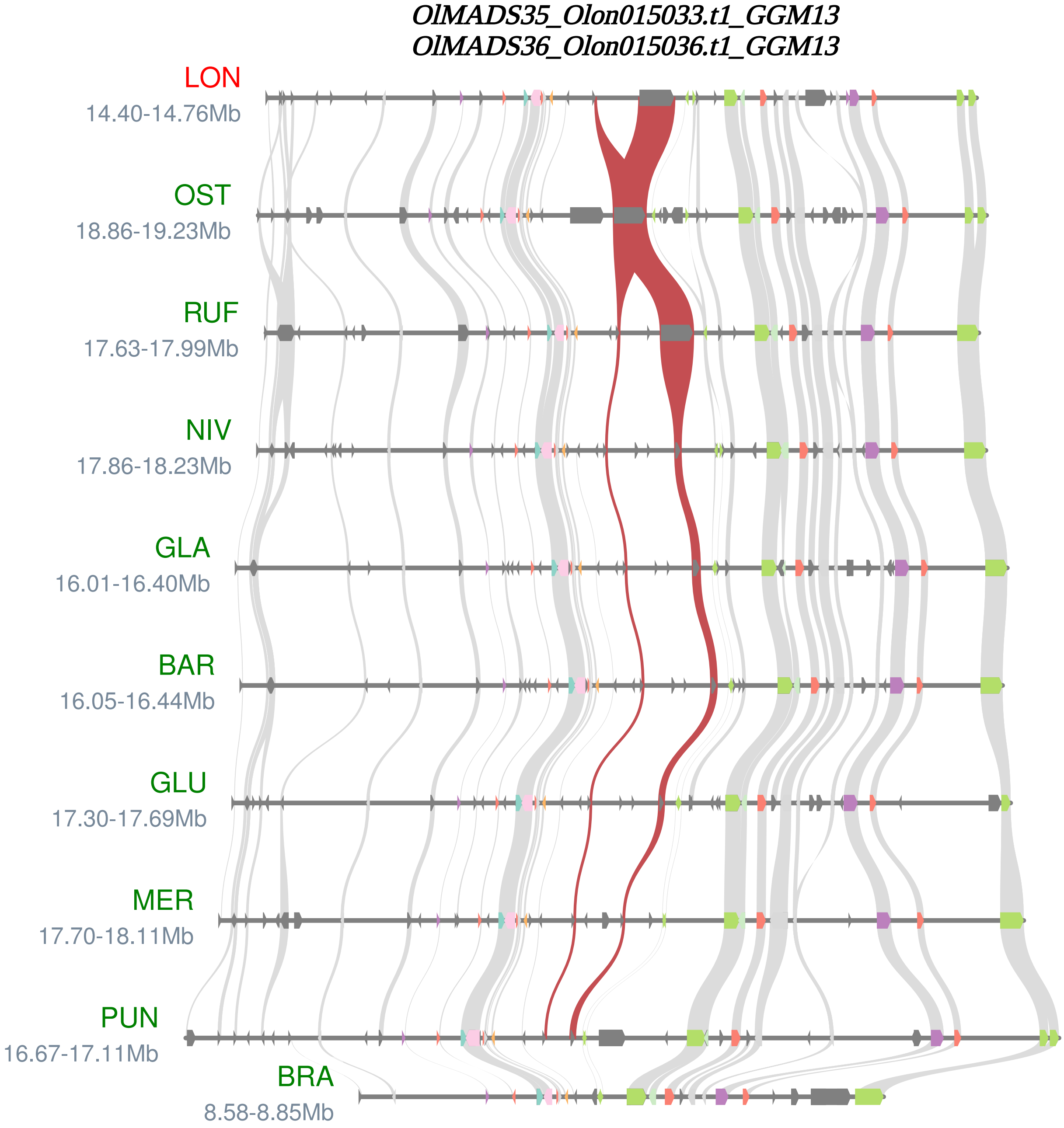

OlMADS35_Olon015033.t1_GGM13
OlMADS36_Olon015036.t1_GGM13
LON
14.40-14.76Mb
OST
18.86-19.23Mb
RUF
17.63-17.99Mb
NIV
17.86-18.23Mb
GLA
16.01-16.40Mb
BAR
16.05-16.44Mb
GLU
17.30-17.69Mb
MER
17.70-18.11Mb
PUN
16.67-17.11Mb
BRA
8.58-8.85Mb

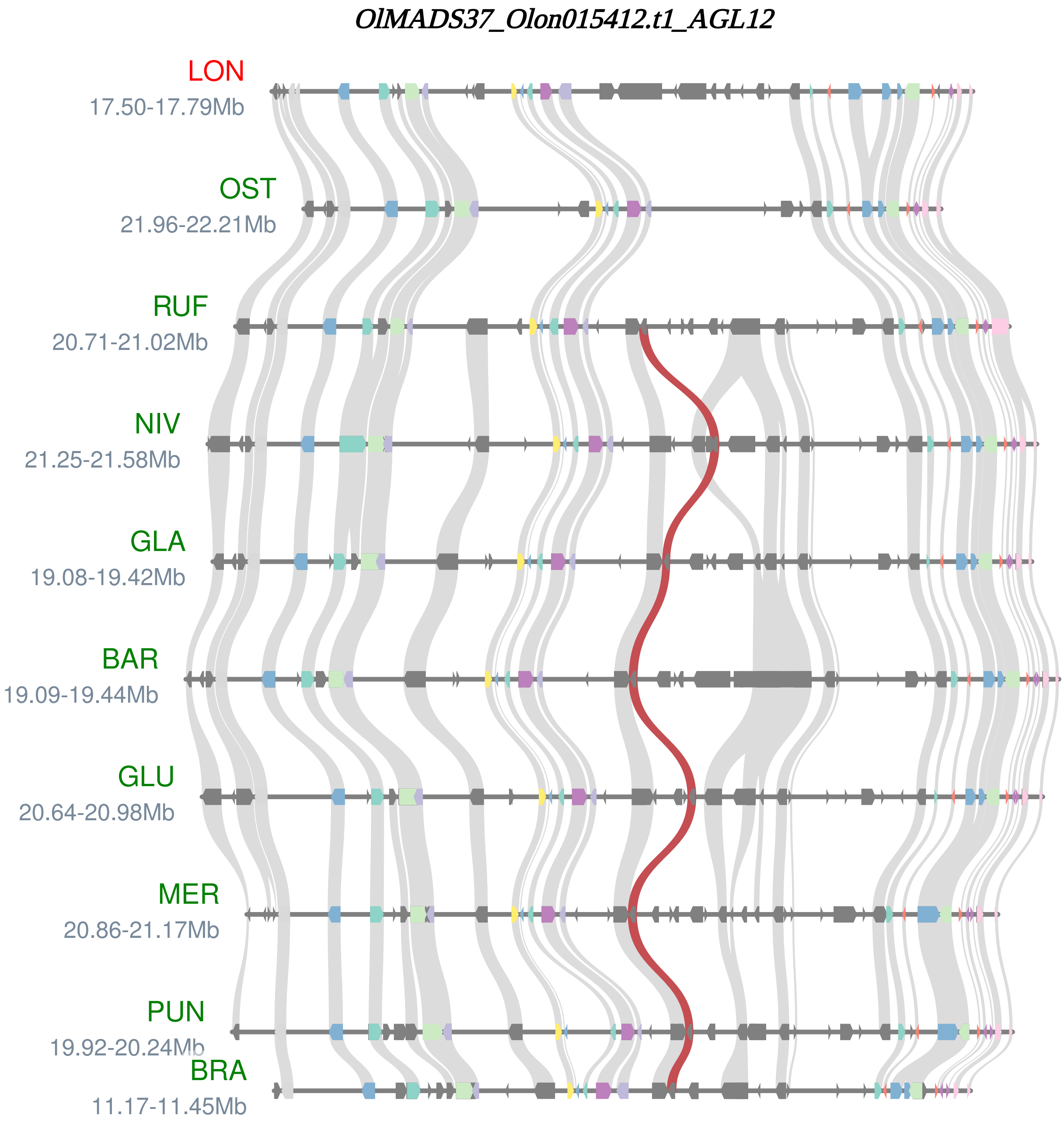
OlMADS37_Olon015412.t1_AGL12
LON
17.50-17.79Mb
OST
21.96-22.21Mb
RUF
20.71-21.02Mb
NIV
21.25-21.58Mb
GLA
19.08-19.42Mb
BAR
19.09-19.44Mb
GLU
20.64-20.98Mb
MER
20.86-21.17Mb
PUN
19.92-20.24Mb
BRA
11.17-11.45Mb

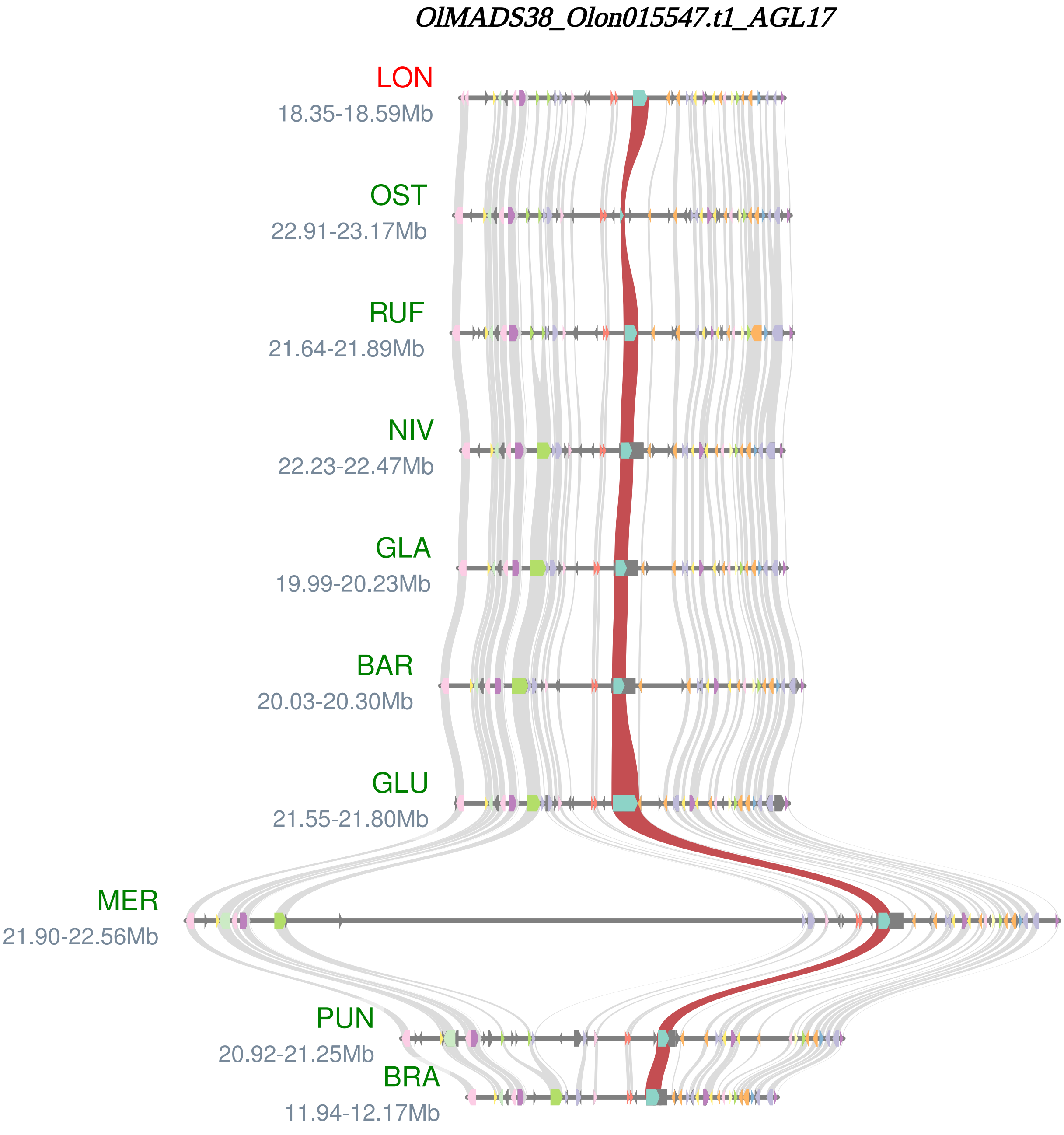

OlMADS38_Olon015547.t1_AGL17
LON
18.35-18.59Mb
OST
22.91-23.17Mb
RUF
21.64-21.89Mb
NIV
22.23-22.47Mb
GLA
19.99-20.23Mb
BAR
20.03-20.30Mb
GLU
21.55-21.80Mb
MER
21.90-22.56Mb
PUN
20.92-21.25Mb
BRA
11.94-12.17Mb

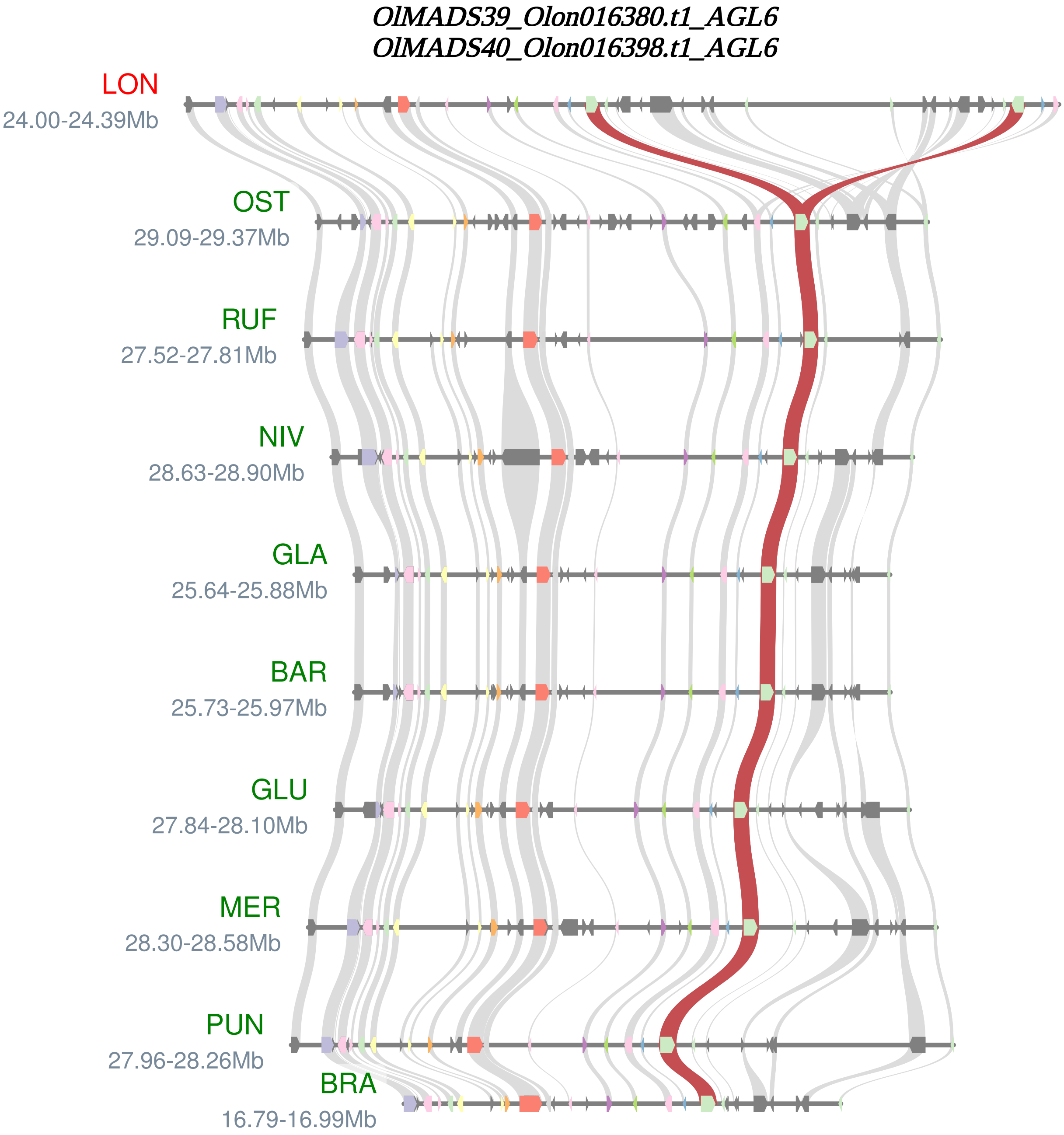
OlMADS39_Olon016380.t1_AGL6
OlMADS40_Olon016398.t1_AGL6
LON
24.00-24.39Mb
OST
29.09-29.37Mb
RUF
27.52-27.81Mb
NIV
28.63-28.90Mb
GLA
25.64-25.88Mb
BAR
25.73-25.97Mb
GLU
27.84-28.10Mb
MER
28.30-28.58Mb
PUN
27.96-28.26Mb
BRA
16.79-16.99Mb

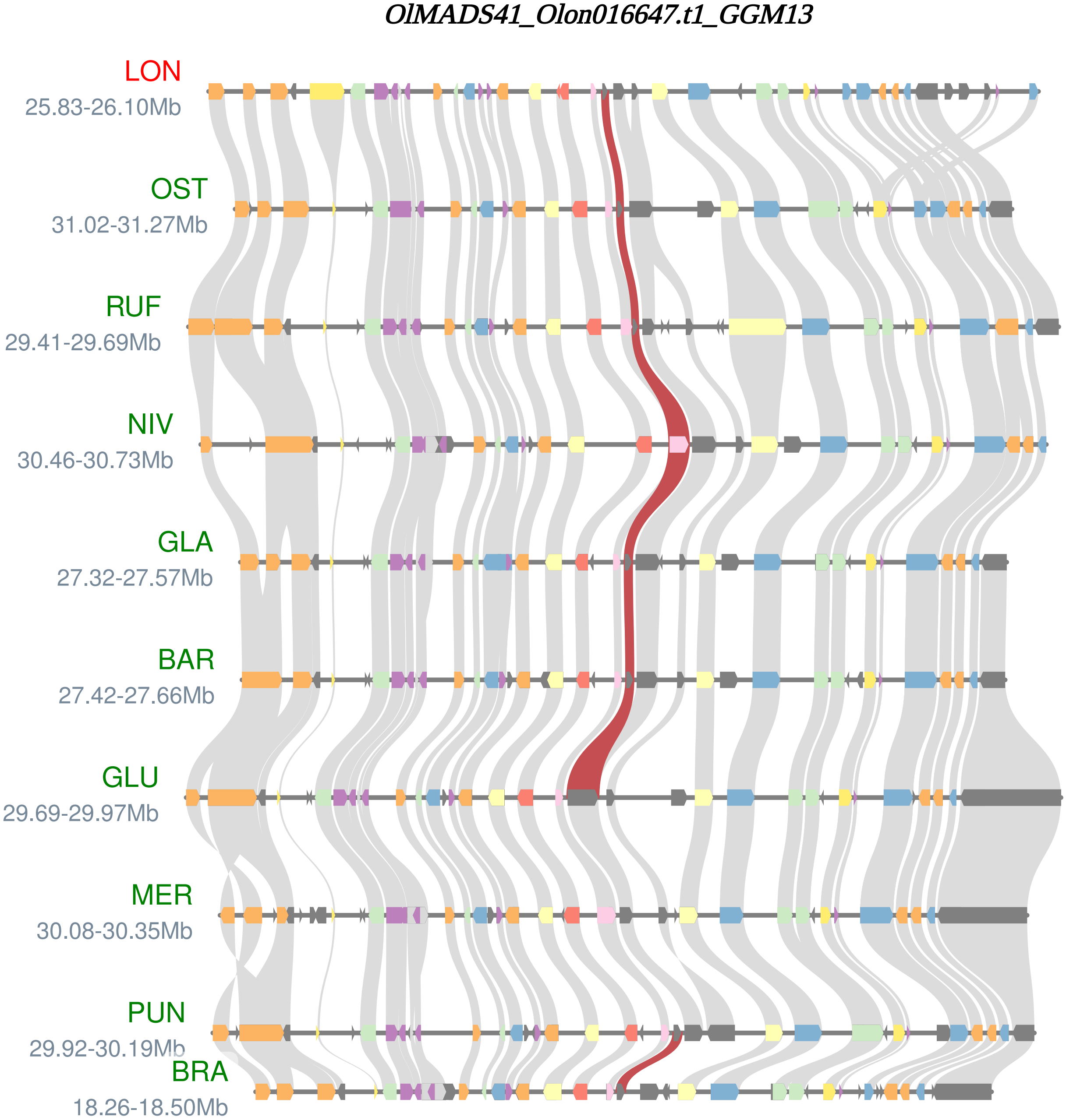

OlMADS41_Olon016647.t1_GGM13
LON
25.83-26.10Mb
OST
31.02-31.27Mb
RUF
29.41-29.69Mb
NIV
30.46-30.73Mb
GLA
27.32-27.57Mb
BAR
27.42-27.66Mb
GLU
29.69-29.97Mb
MER
30.08-30.35Mb
PUN
29.92-30.19Mb
BRA
18.26-18.50Mb

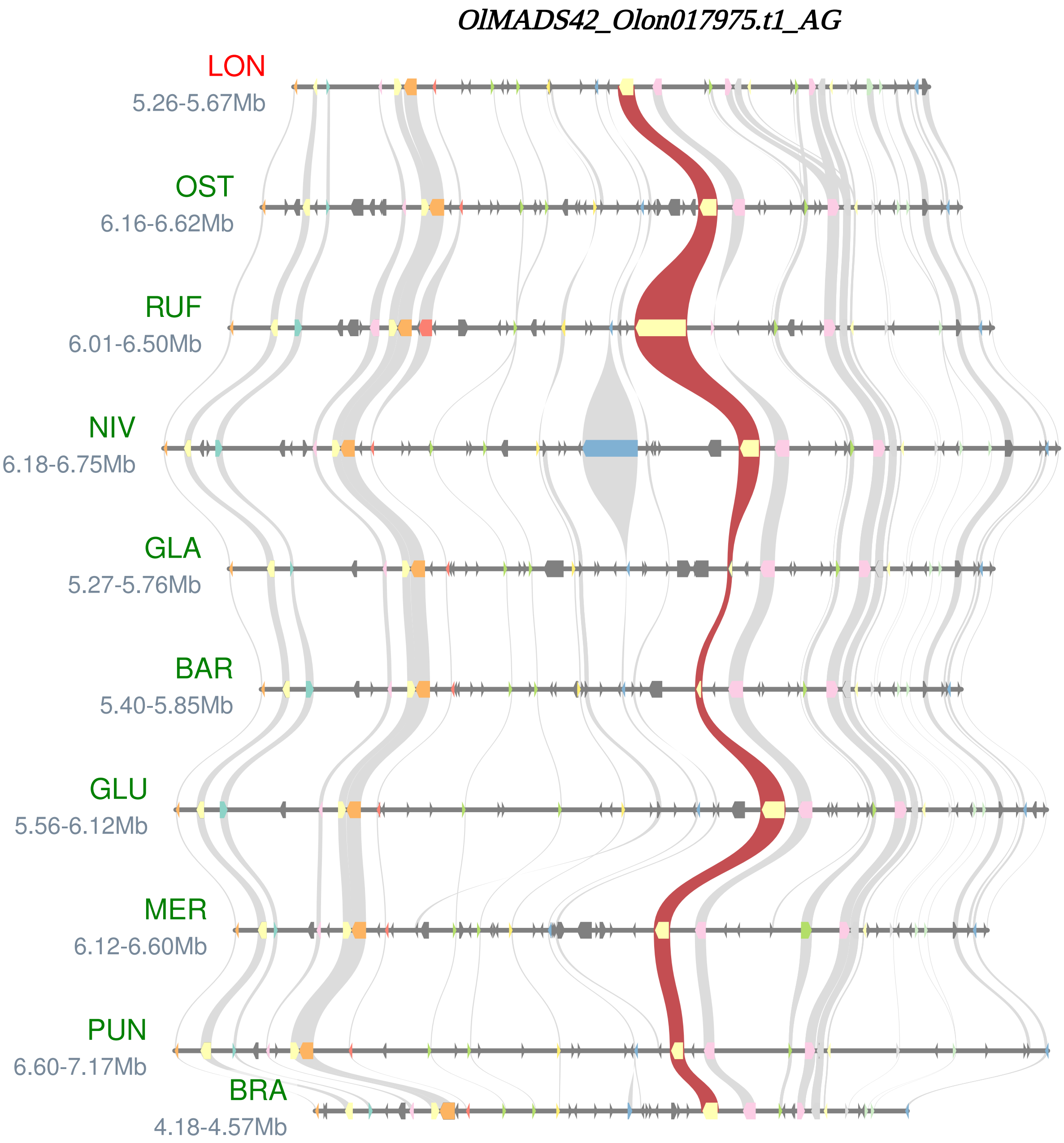

OlMADS42_Olon017975.t1_AG
LON
5.26-5.67Mb
OST
6.16-6.62Mb
RUF
6.01-6.50Mb
NIV
6.18-6.75Mb
GLA
5.27-5.76Mb
BAR
5.40-5.85Mb
GLU
5.56-6.12Mb
MER
6.12-6.60Mb
PUN
6.60-7.17Mb
BRA
4.18-4.57Mb

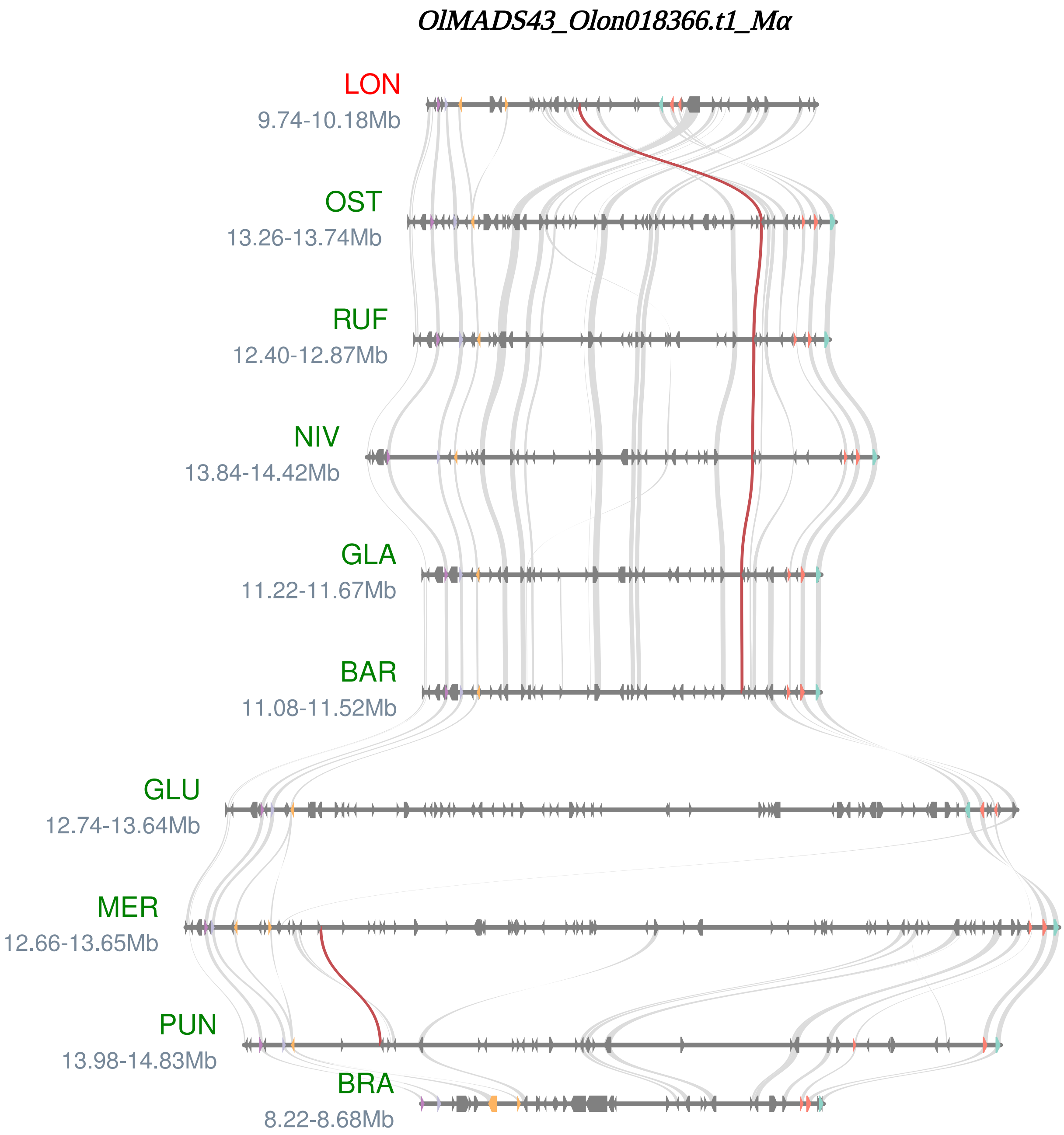
OlMADS43_Olon018366.t1_Mα
LON
9.74-10.18Mb
OST
13.26-13.74Mb
RUF
12.40-12.87Mb
NIV
13.84-14.42Mb
GLA
11.22-11.67Mb
BAR
11.08-11.52Mb
GLU
12.74-13.64Mb
MER
12.66-13.65Mb
PUN
13.98-14.83Mb
BRA
8.22-8.68Mb

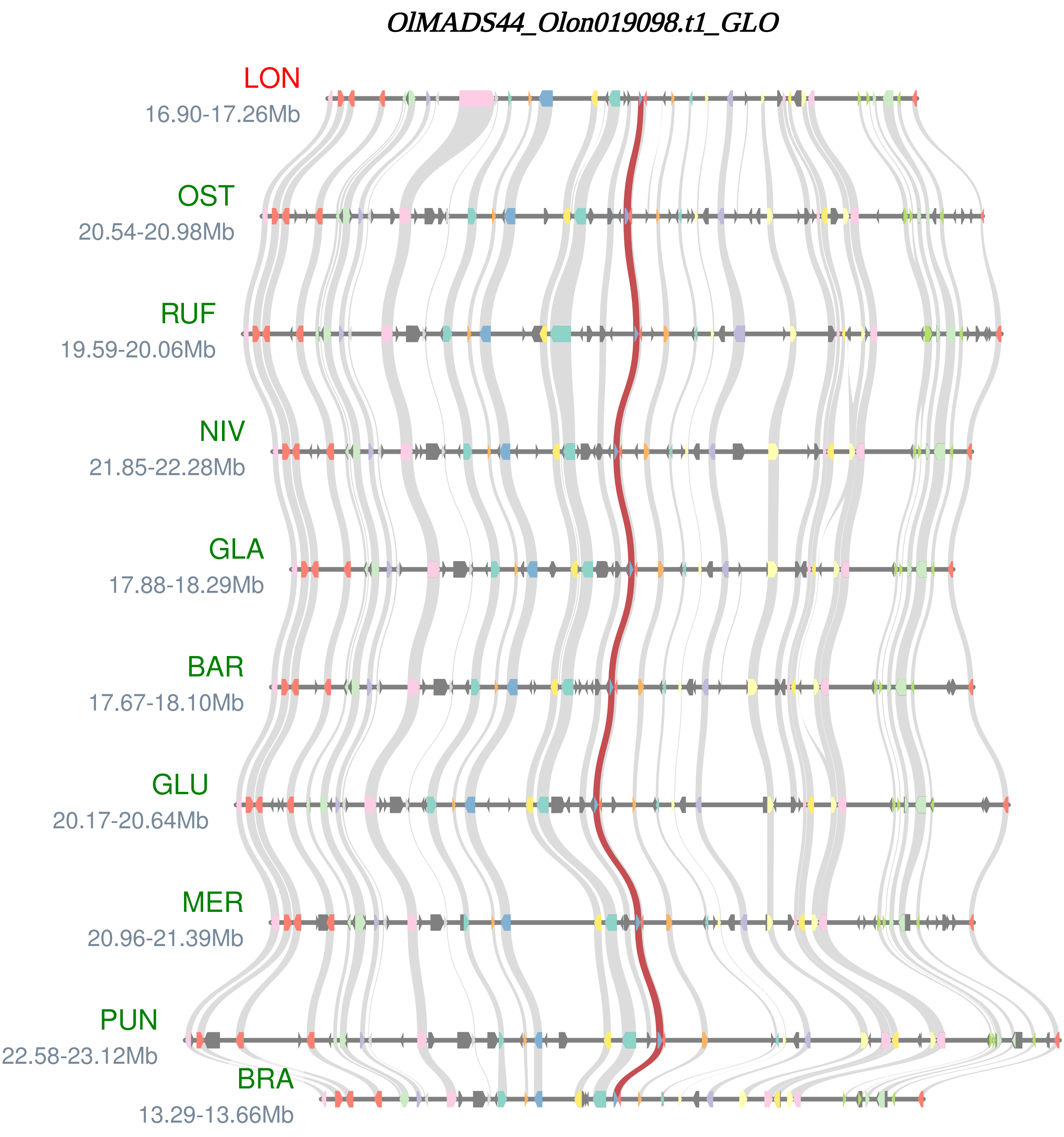

OlMADS44_Olon019098.t1_GLO
LON
16.90-17.26Mb
OST
20.54-20.98Mb
RUF
19.59-20.06Mb
NIV
21.85-22.28Mb
GLA
17.88-18.29Mb
BAR
17.67-18.10Mb
GLU
20.17-20.64Mb
MER
20.96-21.39Mb
PUN
22.58-23.12Mb
BRA
13.29-13.66Mb

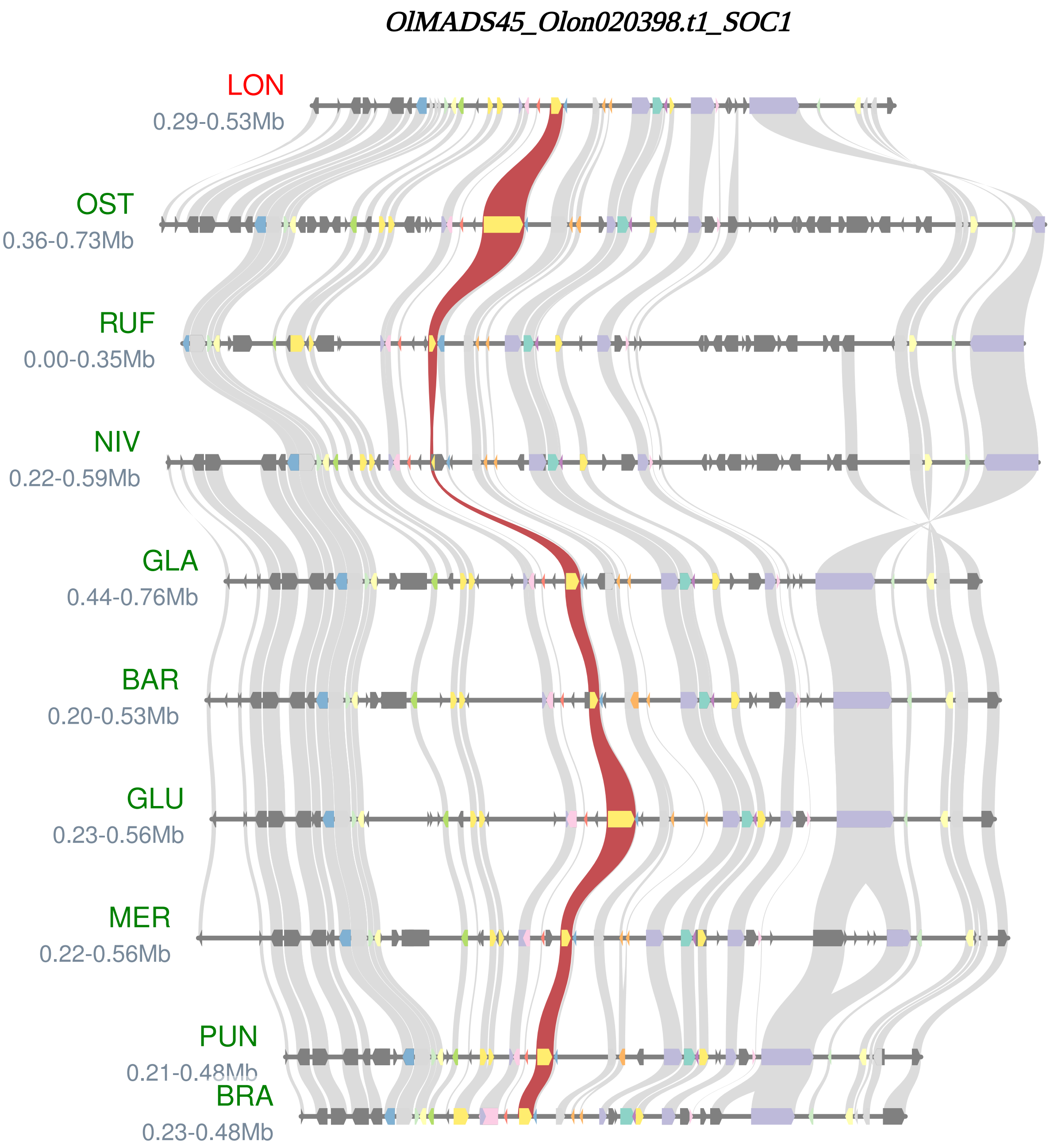
OlMADS45_Olon020398.t1_SOC1
LON
0.29-0.53Mb
OST
0.36-0.73Mb
RUF
0.00-0.35Mb
NIV
0.22-0.59Mb
GLA
0.44-0.76Mb
BAR
0.20-0.53Mb
GLU
0.23-0.56Mb
MER
0.22-0.56Mb
PUN
0.21-0.48Mb
BRA
0.23-0.48Mb

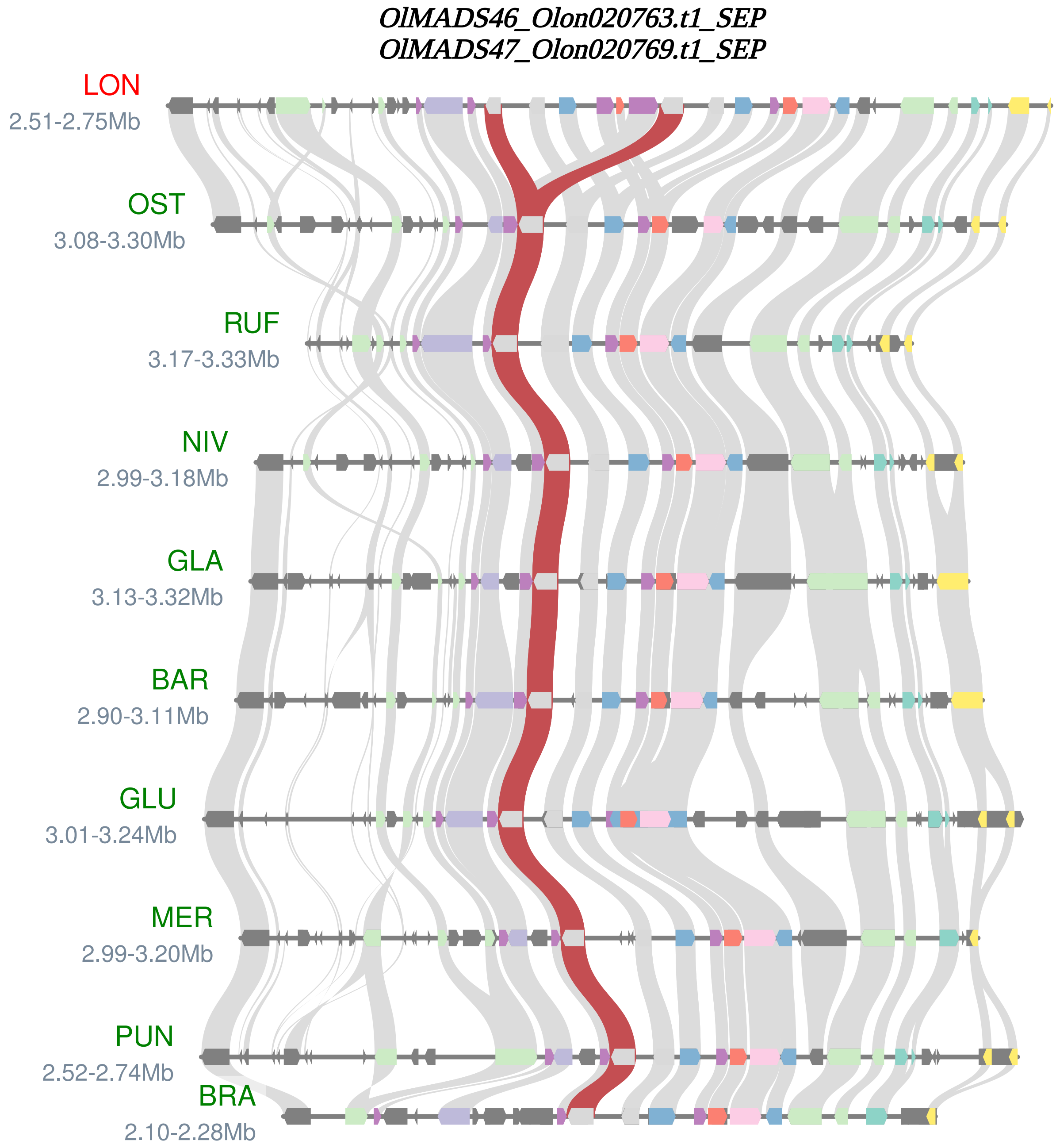
OlMADS46_Olon020763.t1_SEP
OlMADS47_Olon020769.t1_SEP
LON
2.51-2.75Mb
OST
3.08-3.30Mb
RUF
3.17-3.33Mb
NIV
2.99-3.18Mb
GLA
3.13-3.32Mb
BAR
2.90-3.11Mb
GLU
3.01-3.24Mb
MER
2.99-3.20Mb
PUN
2.52-2.74Mb
BRA
2.10-2.28Mb

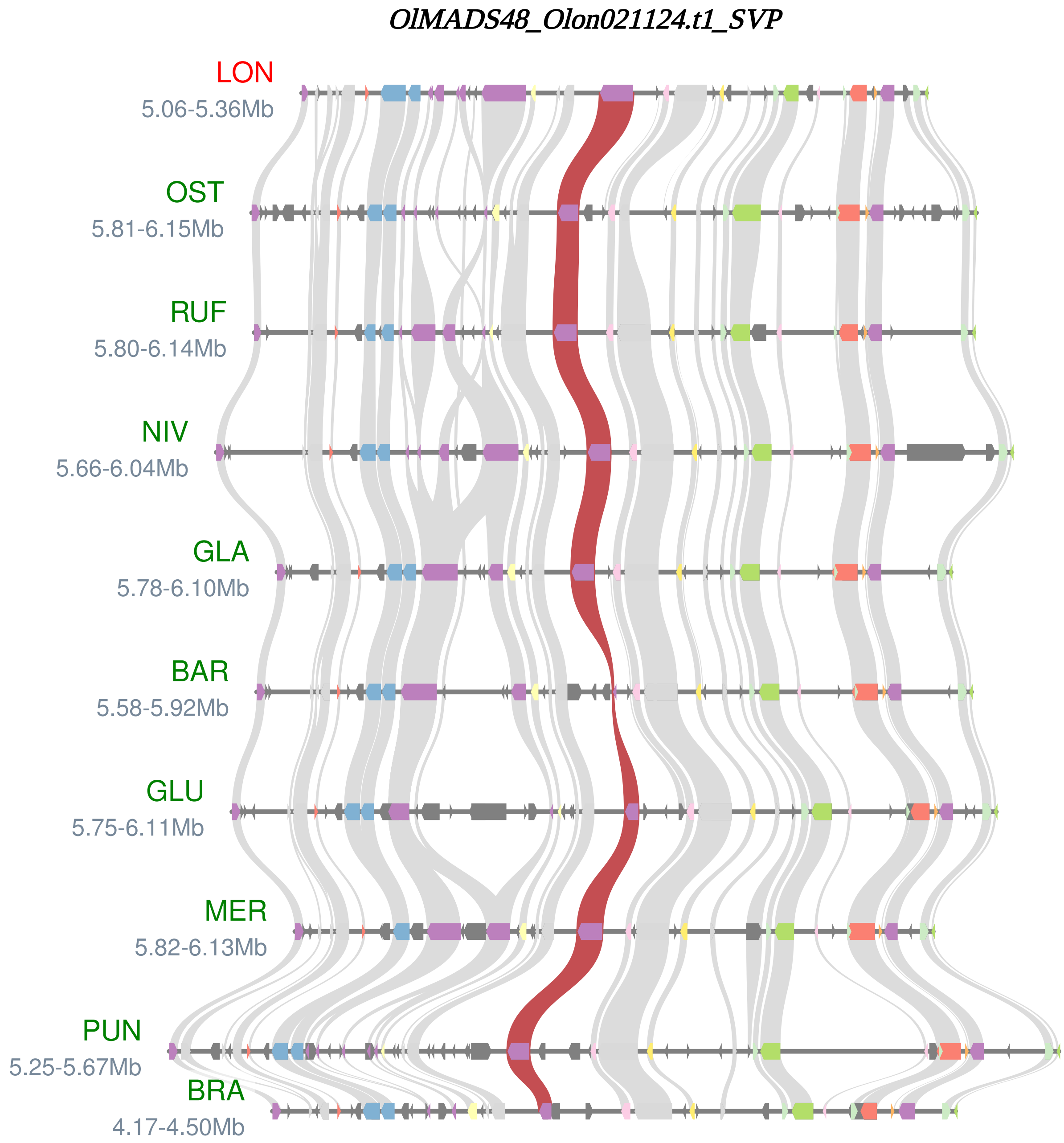
OlMADS48_Olon021124.t1_SVP
LON
5.06-5.36Mb
OST
5.81-6.15Mb
RUF
5.80-6.14Mb
NIV
5.66-6.04Mb
GLA
5.78-6.10Mb
BAR
5.58-5.92Mb
GLU
5.75-6.11Mb
MER
5.82-6.13Mb
PUN
5.25-5.67Mb
BRA
4.17-4.50Mb

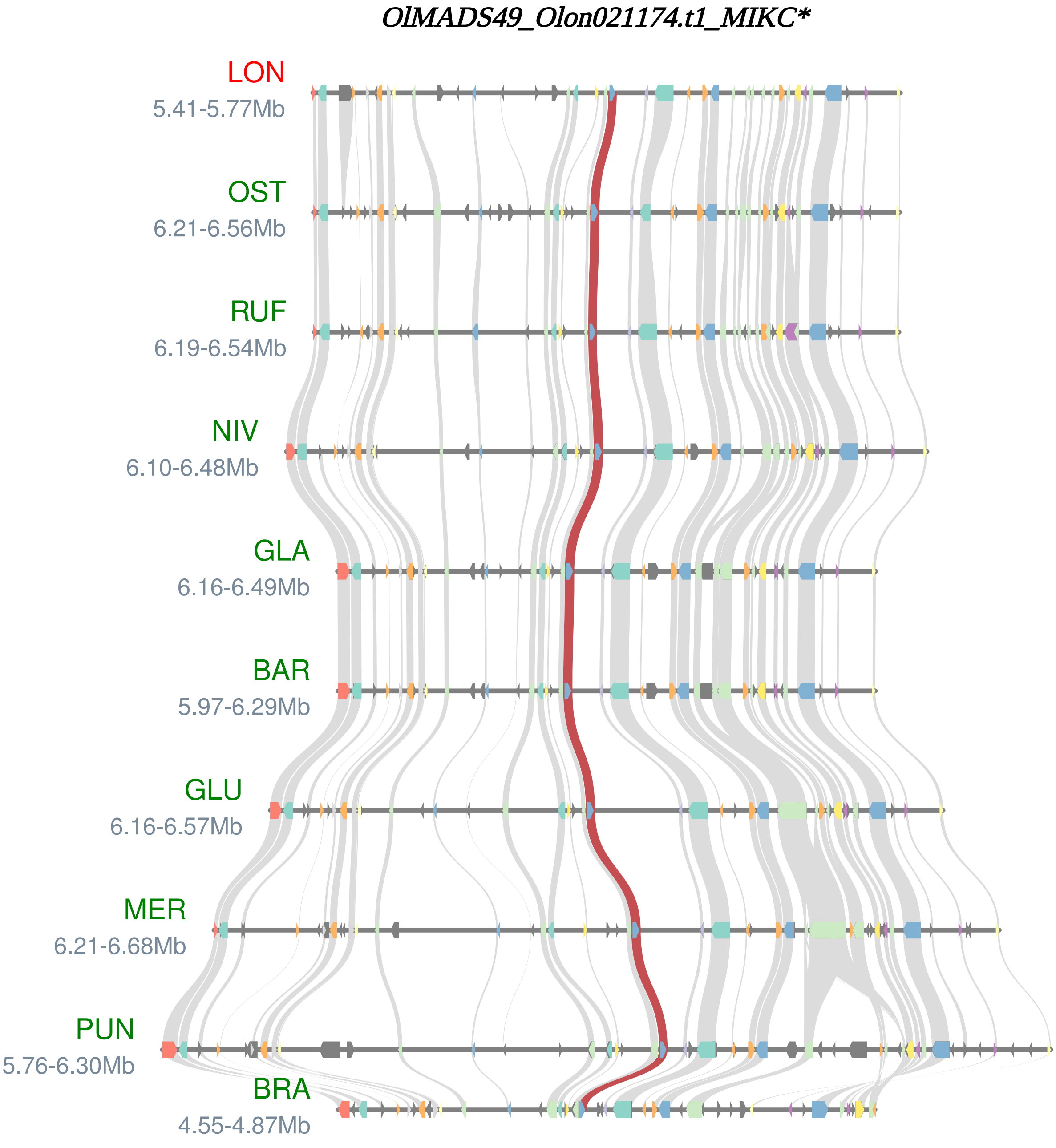
OlMADS49_Olon021174.t1_MIKC*
LON
5.41-5.77Mb
OST
6.21-6.56Mb
RUF
6.19-6.54Mb
NIV
6.10-6.48Mb
GLA
6.16-6.49Mb
BAR
5.97-6.29Mb
GLU
6.16-6.57Mb
MER
6.21-6.68Mb
PUN
5.76-6.30Mb
BRA
4.55-4.87Mb

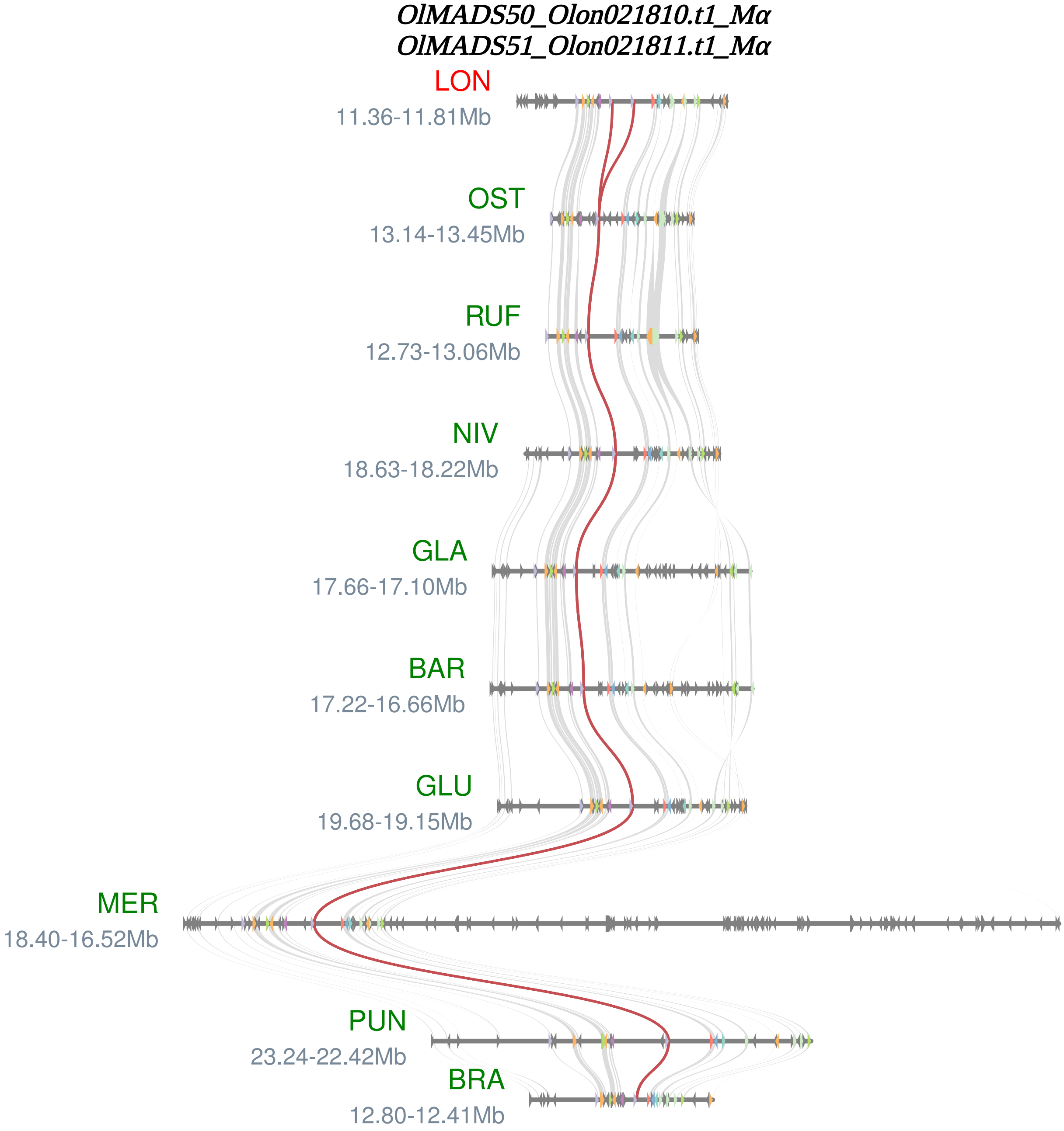

OlMADS50_Olon021810.t1_Mα
OlMADS51_Olon021811.t1_Mα
LON
11.36-11.81Mb
OST
13.14-13.45Mb
RUF
12.73-13.06Mb
NIV
18.63-18.22Mb
GLA
17.66-17.10Mb
BAR
17.22-16.66Mb
GLU
19.68-19.15Mb
MER
18.40-16.52Mb
PUN
23.24-22.42Mb
BRA
12.80-12.41Mb

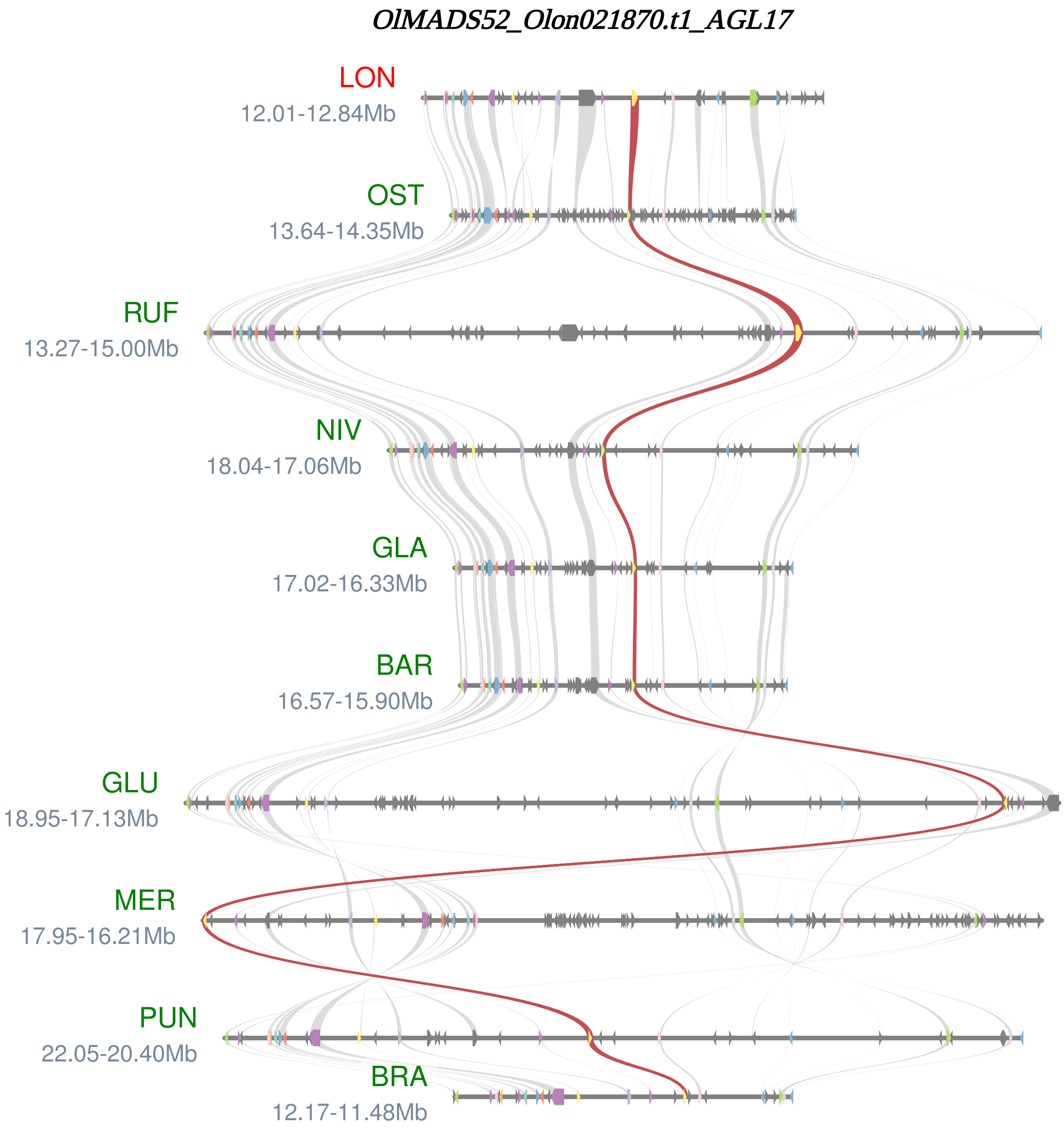
OlMADS52_Olon021870.t1_AGL17
LON
12.01-12.84Mb
OST
13.64-14.35Mb
RUF
13.27-15.00Mb
NIV
18.04-17.06Mb
GLA
17.02-16.33Mb
BAR
16.57-15.90Mb
GLU
18.95-17.13Mb
MER
17.95-16.21Mb
PUN
22.05-20.40Mb
BRA
12.17-11.48Mb

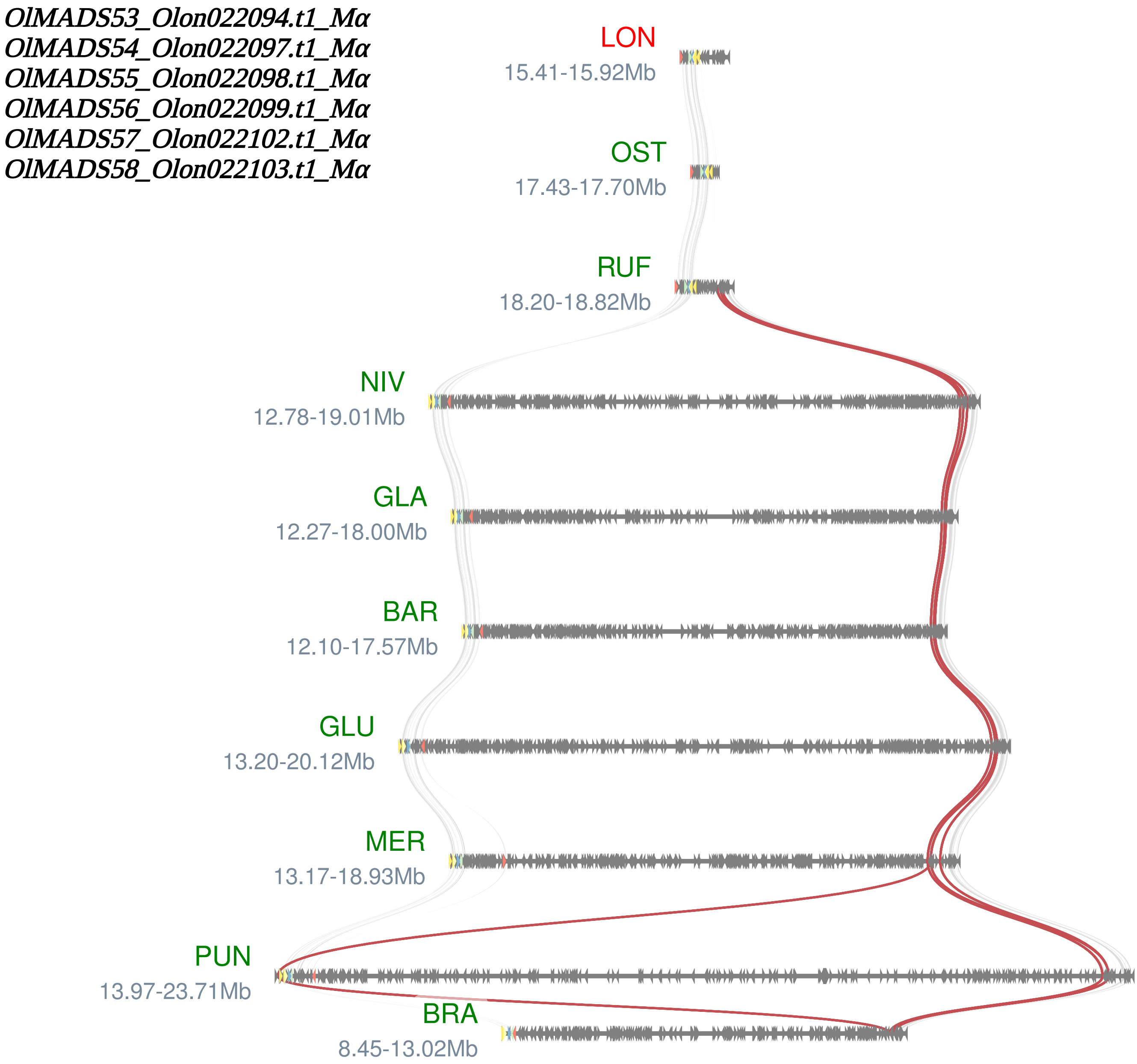

OlMADS53_Olon022094.t1_Mα
OlMADS54_Olon022097.t1_Mα
OlMADS55_Olon022098.t1_Mα
OlMADS56_Olon022099.t1_Mα
OlMADS57_Olon022102.t1_Mα
OlMADS58_Olon022103.t1_Mα
LON
15.41-15.92Mb
OST
17.43-17.70Mb
RUF
18.20-18.82Mb
NIV
12.78-19.01Mb
GLA
12.27-18.00Mb
BAR
12.10-17.57Mb
GLU
13.20-20.12Mb
MER
13.17-18.93Mb
PUN
13.97-23.71Mb
BRA
8.45-13.02Mb

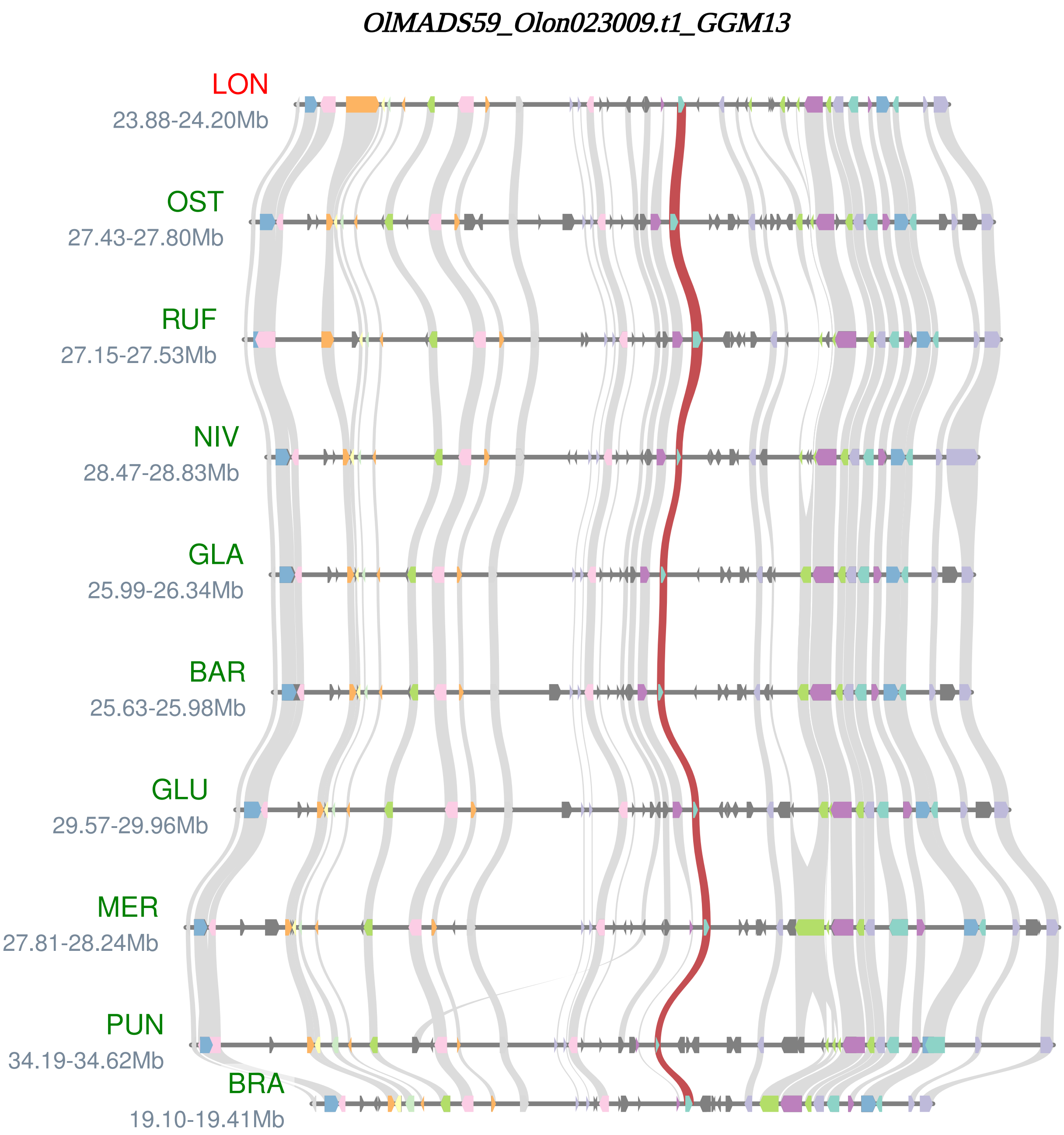
OlMADS59_Olon023009.t1_GGM13
LON
23.88-24.20Mb
OST
27.43-27.80Mb
RUF
27.15-27.53Mb
NIV
28.47-28.83Mb
GLA
25.99-26.34Mb
BAR
25.63-25.98Mb
GLU
29.57-29.96Mb
MER
27.81-28.24Mb
PUN
34.19-34.62Mb
BRA
19.10-19.41Mb

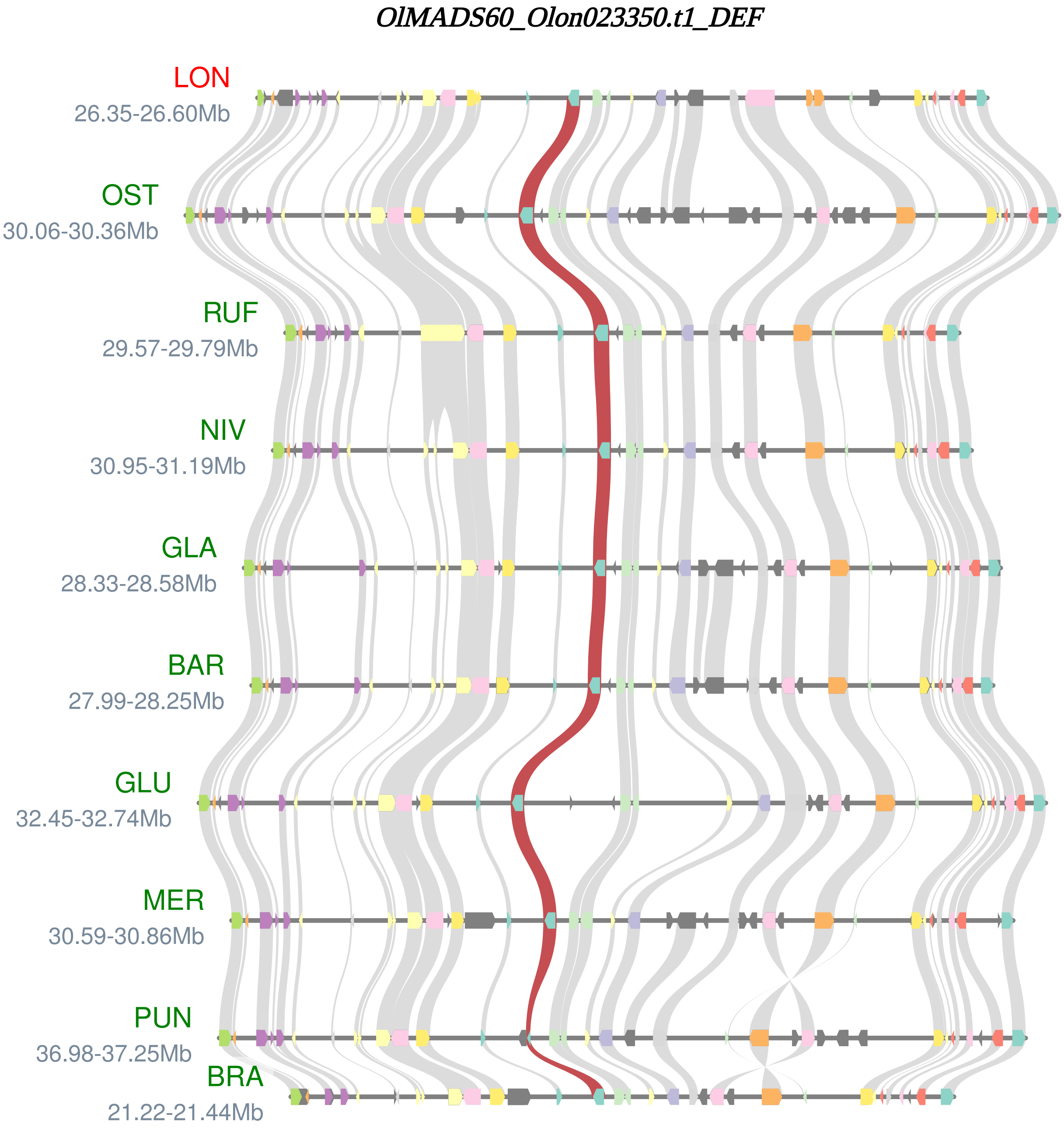
OlMADS60_Olon023350.t1_DEF
LON
26.35-26.60Mb
OST
30.06-30.36Mb
RUF
29.57-29.79Mb
NIV
30.95-31.19Mb
GLA
28.33-28.58Mb
BAR
27.99-28.25Mb
GLU
32.45-32.74Mb
MER
30.59-30.86Mb
PUN
36.98-37.25Mb
BRA
21.22-21.44Mb

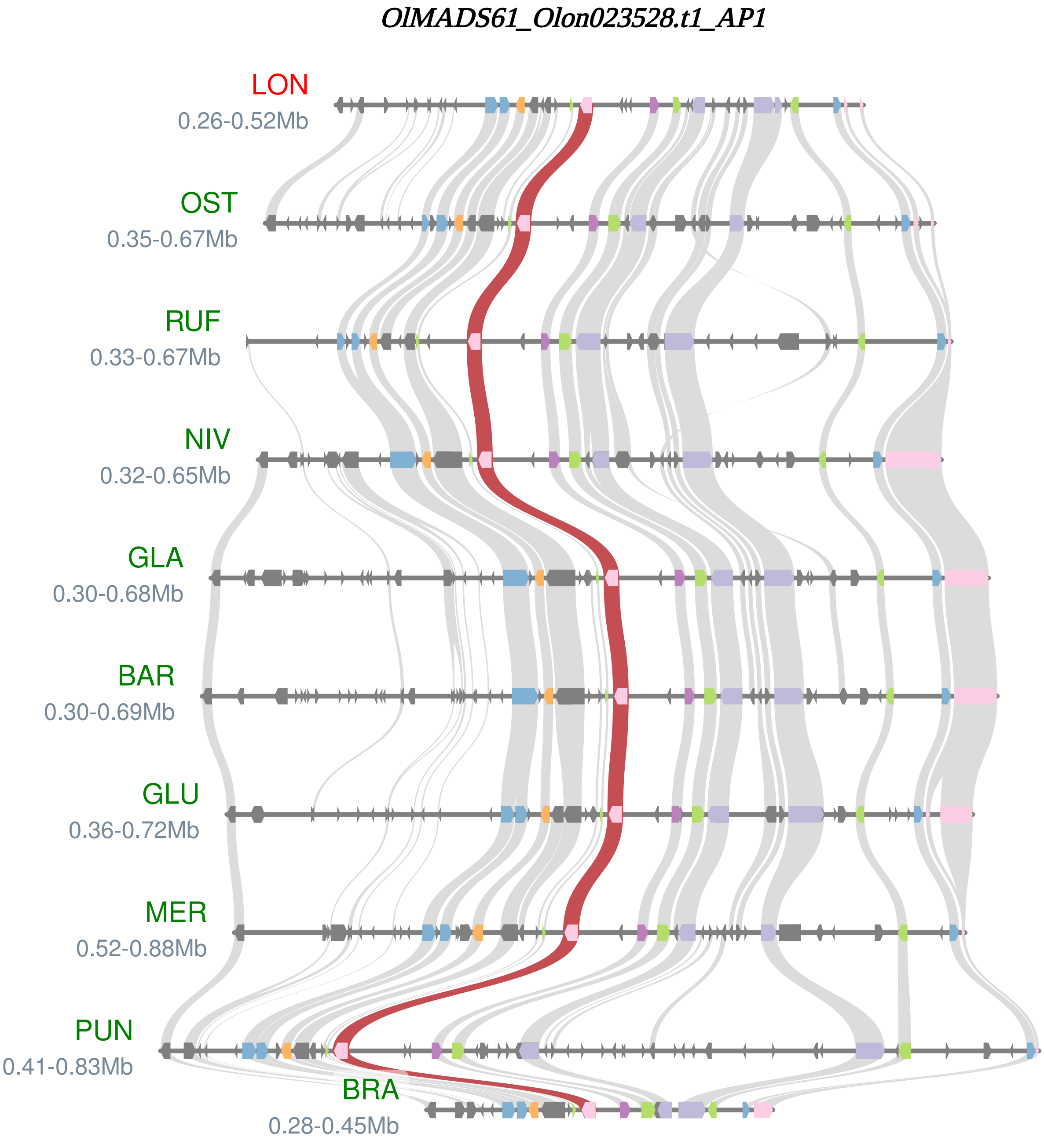
OlMADS61_Olon023528.t1_AP1
LON
0.26-0.52Mb
OST
0.35-0.67Mb
RUF
0.33-0.67Mb
NIV
0.32-0.65Mb
GLA
0.30-0.68Mb
BAR
0.30-0.69Mb
GLU
0.36-0.72Mb
MER
0.52-0.88Mb
PUN
0.41-0.83Mb
BRA
0.28-0.45Mb

OlMADS62_Olon023730.t1_Mβ

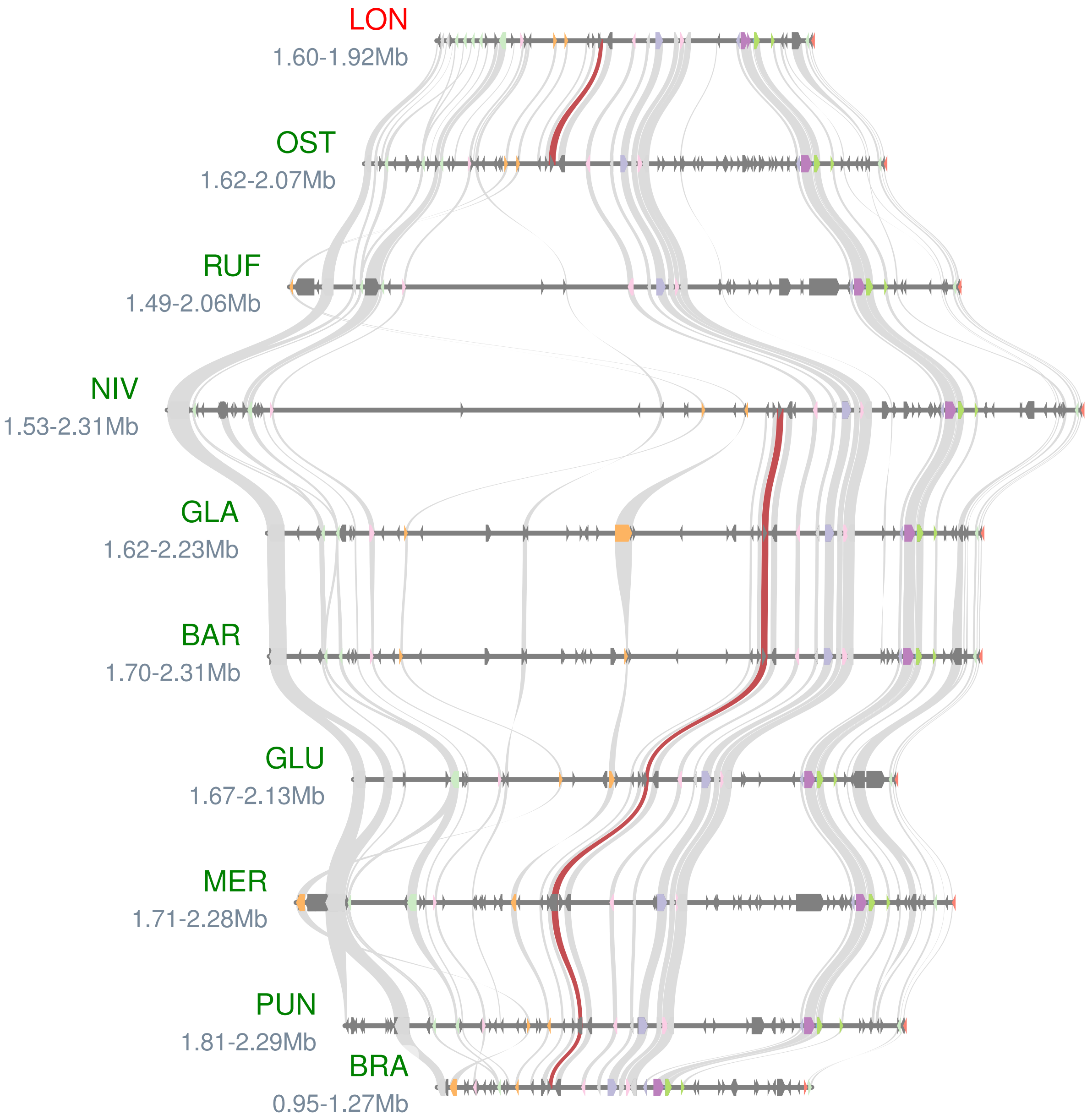

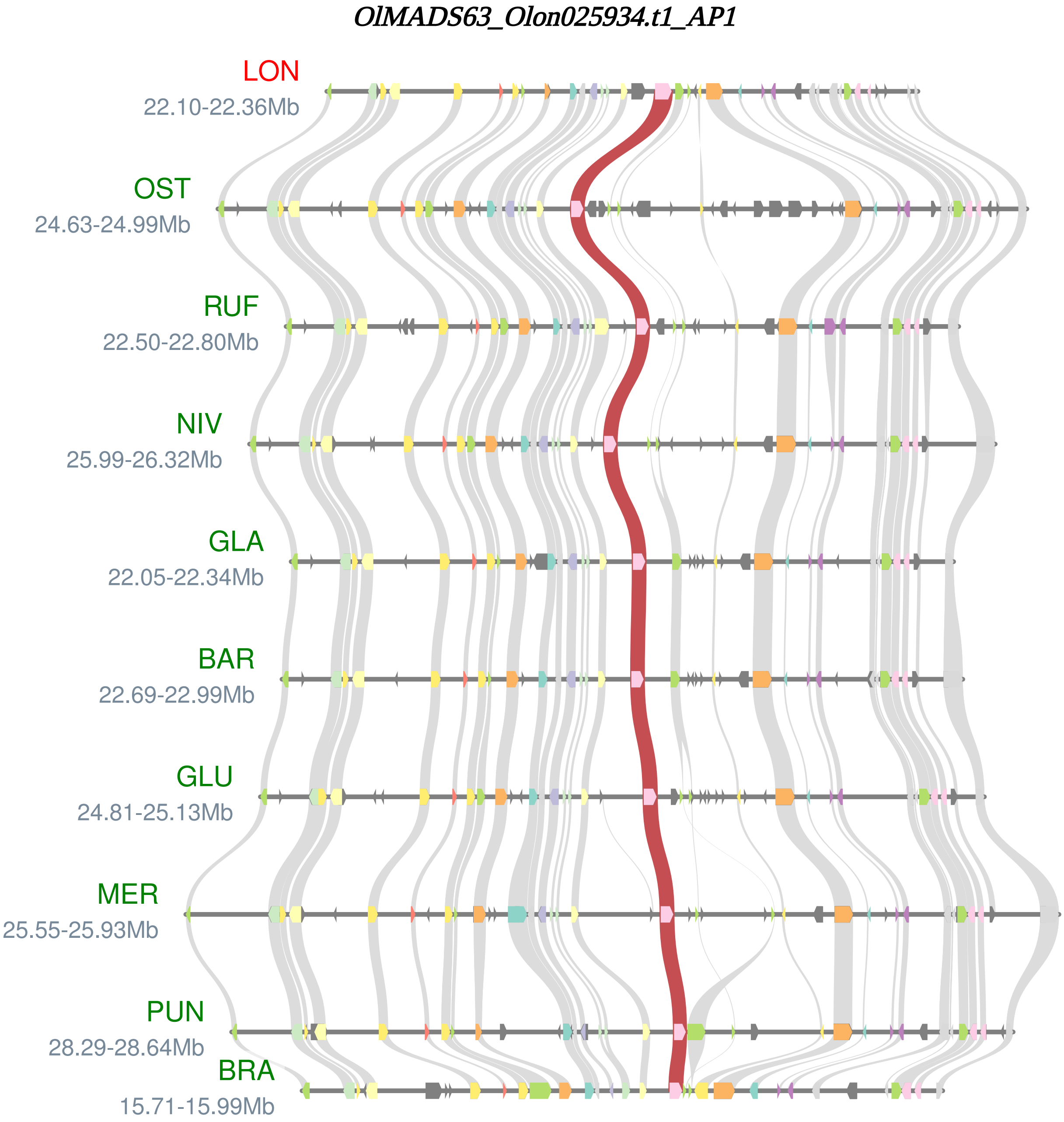

OlMADS63_Olon025934.t1_AP1
LON
22.10-22.36Mb
OST
24.63-24.99Mb
RUF
22.50-22.80Mb
NIV
25.99-26.32Mb
GLA
22.05-22.34Mb
BAR
22.69-22.99Mb
GLU
24.81-25.13Mb
MER
25.55-25.93Mb
PUN
28.29-28.64Mb
BRA
15.71-15.99Mb

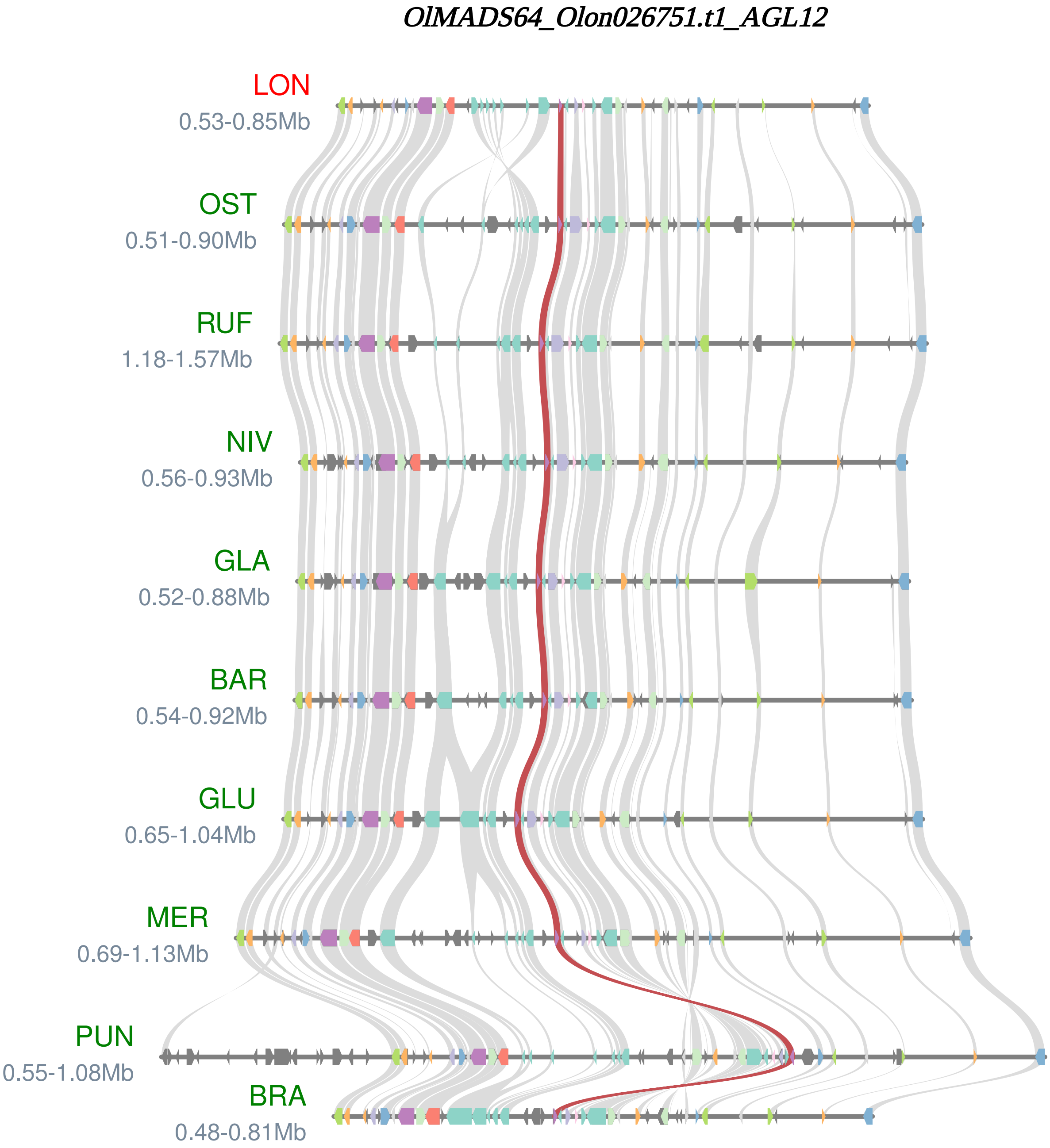
OlMADS64_Olon026751.t1_AGL12
LON
0.53-0.85Mb
OST
0.51-0.90Mb
RUF
1.18-1.57Mb
NIV
0.56-0.93Mb
GLA
0.52-0.88Mb
BAR
0.54-0.92Mb
GLU
0.65-1.04Mb
MER
0.69-1.13Mb
PUN
0.55-1.08Mb
BRA
0.48-0.81Mb

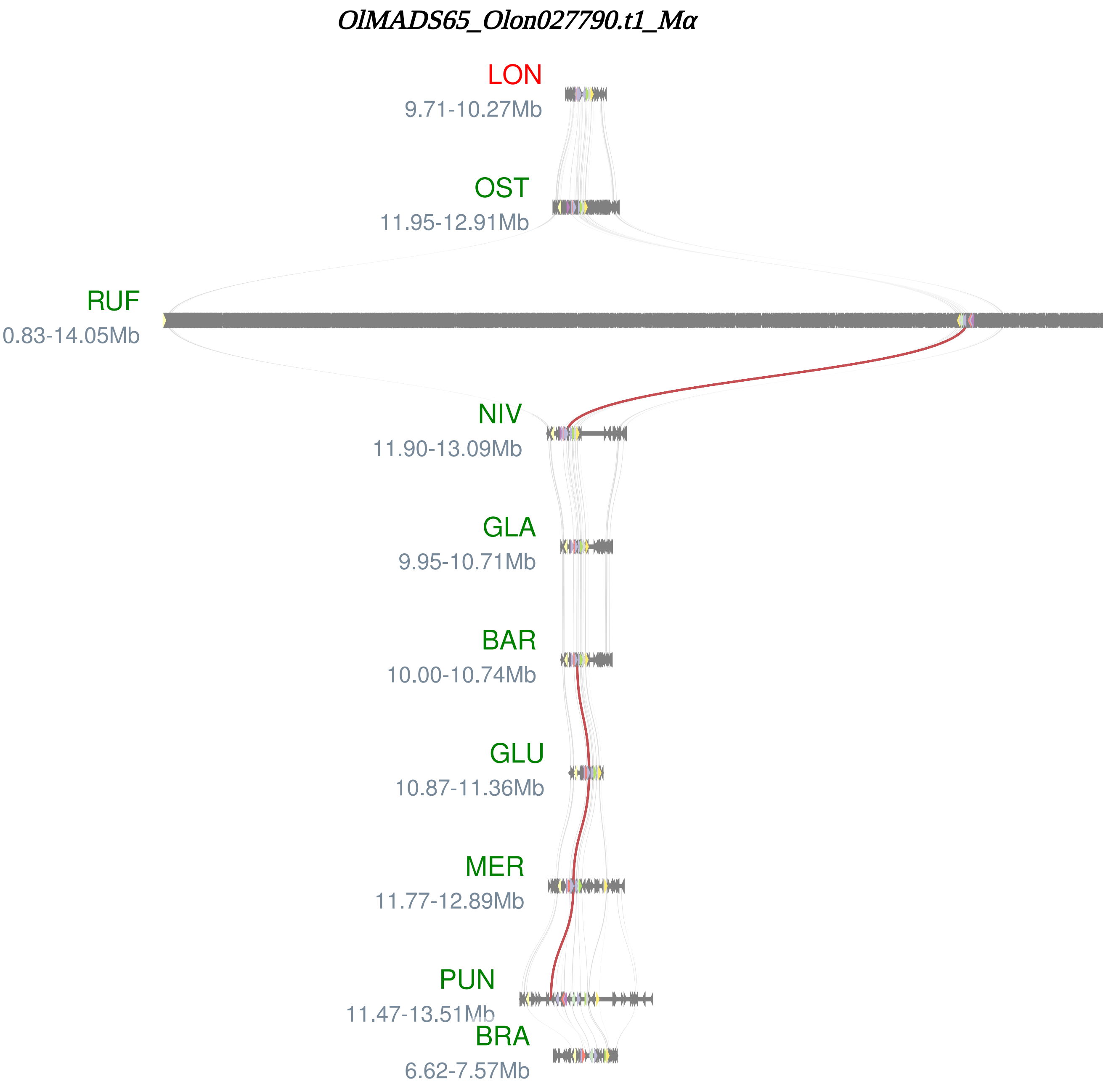
OlMADS65_Olon027790.t1_Mα
LON
9.71-10.27Mb
OST
11.95-12.91Mb
RUF
0.83-14.05Mb
NIV
11.90-13.09Mb
GLA
9.95-10.71Mb
BAR
10.00-10.74Mb
GLU
10.87-11.36Mb
MER
11.77-12.89Mb
PUN
11.47-13.51Mb
BRA
6.62-7.57Mb

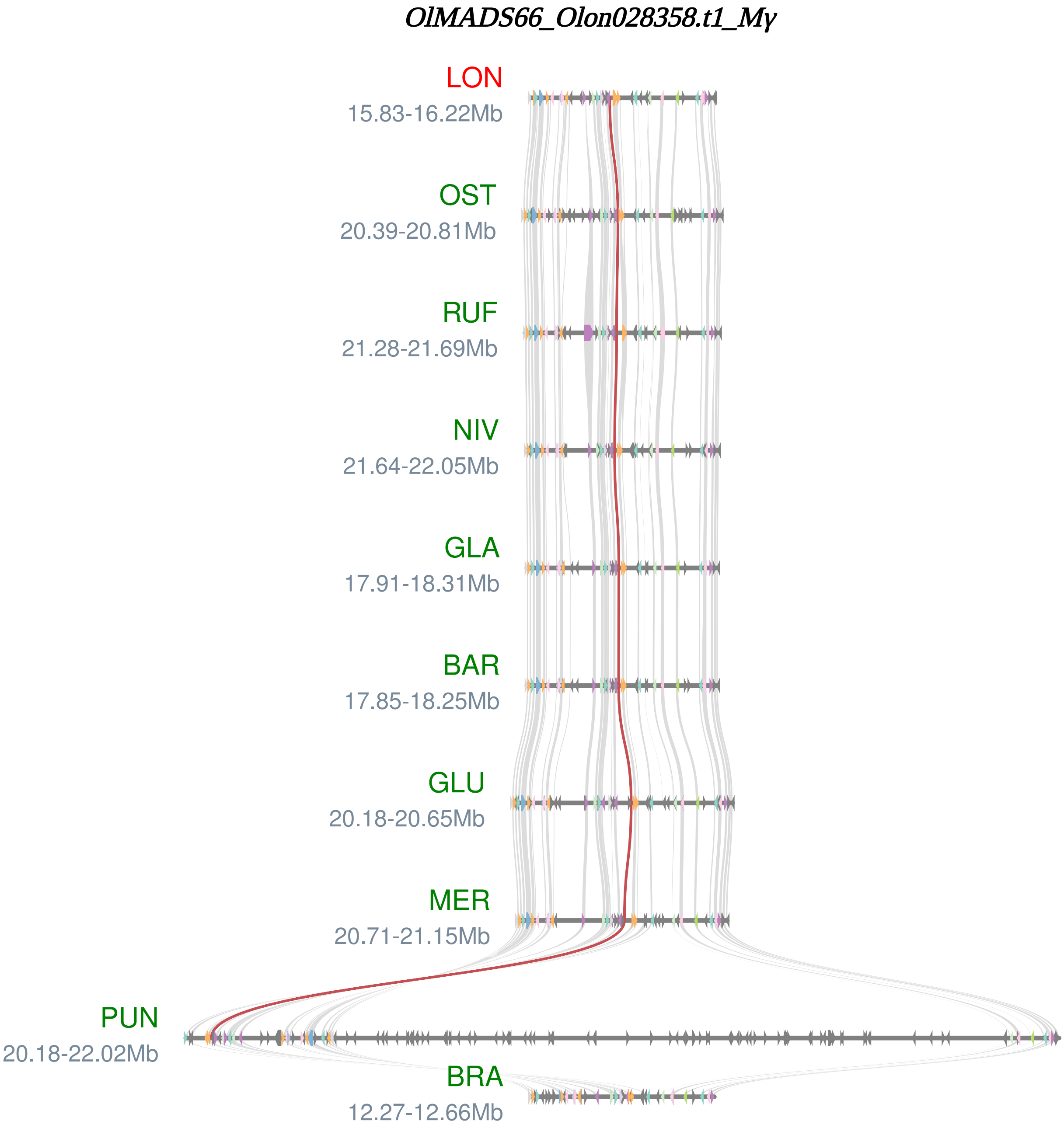
OlMADS66_Olon028358.t1_Mγ
LON
15.83-16.22Mb
OST
20.39-20.81Mb
RUF
21.28-21.69Mb
NIV
21.64-22.05Mb
GLA
17.91-18.31Mb
BAR
17.85-18.25Mb
GLU
20.18-20.65Mb
MER
20.71-21.15Mb
PUN
20.18-22.02Mb
BRA
12.27-12.66Mb

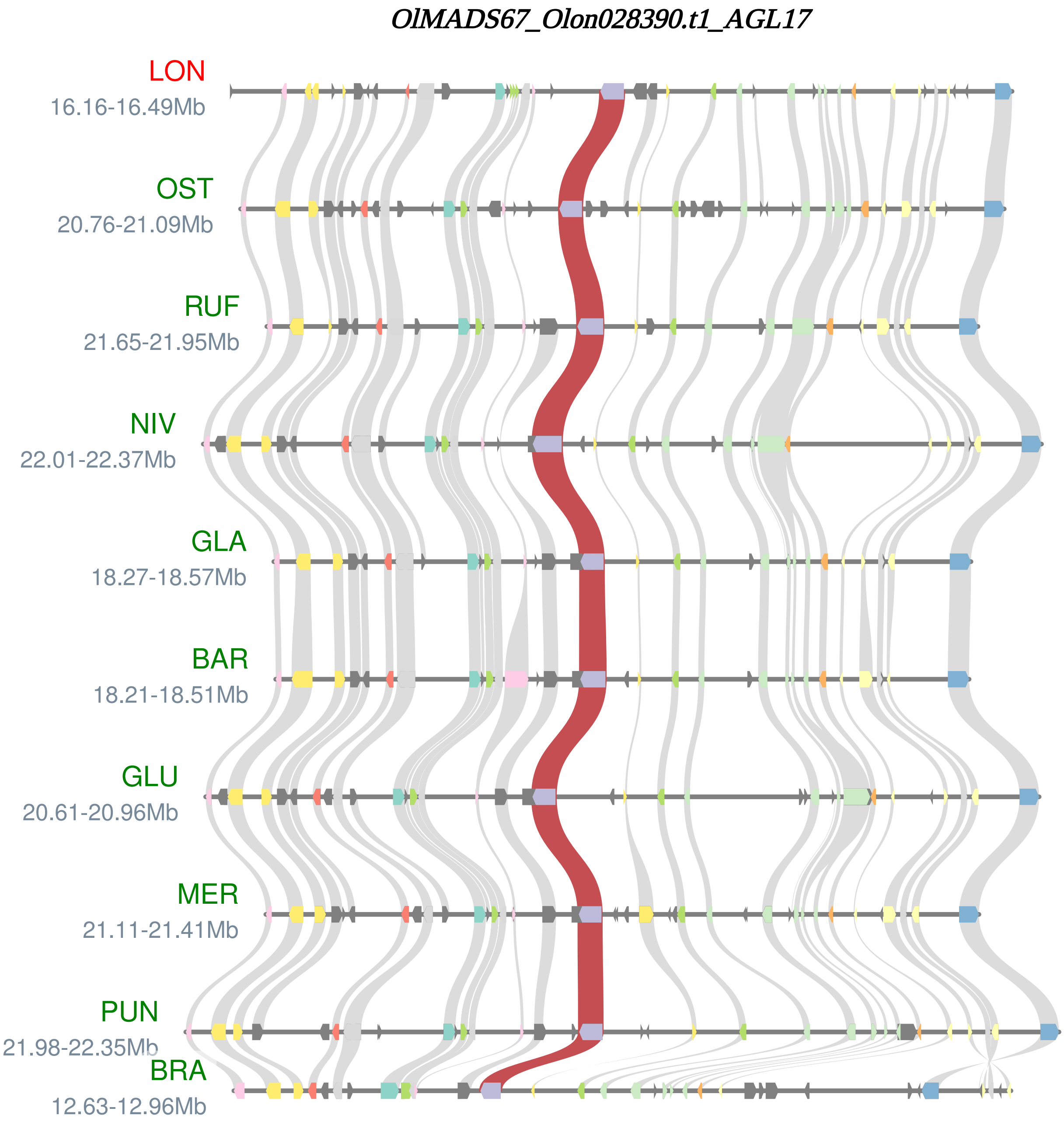
OlMADS67_Olon028390.t1_AGL17
LON
16.16-16.49Mb
OST
20.76-21.09Mb
RUF
21.65-21.95Mb
NIV
22.01-22.37Mb
GLA
18.27-18.57Mb
BAR
18.21-18.51Mb
GLU
20.61-20.96Mb
MER
21.11-21.41Mb
PUN
21.98-22.35Mb
BRA
12.63-12.96Mb

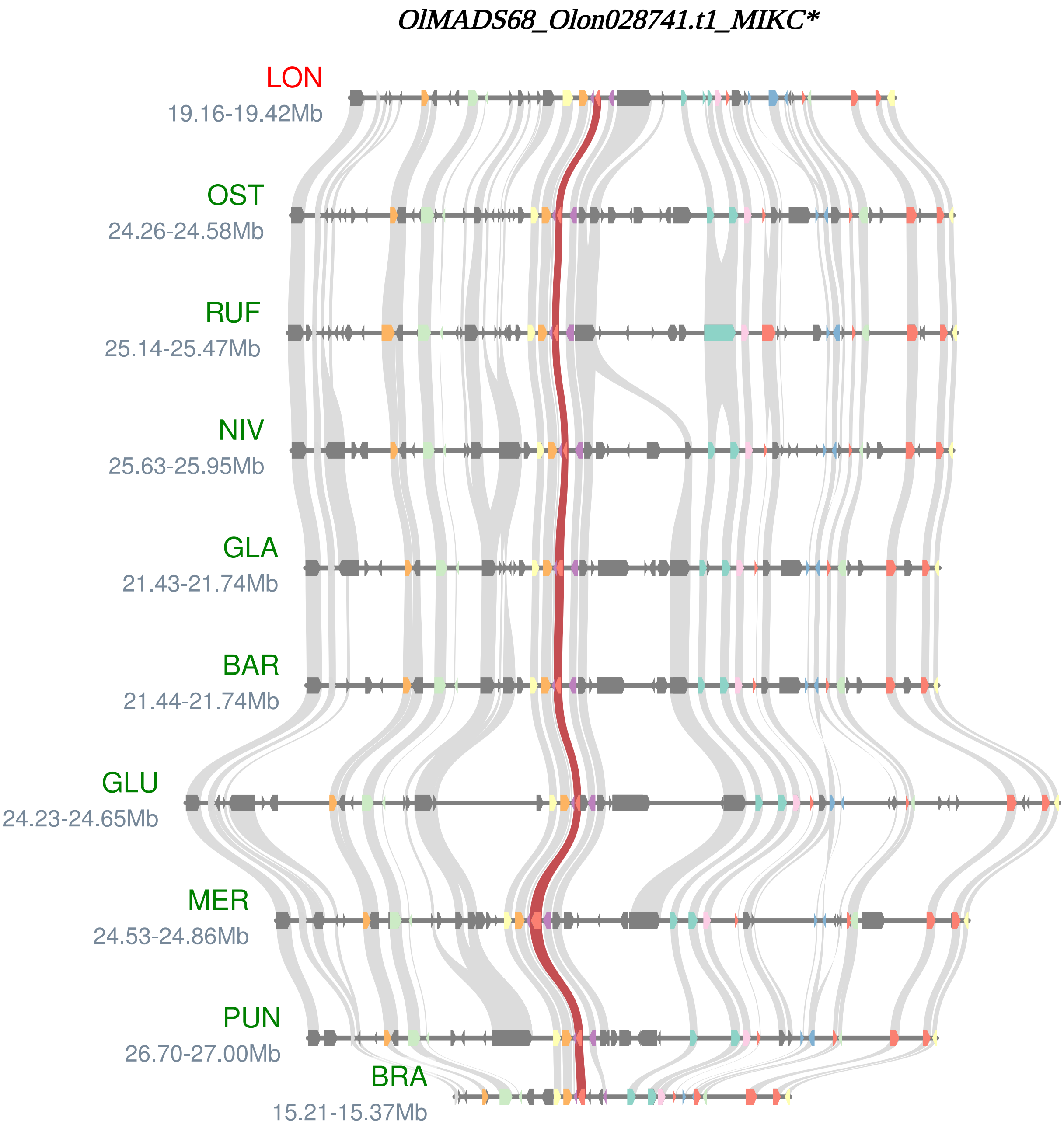
OlMADS68_Olon028741.t1_MIKC*
LON
19.16-19.42Mb
OST
24.26-24.58Mb
RUF
25.14-25.47Mb
NIV
25.63-25.95Mb
GLA
21.43-21.74Mb
BAR
21.44-21.74Mb
GLU
24.23-24.65Mb
MER
24.53-24.86Mb
PUN
26.70-27.00Mb
BRA
15.21-15.37Mb

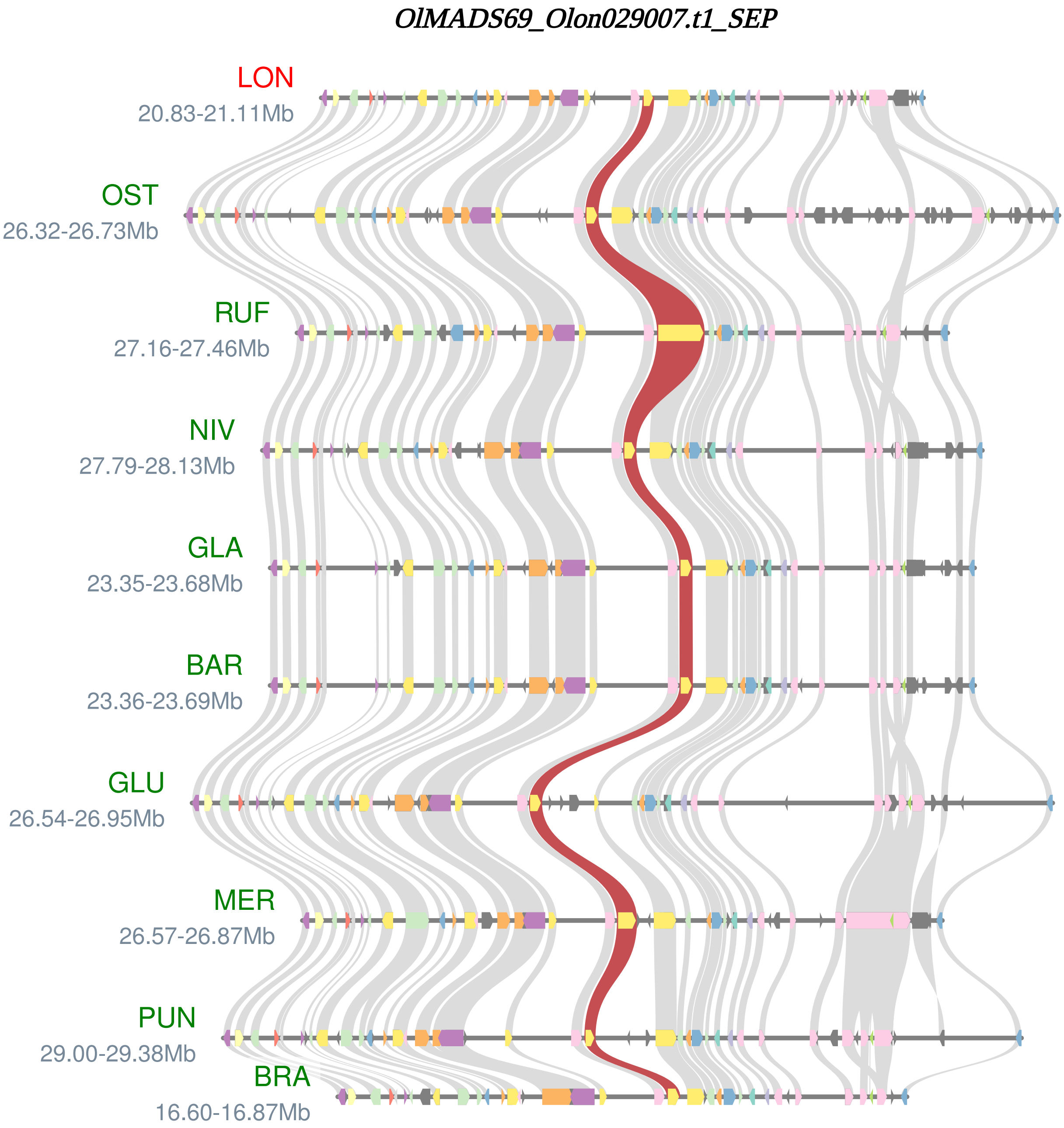

OlMADS69_Olon029007.t1_SEP
LON
20.83-21.11Mb
OST
26.32-26.73Mb
RUF
27.16-27.46Mb
NIV
27.79-28.13Mb
GLA
23.35-23.68Mb
BAR
23.36-23.69Mb
GLU
26.54-26.95Mb
MER
26.57-26.87Mb
PUN
29.00-29.38Mb
BRA
16.60-16.87Mb

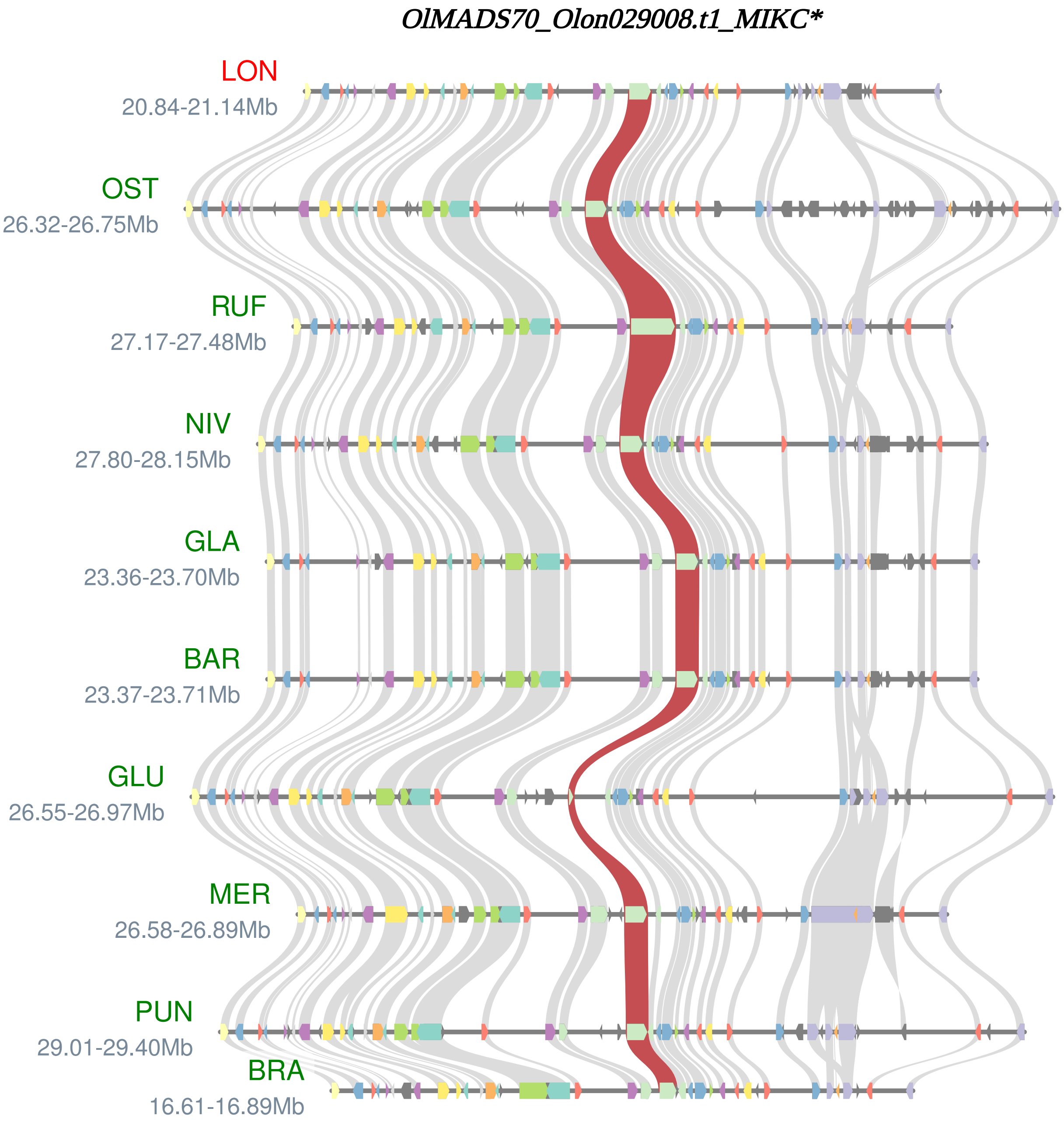
OlMADS70_Olon029008.t1_MIKC*
LON
20.84-21.14Mb
OST
26.32-26.75Mb
RUF
27.17-27.48Mb
NIV
27.80-28.15Mb
GLA
23.36-23.70Mb
BAR
23.37-23.71Mb
GLU
26.55-26.97Mb
MER
26.58-26.89Mb
PUN
29.01-29.40Mb
BRA
16.61-16.89Mb

OlMADS71_Olon029375.t1_Mα
OlMADS72_Olon029378.t1_Mα
（The chromosomal segment in the GLU lacks any detected syntenic genes.）

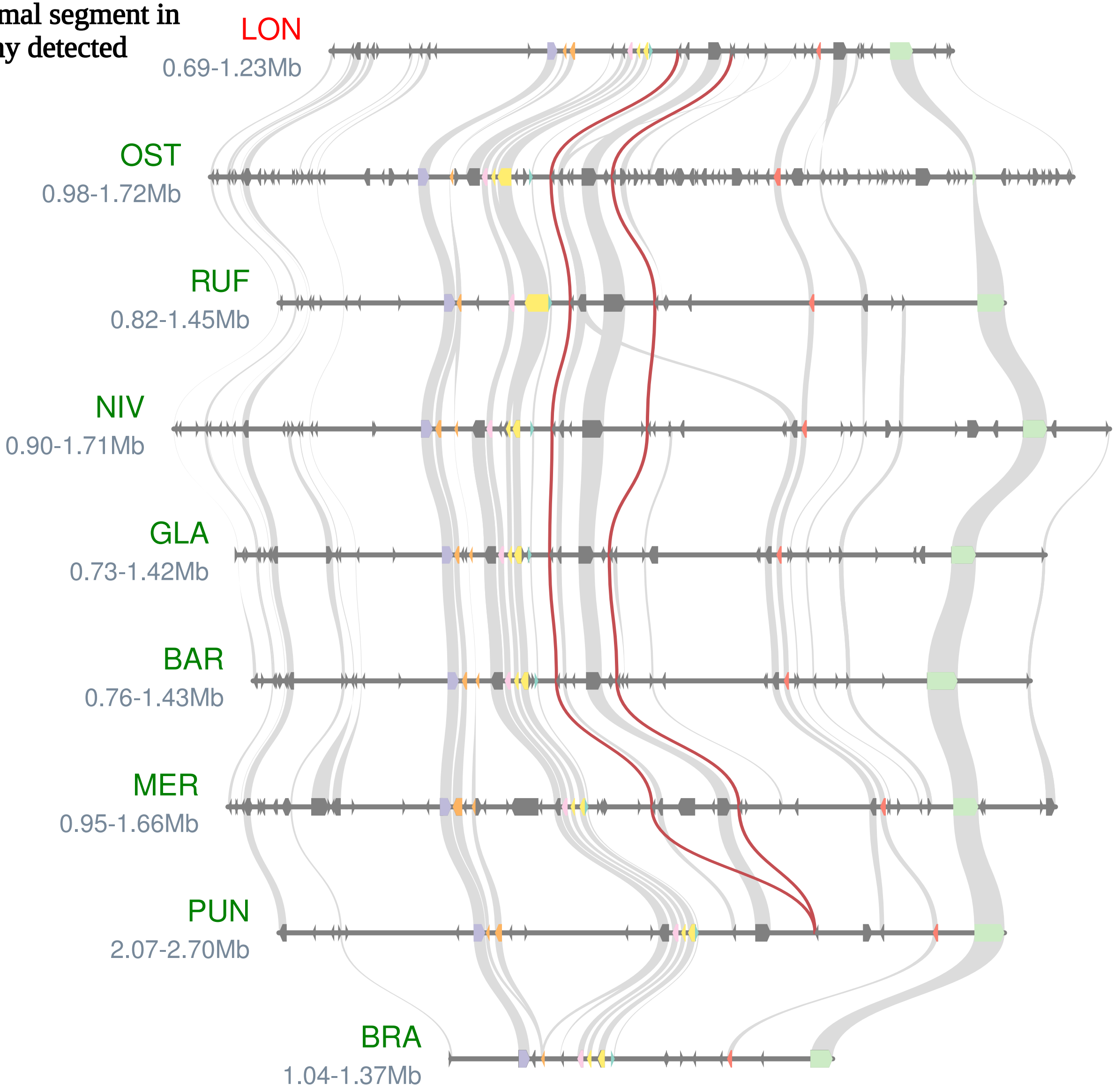

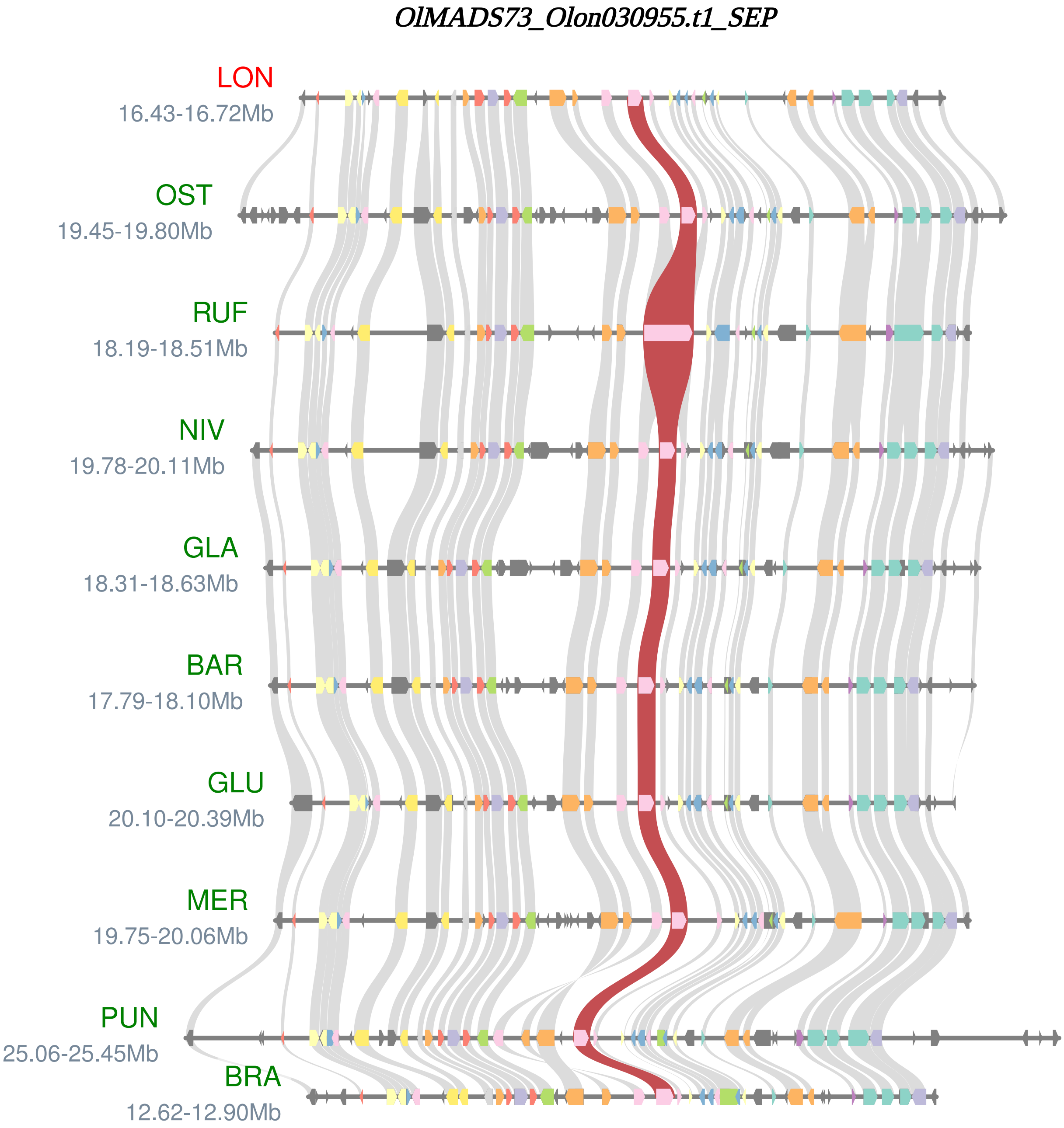

OlMADS73_Olon030955.t1_SEP
LON
16.43-16.72Mb
OST
19.45-19.80Mb
RUF
18.19-18.51Mb
NIV
19.78-20.11Mb
GLA
18.31-18.63Mb
BAR
17.79-18.10Mb
GLU
20.10-20.39Mb
MER
19.75-20.06Mb
PUN
25.06-25.45Mb
BRA
12.62-12.90Mb

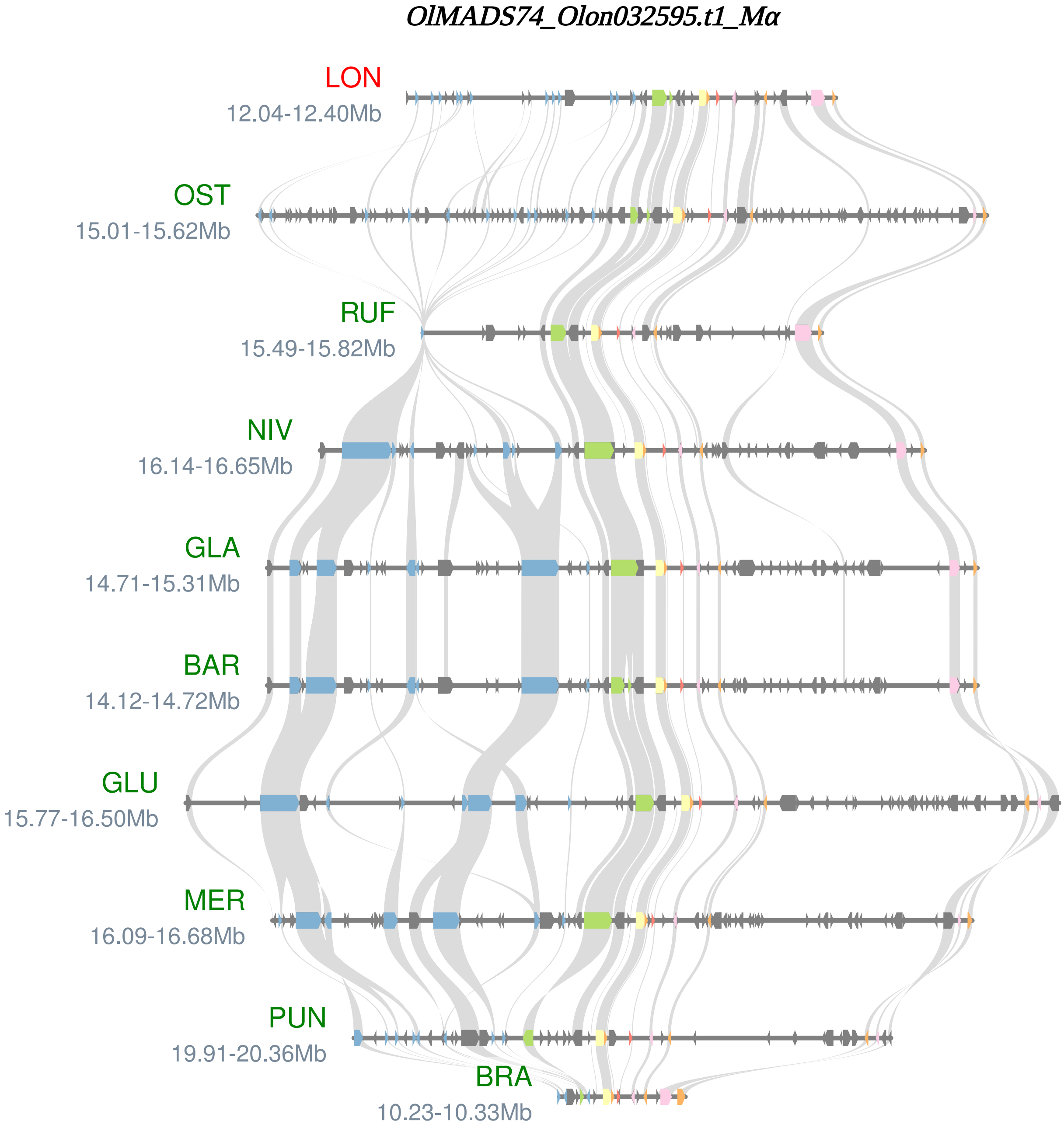

OlMADS74_Olon032595.t1_Mα
LON
12.04-12.40Mb
OST
15.01-15.62Mb
RUF
15.49-15.82Mb
NIV
16.14-16.65Mb
GLA
14.71-15.31Mb
BAR
14.12-14.72Mb
GLU
15.77-16.50Mb
MER
16.09-16.68Mb
PUN
19.91-20.36Mb
BRA
10.23-10.33Mb

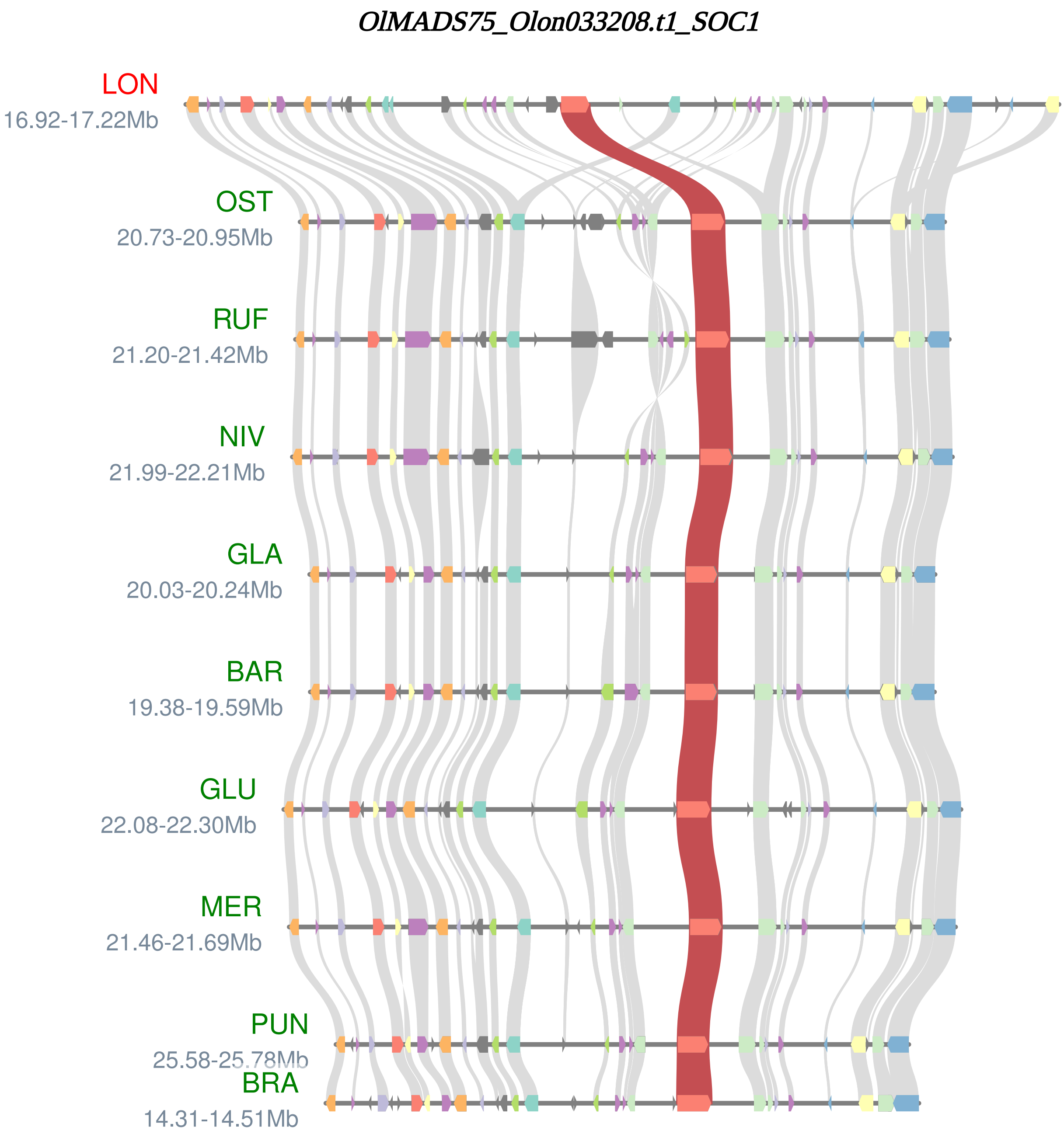
OlMADS75_Olon033208.t1_SOC1
LON
16.92-17.22Mb
OST
20.73-20.95Mb
RUF
21.20-21.42Mb
NIV
21.99-22.21Mb
GLA
20.03-20.24Mb
BAR
19.38-19.59Mb
GLU
22.08-22.30Mb
MER
21.46-21.69Mb
PUN
25.58-25.78Mb
BRA
14.31-14.51Mb

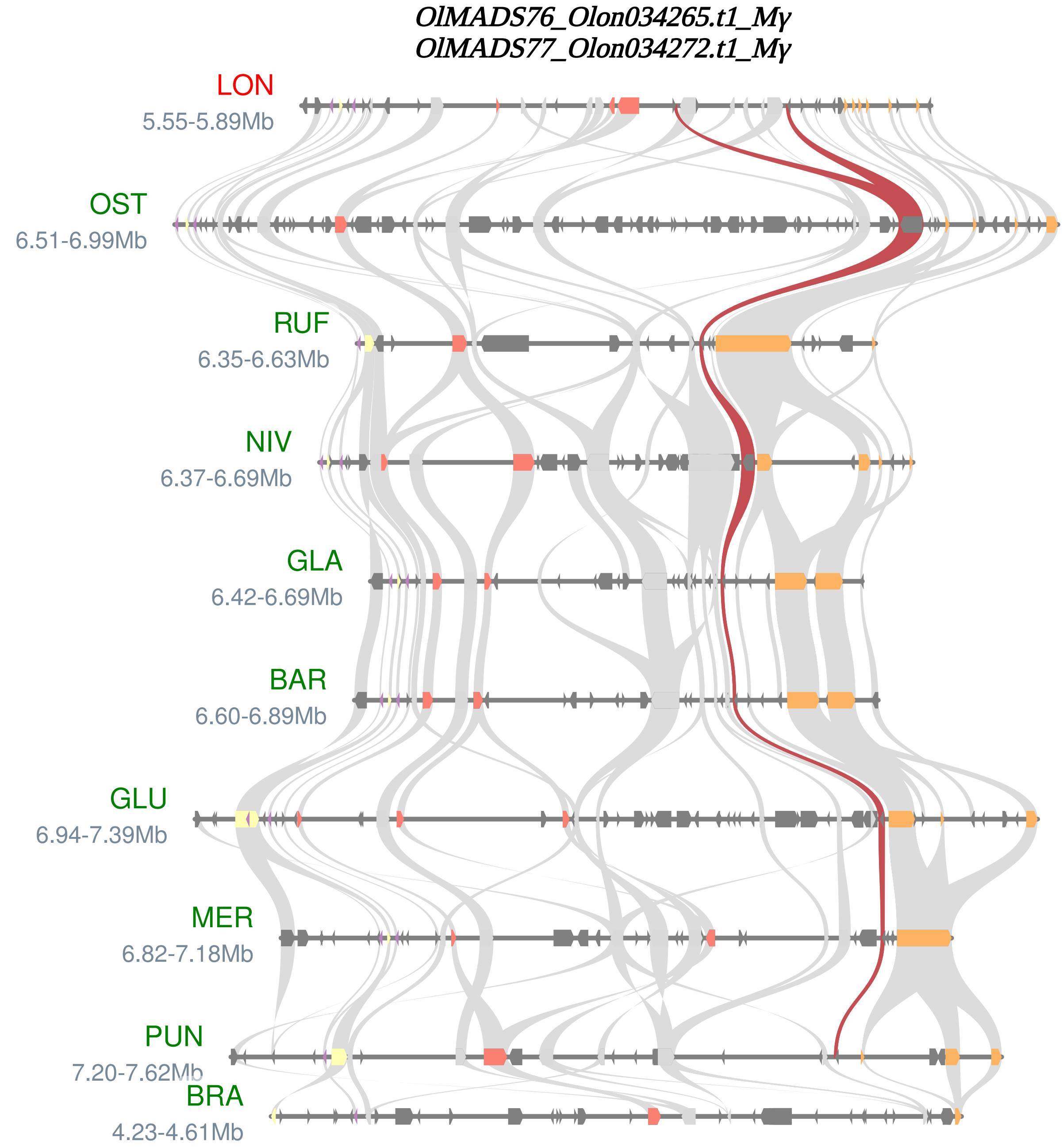
OlMADS76_Olon034265.t1_Mγ
OlMADS77_Olon034272.t1_Mγ
LON
5.55-5.89Mb
OST
6.51-6.99Mb
RUF
6.35-6.63Mb
NIV
6.37-6.69Mb
GLA
6.42-6.69Mb
BAR
6.60-6.89Mb
GLU
6.94-7.39Mb
MER
6.82-7.18Mb
PUN
7.20-7.62Mb
BRA
4.23-4.61Mb

*OlMADS78_Olon035748.t1_MIKC**

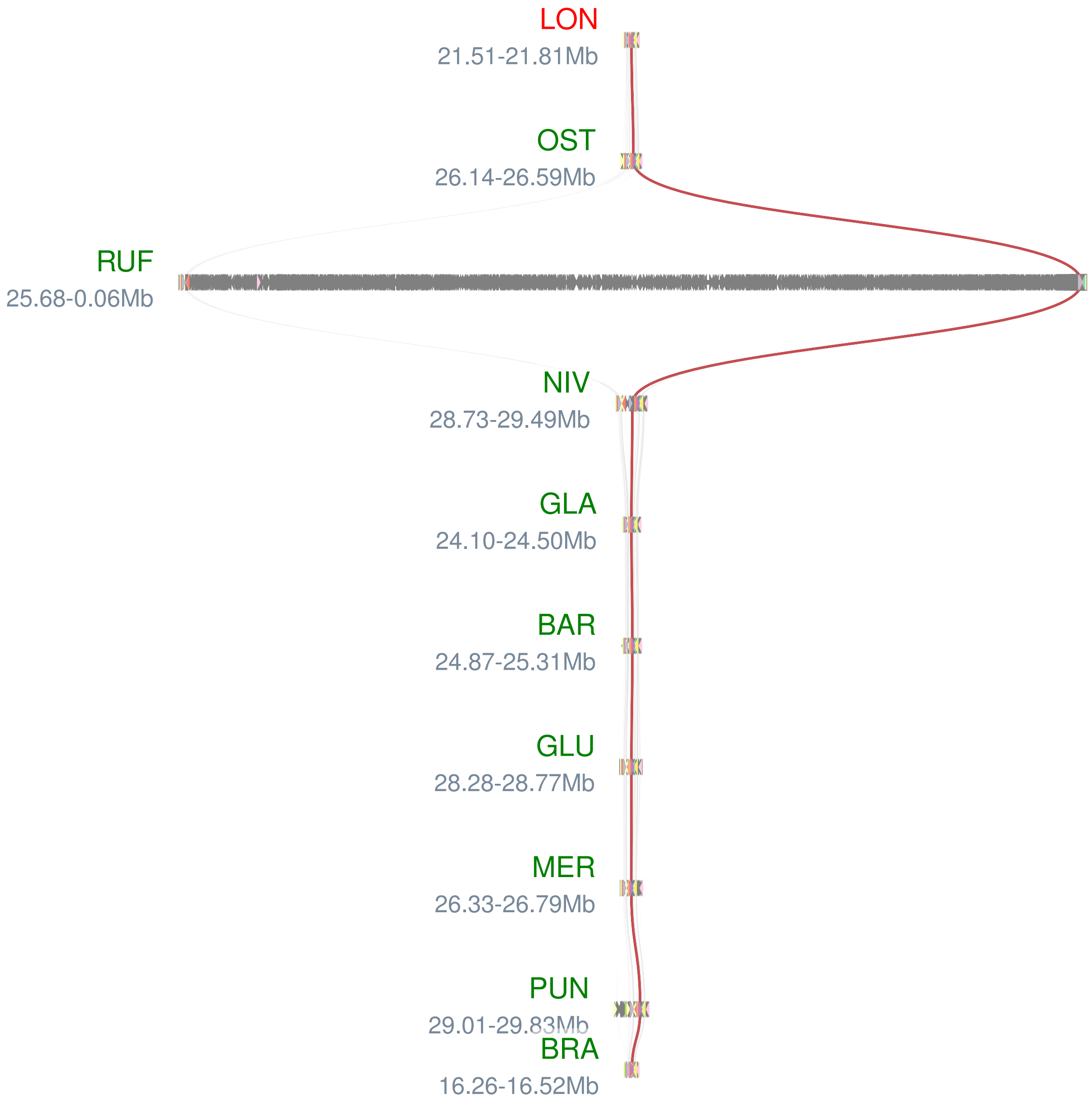

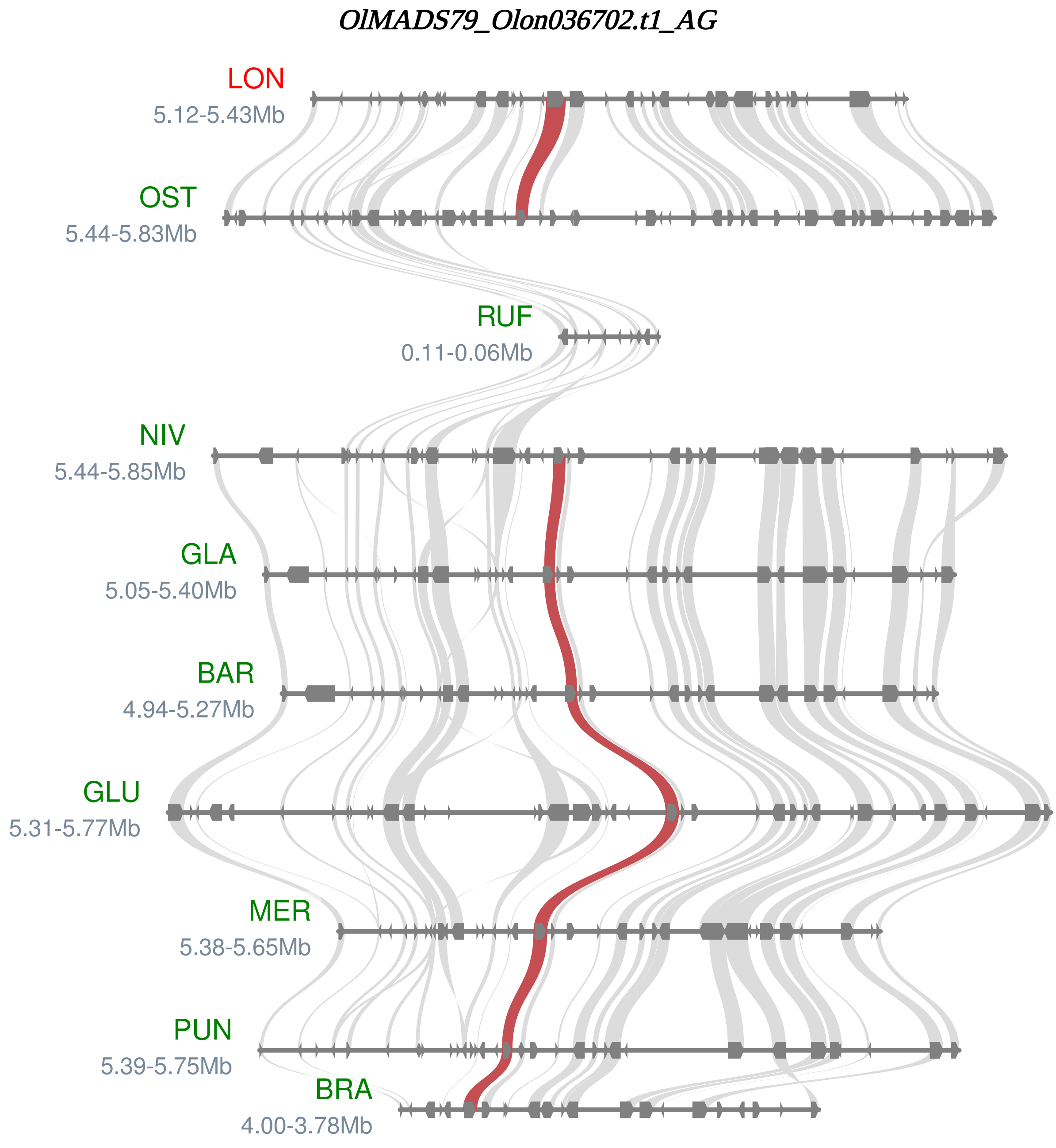
OlMADS79_Olon036702.t1_AG
LON
5.12-5.43Mb
OST
5.44-5.83Mb
RUF
0.11-0.06Mb
NIV
5.44-5.85Mb
GLA
5.05-5.40Mb
BAR
4.94-5.27Mb
GLU
5.31-5.77Mb
MER
5.38-5.65Mb
PUN
5.39-5.75Mb
BRA
4.00-3.78Mb

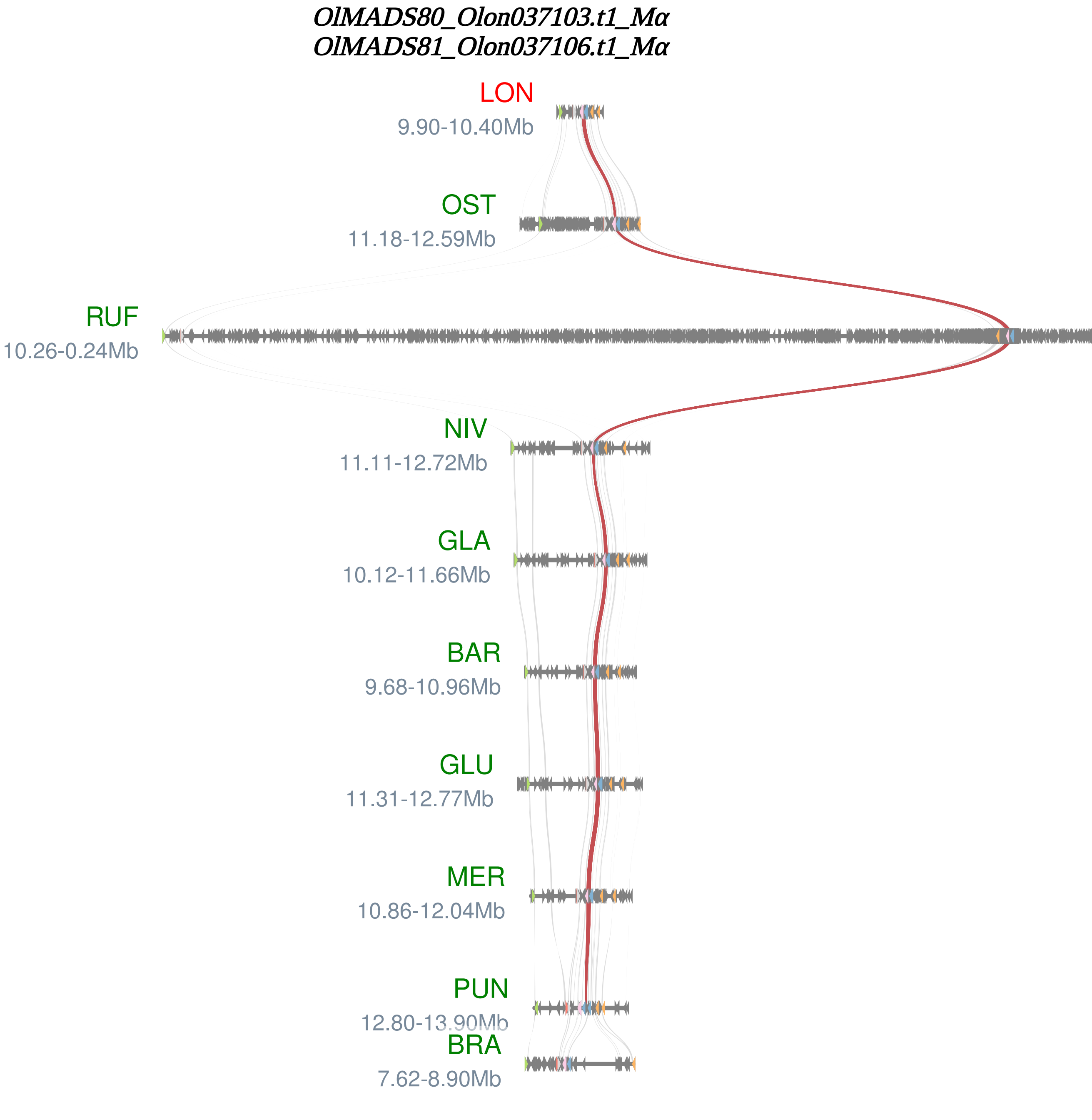

OlMADS80_Olon037103.t1_Mα
OlMADS81_Olon037106.t1_Mα
LON
9.90-10.40Mb
OST
11.18-12.59Mb
RUF
10.26-0.24Mb
NIV
11.11-12.72Mb
GLA
10.12-11.66Mb
BAR
9.68-10.96Mb
GLU
11.31-12.77Mb
MER
10.86-12.04Mb
PUN
12.80-13.90Mb
BRA
7.62-8.90Mb

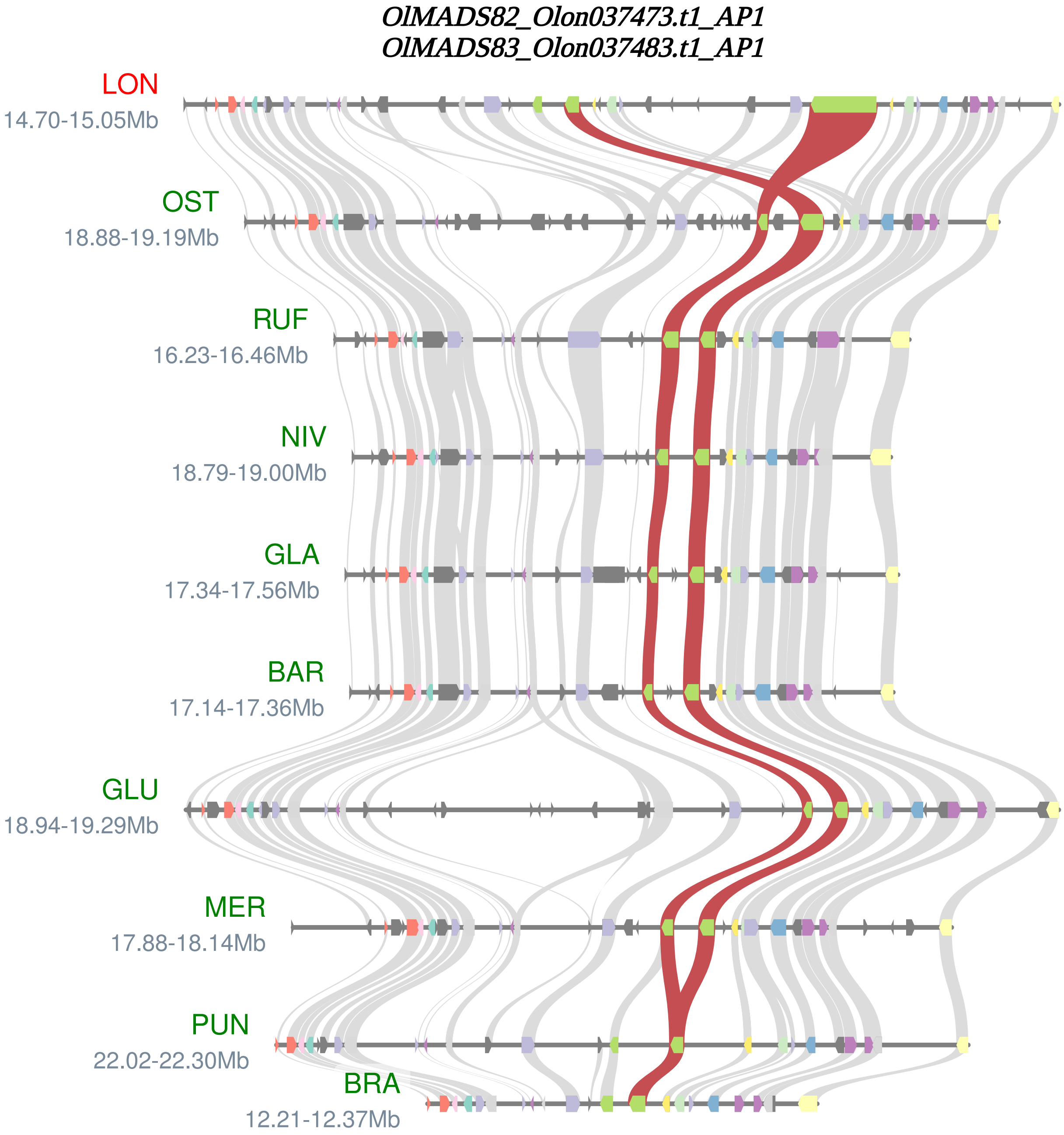

OlMADS82_Olon037473.t1_AP1
OlMADS83_Olon037483.t1_AP1
LON
14.70-15.05Mb
OST
18.88-19.19Mb
RUF
16.23-16.46Mb
NIV
18.79-19.00Mb
GLA
17.34-17.56Mb
BAR
17.14-17.36Mb
GLU
18.94-19.29Mb
MER
17.88-18.14Mb
PUN
22.02-22.30Mb
BRA
12.21-12.37Mb

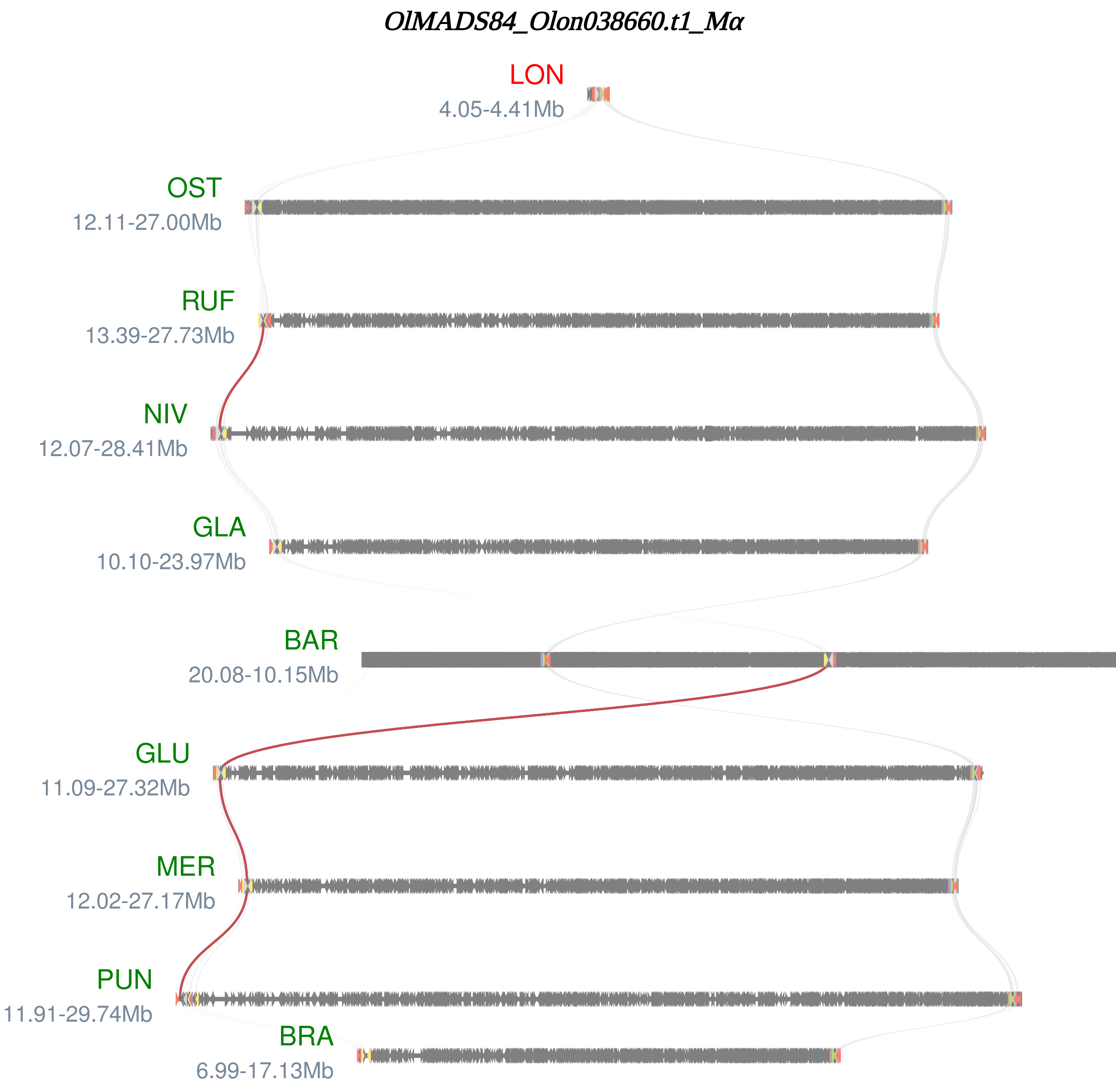

OlMADS84_Olon038660.t1_Mα
LON
4.05-4.41Mb
OST
12.11-27.00Mb
RUF
13.39-27.73Mb
NIV
12.07-28.41Mb
GLA
10.10-23.97Mb
BAR
20.08-10.15Mb
GLU
11.09-27.32Mb
MER
12.02-27.17Mb
PUN
11.91-29.74Mb
BRA
6.99-17.13Mb

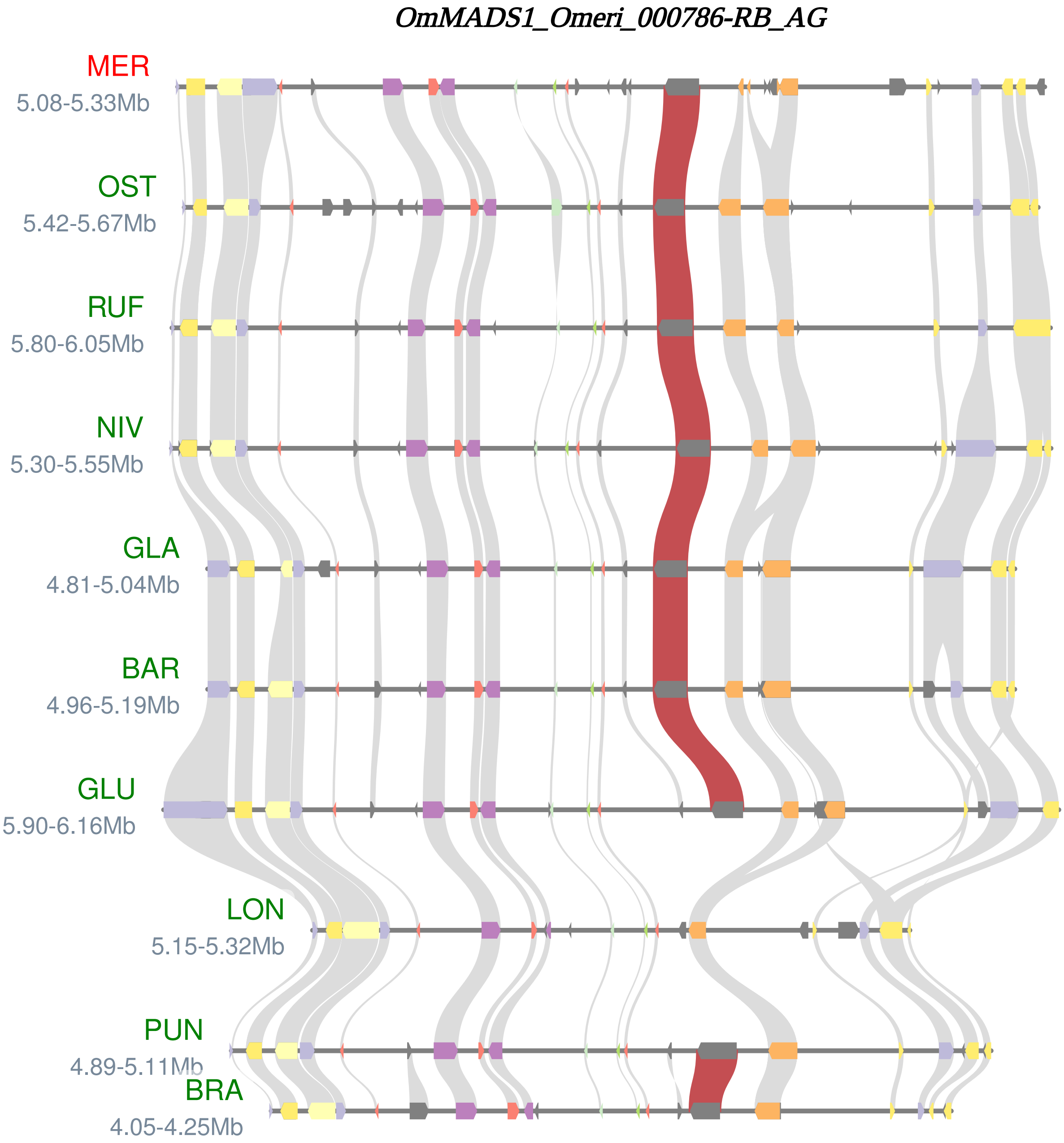
OmMADS1_Omeri_000786-RB_AG
MER
5.08-5.33Mb
OST
5.42-5.67Mb
RUF
5.80-6.05Mb
NIV
5.30-5.55Mb
GLA
4.81-5.04Mb
BAR
4.96-5.19Mb
GLU
5.90-6.16Mb
LON
5.15-5.32Mb
PUN
4.89-5.11Mb
BRA
4.05-4.25Mb

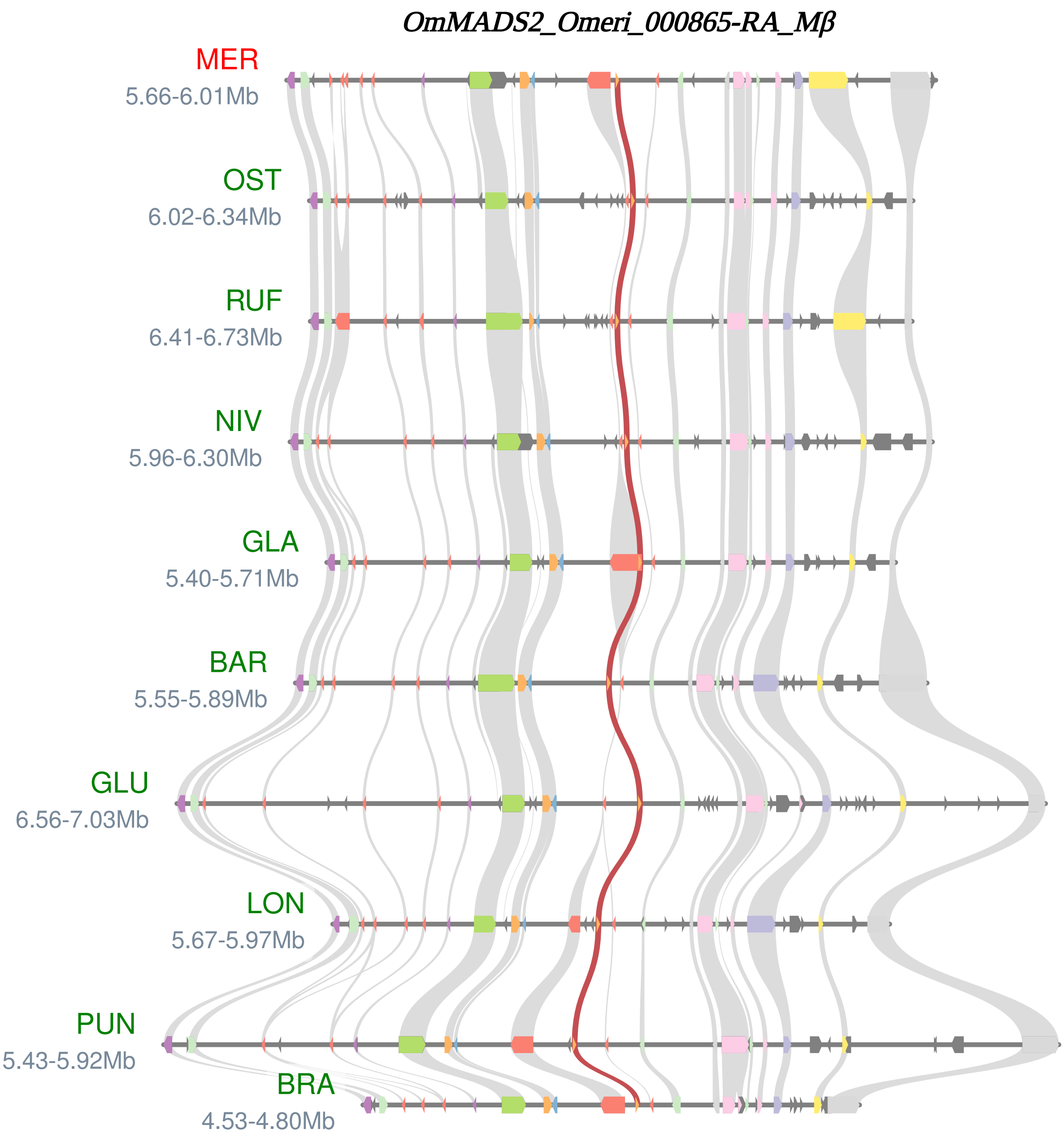
OmMADS2_Omeri_000865-RA_Mβ
MER
5.66-6.01Mb
OST
6.02-6.34Mb
RUF
6.41-6.73Mb
NIV
5.96-6.30Mb
GLA
5.40-5.71Mb
BAR
5.55-5.89Mb
GLU
6.56-7.03Mb
LON
5.67-5.97Mb
PUN
5.43-5.92Mb
BRA
4.53-4.80Mb

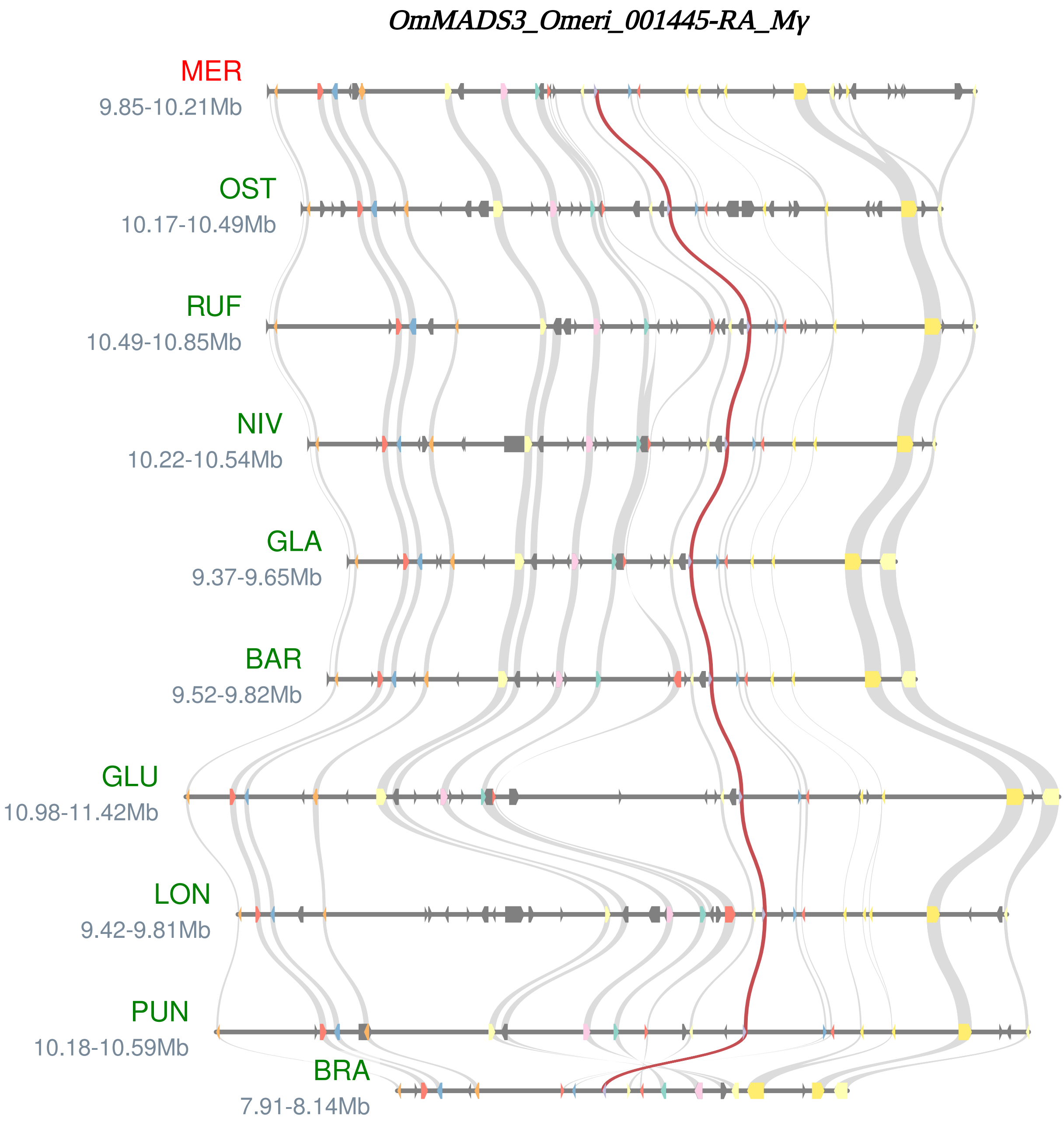
OmMADS3_Omeri_001445-RA_Mγ
MER
9.85-10.21Mb
OST
10.17-10.49Mb
RUF
10.49-10.85Mb
NIV
10.22-10.54Mb
GLA
9.37-9.65Mb
BAR
9.52-9.82Mb
GLU
10.98-11.42Mb
LON
9.42-9.81Mb
PUN
10.18-10.59Mb
BRA
7.91-8.14Mb

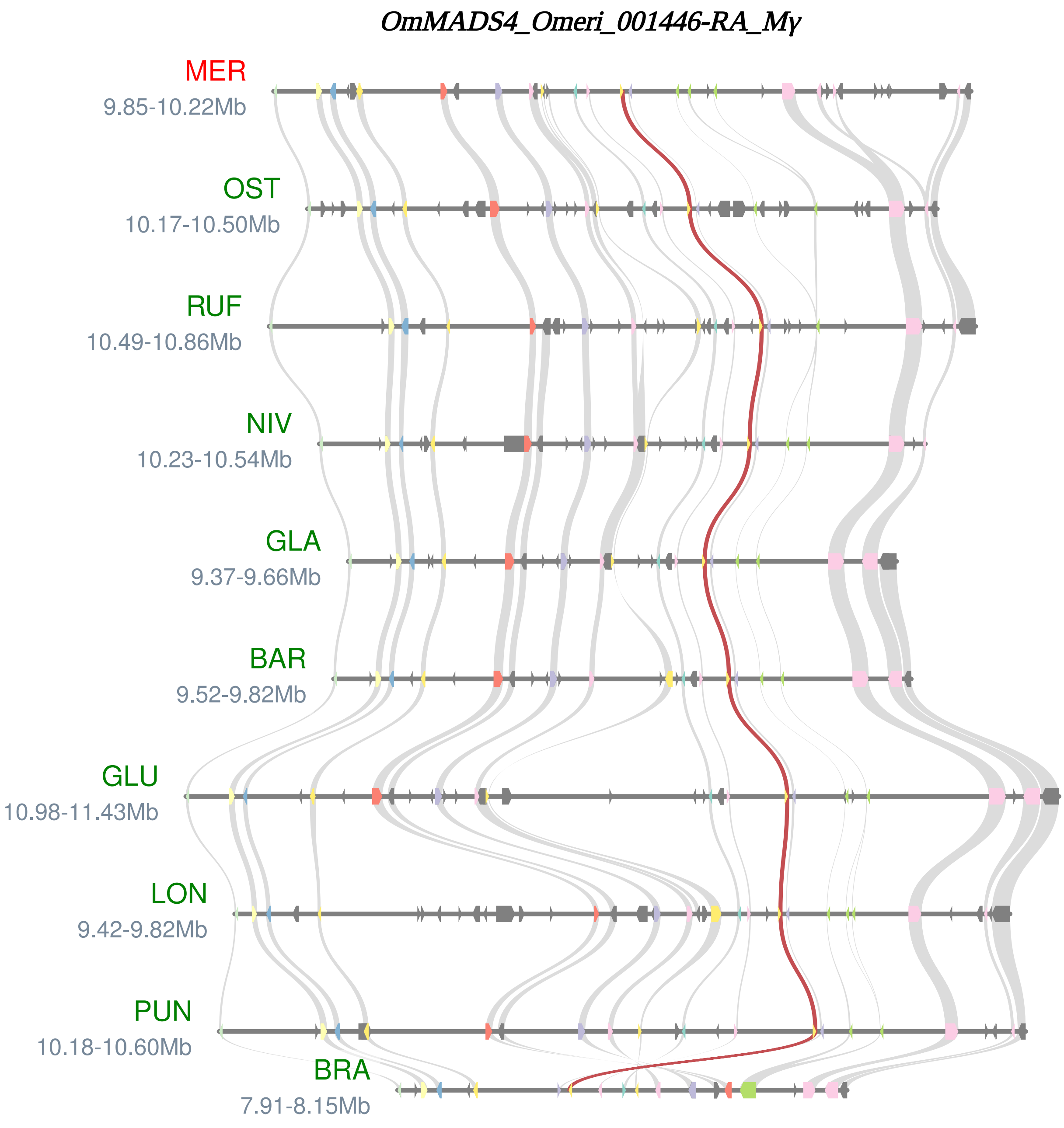

OmMADS4_Omeri_001446-RA_Mγ
MER
9.85-10.22Mb
OST
10.17-10.50Mb
RUF
10.49-10.86Mb
NIV
10.23-10.54Mb
GLA
9.37-9.66Mb
BAR
9.52-9.82Mb
GLU
10.98-11.43Mb
LON
9.42-9.82Mb
PUN
10.18-10.60Mb
BRA
7.91-8.15Mb

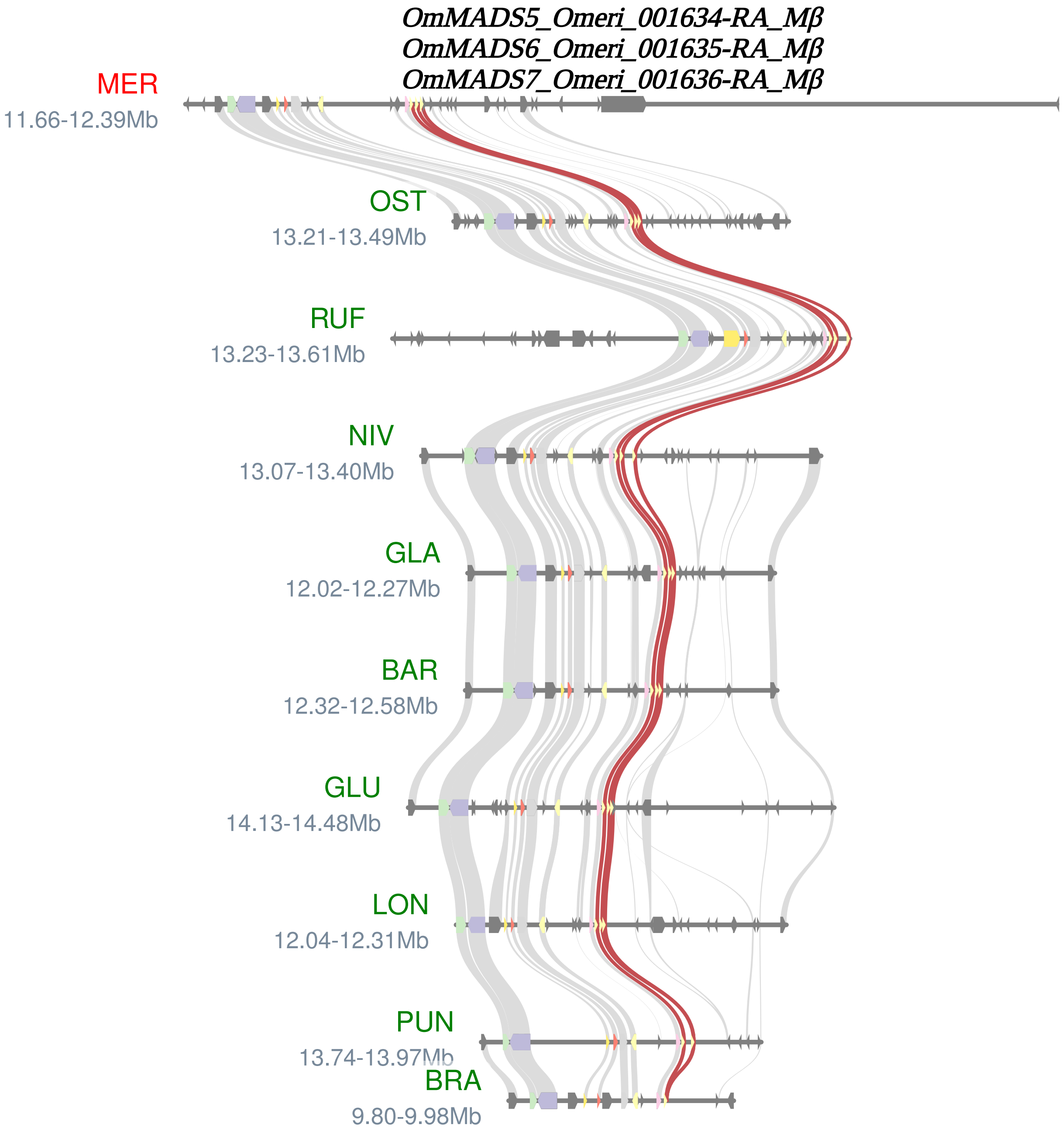
OmMADS5_Omeri_001634-RA_Mβ
OmMADS6_Omeri_001635-RA_Mβ
OmMADS7_Omeri_001636-RA_Mβ
MER
11.66-12.39Mb
OST
13.21-13.49Mb
RUF
13.23-13.61Mb
NIV
13.07-13.40Mb
GLA
12.02-12.27Mb
BAR
12.32-12.58Mb
GLU
14.13-14.48Mb
LON
12.04-12.31Mb
PUN
13.74-13.97Mb
BRA
9.80-9.98Mb

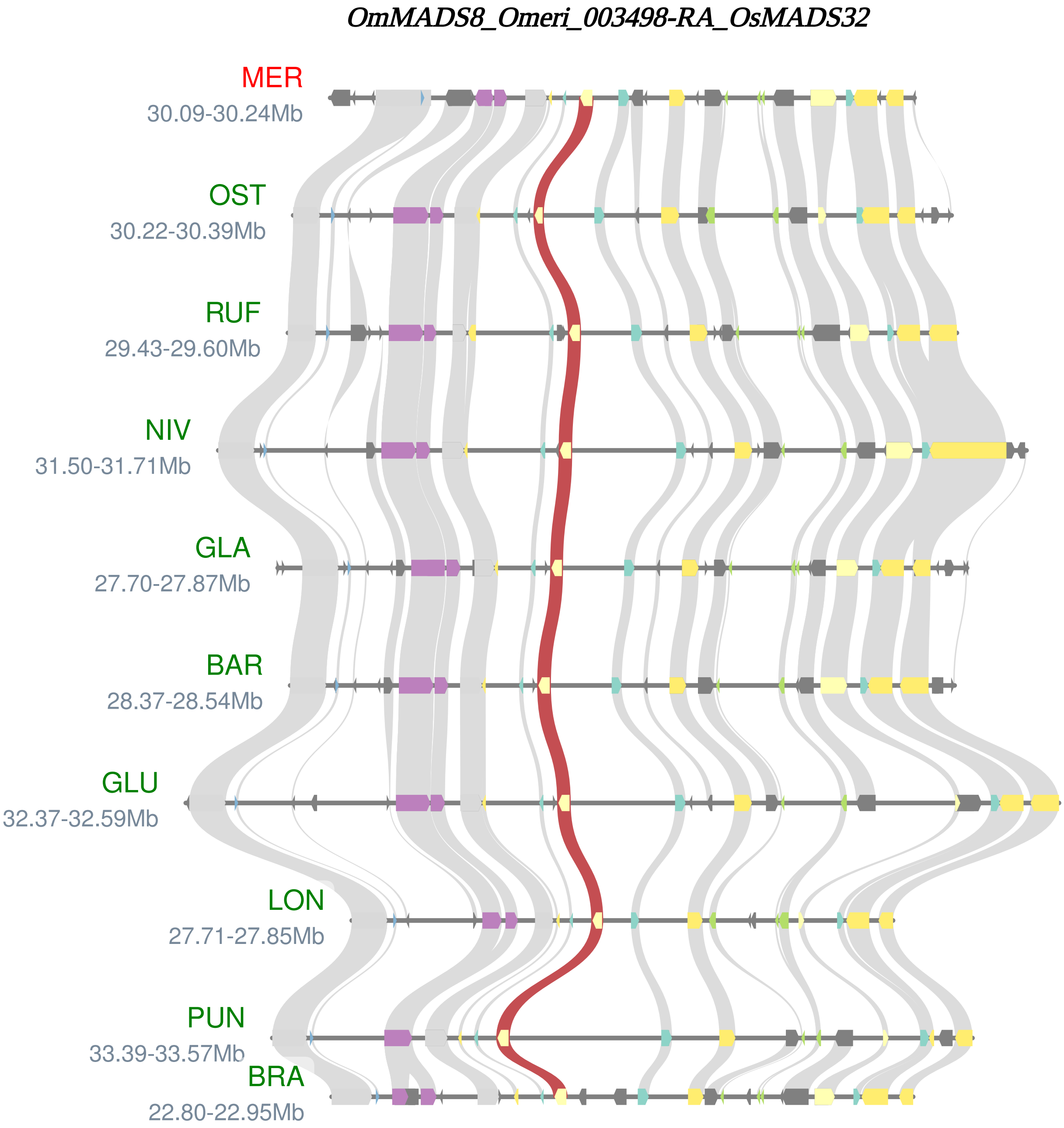

OmMADS8_Omeri_003498-RA_OsMADS32
MER
30.09-30.24Mb
OST
30.22-30.39Mb
RUF
29.43-29.60Mb
NIV
31.50-31.71Mb
GLA
27.70-27.87Mb
BAR
28.37-28.54Mb
GLU
32.37-32.59Mb
LON
27.71-27.85Mb
PUN
33.39-33.57Mb
BRA
22.80-22.95Mb

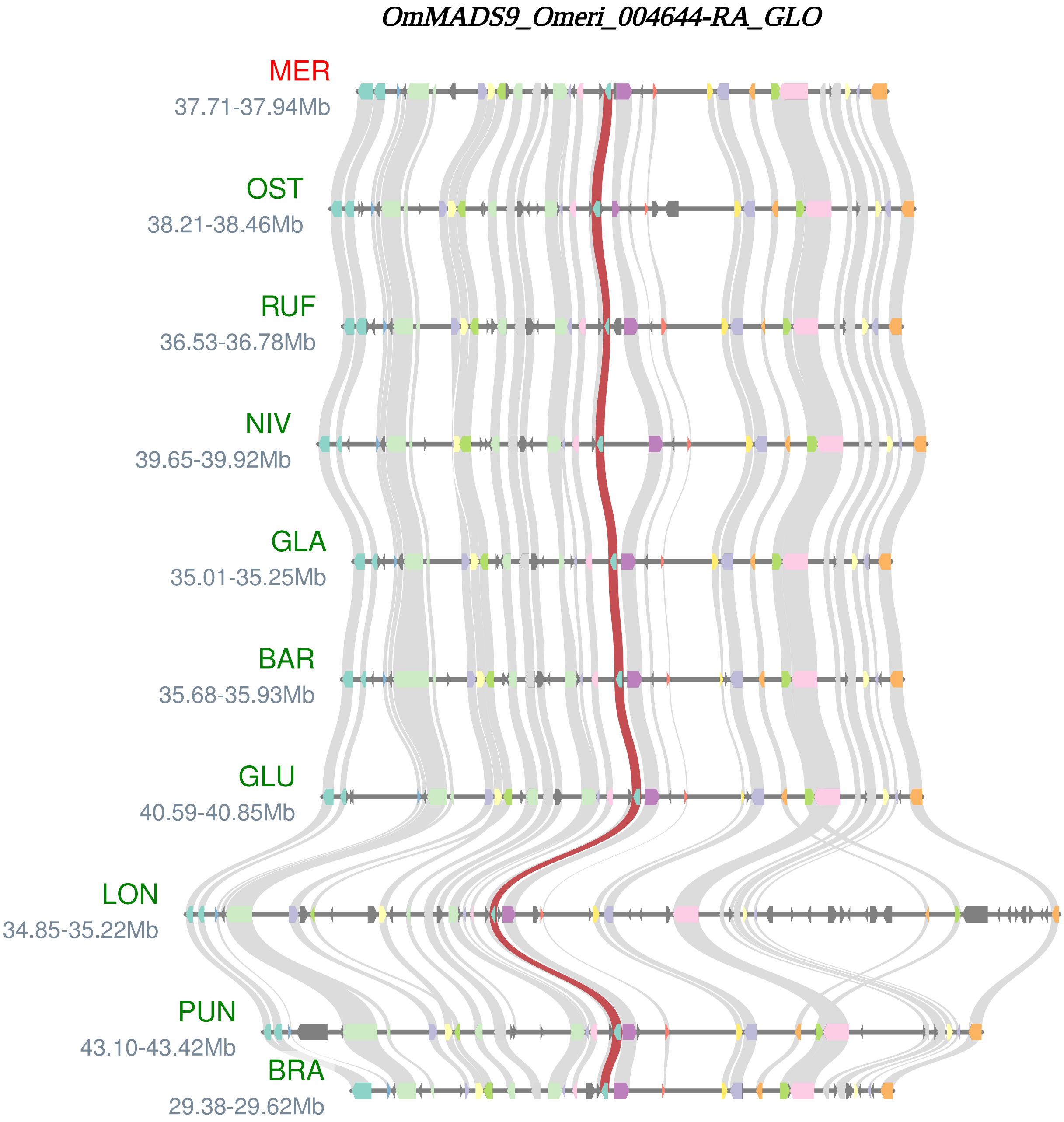

OmMADS9_Omeri_004644-RA_GLO
MER
37.71-37.94Mb
OST
38.21-38.46Mb
RUF
36.53-36.78Mb
NIV
39.65-39.92Mb
GLA
35.01-35.25Mb
BAR
35.68-35.93Mb
GLU
40.59-40.85Mb
LON
34.85-35.22Mb
PUN
43.10-43.42Mb
BRA
29.38-29.62Mb

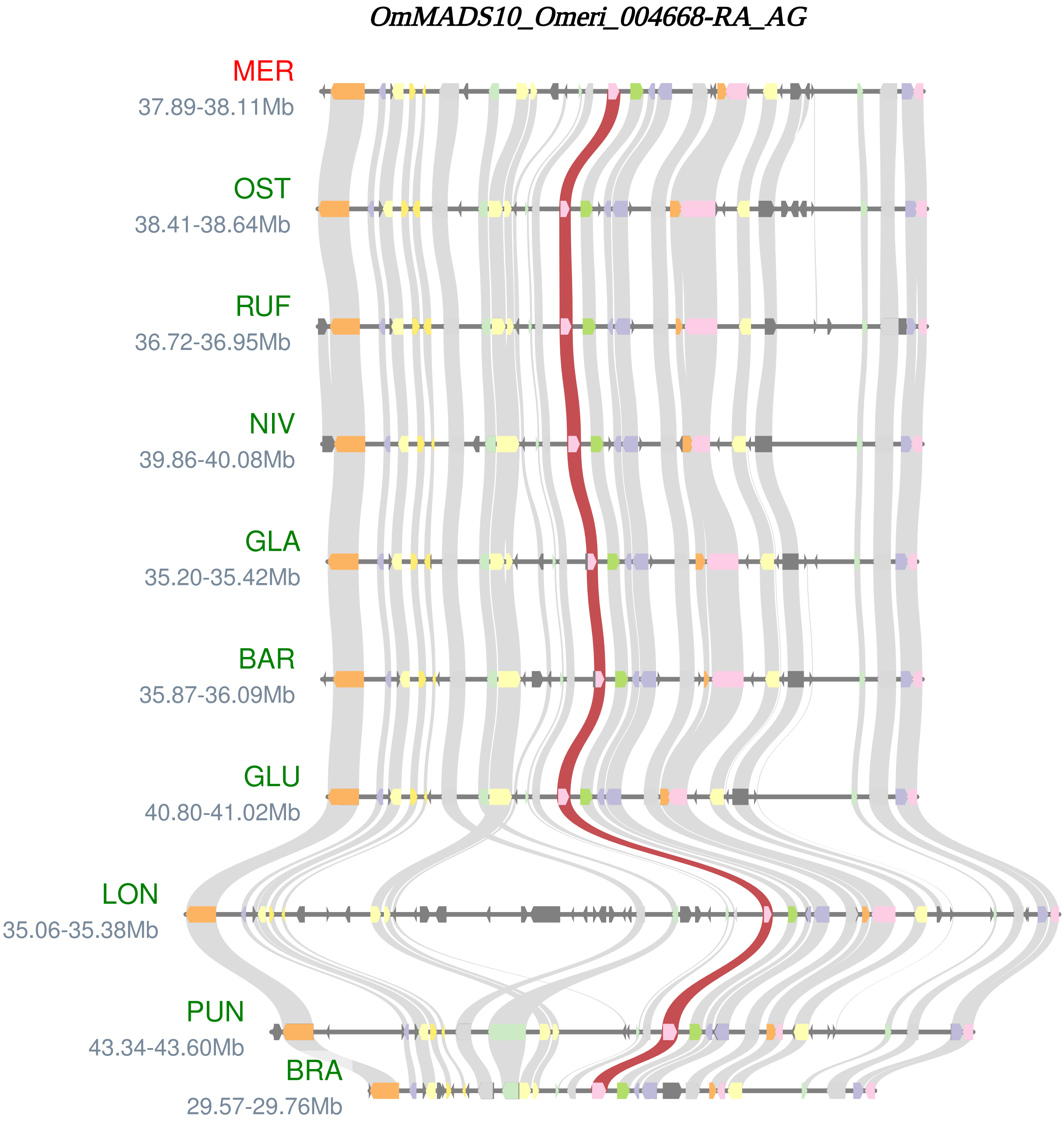
OmMADS10_Omeri_004668-RA_AG
MER
37.89-38.11Mb
OST
38.41-38.64Mb
RUF
36.72-36.95Mb
NIV
39.86-40.08Mb
GLA
35.20-35.42Mb
BAR
35.87-36.09Mb
GLU
40.80-41.02Mb
LON
35.06-35.38Mb
PUN
43.34-43.60Mb
BRA
29.57-29.76Mb

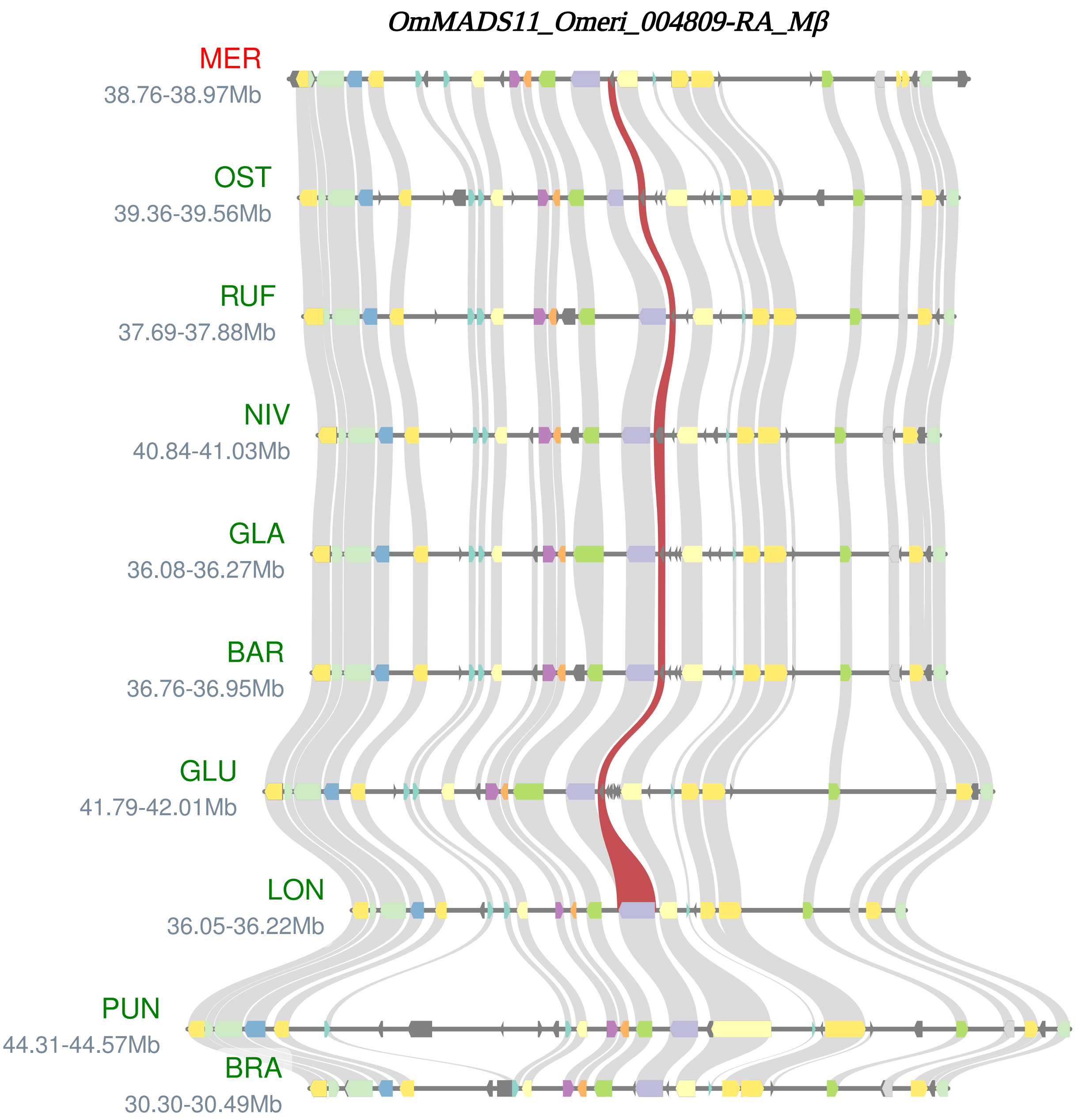
OmMADS11_Omeri_004809-RA_Mβ
MER
38.76-38.97Mb
OST
39.36-39.56Mb
RUF
37.69-37.88Mb
NIV
40.84-41.03Mb
GLA
36.08-36.27Mb
BAR
36.76-36.95Mb
GLU
41.79-42.01Mb
LON
36.05-36.22Mb
PUN
44.31-44.57Mb
BRA
30.30-30.49Mb

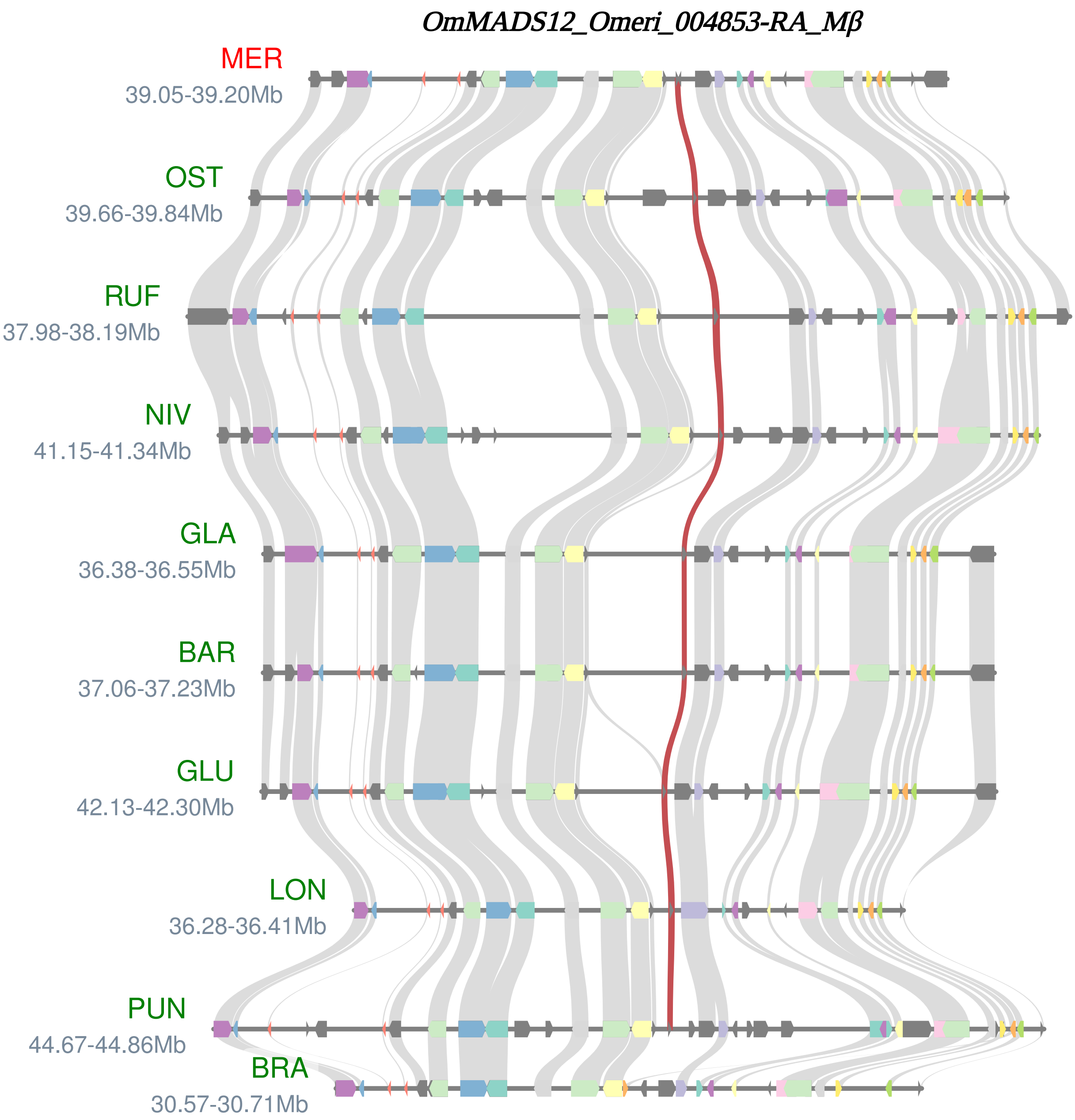
OmMADS12_Omeri_004853-RA_Mβ
MER
39.05-39.20Mb
OST
39.66-39.84Mb
RUF
37.98-38.19Mb
NIV
41.15-41.34Mb
GLA
36.38-36.55Mb
BAR
37.06-37.23Mb
GLU
42.13-42.30Mb
LON
36.28-36.41Mb
PUN
44.67-44.86Mb
BRA
30.57-30.71Mb

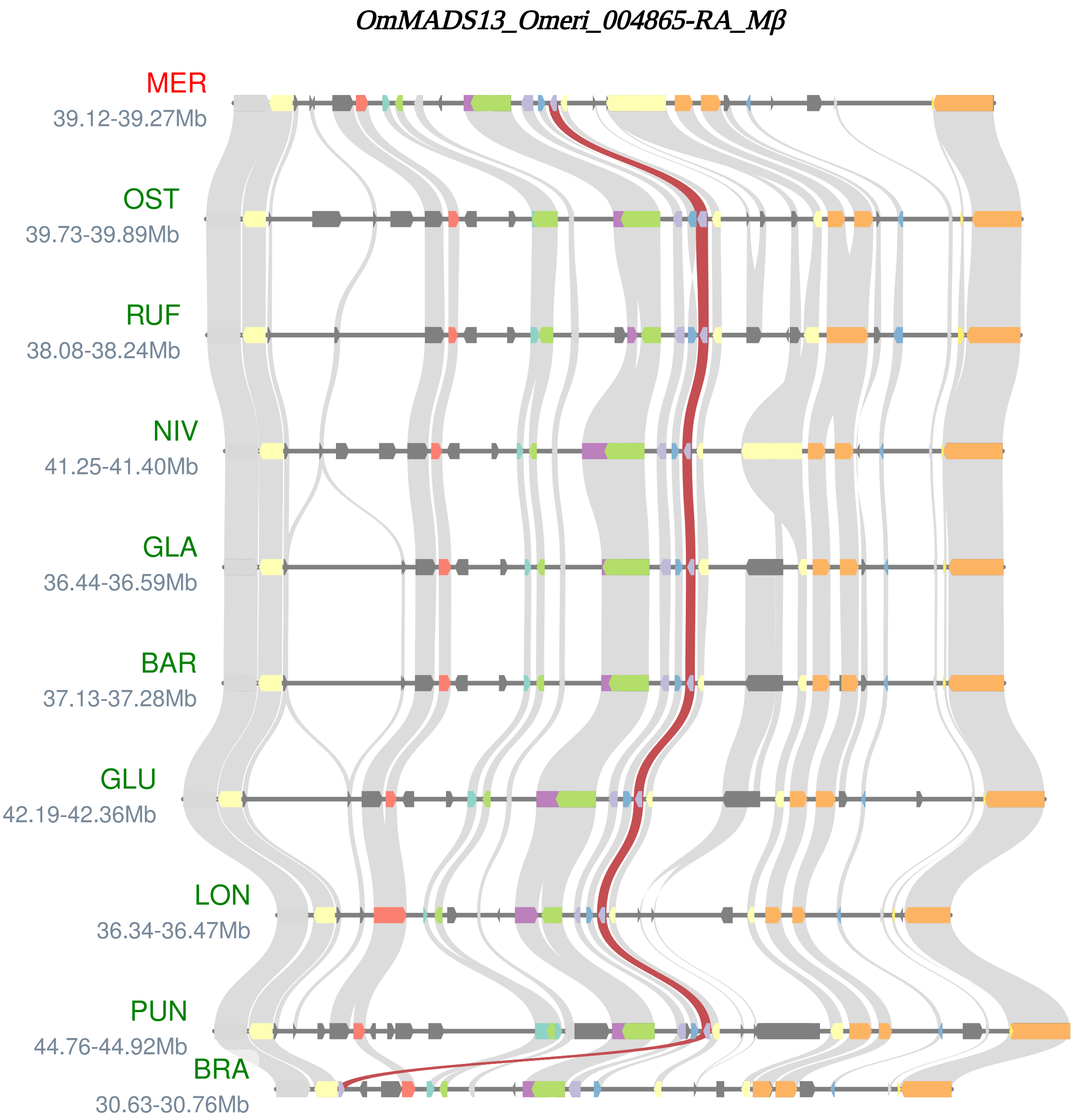

OmMADS13_Omeri_004865-RA_Mβ
MER
39.12-39.27Mb
OST
39.73-39.89Mb
RUF
38.08-38.24Mb
NIV
41.25-41.40Mb
GLA
36.44-36.59Mb
BAR
37.13-37.28Mb
GLU
42.19-42.36Mb
LON
36.34-36.47Mb
PUN
44.76-44.92Mb
BRA
30.63-30.76Mb

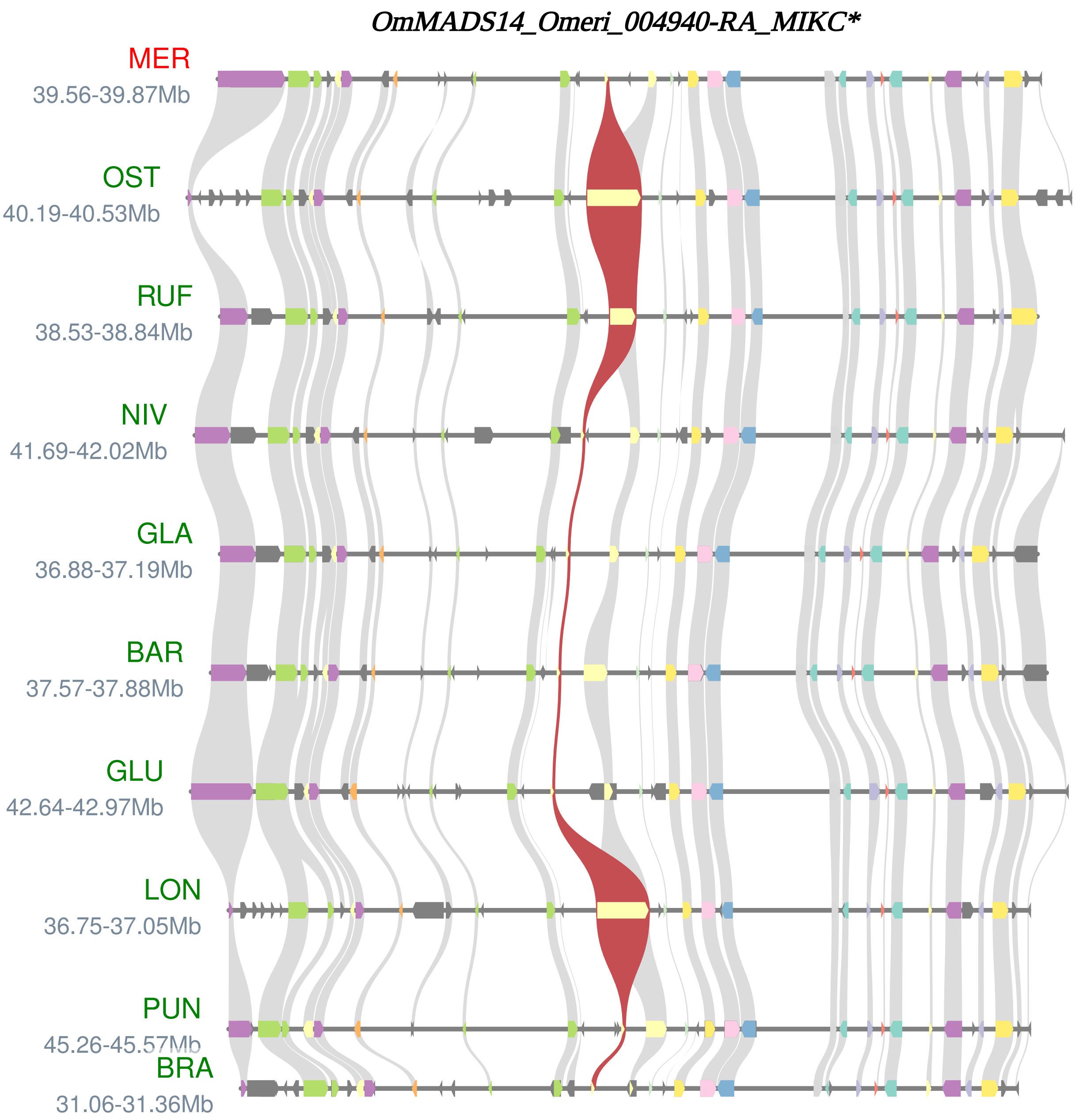
OmMADS14_Omeri_004940-RA_MIKC*
MER
39.56-39.87Mb
OST
40.19-40.53Mb
RUF
38.53-38.84Mb
NIV
41.69-42.02Mb
GLA
36.88-37.19Mb
BAR
37.57-37.88Mb
GLU
42.64-42.97Mb
LON
36.75-37.05Mb
PUN
45.26-45.57Mb
BRA
31.06-31.36Mb

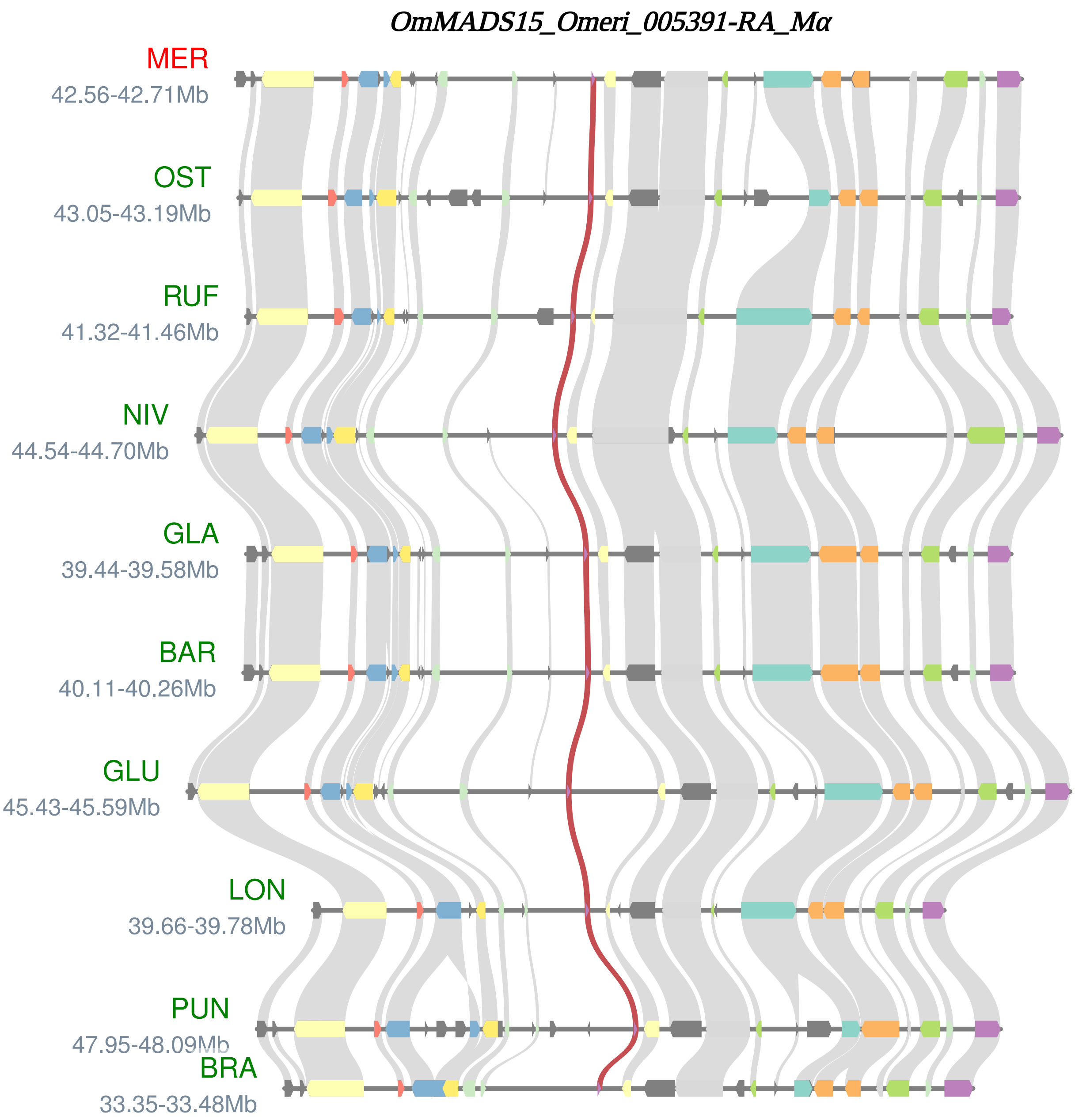

OmMADS15_Omeri_005391-RA_Mα
MER
42.56-42.71Mb
OST
43.05-43.19Mb
RUF
41.32-41.46Mb
NIV
44.54-44.70Mb
GLA
39.44-39.58Mb
BAR
40.11-40.26Mb
GLU
45.43-45.59Mb
LON
39.66-39.78Mb
PUN
47.95-48.09Mb
BRA
33.35-33.48Mb

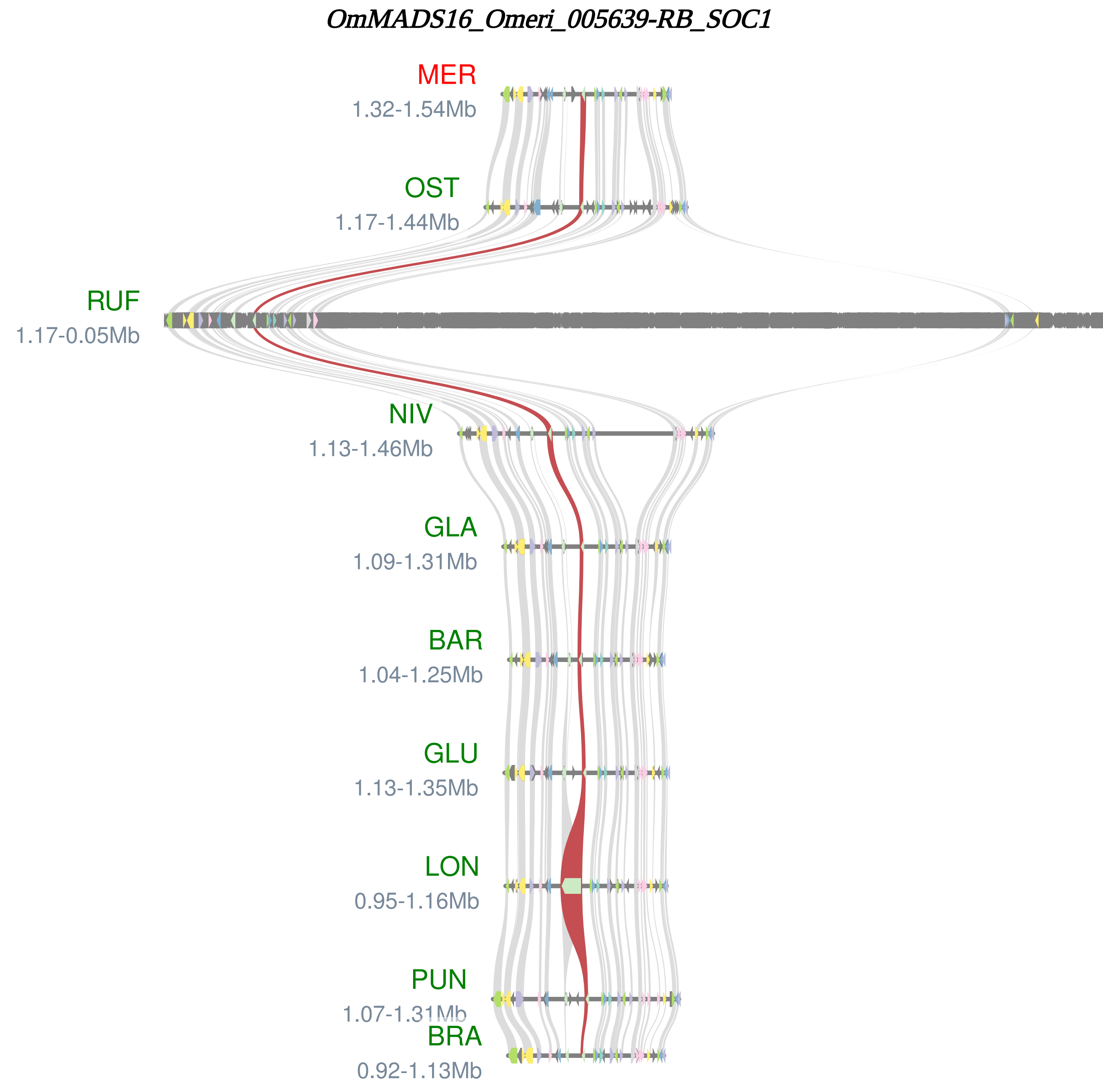
OmMADS16_Omeri_005639-RB_SOC1
MER
1.32-1.54Mb
OST
1.17-1.44Mb
RUF
1.17-0.05Mb
NIV
1.13-1.46Mb
GLA
1.09-1.31Mb
BAR
1.04-1.25Mb
GLU
1.13-1.35Mb
LON
0.95-1.16Mb
PUN
1.07-1.31Mb
BRA
0.92-1.13Mb

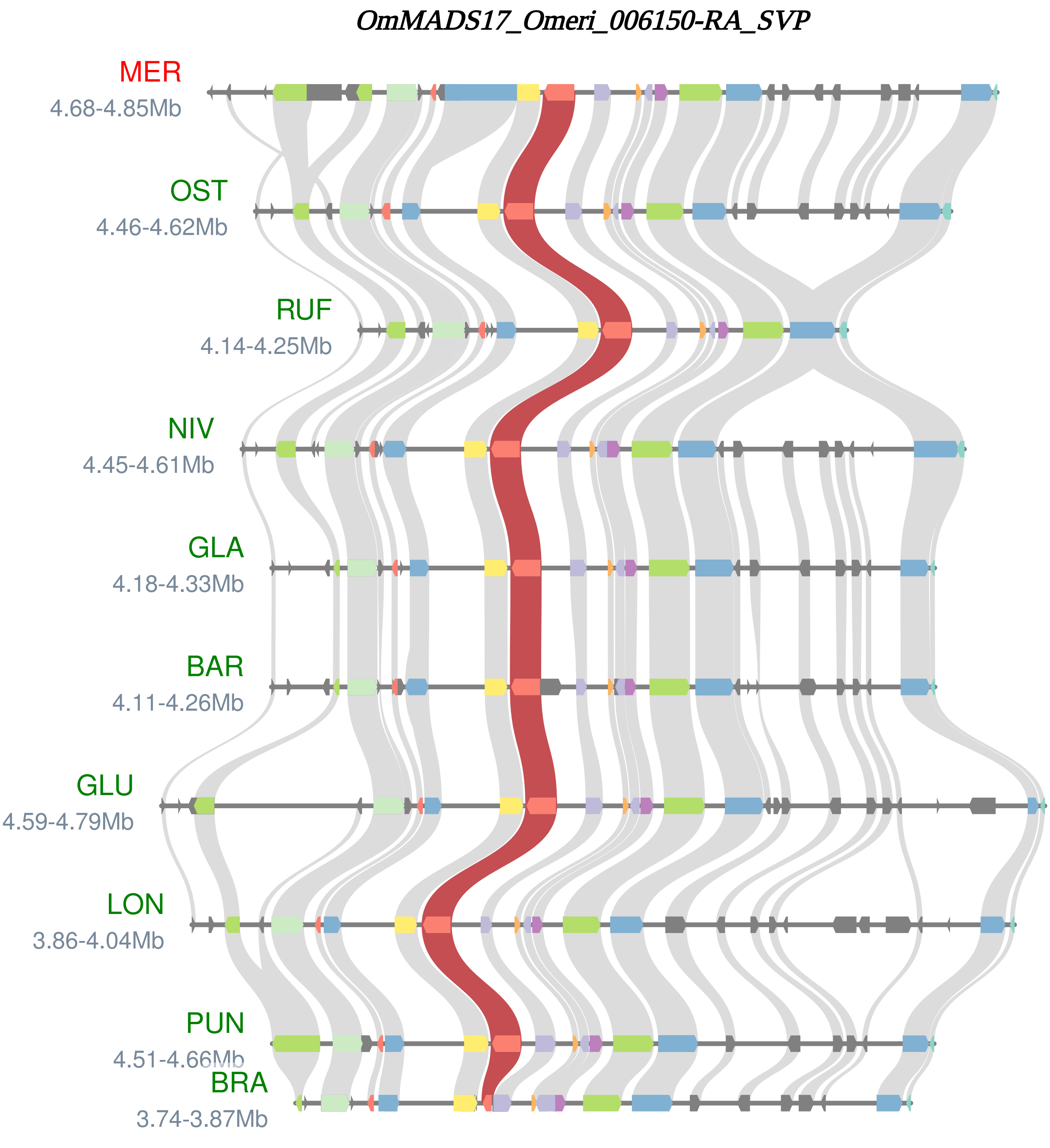
OmMADS17_Omeri_006150-RA_SVP
MER
4.68-4.85Mb
OST
4.46-4.62Mb
RUF
4.14-4.25Mb
NIV
4.45-4.61Mb
GLA
4.18-4.33Mb
BAR
4.11-4.26Mb
GLU
4.59-4.79Mb
LON
3.86-4.04Mb
PUN
4.51-4.66Mb
BRA
3.74-3.87Mb

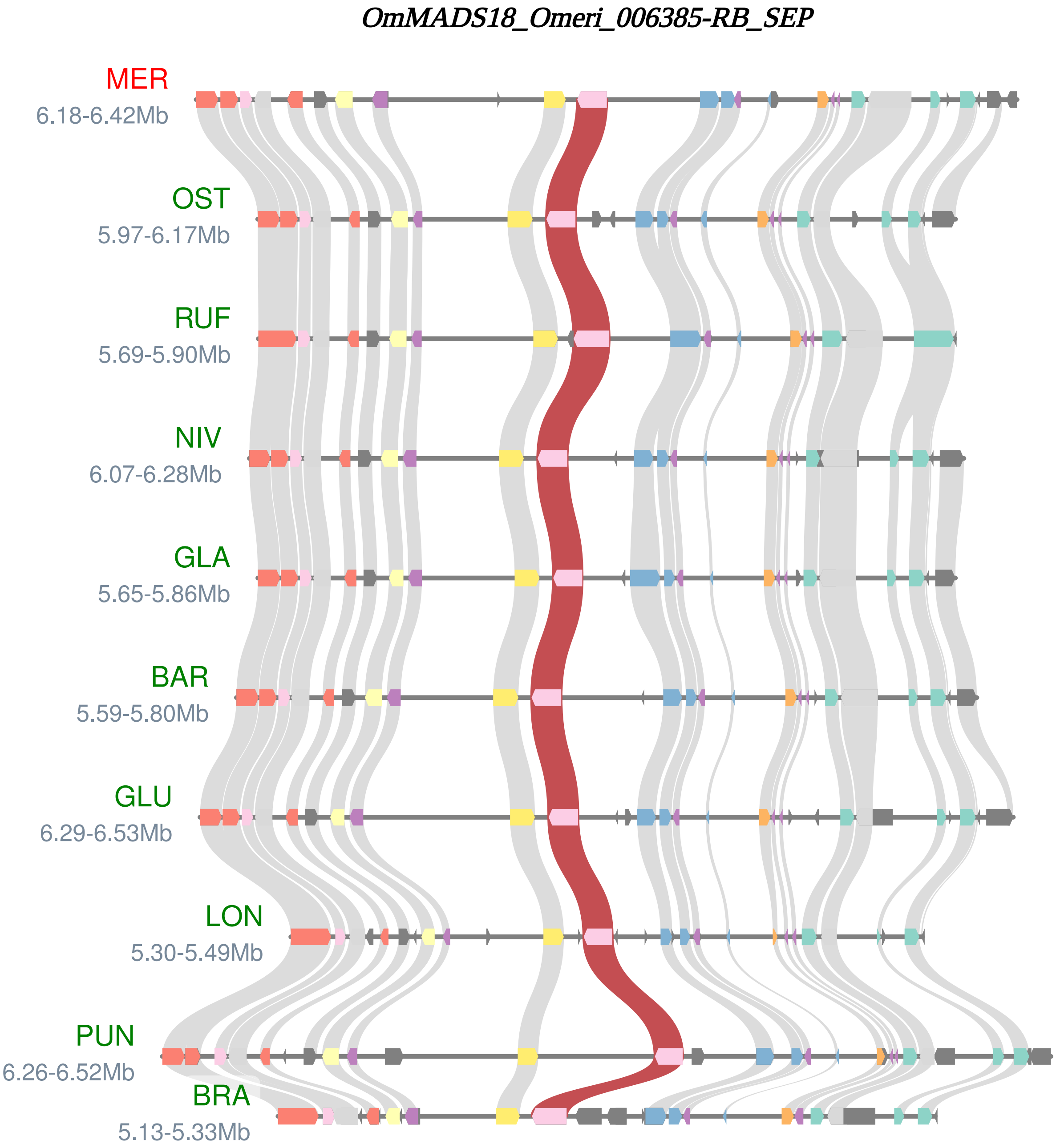
OmMADS18_Omeri_006385-RB_SEP
MER
6.18-6.42Mb
OST
5.97-6.17Mb
RUF
5.69-5.90Mb
NIV
6.07-6.28Mb
GLA
5.65-5.86Mb
BAR
5.59-5.80Mb
GLU
6.29-6.53Mb
LON
5.30-5.49Mb
PUN
6.26-6.52Mb
BRA
5.13-5.33Mb

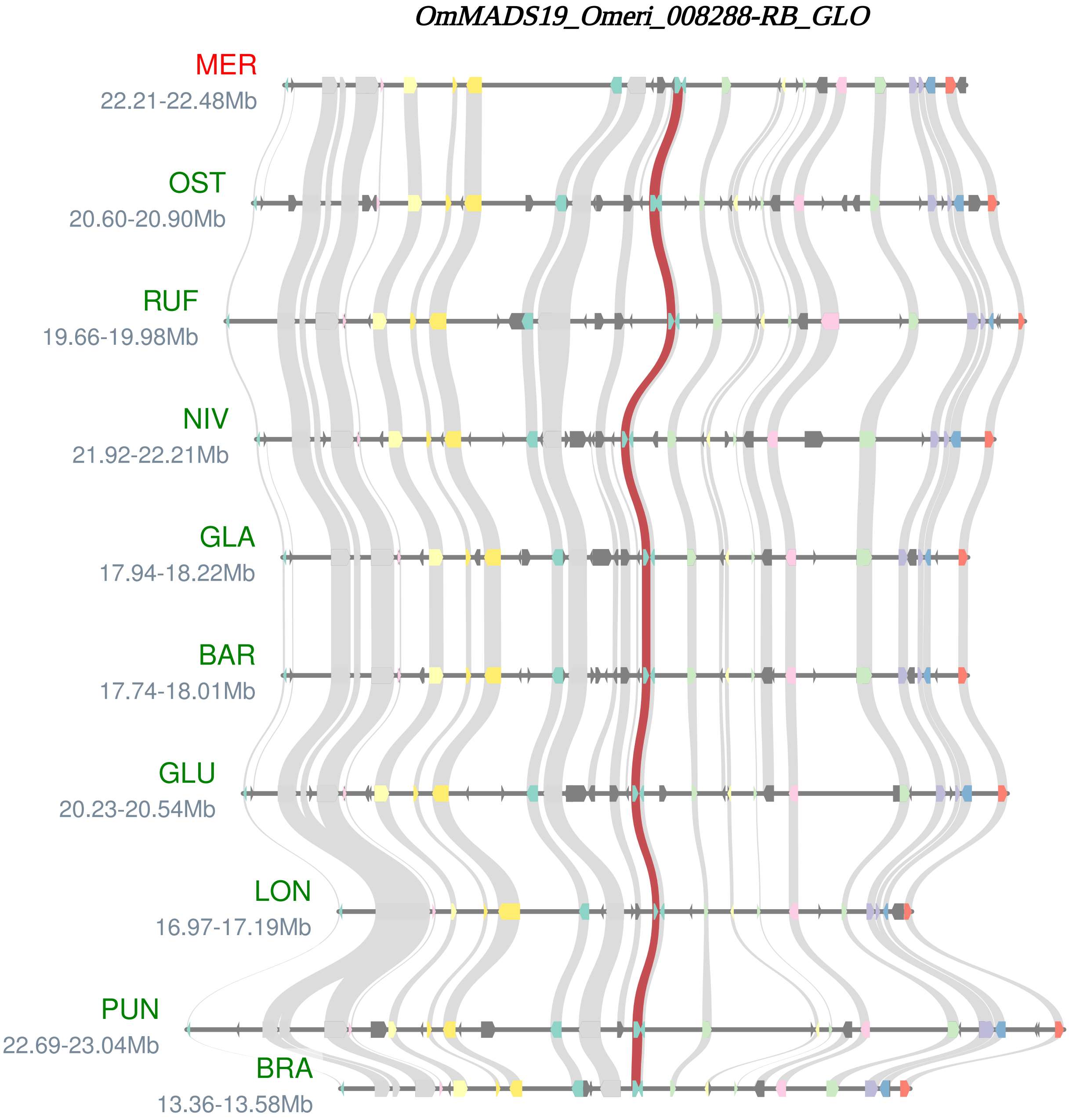
OmMADS19_Omeri_008288-RB_GLO
MER
22.21-22.48Mb
OST
20.60-20.90Mb
RUF
19.66-19.98Mb
NIV
21.92-22.21Mb
GLA
17.94-18.22Mb
BAR
17.74-18.01Mb
GLU
20.23-20.54Mb
LON
16.97-17.19Mb
PUN
22.69-23.04Mb
BRA
13.36-13.58Mb

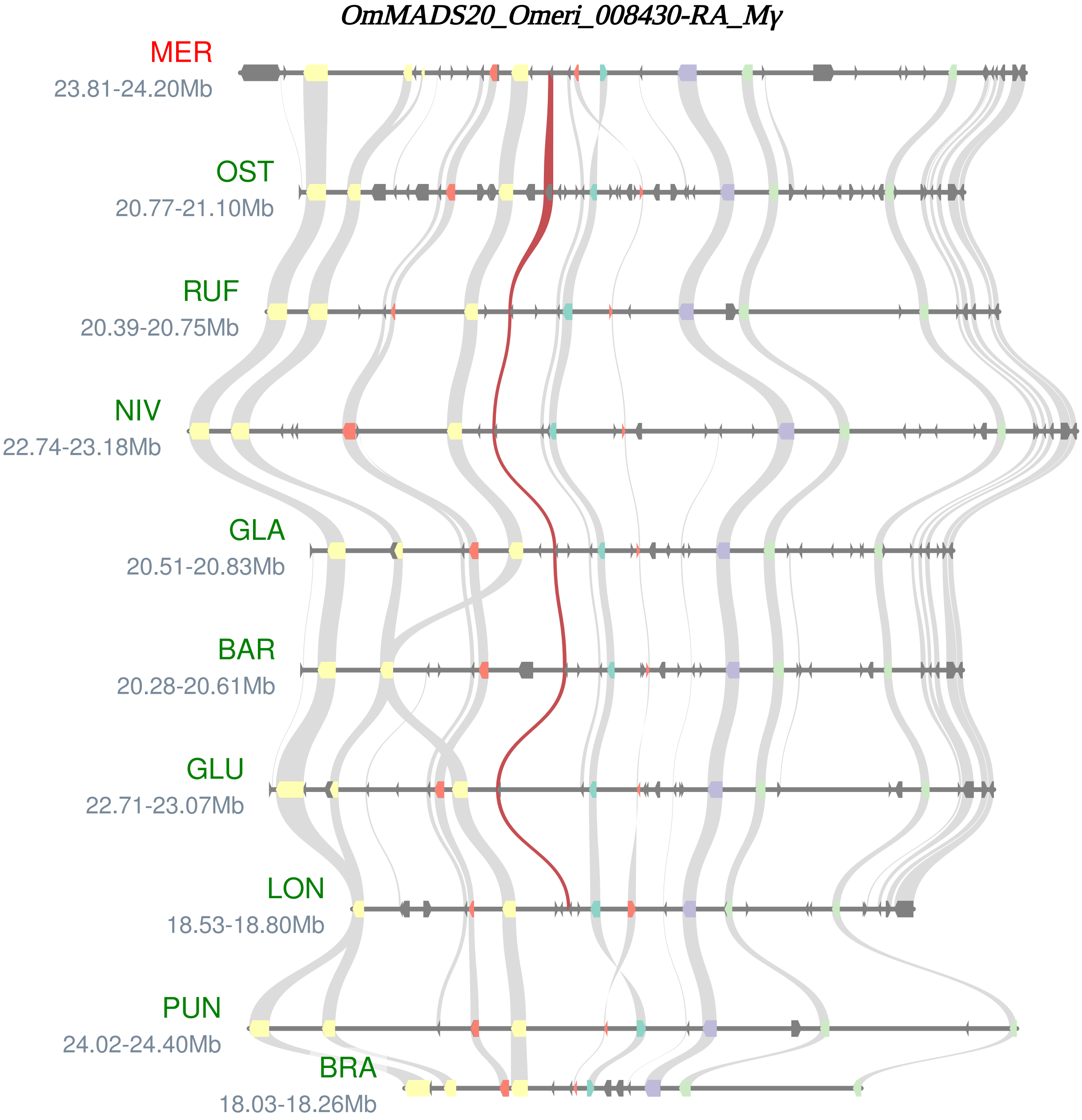

OmMADS20_Omeri_008430-RA_Mγ
MER
23.81-24.20Mb
OST
20.77-21.10Mb
RUF
20.39-20.75Mb
NIV
22.74-23.18Mb
GLA
20.51-20.83Mb
BAR
20.28-20.61Mb
GLU
22.71-23.07Mb
LON
18.53-18.80Mb
PUN
24.02-24.40Mb
BRA
18.03-18.26Mb

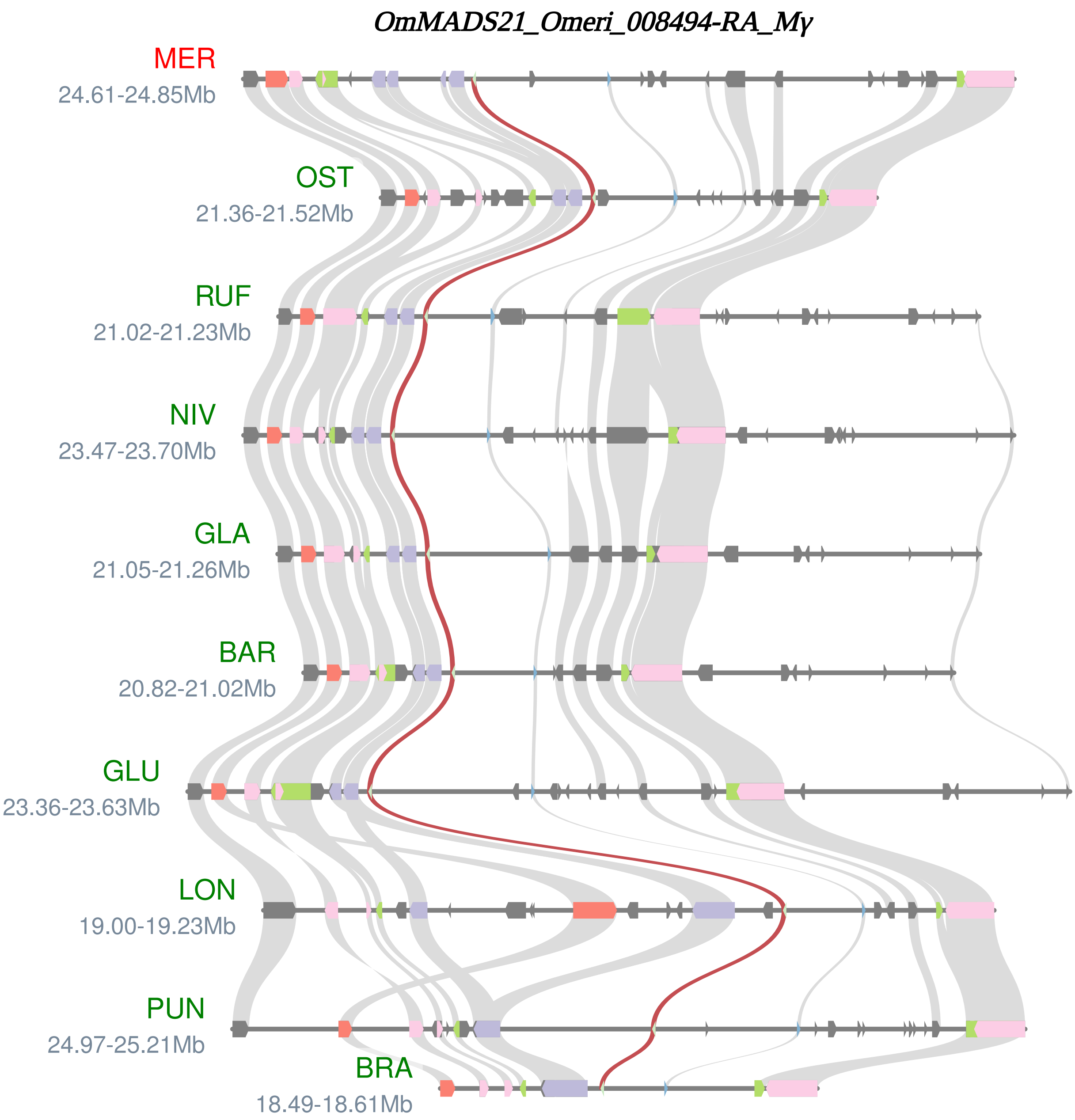

OmMADS21_Omeri_008494-RA_Mγ
MER
24.61-24.85Mb
OST
21.36-21.52Mb
RUF
21.02-21.23Mb
NIV
23.47-23.70Mb
GLA
21.05-21.26Mb
BAR
20.82-21.02Mb
GLU
23.36-23.63Mb
LON
19.00-19.23Mb
PUN
24.97-25.21Mb
BRA
18.49-18.61Mb

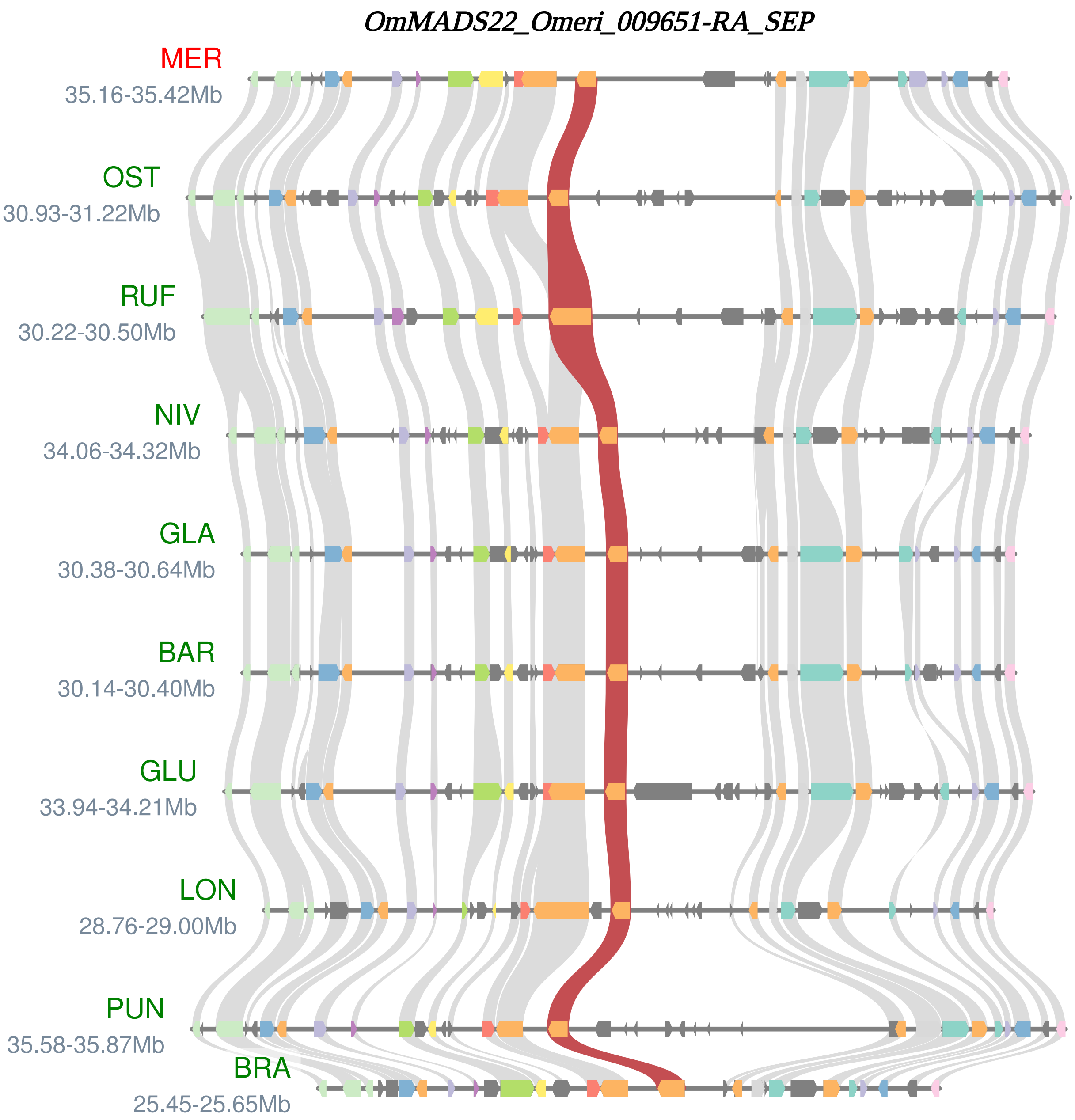

OmMADS22_Omeri_009651-RA_SEP
MER
35.16-35.42Mb
OST
30.93-31.22Mb
RUF
30.22-30.50Mb
NIV
34.06-34.32Mb
GLA
30.38-30.64Mb
BAR
30.14-30.40Mb
GLU
33.94-34.21Mb
LON
28.76-29.00Mb
PUN
35.58-35.87Mb
BRA
25.45-25.65Mb

OmMADS23_Omeri_010551-RA_SOC1
(The chromosomal segment in the BAR lacks any detected syntenic genes.)

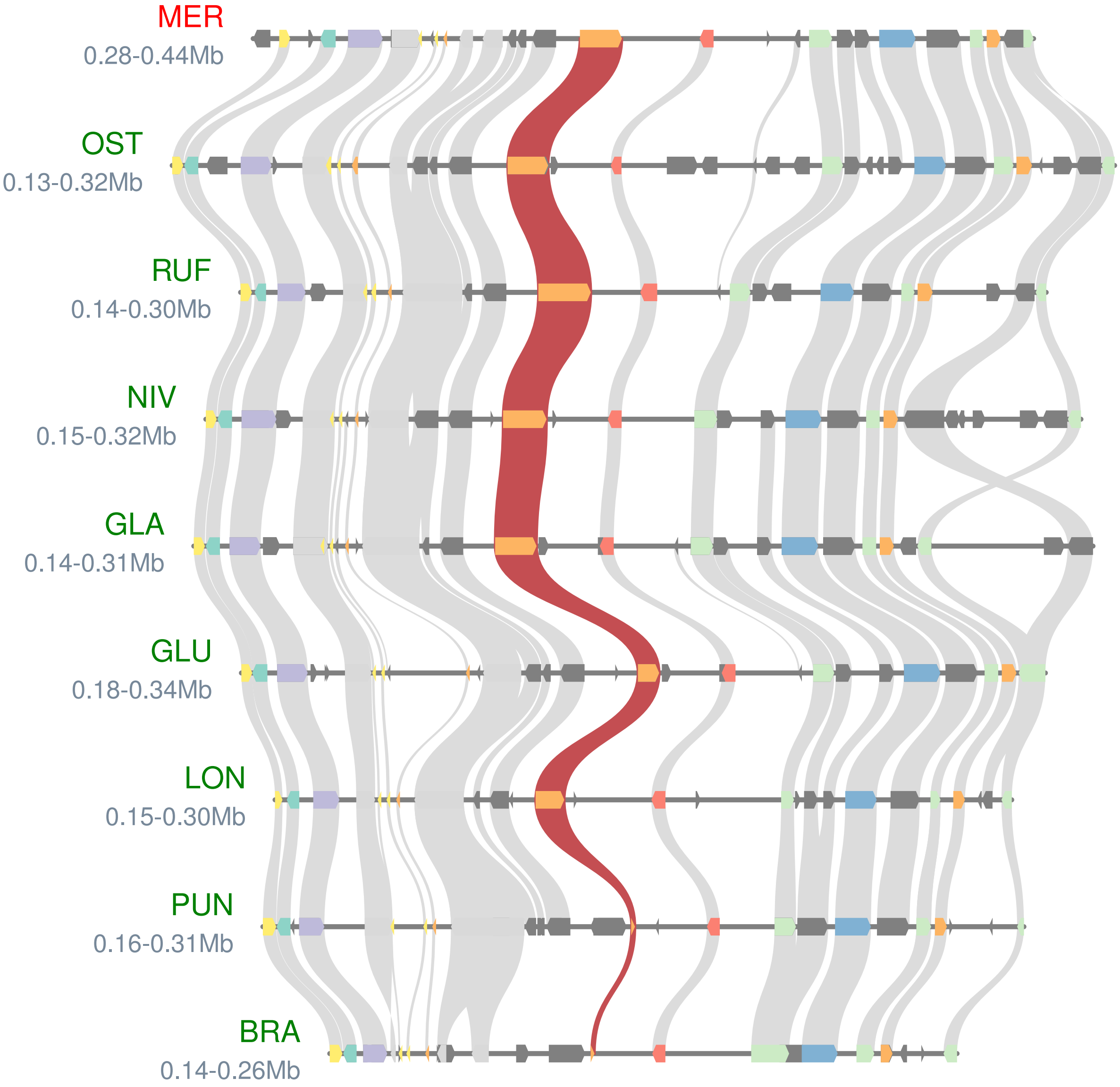

OmMADS24_Omeri_011091-RA_Mα
(The chromosomal segment in the BAR lacks any detected syntenic genes.)

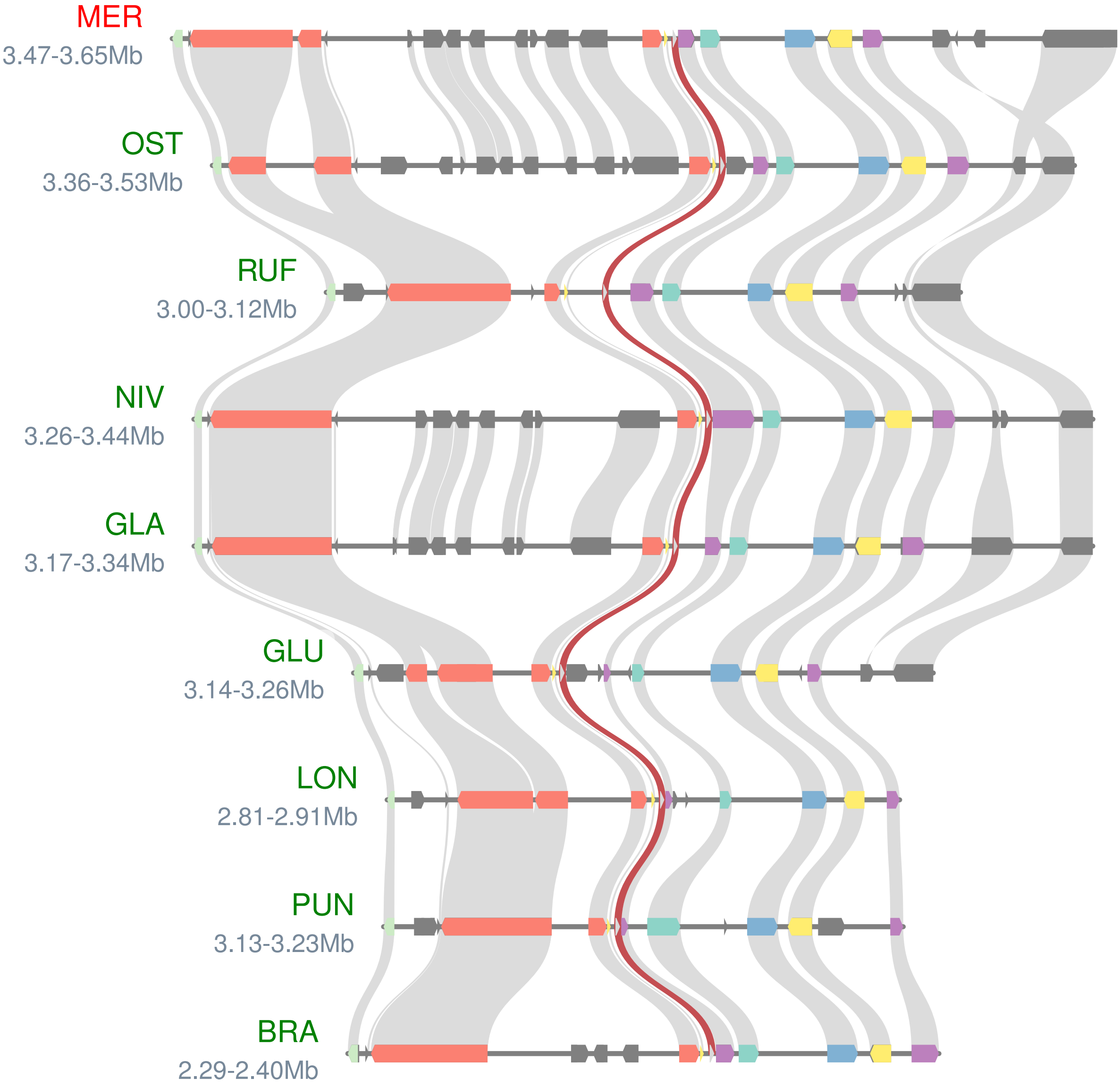

OmMADS25_Omeri_011149-RA_GGM13
(The chromosomal segment in the BAR lacks any detected syntenic genes.)

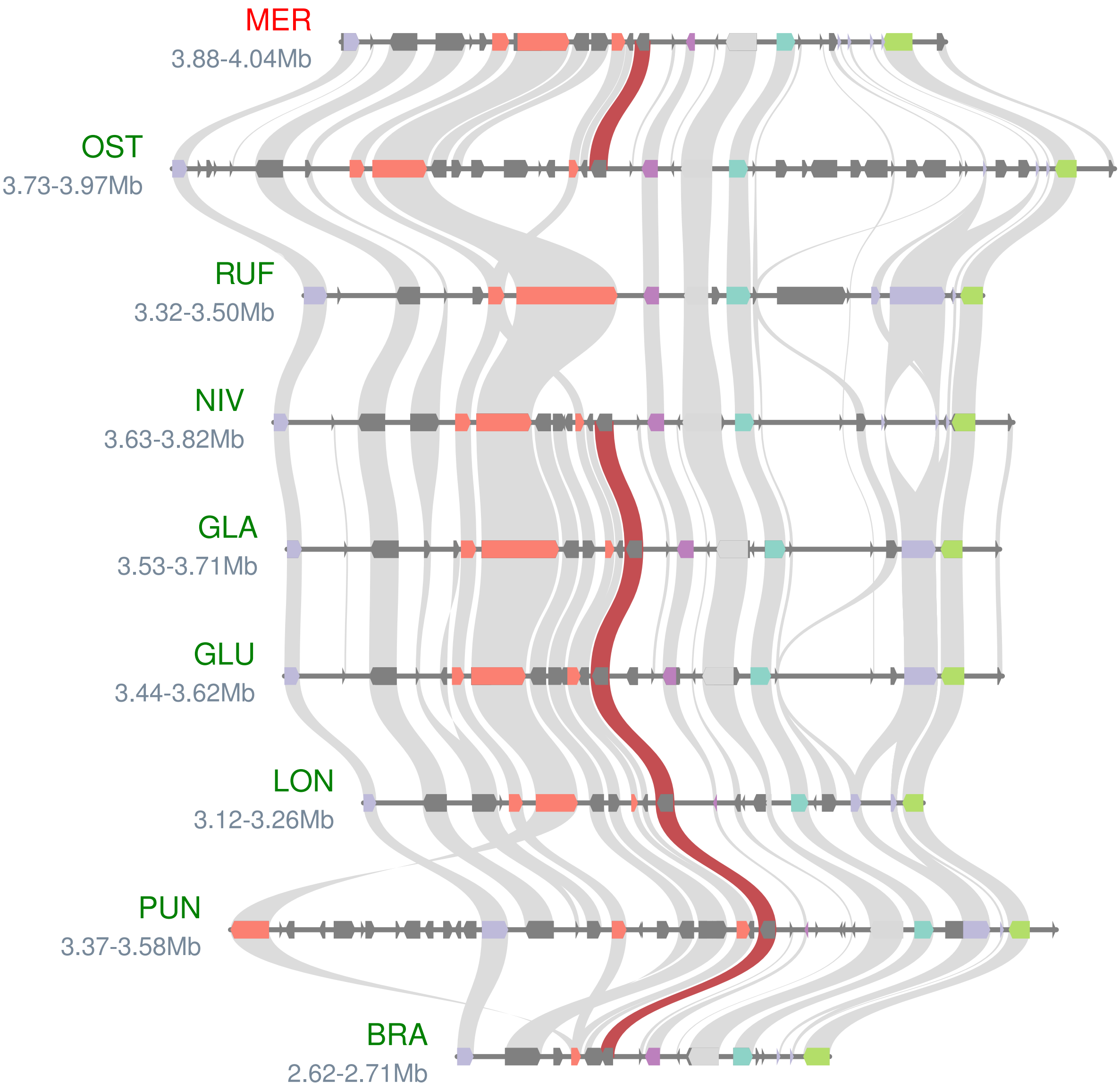

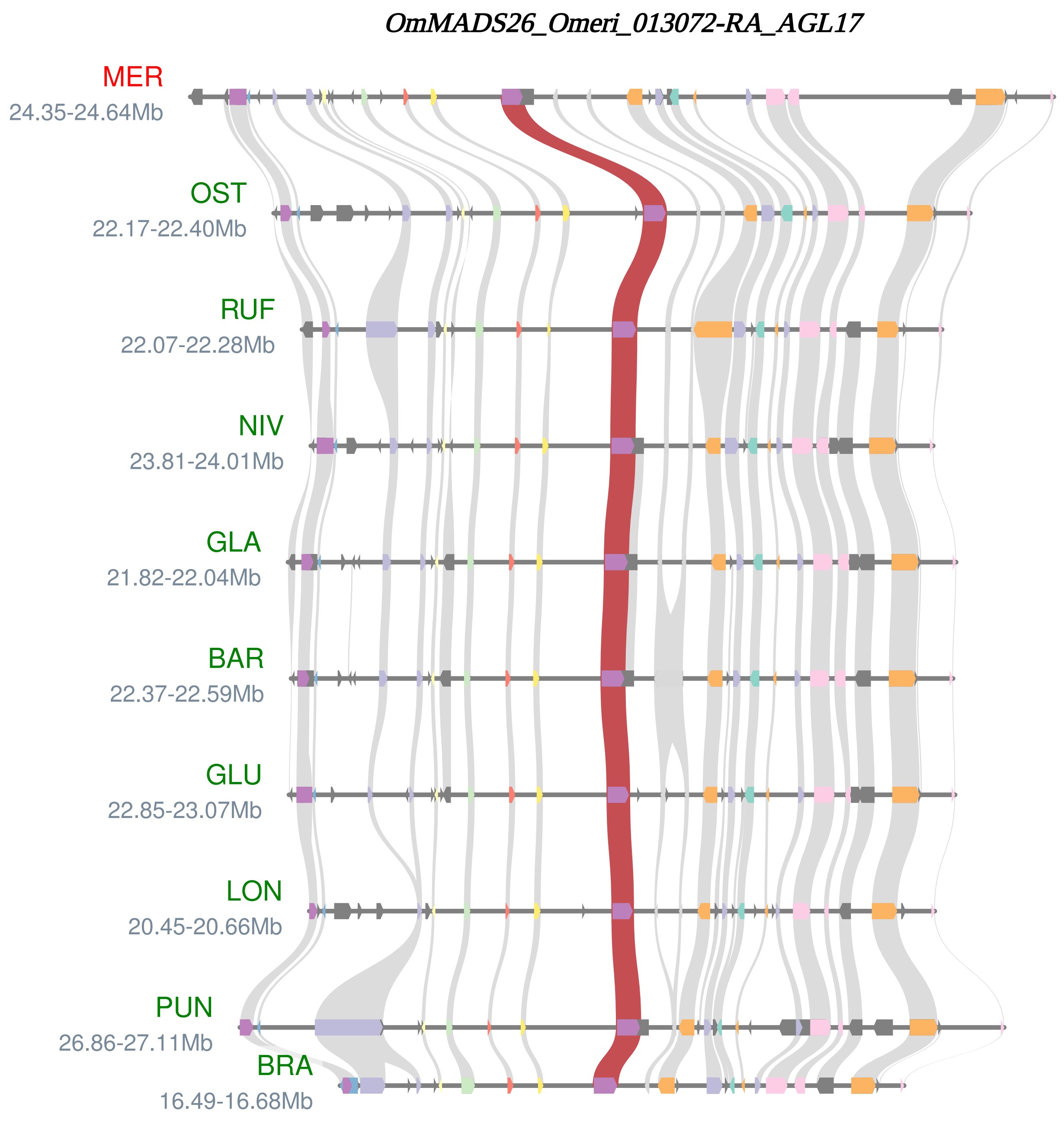

OmMADS26_Omeri_013072-RA_AGL17
MER
24.35-24.64Mb
OST
22.17-22.40Mb
RUF
22.07-22.28Mb
NIV
23.81-24.01Mb
GLA
21.82-22.04Mb
BAR
22.37-22.59Mb
GLU
22.85-23.07Mb
LON
20.45-20.66Mb
PUN
26.86-27.11Mb
BRA
16.49-16.68Mb

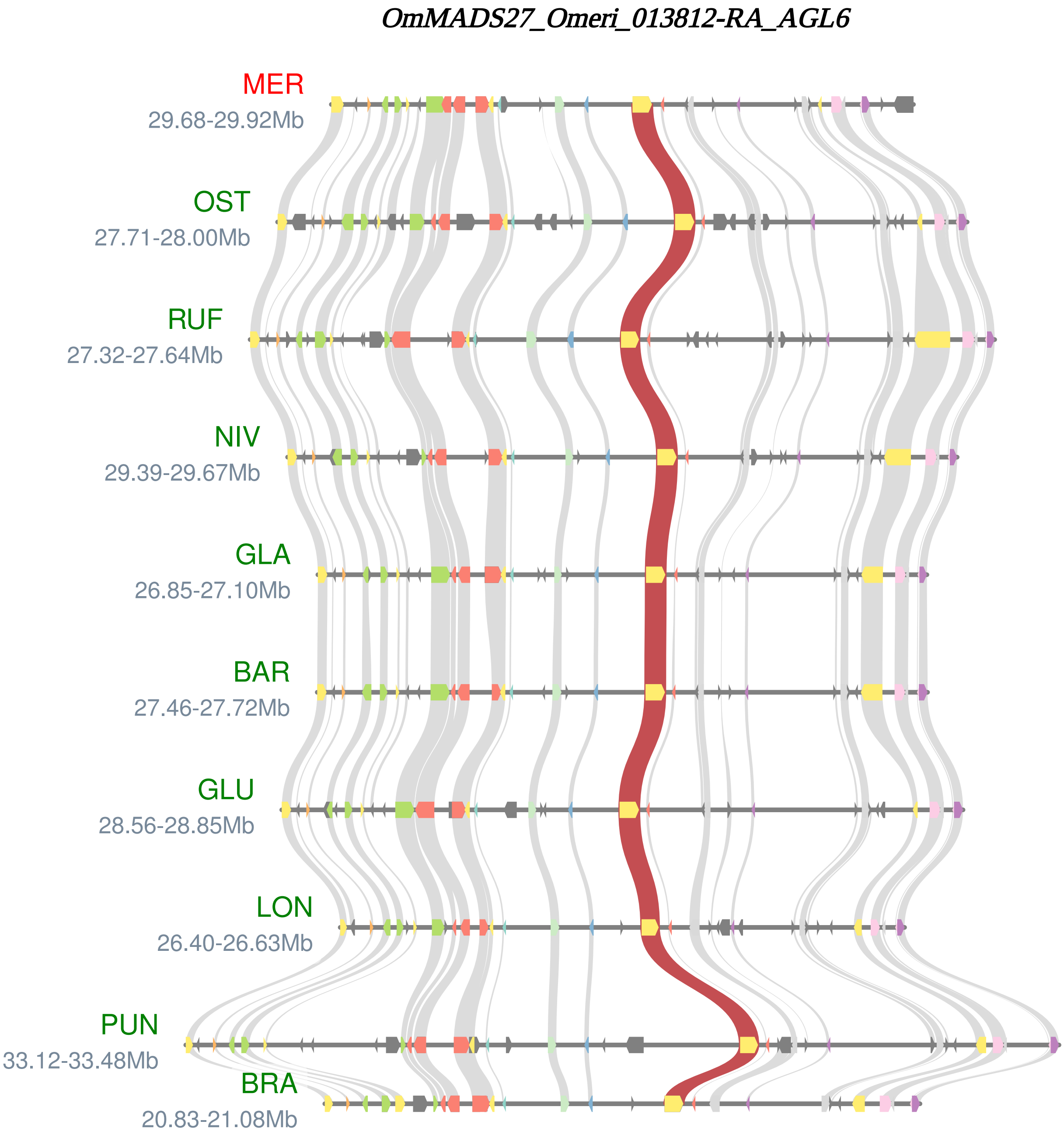
OmMADS27_Omeri_013812-RA_AGL6
MER
29.68-29.92Mb
OST
27.71-28.00Mb
RUF
27.32-27.64Mb
NIV
29.39-29.67Mb
GLA
26.85-27.10Mb
BAR
27.46-27.72Mb
GLU
28.56-28.85Mb
LON
26.40-26.63Mb
PUN
33.12-33.48Mb
BRA
20.83-21.08Mb

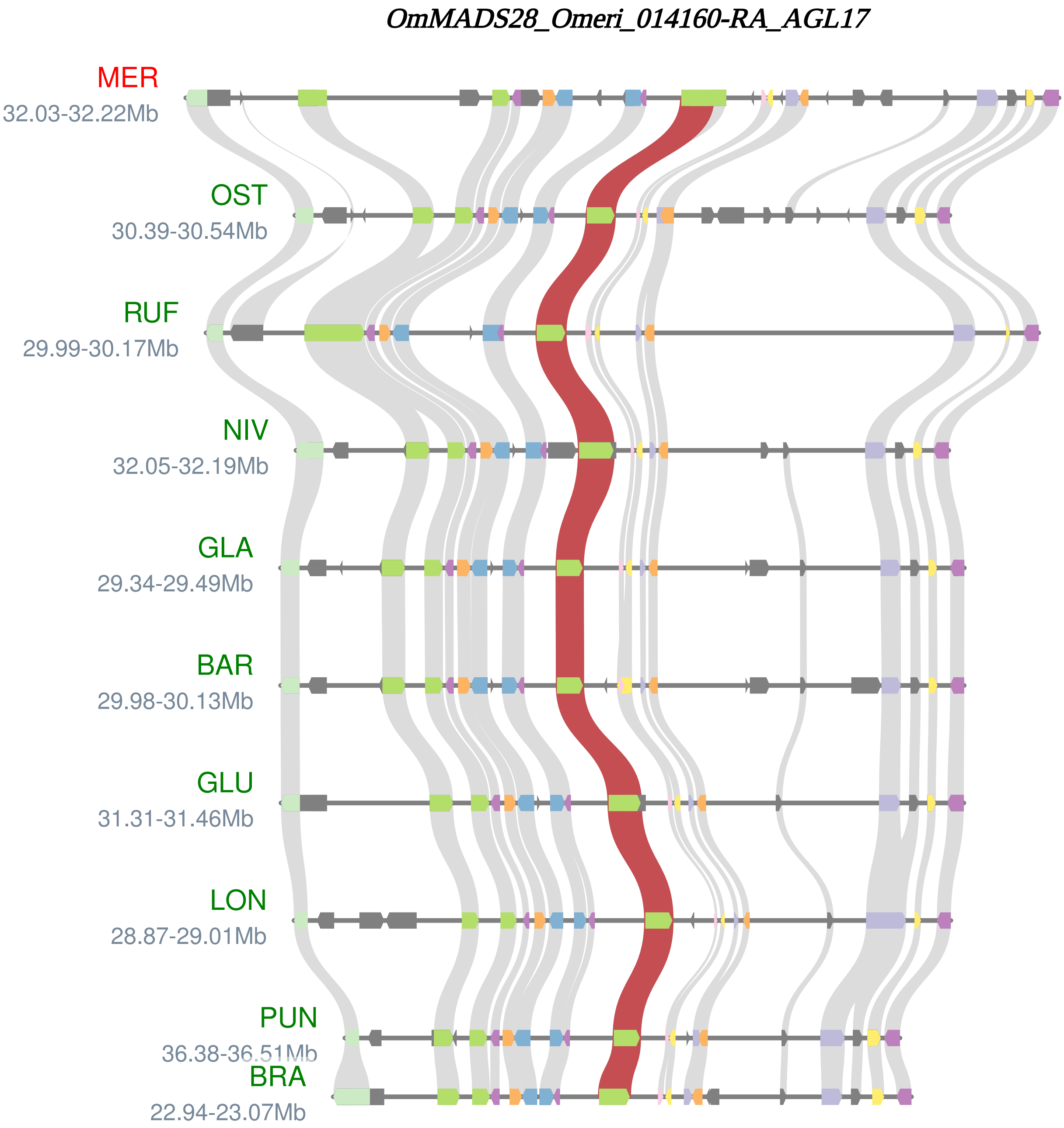
OmMADS28_Omeri_014160-RA_AGL17
MER
32.03-32.22Mb
OST
30.39-30.54Mb
RUF
29.99-30.17Mb
NIV
32.05-32.19Mb
GLA
29.34-29.49Mb
BAR
29.98-30.13Mb
GLU
31.31-31.46Mb
LON
28.87-29.01Mb
PUN
36.38-36.51Mb
BRA
22.94-23.07Mb

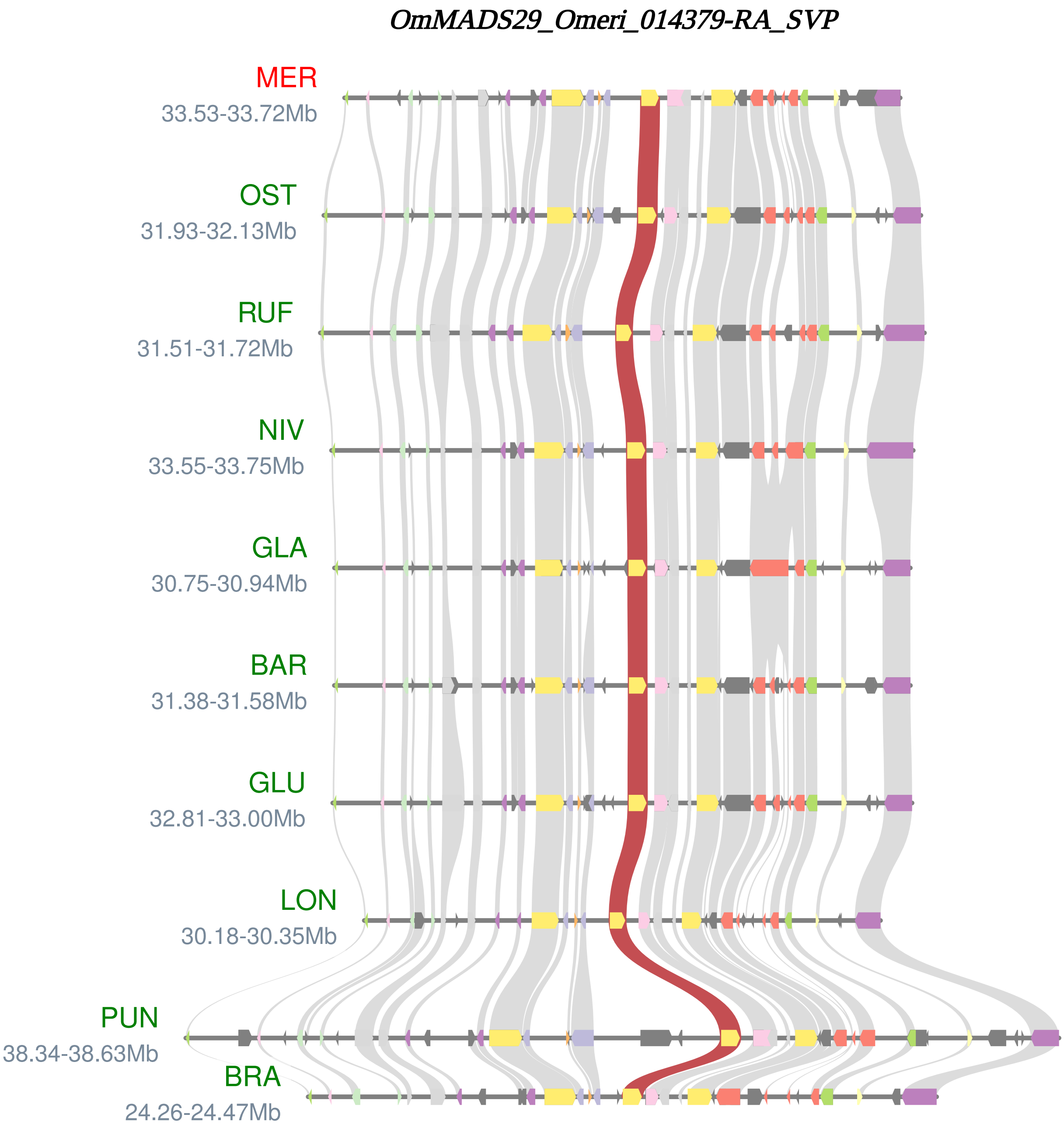
OmMADS29_Omeri_014379-RA_SVP
MER
33.53-33.72Mb
OST
31.93-32.13Mb
RUF
31.51-31.72Mb
NIV
33.55-33.75Mb
GLA
30.75-30.94Mb
BAR
31.38-31.58Mb
GLU
32.81-33.00Mb
LON
30.18-30.35Mb
PUN
38.34-38.63Mb
BRA
24.26-24.47Mb

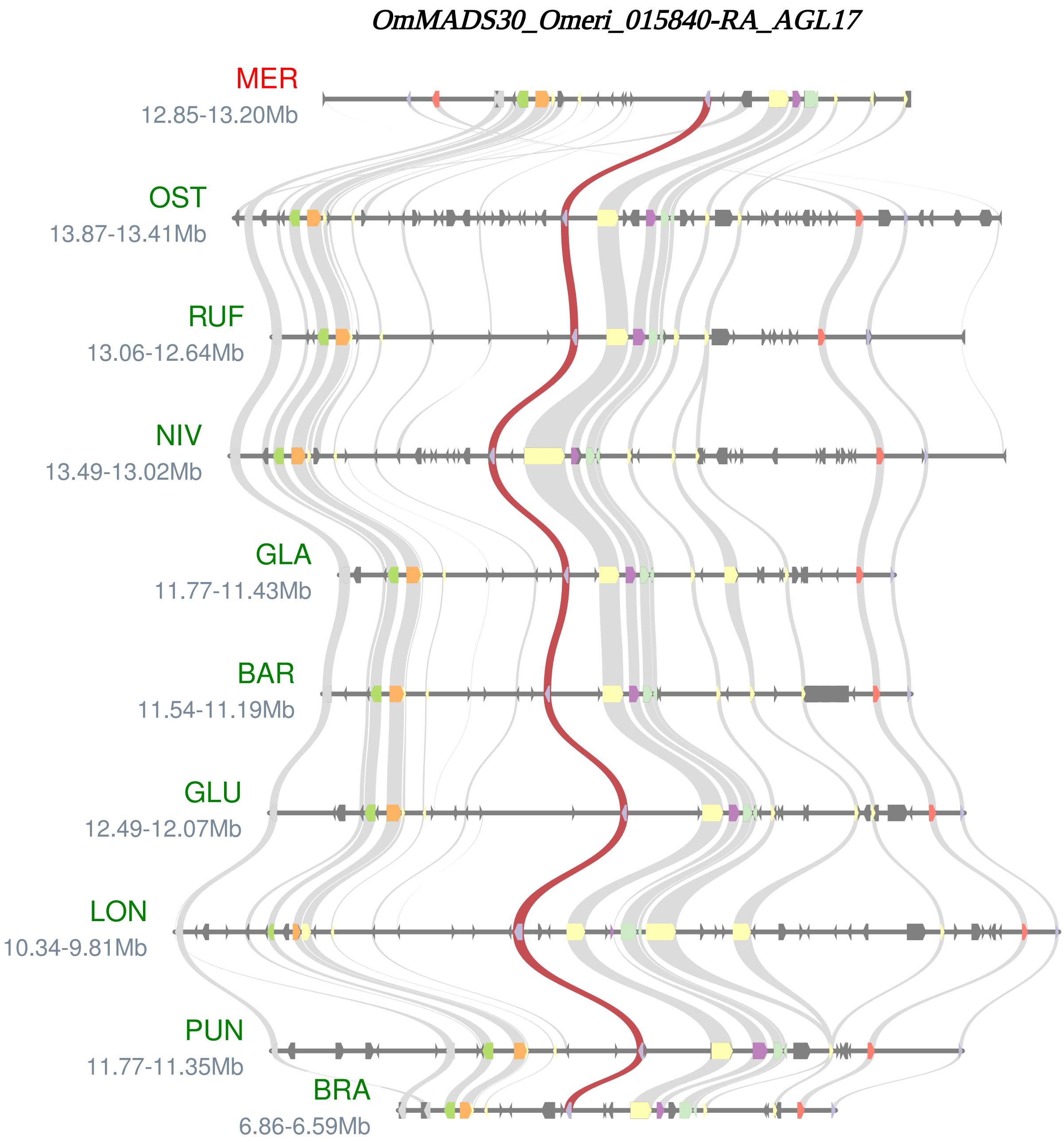

OmMADS30_Omeri_015840-RA_AGL17
MER
12.85-13.20Mb
OST
13.87-13.41Mb
RUF
13.06-12.64Mb
NIV
13.49-13.02Mb
GLA
11.77-11.43Mb
BAR
11.54-11.19Mb
GLU
12.49-12.07Mb
LON
10.34-9.81Mb
PUN
11.77-11.35Mb
BRA
6.86-6.59Mb

OmMADS31_Omeri_015895-RA_Mγ
OmMADS32_Omeri_015903-RA_Mγ
（The chromosomal segment in the GLU lacks any detected syntenic genes.)

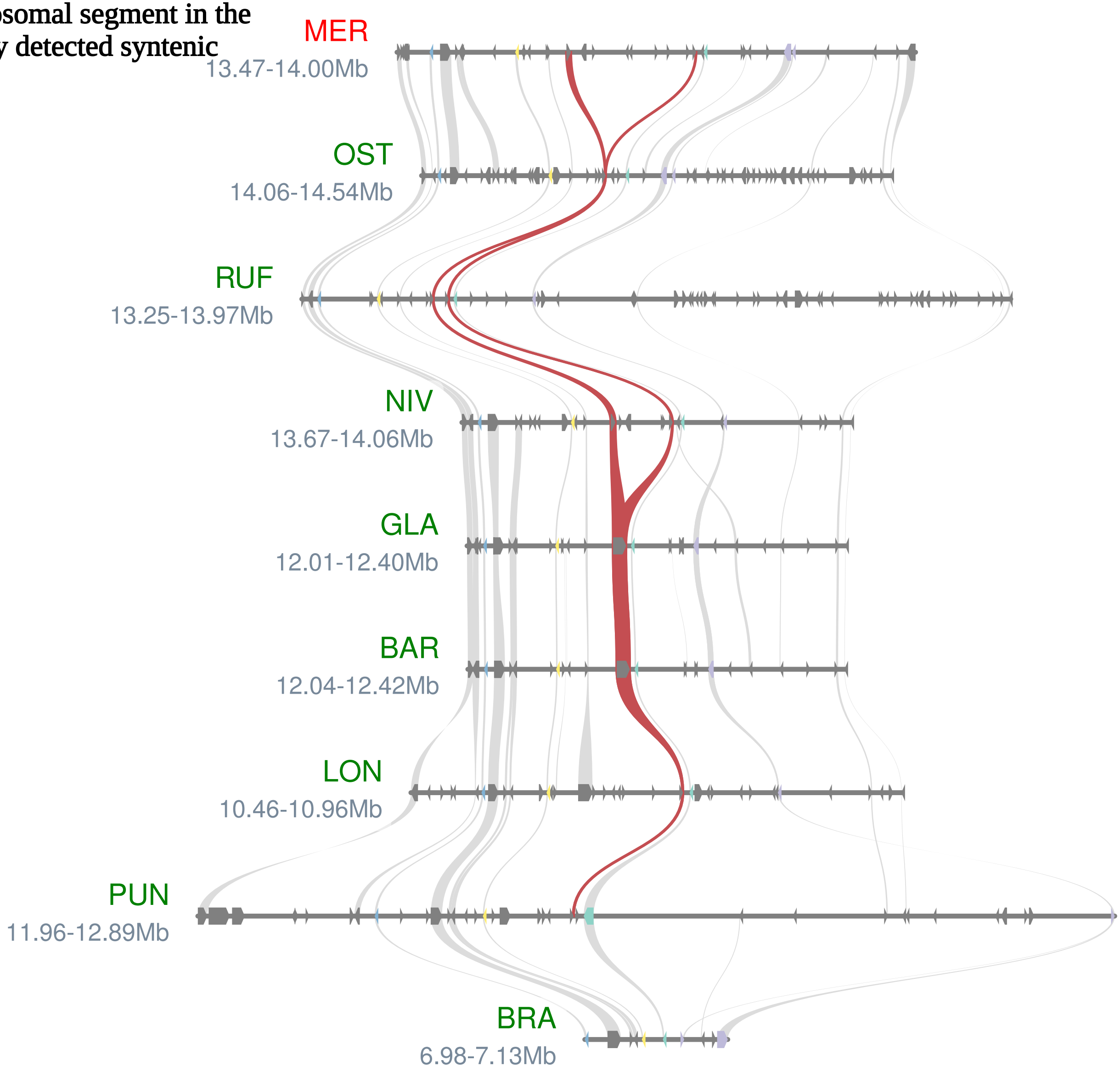

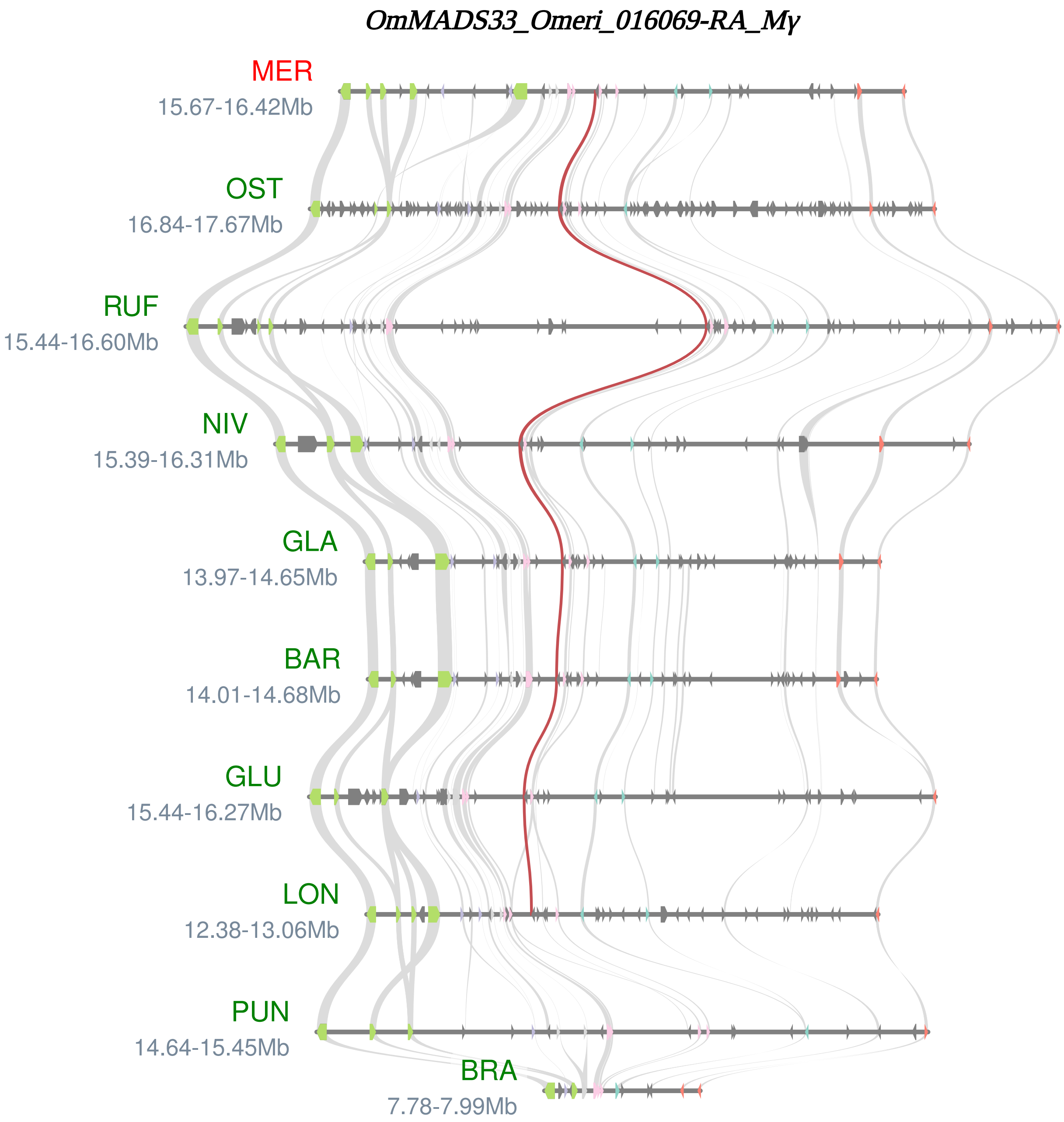
OmMADS33_Omeri_016069-RA_Mγ
MER
15.67-16.42Mb
OST
16.84-17.67Mb
RUF
15.44-16.60Mb
NIV
15.39-16.31Mb
GLA
13.97-14.65Mb
BAR
14.01-14.68Mb
GLU
15.44-16.27Mb
LON
12.38-13.06Mb
PUN
14.64-15.45Mb
BRA
7.78-7.99Mb

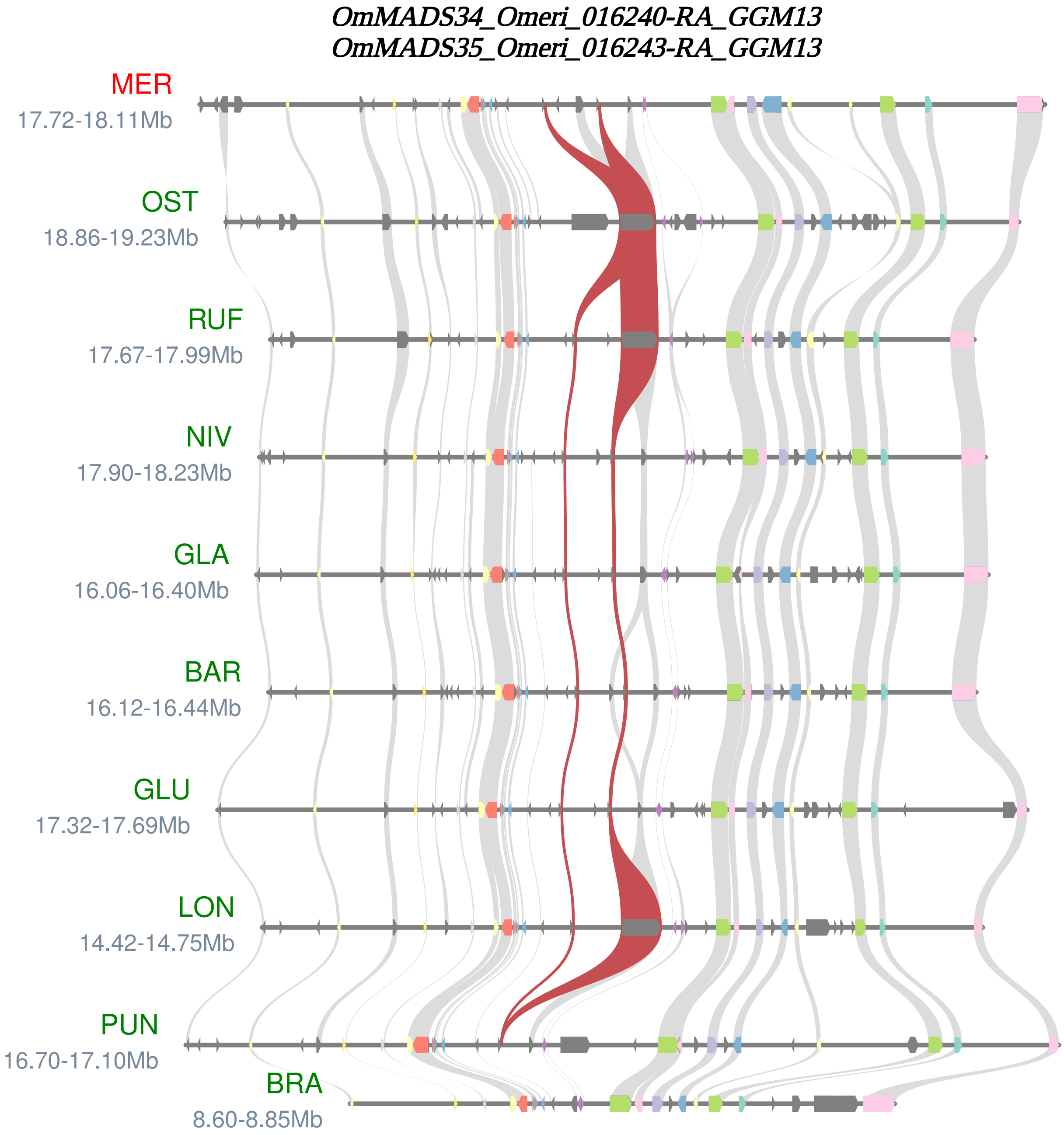
OmMADS34_Omeri_016240-RA_GGM13
OmMADS35_Omeri_016243-RA_GGM13
MER
17.72-18.11Mb
OST
18.86-19.23Mb
RUF
17.67-17.99Mb
NIV
17.90-18.23Mb
GLA
16.06-16.40Mb
BAR
16.12-16.44Mb
GLU
17.32-17.69Mb
LON
14.42-14.75Mb
PUN
16.70-17.10Mb
BRA
8.60-8.85Mb

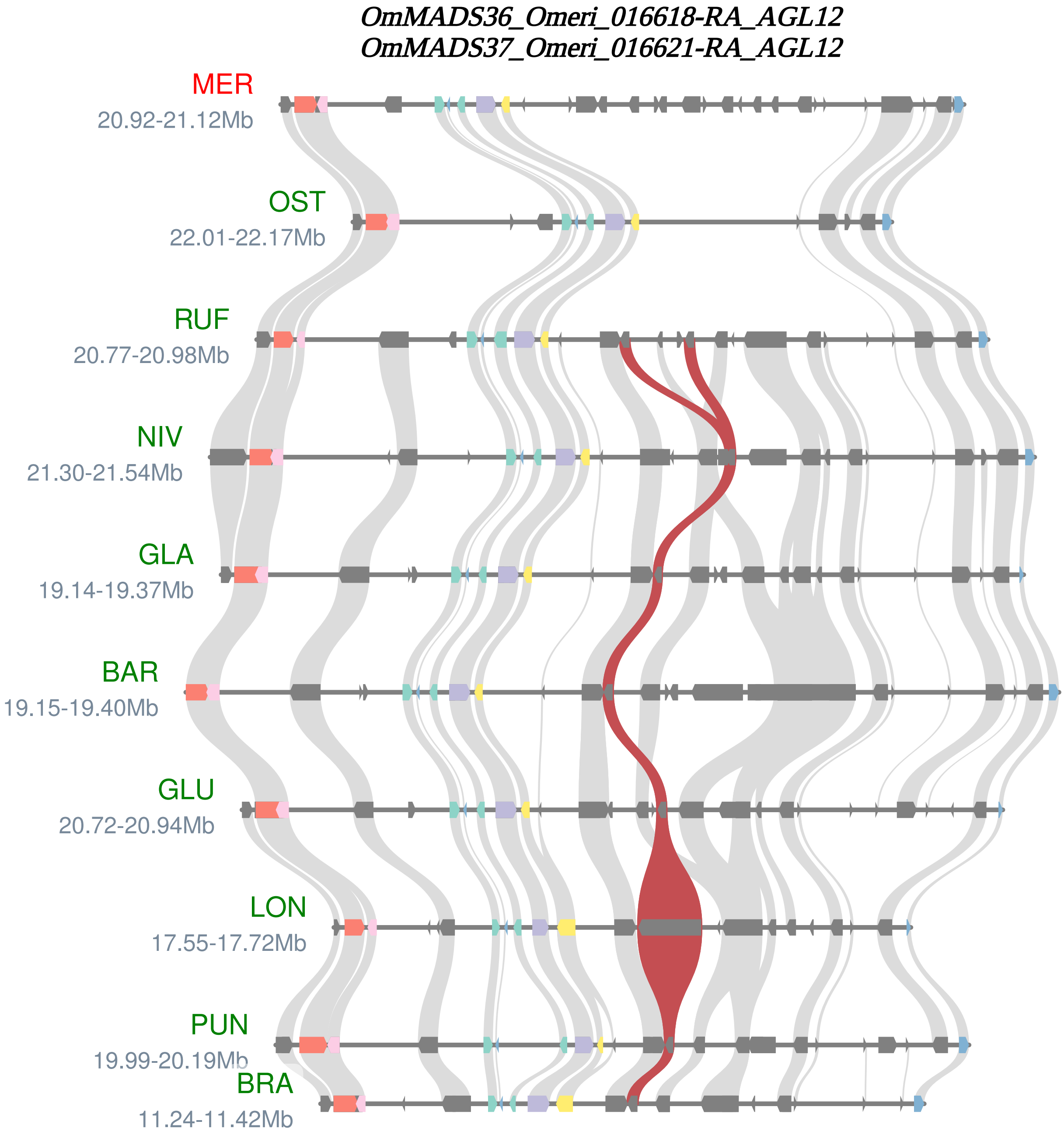

OmMADS36_Omeri_016618-RA_AGL12
OmMADS37_Omeri_016621-RA_AGL12
MER
20.92-21.12Mb
OST
22.01-22.17Mb
RUF
20.77-20.98Mb
NIV
21.30-21.54Mb
GLA
19.14-19.37Mb
BAR
19.15-19.40Mb
GLU
20.72-20.94Mb
LON
17.55-17.72Mb
PUN
19.99-20.19Mb
BRA
11.24-11.42Mb

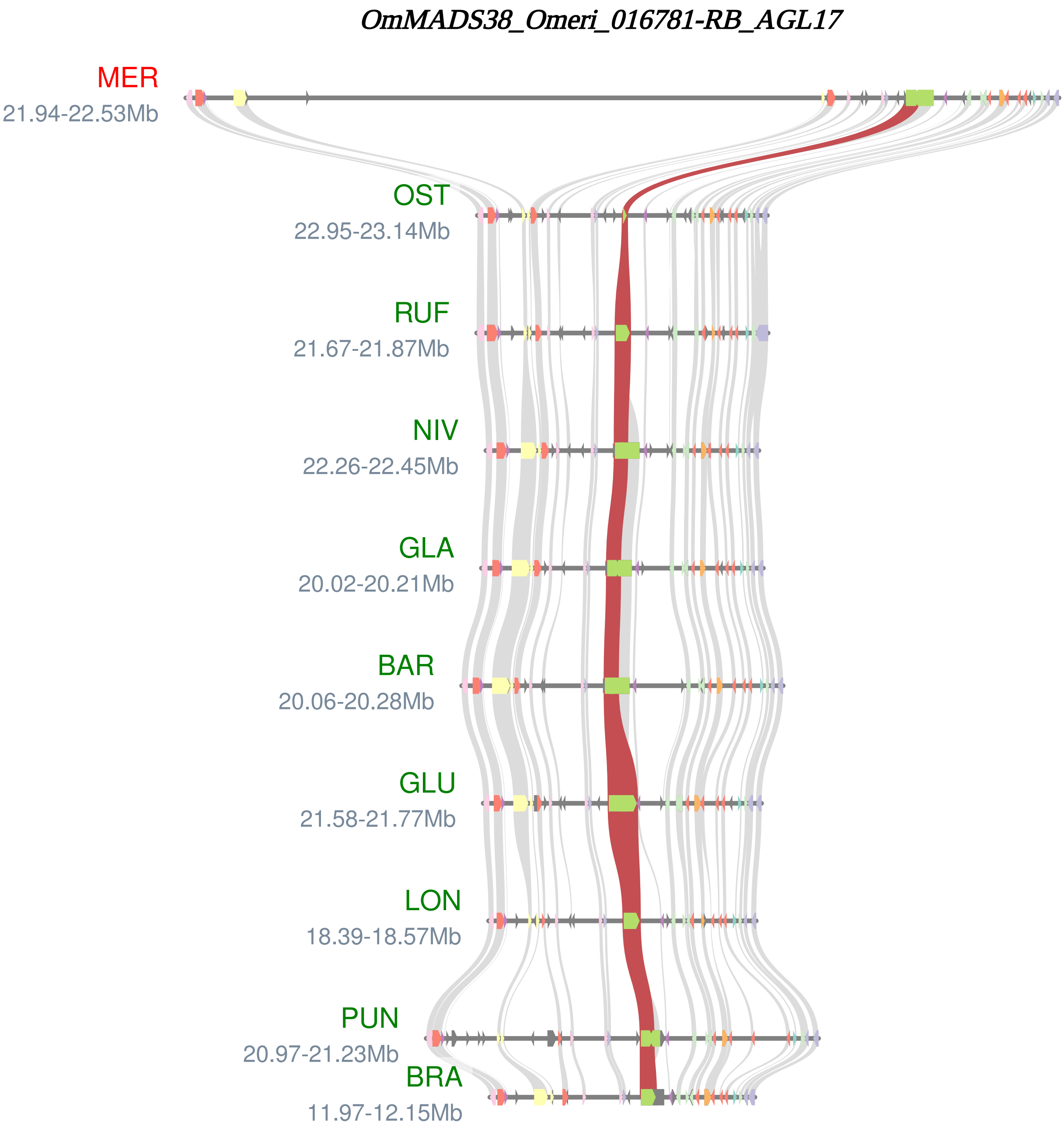
OmMADS38_Omeri_016781-RB_AGL17
MER
21.94-22.53Mb
OST
22.95-23.14Mb
RUF
21.67-21.87Mb
NIV
22.26-22.45Mb
GLA
20.02-20.21Mb
BAR
20.06-20.28Mb
GLU
21.58-21.77Mb
LON
18.39-18.57Mb
PUN
20.97-21.23Mb
BRA
11.97-12.15Mb

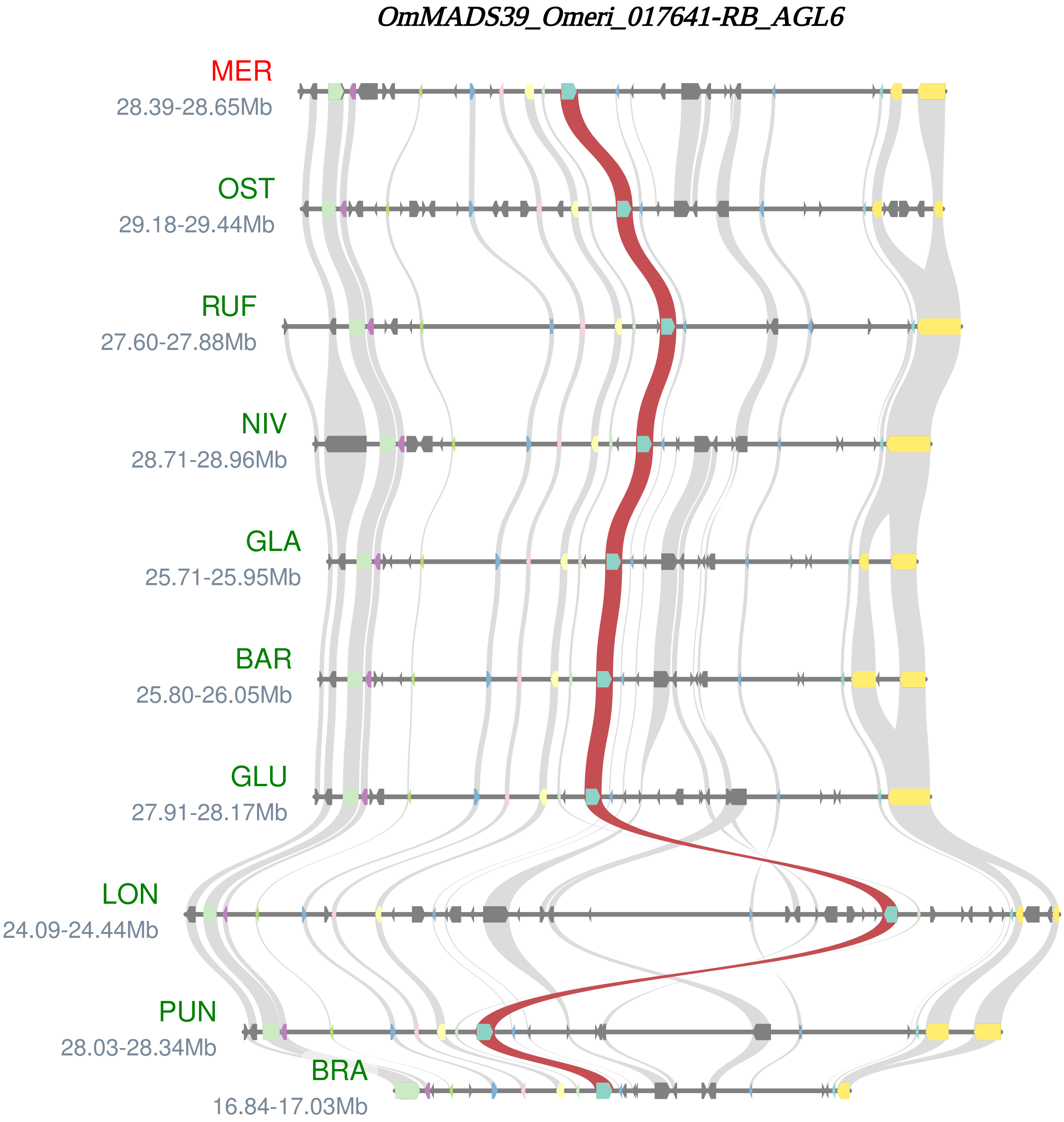

OmMADS39_Omeri_017641-RB_AGL6
MER
28.39-28.65Mb
OST
29.18-29.44Mb
RUF
27.60-27.88Mb
NIV
28.71-28.96Mb
GLA
25.71-25.95Mb
BAR
25.80-26.05Mb
GLU
27.91-28.17Mb
LON
24.09-24.44Mb
PUN
28.03-28.34Mb
BRA
16.84-17.03Mb

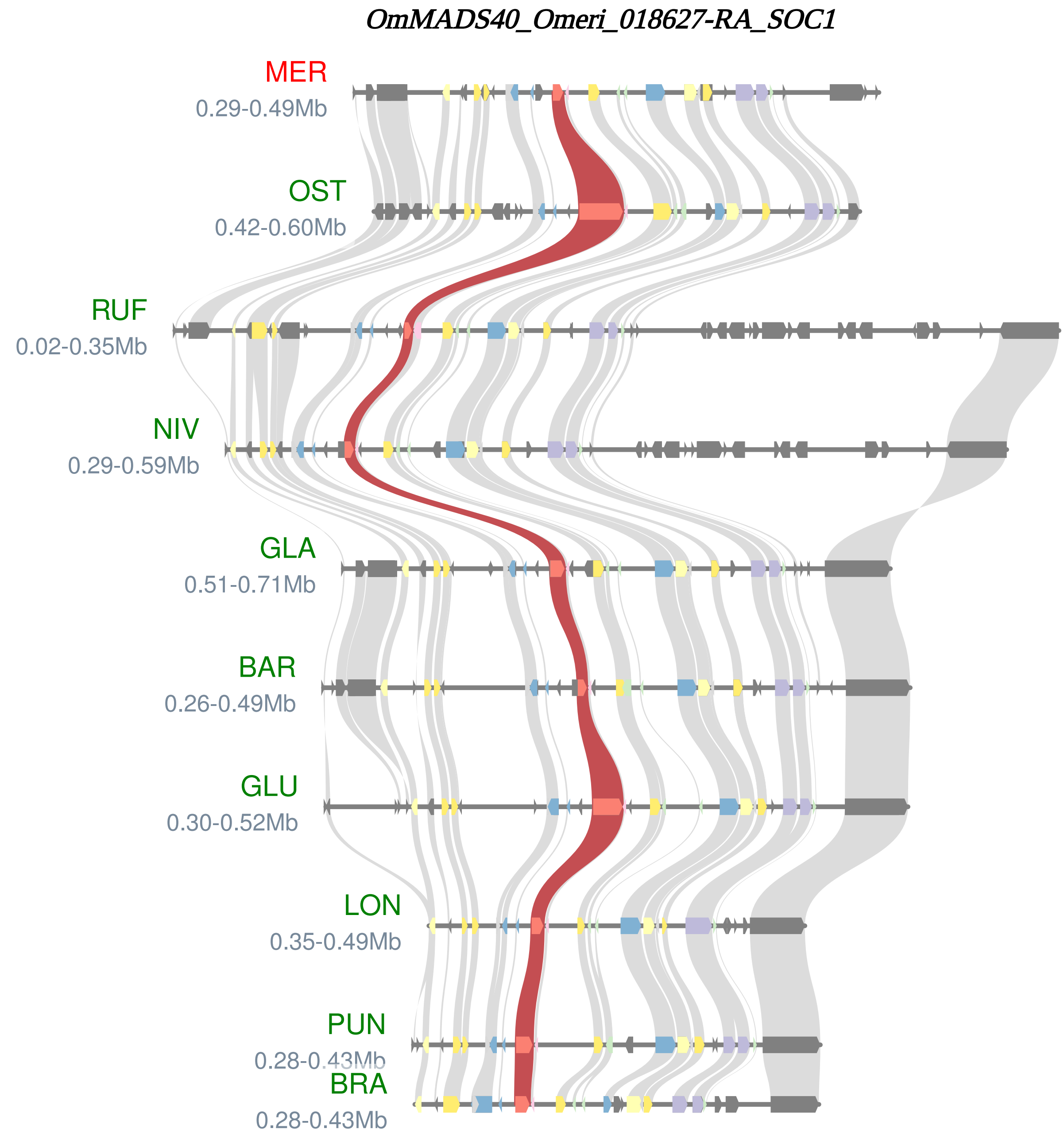
OmMADS40_Omeri_018627-RA_SOC1
MER
0.29-0.49Mb
OST
0.42-0.60Mb
RUF
0.02-0.35Mb
NIV
0.29-0.59Mb
GLA
0.51-0.71Mb
BAR
0.26-0.49Mb
GLU
0.30-0.52Mb
LON
0.35-0.49Mb
PUN
0.28-0.43Mb
BRA
0.28-0.43Mb

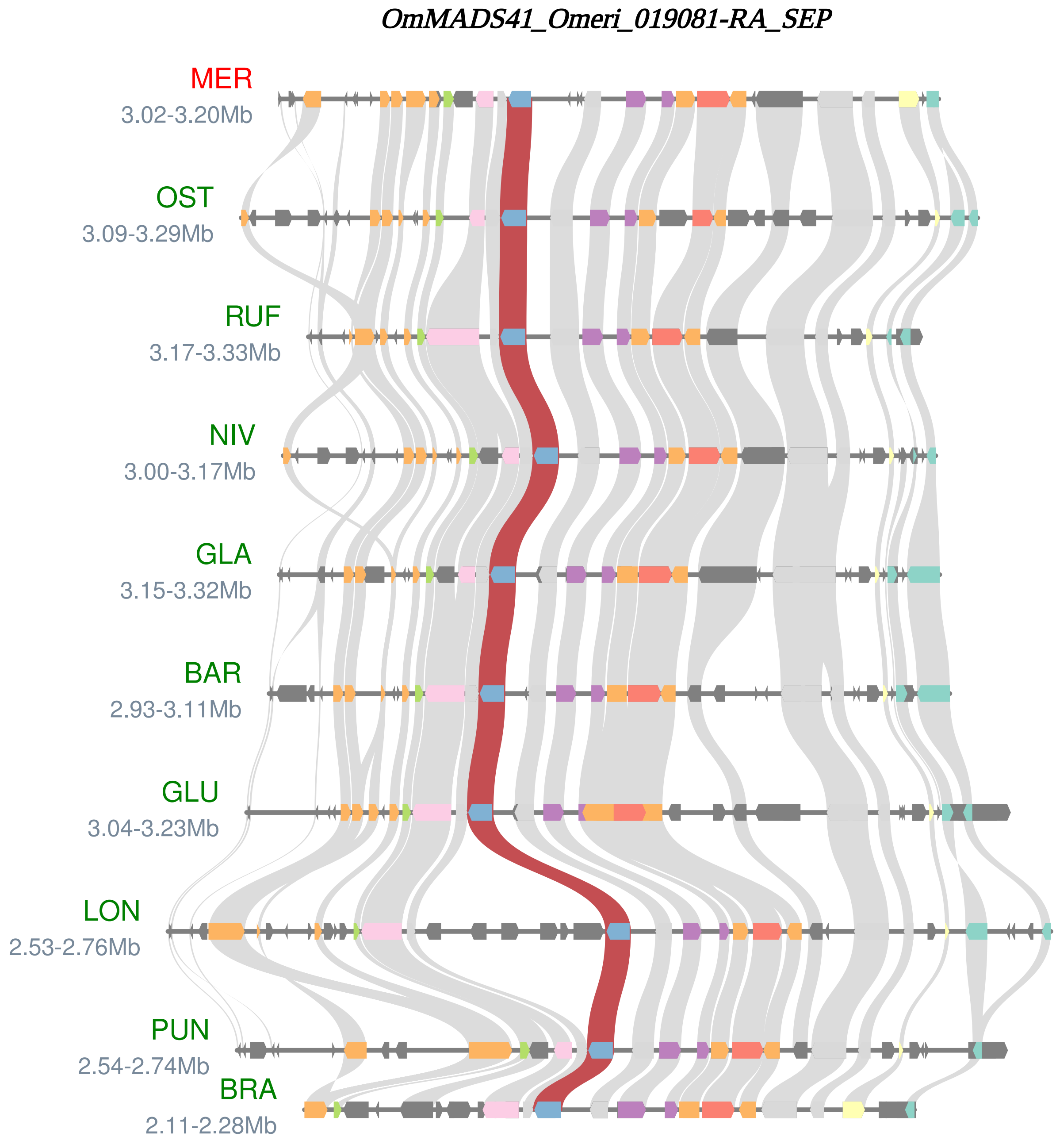
OmMADS41_Omeri_019081-RA_SEP
MER
3.02-3.20Mb
OST
3.09-3.29Mb
RUF
3.17-3.33Mb
NIV
3.00-3.17Mb
GLA
3.15-3.32Mb
BAR
2.93-3.11Mb
GLU
3.04-3.23Mb
LON
2.53-2.76Mb
PUN
2.54-2.74Mb
BRA
2.11-2.28Mb

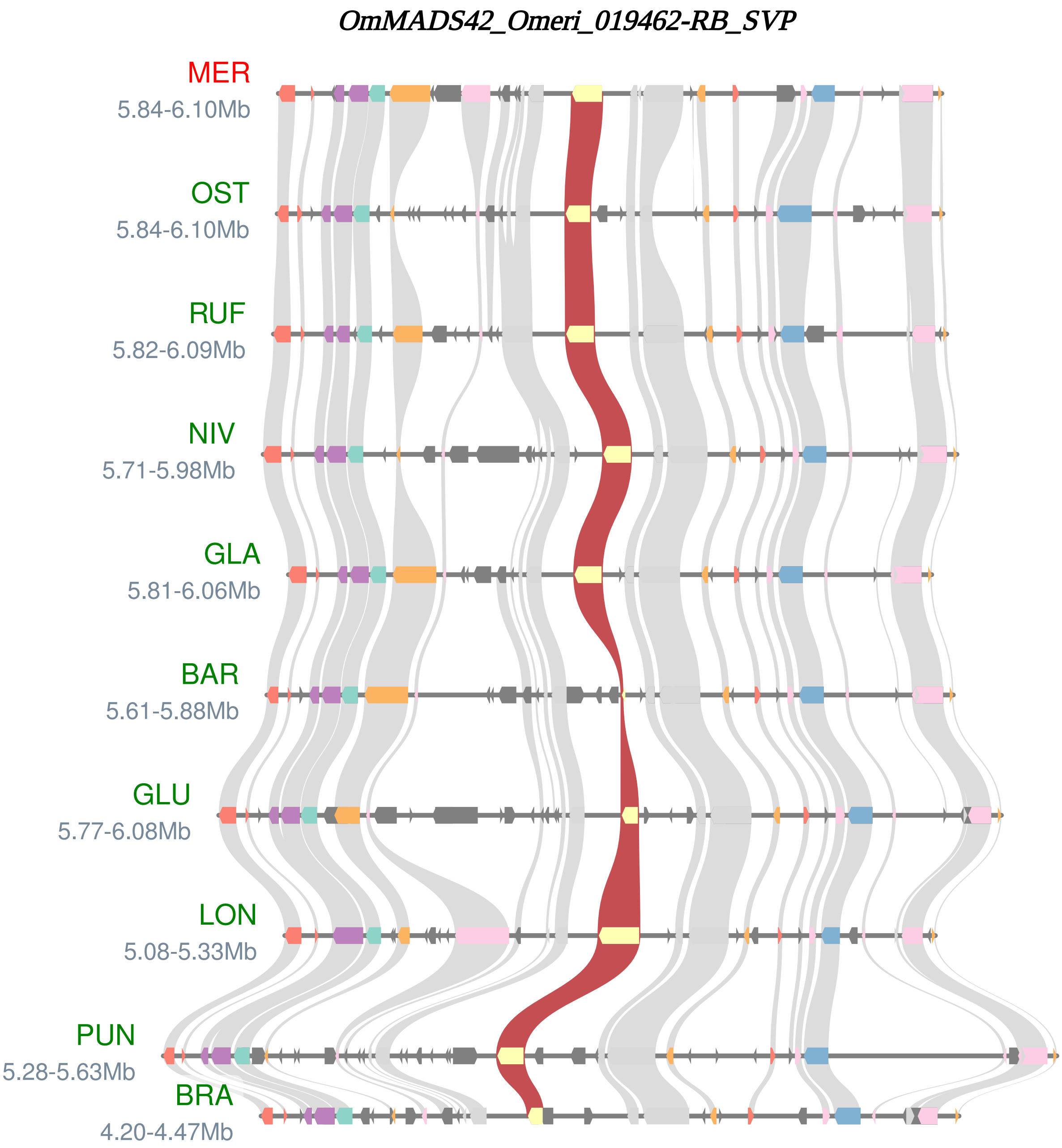

OmMADS42_Omeri_019462-RB_SVP
MER
5.84-6.10Mb
OST
5.84-6.10Mb
RUF
5.82-6.09Mb
NIV
5.71-5.98Mb
GLA
5.81-6.06Mb
BAR
5.61-5.88Mb
GLU
5.77-6.08Mb
LON
5.08-5.33Mb
PUN
5.28-5.63Mb
BRA
4.20-4.47Mb

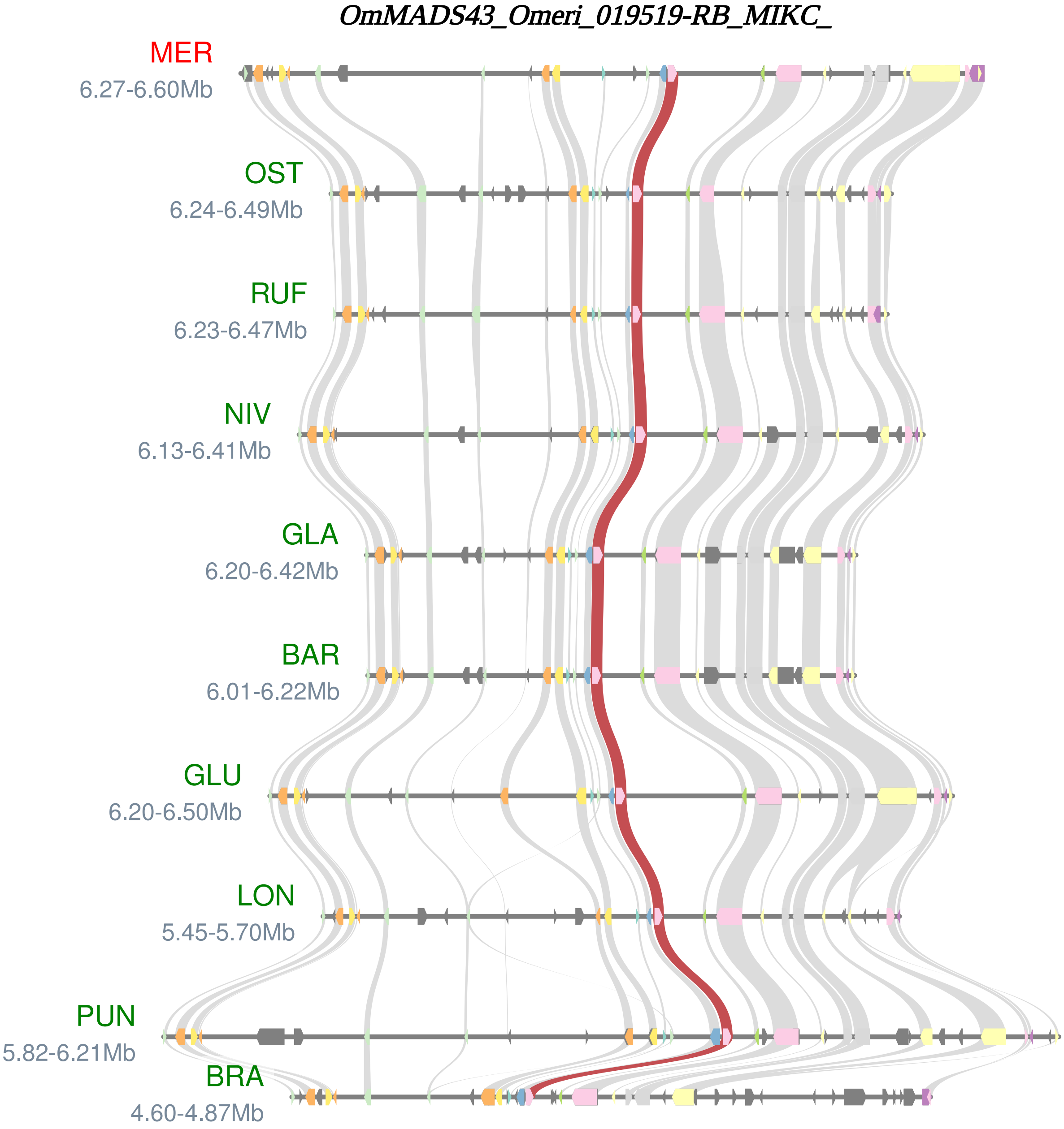
OmMADS43_Omeri_019519-RB_MIKC_
MER
6.27-6.60Mb
OST
6.24-6.49Mb
RUF
6.23-6.47Mb
NIV
6.13-6.41Mb
GLA
6.20-6.42Mb
BAR
6.01-6.22Mb
GLU
6.20-6.50Mb
LON
5.45-5.70Mb
PUN
5.82-6.21Mb
BRA
4.60-4.87Mb

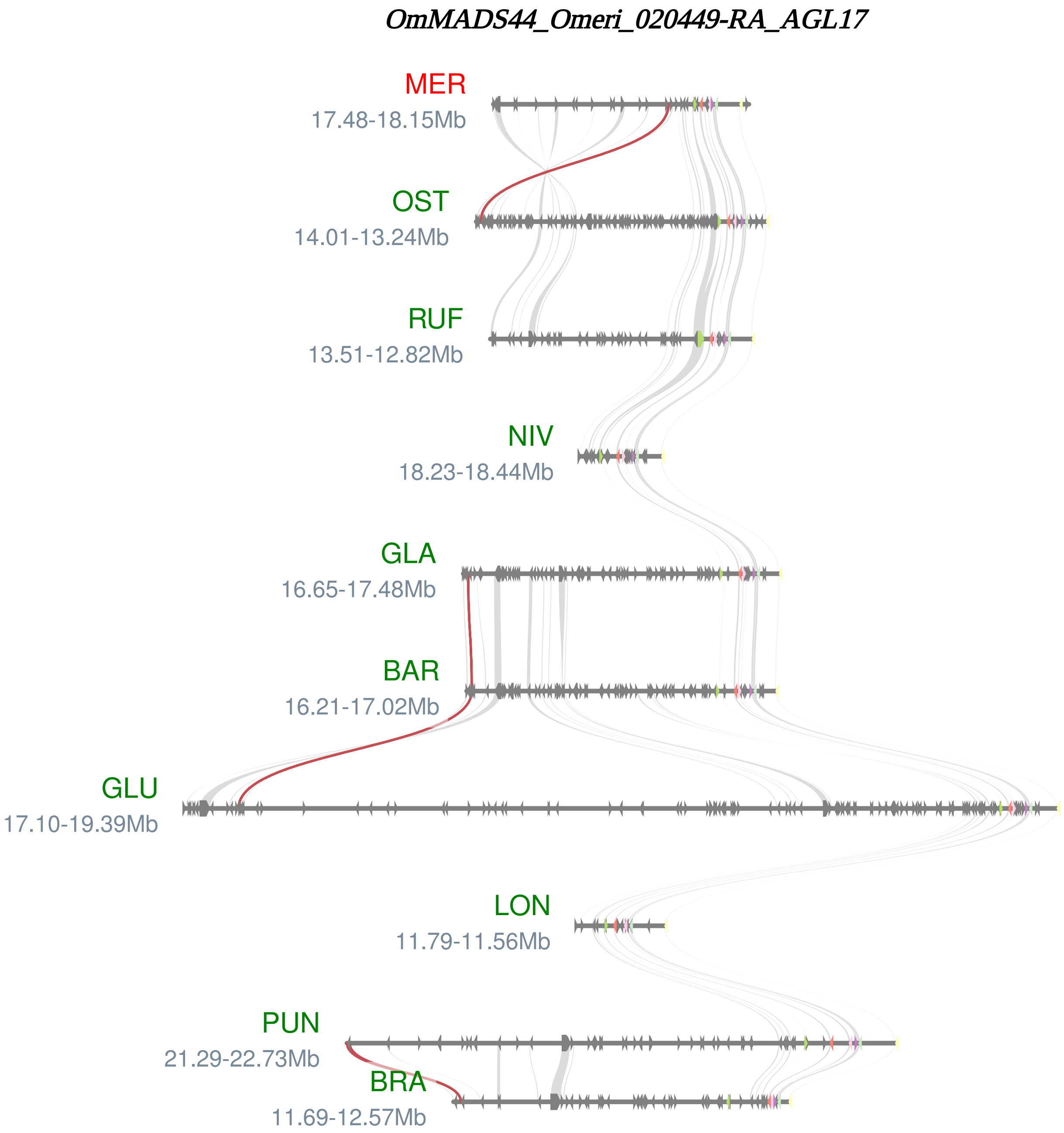
OmMADS44_Omeri_020449-RA_AGL17
MER
17.48-18.15Mb
OST
14.01-13.24Mb
RUF
13.51-12.82Mb
NIV
18.23-18.44Mb
GLA
16.65-17.48Mb
BAR
16.21-17.02Mb
GLU
17.10-19.39Mb
LON
11.79-11.56Mb
PUN
21.29-22.73Mb
BRA
11.69-12.57Mb

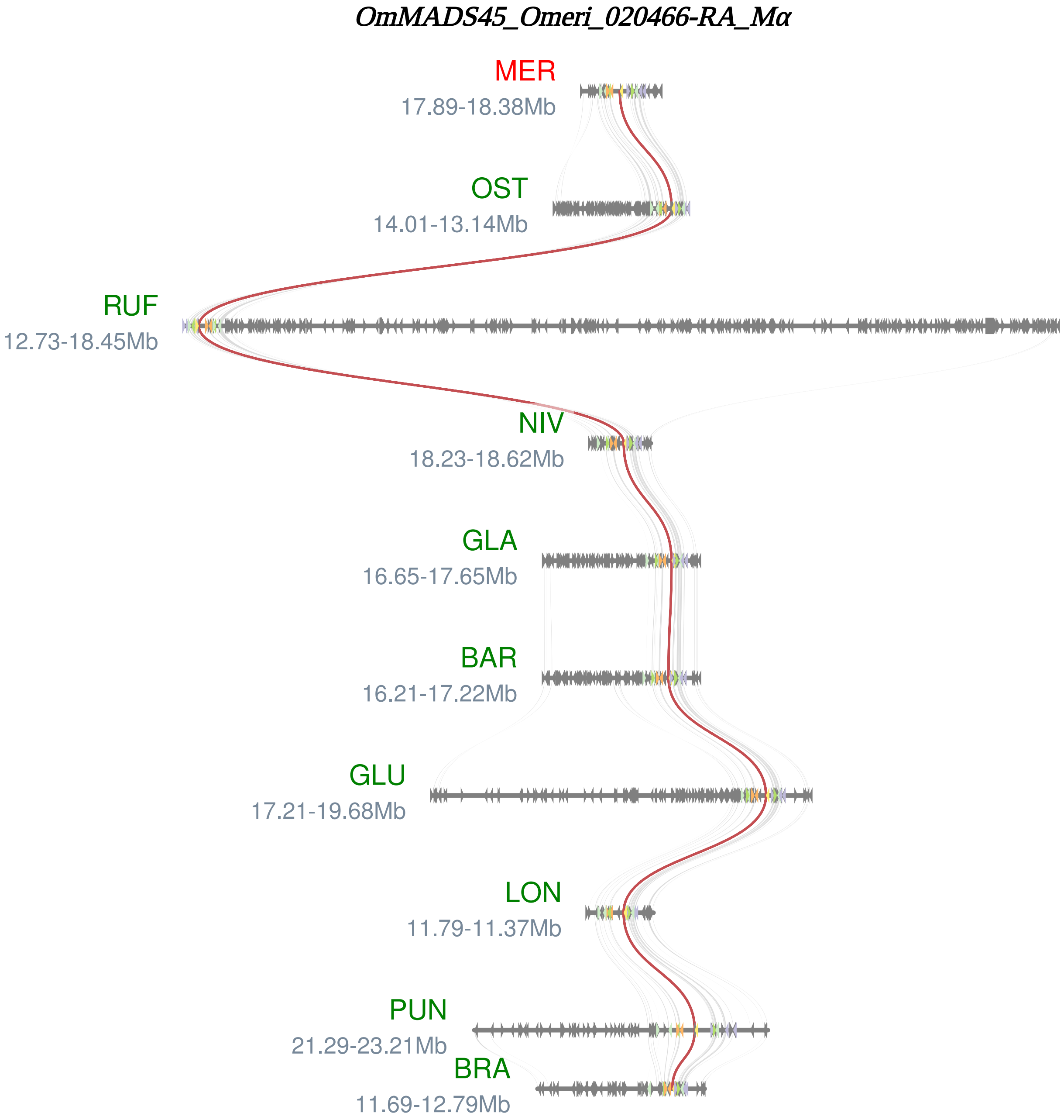

OmMADS45_Omeri_020466-RA_Mα
MER
17.89-18.38Mb
OST
14.01-13.14Mb
RUF
12.73-18.45Mb
NIV
18.23-18.62Mb
GLA
16.65-17.65Mb
BAR
16.21-17.22Mb
GLU
17.21-19.68Mb
LON
11.79-11.37Mb
PUN
21.29-23.21Mb
BRA
11.69-12.79Mb

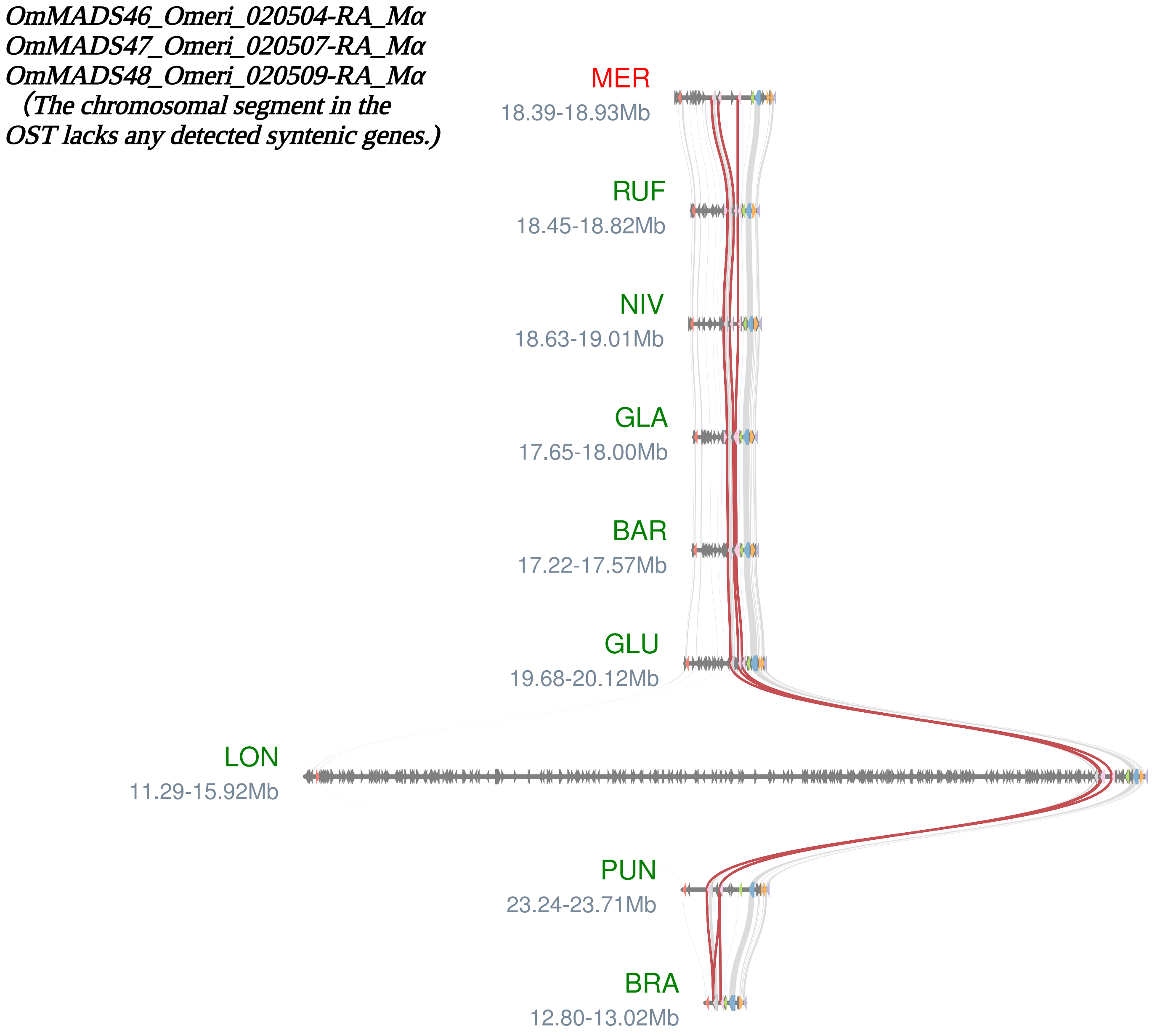
OmMADS46_Omeri_020504-RA_Mα
OmMADS47_Omeri_020507-RA_Mα
OmMADS48_Omeri_020509-RA_Mα
(The chromosomal segment in the OST lacks any detected syntenic genes.)
MER
18.39-18.93Mb
RUF
18.45-18.82Mb
NIV
18.63-19.01Mb
GLA
17.65-18.00Mb
BAR
17.22-17.57Mb
GLU
19.68-20.12Mb
LON
11.29-15.92Mb
PUN
23.24-23.71Mb
BRA
12.80-13.02Mb

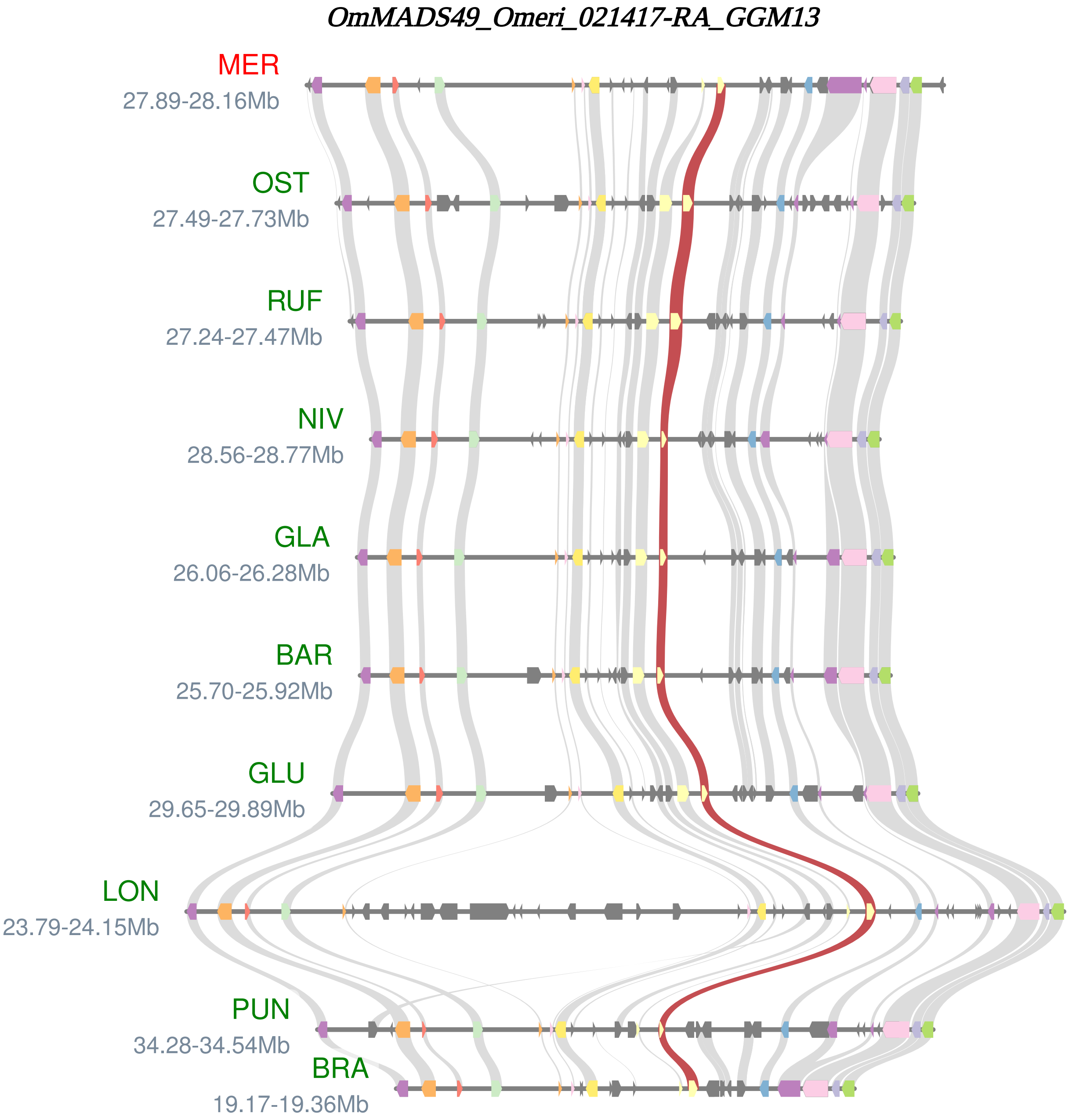

OmMADS49_Omeri_021417-RA_GGM13
MER
27.89-28.16Mb
OST
27.49-27.73Mb
RUF
27.24-27.47Mb
NIV
28.56-28.77Mb
GLA
26.06-26.28Mb
BAR
25.70-25.92Mb
GLU
29.65-29.89Mb
LON
23.79-24.15Mb
PUN
34.28-34.54Mb
BRA
19.17-19.36Mb

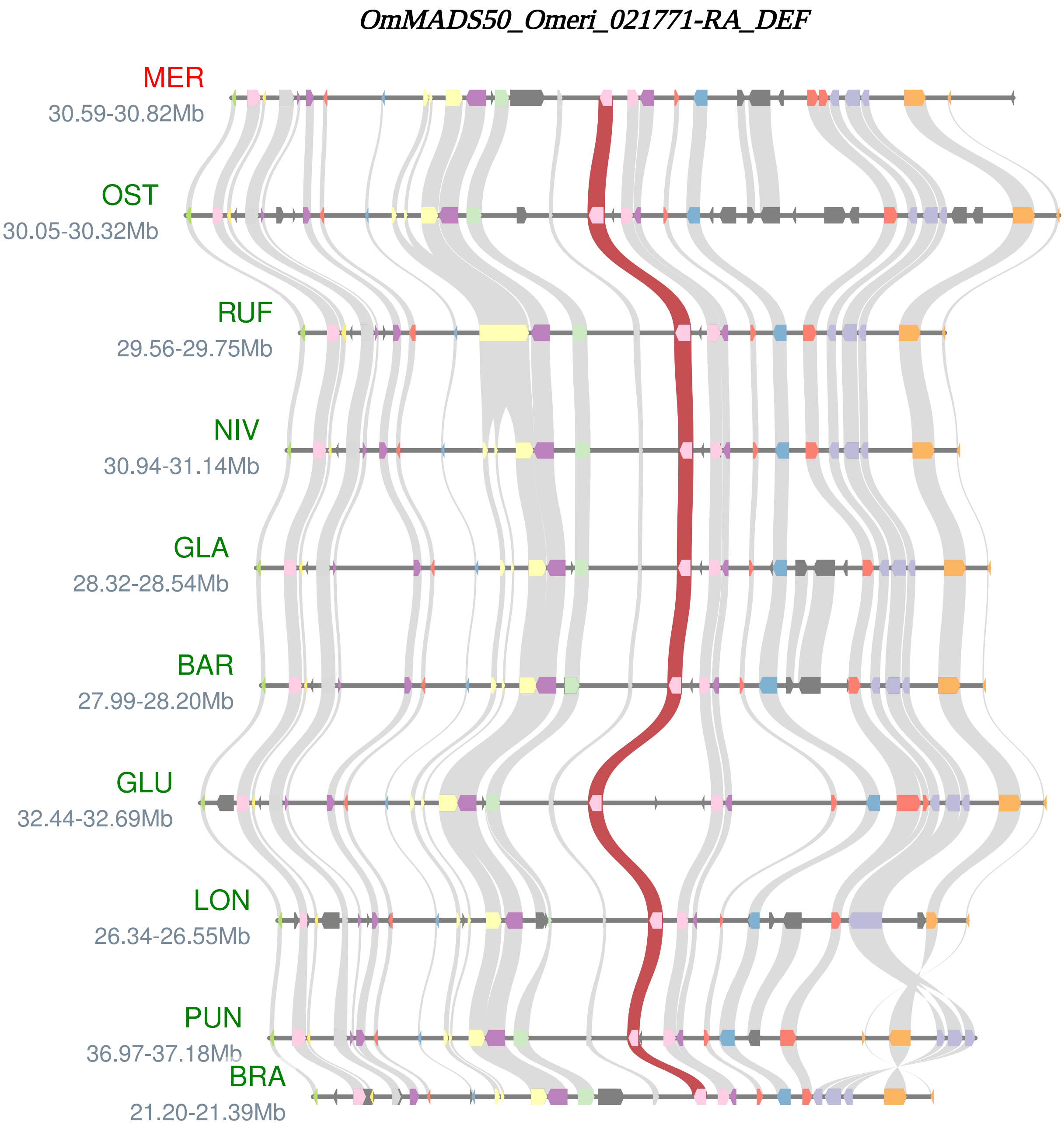
OmMADS50_Omeri_021771-RA_DEF
MER
30.59-30.82Mb
OST
30.05-30.32Mb
RUF
29.56-29.75Mb
NIV
30.94-31.14Mb
GLA
28.32-28.54Mb
BAR
27.99-28.20Mb
GLU
32.44-32.69Mb
LON
26.34-26.55Mb
PUN
36.97-37.18Mb
BRA
21.20-21.39Mb

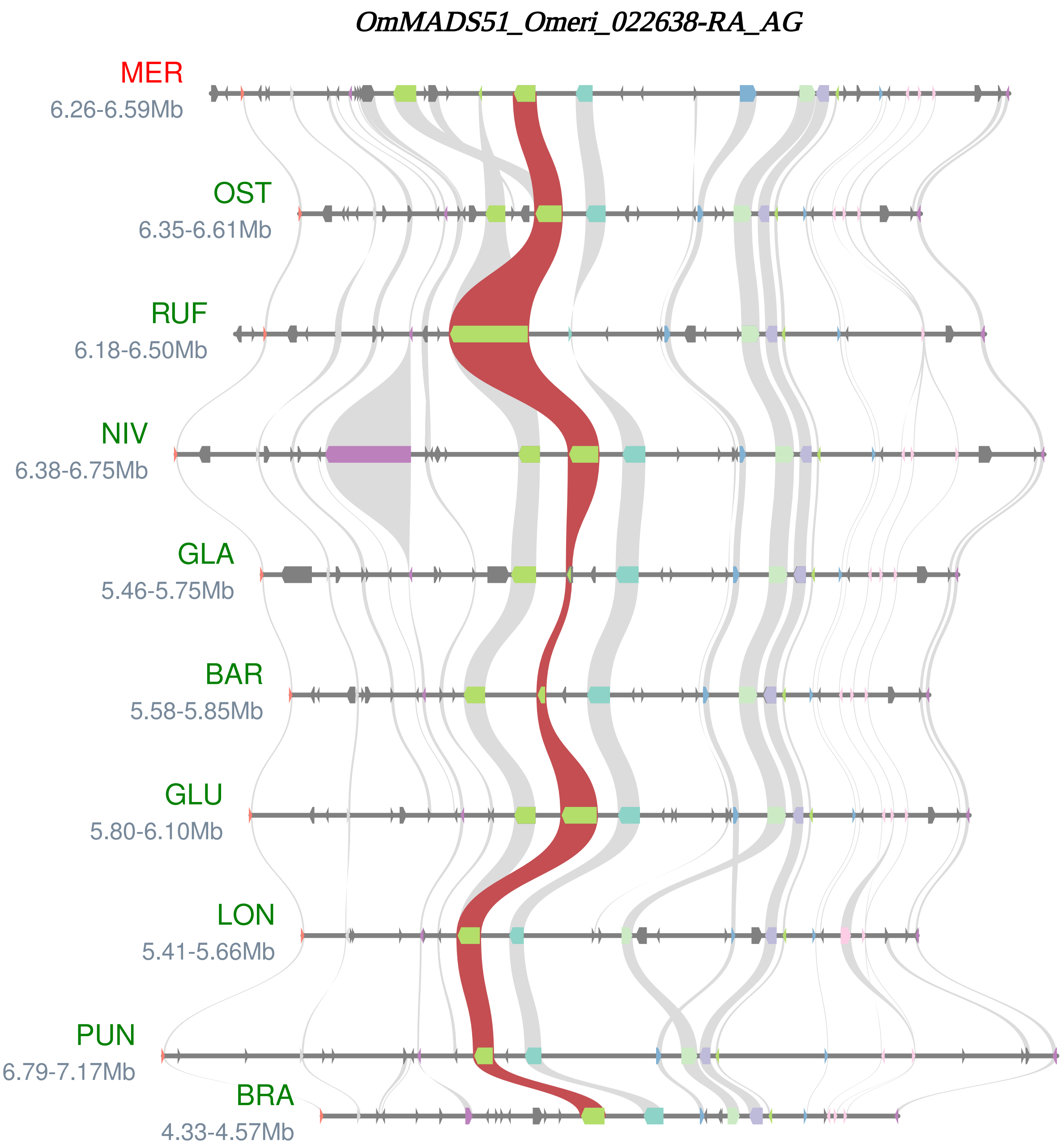

OmMADS51_Omeri_022638-RA_AG
MER
6.26-6.59Mb
OST
6.35-6.61Mb
RUF
6.18-6.50Mb
NIV
6.38-6.75Mb
GLA
5.46-5.75Mb
BAR
5.58-5.85Mb
GLU
5.80-6.10Mb
LON
5.41-5.66Mb
PUN
6.79-7.17Mb
BRA
4.33-4.57Mb

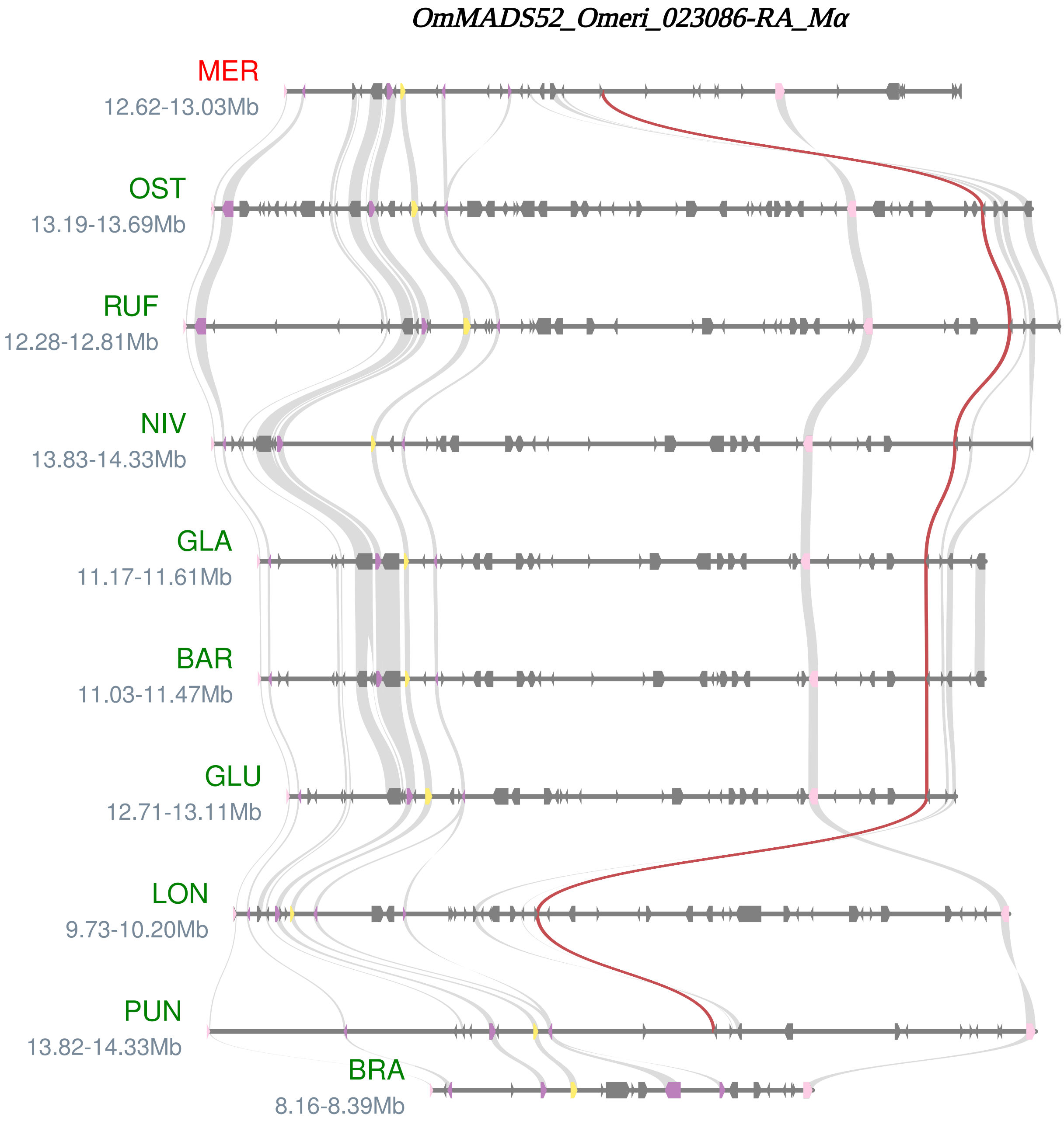
OmMADS52_Omeri_023086-RA_Mα
MER
12.62-13.03Mb
OST
13.19-13.69Mb
RUF
12.28-12.81Mb
NIV
13.83-14.33Mb
GLA
11.17-11.61Mb
BAR
11.03-11.47Mb
GLU
12.71-13.11Mb
LON
9.73-10.20Mb
PUN
13.82-14.33Mb
BRA
8.16-8.39Mb

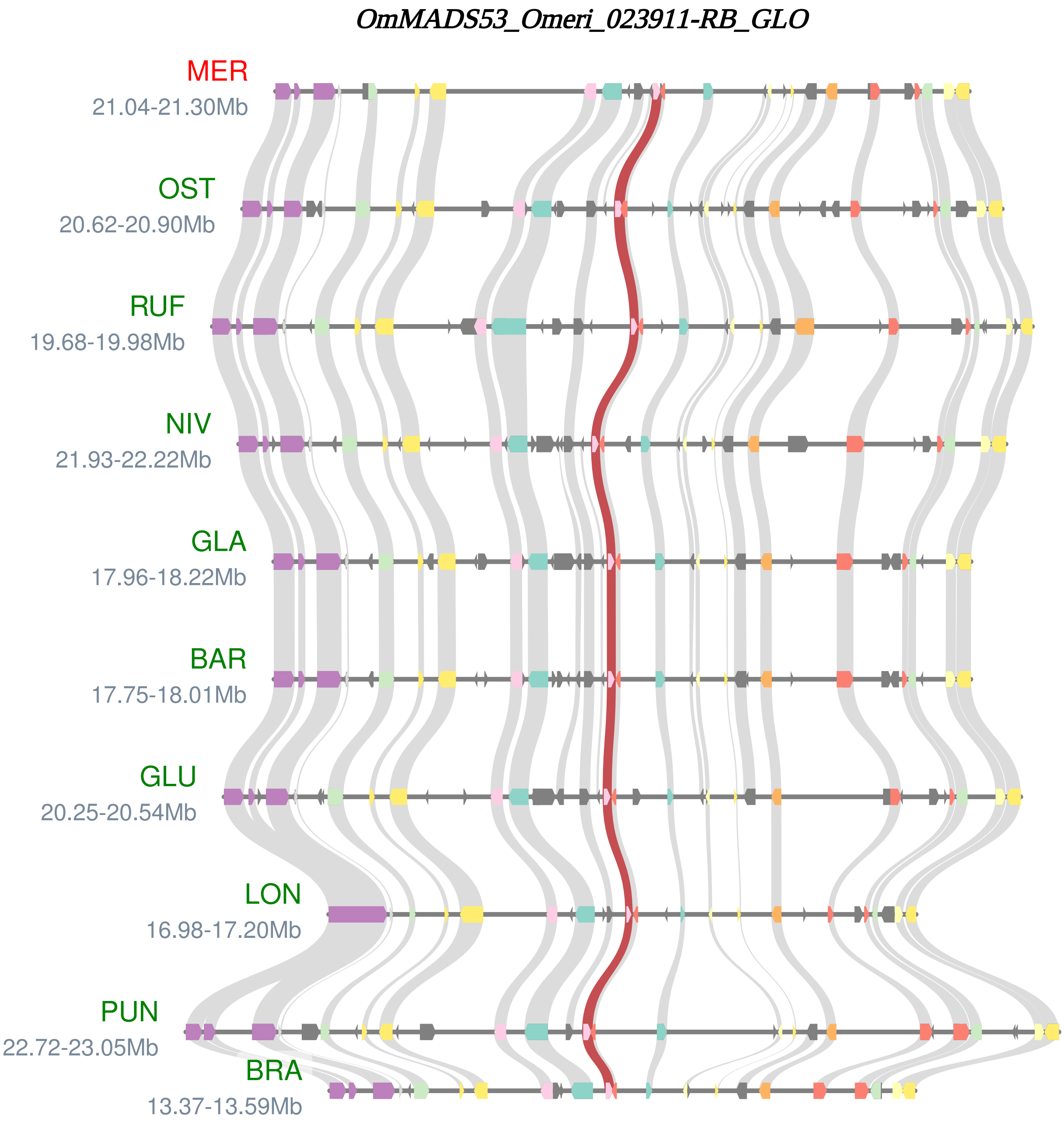
OmMADS53_Omeri_023911-RB_GLO
MER
21.04-21.30Mb
OST
20.62-20.90Mb
RUF
19.68-19.98Mb
NIV
21.93-22.22Mb
GLA
17.96-18.22Mb
BAR
17.75-18.01Mb
GLU
20.25-20.54Mb
LON
16.98-17.20Mb
PUN
22.72-23.05Mb
BRA
13.37-13.59Mb

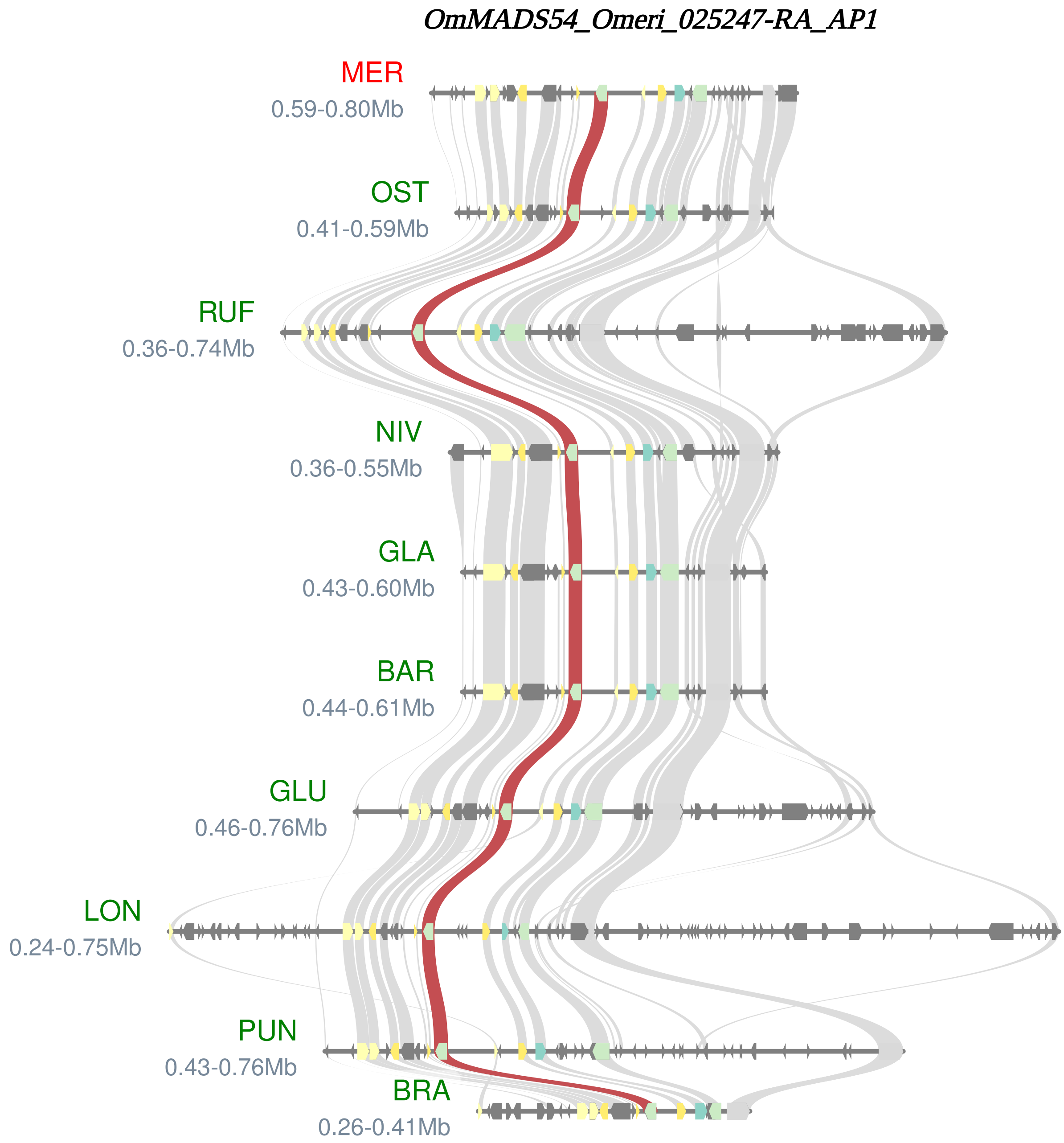

OmMADS54_Omeri_025247-RA_AP1
MER
0.59-0.80Mb
OST
0.41-0.59Mb
RUF
0.36-0.74Mb
NIV
0.36-0.55Mb
GLA
0.43-0.60Mb
BAR
0.44-0.61Mb
GLU
0.46-0.76Mb
LON
0.24-0.75Mb
PUN
0.43-0.76Mb
BRA
0.26-0.41Mb

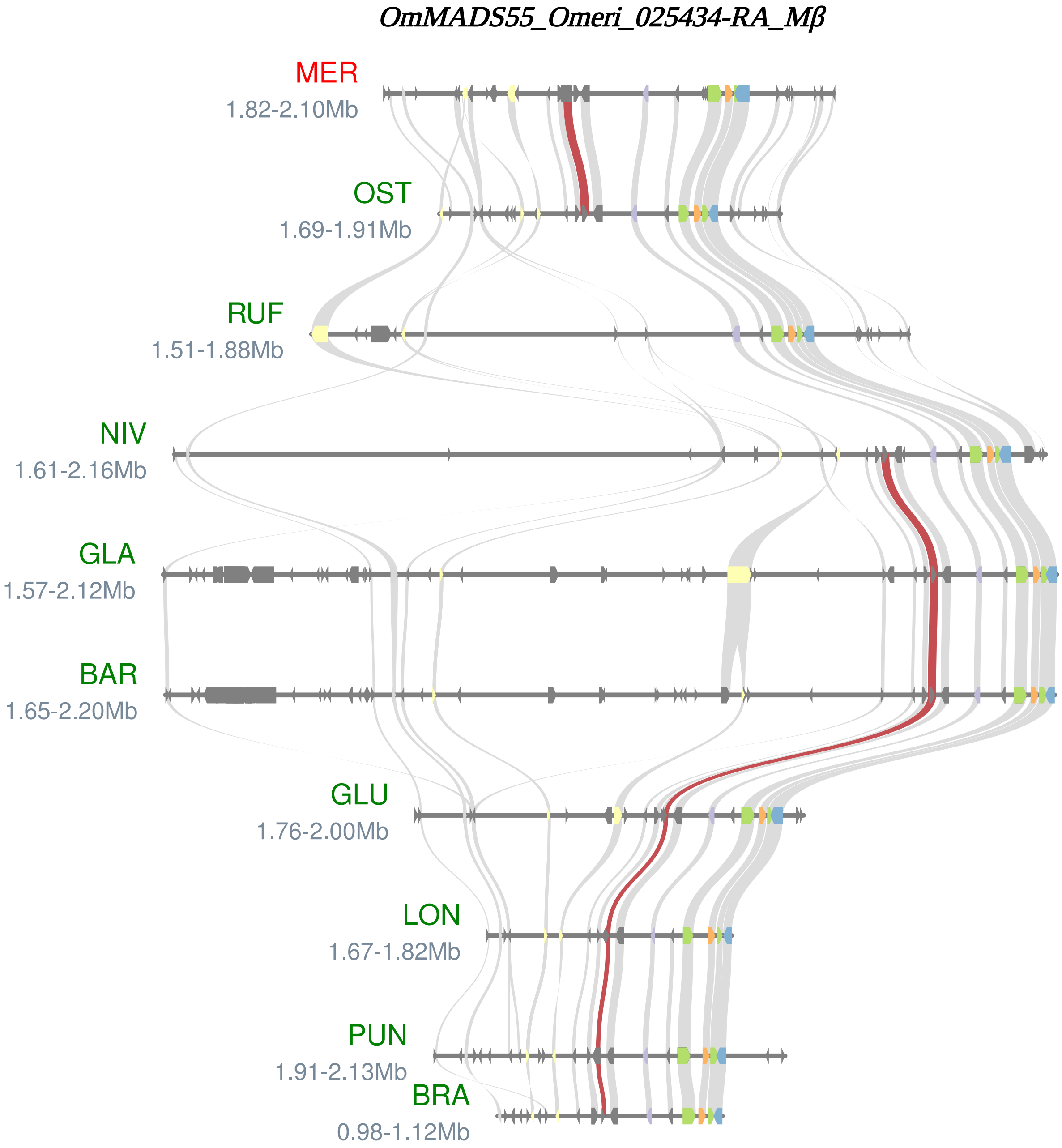
OmMADS55_Omeri_025434-RA_Mβ
MER
1.82-2.10Mb
OST
1.69-1.91Mb
RUF
1.51-1.88Mb
NIV
1.61-2.16Mb
GLA
1.57-2.12Mb
BAR
1.65-2.20Mb
GLU
1.76-2.00Mb
LON
1.67-1.82Mb
PUN
1.91-2.13Mb
BRA
0.98-1.12Mb

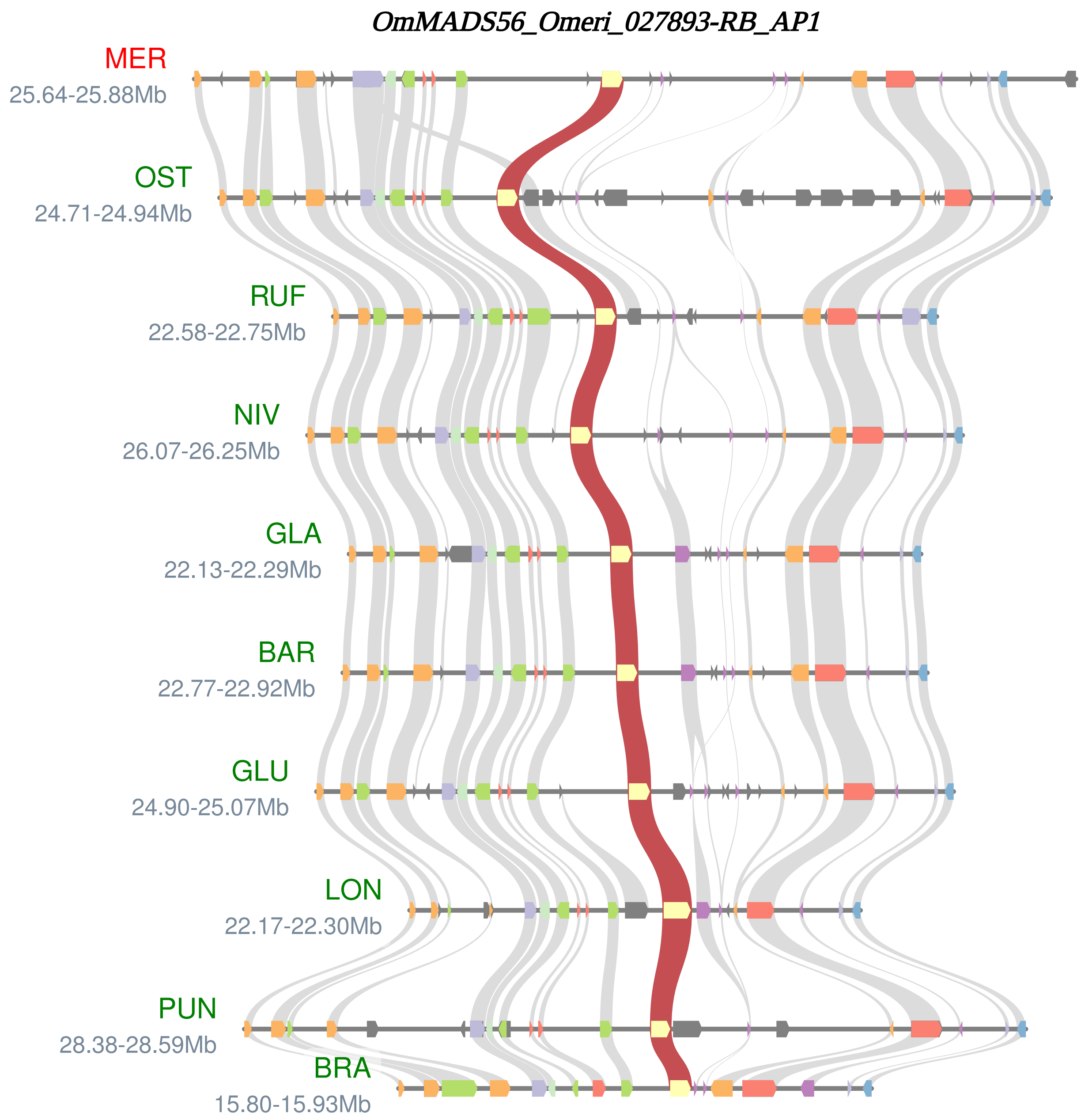
OmMADS56_Omeri_027893-RB_AP1
MER
25.64-25.88Mb
OST
24.71-24.94Mb
RUF
22.58-22.75Mb
NIV
26.07-26.25Mb
GLA
22.13-22.29Mb
BAR
22.77-22.92Mb
GLU
24.90-25.07Mb
LON
22.17-22.30Mb
PUN
28.38-28.59Mb
BRA
15.80-15.93Mb

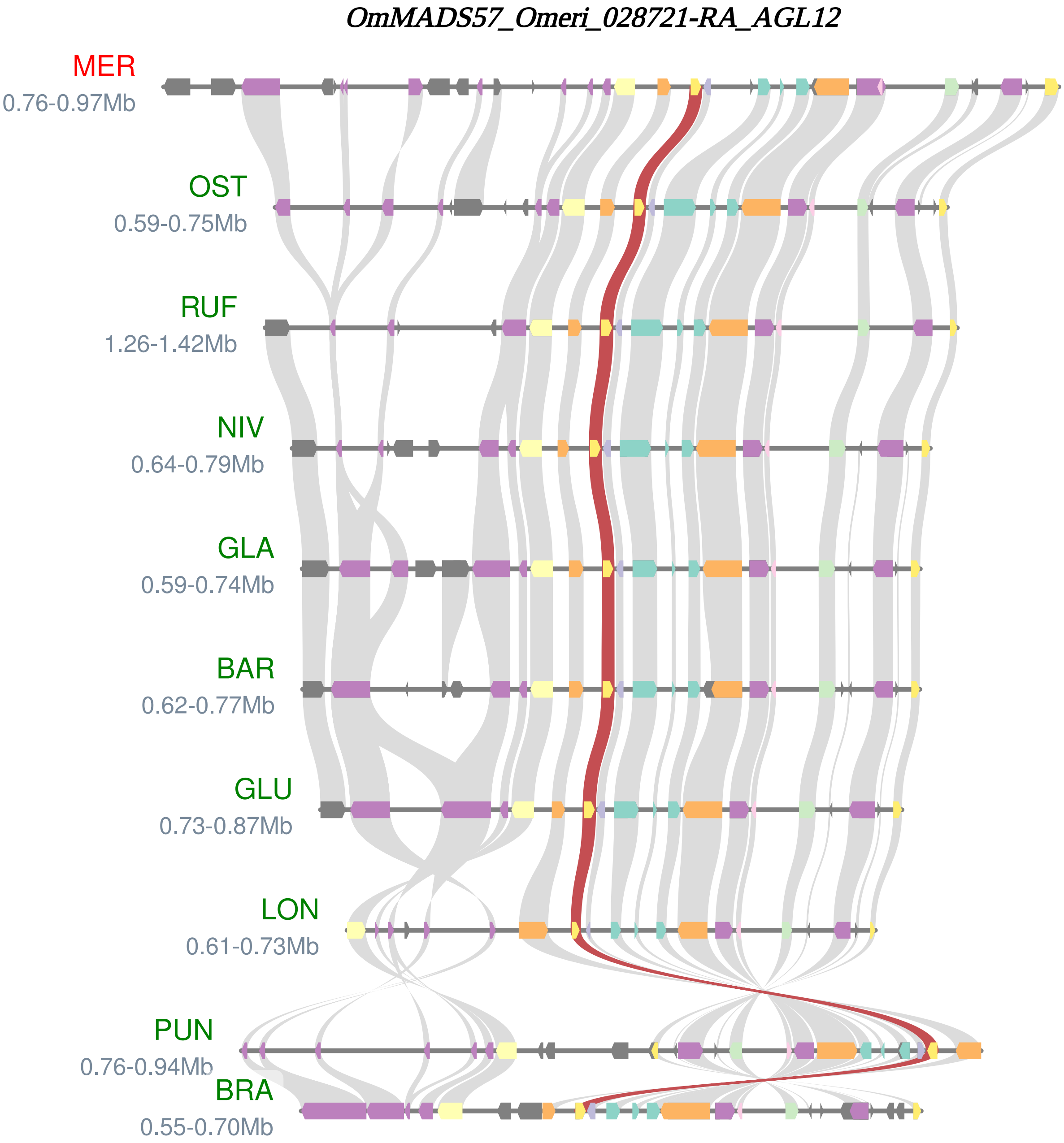

OmMADS57_Omeri_028721-RA_AGL12
MER
0.76-0.97Mb
OST
0.59-0.75Mb
RUF
1.26-1.42Mb
NIV
0.64-0.79Mb
GLA
0.59-0.74Mb
BAR
0.62-0.77Mb
GLU
0.73-0.87Mb
LON
0.61-0.73Mb
PUN
0.76-0.94Mb
BRA
0.55-0.70Mb

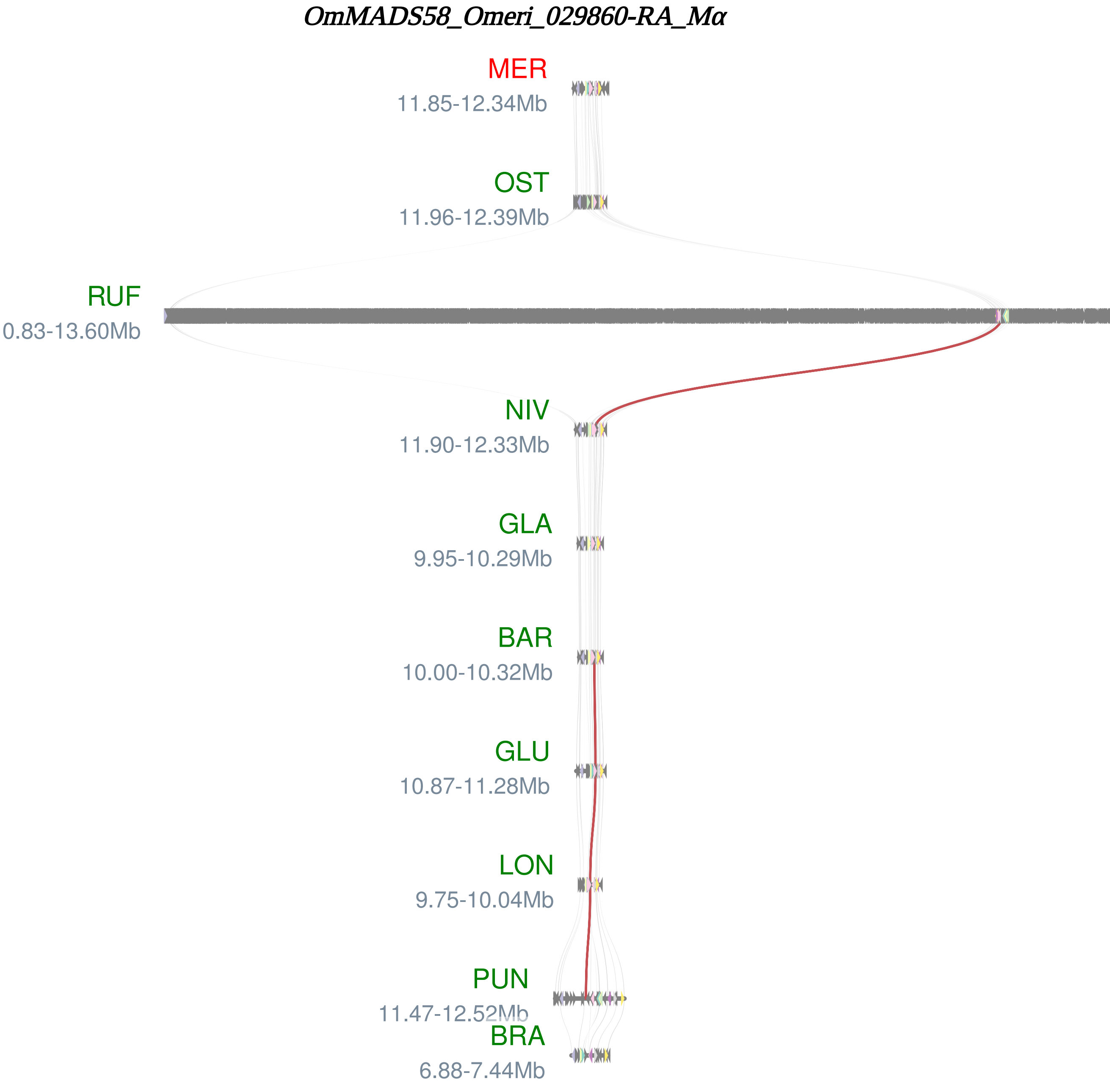
OmMADS58_Omeri_029860-RA_Mα
MER
11.85-12.34Mb
OST
11.96-12.39Mb
RUF
0.83-13.60Mb
NIV
11.90-12.33Mb
GLA
9.95-10.29Mb
BAR
10.00-10.32Mb
GLU
10.87-11.28Mb
LON
9.75-10.04Mb
PUN
11.47-12.52Mb
BRA
6.88-7.44Mb

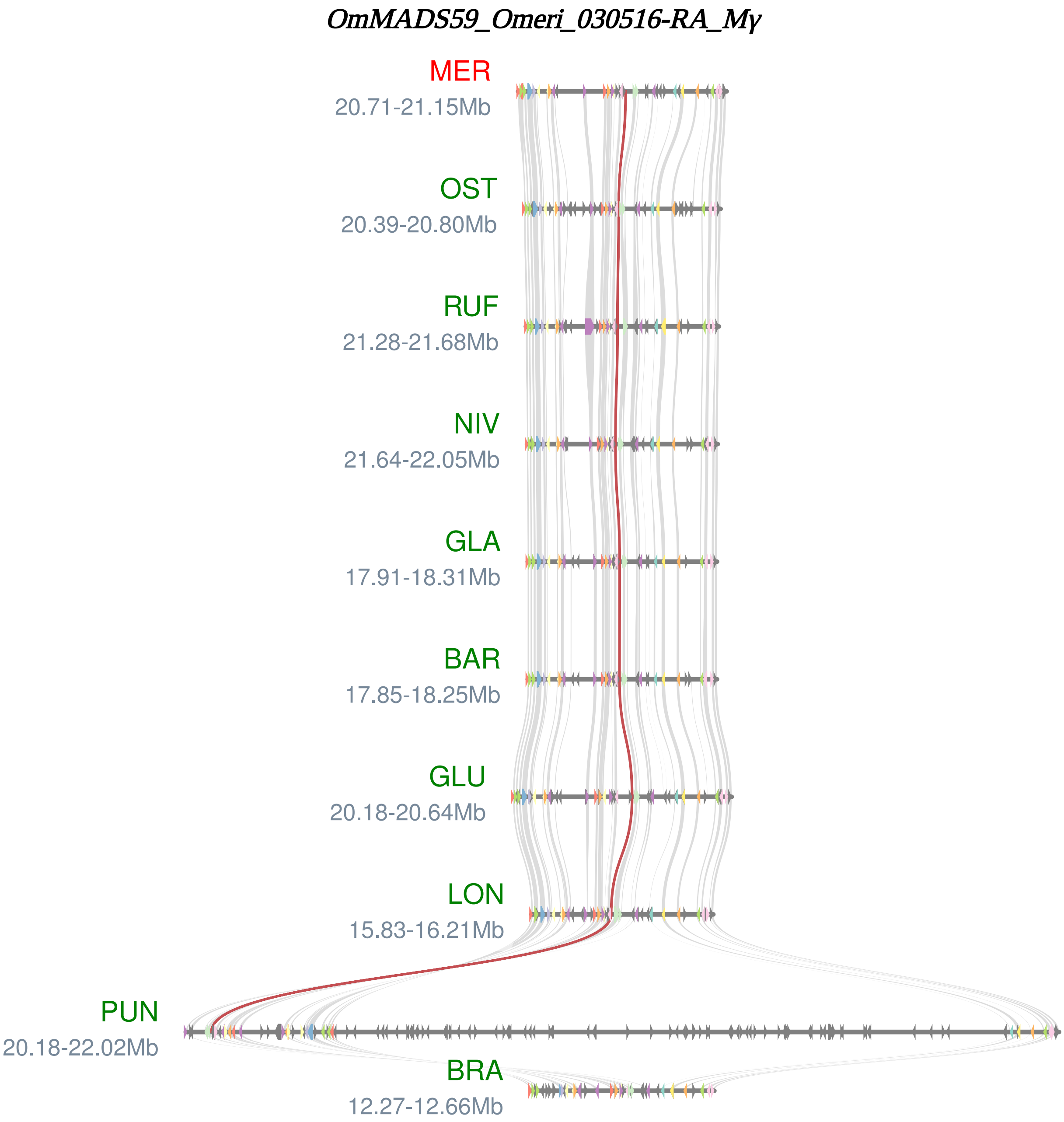
OmMADS59_Omeri_030516-RA_Mγ
MER
20.71-21.15Mb
OST
20.39-20.80Mb
RUF
21.28-21.68Mb
NIV
21.64-22.05Mb
GLA
17.91-18.31Mb
BAR
17.85-18.25Mb
GLU
20.18-20.64Mb
LON
15.83-16.21Mb
PUN
20.18-22.02Mb
BRA
12.27-12.66Mb

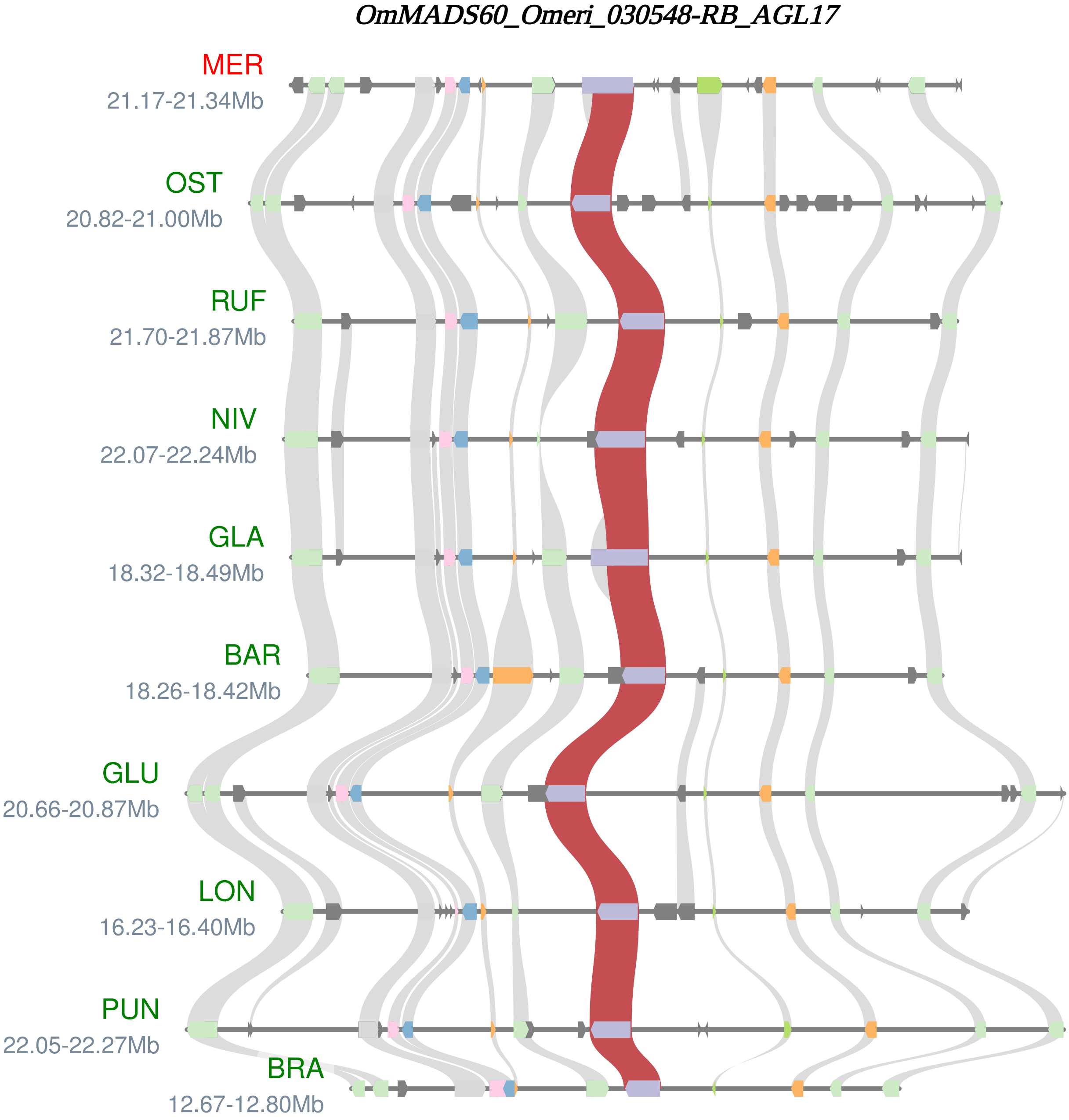
OmMADS60_Omeri_030548-RB_AGL17
MER
21.17-21.34Mb
OST
20.82-21.00Mb
RUF
21.70-21.87Mb
NIV
22.07-22.24Mb
GLA
18.32-18.49Mb
BAR
18.26-18.42Mb
GLU
20.66-20.87Mb
LON
16.23-16.40Mb
PUN
22.05-22.27Mb
BRA
12.67-12.80Mb

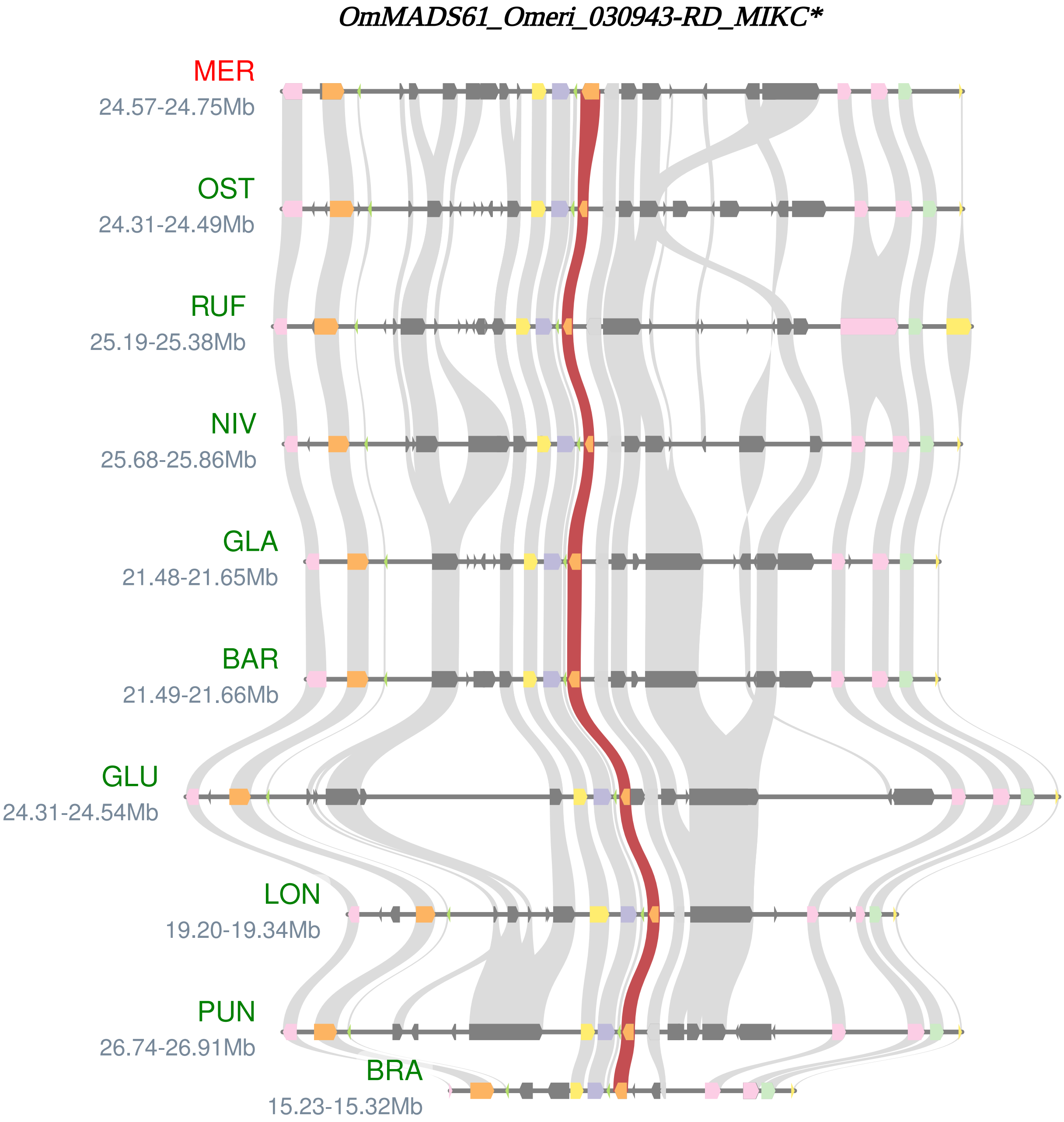
OmMADS61_Omeri_030943-RD_MIKC*
MER
24.57-24.75Mb
OST
24.31-24.49Mb
RUF
25.19-25.38Mb
NIV
25.68-25.86Mb
GLA
21.48-21.65Mb
BAR
21.49-21.66Mb
GLU
24.31-24.54Mb
LON
19.20-19.34Mb
PUN
26.74-26.91Mb
BRA
15.23-15.32Mb

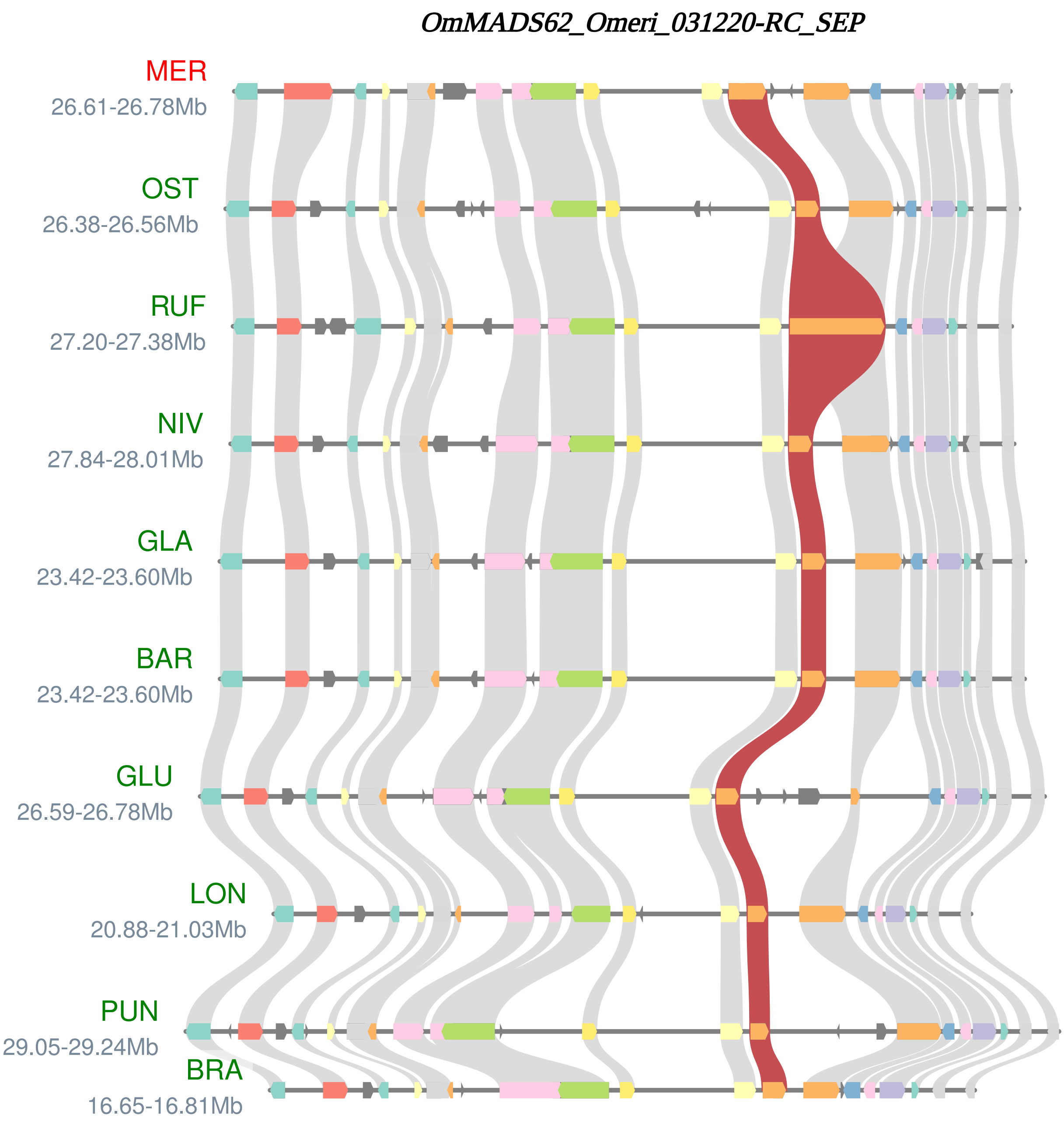
OmMADS62_Omeri_031220-RC_SEP
MER
26.61-26.78Mb
OST
26.38-26.56Mb
RUF
27.20-27.38Mb
NIV
27.84-28.01Mb
GLA
23.42-23.60Mb
BAR
23.42-23.60Mb
GLU
26.59-26.78Mb
LON
20.88-21.03Mb
PUN
29.05-29.24Mb
BRA
16.65-16.81Mb

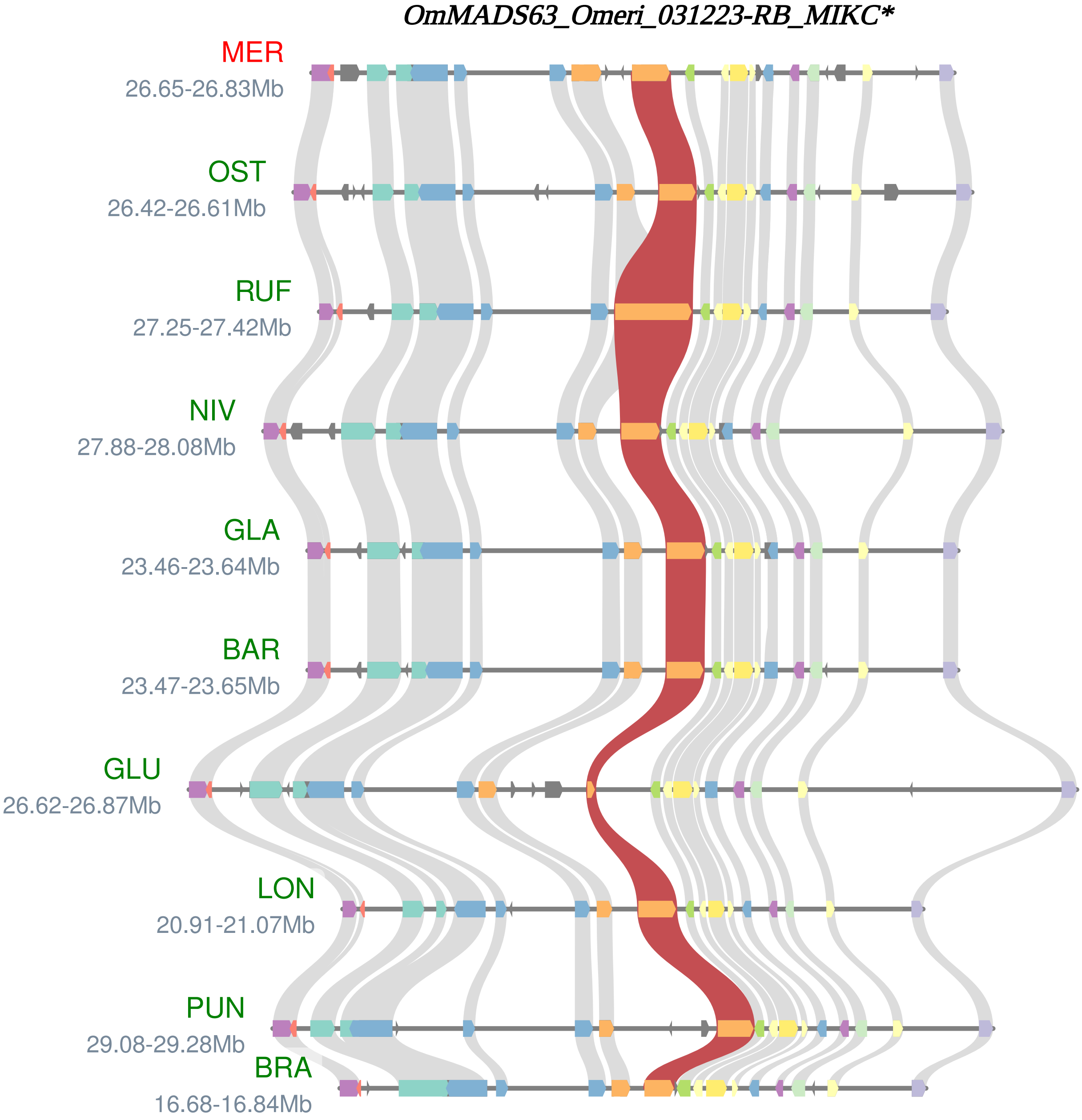
OmMADS63_Omeri_031223-RB_MIKC*
MER
26.65-26.83Mb
OST
26.42-26.61Mb
RUF
27.25-27.42Mb
NIV
27.88-28.08Mb
GLA
23.46-23.64Mb
BAR
23.47-23.65Mb
GLU
26.62-26.87Mb
LON
20.91-21.07Mb
PUN
29.08-29.28Mb
BRA
16.68-16.84Mb

OmMADS64_Omeri_034099-RB_MIKC
(The chromosomal segment in the LON lacks any detected syntenic genes.)

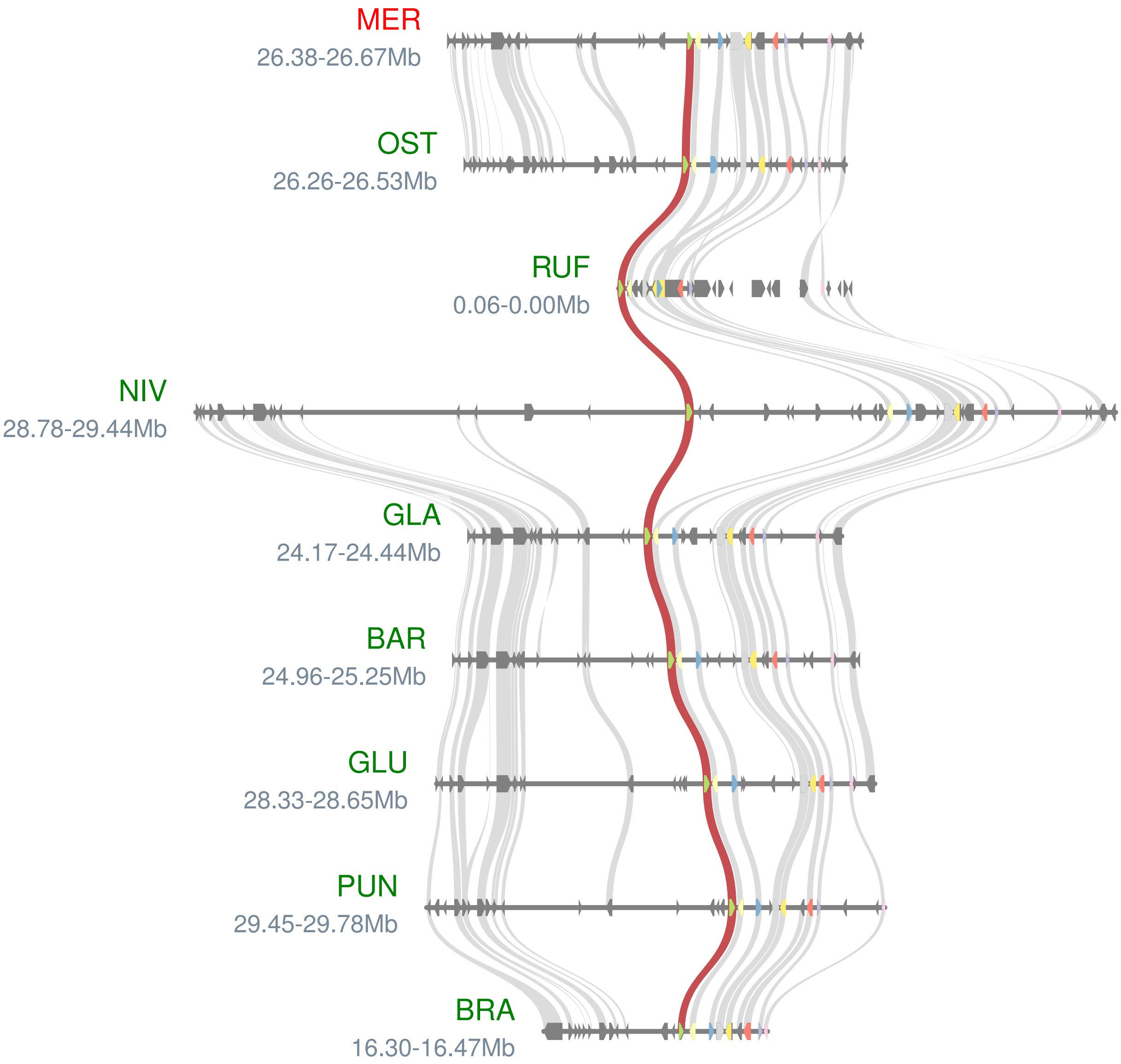

OmMADS65_Omeri_035119-RA_AGL12
(The chromosomal segment in the OST lacks any detected syntenic genes.)

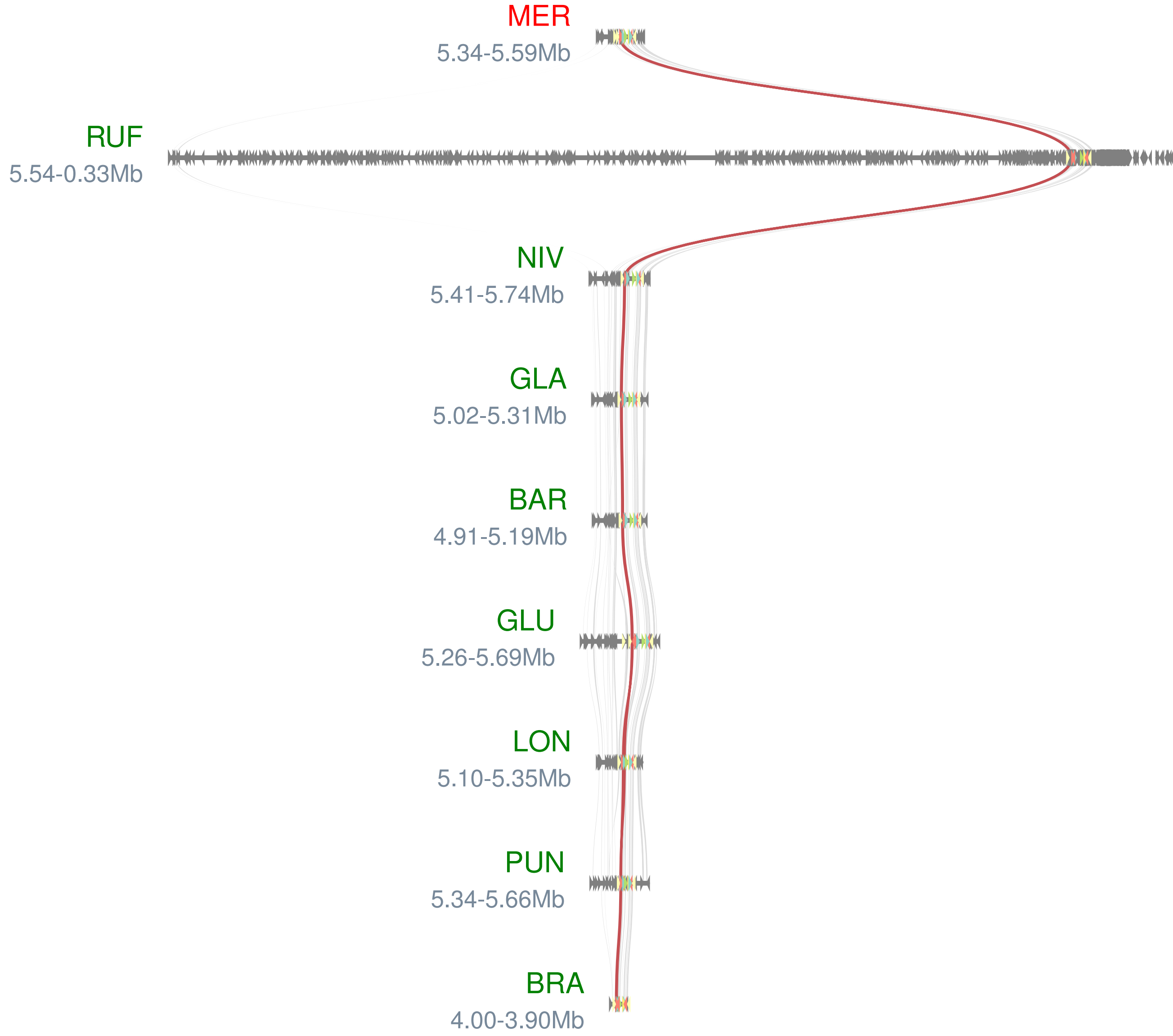

OmMADS66_Omeri_035120-RB_AG

(The chromosomal segment in the OST lacks any detected syntenic genes.)

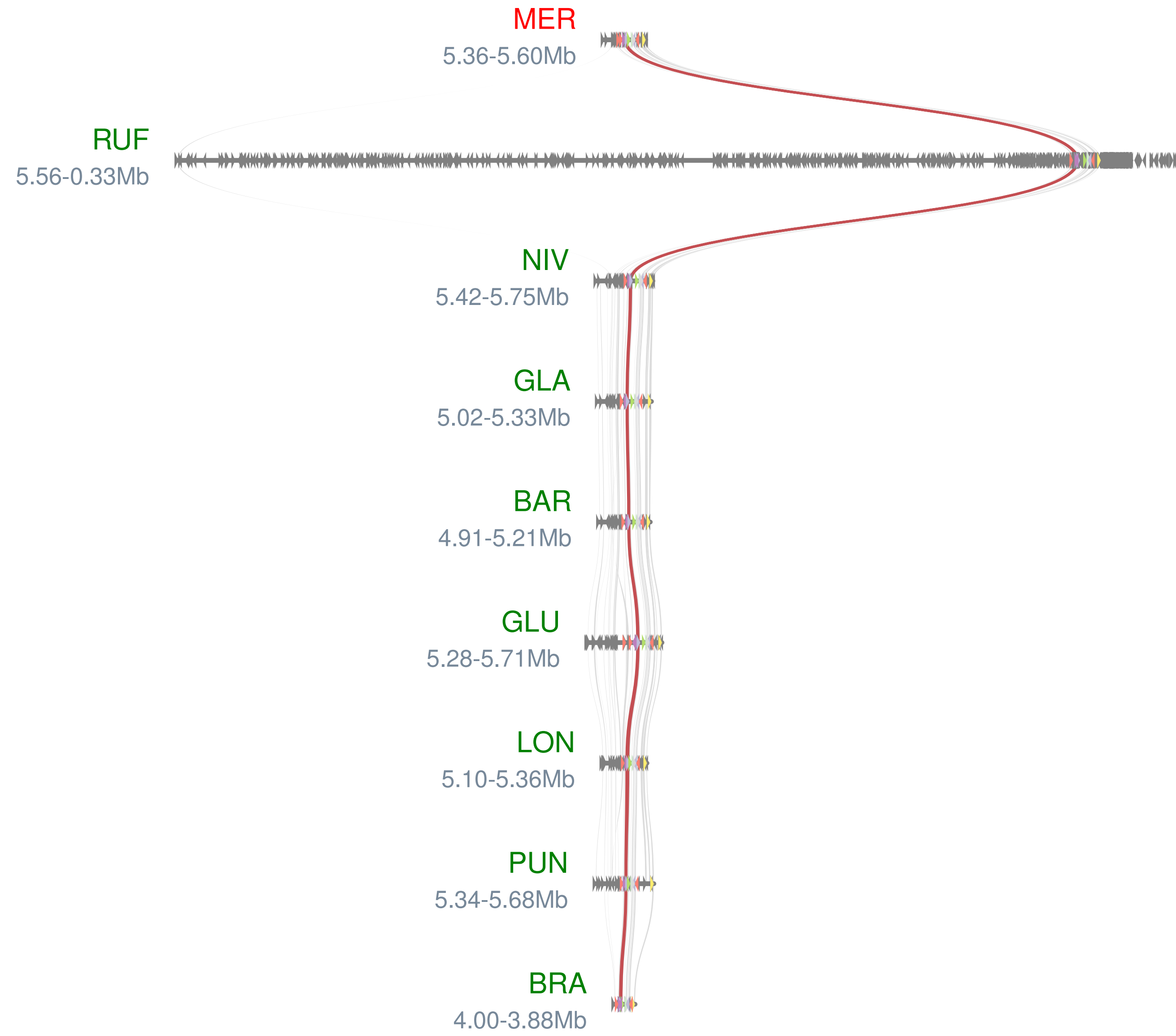

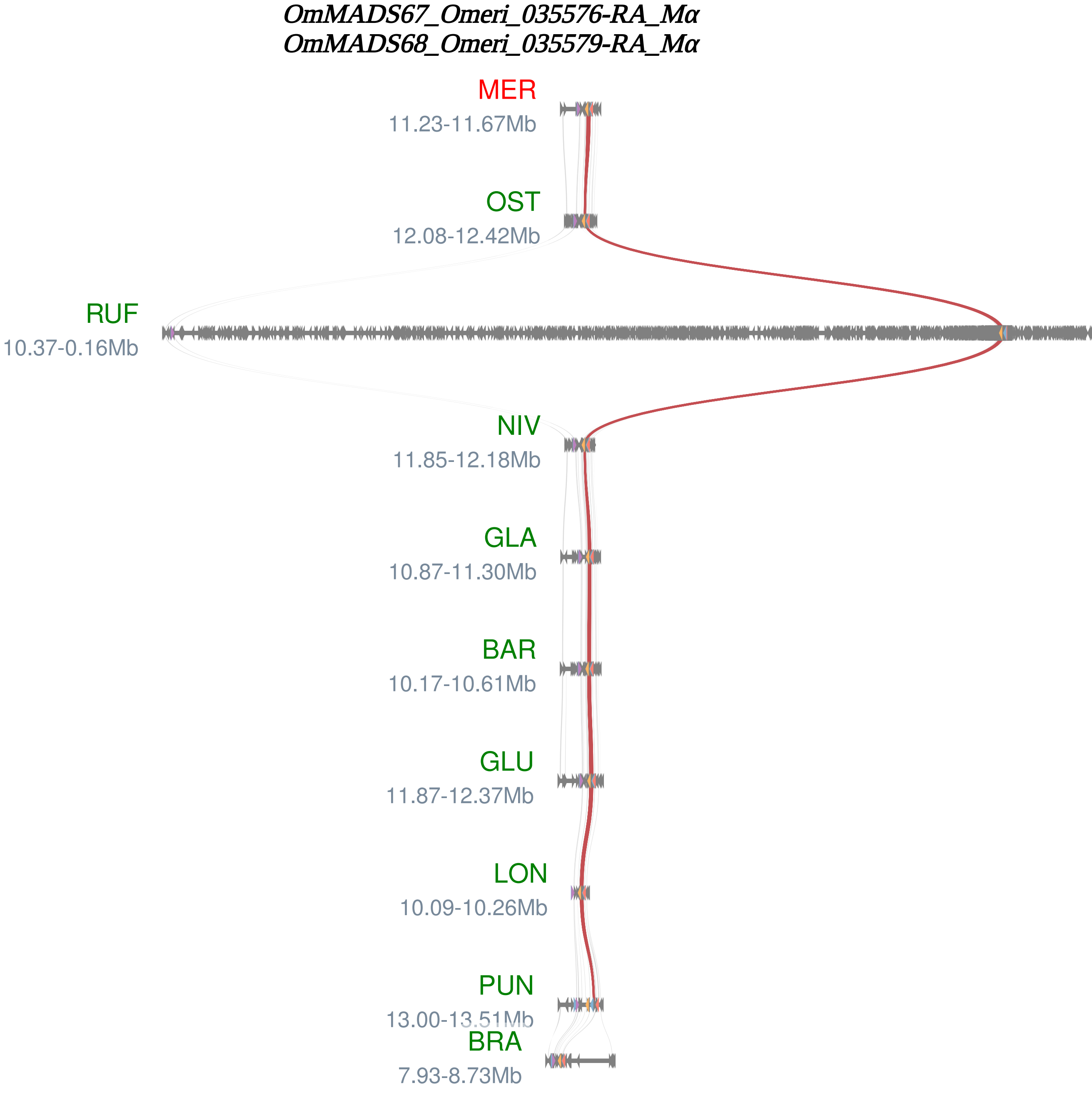
OmMADS67_Omeri_035576-RA_Mα
OmMADS68_Omeri_035579-RA_Mα
MER
11.23-11.67Mb
OST
12.08-12.42Mb
RUF
10.37-0.16Mb
NIV
11.85-12.18Mb
GLA
10.87-11.30Mb
BAR
10.17-10.61Mb
GLU
11.87-12.37Mb
LON
10.09-10.26Mb
PUN
13.00-13.51Mb
BRA
7.93-8.73Mb

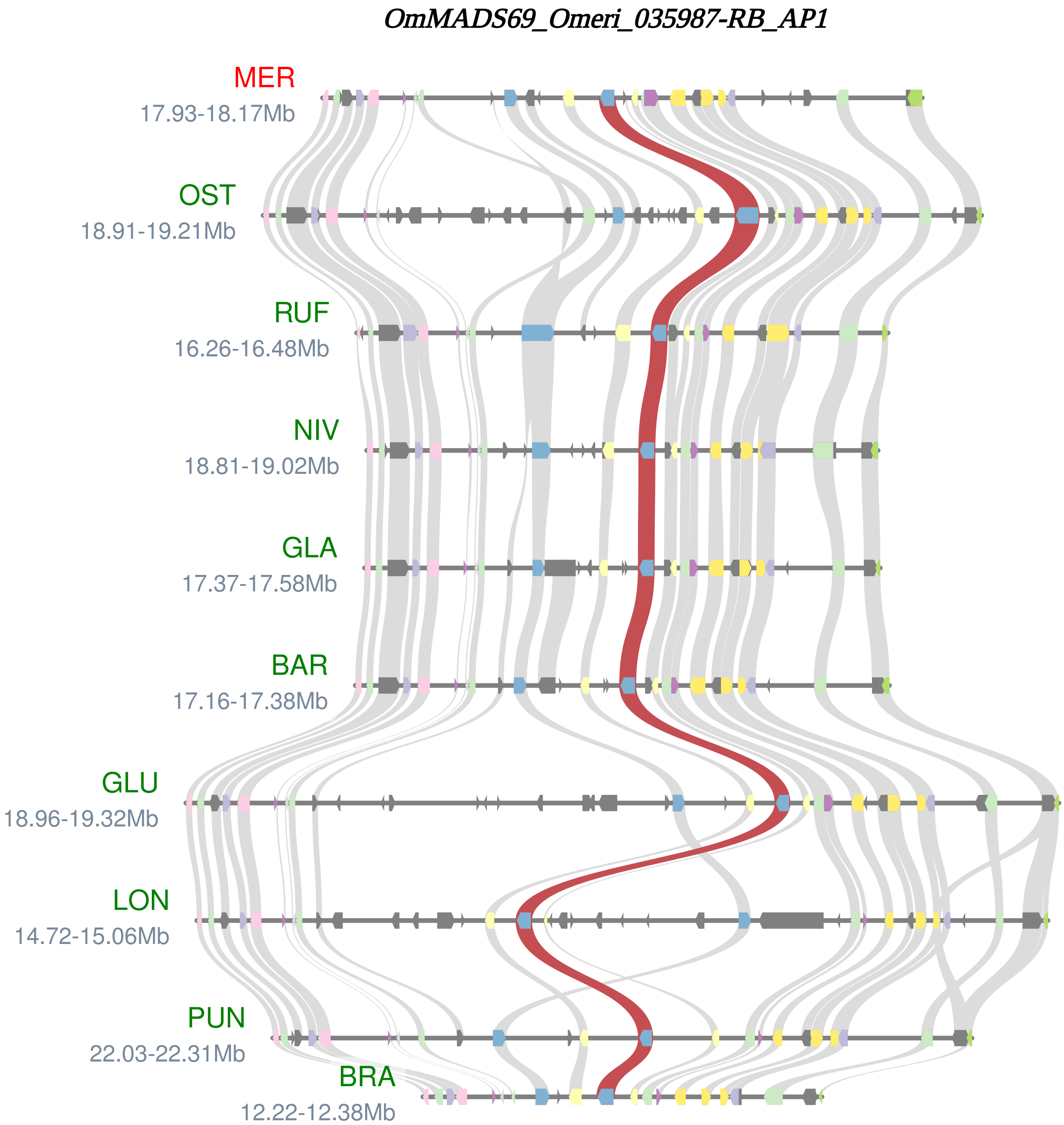

OmMADS69_Omeri_035987-RB_AP1
MER
17.93-18.17Mb
OST
18.91-19.21Mb
RUF
16.26-16.48Mb
NIV
18.81-19.02Mb
GLA
17.37-17.58Mb
BAR
17.16-17.38Mb
GLU
18.96-19.32Mb
LON
14.72-15.06Mb
PUN
22.03-22.31Mb
BRA
12.22-12.38Mb

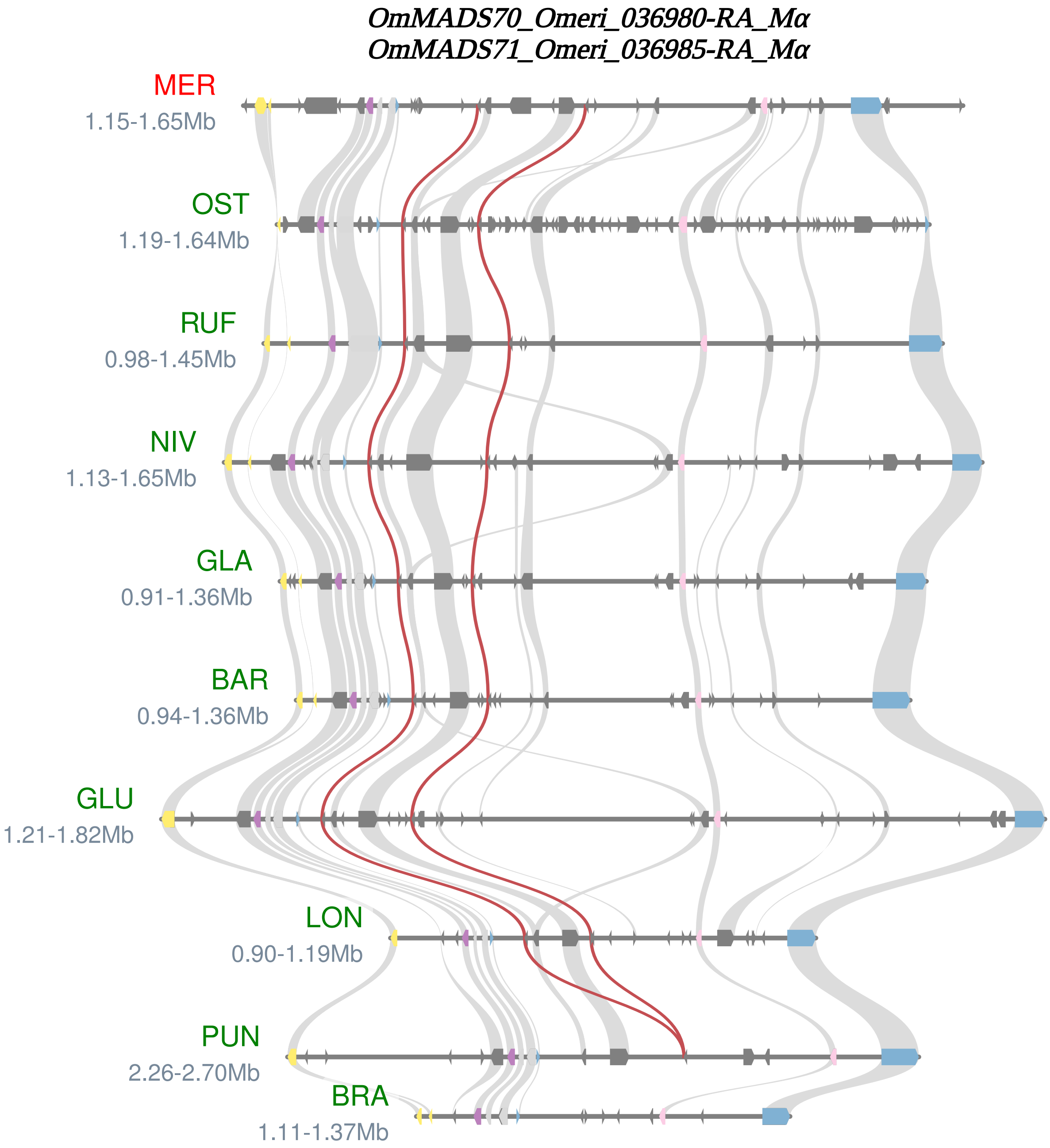

OmMADS70_Omeri_036980-RA_Mα
OmMADS71_Omeri_036985-RA_Mα
MER
1.15-1.65Mb
OST
1.19-1.64Mb
RUF
0.98-1.45Mb
NIV
1.13-1.65Mb
GLA
0.91-1.36Mb
BAR
0.94-1.36Mb
GLU
1.21-1.82Mb
LON
0.90-1.19Mb
PUN
2.26-2.70Mb
BRA
1.11-1.37Mb

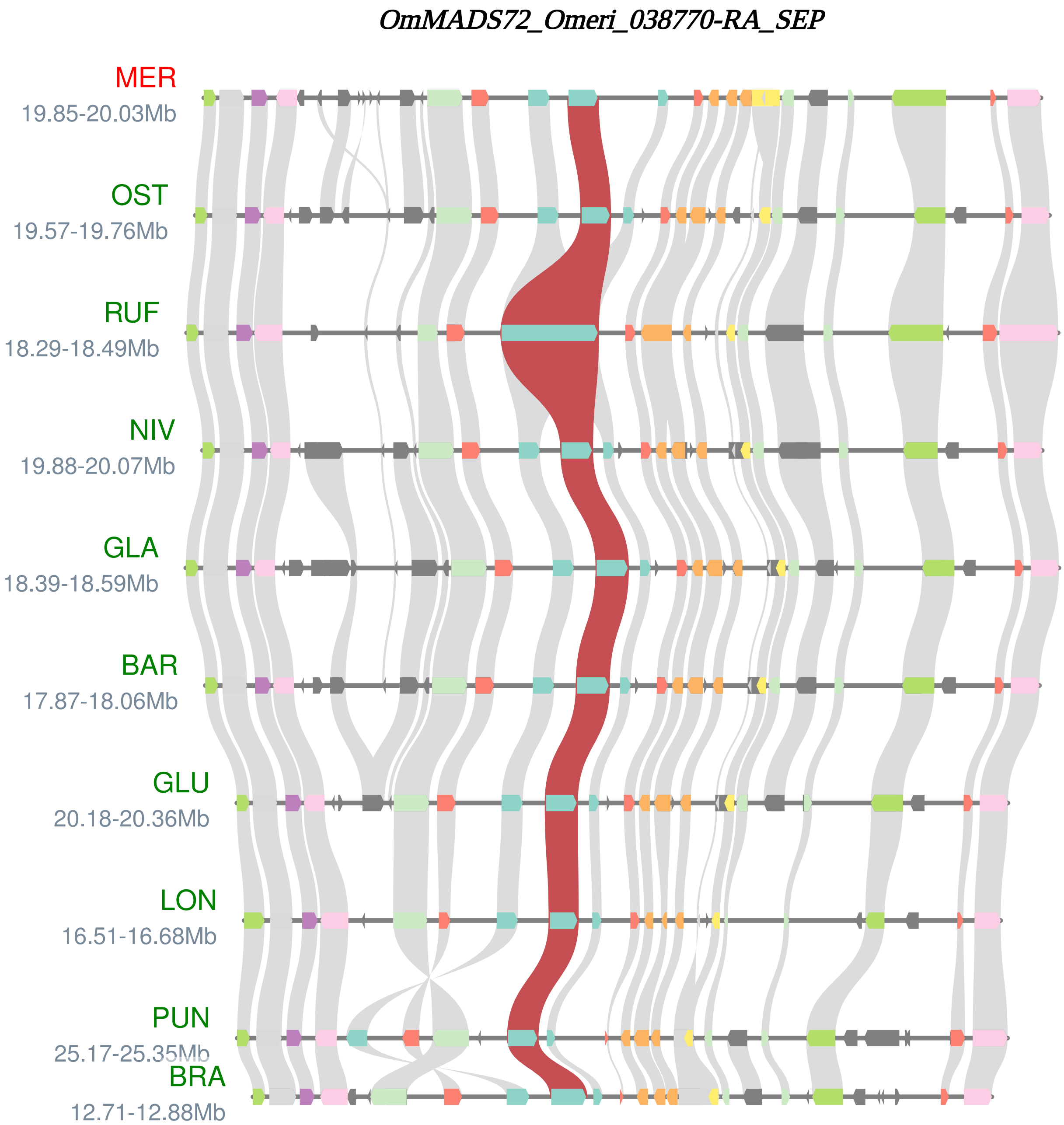
OmMADS72_Omeri_038770-RA_SEP
MER
19.85-20.03Mb
OST
19.57-19.76Mb
RUF
18.29-18.49Mb
NIV
19.88-20.07Mb
GLA
18.39-18.59Mb
BAR
17.87-18.06Mb
GLU
20.18-20.36Mb
LON
16.51-16.68Mb
PUN
25.17-25.35Mb
BRA
12.71-12.88Mb

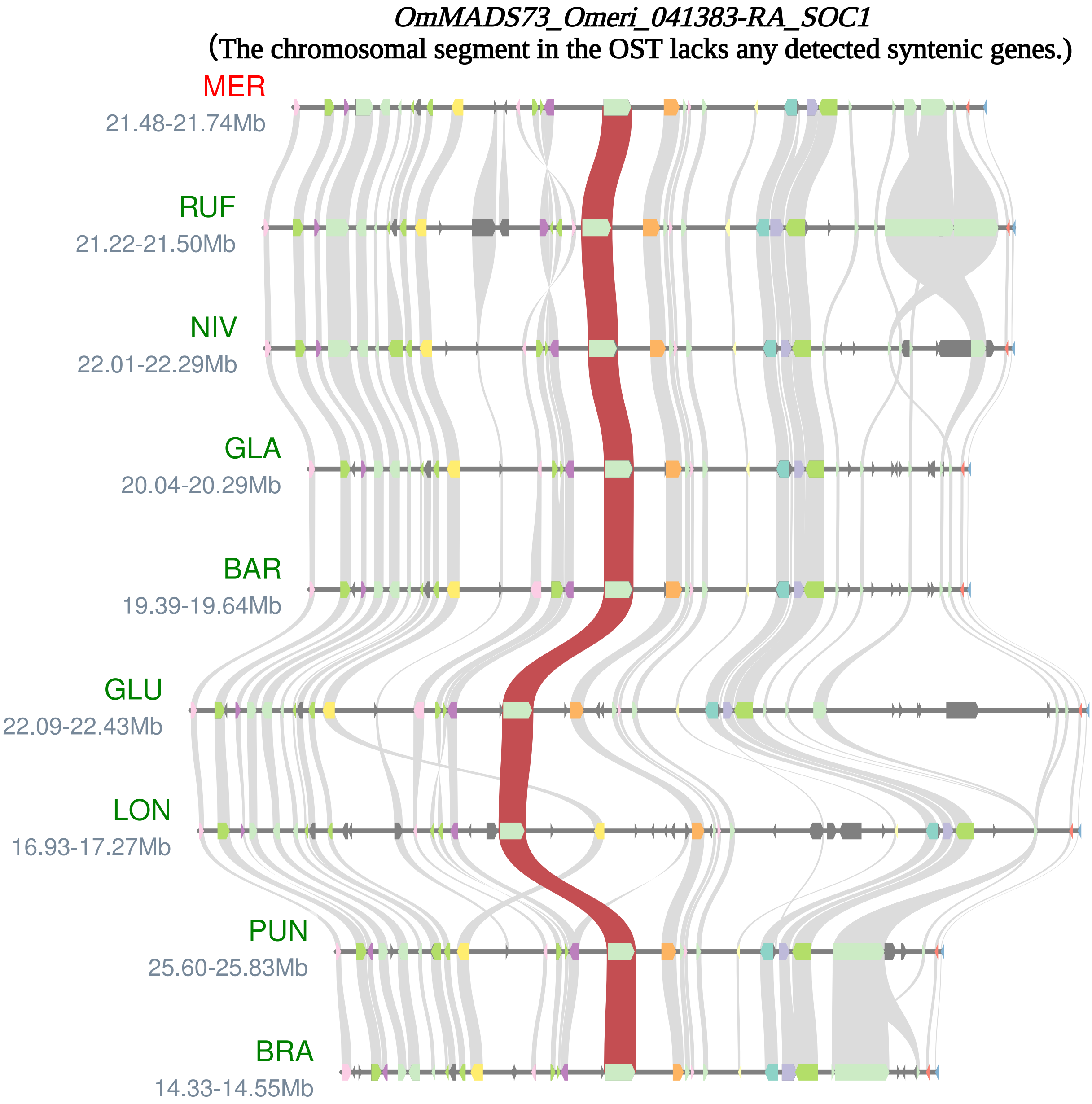
OmMADS73_Omeri_041383-RA_SOC1
(The chromosomal segment in the OST lacks any detected syntenic genes.)
MER
21.48-21.74Mb
RUF
21.22-21.50Mb
NIV
22.01-22.29Mb
GLA
20.04-20.29Mb
BAR
19.39-19.64Mb
GLU
22.09-22.43Mb
LON
16.93-17.27Mb
PUN
25.60-25.83Mb
BRA
14.33-14.55Mb
