# Supplementary material for: Evolutionary Dynamics and Expression Divergence of the MADS-Box Gene Family During Recent Speciation of AA-Genome Oryza Species
Source: Plants (Basel). 2025 Jan 26;14(3):379. doi: 10.3390/plants14030379 (PMC11820988; doi:10.3390/plants14030379)
Supplement: Supplementary file 1 [file plants-14-00379-s001.zip › Supplementary Figure S5.pdf]

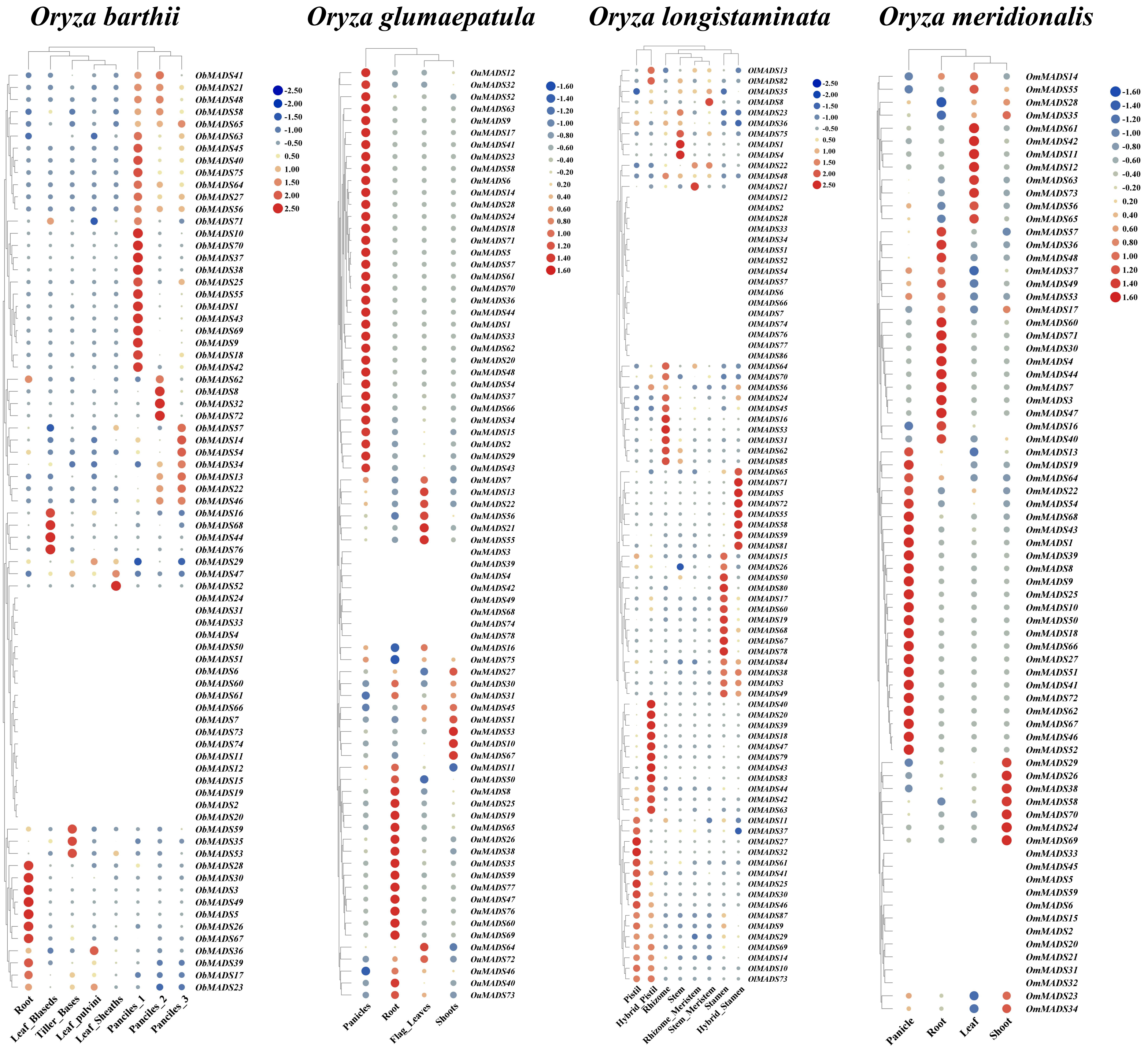
Oryza barthii
ObMADS41
ObMADS21
ObMADS48
ObMADS58
ObMADS65
ObMADS63
ObMADS45
ObMADS40
ObMADS75
ObMADS64
ObMADS27
ObMADS56
ObMADS71
ObMADS10
ObMADS70
ObMADS37
ObMADS38
ObMADS25
ObMADS55
ObMADS1
ObMADS43
ObMADS69
ObMADS9
ObMADS18
ObMADS42
ObMADS62
ObMADS8
ObMADS32
ObMADS72
ObMADS57
ObMADS14
ObMADS54
ObMADS34
ObMADS13
ObMADS22
ObMADS46
ObMADS16
ObMADS68
ObMADS44
ObMADS76
ObMADS29
ObMADS47
ObMADS52
ObMADS24
ObMADS31
ObMADS33
ObMADS4
ObMADS50
ObMADS51
ObMADS6
ObMADS60
ObMADS61
ObMADS66
ObMADS7
ObMADS73
ObMADS74
ObMADS11
ObMADS12
ObMADS15
ObMADS19
ObMADS2
ObMADS20
ObMADS59
ObMADS35
ObMADS53
ObMADS28
ObMADS30
ObMADS3
ObMADS49
ObMADS5
ObMADS26
ObMADS67
ObMADS36
ObMADS39
ObMADS17
ObMADS23
-2.50
-2.00
-1.50
-1.00
-0.50
0.50
1.00
1.50
2.00
2.50
Root
Leaf_Blaseds
Tiller_Bases
Leaf_pulvini
Leaf_Sheaths
Panciles_1
Panciles_2
Panciles_3
Oryza glumaepatula
OuMADS12
OuMADS32
OuMADS52
OuMADS63
OuMADS9
OuMADS17
OuMADS41
OuMADS23
OuMADS58
OuMADS6
OuMADS14
OuMADS28
OuMADS24
OuMADS18
OuMADS71
OuMADS5
OuMADS57
OuMADS61
OuMADS70
OuMADS36
OuMADS44
OuMADS1
OuMADS33
OuMADS62
OuMADS20
OuMADS48
OuMADS54
OuMADS37
OuMADS66
OuMADS34
OuMADS15
OuMADS2
OuMADS29
OuMADS43
OuMADS7
OuMADS13
OuMADS22
OuMADS56
OuMADS21
OuMADS55
OuMADS3
OuMADS39
OuMADS4
OuMADS42
OuMADS49
OuMADS68
OuMADS74
OuMADS78
OuMADS16
OuMADS75
OuMADS27
OuMADS30
OuMADS31
OuMADS45
OuMADS51
OuMADS53
OuMADS10
OuMADS67
OuMADS11
OuMADS50
OuMADS8
OuMADS25
OuMADS19
OuMADS65
OuMADS26
OuMADS38
OuMADS35
OuMADS59
OuMADS77
OuMADS47
OuMADS76
OuMADS60
OuMADS69
OuMADS64
OuMADS72
OuMADS46
OuMADS40
OuMADS73
-1.60
-1.40
-1.20
-1.00
-0.80
-0.60
-0.40
-0.20
0.20
0.40
0.60
0.80
1.00
1.20
1.40
1.60
Panicles
Root
Flag_Leaves
Shoots
Oryza longistaminata
OlMADS13
OlMADS82
OlMADS35
OlMADS8
OlMADS23
OlMADS36
OlMADS75
OlMADS1
OlMADS4
OlMADS22
OlMADS48
OlMADS21
OlMADS12
OlMADS2
OlMADS28
OlMADS33
OlMADS34
OlMADS51
OlMADS52
OlMADS54
OlMADS57
OlMADS6
OlMADS66
OlMADS7
OlMADS74
OlMADS76
OlMADS77
OlMADS86
OlMADS64
OlMADS70
OlMADS56
OlMADS24
OlMADS45
OlMADS16
OlMADS53
OlMADS31
OlMADS62
OlMADS85
OlMADS65
OlMADS71
OlMADS5
OlMADS72
OlMADS55
OlMADS58
OlMADS59
OlMADS81
OlMADS15
OlMADS26
OlMADS50
OlMADS80
OlMADS17
OlMADS60
OlMADS19
OlMADS68
OlMADS67
OlMADS78
OlMADS84
OlMADS38
OlMADS3
OlMADS49
OlMADS40
OlMADS20
OlMADS39
OlMADS18
OlMADS47
OlMADS79
OlMADS43
OlMADS83
OlMADS44
OlMADS42
OlMADS63
OlMADS11
OlMADS37
OlMADS27
OlMADS32
OlMADS61
OlMADS41
OlMADS25
OlMADS30
OlMADS46
OlMADS87
OlMADS9
OlMADS29
OlMADS69
OlMADS14
OlMADS10
OlMADS73
-2.50
-2.00
-1.50
-1.00
-0.50
0.50
1.00
1.50
2.00
2.50
Pistil
Hybrid_Pistil
Rhizome
Stem
Rhizome_Meristem
Stem_Meristem
Stamen
Hybrid_Stamen
Oryza meridionalis
OmMADS14
OmMADS55
OmMADS28
OmMADS35
OmMADS61
OmMADS42
OmMADS11
OmMADS12
OmMADS63
OmMADS73
OmMADS56
OmMADS65
OmMADS57
OmMADS36
OmMADS48
OmMADS37
OmMADS49
OmMADS53
OmMADS17
OmMADS60
OmMADS71
OmMADS30
OmMADS4
OmMADS44
OmMADS7
OmMADS3
OmMADS47
OmMADS16
OmMADS40
OmMADS13
OmMADS19
OmMADS64
OmMADS22
OmMADS54
OmMADS68
OmMADS43
OmMADS1
OmMADS39
OmMADS8
OmMADS9
OmMADS25
OmMADS10
OmMADS50
OmMADS18
OmMADS66
OmMADS27
OmMADS51
OmMADS41
OmMADS72
OmMADS62
OmMADS67
OmMADS46
OmMADS52
OmMADS29
OmMADS26
OmMADS38
OmMADS58
OmMADS70
OmMADS24
OmMADS69
OmMADS33
OmMADS45
OmMADS5
OmMADS59
OmMADS6
OmMADS15
OmMADS2
OmMADS20
OmMADS21
OmMADS31
OmMADS32
OmMADS23
OmMADS34
-1.60
-1.40
-1.20
-1.00
-0.80
-0.60
-0.40
-0.20
0.20
0.40
0.60
0.80
1.00
1.20
1.40
1.60
Panicle
Root
Leaf
Shoot

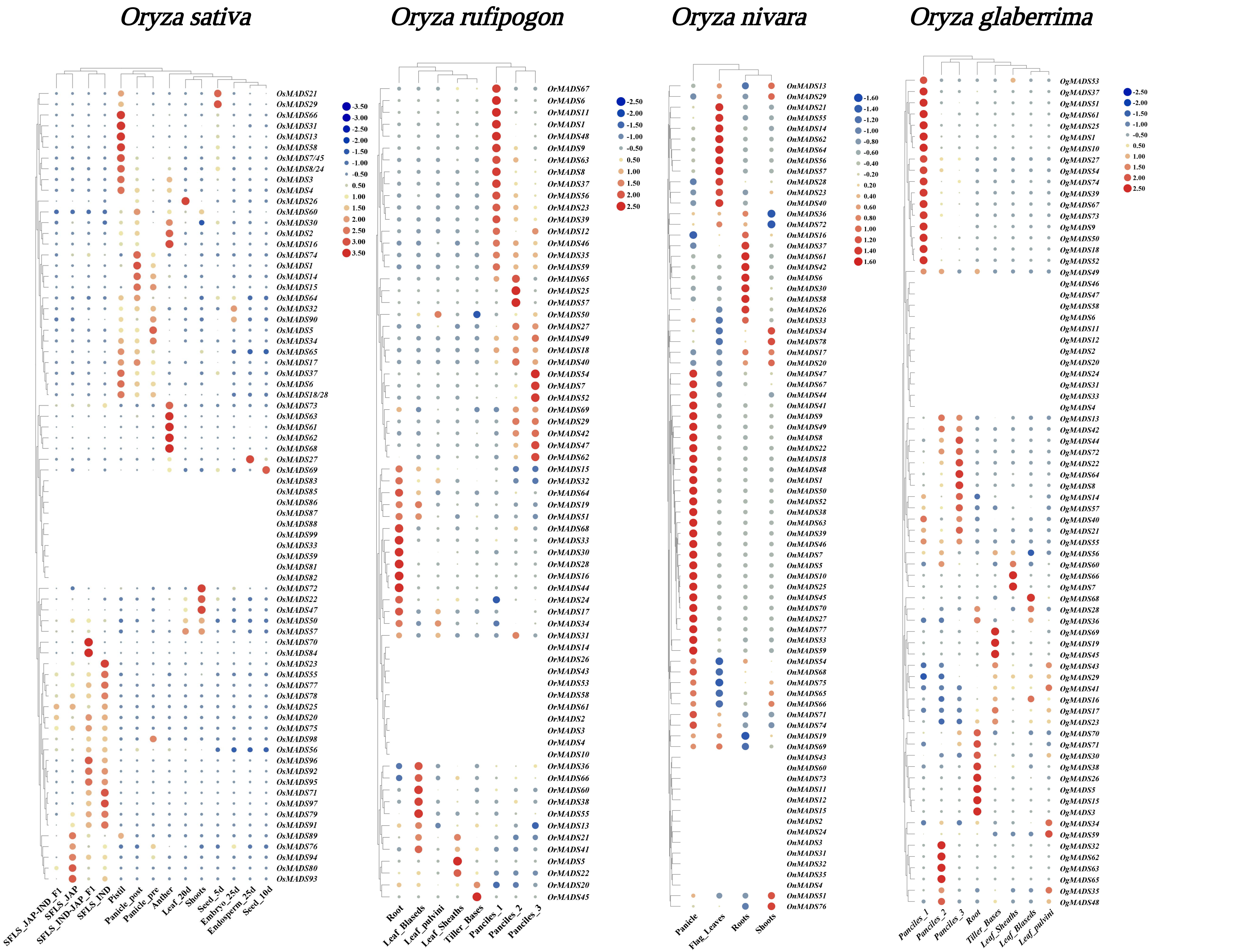
Oryza sativa
Oryza rufipogon
Oryza nivara
Oryza glaberrima
SFLS_JAP-IND_F1
SFLS_JAP
SFLS_IND-JAP_F1
SFLS_IND
Pistil
Panicle_post
Panicle_pre
Anther
Leaf_20d
Shoots
Seed_5d
Embryo_25d
Endosperm_25d
Seed_10d
Root
Leaf_Blaseds
Leaf_pulvini
Leaf_Sheaths
Tiller_Bases
Panciles_1
Panciles_2
Panciles_3
Panicle
Flag_Leaves
Roots
Shoots
Panciles_1
Panciles_2
Panciles_3
Root
Tiller_Bases
Leaf_Sheaths
Leaf_Blaseds
Leaf_pulvini
-3.50
-3.00
-2.50
-2.00
-1.50
-1.00
-0.50
0.50
1.00
1.50
2.00
2.50
3.00
3.50
-2.50
-2.00
-1.50
-1.00
-0.50
0.50
1.00
1.50
2.00
2.50
-1.60
-1.40
-1.20
-1.00
-0.80
-0.60
-0.40
-0.20
0.20
0.40
0.60
0.80
1.00
1.20
1.40
1.60
-2.50
-2.00
-1.50
-1.00
-0.50
0.50
1.00
1.50
2.00
2.50
