# Supplementary material for: Evolutionary Dynamics and Expression Divergence of the MADS-Box Gene Family During Recent Speciation of AA-Genome Oryza Species
Source: Plants (Basel). 2025 Jan 26;14(3):379. doi: 10.3390/plants14030379 (PMC11820988; doi:10.3390/plants14030379)

Oryza meridionalis

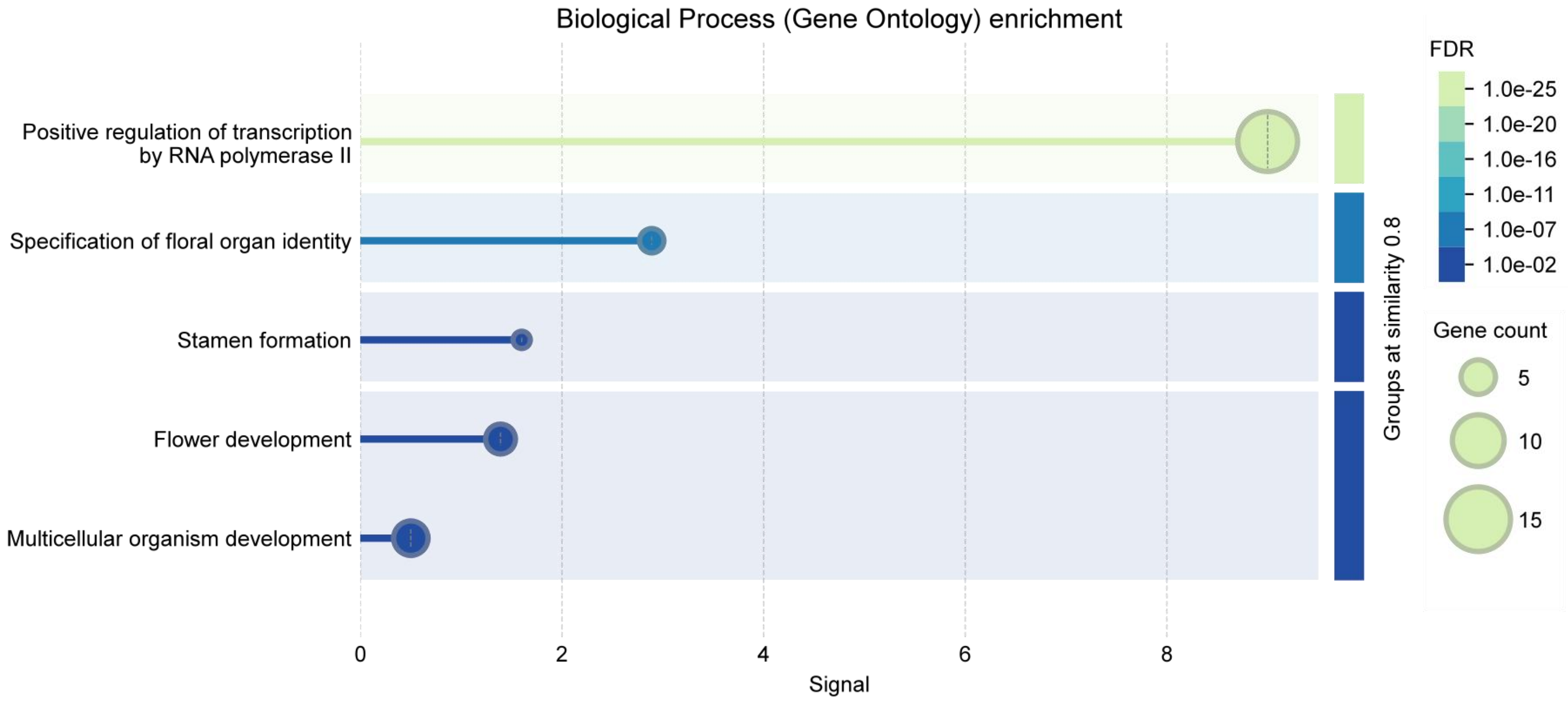

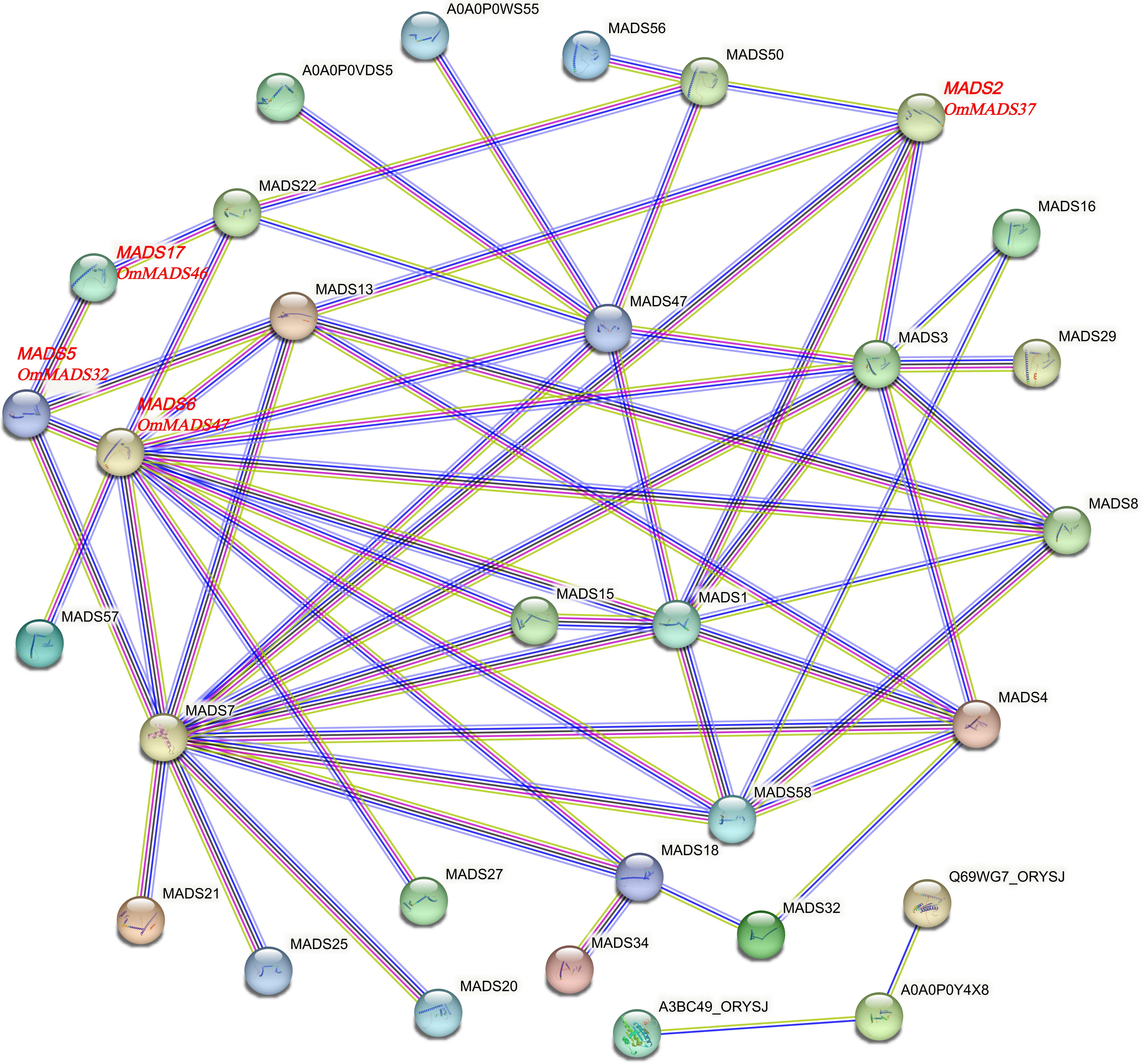

Oryza longistaminata

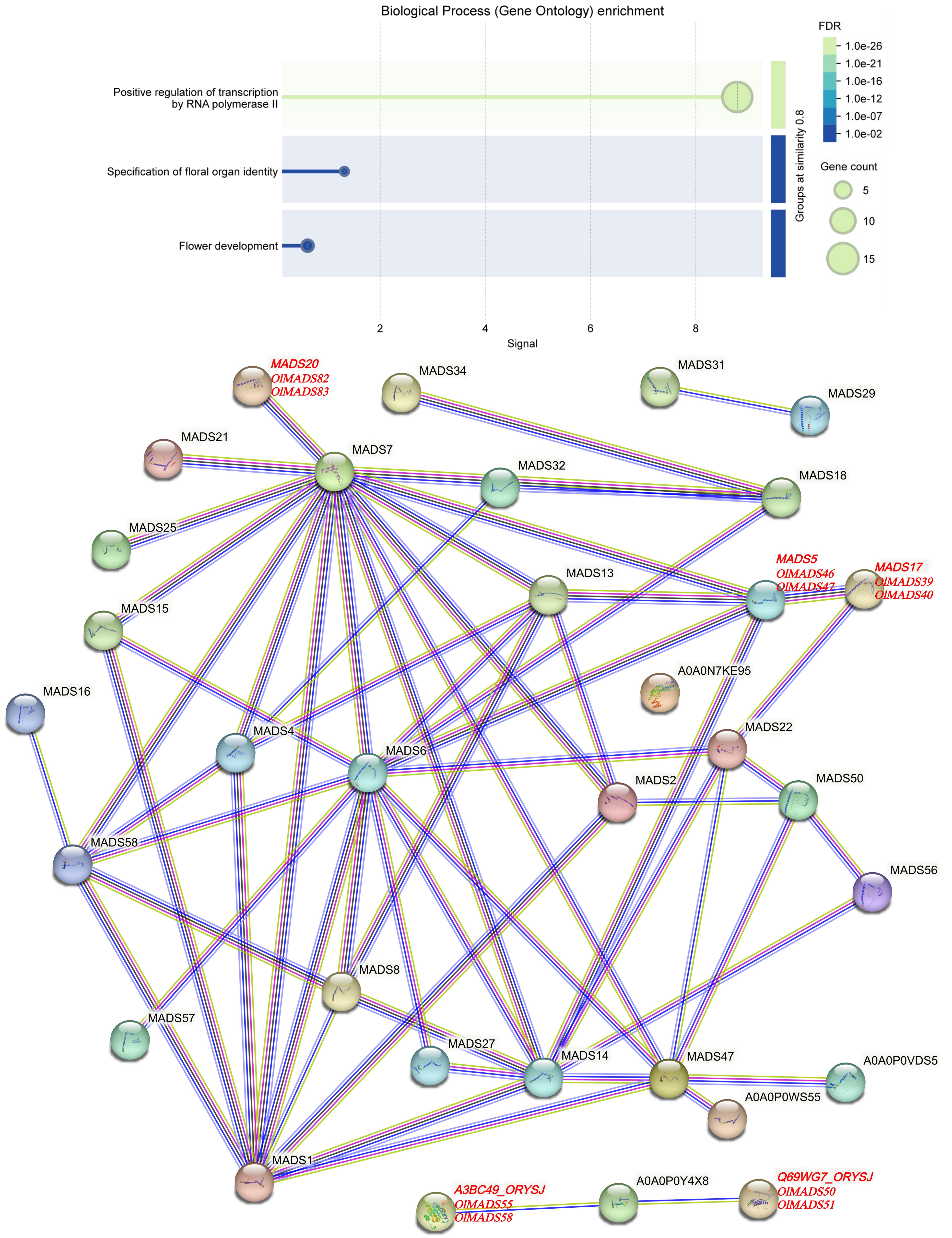

Oryza glumipatula

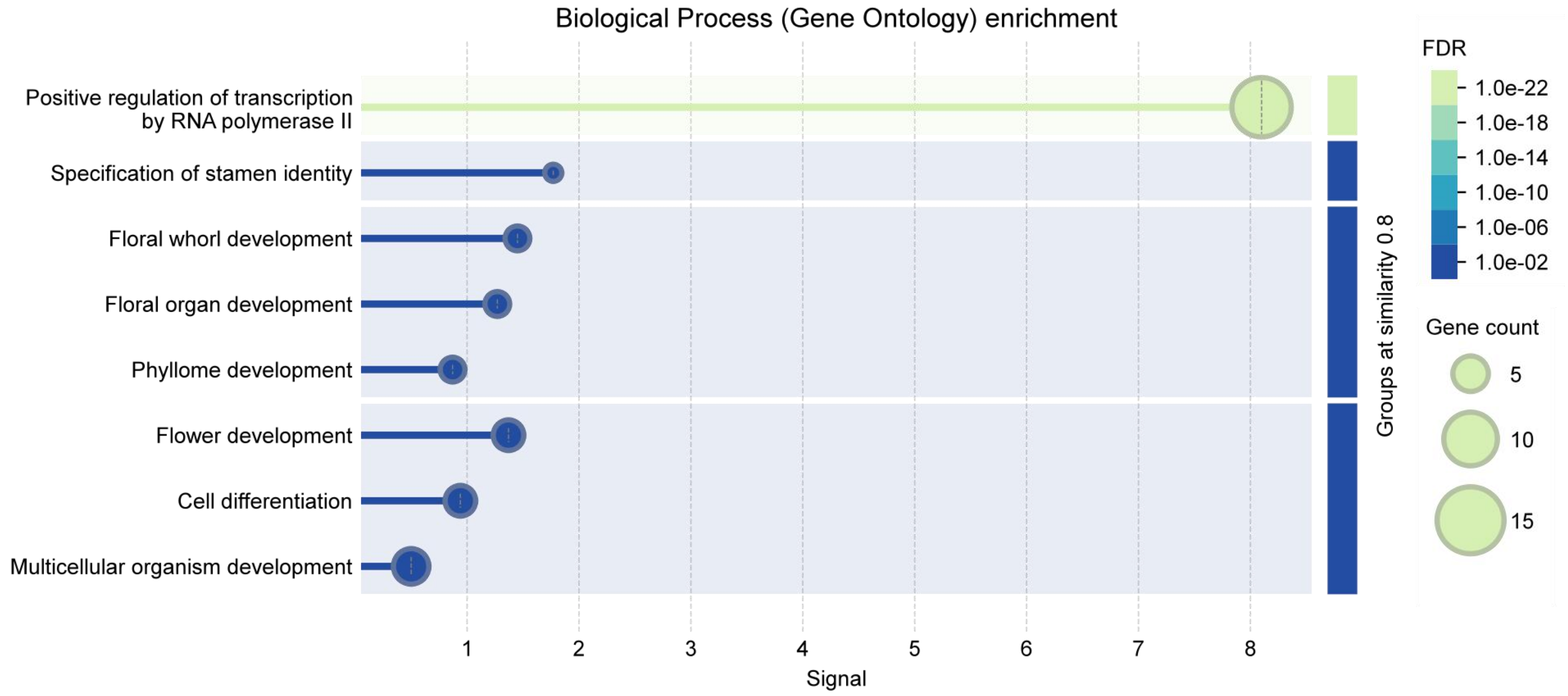

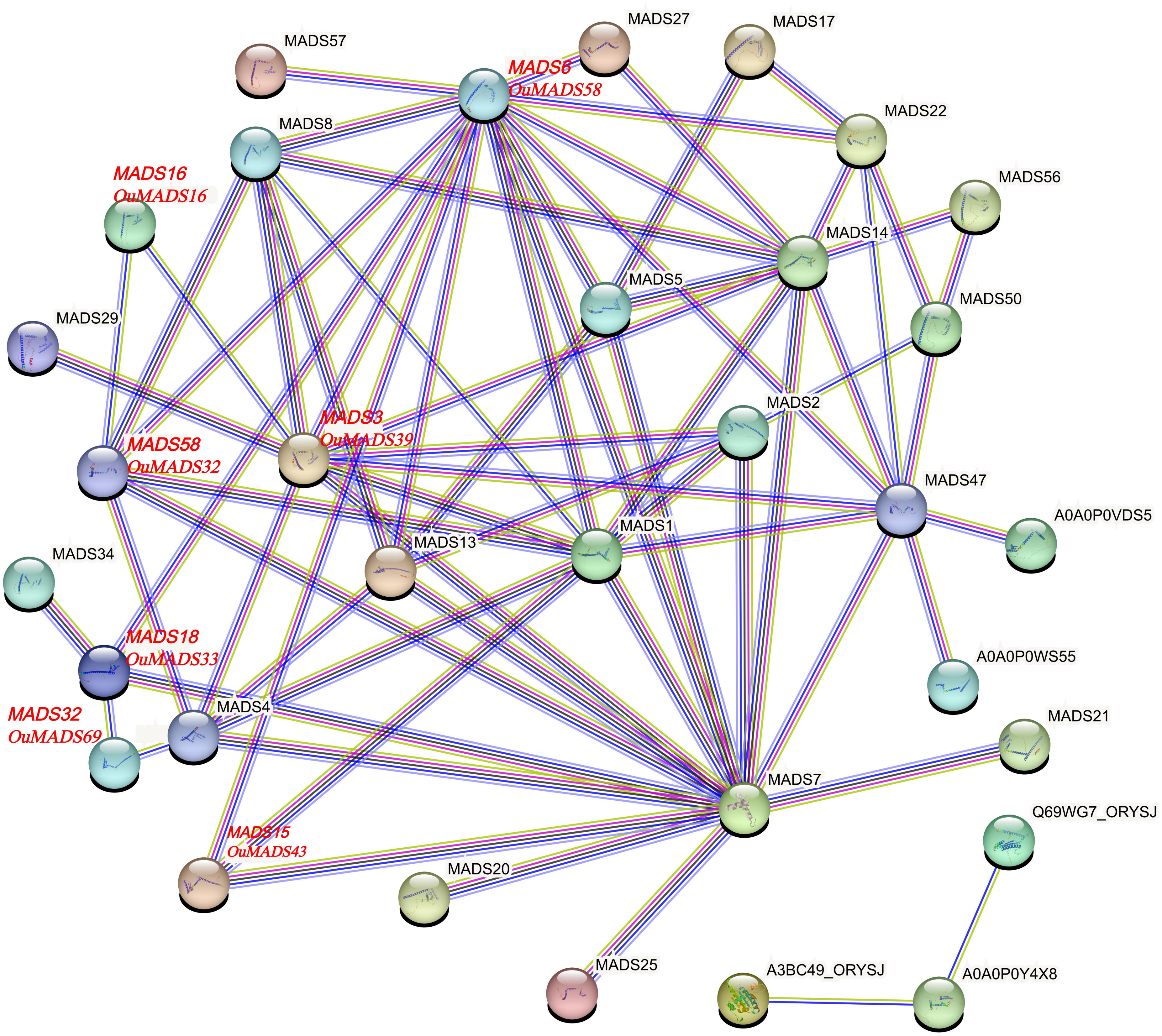

Oryza barthii

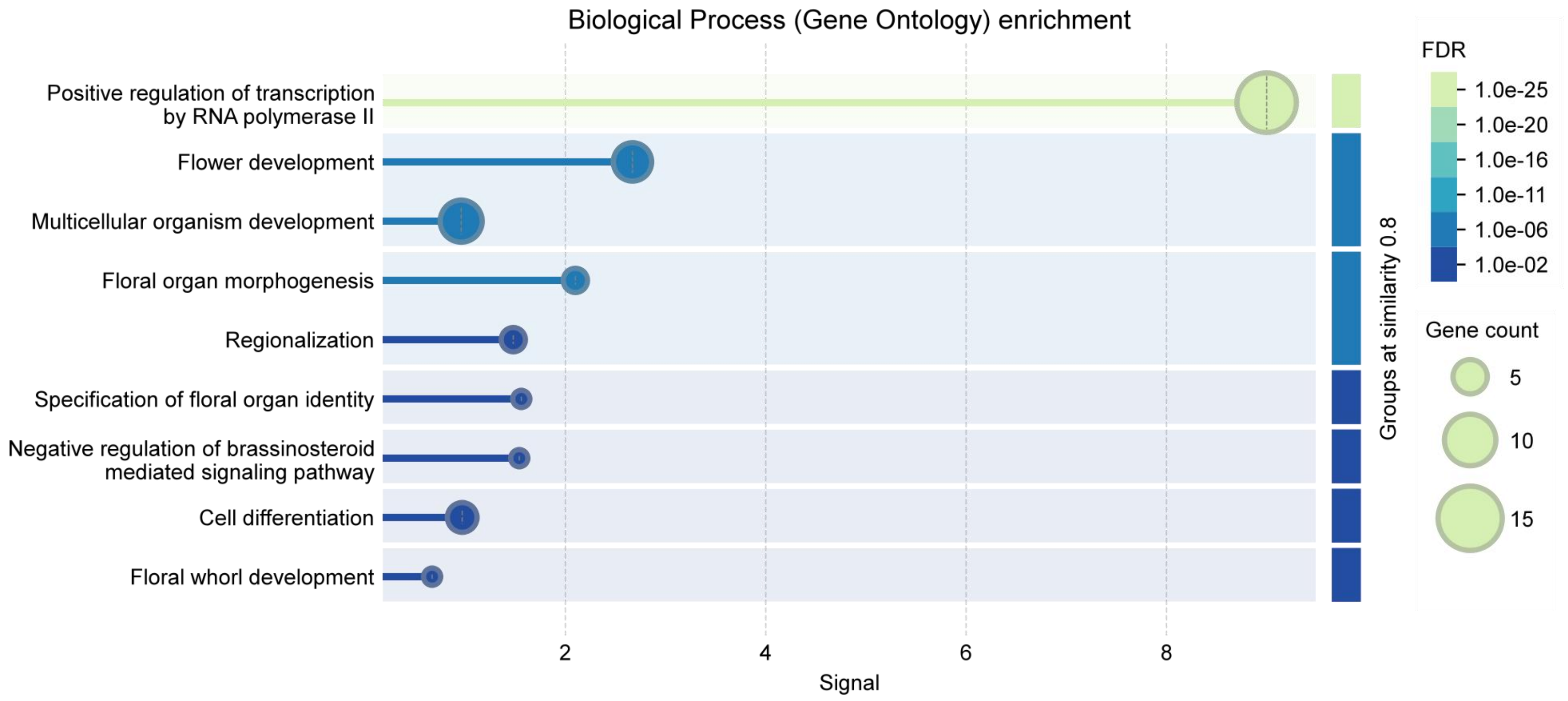

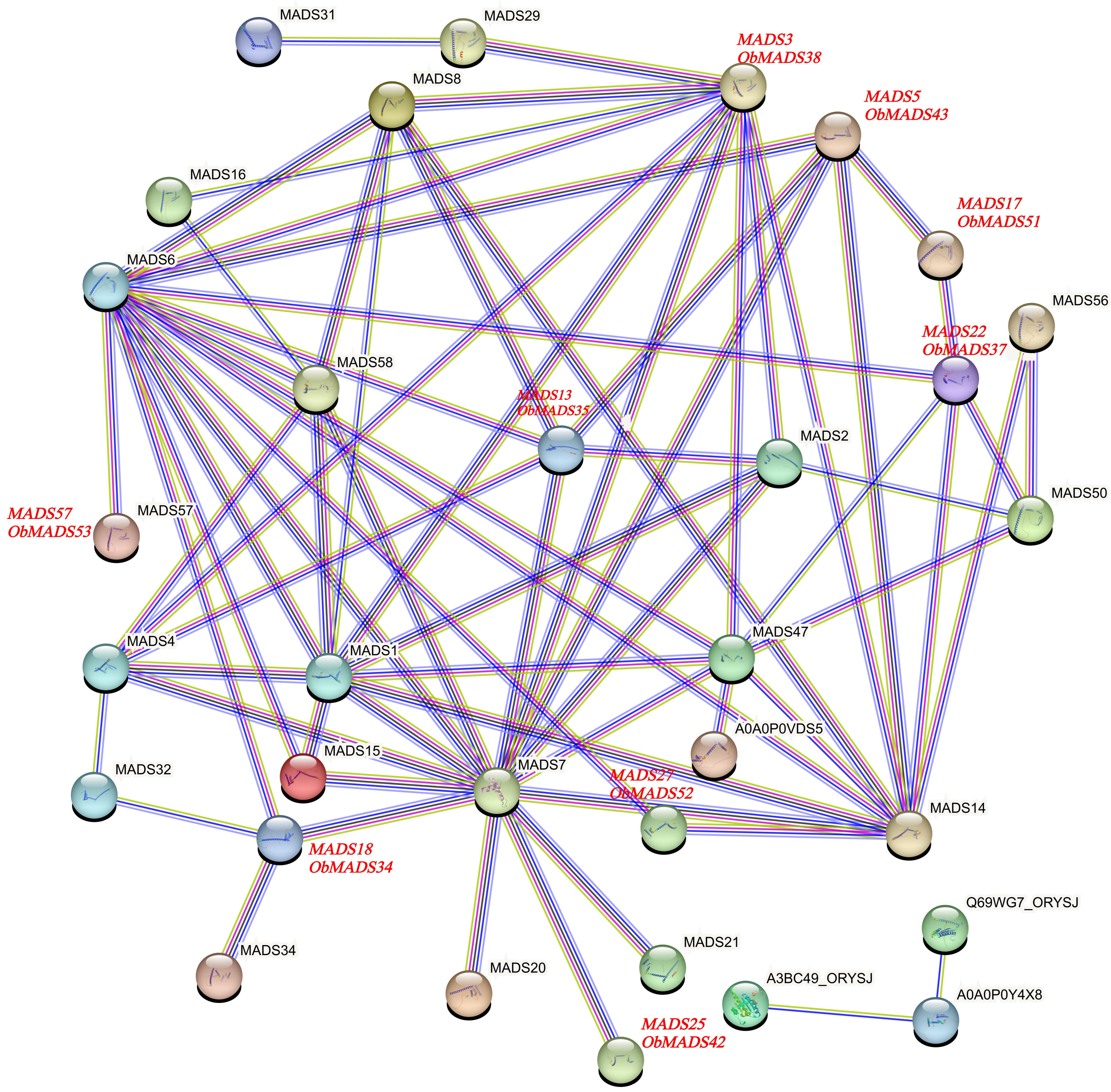

Oryza glaberrima

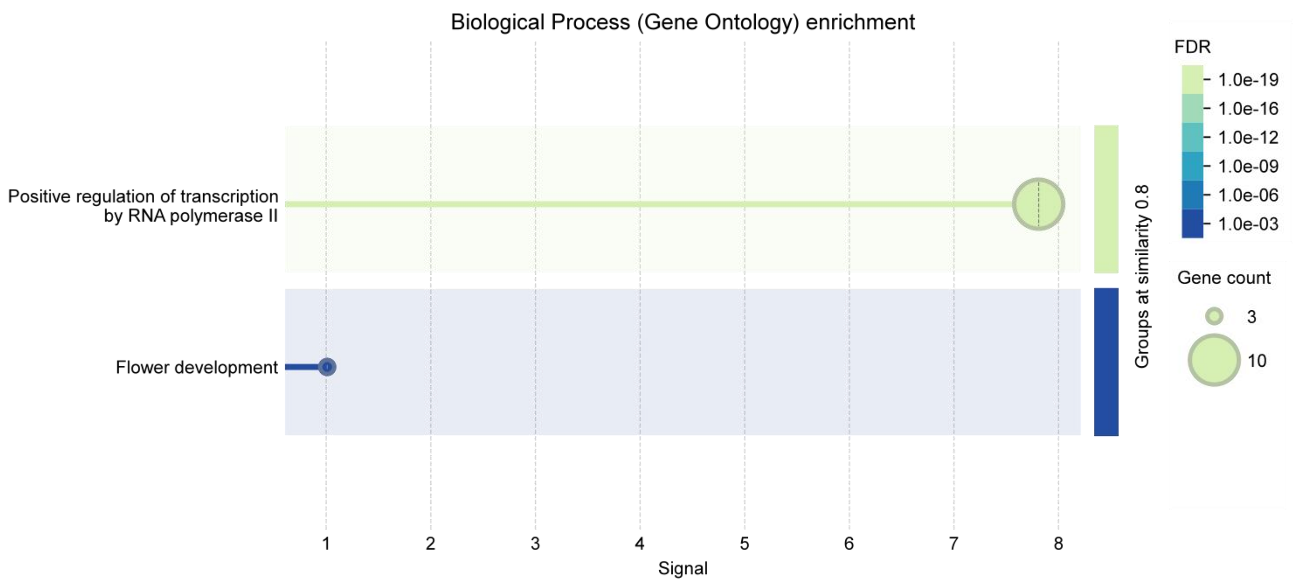

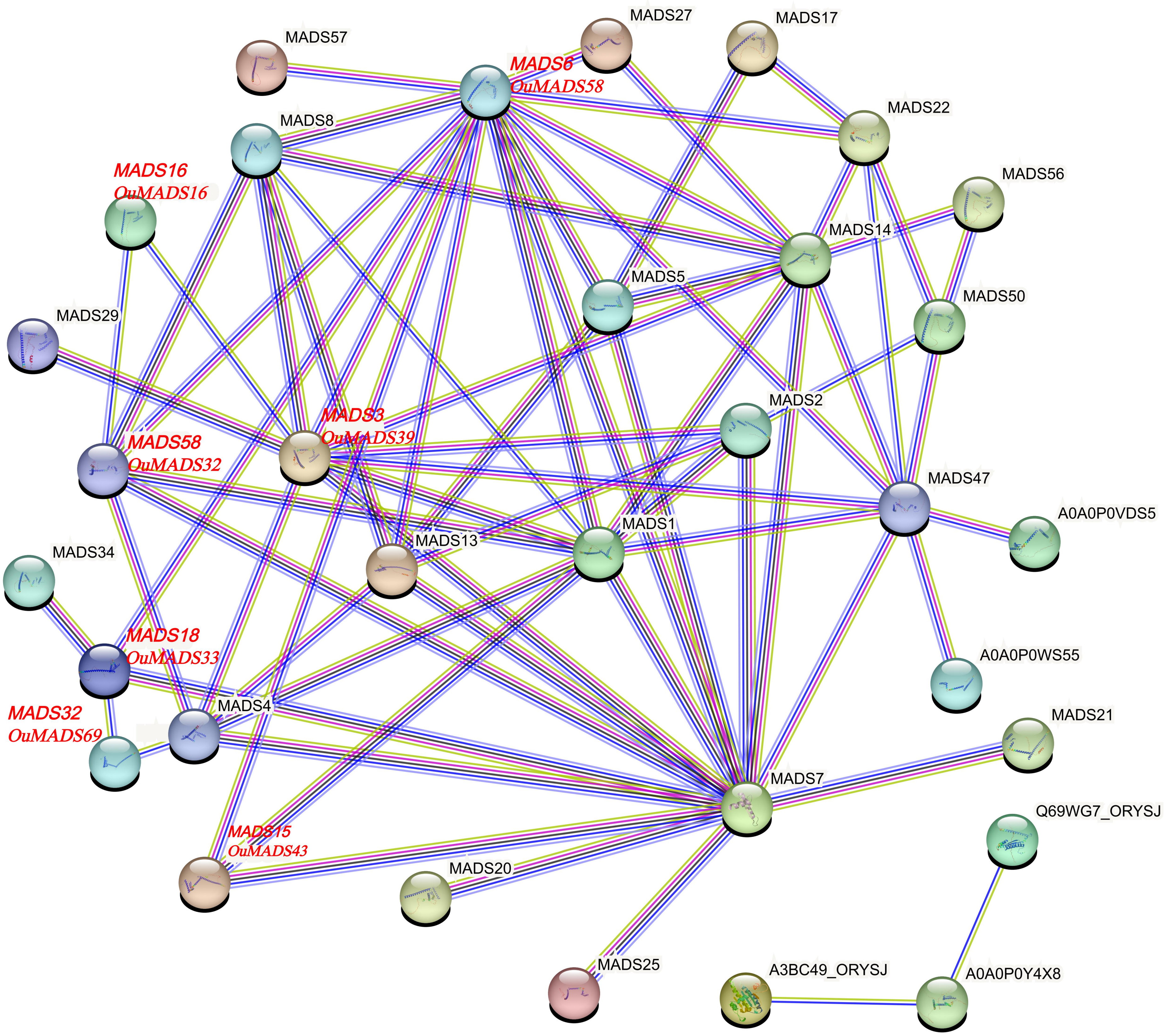

Oryza rufipogon

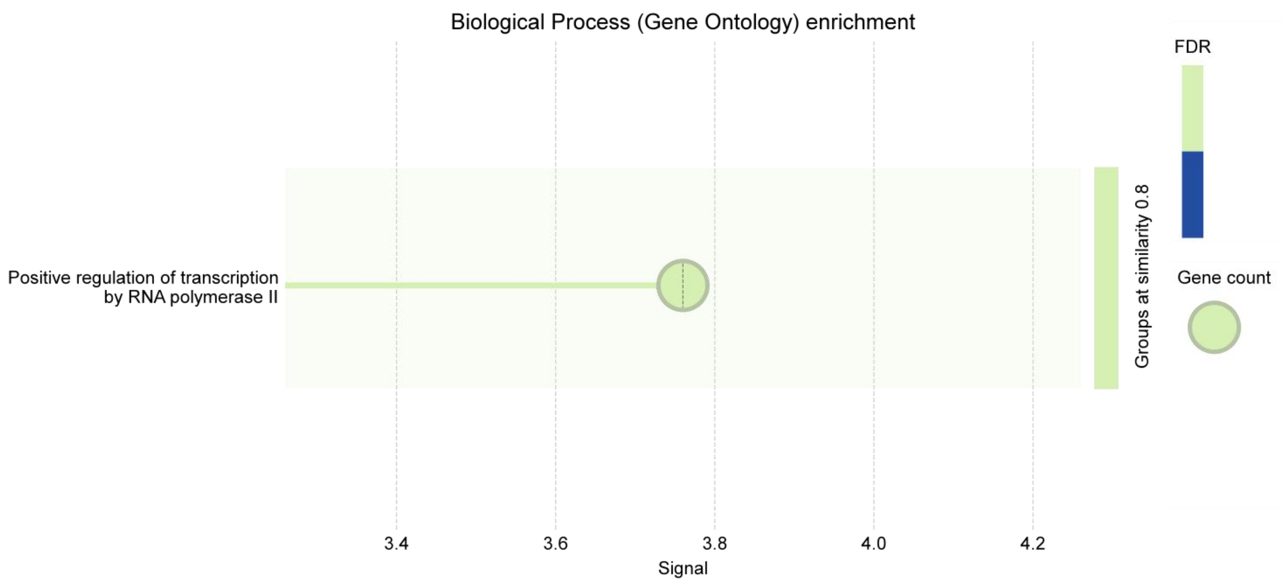

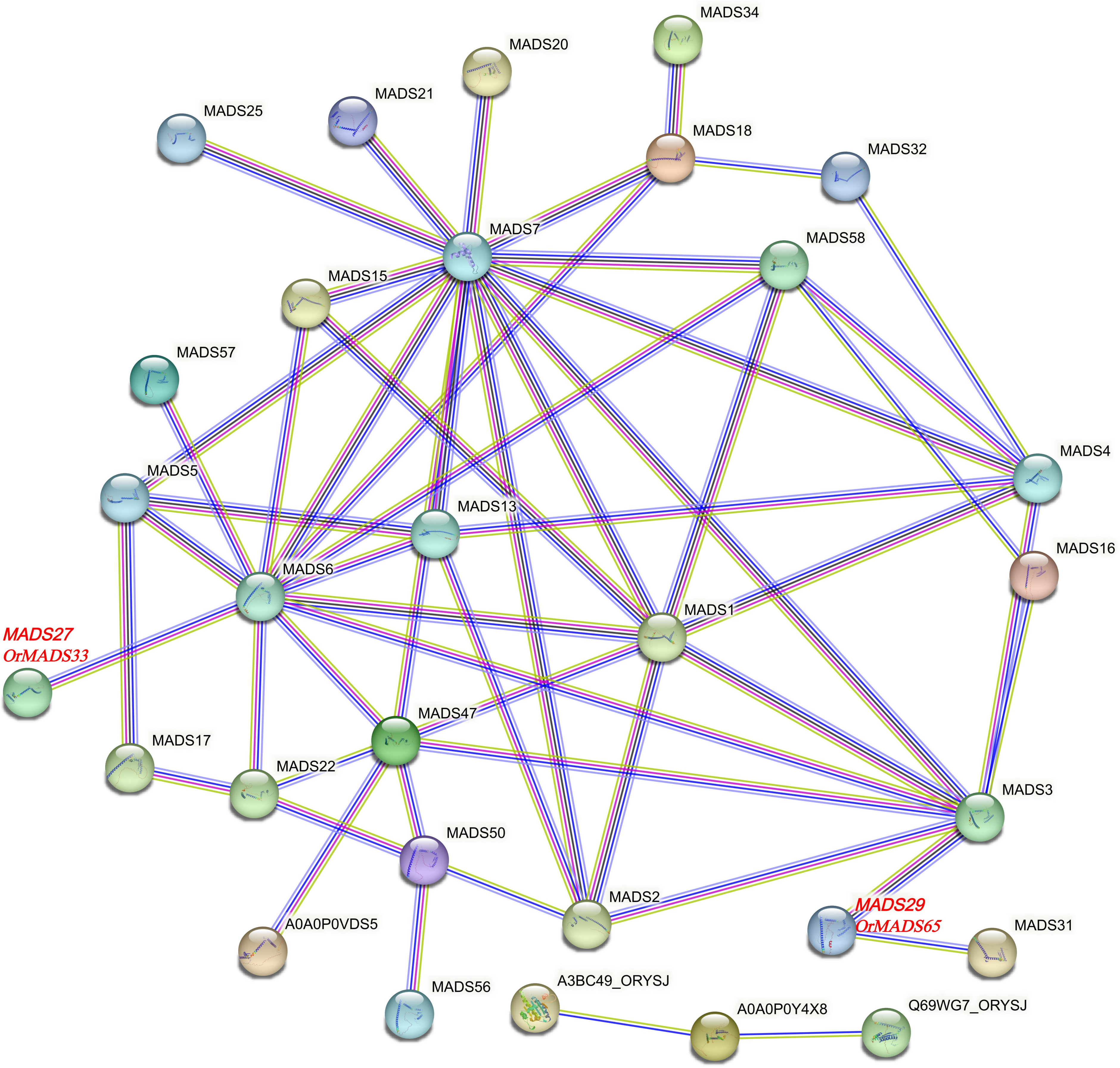

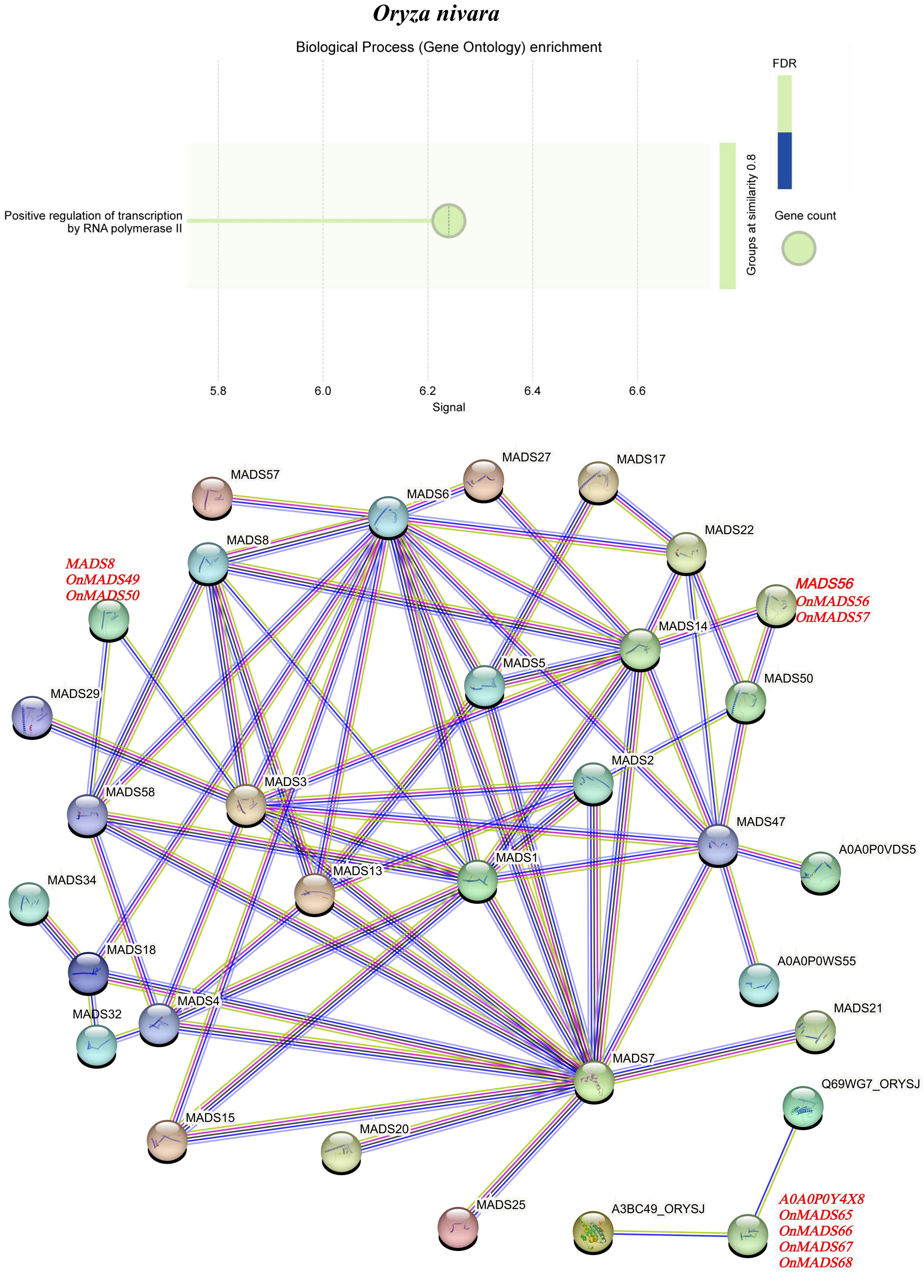
Oryza nivara
Biological Process (Gene Ontology) enrichment
FDR
Gene count
Groups at similarity 0.8
Positive regulation of transcription by RNA polymerase II
5.8
6.0
6.2
6.4
6.6
Signal
MADS57
MADS6
MADS27
MADS17
MADS22
MADS8
MADS8
OnMADS49
OnMADS50
MADS56
OnMADS56
OnMADS57
MADS14
MADS5
MADS50
MADS29
MADS2
MADS58
MADS3
MADS47
A0A0P0VDS5
MADS1
MADS13
MADS34
A0A0P0WS55
MADS18
MADS4
MADS32
MADS21
MADS7
Q69WG7_ORYSJ
MADS15
MADS20
MADS25
A3BC49_ORYSJ
A0A0P0Y4X8
OnMADS65
OnMADS66
OnMADS67
OnMADS68

Oryza sativa

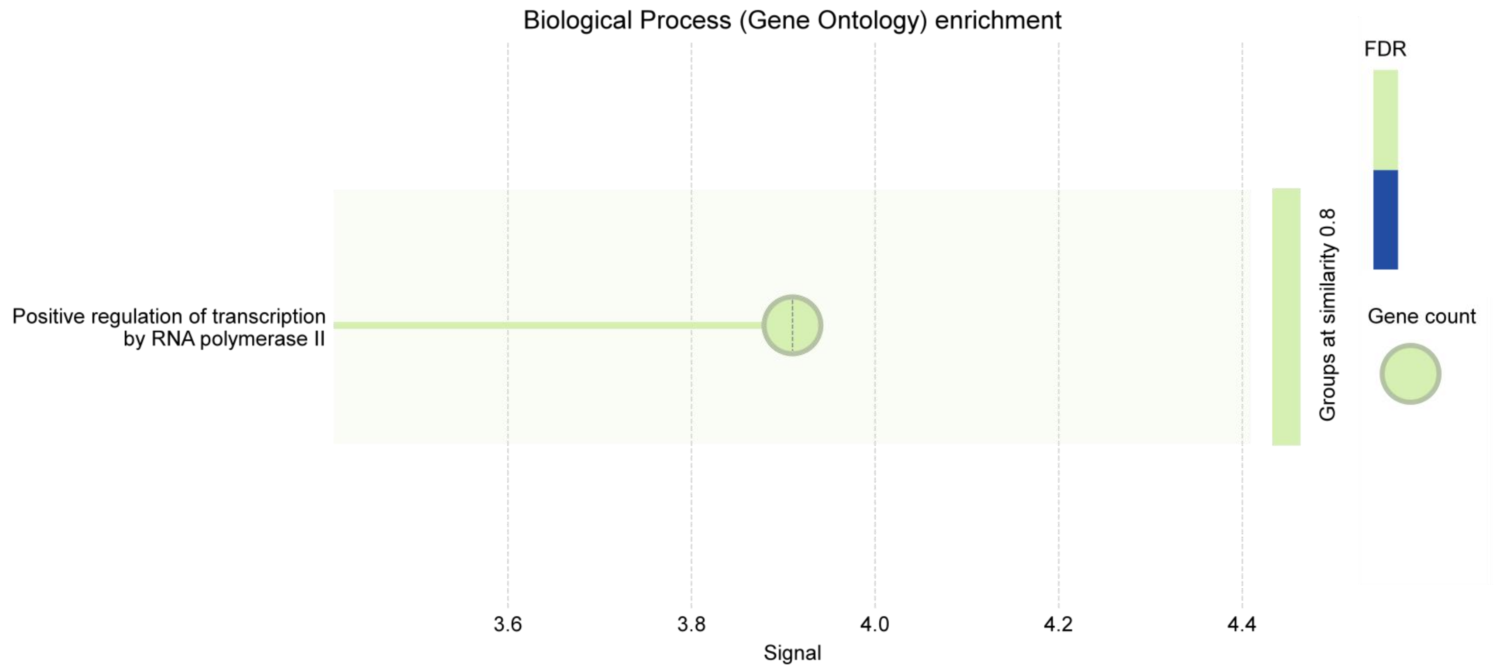

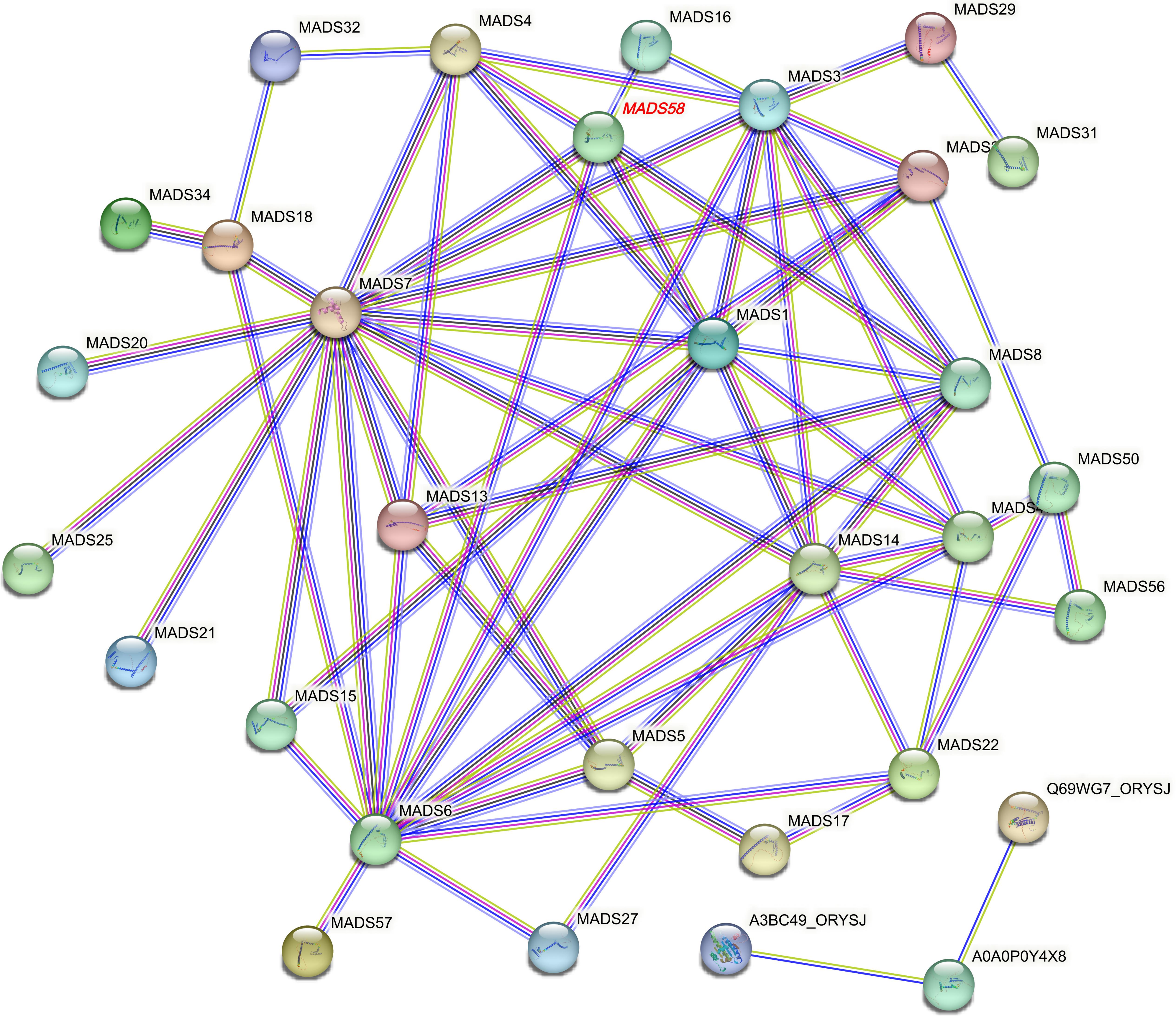

Supplement: Supplementary file 1 [file plants-14-00379-s001.zip › Supplementary Figure S6.pdf]
